# Supplementary material for: Highly regulated, diversifying NTP-dependent biological conflict systems with implications for the emergence of multicellularity
Source: eLife. 2020 Feb 26;9:e52696. doi: 10.7554/eLife.52696 (PMC7159879; doi:10.7554/eLife.52696)
Supplement: Figure 1—source data 1. [file elife-52696-fig1-data1.pdf]

Highly-regulated, diversifying NTP-dependent biological conflict systems with implications for the emergence of multicellularity

SOURCE DATA

Gurmeet Kaur†, A. Maxwell Burroughs†, Lakshminarayan M. Iyer, L. Aravind*

Computational Biology Branch, National Center for Biotechnology Information, National Library of Medicine, National Institutes of Health, Bethesda, MD 20894, USA

†These authors contributed equally to the manuscript

Source Data

I. Source data - genome identifiers, multicellular status, and systems in genome
II. Source data - domain architectures, genome identifiers, gene neighborhoods
III. Source data - profile-profile comparisons
IV. Source data - pairwise systems comparison in same genomes
V. Source data - organisms with multiple systems in same genomes
VI. Source data - genome identifiers and systems for all EAD1-containing organisms
VII. Source data - organism abbreviations
VIII. Source data - multiple sequence alignments

I. Source data - genome identifiers, multicellular status, and systems in genome

VMAP systems

■ **GCA_000349325.1; Streptomyces davawensis JCM 4913; counts= 6; multicellularity status: TRUE**

- AB_hydrolase->CASPASE+VMAP-M0+VMAP-C->MoxR->vWA+STAND_NTPase+Trypsin+CASPASE+S1COLD*->
- AB_hydrolase->EAD2+VMAP-M0+VMAP-C->MoxR->vWA+APATPase+wHTH+TPRs*->||<-CASPASE+EAD2
- CASPASE+VMAP-M0+VMAP-C->vWA+HTH*->
- EAD2+VMAP-M0+VMAP-C->vWA+APATPase+wHTH+TPRs*->||<-METALLOPROTEASE+TPRs<-FXSXX-COOH-like<-Trypsin+EAD2||FXSXX-COOH-like->METALLOPROTEASE+TPRs->
- Trypco1->Trypsin+VMAP-M0+VMAP-C->MoxR->vWA+Pkinase+wHTH+TPRs+cNMP_cyclase+Band7+HAD*->
- Trypco1->Trypsin+VMAP-M7+VMAP-C->MoxR->vWA+EAD2+VMAP-M0+VMAP-C*->

■ **GCA_000014265.1; Trichodesmium erythraeum IMS101; counts= 5; multicellularity status: TRUE**

- CASPASE+VMAP-C->MoxR->?->vWA(partial)->vWA+HTH+HTH+HTH+HTH+HTH+HTH+HTH+HTH+HTH+FGS-fold_domain*->
- EAD1+Trypsin->?->EAD1+VMAP-M0+VMAP-C->MoxR->?->||<-?||vWA+FGS-fold_domain*->
- EAD6+VMAP-M12+VMAP-C->MoxR->vWA+FGS-fold_domain+FGS-fold_domain*->
- EAD7+VMAP-M1+VMAP-C->MoxR->?->?->vWA+FGS-fold_domain*->FGS-fold_domain->
- Trypco1->Trypsin+VMAP-M1+VMAP-C->MoxR->vWA+FGS-fold_domain*->four_helix_bundle_protein->PSE->FGS-fold_domain->

■ **GCA_001685355.1; Streptomyces lincolnensis; counts= 5; multicellularity status: TRUE**

- AB_hydrolase->CASPASE+VMAP-M0+VMAP-C->MoxR->vWA+STAND_NTPase+Trypsin+TIR+CASPASE+EAD2*->
- AB_hydrolase->CASPASE+VMAP-M2+VMAP-C->MoxR->vWA+SFII-helicase+Z1*->
- EAD2+VMAP-M0+VMAP-C->MoxR->vWA+APATPase+wHTH+TPRs*->||<-CASPASE+EAD2
- EAD2+VMAP-M0+VMAP-C->MoxR->vWA+APATPase+wHTH+TPRs*->||<-Na_H_Exchanger||AB_hydrolase->CASPASE+EAD2->FXSXX-COOH-like->METALLOPROTEASE+TPRs->
- Trypco1->Trypsin+VMAP-M0+VMAP-C->MoxR->vWA+Pkinase+wHTH+TPRs+cNMP_cyclase+Band7+HAD*->?->?->?->||<-?||Radical_SAM->Antibiotic_NAT->TIR+FxsC_Cter->

■ **GCA_000092385.1; Streptomyces bingchenggensis BCW-1; counts= 4; multicellularity status: TRUE**

- EAD2+VMAP-M0+VMAP-C->MoxR->vWA+APATPase+wHTH+TPRs*->||<-METALLOPROTEASE+TPRs<-FXSXX-COOH-like<-?<-?<-Na_H_Exchanger
- PNP_UDP_1+EAD2->?->vWA->?->?->EAD2+VMAP-M0+VMAP-C->MoxR->vWA+APATPase+wHTH+TPRs*->||<-METALLOPROTEASE+TPRs
- PNP_UDP_1+EAD2->?->vWA*->?->?->EAD2+VMAP-M0+VMAP-C->MoxR->vWA+APATPase+wHTH+TPRs->||<-METALLOPROTEASE+TPRs
- Trypco1->Trypsin+EAD2+VMAP-M0+VMAP-C->MoxR->vWA+Pkinase+Pkinase+ParA-Soj-PloopNTPase(SIMIBI)*->Porphobil_deam+Porphobil_deamC->

■ **GCA_000761215.1; Streptomyces glaucescens; counts= 4; multicellularity status: TRUE**

- <-TCAD15||PSE->Trypsin+HDxxH+VMAP-M0+VMAP-C->MoxR->vWA+TIR+APATPase+Trypsin+HDxxH+VMAP-M0+VMAP-C*->
- DRHyd+VMAP-M0+VMAP-C->MoxR->vWA+TIR+APATPase+wHTH+TPRs*->
- EAD2+VMAP-M0+VMAP-C->MoxR->vWA+APATPase+wHTH+TPRs*->||<-METALLOPROTEASE+TPRs<-FXSXX-COOH-like
- Trypco1->Trypsin+VMAP-M0+VMAP-C->MoxR->vWA+Pkinase+wHTH+TPRs+cNMP_cyclase+Band7+HAD*->

■ **GCA_001485105.1; Streptomyces acidiscabies; counts= 4; multicellularity status: TRUE**

- Trypco1->Trypsin+EAD2+VMAP-M0+VMAP-C->MoxR->vWA+Pkinase+ParA-Soj-PloopNTPase(SIMIBI)*->
- Trypco2->Trypsin+EAD2+VMAP-M0+VMAP-C->MoxR->vWA+Pkinase+TPRs*->
- vWA+Pkinase+wHTH+cNMP_cyclase+Band7+HAD*->
- vWA+TPRs*->FXSXX-COOH->Radical_SAM->METALLOPROTEASE+TPRs->TIR+APATPase+TPRs->

■ **GCA_001298565.1; Actinobacteria bacterium OK006; counts= 4; multicellularity status: NA**

- AB_hydrolase->CASPASE+VMAP-M2+VMAP-C->MoxR->vWA+BetaPropeller*->
- ADP_ribosyl_GH->NUDIX->Trypco1->Trypsin+EAD2+VMAP-M0+VMAP-C->MoxR->vWA+Pkinase+ParA-Soj-PloopNTPase(SIMIBI)*->
- EAD2+TIR->EAD2+VMAP-M0+VMAP-C->MoxR->vWA+APATPase+wHTH+TPRs*->||<-METALLOPROTEASE+TPRs
- EAD2+VMAP-M0+VMAP-C->vWA+APATPase+wHTH+TPRs*->||<-METALLOPROTEASE+TPRs<-FXSXX-COOH-like<-Trypsin+EAD2<-Trypco2||FXSXX-COOH-like->METALLOPROTEASE+TPRs->

■ **GCA_001514305.1; Streptomyces sp. RV15; counts= 4; multicellularity status: TRUE**

- EAD2+VMAP-M0+VMAP-C->MoxR->vWA+APATPase+wHTH+TPRs*->
- TIR+EAD2->EAD2+VMAP-M10+VMAP-C->MoxR->vWA+APATPase+Trypsin+TIR+FxsC_Cter(partial)*->||<-DUF397
- Trypco1->Trypsin+EAD2+VMAP-M0+VMAP-C->MoxR->vWA+Pkinase+ParA-Soj-PloopNTPase(SIMIBI)*->
- Trypco1->Trypsin+VMAP-M0+VMAP-C->MoxR->vWA+Pkinase+wHTH+TPRs+cNMP_cyclase+Band7+HAD*->

■ **GCA_002019855.1; Streptomyces antibioticus; counts= 4; multicellularity status: TRUE**

- TCAD9+Pkinase+NACHT+HEAT_repeats->EAD2+VMAP-M0+VMAP-C->vWA*->MoxR->vWA+APATPase+TPRs->
- Trypco1->Trypsin+VMAP-M0+VMAP-C->MoxR->vWA+Pkinase+wHTH+TPRs+cNMP_cyclase+Band7+HAD*->
- Trypsin+EAD2+VMAP-C->vWA+APATPase+wHTH+TPRs*->KAP_NTPase->
- vWA+APATPase+wHTH+TPRs*->||<-METALLOPROTEASE+TPRs<-FXSXX-COOH-like<-Trypsin+EAD2||METALLOPROTEASE+TPRs->

■ **GCA_900091315.1; Streptomyces sp. DI166; counts= 4; multicellularity status: TRUE**

- AB_hydrolase->CASPASE+VMAP-M0+VMAP-C->MoxR->vWA_protein*->Trypsin+CASPASE+S1COLD->
- CASPASE+VMAP-M0+VMAP-C->vWA+HTH*->
- EAD2+VMAP-M0+VMAP-C->vWA+APATPase+wHTH+TPRs*->
- Trypco1->Trypsin+VMAP-M0+VMAP-C->MoxR->vWA+Pkinase+wHTH+TPRs+cNMP_cyclase+Band7+HAD*->

■ **GCA_900129855.1; Streptomyces sp. 3124.6; counts= 4; multicellularity status: TRUE**

- <-TCAD15||Trypco1->Trypsin+HDxxH+VMAP-M0+VMAP-C->MoxR->vWA+TPRs*->FXSXX-COOH->Radical_SAM->METALLOPROTEASE+TPRs->TIR+APATPase+TPRs->
- AB_hydrolase->CASPASE+VMAP-M2+VMAP-C->MoxR->vWA+SFII-helicase+Z1*->
- Trypco1->Trypsin+EAD2+VMAP-M0+VMAP-C->MoxR->vWA+Pkinase+ParA-Soj-PloopNTPase(SIMIBI)*->
- Trypco1->Trypsin+VMAP-M0+VMAP-C->MoxR->vWA+cNMP_cyclase+Band7+HAD*->

■ **GCA_000346485.2; Scytonema hofmannii; counts= 4; multicellularity status: TRUE**

- EAD1+VMAP-M1+VMAP-C->MoxR->vWA+FGS-fold_domain*->four_helix_bundle_protein->RVT_1->
- low-complexity+VMAP-M26+VMAP-C->MoxR->vWA+CASPASE+APATPase*->
- TCAD3+EAD1+EAD9->EAD9+EAD1+VMAP-C->MoxR->vWA+FGS-fold_domain*->FGS-fold_domain->four_helix_bundle_protein->RVT_1->
- vWA_protein*->

■ **GCF_001189025.1; Streptomyces europaeiscabiei; counts= 4; multicellularity status: TRUE**

- <-TCAD15||Trypco1->Trypsin+HDxxH+VMAP-M0+VMAP-C->MoxR->vWA+TPRs*->Radical_SAM->TIR+APATPase+TPRs->

- AB_hydrolase->CASPASE+EAD2+VMAP-M0+VMAP-C->MoxR->vWA+TPRs*->
- EAD2+VMAP-M0+VMAP-C->MoxR->vWA+APATPase+wHTH+TPRs*->AB_hydrolase->CASPASE+EAD2->FXSXX-COOH-like->METALLOPROTEASE+TPRs->
- Trypco1->Trypsin+EAD2+VMAP-M0+VMAP-C->MoxR->vWA+Pkinase+ParA-Soj-PloopNTPase(SIMIBI)*->

■ **GCA_001426425.1; Streptomyces sp. Root1310; counts= 4; multicellularity status: TRUE**

- <-TCAD15||Trypco1->Trypsin+HDxxH+VMAP-M0+VMAP-C->MoxR->vWA+TPRs*->FXSXX-COOH->Radical_SAM->METALLOPROTEASE+TPRs->TIR+APATPase+TPRs->
- EAD2+VMAP-M0+VMAP-C->vWA+APATPase+wHTH+TPRs*->||<-METALLOPROTEASE+TPRs<-Trypsin+EAD2||Trypco2->
- Trypco1->Trypsin+EAD2+VMAP-M0+VMAP-C->MoxR->vWA+Pkinase+ParA-Soj-PloopNTPase(SIMIBI)*->
- Trypco1->Trypsin+VMAP-M0+VMAP-C->MoxR->vWA+cNMP_cyclase+Band7+HAD*->

■ **GCF_001650215.1; Streptomyces sp. RTd22; counts= 4; multicellularity status: TRUE**

- AB_hydrolase->CASPASE+EAD2+VMAP-M0+VMAP-C->MoxR->vWA+TPRs*->
- EAD2+VMAP-M0+VMAP-C->MoxR->vWA+APATPase+wHTH+TPRs*->||<-METALLOPROTEASE+TPRs<-Radical_SAM<-FXSXX-COOH||Voltage_CLC->||<-Trypco1
- PNP_UDP_1+EAD2->EAD2+VMAP-M0+VMAP-C->MoxR->vWA+APATPase+wHTH+TPRs*->||<-METALLOPROTEASE+TPRs
- Trypco1->Trypsin+EAD2+VMAP-M0+VMAP-C->MoxR->vWA+Pkinase+Pkinase+ParA-Soj-PloopNTPase(SIMIBI)*->Porphobil_deam+Porphobil_deamC->

■ **GCF_001941325.1; Streptomyces acidiscabies; counts= 4; multicellularity status: TRUE**

- Trypco1->Trypsin+EAD2+VMAP-M0+VMAP-C->MoxR->vWA+Pkinase+ParA-Soj-PloopNTPase(SIMIBI)*->
- Trypco2->Trypsin+EAD2+VMAP-M0+VMAP-C->MoxR->vWA+Pkinase+TPRs*->
- vWA+Pkinase+wHTH+cNMP_cyclase+Band7+HAD*->
- vWA+TIR+FxsC_Cter(partial)*->

■ **GCA_001027285.1; Archangium gephyra; counts= 3; multicellularity status: TRUE**

- EAD1+VMAP-M1+VMAP-C->MoxR->vWA+CASPASE+SLOG+TPRs*->
- EAD1+VMAP-M1+VMAP-C->MoxR->vWA+SLOG+SLOG+TPRs*->EAD1+Trypsin->
- EAD1+VMAP-M1+VMAP-C->vWA+SLOG*->

■ **GCA_001264245.1; Microcystis panniformis FACHB-1757; counts= 3; multicellularity status: TRUE**

- AB_hydrolase->CASPASE+VMAP-M14+VMAP-C->MoxR->vWA+FGS-fold_domain*->
- HTH+VMAP-M9+VMAP-C->MoxR->vWA+FGS-fold_domain*->?->||<-?||FGS-fold_domain->
- Trypco1->Trypsin+VMAP-M1+VMAP-C->MoxR->vWA+FGS-fold_domain*->?->||<-?<-?||FGS-fold_domain->

■ **GCA_001302585.1; Kibdelosporangium phytohabitans; counts= 3; multicellularity status: TRUE**

- AB_hydrolase->CASPASE+VMAP-M2+VMAP-C->MoxR->vWA+SWACOS*->
- EAD2+VMAP-M0+VMAP-C->MoxR->vWA+APATPase+wHTH+TPRs->||<-METALLOPROTEASE+TPRs<-FXSXX-COOH-like||EAD2+VMAP-M0+VMAP-C->MoxR->vWA+APATPase+wHTH+TPRs*->||<-METALLOPROTEASE+TPRs<-FXSXX-COOH-like
- EAD2+VMAP-M0+VMAP-C->MoxR->vWA+APATPase+wHTH+TPRs*->||<-METALLOPROTEASE+TPRs||EAD2+VMAP-M0+VMAP-C->MoxR->vWA+APATPase+wHTH+TPRs->||<-METALLOPROTEASE+TPRs<-FXSXX-COOH-like

■ **GCA_001735805.1; Streptomyces puniciscabiei; counts= 3; multicellularity status: TRUE**

- Trypco1->Trypsin+EAD2+VMAP-M0+VMAP-C->MoxR->vWA+Pkinase+ParA-Soj-PloopNTPase(SIMIBI)*->
- Trypsin+EAD2->EAD2+VMAP-M0+VMAP-C->MoxR->vWA+APATPase+wHTH+TPRs*->||<-METALLOPROTEASE+TPRs<-FXSXX-COOH-like||Trypco1->
- Trypsin+EAD2+VMAP-M0+VMAP-C->vWA+APATPase+wHTH+TPRs*->

■ **GCA_000010605.1; Streptomyces griseus subsp. griseus NBRC 13350; counts= 3; multicellularity status: TRUE**

- AB_hydrolase->CASPASE+VMAP-M2+VMAP-C->MoxR->vWA+TIR+FxsC_Cter*->
- AB_hydrolase->CASPASE+VMAP-M2+VMAP-C->MoxR->vWA+TPRs+TIR+FxsC_Cter(partial)*->
- Trypco1->Trypsin+EAD2+VMAP-M0+VMAP-C->MoxR->vWA+Pkinase+ParA-Soj-PloopNTPase(SIMIBI)*->||<-?<-?<-PSE||?->||<-?<-PSE<-?<-Trypco1

■ **GCA_000328705.1; Saccharothrix espanaensis DSM 44229; counts= 3; multicellularity status: TRUE**

- cNMP_cyclase+EAD2->EAD2+VMAP-M0+VMAP-C->MoxR->vWA+APATPase+wHTH+TPRs*->||<-METALLOPROTEASE+TPRs
- EAD2+VMAP-M0+VMAP-C->MoxR->vWA+APATPase+wHTH+TPRs*->||<-?<-?<-TCAD15
- EAD2+VMAP-M0+VMAP-C->MoxR->vWA+APATPase+wHTH+TPRs*->||<-METALLOPROTEASE+TPRs<-FXSXX-COOH-like<-cNMP_cyclase+EAD2

■ **GCA_000312185.1; Microcystis aeruginosa PCC 9443; counts= 3; multicellularity status: TRUE**

- CASPASE+VMAP-M14+VMAP-C->MoxR->vWA+FGS-fold_domain*->
- HTH+VMAP-M9+VMAP-C->MoxR->vWA+FGS-fold_domain*->?->?->FGS-fold_domain->
- Trypsin+VMAP-M1+VMAP-C->MoxR->vWA*->

■ **GCA_000826545.1; Kibdelosporangium sp. MJ126-NF4; counts= 3; multicellularity status: TRUE**

- AB_hydrolase->CASPASE+VMAP-M2+VMAP-C->MoxR->vWA+EAD2+VMAP-M0+VMAP-C*->
- EAD2+VMAP-M0+VMAP-C->MoxR->vWA+APATPase+wHTH+TPRs*->||<-METALLOPROTEASE+TPRs<-FXSXX-COOH-like||?->||<-?||EAD2+VMAP-M0+VMAP-C->MoxR->vWA+APATPase+wHTH+TPRs->
- vWA+APATPase+wHTH+TPRs->||<-METALLOPROTEASE+TPRs<-FXSXX-COOH-like||?->||<-?||EAD2+VMAP-M0+VMAP-C->MoxR->vWA+APATPase+wHTH+TPRs*->||<-METALLOPROTEASE+TPRs

■ **GCA_000177675.1; Frankia sp. EUN1f; counts= 3; multicellularity status: TRUE**

- EAD2+EAD1->EAD2+VMAP-M0+VMAP-C->MoxR->vWA+APATPase+wHTH+TPRs*->||<-METALLOPROTEASE+TPRs
- TIR+VMAP-M2+VMAP-C->MoxR->vWA+DO-GTPase2*->
- vWA+APATPase+wHTH+TPRs*->||<-METALLOPROTEASE+TPRs<-FXSXX-COOH-like

■ **GCA_000158935.1; Streptomyces lividans TK24; counts= 3; multicellularity status: TRUE**

- <-TCAD15||Trypco1->Trypsin+HDxxH+VMAP-M0+VMAP-C->MoxR->vWA+TPRs*->FXSXX-COOH->Radical_SAM->METALLOPROTEASE+TPRs->TIR+APATPase+TPRs->Trypco1->
- Trypco1->Trypsin+VMAP-M0+VMAP-C->MoxR->vWA->vWA+Pkinase+HTH+TPRs+cNMP_cyclase*->
- Trypco1->Trypsin+VMAP-M0+VMAP-C->MoxR->vWA*->vWA+Pkinase+HTH+TPRs+cNMP_cyclase->

■ **GCA_000158955.1; Streptomyces viridochromogenes DSM 40736; counts= 3; multicellularity status: TRUE**

- <-TCAD15||Trypco1->Trypsin+HDxxH+VMAP-M0+VMAP-C->MoxR->vWA+TPRs*->
- EAD2+VMAP-M0+VMAP-C->MoxR->vWA*->TIR+APATPase+TPRs->?->CASPASE+EAD2->METALLOPROTEASE+TPRs->
- Trypco1->Trypsin+VMAP-M0+VMAP-C->MoxR->vWA*->

■ **GCA_000316115.1; Leptolyngbya sp. PCC 7375; counts= 3; multicellularity status: TRUE**

- AB_hydrolase->CASPASE+EAD9->EAD9+VMAP-M1+VMAP-C->MoxR->vWA+FGS-fold_domain*->FGS-fold_domain->four_helix_bundle_protein->RVT_1->FGS-fold_domain->
- FGS-fold_domain->||<-?||PNP_UDP_1+VMAP-M1+VMAP-C->MoxR->vWA+Cas_csx3*->
- FGS-fold_domain->EAD1+Trypsin->?->EAD1+VMAP-M1+VMAP-C->MoxR->vWA+BetaPropeller+FGS-fold_domain*->FGS-fold_domain->||<-RVT_1<-four_helix_bundle_protein

■ **GCA_000412595.1; Microcystis aeruginosa SPC777; counts= 3; multicellularity status: TRUE**

- AB_hydrolase->CASPASE+VMAP-M14+VMAP-C->MoxR->vWA(partial)->vWA+FGS-fold_domain*->
- HTH+VMAP-M9+VMAP-C->MoxR->vWA+FGS-fold_domain*->
- MoxR->vWA(partial)->vWA+FGS-fold_domain*->

- **GCA_000414115.1; Streptomyces aurantiacus JA 4570; counts= 3; multicellularity status: TRUE**
  - Trypco1->Trypsin+EAD2+VMAP-M0+VMAP-C->MoxR->?->vWA+Pkinase+ParA-Soj-PloopNTPase(SIMIBI)*->
  - Trypco1->Trypsin+VMAP-M0+VMAP-C->MoxR->||<-?||vWA+Pkinase+wHTH+TPRs+cNMP_cyclase+HAD*->
  - vWA+APATPase+wHTH+TPRs*->||<-METALLOPROTEASE+TPRs<-FXSXX-COOH-like<-CASPASE+EAD2
- **GCA_000415505.1; Streptomyces afghaniensis 772; counts= 3; multicellularity status: TRUE**
  - <-TCAD15||Trypco1->Trypsin+HDxxH+VMAP-M0+VMAP-C->MoxR->vWA+TPRs*->
  - EAD2+VMAP-M0+VMAP-C->MoxR->vWA+EAD2+CASPASE+EAD2*->
  - Trypco1->Trypsin+VMAP-M0+VMAP-C->MoxR->?->vWA+cNMP_cyclase+Band7*->
- **GCA_000590515.1; Streptomyces sp. PRh5; counts= 3; multicellularity status: TRUE**
  - AB_hydrolase->CASPASE+EAD2+VMAP-M0+VMAP-C->MoxR->vWA+TPRs*->
  - AB_hydrolase->CASPASE+VMAP-M2+VMAP-C->MoxR->vWA+CASPASE+TCAD13*->
  - PNP_UDP_1+EAD2->EAD2+VMAP-M0+VMAP-C->MoxR->vWA+APATPase+wHTH+TPRs*->||<-METALLOPROTEASE+TPRs<-FXSXX-COOH-like
- **GCA_000829715.2; Streptomyces sp. NBRC 110027; counts= 3; multicellularity status: TRUE**
  - <-TCAD15||Trypco1->Trypsin+HDxxH+VMAP-M0+VMAP-C->MoxR->vWA+TPRs*->FXSXX-COOH->Radical_SAM->TIR+APATPase+TPRs->
  - Trypco1->Trypsin+EAD2+VMAP-M0+VMAP-C->MoxR->vWA+Pkinase+ParA-Soj-PloopNTPase(SIMIBI)*->
  - Trypco1->Trypsin+EAD2+VMAP-M0+VMAP-C->MoxR->vWA+wHTH+TPRs+APATPase+wHTH+TPRs*->||<-?||?->||<-AB_hydrolase
- **GCA_001270025.1; Streptomyces azureus; counts= 3; multicellularity status: TRUE**
  - EAD2+VMAP-M0+VMAP-C->MoxR->vWA+APATPase+wHTH+TPRs*->AB_hydrolase->CASPASE+EAD2->FXSXX-COOH-like->METALLOPROTEASE+TPRs->
  - MoxR->vWA+Pkinase+ParA-Soj-PloopNTPase(SIMIBI)*->
  - Trypco1->Trypsin+VMAP-M0+VMAP-C->MoxR->vWA+Pkinase+wHTH+TPRs+cNMP_cyclase+Band7+HAD*->
- **GCA_001949645.1; Streptomyces acidiscabies; counts= 3; multicellularity status: TRUE**
  - vWA+Pkinase+wHTH+cNMP_cyclase+Band7+HAD*->
  - vWA+TIR+FxsC_Cter(partial)*->
  - vWA+TPRs*->FXSXX-COOH->Radical_SAM->METALLOPROTEASE+TPRs->TIR+APATPase+TPRs->
- **GCA_000725125.1; Streptomyces toyocaensis; counts= 3; multicellularity status: TRUE**
  - EAD2+VMAP-M0+VMAP-C->MoxR->vWA+APATPase+wHTH+TPRs*->||<-Na_H_Exchanger||FXSXX-COOH-like->METALLOPROTEASE+TPRs->
  - Trypco1->Trypsin+EAD2+VMAP-M0+VMAP-C->MoxR->vWA+Pkinase+ParA-Soj-PloopNTPase(SIMIBI)*->
  - Trypco1->Trypsin+VMAP-M0+VMAP-C->MoxR->vWA+Pkinase+wHTH+TPRs+cNMP_cyclase+Band7+HAD*->
- **GCA_000966965.1; Streptomyces sp. NRRL S-104; counts= 3; multicellularity status: TRUE**
  - <-TCAD15||Trypsin+HDxxH+VMAP-M0+VMAP-C->MoxR->vWA+TPRs*->FXSXX-COOH->
  - AB_hydrolase->CASPASE+VMAP-M2+VMAP-C->MoxR->vWA+CASPASE+TCAD12*->
  - VMAP-M0+VMAP-C->MoxR->vWA+Trypsin+cNMP_cyclase+CCCH(TrebleClef)+Pentapeptide_repeats*->
- **GCA_001279445.1; Streptomyces sp. WM6349; counts= 3; multicellularity status: TRUE**
  - <-TCAD15||Trypsin+HDxxH+VMAP-M0+VMAP-C->MoxR->vWA+TPRs*->FXSXX-COOH->
  - AB_hydrolase->CASPASE+VMAP-M0+VMAP-C->MoxR->vWA+Trypsin+cNMP_cyclase+CCCH(TrebleClef)+Pentapeptide_repeats*->

- AB_hydrolase->CASPASE+VMAP-M2+VMAP-C->MoxR->vWA+CASPASE+TCAD12*->

■ **GCA_001279685.1; Streptomyces sp. WM6373; counts= 3; multicellularity status: TRUE**

- <-TCAD15||Trypsin+HDxxH+VMAP-M0+VMAP-C->MoxR->vWA+TPRs*->FXSXX-COOH->
- AB_hydrolase->CASPASE+VMAP-M0+VMAP-C->MoxR->vWA+Trypsin+cNMP_cyclase+CCCH(TrebleClef)+Pentapeptide_repeats*->
- AB_hydrolase->CASPASE+VMAP-M2+VMAP-C->MoxR->vWA+CASPASE+TCAD12*->

■ **GCA_001279905.1; Streptomyces sp. XY66; counts= 3; multicellularity status: TRUE**

- <-TCAD15||Trypsin+HDxxH+VMAP-M0+VMAP-C->MoxR->vWA+TPRs*->FXSXX-COOH->
- AB_hydrolase->CASPASE+VMAP-M0+VMAP-C->MoxR->vWA+Trypsin+cNMP_cyclase+CCCH(TrebleClef)+Pentapeptide_repeats*->
- AB_hydrolase->CASPASE+VMAP-M2+VMAP-C->MoxR->vWA+CASPASE+TCAD12*->

■ **GCA_001279485.1; Streptomyces sp. XY511; counts= 3; multicellularity status: TRUE**

- <-TCAD15||Trypsin+HDxxH+VMAP-M0+VMAP-C->MoxR->vWA+TPRs*->FXSXX-COOH->
- AB_hydrolase->CASPASE+VMAP-M0+VMAP-C->MoxR->vWA+Trypsin+cNMP_cyclase+CCCH(TrebleClef)+Pentapeptide_repeats*->
- AB_hydrolase->CASPASE+VMAP-M2+VMAP-C->MoxR->vWA+CASPASE+TCAD12*->

■ **GCA_001279765.1; Streptomyces sp. XY413; counts= 3; multicellularity status: TRUE**

- <-TCAD15||Trypsin+HDxxH+VMAP-M0+VMAP-C->MoxR->vWA+TPRs*->FXSXX-COOH->
- AB_hydrolase->CASPASE+VMAP-M0+VMAP-C->MoxR->vWA+Trypsin+cNMP_cyclase+CCCH(TrebleClef)+Pentapeptide_repeats*->
- AB_hydrolase->CASPASE+VMAP-M2+VMAP-C->MoxR->vWA+CASPASE+TCAD12*->

■ **GCA_001279495.1; Streptomyces sp. H021; counts= 3; multicellularity status: TRUE**

- <-TCAD15||Trypsin+HDxxH+VMAP-M0+VMAP-C->MoxR->vWA+TPRs*->FXSXX-COOH->METALLOPROTEASE+TPRs->TIR+APATPase+TPRs->
- AB_hydrolase->CASPASE+VMAP-M0+VMAP-C->MoxR->vWA+Trypsin+cNMP_cyclase+CCCH(TrebleClef)+Pentapeptide_repeats*->
- AB_hydrolase->CASPASE+VMAP-M2+VMAP-C->MoxR->vWA+CASPASE+TCAD12*->

■ **GCA_001298545.1; Actinobacteria bacterium OV320; counts= 3; multicellularity status: NA**

- <-TCAD15||Trypco1->Trypsin+HDxxH+VMAP-M0+VMAP-C->MoxR->vWA+TPRs*->FXSXX-COOH->Radical_SAM->METALLOPROTEASE+TPRs->TIR+APATPase+TPRs->
- DUF397->Trypco1->Trypsin+EAD2+VMAP-M0+VMAP-C->MoxR->vWA+Pkinase+ParA-Soj-PloopNTPase(SIMIBI)*->
- Trypco1->Trypsin+VMAP-M0+VMAP-C->MoxR->vWA+cNMP_cyclase+Band7+HAD*->

■ **GCA_001428885.1; Streptomyces sp. Root264; counts= 3; multicellularity status: TRUE**

- <-TCAD15||Trypco1->Trypsin+HDxxH+VMAP-M0+VMAP-C->MoxR->vWA+TPRs*->FXSXX-COOH->Radical_SAM->METALLOPROTEASE+TPRs->TIR+APATPase+TPRs->
- DUF397->MoxR->vWA+APATPase+wHTH+TPRs*->||<-vWA<-EAD2+VMAP-M0+VMAP-C
- EAD2+VMAP-M0+VMAP-C->vWA*->||<-vWA+APATPase+wHTH+TPRs<-MoxR<-DUF397

■ **GCA_001456025.1; Mastigocoleus testarum BC008; counts= 3; multicellularity status: TRUE**

- CASPASE+AB_hydrolase->CASPASE+VMAP-M28+VMAP-C->MoxR->vWA+CASPASE+FGS-fold_domain*->
- EAD1+VMAP-M1+VMAP-C->MoxR->MoxR->vWA_protein*->vWA(partial)->FGS-fold_domain->FGS-fold_domain->
- EAD1+VMAP-M1+VMAP-C->MoxR->vWA+Ntox3+MACRODOMAIN+FGS-fold_domain*->

■ **GCA_001509775.1; Streptomyces violaceusniger; counts= 3; multicellularity status: TRUE**

- AB_hydrolase->CASPASE+EAD2+VMAP-M0+VMAP-C->MoxR->vWA+TPRs*->

- PNP_UDP_1+EAD2->EAD2+VMAP-M0+VMAP-C->MoxR->vWA+APATPase+wHTH+TPRs*->||<-METALLOPROTEASE+TPRs
- SIR2->||<-?<-?||Trypco1->Trypsin+EAD2+VMAP-M0+VMAP-C->MoxR->vWA+Pkinase+ZNR+Pkinase+ParA-Soj-PloopNTPase(SIMIBI)*->Porphobil_deam+Porphobil_deamC->

■ **GCA_001514205.1; Streptomyces griseoruber; counts= 3; multicellularity status: TRUE**

- AB_hydrolase->CASPASE+EAD2+VMAP-M0+VMAP-C->MoxR->vWA+REase+cNMP_cyclase+CASPASE*->
- Trypco1->Trypsin+VMAP-M0+VMAP-C->MoxR->vWA+Pkinase+wHTH+TPRs+cNMP_cyclase+Band7+HAD*->
- Trypsin+EAD2+VMAP-M0+VMAP-C->vWA+APATPase+wHTH+TPRs*->KAP_NTPase->

■ **GCA_001514235.1; Streptomyces caeruleatus; counts= 3; multicellularity status: TRUE**

- AB_hydrolase->CASPASE+EAD2+VMAP-M0+VMAP-C->MoxR->vWA+TIR+FxsC_Cter(partial)*->
- TCAD15->Trypco1->Trypsin+HDxxH+VMAP-M0+VMAP-C->MoxR->vWA+TPRs*->Radical_SAM->TIR+APATPase+TPRs->
- Trypco1->Trypsin+EAD2+VMAP-M0+VMAP-C->MoxR->vWA+Pkinase+ParA-Soj-PloopNTPase(SIMIBI)*->

■ **GCA_001751305.1; Streptomyces nanshensis; counts= 3; multicellularity status: TRUE**

- AB_hydrolase->CASPASE+VMAP-M2+VMAP-C->MoxR->vWA->vWA*->
- AB_hydrolase->CASPASE+VMAP-M2+VMAP-C->MoxR->vWA*->vWA->
- CASPASE+VMAP-M2+VMAP-C->MoxR->PSE->PSE->vWA(partial)+REase+STAND_NTPase+TPRs+cNMP_cyclase*->

■ **GCA_001905005.1; Streptomyces sp. CB02923; counts= 3; multicellularity status: TRUE**

- <-TCAD15||Trypco1->Trypsin+HDxxH+VMAP-M0+VMAP-C->MoxR->vWA+TPRs*->FXSXX-COOH->Radical_SAM->TIR+APATPase+TPRs->
- Trypco1->Trypsin+EAD2+VMAP-M0+VMAP-C->MoxR->vWA+Pkinase+ParA-Soj-PloopNTPase(SIMIBI)*->?->NUDIX->
- Trypco1->Trypsin+EAD2+VMAP-M0+VMAP-C->MoxR->vWA+wHTH+TPRs+wHTH+TPRs+APATPase+wHTH+TPRs*->||<-AB_hydrolase

■ **GCA_000935135.2; Streptomyces sp. MNU77; counts= 3; multicellularity status: TRUE**

- <-PNP_UDP_1+EAD2||AB_hydrolase->CASPASE+VMAP-M2+VMAP-C->MoxR->vWA+TIR+FxsC_Cter*->
- AB_hydrolase->CASPASE+EAD2+VMAP-M0+VMAP-C->MoxR->vWA+TPRs*->
- EAD2+VMAP-M0+VMAP-C->MoxR->vWA+APATPase+wHTH+TPRs*->AB_hydrolase->CASPASE+EAD2->FXSXX-COOH-like->METALLOPROTEASE+TPRs->

■ **GCA_001969965.1; Streptomyces sparsogenes DSM 40356; counts= 3; multicellularity status: TRUE**

- AB_hydrolase->CASPASE+EAD2+VMAP-M0+VMAP-C->MoxR->vWA+TPRs*->
- PNP_UDP_1+EAD2->EAD2+VMAP-M0+VMAP-C->MoxR->vWA+APATPase+wHTH+TPRs*->||<-METALLOPROTEASE+TPRs<-FXSXX-COOH-like
- vWA+Pkinase+Pkinase*->Porphobil_deam+Porphobil_deamC->

■ **GCA_001983105.1; Frankia sp. M16386; counts= 3; multicellularity status: TRUE**

- EAD2+EAD1->EAD2+VMAP-M0+VMAP-C->MoxR->vWA+APATPase+wHTH+TPRs*->||<-METALLOPROTEASE+TPRs
- EAD2+VMAP-M0+VMAP-C->MoxR->vWA+APATPase+wHTH+TPRs*->TIR+APATPase+TPRs->||<-METALLOPROTEASE+TPRs
- Trypco1->Trypsin+HDxxH+VMAP-M0+VMAP-C->MoxR->vWA+TPRs*->Voltage_CLC->FXSXX-COOH-like->METALLOPROTEASE+TPRs->TIR+APATPase+TPRs->

■ **GCA_900090075.1; Streptomyces sp. MnatMP-M77; counts= 3; multicellularity status: TRUE**

- AB_hydrolase->CASPASE+VMAP-M2+VMAP-C->MoxR->vWA+TIR+FxsC_Cter*->
- AB_hydrolase->CASPASE+VMAP-M2+VMAP-C->MoxR->vWA+TPRs+TIR+FxsC_Cter(partial)*->
- Trypco1->Trypsin+EAD2+VMAP-M0+VMAP-C->MoxR->vWA+Pkinase+ParA-Soj-PloopNTPase(SIMIBI)*->

■ **GCA_900091955.1; Streptomyces sp. Ncost-T10-10d; counts= 3; multicellularity status: TRUE**

- AB_hydrolase->CASPASE+VMAP-M0+VMAP-C->MoxR->vWA+STAND_NTPase+BetaPropeller+REase(Mrr_cat-PDDEXK)*->
- Trypsin+EAD2+VMAP-M0+VMAP-C->MoxR->vWA+REase+SFI-helicase+DUF4729*->
- vWA+TPRs*->FXSXX-COOH->Radical_SAM->METALLOPROTEASE+TPRs->TIR+APATPase+TPRs->

■ **GCA_900090095.1; Streptomyces sp. SceaMP-e96; counts= 3; multicellularity status: TRUE**

- <-TCAD15||Trypco1->Trypsin+HDxxH+VMAP-M0+VMAP-C->MoxR->vWA+TPRs*->FXSXX-COOH->Radical_SAM->TIR+APATPase+TPRs->
- Trypco1->Trypsin+EAD2+VMAP-M0+VMAP-C->MoxR->vWA+Pkinase+ParA-Soj-PloopNTPase(SIMIBI)*->
- Trypco1->Trypsin+EAD2+VMAP-M0+VMAP-C->MoxR->vWA+wHTH+TPRs+APATPase+wHTH+TPRs*->?->?->||<-AB_hydrolase

■ **GCA_900105695.1; Streptomyces melanosporofaciens; counts= 3; multicellularity status: TRUE**

- AB_hydrolase->CASPASE+EAD2+VMAP-M0+VMAP-C->MoxR->vWA+TPRs*->
- PNP_UDP_1+EAD2->MoxR->vWA+APATPase+wHTH+TPRs*->||<-METALLOPROTEASE+TPRs<-FXSXX-COOH-like
- SIR2->||<-?<-?||Trypco1->Trypsin+EAD2+VMAP-M0+VMAP-C->MoxR->vWA+Pkinase+ZNR+Pkinase+ParA-Soj-PloopNTPase(SIMIBI)*->Porphobil_deam+Porphobil_deamC->

■ **GCA_900107925.1; Amycolatopsis pretoriensis; counts= 3; multicellularity status: TRUE**

- cNMP_cyclase+EAD2->EAD2+VMAP-M0+VMAP-C->vWA+APATPase+wHTH+TPRs->||<-METALLOPROTEASE+TPRs<-FXSXX-COOH-like||EAD2+VMAP-M0+VMAP-C->MoxR->vWA+APATPase+wHTH+TPRs*->||<-METALLOPROTEASE+TPRs<-FXSXX-COOH-like
- cNMP_cyclase+EAD2->EAD2+VMAP-M0+VMAP-C->vWA+APATPase+wHTH+TPRs*->||<-METALLOPROTEASE+TPRs<-FXSXX-COOH-like||EAD2+VMAP-M0+VMAP-C->MoxR->vWA+APATPase+wHTH+TPRs->||<-METALLOPROTEASE+TPRs<-FXSXX-COOH-like
- EAD2+VMAP-M0+VMAP-C->MoxR->vWA+APATPase+wHTH+TPRs*->||<-METALLOPROTEASE+TPRs<-FXSXX-COOH-like

■ **GCA_900107965.1; Streptomyces yanglinensis; counts= 3; multicellularity status: TRUE**

- <-TCAD15||Trypsin+HDxxH+VMAP-M0+VMAP-C->MoxR->vWA+TPRs*->FXSXX-COOH->Radical_SAM->METALLOPROTEASE+TPRs->TIR+APATPase+TPRs->Trypco1->
- PNP_UDP_1+EAD2->EAD2+VMAP-M0+VMAP-C->MoxR->vWA+APATPase+wHTH+TPRs*->||<-METALLOPROTEASE+TPRs<-FXSXX-COOH-like
- Trypco1->Trypsin+EAD2+VMAP-M0+VMAP-C->MoxR->vWA+Pkinase+ParA-Soj-PloopNTPase(SIMIBI)*->

■ **GCA_900112965.1; Streptomyces mirabilis; counts= 3; multicellularity status: TRUE**

- AB_hydrolase->CASPASE+VMAP-M2+VMAP-C->VMAP-C(partial)->MoxR->vWA+SFII-helicase+Z1*->
- ADP_ribosyl_GH->NUDIX->Trypco1->Trypsin+EAD2+VMAP-M0+VMAP-C->MoxR->vWA+Pkinase+ParA-Soj-PloopNTPase(SIMIBI)*->SIR2->
- EAD2+VMAP-M0+VMAP-C->vWA+APATPase+wHTH+TPRs*->||<-METALLOPROTEASE+TPRs<-Trypsin+EAD2<-Trypco2||METALLOPROTEASE+TPRs->

■ **GCA_900177255.1; Streptomyces sp. Amel2xC10; counts= 3; multicellularity status: TRUE**

- AB_hydrolase->CASPASE+EAD2+VMAP-M0+VMAP-C->MoxR->vWA+REase+cNMP_cyclase+CASPASE*->
- TCAD9+Pkinase+NACHT+HEAT_repeats->EAD2+VMAP-M0+VMAP-C->vWA*->MoxR->vWA+APATPase+TPRs->
- Trypco1->Trypsin+VMAP-M0+VMAP-C->MoxR->vWA+Pkinase+wHTH+TPRs+cNMP_cyclase+Band7+HAD*->

■ **GCA_000307995.2; Microcystis aeruginosa; counts= 3; multicellularity status: TRUE**

- AB_hydrolase->CASPASE+VMAP-M14+VMAP-C->MoxR->vWA+FGS-fold_domain*->
- HTH+VMAP-M9+VMAP-C->MoxR->vWA+FGS-fold_domain*->?->FGS-fold_domain->
- Trypco1->Trypsin+VMAP-M1+VMAP-C->MoxR->vWA+FGS-fold_domain*->?->PSE->||<-PSE||PSE->FGS-fold_domain->

■ **GCA_000312165.1; Microcystis aeruginosa; counts= 3; multicellularity status: TRUE**

- AB_hydrolase->CASPASE+EAD2->EAD2+VMAP-M11+VMAP-C->MoxR->vWA+FGS-fold_domain*->
- AB_hydrolase->CASPASE+VMAP-M14+VMAP-C->MoxR->vWA+FGS-fold_domain*->

- HTH+VMAP-M9+VMAP-C->MoxR->vWA+FGS-fold_domain*->?->?->?->PSE->FGS-fold_domain->

■ **GCA_000312225.1; Microcystis aeruginosa; counts= 3; multicellularity status: TRUE**

- AB_hydrolase->CASPASE+VMAP-M14+VMAP-C->MoxR->vWA+FGS-fold_domain*->
- HTH+VMAP-M9+VMAP-C->MoxR->vWA+FGS-fold_domain*->
- Trypsin+VMAP-M1+VMAP-C->MoxR->vWA+FGS-fold_domain*->FGS-fold_domain->

■ **GCA_000312265.1; Microcystis aeruginosa; counts= 3; multicellularity status: TRUE**

- CASPASE+EAD2->EAD2+VMAP-M11+VMAP-C->MoxR->vWA+FGS-fold_domain*->
- HTH+VMAP-M9+VMAP-C->MoxR->vWA+FGS-fold_domain*->?->?->FGS-fold_domain->
- Trypco1->Trypsin+VMAP-M1+VMAP-C->MoxR->vWA+FGS-fold_domain*->

■ **GCF_000242715.1; Streptomyces acidiscabies; counts= 3; multicellularity status: TRUE**

- AB_hydrolase->CASPASE+EAD2+VMAP-M0+VMAP-C->MoxR->vWA+TIR+FxsC_Cter(partial)*->
- Trypco1->Trypsin+EAD2+VMAP-M0+VMAP-C->MoxR->vWA+Pkinase+ParA-Soj-PloopNTPase(SIMIBI)*->
- Trypco1->Trypsin+VMAP-M0+VMAP-C->MoxR->vWA+Pkinase+wHTH+cNMP_cyclase+Band7+HAD*->

■ **GCA_000240165.1; Streptomyces cattleya; counts= 3; multicellularity status: TRUE**

- <-TCAD15||Trypco1->Trypsin+HDxxH+VMAP-M0+VMAP-C->MoxR->vWA+TPRs*->FXSXX-COOH->Radical_SAM->TIR+APATPase+TPRs->
- AB_hydrolase->CASPASE+EAD2+VMAP-M0+VMAP-C->MoxR->vWA+TPRs*->
- Trypco1->Trypsin+EAD2+VMAP-M0+VMAP-C->MoxR->vWA+Pkinase+ParA-Soj-PloopNTPase(SIMIBI)*->

■ **GCA_000312705.1; Anabaena sp. 90; counts= 3; multicellularity status: TRUE**

- <-CASPASE<-?||?->?->EAD10+VMAP-M1+VMAP-C->MoxR->vWA+TPRs*->
- vWA*->
- vWA+FGS-fold_domain*->

■ **GCF_000377965.1; Streptomyces sp. CNB091; counts= 3; multicellularity status: TRUE**

- CASPASE+VMAP-M2+VMAP-C->MoxR->vWA+HTH+HTH+ParA-Soj-PloopNTPase(SIMIBI)*->PSE->PSE->||<-?||?->||<-?||?->DUF397->
- EAD2+VMAP-M0+VMAP-C->MoxR->vWA+APATPase+wHTH+TPRs*->AB_hydrolase->CASPASE+EAD2->FXSXX-COOH-like->METALLOPROTEASE+TPRs->
- Trypco1->Trypsin+EAD2+VMAP-M0+VMAP-C->MoxR->vWA+Pkinase+ParA-Soj-PloopNTPase(SIMIBI)*->

■ **GCF_000331305.1; Calothrix sp. PCC 7103; counts= 3; multicellularity status: TRUE**

- EAD1+VMAP-M23+VMAP-C->MoxR->vWA+Glyco_trans_4_4+Glycos_transf_1*->
- EAD9+VMAP-M1+VMAP-C->MoxR->vWA+FGS-fold_domain*->FGS-fold_domain->EAD1+APATPase+TPRs->
- VMAP-M1+VMAP-C->MoxR->vWA+MNS+CASPASE+MNS*->

■ **GCF_000599945.1; Microcystis aeruginosa; counts= 3; multicellularity status: TRUE**

- AB_hydrolase->CASPASE+VMAP-M14+VMAP-C->MoxR->vWA+FGS-fold_domain*->
- MoxR->vWA+FGS-fold_domain*->
- vWA+FGS-fold_domain*->

■ **GCA_003947255.1; Amycolatopsis balhimycina; counts= 3; multicellularity status: TRUE**

- cNMP_cyclase+EAD2->EAD2+VMAP-M0+VMAP-C->MoxR->vWA+APATPase+wHTH+TPRs*->||<-METALLOPROTEASE+TPRs||cNMP_cyclase+EAD2->EAD2+VMAP-M0+VMAP-C->MoxR->TIR+APATPase+TPRs->||<-METALLOPROTEASE+TPRs

- vWA+APATPase+wHTH*->
- vWA+APATPase+wHTH+TPRs->||<-METALLOPROTEASE+TPRs||cNMP_cyclase+EAD2->EAD2+VMAP-M0+VMAP-C->MoxR->vWA+APATPase+wHTH+TPRs*->||<-METALLOPROTEASE+TPRs

■ **GCF_000383635.1; Streptomyces sp. 303MFCol5.2; counts= 3; multicellularity status: TRUE**

- <-TCAD15||Trypco1->Trypsin+HDxxH+VMAP-M0+VMAP-C->MoxR->vWA+TPRs*->FXSXX-COOH->Radical_SAM->METALLOPROTEASE+TPRs->TIR+APATPase+TPRs->
- DUF397->||<-?||Trypsin+EAD2+VMAP-M0+VMAP-C->MoxR->vWA+Pkinase+ParA-Soj-PloopNTPase(SIMIBI)*->
- Trypco1->Trypsin+VMAP-M0+VMAP-C->MoxR->vWA+cNMP_cyclase+Band7+HAD*->

■ **GCF_000717815.1; Streptomyces rimosus; counts= 3; multicellularity status: TRUE**

- <-FXSXX-COOH-like||Trypco1->Trypsin+HDxxH+VMAP-M0+VMAP-C->MoxR->vWA+TPRs*->FXSXX-COOH->Radical_SAM->TIR+APATPase+TPRs->
- Trypco1->Trypsin+EAD2+VMAP-M0+VMAP-C->MoxR->vWA+Pkinase+ParA-Soj-PloopNTPase(SIMIBI)*->?->NUDIX->
- Trypco1->Trypsin+EAD2+VMAP-M0+VMAP-C->MoxR->vWA+wHTH+TPRs+wHTH+TPRs+APATPase+wHTH+TPRs*->||<-AB_hydrolase

■ **GCF_000718675.1; Streptomyces rimosus; counts= 3; multicellularity status: TRUE**

- <-TCAD15||Trypco1->Trypsin+HDxxH+VMAP-M0+VMAP-C->MoxR->vWA+TPRs*->FXSXX-COOH->Radical_SAM->TIR+APATPase+TPRs->
- Trypco1->Trypsin+EAD2+VMAP-M0+VMAP-C->MoxR->vWA+Pkinase+ParA-Soj-PloopNTPase(SIMIBI)*->?->NUDIX->
- Trypco1->Trypsin+EAD2+VMAP-M0+VMAP-C->MoxR->vWA+wHTH+TPRs+wHTH+TPRs+APATPase+wHTH+TPRs*->||<-AB_hydrolase

■ **GCF_000721445.1; Streptomyces sp. NRRL F-5053; counts= 3; multicellularity status: TRUE**

- TCAD15->?->?->PSE->?->MoxR->vWA+Trypsin+HDxxH+VMAP-M0+VMAP-C->PSE->MoxR->vWA+TPRs*->FXSXX-COOH->METALLOPROTEASE+TPRs->TIR+APATPase+TPRs->
- TCAD15->?->?->PSE->?->MoxR->vWA+Trypsin+HDxxH+VMAP-M0+VMAP-C*->PSE->MoxR->vWA+TPRs->FXSXX-COOH->METALLOPROTEASE+TPRs->TIR+APATPase+TPRs->
- Trypco1->PSE->vWA+Pkinase+ParA-Soj-PloopNTPase(SIMIBI)*->

■ **GCF_000720675.1; Streptomyces sp. NRRL WC-3795; counts= 3; multicellularity status: TRUE**

- EAD2+VMAP-M0+VMAP-C->MoxR->vWA+APATPase+wHTH+TPRs*->||<-Na_H_Exchanger||AB_hydrolase->CASPASE+EAD2->FXSXX-COOH-like->METALLOPROTEASE+TPRs->
- Trypco1->Trypsin+EAD2+VMAP-M0+VMAP-C->MoxR->vWA+Pkinase+ParA-Soj-PloopNTPase(SIMIBI)*->
- Trypco1->Trypsin+VMAP-M0+VMAP-C->MoxR->vWA+Pkinase+wHTH+TPRs+cNMP_cyclase*->

■ **GCF_000716755.1; Saccharothrix syringae; counts= 3; multicellularity status: TRUE**

- EAD2+VMAP-M0+VMAP-C->MoxR->vWA+APATPase+wHTH+TPRs*->||<-METALLOPROTEASE+TPRs<-FXSXX-COOH-like||?->||<-?||EAD2+VMAP-M0+VMAP-C->MoxR->vWA+APATPase+wHTH+TPRs->
- EAD2+VMAP-M0+VMAP-C->MoxR->vWA+APATPase+wHTH+TPRs*->||<-TCAD15
- vWA+APATPase+wHTH+TPRs->||<-METALLOPROTEASE+TPRs<-FXSXX-COOH-like||?->||<-?||EAD2+VMAP-M0+VMAP-C->MoxR->vWA+APATPase+wHTH+TPRs*->||<-METALLOPROTEASE+TPRs

■ **GCF_000404005.1; Streptomyces sp. FXJ7.023; counts= 3; multicellularity status: TRUE**

- <-TCAD15||Trypco1->Trypsin+HDxxH+VMAP-M0+VMAP-C->MoxR->vWA+TPRs*->FXSXX-COOH->Radical_SAM->METALLOPROTEASE+TPRs->
- AB_hydrolase->CASPASE+EAD2+VMAP-M0+VMAP-C->MoxR->||<-?||vWA+TPRs*->||<-DUF397
- Trypco1->Trypsin+EAD2+VMAP-M0+VMAP-C->MoxR->vWA+Pkinase+ParA-Soj-PloopNTPase(SIMIBI)*->

■ **GCA_000938975.1; Streptomyces iranensis; counts= 3; multicellularity status: TRUE**

- AB_hydrolase->CASPASE+EAD2+VMAP-M0+VMAP-C->MoxR->vWA+TPRs*->
- PNP_UDP_1+EAD2->EAD2+VMAP-M0+VMAP-C->MoxR->vWA+APATPase+wHTH+TPRs*->||<-METALLOPROTEASE+TPRs
- SIR2->||<-?<-?||Trypco1->Trypsin+EAD2+VMAP-M0+VMAP-C->MoxR->vWA+ZNR+Pkinase+ParA-Soj-PloopNTPase(SIMIBI)*->Porphobil_deam+Porphobil_deamC->

■ **GCF_001189015.1; Streptomyces acidiscabies; counts= 3; multicellularity status: TRUE**

- <-TCAD15||PSE->Trypsin+HDxxH+VMAP-M0+VMAP-C->MoxR->vWA+TPRs*->FXSXX-COOH->Radical_SAM->METALLOPROTEASE+TPRs->TIR+APATPase+TPRs->
- Trypco1->Trypsin+VMAP-M0+VMAP-C->MoxR->vWA+Pkinase+wHTH+cNMP_cyclase+Band7+HAD*->
- Trypco2->Trypsin+EAD2+VMAP-M0+VMAP-C->MoxR->vWA+Pkinase+TPRs*->

■ **GCF_000717185.1; Kitasatospora phosalacinea; counts= 3; multicellularity status: TRUE**

- AB_hydrolase->CASPASE+VMAP-M2+VMAP-C->MoxR->vWA+CASPASE+TCAD6+cNMP_cyclase+Antibiotic_NAT*->
- Trypco1->Trypsin+EAD2+VMAP-M0+VMAP-C->MoxR->vWA+Coiled-coil+TIR+FxsC_Cter(partial)*->
- Trypco1->Trypsin+HDxxH+VMAP-M0+VMAP-C->MoxR->vWA+CASPASE+TCAD14+PIN+CCCH(ZNR)+FHA*->

■ **GCF_000720325.1; Streptomyces sp. NRRL B-1347; counts= 3; multicellularity status: TRUE**

- DUF397->||<-?||Trypco1->Trypsin+EAD2+VMAP-M0+VMAP-C->MoxR->vWA+Pkinase+ParA-Soj-PloopNTPase(SIMIBI)*->
- Trypco1->Trypsin+VMAP-M0+VMAP-C->MoxR->vWA+Pkinase+wHTH+TPRs+cNMP_cyclase+HAD*->
- Trypco1->Trypsin+VMAP-M8+VMAP-C->MoxR->vWA+APATPase+wHTH+TPRs*->

■ **GCF_001418335.1; Streptomyces aurantiacus; counts= 3; multicellularity status: TRUE**

- EAD2+VMAP-M0+VMAP-C->MoxR->vWA+APATPase+wHTH+TPRs*->FXSXX-COOH-like->METALLOPROTEASE+TPRs->AB_hydrolase->CASPASE+EAD2->
- Trypco1->Trypsin+EAD2+VMAP-M0+VMAP-C->MoxR->vWA+Pkinase+ParA-Soj-PloopNTPase(SIMIBI)*->
- Trypsin+EAD2->EAD2+VMAP-M0+VMAP-C->MoxR->vWA+APATPase+wHTH+TPRs*->||<-METALLOPROTEASE+TPRs||Trypco1->

■ **GCF_001434355.1; Streptomyces anulatus; counts= 3; multicellularity status: TRUE**

- AB_hydrolase->CASPASE+EAD2+VMAP-M0+VMAP-C->MoxR->vWA+TPRs*->
- EAD2+VMAP-M0+VMAP-C->MoxR->vWA+APATPase+wHTH+TPRs*->AB_hydrolase->CASPASE+EAD2->FXSXX-COOH-like->METALLOPROTEASE+TPRs->
- Trypco1->Trypsin+EAD2+VMAP-M0+VMAP-C->MoxR->vWA+Pkinase+ParA-Soj-PloopNTPase(SIMIBI)*->

■ **GCA_001578075.1; Microcystis aeruginosa; counts= 3; multicellularity status: TRUE**

- AB_hydrolase->CASPASE+VMAP-M14+VMAP-C->MoxR->vWA+FGS-fold_domain*->
- HTH+VMAP-M9+VMAP-C->MoxR->vWA+FGS-fold_domain*->?->?->FGS-fold_domain->
- Trypco1->Trypsin+VMAP-M1+VMAP-C->MoxR->vWA+FGS-fold_domain*->

■ **GCF_001552135.1; Actinomadura chibensis; counts= 3; multicellularity status: TRUE**

- AB_hydrolase->BTLCP+VMAP-M0+VMAP-C->MoxR->vWA+Integrin_beta(vWA)*->
- Radical_SAM->METALLOPROTEASE+TPRs->||<-TIR+APATPase+TPRs||FXSXX-COOH->?->EAD2+VMAP-M0+VMAP-C->MoxR->vWA+APATPase+wHTH+TPRs*->cNMP_cyclase+EAD2->
- Trypsin+EAD2+VMAP-M0+VMAP-C->MoxR->vWA+APATPase+wHTH*->?->||<-?<-?<-?||?->?->DUF397->

■ **GCF_001895325.1; Microcystis aeruginosa; counts= 3; multicellularity status: TRUE**

- AB_hydrolase->CASPASE+VMAP-M14+VMAP-C->MoxR->vWA+FGS-fold_domain*->
- HTH+VMAP-M9+VMAP-C->MoxR->vWA+FGS-fold_domain*->
- Trypco1->Trypsin+VMAP-M1+VMAP-C->MoxR->vWA+FGS-fold_domain*->

■ **GCF_001905525.1; Streptomyces sp. TSRI0395; counts= 3; multicellularity status: TRUE**

- AB_hydrolase->CASPASE+EAD2+VMAP-M0+VMAP-C->MoxR->vWA+TPRs*->
- EAD2+VMAP-M0+VMAP-C->MoxR->vWA+APATPase+wHTH+TPRs*->AB_hydrolase->CASPASE+EAD2->FXSXX-COOH-like->METALLOPROTEASE+TPRs->
- Trypco1->Trypsin+EAD2+VMAP-M0+VMAP-C->MoxR->vWA+Pkinase+ParA-Soj-PloopNTPase(SIMIBI)*->

- **GCF_002019855.1; Streptomyces antibioticus; counts= 3; multicellularity status: TRUE**
  - AB_hydrolase->CASPASE+EAD2+VMAP-M0+VMAP-C->MoxR->vWA+REase+cNMP_cyclase+CASPASE*->
  - EAD2+VMAP-M0+VMAP-C->vWA+APATPase+wHTH+TPRs*->||<-METALLOPROTEASE+TPRs<-FXSXX-COOH-like<-PSE||METALLOPROTEASE+TPRs->
  - TCAD9+Pkinase+NACHT+HEAT_repeats->EAD2+VMAP-M0+VMAP-C->vWA->MoxR->vWA+APATPase+TPRs*->
- **GCA_002021875.1; Streptomyces hygroscopicus; counts= 3; multicellularity status: TRUE**
  - AB_hydrolase->CASPASE+EAD2+VMAP-M0+VMAP-C->MoxR->vWA+TPRs*->
  - PNP_UDP_1+EAD2->EAD2+VMAP-M0+VMAP-C->MoxR->vWA+APATPase+wHTH+TPRs*->||<-METALLOPROTEASE+TPRs
  - SIR2->||<-?<-?||Trypco1->Trypsin+EAD2+VMAP-M0+VMAP-C->MoxR->vWA+Pkinase+ZNR+Pkinase+ParA-Soj-PloopNTPase(SIMIBI)*->Porphobil_deam+Porphobil_deamC->
- **GCA_000018105.1; Acaryochloris marina MBIC11017; counts= 2; multicellularity status: TRUE**
  - FGS-fold_domain->||<-?||PNP_UDP_1+VMAP-M1+VMAP-C->MoxR->vWA+VMAP-C+FGS-fold_domain*->FGS-fold_domain->||<-PSE||FGS-fold_domain->
  - Trypco1->Trypsin+VMAP-M1+VMAP-C->MoxR->vWA+FGS-fold_domain*->FGS-fold_domain->FGS-fold_domain->
- **GCA_000092985.1; Nocardiopsis dassonvillei subsp. dassonvillei DSM 43111; counts= 2; multicellularity status: TRUE**
  - Trypsin+EAD2->EAD2+VMAP-M0+VMAP-C->MoxR->vWA+APATPase+wHTH+TPRs*->||<-DUF397
  - Trypsin+VMAP-M0+VMAP-C->vWA+APATPase+wHTH+TPRs*->||<-DUF397||Trypco1->
- **GCA_000316665.1; Rivularia sp. PCC 7116; counts= 2; multicellularity status: TRUE**
  - EAD1+Trypsin->EAD1+VMAP-M1+VMAP-C->MoxR->vWA+FGS-fold_domain*->FGS-fold_domain->
  - EAD1+VMAP-M1+VMAP-C->MoxR->?->vWA+FGS-fold_domain*->FGS-fold_domain->
- **GCA_000317435.1; Calothrix sp. PCC 6303; counts= 2; multicellularity status: TRUE**
  - <-MoxR<-EAD10+VMAP-M1+VMAP-C<-CASPASE<-?||EAD10+VMAP-M1+VMAP-C->MoxR->vWA+MNS+CASPASE+MNS*->
  - <-vWA+MNS+CASPASE+MNS<-MoxR<-EAD10+VMAP-M1+VMAP-C||?->CASPASE->EAD10+VMAP-M1+VMAP-C->MoxR->vWA+AP-GTPase+COR*->
- **GCA_000400635.2; Amycolatopsis orientalis HCCB10007; counts= 2; multicellularity status: TRUE**
  - AB_hydrolase->CASPASE+VMAP-M0+VMAP-C->MoxR->vWA+GCV_H+HTH+cNMP_cyclase+cNMP_cyclase+TIR+CASPASE*->
  - VMAP-M0+VMAP-C->MoxR->vWA+APATPase+wHTH+TPRs*->||<-METALLOPROTEASE+TPRs<-FXSXX-COOH-like<-cNMP_binding+HTH_Crp_2
- **GCA_000739105.1; Streptomyces lividans TK24; counts= 2; multicellularity status: TRUE**
  - <-TCAD15||Trypco1->Trypsin+HDxxH+VMAP-M0+VMAP-C->MoxR->vWA+TPRs*->FXSXX-COOH->Radical_SAM->METALLOPROTEASE+TPRs->TIR+APATPase+TPRs->Trypco1->
  - Trypco1->Trypsin+VMAP-M0+VMAP-C->MoxR->vWA+Pkinase+wHTH+TPRs+cNMP_cyclase*->
- **GCA_000772045.1; Streptomyces sp. CCM_MD2014; counts= 2; multicellularity status: TRUE**
  - <-TCAD15||Trypco1->Trypsin+HDxxH+VMAP-M0+VMAP-C->MoxR->vWA+TPRs*->FXSXX-COOH->Radical_SAM->METALLOPROTEASE+TPRs->TIR+APATPase+TPRs->Trypco1->
  - EAD2+VMAP-M0+VMAP-C->vWA*->
- **GCA_001040905.1; Streptomyces sp. PBH53; counts= 2; multicellularity status: TRUE**
  - TCAD15->Trypco1->Trypsin+HDxxH+VMAP-M0+VMAP-C->MoxR->vWA+TPRs*->FXSXX-COOH-like->
  - Trypco1->Trypsin+VMAP-M0+VMAP-C->MoxR->vWA+wHTH+cNMP_cyclase+Band7+HAD*->
- **GCA_001278075.1; Streptomyces pristinaespiralis; counts= 2; multicellularity status: TRUE**
  - Trypco1->Trypsin+VMAP-M7+VMAP-C->MoxR->vWA+Tubulin*->

- VMAP-C->MoxR->vWA+Trypsin+cNMP_cyclase+CCCH(TrebleClef)+Pentapeptide_repeats*->

■ **GCA_001447075.1; Streptomyces hygroscopicus subsp. limoneus; counts= 2; multicellularity status: TRUE**

- TCAD15->Trypco1->Trypsin+HDxxH+VMAP-M0+VMAP-C->MoxR->vWA+TPRs*->FXSXX-COOH-like->METALLOPROTEASE+TPRs->TIR+APATPase+TPRs->
- Trypco1->Trypsin+VMAP-M0+VMAP-C->MoxR->vWA+Pkinase+cNMP_cyclase+Band7+HAD*->

■ **GCA_001484565.1; Streptomyces sp. CdTB01; counts= 2; multicellularity status: TRUE**

- <-TCAD15||Trypco1->Trypsin+HDxxH+VMAP-M0+VMAP-C->MoxR->vWA+TPRs*->FXSXX-COOH->Radical_SAM->METALLOPROTEASE+TPRs->TIR+APATPase+TPRs->
- Trypco1->Trypsin+VMAP-M0+VMAP-C->MoxR->vWA+cNMP_cyclase+Band7+HAD*->

■ **GCA_001611795.1; Streptomyces sp. S10(2016); counts= 2; multicellularity status: TRUE**

- EAD2+VMAP-M0+VMAP-C->MoxR->vWA+APATPase+wHTH+TPRs*->AB_hydrolase->CASPASE+EAD2->FXSXX-COOH-like->METALLOPROTEASE+TPRs->
- Trypco1->Trypsin+VMAP-M0+VMAP-C->MoxR->vWA+Pkinase+wHTH+TPRs+cNMP_cyclase+Band7+HAD*->

■ **GCA_001877055.1; Nocardiopsis dassonvillei; counts= 2; multicellularity status: TRUE**

- Trypsin+EAD2->EAD2+VMAP-M0+VMAP-C->MoxR->vWA+APATPase+wHTH+TPRs*->||<-DUF397
- Trypsin+VMAP-M0+VMAP-C->vWA+APATPase+wHTH+TPRs*->||<-DUF397||Trypco1->

■ **GCA_001941845.1; Streptomyces sp. TN58; counts= 2; multicellularity status: TRUE**

- <-TCAD15||Trypsin+HDxxH+VMAP-M0+VMAP-C->MoxR->vWA+TPRs*->FXSXX-COOH->Radical_SAM->METALLOPROTEASE+TPRs->TIR+APATPase+TPRs->
- AB_hydrolase->CASPASE+VMAP-M0+VMAP-C->MoxR->vWA+Trypsin+cNMP_cyclase+CCCH(TrebleClef)+Pentapeptide_repeats*->

■ **GCA_001983975.1; Streptomyces autolyticus; counts= 2; multicellularity status: TRUE**

- AB_hydrolase->CASPASE+EAD2+VMAP-M0+VMAP-C->MoxR->vWA+TPRs*->
- PNP_UDP_1+EAD2->?->EAD2+VMAP-M0+VMAP-C->MoxR->vWA+APATPase+wHTH+TPRs*->||<-METALLOPROTEASE+TPRs<-FXSXX-COOH-like

■ **GCA_002007685.1; Streptomyces sp. fd1-xmd; counts= 2; multicellularity status: TRUE**

- <-TCAD15||Trypsin+HDxxH+VMAP-M0+VMAP-C->MoxR->vWA+TPRs*->FXSXX-COOH->Radical_SAM->METALLOPROTEASE+TPRs->TIR+APATPase+TPRs->
- AB_hydrolase->CASPASE+VMAP-M0+VMAP-C->MoxR->vWA+Trypsin+cNMP_cyclase+CCCH(TrebleClef)+Pentapeptide_repeats*->

■ **GCA_002082195.1; Streptomyces gilvosporeus; counts= 2; multicellularity status: TRUE**

- EAD2+VMAP-M0+VMAP-C->vWA*->
- Trypco1->Trypsin+EAD2+VMAP-M0+VMAP-C->MoxR->vWA+APATPase+wHTH+TPRs*->

■ **GCA_002090335.1; Streptomyces sp. MOE7; counts= 2; multicellularity status: TRUE**

- <-TCAD15||Trypco1->PSE->MoxR->vWA+TPRs*->FXSXX-COOH->Radical_SAM->TIR+APATPase+TPRs->
- EAD2+VMAP-M0+VMAP-C->vWA(partial)->vWA*->

■ **GCA_002355495.1; Streptomyces laurentii; counts= 2; multicellularity status: TRUE**

- <-TCAD15||Trypco1->Trypsin+HDxxH+VMAP-M0+VMAP-C->MoxR->vWA+TPRs*->FXSXX-COOH->METALLOPROTEASE+TPRs->TIR+APATPase+TPRs->
- AB_hydrolase->CASPASE+VMAP-M2+VMAP-C->MoxR->vWA+APATPase+wHTH+TPRs+cNMP_cyclase+NaeI*->

■ **GCA_000091305.1; Streptomyces scabiei 87.22; counts= 2; multicellularity status: TRUE**

- TCAD9+Pkinase+NACHT+HEAT_repeats->EAD2+VMAP-M0+VMAP-C->vWA->MoxR->vWA+APATPase+TPRs*->

- TCAD9+Pkinase+NACHT+HEAT_repeats->EAD2+VMAP-M0+VMAP-C->vWA*->MoxR->vWA+APATPase+TPRs->

- **GCA_001013905.1; Streptomyces leeuwenhoekii; counts= 2; multicellularity status: TRUE**
  - <-TCAD15||Trypco1->Trypsin+HDxxH+VMAP-M0+VMAP-C->MoxR->vWA+TPRs*->FXSXX-COOH->Radical_SAM->METALLOPROTEASE+TPRs->TIR+APATPase+TPRs->
  - VMAP-M2+VMAP-C->MoxR->vWA+Pkinase+NUDIX*->
- **GCA_001406115.1; Streptomyces venezuelae; counts= 2; multicellularity status: TRUE**
  - AB_hydrolase->CASPASE+EAD2+VMAP-M0+VMAP-C->MoxR->vWA+TPRs*->
  - EAD2+VMAP-M0+VMAP-C->MoxR->vWA+APATPase+wHTH+TPRs*->||<-Na_H_Exchanger||AB_hydrolase->CASPASE+EAD2->FXSXX-COOH-like->METALLOPROTEASE+TPRs->
- **GCA_001511815.1; Streptomyces reticuli; counts= 2; multicellularity status: TRUE**
  - TCAD15->Trypco1->Trypsin+HDxxH+VMAP-M0+VMAP-C->MoxR->vWA+TPRs*->FXSXX-COOH-like->METALLOPROTEASE+TPRs->TIR+APATPase+TPRs->
  - Trypco1->Trypsin+VMAP-M0+VMAP-C->MoxR->vWA+wHTH+cNMP_cyclase+Band7+HAD*->
- **GCA_000169095.1; Lyngbya sp. PCC 8106; counts= 2; multicellularity status: TRUE**
  - EAD4+VMAP-M3+VMAP-C->MoxR->vWA+FGS-fold_domain*->FGS-fold_domain->
  - vWA+CASPASE*->
- **GCA_000158915.1; Streptomyces himastatinicus ATCC 53653; counts= 2; multicellularity status: TRUE**
  - EAD2+VMAP-M0+VMAP-C->MoxR->vWA+APATPase+wHTH+TPRs*->||<-METALLOPROTEASE+TPRs<-FXSXX-COOH-like
  - SIR2->||<-?<-?<-?<-PSE||Trypco1->PSE->MoxR->vWA+Pkinase+Pkinase+ParA-Soj-PloopNTPase(SIMIBI)*->Porphobil_deam+Porphobil_deamC->
- **GCA_000177175.2; Streptomyces sp. ACT-1; counts= 2; multicellularity status: TRUE**
  - AB_hydrolase->CASPASE+VMAP-M2+VMAP-C->MoxR->vWA+TIR+FxsC_Cter*->
  - AB_hydrolase->CASPASE+VMAP-M2+VMAP-C->MoxR->vWA+TPRs+TIR+FxsC_Cter(partial)*->
- **GCA_000239075.2; Streptomyces sp. W007; counts= 2; multicellularity status: TRUE**
  - <-TCAD15||Trypco1->Trypsin+HDxxH+VMAP-M0+VMAP-C->MoxR->vWA+TPRs*->Radical_SAM->TIR+APATPase+TPRs->
  - EAD2+VMAP-M0+VMAP-C->MoxR->vWA+APATPase+wHTH+TPRs*->AB_hydrolase->CASPASE+EAD2->METALLOPROTEASE+TPRs->
- **GCA_000297155.2; Streptomyces tsukubensis NRRL18488; counts= 2; multicellularity status: TRUE**
  - vWA+Pkinase*->
  - vWA+TPRs*->
- **GCA_000331185.1; Streptomyces rimosus subsp. rimosus ATCC 10970; counts= 2; multicellularity status: TRUE**
  - <-TCAD15||Trypco1->Trypsin+HDxxH+VMAP-M0+VMAP-C->MoxR->vWA*->
  - vWA+wHTH+TPRs+wHTH+TPRs+APATPase+wHTH+TPRs*->
- **GCA_000403665.1; Streptomyces lividans 1326; counts= 2; multicellularity status: TRUE**
  - <-TCAD15||Trypco1->Trypsin+HDxxH+VMAP-M0+VMAP-C->MoxR->vWA*->?->?->FXSXX-COOH->Radical_SAM->METALLOPROTEASE+TPRs->TIR+APATPase+TPRs->Trypco1->
  - Trypco1->Trypsin+VMAP-M0+VMAP-C->MoxR->vWA+Pkinase*->
- **GCA_000411315.1; Streptomyces sp. HGB0020; counts= 2; multicellularity status: TRUE**
  - <-TCAD15||Trypco1->Trypsin+HDxxH+VMAP-M0+VMAP-C->MoxR->vWA+TPRs*->FXSXX-COOH->Radical_SAM->METALLOPROTEASE+TPRs->TIR+APATPase+TPRs->

- Trypco1->Trypsin+VMAP-M0+VMAP-C->MoxR->vWA+cNMP_cyclase+Band7+HAD*->

■ **GCA_000497425.1; Streptomyces niveus NCIMB 11891; counts= 2; multicellularity status: TRUE**

- <-TCAD15||PSE->Trypsin+HDxxH+VMAP-M0+VMAP-C->MoxR->vWA+TPRs*->FXSXX-COOH->METALLOPROTEASE+TPRs->TIR+APATPase+TPRs->
- Trypco2->Trypsin+EAD2+VMAP-M0+VMAP-C->MoxR->vWA+Pkinase+TPRs*->

■ **GCA_000497445.1; Streptomyces roseochromogenus subsp. oscitans DS 12.976; counts= 2; multicellularity status: TRUE**

- EAD2+VMAP-M0+VMAP-C->MoxR->vWA+APATPase+wHTH+TPRs*->||<-METALLOPROTEASE+TPRs<-FXSXX-COOH-like
- TCAD15->Trypco1->Trypsin+HDxxH+VMAP-M0+VMAP-C->MoxR->vWA+TPRs*->PSE->PSE->TIR+APATPase+TPRs->

■ **GCA_000612545.1; Streptomyces sp. PCS3-D2; counts= 2; multicellularity status: TRUE**

- AB_hydrolase->CASPASE+VMAP-M0+VMAP-C->MoxR->vWA+Trypsin+cNMP_cyclase+CCCH(TrebleClef)+Pentapeptide_repeats*->
- AB_hydrolase->CASPASE+VMAP-M2+VMAP-C->MoxR->vWA+TIR+FxsC_Cter*->

■ **GCA_000955965.1; Streptomyces variegatus; counts= 2; multicellularity status: TRUE**

- EAD2+VMAP-M0+VMAP-C->MoxR->vWA+APATPase+wHTH+TPRs*->AB_hydrolase->CASPASE+EAD2->FXSXX-COOH-like->METALLOPROTEASE+TPRs->
- Trypco1->Trypsin+VMAP-M0+VMAP-C->MoxR->vWA+Pkinase+wHTH+TPRs+cNMP_cyclase+Band7+HAD*->

■ **GCA_001047335.1; Streptomyces regensis; counts= 2; multicellularity status: TRUE**

- Trypco1->Trypsin+HDxxH+VMAP-M0+VMAP-C->MoxR->vWA+TPRs*->FXSXX-COOH-like->METALLOPROTEASE+TPRs->TIR+APATPase+TPRs->
- Trypco1->Trypsin+VMAP-M0+VMAP-C->MoxR->vWA+Pkinase+cNMP_cyclase+Band7+HAD*->

■ **GCA_000972705.2; Limnoraphis robusta CS-951; counts= 2; multicellularity status: TRUE**

- FGS-fold_domain->||<-?<-?||EAD4+VMAP-M3+VMAP-C->MoxR->vWA_protein*->
- FGS-fold_domain->||<-?<-?||EAD4+VMAP-M3+VMAP-C->MoxR->vWA+FGS-fold_domain*->?->FGS-fold_domain->

■ **GCA_001189015.1; Streptomyces acidiscabies; counts= 2; multicellularity status: TRUE**

- <-TCAD15||Trypsin+HDxxH+VMAP-M0+VMAP-C->MoxR->vWA+TPRs*->Radical_SAM->METALLOPROTEASE+TPRs->TIR+APATPase+TPRs->
- Trypco1->Trypsin+VMAP-M0+VMAP-C->MoxR->?->vWA+Pkinase+wHTH+cNMP_cyclase+Band7+HAD*->

■ **GCA_001270515.1; Streptomyces viridochromogenes; counts= 2; multicellularity status: TRUE**

- EAD2+VMAP-M0+VMAP-C->MoxR->vWA+APATPase+wHTH+TPRs*->AB_hydrolase->CASPASE+EAD2->FXSXX-COOH-like->METALLOPROTEASE+TPRs->
- Trypco2->Trypsin+EAD2+VMAP-M0+VMAP-C->MoxR->vWA_protein*->

■ **GCA_001270565.1; Streptomyces virginiae; counts= 2; multicellularity status: TRUE**

- <-TCAD15||Trypsin+HDxxH+VMAP-M0+VMAP-C->MoxR->vWA+TPRs*->FXSXX-COOH->Radical_SAM->METALLOPROTEASE+TPRs->TIR+APATPase+TPRs->
- vWA+Trypsin+cNMP_cyclase+CCCH(TrebleClef)+Pentapeptide_repeats*->

■ **GCA_001270635.1; Streptomyces antibioticus; counts= 2; multicellularity status: TRUE**

- TCAD15->Trypco1->Trypsin+HDxxH+VMAP-M0+VMAP-C->MoxR->vWA+TPRs*->FXSXX-COOH-like->METALLOPROTEASE+TPRs->TIR+APATPase+TPRs->
- Trypco1->Trypsin+VMAP-M0+VMAP-C->MoxR->vWA+Pkinase+cNMP_cyclase+Band7+HAD*->

■ **GCA_001279065.1; Streptomyces rimosus subsp. rimosus; counts= 2; multicellularity status: TRUE**

- <-TCAD15||Trypco1->Trypsin+HDxxH+VMAP-M0+VMAP-C->MoxR->vWA+TPRs*->Radical_SAM->TIR+APATPase+TPRs->

- Trypco1->Trypsin+EAD2+VMAP-M0+VMAP-C->MoxR->vWA+wHTH+TPRs+wHTH+TPRs+APATPase+wHTH+TPRs*->||<-AB_hydrolase

■ **GCA_001279365.1; Streptomyces sp. NRRL F-5755; counts= 2; multicellularity status: TRUE**

- <-TCAD15||Trypco1->Trypsin+HDxxH+VMAP-M0+VMAP-C->MoxR->vWA+TPRs*->Radical_SAM->TIR+APATPase+TPRs->TIR+APATPase+TPRs->
- Trypco1->Trypsin+EAD2+VMAP-M0+VMAP-C->MoxR->vWA+wHTH+TPRs+wHTH+TPRs+APATPase+wHTH+TPRs*->||<-AB_hydrolase

■ **GCA_001279345.1; Streptomyces rimosus subsp. pseudoverticillatus; counts= 2; multicellularity status: TRUE**

- <-TCAD15||Trypco1->Trypsin+HDxxH+VMAP-M0+VMAP-C->MoxR->vWA+TPRs*->Radical_SAM->TIR+APATPase+TPRs->
- Trypco1->Trypsin+EAD2+VMAP-M0+VMAP-C->MoxR->vWA+wHTH+TPRs+wHTH+TPRs+APATPase+wHTH+TPRs*->||<-AB_hydrolase

■ **GCA_001279625.1; Streptomyces sp. WM6368; counts= 2; multicellularity status: TRUE**

- <-TCAD15||Trypsin+HDxxH+VMAP-M0+VMAP-C->MoxR->vWA+TPRs*->FXSXX-COOH->
- AB_hydrolase->CASPASE+VMAP-M0+VMAP-C->MoxR->vWA+Trypsin+cNMP_cyclase+CCCH(TrebleClef)+Pentapeptide_repeats*->

■ **GCA_001279775.1; Streptomyces sp. MMG1533; counts= 2; multicellularity status: TRUE**

- EAD2+VMAP-M0+VMAP-C->MoxR->vWA+APATPase+wHTH+TPRs*->||<-Na_H_Exchanger||AB_hydrolase->CASPASE+EAD2->FXSXX-COOH-like->METALLOPROTEASE+TPRs->
- vWA+APATPase+Trypsin+TIR+FxsC_Cter(partial)*->||<-DUF397

■ **GCA_001279935.1; Streptomyces sp. IGB124; counts= 2; multicellularity status: TRUE**

- <-TCAD15||Trypsin+HDxxH+VMAP-M0+VMAP-C->MoxR->vWA+TPRs*->FXSXX-COOH->
- AB_hydrolase->CASPASE+VMAP-M0+VMAP-C->MoxR->vWA+Trypsin+cNMP_cyclase+CCCH(TrebleClef)+Pentapeptide_repeats*->

■ **GCA_001279855.1; Streptomyces sp. XY58; counts= 2; multicellularity status: TRUE**

- <-TCAD15||Trypsin+HDxxH+VMAP-M0+VMAP-C->MoxR->vWA+TPRs*->FXSXX-COOH->
- AB_hydrolase->CASPASE+VMAP-M2+VMAP-C->MoxR->vWA+CASPASE+TCAD12*->

■ **GCA_001280045.1; Streptomyces sp. NRRL WC-3723; counts= 2; multicellularity status: TRUE**

- TCAD15->Trypco1->Trypsin+HDxxH+VMAP-M0+VMAP-C->MoxR->vWA+TPRs*->FXSXX-COOH-like->METALLOPROTEASE+TPRs->TIR+APATPase+TPRs->
- Trypco1->Trypsin+VMAP-M0+VMAP-C->MoxR->vWA+Pkinase+cNMP_cyclase+Band7+HAD*->

■ **GCA_001279555.1; Micromonospora sp. NRRL B-16802; counts= 2; multicellularity status: TRUE**

- CASPASE+TPRs->EAD2+VMAP-M0+VMAP-C->MoxR->vWA+APATPase+wHTH+TPRs*->||<-METALLOPROTEASE+TPRs<-Radical_SAM<-FXSXX-COOH-like
- EAD2+VMAP-M0+VMAP-C->vWA+APATPase+wHTH+TPRs*->||<-METALLOPROTEASE+TPRs

■ **GCA_001298555.1; Actinobacteria bacterium OV450; counts= 2; multicellularity status: NA**

- <-TCAD15||Trypsin+HDxxH+VMAP-M0+VMAP-C->MoxR->vWA+TPRs*->FXSXX-COOH->Radical_SAM->METALLOPROTEASE+TPRs->TIR+APATPase+TPRs->
- EAD2+VMAP-M0+VMAP-C->MoxR->vWA+APATPase+wHTH+TPRs*->||<-Na_H_Exchanger||AB_hydrolase->CASPASE+EAD2->METALLOPROTEASE+TPRs->

■ **GCA_001314865.1; Phormidesmis priestleyi Ana; counts= 2; multicellularity status: TRUE**

- EAD1+Trypsin->?->EAD1+VMAP-M1+VMAP-C->MoxR->vWA+FGS-fold_domain*->FGS-fold_domain->FGS-fold_domain->
- Trypco1->Trypsin+VMAP-M1+VMAP-C->MoxR->vWA+FGS-fold_domain*->FGS-fold_domain->||<-RVT_1<-four_helix_bundle_protein

■ **GCA_001514035.1; Streptomyces yokosukanensis; counts= 2; multicellularity status: TRUE**

- TCAD15->Trypco1->Trypsin+HDxxH+VMAP-M0+VMAP-C->MoxR->vWA+TPRs*->FXSXX-COOH->METALLOPROTEASE+TPRs->TIR+APATPase+TPRs->

- Trypco1->Trypsin+EAD2+VMAP-M0+VMAP-C->MoxR->vWA+Pkinase+ParA-Soj-PloopNTPase(SIMIBI)*->

■ **GCA_001514065.1; Streptomyces antibioticus; counts= 2; multicellularity status: TRUE**

- DRHyd+VMAP-M0+VMAP-C->MoxR->vWA+TIR+APATPase+wHTH+TPRs*->
- Trypco1->PSE->MoxR->vWA+Pkinase+wHTH+TPRs+cNMP_cyclase+Band7+HAD*->

■ **GCA_001514125.1; Streptomyces longwoodensis; counts= 2; multicellularity status: TRUE**

- <-TCAD15||Trypco1->Trypsin+HDxxH+VMAP-M0+VMAP-C->MoxR->vWA+TPRs*->FXSXX-COOH->Radical_SAM->METALLOPROTEASE+TPRs->TIR+APATPase+TPRs->
- Trypco1->Trypsin+VMAP-M0+VMAP-C->MoxR->vWA+Pkinase+wHTH+TPRs+cNMP_cyclase+Band7+HAD*->

■ **GCA_001595515.1; Streptomyces sp. CC71; counts= 2; multicellularity status: TRUE**

- Trypco1->MoxR->vWA+Pkinase+wHTH+TPRs+cNMP_cyclase*->
- vWA+APATPase+wHTH+TPRs*->||<-Na_H_Exchanger

■ **GCA_001854645.1; Frankia sp. EUN1h; counts= 2; multicellularity status: TRUE**

- EAD2+VMAP-M0+VMAP-C->MoxR->vWA+APATPase+wHTH+TPRs->||<-?||EAD2+VMAP-M0+VMAP-C->MoxR->vWA+APATPase+wHTH+TPRs*->||<-METALLOPROTEASE+TPRs
- EAD2+VMAP-M0+VMAP-C->MoxR->vWA+APATPase+wHTH+TPRs*->||<-?||EAD2+VMAP-M0+VMAP-C->MoxR->vWA+APATPase+wHTH+TPRs->||<-METALLOPROTEASE+TPRs

■ **GCA_000980885.2; Streptomyces malaysiense; counts= 2; multicellularity status: TRUE**

- Trypco1->Trypsin+VMAP-M0+VMAP-C->MoxR->vWA+Pkinase+cNMP_cyclase+Band7+HAD*->
- Trypco2->EAD2+VMAP-M0+VMAP-C->vWA+APATPase+wHTH+TPRs*->||<-PSE<-FXSXX-COOH-like<-Trypsin+EAD2

■ **GCA_001905065.1; Saccharothrix sp. CB00851; counts= 2; multicellularity status: TRUE**

- EAD2+VMAP-M0+VMAP-C->MoxR->vWA+APATPase+wHTH+TPRs*->||<-METALLOPROTEASE+TPRs<-FXSXX-COOH-like
- EAD2+VMAP-M0+VMAP-C->vWA+APATPase+wHTH+TPRs*->||<-METALLOPROTEASE+TPRs<-cNMP_cyclase+EAD2

■ **GCA_001905025.1; Streptomyces sp. CB03578; counts= 2; multicellularity status: TRUE**

- <-cNMP_binding+HTH_Crp_2<-?||EAD2+VMAP-M0+VMAP-C->MoxR->vWA+APATPase+wHTH+TPRs*->||<-Na_H_Exchanger||AB_hydrolase->CASPASE+EAD2->FXSXX-COOH-like->METALLOPROTEASE+TPRs->
- <-TCAD15||Trypsin+HDxxH+VMAP-M0+VMAP-C->MoxR->vWA+TPRs*->FXSXX-COOH->Radical_SAM->METALLOPROTEASE+TPRs->TIR+APATPase+TPRs->

■ **GCA_001905855.1; Streptomyces sp. CB01883; counts= 2; multicellularity status: TRUE**

- EAD2+VMAP-M0+VMAP-C->MoxR->vWA+APATPase+wHTH+TPRs*->AB_hydrolase->CASPASE+EAD2->FXSXX-COOH-like->METALLOPROTEASE+TPRs->
- Trypco1->Trypsin+VMAP-M0+VMAP-C->MoxR->vWA+wHTH+cNMP_cyclase+Band7+HAD*->

■ **GCA_001905725.1; Streptomyces sp. CB02400; counts= 2; multicellularity status: TRUE**

- EAD2+VMAP-M0+VMAP-C->MoxR->vWA+APATPase+wHTH+TPRs*->FXSXX-COOH-like->METALLOPROTEASE+TPRs->
- Trypsin+EAD2->EAD2+VMAP-M0+VMAP-C->MoxR->vWA+APATPase+wHTH+TPRs*->||<-METALLOPROTEASE+TPRs<-Radical_SAM<-FXSXX-COOH||Voltage_CLC->

■ **GCA_001953885.1; Streptomyces sp. IMTB 2501; counts= 2; multicellularity status: TRUE**

- EAD2+VMAP-M0+VMAP-C->MoxR->vWA+APATPase+wHTH+TPRs*->||<-METALLOPROTEASE+TPRs<-FXSXX-COOH-like
- TCAD15->Trypco1->Trypsin+HDxxH+VMAP-M0+VMAP-C->MoxR->vWA+TPRs*->Radical_SAM->TIR+APATPase+TPRs->

■ **GCA_001953875.1; Streptomyces amritsarensis; counts= 2; multicellularity status: TRUE**

- <-TCAD15||Trypsin+HDxxH+VMAP-M0+VMAP-C->MoxR->vWA+TPRs*->FXSXX-COOH->Radical_SAM->METALLOPROTEASE+TPRs->TIR+APATPase+TPRs->

  - AB_hydrolase->CASPASE+VMAP-M0+VMAP-C->MoxR->vWA+Trypsin+cNMP_cyclase+CCCH(TrebleClef)+Pentapeptide_repeats*->

- **GCA_002128465.1; Streptomyces pharetrae CZA14; counts= 2; multicellularity status: TRUE**
  - <-TCAD15||PSE->Trypsin+HDxxH+VMAP-M0+VMAP-C->MoxR->vWA+TIR+APATPase+Trypsin+HDxxH+VMAP-M0+VMAP-C*->
  - Trypco1->Trypsin+EAD2+VMAP-M0+VMAP-C->MoxR->vWA+Pkinase+ParA-Soj-PloopNTPase(SIMIBI)*->
- **GCA_900091975.1; Streptomyces sp. LamerLS-31b; counts= 2; multicellularity status: TRUE**
  - Trypco2->EAD2+VMAP-M0+VMAP-C->vWA+APATPase+wHTH+TPRs*->||<-METALLOPROTEASE+TPRs<-FXSXX-COOH-like<-Trypsin+EAD2<-HTH_31
  - VMAP-C(partial)->MoxR->vWA+Pkinase+cNMP_cyclase+Band7+HAD*->
- **GCA_900090105.1; Micromonospora humi; counts= 2; multicellularity status: TRUE**
  - EAD2+VMAP-M0+VMAP-C->MoxR->vWA+APATPase+wHTH+TPRs*->||<-METALLOPROTEASE+TPRs
  - Trypco1->Trypsin+HDxxH+VMAP-M0+VMAP-C->MoxR->vWA+Trypsin+HDxxH+VMAP-M0+VMAP-C*->?->||<-?<-?||?->DUF397->
- **GCA_900103455.1; Streptomyces wuyuanensis; counts= 2; multicellularity status: TRUE**
  - <-TCAD15||Trypco1->Trypsin+HDxxH+VMAP-M0+VMAP-C->MoxR->vWA+TPRs*->FXSXX-COOH->Radical_SAM->METALLOPROTEASE+TPRs->TIR+APATPase+TPRs->
  - ADP_ribosyl_GH->Trypco1->Trypsin+EAD2+VMAP-M0+VMAP-C->MoxR->vWA+Pkinase+FtsK+TPRs*->NUDIX->SIR2->
- **GCA_900103585.1; Streptomyces sp. cf386; counts= 2; multicellularity status: TRUE**
  - EAD2+VMAP-M0+VMAP-C->vWA+APATPase+wHTH+TPRs*->||<-METALLOPROTEASE+TPRs<-FXSXX-COOH-like<-Trypsin+EAD2<-Trypco2||FXSXX-COOH-like->METALLOPROTEASE+TPRs->
  - Trypsin+VMAP-M0+VMAP-C->MoxR->vWA+Pkinase+wHTH+TPRs+cNMP_cyclase+Band7+HAD*->
- **GCA_900104935.1; Streptomyces sp. 2114.2; counts= 2; multicellularity status: TRUE**
  - <-TCAD15||Trypco1->Trypsin+HDxxH+VMAP-M0+VMAP-C->MoxR->vWA+TPRs*->FXSXX-COOH->Radical_SAM->METALLOPROTEASE+TPRs->TIR+APATPase+TPRs->Trypco1->
  - Trypco1->Trypsin+VMAP-M0+VMAP-C->MoxR->vWA+Pkinase+wHTH+TPRs+cNMP_cyclase*->
- **GCA_900107045.1; Amycolatopsis xylanica; counts= 2; multicellularity status: TRUE**
  - <-cNMP_binding+HTH_Crp_2||cNMP_cyclase+EAD2->EAD2+VMAP-M0+VMAP-C->MoxR->vWA+APATPase+wHTH+TPRs*->||<-METALLOPROTEASE+TPRs
  - CASPASE+VMAP-M2+VMAP-C->MoxR->vWA+APATPase+wHTH+Pkinase*->
- **GCA_900105465.1; Streptomyces sp. PAN_FS17; counts= 2; multicellularity status: TRUE**
  - AB_hydrolase->CASPASE+EAD2+VMAP-M0+VMAP-C->MoxR->vWA_protein*->vWA+TIR+FxsC_Cter->
  - Trypsin+VMAP-M0+VMAP-C->MoxR->vWA+Pkinase+wHTH+TPRs+cNMP_cyclase+Band7+HAD*->
- **GCA_900105515.1; Streptomyces sp. 2131.1; counts= 2; multicellularity status: TRUE**
  - <-TCAD15||Trypco1->Trypsin+HDxxH+VMAP-M0+VMAP-C->MoxR->vWA+TPRs*->FXSXX-COOH->Radical_SAM->METALLOPROTEASE+TPRs->TIR+APATPase+TPRs->
  - Trypco1->Trypsin+HDxxH+VMAP-M0+VMAP-C->MoxR->vWA+CASPASE+NACHT+PIN*->
- **GCA_900105945.1; Amycolatopsis tolypomycina; counts= 2; multicellularity status: TRUE**
  - cNMP_cyclase+EAD2->EAD2+VMAP-M0+VMAP-C->MoxR->vWA+APATPase+wHTH+TPRs*->||<-METALLOPROTEASE+TPRs<-FXSXX-COOH-like
  - HTH+EAD2+VMAP-M0+VMAP-C->MoxR->vWA+APATPase+wHTH*->
- **GCA_900105245.1; Streptomyces sp. 1222.5; counts= 2; multicellularity status: TRUE**
  - EAD2+VMAP-M0+VMAP-C->MoxR->vWA+APATPase+wHTH+TPRs*->||<-Na_H_Exchanger||AB_hydrolase->CASPASE+EAD2->FXSXX-COOH-like->METALLOPROTEASE+TPRs->

- Trypco1->Trypsin+VMAP-M0+VMAP-C->MoxR->vWA+wHTH+cNMP_cyclase+Band7+HAD*->

■ **GCA_900105605.1; Streptomyces sp. 2231.1; counts= 2; multicellularity status: TRUE**

- EAD2+VMAP-M0+VMAP-C->MoxR->vWA+APATPase+wHTH+TPRs*->||<-Na_H_Exchanger||AB_hydrolase->CASPASE+EAD2->FXSXX-COOH-like->METALLOPROTEASE+TPRs->
- Trypco1->Trypsin+VMAP-M0+VMAP-C->MoxR->vWA+wHTH+cNMP_cyclase+Band7+HAD*->

■ **GCA_900105275.1; Streptomyces sp. KS_5; counts= 2; multicellularity status: TRUE**

- Trypsin+VMAP-M0+VMAP-C->MoxR->vWA+Pkinase+wHTH+TPRs+cNMP_cyclase+Band7+HAD*->
- vWA+APATPase+wHTH+TPRs*->?->||<-?<-?<-S1COLD

■ **GCA_900104815.1; Streptomyces misionensis; counts= 2; multicellularity status: TRUE**

- Trypco1->Trypsin+VMAP-M0+VMAP-C->MoxR->vWA+Pkinase+cNMP_cyclase+Band7+HAD*->
- Trypco2->EAD2+VMAP-M0+VMAP-C->vWA+APATPase+wHTH+TPRs*->||<-METALLOPROTEASE+TPRs<-FXSXX-COOH-like<-Trypsin+EAD2

■ **GCA_900105525.1; Streptomyces sp. 2314.4; counts= 2; multicellularity status: TRUE**

- <-TCAD15||Trypco1->Trypsin+HDxxH+VMAP-M0+VMAP-C->MoxR->vWA+TPRs*->FXSXX-COOH->Radical_SAM->TIR+APATPase+TPRs->
- Trypco1->Trypsin+EAD2+VMAP-M0+VMAP-C->MoxR->vWA+TPRs*->

■ **GCA_900108175.1; Actinomadura echinospora; counts= 2; multicellularity status: TRUE**

- Radical_SAM->METALLOPROTEASE+TPRs->||<-TIR+APATPase+TPRs||FXSXX-COOH-like->?->EAD2+VMAP-M0+VMAP-C->MoxR->vWA+APATPase+wHTH+TPRs*->||<-METALLOPROTEASE+TPRs<-Radical_SAM<-FXSXX-COOH
- Trypco1->Trypsin+VMAP-M8+VMAP-C->MoxR->vWA+BetaPropeller+SWACOS+STAND_NTPase*->

■ **GCA_900110755.1; Streptomyces sp. yr375; counts= 2; multicellularity status: TRUE**

- <-TCAD15||Trypco1->Trypsin+HDxxH+VMAP-M0+VMAP-C->MoxR->vWA+TPRs*->FXSXX-COOH->Radical_SAM->
- Trypco1->Trypsin+VMAP-M0+VMAP-C->MoxR->vWA+wHTH+cNMP_cyclase+Band7+HAD*->

■ **GCA_900115095.1; Actinomadura madurae; counts= 2; multicellularity status: TRUE**

- Radical_SAM->||<-TIR+APATPase+TPRs||?->SWACOS->?->EAD2+VMAP-M0+VMAP-C->MoxR->vWA+APATPase+wHTH+TPRs*->||<-Radical_SAM<-?||cNMP_cyclase+EAD2->
- Trypco1->Trypsin+VMAP-M8+VMAP-C->MoxR->vWA+FtsK+FtsK+FtsK*->

■ **GCA_900114955.1; Streptomyces sp. cf124; counts= 2; multicellularity status: TRUE**

- Trypco1->Trypsin+EAD2+VMAP-M0+VMAP-C->MoxR->vWA+APATPase+GGDEF+EAD2+VMAP-M0->EAD2+VMAP-M0+VMAP-C->MoxR->vWA+APATPase+wHTH+TPRs*->
- Trypco1->Trypsin+EAD2+VMAP-M0+VMAP-C->MoxR->vWA+APATPase+GGDEF+EAD2+VMAP-M0*->EAD2+VMAP-M0+VMAP-C->MoxR->vWA+APATPase+wHTH+TPRs->

■ **GCA_900115955.1; Lentzea waywayandensis; counts= 2; multicellularity status: TRUE**

- <-cNMP_binding+HTH_Crp_2||cNMP_cyclase+VMAP-M0+VMAP-C->MoxR->vWA+APATPase+wHTH+TPRs*->||<-METALLOPROTEASE+TPRs<-FXSXX-COOH-like
- AB_hydrolase->BTLCP+VMAP-M2+VMAP-C->MoxR->vWA+CASPASE+NACHT+TPRs*->

■ **GCA_900079405.3; Streptomyces sp. F-1; counts= 2; multicellularity status: TRUE**

- Trypco1->Trypsin+VMAP-M0+VMAP-C->MoxR->vWA+Pkinase+cNMP_cyclase+Band7+HAD*->
- Trypco2->tRNA->vWA+APATPase+wHTH+TPRs*->||<-METALLOPROTEASE+TPRs<-FXSXX-COOH-like<-Trypsin+EAD2

■ **GCA_000330925.1; Microcystis aeruginosa; counts= 2; multicellularity status: TRUE**

- HTH+VMAP-M9+VMAP-C->MoxR->vWA+FGS-fold_domain*->||<-?||?->?->PSE->FGS-fold_domain->

- Trypco1->Trypsin+VMAP-M1+VMAP-C->MoxR->vWA+FGS-fold_domain*->

■ **GCA_000312725.1; Microcystis aeruginosa; counts= 2; multicellularity status: TRUE**

- AB_hydrolase->CASPASE+VMAP-M14+VMAP-C->MoxR->vWA+FGS-fold_domain*->
- vWA+FGS-fold_domain*->

■ **GCA_000312245.1; Microcystis aeruginosa; counts= 2; multicellularity status: TRUE**

- AB_hydrolase->PSE->MoxR->vWA+FGS-fold_domain*->
- Trypsin+VMAP-M1+VMAP-C->MoxR->vWA+FGS-fold_domain*->

■ **GCA_000163875.1; Streptomyces clavuligerus; counts= 2; multicellularity status: TRUE**

- <-TCAD15||Trypco1->Trypsin+HDxxH+VMAP-M0+VMAP-C->MoxR->?->vWA+TPRs*->
- Trypco1->Trypsin+EAD2+VMAP-M0+VMAP-C->MoxR->vWA+wHTH+TPRs+wHTH+TPRs+APATPase+wHTH+TPRs*->

■ **GCA_000332625.1; Streptomyces viridochromogenes; counts= 2; multicellularity status: TRUE**

- Trypsin+EAD2->EAD2+VMAP-M0+VMAP-C->MoxR->vWA+APATPase+wHTH+TPRs*->||<-METALLOPROTEASE+TPRs<-FXSXX-COOH-like
- vWA+Pkinase+wHTH+TPRs+cNMP_cyclase+Band7+HAD*->

■ **GCA_000312285.1; Microcystis aeruginosa; counts= 2; multicellularity status: TRUE**

- AB_hydrolase->CASPASE+VMAP-M14+VMAP-C->MoxR->vWA+FGS-fold_domain*->
- HTH+VMAP-M9+VMAP-C->MoxR->vWA+FGS-fold_domain*->PSE->FGS-fold_domain->

■ **GCA_000280865.1; Streptomyces auratus; counts= 2; multicellularity status: TRUE**

- <-TCAD15||Trypco1->Trypsin+HDxxH+VMAP-M0+VMAP-C->MoxR->vWA+TPRs*->FXSXX-COOH->Radical_SAM->TIR+APATPase+TPRs->
- Trypco1->Trypsin+EAD2+VMAP-M0+VMAP-C->?->PSE->vWA+TPRs*->||<-AB_hydrolase

■ **GCA_000211815.1; Moorea producens; counts= 2; multicellularity status: TRUE**

- AB_hydrolase->CASPASE+VMAP-M14+VMAP-C->MoxR->vWA+FGS-fold_domain*->FGS-fold_domain->four_helix_bundle_protein->FGS-fold_domain->four_helix_bundle_protein->
- EAD1+Trypsin->EAD1+VMAP-M1+Calcineurin-like->EAD1+VMAP-M1+VMAP-C->MoxR->vWA+TPRs+CASPASE*->

■ **GCF_000332095.2; Leptolyngbya sp. PCC 6406; counts= 2; multicellularity status: TRUE**

- EAD1+Trypsin->?->EAD1+VMAP-C->MoxR->vWA+FGS-fold_domain*->four_helix_bundle_protein->PSE->?->RVT_1->FGS-fold_domain->
- FGS-fold_domain->||<-?||?->?->PNP_UDP_1+VMAP-M1+VMAP-C->MoxR->vWA+FGS-fold_domain*->||<-PSE||?->||<-?||?->FGS-fold_domain->FGS-fold_domain->

■ **GCA_900236475.1; Streptomyces chartreusis; counts= 2; multicellularity status: TRUE**

- EAD2+VMAP-M0+VMAP-C->MoxR->vWA+APATPase+wHTH+TPRs*->||<-Na_H_Exchanger||AB_hydrolase->CASPASE+EAD2->FXSXX-COOH-like->METALLOPROTEASE+TPRs->
- Trypco1->Trypsin+VMAP-M0+VMAP-C->MoxR->vWA+cNMP_cyclase+Band7+HAD*->

■ **GCF_000203835.1; Streptomyces coelicolor; counts= 2; multicellularity status: TRUE**

- <-TCAD15||Trypco1->Trypsin+HDxxH+VMAP-M0+VMAP-C->MoxR->vWA+TPRs*->FXSXX-COOH->Radical_SAM->METALLOPROTEASE+TPRs->TIR+APATPase+TPRs->Trypco1->
- Trypco1->Trypsin+VMAP-M0+VMAP-C->MoxR->vWA+Pkinase+wHTH+TPRs+cNMP_cyclase*->

■ **GCA_000010625.1; Microcystis aeruginosa; counts= 2; multicellularity status: TRUE**

- HTH+VMAP-M9+VMAP-C->VMAP-C(partial)->MoxR->vWA+FGS-fold_domain*->?->?->?->FGS-fold_domain->

- Trypco1->||<-?||Trypsin+VMAP-M1+VMAP-C->PSE->?->vWA+FGS-fold_domain*->

■ **GCA_000024025.1; Catenulispora acidiphila; counts= 2; multicellularity status: TRUE**

- AB_hydrolase->CASPASE+VMAP-M0+VMAP-C->MoxR->vWA+TPRs+CASPASE*->
- Trypsin+HDxxH+VMAP-M0+VMAP-C->MoxR->vWA+TPRs*->?->Radical_SAM->

■ **GCA_000177195.2; Streptomyces; counts= 2; multicellularity status: TRUE**

- AB_hydrolase->CASPASE+VMAP-M2+VMAP-C->MoxR->vWA+SFII-helicase+Z1*->
- EAD2+VMAP-M0+VMAP-C->MoxR->vWA+APATPase+wHTH+TPRs*->AB_hydrolase->CASPASE+EAD2->FXSXX-COOH-like->METALLOPROTEASE+TPRs->

■ **GCA_000147815.3; Streptomyces violaceusniger; counts= 2; multicellularity status: TRUE**

- ADP_ribosyl_GH->SIR2->||<-?<-?||Trypco1->Trypsin+EAD2+VMAP-M0+VMAP-C->MoxR->vWA+Pkinase+ZNR+Pkinase+ParA-Soj-PloopNTPase(SIMIBI)*->Porphobil_deam+Porphobil_deamC->
- PNP_UDP_1+EAD2->EAD2+VMAP-M0+VMAP-C->MoxR->vWA+APATPase+wHTH+TPRs*->||<-METALLOPROTEASE+TPRs

■ **GCA_000245355.1; Streptomyces hygroscopicus; counts= 2; multicellularity status: TRUE**

- TCAD15->Trypco1->Trypsin+HDxxH+VMAP-M0+VMAP-C->MoxR->vWA+TPRs*->FXSXX-COOH-like->METALLOPROTEASE+TPRs->TIR+APATPase+TPRs->
- Trypco1->Trypsin+VMAP-M0+VMAP-C->MoxR->vWA+Pkinase+cNMP_cyclase+Band7+HAD*->

■ **GCA_001886595.1; Streptomyces venezuelae; counts= 2; multicellularity status: TRUE**

- <-TCAD15||Trypco1->Trypsin+HDxxH+VMAP-M0+VMAP-C->MoxR->vWA+TPRs*->FXSXX-COOH->METALLOPROTEASE+TPRs->TIR+APATPase+TPRs->
- Trypsin+EAD2+VMAP-M0+VMAP-C->MoxR->vWA+wHTH+TPRs+wHTH+TPRs+APATPase+wHTH+TPRs*->

■ **GCF_000340945.1; Nocardiopsis synnemataformans; counts= 2; multicellularity status: TRUE**

- Trypsin+EAD2->EAD2+VMAP-M0+VMAP-C->MoxR->vWA+APATPase+wHTH+TPRs*->||<-DUF397
- Trypsin+VMAP-M0+VMAP-C->vWA+APATPase+wHTH+TPRs*->||<-DUF397||Trypco1->

■ **GCF_000341065.1; Nocardiopsis halotolerans; counts= 2; multicellularity status: TRUE**

- Trypsin+EAD2->EAD2+VMAP-M0+VMAP-C->MoxR->vWA+APATPase+wHTH+TPRs*->||<-DUF397
- Trypsin+VMAP-M0+VMAP-C->vWA+APATPase+wHTH+TPRs*->||<-DUF397||Trypco1->

■ **GCF_000341105.1; Nocardiopsis potens; counts= 2; multicellularity status: TRUE**

- Trypsin+EAD2+VMAP-M0+VMAP-C->MoxR->vWA+APATPase+wHTH+TPRs*->||<-DUF397
- Trypsin+VMAP-M0+VMAP-C->MoxR->vWA+APATPase+wHTH+TPRs*->||<-DUF397||Trypco1->

■ **GCF_000341145.1; Nocardiopsis xinjiangensis; counts= 2; multicellularity status: TRUE**

- Trypsin+EAD2->EAD2+VMAP-M0+VMAP-C->MoxR->vWA*->Trypco1->
- Trypsin+VMAP-M0+VMAP-C->vWA+APATPase+wHTH+TPRs*->||<-DUF397||Trypco1->

■ **GCF_000341025.1; Nocardiopsis salina; counts= 2; multicellularity status: TRUE**

- EAD2+VMAP-M0+VMAP-C->MoxR->vWA*->Trypco1->
- Trypsin+VMAP-M0+VMAP-C->vWA+APATPase+wHTH+TPRs*->||<-DUF397||Trypco1->

■ **GCA_001445835.1; Streptomyces vitaminophilus; counts= 2; multicellularity status: TRUE**

- AB_hydrolase->EAD2+VMAP-M0+VMAP-C->MoxR->vWA+APATPase+wHTH+TPRs*->||<-METALLOPROTEASE+TPRs<-CASPASE+EAD2

- Trypco1->Trypsin+EAD2+VMAP-M0+VMAP-C->MoxR->vWA+Pkinase+ParA-Soj-PloopNTPase(SIMIBI)*->

- **GCF_000373565.1; Streptomyces sp. HmicA12; counts= 2; multicellularity status: TRUE**
  - EAD2+VMAP-M0+VMAP-C->MoxR->vWA+APATPase+wHTH+TPRs*->AB_hydrolase->CASPASE+EAD2->FXSXX-COOH-like->METALLOPROTEASE+TPRs->
  - Trypco1->Trypsin+VMAP-M0+VMAP-C->MoxR->vWA+Pkinase+wHTH+TPRs+cNMP_cyclase+EAD2+Band7+HAD*->

- **GCF_000373625.1; Streptomyces sp. LaPpAH-108; counts= 2; multicellularity status: TRUE**
  - Trypco1->Trypsin+EAD2+VMAP-M0+VMAP-C->MoxR->vWA+Pkinase+ParA-Soj-PloopNTPase(SIMIBI)*->
  - Trypsin+EAD2->EAD2+VMAP-M0+VMAP-C->MoxR->vWA+APATPase+wHTH+TPRs*->||<-METALLOPROTEASE+TPRs<-FXSXX-COOH-like||Trypco1->

- **GCF_000377165.1; Streptomyces sp. CNY243; counts= 2; multicellularity status: TRUE**
  - <-TCAD15||Trypco1->Trypsin+HDxxH+VMAP-M0+VMAP-C->MoxR->vWA+TPRs*->FXSXX-COOH->Radical_SAM->TIR+APATPase+TPRs->
  - Trypco1->Trypsin+EAD2+VMAP-M0+VMAP-C->MoxR->vWA+Pkinase+ParA-Soj-PloopNTPase(SIMIBI)*->

- **GCF_000381685.1; Nocardiopsis sp. CNS-639; counts= 2; multicellularity status: TRUE**
  - Trypsin+EAD2->EAD2+VMAP-M0+VMAP-C->MoxR->vWA+APATPase+wHTH+TPRs*->||<-DUF397
  - Trypsin+VMAP-M0+VMAP-C->vWA+APATPase+wHTH+TPRs*->||<-DUF397||Trypco1->

- **GCF_000468475.2; Actinomadura madurae; counts= 2; multicellularity status: TRUE**
  - Radical_SAM->||<-TIR+APATPase+TPRs||SWACOS->?->EAD2+VMAP-M0+VMAP-C->MoxR->vWA+APATPase+wHTH+TPRs*->||<-Radical_SAM<-?||cNMP_cyclase+EAD2->||<-?<-?||cNMP_binding+HTH_Crp_2->
  - Trypco1->Trypsin+VMAP-M8+VMAP-C->MoxR->vWA+FtsK+FtsK+FtsK*->

- **GCF_000376565.1; Streptomyces hokutonensis; counts= 2; multicellularity status: TRUE**
  - TIR+EAD2->EAD2+VMAP-M10+VMAP-C->MoxR->vWA+APATPase+Trypsin+TIR+FxsC_Cter(partial)*->
  - Trypco1->Trypsin+EAD2+VMAP-M0+VMAP-C->MoxR->vWA+Pkinase+ParA-Soj-PloopNTPase(SIMIBI)*->

- **GCF_000377125.1; Streptomyces sp. CNS335; counts= 2; multicellularity status: TRUE**
  - <-TCAD15||Trypco1->Trypsin+HDxxH+VMAP-M0+VMAP-C->MoxR->vWA+TPRs*->FXSXX-COOH->Radical_SAM->TIR+APATPase+TPRs->
  - Trypco1->Trypsin+EAD2+VMAP-M0+VMAP-C->MoxR->vWA+Pkinase+ParA-Soj-PloopNTPase(SIMIBI)*->

- **GCF_000425065.1; Actinomadura rifamycini; counts= 2; multicellularity status: TRUE**
  - Radical_SAM->METALLOPROTEASE+TPRs->||<-TIR+APATPase+TPRs||?->?->EAD2+VMAP-M0+VMAP-C->MoxR->vWA+APATPase+wHTH+TPRs*->||<-FXSXX-COOH-like||cNMP_cyclase+EAD2->
  - Trypco1->Trypsin+VMAP-M8+VMAP-C->MoxR->vWA+FtsK+FtsK+cNMP_cyclase+FtsK*->

- **GCF_000424805.1; Streptomyces sp. CNQ865; counts= 2; multicellularity status: TRUE**
  - <-TCAD15||Trypco1->Trypsin+HDxxH+VMAP-M0+VMAP-C->MoxR->vWA+TPRs*->FXSXX-COOH->Radical_SAM->TIR+APATPase+TPRs->
  - Trypco1->Trypsin+EAD2+VMAP-M0+VMAP-C->MoxR->vWA+Pkinase+ParA-Soj-PloopNTPase(SIMIBI)*->

- **GCA_000707945.1; Streptomyces; counts= 2; multicellularity status: TRUE**
  - <-TCAD15||Trypco1->Trypsin+HDxxH+VMAP-M0+VMAP-C->MoxR->vWA+TPRs*->FXSXX-COOH->Radical_SAM->TIR+APATPase+TPRs->
  - Trypco1->Trypsin+EAD2+VMAP-M0+VMAP-C->MoxR->vWA+wHTH+TPRs+wHTH+TPRs+APATPase+wHTH+TPRs*->||<-AB_hydrolase

- **GCF_000717995.1; Streptomyces violaceorubidus; counts= 2; multicellularity status: TRUE**
  - TCAD15->Trypco1->Trypsin+HDxxH+VMAP-M0+VMAP-C->MoxR->vWA+TPRs*->FXSXX-COOH-like->METALLOPROTEASE+TPRs->TIR+APATPase+TPRs->

  - Trypco1->Trypsin+EAD2+VMAP-M0+VMAP-C->MoxR->vWA+Pkinase+ParA-Soj-PloopNTPase(SIMIBI)*->

- **GCF_000718785.1; Streptomyces sp. NRRL WC-3626; counts= 2; multicellularity status: TRUE**
  - EAD2+VMAP-M0+VMAP-C->MoxR->vWA+APATPase+wHTH+TPRs*->FXSXX-COOH-like->METALLOPROTEASE+TPRs->
  - Trypco1->Trypsin+VMAP-M0+VMAP-C->MoxR->vWA+Pkinase+wHTH+TPRs+cNMP_cyclase+Band7+HAD*->

- **GCF_000717925.1; Streptomyces sp. NRRL S-241; counts= 2; multicellularity status: TRUE**
  - <-TCAD15||Trypsin+HDxxH+VMAP-M0+VMAP-C->MoxR->vWA+TPRs*->FXSXX-COOH->?->Radical_SAM->
  - AB_hydrolase->CASPASE+VMAP-M0+VMAP-C->MoxR->vWA+Trypsin+cNMP_cyclase+CCCH(TrebleClef)+Pentapeptide_repeats*->

- **GCF_000718025.1; Streptomyces purpeofuscus; counts= 2; multicellularity status: TRUE**
  - AB_hydrolase->?->||<-?||MoxR->vWA+CASPASE+TCAD6+cNMP_cyclase+Antibiotic_NAT*->
  - Trypsin+EAD2->EAD2+VMAP-M10+VMAP-C->MoxR->vWA+APATPase+SWACOS+wHTH+TPRs+Beta-rich-tail*->

- **GCA_001270585.1; Streptomyces; counts= 2; multicellularity status: TRUE**
  - <-TCAD15||Trypco1->Trypsin+HDxxH+VMAP-M0+VMAP-C->MoxR->vWA+TPRs*->FXSXX-COOH->Radical_SAM->TIR+APATPase+TPRs->
  - Trypco1->Trypsin+EAD2+VMAP-M0+VMAP-C->MoxR->vWA+wHTH+TPRs+wHTH+TPRs+APATPase+wHTH+TPRs*->||<-AB_hydrolase

- **GCF_000716455.1; Streptomyces; counts= 2; multicellularity status: TRUE**
  - <-TCAD15||Trypco1->Trypsin+HDxxH+VMAP-M0+VMAP-C->MoxR->vWA+TPRs*->FXSXX-COOH->Radical_SAM->TIR+APATPase+TPRs->
  - Trypco1->Trypsin+EAD2+VMAP-M0+VMAP-C->MoxR->vWA+Pkinase+ParA-Soj-PloopNTPase(SIMIBI)*->?->NUDIX->

- **GCF_000720595.1; Streptomyces rimosus; counts= 2; multicellularity status: TRUE**
  - Trypco1->Trypsin+EAD2+VMAP-M0+VMAP-C->MoxR->vWA+Pkinase+ParA-Soj-PloopNTPase(SIMIBI)*->?->NUDIX->
  - Trypco1->Trypsin+EAD2+VMAP-M0+VMAP-C->MoxR->vWA+wHTH+TPRs+wHTH+TPRs+APATPase+wHTH+TPRs*->||<-AB_hydrolase

- **GCF_000720725.1; Streptomyces rimosus; counts= 2; multicellularity status: TRUE**
  - <-TCAD15||Trypco1->Trypsin+HDxxH+VMAP-M0+VMAP-C->MoxR->vWA+TPRs*->FXSXX-COOH->Radical_SAM->TIR+APATPase+TPRs->
  - Trypco1->Trypsin+EAD2+VMAP-M0+VMAP-C->MoxR->vWA+Pkinase+ParA-Soj-PloopNTPase(SIMIBI)*->?->NUDIX->

- **GCF_000718935.1; Streptomyces sp. NRRL S-237; counts= 2; multicellularity status: TRUE**
  - <-TCAD15||Trypsin+HDxxH+VMAP-M0+VMAP-C->MoxR->vWA+TPRs*->FXSXX-COOH->Radical_SAM->METALLOPROTEASE+TPRs->TIR+APATPase+TPRs->
  - AB_hydrolase->CASPASE+VMAP-M0+VMAP-C->MoxR->vWA+Trypsin+cNMP_cyclase+CCCH(TrebleClef)+Pentapeptide_repeats*->

- **GCF_000720115.1; Streptomyces sp. NRRL F-2580; counts= 2; multicellularity status: TRUE**
  - <-TCAD15||Trypsin+HDxxH+VMAP-M0+VMAP-C->MoxR->vWA+TPRs*->FXSXX-COOH->Radical_SAM->METALLOPROTEASE+TPRs->TIR+APATPase+TPRs->
  - AB_hydrolase->CASPASE+VMAP-M0+VMAP-C->MoxR->vWA+Trypsin+cNMP_cyclase+CCCH(TrebleClef)+Pentapeptide_repeats*->

- **GCF_000719345.1; Streptomyces sp. NRRL F-2664; counts= 2; multicellularity status: TRUE**
  - <-TCAD15||Trypsin+HDxxH+VMAP-M0+VMAP-C->MoxR->vWA+TPRs*->
  - AB_hydrolase->CASPASE+VMAP-M0+VMAP-C->MoxR->vWA+Trypsin+cNMP_cyclase+CCCH(TrebleClef)+Pentapeptide_repeats*->

- **GCF_000720795.1; Streptomyces; counts= 2; multicellularity status: TRUE**
  - <-TCAD15||Trypsin+HDxxH+VMAP-M0+VMAP-C->MoxR->vWA+TPRs*->FXSXX-COOH->Radical_SAM->METALLOPROTEASE+TPRs->TIR+APATPase+TPRs->

- AB_hydrolase->CASPASE+VMAP-M0+VMAP-C->MoxR->vWA+Trypsin+cNMP_cyclase+CCCH(TrebleClef)+Pentapeptide_repeats*->

■ **GCF_000721535.1; Streptomyces hygroscopicus; counts= 2; multicellularity status: TRUE**

- AB_hydrolase->CASPASE+EAD2+VMAP-M0+VMAP-C->MoxR->vWA+TPRs*->
- PNP_UDP_1+EAD2->EAD2+VMAP-M0+VMAP-C->MoxR->vWA+APATPase+wHTH+TPRs*->||<-METALLOPROTEASE+TPRs

■ **GCF_000720215.1; Streptomyces sp. NRRL S-475; counts= 2; multicellularity status: TRUE**

- <-TCAD15||Trypco1->Trypsin+HDxxH+VMAP-M0+VMAP-C->MoxR->vWA+TPRs*->Radical_SAM->TIR+APATPase+TPRs->
- Trypco1->Trypsin+VMAP-M0+VMAP-C->MoxR->vWA+cNMP_cyclase+Band7+HAD*->

■ **GCF_000719855.1; Streptomyces sp. NRRL F-4474; counts= 2; multicellularity status: TRUE**

- <-TCAD15||Trypsin+HDxxH+VMAP-M0+VMAP-C->MoxR->vWA+TPRs*->FXSXX-COOH->
- AB_hydrolase->CASPASE+VMAP-M0+VMAP-C->MoxR->vWA+Trypsin+cNMP_cyclase+CCCH(TrebleClef)+Pentapeptide_repeats*->

■ **GCF_000717845.1; Streptomyces violaceoruber; counts= 2; multicellularity status: TRUE**

- <-TCAD15||Trypco1->Trypsin+HDxxH+VMAP-M0+VMAP-C->MoxR->vWA+TPRs*->FXSXX-COOH->Radical_SAM->METALLOPROTEASE+TPRs->TIR+APATPase+TPRs->Trypco1->
- Trypco1->Trypsin+VMAP-M0+VMAP-C->MoxR->vWA+Pkinase+wHTH+TPRs+cNMP_cyclase*->

■ **GCF_000720455.1; Streptomyces virginiae; counts= 2; multicellularity status: TRUE**

- <-TCAD15||Trypsin+HDxxH+VMAP-M0+VMAP-C->MoxR->vWA+TPRs*->FXSXX-COOH->Radical_SAM->METALLOPROTEASE+TPRs->TIR+APATPase+TPRs->
- AB_hydrolase->CASPASE+VMAP-M0+VMAP-C->MoxR->vWA+Trypsin+cNMP_cyclase+CCCH(TrebleClef)+Pentapeptide_repeats*->

■ **GCF_000720805.1; Streptomyces violaceoruber; counts= 2; multicellularity status: TRUE**

- AB_hydrolase->CASPASE+VMAP-M0+VMAP-C->MoxR->vWA+Trypsin+cNMP_cyclase+CCCH(TrebleClef)+Pentapeptide_repeats+HAD*->
- EAD2+VMAP-M0+VMAP-C->MoxR->vWA+APATPase+wHTH+TPRs*->

■ **GCF_000720765.1; Streptomyces sp. NRRL S-646; counts= 2; multicellularity status: TRUE**

- <-TCAD15||Trypco1->Trypsin+HDxxH+VMAP-M0+VMAP-C->MoxR->vWA+TIR+APATPase+Trypsin+HDxxH+VMAP-M0+VMAP-C*->
- vWA+APATPase+wHTH+TPRs*->KAP_NTPase->

■ **GCF_000719605.1; Streptomyces sp. NRRL S-1813; counts= 2; multicellularity status: TRUE**

- <-TCAD15||Trypco1->Trypsin+HDxxH+VMAP-M0+VMAP-C->MoxR->vWA+TPRs*->FXSXX-COOH->Radical_SAM->TIR+APATPase+TPRs->
- AB_hydrolase->CASPASE+VMAP-M2+VMAP-C->MoxR->vWA+APATPase+wHTH+TPRs+SFI-helicase*->

■ **GCF_000721365.1; Streptomyces sp. NRRL F-5650; counts= 2; multicellularity status: TRUE**

- <-TCAD15||Trypco1->Trypsin+HDxxH+VMAP-M0+VMAP-C->MoxR->vWA+TPRs*->FXSXX-COOH->Radical_SAM->METALLOPROTEASE+TPRs->TIR+APATPase+TPRs->Trypco1->
- Trypco1->Trypsin+EAD2+VMAP-M0+VMAP-C->MoxR->vWA+Pkinase+ParA-Soj-PloopNTPase(SIMIBI)*->

■ **GCF_000725555.1; Streptomyces erythrochromogenes; counts= 2; multicellularity status: TRUE**

- <-TCAD15||Trypsin+HDxxH+VMAP-M0+VMAP-C->MoxR->vWA+TPRs*->FXSXX-COOH->?->Radical_SAM->METALLOPROTEASE+TPRs->TIR+APATPase+TPRs->
- AB_hydrolase->CASPASE+VMAP-M0+VMAP-C->MoxR->vWA+Trypsin+cNMP_cyclase+CCCH(TrebleClef)+Pentapeptide_repeats*->

■ **GCF_000725725.1; Streptomyces xanthophaeus; counts= 2; multicellularity status: TRUE**

- <-TCAD15||Trypsin+HDxxH+VMAP-M0+VMAP-C->MoxR->vWA+TPRs*->FXSXX-COOH->Radical_SAM->METALLOPROTEASE+TPRs->TIR+APATPase+TPRs->

- AB_hydrolase->CASPASE+VMAP-M0+VMAP-C->MoxR->vWA+Trypsin+cNMP_cyclase+CCCH(TrebleClef)+Pentapeptide_repeats*->

■ **GCF_000716515.1; Streptomyces rimosus; counts= 2; multicellularity status: TRUE**

- <-TCAD15||Trypco1->Trypsin+HDxxH+VMAP-M0+VMAP-C->MoxR->vWA+TPRs*->FXSXX-COOH->Radical_SAM->TIR+APATPase+TPRs->
- Trypco1->Trypsin+EAD2+VMAP-M0+VMAP-C->MoxR->vWA+wHTH+TPRs+wHTH+TPRs+APATPase+wHTH+TPRs*->||<-AB_hydrolase

■ **GCF_001984445.1; Streptomyces lydicus; counts= 2; multicellularity status: TRUE**

- <-TCAD15||Trypco1->Trypsin+HDxxH+VMAP-M0+VMAP-C->MoxR->vWA+TPRs*->FXSXX-COOH->Radical_SAM->TIR+APATPase+TPRs->
- EAD2+VMAP-M0+VMAP-C->vWA*->

■ **GCF_000725495.1; Streptomyces aureus; counts= 2; multicellularity status: TRUE**

- <-TCAD15||Trypco1->Trypsin+HDxxH+VMAP-M0+VMAP-C->MoxR->vWA+TPRs*->FXSXX-COOH->Radical_SAM->METALLOPROTEASE+TPRs->TIR+APATPase+TPRs->
- AB_hydrolase->CASPASE+VMAP-M18+VMAP-C->MoxR->vWA+DO-GTPase2*->

■ **GCF_000718315.1; Streptomyces griseofuscus; counts= 2; multicellularity status: TRUE**

- Trypco1->Trypsin+VMAP-M0+VMAP-C->MoxR->vWA+Pkinase+cNMP_cyclase+Band7+HAD*->
- Trypco2->EAD2+VMAP-M0+VMAP-C->vWA+APATPase+wHTH+TPRs*->||<-METALLOPROTEASE+TPRs<-Trypsin+EAD2<-HTH_31

■ **GCF_000720255.1; Streptomyces griseus; counts= 2; multicellularity status: TRUE**

- Trypco1->Trypsin+VMAP-M0+VMAP-C->MoxR->vWA+cNMP_cyclase+Band7+HAD*->
- Trypco2->?->EAD2+VMAP-M0+VMAP-C->vWA+APATPase+wHTH+TPRs*->||<-METALLOPROTEASE+TPRs<-Trypsin+EAD2

■ **GCF_000745675.1; Streptomyces atratus; counts= 2; multicellularity status: TRUE**

- <-TCAD15||Trypco1->Trypsin+HDxxH+VMAP-M0+VMAP-C->MoxR->vWA+TPRs*->FXSXX-COOH->Radical_SAM->METALLOPROTEASE+TPRs->TIR+APATPase+TPRs->
- EAD2+VMAP-M0+VMAP-C->MoxR->vWA+APATPase+wHTH+TPRs*->AB_hydrolase->CASPASE+EAD2->FXSXX-COOH-like->METALLOPROTEASE+TPRs->

■ **GCF_000725805.1; Streptomyces xanthophaeus; counts= 2; multicellularity status: TRUE**

- <-TCAD15||Trypsin+HDxxH+VMAP-M0+VMAP-C->MoxR->vWA+TPRs*->FXSXX-COOH->Radical_SAM->METALLOPROTEASE+TPRs->TIR+APATPase+TPRs->
- AB_hydrolase->CASPASE+VMAP-M0+VMAP-C->MoxR->vWA+Trypsin+cNMP_cyclase+CCCH(TrebleClef)+Pentapeptide_repeats*->

■ **GCF_000158915.1; Streptomyces himastatinicus; counts= 2; multicellularity status: TRUE**

- EAD2+VMAP-M0+VMAP-C->MoxR->vWA+APATPase+wHTH+TPRs*->||<-METALLOPROTEASE+TPRs<-FXSXX-COOH-like
- vWA+APATPase+wHTH+TPRs*->||<-METALLOPROTEASE+TPRs<-Radical_SAM<-FXSXX-COOH||?->?->||<-Trypco1

■ **GCA_000707965.1; Streptomyces rimosus; counts= 2; multicellularity status: TRUE**

- <-TCAD15||Trypco1->Trypsin+HDxxH+VMAP-M0+VMAP-C->MoxR->vWA+TPRs*->FXSXX-COOH->Radical_SAM->TIR+APATPase+TPRs->
- Trypco1->PSE->MoxR->vWA+wHTH+TPRs+wHTH+TPRs+APATPase+wHTH+TPRs*->||<-AB_hydrolase

■ **GCF_000710405.2; Kitasatospora sp. MBT66; counts= 2; multicellularity status: TRUE**

- AB_hydrolase->CASPASE+VMAP-M2+VMAP-C->MoxR->vWA+CASPASE+TCAD6+cNMP_cyclase+Antibiotic_NAT*->
- Trypsin+EAD2->EAD2+VMAP-M10+VMAP-C->MoxR->||<-?||vWA+APATPase+SWACOS+wHTH+TPRs+Beta-rich-tail*->

■ **GCF_000981225.1; Allosalinactinospora lopnorensis; counts= 2; multicellularity status: TRUE**

- <-Trypco1||Trypsin+EAD2->EAD2+VMAP-M0+VMAP-C->MoxR->vWA+APATPase+wHTH+TPRs*->||<-DUF397

- Trypsin+VMAP-M0+VMAP-C->MoxR->vWA+APATPase+wHTH+TPRs*->||<-DUF397||Trypco1->

■ **GCA_000974985.2; Streptomyces mangrovisoli; counts= 2; multicellularity status: TRUE**

- <-vWA+CASPASE+NACHT+PIN<-MoxR<-Trypsin+HDxxH+VMAP-M0+VMAP-C<-Trypco1||Trypco1->Trypsin+HDxxH+VMAP-M0+VMAP-C->MoxR->vWA+TPRs*->FXSXX-COOH->Radical_SAM->TIR+APATPase+TPRs->
- <-vWA+TPRs<-MoxR<-Trypsin+HDxxH+VMAP-M0+VMAP-C<-Trypco1||Trypco1->Trypsin+HDxxH+VMAP-M0+VMAP-C->MoxR->vWA+CASPASE+NACHT+PIN*->

■ **GCF_001005295.1; Streptomyces yangpuensis; counts= 2; multicellularity status: TRUE**

- <-TCAD15||Trypsin+HDxxH+VMAP-M0+VMAP-C->MoxR->vWA+TPRs*->FXSXX-COOH->PSE->PSE->METALLOPROTEASE+TPRs->TIR+APATPase+TPRs->
- AB_hydrolase->CASPASE+VMAP-M0+VMAP-C->MoxR->vWA+Trypsin+cNMP_cyclase+CCCH(TrebleClef)+Pentapeptide_repeats*->

■ **GCF_001047325.1; Streptomyces viridochromogenes; counts= 2; multicellularity status: TRUE**

- TCAD15->Trypco1->Trypsin+HDxxH+VMAP-M0+VMAP-C->MoxR->vWA+TPRs*->Radical_SAM->TIR+APATPase+TPRs->
- Trypco2->EAD2+VMAP-M0+VMAP-C->vWA+APATPase+wHTH+TPRs*->||<-METALLOPROTEASE+TPRs<-FXSXX-COOH-like<-Trypsin+EAD2

■ **GCF_000302615.1; Streptomyces sp. SS; counts= 2; multicellularity status: TRUE**

- EAD2+VMAP-M0+VMAP-C->MoxR->vWA+APATPase+wHTH+TPRs*->||<-Na_H_Exchanger||AB_hydrolase->CASPASE+EAD2->FXSXX-COOH-like->METALLOPROTEASE+TPRs->
- Trypco2->Trypsin+EAD2+VMAP-M0+VMAP-C->MoxR->vWA+TPRs*->AB_hydrolase->

■ **GCF_000424945.1; Streptomyces sp. 142MFCol3.1; counts= 2; multicellularity status: TRUE**

- <-TCAD15||Trypco1->Trypsin+HDxxH+VMAP-M0+VMAP-C->MoxR->vWA+TPRs*->FXSXX-COOH->Radical_SAM->METALLOPROTEASE+TPRs->TIR+APATPase+TPRs->
- Trypco1->Trypsin+VMAP-M0+VMAP-C->MoxR->vWA+Pkinase+wHTH+TPRs+cNMP_cyclase+Band7+HAD*->?->?->?->Radical_SAM->Antibiotic_NAT->TIR+FxsC_Cter->

■ **GCF_000717915.1; Streptomyces sp. NRRL S-244; counts= 2; multicellularity status: TRUE**

- <-TCAD15||Trypsin+HDxxH+VMAP-M0+VMAP-C->MoxR->vWA+TPRs*->FXSXX-COOH->PSE->METALLOPROTEASE+TPRs->TIR+APATPase+TPRs->
- Trypco1->Trypsin+EAD2+VMAP-M0+VMAP-C->MoxR->vWA+Pkinase+ParA-Soj-PloopNTPase(SIMIBI)*->

■ **GCF_000716595.1; Saccharothrix sp. NRRL B-16314; counts= 2; multicellularity status: TRUE**

- EAD2+VMAP-M0+VMAP-C->MoxR->vWA+APATPase+wHTH+TPRs*->?->||<-METALLOPROTEASE+TPRs
- EAD2+VMAP-M0+VMAP-C->vWA+APATPase+wHTH+TPRs*->||<-METALLOPROTEASE+TPRs<-cNMP_cyclase+EAD2

■ **GCF_001417675.1; Streptomyces; counts= 2; multicellularity status: TRUE**

- <-TCAD15||Trypco1->Trypsin+HDxxH+VMAP-M0+VMAP-C->MoxR->vWA+TPRs*->FXSXX-COOH->Radical_SAM->METALLOPROTEASE+TPRs->TIR+APATPase+TPRs->Trypco1->
- Trypco1->Trypsin+EAD2+VMAP-M0+VMAP-C->MoxR->vWA+Pkinase+ParA-Soj-PloopNTPase(SIMIBI)*->

■ **GCF_000720135.1; Streptomyces sp. NRRL S-1022; counts= 2; multicellularity status: TRUE**

- AB_hydrolase->CASPASE+EAD2+VMAP-M0+VMAP-C->MoxR->vWA+PNP_UDP_1+APATPase+wHTH+TPRs*->
- Trypco1->Trypsin+VMAP-M0+VMAP-C->MoxR->vWA+Pkinase+wHTH+TPRs*->

■ **GCF_000716675.1; Streptomyces; counts= 2; multicellularity status: TRUE**

- ADP_ribosyl_GH->Trypco1->Trypsin+EAD2+VMAP-M0+VMAP-C->PSE->PSE->vWA+Pkinase+FtsK+TPRs*->NUDIX->
- MoxR->vWA+Pkinase+wHTH+TPRs+cNMP_cyclase+HAD*->

■ **GCF_000717595.1; Streptomyces flavochromogenes; counts= 2; multicellularity status: TRUE**

- <-TCAD15||Trypco1->Trypsin+HDxxH+VMAP-M0+VMAP-C->MoxR->vWA+TPRs*->FXSXX-COOH->METALLOPROTEASE+TPRs->TIR+APATPase+TPRs->

- Trypco1->Trypsin+EAD2+VMAP-M0+VMAP-C->MoxR->vWA+Pkinase+ParA-Soj-PloopNTPase(SIMIBI)*->

■ **GCF_000717745.1; Streptomyces violens; counts= 2; multicellularity status: TRUE**

- <-TCAD15<-?||Trypsin+HDxxH+VMAP-M0+VMAP-C->MoxR->vWA+TPRs*->FXSXX-COOH->Radical_SAM->METALLOPROTEASE+TPRs->TIR+APATPase+TPRs->TIR+APATPase+TPRs->
- Trypco1->Trypsin+EAD2+VMAP-M0+VMAP-C->MoxR->vWA+SWACOS+wHTH*->

■ **GCF_000725475.1; Streptomyces durhamensis; counts= 2; multicellularity status: TRUE**

- Trypco1->Trypsin+EAD2+VMAP-M0+VMAP-C->MoxR->vWA+Pkinase+TIR+APATPase+wHTH+TPRs*->
- Trypco1->Trypsin+VMAP-M0+VMAP-C->MoxR->vWA+Pkinase+wHTH+TPRs+cNMP_cyclase+Band7+HAD*->

■ **GCF_000785985.1; Actinoplanes utahensis; counts= 2; multicellularity status: TRUE**

- AB_hydrolase->CASPASE+EAD2->VMAP-M0+VMAP-C->MoxR->vWA+CASPASE(partial)*->
- EAD2+VMAP-M0+VMAP-C->MoxR->vWA+cNMP_cyclase+HTH+HTH+TPRs*->

■ **GCF_000716335.1; Streptomyces sp. NRRL S-118; counts= 2; multicellularity status: TRUE**

- Trypco1->MoxR->vWA+NACHT+CASPASE+BetaPropeller*->
- Trypsin+EAD2+VMAP-M0+VMAP-C->MoxR->vWA+Pkinase+ParA-Soj-PloopNTPase(SIMIBI)*->

■ **GCF_000830005.1; Streptomyces vietnamensis; counts= 2; multicellularity status: TRUE**

- <-TCAD15||Trypco1->Trypsin+HDxxH+VMAP-M0+VMAP-C->MoxR->vWA+TPRs*->FXSXX-COOH->METALLOPROTEASE+TPRs->TIR+APATPase+TPRs->
- AB_hydrolase->CASPASE+VMAP-M2+VMAP-C->MoxR->vWA+REase+SWACOS+TPRs+cNMP_cyclase*->

■ **GCF_000956015.1; Streptomyces sp. NRRL F-4428; counts= 2; multicellularity status: TRUE**

- AB_hydrolase->CASPASE+VMAP-M0+VMAP-C->MoxR->vWA+Trypsin+cNMP_cyclase+CCCH(TrebleClef)+Pentapeptide_repeats*->
- Trypco2->Trypsin+EAD2+VMAP-M0+VMAP-C->MoxR->vWA+Pkinase+TPRs*->

■ **GCA_000952035.2; Streptomyces lydicus; counts= 2; multicellularity status: TRUE**

- <-TCAD15||Trypco1->Trypsin+HDxxH+VMAP-M0+VMAP-C->MoxR->vWA+TPRs*->FXSXX-COOH->Radical_SAM->TIR+APATPase+TPRs->
- Trypco1->Trypsin+EAD2+VMAP-M0+VMAP-C->MoxR->vWA+APATPase+wHTH+TPRs*->||<-AB_hydrolase

■ **GCF_000962825.1; Actinoplanes rectilineatus; counts= 2; multicellularity status: TRUE**

- TIR+EAD2->METALLOPROTEASE+TPRs->EAD2+VMAP-M0+VMAP-C->vWA+APATPase+wHTH+TPRs*->
- Trypco1->Trypsin+VMAP-M8+VMAP-C->MoxR->vWA+SWACOS+TPRs*->

■ **GCF_000718305.1; Streptomyces niger; counts= 2; multicellularity status: TRUE**

- <-TCAD15||Trypco1->Trypsin+HDxxH+VMAP-M0+VMAP-C->MoxR->vWA+TPRs*->FXSXX-COOH->Radical_SAM->METALLOPROTEASE+TPRs->TIR+APATPase+TPRs->Trypco1->
- Trypco1->Trypsin+EAD2+VMAP-M0+VMAP-C->MoxR->vWA+SWACOS+wHTH*->

■ **GCA_001267885.1; Streptomyces ambofaciens; counts= 2; multicellularity status: TRUE**

- EAD2+VMAP-M0+VMAP-C->MoxR->vWA+APATPase+wHTH+TPRs*->||<-Na_H_Exchanger||AB_hydrolase->CASPASE+EAD2->FXSXX-COOH-like->PSE->METALLOPROTEASE+TPRs->
- Trypco1->Trypsin+VMAP-M0+VMAP-C->?->MoxR->vWA+Pkinase+wHTH+TPRs+cNMP_cyclase+HAD*->

■ **GCF_001279805.1; Streptomyces sp. XY431; counts= 2; multicellularity status: TRUE**

- AB_hydrolase->CASPASE+EAD2+VMAP-C->MoxR->vWA+TPRs*->

- vWA+APATPase+SWACOS+wHTH+TPRs+Beta-rich-tail*->

■ **GCF_001279615.1; Streptomyces sp. NRRL F-7442; counts= 2; multicellularity status: TRUE**

- <-TCAD15||Trypco1->Trypsin+HDxxH+VMAP-M0+VMAP-C->MoxR->vWA+TPRs*->FXSXX-COOH->Radical_SAM->METALLOPROTEASE+TPRs->TIR+APATPase+TPRs->Trypco1->PSE->?->CASPASE->
- Trypco1->Trypsin+EAD2+VMAP-M0+VMAP-C->MoxR->vWA+Pkinase+ParA-Soj-PloopNTPase(SIMIBI)*->

■ **GCF_001280095.1; Streptomyces sp. NRRL F-6491; counts= 2; multicellularity status: TRUE**

- <-TCAD15||PSE->Trypsin+HDxxH+VMAP-M0+VMAP-C->MoxR->vWA+TPRs*->FXSXX-COOH->METALLOPROTEASE+TPRs->TIR+APATPase+TPRs->
- AB_hydrolase->CASPASE+VMAP-M2+VMAP-C->MoxR->vWA+SFII-helicase+Trypsin+cNMP_cyclase+Coiled-coil+CASPASE*->

■ **GCA_001279885.1; Streptomyces sp. XY593; counts= 2; multicellularity status: TRUE**

- AB_hydrolase->CASPASE+VMAP-M0+VMAP-C->MoxR->vWA+Trypsin+cNMP_cyclase+CCCH(TrebleClef)+Pentapeptide_repeats*->
- AB_hydrolase->CASPASE+VMAP-M2+VMAP-C->MoxR->vWA+CASPASE+TCAD12*->

■ **GCA_001279525.1; Nocardia sp. NRRL S-836; counts= 2; multicellularity status: TRUE**

- <-cNMP_binding+HTH_Crp_2||cNMP_cyclase+EAD2+VMAP-M0+VMAP-C->MoxR->vWA+APATPase+wHTH+TPRs*->||<-METALLOPROTEASE+TPRs<-FXSXX-COOH-like
- AB_hydrolase->BTLCP+VMAP-M2+VMAP-C->MoxR->vWA+CASPASE+APATPase+TPRs*->

■ **GCA_002005225.1; Streptomyces pactum; counts= 2; multicellularity status: TRUE**

- EAD2+VMAP-M0+VMAP-C->MoxR->vWA+APATPase+wHTH+TPRs*->
- EAD2+VMAP-M0+VMAP-C->MoxR->vWA+APATPase+wHTH+TPRs*->||<-Na_H_Exchanger||AB_hydrolase->CASPASE+EAD2->FXSXX-COOH-like->METALLOPROTEASE+TPRs->

■ **GCF_001417775.1; Streptomyces sp. TP-A0356; counts= 2; multicellularity status: TRUE**

- Trypsin+EAD2->EAD2+VMAP-M0+VMAP-C->MoxR->vWA+APATPase+wHTH+TPRs*->||<-METALLOPROTEASE+TPRs<-Radical_SAM<-FXSXX-COOH||Voltage_CLC->
- Trypsin+VMAP-M6+VMAP-C->MoxR->vWA+APATPase+wHTH+TPRs*->||<-METALLOPROTEASE+TPRs<-Radical_SAM<-FXSXX-COOH-like<-Trypco1

■ **GCF_001417735.1; Streptomyces sp. NBRC 110028; counts= 2; multicellularity status: TRUE**

- AB_hydrolase->CASPASE+EAD2+VMAP-M0+VMAP-C->MoxR->vWA+TPRs*->
- PNP_UDP_1+EAD2->EAD2+VMAP-M0+VMAP-C->MoxR->vWA+APATPase+wHTH+TPRs*->||<-METALLOPROTEASE+TPRs

■ **GCF_001418135.1; Streptomyces aureus; counts= 2; multicellularity status: TRUE**

- vWA+Pkinase+TPRs*->
- vWA+TPRs*->FXSXX-COOH->METALLOPROTEASE+TPRs->

■ **GCF_001418655.1; Streptomyces phaeochromogenes; counts= 2; multicellularity status: TRUE**

- Trypsin+EAD2->EAD2+VMAP-M0+VMAP-C->MoxR->vWA+APATPase+wHTH+TPRs*->||<-METALLOPROTEASE+TPRs<-FXSXX-COOH-like||Trypco1->
- vWA+Pkinase+wHTH+TPRs+cNMP_cyclase+Band7+HAD*->

■ **GCA_001443625.1; Streptomyces venezuelae; counts= 2; multicellularity status: TRUE**

- AB_hydrolase->CASPASE+EAD2+VMAP-M0+VMAP-C->MoxR->vWA+TPRs*->
- EAD2+VMAP-M0+VMAP-C->MoxR->vWA+APATPase+wHTH+TPRs*->||<-Na_H_Exchanger||AB_hydrolase->CASPASE+EAD2->FXSXX-COOH-like->METALLOPROTEASE+TPRs->

■ **GCF_001419685.1; Streptomyces puniciscabiei; counts= 2; multicellularity status: TRUE**

- Trypco1->Trypsin+EAD2+VMAP-M0+VMAP-C->MoxR->vWA+DNA-primase-like_ZnR+S1+KH+KH*->

- Trypco2->EAD2+VMAP-M0+VMAP-C->vWA+APATPase+wHTH+TPRs*->||<-METALLOPROTEASE+TPRs<-Trypsin+EAD2

■ **GCA_001426325.1; Streptomyces; counts= 2; multicellularity status: TRUE**

- AB_hydrolase->CASPASE+EAD2+VMAP-M0+VMAP-C->MoxR->vWA+TPRs*->
- Trypco1->Trypsin+EAD2+VMAP-M0+VMAP-C->MoxR->vWA+Pkinase+ParA-Soj-PloopNTPase(SIMIBI)*->

■ **GCA_001424875.1; Kitasatospora sp. Root107; counts= 2; multicellularity status: TRUE**

- Trypco1->Trypsin+EAD2->EAD2+VMAP-M10+VMAP-C->MoxR->vWA+APATPase+BetaPropeller+Pkinase+wHTH+TPRs+wHTH+TPRs+FtsK*->
- Trypco1->Trypsin+VMAP-M0+VMAP-C->MoxR->vWA+CASPASE+TIR+FxsC_Cter(partial)*->

■ **GCA_001484705.1; Streptomyces sp. 4F; counts= 2; multicellularity status: TRUE**

- EAD2+VMAP-M0+VMAP-C->vWA*->
- EAD2+VMAP-M0+VMAP-C->vWA*->

■ **GCA_001514055.1; Streptomyces corchorusii; counts= 2; multicellularity status: TRUE**

- TCAD15->Trypco1->Trypsin+HDxxH+VMAP-M0+VMAP-C->MoxR->vWA+TPRs*->FXSXX-COOH-like->METALLOPROTEASE+TPRs->TIR+APATPase+TPRs->
- Trypco1->Trypsin+VMAP-M0+VMAP-C->MoxR->vWA+Pkinase+cNMP_cyclase+Band7+HAD*->

■ **GCA_001541145.1; Streptomyces albus; counts= 2; multicellularity status: TRUE**

- <-TCAD15||Trypco1->Trypsin+HDxxH+VMAP-M0+VMAP-C->MoxR->vWA+TPRs*->FXSXX-COOH->Radical_SAM->TIR+APATPase+TPRs->
- Trypco1->Trypsin+EAD2+VMAP-M0+VMAP-C->MoxR->vWA+wHTH+TPRs+wHTH+TPRs+APATPase+wHTH+TPRs*->||<-AB_hydrolase

■ **GCF_001553455.1; Streptomyces hygroscopicus; counts= 2; multicellularity status: TRUE**

- vWA+APATPase+wHTH+TPRs*->||<-METALLOPROTEASE+TPRs
- vWA+TPRs*->

■ **GCF_001553435.1; Streptomyces hygroscopicus; counts= 2; multicellularity status: TRUE**

- AB_hydrolase->CASPASE+EAD2+VMAP-M0+VMAP-C->MoxR->vWA+TPRs*->
- PNP_UDP_1+EAD2->EAD2+VMAP-M0+VMAP-C->MoxR->vWA+APATPase+wHTH+TPRs*->||<-METALLOPROTEASE+TPRs

■ **GCF_000772045.1; Streptomyces sp. CCM_MD2014; counts= 2; multicellularity status: TRUE**

- <-TCAD15||Trypco1->Trypsin+HDxxH+VMAP-M0+VMAP-C->MoxR->vWA+TPRs*->FXSXX-COOH->Radical_SAM->METALLOPROTEASE+TPRs->TIR+APATPase+TPRs->Trypco1->
- Trypco1->Trypsin+EAD2+VMAP-M0+VMAP-C->MoxR->vWA+Pkinase+ParA-Soj-PloopNTPase(SIMIBI)*->

■ **GCF_001184025.1; Streptomyces sp. NBRC 110468; counts= 2; multicellularity status: TRUE**

- <-TCAD15||Trypco1->Trypsin+HDxxH+VMAP-M0+VMAP-C->MoxR->vWA+TPRs*->FXSXX-COOH->Radical_SAM->METALLOPROTEASE+TPRs->TIR+APATPase+TPRs->Trypco1->
- DUF397->Trypco1->Trypsin+EAD2+VMAP-M0+VMAP-C->MoxR->vWA+Pkinase+ParA-Soj-PloopNTPase(SIMIBI)*->

■ **GCA_001632865.1; Streptomyces ambofaciens; counts= 2; multicellularity status: TRUE**

- EAD2+VMAP-M0+VMAP-C->MoxR->vWA+APATPase+wHTH+TPRs*->||<-Na_H_Exchanger||AB_hydrolase->CASPASE+EAD2->FXSXX-COOH-like->METALLOPROTEASE+TPRs->
- Trypco1->Trypsin+VMAP-M0+VMAP-C->MoxR->vWA+Pkinase+wHTH+TPRs+cNMP_cyclase+HAD*->

■ **GCF_001640565.1; Streptomyces sp. RTd22; counts= 2; multicellularity status: TRUE**

- EAD1+Trypsin->EAD2+VMAP-M0+VMAP-C->MoxR->vWA+BetaPropeller+CASPASE+APATPase+wHTH+TPRs+AB_hydrolase*->

- vWA+APATPase+wHTH+TPRs*->||<-METALLOPROTEASE+TPRs<-Radical_SAM<-FXSXX-COOH||Voltage_CLC->PSE->||<-Trypco1

■ **GCF_001652895.1; Streptomyces sp. NBRC 109436; counts= 2; multicellularity status: TRUE**

- CASPASE+EAD2+VMAP-M0+VMAP-C->MoxR->vWA+TPRs*->
- PNP_UDP_1+EAD2->EAD2+VMAP-M0+VMAP-C->MoxR->vWA+APATPase+wHTH+TPRs*->||<-METALLOPROTEASE+TPRs

■ **GCA_001701025.1; Lentzea guizhouensis; counts= 2; multicellularity status: TRUE**

- <-cNMP_binding+HTH_Crp_2||cNMP_cyclase+EAD2+VMAP-M0+VMAP-C->MoxR->vWA+APATPase+wHTH+TPRs*->||<-METALLOPROTEASE+TPRs<-FXSXX-COOH-like
- AB_hydrolase->BTLCP+VMAP-M2+VMAP-C->MoxR->vWA+CASPASE+NACHT+TPRs*->

■ **GCF_001704635.1; Streptomyces sparsogenes; counts= 2; multicellularity status: TRUE**

- PNP_UDP_1+EAD2->EAD2+VMAP-M0+VMAP-C->MoxR->vWA+APATPase+wHTH+TPRs*->||<-METALLOPROTEASE+TPRs
- Trypco1->Trypsin+EAD2+VMAP-M0+VMAP-C->MoxR->vWA+Pkinase+Pkinase*->Porphobil_deam+Porphobil_deamC->

■ **GCF_001705785.1; Streptomyces sporocinereus; counts= 2; multicellularity status: TRUE**

- AB_hydrolase->CASPASE+EAD2+VMAP-M0+VMAP-C->MoxR->vWA+TPRs*->
- PNP_UDP_1+EAD2->||<-?||EAD2+VMAP-M0+VMAP-C->MoxR->vWA+APATPase+wHTH+TPRs*->||<-METALLOPROTEASE+TPRs

■ **GCF_001590865.1; Streptomyces sp. NBRC 110611; counts= 2; multicellularity status: TRUE**

- <-TCAD15||Trypco1->Trypsin+HDxxH+VMAP-M0+VMAP-C->MoxR->vWA+TPRs*->FXSXX-COOH->Radical_SAM->TIR+APATPase+TPRs->
- Trypsin+EAD2+VMAP-M0+VMAP-C->MoxR->?->vWA+wHTH+TPRs+APATPase+wHTH+TPRs*->

■ **GCA_001513965.1; Streptomyces cellostaticus; counts= 2; multicellularity status: TRUE**

- DUF397->?->?->?->Trypco1->Trypsin+VMAP-M0+VMAP-C->MoxR->vWA+Pkinase+wHTH+TPRs+cNMP_cyclase+Band7+HAD*->
- Trypco2->EAD2+VMAP-M0+VMAP-C->vWA+APATPase+wHTH+TPRs*->||<-METALLOPROTEASE+TPRs<-Trypsin+EAD2

■ **GCA_001636945.1; Streptomyces sp. FXJ1.172; counts= 2; multicellularity status: TRUE**

- TCAD15->Trypco1->Trypsin+HDxxH+VMAP-M0+VMAP-C->MoxR->vWA+TPRs*->FXSXX-COOH->METALLOPROTEASE+TPRs->TIR+APATPase+TPRs->
- Trypco1->Trypsin+EAD2+VMAP-M0+VMAP-C->MoxR->vWA+Pkinase+ParA-Soj-PloopNTPase(SIMIBI)*->

■ **GCF_001553475.1; Streptomyces endus; counts= 2; multicellularity status: TRUE**

- AB_hydrolase->CASPASE+EAD2+VMAP-M0+VMAP-C->MoxR->vWA+TPRs*->
- PNP_UDP_1+EAD2->EAD2+VMAP-M0+VMAP-C->MoxR->vWA+APATPase+wHTH+TPRs*->||<-METALLOPROTEASE+TPRs

■ **GCA_001653515.1; Streptomyces sp. ERV7; counts= 2; multicellularity status: TRUE**

- NUDIX->Trypco1->Trypsin+EAD2+VMAP-M0+VMAP-C->MoxR->vWA+Pkinase+ParA-Soj-PloopNTPase(SIMIBI)*->
- vWA+TPRs*->Radical_SAM->TIR+APATPase+TPRs->

■ **GCA_002335465.1; Streptomyces olivochromogenes; counts= 2; multicellularity status: TRUE**

- ADP_ribosyl_GH->NUDIX->Trypco1->Trypsin+EAD2+VMAP-M0+VMAP-C->MoxR->vWA+Pkinase+ParA-Soj-PloopNTPase(SIMIBI)*->
- Trypco1->Trypsin+HDxxH+VMAP-M0+VMAP-C->MoxR->vWA+CASPASE+TIR+NUDIX*->

■ **GCF_001552215.1; Actinomadura macra; counts= 2; multicellularity status: TRUE**

- Trypsin+VMAP-M8+VMAP-C->MoxR->vWA+FtsK+FtsK+FtsK*->

- Trypsin+VMAP-M8+VMAP-C->MoxR->vWA+Pkinase+DO-GTPase2*->

■ **GCF_001552195.1; Actinomadura latina; counts= 2; multicellularity status: TRUE**

- Radical_SAM->||<-TIR+APATPase+TPRs||SWACOS->PSE->?->EAD2+VMAP-M0+VMAP-C->MoxR->vWA+APATPase+wHTH+TPRs*->||<-Radical_SAM<-?||cNMP_cyclase+EAD2->
- Trypco1->Trypsin+VMAP-M8+VMAP-C->MoxR->vWA+FtsK+FtsK+CASPASE+FtsK*->

■ **GCF_001723065.1; Streptomyces rubidus; counts= 2; multicellularity status: TRUE**

- EAD2+VMAP-M0+VMAP-C->vWA+APATPase+wHTH+TPRs*->||<-METALLOPROTEASE+TPRs<-Trypsin+EAD2<-Trypco2
- vWA+APATPase+wHTH+TPRs*->||<-METALLOPROTEASE+TPRs<-Trypsin+EAD2<-Trypco2

■ **GCA_001729485.1; Streptomyces lydicus; counts= 2; multicellularity status: TRUE**

- <-TCAD15||Trypco1->Trypsin+HDxxH+VMAP-M0+VMAP-C->MoxR->vWA+TPRs*->FXSXX-COOH->Radical_SAM->
- EAD2+VMAP-M0+VMAP-C->vWA*->

■ **GCF_001723075.1; Streptomyces niveus; counts= 2; multicellularity status: TRUE**

- Trypco2->Trypsin+EAD2+VMAP-M0+VMAP-C->MoxR->vWA+Pkinase*->
- vWA+TPRs*->FXSXX-COOH->METALLOPROTEASE+TPRs->

■ **GCF_001723055.1; Streptomyces niveus; counts= 2; multicellularity status: TRUE**

- AB_hydrolase->CASPASE+VMAP-M0+VMAP-C->MoxR->vWA+STAND_NTPase+BetaPropeller+REase(Mrr_cat-PDDEXK)*->
- Trypco2->Trypsin+EAD2+VMAP-M0+VMAP-C->MoxR->vWA+Pkinase+TPRs*->

■ **GCF_001748165.1; Streptomyces sp. SPMA113; counts= 2; multicellularity status: TRUE**

- AB_hydrolase->CASPASE+EAD2+VMAP-M0+VMAP-C->MoxR->vWA+TPRs*->
- PNP_UDP_1+EAD2->EAD2+VMAP-M0+VMAP-C->MoxR->vWA+APATPase+wHTH+TPRs*->||<-METALLOPROTEASE+TPRs<-FXSXX-COOH-like

■ **GCA_001767375.1; Streptomyces pactum; counts= 2; multicellularity status: TRUE**

- <-TCAD15||PSE->Trypsin+HDxxH+VMAP-M0+VMAP-C->MoxR->vWA+TPRs*->PSE->Radical_SAM->METALLOPROTEASE+TPRs->TIR+APATPase+TPRs->
- vWA+Pkinase+ParA-Soj-PloopNTPase(SIMIBI)*->

■ **GCA_001854205.1; Moorea producens; counts= 2; multicellularity status: TRUE**

- AB_hydrolase->CASPASE+VMAP-M14+VMAP-C->MoxR->vWA+FGS-fold_domain*->FGS-fold_domain->four_helix_bundle_protein->
- EAD1+Trypsin->EAD1+VMAP-M1+Calcineurin-like->EAD1+VMAP-M1+VMAP-C->MoxR->vWA+FGS-fold_domain*->

■ **GCA_001866675.1; Streptomyces sp. MUSC 14; counts= 2; multicellularity status: TRUE**

- <-TCAD15||Trypco1->Trypsin+HDxxH+VMAP-M0+VMAP-C->MoxR->vWA+TPRs*->FXSXX-COOH->Radical_SAM->METALLOPROTEASE+TPRs->TIR+APATPase+TPRs->
- Trypco1->Trypsin+VMAP-M0+VMAP-C->MoxR->vWA+cNMP_cyclase+Band7+HAD*->

■ **GCA_001866665.1; Streptomyces sp. MUSC 1; counts= 2; multicellularity status: TRUE**

- <-TCAD15||Trypco1->Trypsin+HDxxH+VMAP-M0+VMAP-C->MoxR->vWA+TPRs*->FXSXX-COOH->Radical_SAM->METALLOPROTEASE+TPRs->TIR+APATPase+TPRs->
- Trypco1->Trypsin+VMAP-M0+VMAP-C->MoxR->vWA+cNMP_cyclase+Band7+HAD*->

■ **GCA_002368235.1; Scytonema sp. HK-05; counts= 2; multicellularity status: TRUE**

- Trypco1->Trypsin+EAD9->EAD9+EAD1+VMAP-C->MoxR->vWA+FGS-fold_domain*->||<-?||FGS-fold_domain->

- Trypco2->TCAD3+TCAD4+CASPASE+EAD1->EAD1+VMAP-M1+VMAP-C->MoxR->vWA+FGS-fold_domain*->FGS-fold_domain->

■ **GCA_001905125.1; Streptomyces sp. TSRI0281; counts= 2; multicellularity status: TRUE**

- <-TCAD15||Trypco1->Trypsin+HDxxH+VMAP-M0+VMAP-C->MoxR->vWA+TPRs*->Radical_SAM->TIR+APATPase+TPRs->
- EAD2+VMAP-M0+VMAP-C->MoxR->vWA+APATPase+wHTH+TPRs*->AB_hydrolase->CASPASE+EAD2->FXSXX-COOH-like->METALLOPROTEASE+TPRs->

■ **GCF_001905125.1; Streptomyces sp. TSRI0281; counts= 2; multicellularity status: TRUE**

- Trypco1->Trypsin+EAD2+VMAP-M0+VMAP-C->MoxR->vWA+wHTH+TPRs+wHTH+TPRs+APATPase+wHTH+TPRs*->
- Trypco1->Trypsin+VMAP-M0+VMAP-C->MoxR->vWA+Pkinase+NaeI*->

■ **GCA_001905385.1; Streptomyces sp. CB02414; counts= 2; multicellularity status: TRUE**

- EAD2+VMAP-M0+VMAP-C->MoxR->vWA+APATPase+wHTH+TPRs*->||<-Na_H_Exchanger||AB_hydrolase->CASPASE+EAD2->FXSXX-COOH-like->PSE->METALLOPROTEASE+TPRs->
- Trypco1->Trypsin+VMAP-M0+VMAP-C->MoxR->vWA+Pkinase+wHTH+TPRs+cNMP_cyclase*->

■ **GCF_001905885.1; Streptomyces sp. CB03234; counts= 2; multicellularity status: TRUE**

- AB_hydrolase->EAD2+VMAP-M0+VMAP-C->MoxR->vWA+APATPase+wHTH+TPRs*->||<-METALLOPROTEASE+TPRs<-FXSXX-COOH-like<-CASPASE+EAD2
- Trypsin+EAD2+VMAP-M0+VMAP-C->MoxR->vWA+wHTH+TPRs+wHTH+TPRs+APATPase+wHTH+TPRs*->

■ **GCF_001905575.1; Streptomyces sp. CB00072; counts= 2; multicellularity status: TRUE**

- <-TCAD15||Trypco1->Trypsin+HDxxH+VMAP-M0+VMAP-C->MoxR->vWA+TPRs*->Radical_SAM->TIR+APATPase+TPRs->
- EAD2+VMAP-M0+VMAP-C->MoxR->vWA+APATPase+wHTH+TPRs*->AB_hydrolase->CASPASE+EAD2->FXSXX-COOH-like->METALLOPROTEASE+TPRs->

■ **GCF_001905845.1; Streptomyces sp. TSRI0107; counts= 2; multicellularity status: TRUE**

- EAD2+VMAP-M0+VMAP-C->MoxR->vWA+APATPase+wHTH+TPRs*->||<-Na_H_Exchanger||CASPASE+EAD2->FXSXX-COOH-like->METALLOPROTEASE+TPRs->
- Trypco1->Trypsin+VMAP-M0+VMAP-C->MoxR->vWA+Pkinase+wHTH+TPRs+cNMP_cyclase+Band7+HAD*->

■ **GCF_001941265.1; Streptomyces acidiscabies; counts= 2; multicellularity status: TRUE**

- vWA+Pkinase+wHTH+cNMP_cyclase+Band7+HAD*->
- vWA+TIR+FxsC_Cter(partial)*->

■ **GCF_001941285.1; Streptomyces acidiscabies; counts= 2; multicellularity status: TRUE**

- vWA+Pkinase+wHTH+cNMP_cyclase+Band7+HAD*->
- vWA+TIR+FxsC_Cter(partial)*->

■ **GCA_001942465.1; Actinomadura sp. CNU-125; counts= 2; multicellularity status: TRUE**

- AB_hydrolase->BTLCP+VMAP-M2+VMAP-C->MoxR->vWA_protein*->
- Radical_SAM->PSE->||<-PSE||FXSXX-COOH-like->?->EAD2+VMAP-M0+VMAP-C->MoxR->vWA+APATPase+wHTH+TPRs*->||<-FXSXX-COOH-like||cNMP_cyclase+EAD2->

■ **GCA_001942495.1; Moorea bouillonii; counts= 2; multicellularity status: TRUE**

- AB_hydrolase->CASPASE+VMAP-M14+VMAP-C->MoxR->vWA+FGS-fold_domain*->FGS-fold_domain->four_helix_bundle_protein->
- EAD1+Trypsin->EAD1+VMAP-M1+Calcineurin-like->EAD1+VMAP-M1+VMAP-C->MoxR->vWA+FGS-fold_domain*->

■ **GCA_001942255.1; Nocardiopsis sp. CNR-923; counts= 2; multicellularity status: TRUE**

- Trypsin+EAD2->VMAP-C->MoxR->?->vWA_protein*->TIR+APATPase+TPRs->||<-DUF397||Trypco1->||<-PSE<-?<-PSE<-PSE<-DUF397

- Trypsin+VMAP-M8->VMAP-C->MoxR->vWA+APATPase+wHTH*->

- **GCA_001953865.1; Amycolatopsis coloradensis; counts= 2; multicellularity status: TRUE**
  - AB_hydrolase->CASPASE+VMAP-M2+VMAP-C->MoxR->vWA+TPRs*->
  - cNMP_cyclase+VMAP-M0+VMAP-C->MoxR->vWA+APATPase+wHTH+TPRs*->||<-METALLOPROTEASE+TPRs<-FXSXX-COOH-like<-cNMP_binding+HTH_Crp_2

- **GCA_001970135.1; Streptomyces sp. M1013; counts= 2; multicellularity status: TRUE**
  - TCAD15->Trypco1->Trypsin+HDxxH+VMAP-M0+VMAP-C->MoxR->vWA+TPRs*->FXSXX-COOH-like->METALLOPROTEASE+TPRs->TIR+APATPase+TPRs->
  - Trypsin+VMAP-M6+VMAP-C->MoxR->vWA+APATPase+wHTH+TPRs*->||<-METALLOPROTEASE+TPRs<-Radical_SAM<-Trypco1

- **GCA_001984155.1; Actinosynnema sp. ALI-1.44; counts= 2; multicellularity status: TRUE**
  - EAD2+VMAP-M0+VMAP-C->MoxR->vWA+APATPase+wHTH+TPRs->||<-METALLOPROTEASE+TPRs<-FXSXX-COOH-like||EAD2+VMAP-M0+VMAP-C->MoxR->vWA+APATPase+wHTH+TPRs*->||<-METALLOPROTEASE+TPRs<-FXSXX-COOH-like
  - EAD2+VMAP-M0+VMAP-C->MoxR->vWA+APATPase+wHTH+TPRs*->||<-METALLOPROTEASE+TPRs<-FXSXX-COOH-like||EAD2+VMAP-M0+VMAP-C->MoxR->vWA+APATPase+wHTH+TPRs->||<-METALLOPROTEASE+TPRs<-FXSXX-COOH-like

- **GCA_002025445.1; Microcystis aeruginosa; counts= 2; multicellularity status: TRUE**
  - AB_hydrolase->PSE->||<-PSE||EAD2+VMAP-M11+VMAP-C->MoxR->vWA+FGS-fold_domain*->
  - Trypco1->||<-?||Trypsin+VMAP-M1+VMAP-C->MoxR->vWA+FGS-fold_domain*->

- **GCF_002028385.1; Streptomyces sp. 3211; counts= 2; multicellularity status: TRUE**
  - <-TCAD15||Trypsin+HDxxH+VMAP-M0+VMAP-C->MoxR->vWA+TPRs*->FXSXX-COOH->Radical_SAM->METALLOPROTEASE+TPRs->TIR+APATPase+TPRs->
  - AB_hydrolase->CASPASE+VMAP-M0+VMAP-C->MoxR->vWA+Trypsin+cNMP_cyclase+CCCH(TrebleClef)+Pentapeptide_repeats*->

- **GCF_900129855.1; Streptomyces sp. 3124.6; counts= 2; multicellularity status: TRUE**
  - <-TCAD15||Trypco1->Trypsin+HDxxH+VMAP-M0+VMAP-C->MoxR->vWA+TPRs*->FXSXX-COOH->Radical_SAM->METALLOPROTEASE+TPRs->TIR+APATPase+TPRs->
  - Trypco1->Trypsin+EAD2+VMAP-M0+VMAP-C->MoxR->vWA+Pkinase+ParA-Soj-PloopNTPase(SIMIBI)*->

- **GCA_002080455.1; Streptomyces sp. Sge12; counts= 2; multicellularity status: TRUE**
  - <-TCAD15||Trypsin+HDxxH+VMAP-M0+VMAP-C->MoxR->vWA+TPRs*->FXSXX-COOH->Radical_SAM->METALLOPROTEASE+TPRs->TIR+APATPase+TPRs->
  - AB_hydrolase->CASPASE+VMAP-M0+VMAP-C->MoxR->vWA+Trypsin+cNMP_cyclase+CCCH(TrebleClef)+Pentapeptide_repeats*->

- **GCA_000012405.1; Thermobifida fusca YX; counts= 1; multicellularity status: TRUE**
  - Trypsin+EAD2->EAD2+VMAP-M0+VMAP-C->MoxR->vWA+APATPase+wHTH+TPRs*->Trypco1->

- **GCA_000018005.1; Frankia sp. EAN1pec; counts= 1; multicellularity status: TRUE**
  - EAD2+EAD1->EAD2+VMAP-M0+VMAP-C->MoxR->vWA+APATPase+wHTH+TPRs*->||<-METALLOPROTEASE+TPRs

- **GCA_000020025.1; Nostoc punctiforme PCC 73102; counts= 1; multicellularity status: TRUE**
  - VMAP-M1+VMAP-C->MoxR->vWA+Calcineurin-like+SAVED*->

- **GCA_000022045.1; Cyanothece sp. PCC 7425; counts= 1; multicellularity status: TRUE**
  - <-FGS-fold_domain<-?<-?<-?||VMAP-M1+VMAP-C->MoxR->vWA*->||<-zf-IS66+DDE_Tnp_IS66<-TIR+EAD9<-?<-Trypsin<-Trypco1

- **GCA_000024385.1; Thermomonospora curvata DSM 43183; counts= 1; multicellularity status: TRUE**
  - Radical_SAM->METALLOPROTEASE+TPRs->||<-TIR+APATPase+TPRs||EAD2+VMAP-M0+VMAP-C->MoxR->vWA+APATPase+wHTH+TPRs*->

- **GCA_000237145.1; Actinoplanes sp. SE50/110; counts= 1; multicellularity status: TRUE**
  - EAD1+VMAP-M0+VMAP-C->MoxR->vWA+TPRs*->
- **GCA_000359525.1; Streptomyces albus J1074; counts= 1; multicellularity status: TRUE**
  - <-TCAD15||Trypco1->Trypsin+HDxxH+VMAP-M0+VMAP-C->MoxR->vWA+TPRs*->FXSXX-COOH->Radical_SAM->METALLOPROTEASE+TPRs->TIR+APATPase+TPRs->
- **GCA_000418455.1; Streptomyces rapamycinicus NRRL 5491; counts= 1; multicellularity status: TRUE**
  - PNP_UDP_1+EAD2->EAD2+VMAP-M0+VMAP-C->MoxR->vWA+APATPase+wHTH+TPRs*->||<-METALLOPROTEASE+TPRs<-FXSXX-COOH-like
- **GCA_000444875.1; Streptomyces collinus Tu 365; counts= 1; multicellularity status: TRUE**
  - TCAD15->Trypco1->Trypsin+HDxxH+VMAP-M0+VMAP-C->MoxR->vWA+TPRs*->FXSXX-COOH-like->METALLOPROTEASE+TPRs->TIR+APATPase+TPRs->
- **GCA_000525635.1; Kutzneria albida DSM 43870; counts= 1; multicellularity status: TRUE**
  - EAD2+VMAP-M0+VMAP-C->MoxR->?->vWA+TIR+BetaPropeller*->?->?->||<-cNMP_cyclase+EAD2||cNMP_binding+HTH_Crp_2->?->SIR2->
- **GCA_000340565.3; Nodularia spumigena CCY9414; counts= 1; multicellularity status: TRUE**
  - TIR+EAD6->EAD6+VMAP-M5->VMAP-C->MoxR->vWA+beta-rich-region+HTH+FGS-fold_domain*->four_helix_bundle_protein->RVT_1->
- **GCA_000732925.1; Amycolatopsis japonica; counts= 1; multicellularity status: TRUE**
  - cNMP_cyclase+VMAP-M0+VMAP-C->MoxR->vWA+APATPase+wHTH+TPRs*->||<-METALLOPROTEASE+TPRs<-FXSXX-COOH-like<-cNMP_binding+HTH_Crp_2
- **GCA_000749465.2; Amycolatopsis lurida NRRL 2430; counts= 1; multicellularity status: TRUE**
  - cNMP_cyclase+VMAP-M0+VMAP-C->MoxR->vWA+APATPase+wHTH+TPRs*->||<-?||?->||<-FXSXX-COOH-like<-cNMP_binding+HTH_Crp_2
- **GCA_000931445.1; Streptomyces cyaneogriseus subsp. noncyanogenus; counts= 1; multicellularity status: TRUE**
  - <-TCAD15||Trypco1->Trypsin+HDxxH+VMAP-M0+VMAP-C->MoxR->vWA+TPRs*->FXSXX-COOH->METALLOPROTEASE+TPRs->TIR+APATPase+TPRs->
- **GCA_001027185.1; Streptomyces incarnatus; counts= 1; multicellularity status: TRUE**
  - Trypco2->EAD2+VMAP-M0+VMAP-C->vWA+APATPase+wHTH+TPRs*->||<-FXSXX-COOH-like<-Trypsin+EAD2||?->?->?->?->DUF397->
- **GCA_001277295.1; Anabaena sp. WA102; counts= 1; multicellularity status: TRUE**
  - EAD6+VMAP-C->MoxR->vWA+FGS-fold_domain*->FGS-fold_domain->
- **GCA_001278095.1; Streptomyces sp. CFMR 7; counts= 1; multicellularity status: TRUE**
  - CASPASE+VMAP-M2+VMAP-C->MoxR->vWA+REase+SWACOS+TPRs+cNMP_cyclase*->
- **GCA_001431765.1; Streptomyces sp. FR-008; counts= 1; multicellularity status: TRUE**
  - low-complexity+HDxxH+VMAP-M0->VMAP-C->MoxR->vWA+TPRs*->FXSXX-COOH->?->METALLOPROTEASE+TPRs->TIR+APATPase+TPRs->
- **GCA_001577385.1; Streptomyces albus; counts= 1; multicellularity status: TRUE**
  - <-TCAD15||Trypco1->VMAP-C->MoxR->vWA+TPRs*->FXSXX-COOH->Radical_SAM->METALLOPROTEASE+TPRs->TIR+APATPase+TPRs->
- **GCA_001610855.1; Gemmata sp. SH-PL17; counts= 1; multicellularity status: FALSE**
  - <-EAD1+HD<-EAD1+Trypsin||VMAP-M21+VMAP-C->MoxR->EAD1+HD+vWA+NEFF1*->

- **GCA_001660045.1; Streptomyces parvulus; counts= 1; multicellularity status: TRUE**
  - Trypco2->EAD2+VMAP-M0+VMAP-C->vWA+APATPase+wHTH+TPRs*->||<-METALLOPROTEASE+TPRs<-FXSXX-COOH-like<-Trypsin+EAD2
- **GCA_001664405.1; Rhizobium phaseoli; counts= 1; multicellularity status: TRUE**
  - EAD5+Trypsin->EAD5+VMAP-M4+VMAP-C->MoxR->vWA+REC+SLOG+CASPASE*->
- **GCA_000943515.2; Amycolatopsis orientalis; counts= 1; multicellularity status: TRUE**
  - cNMP_cyclase+VMAP-M0+VMAP-C->MoxR->vWA+APATPase+wHTH+TPRs*->||<-METALLOPROTEASE+TPRs<-FXSXX-COOH-like<-cNMP_binding+HTH_Crp_2
- **GCA_001542625.2; Streptomyces griseochromogenes; counts= 1; multicellularity status: TRUE**
  - Trypco2->EAD2+VMAP-M0+VMAP-C->vWA+APATPase+wHTH+TPRs*->||<-METALLOPROTEASE+TPRs<-Trypsin+EAD2||?->Trypco2->
- **GCA_001750785.1; Streptomyces rubrolavendulae; counts= 1; multicellularity status: TRUE**
  - Trypco1->?->?->AB_hydrolase->CASPASE+VMAP-M2+VMAP-C->MoxR->vWA+APATPase+wHTH+TPRs+cNMP_cyclase+NaeI*->
- **GCA_002128305.1; Streptomyces sp. SCSIO 03032; counts= 1; multicellularity status: TRUE**
  - <-TCAD15||PSE->Trypsin+HDxxH+VMAP-M0+VMAP-C->MoxR->vWA+TPRs*->FXSXX-COOH->Radical_SAM->METALLOPROTEASE+TPRs->TIR+APATPase+TPRs->
- **GCA_002142475.1; Leptolyngbya boryana IAM M-101; counts= 1; multicellularity status: TRUE**
  - TIR+EAD9->EAD9+VMAP-M1+VMAP-C->MoxR->vWA+FGS-fold_domain*->
- **GCA_002368175.1; Calothrix sp. NIES-2098; counts= 1; multicellularity status: TRUE**
  - EAD8+VMAP-M1+VMAP-C->MoxR->?->vWA+MNS*->
- **GCA_002368455.1; Calothrix sp. NIES-4071; counts= 1; multicellularity status: TRUE**
  - EAD1+Trypsin->EAD1+VMAP-M1+VMAP-C->MoxR->vWA+GUN4+FGS-fold_domain*->
- **GCA_003994795.1; Cylindrospermum sp. NIES-4074; counts= 1; multicellularity status: TRUE**
  - cNMP_cyclase+VMAP-M1+VMAP-C->MoxR->vWA+Glyco_trans_4_4+Glycos_transf_1*->FGS-fold_domain->
- **GCA_001160225.1; Mycobacterium tuberculosis; counts= 1; multicellularity status: FALSE**
  - Trypco1->Trypsin+VMAP-M8+VMAP-C->MoxR->vWA+FtsK+FtsK+CASPASE+FtsK*->
- **GCA_001536285.1; Frankia sp. G2; counts= 1; multicellularity status: TRUE**
  - EAD2+EAD1->EAD2+VMAP-M0+VMAP-C->MoxR->vWA+APATPase+wHTH+TPRs*->||<-METALLOPROTEASE+TPRs<-FXSXX-COOH-like
- **GCA_000154885.1; Streptomyces sp. Mg1; counts= 1; multicellularity status: TRUE**
  - <-cNMP_binding+HTH_Crp_2<-?<-?||EAD2+VMAP-M0+VMAP-C->MoxR->vWA*->
- **GCA_000156455.1; Streptomyces roseosporus NRRL 15998; counts= 1; multicellularity status: TRUE**
  - EAD2+VMAP-M0+VMAP-C->MoxR->vWA+APATPase+wHTH+TPRs*->AB_hydrolase->CASPASE+EAD2->FXSXX-COOH-like->METALLOPROTEASE+TPRs->
- **GCA_000154945.1; Streptomyces pristinaespiralis ATCC 25486; counts= 1; multicellularity status: TRUE**
  - AB_hydrolase->CASPASE+VMAP-M2+VMAP-C->MoxR->vWA+TIR+FxsC_Cter*->||<-DUF397

- **GCA_000158895.1; Streptomyces sp. C; counts= 1; multicellularity status: TRUE**
  - <-TCAD15||Trypsin+HDxxH+VMAP-M0+VMAP-C->MoxR->vWA*->FXSXX-COOH->Radical_SAM->METALLOPROTEASE+TPRs->TIR+APATPase+TPRs->
- **GCA_000152145.1; Oscillochloris trichoides DG-6; counts= 1; multicellularity status: TRUE**
  - <-CASPASE+TCAD1+TCAD2||CASPASE+TCAD1+DUF4404->VMAP-M13+VMAP-C->MoxR->vWA+TPRs*->
- **GCA_000244995.1; Burkholderiales bacterium JOSHI_001; counts= 1; multicellularity status: FALSE**
  - AB_hydrolase->CASPASE+VMAP-M15+VMAP-C->MoxR->vWA+HTH*->
- **GCA_000243495.3; Opitutaceae bacterium TAV1; counts= 1; multicellularity status: FALSE**
  - TCAD5+AB_hydrolase+TCAD7+macroglobulin-like+CASPASE->CASPASE+VMAP-C->MoxR->vWA+REC+HTH+SLOG*->
- **GCA_000262465.1; Frankia sp. QA3; counts= 1; multicellularity status: TRUE**
  - EAD2+VMAP-M0+VMAP-C->MoxR->vWA+APATPase+wHTH+TPRs*->||<-METALLOPROTEASE+TPRs<-?<-EAD5+TIR
- **GCA_000342005.1; Amycolatopsis decaplanina DSM 44594; counts= 1; multicellularity status: TRUE**
  - cNMP_cyclase+VMAP-M0+VMAP-C->MoxR->vWA+APATPase+wHTH+TPRs*->||<-METALLOPROTEASE+TPRs<-FXSXX-COOH-like<-cNMP_binding+HTH_Crp_2
- **GCA_000342125.1; Streptomyces mobaraensis NBRC 13819 = DSM 40847; counts= 1; multicellularity status: TRUE**
  - <-TCAD15||Trypco1->Trypsin+HDxxH+VMAP-M0+VMAP-C->MoxR->vWA+TPRs*->FXSXX-COOH-like->METALLOPROTEASE+TPRs->TIR+APATPase+TPRs->
- **GCA_000495635.1; Streptomyces sp. GBA 94-10; counts= 1; multicellularity status: TRUE**
  - <-TCAD15||Trypco1->Trypsin+HDxxH+VMAP-M0+VMAP-C->MoxR->vWA+TPRs*->FXSXX-COOH->Radical_SAM->METALLOPROTEASE+TPRs->TIR+APATPase+TPRs->
- **GCA_000495755.1; Streptomyces sp. PVA 94-07; counts= 1; multicellularity status: TRUE**
  - <-TCAD15||Trypco1->Trypsin+HDxxH+VMAP-M0+VMAP-C->MoxR->vWA+TPRs*->FXSXX-COOH->Radical_SAM->METALLOPROTEASE+TPRs->TIR+APATPase+TPRs->
- **GCA_000495615.1; uncultured Thiohalocapsa sp. PB-PSB1; counts= 1; multicellularity status: NA**
  - Trypco1->Trypsin+VMAP-M0+VMAP-C->MoxR->vWA_protein*->
- **GCA_000568255.1; Kutzneria sp. 744; counts= 1; multicellularity status: TRUE**
  - EAD2+VMAP-M0+VMAP-C->vWA+TIR+BetaPropeller*->?->?->cNMP_binding+HTH_Crp_2->
- **GCA_001485125.1; Streptomyces scabiei; counts= 1; multicellularity status: TRUE**
  - AB_hydrolase->CASPASE+VMAP-M2+VMAP-C->MoxR->vWA+SFII-helicase+Trypsin+cNMP_cyclase+Coiled-coil+CASPASE*->||<-?<-?<-TIR+APATPase+TPRs
- **GCA_001653115.1; Streptomyces sp. F-3; counts= 1; multicellularity status: TRUE**
  - Trypsin+EAD2->EAD2+VMAP-M0+VMAP-C->MoxR->vWA+APATPase+wHTH+TPRs*->||<-METALLOPROTEASE+TPRs||?->Trypco1->
- **GCA_000175415.3; Arthrospira platensis str. Paraca; counts= 1; multicellularity status: TRUE**
  - VMAP-C_protein->MoxR->vWA*->
- **GCA_000817745.1; Aphanocapsa montana BDHKU210001; counts= 1; multicellularity status: TRUE**
  - Trypco1->Trypsin+VMAP-M1+VMAP-C->MoxR->vWA+FGS-fold_domain*->FGS-fold_domain->

- **GCA_000818195.1; Streptomyces sp. 150FB; counts= 1; multicellularity status: TRUE**
  - <-TCAD15||Trypsin+HDxxH+VMAP-M0+VMAP-C->MoxR->vWA+TPRs*->FXSXX-COOH->METALLOPROTEASE+TPRs->TIR+APATPase+TPRs->
- **GCA_000826685.1; Streptomonospora alba; counts= 1; multicellularity status: TRUE**
  - Trypco1->Trypsin+EAD2+VMAP-M0+VMAP-C->MoxR->vWA+APATPase+wHTH+TPRs*->||<-Radical_SAM<-FXSXX-COOH-like
- **GCA_000876225.1; Micromonospora carbonacea; counts= 1; multicellularity status: TRUE**
  - Trypsin+HDxxH+VMAP-M0+VMAP-C->MoxR->vWA+TPRs*->?->TIR+APATPase+TPRs->
- **GCA_000955955.1; Lechevalieria aerocolonigenes; counts= 1; multicellularity status: TRUE**
  - <-cNMP_binding+HTH_Crp_2||MoxR->vWA+APATPase+wHTH+TPRs*->||<-METALLOPROTEASE+TPRs<-FXSXX-COOH-like
- **GCA_000966975.1; Streptomyces katrae; counts= 1; multicellularity status: TRUE**
  - vWA*->
- **GCA_000966985.1; Streptomyces sp. NRRL S-444; counts= 1; multicellularity status: TRUE**
  - <-TCAD15||Trypsin+HDxxH+VMAP-M0+VMAP-C->MoxR->vWA*->
- **GCA_001044425.1; Streptomyces roseus; counts= 1; multicellularity status: TRUE**
  - <-TCAD15||Trypsin+HDxxH+VMAP-M0+VMAP-C->MoxR->vWA+TPRs*->METALLOPROTEASE+TPRs->TIR+APATPase+TPRs->
- **GCA_001047325.1; Streptomyces viridochromogenes; counts= 1; multicellularity status: TRUE**
  - Trypco2->EAD2+VMAP-M0+VMAP-C->vWA+APATPase+wHTH+TPRs*->||<-FXSXX-COOH-like<-Trypsin+EAD2
- **GCA_001047315.1; Streptomyces leeuwenhoekii; counts= 1; multicellularity status: TRUE**
  - <-TCAD15||Trypco1->Trypsin+HDxxH+VMAP-M0+VMAP-C->MoxR->vWA+TPRs*->FXSXX-COOH->METALLOPROTEASE+TPRs->TIR+APATPase+TPRs->
- **GCA_001189025.1; Streptomyces europaeiscabiei; counts= 1; multicellularity status: TRUE**
  - EAD2+VMAP-M0+VMAP-C->MoxR->vWA+APATPase+wHTH+TPRs*->AB_hydrolase->CASPASE+EAD2->FXSXX-COOH-like->METALLOPROTEASE+TPRs->
- **GCA_001279025.1; Streptomyces sp. NRRL WC-3701; counts= 1; multicellularity status: TRUE**
  - Trypco1->Trypsin+EAD2+VMAP-M0+VMAP-C->MoxR->vWA+wHTH+TPRs+wHTH+TPRs+APATPase+wHTH+TPRs*->||<-AB_hydrolase
- **GCA_001279015.1; Streptomyces rimosus subsp. rimosus; counts= 1; multicellularity status: TRUE**
  - <-TCAD15||Trypco1->Trypsin+HDxxH+VMAP-M0+VMAP-C->MoxR->vWA+TPRs*->Radical_SAM->TIR+APATPase+TPRs->
- **GCA_001279355.1; Streptomyces sp. NRRL F-4711; counts= 1; multicellularity status: TRUE**
  - <-TCAD15||Trypco1->Trypsin+HDxxH+VMAP-M0+VMAP-C->MoxR->vWA+TPRs*->FXSXX-COOH->Radical_SAM->METALLOPROTEASE+TPRs->TIR+APATPase+TPRs->Trypco1->
- **GCA_001279725.1; Streptomyces sp. WM4235; counts= 1; multicellularity status: TRUE**
  - <-TCAD15||Trypsin+HDxxH+VMAP-M0+VMAP-C->vWA+TPRs*->Radical_SAM->METALLOPROTEASE+TPRs->TIR+APATPase+TPRs->
- **GCA_001279805.1; Streptomyces sp. XY431; counts= 1; multicellularity status: TRUE**
  - AB_hydrolase->CASPASE+EAD2+VMAP-C->MoxR->vWA+TPRs*->

■ **GCA_001279985.1; Streptomyces sp. AS58; counts= 1; multicellularity status: TRUE**

- EAD2+VMAP-M0+VMAP-C->vWA+APATPase+wHTH+TPRs*->||<-METALLOPROTEASE+TPRs<-FXSXX-COOH-like<-Trypsin+EAD2<-Trypco2||METALLOPROTEASE+TPRs->

■ **GCA_001280005.1; Streptomyces sp. NRRL WC-3618; counts= 1; multicellularity status: TRUE**

- MoxR->vWA+TPRs*->Radical_SAM->TIR+APATPase+TPRs->Radical_SAM->?->STAND_NTPase->TIR+APATPase+TPRs->||<-DUF397

■ **GCA_001279605.1; Streptomyces sp. NRRL F-4707; counts= 1; multicellularity status: TRUE**

- <-TCAD15||Trypco1->Trypsin+HDxxH+VMAP-M0+VMAP-C->MoxR->vWA+TPRs*->FXSXX-COOH->Radical_SAM->METALLOPROTEASE+TPRs->TIR+APATPase+TPRs->?->?->CASPASE->

■ **GCA_001279615.1; Streptomyces sp. NRRL F-7442; counts= 1; multicellularity status: TRUE**

- <-TCAD15||Trypco1->Trypsin+HDxxH+VMAP-M0+VMAP-C->MoxR->vWA+TPRs*->FXSXX-COOH->Radical_SAM->METALLOPROTEASE+TPRs->TIR+APATPase+TPRs->?->CASPASE->TIR+APATPase+TPRs->

■ **GCA_001294335.1; Streptomyces chattanoogensis; counts= 1; multicellularity status: TRUE**

- <-TCAD15||Trypco1->Trypsin+HDxxH+VMAP-M0+VMAP-C->MoxR->vWA+TPRs*->FXSXX-COOH->Radical_SAM->TIR+APATPase+TPRs->

■ **GCA_001294395.1; Streptomyces sp. NRRL WC-3753; counts= 1; multicellularity status: TRUE**

- EAD2+VMAP-M0+VMAP-C->MoxR->vWA+APATPase+wHTH+TPRs*->||<-Na_H_Exchanger||AB_hydrolase->CASPASE+EAD2->FXSXX-COOH-like->METALLOPROTEASE+TPRs->

■ **GCA_001294325.1; Streptomyces sp. NRRL F-6602; counts= 1; multicellularity status: TRUE**

- TCAD15->?->?->Trypco1->Trypsin+HDxxH+VMAP-M0+VMAP-C->MoxR->vWA+TPRs*->METALLOPROTEASE+TPRs->TIR+APATPase+TPRs->

■ **GCA_001298575.1; Actinobacteria bacterium OK074; counts= 1; multicellularity status: NA**

- NUDIX->Trypco1->Trypsin+EAD2+VMAP-M0+VMAP-C->MoxR->vWA+Pkinase+ParA-Soj-PloopNTPase(SIMIBI)*->SIR2->

■ **GCA_001414035.1; Frankia sp. ACN1ag; counts= 1; multicellularity status: TRUE**

- EAD2+EAD1->EAD2+VMAP-M0+VMAP-C->MoxR->vWA+APATPase+wHTH+TPRs*->||<-FXSXX-COOH-like

■ **GCA_001421075.1; Frankia sp. CpI1-P; counts= 1; multicellularity status: TRUE**

- EAD2+EAD1->EAD2+VMAP-M0+VMAP-C->MoxR->vWA+APATPase+wHTH+TPRs*->||<-METALLOPROTEASE+TPRs<-FXSXX-COOH-like

■ **GCA_001482415.1; Streptomyces silvensis; counts= 1; multicellularity status: TRUE**

- Trypsin+HDxxH+VMAP-M0+VMAP-C->MoxR->vWA+TPRs*->

■ **GCA_001484625.1; Streptomyces kanasensis; counts= 1; multicellularity status: TRUE**

- AB_hydrolase->CASPASE+VMAP-M2+VMAP-C->MoxR->vWA_protein*->vWA(partial)+APATPase+wHTH+cNMP_cyclase+NaeI->

■ **GCA_001507455.1; Streptomyces albus subsp. albus; counts= 1; multicellularity status: TRUE**

- PNP_UDP_1+EAD2->EAD2+VMAP-M0+VMAP-C->MoxR->vWA+APATPase+wHTH+TPRs*->||<-PSE<-FXSXX-COOH-like

■ **GCA_001509495.1; Actinoplanes awajinensis subsp. mycoplanecinus; counts= 1; multicellularity status: TRUE**

- FXSXX-COOH-like->METALLOPROTEASE+TPRs->PNP_UDP_1+VMAP-M0+VMAP-C->vWA+APATPase+wHTH+TPRs*->

■ **GCA_001514115.1; Streptomyces olivochromogenes; counts= 1; multicellularity status: TRUE**

- EAD2+VMAP-M0+VMAP-C->vWA+APATPase+wHTH+TPRs*->||<-METALLOPROTEASE+TPRs<-Trypsin+EAD2<-Trypco2||METALLOPROTEASE+TPRs->

- **GCA_001514135.1; Streptomyces avermitilis; counts= 1; multicellularity status: TRUE**
  - AB_hydrolase->CASPASE+VMAP-M2+VMAP-C->MoxR->vWA+APATPase+wHTH+TPRs+cNMP_cyclase+NaeI*->
- **GCA_001672255.1; Anabaena sp. AL09; counts= 1; multicellularity status: TRUE**
  - Trypco1->Trypsin+VMAP-M1+VMAP-C->MoxR->vWA+FGS-fold_domain*->
- **GCA_001672225.1; Anabaena sp. LE011-02; counts= 1; multicellularity status: TRUE**
  - Trypco1->Trypsin+VMAP-M1+VMAP-C->MoxR->vWA+FGS-fold_domain*->
- **GCA_001672085.1; Anabaena sp. AL93; counts= 1; multicellularity status: TRUE**
  - vWA+FGS-fold_domain*->
- **GCA_001672075.1; Anabaena sp. CRKS33; counts= 1; multicellularity status: TRUE**
  - Trypco1->VMAP-M1+EAD6+VMAP-C->MoxR->vWA+FGS-fold_domain*->
- **GCA_001672195.1; Anabaena sp. MDT14b; counts= 1; multicellularity status: TRUE**
  - Trypco1->MoxR->vWA+FGS-fold_domain*->
- **GCA_001672105.1; Aphanizomenon flos-aquae WA102; counts= 1; multicellularity status: TRUE**
  - cNMP_cyclase+EAD1->EAD1+VMAP-M24+VMAP-C->MoxR->vWA+beta-rich-region+HTH+FGS-fold_domain*->?->||<-RVT_1<-four_helix_bundle_protein<-FGS-fold_domain
- **GCA_001751255.1; Streptomyces nanshensis; counts= 1; multicellularity status: TRUE**
  - <-TCAD15||Trypco1->Trypsin+HDxxH+VMAP-M0+VMAP-C->MoxR->vWA*->
- **GCA_001751265.1; Streptomyces nanshensis; counts= 1; multicellularity status: TRUE**
  - Trypsin+HDxxH+VMAP-M0+VMAP-C->Trypsin+HDxxH+VMAP-M0+VMAP-C->MoxR->vWA*->?->TIR+APATPase+TPRs->
- **GCA_001866645.1; Streptomyces sp. MUSC 93; counts= 1; multicellularity status: TRUE**
  - AB_hydrolase->CASPASE+VMAP-M2+VMAP-C->MoxR->vWA+SFII-helicase+DUF3427*->
- **GCA_001904725.1; Phormidium ambiguum IAM M-71; counts= 1; multicellularity status: TRUE**
  - cNMP_cyclase+VMAP-M1+VMAP-C->MoxR->vWA+FGS-fold_domain*->FGS-fold_domain->
- **GCA_001905575.1; Streptomyces sp. CB00072; counts= 1; multicellularity status: TRUE**
  - EAD2+VMAP-M0+VMAP-C->MoxR->vWA+APATPase+wHTH+TPRs*->AB_hydrolase->CASPASE+EAD2->METALLOPROTEASE+TPRs->
- **GCA_001905425.1; Streptomyces sp. MJM1172; counts= 1; multicellularity status: TRUE**
  - EAD2+VMAP-M0+VMAP-C->MoxR->vWA+APATPase+wHTH+TPRs*->||<-Na_H_Exchanger||AB_hydrolase->CASPASE+EAD2->METALLOPROTEASE+TPRs->
- **GCA_001905405.1; Streptomyces sp. TSRI0445; counts= 1; multicellularity status: TRUE**
  - EAD2+VMAP-M0+VMAP-C->MoxR->vWA+APATPase+wHTH+TPRs*->AB_hydrolase->CASPASE+EAD2->FXSXX-COOH-like->METALLOPROTEASE+TPRs->
- **GCA_001905525.1; Streptomyces sp. TSRI0395; counts= 1; multicellularity status: TRUE**
  - EAD2+VMAP-M0+VMAP-C->MoxR->vWA+APATPase+wHTH+TPRs*->AB_hydrolase->CASPASE+EAD2->FXSXX-COOH-like->METALLOPROTEASE+TPRs->

■ **GCA_001905795.1; Streptomyces sp. CB01580; counts= 1; multicellularity status: TRUE**

- <-TCAD15||Trypco1->Trypsin+HDxxH+VMAP-M0+VMAP-C->MoxR->vWA+TPRs*->FXSXX-COOH->Radical_SAM->METALLOPROTEASE+TPRs->TIR+APATPase+TPRs->

■ **GCA_001905645.1; Streptomyces sp. CB02115; counts= 1; multicellularity status: TRUE**

- EAD2+VMAP-M0+VMAP-C->MoxR->vWA+APATPase+wHTH+TPRs*->AB_hydrolase->CASPASE+EAD2->FXSXX-COOH-like->METALLOPROTEASE+TPRs->

■ **GCA_001905845.1; Streptomyces sp. TSRI0107; counts= 1; multicellularity status: TRUE**

- EAD2+VMAP-M0+VMAP-C->MoxR->vWA+APATPase+wHTH+TPRs*->||<-Na_H_Exchanger||AB_hydrolase->CASPASE+EAD2->FXSXX-COOH-like->METALLOPROTEASE+TPRs->

■ **GCA_001905885.1; Streptomyces sp. CB03234; counts= 1; multicellularity status: TRUE**

- AB_hydrolase->EAD2+VMAP-M0+VMAP-C->MoxR->vWA+APATPase+wHTH+TPRs*->||<-METALLOPROTEASE+TPRs<-FXSXX-COOH-like

■ **GCA_001905445.1; Streptomyces sp. CB00455; counts= 1; multicellularity status: TRUE**

- <-TCAD15||Trypsin+HDxxH+VMAP-M0+VMAP-C->MoxR->vWA+TPRs*->FXSXX-COOH->Radical_SAM->METALLOPROTEASE+TPRs->TIR+APATPase+TPRs->

■ **GCA_001918255.1; Ktedonobacter sp. 13_1_40CM_4_52_4; counts= 1; multicellularity status: TRUE**

- MoxR->vWA+Glyco_transf_20*->

■ **GCA_001995215.1; Amycolatopsis azurea DSM 43854; counts= 1; multicellularity status: TRUE**

- cNMP_cyclase+VMAP-M0+VMAP-C->MoxR->vWA+APATPase+wHTH+TPRs*->||<-METALLOPROTEASE+TPRs<-FXSXX-COOH-like<-cNMP_binding+HTH_Crp_2

■ **GCA_002085445.1; Beggiatoa sp. 4572_84; counts= 1; multicellularity status: TRUE**

- VMAP-C(partial)->VMAP-C(partial)->?->?->?->MoxR->vWA_protein*->

■ **GCA_002104855.1; Streptomyces sp. 4F; counts= 1; multicellularity status: TRUE**

- AB_hydrolase->CASPASE+EAD2->VMAP-M0+VMAP-C->MoxR->vWA+TPRs*->

■ **GCA_002104865.1; Streptomyces sp. BF-3; counts= 1; multicellularity status: TRUE**

- vWA(partial)+REase+STAND_NTPase+TPRs+cNMP_cyclase*->

■ **GCA_002148405.1; Alkalinema sp. CACIAM 70d; counts= 1; multicellularity status: TRUE**

- Trypsin+VMAP-M1+VMAP-C->MoxR->vWA_protein*->FGS-fold_domain->FGS-fold_domain->

■ **GCA_002300485.1; Streptomyces sp. Ag82_O1-15; counts= 1; multicellularity status: TRUE**

- AB_hydrolase->CASPASE+VMAP-M0+VMAP-C->MoxR->vWA+cNMP_binding+HTH_Crp_2+TerD*->

■ **GCA_002532535.1; Chloroflexi bacterium Kir15-3F; counts= 1; multicellularity status: TRUE**

- vWA+Pkinase+REC+TCAD9+APHkinase*->

■ **GCA_002803145.1; Kitasatospora sp. CB02891; counts= 1; multicellularity status: TRUE**

- Trypco1->Trypsin+EAD2+VMAP-M0+VMAP-C->MoxR->vWA+BetaPropeller+Pkinase+TIR*->

■ **GCA_900090065.1; Streptomyces sp. OspMP-M45; counts= 1; multicellularity status: TRUE**

- EAD2+VMAP-M0+VMAP-C->MoxR->vWA+APATPase+wHTH+TPRs*->AB_hydrolase->CASPASE+EAD2->FXSXX-COOH-like->METALLOPROTEASE+TPRs->

■ **GCA_900090085.1; Streptomyces sp. Ncost-T6T-1; counts= 1; multicellularity status: TRUE**

- DUF397->Trypco1->Trypsin+EAD2+VMAP-M0+VMAP-C->MoxR->vWA+REase+STAND_NTPase+SFI-helicase*->

■ **GCA_900091765.1; Streptomyces sp. PalvLS-984; counts= 1; multicellularity status: TRUE**

- EAD2+VMAP-M0+VMAP-C->MoxR->vWA+APATPase+wHTH+TPRs*->AB_hydrolase->CASPASE+EAD2->FXSXX-COOH-like->METALLOPROTEASE+TPRs->

■ **GCA_900091835.1; Streptomyces sp. PpalLS-921; counts= 1; multicellularity status: TRUE**

- EAD2+VMAP-M0+VMAP-C->MoxR->vWA+APATPase+wHTH+TPRs*->AB_hydrolase->CASPASE+EAD2->FXSXX-COOH-like->METALLOPROTEASE+TPRs->

■ **GCA_900091875.1; Streptomyces sp. BpilaLS-43; counts= 1; multicellularity status: TRUE**

- EAD2+VMAP-M0+VMAP-C->MoxR->?->vWA+APATPase+wHTH+TPRs*->AB_hydrolase->

■ **GCA_900091865.1; Streptomyces sp. DvalAA-19; counts= 1; multicellularity status: TRUE**

- AB_hydrolase->CASPASE+VMAP-M2+VMAP-C->MoxR->vWA+REase+SWACOS+TPRs+cNMP_cyclase*->

■ **GCA_900091755.1; Streptomyces sp. TverLS-915; counts= 1; multicellularity status: TRUE**

- VMAP-C->MoxR->vWA_protein*->

■ **GCA_900091785.1; Streptomyces sp. BvitLS-983; counts= 1; multicellularity status: TRUE**

- vWA+TPRs*->FXSXX-COOH->Radical_SAM->

■ **GCA_900091885.1; Streptomyces sp. ScaeMP-6W; counts= 1; multicellularity status: TRUE**

- <-TCAD15||Trypco1->Trypsin+HDxxH+VMAP-M0+VMAP-C->MoxR->vWA+TPRs*->FXSXX-COOH->

■ **GCA_900091845.1; Streptomyces sp. SolWspMP-5a-2; counts= 1; multicellularity status: TRUE**

- vWA+APATPase+wHTH+TPRs*->||<-METALLOPROTEASE+TPRs<-FXSXX-COOH-like||Trypco1->

■ **GCA_900091855.1; Streptomyces sp. DvalAA-14; counts= 1; multicellularity status: TRUE**

- vWA+TPRs*->

■ **GCA_900091905.1; Streptomyces sp. OspMP-M43; counts= 1; multicellularity status: TRUE**

- AB_hydrolase->CASPASE+VMAP-M2+VMAP-C->MoxR->vWA+TPRs*->

■ **GCA_900091725.1; Streptomyces sp. DvalAA-43; counts= 1; multicellularity status: TRUE**

- <-TCAD15||Trypco1->Trypsin+HDxxH+VMAP-M0+VMAP-C->MoxR->vWA+TPRs*->FXSXX-COOH->Radical_SAM->METALLOPROTEASE+TPRs->TIR+APATPase+TPRs->Trypco1->

■ **GCA_900091595.1; Micromonospora haikouensis; counts= 1; multicellularity status: TRUE**

- Trypsin+HDxxH+VMAP-M0+VMAP-C->MoxR->vWA+TPRs*->?->Radical_SAM->TIR+APATPase+TPRs->||<-Voltage_CLC

■ **GCA_900091525.1; Micromonospora matsumotoense; counts= 1; multicellularity status: TRUE**

- CASPASE(partial)+VMAP-M2+VMAP-C->MoxR->vWA_protein*->

■ **GCA_900091485.1; Streptomyces sp. LcepLS; counts= 1; multicellularity status: TRUE**

- VMAP-C->MoxR->?->vWA+TPRs*->FXSXX-COOH->Radical_SAM->METALLOPROTEASE+TPRs->TIR+APATPase+TPRs->Trypco1->

■ **GCA_900091995.1; Streptomyces sp. MnatMP-M17; counts= 1; multicellularity status: TRUE**

- AB_hydrolase->CASPASE+VMAP-M2+VMAP-C->MoxR->vWA+TIR+FxsC_Cter*->

■ **GCA_900092005.1; Streptomyces sp. Cmuel-A718b; counts= 1; multicellularity status: TRUE**

- AB_hydrolase->CASPASE+VMAP-M2+VMAP-C->MoxR->vWA+REase+SWACOS+TPRs+cNMP_cyclase*->

■ **GCA_900091985.1; Streptomyces sp. DconLS; counts= 1; multicellularity status: TRUE**

- vWA+Pkinase+cNMP_cyclase+Band7*->

■ **GCA_900091445.1; Micromonospora echinofusca; counts= 1; multicellularity status: TRUE**

- Trypco1->Trypsin+VMAP-M8+VMAP-C->MoxR->vWA+SWACOS+TPRs*->

■ **GCA_900090145.1; Streptomyces sp. WMMB 322; counts= 1; multicellularity status: TRUE**

- <-TCAD15||Trypco1->Trypsin+HDxxH+VMAP-M0+VMAP-C->MoxR->vWA+TPRs*->FXSXX-COOH->METALLOPROTEASE+TPRs->TIR+APATPase+TPRs->

■ **GCA_900090165.1; Streptomyces; counts= 1; multicellularity status: TRUE**

- Trypco1->Trypsin+VMAP-M0+VMAP-C->MoxR->vWA+Pkinase+wHTH+TPRs+cNMP_cyclase*->

■ **GCA_900091415.1; Micromonospora inyonensis; counts= 1; multicellularity status: TRUE**

- Trypco1->Trypsin+HDxxH+VMAP-M0+VMAP-C->MoxR->vWA+Calcineurin-like+NACHT*->

■ **GCA_900091585.1; Micromonospora nigra; counts= 1; multicellularity status: TRUE**

- EAD2+VMAP-M0+VMAP-C->MoxR->vWA+APATPase+wHTH+TPRs*->||<-METALLOPROTEASE+TPRs<-FXSXX-COOH-like

■ **GCA_900095575.1; Streptomyces sp. DpondAA-F4; counts= 1; multicellularity status: TRUE**

- EAD2+VMAP-M0+VMAP-C->MoxR->vWA+APATPase+wHTH+TPRs*->AB_hydrolase->

■ **GCA_900101975.1; Streptomyces sp. 136MFCol5.1; counts= 1; multicellularity status: TRUE**

- <-TCAD15||Trypco1->Trypsin+HDxxH+VMAP-M0+VMAP-C->MoxR->vWA+TPRs*->FXSXX-COOH->Radical_SAM->METALLOPROTEASE+TPRs->TIR+APATPase+TPRs->

■ **GCA_900103675.1; Janthinobacterium sp. 551a; counts= 1; multicellularity status: FALSE**

- AB_hydrolase->CASPASE+VMAP-M4+VMAP-C->MoxR->vWA+Calcineurin-like+APATPase+SFII-helicase*->

■ **GCA_900101585.1; Streptomyces emeiensis; counts= 1; multicellularity status: TRUE**

- AB_hydrolase->CASPASE+VMAP-M2+VMAP-C->MoxR->vWA+SFII-helicase+Trypsin+cNMP_cyclase*->

■ **GCA_900100955.1; Lechevalieria fradiae; counts= 1; multicellularity status: TRUE**

- cNMP_cyclase+VMAP-M0+VMAP-C->MoxR->vWA+APATPase+wHTH+TPRs*->||<-METALLOPROTEASE+TPRs<-FXSXX-COOH-like

■ **GCA_900102095.1; Streptomyces jietaisiensis; counts= 1; multicellularity status: TRUE**

- AB_hydrolase->CASPASE+VMAP-M0+VMAP-C->MoxR->vWA+STAND_NTPase+Trypsin+CASPASE+S1COLD*->

■ **GCA_900100315.1; Streptomyces indicus; counts= 1; multicellularity status: TRUE**

- Trypco1->Trypsin+VMAP-M0+VMAP-C->MoxR->vWA+Pkinase+wHTH+TPRs+cNMP_cyclase+Band7+HAD*->

■ **GCA_900100275.1; Lentzea violacea; counts= 1; multicellularity status: TRUE**

- <-cNMP_binding+HTH_Crp_2||cNMP_cyclase+EAD2+VMAP-M0+VMAP-C->MoxR->vWA+APATPase+wHTH+TPRs*->||<-METALLOPROTEASE+TPRs<-FXSXX-COOH-like||?->?->TIR+TPRs->

■ **GCA_900103985.1; Streptomyces guanduensis; counts= 1; multicellularity status: TRUE**

- PNP_UDP_1+EAD2->EAD2+VMAP-M0+VMAP-C->MoxR->vWA+APATPase+wHTH+TPRs*->||<-METALLOPROTEASE+TPRs<-FXSXX-COOH-like

■ **GCA_900102245.1; Streptomyces sp. KS_16; counts= 1; multicellularity status: TRUE**

- <-TCAD15||Trypco1->Trypsin+HDxxH+VMAP-M0+VMAP-C->MoxR->vWA+TPRs*->FXSXX-COOH->Radical_SAM->TIR+APATPase+TPRs->

■ **GCA_900105395.1; Streptomyces sp. TLI_053; counts= 1; multicellularity status: TRUE**

- Trypco1->Trypsin+EAD2->EAD2+VMAP-M10+VMAP-C->MoxR->vWA+APATPase+SWACOS+wHTH+TPRs+Beta-rich-tail*->

■ **GCA_900105855.1; Amycolatopsis keratiniphila; counts= 1; multicellularity status: TRUE**

- cNMP_cyclase+VMAP-M0+VMAP-C->MoxR->vWA+APATPase+wHTH+TPRs*->||<-METALLOPROTEASE+TPRs<-FXSXX-COOH-like<-cNMP_binding+HTH_Crp_2

■ **GCA_900106925.1; Thiocapsa roseopersicina; counts= 1; multicellularity status: TRUE**

- AB_hydrolase->CASPASE+VMAP-M4+VMAP-C->MoxR->vWA+FGS-fold_domain+FGS-fold_domain+FGS-fold_domain*->

■ **GCA_900105915.1; Streptomyces sp. 2224.1; counts= 1; multicellularity status: TRUE**

- <-TCAD15||Trypco1->Trypsin+HDxxH+VMAP-M0+VMAP-C->MoxR->vWA+TPRs*->FXSXX-COOH->Radical_SAM->TIR+APATPase+TPRs->

■ **GCA_900105415.1; Streptomyces sp. TLI_105; counts= 1; multicellularity status: TRUE**

- <-TCAD15||Trypco1->Trypsin+HDxxH+VMAP-M0+VMAP-C->MoxR->vWA+TPRs*->FXSXX-COOH->METALLOPROTEASE+TPRs->TIR+APATPase+TPRs->

■ **GCA_900105705.1; Streptomyces griseus; counts= 1; multicellularity status: TRUE**

- AB_hydrolase->CASPASE+VMAP-M2+VMAP-C->MoxR->vWA+TIR+FxsC_Cter*->

■ **GCA_900105775.1; Streptomyces sp. 2112.3; counts= 1; multicellularity status: TRUE**

- <-TCAD15||Trypco1->Trypsin+HDxxH+VMAP-M0+VMAP-C->MoxR->vWA+TPRs*->FXSXX-COOH->Radical_SAM->TIR+APATPase+TPRs->

■ **GCA_900108335.1; Nonomuraea solani; counts= 1; multicellularity status: TRUE**

- Trypsin+EAD2->EAD2+VMAP-M0+VMAP-C->MoxR->vWA+APATPase+wHTH+TPRs*->||<-METALLOPROTEASE+TPRs<-FXSXX-COOH-like

■ **GCA_900110255.1; Streptomyces rubidus; counts= 1; multicellularity status: TRUE**

- EAD2+VMAP-M0+VMAP-C->vWA+APATPase+wHTH+TPRs*->||<-METALLOPROTEASE+TPRs<-FXSXX-COOH-like<-Trypsin+EAD2<-Trypco2

■ **GCA_900110955.1; Lechevalieria xinjiangensis; counts= 1; multicellularity status: TRUE**

- <-cNMP_binding+HTH_Crp_2||cNMP_cyclase+VMAP-M0+VMAP-C->MoxR->vWA+APATPase+wHTH+TPRs*->||<-METALLOPROTEASE+TPRs<-FXSXX-COOH-like

■ **GCA_900111005.1; Lentzea albida; counts= 1; multicellularity status: TRUE**

- <-cNMP_binding+HTH_Crp_2||cNMP_cyclase+VMAP-M0+VMAP-C->MoxR->vWA+APATPase+wHTH+TPRs*->||<-METALLOPROTEASE+TPRs<-FXSXX-COOH-like

■ **GCA_900111175.1; Actinokineospora terrae; counts= 1; multicellularity status: TRUE**

- CASPASE+VMAP-M2+VMAP-C->MoxR->vWA+Trypsin+TCAD11+TPRs*->

■ **GCA_900110985.1; Lentzea flaviverrucosa; counts= 1; multicellularity status: TRUE**

- <-cNMP_binding+HTH_Crp_2||cNMP_cyclase+VMAP-M0+VMAP-C->MoxR->vWA+APATPase+wHTH+TPRs*->||<-METALLOPROTEASE+TPRs<-FXSXX-COOH-like

■ **GCA_900113015.1; Actinoplanes philippinensis; counts= 1; multicellularity status: TRUE**

- EAD2+VMAP-M0+VMAP-C->vWA+APATPase+wHTH+TPRs*->||<-METALLOPROTEASE+TPRs<-FXSXX-COOH-like

■ **GCA_900112845.1; Streptomyces alni; counts= 1; multicellularity status: TRUE**

- <-TCAD15||Trypsin+HDxxH+VMAP-M0+VMAP-C->MoxR->vWA+TPRs*->FXSXX-COOH->Radical_SAM->METALLOPROTEASE+TPRs->TIR+APATPase+TPRs->

■ **GCA_900114705.1; Rugamonas rubra; counts= 1; multicellularity status: FALSE**

- AB_hydrolase->CASPASE+VMAP-M4+VMAP-C->MoxR->vWA+METALLOPROTEASE+TCAD8*->

■ **GCA_900116325.1; Streptomyces sp. ok210; counts= 1; multicellularity status: TRUE**

- <-TCAD15||Trypco1->Trypsin+HDxxH+VMAP-M0+VMAP-C->MoxR->vWA+TPRs*->FXSXX-COOH->Radical_SAM->METALLOPROTEASE+TPRs->TIR+APATPase+TPRs->

■ **GCA_900116135.1; Saccharopolyspora flava; counts= 1; multicellularity status: TRUE**

- EAD2+VMAP-M0+VMAP-C->MoxR->vWA_protein*->TIR+APATPase+TPRs->||<-METALLOPROTEASE+TPRs<-FXSXX-COOH-like<-TIR+EAD2

■ **GCA_900116145.1; Streptomyces harbinensis; counts= 1; multicellularity status: TRUE**

- <-TCAD15||Trypco1->Trypsin+HDxxH+VMAP-M0+VMAP-C->MoxR->vWA+TPRs*->FXSXX-COOH->Radical_SAM->METALLOPROTEASE+TPRs->TIR+APATPase+TPRs->

■ **GCA_900116675.1; Bradyrhizobium arachidis; counts= 1; multicellularity status: TRUE**

- AB_hydrolase->CASPASE+VMAP-M4+VMAP-C->MoxR->vWA+CASPASE+TPRs*->

■ **GCA_900119165.1; Amycolatopsis australiensis; counts= 1; multicellularity status: TRUE**

- EAD2+VMAP-M0+VMAP-C->MoxR->vWA+APATPase+wHTH+TPRs*->||<-METALLOPROTEASE+TPRs<-FXSXX-COOH-like

■ **GCA_900119365.1; Streptomyces atratus; counts= 1; multicellularity status: TRUE**

- Trypco1->Trypsin+HDxxH+VMAP-M0+VMAP-C->MoxR->vWA+TPRs*->FXSXX-COOH->Radical_SAM->METALLOPROTEASE+TPRs->TIR+APATPase+TPRs->Trypco1->

■ **GCA_900142575.1; Streptomyces paucisporeus; counts= 1; multicellularity status: TRUE**

- <-TCAD15||Trypsin+HDxxH+VMAP-M0+VMAP-C->MoxR->vWA+TPRs*->FXSXX-COOH->Radical_SAM->METALLOPROTEASE+TPRs->TIR+APATPase+TPRs->

■ **GCA_900176515.1; Kibdelosporangium aridum; counts= 1; multicellularity status: TRUE**

- cNMP_cyclase+VMAP-M0+VMAP-C->MoxR->vWA+APATPase+wHTH+TPRs*->||<-METALLOPROTEASE+TPRs

■ **GCA_900230275.1; Streptomyces sp. OV198; counts= 1; multicellularity status: TRUE**

- AB_hydrolase->CASPASE+VMAP-M0+VMAP-C->MoxR->vWA+cNMP_binding+HTH_Crp_2+TerD*->

■ **GCA_000332585.1; Microcystis aeruginosa; counts= 1; multicellularity status: TRUE**

- HTH+VMAP-M9+VMAP-C->MoxR->vWA+FGS-fold_domain*->

■ **GCA_000312205.1; Microcystis aeruginosa; counts= 1; multicellularity status: TRUE**

- AB_hydrolase->CASPASE+VMAP-M14+VMAP-C->MoxR->vWA+FGS-fold_domain*->

■ **GCA_000388135.1; Amycolatopsis vancoresmycina; counts= 1; multicellularity status: TRUE**

- cNMP_cyclase+EAD2->EAD2+VMAP-M0+VMAP-C->MoxR->vWA+APATPase+wHTH+TPRs*->||<-METALLOPROTEASE+TPRs

■ **GCA_000340415.1; Amycolatopsis azurea; counts= 1; multicellularity status: TRUE**

- cNMP_cyclase+VMAP-M0+VMAP-C->MoxR->vWA+APATPase+wHTH+TPRs*->||<-METALLOPROTEASE+TPRs<-FXSXX-COOH-like<-cNMP_binding+HTH_Crp_2

■ **GCA_000155555.1; Coleofasciculus chthonoplastes; counts= 1; multicellularity status: TRUE**

- EAD9+EAD1+VMAP-C->?->MoxR->||<-?||?->?->vWA+FGS-fold_domain*->FGS-fold_domain->||<-?<-?||FGS-fold_domain->FGS-fold_domain->?->FGS-fold_domain->

■ **GCA_000307915.1; Arthrospira platensis; counts= 1; multicellularity status: TRUE**

- VMAP-C_protein->MoxR->vWA+TPRs*->

■ **GCA_000173555.1; Arthrospira maxima; counts= 1; multicellularity status: TRUE**

- VMAP-C_protein->MoxR->vWA+TPRs+CASPASE*->

■ **GCA_000241835.2; Streptomyces coelicoflavus; counts= 1; multicellularity status: TRUE**

- EAD2+VMAP-M0+VMAP-C->MoxR->vWA+APATPase+wHTH+TPRs*->||<-Na_H_Exchanger

■ **GCF_000235425.2; Frankia sp. CN3; counts= 1; multicellularity status: TRUE**

- EAD2+VMAP-M0+VMAP-C->MoxR->vWA+APATPase+wHTH+TPRs->||<-METALLOPROTEASE+TPRs||EAD2+VMAP-M0+VMAP-C->MoxR->vWA+APATPase+wHTH+TPRs*->||<-METALLOPROTEASE+TPRs

■ **GCA_000213055.1; Streptomyces sp. Tu6071; counts= 1; multicellularity status: TRUE**

- <-TCAD15||Trypsin+HDxxH+VMAP-M0+VMAP-C->MoxR->vWA+TPRs*->FXSXX-COOH->Radical_SAM->METALLOPROTEASE+TPRs->TIR+APATPase+TPRs->Trypco1->

■ **GCA_000176895.2; Arthrospira sp. PCC 8005; counts= 1; multicellularity status: TRUE**

- VMAP-C_protein->MoxR->vWA+TPRs+CASPASE*->

■ **GCA_000297435.1; Microcystis sp. T1-4; counts= 1; multicellularity status: TRUE**

- AB_hydrolase->CASPASE+VMAP-M14+VMAP-C->MoxR->vWA+FGS-fold_domain*->

■ **GCA_900091815.1; Streptomyces sp. SA3_actG; counts= 1; multicellularity status: TRUE**

- <-TCAD15||Trypsin+HDxxH+VMAP-M0+VMAP-C->MoxR->vWA+TPRs*->FXSXX-COOH->Radical_SAM->METALLOPROTEASE+TPRs->TIR+APATPase+TPRs->Trypco1->

■ **GCA_002846475.1; Saccharopolyspora spinosa; counts= 1; multicellularity status: TRUE**

- S1COLD->PSE->?->PSE->||<-?||?->Trypco1->Trypsin+HDxxH+VMAP-M0+VMAP-C->MoxR->vWA+TPRs*->Voltage_CLC->FXSXX-COOH-like->Radical_SAM->METALLOPROTEASE+TPRs->TIR+APATPase+TPRs->

■ **GCF_000258595.1; Streptomyces somaliensis; counts= 1; multicellularity status: TRUE**

- Trypco1->AB_hydrolase->CASPASE+VMAP-M2+VMAP-C->MoxR->vWA+APATPase+wHTH+TPRs+cNMP_cyclase+NaeI*->

■ **GCA_000009765.2; Streptomyces avermitilis; counts= 1; multicellularity status: TRUE**

- AB_hydrolase->CASPASE+VMAP-M2+VMAP-C->MoxR->vWA+APATPase+wHTH+TPRs+cNMP_cyclase+NaeI*->

■ **GCA_000058485.1; Frankia alni; counts= 1; multicellularity status: TRUE**

- EAD2->EAD2+VMAP-M0+VMAP-C->MoxR->vWA+APATPase+wHTH+TPRs*->||<-METALLOPROTEASE+TPRs

■ **GCA_000196835.1; Amycolatopsis mediterranei; counts= 1; multicellularity status: TRUE**

- cNMP_cyclase+EAD2->EAD2+VMAP-M0+VMAP-C->MoxR->vWA+APATPase+wHTH+TPRs*->||<-METALLOPROTEASE+TPRs<-FXSXX-COOH-like

■ **GCA_000166135.1; Frankia inefficax; counts= 1; multicellularity status: TRUE**

- EAD2+VMAP-M0+VMAP-C->MoxR->vWA+APATPase+wHTH+TPRs*->EAD2+EAD1->

■ **GCA_000269985.1; Kitasatospora setae; counts= 1; multicellularity status: TRUE**

- Trypco1->Trypsin+HDxxH+VMAP-M0+VMAP-C->MoxR->vWA+CASPASE+TCAD14+PIN+CCCH(ZNR)+FHA*->

■ **GCA_000210375.1; Arthrospira platensis; counts= 1; multicellularity status: TRUE**

- VMAP-C_protein->MoxR->vWA+TPRs+CASPASE*->

■ **GCA_000316605.1; Leptolyngbya sp. PCC 7376; counts= 1; multicellularity status: TRUE**

- FGS-fold_domain->||<-RVT_1<-four_helix_bundle_protein||FGS-fold_domain->PSE->Trypsin+VMAP-M1+VMAP-C->MoxR->vWA+GAF+FGS-fold_domain*->

■ **GCA_000389965.1; Actinoplanes sp. N902-109; counts= 1; multicellularity status: TRUE**

- AB_hydrolase->CASPASE+VMAP-M2+VMAP-C->MoxR->vWA+TCAD13+NaeI+TPRs*->

■ **GCA_000401915.1; Thermobifida fusca; counts= 1; multicellularity status: TRUE**

- vWA+APATPase+wHTH+TPRs*->||<-DUF397||Trypco1->

■ **GCA_000411495.1; Streptomyces; counts= 1; multicellularity status: TRUE**

- TCAD15->?->?->Trypco1->Trypsin+HDxxH+VMAP-M0+VMAP-C->MoxR->vWA+TPRs*->FXSXX-COOH-like->METALLOPROTEASE+TPRs->TIR+APATPase+TPRs->

■ **GCF_000262345.1; Streptomyces xiaopingdaonensis; counts= 1; multicellularity status: TRUE**

- <-Trypco1||Trypsin+HDxxH+VMAP-M0+VMAP-C->MoxR->vWA+TPRs*->FXSXX-COOH->METALLOPROTEASE+TPRs->TIR+APATPase+TPRs->

■ **GCF_000308175.1; Pseudomonas fluorescens; counts= 1; multicellularity status: FALSE**

- AB_hydrolase->CASPASE+VMAP-M16+VMAP-C->MoxR->vWA+BetaPropeller+TCAD10+REC*->

■ **GCF_000340965.1; Nocardiopsis kunsanensis; counts= 1; multicellularity status: TRUE**

- Trypsin+VMAP-M0+VMAP-C->vWA+APATPase+wHTH+TPRs*->||<-DUF397||Trypco1->

■ **GCF_000341085.1; Nocardiopsis ganjiahuensis; counts= 1; multicellularity status: TRUE**

- Trypsin+EAD2->EAD2+VMAP-M0+VMAP-C->MoxR->vWA+APATPase+wHTH+TPRs*->||<-DUF397

■ **GCF_000341125.1; Nocardiopsis lucentensis; counts= 1; multicellularity status: TRUE**

- vWA+APATPase+wHTH+TPRs*->||<-DUF397||Trypco1->

■ **GCF_000341185.1; Nocardiopsis chromatogenes; counts= 1; multicellularity status: TRUE**

- Trypsin+VMAP-M0+VMAP-C->MoxR->vWA+APATPase+wHTH+TPRs*->||<-DUF397||Trypco1->

■ **GCF_000375625.1; Streptomyces sp. FxanaC1; counts= 1; multicellularity status: TRUE**

- Trypco1->Trypsin+EAD2+VMAP-M0+VMAP-C->MoxR->vWA+wHTH+TPRs+APATPase+wHTH+TPRs*->?->?->||<-AB_hydrolase

- **GCF_000375725.1; Streptomyces; counts= 1; multicellularity status: TRUE**
  - <-TCAD15||Trypco1->Trypsin+HDxxH+VMAP-M0+VMAP-C->MoxR->vWA+TPRs*->FXSXX-COOH->Radical_SAM->METALLOPROTEASE+TPRs->TIR+APATPase+TPRs->Trypco1->
- **GCA_003688775.1; Streptomyces sp. LaPpAH-202; counts= 1; multicellularity status: TRUE**
  - <-TCAD15||Trypco1->Trypsin+HDxxH+VMAP-M0+VMAP-C->MoxR->vWA+TPRs*->FXSXX-COOH->Radical_SAM->METALLOPROTEASE+TPRs->TIR+APATPase+TPRs->
- **GCF_000373665.1; Streptomyces sp. BoleA5; counts= 1; multicellularity status: TRUE**
  - Trypco1->Trypsin+EAD2->EAD2+VMAP-M0+VMAP-C->MoxR->vWA+APATPase+wHTH+TPRs*->||<-METALLOPROTEASE+TPRs<-FXSXX-COOH-like
- **GCF_000377105.1; Streptomyces sp. CNQ766; counts= 1; multicellularity status: TRUE**
  - <-TCAD15||Trypco1->Trypsin+HDxxH+VMAP-M0+VMAP-C->MoxR->vWA+TPRs*->FXSXX-COOH->Radical_SAM->TIR+APATPase+TPRs->
- **GCF_000377545.1; Streptomyces sp. CNY228; counts= 1; multicellularity status: TRUE**
  - <-TCAD15||Trypco1->Trypsin+HDxxH+VMAP-M0+VMAP-C->MoxR->vWA+TPRs*->FXSXX-COOH->Radical_SAM->METALLOPROTEASE+TPRs->TIR+APATPase+TPRs->
- **GCA_003984925.1; Streptomyces sp. TOR3209; counts= 1; multicellularity status: TRUE**
  - EAD2+VMAP-M0+VMAP-C->MoxR->vWA+APATPase+wHTH+TPRs*->||<-Na_H_Exchanger
- **GCF_000280905.1; Streptomyces sp. AA1529; counts= 1; multicellularity status: TRUE**
  - Trypco1->Trypsin+HDxxH+VMAP-M0+VMAP-C->MoxR->vWA+TPRs*->FXSXX-COOH-like->METALLOPROTEASE+TPRs->TIR+APATPase+TPRs->
- **GCF_000297635.1; Streptomyces sp. AA0539; counts= 1; multicellularity status: TRUE**
  - <-TCAD15||Trypco1->Trypsin+HDxxH+VMAP-M0+VMAP-C->MoxR->vWA+TPRs*->FXSXX-COOH->Radical_SAM->
- **GCF_000381025.1; Streptomyces sulphureus; counts= 1; multicellularity status: TRUE**
  - <-TCAD15<-?||?->||<-Trypco1||Trypsin+HDxxH+VMAP-M0+VMAP-C->MoxR->vWA+TPRs*->FXSXX-COOH->METALLOPROTEASE+TPRs->TIR+APATPase+TPRs->
- **GCF_000384175.1; Streptomyces purpureus; counts= 1; multicellularity status: TRUE**
  - <-TCAD15||Trypco1->Trypsin+HDxxH+VMAP-M0+VMAP-C->MoxR->vWA+TPRs*->Radical_SAM->TIR+APATPase+TPRs->
- **GCF_000379525.1; Lamprocystis purpurea; counts= 1; multicellularity status: TRUE**
  - CASPASE+VMAP-M27+VMAP-C->MoxR->vWA+FGS-fold_domain+FGS-fold_domain+PilP+FGS-fold_domain*->
- **GCA_002234385.1; Amycolatopsis alba; counts= 1; multicellularity status: TRUE**
  - cNMP_cyclase+VMAP-M0+VMAP-C->PSE->vWA+APATPase+wHTH+TPRs*->||<-METALLOPROTEASE+TPRs<-FXSXX-COOH-like<-cNMP_binding+HTH_Crp_2
- **GCF_000384295.1; Amycolatopsis balhimycina; counts= 1; multicellularity status: TRUE**
  - <-Trypco1<-?||EAD2+VMAP-M0+VMAP-C->MoxR->vWA+APATPase+wHTH+TPRs*->||<-METALLOPROTEASE+TPRs
- **GCA_000478195.2; Lyngbya aestuarii; counts= 1; multicellularity status: TRUE**
  - Trypco1->Trypsin+VMAP-M1+VMAP-C->MoxR->vWA+FGS-fold_domain*->||<-?||FGS-fold_domain->
- **GCA_000482245.1; Leptolyngbya sp. Heron Island J; counts= 1; multicellularity status: TRUE**
  - FGS-fold_domain->||<-?||PNP_UDP_1+VMAP-M1+VMAP-C->MoxR->vWA+FGS-fold_domain*->FGS-fold_domain->

- **GCA_000478605.2; Streptomyces thermolilacinus; counts= 1; multicellularity status: TRUE**
  - Trypco1->?->?->AB_hydrolase->CASPASE+VMAP-M2+VMAP-C->MoxR->vWA+APATPase+wHTH+TPRs+cNMP_cyclase+NaeI*->
- **GCA_000508205.1; Pseudomonas; counts= 1; multicellularity status: FALSE**
  - AB_hydrolase->CASPASE+VMAP-M16+VMAP-C->MoxR->vWA+BetaPropeller+TCAD10+REC*->
- **GCF_000470535.1; Streptomyces sp. AW19M42; counts= 1; multicellularity status: TRUE**
  - AB_hydrolase->CASPASE+EAD2+VMAP-M0+VMAP-C->MoxR->vWA+TPRs*->
- **GCF_000518045.1; Streptomyces exfoliatus; counts= 1; multicellularity status: TRUE**
  - Trypsin+EAD2->EAD2+VMAP-M0+VMAP-C->MoxR->vWA+APATPase+wHTH+TPRs*->||<-METALLOPROTEASE+TPRs
- **GCF_000239155.1; Actinoalloteichus spitiensis; counts= 1; multicellularity status: TRUE**
  - Trypco1->Trypsin+HDxxH+VMAP-M0+VMAP-C->MoxR->vWA+TPRs*->FXSXX-COOH-like->
- **GCF_000341205.1; Nocardiopsis baichengensis; counts= 1; multicellularity status: TRUE**
  - Trypsin+VMAP-M0+VMAP-C->MoxR->vWA+APATPase+wHTH+TPRs*->||<-DUF397||Trypco1->
- **GCF_000379645.1; Actinoplanes globisporus; counts= 1; multicellularity status: TRUE**
  - AB_hydrolase->CASPASE+VMAP-M2+VMAP-C->MoxR->vWA+CASPASE*->
- **GCF_000379825.1; Longispora albida; counts= 1; multicellularity status: TRUE**
  - Trypsin+HDxxH+VMAP-M0+VMAP-C->MoxR->vWA+TPRs+CASPASE*->
- **GCF_000372745.1; Streptomyces scabrisporus; counts= 1; multicellularity status: TRUE**
  - CASPASE+VMAP-M19->VMAP-C->MoxR->vWA+BetaPropeller*->
- **GCF_000373645.1; Streptomyces sp. ATexAB-D23; counts= 1; multicellularity status: TRUE**
  - <-TCAD15||Trypco1->Trypsin+HDxxH+VMAP-M0+VMAP-C->MoxR->vWA+TPRs*->FXSXX-COOH->Radical_SAM->METALLOPROTEASE+TPRs->TIR+APATPase+TPRs->Trypco1->
- **GCA_002895405.1; Actinoalloteichus cyanogriseus; counts= 1; multicellularity status: TRUE**
  - Trypco1->Trypsin+HDxxH+VMAP-M0+VMAP-C->MoxR->vWA+TPRs*->FXSXX-COOH-like->Radical_SAM->METALLOPROTEASE+TPRs->TIR+APATPase+TPRs->
- **GCF_000482865.1; Actinokineospora inagensis; counts= 1; multicellularity status: TRUE**
  - CASPASE+VMAP-M2+VMAP-C->MoxR->vWA+Trypsin+TCAD11*->
- **GCF_000428945.1; Hamadaea tsunoensis; counts= 1; multicellularity status: TRUE**
  - Trypco1->Trypsin+VMAP-M8+VMAP-C->MoxR->vWA+Trypsin+APATPase+wHTH+TPRs*->
- **GCF_000472685.1; Bradyrhizobium; counts= 1; multicellularity status: TRUE**
  - AB_hydrolase->CASPASE+VMAP-M4+VMAP-C->MoxR->vWA+CASPASE+TPRs*->
- **GCF_000514995.1; Streptomyces sp. CNT371; counts= 1; multicellularity status: TRUE**
  - <-TCAD15||Trypco1->Trypsin+HDxxH+VMAP-M0+VMAP-C->MoxR->vWA+TPRs*->FXSXX-COOH->Radical_SAM->TIR+APATPase+TPRs->

- **GCF_000514715.1; Streptomyces sp. CNH099; counts= 1; multicellularity status: TRUE**
  - <-TCAD15||Trypco1->Trypsin+HDxxH+VMAP-M0+VMAP-C->MoxR->vWA+TPRs*->FXSXX-COOH->Radical_SAM->TIR+APATPase+TPRs->
- **GCF_000424765.1; Streptomyces sp. CNT318; counts= 1; multicellularity status: TRUE**
  - Trypco1->Trypsin+HDxxH+VMAP-M0+VMAP-C->MoxR->vWA+TPRs*->FXSXX-COOH-like->METALLOPROTEASE+TPRs->TIR+APATPase+TPRs->
- **GCF_000426325.1; Streptomyces sp. CNS606; counts= 1; multicellularity status: TRUE**
  - AB_hydrolase->CASPASE+EAD2+VMAP-M0+VMAP-C->MoxR->vWA+TPRs*->
- **GCF_000482585.1; Streptomyces sp. CNQ329; counts= 1; multicellularity status: TRUE**
  - <-TCAD15||Trypco1->Trypsin+HDxxH+VMAP-M0+VMAP-C->MoxR->vWA+TPRs*->FXSXX-COOH->Radical_SAM->TIR+APATPase+TPRs->
- **GCA_900091745.1; Streptomyces sp. DpondAA-B6; counts= 1; multicellularity status: TRUE**
  - EAD2+VMAP-M0+VMAP-C->MoxR->vWA+APATPase+wHTH+TPRs*->AB_hydrolase->CASPASE+EAD2->FXSXX-COOH-like->METALLOPROTEASE+TPRs->
- **GCF_000383615.1; Streptomyces canus; counts= 1; multicellularity status: TRUE**
  - Trypco1->Trypsin+EAD2+VMAP-M0+VMAP-C->MoxR->vWA+TPRs*->
- **GCF_000429085.1; Streptomyces flavidovirens; counts= 1; multicellularity status: TRUE**
  - <-TCAD15||Trypco1->Trypsin+HDxxH+VMAP-M0+VMAP-C->MoxR->vWA+TPRs*->FXSXX-COOH->METALLOPROTEASE+TPRs->TIR+APATPase+TPRs->
- **GCF_000715845.1; Streptomyces monomycini; counts= 1; multicellularity status: TRUE**
  - <-TCAD15||Trypco1->Trypsin+HDxxH+VMAP-M0+VMAP-C->MoxR->vWA+TPRs*->FXSXX-COOH->Radical_SAM->TIR+APATPase+TPRs->
- **GCF_000716025.1; Streptomyces baarnensis; counts= 1; multicellularity status: TRUE**
  - EAD2+VMAP-M0+VMAP-C->MoxR->vWA+APATPase+wHTH+TPRs*->AB_hydrolase->CASPASE+EAD2->PSE->METALLOPROTEASE+TPRs->
- **GCF_000718945.1; Streptomyces sp. NRRL S-813; counts= 1; multicellularity status: TRUE**
  - Trypsin+EAD2->EAD2+VMAP-M0+VMAP-C->MoxR->vWA+APATPase+wHTH+TPRs*->||<-METALLOPROTEASE+TPRs<-FXSXX-COOH-like
- **GCA_001270665.1; Streptomyces; counts= 1; multicellularity status: TRUE**
  - <-TCAD15||Trypco1->Trypsin+HDxxH+VMAP-M0+VMAP-C->MoxR->vWA+TPRs*->FXSXX-COOH->Radical_SAM->TIR+APATPase+TPRs->
- **GCF_000719335.1; Streptomyces sp. NRRL S-87; counts= 1; multicellularity status: TRUE**
  - AB_hydrolase->CASPASE+VMAP-M0+VMAP-C->MoxR->vWA+Trypsin+cNMP_cyclase+TPRs*->
- **GCF_000717875.1; Streptomyces sp. NRRL S-455; counts= 1; multicellularity status: TRUE**
  - EAD2+VMAP-M0+VMAP-C->MoxR->vWA+APATPase+wHTH+TPRs*->||<-Na_H_Exchanger||AB_hydrolase->CASPASE+EAD2->FXSXX-COOH-like->METALLOPROTEASE+TPRs->
- **GCA_001509565.1; Streptomyces; counts= 1; multicellularity status: TRUE**
  - MoxR->vWA+TPRs*->FXSXX-COOH->Radical_SAM->METALLOPROTEASE+TPRs->TIR+APATPase+TPRs->
- **GCF_000718515.1; Micromonospora parva; counts= 1; multicellularity status: TRUE**
  - CASPASE+TPRs->EAD2+VMAP-M0+VMAP-C->MoxR->vWA+APATPase+wHTH+TPRs*->||<-METALLOPROTEASE+TPRs<-Radical_SAM<-FXSXX-COOH-like

- **GCA_003073355.1; Streptomyces scopuliridis; counts= 1; multicellularity status: TRUE**
  - <-TCAD15||Trypco1->Trypsin+HDxxH+VMAP-M0+VMAP-C->MoxR->vWA+TPRs*->FXSXX-COOH->Radical_SAM->METALLOPROTEASE+TPRs->TIR+APATPase+TPRs->
- **GCF_000721345.1; Kitasatospora sp. NRRL B-11411; counts= 1; multicellularity status: TRUE**
  - MoxR->vWA+CASPASE+TCAD14+PIN+CCCH(ZNR)+FHA*->
- **GCF_000719205.1; Lechevalieria aerocolonigenes; counts= 1; multicellularity status: TRUE**
  - cNMP_cyclase+VMAP-M0+VMAP-C->MoxR->vWA+APATPase+wHTH+TPRs*->||<-METALLOPROTEASE+TPRs<-FXSXX-COOH-like||TIR+APATPase+TPRs->||<-DUF397
- **GCF_000725545.1; Streptomyces exfoliatus; counts= 1; multicellularity status: TRUE**
  - <-TCAD15||Trypco1->Trypsin+HDxxH+VMAP-M0+VMAP-C->MoxR->vWA+TPRs*->FXSXX-COOH->METALLOPROTEASE+TPRs->TIR+APATPase+TPRs->
- **GCF_000720915.1; Streptomyces albus; counts= 1; multicellularity status: TRUE**
  - EAD2+VMAP-M0+VMAP-C->MoxR->vWA+APATPase+wHTH+TPRs*->AB_hydrolase->CASPASE+EAD2->PSE->METALLOPROTEASE+TPRs->
- **GCF_000720535.1; Streptomyces; counts= 1; multicellularity status: TRUE**
  - EAD2+VMAP-M0+VMAP-C->MoxR->vWA+APATPase+wHTH+TPRs*->AB_hydrolase->CASPASE+EAD2->FXSXX-COOH-like->METALLOPROTEASE+TPRs->
- **GCF_000716535.1; Streptomyces; counts= 1; multicellularity status: TRUE**
  - Trypco1->Trypsin+VMAP-M0+VMAP-C->MoxR->vWA+Pkinase+cNMP_cyclase+Band7+HAD*->
- **GCF_000718705.1; Streptomyces; counts= 1; multicellularity status: TRUE**
  - AB_hydrolase->CASPASE+VMAP-M0+VMAP-C->MoxR->vWA+Trypsin+cNMP_cyclase+CCCH(TrebleClef)+Pentapeptide_repeats*->
- **GCF_000721105.1; Streptomyces cellulosae; counts= 1; multicellularity status: TRUE**
  - EAD2+VMAP-M0+VMAP-C->MoxR->vWA+APATPase+wHTH+TPRs*->?->||<-Na_H_Exchanger||AB_hydrolase->CASPASE+EAD2->FXSXX-COOH-like->
- **GCF_000719495.1; Streptomyces griseus; counts= 1; multicellularity status: TRUE**
  - <-TCAD15||Trypco1->Trypsin+HDxxH+VMAP-M0+VMAP-C->MoxR->vWA+TPRs*->FXSXX-COOH->||<-?||Radical_SAM->METALLOPROTEASE+TPRs->TIR+APATPase+TPRs->
- **GCF_000717795.1; Streptomyces griseus; counts= 1; multicellularity status: TRUE**
  - AB_hydrolase->CASPASE+VMAP-M2+VMAP-C->MoxR->vWA_protein*->TIR+FxsC_Cter->
- **GCF_000719355.1; Streptomyces griseus; counts= 1; multicellularity status: TRUE**
  - EAD2+VMAP-M0+VMAP-C->MoxR->vWA+APATPase+wHTH+TPRs*->AB_hydrolase->CASPASE+EAD2->FXSXX-COOH-like->METALLOPROTEASE+TPRs->
- **GCF_000719775.1; Streptomyces sp. NRRL F-5135; counts= 1; multicellularity status: TRUE**
  - <-TCAD15||Trypco1->Trypsin+HDxxH+VMAP-M0+VMAP-C->?->MoxR->vWA+TPRs*->FXSXX-COOH->Radical_SAM->METALLOPROTEASE+TPRs->TIR+APATPase+TPRs->
- **GCF_000719435.1; Streptomyces griseus; counts= 1; multicellularity status: TRUE**
  - <-TCAD15||Trypco1->Trypsin+HDxxH+VMAP-M0+VMAP-C->MoxR->vWA+TPRs*->FXSXX-COOH->Radical_SAM->METALLOPROTEASE+TPRs->TIR+APATPase+TPRs->
- **GCF_000719895.1; Streptomyces sp. NRRL F-2799; counts= 1; multicellularity status: TRUE**
  - Trypsin+HDxxH+VMAP-M0+VMAP-C->MoxR->vWA+TPRs*->FXSXX-COOH->Radical_SAM->METALLOPROTEASE+TPRs->TIR+APATPase+TPRs->

- **GCA_001279005.1; Streptomyces; counts= 1; multicellularity status: TRUE**
  - Trypsin+EAD2->EAD2+VMAP-M0+VMAP-C->MoxR->vWA+APATPase+wHTH+TPRs*->||<-METALLOPROTEASE+TPRs<-Radical_SAM<-FXSXX-COOH||Voltage_CLC->
- **GCF_000721605.1; Streptomyces sp. NRRL S-37; counts= 1; multicellularity status: TRUE**
  - EAD2+VMAP-M0+VMAP-C->MoxR->vWA+APATPase+wHTH+TPRs*->||<-Na_H_Exchanger<-?||METALLOPROTEASE+TPRs->
- **GCF_000722015.1; Streptomyces sp. NRRL S-474; counts= 1; multicellularity status: TRUE**
  - EAD2+VMAP-M0+VMAP-C->MoxR->vWA+APATPase+wHTH+TPRs*->||<-Na_H_Exchanger||AB_hydrolase->CASPASE+EAD2->FXSXX-COOH-like->METALLOPROTEASE+TPRs->
- **GCF_000721265.1; Streptomyces sp. NRRL F-5126; counts= 1; multicellularity status: TRUE**
  - <-TCAD15||Trypco1->Trypsin+HDxxH+VMAP-M0+VMAP-C->MoxR->vWA+TPRs*->FXSXX-COOH->Radical_SAM->METALLOPROTEASE+TPRs->TIR+APATPase+TPRs->
- **GCF_000717895.1; Streptomyces sp. NRRL WC-3744; counts= 1; multicellularity status: TRUE**
  - Trypco1->Trypsin+VMAP-M0+VMAP-C->MoxR->vWA+Pkinase+cNMP_cyclase+Band7+HAD*->
- **GCF_000719475.1; Streptomyces sp. NRRL F-5630; counts= 1; multicellularity status: TRUE**
  - <-TCAD15||Trypsin+HDxxH+VMAP-M0+VMAP-C->MoxR->vWA+TPRs*->FXSXX-COOH->Radical_SAM->METALLOPROTEASE+TPRs->TIR+APATPase+TPRs->Trypco1->
- **GCF_000720295.1; Streptomyces sp. NRRL WC-3725; counts= 1; multicellularity status: TRUE**
  - Trypco1->Trypsin+VMAP-M0+VMAP-C->MoxR->vWA+Pkinase+cNMP_cyclase+Band7+HAD*->
- **GCF_000721935.1; Streptomyces sp. NRRL WC-3549; counts= 1; multicellularity status: TRUE**
  - EAD2+VMAP-M0+VMAP-C->MoxR->vWA+APATPase+wHTH+TPRs*->PSE->PSE->CASPASE+EAD2->FXSXX-COOH-like->METALLOPROTEASE+TPRs->
- **GCF_000722185.1; Streptomyces; counts= 1; multicellularity status: TRUE**
  - EAD2+VMAP-M0+VMAP-C->MoxR->vWA+APATPase+wHTH+TPRs*->AB_hydrolase->CASPASE+EAD2->FXSXX-COOH-like->METALLOPROTEASE+TPRs->
- **GCF_000725635.1; Streptomyces sp. NRRL B-3253; counts= 1; multicellularity status: TRUE**
  - <-TCAD15||Trypco1->Trypsin+HDxxH+VMAP-M0+VMAP-C->MoxR->vWA+TPRs*->FXSXX-COOH->Radical_SAM->METALLOPROTEASE+TPRs->TIR+APATPase+TPRs->
- **GCA_004328625.1; Streptomyces seoulensis; counts= 1; multicellularity status: TRUE**
  - <-TCAD15||PSE->Trypsin+HDxxH+VMAP-M0+VMAP-C->MoxR->vWA+TPRs*->FXSXX-COOH->Radical_SAM->METALLOPROTEASE+TPRs->TIR+APATPase+TPRs->
- **GCF_000725645.1; Streptomyces sp. NRRL F-5635; counts= 1; multicellularity status: TRUE**
  - <-TCAD15||Trypco1->Trypsin+HDxxH+VMAP-M0+VMAP-C->MoxR->vWA+TPRs*->FXSXX-COOH->Radical_SAM->METALLOPROTEASE+TPRs->TIR+APATPase+TPRs->Trypco1->
- **GCF_000156695.2; Streptomyces filamentosus; counts= 1; multicellularity status: TRUE**
  - EAD2+VMAP-M0+VMAP-C->MoxR->vWA+APATPase+wHTH+TPRs*->PSE->CASPASE+EAD2->FXSXX-COOH-like->METALLOPROTEASE+TPRs->
- **GCF_000373305.1; Streptomyces sp. CcalMP-8W; counts= 1; multicellularity status: TRUE**
  - Trypco1->Trypsin+EAD2+VMAP-M0+VMAP-C->MoxR->vWA+Pkinase+ParA-Soj-PloopNTPase(SIMIBI)*->
- **GCF_000156455.1; Streptomyces filamentosus; counts= 1; multicellularity status: TRUE**
  - EAD2+VMAP-M0+VMAP-C->MoxR->vWA+APATPase+wHTH+TPRs*->PSE->PSE->CASPASE+EAD2->FXSXX-COOH-like->

- **GCF_000717375.1; Streptomyces griseus; counts= 1; multicellularity status: TRUE**
  - <-TCAD15||Trypco1->Trypsin+HDxxH+VMAP-M0+VMAP-C->MoxR->vWA+TPRs*->FXSXX-COOH->Radical_SAM->METALLOPROTEASE+TPRs->TIR+APATPase+TPRs->
- **GCF_000717345.1; Streptomyces lydicus; counts= 1; multicellularity status: TRUE**
  - <-TCAD15||Trypco1->Trypsin+HDxxH+VMAP-M0+VMAP-C->MoxR->vWA+TPRs*->FXSXX-COOH->Radical_SAM->TIR+APATPase+TPRs->
- **GCF_000717025.1; Streptomyces atroolivaceus; counts= 1; multicellularity status: TRUE**
  - EAD2+VMAP-M0+VMAP-C->MoxR->vWA+APATPase+wHTH+TPRs*->AB_hydrolase->CASPASE+EAD2->FXSXX-COOH-like->METALLOPROTEASE+TPRs->
- **GCF_000717055.1; Streptomyces iakyrus; counts= 1; multicellularity status: TRUE**
  - Trypco1->Trypsin+VMAP-M0+VMAP-C->MoxR->vWA+Pkinase+wHTH+TPRs+cNMP_cyclase+Band7+HAD*->
- **GCF_000797385.1; Streptomyces sp. CNQ431; counts= 1; multicellularity status: TRUE**
  - <-TCAD15||Trypco1->Trypsin+HDxxH+VMAP-M0+VMAP-C->MoxR->vWA+TPRs*->FXSXX-COOH->Radical_SAM->METALLOPROTEASE+TPRs->TIR+APATPase+TPRs->
- **GCF_000504285.1; Actinospica robiniae; counts= 1; multicellularity status: TRUE**
  - PNP_UDP_1+VMAP-M0+VMAP-C->MoxR->vWA+APATPase+wHTH+TPRs*->||<-METALLOPROTEASE+TPRs||METALLOPROTEASE+TPRs->||<-PNP_UDP_1+EAD2<-EAD2+VMAP-M0+APATPase+TPRs
- **GCF_000744785.1; Kitasatospora azatica; counts= 1; multicellularity status: TRUE**
  - Trypco1->Trypsin+EAD2->EAD2+VMAP-M10+VMAP-C->MoxR->vWA+APATPase+BetaPropeller+cNMP_cyclase+NaeI*->
- **GCA_000696185.1; Kitasatospora cheerisanensis; counts= 1; multicellularity status: TRUE**
  - AB_hydrolase->CASPASE+VMAP-M2+VMAP-C->MoxR->vWA_protein*->
- **GCA_900176525.1; Lentzea albidocapillata; counts= 1; multicellularity status: TRUE**
  - <-cNMP_binding+HTH_Crp_2||cNMP_cyclase+EAD2+VMAP-M0+VMAP-C->MoxR->vWA+APATPase+wHTH+TPRs*->||<-METALLOPROTEASE+TPRs<-FXSXX-COOH-like||TIR+TPRs->
- **GCA_004768505.1; Streptomyces albus; counts= 1; multicellularity status: TRUE**
  - TCAD15->?->?->Trypco1->Trypsin+HDxxH+VMAP-M0+VMAP-C->MoxR->vWA+TPRs*->FXSXX-COOH-like->METALLOPROTEASE+TPRs->TIR+APATPase+TPRs->
- **GCF_000415505.1; Streptomyces afghaniensis; counts= 1; multicellularity status: TRUE**
  - EAD2+VMAP-M0+VMAP-C->PSE->vWA+APATPase+wHTH+TPRs*->
- **GCA_000738695.1; Streptomyces scabiei; counts= 1; multicellularity status: TRUE**
  - vWA_protein*->
- **GCA_000738715.1; Streptomyces scabiei; counts= 1; multicellularity status: TRUE**
  - TCAD9+Pkinase+NACHT+HEAT_repeats->EAD2+VMAP-M0+VMAP-C->vWA*->MoxR->vWA+APATPase+TPRs->
- **GCF_000746395.1; Streptomyces mirabilis; counts= 1; multicellularity status: TRUE**
  - EAD2+VMAP-M0+VMAP-C->MoxR->vWA+APATPase+wHTH+TPRs*->||<-Na_H_Exchanger||AB_hydrolase->CASPASE+EAD2->FXSXX-COOH-like->METALLOPROTEASE+TPRs->
- **GCF_000701285.1; Streptomyces sp. CNQ-525; counts= 1; multicellularity status: TRUE**
  - <-TCAD15||Trypco1->Trypsin+HDxxH+VMAP-M0+VMAP-C->MoxR->vWA+TPRs*->FXSXX-COOH->Radical_SAM->TIR+APATPase+TPRs->

■ **GCA_000696115.1; Streptomyces olindensis; counts= 1; multicellularity status: TRUE**

- EAD2+VMAP-M0+VMAP-C->MoxR->vWA+APATPase+wHTH+TPRs*->AB_hydrolase->CASPASE+EAD2->FXSXX-COOH-like->METALLOPROTEASE+TPRs->

■ **GCF_000495635.1; Streptomyces sp. GBA 94-10; counts= 1; multicellularity status: TRUE**

- <-TCAD15||Trypco1->Trypsin+HDxxH+VMAP-M0+VMAP-C->MoxR->vWA+TPRs*->FXSXX-COOH->Radical_SAM->METALLOPROTEASE+TPRs->TIR+APATPase+TPRs->

■ **GCA_000412265.2; Streptomyces sp. Mg1; counts= 1; multicellularity status: TRUE**

- <-cNMP_binding+HTH_Crp_2<-?||EAD2+VMAP-M0+VMAP-C->MoxR->vWA+APATPase+wHTH+TPRs*->||<-Na_H_Exchanger||AB_hydrolase->CASPASE+EAD2->FXSXX-COOH-like->METALLOPROTEASE+TPRs->

■ **GCF_000719665.1; Streptomyces; counts= 1; multicellularity status: TRUE**

- <-TCAD15||Trypco1->Trypsin+HDxxH+VMAP-M0+VMAP-C->MoxR->vWA+TPRs*->FXSXX-COOH->Radical_SAM->METALLOPROTEASE+TPRs->TIR+APATPase+TPRs->Trypco1->

■ **GCF_000721335.1; Streptomyces sp. NRRL S-1831; counts= 1; multicellularity status: TRUE**

- <-TCAD15||Trypco1->Trypsin+HDxxH+VMAP-M0+VMAP-C->MoxR->vWA+TPRs*->FXSXX-COOH->Radical_SAM->METALLOPROTEASE+TPRs->TIR+APATPase+TPRs->Trypco1->

■ **GCF_000720175.1; Streptomyces sp. NRRL S-337; counts= 1; multicellularity status: TRUE**

- <-TCAD15||PSE->Trypsin+HDxxH+VMAP-M0+VMAP-C->MoxR->vWA+TPRs*->FXSXX-COOH->Radical_SAM->TIR+APATPase+TPRs->

■ **GCF_000721455.1; Streptomyces sp. NRRL F-6628; counts= 1; multicellularity status: TRUE**

- <-TCAD15||Trypco1->Trypsin+HDxxH+VMAP-M0+VMAP-C->MoxR->vWA+TPRs*->FXSXX-COOH->Radical_SAM->METALLOPROTEASE+TPRs->TIR+APATPase+TPRs->

■ **GCA_000698495.1; Streptomyces sp. NTK 937; counts= 1; multicellularity status: TRUE**

- EAD2+VMAP-M0+VMAP-C->MoxR->vWA+APATPase+wHTH+TPRs*->AB_hydrolase->CASPASE+EAD2->FXSXX-COOH-like->METALLOPROTEASE+TPRs->

■ **GCF_000495755.1; Streptomyces sp. PVA 94-07; counts= 1; multicellularity status: TRUE**

- <-TCAD15||Trypco1->Trypsin+HDxxH+VMAP-M0+VMAP-C->MoxR->vWA+TPRs*->FXSXX-COOH->Radical_SAM->METALLOPROTEASE+TPRs->TIR+APATPase+TPRs->

■ **GCA_000802245.2; Streptomyces; counts= 1; multicellularity status: TRUE**

- TCAD15->?->Trypco1->Trypsin+HDxxH+VMAP-M0+VMAP-C->MoxR->vWA+TPRs*->FXSXX-COOH-like->METALLOPROTEASE+TPRs->TIR+APATPase+TPRs->

■ **GCA_004212215.1; Lyngbya confervoides; counts= 1; multicellularity status: TRUE**

- Trypco1->Trypsin+VMAP-M1+VMAP-C->MoxR->vWA+FGS-fold_domain*->?->FGS-fold_domain->

■ **GCF_000308675.1; Nocardia paucivorans; counts= 1; multicellularity status: TRUE**

- Trypco1->Trypsin+HDxxH+VMAP-M0+VMAP-C->MoxR->vWA+TPRs*->Voltage_CLC->METALLOPROTEASE->TIR+APATPase+wHTH+TPRs->

■ **GCF_000308495.1; Nocardia brevicatena; counts= 1; multicellularity status: TRUE**

- Trypsin+HDxxH+VMAP-M0+VMAP-C->MoxR->vWA+TPRs*->Voltage_CLC->METALLOPROTEASE->TIR+APATPase+wHTH+TPRs->

■ **GCA_003182435.1; Streptomyces albus; counts= 1; multicellularity status: TRUE**

- <-TCAD15||Trypco1->Trypsin+HDxxH+VMAP-M0+VMAP-C->MoxR->vWA+TPRs*->FXSXX-COOH->Radical_SAM->METALLOPROTEASE+TPRs->TIR+APATPase+TPRs->

■ **GCA_000818175.1; Streptomyces sp. AcH 505; counts= 1; multicellularity status: TRUE**

- EAD2+VMAP-M0+VMAP-C->MoxR->vWA+APATPase+wHTH+TPRs*->||<-Na_H_Exchanger||AB_hydrolase->CASPASE+EAD2->FXSXX-COOH-like->

- **GCA_900109465.1; Streptacidiphilus jiangxiensis; counts= 1; multicellularity status: TRUE**
  - <-TCAD15||Trypco1->Trypsin+HDxxH+VMAP-M0+VMAP-C->MoxR->vWA+TPRs*->FXSXX-COOH->Radical_SAM->METALLOPROTEASE+TPRs->TIR+APATPase+TPRs->
- **GCF_000719915.1; Streptomyces sp. NRRL F-2890; counts= 1; multicellularity status: TRUE**
  - <-TCAD15||Trypco1->Trypsin+HDxxH+VMAP-M0+VMAP-C->MoxR->vWA+TPRs*->FXSXX-COOH->Radical_SAM->METALLOPROTEASE+TPRs->TIR+APATPase+TPRs->
- **GCF_000805335.1; Streptomyces sp. CT34; counts= 1; multicellularity status: TRUE**
  - <-TCAD15||Trypco1->Trypsin+HDxxH+VMAP-M0+VMAP-C->MoxR->vWA+TPRs*->FXSXX-COOH->Radical_SAM->
- **GCF_000819545.1; Streptomyces nodosus; counts= 1; multicellularity status: TRUE**
  - Trypsin+EAD2->EAD2+VMAP-M0+VMAP-C->MoxR->vWA+APATPase+wHTH+TPRs*->||<-METALLOPROTEASE+TPRs<-Radical_SAM<-FXSXX-COOH||Voltage_CLC->||<-Trypco1
- **GCF_000716435.1; Streptomyces xylophagus; counts= 1; multicellularity status: TRUE**
  - EAD2+VMAP-M0+VMAP-C->MoxR->vWA+APATPase+wHTH+TPRs*->AB_hydrolase->CASPASE+EAD2->FXSXX-COOH-like->METALLOPROTEASE+TPRs->
- **GCA_000695625.1; Amycolatopsis rifamycinica; counts= 1; multicellularity status: TRUE**
  - cNMP_cyclase+EAD2->EAD2+VMAP-M0+VMAP-C->MoxR->vWA+APATPase+wHTH+TPRs*->||<-METALLOPROTEASE+TPRs<-FXSXX-COOH-like
- **GCF_000312185.1; Microcystis aeruginosa; counts= 1; multicellularity status: TRUE**
  - HTH+VMAP-M9+VMAP-C->MoxR->?->vWA+FGS-fold_domain*->?->PSE->FGS-fold_domain->
- **GCA_000935125.1; Streptomyces natalensis; counts= 1; multicellularity status: TRUE**
  - Trypco1->Trypsin+EAD2+VMAP-M0+VMAP-C->MoxR->vWA+APATPase+wHTH+TPRs*->
- **GCA_003147545.1; Streptomyces griseus; counts= 1; multicellularity status: TRUE**
  - EAD2+VMAP-M0+VMAP-C->MoxR->vWA+APATPase+wHTH+TPRs*->AB_hydrolase->CASPASE+EAD2->FXSXX-COOH-like->METALLOPROTEASE+TPRs->
- **GCA_000952105.1; Chromobacterium violaceum; counts= 1; multicellularity status: FALSE**
  - AB_hydrolase->CASPASE+VMAP-M4+VMAP-C->MoxR->vWA+REC+TCAD8*->
- **GCA_000955975.1; Saccharothrix sp. ST-888; counts= 1; multicellularity status: TRUE**
  - Trypco1->Trypsin+EAD2+VMAP-M0+VMAP-C->MoxR->vWA+Pkinase+ParA-Soj-PloopNTPase(SIMIBI)*->
- **GCF_000969605.1; Aquincola tertiaricarbonis; counts= 1; multicellularity status: TRUE**
  - AB_hydrolase->CASPASE+VMAP-M0+VMAP-C->MoxR->vWA+BetaPropeller+REC*->
- **GCF_003060805.1; Arthrospira sp. TJSD091; counts= 1; multicellularity status: TRUE**
  - VMAP-C_protein->MoxR->vWA+TPRs+CASPASE*->
- **GCF_000981895.1; Streptomyces sp. NRRL B-24891; counts= 1; multicellularity status: TRUE**
  - vWA+TPRs*->FXSXX-COOH->Radical_SAM->METALLOPROTEASE+TPRs->TIR+APATPase+TPRs->
- **GCA_000993785.2; Streptomyces xiamenensis; counts= 1; multicellularity status: TRUE**
  - <-TCAD15||Trypco1->Trypsin+HDxxH+VMAP-M0+VMAP-C->MoxR->vWA+TPRs*->FXSXX-COOH->Radical_SAM->METALLOPROTEASE+TPRs->TIR+APATPase+TPRs->

■ **GCF_001005085.2; Streptomyces humi; counts= 1; multicellularity status: TRUE**

- Trypsin+EAD2+VMAP-M0+VMAP-C->vWA+APATPase+wHTH+TPRs*->KAP_NTPase->

■ **GCA_001011035.1; Streptomyces sp. CNQ-509; counts= 1; multicellularity status: TRUE**

- <-TCAD15||Trypco1->Trypsin+HDxxH+VMAP-M0+VMAP-C->MoxR->vWA+TPRs*->FXSXX-COOH->Radical_SAM->TIR+APATPase+TPRs->

■ **GCA_001014595.1; Streptomyces sp. KE1; counts= 1; multicellularity status: TRUE**

- <-TCAD15||Trypco1->Trypsin+HDxxH+VMAP-M0+VMAP-C->MoxR->vWA+TPRs*->FXSXX-COOH->Radical_SAM->

■ **GCF_000800535.1; Streptomyces sp. M10; counts= 1; multicellularity status: TRUE**

- <-TCAD15||Trypco1->Trypsin+HDxxH+VMAP-M0+VMAP-C->MoxR->vWA+TPRs*->FXSXX-COOH->Radical_SAM->METALLOPROTEASE+TPRs->TIR+APATPase+TPRs->

■ **GCF_001027285.1; Archangium gephyra; counts= 1; multicellularity status: TRUE**

- EAD1+VMAP-M1+VMAP-C->vWA+SLOG*->

■ **GCA_001039005.1; Burkholderia cepacia; counts= 1; multicellularity status: FALSE**

- AB_hydrolase->CASPASE+VMAP-M4+VMAP-C->MoxR->vWA+APATPase+wHTH*->

■ **GCF_000718635.1; Streptomyces varsoviensis; counts= 1; multicellularity status: TRUE**

- <-TCAD15||Trypco1->Trypsin+HDxxH+VMAP-M0+VMAP-C->MoxR->vWA+TPRs*->FXSXX-COOH->Radical_SAM->METALLOPROTEASE+TPRs->TIR+APATPase+TPRs->

■ **GCF_001083795.1; Streptomyces sp. SBT349; counts= 1; multicellularity status: TRUE**

- vWA+TPRs*->FXSXX-COOH->

■ **GCA_001187435.1; Streptomyces caatingaensis; counts= 1; multicellularity status: TRUE**

- <-TCAD15||Trypsin+HDxxH+VMAP-M0+VMAP-C->MoxR->vWA+TPRs*->FXSXX-COOH->METALLOPROTEASE+TPRs->TIR+APATPase+TPRs->

■ **GCF_000022045.1; Cyanothece sp. PCC 7425; counts= 1; multicellularity status: TRUE**

- <-FGS-fold_domain<-?<-?<-?||?->VMAP-M1+VMAP-C->MoxR->vWA*->||<-zf-IS66+DDE_Tnp_IS66<-TIR+EAD9<-PSE<-Trypsin<-Trypco1

■ **GCA_000698945.1; Streptomyces wadayamensis; counts= 1; multicellularity status: TRUE**

- <-TCAD15<-?||Trypco1->Trypsin+HDxxH+VMAP-M0+VMAP-C->MoxR->vWA+TPRs*->FXSXX-COOH->Radical_SAM->METALLOPROTEASE+TPRs->TIR+APATPase+TPRs->

■ **GCF_000739635.1; Verrucomicrobium sp. BvORR034; counts= 1; multicellularity status: FALSE**

- VMAP-M22+VMAP-C->MoxR->vWA+AP-GTPase+COR+EAD1+Trypsin*->

■ **GCF_000716945.1; Streptomyces albus; counts= 1; multicellularity status: TRUE**

- Trypco2->Trypsin+EAD2+VMAP-M0+VMAP-C->MoxR->vWA+Pkinase+TPRs*->

■ **GCF_000259895.1; Streptomyces sp. TOR3209; counts= 1; multicellularity status: TRUE**

- Trypco1->Trypsin+VMAP-M0+VMAP-C->MoxR->vWA+Pkinase+wHTH+TPRs+cNMP_cyclase*->

■ **GCF_000373465.1; Streptomyces sp. FxanaD5; counts= 1; multicellularity status: TRUE**

- Trypco1->Trypsin+VMAP-M0+VMAP-C->MoxR->vWA+Pkinase+wHTH+TPRs+cNMP_cyclase+Band7*->

- **GCF_000381885.1; Actinomadura atramentaria; counts= 1; multicellularity status: TRUE**
  - Trypco1->Trypsin+VMAP-M8+VMAP-C->MoxR->vWA+cNMP_cyclase+cNMP_cyclase+FtsK+TPRs*->?->?->||<-DUF397<-DUF397
- **GCF_000349325.1; Streptomyces davawensis; counts= 1; multicellularity status: TRUE**
  - EAD2+VMAP-M0+VMAP-C->vWA+APATPase+wHTH+TPRs*->||<-METALLOPROTEASE+TPRs<-FXSXX-COOH-like<-Trypsin+EAD2||METALLOPROTEASE+TPRs->
- **GCF_000424825.1; Streptomyces sp. CNH287; counts= 1; multicellularity status: TRUE**
  - TCAD15->?->||<-?||Trypco1->Trypsin+HDxxH+VMAP-M0+VMAP-C->MoxR->vWA+TPRs*->FXSXX-COOH->METALLOPROTEASE+TPRs->TIR+APATPase+TPRs->
- **GCF_000497425.1; Streptomyces niveus; counts= 1; multicellularity status: TRUE**
  - Trypco2->Trypsin+EAD2+VMAP-M0+VMAP-C->MoxR->vWA+Pkinase+TPRs*->
- **GCA_001756925.1; Streptomyces fradiae; counts= 1; multicellularity status: TRUE**
  - NUDIX->Trypco1->Trypsin+EAD2+VMAP-M0+VMAP-C->MoxR->vWA+Pkinase+ParA-Soj-PloopNTPase(SIMIBI)*->SIR2->
- **GCF_000696185.1; Kitasatospora cheerisanensis; counts= 1; multicellularity status: TRUE**
  - Trypsin+EAD2+VMAP-M0+VMAP-C->PSE->vWA_protein*->BetaPropeller->
- **GCF_000701325.1; Streptomyces sp. URHA0041; counts= 1; multicellularity status: TRUE**
  - <-TCAD15||Trypsin+HDxxH+VMAP-M0+VMAP-C->MoxR->vWA+TPRs*->FXSXX-COOH->Radical_SAM->METALLOPROTEASE+TPRs->TIR+APATPase+TPRs->
- **GCF_000719555.1; Streptomyces sp. NRRL F-5727; counts= 1; multicellularity status: TRUE**
  - EAD2+VMAP-M0+VMAP-C->MoxR->vWA+APATPase+wHTH+TPRs*->||<-Na_H_Exchanger||AB_hydrolase->CASPASE+EAD2->FXSXX-COOH-like->METALLOPROTEASE+TPRs->
- **GCF_000718985.1; Streptomyces megasporus; counts= 1; multicellularity status: TRUE**
  - Trypco1->Trypsin+HDxxH+VMAP-M0+VMAP-C->MoxR->vWA+TPRs*->METALLOPROTEASE+TPRs->TIR+APATPase+TPRs->
- **GCA_000707925.1; Streptomyces rimosus; counts= 1; multicellularity status: TRUE**
  - <-TCAD15||Trypco1->Trypsin+HDxxH+VMAP-M0+VMAP-C->MoxR->vWA+TPRs*->FXSXX-COOH->Radical_SAM->TIR+APATPase+TPRs->
- **GCF_000716805.1; Streptomyces yerevanensis; counts= 1; multicellularity status: TRUE**
  - Trypco1->PSE->MoxR->vWA+Pkinase+wHTH+TPRs+cNMP_cyclase+Band7+HAD*->
- **GCF_000718475.1; Microbispora rosea; counts= 1; multicellularity status: TRUE**
  - Trypco1->Trypsin+VMAP-M8+VMAP-C->MoxR->vWA+TIR+FxsC_Cter*->
- **GCF_000716685.1; Streptomyces virginiae; counts= 1; multicellularity status: TRUE**
  - <-TCAD15||Trypsin+HDxxH+VMAP-M0+VMAP-C->MoxR->vWA+TPRs*->FXSXX-COOH->Radical_SAM->METALLOPROTEASE+TPRs->TIR+APATPase+TPRs->
- **GCF_000719695.1; Streptomyces sp. NRRL F-6131; counts= 1; multicellularity status: TRUE**
  - Trypco1->Trypsin+EAD2->EAD2+VMAP-M10+VMAP-C->MoxR->vWA+APATPase+SWACOS+wHTH+TPRs+Beta-rich-tail*->
- **GCF_000716445.1; Streptomyces; counts= 1; multicellularity status: TRUE**
  - EAD2+VMAP-M0+VMAP-C->MoxR->vWA+APATPase+wHTH+TPRs*->||<-Na_H_Exchanger||AB_hydrolase->CASPASE+EAD2->FXSXX-COOH-like->METALLOPROTEASE+TPRs->

- **GCF_000717135.1; Catenuloplanes japonicus; counts= 1; multicellularity status: TRUE**
  - METALLOPROTEASE+TPRs->EAD2+VMAP-M0+VMAP-C->vWA+APATPase+wHTH+TPRs*->
- **GCF_000718525.1; Streptomyces griseus; counts= 1; multicellularity status: TRUE**
  - EAD2+VMAP-M0+VMAP-C->MoxR->vWA+APATPase+wHTH+TPRs*->||<-Na_H_Exchanger||AB_hydrolase->CASPASE+EAD2->FXSXX-COOH-like->METALLOPROTEASE+TPRs->
- **GCF_000720145.1; Streptomyces sp. NRRL F-2747; counts= 1; multicellularity status: TRUE**
  - <-TCAD15||Trypsin+HDxxH+VMAP-M0+VMAP-C->MoxR->vWA+TPRs*->FXSXX-COOH->Radical_SAM->METALLOPROTEASE+TPRs->TIR+APATPase+TPRs->
- **GCF_000721705.1; Actinoplanes subtropicus; counts= 1; multicellularity status: TRUE**
  - PNP_UDP_1+VMAP-M0+VMAP-C->MoxR->vWA+APATPase+wHTH+TPRs*->||<-METALLOPROTEASE+TPRs
- **GCF_000725465.1; Streptomyces bicolor; counts= 1; multicellularity status: TRUE**
  - Trypco1->Trypsin+EAD2+VMAP-M0+VMAP-C->MoxR->vWA+Pkinase+ParA-Soj-PloopNTPase(SIMIBI)*->
- **GCF_000717655.1; Streptomyces katrae; counts= 1; multicellularity status: TRUE**
  - <-TCAD15||Trypsin+HDxxH+VMAP-M0+VMAP-C->MoxR->vWA+TPRs*->FXSXX-COOH->Radical_SAM->METALLOPROTEASE+TPRs->TIR+APATPase+TPRs->
- **GCF_000720635.1; Streptomyces sp. NRRL WC-3742; counts= 1; multicellularity status: TRUE**
  - Trypco1->Trypsin+VMAP-M0+VMAP-C->vWA+TIR+FxsC_Cter*->
- **GCA_002803845.1; Streptomyces lavendulae; counts= 1; multicellularity status: TRUE**
  - <-TCAD15||Trypsin+HDxxH+VMAP-M0+VMAP-C->MoxR->vWA+TPRs*->FXSXX-COOH->Radical_SAM->METALLOPROTEASE+TPRs->TIR+APATPase+TPRs->
- **GCF_000721515.1; Streptomyces sp. NRRL F-5193; counts= 1; multicellularity status: TRUE**
  - EAD2+VMAP-M0+VMAP-C->MoxR->vWA+APATPase+wHTH+TPRs*->||<-Na_H_Exchanger||AB_hydrolase->CASPASE+EAD2->FXSXX-COOH-like->METALLOPROTEASE+TPRs->
- **GCF_000721305.1; Streptomyces sp. NRRL S-1868; counts= 1; multicellularity status: TRUE**
  - Trypco1->Trypsin+EAD2+VMAP-M0+VMAP-C->MoxR->vWA+Pkinase+ParA-Soj-PloopNTPase(SIMIBI)*->
- **GCF_000725125.1; Streptomyces toyocaensis; counts= 1; multicellularity status: TRUE**
  - Trypco1->Trypsin+EAD2+VMAP-M0+VMAP-C->MoxR->vWA+Pkinase+ParA-Soj-PloopNTPase(SIMIBI)*->
- **GCF_000720485.1; Streptomyces ochraceiscleroticus; counts= 1; multicellularity status: TRUE**
  - Trypco1->Trypsin+EAD2+VMAP-M0+VMAP-C->MoxR->vWA+SWACOS+wHTH*->
- **GCF_000725785.1; Streptomyces; counts= 1; multicellularity status: TRUE**
  - SIR2->?->||<-?||AB_hydrolase->CASPASE+EAD2+VMAP-M0+VMAP-C->MoxR->vWA+REase+cNMP_cyclase+CASPASE*->
- **GCF_000745715.1; Streptomyces mirabilis; counts= 1; multicellularity status: TRUE**
  - AB_hydrolase->CASPASE+EAD2+VMAP-M0+VMAP-C->MoxR->vWA+PNP_UDP_1+APATPase+wHTH+TPRs*->
- **GCF_000612545.1; Streptomyces sp. PCS3-D2; counts= 1; multicellularity status: TRUE**
  - <-TCAD15||Trypsin+HDxxH+VMAP-M0+VMAP-C->MoxR->vWA+TPRs*->FXSXX-COOH->Radical_SAM->METALLOPROTEASE+TPRs->TIR+APATPase+TPRs->

- **GCF_000772895.1; Streptomyces galbus; counts= 1; multicellularity status: TRUE**
  - Trypco1->Trypsin+VMAP-M0+VMAP-C->MoxR->vWA+Pkinase+wHTH+TPRs+cNMP_cyclase*->?->?->||<-?||Radical_SAM->Antibiotic_NAT->TIR+FxsC_Cter->
- **GCF_000686765.1; Streptomyces sp. UNC401CLCol; counts= 1; multicellularity status: TRUE**
  - Trypco1->Trypsin+VMAP-M0+VMAP-C->MoxR->vWA+Pkinase+wHTH+TPRs+cNMP_cyclase+Band7*->
- **GCF_000814065.1; Pseudomonas sp. C5pp; counts= 1; multicellularity status: FALSE**
  - AB_hydrolase->CASPASE+VMAP-M16+VMAP-C->MoxR->vWA+BetaPropeller+TCAD10+REC*->
- **GCF_000158955.1; Streptomyces viridochromogenes; counts= 1; multicellularity status: TRUE**
  - <-TCAD15||Trypco1->Trypsin+HDxxH+VMAP-M0+VMAP-C->MoxR->vWA+TPRs*->
- **GCF_000525635.1; Kutzneria albida; counts= 1; multicellularity status: TRUE**
  - EAD2+VMAP-M0+VMAP-C->MoxR->?->vWA+TIR+BetaPropeller*->?->?->||<-cNMP_cyclase+EAD2||cNMP_binding+HTH_Crp_2->?->SIR2->
- **GCF_000717715.1; Streptomyces sp. NRRL B-24484; counts= 1; multicellularity status: TRUE**
  - Trypco1->Trypsin+EAD2+VMAP-M0+VMAP-C->MoxR->vWA+TPRs*->
- **GCF_000721495.1; Streptomyces sp. NRRL F-5123; counts= 1; multicellularity status: TRUE**
  - Trypsin+EAD2+VMAP-M0+VMAP-C->MoxR->vWA+APATPase+wHTH+BetaPropeller*->
- **GCF_000721235.1; Streptomyces olivaceus; counts= 1; multicellularity status: TRUE**
  - Trypco1->Trypsin+EAD2+VMAP-M0+VMAP-C->MoxR->vWA+Pkinase+ParA-Soj-PloopNTPase(SIMIBI)*->
- **GCF_000718165.1; Streptomyces fulvoviolaceus; counts= 1; multicellularity status: TRUE**
  - Trypco1->Trypsin+HDxxH+VMAP-M0+VMAP-C->MoxR->vWA+Pkinase+STAND_NTPase+STAND_NTPase*->
- **GCF_000829695.1; Streptomyces sp. NBRC 110035; counts= 1; multicellularity status: TRUE**
  - EAD2+VMAP-M0+VMAP-C->vWA+APATPase+wHTH+TPRs*->||<-METALLOPROTEASE+TPRs<-Trypsin+EAD2<-Trypco2||METALLOPROTEASE+TPRs->
- **GCA_000733295.1; Archangium violaceum; counts= 1; multicellularity status: TRUE**
  - EAD1+Trypsin->EAD1+VMAP-M1+VMAP-C->MoxR->vWA+SLOG+EAD1+TIR*->
- **GCF_000955975.1; Saccharothrix sp. ST-888; counts= 1; multicellularity status: TRUE**
  - Trypco1->Trypsin+VMAP-M0+VMAP-C->MoxR->vWA+CASPASE+TIR+FxsC_Cter(partial)*->
- **GCF_000966955.1; Streptomyces sp. NRRL S-495; counts= 1; multicellularity status: TRUE**
  - Trypsin+EAD2->EAD2+VMAP-M10+VMAP-C->MoxR->vWA+APATPase+SWACOS+wHTH+TPRs+Beta-rich-tail*->
- **GCF_001305595.1; Azospirillum thiophilum; counts= 1; multicellularity status: FALSE**
  - AB_hydrolase->CASPASE+VMAP-M4+VMAP-C->MoxR->vWA+APATPase+wHTH+TPRs*->
- **GCF_000720235.1; Streptomyces; counts= 1; multicellularity status: TRUE**
  - <-TCAD15||Trypsin+HDxxH+VMAP-M0+VMAP-C->MoxR->vWA+TPRs*->FXSXX-COOH->

- **GCF_000829715.2; Streptomyces sp. NBRC 110027; counts= 1; multicellularity status: TRUE**
  - <-TCAD15||Trypco1->Trypsin+HDxxH+VMAP-M0+VMAP-C->MoxR->vWA+TPRs*->FXSXX-COOH->Radical_SAM->TIR+APATPase+TPRs->
- **GCF_000972435.1; Streptomyces sp. MBT28; counts= 1; multicellularity status: TRUE**
  - <-TCAD15||Trypco1->Trypsin+HDxxH+VMAP-M0+VMAP-C->MoxR->vWA+TIR+APATPase+Trypsin+HDxxH+VMAP-M0+VMAP-C*->
- **GCF_000982955.1; Micromonospora sp. HK10; counts= 1; multicellularity status: TRUE**
  - EAD2+VMAP-M0+VMAP-C->vWA+APATPase+wHTH+TPRs*->||<-METALLOPROTEASE+TPRs<-FXSXX-COOH-like<-CASPASE+EAD2
- **GCF_001005405.1; Streptomyces scabiei; counts= 1; multicellularity status: TRUE**
  - MoxR->vWA+APATPase+wHTH+TPRs*->
- **GCF_000718775.1; Streptomyces sp. NRRL S-31; counts= 1; multicellularity status: TRUE**
  - vWA+TPRs*->FXSXX-COOH->Radical_SAM->TIR+APATPase+TPRs->FXSXX-COOH->Radical_SAM->
- **GCF_000720995.1; Streptomyces celluloflavus; counts= 1; multicellularity status: TRUE**
  - <-TCAD15||Trypco1->Trypsin+HDxxH+VMAP-M0+VMAP-C->MoxR->vWA+TPRs*->FXSXX-COOH->Radical_SAM->TIR+APATPase+TPRs->
- **GCF_000980885.2; Streptomyces malaysiense; counts= 1; multicellularity status: TRUE**
  - Trypco2->EAD2+VMAP-M0+VMAP-C->vWA+APATPase+wHTH+TPRs*->||<-METALLOPROTEASE+TPRs<-FXSXX-COOH-like<-Trypsin+EAD2
- **GCF_000412265.2; Streptomyces sp. Mg1; counts= 1; multicellularity status: TRUE**
  - <-TCAD15||Trypsin+HDxxH+VMAP-M0+VMAP-C->MoxR->vWA+TPRs*->FXSXX-COOH->Radical_SAM->METALLOPROTEASE+TPRs->TIR+APATPase+TPRs->
- **GCF_001044425.1; Streptomyces roseus; counts= 1; multicellularity status: TRUE**
  - <-TCAD15||Trypsin+HDxxH+VMAP-M0+VMAP-C->MoxR->vWA+TPRs*->FXSXX-COOH->PSE->METALLOPROTEASE+TPRs->TIR+APATPase+TPRs->
- **GCF_001270565.1; Streptomyces virginiae; counts= 1; multicellularity status: TRUE**
  - <-TCAD15||Trypsin+HDxxH+VMAP-M0+VMAP-C->MoxR->vWA+TPRs*->FXSXX-COOH->Radical_SAM->METALLOPROTEASE+TPRs->TIR+APATPase+TPRs->
- **GCF_001270485.1; Streptomyces viridochromogenes; counts= 1; multicellularity status: TRUE**
  - Trypco2->EAD2+VMAP-M0+VMAP-C->vWA+APATPase+wHTH+TPRs*->||<-METALLOPROTEASE+TPRs<-FXSXX-COOH-like<-Trypsin+EAD2
- **GCA_001279845.1; Streptomyces sp. XY533; counts= 1; multicellularity status: TRUE**
  - AB_hydrolase->CASPASE+VMAP-M0+VMAP-C->MoxR->vWA+Trypsin+cNMP_cyclase+CCCH(TrebleClef)+Pentapeptide_repeats*->
- **GCF_001279445.1; Streptomyces; counts= 1; multicellularity status: TRUE**
  - <-TCAD15||Trypsin+HDxxH+VMAP-M0+VMAP-C->MoxR->vWA+TPRs*->FXSXX-COOH->
- **GCF_001279485.1; Streptomyces sp. XY511; counts= 1; multicellularity status: TRUE**
  - <-TCAD15||Trypsin+HDxxH+VMAP-M0+VMAP-C->MoxR->vWA+TPRs*->FXSXX-COOH->Radical_SAM->
- **GCF_001279495.1; Streptomyces sp. H021; counts= 1; multicellularity status: TRUE**
  - <-TCAD15||Trypsin+HDxxH+VMAP-M0+VMAP-C->MoxR->vWA+TPRs*->FXSXX-COOH->Radical_SAM->METALLOPROTEASE+TPRs->TIR+APATPase+TPRs->

■ **GCF_001279855.1; Streptomyces; counts= 1; multicellularity status: TRUE**

- <-TCAD15||Trypsin+HDxxH+VMAP-M0+VMAP-C->MoxR->vWA+TPRs*->FXSXX-COOH->Radical_SAM->

■ **GCF_001279455.1; Streptomyces sp. XY152; counts= 1; multicellularity status: TRUE**

- Trypco1->Trypsin+VMAP-M0+VMAP-C->MoxR->vWA+Pkinase+wHTH+TPRs+cNMP_cyclase+Band7+HAD*->

■ **GCF_001279605.1; Streptomyces sp. NRRL F-4707; counts= 1; multicellularity status: TRUE**

- <-TCAD15||Trypco1->Trypsin+HDxxH+VMAP-M0+VMAP-C->MoxR->vWA+TPRs*->FXSXX-COOH->Radical_SAM->METALLOPROTEASE+TPRs->TIR+APATPase+TPRs->Trypco1->

■ **GCF_001280125.1; Streptomyces sp. NRRL F-6492; counts= 1; multicellularity status: TRUE**

- vWA+TPRs*->FXSXX-COOH->METALLOPROTEASE+TPRs->TIR+APATPase+TPRs->

■ **GCF_001279555.1; Micromonospora sp. NRRL B-16802; counts= 1; multicellularity status: TRUE**

- EAD2+VMAP-M0+VMAP-C->vWA+APATPase+wHTH+TPRs*->||<-METALLOPROTEASE+TPRs

■ **GCF_001280015.1; Streptomyces sp. MMG1121; counts= 1; multicellularity status: TRUE**

- Trypco1->Trypsin+EAD2+VMAP-M0+VMAP-C->MoxR->vWA+Pkinase+ParA-Soj-PloopNTPase(SIMIBI)*->

■ **GCA_001280015.1; Streptomyces sp. MMG1121; counts= 1; multicellularity status: TRUE**

- TCAD15->Trypco1->Trypsin+HDxxH+VMAP-M0+VMAP-C->MoxR->vWA+TPRs*->FXSXX-COOH-like->METALLOPROTEASE+TPRs->TIR+APATPase+TPRs->

■ **GCF_001279905.1; Streptomyces sp. XY66; counts= 1; multicellularity status: TRUE**

- <-TCAD15||Trypsin+HDxxH+VMAP-M0+VMAP-C->MoxR->vWA+TPRs*->FXSXX-COOH->Radical_SAM->

■ **GCF_001279935.1; Streptomyces sp. IGB124; counts= 1; multicellularity status: TRUE**

- <-TCAD15||Trypsin+HDxxH+VMAP-M0+VMAP-C->MoxR->vWA+TPRs*->FXSXX-COOH->

■ **GCF_001279365.1; Streptomyces sp. NRRL F-5755; counts= 1; multicellularity status: TRUE**

- <-TCAD15||Trypco1->Trypsin+HDxxH+VMAP-M0+VMAP-C->MoxR->vWA+TPRs*->FXSXX-COOH->Radical_SAM->TIR+APATPase+TPRs->TIR+APATPase+TPRs->

■ **GCF_001279625.1; Streptomyces sp. WM6368; counts= 1; multicellularity status: TRUE**

- <-TCAD15||Trypsin+HDxxH+VMAP-M0+VMAP-C->MoxR->vWA+TPRs*->FXSXX-COOH->

■ **GCF_001279685.1; Streptomyces sp. WM6373; counts= 1; multicellularity status: TRUE**

- <-TCAD15||Trypsin+HDxxH+VMAP-M0+VMAP-C->MoxR->vWA+TPRs*->FXSXX-COOH->Radical_SAM->

■ **GCF_001279765.1; Streptomyces sp. XY413; counts= 1; multicellularity status: TRUE**

- <-TCAD15||Trypsin+HDxxH+VMAP-M0+VMAP-C->MoxR->vWA+TPRs*->FXSXX-COOH->

■ **GCF_001280005.1; Streptomyces sp. NRRL WC-3618; counts= 1; multicellularity status: TRUE**

- MoxR->vWA+TPRs*->Radical_SAM->TIR+APATPase+TPRs->Radical_SAM->?->STAND_NTPase->vWA->TIR+APATPase+TPRs->

■ **GCF_001279985.1; Streptomyces sp. AS58; counts= 1; multicellularity status: TRUE**

- EAD2+VMAP-M0+VMAP-C->vWA+APATPase+wHTH+TPRs*->||<-METALLOPROTEASE+TPRs<-FXSXX-COOH-like<-Trypsin+EAD2<-Trypco2||METALLOPROTEASE+TPRs->

- **GCF_001280885.1; Streptomyces sp. XY332; counts= 1; multicellularity status: TRUE**
  - <-TCAD15||Trypsin+HDxxH+VMAP-M0+VMAP-C->MoxR->vWA+TPRs*->FXSXX-COOH->Radical_SAM->METALLOPROTEASE+TPRs->TIR+APATPase+TPRs->
- **GCF_001279345.1; Streptomyces rimosus; counts= 1; multicellularity status: TRUE**
  - <-TCAD15||Trypco1->Trypsin+HDxxH+VMAP-M0+VMAP-C->MoxR->vWA+TPRs*->FXSXX-COOH->Radical_SAM->TIR+APATPase+TPRs->
- **GCF_001294335.1; Streptomyces chattanoogensis; counts= 1; multicellularity status: TRUE**
  - <-TCAD15||Trypco1->Trypsin+HDxxH+VMAP-M0+VMAP-C->MoxR->vWA+TPRs*->FXSXX-COOH->Radical_SAM->TIR+APATPase+TPRs->
- **GCA_001294345.1; Streptomyces sp. NRRL S-4; counts= 1; multicellularity status: TRUE**
  - EAD2+VMAP-M0+VMAP-C->MoxR->vWA+APATPase+wHTH+TPRs*->AB_hydrolase->CASPASE+EAD2->FXSXX-COOH-like->METALLOPROTEASE+TPRs->
- **GCF_001298575.1; Actinobacteria bacterium OK074; counts= 1; multicellularity status: NA**
  - NUDIX->Trypco1->Trypsin+EAD2+VMAP-M0+VMAP-C->MoxR->vWA+Pkinase+ParA-Soj-PloopNTPase(SIMIBI)*->SIR2->
- **GCF_001298565.1; Actinobacteria bacterium OK006; counts= 1; multicellularity status: NA**
  - AB_hydrolase->CASPASE+VMAP-M2+VMAP-C->MoxR->vWA+BetaPropeller*->
- **GCF_001298545.1; Actinobacteria bacterium OV320; counts= 1; multicellularity status: NA**
  - <-TCAD15||Trypco1->Trypsin+HDxxH+VMAP-M0+VMAP-C->MoxR->vWA+TPRs*->FXSXX-COOH->Radical_SAM->METALLOPROTEASE+TPRs->TIR+APATPase+TPRs->
- **GCA_001306465.1; Frankia sp. R43; counts= 1; multicellularity status: TRUE**
  - EAD2+EAD1->EAD2+VMAP-M0+VMAP-C->MoxR->vWA+APATPase+wHTH+TPRs*->||<-METALLOPROTEASE+TPRs
- **GCF_001418505.1; Streptomyces flocculus; counts= 1; multicellularity status: TRUE**
  - vWA+TPRs*->FXSXX-COOH-like->METALLOPROTEASE+TPRs->TIR+APATPase+TPRs->
- **GCF_001418465.1; Streptomyces kanamyceticus; counts= 1; multicellularity status: TRUE**
  - vWA+Pkinase+FtsK+TPRs*->
- **GCF_001418325.1; Streptomyces atriruber; counts= 1; multicellularity status: TRUE**
  - vWA+Pkinase+wHTH+TPRs+cNMP_cyclase+HAD*->
- **GCF_001419765.1; Streptomyces torulosus; counts= 1; multicellularity status: TRUE**
  - Trypsin+EAD2+VMAP-M0+VMAP-C->vWA+APATPase+wHTH+TPRs*->
- **GCF_001420875.1; Frankia sp. AvcI1; counts= 1; multicellularity status: TRUE**
  - EAD2->EAD2+VMAP-M0+VMAP-C->MoxR->vWA+APATPase+wHTH+TPRs*->||<-METALLOPROTEASE+TPRs<-FXSXX-COOH-like
- **GCA_001426405.1; Streptomyces; counts= 1; multicellularity status: TRUE**
  - <-TCAD15||PSE->Trypsin+HDxxH+VMAP-M0+VMAP-C->MoxR->vWA+TPRs*->FXSXX-COOH->METALLOPROTEASE+TPRs->TIR+APATPase+TPRs->
- **GCA_001426585.1; Streptomyces sp. Root431; counts= 1; multicellularity status: TRUE**
  - <-TCAD15||Trypco1->Trypsin+HDxxH+VMAP-M0+VMAP-C->MoxR->vWA+TPRs*->FXSXX-COOH->METALLOPROTEASE+TPRs->TIR+APATPase+TPRs->

■ **GCA_001426615.1; Streptomyces; counts= 1; multicellularity status: TRUE**

- Trypco1->Trypsin+EAD2+VMAP-M0+VMAP-C->MoxR->vWA+Pkinase+ParA-Soj-PloopNTPase(SIMIBI)*->

■ **GCA_001429805.1; Kitasatospora sp. Root187; counts= 1; multicellularity status: TRUE**

- Trypco1->Trypsin+EAD2->EAD2+VMAP-M10+VMAP-C->MoxR->vWA+APATPase+BetaPropeller+Pkinase+wHTH+TPRs+wHTH+TPRs+FtsK*->

■ **GCF_001428885.1; Streptomyces sp. Root264; counts= 1; multicellularity status: TRUE**

- DUF397->MoxR->vWA+APATPase+wHTH+TPRs*->||<-vWA<-EAD2+VMAP-M0+VMAP-C

■ **GCA_001484605.1; Pseudarthrobacter sulfonivorans; counts= 1; multicellularity status: FALSE**

- AB_hydrolase->CASPASE+VMAP-M17+VMAP-C->MoxR->vWA+BetaPropeller+CASPASE+STAND_NTPase*->

■ **GCF_001493375.1; Streptomyces specialis; counts= 1; multicellularity status: TRUE**

- <-TCAD15||Trypco1->Trypsin+HDxxH+VMAP-M0+VMAP-C->MoxR->vWA+TPRs*->FXSXX-COOH->PSE->?->?->TIR+APATPase+TPRs->

■ **GCF_001485105.1; Streptomyces acidiscabies; counts= 1; multicellularity status: TRUE**

- vWA+TPRs*->FXSXX-COOH->Radical_SAM->METALLOPROTEASE+TPRs->TIR+APATPase+TPRs->

■ **GCA_001507435.1; Streptomyces sp. NRRL F-5122; counts= 1; multicellularity status: TRUE**

- <-TCAD15||Trypco1->Trypsin+HDxxH+VMAP-M0+VMAP-C->MoxR->vWA+TPRs*->FXSXX-COOH->Radical_SAM->METALLOPROTEASE+TPRs->TIR+APATPase+TPRs->

■ **GCF_001511815.1; Streptomyces reticuli; counts= 1; multicellularity status: TRUE**

- TCAD15->Trypco1->Trypsin+HDxxH+VMAP-M0+VMAP-C->MoxR->vWA+TPRs*->FXSXX-COOH-like->METALLOPROTEASE+TPRs->TIR+APATPase+TPRs->

■ **GCF_000974445.1; Lechevalieria aerocolonigenes; counts= 1; multicellularity status: TRUE**

- cNMP_cyclase+VMAP-M0+VMAP-C->MoxR->vWA+APATPase+wHTH+TPRs*->||<-METALLOPROTEASE+TPRs<-FXSXX-COOH-like||TIR+APATPase+TPRs->||<-DUF397

■ **GCF_001531145.1; Streptomyces sp. IMTB 1903; counts= 1; multicellularity status: TRUE**

- <-TCAD15||Trypsin+HDxxH+VMAP-M0+VMAP-C->MoxR->vWA+TPRs*->FXSXX-COOH->Radical_SAM->METALLOPROTEASE+TPRs->TIR+APATPase+TPRs->

■ **GCF_001550225.1; Streptomyces scabiei; counts= 1; multicellularity status: TRUE**

- MoxR->vWA+APATPase+wHTH+TPRs*->

■ **GCF_001550315.1; Streptomyces; counts= 1; multicellularity status: TRUE**

- Trypco1->Trypsin+VMAP-M0+VMAP-C->MoxR->vWA+Pkinase+wHTH+TPRs+cNMP_cyclase*->

■ **GCF_001550215.1; Streptomyces scabiei; counts= 1; multicellularity status: TRUE**

- vWA*->

■ **GCF_001572115.1; Streptomyces scabiei; counts= 1; multicellularity status: TRUE**

- MoxR->vWA+APATPase+wHTH+TPRs*->

■ **GCF_001592045.1; Thermobifida fusca; counts= 1; multicellularity status: TRUE**

- Trypsin+EAD2->EAD2+VMAP-M0+VMAP-C->MoxR->vWA+APATPase+wHTH+TPRs*->||<-DUF397||Trypco1->

- **GCA_003719195.1; Herbaspirillum rubrisubalbicans; counts= 1; multicellularity status: FALSE**
  - AB_hydrolase->CASPASE+VMAP-M20+VMAP-C->MoxR->vWA+DRHyd+TCAD8*->
- **GCA_001514215.1; Streptomyces bungoensis; counts= 1; multicellularity status: TRUE**
  - <-TCAD15||PSE->Trypsin+HDxxH+VMAP-M0+VMAP-C->MoxR->vWA+TPRs*->FXSXX-COOH->Radical_SAM->METALLOPROTEASE+TPRs->TIR+APATPase+TPRs->Trypco1->
- **GCA_001613935.1; Amycolatopsis regifaucium; counts= 1; multicellularity status: TRUE**
  - cNMP_cyclase+VMAP-M0+VMAP-C->MoxR->vWA+APATPase+wHTH+TPRs*->||<-METALLOPROTEASE+TPRs<-FXSXX-COOH-like<-cNMP_binding+HTH_Crp_2
- **GCF_001570645.1; Streptomyces sporocinereus; counts= 1; multicellularity status: TRUE**
  - AB_hydrolase->CASPASE+EAD2+VMAP-M0+VMAP-C->MoxR->vWA+TPRs*->
- **GCA_001513975.1; Streptomyces curacoi; counts= 1; multicellularity status: TRUE**
  - Trypsin+EAD2->EAD2+VMAP-M0+VMAP-C->MoxR->vWA+APATPase+wHTH+TPRs*->||<-METALLOPROTEASE+TPRs<-FXSXX-COOH-like
- **GCA_001469455.2; Streptomyces sp. AVP053U2; counts= 1; multicellularity status: TRUE**
  - EAD2+VMAP-M0+VMAP-C->vWA+APATPase+wHTH+TPRs*->||<-METALLOPROTEASE+TPRs<-Trypsin+EAD2
- **GCF_000974485.1; Streptomyces sp. NBRC 109706; counts= 1; multicellularity status: TRUE**
  - <-TCAD15||Trypco1->Trypsin+HDxxH+VMAP-M0+VMAP-C->MoxR->vWA+TPRs*->?->FXSXX-COOH->Radical_SAM->METALLOPROTEASE+TPRs->TIR+APATPase+TPRs->
- **GCA_001509475.1; Streptomyces regalis; counts= 1; multicellularity status: TRUE**
  - Trypco1->Trypsin+HDxxH+VMAP-M0+VMAP-C->MoxR->vWA+TPRs*->Radical_SAM->TIR+APATPase+TPRs->||<-TCAD15
- **GCF_001514235.1; Streptomyces caeruleatus; counts= 1; multicellularity status: TRUE**
  - Trypsin+VMAP-M0+VMAP-C->MoxR->vWA+Pkinase+ParA-Soj-PloopNTPase(SIMIBI)*->
- **GCF_000158935.1; Streptomyces lividans; counts= 1; multicellularity status: TRUE**
  - Trypco1->Trypsin+VMAP-M0+VMAP-C->MoxR->PSE->vWA+Pkinase+wHTH+TPRs+cNMP_cyclase*->
- **GCA_001270575.1; Streptomyces decoyicus; counts= 1; multicellularity status: TRUE**
  - <-TCAD15||Trypco1->Trypsin+HDxxH+VMAP-M0+VMAP-C->MoxR->vWA+TPRs*->FXSXX-COOH->Radical_SAM->TIR+APATPase+TPRs->
- **GCF_001270495.1; Streptomyces viridochromogenes; counts= 1; multicellularity status: TRUE**
  - Trypco1->Trypsin+VMAP-M0+VMAP-C->MoxR->vWA+Pkinase+wHTH+TPRs+cNMP_cyclase+Band7+HAD*->
- **GCF_000373365.1; Frankia discariae; counts= 1; multicellularity status: TRUE**
  - EAD2+EAD1->EAD2+VMAP-M0+VMAP-C->MoxR->vWA+APATPase+wHTH+TPRs*->||<-METALLOPROTEASE+TPRs
- **GCA_001687325.1; Streptomyces sp. PTY087I2; counts= 1; multicellularity status: TRUE**
  - EAD2+VMAP-M0+VMAP-C->MoxR->vWA+APATPase+wHTH+TPRs*->AB_hydrolase->CASPASE+EAD2->FXSXX-COOH-like->METALLOPROTEASE+TPRs->
- **GCA_001693675.1; Streptomyces clavuligerus; counts= 1; multicellularity status: TRUE**
  - <-TCAD15||Trypco1->Trypsin+HDxxH+VMAP-M0+VMAP-C->MoxR->vWA+TPRs*->

- **GCF_001514035.1; Streptomyces yokosukanensis; counts= 1; multicellularity status: TRUE**
  - Trypco1->Trypsin+EAD2+VMAP-M0+VMAP-C->MoxR->vWA+Pkinase+ParA-Soj-PloopNTPase(SIMIBI)*->
- **GCA_001514285.1; Streptomyces sp. DSM 15324; counts= 1; multicellularity status: TRUE**
  - AB_hydrolase->CASPASE+EAD2+VMAP-M0+VMAP-C->MoxR->vWA+REase+cNMP_cyclase+CASPASE*->
- **GCF_001653115.1; Streptomyces sp. F-3; counts= 1; multicellularity status: TRUE**
  - Trypsin+EAD2->EAD2+VMAP-M0+VMAP-C->MoxR->vWA+APATPase+wHTH+TPRs*->||<-METALLOPROTEASE+TPRs<-FXSXX-COOH-like||Trypco1->
- **GCF_001704195.1; Streptomyces sampsonii; counts= 1; multicellularity status: TRUE**
  - <-TCAD15||Trypco1->Trypsin+HDxxH+VMAP-M0+VMAP-C->MoxR->vWA+TPRs*->FXSXX-COOH->Radical_SAM->METALLOPROTEASE+TPRs->TIR+APATPase+TPRs->
- **GCF_001552155.1; Actinomadura formosensis; counts= 1; multicellularity status: TRUE**
  - Trypco1->Trypsin+VMAP-M8+VMAP-C->MoxR->vWA+FtsK+FtsK+FtsK*->
- **GCF_001552175.1; Actinomadura kijaniata; counts= 1; multicellularity status: TRUE**
  - Trypsin+VMAP-M8+VMAP-C->MoxR->vWA+FtsK+FtsK+FtsK+FtsK+FtsK+FtsK*->
- **GCF_001552555.1; Nocardiopsis trehalosi; counts= 1; multicellularity status: TRUE**
  - Trypsin+VMAP-M0+VMAP-C->MoxR->vWA+APATPase+wHTH+TPRs*->||<-DUF397
- **GCA_003350525.1; Nocardia mexicana; counts= 1; multicellularity status: TRUE**
  - Trypco1->Trypsin+HDxxH+VMAP-M0+VMAP-C->MoxR->vWA+TPRs*->Voltage_CLC->METALLOPROTEASE->TIR+APATPase+wHTH+TPRs->Radical_SAM->
- **GCA_001517975.1; Thermobifida cellulosilytica; counts= 1; multicellularity status: TRUE**
  - Trypsin+EAD2->EAD2+VMAP-M0+VMAP-C->MoxR->vWA+APATPase+wHTH+TPRs*->||<-DUF397||Trypco1->
- **GCA_001637315.1; Phormidium willei; counts= 1; multicellularity status: TRUE**
  - VMAP-C->MoxR->vWA+FGS-fold_domain*->FGS-fold_domain->
- **GCF_001715295.1; Streptomyces griseus; counts= 1; multicellularity status: TRUE**
  - EAD2+VMAP-M0+VMAP-C->MoxR->vWA+APATPase+wHTH+TPRs*->AB_hydrolase->CASPASE+EAD2->FXSXX-COOH-like->METALLOPROTEASE+TPRs->
- **GCF_001746315.1; Streptomyces sp. EN27; counts= 1; multicellularity status: TRUE**
  - Trypco1->Trypsin+EAD2+VMAP-M0+VMAP-C->MoxR->vWA+Pkinase+ParA-Soj-PloopNTPase(SIMIBI)*->
- **GCF_001746305.1; Streptomyces sp. EN23; counts= 1; multicellularity status: TRUE**
  - Trypco1->Trypsin+EAD2+VMAP-M0+VMAP-C->MoxR->vWA+Pkinase+ParA-Soj-PloopNTPase(SIMIBI)*->
- **GCF_001746295.1; Streptomyces sp. LUP47B; counts= 1; multicellularity status: TRUE**
  - Trypsin+EAD2->EAD2+VMAP-M0+VMAP-C->MoxR->vWA+APATPase+wHTH+TPRs*->||<-METALLOPROTEASE+TPRs<-FXSXX-COOH-like||Trypco1->
- **GCF_001748305.1; Streptomyces luteocolor; counts= 1; multicellularity status: TRUE**
  - ADP_ribosyl_GH->Trypsin+EAD2+VMAP-M0+VMAP-C->MoxR->vWA+Pkinase+FtsK+TPRs*->NUDIX->

- **GCA_001746425.1; Streptomyces subrutilus; counts= 1; multicellularity status: TRUE**
  - <-TCAD15||Trypsin+HDxxH+VMAP-M0+VMAP-C->MoxR->vWA+TPRs*->FXSXX-COOH->Radical_SAM->METALLOPROTEASE+TPRs->TIR+APATPase+TPRs->
- **GCA_001746465.1; Streptomyces agglomeratus; counts= 1; multicellularity status: TRUE**
  - <-TCAD15||PSE->Trypsin+HDxxH+VMAP-M0+VMAP-C->MoxR->vWA+TPRs*->FXSXX-COOH->METALLOPROTEASE+TPRs->TIR+APATPase+TPRs->
- **GCA_001746415.1; Streptomyces agglomeratus; counts= 1; multicellularity status: TRUE**
  - <-TCAD15||PSE->Trypsin+HDxxH+VMAP-M0+VMAP-C->MoxR->vWA+TPRs*->FXSXX-COOH->METALLOPROTEASE+TPRs->TIR+APATPase+TPRs->
- **GCA_001751375.1; Streptomyces; counts= 1; multicellularity status: TRUE**
  - Trypco1->Trypsin+HDxxH+VMAP-M0+VMAP-C->MoxR->vWA+TPRs*->FXSXX-COOH-like->METALLOPROTEASE+TPRs->TIR+APATPase+TPRs->
- **GCA_001746485.1; Streptomyces agglomeratus; counts= 1; multicellularity status: TRUE**
  - <-TCAD15||PSE->Trypsin+HDxxH+VMAP-M0+VMAP-C->MoxR->vWA+TPRs*->FXSXX-COOH->METALLOPROTEASE+TPRs->TIR+APATPase+TPRs->
- **GCA_001767235.1; Moorea producens; counts= 1; multicellularity status: TRUE**
  - EAD1+Trypsin->||<-?||PSE->EAD1+VMAP-M1+VMAP-C->MoxR->vWA+FGS-fold_domain*->
- **GCF_001865315.1; Streptomyces sampsonii; counts= 1; multicellularity status: TRUE**
  - <-TCAD15||Trypco1->Trypsin+HDxxH+VMAP-M0+VMAP-C->MoxR->vWA+TPRs*->FXSXX-COOH->Radical_SAM->METALLOPROTEASE+TPRs->TIR+APATPase+TPRs->
- **GCF_001865795.1; Streptomyces albidoflavus; counts= 1; multicellularity status: TRUE**
  - <-TCAD15||Trypco1->Trypsin+HDxxH+VMAP-M0+VMAP-C->MoxR->vWA+TPRs*->FXSXX-COOH->Radical_SAM->METALLOPROTEASE+TPRs->TIR+APATPase+TPRs->
- **GCA_001953855.1; Amycolatopsis keratiniphila; counts= 1; multicellularity status: TRUE**
  - cNMP_cyclase+VMAP-M0+VMAP-C->MoxR->vWA+APATPase+wHTH+TPRs*->||<-METALLOPROTEASE+TPRs<-FXSXX-COOH-like<-cNMP_binding+HTH_Crp_2
- **GCF_001578075.1; Microcystis aeruginosa; counts= 1; multicellularity status: TRUE**
  - AB_hydrolase->CASPASE+EAD2->EAD2+VMAP-M11+VMAP-C->MoxR->vWA+FGS-fold_domain*->
- **GCF_001895105.1; Streptomyces sp. NBRC 110465; counts= 1; multicellularity status: TRUE**
  - Trypco1->Trypsin+EAD2+VMAP-M0+VMAP-C->MoxR->vWA+Pkinase+ParA-Soj-PloopNTPase(SIMIBI)*->
- **GCA_900141985.1; Nocardiopsis flavescens; counts= 1; multicellularity status: TRUE**
  - Trypsin+EAD2->EAD2+VMAP-M0+VMAP-C->MoxR->vWA+APATPase+wHTH+TPRs*->||<-DUF397||Trypco1->
- **GCA_001905145.1; Nocardiopsis sp. TSRI0078; counts= 1; multicellularity status: TRUE**
  - Trypsin+VMAP-M0+VMAP-C->vWA+APATPase+wHTH+TPRs*->||<-DUF397||Trypco1->
- **GCA_001905365.1; Streptomyces sp. CB02488; counts= 1; multicellularity status: TRUE**
  - <-TCAD15||Trypco1->Trypsin+HDxxH+VMAP-M0+VMAP-C->MoxR->vWA+TPRs*->FXSXX-COOH->Radical_SAM->METALLOPROTEASE+TPRs->TIR+APATPase+TPRs->Trypco1->
- **GCF_001905645.1; Streptomyces sp. CB02115; counts= 1; multicellularity status: TRUE**
  - EAD2+VMAP-M0+VMAP-C->MoxR->vWA+APATPase+wHTH+TPRs*->AB_hydrolase->CASPASE+EAD2->FXSXX-COOH-like->METALLOPROTEASE+TPRs->

■ **GCF_001905905.1; Streptomyces sp. CB02366; counts= 1; multicellularity status: TRUE**

- AB_hydrolase->CASPASE+VMAP-M0+VMAP-C->MoxR->vWA+STAND_NTPase+Trypsin+CASPASE+S1COLD*->

■ **GCF_001905405.1; Streptomyces sp. TSRI0445; counts= 1; multicellularity status: TRUE**

- EAD2+VMAP-M0+VMAP-C->MoxR->vWA+APATPase+wHTH+TPRs*->AB_hydrolase->CASPASE+EAD2->FXSXX-COOH-like->METALLOPROTEASE+TPRs->

■ **GCF_001905795.1; Streptomyces sp. CB01580; counts= 1; multicellularity status: TRUE**

- AB_hydrolase->CASPASE+VMAP-M2+VMAP-C->MoxR->vWA+cNMP_cyclase+TCAD13*->

■ **GCF_001905345.1; Streptomyces uncialis; counts= 1; multicellularity status: TRUE**

- Trypco1->Trypsin+VMAP-M0+VMAP-C->MoxR->vWA+Pkinase+wHTH+TPRs+cNMP_cyclase*->

■ **GCF_001905485.1; Streptomyces sp. TSRI0261; counts= 1; multicellularity status: TRUE**

- AB_hydrolase->CASPASE+EAD2+VMAP-M0+VMAP-C->MoxR->vWA+TPRs*->

■ **GCA_001905485.1; Streptomyces sp. TSRI0261; counts= 1; multicellularity status: TRUE**

- EAD2+VMAP-M0+VMAP-C->MoxR->vWA+APATPase+wHTH+TPRs*->AB_hydrolase->CASPASE+EAD2->FXSXX-COOH-like->METALLOPROTEASE+TPRs->

■ **GCA_001905755.1; Amycolatopsis sp. CB00013; counts= 1; multicellularity status: TRUE**

- cNMP_cyclase+VMAP-M0+VMAP-C->MoxR->vWA+APATPase+wHTH+TPRs*->||<-METALLOPROTEASE+TPRs<-FXSXX-COOH-like<-cNMP_binding+HTH_Crp_2

■ **GCA_001905735.1; Streptomyces sp. CB01249; counts= 1; multicellularity status: TRUE**

- <-TCAD15||Trypco1->Trypsin+HDxxH+VMAP-M0+VMAP-C->MoxR->vWA+TPRs*->FXSXX-COOH->Radical_SAM->METALLOPROTEASE+TPRs->TIR+APATPase+TPRs->Trypco1->

■ **GCF_001905735.1; Streptomyces sp. CB01249; counts= 1; multicellularity status: TRUE**

- AB_hydrolase->CASPASE+EAD2+VMAP-M0+VMAP-C->MoxR->vWA+TPRs*->

■ **GCF_001905825.1; Streptomyces sp. CB02009; counts= 1; multicellularity status: TRUE**

- <-TCAD15||Trypco1->Trypsin+HDxxH+VMAP-M0+VMAP-C->MoxR->vWA+TPRs*->FXSXX-COOH->METALLOPROTEASE+TPRs->TIR+APATPase+TPRs->

■ **GCA_001905705.1; Streptomyces sp. CB02460; counts= 1; multicellularity status: TRUE**

- <-TCAD15||Trypco1->Trypsin+HDxxH+VMAP-M0+VMAP-C->MoxR->vWA+TPRs*->FXSXX-COOH->Radical_SAM->METALLOPROTEASE+TPRs->TIR+APATPase+TPRs->

■ **GCA_900111245.1; Streptomyces qinglanensis; counts= 1; multicellularity status: TRUE**

- Trypco1->Trypsin+HDxxH+VMAP-M0+VMAP-C->MoxR->vWA+TPRs*->FXSXX-COOH-like->METALLOPROTEASE+TPRs->TIR+APATPase+TPRs->

■ **GCA_001921205.1; Actinophytocola xanthii; counts= 1; multicellularity status: TRUE**

- cNMP_cyclase+VMAP-M0+VMAP-C->MoxR->vWA+APATPase+wHTH+TPRs*->||<-METALLOPROTEASE+TPRs<-FXSXX-COOH-like<-cNMP_binding+HTH_Crp_2

■ **GCA_001921215.1; Actinophytocola xinjiangensis; counts= 1; multicellularity status: TRUE**

- cNMP_cyclase+VMAP-M0+VMAP-C->MoxR->vWA+APATPase+wHTH+TPRs*->||<-METALLOPROTEASE+TPRs<-FXSXX-COOH-like<-cNMP_binding+HTH_Crp_2

■ **GCA_001939115.1; Oscillatoriales cyanobacterium 'hensonii'; counts= 1; multicellularity status: TRUE**

- EAD1+Trypsin->?->EAD1+VMAP-M1+VMAP-C->MoxR->vWA+FGS-fold_domain*->four_helix_bundle_protein->RVT_1->FGS-fold_domain->

- **GCF_001941275.1; Streptomyces acidiscabies; counts= 1; multicellularity status: TRUE**
  - vWA+Pkinase+wHTH+cNMP_cyclase+Band7+HAD*->
- **GCA_001983215.1; Frankia sp. CcI49; counts= 1; multicellularity status: TRUE**
  - EAD2+EAD1->EAD2+VMAP-M0+VMAP-C->MoxR->vWA+APATPase+wHTH+TPRs*->||<-METALLOPROTEASE+TPRs
- **GCA_001977425.1; Burkholderia pseudomallei; counts= 1; multicellularity status: FALSE**
  - AB_hydrolase->CASPASE+VMAP-M4+VMAP-C->MoxR->vWA+REC*->
- **GCA_001984175.1; Saccharothrix sp. ALI-22-I; counts= 1; multicellularity status: TRUE**
  - EAD2+VMAP-M0+VMAP-C->MoxR->vWA+APATPase+wHTH+TPRs*->||<-METALLOPROTEASE+TPRs<-cNMP_cyclase+EAD2
- **GCF_001984575.1; Streptomyces sp. MP131-18; counts= 1; multicellularity status: TRUE**
  - <-TCAD15||Trypco1->Trypsin+HDxxH+VMAP-M0+VMAP-C->MoxR->vWA+TPRs*->FXSXX-COOH->Radical_SAM->METALLOPROTEASE+TPRs->TIR+APATPase+TPRs->
- **GCA_001984575.1; Streptomyces sp. MP131-18; counts= 1; multicellularity status: TRUE**
  - Trypco1->Trypsin+EAD2+VMAP-M0+VMAP-C->MoxR->vWA+Pkinase+ParA-Soj-PloopNTPase(SIMIBI)*->
- **GCA_001989395.1; Nocardia donostiensis; counts= 1; multicellularity status: TRUE**
  - Trypco1->Trypsin+HDxxH+VMAP-M0+VMAP-C->MoxR->vWA+TPRs*->Voltage_CLC->METALLOPROTEASE->TIR+APATPase+wHTH+TPRs->
- **GCA_001998325.1; Nocardiopsis sinuspersici; counts= 1; multicellularity status: TRUE**
  - Trypsin+VMAP-M0+VMAP-C->vWA+APATPase+wHTH+TPRs*->||<-DUF397||Trypco1->
- **GCF_002005565.1; Streptomyces sp. JHA26; counts= 1; multicellularity status: TRUE**
  - Trypco1->Trypsin+EAD2+VMAP-M0+VMAP-C->MoxR->vWA+Pkinase+ParA-Soj-PloopNTPase(SIMIBI)*->
- **GCA_001642995.1; Streptomyces jeddahensis; counts= 1; multicellularity status: TRUE**
  - <-TCAD15||PSE->Trypsin+HDxxH+VMAP-M0+VMAP-C->MoxR->vWA+TPRs*->FXSXX-COOH->Radical_SAM->METALLOPROTEASE+TPRs->TIR+APATPase+TPRs->
- **GCF_002007685.1; Streptomyces sp. fd1-xmd; counts= 1; multicellularity status: TRUE**
  - <-TCAD15||Trypsin+HDxxH+VMAP-M0+VMAP-C->MoxR->vWA+TPRs*->FXSXX-COOH->Radical_SAM->METALLOPROTEASE+TPRs->TIR+APATPase+TPRs->
- **GCF_000239075.1; Streptomyces sp. W007; counts= 1; multicellularity status: TRUE**
  - EAD2+VMAP-M0+VMAP-C->MoxR->vWA+APATPase+wHTH+TPRs*->AB_hydrolase->CASPASE+EAD2->FXSXX-COOH-like->METALLOPROTEASE+TPRs->
- **GCF_000497445.1; Streptomyces roseochromogenus; counts= 1; multicellularity status: TRUE**
  - EAD2+VMAP-M0+VMAP-C->MoxR->vWA+APATPase+wHTH+TPRs*->||<-METALLOPROTEASE+TPRs<-FXSXX-COOH-like
- **GCA_900167435.1; Marinactinospora thermotolerans; counts= 1; multicellularity status: TRUE**
  - Trypsin+VMAP-M0+VMAP-C->MoxR->vWA+APATPase+wHTH+TPRs*->||<-DUF397||Trypco1->
- **GCF_000721685.1; Streptomyces mediolani; counts= 1; multicellularity status: TRUE**
  - EAD2+VMAP-M0+VMAP-C->MoxR->vWA+APATPase+wHTH+TPRs*->AB_hydrolase->CASPASE+EAD2->FXSXX-COOH-like->METALLOPROTEASE+TPRs->

■ **GCF_001279545.1; Streptomyces sp. NRRL B-3648; counts= 1; multicellularity status: TRUE**

- Trypco1->Trypsin+VMAP-M0+VMAP-C->MoxR->vWA+Pkinase+wHTH+TPRs*->

■ **GCF_001270575.1; Streptomyces decoyicus; counts= 1; multicellularity status: TRUE**

- AB_hydrolase->CASPASE+VMAP-M2+VMAP-C->MoxR->vWA+APATPase+wHTH+TPRs+SFI-helicase*->

■ **GCF_000931445.1; Streptomyces cyaneogriseus; counts= 1; multicellularity status: TRUE**

- <-TCAD15||Trypco1->Trypsin+HDxxH+VMAP-M0+VMAP-C->MoxR->vWA+TPRs*->FXSXX-COOH->Radical_SAM->METALLOPROTEASE+TPRs->TIR+APATPase+TPRs->

■ **GCA_002024165.1; Streptomyces scabrisporus; counts= 1; multicellularity status: TRUE**

- CASPASE+VMAP-M19+VMAP-C->MoxR->vWA+BetaPropeller*->

■ **GCF_001047315.1; Streptomyces leeuwenhoekii; counts= 1; multicellularity status: TRUE**

- <-TCAD15||Trypco1->Trypsin+HDxxH+VMAP-M0+VMAP-C->MoxR->vWA+TPRs*->FXSXX-COOH->Radical_SAM->METALLOPROTEASE+TPRs->TIR+APATPase+TPRs->

■ **GCF_001418645.1; Streptomyces neyagawaensis; counts= 1; multicellularity status: TRUE**

- Trypco1->Trypsin+EAD2+VMAP-M0+VMAP-C->MoxR->vWA+Pkinase+ParA-Soj-PloopNTPase(SIMIBI)*->

■ **GCF_001509775.1; Streptomyces violaceusniger; counts= 1; multicellularity status: TRUE**

- AB_hydrolase->CASPASE+EAD2+VMAP-M0+VMAP-C->MoxR->vWA+TIR+cNMP_cyclase+S1COLD+EAD2*->

■ **GCF_001514065.1; Streptomyces antibioticus; counts= 1; multicellularity status: TRUE**

- Trypco1->Trypsin+VMAP-M0+VMAP-C->MoxR->vWA+Pkinase+wHTH+TPRs+cNMP_cyclase+Band7+HAD*->

■ **GCF_001514305.1; Streptomyces sp. RV15; counts= 1; multicellularity status: TRUE**

- Trypco1->Trypsin+VMAP-M0+VMAP-C->MoxR->vWA+Pkinase+wHTH+TPRs+cNMP_cyclase+Band7+HAD*->

■ **GCF_001700515.1; Streptomyces lushanensis; counts= 1; multicellularity status: TRUE**

- <-TCAD15||Trypco1->Trypsin+HDxxH+VMAP-M0+VMAP-C->MoxR->vWA+TPRs*->FXSXX-COOH->Radical_SAM->METALLOPROTEASE+TPRs->TIR+APATPase+TPRs->

■ **GCF_001595515.1; Streptomyces sp. CC71; counts= 1; multicellularity status: TRUE**

- Trypco1->PSE->MoxR->vWA+Pkinase+wHTH+TPRs+cNMP_cyclase*->

■ **GCF_001746285.1; Streptomyces sp. EN16; counts= 1; multicellularity status: TRUE**

- EAD2+VMAP-M0+VMAP-C->MoxR->vWA+APATPase+wHTH+TPRs*->

■ **GCF_001542625.1; Streptomyces griseochromogenes; counts= 1; multicellularity status: TRUE**

- Trypco1->Trypsin+VMAP-M0+VMAP-C->MoxR->vWA+Pkinase+wHTH+TPRs+cNMP_cyclase+Band7*->

■ **GCF_001660045.1; Streptomyces parvulus; counts= 1; multicellularity status: TRUE**

- Trypco2->EAD2+VMAP-M0+VMAP-C->vWA+APATPase+wHTH+TPRs*->||<-METALLOPROTEASE+TPRs<-FXSXX-COOH-like<-Trypsin+EAD2

■ **GCF_001013905.1; Streptomyces leeuwenhoekii; counts= 1; multicellularity status: TRUE**

- <-TCAD15||Trypco1->Trypsin+HDxxH+VMAP-M0+VMAP-C->MoxR->vWA+TPRs*->FXSXX-COOH->Radical_SAM->METALLOPROTEASE+TPRs->TIR+APATPase+TPRs->

- **GCF_001866675.1; Streptomyces sp. MUSC 14; counts= 1; multicellularity status: TRUE**
  - Trypco2->Trypsin+EAD2+VMAP-M0+VMAP-C->MoxR->vWA+Pkinase+Pkinase+NaeI*->
- **GCF_001905855.1; Streptomyces sp. CB01883; counts= 1; multicellularity status: TRUE**
  - EAD2+VMAP-M0+VMAP-C->MoxR->vWA+APATPase+wHTH+TPRs*->||<-Na_H_Exchanger||AB_hydrolase->CASPASE+EAD2->FXSXX-COOH-like->METALLOPROTEASE+TPRs->
- **GCF_001905725.1; Streptomyces sp. CB02400; counts= 1; multicellularity status: TRUE**
  - CASPASE+VMAP-M2+VMAP-C->MoxR->||<-?||vWA+SWACOS+Trypsin+TIR+HTH*->
- **GCF_001953885.1; Streptomyces sp. IMTB 2501; counts= 1; multicellularity status: TRUE**
  - EAD2+VMAP-M0+VMAP-C->MoxR->vWA+APATPase+wHTH+TPRs*->||<-METALLOPROTEASE+TPRs<-FXSXX-COOH-like
- **GCF_001953875.1; Streptomyces amritsarensis; counts= 1; multicellularity status: TRUE**
  - <-TCAD15||Trypsin+HDxxH+VMAP-M0+VMAP-C->MoxR->vWA+TPRs*->FXSXX-COOH->Radical_SAM->METALLOPROTEASE+TPRs->TIR+APATPase+TPRs->
- **GCF_001941845.1; Streptomyces sp. TN58; counts= 1; multicellularity status: TRUE**
  - <-TCAD15||Trypsin+HDxxH+VMAP-M0+VMAP-C->MoxR->vWA+TPRs*->FXSXX-COOH->Radical_SAM->METALLOPROTEASE+TPRs->TIR+APATPase+TPRs->
- **GCA_002027195.1; Streptomyces sp. GKU 895; counts= 1; multicellularity status: TRUE**
  - MoxR->vWA_protein*->
- **GCF_002028425.1; Streptomyces katrae; counts= 1; multicellularity status: TRUE**
  - <-TCAD15||Trypsin+HDxxH+VMAP-M0+VMAP-C->MoxR->vWA+TPRs*->?->FXSXX-COOH->Radical_SAM->METALLOPROTEASE+TPRs->TIR+APATPase+TPRs->
- **GCA_003814735.1; Streptomyces katrae; counts= 1; multicellularity status: TRUE**
  - AB_hydrolase->||<-?||MoxR->vWA+Trypsin+cNMP_cyclase+CCCH(TrebleClef)+Pentapeptide_repeats*->
- **GCF_001570465.1; Mycobacterium fortuitum; counts= 1; multicellularity status: FALSE**
  - AB_hydrolase->CASPASE+VMAP-M2+VMAP-C->MoxR->vWA+HD+RelA+TGS*->
- **GCA_002078175.1; Streptomyces sp. M41(2017); counts= 1; multicellularity status: TRUE**
  - <-TCAD15||Trypco1->Trypsin+HDxxH+VMAP-M0+VMAP-C->MoxR->vWA+TPRs*->FXSXX-COOH->Radical_SAM->METALLOPROTEASE+TPRs->TIR+APATPase+TPRs->
- **GCF_001983105.1; Frankia sp. M16386; counts= 1; multicellularity status: TRUE**
  - EAD2+VMAP-M0+VMAP-C->MoxR->vWA+APATPase+wHTH+TPRs*->TIR+APATPase+TPRs->||<-METALLOPROTEASE+TPRs
- **GCF_000447295.1; Fischerella sp. PCC 9431; counts= 1; multicellularity status: TRUE**
  - Trypco1->Trypsin+EAD9->EAD9+EAD1+VMAP-C->MoxR->vWA+FGS-fold_domain*->FGS-fold_domain->four_helix_bundle_protein->RVT_1->
- **GCF_000464825.1; Planktothrix agardhii; counts= 1; multicellularity status: TRUE**
  - CASPASE+VMAP-M1+VMAP-C->MoxR->vWA+MNS+MNS*->
- **GCF_000478275.1; Amycolatopsis orientalis; counts= 1; multicellularity status: TRUE**
  - cNMP_cyclase+VMAP-M0+VMAP-C->MoxR->vWA+APATPase+wHTH+TPRs*->||<-METALLOPROTEASE+TPRs<-FXSXX-COOH-like<-cNMP_binding+HTH_Crp_2

- **GCA_000754115.1; Amycolatopsis sp. MJM2582; counts= 1; multicellularity status: TRUE**
  - cNMP_cyclase+VMAP-M0+VMAP-C->MoxR->vWA+APATPase+wHTH+TPRs*->||<-METALLOPROTEASE+TPRs<-FXSXX-COOH-like<-cNMP_binding+HTH_Crp_2
- **GCF_000955955.1; Lechevalieria aerocolonigenes; counts= 1; multicellularity status: TRUE**
  - <-cNMP_binding+HTH_Crp_2||cNMP_cyclase+VMAP-M0+VMAP-C->MoxR->vWA+APATPase+wHTH+TPRs*->||<-METALLOPROTEASE+TPRs<-FXSXX-COOH-like
- **GCA_000948395.1; Frankia sp. CpI1-S; counts= 1; multicellularity status: TRUE**
  - EAD2+EAD1->EAD2+VMAP-M0+VMAP-C->MoxR->vWA+APATPase+wHTH+TPRs*->||<-METALLOPROTEASE+TPRs<-FXSXX-COOH-like
- **GCF_000972705.2; Limnoraphis robusta; counts= 1; multicellularity status: TRUE**
  - FGS-fold_domain->||<-?<-?||EAD4+VMAP-M3+VMAP-C->MoxR->vWA+FGS-fold_domain*->?->FGS-fold_domain->
- **GCA_900113515.1; Amycolatopsis regifaucium; counts= 1; multicellularity status: TRUE**
  - cNMP_cyclase+VMAP-M0+VMAP-C->MoxR->vWA+APATPase+wHTH+TPRs*->||<-METALLOPROTEASE+TPRs<-FXSXX-COOH-like<-cNMP_binding+HTH_Crp_2
- **GCF_000943515.2; Amycolatopsis orientalis; counts= 1; multicellularity status: TRUE**
  - cNMP_cyclase+VMAP-M0+VMAP-C->MoxR->vWA+APATPase+wHTH+TPRs*->||<-METALLOPROTEASE+TPRs<-FXSXX-COOH-like<-cNMP_binding+HTH_Crp_2
- **GCF_001854645.1; Frankia sp. EUN1h; counts= 1; multicellularity status: TRUE**
  - EAD2+VMAP-M0+VMAP-C->MoxR->vWA+APATPase+wHTH+TPRs->||<-METALLOPROTEASE+TPRs||EAD2+VMAP-M0+VMAP-C->MoxR->vWA+APATPase+wHTH+TPRs*->||<-METALLOPROTEASE+TPRs
- **GCF_000826545.1; Kibdelosporangium sp. MJ126-NF4; counts= 1; multicellularity status: TRUE**
  - EAD2+VMAP-M0+VMAP-C->MoxR->vWA+APATPase+wHTH+TPRs*->||<-METALLOPROTEASE+TPRs<-FXSXX-COOH-like<-?||EAD2+VMAP-M0+VMAP-C->MoxR->vWA+APATPase+wHTH+TPRs->||<-METALLOPROTEASE+TPRs
- **GCF_001414035.1; Frankia sp. ACN1ag; counts= 1; multicellularity status: TRUE**
  - EAD2+EAD1->EAD2+VMAP-M0+VMAP-C->vWA+APATPase+wHTH+TPRs*->||<-METALLOPROTEASE+TPRs<-FXSXX-COOH-like
- **GCA_900010725.1; Planktothrix serta; counts= 1; multicellularity status: TRUE**
  - vWA+MACRODOMAIN+GUN4+DO-GTPase2*->
- **GCF_001905755.1; Amycolatopsis sp. CB00013; counts= 1; multicellularity status: TRUE**
  - cNMP_cyclase+VMAP-M0+VMAP-C->MoxR->vWA+APATPase+wHTH+TPRs*->||<-METALLOPROTEASE+TPRs<-FXSXX-COOH-like<-cNMP_binding+HTH_Crp_2
- **GCF_000620785.1; Pseudonocardia acaciae; counts= 1; multicellularity status: TRUE**
  - cNMP_binding+HTH_Crp_2->?->cNMP_cyclase+VMAP-M0+VMAP-C->MoxR->vWA+APATPase+wHTH+TPRs*->||<-METALLOPROTEASE+TPRs
- **GCF_001904725.1; Phormidium ambiguum; counts= 1; multicellularity status: TRUE**
  - cNMP_cyclase+VMAP-M1+VMAP-C->MoxR->vWA+FGS-fold_domain*->FGS-fold_domain->
- **GCF_001953865.1; Amycolatopsis coloradensis; counts= 1; multicellularity status: TRUE**
  - cNMP_cyclase+VMAP-M0+VMAP-C->MoxR->vWA+APATPase+wHTH+TPRs*->||<-METALLOPROTEASE+TPRs<-FXSXX-COOH-like<-cNMP_binding+HTH_Crp_2
- **GCF_000582685.1; [Scytonema hofmanni] UTEX B 1581; counts= 1; multicellularity status: TRUE**
  - EAD10+VMAP-M1+VMAP-C->MoxR->vWA+MNS+GUN4+EAD10+BetaPropeller*->

- **GCA_002099185.1; Streptomyces sp. CB03238; counts= 1; multicellularity status: TRUE**
  - Trypco1->Trypsin+EAD2+VMAP-M0+VMAP-C->MoxR->vWA+wHTH+TPRs+wHTH+TPRs+APATPase+wHTH+TPRs*->
- **GCA_002095975.1; Microcystis aeruginosa; counts= 1; multicellularity status: TRUE**
  - HTH+VMAP-M9+VMAP-C->MoxR->vWA+FGS-fold_domain*->||<-?<-?<-?<-PSE||?->FGS-fold_domain->
- **GCA_002114065.1; Streptomyces griseofuscus; counts= 1; multicellularity status: TRUE**
  - Trypco1->Trypsin+VMAP-M0+VMAP-C->MoxR->vWA+Pkinase+cNMP_cyclase+Band7+HAD*->
- **GCF_000328705.1; Saccharothrix espanaensis; counts= 1; multicellularity status: TRUE**
  - EAD2+VMAP-M0+VMAP-C->MoxR->vWA+APATPase+wHTH+TPRs*->||<-METALLOPROTEASE+TPRs<-cNMP_cyclase+EAD2
- **GCF_002150735.1; Streptomyces africanus; counts= 1; multicellularity status: TRUE**
  - Trypsin+EAD2+VMAP-M0+VMAP-C->MoxR->vWA+APATPase+GGDEF+EAD2+VMAP-M0+VMAP-C*->
- **GCF_002154275.1; Streptomyces castelarensis; counts= 1; multicellularity status: TRUE**
  - AB_hydrolase->CASPASE+EAD2+VMAP-M0+VMAP-C->MoxR->vWA+TIR+cNMP_cyclase+S1COLD+EAD2*->
- **GCF_002140155.1; Micromonospora sp. NBS 11-29; counts= 1; multicellularity status: TRUE**
  - Trypco1+Trypsin+VMAP-M0+VMAP-C->MoxR->vWA+Trypsin+Coiled-coil*->Glyco_hydro_16+Ricin_B_lectin->||<-?||?->DUF397->
- **GCA_004296455.1; Calothrix sp. NIES-4101; counts= 1; multicellularity status: TRUE**
  - EAD1+Trypsin->?->EAD1+VMAP-M1+VMAP-C->||<-?||VMAP-C(partial)->MoxR->vWA+REC+CASPASE+FGS-fold_domain*->
- **GCF_002551305.1; Streptomyces sp. or20; counts= 1; multicellularity status: TRUE**
  - Trypsin+EAD2+VMAP-M0+VMAP-C->MoxR->vWA+Pkinase+APATPase+TPRs*->
- **GCA_002573955.1; Streptomyces sp. Ru87; counts= 1; multicellularity status: TRUE**
  - vWA+Pkinase+Pkinase+TPRs*->
- **GCA_002776695.1; Bradyrhizobium sp. TSA1; counts= 1; multicellularity status: TRUE**
  - AB_hydrolase->CASPASE+VMAP-M4+VMAP-C->MoxR->vWA+REC+REC+GAF+GAF+GAF+Acylphosphatase+CASPASE*->

iSTAND systems

- **GCA_001292585.1; Candidatus Magnetomorum sp. HK-1; counts= 9; multicellularity status: TRUE**
  - DRHyd+iSTAND*->
  - iSTAND*->MoxR->??+FGE-sulfatase->
  - iSTAND*->MoxR->||<-tRNA_anti-codon+tRNA-synt_2||REC+PAS+NtrC-AAA+FIS-HTH->REC+cNMP_cyclase->HAM->
  - Nucleoside_deoxyribosyltransferas?->iSTAND(fragment)*->
  - TIR+iSTAND*->MoxR->TM+TM+vWA+TM+PEGA+PEGA+FGS-fold_domain->
  - TIR+iSTAND*->STAND_NTPase->
  - TM+TM+iSTAND*->
  - TM+TM+iSTAND*->MoxR->
  - ZnR+ZnR+TIR+iSTAND*->MoxR->TM+TM+vWA+TM+FGS-fold_domain->
- **GCA_000516475.1; Candidatus Magnetoglobus multicellularis str. Araruama; counts= 5; multicellularity status: TRUE**
  - DRHyd+iSTAND*->??->GspA-ATPase(fragment)->
  - DRHyd+iSTAND*->MoxR->
  - iSTAND(fragment)*->??->??->
  - STNAD_NTPase+REase7->ZnR+ZnR+TIR+iSTAND*->MoxR->
  - TIR+iSTAND*->
- **GCA_000346485.2; Scytonema hofmannii PCC 7110; counts= 4; multicellularity status: TRUE**
  - BetaPropeller+iSTAND*->
  - Coiled-coil+iSTAND*->
  - EAD9+iSTAND*->MoxR->helical_region(leucine-richLWxKmotif)+vWA->??+CHASE+TM+TM->
  - PNP_UDP_1+EAD9->iSTAND*->MoxR->helical_region(leucine-richLWxKmotif)+vWA+FGS-fold_domain->
- **GCA_001698425.1; Oscillatoriales cyanobacterium USR001; counts= 4; multicellularity status: TRUE**
  - ??->??+HTH+iSTAND*->MoxR->
  - CASPASE+iSTAND*->MoxR->helical_region(leucine-richLWxKmotif)+vWA->Ntox45->HEPN->??->
  - EAD1+CASPASE+iSTAND*->MoxR->helical_region(leucine-richLWxKmotif)+vWA->vWA+EAD1-unk5+CASPASE->
  - HTH+iSTAND*->MoxR->
- **GCA_003242955.1; Candidatus Methyloumidiphilus alinensis; counts= 3; multicellularity status: FALSE**
  - ??->iSTAND*->MoxR->TM+TM+vWA+TM+FGE-sulfatase->
  - DrHyd+EAD9->EAD9+iSTAND*->MoxR->helical_region(leucine-richLWxKmotif)+vWA->
  - TIR+iSTAND*->MoxR->
- **GCA_003646175.1; Gammaproteobacteria bacterium; counts= 3; multicellularity status: NA**

- ??->Trypco1->||<-??||Trypsin+iSTAND*->MoxR->HTH->
- EAD-unk2->CASPASE+iSTAND*->HTH->MoxR->??->??+DUF4351->
- TM+TM+iSTAND*->

- **GCA_000212735.1; Haliscomenobacter hydrossis DSM 1100; counts= 2; multicellularity status: TRUE**
  - CASPASE+iSTAND*->MoxR->vWA+??+FGS-fold_domain+FGS-fold_domain+FGS-fold_domain+FGS-fold_domain->
  - TM+TM+TM+TM+STAND_NTPase+TM+TM+TM+TM->||<-STAND_NTPase+REase<-TM||HTH->PIN+DUF3368->EADiS+iSTAND*->MoxR->||<-vWA<-??+beta-sandwich(Ig-like?)
- **GCA_002368175.1; Calothrix sp. NIES-2098; counts= 2; multicellularity status: TRUE**
  - Coiled-coil+iSTAND*->MoxR->helical_region(leucine-richLWxKmotif)+TM+Calx-beta?+TM+vWA->vWA+FGS-fold_domain->
  - EAD9+iSTAND*->MoxR->helical_region(leucine-richLWxKmotif)+vWA->Cation_ATPase->Calcineurin->
- **GCA_000300115.1; Tolypothrix sp. PCC 7601; counts= 2; multicellularity status: TRUE**
  - HTH+iSTAND+HTH+IG-like+CASPASE+IG-like*->CASPASE->Pkinase+STAND_NTPase+macroglobulin-like+CASPASE->CASPASE->Coiled-coil->
  - TM+DUF218->||<-??<-??||Coiled-coil+iSTAND*->MoxR->
- **GCA_000473895.1; Rubidibacter lacunae KORDI 51-2; counts= 2; multicellularity status: TRUE**
  - ??->iSTAND*->MoxR->TM+TM+vWA+TM->
  - <-Trypco1||CASPASE+iSTAND*->MoxR->helical_region(leucine-richLWxKmotif)+vWA->BetaPropeller+TPRs+FGE-sulfatase->FGS-fold_domain->
- **GCA_001314905.1; Phormidium sp. OSCR; counts= 2; multicellularity status: TRUE**
  - <-Trypco1||CASPASE+iSTAND*->MoxR->helical_region(leucine-richLWxKmotif)+vWA->vWA+FGS-fold_domain->
  - Coiled-coil+iSTAND*->MoxR->helical_region(leucine-richLWxKmotif)+vWA->
- **GCA_001698435.1; Nostoc sp. MBR 210; counts= 2; multicellularity status: TRUE**
  - ??+bDLD1+Trypsin->??->bDLD1+iSTAND*->MoxR->helical_region(leucine-richLWxKmotif)+vWA->
  - HTH+iSTAND*->MoxR->helical_region(leucine-richLWxKmotif)+vWA->??+CASPASE->
- **GCA_003695465.1; Bacteroidetes bacterium; counts= 2; multicellularity status: FALSE**
  - Coiled-coil+iSTAND(fragment)*->
  - iSTAND(fragment)*->MoxR->TM+beta-sandwich(Ig-like?)->
- **GCA_003696835.1; Bacteroidetes bacterium; counts= 2; multicellularity status: FALSE**
  - <-??||CASPASE+iSTAND*->TM+TM+vWA+??+BetaPropeller->
  - REC+Alkaline_phosphatase-like->CASPASE+iSTAND*->??->Radical_SAM->
- **GCA_900099115.2; Thiotrichales bacterium HS_08; counts= 2; multicellularity status: TRUE**
  - <-PseudoU_synth||??->??+CASPASE+iSTAND*->
  - Trypsin+iSTAND*->
- **GCF_000380225.1; filamentous cyanobacterium ESFC-1; counts= 2; multicellularity status: TRUE**
  - <-NfeD||??->??+iSTAND*->MoxR->
  - HTH+iSTAND*->MoxR->helical_region(leucine-richLWxKmotif)+vWA->
- **GCF_000373105.1; Lewinella persica; counts= 2; multicellularity status: TRUE**

- Coiled-coil+iSTAND*->MoxR->TM+TM+??TM+??+CASPASE->
- EADiS+iSTAND*->MoxR->

■ **GCF_900185595.1; Calothrix rhizosoleniae; counts= 2; multicellularity status: TRUE**

- EAD4+iSTAND*->MoxR->
- EAD9+iSTAND*->MoxR->helical_region(leucine-richLWxKmotif)+vWA->vWA+FGS-fold_domain->

■ **GCA_000014265.1; Trichodesmium erythraeum IMS101; counts= 1; multicellularity status: TRUE**

- iSTAND*->MoxR->TM+TM+vWA+TM+TM+TM+TM+TM+TM->

■ **GCA_000020025.1; Nostoc punctiforme PCC 73102; counts= 1; multicellularity status: TRUE**

- CASPASE+EAD9->EAD9+iSTAND*->STAND_NTPase->MoxR(fragment)->helical_region(leucine-richLWxKmotif)+vWA->??+REC->GUN4->

■ **GCA_000021825.1; Gloeothece citriformis PCC 7424; counts= 1; multicellularity status: TRUE**

- <-TM||HTH+iSTAND*->MoxR->helical_region(leucine-richLWxKmotif)+vWA->

■ **GCA_000219585.1; Nitrosomonas sp. Is79A3; counts= 1; multicellularity status: FALSE**

- TIR+iSTAND*->MoxR->TM+TM+vWA+TM+FGS-fold_domain->

■ **GCA_000316665.1; Rivularia sp. PCC 7116; counts= 1; multicellularity status: TRUE**

- Trypco1->Trypsin+iSTAND*->MoxR->helical_region(leucine-richLWxKmotif)+vWA->vWA+FGS-fold_domain->

■ **GCA_000317435.1; Calothrix sp. PCC 6303; counts= 1; multicellularity status: TRUE**

- CASPASE+iSTAND*->STAND_NTPase(fragment)->

■ **GCA_000317535.1; Cylindrospermum stagnale PCC 7417; counts= 1; multicellularity status: TRUE**

- CASPASE+EAD9->Coiled-coil+iSTAND*->

■ **GCA_001854205.1; Moorea producens JHB; counts= 1; multicellularity status: TRUE**

- Coiled-coil+iSTAND*->MoxR->helical_region(leucine-richLWxKmotif)+TM+Calx-beta?+TM+vWA->

■ **GCA_002796605.1; Sphingomonas sp. Cra20; counts= 1; multicellularity status: FALSE**

- rve_3->Trypsin+iSTAND*->MoxR->

■ **GCA_002813575.1; Nostoc flagelliforme CCNUN1; counts= 1; multicellularity status: TRUE**

- EAD9+iSTAND*->MoxR->helical_region(leucine-richLWxKmotif)+vWA->??+TM->CASPASE(fragment)->

■ **GCA_000210375.1; Arthrospira platensis NIES-39; counts= 1; multicellularity status: TRUE**

- SLH+OprB->||<-TM||RadC-like_JAB+sGTPase->Coiled-coil+iSTAND*->MoxR->helical_region(leucine-richLWxKmotif)+vWA->TA_antitoxin(DUF1902)->Ntox45->HEPN->

■ **GCA_002368215.1; Nostoc sp. NIES-2111; counts= 1; multicellularity status: TRUE**

- Pentapeptide_repeats+TM+TM+TM+TM+TM+TM+TM+Pentapeptide_repeats->Pentapeptide_repeats->STNAD_NTPase+TM+TM+TM+TM+TM+TM+TM+TM->iSTAND*->MoxR->TM+TM+vWA->||<-DDE_endonuclease<-PAIREDC-HTH||beta-rich_jelly-roll?->

■ **GCA_002368235.1; Scytonema sp. HK-05; counts= 1; multicellularity status: TRUE**

- iSTAND*->MoxR->helical_region(leucine-richLWxKmotif)+vWA->TM+Pentapeptide_repeats+TM+TM+TM+TM+TM+TM+TM+Pentapeptide_repeats->||<-TM+TM+TM+TM+TM

- **GCA_002368435.1; Scytonema sp. NIES-4073; counts= 1; multicellularity status: TRUE**
  - EAD8+Trypsin->EAD8+iSTAND*->MoxR->helical_region(leucine-richLWxKmotif)+vWA->REase->vWA+GTPase-AIG->
- **GCA_002368335.1; Nostoc sp. NIES-4103; counts= 1; multicellularity status: TRUE**
  - HTH+iSTAND*->MoxR->helical_region(leucine-richLWxKmotif)+vWA->
- **GCA_003609755.1; Planktothrix agardhii NIES-204; counts= 1; multicellularity status: TRUE**
  - ??+HTH+iSTAND*->MoxR->helical_region(leucine-richLWxKmotif)+vWA->
- **GCA_000026145.1; Bradyrhizobium sp. ORS 278; counts= 1; multicellularity status: TRUE**
  - Trypco2->Trypsin+iSTAND*->MoxR->2TM+vWA+22TM->??+TM->??->
- **GCA_000283655.1; Azospirillum lipoferum 4B; counts= 1; multicellularity status: FALSE**
  - Trypco2->Trypsin+iSTAND*->MoxR->2TM+vWA+22TM->
- **GCA_900009265.1; Planktothrix paucivesiculata PCC 9631; counts= 1; multicellularity status: TRUE**
  - Death-like.bact-prob+iSTAND*->MoxR->helical_region(leucine-richLWxKmotif)+vWA->vWA+TPRs+CASPASE->??->
- **GCA_900009145.1; Planktothrix tepida PCC 9214; counts= 1; multicellularity status: TRUE**
  - HTH+iSTAND*->MoxR->helical_region(leucine-richLWxKmotif)+vWA->
- **GCA_001458735.1; Candidatus Nitrospira nitrosa; counts= 1; multicellularity status: NA**
  - DUF805(3TM_protein)->??->DUF805(3TM_protein)->TM+TM+TM->TIR+iSTAND*->MoxR->TM+TM+vWA+TM+TM->
- **GCA_000169335.1; Crocosphaera chwakensis CCY0110; counts= 1; multicellularity status: TRUE**
  - AB_hydrolase->CASPASE+iSTAND*->MoxR->helical_region(leucine-richLWxKmotif)+vWA->vWA+FGS-fold_domain->
- **GCA_000170715.1; Beggiatoa sp. PS; counts= 1; multicellularity status: TRUE**
  - PNP_UDP_1->PNP_UDP_1(fragment)->iSTAND*->HTH->MoxR->
- **GCA_000155555.1; Coleofasciculus chthonoplastes PCC 7420; counts= 1; multicellularity status: TRUE**
  - <-TM||HTH+iSTAND*->MoxR->
- **GCA_000307915.1; Arthrospira platensis C1; counts= 1; multicellularity status: TRUE**
  - SLH+OprB->RadC-like_JAB+sGTPase->Coiled-coil+iSTAND*->MoxR->helical_region(leucine-richLWxKmotif)+vWA->TA_antitoxin(DUF1902)->HicA_toxin->Ntox45->
- **GCA_000585055.1; Candidatus Accumulibacter sp. BA-92; counts= 1; multicellularity status: FALSE**
  - TIR+iSTAND*->MoxR->TM+TM->vWA+TIR->TM+FGS-fold_domain->
- **GCA_000739535.1; Candidatus Vecturithrix granuli; counts= 1; multicellularity status: TRUE**
  - TIR+iSTAND*->GspA-ATPase(fragment)->Ploop_inactive->
- **GCA_003864295.1; Aphanothece sacrum FPU1; counts= 1; multicellularity status: TRUE**
  - HTH+iSTAND*->MoxR->

■ **GCA_000710505.1; Planktothrix agardhii NIVA-CYA 126/8; counts= 1; multicellularity status: TRUE**

- ??+HTH+iSTAND*->MoxR->vWA+FGE-sulfatase->

■ **GCA_000584955.2; Candidatus Accumulibacter sp. SK-01; counts= 1; multicellularity status: FALSE**

- iSTAND*->MoxR->

■ **GCA_000817745.1; Aphanocapsa montana BDHKU210001; counts= 1; multicellularity status: TRUE**

- iSTAND*->MoxR->??->

■ **GCA_000817785.1; Hassallia byssoidea VB512170; counts= 1; multicellularity status: TRUE**

- EAD9+iSTAND*->MoxR->??->

■ **GCA_000817775.1; Lyngbya confervoides BDU141951; counts= 1; multicellularity status: TRUE**

- TM+TM+TM+TM+TM->EAD9+iSTAND*->

■ **GCA_000828075.2; Tolypothrix campylonemoides VB511288; counts= 1; multicellularity status: TRUE**

- cyclic_phosphodiesterase->OppC_N+TM+BPD_transp_1->DRHyd+iSTAND*->

■ **GCA_001399705.1; Kouleothrix aurantiaca; counts= 1; multicellularity status: TRUE**

- STAND_NTPase->Coiled-coil+iSTAND*->STAND_NTPase->

■ **GCA_001428505.1; Terrabacter sp. Root181; counts= 1; multicellularity status: TRUE**

- CASPASE->EAD9+iSTAND*->MoxR->helical_region(leucine-richLWxKmotif)+vWA->CASPASE+Pkinase->

■ **GCA_001456025.1; Mastigocoleus testarum BC008; counts= 1; multicellularity status: TRUE**

- <-??+Coiled-coil+TM+TM<-??||CASPASE+APATPase+wTHT+TPR->CASPASE+iSTAND*->MoxR->helical_region(leucine-richLWxKmotif)+vWA->

■ **GCA_001580545.1; Paraburkholderia monticola; counts= 1; multicellularity status: FALSE**

- DUF805(3TM_protein)->TM+TM+TM->TIR+iSTAND*->MoxR->TM+TM+vWA+TM+TM->??->??->||<-??

■ **GCA_001712795.1; Nostoc sp. KVJ20; counts= 1; multicellularity status: TRUE**

- EAD1+VMAP-M1+iSTAND*->MoxR->helical_region(leucine-richLWxKmotif)+vWA->TM+sGTPase->

■ **GCA_000817735.2; Scytonema millei VB511283; counts= 1; multicellularity status: TRUE**

- HTH+iSTAND*->MoxR->helical_region(leucine-richLWxKmotif)+vWA->vWA+FGS-fold_domain->

■ **GCA_001746915.1; Desertifilum sp. IPPAS B-1220; counts= 1; multicellularity status: TRUE**

- iSTAND*->MoxR->helical_region(leucine-richLWxKmotif)+vWA+FGS-fold_domain->Four-helical-protein->RVT->

■ **GCA_001899605.1; Sphingobacteriales bacterium 40-81; counts= 1; multicellularity status: NA**

- <-??||DRHyd+iSTAND*->MoxR->TM+TM+beta-sandwich(Ig-like?)+vWA+TM+??+LRRs?->TM+ATP-synt_A->TM+TM->ATP-synt_B->

■ **GCA_001904675.1; Scytonema sp. HK-05; counts= 1; multicellularity status: TRUE**

- pJV1-spdB3?->cNMP_cyclase->??+iSTAND*->MoxR->helical_region(leucine-richLWxKmotif)+vWA->vWA+FGS-fold_domain->

- **GCA_001942495.1; Moorea bouillonii PNG; counts= 1; multicellularity status: TRUE**
  - Trypsin+iSTAND*->MoxR->helical_region(leucine-richLWxKmotif)+vWA->
- **GCA_002077945.1; Niastella vici; counts= 1; multicellularity status: TRUE**
  - DrHyd+iSTAND*->MoxR->TM+beta-sandwich(Ig-like?)+TM+vWA+TM+??->
- **GCA_002083395.1; Proteobacteria bacterium SG_bin4; counts= 1; multicellularity status: NA**
  - TIR+iSTAND*->MoxR->TM->TM+vWA+TM+FGS-fold_domain->
- **GCA_002084355.1; Bacteroidetes bacterium 4572_128; counts= 1; multicellularity status: FALSE**
  - TIR->iSTAND*->MoxR->Coiled-coil+??->??+DrHyd+APATPase+wHTH+TPR->TPRs->
- **GCA_002212985.1; Sphingobacteriales bacterium TSM_CSS; counts= 1; multicellularity status: NA**
  - TIR+iSTAND*->MoxR->TM+beta-sandwich(Ig-like?)+TM+vWA+TM+TM+TPRs+BetaPropeller->ZnR->||<-TM||MazG+MazG->
- **GCA_002289455.1; Calothrix elsteri CCALA 953; counts= 1; multicellularity status: TRUE**
  - CASPASE+EAD9->EAD9+iSTAND*->MoxR(fragment)->
- **GCA_002591745.1; Lewinellaceae bacterium SD302; counts= 1; multicellularity status: NA**
  - EADiS+iSTAND*->MoxR->beta-sandwich(Ig-like?)+TM+vWA+TM+OmpA->
- **GCA_002842325.1; Betaproteobacteria bacterium HGW-Betaproteobacteria-13; counts= 1; multicellularity status: FALSE**
  - DRHyd+iSTAND*->MoxR->vWA->??+TIR+CASPASE->
- **GCA_002893205.1; Cylindrospermopsis raciborskii S07; counts= 1; multicellularity status: TRUE**
  - Trypco1->Trypsin+iSTAND*->MoxR->
- **GCA_003003725.1; filamentous cyanobacterium Phorm 6; counts= 1; multicellularity status: TRUE**
  - EAD1+CASPASE+iSTAND*->MoxR->
- **GCA_002212765.2; Haliangium sp. UPWRP_2; counts= 1; multicellularity status: TRUE**
  - Coiled-coil??+iSTAND*->MoxR->helical_region(leucine-richLWxKmotif)+vWA->??->EAD7+EAD7+EAD7+EAD7+VWA->||<-??
- **GCA_003017785.1; filamentous cyanobacterium CCP5; counts= 1; multicellularity status: TRUE**
  - iSTAND*->MoxR->helical_region(leucine-richLWxKmotif)+vWA->Coiled-coil->
- **GCA_003023605.1; Bacteroidetes bacterium; counts= 1; multicellularity status: FALSE**
  - EADiS+iSTAND*->MoxR->TM+beta-rich_region+TM+vWA->
- **GCA_003261315.1; Hapalosiphonaceae cyanobacterium JJU2; counts= 1; multicellularity status: TRUE**
  - HTH+iSTAND*->MoxR->
- **GCA_003577195.1; Candidatus Brocadia sp.; counts= 1; multicellularity status: NA**
  - Trypco1->Trypsin+iSTAND*->

■ **GCA_003645185.1; Gammaproteobacteria bacterium; counts= 1; multicellularity status: NA**

- iSTAND*->

■ **GCA_003645315.1; Gammaproteobacteria bacterium; counts= 1; multicellularity status: NA**

- iSTAND*->DUF4065->BECR->MoxR->helical_bundle+TM+LysM+TM+vWA+iSvmX1+TM_region

■ **GCA_003696485.1; Bacteroidetes bacterium; counts= 1; multicellularity status: FALSE**

- EADiS+iSTAND*->STAND(fragment)->

■ **GCA_001544535.2; Caballeronia choica; counts= 1; multicellularity status: FALSE**

- Trypco2->Trypsin+iSTAND*->MoxR->TM+LRRs->

■ **GCA_900107745.1; Variovorax sp. YR216; counts= 1; multicellularity status: FALSE**

- TIR+iSTAND*->MoxR->TM+TM+vWA+BetaPropeller+PASTA->

■ **GCF_000314005.1; Spirulina subsalsa; counts= 1; multicellularity status: TRUE**

- ??+iSTAND*->MoxR->helical_region(leucine-richLWxKmotif)+vWA->TM->||<-Y1_Tnp

■ **GCF_000315565.1; Mastigocladopsis repens; counts= 1; multicellularity status: TRUE**

- EAD9+iSTAND*->MoxR->helical_region(leucine-richLWxKmotif)+vWA->

■ **GCF_000332355.1; Geitlerinema sp. PCC 7105; counts= 1; multicellularity status: TRUE**

- NTP_transferase->BetaPropeller+AB_hydrolase->||<-Trypco1||CASPASE+iSTAND*->MoxR->helical_region(leucine-richLWxKmotif)+vWA->||<-HicB_lk_antitox||vWA+FGS-fold_domain->FGS-fold_domain->

■ **GCF_000331305.1; Calothrix sp. PCC 7103; counts= 1; multicellularity status: TRUE**

- Coiled-coil+iSTAND*->MoxR->helical_region(leucine-richLWxKmotif)+vWA+FGS-fold_domain->

■ **GCF_000379765.1; Flexithrix dorotheae; counts= 1; multicellularity status: TRUE**

- TIR+iSTAND*->MoxR->

■ **GCF_000379805.1; Lewinella cohaerens; counts= 1; multicellularity status: TRUE**

- EADiS+iSTAND*->MoxR->??+beta-sandwich(Ig-like?)+TM+vWA+TM+FGE-sulfatase->||<-TPRs

■ **GCF_000447295.1; Fischerella sp. PCC 9431; counts= 1; multicellularity status: TRUE**

- TIR->iSTAND*->MoxR->helical_region(leucine-richLWxKmotif)+vWA->

■ **GCF_000426905.1; Dolichospermum circinale; counts= 1; multicellularity status: TRUE**

- EAD9+iSTAND*->MoxR->TM+TM+??+Amidase->RVT->

■ **GCF_000472745.1; Bradyrhizobium japonicum; counts= 1; multicellularity status: TRUE**

- TIR+iSTAND*->MoxR->TM+TM+vWA+TM+Sel1->??->

■ **GCF_000423565.1; Runella zeae; counts= 1; multicellularity status: TRUE**

- CASPASE+iSTAND*->MoxR->vWA->CASPASE+LRRs->SUI->??->

- **GCF_000582685.1; [Scytonema hofmanni] UTEX B 1581; counts= 1; multicellularity status: TRUE**
  - EAD9+iSTAND*->MoxR->helical_region(leucine-richLWxKmotif)+vWA->
- **GCF_000828085.2; Scytonema tolypothrichoides; counts= 1; multicellularity status: TRUE**
  - TIR+iSTAND*->MoxR->
- **GCF_001597745.1; Anabaena sp. 4-3; counts= 1; multicellularity status: TRUE**
  - ??+iSTAND*->MoxR->helical_region(leucine-richLWxKmotif)+vWA->
- **GCF_001858025.1; Trichormus sp. NMC-1; counts= 1; multicellularity status: TRUE**
  - Trypco1->Trypsin+EAD9->EAD9+iSTAND*->MoxR->helical_region(leucine-richLWxKmotif)+vWA->7TMR-HDED+Beta-rich+HTH+FGS-fold_domain->
- **GCF_000526455.1; Desulfovibrio magneticus; counts= 1; multicellularity status: FALSE**
  - DRHyd+iSTAND*->MoxR->vWA->??+TIR+APATPase+TPRs+Pentapeptide_repeats->
- **GCF_000744415.1; Ghiorsea bivora; counts= 1; multicellularity status: FALSE**
  - iSTAND+McrB*->REase->||<-SLOG||??->TM->DUF262+??+DUF1524(HNH?)->
- **GCF_000759025.1; Phaeodactylibacter xiamenensis; counts= 1; multicellularity status: TRUE**
  - CASPASE+AB_hydrolase+iSTAND*->MoxR->vWA->??+SGNH_hydrolase+CASPASE->
- **GCF_002198735.1; Ideonella sp. A 288; counts= 1; multicellularity status: FALSE**
  - TIR+iSTAND*->MoxR->BetaPropeller+FGS-fold_domain->
- **GCF_900469875.1; Rhizobiales bacterium; counts= 1; multicellularity status: FALSE**
  - iSTAND*->MoxR->TM+TM+vWA+TM+Sel1->
- **GCF_003838225.1; Okeania hirsuta; counts= 1; multicellularity status: TRUE**
  - ??+iSTAND*->MoxR->

FtsH systems

- **GCA_002720785.1; Deltaproteobacteria bacterium; counts= 2; multicellularity status: NA**
  - inactive_FtsH+FtsH*->X3_analog->
  - Radical_SAM->?->X5->inactive_FtsH+FtsH*->X3_analog->?->MoxR<-?||helical_unk+vWA->
- **GCF_000331735.1; Myxococcus stipitatus; counts= 1; multicellularity status: TRUE**
  - Radical_SAM->X6->X5->inactive_FtsH+FtsH*->X3_analog->a/b_unk+NEFF1+TPR_region->MoxR->?->helical_unk+ivWA->
- **GCA_002402105.1; Candidatus Cloacimonetes bacterium; counts= 1; multicellularity status: NA**
  - Radical_SAM->?->X5->inactive_FtsH+FtsH*->X3_analog->??+NEFF1->PSE->PSE->helical_unk+ivWA->
- **GCF_000186345.1; Isosphaera pallida; counts= 1; multicellularity status: TRUE**
  - inactive_FtsH+FtsH*->X3_analog->??+TPRs+NEFF1->MoxR->helical_unk+ivWA->
- **GCA_002747355.1; Proteobacteria bacterium; counts= 1; multicellularity status: TRUE**
  - Radical_SAM->X6->X5->inactive_FtsH+FtsH*->X3_analog->a/b_unk+NEFF1+TPR_region->MoxR->helical_unk+ivWA->
- **GCF_900101905.1; Myxococcus virescens; counts= 1; multicellularity status: TRUE**
  - Radical_SAM->X6->X5->inactive_FtsH+FtsH*->X3_analog->a/b_unk+NEFF1+TPR_region->MoxR->helical_unk+ivWA->
- **GCF_002305895.1; Myxococcus macrosporus; counts= 1; multicellularity status: TRUE**
  - Radical_SAM->X6->X5->inactive_FtsH+FtsH*->X3_analog->a/b_unk+NEFF1+TPR_region->MoxR->helical_unk+ivWA->
- **GCF_006401215.1; Myxococcus xanthus; counts= 1; multicellularity status: TRUE**
  - Radical_SAM->X6->X5->inactive_FtsH+FtsH*->X3_analog->a/b_unk+NEFF1+TPR_region->MoxR->helical_unk+ivWA->
- **GCF_006400955.1; Myxococcus xanthus; counts= 1; multicellularity status: TRUE**
  - Radical_SAM->X6->X5->inactive_FtsH+FtsH*->X3_analog->a/b_unk+NEFF1+TPR_region->MoxR->helical_unk+ivWA->
- **GCF_006401635.1; Myxococcus xanthus; counts= 1; multicellularity status: TRUE**
  - Radical_SAM->X6->X5->inactive_FtsH+FtsH*->X3_analog->a/b_unk+NEFF1+TPR_region->MoxR->helical_unk+ivWA->
- **GCF_006518195.1; Myxococcus; counts= 1; multicellularity status: TRUE**
  - Radical_SAM->X6->X5->inactive_FtsH+FtsH*->X3_analog->a/b_unk+NEFF1+TPR_region->MoxR->helical_unk+ivWA->
- **GCF_006547365.1; Myxococcus sp. AB025A; counts= 1; multicellularity status: TRUE**
  - Radical_SAM->X6->X5->inactive_FtsH+FtsH*->X3_analog->a/b_unk+NEFF1+TPR_region->MoxR->helical_unk+ivWA->

- **GCF_006547355.1; Myxococcus sp. AB024B; counts= 1; multicellularity status: TRUE**
  - Radical_SAM->X6->X5->inactive_FtsH+FtsH*->X3_analog->a/b_unk+NEFF1+TPR_region->MoxR->helical_unk+ivWA->
- **GCF_000988565.1; Myxococcus fulvus; counts= 1; multicellularity status: TRUE**
  - Radical_SAM->X6->X5->inactive_FtsH+FtsH*->X3_analog->a/b_unk+NEFF1+TPR_region->MoxR->||<-?||helical_unk+ivWA->
- **GCA_003327425.1; Candidatus Ozemobacter sibiricus; counts= 1; multicellularity status: NA**
  - inactive_FtsH+FtsH*->?->?->MoxR->helical_unk+ivWA->Radical_SAM->RadSAM_pep->
- **GCA_002839435.1; Candidatus Riflebacteria bacterium HGW-Riflebacteria-2; counts= 1; multicellularity status: NA**
  - inactive_FtsH+FtsH*->?->MoxR->helical_unk+ivWA->Radical_SAM->RadSAM_pep->?->X5->
- **GCA_002343915.1; Nannocystis exedens; counts= 1; multicellularity status: TRUE**
  - Radical_SAM->?->X5->inactive_FtsH+FtsH*->X3_analog->?->MoxR->helical_unk+ivWA->
- **GCF_900112715.1; Nannocystis exedens; counts= 1; multicellularity status: TRUE**
  - Radical_SAM->?->X5->inactive_FtsH+FtsH*->X3_analog->?->MoxR->helical_unk+ivWA->
- **GCF_000012685.1; Myxococcus; counts= 1; multicellularity status: TRUE**
  - Radical_SAM->X6->X5->inactive_FtsH+FtsH*->X3_analog->a/b_unk+NEFF1+TPR_region->MoxR->?->helical_unk+ivWA->
- **GCF_000280925.3; Myxococcus hansupus; counts= 1; multicellularity status: TRUE**
  - Radical_SAM->X6->X5->inactive_FtsH+FtsH*->X3_analog->a/b_unk+NEFF1+TPR_region->MoxR->helical_unk+ivWA->
- **GCF_002305875.1; Cystobacter fuscus; counts= 1; multicellularity status: TRUE**
  - Radical_SAM->X6->X5->inactive_FtsH+FtsH*->X3_analog->a/b_unk+NEFF1+TPR_region->MoxR->helical_unk+ivWA->?->?->||<-Radical_SAM
- **GCF_000335475.2; Cystobacter fuscus; counts= 1; multicellularity status: TRUE**
  - Radical_SAM->X6->?->X5->inactive_FtsH+FtsH*->X3_analog->a/b_unk+NEFF1+TPR_region->MoxR->helical_unk+ivWA->?->?->||<-Radical_SAM
- **GCF_000219105.1; Myxococcus macrosporus; counts= 1; multicellularity status: TRUE**
  - X6->X5->inactive_FtsH+FtsH*->X3_analog->a/b_unk+NEFF1+TPR_region->MoxR->helical_unk+ivWA->?->?->||<-Radical_SAM
- **GCF_001887355.1; Cystobacter ferrugineus; counts= 1; multicellularity status: TRUE**
  - Radical_SAM->X6->X5->inactive_FtsH+FtsH*->X3_analog->a/b_unk+NEFF1+TPR_region->MoxR->helical_unk+ivWA->?->?->||<-Radical_SAM
- **GCA_002839445.1; Candidatus Riflebacteria bacterium HGW-Riflebacteria-1; counts= 1; multicellularity status: NA**
  - inactive_FtsH+FtsH*->?->MoxR->helical_unk+ivWA->Radical_SAM->RadSAM_pep->?->X5->
- **GCF_900111765.1; Myxococcus fulvus; counts= 1; multicellularity status: TRUE**
  - Radical_SAM->X6->X5->inactive_FtsH+FtsH*->X3_analog->a/b_unk+NEFF1+TPR_region->MoxR->||<-?||helical_unk+vWA->
- **GCA_002722755.1; Proteobacteria bacterium; counts= 1; multicellularity status: TRUE**
  - Radical_SAM->?->X5->inactive_FtsH+FtsH*->X3_analog->?->MoxR->??+ivWA->||<-?||STAND_NTPase->

- **GCF_004138135.1; Oscillochloris sp. Chuk17; counts= 1; multicellularity status: TRUE**
  - TPRs->?->?->??+vWA+FGS-fold_domain->inactive_FtsH+FtsH*->REase->MoxR
- **GCF_000152145.1; Oscillochloris trichoides; counts= 1; multicellularity status: TRUE**
  - TPRs->?->?->??+vWA+FGS-fold_domain->inactive_FtsH+FtsH*->MoxR->?->?->STAND_NTPase->
- **GCF_006518215.1; Myxococcus sp. AB022; counts= 1; multicellularity status: TRUE**
  - Radical_SAM->X6->X5->inactive_FtsH+FtsH*->X3_analog->a/b_unk+NEFF1+TPR_region->MoxR->||<-?||helical_unk+ivWA->
- **GCF_000255705.1; Zavarzinella formosa; counts= 1; multicellularity status: TRUE**
  - Radical_SAM->?->Radical_SAM->X6->X5->inactive_FtsH+FtsH*->X3_analog->?->a/b_unk+NEFF+TPR_region->
- **GCF_001610855.1; Gemmata sp. SH-PL17; counts= 1; multicellularity status: FALSE**
  - Radical_SAM->X6->X5->inactive_FtsH+FtsH*->X3_analog->a/b_unk+NEFF1+TPR_region->a/b_unk+NEFF1+TPR_region->
- **GCF_000720255.1; Streptomyces griseus; counts= 1; multicellularity status: TRUE**
  - EAD1+CASPASE->RING(inactive)+RING->E1+UFD->EAD1+Ubl-Nterm+Ubl+E2->JAB-N+JAB->X1->inactive_FtsH+FtsH*->MoxR->X2->X3->TPRs->low_complexity+a/b_unk+TPR_repeat<-BetaPropeller+vWA<-RadSAM_pep<-RADICAL_SAM.Ammemo
- **GCF_000317435.1; Calothrix parietina; counts= 1; multicellularity status: TRUE**
  - E1+UFD->EAD8+Trypsin->TPRs+FGS-fold_domain->EAD8+EAD1->EAD1+Ubl-Nterm+Ubl+E2->JAB-N+JAB->X1->inactive_FtsH+FtsH*->TPRs+C-type_lectin->MoxR->?->X3->TPRs+C-type_lectin->TPRs+C-type_lectin->?->Clp_N_repeats+vWA<-RADICAL_SAM.Ammemo
- **GCF_002027195.1; Streptomyces sp. GKU 895; counts= 1; multicellularity status: TRUE**
  - E1+UFD->E1+UFD->EAD1+Ubl-Nterm+Ubl+E2->JAB_N+JAB->X1->inactive_FtsH+FtsH*->MoxR->X2->X3->PSE->?->beta_propeller(frag?)
- **GCF_000716715.1; Dactylosporangium aurantiacum; counts= 1; multicellularity status: TRUE**
  - E1+UFD->EAD1+Ubl-Nterm+Ubl+E2->JAB_N+JAB->X1->inactive_FtsH+FtsH*->MoxR->X2->X3->TPRs->low_complexity+a/b_unk+TPR_repeat->BetaPropeller+vWA->||<-RadSAM_pep<-RADICAL_SAM.Ammemo
- **GCF_900100605.1; Sinosporangium album; counts= 1; multicellularity status: TRUE**
  - E1+UFD->EAD1+Ubl-Nterm+Ubl+E2->JAB_N+JAB->X1->inactive_FtsH+FtsH*->MoxR->X2->X3->TPRs->low_complexity+a/b_unk+TPR_repeat->BetaPropeller+vWA->||<-RadSAM_pep
- **GCF_000972385.1; Streptomyces sp. WM6386; counts= 1; multicellularity status: TRUE**
  - <-DO-GTPase2<-Pkinase+TM||EAD1+CASPASE->RING(inactive)+RING->E1+UFD->EAD1+Ubl-Nterm+Ubl+E2->JAB_N+JAB*->X1->inactive_FtsH+FtsH->MoxR->??->X2->X3->TPRs->low_complexity+a/b_unk+TPR_repeat->BetaPropeller+vWA->||<-RadSAM_pep<-RADICAL_SAM.Ammemo<-MPTase<-RadSAM.X1
- **GCF_000746395.1; Streptomyces mirabilis; counts= 1; multicellularity status: TRUE**
  - <-TM+??<-TM+coiled-coil<-GAP1-N2+GAP1-M+??<-DO-GTPase2<-Pkinase+TM||EAD1+CASPASE->RING(inactive)+RING->E1+UFD->EAD1+Ubl-Nterm+Ubl+E2->JAB_N+JAB->X1->inactive_FtsH+FtsH*->MoxR->X2->X3->TPRs->low_complexity+a/b_unk+TPR_repeat->BetaPropeller+vWA->||<-RadSAM_pep<-RADICAL_SAM.Ammemo<-MPTase
- **GCF_001514035.1; Streptomyces yokosukanensis; counts= 1; multicellularity status: TRUE**
  - EAD1+CASPASE->RING(inactive)+RING->E1+UFD->EAD1+Ubl-Nterm+Ubl+E2->JAB_N+JAB->X1->inactive_FtsH+FtsH*->MoxR->X2->X3->TPRs->?->BetaPropeller+vWA<-RadSAM_pep<-RADICAL_SAM.Ammemo<-MPTase
- **GCA_004345645.1; Actinocrispum wychmicini; counts= 1; multicellularity status: TRUE**
  - <-TM+??<-TM+coiled-coil<-GAP1-N2+GAP1-M+??<-DO-GTPase2<-Pkinase+TM||EAD1+CASPASE->RING(inactive)+RING->E1+UFD->EAD1+Ubl-Nterm+Ubl+E2->JAB_N+JAB->X1->inactive_FtsH+FtsH*->MoxR->X2->X3->TPRs->low_complexity+a/b_unk+TPR_repeat->BetaPropeller+vWA->||<-RadSAM_pep<-RADICAL_SAM.Ammemo
- **GCF_000384295.1; Amycolatopsis balhimycina; counts= 1; multicellularity status: TRUE**
  - EAD1+CASPASE->RING(inactive)+RING->E1+UFD->EAD1+Ubl-Nterm+Ubl+E2->JAB_N+JAB->X1->inactive_FtsH+FtsH*->MoxR->X2->X3->TPRs->low_complexity+a/b_unk+TPR_repeat->BetaPropeller+vWA->||<-RadSAM_pep<-RADICAL_SAM.Ammemo<-TM+TM

- **GCF_002234595.1; Amycolatopsis sp. H5; counts= 1; multicellularity status: TRUE**
  - EAD1+CASPASE->RING(inactive)+RING->E1+UFD->EAD1+Ubl-Nterm+Ubl+E2->JAB_N+JAB->X1->inactive_FtsH+FtsH*->MoxR->X2->X3->TPRs->low_complexity+a/b_unk+TPR_repeat->BetaPropeller+vWA->||<-RadSAM_pep<-RADICAL_SAM.Ammemo<-TM+TM
- **GCF_003955715.1; Streptomyces sp. GGCR-6; counts= 1; multicellularity status: TRUE**
  - EAD1+CASPASE->RING(inactive)+RING->E1+UFD->EAD1+Ubl-Nterm+Ubl+E2->JAB_N+JAB->X1->inactive_FtsH+FtsH*->MoxR->X2->X3->TPRs->low_complexity+a/b_unk+TPR_repeat->BetaPropeller+vWA->||<-RadSAM_pep<-RADICAL_SAM.Ammemo
- **GCF_004345645.1; Actinocrispum wychmicini; counts= 1; multicellularity status: TRUE**
  - <-TM+??<-TM+coiled-coil<-GAP1-N2+GAP1-M+??<-DO-GTPase2<-Pkinase+TM||EAD1+CASPASE->RING(inactive)+RING->E1+UFD->EAD1+Ubl-Nterm+Ubl+E2->JAB_N+JAB->X1->inactive_FtsH+FtsH*->MoxR->X2->X3->TPRs->low_complexity+a/b_unk+TPR_repeat->BetaPropeller+vWA->||<-RadSAM_pep<-RADICAL_SAM.Ammemo
- **GCF_004343295.1; Streptomyces sp. BK042; counts= 1; multicellularity status: TRUE**
  - EAD1+CASPASE->RING(inactive)+RING->E1+UFD->EAD1+Ubl-Nterm+Ubl+E2->JAB_N+JAB->X1->inactive_FtsH+FtsH*->MoxR->X2->X3->TPRs->low_complexity+a/b_unk+TPR_repeat->BetaPropeller+vWA->||<-RadSAM_pep<-RADICAL_SAM.Ammemo
- **GCF_900104725.1; Actinoplanes derwentensis; counts= 1; multicellularity status: TRUE**
  - Trypsin+EAD1->BetaPropeller+vWA->RADICAL_SAM.Ammemo->RadSAM_pep->RING(inactive)+RING->E1+UFD->EAD1+Ubl-Nterm+Ubl+E2->JAB_N+JAB->X1->inactive_FtsH+FtsH*->MoxR->X2->X3->TPRs->low_complexity+a/b_unk+TPR_repeat->CASPASE+Ploop_NTPase->BetaPropeller->ABhydrolase+TIR->EAD1+TIR->BetaPropeller+vWA->
- **GCF_000725475.1; Streptomyces durhamensis; counts= 1; multicellularity status: TRUE**
  - EAD1+CASPASE->RING(inactive)+RING->E1+UFD->EAD1+Ubl-Nterm+Ubl+E2->JAB_N+JAB->X1->inactive_FtsH+FtsH*->MoxR->X2->X3->TPRs->low_complexity+a/b_unk+TPR_repeat->BetaPropeller+vWA->||<-RadSAM_pep<-RADICAL_SAM.Ammemo<-MPTase<-RadSAM.X1
- **GCF_001280015.1; Streptomyces sp. MMG1121; counts= 1; multicellularity status: TRUE**
  - EAD1+CASPASE->RING(inactive)+RING->E1+UFD->EAD1+Ubl-Nterm+Ubl+E2->JAB_N+JAB->X1->inactive_FtsH+FtsH*->MoxR->X2->X3->TPRs->low_complexity+a/b_unk+TPR_repeat->BetaPropeller+vWA->||<-RadSAM_pep<-RADICAL_SAM.Ammemo<-MPTase<-RadSAM.X1
- **GCF_001866665.1; Streptomyces sp. MUSC 1; counts= 1; multicellularity status: TRUE**
  - EAD1+CASPASE->RING(inactive)+RING->E1+UFD->EAD1+Ubl-Nterm+Ubl+E2->JAB_N+JAB->X1->inactive_FtsH+FtsH*->MoxR->X2->X3->TPRs->low_complexity+a/b_unk+TPR_repeat->BetaPropeller+vWA->||<-RadSAM_pep<-RADICAL_SAM.Ammemo<-MPTase<-RadSAM.X1
- **GCF_001735805.1; Streptomyces puniciscabiei; counts= 1; multicellularity status: TRUE**
  - <-TM+??<-TM+coiled-coil<-GAP1-N2+GAP1-M+??<-DO-GTPase2<-Pkinase+TM||EAD1+CASPASE->RING(inactive)+RING->E1+UFD->EAD1+Ubl-Nterm+Ubl+E2->JAB_N+JAB->X1->inactive_FtsH+FtsH*->MoxR->X2->X3->TPRs->low_complexity+a/b_unk+TPR_repeat->||<-BetaPropeller+vWA<-RadSAM_pep<-RADICAL_SAM.Ammemo<-MPTase
- **GCF_000379645.1; Actinoplanes globisporus; counts= 1; multicellularity status: TRUE**
  - Clp_N_repeats+vWA->Clp_N_repeats+vWA->Clp_N_repeats+vWA->Clp_N_repeats+vWA->Clp_N_repeats+vWA->Clp_N_repeats+vWA->??->??+4TM->EAD1+CASPASE->RADICAL_SAM.Ammemo->RadSAM_pep->RING(inactive)+RING->E1+UFD->EAD1+Ubl-Nterm+Ubl+E2->JAB_N+JAB->X1->inactive_FtsH+FtsH*->MoxR->X2->?->TPRs->low_complexity+a/b_unk+TPRs->
- **GCF_004364215.1; Streptomyces sp. BK161; counts= 1; multicellularity status: TRUE**
  - EAD1+CASPASE->RING(inactive)+RING->E1+UFD->EAD1+Ubl-Nterm+Ubl+E2->JAB_N+JAB->X1->inactive_FtsH+FtsH*->MoxR->X2->X3->TPRs->low_complexity+a/b_unk+TPRs->||<-BetaPropeller+vWA<-RadSAM_pep<-RADICAL_SAM.Ammemo
- **GCA_006912135.1; Cryptosporangium sp. A-T 5661; counts= 1; multicellularity status: TRUE**
  - BetaPropeller+vWA->BetaPropeller+vWA->BetaPropeller+vWA->||<-TIR+EAD1||BetaPropeller+vWA->RADICAL_SAM.Ammemo->RadSAM_pep->RING(inactive)+RING->E1+UFD->EAD1+Ubl-Nterm+Ubl+E2->JAB_N+JAB->X1->inactive_FtsH+FtsH*->MoxR->X2->X3->TPRs->low_complexity+a/b_unk+TPRs->
- **GCF_005144585.1; Polyangium fumosum; counts= 1; multicellularity status: TRUE**
  - <-RadSAM_pep<-Radical_SAM<-BetaPropeller+vWA||Prok_Ring(degenerate)+Prok_Ring->E1+UFD->EAD1+Ubl-Nterm+Ubl+E2->JAB-N+JAB->X1->inactive_FtsH+FtsH*->MoxR->?->TPRs->low_complexity+a/b_unk+TPRs->
- **GCF_004217135.1; Micromonospora violae; counts= 1; multicellularity status: TRUE**
  - Clp_N_repeats+vWA->??->??+4TM->EAD1+CASPASE->RING(inactive)+RING->E1+UFD->EAD1+Ubl-Nterm+Ubl+E2->JAB_N+JAB->X1->inactive_FtsH+FtsH*->MoxR->TPRs->low_complexity+a/b_unk+TPR_repeat->
- **GCA_001292585.1; Candidatus Magnetomorum sp. HK-1; counts= 1; multicellularity status: TRUE**
  - TIR+??->||<-?||Ubl+E2->JAB-N+JAB->inactive_FtsH+FtsH*->E1+UFD->TPRs+FGS-fold_domain->

- **GCF_002154695.1; Nostoc sp. T09; counts= 1; multicellularity status: TRUE**
  - RadSAM_pep->RING(inactive)+RING->E1+UFD->Trypco2->TCAD4+CASPASE+EAD1->EAD1+Ubl-Nterm+Ubl+E2->JAB_N+JAB->X1->inactive_FtsH+FtsH*->TPRs+FGS-fold_domain->MoxR->?->?->FGS-fold_domain->||<-?||?->||<-BetaPropeller+vWA||RADICAL_SAM.Ammemo->
- **GCF_003143935.1; Streptomyces sp. Act143; counts= 1; multicellularity status: TRUE**
  - EAD1+CASPASE->RING(inactive)+RING->E1+UFD->EAD1+Ubl-Nterm+Ubl+E2->JAB_N+JAB->X1->inactive_FtsH+FtsH*->MoxR->X2->X3->TPRs->low_complexity+a/b_unk+TPR_repeat->||<-BetaPropeller+vWA<-RadSAM_pep<-RADICAL_SAM.Ammemo
- **GCA_000516475.1; Candidatus Magnetoglobus multicellularis str. Araruama; counts= 1; multicellularity status: TRUE**
  - inactive_FtsH+FtsH*->inactive_FtsH+FtsH->Prok_Ring+Prok_Ring->E1+UFD->TIR+??->Ubl+E2->JAB_N2->JAB->
- **GCA_900104725.1; Actinoplanes derwentensis; counts= 1; multicellularity status: TRUE**
  - Trypsin+EAD1->BetaPropeller+vWA->RADICAL_SAM.Ammemo->RadSAM_pep->RING(inactive)+RING->E1+UFD->EAD1+Ubl-Nterm+Ubl+E2->JAB_N+JAB->X1->inactive_FtsH+FtsH*->MoxR->X2->X3->TPRs->low_complexity+a/b_unk+TPR_repeat->CASPASE+Ploop_NTPase->beta_propeller->ABhydrolase+TIR->EAD1+TIR->BetaPropeller+vWA->
- **GCA_003695705.1; Planctomycetes bacterium; counts= 1; multicellularity status: NA**
  - MPTase->||<-Prok-RING+TM<-?<-Prok-RING+TM+TM<-Band7<-Band7||inactive_FtsH+FtsH*->X3_analog->
- **GCF_001767235.1; Moorea producens; counts= 1; multicellularity status: TRUE**
  - RadSAM_pep->Prok_Ring(degenerate)+Prok_Ring->X1->TPRs->inactive_FtsH+FtsH*->MoxR->?->?->TPRs+FGS-fold_domain->->||<-?||?->?->||<-RADICAL_SAM.Ammemo
- **GCF_000262465.1; Frankia sp. QA3; counts= 1; multicellularity status: TRUE**
  - E1+UFD->EAD1+Ubl-Nterm+Ubl+E2->JAB_N+JAB->X1->X1->inactive_FtsH+FtsH*->MoxR->Clathrin+DnaJ_CXXCXGXG+DnaJ_C->low_complexity+a/b_unk+TPR_repeat->||<-?<-TIR
- **GCF_900206255.1; Streptomyces sp. TLI_55; counts= 1; multicellularity status: TRUE**
  - RING(inactive)+RING->E1+UFD->EAD1+Ubl-Nterm+Ubl+E2->||<-PSE->X1->inactive_FtsH+FtsH*->MoxR->?->?->TPRs->low_complexity+a/b_unk+TPR_repeat->BetaPropeller+vWA->||<-RadSAM_pep<-RADICAL_SAM.Ammemo
- **GCF_001469165.1; Thiohalocapsa sp. ML1; counts= 1; multicellularity status: TRUE**
  - RING(inactive)+RING->E1+UFD->helical_X+TIR->EAD1+Ubl-Nterm+Ubl+E2->||<-PSE||?->inactive_FtsH+FtsH*->MoxR->BetaPropeller+vWA->X2->X3?->TPRs->low_complexity+a/b_unk+TPR_repeat->||<-?||BetaPropeller+vWA->HTH->RADICAL_SAM.Ammemo->RadSAM_pep->
- **GCF_900634715.1; Frankia symbiont of Datisca glomerata; counts= 1; multicellularity status: TRUE**
  - RadSAM_pep->RING(inactive)+RING->E1+?->TIR->||<-?<-?<-?||X1->inactive_FtsH+FtsH*->MoxR->
- **GCF_000024805.1; Haliangium ochraceum; counts= 1; multicellularity status: TRUE**
  - beta_propeller+vWA->X1->inactive_FtsH+FtsH*->MoxR->X2->X3->?->Radical_SAM->RadSAM_pep->
- **GCF_001017755.1; Frankia coriariae; counts= 1; multicellularity status: TRUE**
  - X1->inactive_FtsH+FtsH*->MoxR->X2->
- **GCA_001787855.1; Candidatus Riflebacteria bacterium GWC2_50_8; counts= 1; multicellularity status: NA**
  - inactive_FtsH+FtsH*->?->MoxR->
- **GCF_900635405.1; Frankia symbiont of Datisca glomerata; counts= 1; multicellularity status: TRUE**
  - inactive_FtsH+FtsH*->MoxR->
- **GCA_003645185.1; Gammaproteobacteria bacterium; counts= 1; multicellularity status: NA**
  - inactive_FtsH+FtsH*->?->

- **GCA_001873925.1; Deltaproteobacteria bacterium CG2_30_63_29; counts= 1; multicellularity status: NA**
  - X5->inactive_FtsH+FtsH*->
- **GCA_002789955.1; Deltaproteobacteria bacterium CG_4_9_14_3_um_filter_63_12; counts= 1; multicellularity status: NA**
  - X5->inactive_FtsH+FtsH*->
- **GCA_000821145.1; uncultured bacterium; counts= 1; multicellularity status: NA**
  - X6->X5->inactive_FtsH+FtsH*->
- **GCF_006636215.1; Myxococcus sp. AM401; counts= 1; multicellularity status: TRUE**
  - Radical_SAM->X6->X5->inactive_FtsH+FtsH*->X3_analog->a/b_unk+NEFF1+TPR_region->MoxR->
- **GCF_001854205.1; Moorea producens; counts= 1; multicellularity status: TRUE**
  - inactive_FtsH+FtsH*->MoxR->?->||<-?<-?||X2->
- **GCF_000171775.1; Gemmata obscuriglobus; counts= 1; multicellularity status: FALSE**
  - Radical_SAM->X6->X5->inactive_FtsH+FtsH*->X3_analog->a/b_unk+NEFF1+TPR_region->a/b_unk+NEFF1+TPR_region->
- **GCA_003669555.1; Planctomycetes bacterium; counts= 1; multicellularity status: NA**
  - X5->inactive_FtsH+FtsH*->X3_analog->?+NEFF+?->MoxR->
- **GCA_002716585.1; Myxococcales bacterium; counts= 1; multicellularity status: TRUE**
  - inactive_FtsH+FtsH*->X3_analog->
- **GCA_006226375.1; Deltaproteobacteria bacterium; counts= 1; multicellularity status: NA**
  - inactive_FtsH+FtsH*->X3_analog->
- **GCF_002197845.1; Fimbriiglobus ruber; counts= 1; multicellularity status: TRUE**
  - inactive_FtsH+FtsH*->X3_analog->
- **GCA_004376375.1; Planctomycetes bacterium; counts= 1; multicellularity status: NA**
  - X5->inactive_FtsH+FtsH*->?->?->X3_analog->
- **GCA_003243425.1; Archangium gephyra; counts= 1; multicellularity status: TRUE**
  - X5->inactive_FtsH+FtsH*->X3_analog->
- **GCF_000170895.1; Plesiocystis pacifica; counts= 1; multicellularity status: TRUE**
  - X5->inactive_FtsH+FtsH*->X3_analog->?->a/b_unk+NEFF+TPRs
- **GCA_003508275.1; Planctomycetales bacterium; counts= 1; multicellularity status: NA**
  - X6->X5->inactive_FtsH+FtsH*->
- **GCF_002994615.1; Enhygromyxa salina; counts= 1; multicellularity status: TRUE**
  - Radical_SAM->X6->X5->inactive_FtsH+FtsH*->

- **GCF_900635385.1; Frankia symbiont of Coriaria nepalensis; counts= 1; multicellularity status: TRUE**
    - inactive_FtsH+FtsH*->MoxR->X2->||<-?||TPRs->
- **GCF_000177615.2; Frankia; counts= 1; multicellularity status: TRUE**
    - X1->inactive_FtsH+FtsH*->MoxR->X2->||<-?||TPRs->
- **GCF_001983005.1; Frankia sp. BMG5.30; counts= 1; multicellularity status: TRUE**
    - X1->inactive_FtsH+FtsH*->MoxR->X2->?->TPRs->

β-propeller systems

■ **GCA__900104725.1; Actinoplanes derwentensis; counts= 2; multicellularity status: TRUE**

- ??->Trypsin+EAD1->beta__propeller+helical__linker+vWA*->RADICAL__SAM.Ammemo->RadSAM__pep->?->E1+UFD->EAD1+Ubl-Nterm+Ubl+E2->
- low__complexity+a/b__unk+TPR__repeat->CASPASE+Ploop__NTPase->BetaPropeller->a/b__hydrolase->EAD1+TIR->beta__propeller+helical__linker+vWA*->

■ **GCA__000516475.1; Candidatus Magnetoglobus multicellularis str. Araruama; counts= 2; multicellularity status: TRUE**

- vWA*->
- vWA*->

■ **GCA__001769385.1; Bacteroidetes bacterium RIFCSPLOWO2__12__FULL__35__15; counts= 1; multicellularity status: FALSE**

- bpX4->MoxR->bpX2->extended__strand+helical__region+vWA+beta__propeller+beta__rich__region+bpX1+Ribosomal__L12__insert*->bpX3->

■ **GCA__000265505.1; Bernardetia litoralis DSM 6794; counts= 1; multicellularity status: NA**

- bpX4->?->MoxR->?->bpX2->extended__strand+helical__region+vWA+beta__propeller+beta__rich__region+bpX1+Ribosomal__L12__insert*->

■ **GCA__002916795.1; Flavobacterium columnare; counts= 1; multicellularity status: FALSE**

- bpX4->MoxR->bpX2->extended__strand+helical__region+vWA+beta__propeller+beta__rich__region+bpX1+Ribosomal__L12__insert*->abpX3+2TM+TPR__region->

■ **GCA__002217335.1; Flavobacterium hydatis; counts= 1; multicellularity status: FALSE**

- bpX4->MoxR->bpX2->extended__strand+helical__region+vWA+beta__propeller+beta__rich__region+bpX1+Ribosomal__L12__insert*->

■ **GCA__900110335.1; Flavobacterium sp. fv08; counts= 1; multicellularity status: FALSE**

- bpX4->MoxR->bpX2->extended__strand+helical__region+vWA+beta__propeller+beta__rich__region+bpX1+Ribosomal__L12__insert*->abpX3+2TM+TPR__region->

■ **GCA__001281695.1; bacterium 336/3; counts= 1; multicellularity status: NA**

- bpX4->MoxR->bpX2->extended__strand+helical__region+vWA+beta__propeller+beta__rich__region+bpX1*->

■ **GCF__004014855.1; Apibacter sp. HY041; counts= 1; multicellularity status: FALSE**

- bpX4->MoxR->bpX2->extended__strand+helical__region+vWA+beta__propeller+beta__rich__region+bpX1*->bpX3+4TM->2TM+TPR__region->

■ **GCA__003148625.1; Arcicella aurantiaca; counts= 1; multicellularity status: FALSE**

- bpX4->MoxR->bpX2->extended__strand+helical__region+vWA+beta__propeller+beta__rich__region+bpX1*->bpX3+TPR__region->

■ **GCF__000724545.1; Aureispira sp. CCB-QB1; counts= 1; multicellularity status: TRUE**

- bpX4->MoxR->bpX2->extended__strand+helical__region+vWA+beta__propeller+beta__rich__region+bpX1*->bpX3+TPR__region->

■ **GCA__003014755.1; Chitinophaga niastensis; counts= 1; multicellularity status: TRUE**

- MoxR->bpX2->extended__strand+helical__region+vWA+beta__propeller+beta__rich__region+bpX1*->bpX3+TPR__region+beta__propeller->

- **GCA_003308995.1; Chitinophaga sp. GDMCC 1.1325; counts= 1; multicellularity status: TRUE**
  - bpX4->MoxR->bpX2->extended_strand+helical_region+vWA+beta_propeller+beta_rich_region+bpX1*->bpX3+TPR_region+beta_propeller->
- **GCA_003412465.1; Chitinophaga sp. K20C18050901; counts= 1; multicellularity status: TRUE**
  - bpX4->MoxR->bpX2->extended_strand+helical_region+vWA+beta_propeller+beta_rich_region+bpX1*->bpX3+TPR_region+beta_propeller->HTH+TetR_C->
- **GCA_003182155.1; Chitinophaga sp. S165; counts= 1; multicellularity status: TRUE**
  - bpX4->MoxR->bpX2->extended_strand+helical_region+vWA+beta_propeller+beta_rich_region+bpX1*->bpX3+TPR_region+beta_propeller->HTH+TetR_C->
- **GCA_900110995.1; Chitinophaga sp. YR573; counts= 1; multicellularity status: TRUE**
  - bpX4->MoxR->bpX2->extended_strand+helical_region+vWA+beta_propeller+beta_rich_region+bpX1*->bpX3+TPR_region+beta_propeller->HTH+TetR_C->
- **GCA_900114995.1; Chitinophaga sp. YR627; counts= 1; multicellularity status: TRUE**
  - bpX4->MoxR->bpX2->extended_strand+helical_region+vWA+beta_propeller+beta_rich_region+bpX1*->bpX3+TPR_region+beta_propeller->
- **GCA_900100115.1; Chryseobacterium soldanellicola; counts= 1; multicellularity status: FALSE**
  - MoxR->bpX2->extended_strand+helical_region+vWA+beta_propeller+beta_rich_region+bpX1*->bpX3+4TM->2TM+TPR_region->
- **GCA_000014145.1; Cytophaga hutchinsonii ATCC 33406; counts= 1; multicellularity status: FALSE**
  - bpX4->MoxR->bpX2->extended_strand+helical_region+vWA+beta_propeller+beta_rich_region+bpX1*->bpX3+TPR_region->
- **GCF_900343125.1; Dysgonomonas sp. Marseille-P4361; counts= 1; multicellularity status: FALSE**
  - bpX4->MoxR->bpX2->extended_strand+helical_region+vWA+beta_propeller+beta_rich_region+bpX1*->bpX3+TPR_region->
- **GCA_002355595.1; Filimonas lacunae; counts= 1; multicellularity status: TRUE**
  - bpX4->MoxR->bpX2->extended_strand+helical_region+vWA+beta_propeller+beta_rich_region+bpX1-*>extended_strand+helical_region+vWA+beta_propeller+beta_rich_region+bpX1*->bpX3+TPR_region->
- **GCA_003097095.1; Filimonas sp. YR581; counts= 1; multicellularity status: TRUE**
  - bpX4->MoxR->bpX2->extended_strand+helical_region+vWA+beta_propeller+beta_rich_region+bpX1*->bpX3+TPR_region->
- **GCA_000814475.1; Flavihumibacter solisilvae; counts= 1; multicellularity status: FALSE**
  - bpX4->MoxR->bpX2->extended_strand+helical_region+vWA+beta_propeller+beta_rich_region+bpX1*->bpX3+TPR_region->
- **GCA_001601815.1; Flavihumibacter sp. CACIAM 22H1; counts= 1; multicellularity status: FALSE**
  - bpX4->MoxR->bpX2->extended_strand+helical_region+vWA+beta_propeller+beta_rich_region+bpX1*->bpX3+TPR_region->
- **GCA_001644645.1; Flavisolibacter tropicus; counts= 1; multicellularity status: FALSE**
  - bpX4->MoxR->bpX2->extended_strand+helical_region+vWA+beta_propeller+beta_rich_region+bpX1*->bpX3->
- **GCA_003096035.1; Flavobacterium album; counts= 1; multicellularity status: FALSE**
  - bpX4->MoxR->bpX2->extended_strand+helical_region+vWA+beta_propeller+beta_rich_region+bpX1*->abpX3+2TM+TPR_region->
- **GCF_000967805.1; Flavobacterium sp. 316; counts= 1; multicellularity status: FALSE**
  - bpX4->MoxR->bpX2->extended_strand+helical_region+vWA+beta_propeller+beta_rich_region+bpX1*->bpX3+4TM->2TM+TPR_region->

- **GCA_000212735.1; Haliscomenobacter hydrossis DSM 1100; counts= 1; multicellularity status: TRUE**
  - bpX4->MoxR->bpX2->extended_strand+helical_region+vWA+beta_propeller+beta_rich_region+bpX1*->bpX3+TPR_region+beta_propeller->
- **GCA_002797555.1; Hymenobacter chitinivorans DSM 11115; counts= 1; multicellularity status: FALSE**
  - bpX4->MoxR->bpX2->extended_strand+helical_region+vWA+beta_propeller+beta_rich_region+bpX1*->bpX3+2TM+TPR_region+beta_propeller->
- **GCA_003970915.1; Hymenobacter gummosus; counts= 1; multicellularity status: FALSE**
  - MoxR->bpX2->extended_strand+helical_region+vWA+beta_propeller+beta_rich_region+bpX1*->
- **GCA_900188255.1; Hymenobacter mucosus; counts= 1; multicellularity status: FALSE**
  - bpX4->MoxR->bpX2->extended_strand+helical_region+vWA+beta_propeller+beta_rich_region+bpX1*->bpX3+TPR_region+beta_propeller->
- **GCF_000420705.1; Hymenobacter norwichensis; counts= 1; multicellularity status: FALSE**
  - bpX4->MoxR->bpX2->extended_strand+helical_region+vWA+beta_propeller+beta_rich_region+bpX1*->
- **GCA_003944705.1; Hymenobacter sp. 9PBR-2; counts= 1; multicellularity status: FALSE**
  - MoxR->bpX2->extended_strand+helical_region+vWA+beta_propeller+beta_rich_region+bpX1*->
- **GCA_000154725.1; Kordia algicida OT-1; counts= 1; multicellularity status: FALSE**
  - bpX4->MoxR->bpX2->extended_strand+helical_region+vWA+beta_propeller+beta_rich_region+bpX1*->bpX3+4TM->2TM+TPR_region->
- **GCA_003633615.1; Mucilaginibacter gracilis; counts= 1; multicellularity status: FALSE**
  - bpX4->MoxR->bpX2->extended_strand+helical_region+vWA+beta_propeller+beta_rich_region+bpX1*->
- **GCA_000166195.3; Mucilaginibacter paludis DSM 18603; counts= 1; multicellularity status: FALSE**
  - extended_strand+helical_region+vWA+beta_propeller+beta_rich_region+bpX1*->bpX3+TPR_region+beta_propeller->
- **GCA_004005815.1; Mucilaginibacter sp. YBJ-36; counts= 1; multicellularity status: FALSE**
  - bpX4->MoxR->bpX2->extended_strand+helical_region+vWA+beta_propeller+beta_rich_region+bpX1*->bpX3+2TM+TPR_region+beta_propeller->
- **GCA_000246855.1; Niastella koreensis GR20-10; counts= 1; multicellularity status: FALSE**
  - bpX4->MoxR->bpX2->extended_strand+helical_region+vWA+beta_propeller+beta_rich_region+bpX1*->bpX3+TPR_region+beta_propeller->
- **GCA_001442625.1; Pedobacter ginsenosidimutans; counts= 1; multicellularity status: FALSE**
  - bpX4->MoxR->bpX2->extended_strand+helical_region+vWA+beta_propeller+beta_rich_region+bpX1*->bpX3+2TM+TPR_region+beta_propeller->
- **GCA_003852495.1; Taibaiella sp. KBW10; counts= 1; multicellularity status: FALSE**
  - bpX4->MoxR->bpX2->extended_strand+helical_region+vWA+beta_propeller+beta_rich_region+bpX1*->bpX3+4TM->2TM+TPR_region->
- **GCA_002967765.1; Blastopirellula marina; counts= 1; multicellularity status: TRUE**
  - bpX4->MoxR->bpX2->extended_strand+helical_region+vWA+beta_propeller+beta_rich_region+bpX1*->bpX3+TPR_region+beta_propeller->
- **GCA_002197845.1; Fimbriiglobus ruber; counts= 1; multicellularity status: TRUE**
  - bpX2->extended_strand+helical_region+vWA+beta_propeller+beta_rich_region+bpX1*->bpX3+TPR_region+beta_propeller->

- **GCA_001610855.1; Gemmata sp. SH-PL17; counts= 1; multicellularity status: FALSE**
  - bpX4->MoxR->bpX2->extended_strand+helical_region+vWA+beta_propeller+beta_rich_region+bpX1*->bpX3+TPR_region+beta_propeller->
- **GCA_003977685.1; Isosphaeraceae bacterium GM2012; counts= 1; multicellularity status: TRUE**
  - bpX4->MoxR->bpX2->extended_strand+helical_region+vWA+beta_propeller+beta_rich_region+bpX1*->
- **GCA_900113665.1; Planctomicrobium piriforme; counts= 1; multicellularity status: TRUE**
  - bpX4->MoxR->bpX2->extended_strand+helical_region+vWA+beta_propeller+beta_rich_region+bpX1*->bpX3+TPR_region+beta_propeller->
- **GCA_001610835.1; Planctomyces sp. SH-PL14; counts= 1; multicellularity status: FALSE**
  - bpX4->MoxR->bpX2->extended_strand+helical_region+vWA+beta_propeller+beta_rich_region+bpX1*->bpX3+TPR_region+beta_propeller->
- **GCA_003576915.1; Planctomycetes bacterium; counts= 1; multicellularity status: NA**
  - MoxR->bpX2->extended_strand+helical_region+vWA+beta_propeller+beta_rich_region+bpX1*->
- **GCA_003669275.1; Planctomycetes bacterium; counts= 1; multicellularity status: NA**
  - bpX4->MoxR->bpX2->extended_strand+helical_region+vWA+beta_propeller+beta_rich_region+bpX1*->bpX3+2TM+TPR_region+??->
- **GCA_002251265.1; Rhodopirellula sp. MGV; counts= 1; multicellularity status: TRUE**
  - bpX4->MoxR->bpX2->extended_strand+helical_region+vWA+beta_propeller+beta_rich_region+bpX1*->bpX3+TPR_region+beta_propeller->
- **GCF_001642915.1; Roseimaritima ulvae; counts= 1; multicellularity status: TRUE**
  - bpX4->MoxR->bpX2->extended_strand+helical_region+vWA+beta_propeller+beta_rich_region+bpX1*->bpX3+TPR_region+beta_propeller->
- **GCF_000255655.1; Schlesneria paludicola; counts= 1; multicellularity status: TRUE**
  - extended_strand+helical_region+vWA+beta_propeller+beta_rich_region+bpX1*->bpX3+TPR_region+beta_propeller->
- **GCA_900129635.1; Singulisphaera sp. GP187; counts= 1; multicellularity status: TRUE**
  - bpX4->MoxR->bpX2->extended_strand+helical_region+vWA+beta_propeller+beta_rich_region+bpX1*->bpX3+TPR_region+beta_propeller->
- **GCF_000255705.1; Zavarzinella formosa; counts= 1; multicellularity status: TRUE**
  - extended_strand+helical_region+vWA+beta_propeller+beta_rich_region+bpX1*->
- **GCF_003856375.1; Glaciecola sp. THG-3.7; counts= 1; multicellularity status: FALSE**
  - bpX4->MoxR->bpX2->extended_strand+helical_region+vWA+beta_propeller+beta_rich_region+bpX1*->bpX3+TPR_region->
- **GCA_000173075.1; Chthoniobacter flavus Ellin428; counts= 1; multicellularity status: FALSE**
  - bpX4->MoxR->bpX2->extended_strand+helical_region+vWA+beta_propeller+beta_rich_region+bpX1*->bpX3+TPR_region+beta_propeller->
- **GCA_000172555.1; Pedosphaera parvula Ellin514; counts= 1; multicellularity status: FALSE**
  - bpX4->MoxR->bpX2->extended_strand+helical_region+vWA+beta_propeller+beta_rich_region+bpX1*->?->bpX3+TPR_region+beta_propeller->
- **GCA_003315205.1; Roseimicrobium gellanilyticum; counts= 1; multicellularity status: TRUE**
  - bpX4->MoxR->bpX2->extended_strand+helical_region+vWA+beta_propeller+beta_rich_region+bpX1*->bpX3+TPR_region+beta_propeller->

- **GCA_003054695.1; Verrucomicrobia bacterium LW23; counts= 1; multicellularity status: FALSE**
  - extended_strand+helical_region+vWA+beta_propeller+beta_rich_region+bpX1*->
- **GCF_000172155.1; Verrucomicrobium spinosum; counts= 1; multicellularity status: FALSE**
  - bpX4->MoxR->bpX2->extended_strand+helical_region+vWA+beta_propeller+beta_rich_region+bpX1*->bpX3+TPR_region+beta_propeller->
- **GCF_000719695.1; Streptomyces sp. NRRL F-6131; counts= 1; multicellularity status: TRUE**
  - MoxR->bpX5->vWA+beta_propeller*->bpX6+TMs+TPRs+Betapropeller->
- **GCA_002797915.1; Yoonia maricola; counts= 1; multicellularity status: FALSE**
  - MoxR->?->vWA+beta_propeller*->
- **GCA_001707535.1; Achromobacter xylosoxidans; counts= 1; multicellularity status: FALSE**
  - MoxR->bpX5->vWA+beta_propeller*->bpX6+TPRs+Betapropeller->
- **GCA_001294205.1; Amantichitinum ursilacus; counts= 1; multicellularity status: FALSE**
  - MoxR->bpX5->vWA+beta_propeller*->bpX6+TPRs+Betapropeller->
- **GCA_900176275.1; Andreprevotia lacus DSM 23236; counts= 1; multicellularity status: FALSE**
  - MoxR->bpX5->vWA+beta_propeller*->bpX6+TMs+TPRs+Betapropeller->
- **GCA_000007705.1; Chromobacterium violaceum ATCC 12472; counts= 1; multicellularity status: FALSE**
  - MoxR->bpX5->vWA+beta_propeller*->
- **GCA_003416915.1; Duganella sp. BJB489; counts= 1; multicellularity status: FALSE**
  - MoxR->bpX5->vWA+beta_propeller*->bpX6+TMs+TPRs+Betapropeller->
- **GCA_001427305.1; Duganella sp. Root1480D1; counts= 1; multicellularity status: FALSE**
  - MoxR->bpX5->vWA+beta_propeller*->bpX6+TMs+TPRs+Betapropeller->
- **GCF_000333495.1; Herbaspirillum sp. B39; counts= 1; multicellularity status: FALSE**
  - MoxR->bpX5->vWA+beta_propeller*->?->bpX6+TPRs+Betapropeller->
- **GCA_900129765.1; Massilia sp. CF038; counts= 1; multicellularity status: FALSE**
  - MoxR->bpX5->vWA+beta_propeller*->bpX6+TMs+TPRs+Betapropeller->
- **GCA_002752675.1; Massilia violaceinigra; counts= 1; multicellularity status: FALSE**
  - MoxR->bpX5->vWA+beta_propeller->*vWA+beta_propeller*->bpX6+TPRs+Betapropeller->
- **GCA_003269145.1; Microvirgula sp. AG722; counts= 1; multicellularity status: FALSE**
  - MoxR->bpX5->vWA+beta_propeller*->bpX6+TPRs+Betapropeller->||<-?<-BetaPropeller<-BetaPropeller
- **GCA_001653795.1; Mitsuaria sp. 7; counts= 1; multicellularity status: FALSE**
  - MoxR->bpX5->vWA+beta_propeller*->bpX6+TMs+TPRs+Betapropeller->

- **GCA_000427195.1; Ralstonia solanacearum CMR15; counts= 1; multicellularity status: FALSE**
  - MoxR->bpX5->vWA+beta_propeller*->bpX6+TMs+TPRs+Betapropeller->
- **GCA_002116905.1; Rhizobacter gummiphilus; counts= 1; multicellularity status: FALSE**
  - MoxR->bpX5->vWA+beta_propeller*->bpX6+TMs+TPRs+Betapropeller->
- **GCA_001425785.1; Rhizobacter sp. Root1221; counts= 1; multicellularity status: FALSE**
  - MoxR->bpX5->vWA+beta_propeller*->bpX6+TPRs+Betapropeller->
- **GCA_003852895.1; Rhizobacter sp. S-16; counts= 1; multicellularity status: FALSE**
  - MoxR->bpX5->vWA+beta_propeller*->bpX6+TMs+TPRs+Betapropeller->
- **GCA_900106655.1; Variovorax sp. YR634; counts= 1; multicellularity status: FALSE**
  - MoxR->bpX5->vWA+beta_propeller*->bpX6+TMs+TPRs+Betapropeller->
- **GCA_000733295.1; Archangium violaceum Cb vi76; counts= 1; multicellularity status: TRUE**
  - bpX5->vWA+beta_propeller*->bpX6+TPRs+Betapropeller->
- **GCA_003600865.1; Corallococcus sp. H22C18031201; counts= 1; multicellularity status: TRUE**
  - MoxR->bpX5->vWA+beta_propeller*->bpX6+TPRs+Betapropeller->
- **GCA_000988565.1; Myxococcus fulvus 124B02; counts= 1; multicellularity status: TRUE**
  - bpX5->vWA+beta_propeller*->
- **GCA_002305895.1; Myxococcus macrosporus DSM 14697; counts= 1; multicellularity status: TRUE**
  - MoxR->bpX5->vWA+beta_propeller*->bpX6+TPRs+Betapropeller->
- **GCA_000012685.1; Myxococcus xanthus DK 1622; counts= 1; multicellularity status: TRUE**
  - MoxR->bpX5->vWA+beta_propeller*->bpX6+TPRs+Betapropeller->
- **GCA_002141575.1; Gilliamella apicola; counts= 1; multicellularity status: FALSE**
  - MoxR->bpX5->vWA+beta_propeller*->bpX6+TMs+TPRs+Betapropeller->
- **GCA_000012985.1; Hahella chejuensis KCTC 2396; counts= 1; multicellularity status: FALSE**
  - MoxR->bpX5->vWA+beta_propeller*->bpX6+TPRs+Betapropeller->
- **GCF_000419525.1; Leucothrix mucor; counts= 1; multicellularity status: TRUE**
  - MoxR->bpX5->vWA+beta_propeller*->bpX6+TPRs+Betapropeller->
- **GCF_000336385.2; Lysobacter antibioticus; counts= 1; multicellularity status: FALSE**
  - MoxR->bpX5->vWA+beta_propeller*->
- **GCA_002355975.1; Lysobacter enzymogenes; counts= 1; multicellularity status: FALSE**
  - MoxR->bpX5->vWA+beta_propeller*->bpX6+TPRs+Betapropeller->

- **GCA_001427505.1; Lysobacter sp. Root604; counts= 1; multicellularity status: FALSE**
  - MoxR->bpX5->vWA+beta_propeller*->bpX6+TPRs+Betapropeller->
- **GCA_001427785.1; Lysobacter sp. Root690; counts= 1; multicellularity status: FALSE**
  - MoxR->bpX5->vWA+beta_propeller*->bpX6+TPRs+Betapropeller->
- **GCA_002206145.2; Proteus mirabilis; counts= 1; multicellularity status: FALSE**
  - RADICAL_SAM.Ammemo->?->MoxR->bpX5->vWA+beta_propeller*->bpX6+TPRs+Betapropeller->
- **GCA_000314895.2; Providencia sneebia DSM 19967; counts= 1; multicellularity status: FALSE**
  - bpX5->vWA+beta_propeller*->
- **GCA_000820515.1; Pseudomonas batumici; counts= 1; multicellularity status: FALSE**
  - MoxR->bpX5->vWA+beta_propeller*->bpX6+TMs+TPRs+Betapropeller->
- **GCF_003415775.1; Pseudomonas cichorii; counts= 1; multicellularity status: FALSE**
  - MoxR->bpX5->vWA+beta_propeller*->bpX6+TMs+TPRs+Betapropeller->
- **GCF_000467005.1; Pseudomonas fuscovaginae; counts= 1; multicellularity status: FALSE**
  - MoxR->PSE->vWA+beta_propeller*->?->TPRs+Betapropeller->
- **GCA_003700875.1; Pseudomonas marginalis pv. marginalis; counts= 1; multicellularity status: FALSE**
  - MoxR->bpX5->vWA+beta_propeller*->bpX6+TMs+TPRs+Betapropeller->
- **GCA_003701285.1; Pseudomonas savastanoi pv. glycinea; counts= 1; multicellularity status: FALSE**
  - vWA+beta_propeller*->
- **GCF_900582885.1; Pseudomonas sp. 286; counts= 1; multicellularity status: FALSE**
  - MoxR->bpX5->vWA+beta_propeller*->bpX6+TMs+TPRs+Betapropeller->
- **GCA_000931385.1; Pseudomonas sp. FeS53a; counts= 1; multicellularity status: FALSE**
  - MoxR->bpX5->vWA+beta_propeller*->bpX6+TMs+TPRs+Betapropeller->
- **GCF_000812405.1; Pseudomonas sp. PI1; counts= 1; multicellularity status: FALSE**
  - MoxR->bpX5->vWA+beta_propeller*->
- **GCA_001547915.1; Pseudomonas sp. St29; counts= 1; multicellularity status: FALSE**
  - MoxR->bpX5->vWA+beta_propeller*->bpX6+TMs+TPRs+Betapropeller->
- **GCA_900215525.1; Pseudomonas sp. URIL14HWK12:I9; counts= 1; multicellularity status: FALSE**
  - MoxR->bpX5->vWA+beta_propeller*->bpX6+TPRs+Betapropeller->
- **GCA_001725155.1; Xanthomonadaceae bacterium SCN 69-320; counts= 1; multicellularity status: FALSE**
  - MoxR->bpX5->vWA+beta_propeller*->bpX6+TPRs+Betapropeller->

■ **GCA_002940525.1; Xanthomonas arboricola; counts= 1; multicellularity status: FALSE**

- MoxR->bpX5->vWA+beta_propeller*->bpX6+TPRs+Betapropeller->

■ **GCA_001043115.1; Xanthomonas sp. NCPPB 1128; counts= 1; multicellularity status: FALSE**

- MoxR->bpX5->vWA+beta_propeller*->bpX6+TPRs+Betapropeller->

■ **GCF_000379645.1; Actinoplanes globisporus; counts= 1; multicellularity status: TRUE**

- ClpN_repeats+vWA->ClpN_repeats+vWA->ClpN_repeats+vWA->*ClpN_repeats+vWA*->ClpN_repeats+vWA->ClpN_repeats+vWA->?->?->EAD1+CASPASE->

■ **GCA_002234595.1; Amycolatopsis sp. H5; counts= 1; multicellularity status: TRUE**

- MoxR->?->X3->TPRs->low_complexity+TPRs->beta_propeller+helical_linker+vWA*->||<-RadSAM_pep<-RADICAL_SAM.Ammemo

■ **GCF_000716715.1; Dactylosporangium aurantiacum; counts= 1; multicellularity status: TRUE**

- MoxR->?->X3->TPRs->low_complexity+TPRs->beta_propeller+helical_linker+vWA*->||<-RadSAM_pep<-RADICAL_SAM.Ammemo

■ **GCA_001017755.1; Frankia coriariae; counts= 1; multicellularity status: TRUE**

- beta_propeller+helical_linker+vWA*->

■ **GCA_900100605.1; Sinosporangium album; counts= 1; multicellularity status: TRUE**

- TPRs->low_complexity+TPRs->beta_propeller+helical_linker+vWA*->||<-RadSAM_pep

■ **GCA_003955715.1; Streptomyces sp. GGCR-6; counts= 1; multicellularity status: TRUE**

- beta_propeller+helical_linker+vWA*->||<-RadSAM_pep<-RADICAL_SAM.Ammemo

■ **GCA_001280015.1; Streptomyces sp. MMG1121; counts= 1; multicellularity status: TRUE**

- X3->TPRs->low_complexity+TPRs->beta_propeller+helical_linker+vWA*->||<-RadSAM_pep<-RADICAL_SAM.Ammemo

■ **GCA_002154695.1; Nostoc sp. T09; counts= 1; multicellularity status: TRUE**

- X3->TPRs+C-type_lectin->||<-?||low_complexity+TPRs->beta_propeller+helical_linker+vWA*->||<-RADICAL_SAM.Ammemo

■ **GCF_000024805.1; Haliangium ochraceum; counts= 1; multicellularity status: TRUE**

- beta_propeller+helical_linker+vWA*->TPRs->

■ **GCF_001469165.1; Thiohalocapsa sp. ML1; counts= 1; multicellularity status: TRUE**

- X3->TPRs->low_complexity+TPRs->||<-?||beta_propeller+helical_linker+vWA*->

■ **GCA_003645185.1; Gammaproteobacteria bacterium; counts= 1; multicellularity status: NA**

- helical_linker+vWA*->

■ **GCF_000262465.1; Frankia sp. QA3; counts= 1; multicellularity status: TRUE**

- vWA*->

■ **GCF_002156075.1; Streptomyces scabiei; counts= 1; multicellularity status: TRUE**

- vWA*->

DOGTPase systems

- **GCA_000175595.1; Clostridium carboxidivorans P7; counts= 3; multicellularity status: FALSE**
  - Fibronectin-like_repeats+??->ZnR+ZnR+DO-GTPase2.2->GAP1-N2+GAP1-M+GAP1-C*->?->TM+TM+TM+TM+ZnR+ZnR+DO-GTPase2.2->TM+vWA(canonical)+YtKA-like+TM+??->Tubulin+coiled_coil+??->Tubulin+coiled_coil+HTH?+??->TM+TM+TM+vWA(canonical)+TM+TM+TM+polar_tail->PSE->ZnR+ZnR+DO-GTPase2.2+ZnR+ZnR->
  - TM+TM+TM+TM+ZnR+ZnR+DO-GTPase2.2->TM+vWA(canonical)+YtKA-like+TM+??->Tubulin+coiled_coil+??->Tubulin+coiled_coil+HTH?+??->TM+TM+TM+vWA(canonical)+TM+TM+TM+polar_tail->Fibronectin-like_repeats+??->ZnR+ZnR+DO-GTPase2.2+ZnR+ZnR->GAP1-N2+GAP1-M+GAP1-C*->GAP1-N2+GAP1-M->
  - TM+vWA(canonical)+YtKA-like+TM+??->Tubulin+coiled_coil+??->Tubulin+coiled_coil+HTH?+??->TM+TM+TM+vWA(canonical)+TM+TM+TM+polar_tail->Fibronectin-like_repeats+??->ZnR+ZnR+DO-GTPase2.2+ZnR+ZnR->GAP1-N2+GAP1-M+GAP1-C->GAP1-N2+GAP1-M*->
- **GCA_000647895.1; Clostridium tetanomorphum DSM 665; counts= 3; multicellularity status: FALSE**
  - Fibronectin-like_repeats+??->ZnR+ZnR+DO-GTPase2.2->GAP1-N2+GAP1-M+GAP1-C*->TM+TM+TM+TM+ZnR+ZnR+DO-GTPase2.2->TM+vWA(canonical)+YtKA-like+TM+??->Tubulin+coiled_coil+??->Tubulin+coiled_coil+HTH?+??->TM+TM+TM+vWA(canonical)+TM+TM+TM+polar_tail->Fibronectin-like_repeats+??->ZnR+ZnR+DO-GTPase2.2+ZnR+ZnR->
  - TM+TM+TM+TM+ZnR+ZnR+DO-GTPase2.2->TM+vWA(canonical)+YtKA-like+TM+??->Tubulin+coiled_coil+??->Tubulin+coiled_coil+HTH?+??->TM+TM+TM+vWA(canonical)+TM+TM+TM+polar_tail->Fibronectin-like_repeats+??->ZnR+ZnR+DO-GTPase2.2+ZnR+ZnR->GAP1-N2+GAP1-M*->
  - ZnR+ZnR+DO-GTPase2.2+ZnR+ZnR->GAP1-N2+GAP1-M*->
- **GCA_001276615.1; Achromatium sp. WMS3; counts= 3; multicellularity status: FALSE**
  - GAP1-N1+GAP1-M+GAP1-C*->
  - GAP1-N2+GAP1-M+GAP1-C*->
  - GAP1-N2+GAP1-M+GAP1-C*->
- **GCA_002737235.1; Opitutae bacterium; counts= 3; multicellularity status: FALSE**
  - FHA-like?+ZnR+ZnR+DO-GTPase2.2->GAP1-N2+GAP1-M+TM+GAP1-C*->
  - FHA-like?+ZnR+ZnR+DO-GTPase2.2->GAP1-N2+GAP1-M+TM+GAP1-C*->
  - FHA-like?+ZnR+ZnR+DO-GTPase2.2->GAP1-N2+GAP1-M+TM+GAP1-C*->
- **GCA_002794505.1; Chloroflexi bacterium; counts= 3; multicellularity status: TRUE**
  - GAP1-N2+GAP1-M+GAP1-C*->
  - GAP1-N2+GAP1-M+GAP1-C*->Pkinase+helical_region+TM_region->
  - GAP1-N2+GAP1-M+TM+GAP1-C*->
- **GCA_900129635.1; Singulisphaera sp. GP187; counts= 3; multicellularity status: TRUE**
  - Calcineurin+GAPEAD1->GAP2->DO-GTPase2.1->DO-GTPase1->GAP1-N1+GAP1-M+GAP1-C*->
  - DO-GTPase2.1+TM->GAP1-N2+GAP1-M+GAP1-C*->
  - GAP1-N2+GAP1-M+GAP1-C*->
- **GCA_000181475.1; Gimesia maris; counts= 3; multicellularity status: FALSE**
  - GAP1-N2+GAP1-M+TM+GAP1-C->ZnR+ZnR+DO-GTPase2.2->GAP1-N2+GAP1-M+TM+GAP1-C*->??+TM+??+beta-rich??->GYF+GYF+TM+TM+TM->
  - GAP1-N2+GAP1-M+TM+GAP1-C*->ZnR+ZnR+DO-GTPase2.2->GAP1-N2+GAP1-M+TM+GAP1-C->??+TM+??+beta-rich??->GYF+GYF+TM+TM+TM->
  - GrpE->HSP70->HSP70+vWA(canonical)->DnaJ+DnaJ+DZnR+Fibronectin-like_repeats->ZnR+ZnR+DO-GTPase2.2->GAP1-N2+GAP1-M+GAP1-C*->?->TM+TM+TM+TM+ZnR+ZnR+DO-GTPase2.2->
- **GCA_000242455.3; Singulisphaera acidiphila; counts= 3; multicellularity status: TRUE**

- FGS-fold_domain->ZnR+DO-GTPase2.1->GAP1-N2+GAP1-M*->
- GAP1-N2+GAP1-M+GAP1-C*->
- GAP1-N2+GAP1-M+GAP1-C*->

■ **GCA_001270375.1; Clostridium sp. DMHC 10; counts= 3; multicellularity status: NA**

- Fibronectin-like_repeats+??->PSE->GAP1-N2+GAP1-M+GAP1-C(frag)->GAP1-N2+GAP1-M*->?->?->TM+TM+TM+TM+ZnR+ZnR+DO-GTPase2.2->TM+vWA(canonical)+YtKA-like+TM+??->PSE->?->Tubulin+coiled_coil+HTH?+??->TM+TM+TM+vWA(canonical)+TM+TM+TM+polar_tail->
- Fibronectin-like_repeats+??->PSE->GAP1-N2+GAP1-M+GAP1-C(frag)*->GAP1-N2+GAP1-M->?->?->TM+TM+TM+TM+ZnR+ZnR+DO-GTPase2.2->TM+vWA(canonical)+YtKA-like+TM+??->PSE->?->Tubulin+coiled_coil+HTH?+??->
- GAP1-N2+GAP1-M+GAP1-C*->

■ **GCF_001570525.1; Herbidospora sakaeratensis; counts= 3; multicellularity status: TRUE**

- GrpE->HSP70->TPR_containing_alpha_helical+Fibronectin-like_repeats->ZnR+ZnR+DO-GTPase2.2->GAP1-N2+GAP1-M+GAP1-C*->TM+TM+TM+TM+ZnR+ZnR+DO-GTPase2.2+TM->
- GrpE->HSP70->TPR_containing_alpha_helical+Fibronectin-like_repeats->ZnR+ZnR+DO-GTPase2.2->GAP1-N2+GAP1-M+TM+GAP1-C*->
- vWA(canonical)->DO-GTPase1->GAP1-N2+GAP1-M+GAP1-C+TM+low_complexity+TM*->

■ **GCA_000025185.1; Pirellula staleyi DSM 6068; counts= 2; multicellularity status: TRUE**

- FGS-fold_domain->ZnR+DO-GTPase2.1->GAP1-N2+GAP1-M*->
- ZnR+ZnR+DO-GTPase2.2->GAP1-N2+GAP1-M+GAP1-C*->??+TM+??+beta-rich??->GYF+GYF+TM+TM+TM->

■ **GCA_000092385.1; Streptomyces bingchenggensis BCW-1; counts= 2; multicellularity status: TRUE**

- GAP1-N2+GAP1-M+GAP1-C*->
- ZnR+ZnR+DO-GTPase2.2->GAP1-N2+GAP1-M+GAP1-C*->TM+TM+TM+TM+ZnR+ZnR+DO-GTPase2.2->

■ **GCA_000968375.1; Clostridium scatologenes; counts= 2; multicellularity status: FALSE**

- Fibronectin-like_repeats+??->ZnR+ZnR+DO-GTPase2.2->GAP1-N2+GAP1-M+GAP1-C*->TM+TM+TM+TM+ZnR+ZnR+DO-GTPase2.2->TM+vWA(canonical)+YtKA-like+TM+??->Tubulin+coiled_coil+??->Tubulin+coiled_coil+HTH?+??->TM+TM+TM+vWA(canonical)+TM+TM+TM+polar_tail->Fibronectin-like_repeats+??->ZnR+ZnR+DO-GTPase2.2+ZnR+ZnR->
- TM+TM+TM+TM+ZnR+ZnR+DO-GTPase2.2->TM+vWA(canonical)+YtKA-like+TM+??->Tubulin+coiled_coil+??->Tubulin+coiled_coil+HTH?+??->TM+TM+TM+vWA(canonical)+TM+TM+TM+polar_tail->Fibronectin-like_repeats+??->ZnR+ZnR+DO-GTPase2.2+ZnR+ZnR->GAP1-N2+GAP1-M*->

■ **GCA_001038625.1; Clostridium carboxidivorans P7; counts= 2; multicellularity status: FALSE**

- Fibronectin-like_repeats+??->ZnR+ZnR+DO-GTPase2.2->GAP1-N2+GAP1-M+GAP1-C*->TM+TM+TM+TM+ZnR+ZnR+DO-GTPase2.2->TM+vWA(canonical)+YtKA-like+TM+??->Tubulin+coiled_coil+??->Tubulin+coiled_coil+HTH?+??->TM+TM+TM+vWA(canonical)+TM+TM+TM+polar_tail->Fibronectin-like_repeats+??->ZnR+ZnR+DO-GTPase2.2+ZnR+ZnR->
- TM+TM+TM+TM+ZnR+ZnR+DO-GTPase2.2->TM+vWA(canonical)+YtKA-like+TM+??->Tubulin+coiled_coil+??->Tubulin+coiled_coil+HTH?+??->TM+TM+TM+vWA(canonical)+TM+TM+TM+polar_tail->PSE->ZnR+ZnR+DO-GTPase2.2+ZnR+ZnR->GAP1-N2+GAP1-M*->

■ **GCA_001610835.1; Planctomyces sp. SH-PL14; counts= 2; multicellularity status: FALSE**

- DO-GTPase2.2->GAP1-N2+GAP1-M+TM+GAP1-C*->??+TM+??+beta-rich??->GYF+GYF+Trypsin->
- ZnR+ZnR+DO-GTPase2.2->GAP1-N2+GAP1-M+TM+GAP1-C*->??+TM+??+beta-rich??->GYF+GYF+Trypsin->Pkinase+low_complexity+TM->

■ **GCA_002189675.1; Streptomyces alboflavus; counts= 2; multicellularity status: TRUE**

- GAP1-N2+GAP1-M+GAP1-C*->vWA(canonical)->PP2C->Pkinase+ZnR+beta-rich->||<-TPR_containing_alpha_helical+Fibronectin-like_repeats
- Pkinase->?->?->?->?->TPR_containing_alpha_helical+Fibronectin-like_repeats->ZnR+ZnR+DO-GTPase2.2->GAP1-N2+GAP1-M+GAP1-C*->?->TM+TM+TM+TM+ZnR+ZnR+DO-GTPase2.2->

■ **GCA_002355675.1; Corynebacterium glutamicum; counts= 2; multicellularity status: FALSE**

- GrpE->HSP70->??+Fibronectin-like_repeats->ZnR+ZnR+DO-GTPase2.2->GAP1-N2+GAP1-M+GAP1-C*->TM+TM+TM+TM+ZnR+ZnR+DO-GTPase2.2->
- polar_leader+TM+TM+TM->TM+vWA(canonical)+Fibronectin-like_repeats+TM+beta-rich+lc_tail->Tubulin+alpha_helical+??+??->GAP_unknown1+TM+TM+GAP_unknown2->HRDC-like?+Fibronectin-like_repeats->GAP1-N2+GAP1-M+GAP1-C*->beta-rich+vWA(canonical)->??+DO-GTPase2.2->

■ **GCA_000239075.2; Streptomyces sp. W007; counts= 2; multicellularity status: TRUE**

- *

- TM+TM+TM+TM+ZnR+ZnR+DO-GTPase2.2+TM->?->vWA(canonical)->PP2C->Pkinase+ZnR+beta-rich->||<-?<-?||GAP1-N2+GAP1-M+GAP1-C*->

■ **GCA_000295855.1; Mycolicibacterium fortuitum subsp. fortuitum DSM 46621 = ATCC 6841; counts= 2; multicellularity status: FALSE**

- HRDC-like?+Fibronectin-like_repeats->GAP1-N2+GAP1-M+GAP1-C*->
- polar_leader+TM+TM+TM->TM+vWA(canonical)+Fibronectin-like_repeats+TM+beta-rich+lc_tail->Tubulin+alpha_helical+??+??->GAP_unknown1+TM+TM+GAP_unknown2->PSE->PSE->HRDC-like?+Fibronectin-like_repeats->PSE->GAP1-N2+GAP1-M+GAP1-C*->beta-rich+vWA(canonical)->??+DO-GTPase2.2->TM+DUF4190+TM+PknH_C->

■ **GCA_000497445.1; Streptomyces roseochromogenus subsp. oscitans DS 12.976; counts= 2; multicellularity status: TRUE**

- GAP1-N2+GAP1-M+GAP1-C*->TM+TM+TM+TM+ZnR+ZnR+DO-GTPase2.2+Jelly_roll+beta-sandwich->||<-STAND_NTPase+TM+TM+TM+TM+TM+TM+TM
- Pkinase->?->?->?->?->HSP70_domain_2+low_complexity+domain_betaX->ZnR+ZnR+DO-GTPase2.2->GAP1-N2+GAP1-M+GAP1-C*->TM+TM+TM+TM+ZnR+ZnR+DO-GTPase2.2->

■ **GCA_000524095.1; Mycobacterium kansasii 824; counts= 2; multicellularity status: FALSE**

- TM+DUF4190+TM->TM+DUF4190+TM->polar_leader+TM+TM+TM->PSE->PSE->GAP_unknown1+TM+TM+GAP_unknown2->GAP_unknown1+TM+TM+GAP_unknown2->PSE->GAP1-N2+GAP1-M+GAP1-C*->GAP1-N2+GAP1-M+GAP1-C->?->beta-rich+vWA(canonical)->
- Tubulin+alpha_helical+??+??->GAP_unknown1+TM+TM+GAP_unknown2->GAP_unknown1+TM+TM+GAP_unknown2->HRDC-like?+Fibronectin-like_repeats->HRDC-like?+Fibronectin-like_repeats->GAP1-N2+GAP1-M+GAP1-C->GAP1-N2+GAP1-M+GAP1-C*->?->beta-rich+vWA(canonical)->??+DO-GTPase2.2->

■ **GCA_000785145.2; Candidatus Thiomargarita nelsonii; counts= 2; multicellularity status: TRUE**

- DO-GTPase1->?->GAP1-N2+GAP1-M+GAP1-C*->DO-GTPase2?->
- ZnR+ZnR+DO-GTPase2.2->GAP1-N2+GAP1-M+GAP1-C*->TM+TM+TM+TM+ZnR+ZnR+DO-GTPase2.2->

■ **GCA_001898385.1; Planctomycetales bacterium 71-10; counts= 2; multicellularity status: NA**

- GAP1-N2+GAP1-M*->
- ZnR+DO-GTPase2.2->GAP1-N2+GAP1-M+GAP1-C*->

■ **GCA_001905595.1; Streptomyces sp. CB00316; counts= 2; multicellularity status: TRUE**

- TM+TM+TM+TM+ZnR+ZnR+DO-GTPase2.2+TM->vWA(canonical)->PP2C->Pkinase+ZnR+beta-rich->||<-?<-?||GAP1-N2+GAP1-M+GAP1-C*->
- TPR_containing_alpha_helical+Fibronectin-like_repeats->ZnR+ZnR+DO-GTPase2.2->GAP1-N2+GAP1-M+GAP1-C*->TM+TM+TM+TM+ZnR+ZnR+DO-GTPase2.2+TM->vWA(canonical)->PP2C->Pkinase+ZnR+beta-rich->

■ **GCA_001874765.1; Acinetobacter baumannii; counts= 2; multicellularity status: FALSE**

- GAP2->GAP2->ZnR+DO-GTPase2.1->DO-GTPase1->DO-GTPase1->GAP1-N1+GAP1-M+GAP1-C*->EAD1->
- PNP_UDP_1+EAD1->GAP2->ZnR+DO-GTPase2.1->DO-GTPase1->GAP1-N1+GAP1-M+GAP1-C*->EAD1->

■ **GCA_001983595.1; Streptomyces sp. IB2014 011-1; counts= 2; multicellularity status: TRUE**

- TPR_containing_alpha_helical+Fibronectin-like_repeats->ZnR+ZnR+DO-GTPase2.2->GAP1-N2+GAP1-M+GAP1-C*->TM+TM+TM+TM+ZnR+ZnR+DO-GTPase2.2+TM->vWA(canonical)->PP2C->Pkinase+ZnR+beta-rich->
- vWA(canonical)->PP2C->Pkinase+ZnR+beta-rich->||<-?<-?<-?||GAP1-N2+GAP1-M+GAP1-C*->

■ **GCA_002003625.1; Mycobacterium kansasii; counts= 2; multicellularity status: FALSE**

- GAP_unknown1+TM+TM+GAP_unknown2->HRDC-like?+Fibronectin-like_repeats->GAP1-N2+GAP1-M+GAP1-C*->GAP1-M->GAP1-N2+GAP1-M+GAP1-C->beta-rich+vWA(canonical)->??+DO-GTPase2.2->
- Tubulin+alpha_helical+??+??->Tubulin+alpha_helical+??+??->GAP_unknown1+TM+TM+GAP_unknown2->HRDC-like?+Fibronectin-like_repeats->GAP1-N2+GAP1-M+GAP1-C->GAP1-M*->GAP1-N2+GAP1-M+GAP1-C->beta-rich+vWA(canonical)->??+DO-GTPase2.2->

■ **GCA_002167455.1; Rhodopirellula; counts= 2; multicellularity status: TRUE**

- FGS-fold_domain->ZnR+DO-GTPase2.1->GAP1-N2+GAP1-M*->
- ZnR+ZnR+DO-GTPase2.2->GAP1-N2+GAP1-M+TM+GAP1-C*->??+TM+??+beta-rich??->GYF+GYF+TM+TM+TM->

■ **GCA_002279765.1; Verrucomicrobia bacterium 12-59-8; counts= 2; multicellularity status: FALSE**

- FHA-like?+ZnR+ZnR+DO-GTPase2.2->GAP1-N2+GAP1-M+TM*->

- FHA-like?+ZnR+ZnR+DO-GTPase2.2->GAP1-N2+GAP1-M+TM+GAP1-C*->

■ **GCA_002401775.1; Ectothiorhodospiraceae bacterium; counts= 2; multicellularity status: TRUE**

- GAP2->ZnR+DO-GTPase2.1->DO-GTPase1->GAP1-N1+GAP1-M+GAP1-C+CASPASE*->
- GAP2->ZnR+DO-GTPase2.1->DO-GTPase1->GAP1-N1+GAP1-M+GAP1-C+CASPASE*->

■ **GCA_002794585.1; Chloroflexi bacterium; counts= 2; multicellularity status: TRUE**

- GAP1-N2+GAP1-M*->
- GAP1-N2+GAP1-M+GAP1-C*->

■ **GCA_003002825.1; Spirosoma oryzae; counts= 2; multicellularity status: TRUE**

- GAP2->ZnR+DO-GTPase2.1->DO-GTPase1->GAP1-N1+GAP1-M+GAP1-C+EAD1*->PNP_UDP_1+EAD1->
- ZnR+DO-GTPase2.1->DO-GTPase1->GAP1-N1+GAP1-M+GAP1-C+EAD1*->PNP_UDP_1+EAD1->

■ **GCA_900091795.1; Streptomyces sp. Termitarium-T10T-6; counts= 2; multicellularity status: TRUE**

- GAP1-N2+GAP1-M+GAP1-C->GAP1-N2+GAP1-M+GAP1-C*->
- GAP1-N2+GAP1-M+GAP1-C*->GAP1-N2+GAP1-M+GAP1-C->

■ **GCA_900111115.1; Giesbergeria anulus; counts= 2; multicellularity status: FALSE**

- Calcineurin+GAPEAD1->GAP2->ZnR+DO-GTPase2.1->DO-GTPase1->GAP1-N1+GAP1-M+GAP1-C*->
- GAP2->ZnR+DO-GTPase2.1->DO-GTPase1->GAP1-N1+GAP1-M+GAP1-C+EAD1*->CASPASE+EAD1->

■ **GCA_000416365.2; Mycobacteriaceae; counts= 2; multicellularity status: FALSE**

- HRDC-like?+Fibronectin-like_repeats->GAP1-N2+GAP1-M+GAP1-C*->??+DO-GTPase2.2->
- Tubulin+alpha_helical+??+??->GAP_unknown1+TM+TM+GAP_unknown2->HRDC-like?+Fibronectin-like_repeats->GAP1-N2+GAP1-M+GAP1-C*->beta-rich+vWA(canonical)->??+DO-GTPase2.2->TM+DUF4190+TM+PknH_C->TM+DUF4190+TM+PknH_C->

■ **GCA_000062885.1; Saccharopolyspora erythraea; counts= 2; multicellularity status: TRUE**

- GAP1-N2+GAP1-M+GAP1-C*->
- GrpE->HSP70->TPR_containing_alpha_helical+Fibronectin-like_repeats->ZnR+ZnR+DO-GTPase2.2->GAP1-N2+GAP1-M+GAP1-C*->TM+TM+TM+TM+ZnR+ZnR+DO-GTPase2.2->||<-?<-?<-HSP70<-unknown+CASPASE

■ **GCA_003149495.1; Gemmata obscuriglobus; counts= 2; multicellularity status: FALSE**

- DO-GTPase2.1->?->GAP1-N2+GAP1-M+TM+low_complexity+TM*->
- GAP1-N2+GAP1-M+TM*->

■ **GCA_000010265.1; Clostridium kluyveri; counts= 2; multicellularity status: FALSE**

- Fibronectin-like_repeats+??->ZnR+ZnR+DO-GTPase2.2->GAP1-N2+GAP1-M+GAP1-C*->TM+TM+TM+TM+ZnR+ZnR+DO-GTPase2.2->TM+vWA(canonical)+YtKA-like+TM+??->Tubulin+coiled_coil+??->Tubulin+coiled_coil+HTH?+??->Fibronectin-like_repeats+??->ZnR+ZnR+DO-GTPase2.2+ZnR+ZnR->GAP1-N2+GAP1-M->
- GAP1-N2+GAP1-M+GAP1-C->TM+TM+TM+TM+ZnR+ZnR+DO-GTPase2.2->TM+vWA(canonical)+YtKA-like+TM+??->Tubulin+coiled_coil+??->Tubulin+coiled_coil+HTH?+??->Fibronectin-like_repeats+??->ZnR+ZnR+DO-GTPase2.2+ZnR+ZnR->GAP1-N2+GAP1-M*->

■ **GCA_000024385.1; Thermomonospora curvata; counts= 2; multicellularity status: TRUE**

- GrpE->HSP70->TPR_containing_alpha_helical+Fibronectin-like_repeats->ZnR+ZnR+DO-GTPase2.2->GAP1-N2+GAP1-M+GAP1-C*->TM+TM+TM+TM+ZnR+ZnR+DO-GTPase2.2->
- GrpE->HSP70->TPR_containing_alpha_helical+Fibronectin-like_repeats->ZnR+ZnR+DO-GTPase2.2->GAP1-N2+GAP1-M+GAP1-C*->TM+TM+TM+TM+ZnR+ZnR+DO-GTPase2.2+TM->

■ **GCA_000143685.1; Clostridium ljungdahlii; counts= 2; multicellularity status: FALSE**

- Fibronectin-like_repeats+??->ZnR+ZnR+DO-GTPase2.2->GAP1-N2+GAP1-M+GAP1-C*->TM+vWA(canonical)+YtKA-like+TM+??->Tubulin+coiled_coil+??->Tubulin+coiled_coil+HTH?+??->TM+TM+TM+vWA(canonical)+TM+TM+TM+polar_tail->Fibronectin-like_repeats+??->

- Tubulin+coiled_coil+HTH?+??->TM+TM+TM+vWA(canonical)+TM+TM+TM+polar_tail->Fibronectin-like_repeats+??->?->?->?->ZnR+ZnR+DO-GTPase2.2+ZnR+ZnR->GAP1-N2+GAP1-M*->

■ **GCA_000484505.1; Clostridium autoethanogenum; counts= 2; multicellularity status: NA**

- Fibronectin-like_repeats+??->ZnR+ZnR+DO-GTPase2.2->GAP1-N2+GAP1-M+GAP1-C*->TM+vWA(canonical)+YtKA-like+TM+??->Tubulin+coiled_coil+??->PSE->TM+TM+TM+vWA(canonical)+TM+TM+TM+polar_tail->Fibronectin-like_repeats+??->
- Tubulin+coiled_coil+??->PSE->TM+TM+TM+vWA(canonical)+TM+TM+TM+polar_tail->Fibronectin-like_repeats+??->?->?->?->ZnR+ZnR+DO-GTPase2.2+ZnR+ZnR->GAP1-N2+GAP1-M*->

■ **GCF_000619945.1; Clostridium lundense; counts= 2; multicellularity status: FALSE**

- Fibronectin-like_repeats+??->ZnR+ZnR+DO-GTPase2.2->GAP1-N2+GAP1-M+GAP1-C*->TM+TM+TM+TM+ZnR+ZnR+DO-GTPase2.2->TM+vWA(canonical)+YtKA-like+TM+??->Tubulin+coiled_coil+??->Tubulin+coiled_coil+HTH?+??->TM+TM+TM+vWA(canonical)+TM+TM+TM+polar_tail->Fibronectin-like_repeats+??->ZnR+ZnR+DO-GTPase2.2+ZnR+ZnR->
- TM+TM+TM+TM+ZnR+ZnR+DO-GTPase2.2->TM+vWA(canonical)+YtKA-like+TM+??->Tubulin+coiled_coil+??->Tubulin+coiled_coil+HTH?+??->TM+TM+TM+vWA(canonical)+TM+TM+TM+polar_tail->Fibronectin-like_repeats+??->ZnR+ZnR+DO-GTPase2.2+ZnR+ZnR->GAP1-N2+GAP1-M*->

■ **GCF_000686125.1; Ruminococcus sp. NK3A76; counts= 2; multicellularity status: TRUE**

- GrpE->HSP70->HSP70+vWA(canonical)->?->ZnR+ZnR+DO-GTPase2.2->GAP1-N2+GAP1-M+GAP1-C*->
- HSP70->?->HSP70(frag)+vWA(canonical)->?->ZnR+ZnR+Fibronectin-like_repeats->ZnR+ZnR+DO-GTPase2.2->?->GAP1-N2+GAP1-M+GAP1-C*->

■ **GCF_000716675.1; Streptomyces; counts= 2; multicellularity status: TRUE**

- GAP1-N2+GAP1-M+GAP1-C*->
- Pkinase->?->?->?->HSP70_domain_2+low_complexity+domain_betaX->ZnR+ZnR+DO-GTPase2.2->GAP1-N2+GAP1-M+GAP1-C*->TM+TM+TM+TM+ZnR+ZnR+DO-GTPase2.2->

■ **GCF_000721705.1; Actinoplanes subtropicus; counts= 2; multicellularity status: TRUE**

- <-TPR_region+CASPASE||GrpE->HSP70->TPR_containing_alpha_helical+Fibronectin-like_repeats->ZnR+ZnR+DO-GTPase2.2->GAP1-N2+GAP1-M+GAP1-C*->TM+TM+TM+TM+ZnR+ZnR+DO-GTPase2.2->
- ZnR+ZnR+DO-GTPase2.2->GAP1-N2+GAP1-M+TM+GAP1-C*->

■ **GCF_000719065.1; Herbidospora cretacea; counts= 2; multicellularity status: TRUE**

- GrpE->HSP70->TPR_containing_alpha_helical+Fibronectin-like_repeats->ZnR+ZnR+DO-GTPase2.2->GAP1-N2+GAP1-M+GAP1-C*->TM+TM+TM+TM+ZnR+ZnR+DO-GTPase2.2+TM->
- vWA(canonical)->DO-GTPase1->GAP1-N2+GAP1-M+GAP1-C+TM+low_complexity+TM*->

■ **GCF_000974445.1; Lechevalieria aerocolonigenes; counts= 2; multicellularity status: TRUE**

- TM+vWA(canonical)+TM->HSP70->??+Fibronectin-like_repeats->ZnR+ZnR+DO-GTPase2.2->GAP1-N2+GAP1-M+GAP1-C*->TM+TM+TM+TM+ZnR+ZnR+DO-GTPase2.2->||<-GrpE
- vWA(canonical)+beta-rich+low_complexity->Tubulin+alpha_helical+??+??->??+TM+TM+TM+TM+??->Pkinase+TM->ZnR+ZnR+DO-GTPase2.2->GAP1-N2+GAP1-M+SLATT+SLATT*->

■ **GCF_000718135.1; Streptomyces cyaneofuscatus; counts= 2; multicellularity status: TRUE**

- TM+TM+TM+TM+ZnR+ZnR+DO-GTPase2.2+TM->vWA(canonical)->PP2C->Pkinase+ZnR+beta-rich->||<-?<-?<-?||GAP1-N2+GAP1-M+GAP1-C*->
- TPR_containing_alpha_helical+Fibronectin-like_repeats->ZnR+ZnR+DO-GTPase2.2->GAP1-N2+GAP1-M+GAP1-C*->TM+TM+TM+TM+ZnR+ZnR+DO-GTPase2.2+TM->vWA(canonical)->PP2C->Pkinase+ZnR+beta-rich->

■ **GCF_000718165.1; Streptomyces fulvoviolaceus; counts= 2; multicellularity status: TRUE**

- DO-GTPase1->GAP1-N2+GAP1-M+TM+GAP1-C*->?->TM+vWA(canonical)+TM+low_complexity->
- TPR_containing_alpha_helical+Fibronectin-like_repeats->ZnR+ZnR+DO-GTPase2.2->GAP1-N2+GAP1-M+GAP1-C*->TM+TM+TM+TM+ZnR+ZnR+DO-GTPase2.2+TM->vWA(canonical)->PP2C->Pkinase+ZnR+beta-rich->

■ **GCA_001278095.1; Streptomyces sp. CFMR 7; counts= 2; multicellularity status: TRUE**

- TM+TM+TM+TM+ZnR+ZnR+DO-GTPase2.2+TM->vWA(canonical)->PP2C->Pkinase+ZnR+beta-rich->||<-?<-?<-?||GAP1-N2+GAP1-M+GAP1-C*->
- TPR_containing_alpha_helical+Fibronectin-like_repeats->ZnR+ZnR+DO-GTPase2.2->GAP1-N2+GAP1-M+GAP1-C*->TM+TM+TM+TM+ZnR+ZnR+DO-GTPase2.2+TM->vWA(canonical)->PP2C->Pkinase+ZnR+beta-rich->

■ **GCA_001278845.1; Actinomyces sp. oral taxon 414; counts= 2; multicellularity status: NA**

- GrpE->HSP70->??+Fibronectin->ZnR+ZnR+DO-GTPase2.2->GAP1-N2+GAP1-M+GAP1-C*->

- polar__leader+TM+TM+TM->TM+vWA(canonical)+Fibronectin-like__repeats+TM+beta-rich+lc__tail->Tubulin+alpha__helical+??+??->GAP__unknown1+TM+TM+GAP__unknown2->HRDC-like?+Fibronectin-like__repeats->GAP1-N2+GAP1-M+GAP1-C*->?->??+DO-GTPase2.2->

■ **GCA__001279525.1; Nocardia sp. NRRL S-836; counts= 2; multicellularity status: TRUE**

- TM+vWA(canonical)+TM->HSP70->??+Fibronectin-like__repeats->ZnR+ZnR+DO-GTPase2.2->GAP1-N2+GAP1-M+GAP1-C*->TM+TM+TM+TM+ZnR+ZnR+DO-GTPase2.2->||<-GrpE
- vWA(canonical)+beta-rich+low__complexity->?->?->Tubulin+alpha__helical+??+??->??+TM+TM+TM+TM+??->Pkinase+TM->ZnR+ZnR+DO-GTPase2.2->GAP1-N2+GAP1-M+GAP1-C+TM+beta-rich*->?->||<-?||Pkinase+TM+Septum__form->

■ **GCA__001307545.1; Mycolicibacterium fortuitum; counts= 2; multicellularity status: FALSE**

- HRDC-like?+Fibronectin-like__repeats->GAP1-N2+GAP1-M+GAP1-C*->??+DO-GTPase2.2->
- polar__leader+TM+TM+TM->TM+vWA(canonical)+Fibronectin-like__repeats+TM+beta-rich+lc__tail->Tubulin+alpha__helical+??+??->GAP__unknown1+TM+TM+GAP__unknown2->HRDC-like?+Fibronectin-like__repeats->GAP1-N2+GAP1-M+GAP1-C*->beta-rich+vWA(canonical)->??+DO-GTPase2.2->TM+DUF4190+TM+PknH__C->TM+DUF4190+TM+PknH__C->

■ **GCA__001317355.1; Oxobacter pfennigii; counts= 2; multicellularity status: FALSE**

- GAP1-N2+GAP1-M+GAP1-C*->
- Tubulin+coiled__coil+??->Tubulin+coiled__coil+HTH?+??->TM+TM+TM+vWA(canonical)+TM+TM+TM+polar__tail->Fibronectin-like__repeats+??->ZnR+ZnR+DO-GTPase2.2+ZnR+ZnR->GAP1-N2+GAP1-M+GAP1-C+polar__tail+TM+beta-rich*->

■ **GCF__001368795.1; Paenibacillus ihuae; counts= 2; multicellularity status: TRUE**

- TM+vWA(canonical)+YtKA-like+TM+??->PSE->PSE->Tubulin+coiled__coil+HTH?+??->TM+TM+TM+vWA(canonical)+TM+TM+TM+polar__tail->Fibronectin-like__repeats+??->ZnR+ZnR+DO-GTPase2.2+ZnR+ZnR->GAP1-N2+GAP1-M->GAP1-N2+GAP1-M+GAP1-C+polar__tail+TM+beta-rich*->
- TM+vWA(canonical)+YtKA-like+TM+??->PSE->PSE->Tubulin+coiled__coil+HTH?+??->TM+TM+TM+vWA(canonical)+TM+TM+TM+polar__tail->Fibronectin-like__repeats+??->ZnR+ZnR+DO-GTPase2.2+ZnR+ZnR->GAP1-N2+GAP1-M*->GAP1-N2+GAP1-M+GAP1-C+polar__tail+TM+beta-rich->

■ **GCA__002102345.1; Mycolicibacterium peregrinum; counts= 2; multicellularity status: FALSE**

- HRDC-like?+Fibronectin-like__repeats->GAP1-N2+GAP1-M+GAP1-C*->??+DO-GTPase2.2->
- polar__leader+TM+TM+TM->TM+vWA(canonical)+Fibronectin-like__repeats+TM+beta-rich+lc__tail->Tubulin+alpha__helical+??+??->GAP__unknown1+TM+TM+GAP__unknown2->HRDC-like?+Fibronectin-like__repeats->GAP1-N2+GAP1-M+GAP1-C*->beta-rich+vWA(canonical)->??+DO-GTPase2.2->TM+DUF4190+TM+PknH__C->TM+DUF4190+TM+PknH__C->

■ **GCA__001570465.1; Mycolicibacterium fortuitum; counts= 2; multicellularity status: FALSE**

- HRDC-like?+Fibronectin-like__repeats->GAP1-N2+GAP1-M+GAP1-C*->??+DO-GTPase2.2->
- polar__leader+TM+TM+TM->TM+vWA(canonical)+Fibronectin-like__repeats+TM+beta-rich+lc__tail->Tubulin+alpha__helical+??+??->GAP__unknown1+TM+TM+GAP__unknown2->HRDC-like?+Fibronectin-like__repeats->GAP1-N2+GAP1-M+GAP1-C*->beta-rich+vWA(canonical)->??+DO-GTPase2.2->TM+DUF4190+TM+PknH__C->TM+DUF4190+TM+PknH__C->

■ **GCF__001570605.1; Herbidospora cretacea; counts= 2; multicellularity status: TRUE**

- GrpE->HSP70->TPR__containing__alpha__helical+Fibronectin-like__repeats->ZnR+ZnR+DO-GTPase2.2->GAP1-N2+GAP1-M+GAP1-C*->TM+TM+TM+TM+ZnR+ZnR+DO-GTPase2.2+TM->
- vWA(canonical)->DO-GTPase1->GAP1-N2+GAP1-M+GAP1-C+TM+low__complexity+TM*->

■ **GCF__001570565.1; Herbidospora yilanensis; counts= 2; multicellularity status: TRUE**

- GrpE->HSP70->TPR__containing__alpha__helical+Fibronectin-like__repeats->ZnR+ZnR+DO-GTPase2.2->GAP1-N2+GAP1-M+GAP1-C*->TM+TM+TM+TM+ZnR+ZnR+DO-GTPase2.2+TM->
- vWA(canonical)->DO-GTPase1->GAP1-N2+GAP1-M+GAP1-C+TM+low__complexity+TM*->

■ **GCF__001570585.1; Herbidospora daliensis; counts= 2; multicellularity status: TRUE**

- GrpE->HSP70->TPR__containing__alpha__helical+Fibronectin-like__repeats->ZnR+ZnR+DO-GTPase2.2->GAP1-N2+GAP1-M+GAP1-C*->TM+TM+TM+TM+ZnR+ZnR+DO-GTPase2.2+TM->
- vWA(canonical)->DO-GTPase1->GAP1-N2+GAP1-M+GAP1-C+TM+low__complexity+TM*->

■ **GCA__001636845.1; Clostridium ljungdahlii; counts= 2; multicellularity status: FALSE**

- Fibronectin-like__repeats+??->PSE->?->ZnR+ZnR+DO-GTPase2.2->GAP1-N2+GAP1-M+GAP1-C*->TM+vWA(canonical)+YtKA-like+TM+??->Tubulin+coiled__coil+??->Tubulin+coiled__coil+HTH?+??->TM+TM+TM+vWA(canonical)+TM+TM+TM+polar__tail->Fibronectin-like__repeats+??->
- TM+TM+TM+vWA(canonical)+TM+TM+TM+polar__tail->Fibronectin-like__repeats+??->?->?->?->?->ZnR+ZnR+DO-GTPase2.2+ZnR+ZnR->GAP1-N2+GAP1-M*->

■ **GCA__001636835.1; Clostridium coskatii; counts= 2; multicellularity status: FALSE**

- Fibronectin-like__repeats+??->ZnR+ZnR+DO-GTPase2.2->GAP1-N2+GAP1-M+GAP1-C*->TM+vWA(canonical)+YtKA-like+TM+??->Tubulin+coiled__coil+??->Tubulin+coiled__coil+HTH?+??->TM+TM+TM+vWA(canonical)+TM+TM+TM+polar__tail->Fibronectin-like__repeats+??->

- TM+TM+TM+vWA(canonical)+TM+TM+TM+polar_tail->Fibronectin-like_repeats+??->?->?->?->?->ZnR+ZnR+DO-GTPase2.2+ZnR+ZnR->GAP1-N2+GAP1-M*->

■ **GCA_001665455.1; Mycolicibacterium fortuitum; counts= 2; multicellularity status: FALSE**

- HRDC-like?+Fibronectin-like_repeats->GAP1-N2+GAP1-M+GAP1-C*->??+DO-GTPase2.2->
- polar_leader+TM+TM+TM->TM+vWA(canonical)+Fibronectin-like_repeats+TM+beta-rich+lc_tail->Tubulin+alpha_helical+??+??->GAP_unknown1+TM+TM+GAP_unknown2->HRDC-like?+Fibronectin-like_repeats->GAP1-N2+GAP1-M+GAP1-C*->beta-rich+vWA(canonical)->??+DO-GTPase2.2->TM+DUF4190+TM+PknH_C->TM+DUF4190+TM+PknH_C->

■ **GCA_001665415.1; Mycolicibacterium fortuitum; counts= 2; multicellularity status: FALSE**

- HRDC-like?+Fibronectin-like_repeats->GAP1-N2+GAP1-M+GAP1-C*->??+DO-GTPase2.2->
- polar_leader+TM+TM+TM->TM+vWA(canonical)+Fibronectin-like_repeats+TM+beta-rich+lc_tail->Tubulin+alpha_helical+??+??->GAP_unknown1+TM+TM+GAP_unknown2->HRDC-like?+Fibronectin-like_repeats->GAP1-N2+GAP1-M+GAP1-C*->beta-rich+vWA(canonical)->??+DO-GTPase2.2->TM+DUF4190+TM+PknH_C->

■ **GCA_001665625.1; Mycolicibacterium peregrinum; counts= 2; multicellularity status: FALSE**

- HRDC-like?+Fibronectin-like_repeats->GAP1-N2+GAP1-M+GAP1-C*->??+DO-GTPase2.2->
- TM+vWA(canonical)+Fibronectin-like_repeats+TM+beta-rich+lc_tail->Tubulin+alpha_helical+??+??->GAP_unknown1+TM+TM+GAP_unknown2->HRDC-like?+Fibronectin-like_repeats->GAP1-N2+GAP1-M+GAP1-C*->beta-rich+vWA(canonical)->??+DO-GTPase2.2->TM+DUF4190+TM+PknH_C->

■ **GCA_001665315.1; Mycolicibacterium fortuitum; counts= 2; multicellularity status: FALSE**

- HRDC-like?+Fibronectin-like_repeats->GAP1-N2+GAP1-M+GAP1-C*->??+DO-GTPase2.2->
- polar_leader+TM+TM+TM->TM+vWA(canonical)+Fibronectin-like_repeats+TM+beta-rich+lc_tail->Tubulin+alpha_helical+??+??->GAP_unknown1+TM+TM+GAP_unknown2->HRDC-like?+Fibronectin-like_repeats->GAP1-N2+GAP1-M+GAP1-C*->beta-rich+vWA(canonical)->??+DO-GTPase2.2->TM+DUF4190+TM+PknH_C->

■ **GCA_001665785.1; Mycolicibacterium peregrinum; counts= 2; multicellularity status: FALSE**

- HRDC-like?+Fibronectin-like_repeats->GAP1-N2+GAP1-M+GAP1-C*->??+DO-GTPase2.2->
- TM+[lc_insert+]vWA(canonical)+Fibronectin-like_repeats+TM+??+lc_tail->Tubulin+alpha_helical+??+??->GAP_unknown1+TM+TM+GAP_unknown2->HRDC-like?+Fibronectin-like_repeats->GAP1-N2+GAP1-M+GAP1-C*->beta-rich+vWA(canonical)->??+DO-GTPase2.2->TM+DUF4190+TM+PknH_C->

■ **GCA_001667205.1; Mycolicibacterium peregrinum; counts= 2; multicellularity status: FALSE**

- HRDC-like?+Fibronectin-like_repeats->GAP1-N2+GAP1-M+GAP1-C*->??+DO-GTPase2.2->
- polar_leader+TM+TM+TM->TM+vWA(canonical)+Fibronectin-like_repeats+TM+beta-rich+lc_tail->Tubulin+alpha_helical+??+??->GAP_unknown1+TM+TM+GAP_unknown2->HRDC-like?+Fibronectin-like_repeats->GAP1-N2+GAP1-M+GAP1-C*->beta-rich+vWA(canonical)->??+DO-GTPase2.2->TM+DUF4190+TM+PknH_C->TM+DUF4190+TM+PknH_C->

■ **GCA_001673325.1; Mycolicibacterium fortuitum; counts= 2; multicellularity status: FALSE**

- HRDC-like?+Fibronectin-like_repeats->GAP1-N2+GAP1-M+GAP1-C*->??+DO-GTPase2.2->
- polar_leader+TM+TM+TM->TM+vWA(canonical)+Fibronectin-like_repeats+TM+beta-rich+lc_tail->Tubulin+alpha_helical+??+??->GAP_unknown1+TM+TM+GAP_unknown2->HRDC-like?+Fibronectin-like_repeats->GAP1-N2+GAP1-M+GAP1-C*->beta-rich+vWA(canonical)->??+DO-GTPase2.2->TM+DUF4190+TM+PknH_C->TM+DUF4190+TM+PknH_C->

■ **GCA_001673265.1; Mycolicibacterium conceptionense; counts= 2; multicellularity status: FALSE**

- GrpE->HSP70->TPR_containing_alpha_helical+Fibronectin-like_repeats->ZnR+ZnR+DO-GTPase2.2->GAP1-N2+GAP1-M+GAP1-C*->TM+TM+TM+TM+ZnR+ZnR+DO-GTPase2.2->HSP70->vWA(canonical)->
- HRDC-like?+Fibronectin-like_repeats->GAP1-N2+GAP1-M+GAP1-C*->??+DO-GTPase2.2->

■ **GCA_001672845.1; Mycolicibacterium fortuitum; counts= 2; multicellularity status: FALSE**

- HRDC-like?+Fibronectin-like_repeats->GAP1-N2+GAP1-M+GAP1-C*->??+DO-GTPase2.2->
- polar_leader+TM+TM+TM->TM+vWA(canonical)+Fibronectin-like_repeats+TM+beta-rich+lc_tail->Tubulin+alpha_helical+??+??->GAP_unknown1+TM+TM+GAP_unknown2->HRDC-like?+Fibronectin-like_repeats->GAP1-N2+GAP1-M+GAP1-C*->beta-rich+vWA(canonical)->??+DO-GTPase2.2->TM+DUF4190+TM+PknH_C->TM+DUF4190+TM+PknH_C->

■ **GCA_001673275.1; Mycolicibacterium fortuitum; counts= 2; multicellularity status: FALSE**

- HRDC-like?+Fibronectin-like_repeats->GAP1-N2+GAP1-M+GAP1-C*->??+DO-GTPase2.2->
- polar_leader+TM+TM+TM->TM+vWA(canonical)+Fibronectin-like_repeats+TM+beta-rich+lc_tail->Tubulin+alpha_helical+??+??->GAP_unknown1+TM+TM+GAP_unknown2->HRDC-like?+Fibronectin-like_repeats->GAP1-N2+GAP1-M+GAP1-C*->beta-rich+vWA(canonical)->??+DO-GTPase2.2->TM+DUF4190+TM+PknH_C->

■ **GCA_001675165.1; Clostridium ragsdalei; counts= 2; multicellularity status: FALSE**

- Fibronectin-like_repeats+??->ZnR+ZnR+DO-GTPase2.2->GAP1-N2+GAP1-M+GAP1-C*->TM+vWA(canonical)+YtKA-like+TM+??->Tubulin+coiled_coil+??->Tubulin+coiled_coil+HTH?+??->TM+TM+TM+vWA(canonical)+TM+TM+TM+polar_tail->Fibronectin-like_repeats+??->

- TM+TM+TM+vWA(canonical)+TM+TM+TM+polar_tail->Fibronectin-like_repeats+??->?->?->?->?->ZnR+ZnR+DO-GTPase2.2+ZnR+ZnR->GAP1-N2+GAP1-M*->

■ **GCA_001673485.1; Mycolicibacterium fortuitum; counts= 2; multicellularity status: FALSE**

- GAP_unknown1+TM+TM+GAP_unknown2->HRDC-like?+Fibronectin-like_repeats->GAP1-N2+GAP1-M+GAP1-C*->beta-rich+vWA(canonical)->??+DO-GTPase2.2->TM+DUF4190+TM+PknH_C->
- HRDC-like?+Fibronectin-like_repeats->GAP1-N2+GAP1-M+GAP1-C*->??+DO-GTPase2.2->

■ **GCF_001570545.1; Herbidospora mongoliensis; counts= 2; multicellularity status: TRUE**

- GrpE->HSP70->TPR_containing_alpha_helical+Fibronectin-like_repeats->ZnR+ZnR+DO-GTPase2.2->GAP1-N2+GAP1-M+GAP1-C*->TM+TM+TM+TM+ZnR+ZnR+DO-GTPase2.2+TM->
- vWA(canonical)->DO-GTPase1->GAP1-N2+GAP1-M+GAP1-C+TM+low_complexity+TM*->

■ **GCA_001666875.1; Mycobacterium sp. E802; counts= 2; multicellularity status: FALSE**

- GrpE->HSP70->TPR_containing_alpha_helical+Fibronectin-like_repeats->ZnR+ZnR+DO-GTPase2.2->GAP1-N2+GAP1-M+GAP1-C*->TM+TM+TM+TM+ZnR+ZnR+DO-GTPase2.2->HSP70->vWA(canonical)->
- HRDC-like?+Fibronectin-like_repeats->GAP1-N2+GAP1-M+GAP1-C*->??+DO-GTPase2.2->

■ **GCF_001552135.1; Actinomadura chibensis; counts= 2; multicellularity status: TRUE**

- GrpE->HSP70->TPR_containing_alpha_helical+Fibronectin-like_repeats->ZnR+ZnR+DO-GTPase2.2->GAP1-N2+GAP1-M+GAP1-C*->TM+TM+TM+TM+ZnR+ZnR+DO-GTPase2.2+TM->
- vWA(canonical)->DO-GTPase1->GAP1-N2+GAP1-M+GAP1-C+TM*->

■ **GCA_003001795.1; Actinomadura; counts= 2; multicellularity status: TRUE**

- GrpE->HSP70->TPR_containing_alpha_helical+Fibronectin-like_repeats->ZnR+ZnR+DO-GTPase2.2->GAP1-N2+GAP1-M+GAP1-C*->TM+TM+TM+TM+ZnR+ZnR+DO-GTPase2.2->?->unknown+CASPASE->HSP70->
- HSP70->TPR_containing_alpha_helical+Fibronectin-like_repeats->ZnR+ZnR+DO-GTPase2.2->GAP1-N2+GAP1-M+GAP1-C*->TM+TM+TM+TM+ZnR+ZnR+DO-GTPase2.2+TM->

■ **GCA_002300155.1; Rhodococcus; counts= 2; multicellularity status: TRUE**

- polar_leader+TM+TM+TM->TM+vWA(canonical)+Fibronectin-like_repeats+TM+beta-rich+lc_tail->Tubulin+alpha_helical+??+??->GAP_unknown1+TM+TM+GAP_unknown2->HRDC-like?+Fibronectin-like_repeats->GAP1-N2+GAP1-M+GAP1-C*->beta-rich+vWA(canonical)->??+DO-GTPase2.2->
- TM+vWA(canonical)+Fibronectin-like_repeats+TM+beta-rich+lc_tail->Tubulin+alpha_helical+??+??->GAP_unknown1+TM+TM+GAP_unknown2->HRDC-like?+Fibronectin-like_repeats->GAP1-N2+GAP1-M+GAP1-C*->beta-rich+vWA(canonical)->??+DO-GTPase2.2->

■ **GCA_001905575.1; Streptomyces sp. CB00072; counts= 2; multicellularity status: TRUE**

- GAP1-N2+GAP1-M+GAP1-C->TM+TM+TM+TM+ZnR+ZnR+DO-GTPase2.2+TM->vWA(canonical)->PP2C->Pkinase+ZnR+beta-rich->||<-?<-?||GAP1-N2+GAP1-M+GAP1-C*->
- TPR_containing_alpha_helical+Fibronectin-like_repeats->ZnR+ZnR+DO-GTPase2.2->GAP1-N2+GAP1-M+GAP1-C*->TM+TM+TM+TM+ZnR+ZnR+DO-GTPase2.2+TM->vWA(canonical)->PP2C->Pkinase+ZnR+beta-rich->||<-?<-?||GAP1-N2+GAP1-M+GAP1-C->

■ **GCA_900129675.1; Bradyrhizobium erythrophlei; counts= 2; multicellularity status: TRUE**

- Calcineurin+GAPEAD1->GAP2->DO-GTPase2.1->DO-GTPase1->GAP1-N1+GAP1-M+GAP1-C*->
- GAP1-N1+GAP1-M+GAP1-C*->

■ **GCF_001610835.1; Planctomyces sp. SH-PL14; counts= 2; multicellularity status: FALSE**

- DO-GTPase2.2->GAP1-N2+GAP1-M+TM+GAP1-C*->??+TM+??+beta-rich??->GYF+GYF+Trypsin->
- ZnR+ZnR+DO-GTPase2.2->GAP1-N2+GAP1-M+TM+GAP1-C*->??+TM+??+beta-rich??->GYF+GYF+Trypsin->Pkinase+low_complexity+TM->

■ **GCA_001954035.1; Mycolicibacterium fortuitum; counts= 2; multicellularity status: FALSE**

- HRDC-like?+Fibronectin-like_repeats->GAP1-N2+GAP1-M+GAP1-C*->??+DO-GTPase2.2->
- polar_leader+TM+TM+TM->TM+vWA(canonical)+Fibronectin-like_repeats+TM+beta-rich+lc_tail->Tubulin+alpha_helical+??+??->GAP_unknown1+TM+TM+GAP_unknown2->HRDC-like?+Fibronectin-like_repeats->GAP1-N2+GAP1-M+GAP1-C*->beta-rich+vWA(canonical)->??+DO-GTPase2.2->

■ **GCA_001956985.1; Paludisphaera borealis; counts= 2; multicellularity status: TRUE**

- GAP1-N2+GAP1-M*->

- ZnR+ZnR+DO-GTPase2.2->GAP1-N2+GAP1-M+GAP1-C*->??+TM+??+beta-rich??->

■ **GCA_002197845.1; Fimbriiglobus ruber; counts= 2; multicellularity status: TRUE**

- GAP1-N2+GAP1-M+TM+GAP1-C*->
- GAP1-N2+GAP1-M+TM+low_complexity+TM*->

■ **GCA_002224125.1; Streptomyces sp. NBS 14/10; counts= 2; multicellularity status: TRUE**

- GAP1-N2+GAP1-M+GAP1-C*->
- PP2C->Pkinase->?->?->?->HSP70_domain_2+low_complexity+domain_betaX->ZnR+ZnR+DO-GTPase2.2->GAP1-N2+GAP1-M+GAP1-C*->TM+TM+TM+TM+ZnR+ZnR+DO-GTPase2.2->

■ **GCA_900091865.1; Streptomyces sp. DvalAA-19; counts= 2; multicellularity status: TRUE**

- GAP1-N2+GAP1-M+GAP1-C->||<-PSE||vWA(canonical)->PP2C->Pkinase+ZnR+beta-rich->PSE->||<-?<-?<-?||GAP1-N2+GAP1-M+GAP1-C*->
- TPR_containing_alpha_helical+Fibronectin-like_repeats->ZnR+ZnR+DO-GTPase2.2->GAP1-N2+GAP1-M+GAP1-C*->||<-PSE||vWA(canonical)->PP2C->Pkinase+ZnR+beta-rich->PSE->||<-?<-?<-?||GAP1-N2+GAP1-M+GAP1-C->

■ **GCA_002251265.1; Rhodopirellula; counts= 2; multicellularity status: TRUE**

- FGS-fold_domain->ZnR+DO-GTPase2.1->GAP1-N2+GAP1-M*->
- ZnR+ZnR+DO-GTPase2.2->GAP1-N2+GAP1-M+TM+GAP1-C*->??+TM+??+beta-rich??->GYF+GYF+TM+TM+TM->

■ **GCA_002289565.1; Rhodopirellula sp. SM50; counts= 2; multicellularity status: TRUE**

- FGS-fold_domain->ZnR+DO-GTPase2.1->GAP1-N2+GAP1-M*->
- ZnR+ZnR+DO-GTPase2.2->GAP1-N2+GAP1-M+TM+GAP1-C*->??+TM+??+beta-rich??->GYF+GYF+TM+TM+TM->

■ **GCF_002551465.1; Streptomyces sp. b84; counts= 2; multicellularity status: TRUE**

- GAP1-N2+GAP1-M+GAP1-C->TM+TM+TM+TM+ZnR+ZnR+DO-GTPase2.2+TM->vWA(canonical)->PP2C->Pkinase+ZnR+beta-rich->||<-?<-?||GAP1-N2+GAP1-M+GAP1-C*->
- TPR_containing_alpha_helical+Fibronectin-like_repeats->ZnR+ZnR+DO-GTPase2.2->GAP1-N2+GAP1-M+GAP1-C*->TM+TM+TM+TM+ZnR+ZnR+DO-GTPase2.2+TM->vWA(canonical)->PP2C->Pkinase+ZnR+beta-rich->||<-?<-?||GAP1-N2+GAP1-M+GAP1-C->

■ **GCA_000025345.1; Geodermatophilus obscurus DSM 43160; counts= 1; multicellularity status: TRUE**

- Pkinase+TM->TM->ZnR+ZnR+DO-GTPase2.2->GAP1-N2+GAP1-M+GAP1-C*->?->||<-?<-?||?->Pkinase+low_complexity->

■ **GCA_000190575.1; Bacteroides salanitronis DSM 18170; counts= 1; multicellularity status: FALSE**

- Calcineurin+GAPEAD1->?->DO-GTPase2.1->DO-GTPase1->GAP1-N1+GAP1-M+GAP1-C*->

■ **GCA_000221965.1; Xanthomonas campestris pv. raphani 756C; counts= 1; multicellularity status: FALSE**

- GAP2->DO-GTPase1->GAP1-N1+GAP1-M+GAP1-C+EAD1*->

■ **GCA_000283295.1; Mycolicibacterium smegmatis MC2 155; counts= 1; multicellularity status: FALSE**

- HRDC-like?+Fibronectin-like_repeats->GAP1-N2+GAP1-M+GAP1-C*->??+DO-GTPase2.2->

■ **GCA_002603685.1; Leptospira phage vb_LkmZ_Bejolso9-LE1; counts= 1; multicellularity status: FALSE**

- GAP2->ZnR+DO-GTPase2.1->DO-GTPase1->GAP1-N1+GAP1-M+GAP1-C+EAD1*->PNP_UDP_1+EAD1->

■ **GCA_000739375.1; Comamonas testosteroni TK102; counts= 1; multicellularity status: FALSE**

- GAP2->DO-GTPase1->GAP1-N1+GAP1-M+GAP1-C+EAD1*->CASPASE+EAD1->

■ **GCA_000828015.1; Corynebacterium glutamicum; counts= 1; multicellularity status: FALSE**

- polar_leader+TM+TM+TM->TM+vWA(canonical)+Fibronectin-like_repeats+TM+beta-rich+lc_tail->Tubulin+alpha_helical+??+??->GAP_unknown1+TM+TM+GAP_unknown2->HRDC-like?+Fibronectin-like_repeats->GAP1-N2+GAP1-M+GAP1-C*->beta-rich+vWA(canonical)->??+DO-GTPase2.2->

■ **GCA_000833575.1; Corynebacterium singulare; counts= 1; multicellularity status: FALSE**

- polar_leader+TM+TM+TM->TM+vWA(canonical)+Fibronectin-like_repeats+TM+beta-rich+lc_tail->Tubulin+alpha_helical+??+??->GAP_unknown1+TM+TM+GAP_unknown2->HRDC-like?+Fibronectin-like_repeats->GAP1-N2+GAP1-M+GAP1-C*->beta-rich+vWA(canonical)->??+DO-GTPase2.2->polar_leader+TM+TM+TM->

■ **GCA_000959345.1; Burkholderia pseudomallei PB08298010; counts= 1; multicellularity status: FALSE**

- Calcineurin+GAPEAD1->GAP2->ZnR+DO-GTPase2.1->DO-GTPase1->GAP1-N1+GAP1-M+GAP1-C*->

■ **GCA_001040905.1; Streptomyces sp. PBH53; counts= 1; multicellularity status: TRUE**

- TPR_containing_alpha_helical+Fibronectin-like_repeats->ZnR+ZnR+DO-GTPase2.2->GAP1-N2+GAP1-M+GAP1-C*->TM+TM+TM+TM+ZnR+ZnR+DO-GTPase2.2+TM->vWA(canonical)->Pkinase+ZnR+beta-rich->

■ **GCA_001447315.1; Paenibacillus sp. IHB B 3084; counts= 1; multicellularity status: TRUE**

- Tubulin+coiled_coil+??->Tubulin+coiled_coil+HTH?+??->TM+TM+TM+vWA(canonical)+TM+TM+TM+polar_tail->Fibronectin-like_repeats+??->ZnR+ZnR+DO-GTPase2.2+ZnR+ZnR->GAP1-N2+GAP1-M+GAP1-C+polar_tail+TM+beta-rich*->

■ **GCA_001447865.2; Corynebacterium glutamicum; counts= 1; multicellularity status: FALSE**

- polar_leader+TM+TM+TM->TM+vWA(canonical)+Fibronectin-like_repeats+TM+beta-rich+lc_tail->Tubulin+alpha_helical+??+??->GAP_unknown1+TM+TM+GAP_unknown2->HRDC-like?+Fibronectin-like_repeats->GAP1-N2+GAP1-M+GAP1-C*->beta-rich+vWA(canonical)->??+DO-GTPase2.2->

■ **GCA_001518935.2; Corynebacterium glutamicum; counts= 1; multicellularity status: FALSE**

- polar_leader+TM+TM+TM->TM+vWA(canonical)+Fibronectin-like_repeats+TM+beta-rich+lc_tail->Tubulin+alpha_helical+??+??->GAP_unknown1+TM+TM+GAP_unknown2->HRDC-like?+Fibronectin-like_repeats->GAP1-N2+GAP1-M+GAP1-C*->beta-rich+vWA(canonical)->??+DO-GTPase2.2->

■ **GCA_001610875.1; Pirellula sp. SH-Sr6A; counts= 1; multicellularity status: TRUE**

- GAP2->ZnR+DO-GTPase2.1->DO-GTPase1->GAP1-N1+GAP1-M+GAP1-C+EAD1*->||<-EAD1+Trypsin

■ **GCA_001643015.1; Corynebacterium crudilactis; counts= 1; multicellularity status: FALSE**

- polar_leader+TM+TM+TM->TM+vWA(canonical)+Fibronectin-like_repeats+TM+beta-rich+lc_tail->Tubulin+alpha_helical+??+??->GAP_unknown1+TM+TM+GAP_unknown2->HRDC-like?+Fibronectin-like_repeats->GAP1-N2+GAP1-M+GAP1-C*->beta-rich+vWA(canonical)->

■ **GCA_001865855.1; Nocardia seriolae; counts= 1; multicellularity status: TRUE**

- Pkinase+TM->?->ZnR+DO-GTPase2.1->GAP1-N2+GAP1-M+GAP1-C*->

■ **GCA_001874405.2; Paenibacillus polymyxa; counts= 1; multicellularity status: TRUE**

- TM+vWA(canonical)+YtKA-like+TM+??->Tubulin+coiled_coil+??->Tubulin+coiled_coil+HTH?+??->TM+TM+TM+vWA(canonical)+TM+TM+TM+polar_tail->Fibronectin-like_repeats+??->ZnR+ZnR+DO-GTPase2.2+ZnR+ZnR->GAP1-N2+GAP1-M+GAP1-C+polar_tail+TM+beta-rich*->

■ **GCA_001874425.2; Paenibacillus polymyxa; counts= 1; multicellularity status: TRUE**

- TM+vWA(canonical)+YtKA-like+TM+??->PSE->Tubulin+coiled_coil+HTH?+??->TM+TM+TM+vWA(canonical)+TM+TM+TM+polar_tail->Fibronectin-like_repeats+??->ZnR+ZnR+DO-GTPase2.2+ZnR+ZnR->GAP1-N2+GAP1-M+GAP1-C+polar_tail+TM+beta-rich*->

■ **GCA_001931635.1; Streptomyces sp. Tue 6075; counts= 1; multicellularity status: TRUE**

- GAP1-N2+GAP1-M+GAP1-C*->

■ **GCA_001936195.1; Corynebacterium glutamicum; counts= 1; multicellularity status: FALSE**

- polar_leader+TM+TM+TM->TM+vWA(canonical)+Fibronectin-like_repeats+TM+beta-rich+lc_tail->Tubulin+alpha_helical+??+??->GAP_unknown1+TM+TM+GAP_unknown2->HRDC-like?+Fibronectin-like_repeats->GAP1-N2+GAP1-M+GAP1-C*->beta-rich+vWA(canonical)->??+DO-GTPase2.2->

■ **GCA_002094995.1; Streptomyces sp. S8; counts= 1; multicellularity status: TRUE**

- GAP1-N2+GAP1-M+GAP1-C*->

■ **GCA_002220305.1; Burkholderia sp. AD24; counts= 1; multicellularity status: FALSE**

- Calcineurin+GAPEAD1->GAP2->ZnR+DO-GTPase2.1->DO-GTPase1->GAP1-N1+GAP1-M+GAP1-C*->

■ **GCA_003431825.1; Blastomonas fulva; counts= 1; multicellularity status: FALSE**

- Calcineurin+GAPEAD1->GAP2->ZnR+DO-GTPase2.1->DO-GTPase1->GAP1-N1+GAP1-M+GAP1-C*->

■ **GCA_002814075.1; Polaribacter sejongensis; counts= 1; multicellularity status: TRUE**

- GAP2->ZnR+DO-GTPase2.1->DO-GTPase1->GAP1-N1+GAP1-M+GAP1-C+PNP_UDP_1*->

■ **GCA_002943525.1; Kitasatospora sp. MMS16-BH015; counts= 1; multicellularity status: TRUE**

- PP2C->Pkinase->?->?->?->HSP70_domain_2+low_complexity+domain_betaX->ZnR+ZnR+DO-GTPase2.2->GAP1-N2+GAP1-M+GAP1-C*->TM+TM+TM+TM+ZnR+ZnR+DO-GTPase2.2->

■ **GCA_000011325.1; Corynebacterium glutamicum ATCC 13032; counts= 1; multicellularity status: FALSE**

- polar_leader+TM+TM+TM->TM+vWA(canonical)+Fibronectin-like_repeats+TM+beta-rich+lc_tail->Tubulin+alpha_helical+??+??->GAP_unknown1+TM+TM+GAP_unknown2->HRDC-like?+Fibronectin-like_repeats->GAP1-N2+GAP1-M+GAP1-C*->beta-rich+vWA(canonical)->??+DO-GTPase2.2->

■ **GCA_000010225.1; Corynebacterium glutamicum R; counts= 1; multicellularity status: FALSE**

- polar_leader+TM+TM+TM->TM+vWA(canonical)+Fibronectin-like_repeats+TM+beta-rich+lc_tail->Tubulin+alpha_helical+??+??->GAP_unknown1+TM+TM+GAP_unknown2->HRDC-like?+Fibronectin-like_repeats->GAP1-N2+GAP1-M+GAP1-C*->beta-rich+vWA(canonical)->??+DO-GTPase2.2->

■ **GCA_000010605.1; Streptomyces griseus subsp. griseus NBRC 13350; counts= 1; multicellularity status: TRUE**

- GAP1-N2+GAP1-M+GAP1-C*->

■ **GCA_000010805.1; Rhodococcus opacus B4; counts= 1; multicellularity status: TRUE**

- polar_leader+TM+TM+TM->TM+vWA(canonical)+Fibronectin-like_repeats+TM+beta-rich+lc_tail->Tubulin+alpha_helical+??+??->GAP_unknown1+TM+TM+GAP_unknown2->HRDC-like?+Fibronectin-like_repeats->GAP1-N2+GAP1-M+GAP1-C*->beta-rich+vWA(canonical)->??+DO-GTPase2.2->

■ **GCA_002355155.1; Corynebacterium glutamicum; counts= 1; multicellularity status: FALSE**

- polar_leader+TM+TM+TM->TM+vWA(canonical)+Fibronectin-like_repeats+TM+beta-rich+lc_tail->Tubulin+alpha_helical+??+??->GAP_unknown1+TM+TM+GAP_unknown2->HRDC-like?+Fibronectin-like_repeats->GAP1-N2+GAP1-M+GAP1-C*->beta-rich+vWA(canonical)->??+DO-GTPase2.2->

■ **GCA_002355915.1; Actinomyces naeslundii; counts= 1; multicellularity status: TRUE**

- HRDC-like?+Fibronectin-like_repeats->GAP1-N2+GAP1-M+GAP1-C*->

■ **GCA_003966365.1; Acetobacter orientalis; counts= 1; multicellularity status: FALSE**

- AB_hydrolase+GAPEAD1->GAP2->ZnR+DO-GTPase2.1->DO-GTPase1->GAP1-N1+GAP1-M+GAP1-C*->

■ **GCA_003609795.1; Sphingobium sp. YG1; counts= 1; multicellularity status: FALSE**

- Calcineurin+GAPEAD1->GAP2->ZnR+DO-GTPase2.1->DO-GTPase1->GAP1-N1+GAP1-M+GAP1-C*->

■ **GCA_000196335.1; Corynebacterium glutamicum ATCC 13032; counts= 1; multicellularity status: FALSE**

- polar_leader+TM+TM+TM->TM+vWA(canonical)+Fibronectin-like_repeats+TM+beta-rich+lc_tail->Tubulin+alpha_helical+??+??->GAP_unknown1+TM+TM+GAP_unknown2->HRDC-like?+Fibronectin-like_repeats->GAP1-N2+GAP1-M+GAP1-C*->beta-rich+vWA(canonical)->??+DO-GTPase2.2->

■ **GCA_001046875.1; Tetrasphaera jenkinsii Ben 74; counts= 1; multicellularity status: TRUE**

- polar_leader+TM+TM+TM->TM+vWA(canonical)+Fibronectin-like_repeats+TM+beta-rich+lc_tail->Tubulin+alpha_helical+??+??->GAP_unknown1+TM+TM+GAP_unknown2->HRDC-like?+Fibronectin-like_repeats->GAP1-N2+GAP1-M+GAP1-C*->?->??+DO-GTPase2.2->

■ **GCA_000431615.1; Firmicutes bacterium CAG:41; counts= 1; multicellularity status: FALSE**

- GAP1-N2+GAP1-M+TM+GAP1-C*->

■ **GCA_000433415.1; Clostridium sp. CAG:448; counts= 1; multicellularity status: NA**

- GAP1-N2+GAP1-M+TM+GAP1-C*->

■ **GCA_000437175.1; Ruminococcus sp. CAG:330; counts= 1; multicellularity status: TRUE**

- HSP70(frag)+vWA(canonical)->?->DO-GTPase2.1->GAP1-N2+GAP1-M+TM+GAP1-C*->

■ **GCA_000438015.1; Coraliomargarita sp. CAG:312; counts= 1; multicellularity status: FALSE**

- FHA-like?+ZnR+ZnR+DO-GTPase2.2->GAP1-N2+GAP1-M+TM+GAP1-C*->

■ **GCA_000982975.1; Halomonas sp. A3H3; counts= 1; multicellularity status: FALSE**

- GAP2->ZnR+DO-GTPase2.1->DO-GTPase1->GAP1-N1+GAP1-M+GAP1-C+EAD1*->CASPASE+EAD1->

■ **GCA_001160225.1; Mycobacterium tuberculosis; counts= 1; multicellularity status: FALSE**

- TPR_containing_alpha_helical+Fibronectin-like_repeats->ZnR+ZnR+DO-GTPase2.2->GAP1-N2+GAP1-M+GAP1-C*->TM+TM+TM+TM+ZnR+ZnR+DO-GTPase2.2+TM->

■ **GCA_001152565.1; Yersinia pekkanenii; counts= 1; multicellularity status: FALSE**

- Calcineurin+GAPEAD1->GAP2->DO-GTPase2.1->DO-GTPase1->GAP1-N1+GAP1-M+GAP1-C*->

■ **GCA_001261935.1; Corynebacterium striatum; counts= 1; multicellularity status: FALSE**

- polar_leader+TM+TM+TM->TM+vWA(canonical)+Fibronectin-like_repeats+TM+beta-rich+lc_tail->Tubulin+alpha_helical+??+??->GAP_unknown1+TM+TM+GAP_unknown2->HRDC-like?+Fibronectin-like_repeats->GAP1-N2+GAP1-M+GAP1-C*->beta-rich+vWA(canonical)->??+DO-GTPase2.2->polar_leader+TM+TM+TM->

■ **GCA_001319925.1; Yersinia intermedia; counts= 1; multicellularity status: FALSE**

- Calcineurin+GAPEAD1->GAP2->ZnR+DO-GTPase2.1->DO-GTPase1->GAP1-N1+GAP1-M+GAP1-C*->

■ **GCA_001405915.1; Prevotella copri; counts= 1; multicellularity status: FALSE**

- DO-GTPase1->GAP1-N1+GAP1-M+GAP1-C*->

■ **GCA_000016505.1; Clostridium kluyveri DSM 555; counts= 1; multicellularity status: FALSE**

- TM+TM+TM+TM+ZnR+ZnR+DO-GTPase2.2->TM+vWA(canonical)+YtKA-like+TM+??->Tubulin+coiled_coil+??->Tubulin+coiled_coil+HTH?+??->Fibronectin-like_repeats+??->ZnR+ZnR+DO-GTPase2.2+ZnR+ZnR->GAP1-N2+GAP1-M*->

■ **GCA_000173655.1; Corynebacterium amycolatum SK46; counts= 1; multicellularity status: FALSE**

- polar_leader+TM+TM+TM->TM+vWA(canonical)+Fibronectin-like_repeats+TM+beta-rich+lc_tail->Tubulin+alpha_helical+??+??->GAP_unknown1+TM+TM+GAP_unknown2->HRDC-like?+Fibronectin-like_repeats->GAP1-N2+GAP1-M+GAP1-C*->beta-rich+vWA(canonical)->??+DO-GTPase2.2->

■ **GCA_000167995.1; Yersinia mollaretii ATCC 43969; counts= 1; multicellularity status: FALSE**

- DO-GTPase2.1(fragment)->DO-GTPase1->GAP1-N1+GAP1-M+GAP1-C*->

■ **GCA_000156475.1; Streptomyces albus J1074; counts= 1; multicellularity status: TRUE**

- GAP1-N2+GAP1-M+GAP1-C*->

■ **GCA_000158855.1; Streptomyces sp. SPB78; counts= 1; multicellularity status: TRUE**

- GAP1-N2+GAP1-M+GAP1-C*->

■ **GCA_000158895.1; Streptomyces sp. C; counts= 1; multicellularity status: TRUE**

- GAP1-N2+GAP1-M+GAP1-C*->

■ **GCA_000158915.1; Streptomyces himastatinicus ATCC 53653; counts= 1; multicellularity status: TRUE**

- TPR_containing_alpha_helical+Fibronectin-like_repeats->ZnR+ZnR+DO-GTPase2.2->GAP1-N2+GAP1-M+GAP1-C*->TM+TM+TM+TM+ZnR+ZnR+DO-GTPase2.2+TM->vWA(canonical)->PP2C->Pkinase+ZnR+beta-rich->

■ **GCA_000208635.2; Gluconacetobacter sp. SXCC-1; counts= 1; multicellularity status: FALSE**

- Calcineurin+GAPEAD1->GAP2->ZnR+DO-GTPase2.1->DO-GTPase1->GAP1-N1+GAP1-M+GAP1-C*->

■ **GCA_000224315.2; Corynebacterium glutamicum S9114; counts= 1; multicellularity status: FALSE**

- TM+TM+TM->TM+vWA(canonical)+Fibronectin-like_repeats+TM+beta-rich+lc_tail->Tubulin+alpha_helical+??+??->GAP_unknown1+TM+TM+GAP_unknown2->HRDC-like?+Fibronectin-like_repeats->GAP1-N2+GAP1-M+GAP1-C*->beta-rich+vWA(canonical)->??+DO-GTPase2.2->

■ **GCA_000245555.2; Paenibacillus dendritiformis C454; counts= 1; multicellularity status: TRUE**

- TM+vWA(canonical)+YtKA-like+TM+??->Tubulin+coiled_coil+??->Tubulin+coiled_coil+HTH?+??->TM+TM+TM+vWA(canonical)+TM+TM+TM+polar_tail->Fibronectin-like_repeats+??->ZnR+ZnR+DO-GTPase2.2+ZnR+ZnR->GAP1-N2+GAP1-M+GAP1-C+polar_tail+TM+beta-rich*->

■ **GCA_000271305.1; Hydrocarboniphaga effusa AP103; counts= 1; multicellularity status: FALSE**

- Calcineurin+GAPEAD1->DO-GTPase1->GAP1-N1+GAP1-M+GAP1-C*->

■ **GCA_000278565.1; Pseudomonas sp. Ag1; counts= 1; multicellularity status: FALSE**

- Calcineurin+GAPEAD1->GAP2->DO-GTPase1->GAP1-N1+GAP1-M+GAP1-C*->

■ **GCA_000243895.3; Leptospira interrogans str. FPW2026; counts= 1; multicellularity status: FALSE**

- GAP2->ZnR+DO-GTPase2.1->DO-GTPase1->GAP1-N1+GAP1-M+GAP1-C*->

■ **GCA_000244275.3; Leptospira interrogans str. UI 12621; counts= 1; multicellularity status: FALSE**

- Calcineurin+GAPEAD1->GAP2->ZnR+DO-GTPase2.1->DO-GTPase1->GAP1-N1+GAP1-M+GAP1-C*->

■ **GCA_000244135.3; Leptospira interrogans serovar Grippotyphosa str. UI 08368; counts= 1; multicellularity status: FALSE**

- GAP1-N1+GAP1-M+GAP1-C*->

■ **GCA_000344825.1; Salmonella enterica subsp. enterica serovar Newport str. SH111077; counts= 1; multicellularity status: FALSE**

- Calcineurin+GAPEAD1->GAP2->ZnR+DO-GTPase2.1->DO-GTPase1->GAP1-N1+GAP1-M+GAP1-C*->

■ **GCA_000364445.1; Methylobacterium mesophilicum SR1.6/6; counts= 1; multicellularity status: TRUE**

- CASPASE+GAPEAD1->GAP2->ZnR+DO-GTPase2.1->DO-GTPase1->GAP1-N1+GAP1-M+GAP1-C*->

■ **GCA_000368445.1; Acinetobacter sp. CIP 56.2; counts= 1; multicellularity status: FALSE**

- GAP2->ZnR+DO-GTPase2.1->DO-GTPase1->GAP1-N1+GAP1-M+GAP1-C*->EAD1->CASPASE+EAD1->

■ **GCA_000414115.1; Streptomyces aurantiacus JA 4570; counts= 1; multicellularity status: TRUE**

- GAP1-N2+GAP1-M+GAP1-C*->vWA(canonical)->

■ **GCA_001856845.1; Thermoplasmatales archaeon Gpl; counts= 1; multicellularity status: FALSE**

- TM+TM+TM+TM+TM+TM+DO-GTPase2.1->GAP1-N2+GAP1-M+GAP1-C*->??->TM_region+DO-GTPase1->

■ **GCA_000448385.1; Saccharopolyspora erythraea D; counts= 1; multicellularity status: TRUE**

- ZnR+ZnR+DO-GTPase2.2->GAP1-N2+GAP1-M+GAP1-C*->

■ **GCA_000481145.1; Pseudomonas aeruginosa BWHPSA028; counts= 1; multicellularity status: FALSE**

- Calcineurin+GAPEAD1->GAP2->ZnR+DO-GTPase2.1->DO-GTPase1->GAP1-N1+GAP1-M+GAP1-C*->

■ **GCA_000502855.1; Mesorhizobium sp. L2C067A000; counts= 1; multicellularity status: TRUE**

- Calcineurin+GAPEAD1->GAP2->ZnR+DO-GTPase2.1->DO-GTPase1->GAP1-N1+GAP1-M+GAP1-C*->

■ **GCA_000508425.1; Actinomyces urogenitalis DORA_12; counts= 1; multicellularity status: TRUE**

- GAP1-N2+GAP1-M+GAP1-C(frag)*->

■ **GCA_000516475.1; Candidatus Magnetoglobus multicellularis str. Araruama; counts= 1; multicellularity status: TRUE**

- GrpE->?->?->GrpE->DO-GTPase2.1->GAP1-N1+GAP1-M+GAP1-C*->

■ **GCA_000517865.1; Paenibacillus sp. FSL R7-269; counts= 1; multicellularity status: TRUE**

- TM+vWA(canonical)+YtKA-like+TM+??->Tubulin+coiled_coil+??->Tubulin+coiled_coil+HTH?+??->?->TM+TM+TM+vWA(canonical)+TM+TM+TM+polar_tail->Fibronectin-like_repeats+??->ZnR+ZnR+DO-GTPase2.2+ZnR+ZnR->GAP1-N2+GAP1-M+GAP1-C+polar_tail+TM+beta-rich*->

■ **GCA_000517905.1; Paenibacillus sp. FSL H7-689; counts= 1; multicellularity status: TRUE**

- TM+vWA(canonical)+YtKA-like+TM+??->Tubulin+coiled_coil+??->Tubulin+coiled_coil+HTH?+??->TM+TM+TM+vWA(canonical)+TM+TM+TM+polar_tail->Fibronectin-like_repeats+??->ZnR+ZnR+DO-GTPase2.2+ZnR+ZnR->GAP1-N2+GAP1-M+GAP1-C+polar_tail+TM+beta-rich*->

■ **GCA_000517965.1; Paenibacillus sp. FSL R7-277; counts= 1; multicellularity status: TRUE**

- Tubulin+coiled_coil+??->Tubulin+coiled_coil+HTH?+??->?->?->TM+TM+TM+vWA(canonical)+TM+TM+TM+polar_tail->Fibronectin-like_repeats+??->ZnR+ZnR+DO-GTPase2.2+ZnR+ZnR->GAP1-N2+GAP1-M+GAP1-C+polar_tail+TM+beta-rich*->

■ **GCA_000568155.1; Enterobacter sp. DC3; counts= 1; multicellularity status: FALSE**

- Calcineurin+GAPEAD1->GAP2->DO-GTPase2.1->DO-GTPase1->GAP1-N1+GAP1-M+GAP1-C*->

■ **GCA_000349585.1; Dietzia sp. UCD-THP; counts= 1; multicellularity status: FALSE**

- GAP1-N2+GAP1-M+GAP1-C*->

■ **GCA_000739515.1; Candidatus Moduliflexus flocculans; counts= 1; multicellularity status: TRUE**

- DO-GTPase2.1->GAP1-N2+GAP1-M+low_complexity+TM+TM*->

■ **GCA_000974725.1; Methylobacterium sp. ME121; counts= 1; multicellularity status: TRUE**

- Calcineurin+GAPEAD1->GAP2->ZnR+DO-GTPase2.1->DO-GTPase1->GAP1-N1+GAP1-M+GAP1-C*->

■ **GCA_001570725.1; Paenibacillus amylolyticus; counts= 1; multicellularity status: TRUE**

- TM+vWA(canonical)+YtKA-like+TM+??->Tubulin+coiled_coil+??->Tubulin+coiled_coil+HTH?+??->TM+TM+TM+vWA(canonical)+TM+TM+TM+polar_tail->Fibronectin-like_repeats+??->ZnR+ZnR+DO-GTPase2.2+ZnR+ZnR->GAP1-N2+GAP1-M+GAP1-C+polar_tail+TM+beta-rich*->

■ **GCA_001894745.1; Paenibacillus sp. NAIST15-1; counts= 1; multicellularity status: TRUE**

- TM+vWA(canonical)+YtKA-like+TM+??->Tubulin+coiled_coil+??->Tubulin+coiled_coil+HTH?+??->TM+TM+TM+vWA(canonical)+TM+TM+TM+polar_tail->Fibronectin-like_repeats+??->ZnR+ZnR+DO-GTPase2.2+ZnR+ZnR->GAP1-N2+GAP1-M+GAP1-C+polar_tail+TM+beta-rich*->

■ **GCA_002003445.1; Corynebacterium glutamicum; counts= 1; multicellularity status: FALSE**

- polar_leader+TM+TM+TM->TM+vWA(canonical)+Fibronectin-like_repeats+TM+beta-rich+lc_tail->Tubulin+alpha_helical+??+??->GAP_unknown1+TM+TM+GAP_unknown2->HRDC-like?+Fibronectin-like_repeats->GAP1-N2+GAP1-M+GAP1-C*->beta-rich+vWA(canonical)->??+DO-GTPase2.2->

■ **GCA_000698985.1; Bordetella bronchiseptica MBORD731; counts= 1; multicellularity status: FALSE**

- DO-GTPase2.1(fragment)->DO-GTPase1->GAP1-N1+GAP1-M+GAP1-C*->

■ **GCA_000694595.1; Klebsiella variicola; counts= 1; multicellularity status: FALSE**

- Calcineurin+GAPEAD1->GAP2->DO-GTPase2.1->DO-GTPase1->GAP1-N1+GAP1-M+GAP1-C*->

■ **GCA_000698455.1; Rhodococcus qingshengii; counts= 1; multicellularity status: TRUE**

- GrpE->TPR_containing_alpha_helical+Fibronectin-like_repeats->ZnR+ZnR+DO-GTPase2.2->?->GAP1-N2+GAP1-M+GAP1-C*->TM+TM+TM+TM+ZnR+ZnR+DO-GTPase2.2->??->HSP70->vWA(canonical)->

■ **GCA_000705615.1; Rhizobium leguminosarum bv. phaseoli CCGM1; counts= 1; multicellularity status: TRUE**

- Calcineurin+GAPEAD1->GAP2->DO-GTPase2.1->GAP1-N1+GAP1-M+GAP1-C*->

■ **GCA_000219495.2; Medicago truncatula; counts= 1; multicellularity status: FALSE**

- DO-GTPase2+DO-GTPase1+GAP1-N1+GAP1-M+GAP1-C*->

■ **GCA_000584955.2; Candidatus Accumulibacter sp. SK-01; counts= 1; multicellularity status: FALSE**

- GAP2->ZnR+DO-GTPase2.1->DO-GTPase1->GAP1-N1+GAP1-M+GAP1-C+EAD1*->CASPASE+EAD1->

■ **GCA_000741785.1; Bifidobacterium stellenboschense; counts= 1; multicellularity status: FALSE**

- TM+TM+TM+TM+ZnR+ZnR+DO-GTPase2.2->GrpE->HSP70->HSP70+vWA(canonical)->DnaJ+DnaJ+ZnR+ZnR+coiled_coil+Fibronectin-like_repeats->ZnR+ZnR+DO-GTPase2.2->GAP1-N2+GAP1-M+GAP1-C*->

■ **GCA_000758965.1; Corynebacterium freneyi DNF00450; counts= 1; multicellularity status: FALSE**

- TM+vWA(canonical)+Fibronectin-like_repeats+TM+beta-rich+lc_tail->Tubulin+alpha_helical+??+??->GAP_unknown1+TM+TM+GAP_unknown2->HRDC-like?+Fibronectin-like_repeats->GAP1-N2+GAP1-M+GAP1-C*->??+DO-GTPase2.2->

■ **GCA_000812585.1; Chromobacterium piscinae; counts= 1; multicellularity status: FALSE**

- Calcineurin+GAPEAD1->GAP2->ZnR+DO-GTPase2.1->GAP1-N1+GAP1-M+GAP1-C*->

■ **GCA_000817655.1; Streptomyces sp. RSD-27; counts= 1; multicellularity status: TRUE**

- GAP1-N2+GAP1-M+GAP1-C*->

■ **GCA_000818175.1; Streptomyces sp. AcH 505; counts= 1; multicellularity status: TRUE**

- GAP1-N2+GAP1-M+GAP1-C*->

■ **GCA_000819915.1; Corynebacterium glutamicum; counts= 1; multicellularity status: FALSE**

- polar_leader+TM+TM+TM->TM+vWA(canonical)+Fibronectin-like_repeats+TM+beta-rich+lc_tail->Tubulin+alpha_helical+??+??->GAP_unknown1+TM+TM+GAP_unknown2->HRDC-like?+Fibronectin-like_repeats->GAP1-N2+GAP1-M+GAP1-C*->beta-rich+vWA(canonical)->??+DO-GTPase2.2->

■ **GCA_000834545.1; Pseudomonas fluorescens; counts= 1; multicellularity status: FALSE**

- GAP2->ZnR+DO-GTPase2.1->DO-GTPase1->GAP1-N1+GAP1-M+GAP1-C+EAD1*->CASPASE+EAD1->

■ **GCA_000878385.1; Methylobacterium radiotolerans; counts= 1; multicellularity status: TRUE**

- Calcineurin+GAPEAD1->GAP2->ZnR+DO-GTPase2.1->DO-GTPase1->GAP1-N1+GAP1-M+GAP1-C*->

■ **GCA_000932205.1; Streptomyces sp. MBRL 601; counts= 1; multicellularity status: TRUE**

- GAP1-N2+GAP1-M+GAP1-C*->

■ **GCA_000961745.1; Rhodospirillaceae bacterium BRH_c57; counts= 1; multicellularity status: FALSE**

- GAP2->ZnR+DO-GTPase2.1->DO-GTPase1->GAP1-N1+GAP1-M+GAP1-C+EAD1*->

■ **GCA_000961795.1; Hyphomonas sp. BRH_c22; counts= 1; multicellularity status: FALSE**

- Calcineurin+GAPEAD1->GAP2->DO-GTPase2.1->DO-GTPase1->GAP1-N1+GAP1-M+GAP1-C*->

■ **GCA_000966975.1; Streptomyces katrae; counts= 1; multicellularity status: TRUE**

- GAP1-N2+GAP1-M+GAP1-C*->

■ **GCA_000966985.1; Streptomyces sp. NRRL S-444; counts= 1; multicellularity status: TRUE**

- GAP1-N2+GAP1-M+GAP1-C*->

■ **GCA_001038505.1; Puniceibacterium sp. IMCC21224; counts= 1; multicellularity status: FALSE**

- DO-GTPase2.2->GAP1-N2+GAP1-M+TM+GAP1-C*->

■ **GCA_001047325.1; Streptomyces viridochromogenes; counts= 1; multicellularity status: TRUE**

- GAP2->ZnR+DO-GTPase2.1->DO-GTPase1->GAP1-N1+GAP1-M+GAP1-C+EAD1*->CASPASE+EAD1->

■ **GCA_001047335.1; Streptomyces regensis; counts= 1; multicellularity status: TRUE**

- TPR_containing_alpha_helical+Fibronectin-like_repeats->ZnR+ZnR+DO-GTPase2.2->GAP1-N2+GAP1-M+GAP1-C*->vWA(canonical)->Pkinase+ZnR+beta-rich->

■ **GCA_001187725.1; Brevibacillus reuszeri; counts= 1; multicellularity status: FALSE**

- TM+vWA(canonical)+YtKA-like+TM+??->Tubulin+coiled_coil+??->Tubulin+coiled_coil+HTH?+??->TM+TM+TM+vWA(canonical)+TM+TM+TM+polar_tail->Fibronectin-like_repeats+??->ZnR+ZnR+DO-GTPase2.2+ZnR+ZnR->GAP1-N2+GAP1-M+GAP1-C+polar_tail+TM+beta-rich*->

■ **GCA_001270675.1; Streptomyces griseus subsp. rhodochrous; counts= 1; multicellularity status: TRUE**

- GAP1-N2+GAP1-M+GAP1-C*->

■ **GCA_001275645.1; Bacillus sp. FJAT-18019; counts= 1; multicellularity status: NA**

- TM+vWA(canonical)+YtKA-like+TM+??->Tubulin+coiled_coil+??->Tubulin+coiled_coil+HTH?+??->TM+TM+TM+vWA(canonical)+TM+TM+TM+polar_tail->Fibronectin-like_repeats+??->ZnR+ZnR+DO-GTPase2.2+ZnR+ZnR->GAP1-N2+GAP1-M+GAP1-C+polar_tail+TM+beta-rich*->

■ **GCA_001277345.1; Paenibacillus solani; counts= 1; multicellularity status: TRUE**

- TM+vWA(canonical)+YtKA-like+TM+??->Tubulin+coiled_coil+??->Tubulin+coiled_coil+HTH?+??->TM+TM+TM+vWA(canonical)+TM+TM+TM+polar_tail->Fibronectin-like_repeats+??->ZnR+ZnR+DO-GTPase2.2+ZnR+ZnR->GAP1-N2+GAP1-M+GAP1-C+polar_tail+TM+beta-rich*->

■ **GCA_001277855.1; Paenibacillus polymyxa; counts= 1; multicellularity status: TRUE**

- TM+vWA(canonical)+YtKA-like+TM+??->Tubulin+coiled_coil+??->Tubulin+coiled_coil+HTH?+??->TM+TM+TM+vWA(canonical)+TM+TM+TM+polar_tail->Fibronectin-like_repeats+??->ZnR+ZnR+DO-GTPase2.2+ZnR+ZnR->GAP1-N2+GAP1-M+GAP1-C+polar_tail+TM+beta-rich*->

■ **GCA_001279425.1; Streptomyces sp. NRRL F-2295; counts= 1; multicellularity status: TRUE**

- GAP1-N2+GAP1-M+GAP1-C*->

■ **GCA_001279735.1; Streptomyces sp. MMG1522; counts= 1; multicellularity status: TRUE**

- GAP1-N2+GAP1-M+GAP1-C*->

■ **GCA_001280045.1; Streptomyces sp. NRRL WC-3723; counts= 1; multicellularity status: TRUE**

- TPR_containing_alpha_helical+Fibronectin-like_repeats->ZnR+ZnR+DO-GTPase2.2->GAP1-N2+GAP1-M+GAP1-C*->vWA(canonical)->Pkinase+ZnR+beta-rich->

■ **GCA_001280595.1; Paenibacillus sp. A59; counts= 1; multicellularity status: TRUE**

- TM+vWA(canonical)+YtKA-like+TM+??->Tubulin+coiled_coil+??->Tubulin+coiled_coil+HTH?+??->TM+TM+TM+vWA(canonical)+TM+TM+TM+polar_tail->Fibronectin-like_repeats+??->ZnR+ZnR+DO-GTPase2.2+ZnR+ZnR->GAP1-N2+GAP1-M+GAP1-C+polar_tail+TM+beta-rich*->

■ **GCA_001280885.1; Streptomyces sp. XY332; counts= 1; multicellularity status: TRUE**

- TPR_containing_alpha_helical+Fibronectin-like_repeats->GAP1-N2+GAP1-M+GAP1-C*->TM+TM+TM+TM+ZnR+ZnR+DO-GTPase2.2+TM->vWA(canonical)->PP2C->Pkinase+ZnR+beta-rich->

■ **GCA_001298555.1; Actinobacteria bacterium OV450; counts= 1; multicellularity status: NA**

- Fibronectin-like_repeats->ZnR+ZnR+DO-GTPase2.2->GAP1-N2+GAP1-M+GAP1-C*->

■ **GCA_001304075.1; candidate division BRC1 bacterium SM23_51; counts= 1; multicellularity status: NA**

- ZnR+DO-GTPase2.1->GAP1-N2+GAP1-M+TM+GAP1-C*->

■ **GCA_001400775.1; Pseudomonas congelans; counts= 1; multicellularity status: FALSE**

- Calcineurin+GAPEAD1->GAP2->ZnR+DO-GTPase2.1->GAP1-N1+GAP1-M+GAP1-C*->

■ **GCA_001426045.1; Methylobacterium sp. Leaf94; counts= 1; multicellularity status: TRUE**

- GAP1-N1+GAP1-M+GAP1-C*->

■ **GCA_001425705.1; Pelomonas sp. Root1217; counts= 1; multicellularity status: FALSE**

- Calcineurin+GAPEAD1->GAP2->ZnR+DO-GTPase2.1->DO-GTPase1->GAP1-N1+GAP1-M+GAP1-C*->

■ **GCA_001514305.1; Streptomyces sp. RV15; counts= 1; multicellularity status: TRUE**

- TPR_containing_alpha_helical+Fibronectin-like_repeats->ZnR+ZnR+DO-GTPase2.2->GAP1-N2+GAP1-M+GAP1-C*->TM+TM+TM+TM+ZnR+ZnR+DO-GTPase2.2+TM->vWA(canonical)->PP2C->Pkinase+ZnR+beta-rich->

■ **GCA_001526225.1; Burkholderia ubonensis; counts= 1; multicellularity status: FALSE**

- Calcineurin+GAPEAD1->GAP2->||<-?||DO-GTPase1->GAP1-N1+GAP1-M+GAP1-C*->

■ **GCA_001527385.1; Burkholderia ubonensis; counts= 1; multicellularity status: FALSE**

- Calcineurin+GAPEAD1->GAP2->||<-?||DO-GTPase1->GAP1-N1+GAP1-M+GAP1-C*->

■ **GCA_001528875.1; Burkholderia ubonensis; counts= 1; multicellularity status: FALSE**

- Calcineurin+GAPEAD1->GAP2->DO-GTPase2.1->DO-GTPase1->GAP1-N1+GAP1-M+GAP1-C*->

■ **GCA_001546485.1; Gardnerella vaginalis; counts= 1; multicellularity status: FALSE**

- TM+TM+TM+TM+ZnR+ZnR+DO-GTPase2.2->?->HSP70->HSP70+vWA(canonical)->DnaJ+DnaJ+ZnR+ZnR+coiled_coil+Fibronectin-like_repeats->ZnR+ZnR+DO-GTPase2.2->GAP1-N2+GAP1-M+GAP1-C*->

■ **GCA_001560865.1; Pseudomonas aeruginosa; counts= 1; multicellularity status: FALSE**

- GAP2->ZnR+DO-GTPase2.1->DO-GTPase1->GAP1-N1+GAP1-M+GAP1-C+EAD1*->CASPASE+EAD1->

■ **GCA_001567085.1; Chloroflexi bacterium OLB15; counts= 1; multicellularity status: TRUE**

- GAP1-N2+GAP1-M+GAP1-C*->

■ **GCA_001589295.1; Sorangium cellulosum; counts= 1; multicellularity status: TRUE**

- GAP1-N1+GAP1-M+GAP1-C*->||<-DO-GTPase1<-ZnR+DO-GTPase2.1<-GAP2

■ **GCA_001613855.1; Thalassospira xiamenensis; counts= 1; multicellularity status: FALSE**

- Calcineurin+GAPEAD1->GAP2->ZnR(fragment)+DO-GTPase2.1->DO-GTPase1->GAP1-N1+GAP1-M+GAP1-C*->

■ **GCA_001636565.1; Frankia sp. EI5c; counts= 1; multicellularity status: TRUE**

- GAP1-N2+GAP1-M+GAP1-C+ZnR+DO-GTPase2*->

■ **GCA_001644415.1; Planctomycetaceae bacterium SCGC AG-212-F19; counts= 1; multicellularity status: NA**

- GAP1-N2+GAP1-M+TM+low_complexity*->

■ **GCA_001705105.1; Bradyrhizobium sp. UASWS1016; counts= 1; multicellularity status: TRUE**

- DO-GTPase1->GAP1-N1+GAP1-M+GAP1-C*->

■ **GCA_001723765.1; Candidatus Scalindua rubra; counts= 1; multicellularity status: NA**

- DO-GTPase2.1->GAP1-N2+GAP1-M+GAP1-C*->

■ **GCA_001807225.1; Corynebacterium sp. HMSC05E07; counts= 1; multicellularity status: FALSE**

- polar_leader+TM+TM+TM->TM+vWA(canonical)+Fibronectin-like_repeats+TM+beta-rich+lc_tail->Tubulin+alpha_helical+??+??->GAP_unknown1+TM+TM+GAP_unknown2->HRDC-like?+Fibronectin-like_repeats->GAP1-N2+GAP1-M+GAP1-C*->beta-rich+vWA(canonical)->??+DO-GTPase2.2->polar_leader+TM+TM+TM->

■ **GCA_001807025.1; Corynebacterium sp. HMSC11E11; counts= 1; multicellularity status: FALSE**

- polar_leader+TM+TM+TM->TM+vWA(canonical)+Fibronectin-like_repeats+TM+beta-rich+lc_tail->Tubulin+alpha_helical+??+??->GAP_unknown1+TM+TM+GAP_unknown2->HRDC-like?+Fibronectin-like_repeats->GAP1-N2+GAP1-M+GAP1-C*->beta-rich+vWA(canonical)->??+DO-GTPase2.2->polar_leader+TM+TM+TM->

■ **GCA_001767115.1; Acidobacteria bacterium RIFCSPLOWO2_12_FULL_59_11; counts= 1; multicellularity status: FALSE**

- DO-GTPase1->GAP1-N1+GAP1-M+GAP1-C*->

■ **GCA_001772075.1; Burkholderiales bacterium RIFCSPHIGHO2_02_FULL_64_19; counts= 1; multicellularity status: FALSE**

- Calcineurin+GAPEAD1->GAP2->ZnR+DO-GTPase2.1->DO-GTPase1->GAP1-N1+GAP1-M+GAP1-C*->

■ **GCA_001804865.1; Lentisphaerae bacterium GWF2_57_35; counts= 1; multicellularity status: FALSE**

- FHA-like+??+ZnR+ZnR+DO-GTPase2.2+??->GAP1-N2+GAP1-M+TM+GAP1-C*->

■ **GCA_001803235.1; Lentisphaerae bacterium RIFOXYA12_64_32; counts= 1; multicellularity status: FALSE**

- GAP1-N2+GAP1-M*->??+TM+??+beta-rich??->

■ **GCA_001828265.1; Planctomycetes bacterium RBG_13_63_9; counts= 1; multicellularity status: NA**

- FGS-fold_domain->ZnR+DO-GTPase2.1->GAP1-N2+GAP1-M+GAP1-C*->

■ **GCA_001828305.1; Planctomycetes bacterium RBG_16_64_10; counts= 1; multicellularity status: NA**

- FGS-fold_domain->ZnR+DO-GTPase2.1->GAP1-N2+GAP1-M+GAP1-C(frag)*->

■ **GCA_001828335.1; Planctomycetes bacterium RBG_16_64_12; counts= 1; multicellularity status: NA**

- ZnR+DO-GTPase2.1->GAP1-N2+GAP1-M+GAP1-C*->

■ **GCA_001830395.1; Pseudomonadales bacterium RIFCSPLOWO2_12_59_9; counts= 1; multicellularity status: FALSE**

- Calcineurin+GAPEAD1->GAP2->DO-GTPase2.1->DO-GTPase1->GAP1-N1+GAP1-M+GAP1-C*->

■ **GCA_001831115.1; Verrucomicrobia bacterium GWF2_62_7; counts= 1; multicellularity status: FALSE**

- GAP1-N2+GAP1-M+TM+GAP1-C*->

■ **GCA_001854655.1; Frankia sp. Cc1.17; counts= 1; multicellularity status: TRUE**

- GAP1-N2+GAP1-M+GAP1-C+ZnR+DO-GTPase2*->

- **GCA_001898335.1; Mucilaginibacter sp. 44-25; counts= 1; multicellularity status: FALSE**
  - TIR+GAPEAD1->GAP2->ZnR+DO-GTPase2.1->DO-GTPase1->GAP1-N1+GAP1-M+GAP1-C*->
- **GCA_001897835.1; Bacteroidetes bacterium 46-16; counts= 1; multicellularity status: FALSE**
  - GAP2->ZnR+DO-GTPase2.1->DO-GTPase1->GAP1-N1+GAP1-M+GAP1-C+EAD1*->PNP_UDP_1+EAD1->
- **GCA_001898665.1; Burkholderiales bacterium 68-20; counts= 1; multicellularity status: FALSE**
  - GAP2->DO-GTPase2.1->DO-GTPase1->GAP1-N1+GAP1-M+GAP1-C+EAD1*->CASPASE+EAD1->
- **GCA_001905305.1; Mycobacterium sp. SWH-M5; counts= 1; multicellularity status: FALSE**
  - HRDC-like?+Fibronectin-like_repeats->GAP1-N2+GAP1-M+GAP1-C(frag)*->
- **GCA_001905065.1; Saccharothrix sp. CB00851; counts= 1; multicellularity status: TRUE**
  - HSP70->||<-?||GrpE->HSP70->TPR_containing_alpha_helical+Fibronectin-like_repeats->ZnR+ZnR+DO-GTPase2.2->GAP1-N2+GAP1-M+GAP1-C*->TM+TM+TM+TM+ZnR+ZnR+DO-GTPase2.2->
- **GCA_001905525.1; Streptomyces sp. TSRI0395; counts= 1; multicellularity status: TRUE**
  - GAP1-N2+GAP1-M+GAP1-C*->
- **GCA_001905905.1; Streptomyces sp. CB02366; counts= 1; multicellularity status: TRUE**
  - GAP1-N2+GAP1-M+GAP1-C*->
- **GCA_001912755.2; Corynebacterium glutamicum; counts= 1; multicellularity status: FALSE**
  - polar_leader+TM+TM+TM->TM+vWA(canonical)+Fibronectin-like_repeats+TM+beta-rich+lc_tail->Tubulin+alpha_helical+??+??->GAP_unknown1+TM+TM+GAP_unknown2->HRDC-like?+Fibronectin-like_repeats->GAP1-N2+GAP1-M+GAP1-C*->beta-rich+vWA(canonical)->??+DO-GTPase2.2->
- **GCA_001912725.1; Corynebacterium glutamicum; counts= 1; multicellularity status: FALSE**
  - polar_leader+TM+TM+TM->TM+vWA(canonical)+Fibronectin-like_repeats+TM+beta-rich+lc_tail->Tubulin+alpha_helical+??+??->GAP_unknown1+TM+TM+GAP_unknown2->HRDC-like?+Fibronectin-like_repeats->GAP1-N2+GAP1-M+GAP1-C*->beta-rich+vWA(canonical)->??+DO-GTPase2.2->
- **GCA_001917305.1; Verrucomicrobia bacterium CAG:312_58_20; counts= 1; multicellularity status: FALSE**
  - FHA-like?+ZnR+ZnR+DO-GTPase2.2->GAP1-N2+GAP1-M+TM+GAP1-C*->
- **GCA_001921865.1; Pseudomonas chlororaphis; counts= 1; multicellularity status: FALSE**
  - GAP2->ZnR+DO-GTPase2.1->DO-GTPase1->GAP1-N1+GAP1-M+GAP1-C+EAD1*->CASPASE+EAD1->
- **GCA_001955985.1; Paenibacillus pabuli; counts= 1; multicellularity status: TRUE**
  - TM+vWA(canonical)+YtKA-like+TM+??->Tubulin+coiled_coil+??->Tubulin+coiled_coil+HTH?+??->TM+TM+TM+vWA(canonical)+TM+TM+TM+polar_tail->Fibronectin-like_repeats+??->ZnR+ZnR+DO-GTPase2.2+ZnR+ZnR->GAP1-N2+GAP1-M+GAP1-C+polar_tail+TM+beta-rich*->
- **GCA_001956035.1; Paenibacillus amylolyticus; counts= 1; multicellularity status: TRUE**
  - TM+vWA(canonical)+YtKA-like+TM+??->Tubulin+coiled_coil+??->Tubulin+coiled_coil+HTH?+??->TM+TM+TM+vWA(canonical)+TM+TM+TM+polar_tail->Fibronectin-like_repeats+??->ZnR+ZnR+DO-GTPase2.2+ZnR+ZnR->GAP1-N2+GAP1-M+GAP1-C+polar_tail+TM+beta-rich*->
- **GCA_001956835.1; Paenibacillus macerans; counts= 1; multicellularity status: TRUE**
  - TM+TM+TM+vWA(canonical)+TM+TM+TM+polar_tail->Fibronectin-like_repeats+??->ZnR+ZnR+DO-GTPase2.2+ZnR+ZnR->GAP1-N2+GAP1-M+GAP1-C+polar_tail+TM+beta-rich*->
- **GCA_001974825.1; Corchorus olitorius; counts= 1; multicellularity status: NA**

- TM+TM+TM+vWA+TM+TM+TM+ZnR+GTPase+GAP1-N2+GAP1-M+GAP1-C+ATP__grasp+ATP__grasp+Rossmann-like+thioredoxin__fold*->

■ **GCA__002083655.1; Proteobacteria bacterium ST__bin14; counts= 1; multicellularity status: FALSE**

- Calcineurin+GAPEAD1->GAP2->ZnR+DO-GTPase2.1->DO-GTPase1->GAP1-N1+GAP1-M+GAP1-C*->

■ **GCA__002085445.1; Beggiatoa sp. 4572__84; counts= 1; multicellularity status: TRUE**

- GAP1-N2+GAP1-M+GAP1-C*->TM+TM+TM+TM+ZnR+ZnR+DO-GTPase2.2->||<-HSP70<-GrpE

■ **GCA__002086865.1; Mycobacterium kansasii; counts= 1; multicellularity status: FALSE**

- TM+DUF4190+TM->TM+DUF4190+TM->polar__leader+TM+TM+TM->TM+vWA(canonical)+Fibronectin-like__repeats+TM+beta-rich+lc__tail->Tubulin+alpha__helical+??+??->GAP__unknown1+TM+TM+GAP__unknown2->HRDC-like?+Fibronectin-like__repeats->GAP1-N2+GAP1-M+GAP1-C*->beta-rich+vWA(canonical)->??+DO-GTPase2.2->

■ **GCA__002086855.1; Mycobacterium kansasii; counts= 1; multicellularity status: FALSE**

- TM+DUF4190+TM->TM+DUF4190+TM->polar__leader+TM+TM+TM->TM+vWA(canonical)+Fibronectin-like__repeats+TM+beta-rich+lc__tail->Tubulin+alpha__helical+??+??->GAP__unknown1+TM+TM+GAP__unknown2->HRDC-like?+Fibronectin-like__repeats->GAP1-N2+GAP1-M+GAP1-C*->beta-rich+vWA(canonical)->??+DO-GTPase2.2->

■ **GCA__002086925.1; Mycobacterium kansasii; counts= 1; multicellularity status: FALSE**

- TM+DUF4190+TM->TM+DUF4190+TM->polar__leader+TM+TM+TM->TM+vWA(canonical)+Fibronectin-like__repeats+TM+beta-rich+lc__tail->Tubulin+alpha__helical+??+??->GAP__unknown1+TM+TM+GAP__unknown2->HRDC-like?+Fibronectin-like__repeats->GAP1-N2+GAP1-M+GAP1-C*->beta-rich+vWA(canonical)->??+DO-GTPase2.2->

■ **GCA__002086935.1; Mycobacterium kansasii; counts= 1; multicellularity status: FALSE**

- TM+DUF4190+TM->TM+DUF4190+TM->polar__leader+TM+TM+TM->TM+vWA(canonical)+Fibronectin-like__repeats+TM+beta-rich+lc__tail->Tubulin+alpha__helical+??+??->GAP__unknown1+TM+TM+GAP__unknown2->HRDC-like?+Fibronectin-like__repeats->GAP1-N2+GAP1-M+GAP1-C*->beta-rich+vWA(canonical)->??+DO-GTPase2.2->

■ **GCA__002104865.1; Streptomyces sp. BF-3; counts= 1; multicellularity status: TRUE**

- GAP1-N2+GAP1-M+GAP1-C*->

■ **GCA__001432265.1; Acinetobacter baumannii; counts= 1; multicellularity status: FALSE**

- GAP2->ZnR+DO-GTPase2.1->DO-GTPase1->GAP1-N1+GAP1-M+GAP1-C*->

■ **GCA__002159375.1; Alistipes sp. An54; counts= 1; multicellularity status: FALSE**

- Calcineurin+GAPEAD1->?->DO-GTPase2.1->DO-GTPase1->GAP1-N1+GAP1-M+GAP1-C*->

■ **GCA__002170515.1; Verrucomicrobia bacterium TMED71; counts= 1; multicellularity status: FALSE**

- FHA-like?+ZnR+ZnR+DO-GTPase2.2->GAP1-N2+GAP1-M+low__complexity+TM+TM*->

■ **GCA__002170435.1; Verrucomicrobiaceae bacterium TMED76; counts= 1; multicellularity status: NA**

- FHA-like?+ZnR+ZnR+DO-GTPase2.2->GAP1-N2+GAP1-M+TM+beta-rich+??*->??+TM+??->

■ **GCA__002200935.1; Rhizobium yanglingense; counts= 1; multicellularity status: TRUE**

- GAPEAD1->GAP2->GAP1-N1+GAP1-M+GAP1-C*->?->Calcineurin->

■ **GCA__002204655.1; Cuniculiplasma sp. C__DKE; counts= 1; multicellularity status: TRUE**

- TM+TM+TM+TM+TM+TM+DO-GTPase2.1->GAP1-N2+GAP1-M+GAP1-C*->??->TM__region+DO-GTPase1->

■ **GCA__002242735.1; Streptomyces sp. 2R; counts= 1; multicellularity status: TRUE**

- GAP1-N2+GAP1-M+GAP1-C*->

■ **GCA__002254715.1; archaeon ex4484__74; counts= 1; multicellularity status: NA**

- GAP1-N2+GAP1-M*->

- **GCA_002279215.1; Planctomycetia bacterium 21-64-5; counts= 1; multicellularity status: NA**
  - ??+GAP1-N2+GAP1-M+??+TM+TM*->
- **GCA_002280395.1; Flavobacteriales bacterium 32-35-8; counts= 1; multicellularity status: FALSE**
  - GAP2->ZnR+DO-GTPase2.1->DO-GTPase1->GAP1-N1+GAP1-M+GAP1-C+EAD1*->??+EAD1->EAD1+Trypsin->
- **GCA_002281555.1; Hydrogenophilales bacterium 28-61-23; counts= 1; multicellularity status: FALSE**
  - Calcineurin+EAD1->||<-?||GAP2->ZnR+DO-GTPase2.1->DO-GTPase1->GAP1-N1+GAP1-M+GAP1-C+EAD1*->
- **GCA_002264305.1; Paenibacillus taichungensis; counts= 1; multicellularity status: TRUE**
  - ZnR+ZnR+DO-GTPase2.2+ZnR+ZnR->GAP1-N2+GAP1-M+GAP1-C+polar_tail+TM+beta-rich*->
- **GCA_002286935.1; Bifidobacterium criceti; counts= 1; multicellularity status: FALSE**
  - TM+TM+TM+TM+ZnR+ZnR+DO-GTPase2.2->GrpE->HSP70->HSP70+vWA(canonical)->DnaJ+DnaJ+ZnR+ZnR+coiled_coil+Fibronectin-like_repeats->ZnR+ZnR+DO-GTPase2.2->GAP1-N2+GAP1-M+GAP1-C*->
- **GCA_002304375.1; Opitutae bacterium Tous-C2FEB; counts= 1; multicellularity status: FALSE**
  - ZnR+ZnR+DO-GTPase2.2->GAP1-N2+GAP1-M+TM+GAP1-C*->
- **GCA_002290585.1; Opitutae bacterium AMD-G3; counts= 1; multicellularity status: FALSE**
  - FHA-like?+ZnR+ZnR+DO-GTPase2.2->GAP1-N2+GAP1-M+TM+GAP1-C*->
- **GCA_002290595.1; Opitutae bacterium AMD-G1; counts= 1; multicellularity status: FALSE**
  - FHA-like?+ZnR+ZnR+DO-GTPase2.2->GAP1-N2+GAP1-M+TM+GAP1-C*->
- **GCA_002401295.1; SAR324 cluster bacterium; counts= 1; multicellularity status: NA**
  - GAP2->DO-GTPase2.1->DO-GTPase1->GAP1-N1+GAP1-M+GAP1-C+PNP_UDP_1*->
- **GCA_002401655.1; Gammaproteobacteria bacterium; counts= 1; multicellularity status: NA**
  - GAP2->ZnR+DO-GTPase2.1->DO-GTPase1->GAP1-N1+GAP1-M+GAP1-C+EAD1*->
- **GCA_002733575.1; Blastopirellula sp.; counts= 1; multicellularity status: TRUE**
  - FGS-fold_domain->ZnR+DO-GTPase2.1->GAP1-N2+GAP1-M+GAP1-C*->
- **GCA_002735695.1; Chitinimonas sp. BJB300; counts= 1; multicellularity status: FALSE**
  - Calcineurin+GAPEAD1->GAP2->ZnR+DO-GTPase2.1->DO-GTPase1->GAP1-N1+GAP1-M+GAP1-C*->
- **GCA_002735705.1; Janthinobacterium sp. BJB426; counts= 1; multicellularity status: FALSE**
  - GAP2->DO-GTPase2.1->DO-GTPase1->GAP1-N1+GAP1-M+GAP1-C+EAD1*->CASPASE+EAD1->
- **GCA_002737185.1; Opitutae bacterium; counts= 1; multicellularity status: FALSE**
  - FHA-like?+ZnR+ZnR+DO-GTPase2.2->GAP1-N2+GAP1-M+TM+GAP1-C*->
- **GCA_002737585.1; Planctomycetaceae bacterium; counts= 1; multicellularity status: NA**

- ZnR+ZnR+DO-GTPase2.2->GAP1-N2+GAP1-M+TM*->

■ **GCA_002748445.1; candidate division KSB3 bacterium; counts= 1; multicellularity status: NA**

- GAP1-N2+GAP1-M+GAP1-C*->

■ **GCA_002754535.1; Streptomyces sp. 61; counts= 1; multicellularity status: TRUE**

- TPR_containing_alpha_helical+Fibronectin-like_repeats->ZnR+ZnR+DO-GTPase2.2->GAP1-N2+GAP1-M+GAP1-C*->TM+TM+TM+TM+ZnR+ZnR+DO-GTPase2.2+TM->?->vWA(canonical)->PP2C->Pkinase+ZnR+beta-rich->

■ **GCA_002750415.1; Paenibacillus sp. LK1; counts= 1; multicellularity status: TRUE**

- TM+vWA(canonical)+YtKA-like+TM+??->Tubulin+coiled_coil+??->Tubulin+coiled_coil+HTH?+??->TM+TM+TM+vWA(canonical)+TM+TM+TM+polar_tail->Fibronectin-like_repeats+??->ZnR+ZnR+DO-GTPase2.2+ZnR+ZnR->GAP1-N2+GAP1-M+GAP1-C+polar_tail+TM+beta-rich*->

■ **GCA_002760695.1; Acinetobacter baumannii; counts= 1; multicellularity status: FALSE**

- PNP_UDP_1+EAD1->GAP2->ZnR+DO-GTPase2.1->DO-GTPase1->GAP1-N1+GAP1-M+GAP1-C*->

■ **GCA_002760725.1; Acinetobacter baumannii; counts= 1; multicellularity status: FALSE**

- PNP_UDP_1+EAD1->GAP2->ZnR+DO-GTPase2.1->DO-GTPase1->GAP1-N1+GAP1-M+GAP1-C*->

■ **GCA_002760715.1; Acinetobacter baumannii; counts= 1; multicellularity status: FALSE**

- PNP_UDP_1+EAD1->GAP2->ZnR+DO-GTPase2.1->DO-GTPase1->GAP1-N1+GAP1-M+GAP1-C*->

■ **GCA_002760785.1; Acinetobacter baumannii; counts= 1; multicellularity status: FALSE**

- PNP_UDP_1+EAD1->GAP2->ZnR+DO-GTPase2.1->DO-GTPase1->GAP1-N1+GAP1-M+GAP1-C*->

■ **GCA_002760765.1; Acinetobacter baumannii; counts= 1; multicellularity status: FALSE**

- GAP2->ZnR+DO-GTPase2.1->DO-GTPase1->GAP1-N1+GAP1-M+GAP1-C*->

■ **GCA_002760825.1; Acinetobacter baumannii; counts= 1; multicellularity status: FALSE**

- ZnR+DO-GTPase2.1->DO-GTPase1->GAP1-N1+GAP1-M+GAP1-C*->

■ **GCA_002760895.1; Acinetobacter baumannii; counts= 1; multicellularity status: FALSE**

- GAP1-N1+GAP1-M+GAP1-C*->

■ **GCA_002775095.1; Corynebacterium striatum; counts= 1; multicellularity status: FALSE**

- polar_leader+TM+TM+TM->PSE->PSE->GAP_unknown1+TM+TM+GAP_unknown2->HRDC-like?+Fibronectin-like_repeats->HRDC-like?+Fibronectin-like_repeats->GAP1-N2+GAP1-M+GAP1-C(frag)*->?->beta-rich+vWA(canonical)->??+DO-GTPase2.2->polar_leader+TM+TM+TM->

■ **GCA_002775105.1; Corynebacterium striatum; counts= 1; multicellularity status: FALSE**

- polar_leader+TM+TM+TM->PSE->PSE->GAP_unknown1+TM+TM+GAP_unknown2->HRDC-like?+Fibronectin-like_repeats->GAP1-N2+GAP1-M+GAP1-C*->beta-rich+vWA(canonical)->??+DO-GTPase2.2->polar_leader+TM+TM+TM->

■ **GCA_002794595.1; Chloroflexi bacterium; counts= 1; multicellularity status: TRUE**

- GAP1-N2+GAP1-M+GAP1-C*->

■ **GCA_002808645.1; Streptomyces albidoflavus; counts= 1; multicellularity status: TRUE**

- GAP1-N2+GAP1-M+GAP1-C*->

■ **GCA_002813225.1; Streptomyces sp. Ag109_G2-1; counts= 1; multicellularity status: TRUE**

- GAP1-N2+GAP1-M+GAP1-C*->

■ **GCA_002836235.1; Psychrobacter sp. Choline-02u-13; counts= 1; multicellularity status: FALSE**

- DO-GTPase1->GAP1-N1+GAP1-M+GAP1-C*->

■ **GCA_001896795.2; Thermomonospora sp. CIF 1; counts= 1; multicellularity status: TRUE**

- GrpE->HSP70->TPR_containing_alpha_helical+Fibronectin-like_repeats->ZnR+ZnR+DO-GTPase2.2->GAP1-N2+GAP1-M+GAP1-C*->TM+TM+TM+TM+ZnR+ZnR+DO-GTPase2.2+TM->

■ **GCA_002840095.1; Gammaproteobacteria bacterium HGW-Gammaproteobacteria-1; counts= 1; multicellularity status: FALSE**

- Calcineurin+GAPEAD1->GAP2->DO-GTPase2.1->DO-GTPase1->GAP1-N1+GAP1-M+GAP1-C*->

■ **GCA_002841525.1; Firmicutes bacterium HGW-Firmicutes-17; counts= 1; multicellularity status: FALSE**

- DO-GTPase1->GAP1-N1+GAP1-M+GAP1-C*->

■ **GCA_002842145.1; Betaproteobacteria bacterium HGW-Betaproteobacteria-6; counts= 1; multicellularity status: FALSE**

- Calcineurin+GAPEAD1->GAP2->ZnR+DO-GTPase2.1->PSE->GAP1-N1+GAP1-M+GAP1-C*->

■ **GCA_002842665.1; Bacteroidetes bacterium HGW-Bacteroidetes-11; counts= 1; multicellularity status: FALSE**

- GAP2->ZnR+DO-GTPase2.1->DO-GTPase1->GAP1-N1+GAP1-M+GAP1-C+EAD1*->PNP_UDP_1+EAD1->

■ **GCA_002843035.1; Actinobacteria bacterium HGW-Actinobacteria-5; counts= 1; multicellularity status: NA**

- ZnR+ZnR+DO-GTPase2.2->GAP1-N2+GAP1-M+low_complexity*->

■ **GCA_002860365.1; Bifidobacterium parmae; counts= 1; multicellularity status: FALSE**

- TM+TM+TM+TM+ZnR+ZnR+DO-GTPase2.2->GrpE->HSP70->HSP70+vWA(canonical)->DnaJ+DnaJ+ZnR+ZnR+coiled_coil+Fibronectin-like_repeats->ZnR+ZnR+DO-GTPase2.2->GAP1-N2+GAP1-M+GAP1-C*->

■ **GCA_002863645.1; Psychrobacter sp. MES7-P7E; counts= 1; multicellularity status: FALSE**

- GAP2->ZnR+DO-GTPase2.1->DO-GTPase1->GAP1-N1+GAP1-M+GAP1-C*->

■ **GCA_002873265.1; Vibrio lentus; counts= 1; multicellularity status: FALSE**

- Calcineurin+GAPEAD1->GAP2->ZnR+DO-GTPase2.1->DO-GTPase1->GAP1-N1+GAP1-M+GAP1-C*->

■ **GCA_002878555.1; Vibrio splendidus; counts= 1; multicellularity status: FALSE**

- PNP_UDP_1+EAD1->GAP2->ZnR+DO-GTPase2.1->DO-GTPase1->GAP1-N1+GAP1-M+GAP1-C+EAD1*->SLATT->

■ **GCA_003002065.1; Pseudosporangium ferrugineum; counts= 1; multicellularity status: TRUE**

- GAP2->ZnR+DO-GTPase2.1->DO-GTPase1->GAP1-N1+GAP1-M+GAP1-C+EAD1*->PNP_UDP_1+EAD1->

■ **GCA_003010405.1; Acidobacteria bacterium; counts= 1; multicellularity status: FALSE**

- DO-GTPase1->GAP1-N1+GAP1-M+GAP1-C*->

■ **GCA_900084005.1; Klebsiella oxytoca; counts= 1; multicellularity status: FALSE**

- Calcineurin+GAPEAD1->GAP2->DO-GTPase2.1->DO-GTPase1->GAP1-N1+GAP1-M+GAP1-C*->

■ **GCA_900086575.1; Komagataeibacter rhaeticus; counts= 1; multicellularity status: FALSE**

  - Calcineurin+GAPEAD1->GAP2->ZnR+DO-GTPase2.1->DO-GTPase1->GAP1-N1+GAP1-M+GAP1-C*->

- **GCA_900091315.1; Streptomyces sp. DI166; counts= 1; multicellularity status: TRUE**
  - TPR_containing_alpha_helical+Fibronectin-like_repeats->ZnR+ZnR+DO-GTPase2.2->GAP1-N2+GAP1-M+GAP1-C*->
- **GCA_900091875.1; Streptomyces sp. BpilaLS-43; counts= 1; multicellularity status: TRUE**
  - GAP1-N2+GAP1-M+GAP1-C*->
- **GCA_900091755.1; Streptomyces sp. TverLS-915; counts= 1; multicellularity status: TRUE**
  - GAP1-N2+GAP1-M+GAP1-C*->
- **GCA_900091715.1; Streptomyces sp. DpondAA-F4a; counts= 1; multicellularity status: TRUE**
  - GAP1-N2+GAP1-M+GAP1-C*->
- **GCA_900091895.1; Streptomyces sp. IgraMP-1; counts= 1; multicellularity status: TRUE**
  - GAP1-N2+GAP1-M+GAP1-C*->
- **GCA_900091965.1; Streptomyces sp. Ncost-T6T-2b; counts= 1; multicellularity status: TRUE**
  - GAP1-N2+GAP1-M+GAP1-C*->
- **GCA_900090155.1; Streptomyces sp. ScaeMP-e48; counts= 1; multicellularity status: TRUE**
  - GAP1-N2+GAP1-M+GAP1-C*->
- **GCA_900101545.1; Variovorax sp. CF079; counts= 1; multicellularity status: FALSE**
  - Calcineurin+GAPEAD1->GAP2->ZnR+DO-GTPase2.1->DO-GTPase1->GAP1-N1+GAP1-M+GAP1-C*->
- **GCA_900101225.1; Paenibacillus sp. CF095; counts= 1; multicellularity status: TRUE**
  - TM+vWA(canonical)+YtKA-like+TM+??->Tubulin+coiled_coil+??->Tubulin+coiled_coil+HTH?+??->TM+TM+TM+vWA(canonical)+TM+TM+TM+polar_tail->Fibronectin-like_repeats+??->ZnR+ZnR+DO-GTPase2.2+ZnR+ZnR->GAP1-N2+GAP1-M+GAP1-C+polar_tail+TM+beta-rich*->
- **GCA_900101405.1; Bradyrhizobium sp. R5; counts= 1; multicellularity status: TRUE**
  - Calcineurin+GAPEAD1->GAP2->ZnR+DO-GTPase2.1->DO-GTPase1->GAP1-N1+GAP1-M+GAP1-C*->
- **GCA_900103785.1; Geodermatophilus siccatus; counts= 1; multicellularity status: TRUE**
  - Pkinase+TM->TM->ZnR+ZnR+DO-GTPase2.2->GAP1-N2+GAP1-M+GAP1-C*->?->||<-?<-?||?->Pkinase+low_complexity->
- **GCA_900104025.1; Geodermatophilus sp. DSM 45219; counts= 1; multicellularity status: TRUE**
  - Pkinase+TM->TM->ZnR+ZnR+DO-GTPase2.2->GAP1-N2+GAP1-M+GAP1-C*->?->||<-?<-?||?->Pkinase+low_complexity->
- **GCA_900103225.1; Pseudomonas congelans; counts= 1; multicellularity status: FALSE**
  - Calcineurin+GAPEAD1->GAP2->ZnR+DO-GTPase2.1->DO-GTPase1->GAP1-N1+GAP1-M+GAP1-C*->
- **GCA_900101695.1; Pseudomonas sp. UC 17F4; counts= 1; multicellularity status: FALSE**
  - Calcineurin+GAPEAD1->GAP2->ZnR+DO-GTPase2.1->DO-GTPase1->GAP1-N1+GAP1-M+GAP1-C*->
- **GCA_900106525.1; Lysobacter enzymogenes; counts= 1; multicellularity status: FALSE**

- Calcineurin+GAPEAD1->GAP2->ZnR+DO-GTPase2.1->DO-GTPase1->GAP1-N1+GAP1-M+GAP1-C*->

■ **GCA_900107105.1; Geodermatophilus africanus; counts= 1; multicellularity status: TRUE**

- Pkinase+TM->TM->ZnR+ZnR+DO-GTPase2.2->GAP1-N2+GAP1-M+GAP1-C*->?->||<-?<-?||?->Pkinase+low_complexity->

■ **GCA_900107345.1; Pseudomonas syringae; counts= 1; multicellularity status: FALSE**

- Calcineurin+GAPEAD1->GAP2->ZnR+DO-GTPase2.1->DO-GTPase1->GAP1-N1+GAP1-M+GAP1-C*->

■ **GCA_900107605.1; Acidovorax soli; counts= 1; multicellularity status: FALSE**

- Calcineurin+GAPEAD1->GAP2->ZnR+DO-GTPase2.1->DO-GTPase1->GAP1-N1+GAP1-M+GAP1-C*->

■ **GCA_900105705.1; Streptomyces griseus; counts= 1; multicellularity status: TRUE**

- GAP1-N2+GAP1-M+GAP1-C*->

■ **GCA_900104845.1; Paraburkholderia caballeronis; counts= 1; multicellularity status: FALSE**

- Calcineurin+GAPEAD1->GAP2->ZnR+DO-GTPase2.1->DO-GTPase1->GAP1-N1+GAP1-M+GAP1-C*->

■ **GCA_900105265.1; Streptomyces sp. 3213.3; counts= 1; multicellularity status: TRUE**

- TPR_containing_alpha_helical+Fibronectin-like_repeats->ZnR+ZnR+DO-GTPase2.2->GAP1-N2+GAP1-M+GAP1-C*->TM+TM+TM+TM+ZnR+ZnR+DO-GTPase2.2+TM->?->vWA(canonical)->PP2C->Pkinase+ZnR+beta-rich->

■ **GCA_900107975.1; Paenibacillus sp. UNC499MF; counts= 1; multicellularity status: TRUE**

- TM+vWA(canonical)+YtKA-like+TM+??->Tubulin+coiled_coil+??->Tubulin+coiled_coil+HTH?+??->TM+TM+TM+vWA(canonical)+TM+TM+TM+polar_tail->Fibronectin-like_repeats+??->ZnR+ZnR+DO-GTPase2.2+ZnR+ZnR->GAP1-N2+GAP1-M+GAP1-C+polar_tail+TM+Cadherin-like*->

■ **GCA_900108175.1; Actinomadura echinospora; counts= 1; multicellularity status: TRUE**

- GrpE->HSP70->TPR_containing_alpha_helical+Fibronectin-like_repeats->ZnR+ZnR+DO-GTPase2.2->GAP1-N2+GAP1-M+GAP1-C*->TM+TM+TM+TM+ZnR+ZnR+DO-GTPase2.2+TM->

■ **GCA_900108335.1; Nonomuraea solani; counts= 1; multicellularity status: TRUE**

- GrpE->HSP70->TPR_containing_alpha_helical+Fibronectin-like_repeats->ZnR+ZnR+DO-GTPase2.2->GAP1-N2+GAP1-M+GAP1-C*->TM+TM+TM+TM+ZnR+ZnR+DO-GTPase2.2+TM->

■ **GCA_900109515.1; Paenibacillus sp. OK003; counts= 1; multicellularity status: TRUE**

- TM+vWA(canonical)+YtKA-like+TM+??->Tubulin+coiled_coil+??->Tubulin+coiled_coil+HTH?+??->TM+TM+TM+vWA(canonical)+TM+TM+TM+polar_tail->Fibronectin-like_repeats+??->ZnR+ZnR+DO-GTPase2.2+ZnR+ZnR->GAP1-N2+GAP1-M+GAP1-C+polar_tail+TM+beta-rich*->

■ **GCA_900110055.1; Paenibacillus sp. OK076; counts= 1; multicellularity status: TRUE**

- TM+vWA(canonical)+YtKA-like+TM+??->Tubulin+coiled_coil+??->Tubulin+coiled_coil+HTH?+??->TM+TM+TM+vWA(canonical)+TM+TM+TM+polar_tail->Fibronectin-like_repeats+??->ZnR+ZnR+DO-GTPase2.2+ZnR+ZnR->GAP1-N2+GAP1-M+GAP1-C+polar_tail+TM+beta-rich*->

■ **GCA_900110775.1; Litorimicrobium taeanense; counts= 1; multicellularity status: NA**

- Calcineurin+GAPEAD1->GAP2->ZnR+DO-GTPase2.1->DO-GTPase1->GAP1-N1+GAP1-M+GAP1-C*->

■ **GCA_900111175.1; Actinokineospora terrae; counts= 1; multicellularity status: TRUE**

- CASPASE+TPR_region->?->GAP1-N2+GAP1-M*->

■ **GCA_900111455.1; Geodermatophilus poikilotrophus; counts= 1; multicellularity status: TRUE**

- Pkinase+TM->TM->ZnR+ZnR+DO-GTPase2.2->GAP1-N2+GAP1-M+GAP1-C*->?->||<-?<-?||?->Pkinase+low_complexity->

■ **GCA_900111565.1; Paenibacillus sp. NFR01; counts= 1; multicellularity status: TRUE**

- TM+vWA(canonical)+YtKA-like+TM+??->Tubulin+coiled_coil+??->Tubulin+coiled_coil+HTH?+??->TM+TM+TM+vWA(canonical)+TM+TM+TM+polar_tail->Fibronectin-like_repeats+??->ZnR+ZnR+DO-GTPase2.2+ZnR+ZnR->GAP1-N2+GAP1-M+GAP1-C+polar_tail+TM+beta-rich*->

■ **GCA_900112015.1; Azotobacter beijerinckii; counts= 1; multicellularity status: FALSE**

- Calcineurin+GAPEAD1->GAP2->ZnR+DO-GTPase2.1->DO-GTPase1->GAP1-N1+GAP1-M+GAP1-C*->SLATT->

■ **GCA_900112815.1; Blastococcus sp. DSM 46838; counts= 1; multicellularity status: TRUE**

- Calcineurin+GAPEAD1->GAP2->DO-GTPase2.1->DO-GTPase1->GAP1-N1+GAP1-M+GAP1-C*->

■ **GCA_900114385.1; Geodermatophilus ruber; counts= 1; multicellularity status: TRUE**

- Pkinase+TM->TM->ZnR+ZnR+DO-GTPase2.2->GAP1-N2+GAP1-M+GAP1-C*->

■ **GCA_900115095.1; Actinomadura madurae; counts= 1; multicellularity status: TRUE**

- GrpE->HSP70->TPR_containing_alpha_helical+Fibronectin-like_repeats->ZnR+ZnR+DO-GTPase2.2->GAP1-N2+GAP1-M+GAP1-C*->TM+TM+TM+TM+ZnR+ZnR+DO-GTPase2.2+TM->

■ **GCA_900116845.1; Pseudomonas sp. OV546; counts= 1; multicellularity status: FALSE**

- Calcineurin+GAPEAD1->GAP2->ZnR+DO-GTPase2.1->DO-GTPase1->GAP1-N1+GAP1-M+GAP1-C*->

■ **GCA_900130065.1; Sporobacter termitidis DSM 10068; counts= 1; multicellularity status: FALSE**

- Calcineurin+GAPEAD1->GAP2->DO-GTPase2.1->DO-GTPase1->GAP1-N1+GAP1-M+GAP1-C*->

■ **GCA_900143215.1; Geodermatophilus obscurus; counts= 1; multicellularity status: TRUE**

- Pkinase+TM->TM->ZnR+ZnR+DO-GTPase2.2->GAP1-N2+GAP1-M+GAP1-C*->?->||<-?<-?||?->Pkinase+low_complexity->

■ **GCA_900134035.1; Mycobacteroides abscessus subsp. abscessus; counts= 1; multicellularity status: FALSE**

- HSP70->TPR_containing_alpha_helical+Fibronectin-like_repeats->ZnR+ZnR+DO-GTPase2.2->GAP1-N2+GAP1-M+GAP1-C*->

■ **GCA_900083515.1; Cuniculiplasma divulgatum; counts= 1; multicellularity status: TRUE**

- TM+TM+TM+TM+TM+TM+DO-GTPase2.1->GAP1-N2+GAP1-M+GAP1-C*->??->TM_region+DO-GTPase1->

■ **GCA_900143625.1; Nocardiopsis sp. JB363; counts= 1; multicellularity status: TRUE**

- TPR_containing_alpha_helical+Fibronectin-like_repeats->ZnR+ZnR+DO-GTPase2.2->GAP1-N2+GAP1-M+GAP1-C*->TM+TM+TM+TM+ZnR+ZnR+DO-GTPase2.2->

■ **GCA_900156185.1; Pseudomonas putida; counts= 1; multicellularity status: FALSE**

- Calcineurin+GAPEAD1->GAP2->ZnR+DO-GTPase2.1->DO-GTPase1->GAP1-N1+GAP1-M+GAP1-C*->

■ **GCA_900163685.1; Corynebacterium glutamicum; counts= 1; multicellularity status: FALSE**

- TM+TM+TM->TM+vWA(canonical)+Fibronectin-like_repeats+TM+beta-rich+lc_tail->Tubulin+alpha_helical+??+??->GAP_unknown1+TM+TM+GAP_unknown2->HRDC-like?+Fibronectin-like_repeats->GAP1-N2+GAP1-M+GAP1-C*->beta-rich+vWA(canonical)->??+DO-GTPase2.2->

■ **GCA_900132645.1; Mycobacteroides abscessus subsp. abscessus; counts= 1; multicellularity status: FALSE**

- GAP1-N2+GAP1-M+GAP1-C+polar_tail+TM+beta-rich*->

■ **GCA_900174015.1; Klebsiella pneumoniae; counts= 1; multicellularity status: FALSE**

- GAPEAD1->GAP2->DO-GTPase1->GAP1-N1+GAP1-M+GAP1-C*->

■ **GCA_900177425.1; Paenibacillus uliginis N3/975; counts= 1; multicellularity status: TRUE**

- TM+vWA(canonical)+YtKA-like+TM+??->Tubulin+coiled_coil+??->Tubulin+coiled_coil+HTH?+??->TM+TM+TM+vWA(canonical)+TM+TM+TM+polar_tail->Fibronectin-like_repeats+??->ZnR+ZnR+DO-GTPase2.2+ZnR+ZnR->GAP1-N2+GAP1-M+GAP1-C+polar_tail+TM+beta-rich*->

■ **GCA_900188375.1; Geodermatophilus pulveris; counts= 1; multicellularity status: TRUE**

- Pkinase+TM->TM->ZnR+ZnR+DO-GTPase2.2->GAP1-N2+GAP1-M+GAP1-C*->Pkinase+low_complexity->

■ **GCA_900221015.1; Ornithinimicrobium pekingense; counts= 1; multicellularity status: FALSE**

- Pkinase+TM_region->ZnR+ZnR+DO-GTPase2.2->GAP1-N2+GAP1-M+TM+GAP1-C+TM*->

■ **GCA_900215445.1; Serratia sp. JKS296; counts= 1; multicellularity status: FALSE**

- GAP2->ZnR+DO-GTPase2.1->DO-GTPase1->GAP1-N1+GAP1-M+GAP1-C+EAD1*->CASPASE+EAD1->

■ **GCA_000216135.3; Leptospira interrogans; counts= 1; multicellularity status: FALSE**

- GAP2->ZnR+DO-GTPase2.1->DO-GTPase1->GAP1-N1+GAP1-M+GAP1-C+EAD1*->PNP_UDP_1+EAD1->

■ **GCA_000306495.2; Leptospira interrogans; counts= 1; multicellularity status: FALSE**

- GAP2->ZnR+DO-GTPase2.1->DO-GTPase1->GAP1-N1+GAP1-M+GAP1-C+EAD1*->PNP_UDP_1+EAD1->

■ **GCA_000164015.3; Pseudomonas savastanoi; counts= 1; multicellularity status: FALSE**

- GAP2->DO-GTPase2.1->DO-GTPase1->GAP1-N1+GAP1-M+GAP1-C+EAD1*->CASPASE(frag)->PSE->CASPASE+EAD1->

■ **GCA_000153105.1; Blastopirellula marina; counts= 1; multicellularity status: TRUE**

- FGS-fold_domain->ZnR+DO-GTPase2.1->GAP1-N2+GAP1-M+GAP1-C*->

■ **GCA_000219535.3; Brevibacillus laterosporus; counts= 1; multicellularity status: FALSE**

- TM+vWA(canonical)+YtKA-like+TM+??->Tubulin+coiled_coil+??->Tubulin+coiled_coil+HTH?+??->TM+TM+TM+vWA(canonical)+TM+TM+TM+polar_tail->Fibronectin-like_repeats+??->ZnR+ZnR+DO-GTPase2.2+ZnR+ZnR->GAP1-N2+GAP1-M+GAP1-C+polar_tail+TM+beta-rich*->

■ **GCA_000237005.2; Brevibacillus laterosporus; counts= 1; multicellularity status: FALSE**

- TM+vWA(canonical)+YtKA-like+TM+??->Tubulin+coiled_coil+??->Tubulin+coiled_coil+HTH?+??->TM+TM+TM+vWA(canonical)+TM+TM+TM+polar_tail->Fibronectin-like_repeats+??->ZnR+ZnR+DO-GTPase2.2+ZnR+ZnR->GAP1-N2+GAP1-M+GAP1-C+polar_tail+TM+beta-rich*->

■ **GCA_000353565.1; Brevibacillus borstelensis; counts= 1; multicellularity status: FALSE**

- TM+vWA(canonical)+YtKA-like+TM+??->Tubulin+coiled_coil+??->Tubulin+coiled_coil+HTH?+??->TM+TM+TM+vWA(canonical)+TM+TM+TM+polar_tail->Fibronectin-like_repeats+??->ZnR+ZnR+DO-GTPase2.2+ZnR+ZnR->GAP1-N2+GAP1-M+GAP1-C+polar_tail+TM+beta-rich*->

■ **GCA_000271785.1; Rhizobium leguminosarum; counts= 1; multicellularity status: TRUE**

- Calcineurin+GAPEAD1->GAP2->DO-GTPase2.1->DO-GTPase1->GAP1-N1+GAP1-M+GAP1-C*->

■ **GCA_002243555.1; Corynebacterium; counts= 1; multicellularity status: FALSE**

- polar_leader+TM+TM+TM->TM+vWA(canonical)+Fibronectin-like_repeats+TM+beta-rich+lc_tail->Tubulin+alpha_helical+??+??->GAP_unknown1+TM+TM+GAP_unknown2->HRDC-like?+Fibronectin-like_repeats->GAP1-N2+GAP1-M+GAP1-C*->beta-rich+vWA(canonical)->??+DO-GTPase2.2->

■ **GCA_000177175.2; Streptomyces sp. ACT-1; counts= 1; multicellularity status: TRUE**

- GAP1-N2+GAP1-M+GAP1-C*->

■ **GCA_000364245.1; Clostridium sp. ASF502; counts= 1; multicellularity status: NA**

- HSP70->HSP70+vWA(canonical)->DnaJ+DnaJ+ZnR+ZnR+coiled_coil+Fibronectin-like_repeats->ZnR+ZnR+DO-GTPase2.2->GAP1-N2+GAP1-M+GAP1-C*->?->?->TM+TM+TM+TM+ZnR+ZnR+DO-GTPase2.2+TPR_region->

■ **GCA_000738065.1; Bacteroides; counts= 1; multicellularity status: FALSE**

- Calcineurin+GAPEAD1->?->DO-GTPase2.1->DO-GTPase1->GAP1-N1+GAP1-M+GAP1-C*->

■ **GCA_000350485.1; [Clostridium] termitidis; counts= 1; multicellularity status: FALSE**

- Calcineurin+GAPEAD1->GAP2->DO-GTPase2.1->DO-GTPase1->GAP1-N1+GAP1-M+GAP1-C*->

■ **GCA_000368665.1; Acinetobacter junii; counts= 1; multicellularity status: FALSE**

- GAP2->ZnR+DO-GTPase2.1->DO-GTPase1->GAP1-N1+GAP1-M+GAP1-C+EAD1*->CASPASE+EAD1->

■ **GCA_000342125.1; Streptomyces mobaraensis; counts= 1; multicellularity status: TRUE**

- GAP1-N2+GAP1-M+GAP1-C*->

■ **GCA_001896005.1; Acinetobacter; counts= 1; multicellularity status: FALSE**

- PNP_UDP_1+EAD1->GAP2->ZnR+DO-GTPase2.1->DO-GTPase1->GAP1-N1+GAP1-M+GAP1-C+EAD1*->

■ **GCA_000241345.2; Gordonia amarae; counts= 1; multicellularity status: FALSE**

- polar_leader+TM+TM+TM->TM+vWA(canonical)+Fibronectin-like_repeats+TM+beta-rich+lc_tail->Tubulin+alpha_helical+??+??->GAP_unknown1+TM+TM+GAP_unknown2->HRDC-like?+Fibronectin-like_repeats->GAP1-N2+GAP1-M+GAP1-C*->beta-rich+vWA(canonical)->??+DO-GTPase2.2->

■ **GCA_000599545.1; Rhodococcus opacus; counts= 1; multicellularity status: TRUE**

- polar_leader+TM+TM+TM->TM+vWA(canonical)+Fibronectin-like_repeats+TM+beta-rich+lc_tail->Tubulin+alpha_helical+??+??->GAP_unknown1+TM+TM+GAP_unknown2->HRDC-like?+Fibronectin-like_repeats->GAP1-N2+GAP1-M+GAP1-C*->beta-rich+vWA(canonical)->??+DO-GTPase2.2->

■ **GCA_000369745.1; Acinetobacter; counts= 1; multicellularity status: FALSE**

- PNP_UDP_1+EAD1->GAP2->ZnR+DO-GTPase2.1->DO-GTPase1->GAP1-N1+GAP1-M+GAP1-C+EAD1*->

■ **GCA_000159595.1; Corynebacterium glucuronolyticum; counts= 1; multicellularity status: FALSE**

- GrpE->HSP70->TPR_containing_alpha_helical+Fibronectin-like_repeats->ZnR+ZnR+DO-GTPase2.2->GAP1-N2+GAP1-M+GAP1-C*->TM+TM+TM+TM+ZnR+ZnR+DO-GTPase2.2->

■ **GCA_000164155.2; Rhodococcus hoagii; counts= 1; multicellularity status: TRUE**

- polar_leader+TM+TM+TM->TM+vWA(canonical)+Fibronectin-like_repeats+TM+beta-rich+lc_tail->Tubulin+alpha_helical+??+??->GAP_unknown1+TM+TM+GAP_unknown2->HRDC-like?+Fibronectin-like_repeats->GAP1-N2+GAP1-M+GAP1-C*->beta-rich+vWA(canonical)->??+DO-GTPase2.2->

■ **GCA_000158635.1; Corynebacterium matruchotii; counts= 1; multicellularity status: FALSE**

- polar_leader+TM+TM+TM->TM+vWA(canonical)+Fibronectin-like_repeats+TM+beta-rich+lc_tail->Tubulin+alpha_helical+??+??->GAP_unknown1+TM+TM+GAP_unknown2->HRDC-like?+Fibronectin-like_repeats->GAP1-N2+GAP1-M+GAP1-C*->beta-rich+vWA(canonical)->??+DO-GTPase2.2->polar_leader+TM+TM+TM->

■ **GCA_000175375.1; Corynebacterium matruchotii; counts= 1; multicellularity status: FALSE**

- polar_leader+TM+TM+TM->TM+vWA(canonical)+Fibronectin-like_repeats+TM+beta-rich+lc_tail->Tubulin+alpha_helical+??+??->GAP_unknown1+TM+TM+GAP_unknown2->HRDC-like?+Fibronectin-like_repeats->GAP1-N2+GAP1-M+GAP1-C*->beta-rich+vWA(canonical)->??+DO-GTPase2.2->polar_leader+TM+TM+TM->

■ **GCA_000159135.1; Corynebacterium striatum; counts= 1; multicellularity status: FALSE**

- polar_leader+TM+TM+TM->TM+vWA(canonical)+Fibronectin-like_repeats+TM+beta-rich+lc_tail->Tubulin+alpha_helical+??+??->GAP_unknown1+TM+TM+GAP_unknown2->HRDC-like?+Fibronectin-like_repeats->GAP1-N2+GAP1-M+GAP1-C*->beta-rich+vWA(canonical)->??+DO-GTPase2.2->polar_leader+TM+TM+TM->

■ **GCA_001404375.1; Bacteroides; counts= 1; multicellularity status: FALSE**

- Calcineurin+GAPEAD1->?->DO-GTPase2.1->DO-GTPase1->GAP1-N1+GAP1-M+GAP1-C*->

■ **GCA_000175235.1; Acidovorax delafieldii; counts= 1; multicellularity status: FALSE**

- Calcineurin+GAPEAD1->GAP2->ZnR+DO-GTPase2.1->DO-GTPase1->GAP1-N1+GAP1-M+GAP1-C*->

■ **GCA_000328345.1; Brevibacillus agri; counts= 1; multicellularity status: FALSE**

- Tubulin+coiled_coil+??->PSE->TM+TM+TM+vWA(canonical)+TM+TM+TM+polar_tail->Fibronectin-like_repeats+??->ZnR+ZnR+DO-GTPase2.2+ZnR+ZnR->GAP1-N2+GAP1-M+GAP1-C+polar_tail+TM+beta-rich*->

■ **GCA_000178455.1; Brevibacterium mcbrellneri; counts= 1; multicellularity status: FALSE**

- TM+vWA(canonical)+Fibronectin-like_repeats+TM+beta-rich+lc_tail->Tubulin+alpha_helical+??+??->GAP_unknown1+TM+TM+GAP_unknown2->HRDC-like?+Fibronectin-like_repeats->GAP1-N2+GAP1-M+GAP1-C*->?->??+DO-GTPase2.2->?->||<-?<-?<-TM+TM+vWA(canonical)+TM<-TM+TM+vWA(canonical)+TM

■ **GCA_000239695.1; Actinomyces graevenitzii; counts= 1; multicellularity status: TRUE**

- GrpE->HSP70->TPR_containing_alpha_helical+Fibronectin-like_repeats->ZnR+ZnR+DO-GTPase2.2->GAP1-N2+GAP1-M+GAP1-C*->TM+TM+TM+TM+ZnR+ZnR+DO-GTPase2.2->

■ **GCA_000156455.1; Streptomyces filamentosus; counts= 1; multicellularity status: TRUE**

- *

■ **GCF_000243415.2; Mycolicibacterium tusciae; counts= 1; multicellularity status: FALSE**

- GrpE->HSP70->TPR_containing_alpha_helical+Fibronectin-like_repeats->ZnR+ZnR+DO-GTPase2.2->GAP1-N2+GAP1-M+GAP1-C*->TM+TM+TM+TM+ZnR+ZnR+DO-GTPase2.2->?->?->?->?->HSP70->vWA(canonical)->

■ **GCA_000315235.1; Paenibacillus popilliae; counts= 1; multicellularity status: TRUE**

- TM+vWA(canonical)+YtKA-like+TM+??->Tubulin+coiled_coil+??->Tubulin+coiled_coil+HTH?+??->TM+TM+TM+vWA(canonical)+TM+TM+TM+polar_tail->Fibronectin-like_repeats+??->ZnR+ZnR+DO-GTPase2.2+ZnR+ZnR->GAP1-N2+GAP1-M+GAP1-C+polar_tail+TM+beta-rich*->

■ **GCA_000297155.2; Streptomyces tsukubensis; counts= 1; multicellularity status: TRUE**

- ZnR+ZnR+DO-GTPase2.2->GAP1-N2+GAP1-M+GAP1-C*->TM+TM+TM+TM+ZnR+ZnR+DO-GTPase2.2->

■ **GCA_000177675.1; Frankia sp. EUN1f; counts= 1; multicellularity status: TRUE**

- GAP1-N2+GAP1-M+GAP1-C+ZnR+DO-GTPase2*->

■ **GCA_000159035.1; Actinomyces urogenitalis; counts= 1; multicellularity status: TRUE**

- polar_leader+TM+TM+TM->TM+vWA(canonical)+Fibronectin-like_repeats+TM+beta-rich+lc_tail->Tubulin+alpha_helical+??+??->GAP_unknown1+TM+TM+GAP_unknown2->HRDC-like?+Fibronectin-like_repeats->GAP1-N2+GAP1-M+GAP1-C*->?->??+DO-GTPase2.2->

■ **GCA_000236965.2; Rhodococcus pyridinivorans; counts= 1; multicellularity status: TRUE**

- polar_leader+TM+TM+TM->TM+vWA(canonical)+Fibronectin-like_repeats+TM+beta-rich+lc_tail->Tubulin+alpha_helical+??+??->PSE->HRDC-like?+Fibronectin-like_repeats->GAP1-N2+GAP1-M+GAP1-C*->beta-rich+vWA(canonical)->??+DO-GTPase2.2->

■ **GCA_000190755.3; Methylobacter tundripaludum; counts= 1; multicellularity status: TRUE**

- PNP_UDP_1+EAD1->GAP2->||<-?||GAP2->ZnR+DO-GTPase2.1->DO-GTPase1->GAP1-N1+GAP1-M+GAP1-C+EAD1*->

■ **GCA_000350545.1; Desulfotignum phosphitoxidans; counts= 1; multicellularity status: FALSE**

- ??+PNP_UDP_1+EAD1->GAP2->ZnR+DO-GTPase2.1->DO-GTPase1->GAP1-N1+GAP1-M+GAP1-C*->?->EAD1->

■ **GCA_000307875.1; Bacillus bataviensis; counts= 1; multicellularity status: FALSE**

- TM+vWA(canonical)+YtKA-like+TM+??->Tubulin+coiled_coil+??->Tubulin+coiled_coil+HTH?+??->TM+TM+TM+vWA(canonical)+TM+TM+TM+polar_tail->Fibronectin-like_repeats+??->ZnR+ZnR+DO-GTPase2.2+ZnR+ZnR->GAP1-N2+GAP1-M+GAP1-C+polar_tail+TM+beta-rich*->

■ **GCA_000164135.1; Mycobacterium; counts= 1; multicellularity status: FALSE**

- TM+DUF4190+TM->TM+DUF4190+TM->polar_leader+TM+TM+TM->TM+vWA(canonical)+Fibronectin-like_repeats+TM+beta-rich+lc_tail->Tubulin+alpha_helical+??+??->GAP_unknown1+TM+TM+GAP_unknown2->HRDC-like?+Fibronectin-like_repeats->GAP1-N2+GAP1-M+GAP1-C(frag)*->beta-rich+vWA(canonical)->??+DO-GTPase2.2->

■ **GCA_002966555.1; Pseudomonas syringae group genomosp. 3; counts= 1; multicellularity status: FALSE**

- Calcineurin+GAPEAD1->GAP2->ZnR+DO-GTPase2.1->GAP1-N1+GAP1-M+GAP1-C*->

■ **GCA_000241305.2; Gordonia effusa; counts= 1; multicellularity status: FALSE**

- polar_leader+TM+TM+TM->TM+vWA(canonical)+Fibronectin-like_repeats+TM+beta-rich+lc_tail->Tubulin+alpha_helical+??+??->GAP_unknown1+TM+TM+GAP_unknown2->HRDC-like?+Fibronectin-like_repeats->GAP1-N2+GAP1-M+GAP1-C*->beta-rich+vWA(canonical)->??+DO-GTPase2.2->

■ **GCA_000241265.2; Gordonia araii; counts= 1; multicellularity status: FALSE**

- polar_leader+TM+TM+TM->TM+vWA(canonical)+Fibronectin-like_repeats+TM+beta-rich+lc_tail->Tubulin+alpha_helical+??+??->GAP_unknown1+TM+TM+GAP_unknown2->HRDC-like?+Fibronectin-like_repeats->GAP1-N2+GAP1-M+GAP1-C*->beta-rich+vWA(canonical)->??+DO-GTPase2.2->

■ **GCA_000245715.1; Paenibacillus sp. Aloe-11; counts= 1; multicellularity status: TRUE**

- Tubulin+coiled_coil+??->PSE->TM+TM+TM+vWA(canonical)+TM+TM+TM+polar_tail->Fibronectin-like_repeats+??->ZnR+ZnR+DO-GTPase2.2+ZnR+ZnR->GAP1-N2+GAP1-M+GAP1-C+polar_tail+TM+beta-rich*->

■ **GCA_000282075.1; Brevibacillus sp. BC25; counts= 1; multicellularity status: FALSE**

- TM+vWA(canonical)+YtKA-like+TM+??->Tubulin+coiled_coil+??->Tubulin+coiled_coil+HTH?+??->TM+TM+TM+vWA(canonical)+TM+TM+TM+polar_tail->Fibronectin-like_repeats+??->ZnR+ZnR+DO-GTPase2.2+ZnR+ZnR->GAP1-N2+GAP1-M+GAP1-C+polar_tail+TM+beta-rich*->

■ **GCA_000282095.2; Rhizobium sp. CF080; counts= 1; multicellularity status: TRUE**

- GAP2->PSE->DO-GTPase1->GAP1-N1+GAP1-M+GAP1-C+EAD1*->CASPASE+EAD1->

■ **GCA_000222105.4; Mycobacterium colombiense; counts= 1; multicellularity status: FALSE**

- TM+DUF4190+TM->TM+DUF4190+TM->polar_leader+TM+TM+TM->TM+vWA(canonical)+Fibronectin-like_repeats+TM+beta-rich+lc_tail->Tubulin+alpha_helical+??+??->PSE->PSE->GAP1-N2+GAP1-M+GAP1-C*->beta-rich+vWA(canonical)->??+DO-GTPase2.2->TM+DUF4190+TM+PknH_C->TM+DUF4190+TM+PknH_C->?->?->TM+DUF4190+TM+PknH_C->

■ **GCA_000282015.1; Brevibacillus sp. CF112; counts= 1; multicellularity status: FALSE**

- TM+vWA(canonical)+YtKA-like+TM+??->Tubulin+coiled_coil+??->Tubulin+coiled_coil+HTH?+??->TM+TM+TM+vWA(canonical)+TM+TM+TM+polar_tail->Fibronectin-like_repeats+??->ZnR+ZnR+DO-GTPase2.2+ZnR+ZnR->GAP1-N2+GAP1-M+GAP1-C+polar_tail+TM+beta-rich*->

■ **GCA_000213055.1; Streptomyces sp. Tu6071; counts= 1; multicellularity status: TRUE**

- GAP1-N2+GAP1-M+GAP1-C*->

■ **GCA_000174195.1; Roseburia inulinivorans; counts= 1; multicellularity status: FALSE**

- Calcineurin+GAPEAD1->GAP2->DO-GTPase2.1->DO-GTPase1->GAP1-N1+GAP1-M+GAP1-C*->

■ **GCA_000155695.1; Verrucomicrobiae bacterium DG1235; counts= 1; multicellularity status: NA**

- FHA-like?+ZnR+ZnR+DO-GTPase2.2->GAP1-N2+GAP1-M+TM+GAP1-C*->

■ **GCA_000297435.1; Microcystis sp. T1-4; counts= 1; multicellularity status: TRUE**

- ZnR+ZnR+DO-GTPase2.2->GAP1-N2+GAP1-M+GAP1-C*->TM+TM+TM+TM+TM+ZnR+ZnR+DO-GTPase2.2+TM+beta-sandwich+NTF-like->GrpE->

■ **GCA_000344135.1; Gordonia malaquae; counts= 1; multicellularity status: FALSE**

- polar_leader+TM+TM+TM->TM+vWA(canonical)+Fibronectin-like_repeats+TM+beta-rich+lc_tail->Tubulin+alpha_helical+??+??->GAP_unknown1+TM+TM+GAP_unknown2->HRDC-like?+Fibronectin-like_repeats->GAP1-N2+GAP1-M+GAP1-C*->beta-rich+vWA(canonical)->??+DO-GTPase2.2->

■ **GCA_000299175.2; Streptomyces; counts= 1; multicellularity status: TRUE**

- GAP1-N2+GAP1-M+GAP1-C*->

■ **GCA_000333955.1; Rhodococcus sp. AW25M09; counts= 1; multicellularity status: TRUE**

- polar_leader+TM+TM+TM->TM+vWA(canonical)+Fibronectin-like_repeats+TM+beta-rich+lc_tail->Tubulin+alpha_helical+??+??->GAP_unknown1+TM+TM+GAP_unknown2->HRDC-like?+Fibronectin-like_repeats->GAP1-N2+GAP1-M+GAP1-C*->beta-rich+vWA(canonical)->??+DO-GTPase2.2->

■ **GCA_000296275.1; Actinomyces massiliensis; counts= 1; multicellularity status: TRUE**

- polar_leader+TM+TM+TM->TM+vWA(canonical)+Fibronectin-like_repeats+TM+beta-rich+lc_tail->Tubulin+alpha_helical+??+??->GAP_unknown1+TM+TM+GAP_unknown2->HRDC-like?+Fibronectin-like_repeats->GAP1-N2+GAP1-M+GAP1-C*->?->??+DO-GTPase2.2->

■ **GCA_000230835.1; Clostridium sp. DL-VIII; counts= 1; multicellularity status: NA**

- Calcineurin+GAPEAD1->GAP2->DO-GTPase2.1->DO-GTPase1->GAP1-N1+GAP1-M+GAP1-C*->

- **GCA_000296815.2; Fibrisoma limi; counts= 1; multicellularity status: TRUE**
  - GAP2->DO-GTPase2.1->DO-GTPase1->GAP1-N1+GAP1-M+GAP1-C+EAD1*->PNP_UDP_1+EAD1->
- **GCA_000280725.1; Rhodococcus sp. JVH1; counts= 1; multicellularity status: TRUE**
  - polar_leader+TM+TM+TM->TM+vWA(canonical)+Fibronectin-like_repeats+TM+beta-rich+lc_tail->Tubulin+alpha_helical+??+??->GAP_unknown1+TM+TM+GAP_unknown2->HRDC-like?+Fibronectin-like_repeats->GAP1-N2+GAP1-M+GAP1-C*->beta-rich+vWA(canonical)->??+DO-GTPase2.2->
- **GCA_000314635.1; Acinetobacter baumannii; counts= 1; multicellularity status: FALSE**
  - GAP2->ZnR+DO-GTPase2.1->DO-GTPase1->GAP1-N1+GAP1-M+GAP1-C+EAD1*->CASPASE+EAD1->
- **GCA_000238095.2; Peptoanaerobacter stomatis; counts= 1; multicellularity status: FALSE**
  - TM+TM+TM+TM+ZnR+ZnR+DO-GTPase2.2->GrpE->HSP70->HSP70+vWA(canonical)->DnaJ+DnaJ+ZnR+ZnR+coiled_coil+Fibronectin-like_repeats->ZnR+ZnR+DO-GTPase2.2->GAP1-N2+GAP1-M+GAP1-C*->?->?->?->TM+TM+TM+TM+ZnR+ZnR+DO-GTPase2.2->
- **GCA_000293035.1; Peptoanaerobacter stomatis; counts= 1; multicellularity status: FALSE**
  - HSP70->HSP70+vWA(canonical)->DnaJ+DnaJ+ZnR+ZnR+coiled_coil+Fibronectin-like_repeats->ZnR+ZnR+DO-GTPase2.2->GAP1-N2+GAP1-M+GAP1-C*->?->?->TM+TM+TM+TM+ZnR+ZnR+DO-GTPase2.2->
- **GCA_000204455.2; Paenibacillus sp. HGF5; counts= 1; multicellularity status: TRUE**
  - TM+vWA(canonical)+YtKA-like+TM+??->Tubulin+coiled_coil+??->Tubulin+coiled_coil+HTH?+??->TM+TM+TM+vWA(canonical)+TM+TM+TM+polar_tail->Fibronectin-like_repeats+??->ZnR+ZnR+DO-GTPase2.2+ZnR+ZnR->GAP1-N2+GAP1-M+GAP1-C+polar_tail+TM+beta-rich*->
- **GCA_000278725.1; Actinomyces sp. ICM47; counts= 1; multicellularity status: NA**
  - GrpE->HSP70->TPR_containing_alpha_helical+Fibronectin-like_repeats->ZnR+ZnR+DO-GTPase2.2->GAP1-N2+GAP1-M+GAP1-C*->TM+TM+TM+TM+ZnR+ZnR+DO-GTPase2.2->??->?->?->?->HSP70->vWA(canonical)->
- **GCA_000214295.2; Paenibacillus; counts= 1; multicellularity status: TRUE**
  - TM+vWA(canonical)+YtKA-like+TM+??->Tubulin+coiled_coil+??->Tubulin+coiled_coil+HTH?+??->TM+TM+TM+vWA(canonical)+TM+TM+TM+polar_tail->Fibronectin-like_repeats+??->ZnR+ZnR+DO-GTPase2.2+ZnR+ZnR->GAP1-N2+GAP1-M+GAP1-C+polar_tail+TM+Cadherin-like*->
- **GCA_000262465.1; Frankia sp. QA3; counts= 1; multicellularity status: TRUE**
  - GrpE->HSP70->TPR_containing_alpha_helical+Fibronectin-like_repeats->ZnR+ZnR+DO-GTPase2.2->GAP1-N2+GAP1-M+GAP1-C*->TM+TM+TM+TM+ZnR+ZnR+DO-GTPase2.2->??->HSP70->
- **GCA_000220835.1; Actinomyces sp. oral taxon 448; counts= 1; multicellularity status: NA**
  - polar_leader+TM+TM+TM->TM+vWA(canonical)+Fibronectin-like_repeats+TM+beta-rich+lc_tail->Tubulin+alpha_helical+??+??->PSE->HRDC-like?+Fibronectin-like_repeats->GAP1-N2+GAP1-M+GAP1-C*->?->??+DO-GTPase2.2->
- **GCA_000022905.1; Corynebacterium aurimucosum; counts= 1; multicellularity status: FALSE**
  - polar_leader+TM+TM+TM->TM+vWA(canonical)+Fibronectin-like_repeats+TM+beta-rich+lc_tail->Tubulin+alpha_helical+??+??->GAP_unknown1+TM+TM+GAP_unknown2->HRDC-like?+Fibronectin-like_repeats->GAP1-N2+GAP1-M+GAP1-C*->beta-rich+vWA(canonical)->??+DO-GTPase2.2->polar_leader+TM+TM+TM->
- **GCF_000177455.1; Pseudomonas syringae group genomosp. 3; counts= 1; multicellularity status: FALSE**
  - Calcineurin+GAPEAD1->GAP2->ZnR+DO-GTPase2.1->GAP1-N1+GAP1-M+GAP1-C*->
- **GCF_000179195.1; Streptomyces sp. SA3_actG; counts= 1; multicellularity status: TRUE**
  - GAP1-N2+GAP1-M+GAP1-C*->
- **GCF_000236805.1; Paenibacillus peoriae; counts= 1; multicellularity status: TRUE**
  - TM+vWA(canonical)+YtKA-like+TM+??->Tubulin+coiled_coil+??->Tubulin+coiled_coil+HTH?+??->TM+TM+TM+vWA(canonical)+TM+TM+TM+polar_tail->Fibronectin-like_repeats+??->ZnR+ZnR+DO-GTPase2.2+ZnR+ZnR->GAP1-N2+GAP1-M+GAP1-C+polar_tail+TM+beta-rich*->
- **GCF_000213315.1; Paenibacillus elgii; counts= 1; multicellularity status: TRUE**

- TM+vWA(canonical)+YtKA-like+TM+??->Tubulin+coiled_coil+??->Tubulin+coiled_coil+HTH?+??->?->TM+TM+TM+vWA(canonical)+TM+TM+TM+polar_tail->Fibronectin-like_repeats+??->ZnR+ZnR+DO-GTPase2.2+ZnR+ZnR->GAP1-N2+GAP1-M+GAP1-C+polar_tail+TM+Cadherin-like*->

■ **GCF_000255655.1; Schlesneria paludicola; counts= 1; multicellularity status: TRUE**

- ZnR+ZnR+DO-GTPase2.2->GAP1-N2+GAP1-M+TM+GAP1-C*->??+TM+??+beta-rich??->GYF+GYF+ZnR+TM+TM+TM->

■ **GCA_002846475.1; Saccharopolyspora spinosa; counts= 1; multicellularity status: TRUE**

- GrpE->HSP70->TPR_containing_alpha_helical+Fibronectin-like_repeats->ZnR+ZnR+DO-GTPase2.2->GAP1-N2+GAP1-M+GAP1-C*->TM+TM+TM+TM+ZnR+ZnR+DO-GTPase2.2->HSP70->vWA(canonical)->

■ **GCA_000364265.1; Parabacteroides; counts= 1; multicellularity status: FALSE**

- Calcineurin+GAPEAD1->?->DO-GTPase2.1->DO-GTPase1->GAP1-N1+GAP1-M+GAP1-C*->

■ **GCF_000011325.1; Corynebacterium glutamicum; counts= 1; multicellularity status: FALSE**

- polar_leader+TM+TM+TM->TM+vWA(canonical)+Fibronectin-like_repeats+TM+beta-rich+lc_tail->Tubulin+alpha_helical+??+??->GAP_unknown1+TM+TM+GAP_unknown2->HRDC-like?+Fibronectin-like_repeats->GAP1-N2+GAP1-M+GAP1-C*->beta-rich+vWA(canonical)->??+DO-GTPase2.2->

■ **GCF_000007805.1; Pseudomonas syringae group genomosp. 3; counts= 1; multicellularity status: FALSE**

- DO-GTPase2.1->DO-GTPase1->GAP1-N1+GAP1-M+GAP1-C*->

■ **GCA_000014165.1; Mycobacterium; counts= 1; multicellularity status: FALSE**

- HRDC-like?+Fibronectin-like_repeats->GAP1-N2+GAP1-M+GAP1-C*->??+DO-GTPase2.2->

■ **GCA_000014565.1; Rhodococcus jostii; counts= 1; multicellularity status: TRUE**

- polar_leader+TM+TM+TM->TM+vWA(canonical)+Fibronectin-like_repeats+TM+beta-rich+lc_tail->Tubulin+alpha_helical+??+??->GAP_unknown1+TM+TM+GAP_unknown2->HRDC-like?+Fibronectin-like_repeats->GAP1-N2+GAP1-M+GAP1-C*->beta-rich+vWA(canonical)->??+DO-GTPase2.2->

■ **GCA_000014925.1; Paenarthrobacter aurescens; counts= 1; multicellularity status: FALSE**

- PP2C->?->ZnR+ZnR+DO-GTPase2.2->GAP1-N2+GAP1-M+TM+GAP1-C+TM+TM*->

■ **GCA_000016005.1; Mycobacteriaceae; counts= 1; multicellularity status: FALSE**

- HRDC-like?+Fibronectin-like_repeats->GAP1-N2+GAP1-M+GAP1-C*->??+DO-GTPase2.2->

■ **GCA_000012825.1; Bacteroides vulgatus; counts= 1; multicellularity status: FALSE**

- Calcineurin+GAPEAD1->?->DO-GTPase2.1->DO-GTPase1->GAP1-N1+GAP1-M+GAP1-C*->

■ **GCA_000017645.1; Xanthobacter autotrophicus; counts= 1; multicellularity status: FALSE**

- GAP2->ZnR+DO-GTPase2.1->DO-GTPase1->GAP1-N1+GAP1-M+GAP1-C+EAD1*->CASPASE+EAD1->

■ **GCA_000020045.1; Paraburkholderia phymatum; counts= 1; multicellularity status: FALSE**

- Calcineurin+GAPEAD1->GAP2->ZnR+DO-GTPase2.1->DO-GTPase1->GAP1-N1+GAP1-M+GAP1-C*->

■ **GCA_000196495.1; Aliivibrio salmonicida; counts= 1; multicellularity status: FALSE**

- Calcineurin+GAPEAD1->GAP2->?->GAP2->ZnR+DO-GTPase2.1->DO-GTPase1->GAP1-N1+GAP1-M+GAP1-C*->

■ **GCA_000009225.1; Pseudomonas fluorescens; counts= 1; multicellularity status: FALSE**

- Calcineurin+GAPEAD1->GAP2->ZnR+DO-GTPase2.1->DO-GTPase1->GAP1-N1+GAP1-M+GAP1-C*->

■ **GCA_000024165.1; Candidatus Accumulibacter phosphatis; counts= 1; multicellularity status: FALSE**

- GAP1-N1+GAP1-M+GAP1-C*->

■ **GCA_000146875.2; Paenibacillus polymyxa; counts= 1; multicellularity status: TRUE**

- TM+vWA(canonical)+YtKA-like+TM+??->Tubulin+coiled_coil+??->Tubulin+coiled_coil+HTH?+??->TM+TM+TM+vWA(canonical)+TM+TM+TM+polar_tail->Fibronectin-like_repeats+??->ZnR+ZnR+DO-GTPase2.2+ZnR+ZnR->GAP1-N2+GAP1-M+GAP1-C+polar_tail+TM+beta-rich*->

■ **GCA_000164985.2; Paenibacillus polymyxa; counts= 1; multicellularity status: TRUE**

- TM+vWA(canonical)+YtKA-like+TM+??->Tubulin+coiled_coil+??->Tubulin+coiled_coil+HTH?+??->TM+TM+TM+vWA(canonical)+TM+TM+TM+polar_tail->Fibronectin-like_repeats+??->ZnR+ZnR+DO-GTPase2.2+ZnR+ZnR->GAP1-N2+GAP1-M+GAP1-C+polar_tail+TM+beta-rich*->

■ **GCA_000186345.1; Isosphaera pallida; counts= 1; multicellularity status: TRUE**

- FGS-fold_domain->ZnR+DO-GTPase2.1->GAP1-N2+GAP1-M*->

■ **GCA_000176855.2; Acidovorax avenae; counts= 1; multicellularity status: FALSE**

- GAP2->DO-GTPase2.1->DO-GTPase1->GAP1-N1+GAP1-M+GAP1-C+EAD1*->CASPASE+EAD1->

■ **GCA_000178835.2; Cellulosilyticum lentocellum; counts= 1; multicellularity status: FALSE**

- TM+vWA(canonical)+YtKA-like+TM+??->Tubulin+coiled_coil+??->Tubulin+coiled_coil+HTH?+??->TM+TM+TM+vWA(canonical)+TM+TM+TM+polar_tail->Fibronectin-like_repeats+??->ZnR+ZnR+DO-GTPase2.2+ZnR+ZnR->GAP1-N2+GAP1-M+GAP1-C+TM+polar_tail+beta-rich*->

■ **GCA_000218915.1; Paenibacillus mucilaginosus; counts= 1; multicellularity status: TRUE**

- TM+vWA(canonical)+YtKA-like+TM+??->Tubulin+coiled_coil+??->Tubulin+coiled_coil+HTH?+??->TM+TM+TM+vWA(canonical)+TM+TM+TM+polar_tail->Fibronectin-like_repeats+??->ZnR+ZnR+DO-GTPase2.2+ZnR+ZnR->GAP1-N2+GAP1-M+GAP1-C+polar_tail+TM+Cadherin-like*->

■ **GCA_000179395.2; Corynebacterium variabile; counts= 1; multicellularity status: FALSE**

- polar_leader+TM+TM+TM->TM+vWA(canonical)+Fibronectin-like_repeats+TM+beta-rich+lc_tail->Tubulin+alpha_helical+??+??->GAP_unknown1+TM+TM+GAP_unknown2->HRDC-like?+Fibronectin-like_repeats->GAP1-N2+GAP1-M+GAP1-C*->beta-rich+vWA(canonical)->??+DO-GTPase2.2->

■ **GCA_000177195.2; Streptomyces; counts= 1; multicellularity status: TRUE**

- GAP1-N2+GAP1-M+GAP1-C*->

■ **GCA_000176115.2; Streptomyces pratensis; counts= 1; multicellularity status: TRUE**

- GAP1-N2+GAP1-M+GAP1-C*->

■ **GCA_000235585.1; Paenibacillus terrae; counts= 1; multicellularity status: TRUE**

- TM+vWA(canonical)+YtKA-like+TM+??->Tubulin+coiled_coil+??->Tubulin+coiled_coil+HTH?+??->TM+TM+TM+vWA(canonical)+TM+TM+TM+polar_tail->Fibronectin-like_repeats+??->ZnR+ZnR+DO-GTPase2.2+ZnR+ZnR->GAP1-N2+GAP1-M+GAP1-C+polar_tail+TM+beta-rich*->

■ **GCA_000250655.1; Paenibacillus mucilaginosus; counts= 1; multicellularity status: TRUE**

- TM+vWA(canonical)+YtKA-like+TM+??->PSE->PSE->TM+TM+TM+vWA(canonical)+TM+TM+TM+polar_tail->Fibronectin-like_repeats+??->ZnR+ZnR+DO-GTPase2.2+ZnR+ZnR->GAP1-N2+GAP1-M+GAP1-C+polar_tail+TM+Cadherin-like*->

■ **GCA_000252445.1; Deinococcus gobiensis; counts= 1; multicellularity status: FALSE**

- GAP2->DO-GTPase2.1->DO-GTPase1->GAP1-N1+GAP1-M+GAP1-C*->

■ **GCA_000237145.1; Actinoplanes; counts= 1; multicellularity status: TRUE**

- GrpE->HSP70->TPR_containing_alpha_helical+Fibronectin-like_repeats->ZnR+ZnR+DO-GTPase2.2->GAP1-N2+GAP1-M+GAP1-C*->TM+TM+TM+TM+ZnR+ZnR+DO-GTPase2.2->

■ **GCA_000294515.1; Nocardiopsis alba; counts= 1; multicellularity status: TRUE**

- TPR_containing_alpha_helical+Fibronectin-like_repeats->ZnR+ZnR+DO-GTPase2.2->GAP1-N2+GAP1-M+GAP1-C*->TM+TM+TM+TM+ZnR+ZnR+DO-GTPase2.2->

■ **GCA_000299395.1; Nitrosopumilus; counts= 1; multicellularity status: FALSE**

- GAP1-N2+GAP1-M+TM+low_complexity*->

- **GCA_000311765.1; Gluconobacter oxydans; counts= 1; multicellularity status: FALSE**
  - CASPASE+GAPEAD1->GAP2->ZnR+DO-GTPase2.1->DO-GTPase1->GAP1-N1+GAP1-M+GAP1-C*->
- **GCA_000320385.2; Sinorhizobium meliloti; counts= 1; multicellularity status: TRUE**
  - GAP2->ZnR+DO-GTPase2.1->DO-GTPase1->GAP1-N1+GAP1-M+GAP1-C+EAD1*->CASPASE+EAD1->
- **GCA_000359525.1; Streptomyces; counts= 1; multicellularity status: TRUE**
  - GAP1-N2+GAP1-M+GAP1-C*->
- **GCA_000364805.1; Streptomyces sp. PAMC 26508; counts= 1; multicellularity status: TRUE**
  - GAP1-N2+GAP1-M+GAP1-C*->
- **GCA_000385945.1; Streptomyces fulvissimus; counts= 1; multicellularity status: TRUE**
  - GAP1-N2+GAP1-M+GAP1-C*->
- **GCA_000344785.1; Corynebacterium callunae; counts= 1; multicellularity status: FALSE**
  - polar_leader+TM+TM+TM->TM+vWA(canonical)+Fibronectin-like_repeats+TM+beta-rich+lc_tail->Tubulin+alpha_helical+??+??->GAP_unknown1+TM+TM+GAP_unknown2->HRDC-like?+Fibronectin-like_repeats->GAP1-N2+GAP1-M+GAP1-C*->
- **GCA_000349325.1; Streptomyces davaonensis; counts= 1; multicellularity status: TRUE**
  - TPR_containing_alpha_helical+Fibronectin-like_repeats->ZnR+ZnR+DO-GTPase2.2->GAP1-N2+GAP1-M+GAP1-C*->TM+TM+TM+TM+ZnR+ZnR+DO-GTPase2.2+TM->vWA(canonical)->PP2C->Pkinase+ZnR+beta-rich->
- **GCA_000214355.1; Treponema azotonutricium; counts= 1; multicellularity status: FALSE**
  - TM+TM+TM+TM+ZnR+ZnR+DO-GTPase2.2->?->HSP70->HSP70+vWA(canonical)->?->ZnR+ZnR+DO-GTPase2.2->GAP1-N2+GAP1-M+GAP1-C*->
- **GCA_000024685.1; Paenibacillus sp. Y412MC10; counts= 1; multicellularity status: TRUE**
  - TM+vWA(canonical)+YtKA-like+TM+??->?->Tubulin+coiled_coil+??->Tubulin+coiled_coil+HTH?+??->TM+TM+TM+vWA(canonical)+TM+TM+TM+polar_tail->Fibronectin-like_repeats+??->ZnR+ZnR+DO-GTPase2.2+ZnR+ZnR->GAP1-N2+GAP1-M+GAP1-C+polar_tail+TM+beta-rich*->
- **GCA_000010165.1; Brevibacillus brevis; counts= 1; multicellularity status: FALSE**
  - TM+vWA(canonical)+YtKA-like+TM+??->Tubulin+coiled_coil+??->Tubulin+coiled_coil+HTH?+??->TM+TM+TM+vWA(canonical)+TM+TM+TM+polar_tail->Fibronectin-like_repeats+??->ZnR+ZnR+DO-GTPase2.2+ZnR+ZnR->GAP1-N2+GAP1-M+GAP1-C+polar_tail+TM+beta-rich*->
- **GCA_000401235.1; Bacillus nealsonii; counts= 1; multicellularity status: FALSE**
  - GAP1-N2+GAP1-M+GAP1-C+polar_tail+TM+beta-rich*->
- **GCA_000258535.2; Paenibacillus mucilaginosus; counts= 1; multicellularity status: TRUE**
  - TM+vWA(canonical)+YtKA-like+TM+??->Tubulin+coiled_coil+??->Tubulin+coiled_coil+HTH?+??->TM+TM+TM+vWA(canonical)+TM+TM+TM+polar_tail->Fibronectin-like_repeats+??->ZnR+ZnR+DO-GTPase2.2+ZnR+ZnR->GAP1-N2+GAP1-M+GAP1-C+polar_tail+TM+Cadherin-like*->
- **GCA_000411315.1; Streptomyces sp. HGB0020; counts= 1; multicellularity status: TRUE**
  - GAP1-N2+GAP1-M+GAP1-C*->vWA(canonical)->PP2C->Pkinase+ZnR+beta-rich->||<-TPR_containing_alpha_helical+Fibronectin-like_repeats
- **GCA_001184755.1; Bacillales; counts= 1; multicellularity status: NA**
  - TM+vWA(canonical)+YtKA-like+TM+??->Tubulin+coiled_coil+??->Tubulin+coiled_coil+HTH?+??->TM+TM+TM+vWA(canonical)+TM+TM+TM+polar_tail->Fibronectin-like_repeats+??->ZnR+ZnR+DO-GTPase2.2+ZnR+ZnR->GAP1-N2+GAP1-M+GAP1-C+polar_tail+TM+beta-rich*->
- **GCA_000597985.1; Paenibacillus polymyxa; counts= 1; multicellularity status: TRUE**

- Tubulin+coiled_coil+??->Tubulin+coiled_coil+HTH?+??->TM+TM+TM+vWA(canonical)+TM+TM+TM+polar_tail->Fibronectin-like_repeats+??->ZnR+ZnR+DO-GTPase2.2+ZnR+ZnR->GAP1-N2+GAP1-M+GAP1-C+polar_tail+TM+beta-rich*->

■ **GCF_000263875.1; Rhodococcus sp. DK17; counts= 1; multicellularity status: TRUE**

- polar_leader+TM+TM+TM->TM+vWA(canonical)+Fibronectin-like_repeats+TM+beta-rich+lc_tail->Tubulin+alpha_helical+??+??->GAP_unknown1+TM+TM+GAP_unknown2->HRDC-like?+Fibronectin-like_repeats->GAP1-N2+GAP1-M+GAP1-C*->beta-rich+vWA(canonical)->??+DO-GTPase2.2->

■ **GCA_000287135.2; Vibrio ordalii; counts= 1; multicellularity status: FALSE**

- PNP_UDP_1+EAD1->GAP2->ZnR(fragment)+DO-GTPase2.1->DO-GTPase1->GAP1-N1+GAP1-M+GAP1-C+EAD1*->SLATT->

■ **GCF_000269805.1; Actinomyces massiliensis; counts= 1; multicellularity status: TRUE**

- polar_leader+TM+TM+TM->TM+vWA(canonical)+Fibronectin-like_repeats+TM+beta-rich+lc_tail->Tubulin+alpha_helical+??+??->GAP_unknown1+TM+TM+GAP_unknown2->HRDC-like?+Fibronectin-like_repeats->GAP1-N2+GAP1-M+GAP1-C*->?->??+DO-GTPase2.2->

■ **GCA_000296715.2; Brevibacillus brevis; counts= 1; multicellularity status: FALSE**

- TM+vWA(canonical)+YtKA-like+TM+??->Tubulin+coiled_coil+??->Tubulin+coiled_coil+HTH?+??->TM+TM+TM+vWA(canonical)+TM+TM+TM+polar_tail->Fibronectin-like_repeats+??->ZnR+ZnR+DO-GTPase2.2+ZnR+ZnR->GAP1-N2+GAP1-M+GAP1-C+polar_tail+TM+beta-rich*->

■ **GCF_000302595.1; Pedobacter arcticus; counts= 1; multicellularity status: FALSE**

- Calcineurin+GAPEAD1->GAP2->ZnR+DO-GTPase2.1->DO-GTPase1->GAP1-N1+GAP1-M+GAP1-C*->

■ **GCA_000307675.1; Paenibacillus polymyxa; counts= 1; multicellularity status: TRUE**

- TM+vWA(canonical)+YtKA-like+TM+??->Tubulin+coiled_coil+??->Tubulin+coiled_coil+HTH?+??->TM+TM+TM+vWA(canonical)+TM+TM+TM+polar_tail->Fibronectin-like_repeats+??->ZnR+ZnR+DO-GTPase2.2+ZnR+ZnR->GAP1-N2+GAP1-M+GAP1-C+polar_tail+TM+beta-rich*->

■ **GCF_000341225.1; Nocardiopsis alba; counts= 1; multicellularity status: TRUE**

- TPR_containing_alpha_helical+Fibronectin-like_repeats->ZnR+ZnR+DO-GTPase2.2->GAP1-N2+GAP1-M+GAP1-C*->TM+TM+TM+TM+ZnR+ZnR+DO-GTPase2.2->

■ **GCF_000341265.1; Nocardiopsis prasina; counts= 1; multicellularity status: TRUE**

- GrpE->HSP70->TPR_containing_alpha_helical+Fibronectin-like_repeats->ZnR+ZnR+DO-GTPase2.2->GAP1-N2+GAP1-M+GAP1-C*->TM+TM+TM+TM+ZnR+ZnR+DO-GTPase2.2->??->

■ **GCF_000340945.1; Nocardiopsis synnemataformans; counts= 1; multicellularity status: TRUE**

- TPR_containing_alpha_helical+Fibronectin-like_repeats+extended_insert+Fibronectin-like_repeats->ZnR+ZnR+DO-GTPase2.2->GAP1-N2+GAP1-M+GAP1-C*->TM+TM+ZnR+ZnR+DO-GTPase2.2->

■ **GCF_000341065.1; Nocardiopsis halotolerans; counts= 1; multicellularity status: TRUE**

- GAP1-N2+GAP1-M+GAP1-C*->

■ **GCF_000340965.1; Nocardiopsis kunsanensis; counts= 1; multicellularity status: TRUE**

- HSP70->TPR_containing_alpha_helical+Fibronectin-like_repeats->ZnR+ZnR+DO-GTPase2.2->GAP1-N2+GAP1-M+GAP1-C*->TM+TM+TM+TM+ZnR+ZnR+DO-GTPase2.2->

■ **GCF_000340985.1; Nocardiopsis valliformis; counts= 1; multicellularity status: TRUE**

- GrpE->HSP70->TPR_containing_alpha_helical+Fibronectin-like_repeats->ZnR+ZnR+DO-GTPase2.2->GAP1-N2+GAP1-M+GAP1-C*->TM+TM+TM+TM+ZnR+ZnR+DO-GTPase2.2->??->HSP70->vWA(canonical)->

■ **GCF_000341085.1; Nocardiopsis ganjiahuensis; counts= 1; multicellularity status: TRUE**

- TPR_containing_alpha_helical+Fibronectin-like_repeats->ZnR+ZnR+DO-GTPase2.2->GAP1-N2+GAP1-M+GAP1-C*->TM+TM+TM+TM+ZnR+ZnR+DO-GTPase2.2->

■ **GCF_000341005.1; Nocardiopsis alkaliphila; counts= 1; multicellularity status: TRUE**

- TPR_containing_alpha_helical+Fibronectin-like_repeats->ZnR+ZnR+DO-GTPase2.2->GAP1-N2+GAP1-M+GAP1-C*->

■ **GCF_000341145.1; Nocardiopsis xinjiangensis; counts= 1; multicellularity status: TRUE**

- HSP70->PSE->ZnR+ZnR+DO-GTPase2.2->GAP1-N2+GAP1-M+GAP1-C*->TM+TM+TM+TM+ZnR+ZnR+DO-GTPase2.2->

■ **GCF_000346635.1; Paenibacillus sp. A9; counts= 1; multicellularity status: TRUE**

- TM+vWA(canonical)+YtKA-like+TM+??->Tubulin+coiled_coil+??->Tubulin+coiled_coil+HTH?+??->TM+TM+TM+vWA(canonical)+TM+TM+TM+polar_tail->Fibronectin-like_repeats+??->ZnR+ZnR+DO-GTPase2.2+ZnR+ZnR->GAP1-N2+GAP1-M+GAP1-C+polar_tail+TM+beta-rich*->

■ **GCF_000372385.1; Corynebacterium ciconiae; counts= 1; multicellularity status: FALSE**

- polar_leader+TM+TM+TM->TM+vWA(canonical)+Fibronectin-like_repeats+TM+beta-rich+lc_tail->Tubulin+alpha_helical+??+??->GAP_unknown1+TM+TM+GAP_unknown2->HRDC-like?+Fibronectin-like_repeats->GAP1-N2+GAP1-M+GAP1-C*->beta-rich+vWA(canonical)->??+DO-GTPase2.2->polar_leader+TM+TM+TM->

■ **GCF_000372445.1; Corynebacterium ulceribovis; counts= 1; multicellularity status: FALSE**

- polar_leader+TM+TM+TM->TM+vWA(canonical)+Fibronectin-like_repeats+TM+beta-rich+lc_tail->Tubulin+alpha_helical+??+??->GAP_unknown1+TM+TM+GAP_unknown2->HRDC-like?+Fibronectin-like_repeats->GAP1-N2+GAP1-M+GAP1-C*->beta-rich+vWA(canonical)->??+DO-GTPase2.2->?->TM+TM+TM->

■ **GCF_000375725.1; Streptomyces; counts= 1; multicellularity status: TRUE**

- GAP1-N2+GAP1-M+GAP1-C*->

■ **GCA_001445835.1; Streptomyces vitaminophilus; counts= 1; multicellularity status: TRUE**

- GAP1-N2+GAP1-M+GAP1-C*->vWA(canonical)->PSE->Pkinase+ZnR+beta-rich->||<-TPR_containing_alpha_helical+Fibronectin-like_repeats

■ **GCA_003688775.1; Streptomyces sp. LaPpAH-202; counts= 1; multicellularity status: TRUE**

- GAP1-N2+GAP1-M+GAP1-C*->

■ **GCF_000373645.1; Streptomyces sp. ATexAB-D23; counts= 1; multicellularity status: TRUE**

- GAP1-N2+GAP1-M+GAP1-C*->

■ **GCF_000374145.1; Neomegalonema perideroedes; counts= 1; multicellularity status: TRUE**

- Calcineurin+GAPEAD1->GAP2->ZnR+DO-GTPase2.1->DO-GTPase1->GAP1-N1+GAP1-M+GAP1-C*->

■ **GCF_000374305.1; Actinomadura flavalba; counts= 1; multicellularity status: TRUE**

- GrpE->HSP70->TPR_containing_alpha_helical+Fibronectin-like_repeats->ZnR+ZnR+DO-GTPase2.2->GAP1-N2+GAP1-M+GAP1-C*->TM+TM+TM+TM+ZnR+ZnR+DO-GTPase2.2+TM->

■ **GCA_002706795.1; Brevibacillus laterosporus; counts= 1; multicellularity status: FALSE**

- TM+vWA(canonical)+YtKA-like+TM+??->Tubulin+coiled_coil+??->Tubulin+coiled_coil+HTH?+??->TM+TM+TM+vWA(canonical)+TM+TM+TM+polar_tail->Fibronectin-like_repeats+??->ZnR+ZnR+DO-GTPase2.2+ZnR+ZnR->GAP1-N2+GAP1-M+GAP1-C+polar_tail+TM+beta-rich*->

■ **GCF_000374445.1; Actinokineospora enzanensis; counts= 1; multicellularity status: TRUE**

- GrpE->HSP70->TPR_containing_alpha_helical+Fibronectin-like_repeats->ZnR+ZnR+DO-GTPase2.2->GAP1-N2+GAP1-M+GAP1-C*->TM+TM+TM+TM+ZnR+ZnR+DO-GTPase2.2->HSP70->vWA(canonical)->

■ **GCF_000374845.1; Paenibacillus terrigena; counts= 1; multicellularity status: TRUE**

- TM+vWA(canonical)+YtKA-like+TM+??->Tubulin+coiled_coil+??->Tubulin+coiled_coil+HTH?+??->TM+TM+TM+vWA(canonical)+TM+TM+TM+polar_tail->Fibronectin-like_repeats+??->ZnR+ZnR+DO-GTPase2.2+ZnR+ZnR->GAP1-N2+GAP1-M+GAP1-C+polar_tail+TM+beta-rich*->

■ **GCF_000377505.1; Paenibacillus massiliensis; counts= 1; multicellularity status: TRUE**

- TM+vWA(canonical)+YtKA-like+TM+??->Tubulin+coiled_coil+??->Tubulin+coiled_coil+HTH?+??->TM+TM+TM+vWA(canonical)+TM+TM+TM+polar_tail->Fibronectin-like_repeats+??->ZnR+ZnR+DO-GTPase2.2+ZnR+ZnR->GAP1-N2+GAP1-M+GAP1-C+polar_tail+TM+beta-rich*->

■ **GCF_000377545.1; Streptomyces sp. CNY228; counts= 1; multicellularity status: TRUE**

- GAP1-N2+GAP1-M+GAP1-C*->

■ **GCF_000378145.1; Saccharibacillus kuerlensis; counts= 1; multicellularity status: TRUE**

- TM+vWA(canonical)+YtKA-like+TM+??->Tubulin+coiled_coil+??->Tubulin+coiled_coil+HTH?+??->TM+TM+TM+vWA(canonical)+TM+TM+TM+polar_tail->Fibronectin-like_repeats+??->ZnR+ZnR+DO-GTPase2.2+ZnR+ZnR->GAP1-N2+GAP1-M+GAP1-C+polar_tail+TM+beta-rich*->

■ **GCF_000367365.1; Streptomyces prunicolor; counts= 1; multicellularity status: TRUE**

- GrpE->HSP70->TPR_containing_alpha_helical+Fibronectin-like_repeats->ZnR+ZnR+DO-GTPase2.2->GAP1-N2+GAP1-M+GAP1-C*->TM+TM+TM+TM+ZnR+ZnR+DO-GTPase2.2+Jelly_roll+beta-sandwich->||<-STAND_NTPase+TM+TM+TM+TM+TM+TM+TM

■ **GCF_000376565.1; Streptomyces hokutonensis; counts= 1; multicellularity status: TRUE**

- TPR_containing_alpha_helical+Fibronectin-like_repeats->ZnR+ZnR+DO-GTPase2.2->GAP1-N2+GAP1-M+GAP1-C*->TM+TM+TM+TM+ZnR+ZnR+DO-GTPase2.2+TM->vWA(canonical)->PP2C->Pkinase+ZnR+beta-rich->

■ **GCF_000283315.1; Paenibacillus sp. OSY-SE; counts= 1; multicellularity status: TRUE**

- TM+TM+TM+vWA+TM+TM+TM+polar_tail->Fibronectin-like_repeats+??->ZnR+ZnR+DO-GTPase2.2->GAP1-N2+GAP1-M+GAP1-C+polar_tail*->

■ **GCA_000317305.3; Mycobacteriaceae; counts= 1; multicellularity status: FALSE**

- TM+DUF4190+TM->TM+DUF4190+TM->polar_leader+TM+TM+TM->TM+vWA(canonical)+Fibronectin-like_repeats+TM+beta-rich+lc_tail->Tubulin+alpha_helical+??+??->GAP_unknown1+TM+TM+GAP_unknown2->HRDC-like?+Fibronectin-like_repeats->GAP1-N2+GAP1-M+GAP1-C*->beta-rich+vWA(canonical)->??+DO-GTPase2.2->PSE->TM+DUF4190+TM+PknH_C->TM+DUF4190+TM+PknH_C->

■ **GCA_000473765.1; Rhodonellum; counts= 1; multicellularity status: FALSE**

- GAP2->ZnR+DO-GTPase2.1->DO-GTPase1->GAP1-N1+GAP1-M+GAP1-C+PNP_UDP_1*->

■ **GCF_000381685.1; Nocardiopsis sp. CNS-639; counts= 1; multicellularity status: TRUE**

- TPR_containing_alpha_helical+Fibronectin-like_repeats+extended_insert+Fibronectin-like_repeats->ZnR+ZnR+DO-GTPase2.2->GAP1-N2+GAP1-M+GAP1-C*->TM+TM+TM+TM+ZnR+ZnR+DO-GTPase2.2->

■ **GCF_000381885.1; Actinomadura atramentaria; counts= 1; multicellularity status: TRUE**

- GrpE->HSP70->TPR_containing_alpha_helical+Fibronectin-like_repeats->ZnR+ZnR+DO-GTPase2.2->GAP1-N2+GAP1-M+GAP1-C*->TM+TM+TM+TM+ZnR+ZnR+DO-GTPase2.2+TM->

■ **GCF_000381905.1; Paenibacillus fonticola; counts= 1; multicellularity status: TRUE**

- TM+vWA(canonical)+YtKA-like+TM+??->Tubulin+coiled_coil+??->Tubulin+coiled_coil+HTH?+??->TM+TM+TM+vWA(canonical)+TM+TM+TM+polar_tail->Fibronectin-like_repeats+??->ZnR+ZnR+DO-GTPase2.2+ZnR+ZnR->GAP1-N2+GAP1-M+GAP1-C+polar_tail+TM+beta-rich*->

■ **GCA_900454525.1; Paenibacillus polymyxa; counts= 1; multicellularity status: TRUE**

- TM+vWA(canonical)+YtKA-like+TM+??->Tubulin+coiled_coil+??->Tubulin+coiled_coil+HTH?+??->TM+TM+TM+vWA(canonical)+TM+TM+TM+polar_tail->Fibronectin-like_repeats+??->ZnR+ZnR+DO-GTPase2.2+ZnR+ZnR->GAP1-N2+GAP1-M+GAP1-C+polar_tail+TM+beta-rich*->

■ **GCF_000374185.1; Paenibacillus sp. HW567; counts= 1; multicellularity status: TRUE**

- TM+vWA(canonical)+YtKA-like+TM+??->Tubulin+coiled_coil+??->Tubulin+coiled_coil+HTH?+??->TM+TM+TM+vWA(canonical)+TM+TM+TM+polar_tail->Fibronectin-like_repeats+??->ZnR+ZnR+DO-GTPase2.2+ZnR+ZnR->GAP1-N2+GAP1-M+GAP1-C+polar_tail+TM+beta-rich*->

■ **GCF_000383615.1; Streptomyces canus; counts= 1; multicellularity status: TRUE**

- GAP2->ZnR+DO-GTPase2.1->DO-GTPase1->GAP1-N1+GAP1-M+GAP1-C+EAD1*->CASPASE+EAD1->

■ **GCA_000767055.1; Corynebacterium doosanense; counts= 1; multicellularity status: FALSE**

- polar_leader+TM+TM+TM->TM+vWA(canonical)+Fibronectin-like_repeats+TM+beta-rich+lc_tail->Tubulin+alpha_helical+??+??->GAP_unknown1+TM+TM+GAP_unknown2->HRDC-like?+Fibronectin-like_repeats->GAP1-N2+GAP1-M+GAP1-C*->beta-rich+vWA(canonical)->??+DO-GTPase2.2->TM+TM+TM->

■ **GCA_000418365.1; Corynebacterium terpenotabidum; counts= 1; multicellularity status: FALSE**

- polar_leader+TM+TM+TM->TM+vWA(canonical)+Fibronectin-like_repeats+TM+beta-rich+lc_tail->Tubulin+alpha_helical+??+??->GAP_unknown1+TM+TM+GAP_unknown2->HRDC-like?+Fibronectin-like_repeats->GAP1-N2+GAP1-M+GAP1-C*->beta-rich+vWA(canonical)->??+DO-GTPase2.2->

■ **GCA_000018005.1; Frankia sp. EAN1pec; counts= 1; multicellularity status: TRUE**

- HSP70->TPR_containing_alpha_helical+Fibronectin-like_repeats->ZnR+ZnR+DO-GTPase2.2->GAP1-N2+GAP1-M+GAP1-C*->TM+TM+TM+TM+ZnR+ZnR+DO-GTPase2.2->

■ **GCF_000255705.1; Zavarzinella formosa; counts= 1; multicellularity status: TRUE**

- GAP1-N2+GAP1-M+GAP1-C*->

■ **GCF_000379525.1; Lamprocystis purpurea; counts= 1; multicellularity status: TRUE**

- GAP1-N2+GAP1-M+GAP1-C*->

■ **GCF_000379645.1; Actinoplanes globisporus; counts= 1; multicellularity status: TRUE**

- HSP70->||<-?||GrpE->HSP70->TPR_containing_alpha_helical+Fibronectin-like_repeats->ZnR+ZnR+DO-GTPase2.2->GAP1-N2+GAP1-M+GAP1-C*->TM+TM+TM+TM+ZnR+ZnR+DO-GTPase2.2->

■ **GCF_000377165.1; Streptomyces sp. CNY243; counts= 1; multicellularity status: TRUE**

- PP2C->Pkinase->?->?->?->HSP70_domain_2+low_complexity+domain_betaX->ZnR+ZnR+DO-GTPase2.2->GAP1-N2+GAP1-M+GAP1-C*->TM+TM+TM+TM+ZnR+ZnR+DO-GTPase2.2->

■ **GCF_000378385.1; Paenibacillus daejeonensis; counts= 1; multicellularity status: TRUE**

- TM+vWA(canonical)+YtKA-like+TM+??->Tubulin+coiled_coil+??->Tubulin+coiled_coil+HTH?+??->TM+TM+TM+vWA(canonical)+TM+TM+TM+polar_tail->Fibronectin-like_repeats+??->?->ZnR+ZnR+DO-GTPase2.2+ZnR+ZnR->GAP1-N2+GAP1-M+GAP1-C+polar_tail+TM+beta-rich*->

■ **GCA_002234385.1; Amycolatopsis alba; counts= 1; multicellularity status: TRUE**

- GrpE->HSP70->TPR_containing_alpha_helical+Fibronectin-like_repeats->ZnR+ZnR+DO-GTPase2.2->GAP1-N2+GAP1-M+GAP1-C*->TM+TM+TM+TM+ZnR+ZnR+DO-GTPase2.2->?->HSP70->vWA(canonical)->||<-?<-?<-HSP70

■ **GCF_000377105.1; Streptomyces sp. CNQ766; counts= 1; multicellularity status: TRUE**

- PP2C->Pkinase->?->?->?->HSP70_domain_2+low_complexity+domain_betaX->ZnR+ZnR+DO-GTPase2.2->GAP1-N2+GAP1-M+GAP1-C*->TM+TM+TM+TM+ZnR+ZnR+DO-GTPase2.2->

■ **GCF_000377125.1; Streptomyces sp. CNS335; counts= 1; multicellularity status: TRUE**

- PP2C->Pkinase->?->?->?->HSP70_domain_2+low_complexity+domain_betaX->ZnR+ZnR+DO-GTPase2.2->GAP1-N2+GAP1-M+GAP1-C*->TM+TM+TM+TM+ZnR+ZnR+DO-GTPase2.2->

■ **GCA_000442645.1; Corynebacterium maris; counts= 1; multicellularity status: FALSE**

- polar_leader+TM+TM+TM->TM+vWA(canonical)+Fibronectin-like_repeats+TM+beta-rich+lc_tail->Tubulin+alpha_helical+??+??->GAP_unknown1+TM+TM+GAP_unknown2->HRDC-like?+Fibronectin-like_repeats->GAP1-N2+GAP1-M+GAP1-C*->beta-rich+vWA(canonical)->??+DO-GTPase2.2->TM+TM+TM->

■ **GCA_000444875.1; Streptomyces collinus; counts= 1; multicellularity status: TRUE**

- *

■ **GCA_000455705.1; Pseudomonas aeruginosa; counts= 1; multicellularity status: FALSE**

- Calcineurin+GAPEAD1->GAP2->ZnR+DO-GTPase2.1->DO-GTPase1->GAP1-N1+GAP1-M+GAP1-C*->

■ **GCA_000465875.3; Alcaligenes sp. EGD-AK7; counts= 1; multicellularity status: FALSE**

- GAP2->ZnR+DO-GTPase2.1->DO-GTPase1->GAP1-N1+GAP1-M+GAP1-C+EAD1*->CASPASE+EAD1->

■ **GCA_001039615.1; Crocosphaera watsonii; counts= 1; multicellularity status: TRUE**

- DnaJ+DnaJ+ZnR+ZnR+coiled_coil+Fibronectin-like_repeats->ZnR+ZnR+DO-GTPase2.2(frag)->?->GAP1-N2+GAP1-M+GAP1-C*->TM+TM+TM+TM+TM+ZnR+ZnR+DO-GTPase2.2+TM+beta-sandwich+NTF-like->GrpE->HSP70->?->?->HSP70+vWA(canonical)->

■ **GCA_000472325.2; Brevibacillus laterosporus; counts= 1; multicellularity status: FALSE**

- TM+vWA(canonical)+YtKA-like+TM+??->Tubulin+coiled_coil+??->Tubulin+coiled_coil+HTH?+??->TM+TM+TM+vWA(canonical)+TM+TM+TM+polar_tail->Fibronectin-like_repeats+??->ZnR+ZnR+DO-GTPase2.2+ZnR+ZnR->GAP1-N2+GAP1-M+GAP1-C+polar_tail+TM+beta-rich*->

■ **GCF_000412185.1; Euryhalocaulis caribicus; counts= 1; multicellularity status: FALSE**

- GAP2->DO-GTPase2.1->DO-GTPase1->GAP1-N1+GAP1-M+GAP1-C+DNA-glycosylase+EAD1*->CASPASE+EAD1->

■ **GCA_000157895.2; Mycobacterium kansasii; counts= 1; multicellularity status: FALSE**

- TM+DUF4190+TM->TM+DUF4190+TM->polar_leader+TM+TM+TM->TM+vWA(canonical)+Fibronectin-like_repeats+TM+beta-rich+lc_tail->Tubulin+alpha_helical+??+??->GAP_unknown1+TM+TM+GAP_unknown2->HRDC-like?+Fibronectin-like_repeats->GAP1-N2+GAP1-M+GAP1-C*->beta-rich+vWA(canonical)->??+DO-GTPase2.2->

■ **GCA_000495755.1; Streptomyces sp. PVA 94-07; counts= 1; multicellularity status: TRUE**

- GAP1-N2+GAP1-M+GAP1-C*->

■ **GCA_000495635.1; Streptomyces sp. GBA 94-10; counts= 1; multicellularity status: TRUE**

- GAP1-N2+GAP1-M+GAP1-C*->

■ **GCA_000497425.1; Streptomyces niveus; counts= 1; multicellularity status: TRUE**

- GAP1-N2+GAP1-M+GAP1-C*->vWA(canonical)->PP2C->Pkinase+ZnR+beta-rich->||<-TPR_containing_alpha_helical+Fibronectin-like_repeats

■ **GCA_000503775.1; Brevibacillus panacihumi; counts= 1; multicellularity status: FALSE**

- TM+vWA(canonical)+YtKA-like+TM+??->Tubulin+coiled_coil+??->Tubulin+coiled_coil+HTH?+??->TM+TM+TM+vWA(canonical)+TM+TM+TM+polar_tail->Fibronectin-like_repeats+??->ZnR+ZnR+DO-GTPase2.2+ZnR+ZnR->GAP1-N2+GAP1-M+GAP1-C+polar_tail+TM+beta-rich*->

■ **GCA_000502415.1; Mesorhizobium sp. LNHC229A00; counts= 1; multicellularity status: TRUE**

- GAP1-N1+GAP1-M+GAP1-C*->

■ **GCA_000502395.1; Mesorhizobium sp. LNHC220B00; counts= 1; multicellularity status: TRUE**

- Calcineurin+GAPEAD1->GAP2->DO-GTPase2.1->DO-GTPase1->GAP1-N1+GAP1-M+GAP1-C*->

■ **GCA_000502375.1; Mesorhizobium sp. L48C026A00; counts= 1; multicellularity status: TRUE**

- Calcineurin+GAPEAD1->GAP2->ZnR(fragment)+DO-GTPase2.1->DO-GTPase1->GAP1-N1+GAP1-M+GAP1-C*->

■ **GCA_000502895.1; Mesorhizobium sp. L2C085B000; counts= 1; multicellularity status: TRUE**

- Calcineurin+GAPEAD1->GAP2->ZnR+DO-GTPase2.1->DO-GTPase1->GAP1-N1+GAP1-M+GAP1-C*->

■ **GCA_000507785.1; Ruminococcus lactaris; counts= 1; multicellularity status: TRUE**

- ??->??+HTH?+GrpE->HSP70->DnaJ+DnaJ+ZnR+Fibronectin-like_repeats->ZnR+ZnR+DO-GTPase2.2->GAP1-N2+GAP1-M+GAP1-C*->?->TM+TM+TM+TM+ZnR+ZnR+DO-GTPase2.2->

■ **GCA_000509425.1; Paenibacillus sp. JCM 10914; counts= 1; multicellularity status: TRUE**

- Tubulin+coiled_coil+??->PSE->TM+TM+TM+vWA(canonical)+TM+TM+TM+polar_tail->Fibronectin-like_repeats+??->ZnR+ZnR+DO-GTPase2.2+ZnR+ZnR->GAP1-N2+GAP1-M+GAP1-C+polar_tail+TM+beta-rich*->

■ **GCA_000507205.2; Paenibacillus polymyxa; counts= 1; multicellularity status: TRUE**

- TM+vWA(canonical)+YtKA-like+TM+??->Tubulin+coiled_coil+??->Tubulin+coiled_coil+HTH?+??->TM+TM+TM+vWA(canonical)+TM+TM+TM+polar_tail->Fibronectin-like_repeats+??->ZnR+ZnR+DO-GTPase2.2+ZnR+ZnR->GAP1-N2+GAP1-M+GAP1-C+polar_tail+TM+beta-rich*->

■ **GCA_000508325.2; Bacillus vireti; counts= 1; multicellularity status: NA**

- TM+vWA(canonical)+YtKA-like+TM+??->Tubulin+coiled_coil+??->Tubulin+coiled_coil+HTH?+??->TM+TM+TM+vWA(canonical)+TM+TM+TM+polar_tail->Fibronectin-like_repeats+??->ZnR+ZnR+DO-GTPase2.2+ZnR+ZnR->GAP1-N2+GAP1-M+GAP1-C+polar_tail+TM+beta-rich*->

■ **GCF_000344825.1; Salmonella enterica; counts= 1; multicellularity status: FALSE**

- Calcineurin+GAPEAD1->GAP2->DO-GTPase2.1(fragment)->DO-GTPase1->GAP1-N1+GAP1-M+GAP1-C*->

■ **GCA_000156695.2; Streptomyces filamentosus; counts= 1; multicellularity status: TRUE**

- GAP1-N2+GAP1-M+GAP1-C*->

■ **GCF_000440555.1; Streptococcus suis; counts= 1; multicellularity status: FALSE**

- HSP70->?->ZnR+ZnR+DO-GTPase2.2->GAP1-N2+GAP1-M+GAP1-C*->

■ **GCF_000470535.1; Streptomyces sp. AW19M42; counts= 1; multicellularity status: TRUE**

- GAP1-N2+GAP1-M+GAP1-C*->

■ **GCA_000499205.1; Paenibacillus; counts= 1; multicellularity status: TRUE**

- TM+TM+TM+vWA+TM+TM+TM+polar_tail->Fibronectin-like_repeats+??->ZnR+ZnR+DO-GTPase2.2->GAP1-N2+GAP1-M+GAP1-C+polar_tail*->

■ **GCF_000452545.1; Pseudomonas syringae; counts= 1; multicellularity status: FALSE**

- Calcineurin+GAPEAD1->GAP2->ZnR+DO-GTPase2.1->DO-GTPase1->GAP1-N1+GAP1-M+GAP1-C*->

■ **GCF_000612185.1; Brevibacillus borstelensis; counts= 1; multicellularity status: FALSE**

- TM+vWA(canonical)+YtKA-like+TM+??->Tubulin+coiled_coil+??->Tubulin+coiled_coil+HTH?+??->TM+TM+TM+vWA(canonical)+TM+TM+TM+polar_tail->Fibronectin-like_repeats+??->ZnR+ZnR+DO-GTPase2.2+ZnR+ZnR->GAP1-N2+GAP1-M+GAP1-C+polar_tail+TM+beta-rich*->

■ **GCA_002074835.1; Leptospira interrogans; counts= 1; multicellularity status: FALSE**

- Calcineurin+GAPEAD1->GAP2->ZnR+DO-GTPase2.1->DO-GTPase1->GAP1-N1+GAP1-M+GAP1-C*->

■ **GCA_000612505.1; Paenibacillus sabinae; counts= 1; multicellularity status: TRUE**

- TM+vWA(canonical)+YtKA-like+TM+??->Tubulin+coiled_coil+??->Tubulin+coiled_coil+HTH?+??->TM+TM+TM+vWA(canonical)+TM+TM+TM+polar_tail->Fibronectin-like_repeats+??->ZnR+ZnR+DO-GTPase2.2+ZnR+ZnR->GAP1-N2+GAP1-M+GAP1-C+polar_tail+TM+beta-rich*->

■ **GCA_000582515.1; Yersinia similis; counts= 1; multicellularity status: FALSE**

- GAP2->ZnR+DO-GTPase2.1->DO-GTPase1->GAP1-N1+GAP1-M+GAP1-C+EAD1*->CASPASE+EAD1->

■ **GCF_000490295.1; Vibrio parahaemolyticus; counts= 1; multicellularity status: FALSE**

- GAP2->ZnR+DO-GTPase2.1->DO-GTPase1->GAP1-N1+GAP1-M+GAP1-C+EAD1*->

■ **GCF_000520695.1; Paenibacillus massiliensis; counts= 1; multicellularity status: TRUE**

- Tubulin+coiled_coil+??->Tubulin+coiled_coil+HTH?+??->TM+TM+TM+vWA(canonical)+TM+TM+TM+polar_tail->Fibronectin-like_repeats+??->ZnR+ZnR+DO-GTPase2.2+ZnR+ZnR->GAP1-N2+GAP1-M+GAP1-C+polar_tail+TM+beta-rich*->

■ **GCF_000520795.1; Paenibacillus polymyxa; counts= 1; multicellularity status: TRUE**

- Tubulin+coiled_coil+??->Tubulin+coiled_coil+HTH?+??->TM+TM+TM+vWA+TM+TM+TM+polar_tail->Fibronectin-like_repeats+??->ZnR+ZnR+DO-GTPase2.2->GAP1-N2+GAP1-M+GAP1-C+polar_tail*->

■ **GCF_000520815.1; Paenibacillus sp. 1-49; counts= 1; multicellularity status: TRUE**

- TM+vWA(canonical)+YtKA-like+TM+??->Tubulin+coiled_coil+??->Tubulin+coiled_coil+HTH?+??->TM+TM+TM+vWA(canonical)+TM+TM+TM+polar_tail->Fibronectin-like_repeats+??->ZnR+ZnR+DO-GTPase2.2+ZnR+ZnR->GAP1-N2+GAP1-M+GAP1-C+polar_tail+TM+beta-rich*->

■ **GCF_000520735.1; Paenibacillus forsythiae; counts= 1; multicellularity status: TRUE**

- TM+TM+TM+vWA(canonical)+TM+TM+TM+polar_tail->Fibronectin-like_repeats+??->ZnR+ZnR+DO-GTPase2.2+ZnR+ZnR->GAP1-N2+GAP1-M+GAP1-C+polar_tail+TM+beta-rich*->

■ **GCF_000520755.1; Paenibacillus sp. 1-18; counts= 1; multicellularity status: TRUE**

- TM+vWA(canonical)+YtKA-like+TM+??->Tubulin+coiled_coil+??->Tubulin+coiled_coil+HTH?+??->TM+TM+TM+vWA(canonical)+TM+TM+TM+polar_tail->Fibronectin-like_repeats+??->ZnR+ZnR+DO-GTPase2.2+ZnR+ZnR->GAP1-N2+GAP1-M+GAP1-C+polar_tail+TM+beta-rich*->

■ **GCF_000520775.1; Paenibacillus polymyxa; counts= 1; multicellularity status: TRUE**

- TM+vWA(canonical)+YtKA-like+TM+??->Tubulin+coiled_coil+??->Tubulin+coiled_coil+HTH?+??->TM+TM+TM+vWA(canonical)+TM+TM+TM+polar_tail->Fibronectin-like_repeats+??->ZnR+ZnR+DO-GTPase2.2+ZnR+ZnR->GAP1-N2+GAP1-M+GAP1-C+polar_tail+TM+beta-rich*->

■ **GCF_000526555.1; Bacteroides sp. 14(A); counts= 1; multicellularity status: FALSE**

- Calcineurin+GAPEAD1->?->DO-GTPase2.1->DO-GTPase1->GAP1-N1+GAP1-M+GAP1-C*->

■ **GCF_000612125.1; Brevibacillus agri; counts= 1; multicellularity status: FALSE**

- TM+vWA(canonical)+YtKA-like+TM+??->Tubulin+coiled_coil+??->Tubulin+coiled_coil+HTH?+??->TM+TM+TM+vWA(canonical)+TM+TM+TM+polar_tail->Fibronectin-like_repeats+??->ZnR+ZnR+DO-GTPase2.2+ZnR+ZnR->GAP1-N2+GAP1-M+GAP1-C+polar_tail+TM+beta-rich*->

■ **GCF_000612225.1; Paenibacillus ehimensis; counts= 1; multicellularity status: TRUE**

- TM+vWA(canonical)+YtKA-like+TM+??->Tubulin+coiled_coil+??->Tubulin+coiled_coil+HTH?+??->?->TM+TM+TM+vWA(canonical)+TM+TM+TM+polar_tail->Fibronectin-like_repeats+??->ZnR+ZnR+DO-GTPase2.2+ZnR+ZnR->GAP1-N2+GAP1-M+GAP1-C+polar_tail+TM+Cadherin-like*->

■ **GCF_000425065.1; Actinomadura rifamycini; counts= 1; multicellularity status: TRUE**

- GrpE->HSP70->TPR_containing_alpha_helical+Fibronectin-like_repeats->ZnR+ZnR+DO-GTPase2.2->GAP1-N2+GAP1-M+GAP1-C*->TM+TM+TM+TM+ZnR+ZnR+DO-GTPase2.2+TM->

■ **GCF_000429225.1; Actinomyces dentalis; counts= 1; multicellularity status: TRUE**

- GrpE->HSP70->??+Fibronectin->ZnR+ZnR+DO-GTPase2.2->GAP1-N2+GAP1-M+GAP1-C*->TM+TM+TM+TM+ZnR+ZnR+DO-GTPase2.2->

■ **GCF_000518265.1; Actinomadura oligospora; counts= 1; multicellularity status: TRUE**

- GrpE->HSP70->TPR_containing_alpha_helical+Fibronectin-like_repeats->ZnR+ZnR+DO-GTPase2.2->GAP1-N2+GAP1-M+GAP1-C*->

■ **GCF_000482865.1; Actinokineospora inagensis; counts= 1; multicellularity status: TRUE**

- vWA(canonical)+beta-rich+low_complexity->?->?->Tubulin+alpha_helical+??+??->??+TM+TM+TM+TM+??->Pkinase+TM->ZnR+ZnR+DO-GTPase2.2->GAP1-N2+GAP1-M+GAP1-C+TM+beta-rich*->

■ **GCF_000470975.1; Bacillus sp. NSP2.1; counts= 1; multicellularity status: NA**

- TM+vWA(canonical)+YtKA-like+TM+??->Tubulin+coiled_coil+??->Tubulin+coiled_coil+HTH?+??->TM+TM+TM+vWA(canonical)+TM+TM+TM+polar_tail->Fibronectin-like_repeats+??->ZnR+ZnR+DO-GTPase2.2+ZnR+ZnR->GAP1-N2+GAP1-M+GAP1-C+polar_tail+TM+beta-rich*->

■ **GCF_000686805.1; Bacillus sp. UNC41MFS5; counts= 1; multicellularity status: NA**

- TM+vWA(canonical)+YtKA-like+TM+??->Tubulin+coiled_coil+??->Tubulin+coiled_coil+HTH?+??->Fibronectin-like_repeats+??->ZnR+ZnR+DO-GTPase2.2+ZnR+ZnR->GAP1-N2+GAP1-M+GAP1-C+polar_tail+TM+beta-rich*->

■ **GCF_000515315.1; Asinibacterium sp. OR53; counts= 1; multicellularity status: FALSE**

- GAP2->ZnR+DO-GTPase2.1->DO-GTPase1->GAP1-N1+GAP1-M+GAP1-C+EAD1*->PNP_UDP_1+EAD1->

■ **GCF_000428805.1; Corynebacterium freiburgense; counts= 1; multicellularity status: FALSE**

- polar_leader+TM+TM+TM->TM+vWA(canonical)+Fibronectin-like_repeats+TM+beta-rich+lc_tail->Tubulin+alpha_helical+??+??->GAP_unknown1+TM+TM+GAP_unknown2->HRDC-like?+Fibronectin-like_repeats->GAP1-N2+GAP1-M+GAP1-C*->beta-rich+vWA(canonical)->??+DO-GTPase2.2->

■ **GCF_000427865.1; Corynebacterium sputi; counts= 1; multicellularity status: FALSE**

- polar_leader+TM+TM+TM->||<-?||TM+vWA(canonical)+Fibronectin-like_repeats+TM+beta-rich+lc_tail->Tubulin+alpha_helical+??+??->GAP_unknown1+TM+TM+GAP_unknown2->HRDC-like?+Fibronectin-like_repeats->GAP1-N2+GAP1-M+GAP1-C*->beta-rich+vWA(canonical)->??+DO-GTPase2.2->

■ **GCF_000428945.1; Hamadaea tsunoensis; counts= 1; multicellularity status: TRUE**

- TPR_containing_alpha_helical+Fibronectin-like_repeats->ZnR+ZnR+DO-GTPase2.2->GAP1-N2+GAP1-M+GAP1-C*->TM+TM+TM+TM+ZnR+ZnR+DO-GTPase2.2->

■ **GCA_900177315.1; Pseudomonas; counts= 1; multicellularity status: FALSE**

- Calcineurin+GAPEAD1->GAP2->ZnR+DO-GTPase2.1->DO-GTPase1->GAP1-N1+GAP1-M+GAP1-C*->

■ **GCF_000514995.1; Streptomyces sp. CNT371; counts= 1; multicellularity status: TRUE**

- PP2C->Pkinase->?->?->?->HSP70_domain_2+low_complexity+domain_betaX->ZnR+ZnR+DO-GTPase2.2->GAP1-N2+GAP1-M+GAP1-C*->TM+TM+TM+TM+ZnR+ZnR+DO-GTPase2.2->

■ **GCF_000514715.1; Streptomyces sp. CNH099; counts= 1; multicellularity status: TRUE**

- PP2C->Pkinase->?->?->?->HSP70_domain_2+low_complexity+domain_betaX->ZnR+ZnR+DO-GTPase2.2->GAP1-N2+GAP1-M+GAP1-C*->TM+TM+TM+TM+ZnR+ZnR+DO-GTPase2.2->

■ **GCF_000424765.1; Streptomyces sp. CNT318; counts= 1; multicellularity status: TRUE**

- PP2C->Pkinase->?->?->?->HSP70_domain_2+low_complexity+domain_betaX->ZnR+ZnR+DO-GTPase2.2->GAP1-N2+GAP1-M+GAP1-C*->TM+TM+TM+TM+ZnR+ZnR+DO-GTPase2.2->

■ **GCF_000424805.1; Streptomyces sp. CNQ865; counts= 1; multicellularity status: TRUE**

- PP2C->Pkinase->?->?->?->HSP70_domain_2+low_complexity+domain_betaX->ZnR+ZnR+DO-GTPase2.2->GAP1-N2+GAP1-M+GAP1-C*->TM+TM+TM+TM+ZnR+ZnR+DO-GTPase2.2->

■ **GCF_000621585.1; [Eubacterium] cellulosolvens; counts= 1; multicellularity status: FALSE**

- TM+vWA(canonical)+YtKA-like+IG-like+TM+??->Tubulin+coiled_coil+??->?->?->ZnR+insert+ZnR+DO-GTPase2.2+ZnR->GAP1-N2+GAP1-M+low_complexity+TM+TM*->

■ **GCA_004001485.1; Sinorhizobium meliloti; counts= 1; multicellularity status: TRUE**

- GAP2->ZnR+DO-GTPase2.1->DO-GTPase1->GAP1-N1+GAP1-M+GAP1-C+EAD1*->CASPASE+EAD1->

■ **GCF_000686945.1; Selenomonas sp. AE3005; counts= 1; multicellularity status: FALSE**

- TM+TM+TM+TM+ZnR+ZnR+DO-GTPase2.2->GrpE->HSP70->HSP70+vWA(canonical)->?->ZnR+ZnR+DO-GTPase2.2->GAP1-N2+GAP1-M+GAP1-C*->

■ **GCA_000807315.1; Bradyrhizobium japonicum; counts= 1; multicellularity status: TRUE**

- Calcineurin+GAPEAD1->GAP2->ZnR+DO-GTPase2.1->DO-GTPase1->GAP1-N1+GAP1-M+GAP1-C*->

■ **GCF_000647875.1; Streptomyces sp. SolWspMP-sol2th; counts= 1; multicellularity status: TRUE**

- GAP1-N2+GAP1-M+GAP1-C*->

■ **GCA_900091745.1; Streptomyces; counts= 1; multicellularity status: TRUE**

- GAP1-N2+GAP1-M+GAP1-C*->

■ **GCF_000621765.1; Ruminococcaceae bacterium AB4001; counts= 1; multicellularity status: NA**

- TM+vWA(canonical)+YtKA-like+IG-like+TM+??->Tubulin+coiled_coil+??->?->?->ZnR+ZnR+DO-GTPase2.2+ZnR+ZnR->GAP1-N2+GAP1-M+GAP1-C*->?->?->?->Tubulin+coiled_coil+??->

■ **GCA_900116035.1; Paenibacillus; counts= 1; multicellularity status: TRUE**

- TM+vWA(canonical)+YtKA-like+TM+??->Tubulin+coiled_coil+??->Tubulin+coiled_coil+HTH?+??->TM+TM+TM+vWA(canonical)+TM+TM+TM+polar_tail->Fibronectin-like_repeats+??->ZnR+ZnR+DO-GTPase2.2+ZnR+ZnR->GAP1-N2+GAP1-M+GAP1-C+polar_tail+TM+beta-rich*->

■ **GCF_000425125.1; Paenibacillus taiwanensis; counts= 1; multicellularity status: TRUE**

- TM+vWA(canonical)+YtKA-like+TM+??->Tubulin+coiled_coil+??->Tubulin+coiled_coil+HTH?+??->TM+TM+TM+vWA(canonical)+TM+TM+TM+polar_tail->Fibronectin-like_repeats+??->ZnR+ZnR+DO-GTPase2.2+ZnR+ZnR->GAP1-N2+GAP1-M+GAP1-C+polar_tail+TM+beta-rich*->

■ **GCF_000620645.1; Nocardioides sp. URHA0032; counts= 1; multicellularity status: TRUE**

- polar_leader+TM+TM+TM->TM+vWA(canonical)+Fibronectin-like_repeats+TM+beta-rich+lc_tail->Tubulin+alpha_helical+??+??->GAP_unknown1+TM+TM+GAP_unknown2->?->HRDC-like?+Fibronectin-like_repeats->GAP1-N2+GAP1-M+GAP1-C*->?->??+DO-GTPase2.2->

■ **GCF_000423605.1; Saccharospirillum impatiens; counts= 1; multicellularity status: FALSE**

- DO-GTPase1->GAP1-N1+GAP1-M+GAP1-C*->

■ **GCA_002591785.1; Budvicia aquatica; counts= 1; multicellularity status: FALSE**

- Calcineurin+GAPEAD1->GAP2->ZnR+DO-GTPase2.1->DO-GTPase1->GAP1-N1+GAP1-M+GAP1-C*->

■ **GCF_000426065.1; Mycobacterium sp. URHD0025; counts= 1; multicellularity status: FALSE**

- HRDC-like?+Fibronectin-like_repeats->GAP1-N2+GAP1-M+GAP1-C*->??+DO-GTPase2.2->

- **GCF_000455145.1; Mycobacterium sp. UM_WWY; counts= 1; multicellularity status: FALSE**
  - TM+DUF4190+TM->polar_leader+TM+TM+TM->PSE->Tubulin+alpha_helical+??+??->GAP_unknown1+TM+TM+GAP_unknown2->HRDC-like?+Fibronectin-like_repeats->GAP1-N2+GAP1-M+GAP1-C*->beta-rich+vWA(canonical)->??+DO-GTPase2.2->TM+DUF4190+TM+PknH_C->TM+DUF4190+TM+PknH_C->TM+DUF4190+TM+PknH_C->
- **GCA_003941845.1; Paenibacillus polymyxa; counts= 1; multicellularity status: TRUE**
  - TM+vWA(canonical)+YtKA-like+TM+??->Tubulin+coiled_coil+??->Tubulin+coiled_coil+HTH?+??->TM+TM+TM+vWA(canonical)+TM+TM+TM+polar_tail->Fibronectin-like_repeats+??->ZnR+ZnR+DO-GTPase2.2+ZnR+ZnR->GAP1-N2+GAP1-M+GAP1-C+polar_tail+TM+beta-rich*->
- **GCF_000716815.1; Streptomyces; counts= 1; multicellularity status: TRUE**
  - GAP1-N2+GAP1-M+GAP1-C*->
- **GCF_000718255.1; Spirillospora albida; counts= 1; multicellularity status: TRUE**
  - GrpE->HSP70->TPR_containing_alpha_helical+Fibronectin-like_repeats->ZnR+ZnR+DO-GTPase2.2->GAP1-N2+GAP1-M+GAP1-C*->TM+TM+TM+TM+ZnR+ZnR+DO-GTPase2.2+TM->
- **GCA_003073355.1; Streptomyces scopuliridis; counts= 1; multicellularity status: TRUE**
  - GAP1-N2+GAP1-M+GAP1-C*->
- **GCF_000717925.1; Streptomyces sp. NRRL S-241; counts= 1; multicellularity status: TRUE**
  - TPR_containing_alpha_helical+Fibronectin-like_repeats->PSE->GAP1-N2+GAP1-M+GAP1-C*->TM+TM+TM+TM+ZnR+ZnR+DO-GTPase2.2+TM->vWA(canonical)->PP2C->Pkinase+ZnR+beta-rich->
- **GCF_000720535.1; Streptomyces; counts= 1; multicellularity status: TRUE**
  - GAP1-N2+GAP1-M+GAP1-C*->
- **GCF_000720395.1; Streptomyces flavovirens; counts= 1; multicellularity status: TRUE**
  - GAP1-N2+GAP1-M+GAP1-C*->
- **GCF_000721105.1; Streptomyces cellulosae; counts= 1; multicellularity status: TRUE**
  - GrpE->HSP70->TPR_containing_alpha_helical+Fibronectin-like_repeats->ZnR+ZnR+DO-GTPase2.2->GAP1-N2+GAP1-M+GAP1-C*->TM+TM+TM+TM+ZnR+ZnR+DO-GTPase2.2+Jelly_roll+beta-sandwich->||<-STAND_NTPase+TM+TM+TM+TM+TM+TM+TM
- **GCF_000720325.1; Streptomyces sp. NRRL B-1347; counts= 1; multicellularity status: TRUE**
  - GAP1-N2+GAP1-M+GAP1-C*->vWA(canonical)->PP2C->Pkinase+ZnR+beta-rich->
- **GCF_000719495.1; Streptomyces griseus; counts= 1; multicellularity status: TRUE**
  - GAP1-N2+GAP1-M+GAP1-C*->
- **GCA_003989035.1; Streptomyces griseus; counts= 1; multicellularity status: TRUE**
  - GAP1-N2+GAP1-M+GAP1-C*->
- **GCF_000718935.1; Streptomyces sp. NRRL S-237; counts= 1; multicellularity status: TRUE**
  - TPR_containing_alpha_helical+Fibronectin-like_repeats->ZnR+ZnR+DO-GTPase2.2->GAP1-N2+GAP1-M+GAP1-C*->PSE->vWA(canonical)->PP2C->Pkinase+ZnR+beta-rich->
- **GCF_000718955.1; Streptomyces sp. NRRL S-575; counts= 1; multicellularity status: TRUE**
  - GAP1-N2+GAP1-M+GAP1-C*->||<-PSE||vWA(canonical)->PP2C->Pkinase+ZnR+beta-rich->
- **GCF_000719435.1; Streptomyces griseus; counts= 1; multicellularity status: TRUE**

- GAP1-N2+GAP1-M+GAP1-C*->

■ **GCF_000717945.1; Streptomyces sp. NRRL S-920; counts= 1; multicellularity status: TRUE**

- GAP1-N2+GAP1-M+GAP1-C*->vWA(canonical)->PP2C->Pkinase+ZnR+beta-rich->||<-TPR_containing_alpha_helical+Fibronectin-like_repeats

■ **GCF_000721685.1; Streptomyces mediolani; counts= 1; multicellularity status: TRUE**

- GAP1-N2+GAP1-M+GAP1-C*->

■ **GCF_000721305.1; Streptomyces sp. NRRL S-1868; counts= 1; multicellularity status: TRUE**

- GAP1-N2+GAP1-M+GAP1-C*->PSE->?->||<-TPR_containing_alpha_helical+Fibronectin-like_repeats

■ **GCF_000720405.1; Streptomyces sp. NRRL B-24051; counts= 1; multicellularity status: TRUE**

- GAP1-N2+GAP1-M+GAP1-C*->

■ **GCF_000721445.1; Streptomyces sp. NRRL F-5053; counts= 1; multicellularity status: TRUE**

- GAP1-N2+GAP1-M+GAP1-C*->PSE->?->||<-TPR_containing_alpha_helical+Fibronectin-like_repeats

■ **GCF_000721265.1; Streptomyces sp. NRRL F-5126; counts= 1; multicellularity status: TRUE**

- GAP1-N2+GAP1-M+GAP1-C*->

■ **GCF_000720795.1; Streptomyces sp. NRRL S-515; counts= 1; multicellularity status: TRUE**

- GAP1-N2+GAP1-M+GAP1-C*->||<-PSE||vWA(canonical)->PP2C->Pkinase+ZnR+beta-rich->

■ **GCF_000721715.1; Streptomyces sp. NRRL B-24720; counts= 1; multicellularity status: TRUE**

- GAP1-N2+GAP1-M+GAP1-C*->

■ **GCF_000719575.1; Streptomyces sp. NRRL S-1824; counts= 1; multicellularity status: TRUE**

- GAP1-N2+GAP1-M+GAP1-C*->

■ **GCF_000719475.1; Streptomyces sp. NRRL F-5630; counts= 1; multicellularity status: TRUE**

- GAP1-N2+GAP1-M+GAP1-C*->

■ **GCF_000721935.1; Streptomyces sp. NRRL WC-3549; counts= 1; multicellularity status: TRUE**

- GAP1-N2+GAP1-M+GAP1-C*->

■ **GCA_001294345.1; Streptomyces; counts= 1; multicellularity status: TRUE**

- GAP1-N2+GAP1-M+GAP1-C*->

■ **GCF_000725635.1; Streptomyces sp. NRRL B-3253; counts= 1; multicellularity status: TRUE**

- GAP1-N2+GAP1-M+GAP1-C*->

■ **GCF_000725655.1; Streptomyces; counts= 1; multicellularity status: TRUE**

- TPR_containing_alpha_helical+Fibronectin-like_repeats->ZnR+ZnR+DO-GTPase2.2->GAP1-N2+GAP1-M+GAP1-C*->PSE->vWA(canonical)->PP2C->Pkinase+ZnR+beta-rich->

■ **GCF_000735775.1; Paenibacillus polymyxa; counts= 1; multicellularity status: TRUE**

- TM+vWA(canonical)+YtKA-like+TM+??->Tubulin+coiled_coil+??->Tubulin+coiled_coil+HTH?+??->TM+TM+TM+vWA(canonical)+TM+TM+TM+polar_tail->Fibronectin-like_repeats+??->ZnR+ZnR+DO-GTPase2.2+ZnR+ZnR->GAP1-N2+GAP1-M+GAP1-C+polar_tail+TM+beta-rich*->

- **GCF_000725465.1; Streptomyces bicolor; counts= 1; multicellularity status: TRUE**
  - TPR_containing_alpha_helical+Fibronectin-like_repeats->ZnR+ZnR+DO-GTPase2.2->GAP1-N2+GAP1-M+GAP1-C*->TM+TM+TM+TM+ZnR+ZnR+DO-GTPase2.2+TM->vWA(canonical)->PP2C->Pkinase+ZnR+beta-rich->
- **GCF_001076885.1; Bacillus; counts= 1; multicellularity status: NA**
  - GAP1-N2+GAP1-M+GAP1-C+polar_tail+TM+beta-rich*->
- **GCA_000738785.1; Brevibacillus borstelensis; counts= 1; multicellularity status: FALSE**
  - TM+vWA(canonical)+YtKA-like+TM+??->Tubulin+coiled_coil+??->Tubulin+coiled_coil+HTH?+??->TM+TM+TM+vWA(canonical)+TM+TM+TM+polar_tail->Fibronectin-like_repeats+??->ZnR+ZnR+DO-GTPase2.2+ZnR+ZnR->GAP1-N2+GAP1-M+GAP1-C+polar_tail+TM+beta-rich*->
- **GCA_003096175.1; Clostridium drakei; counts= 1; multicellularity status: FALSE**
  - TM+TM+TM+TM+ZnR+ZnR+DO-GTPase2.2->TM+vWA(canonical)+YtKA-like+TM+??->Tubulin+coiled_coil+??->Tubulin+coiled_coil+HTH?+??->TM+TM+TM+vWA(canonical)+TM+TM+TM+polar_tail->Fibronectin-like_repeats+??->ZnR+ZnR+DO-GTPase2.2+ZnR+ZnR->GAP1-N2+GAP1-M*->
- **GCF_000760995.1; Rhodococcus fascians; counts= 1; multicellularity status: TRUE**
  - GAP2->?->DO-GTPase1->GAP1-N1+GAP1-M+GAP1-C+EAD1*->PNP_UDP_1+EAD1->PNP_UDP_1->
- **GCF_000761035.1; Rhodococcus fascians; counts= 1; multicellularity status: TRUE**
  - GAP2->?->DO-GTPase1->GAP1-N1+GAP1-M+GAP1-C+EAD1*->PNP_UDP_1+EAD1->PNP_UDP_1->
- **GCA_000261345.2; Streptomyces globisporus; counts= 1; multicellularity status: TRUE**
  - GAP1-N2+GAP1-M+GAP1-C*->
- **GCA_000743295.1; Streptomyces sp. JS01; counts= 1; multicellularity status: TRUE**
  - GAP1-N2+GAP1-M+GAP1-C*->
- **GCF_000167995.1; Yersinia mollaretii; counts= 1; multicellularity status: FALSE**
  - DO-GTPase2.1(fragment)->DO-GTPase1->GAP1-N1+GAP1-M+GAP1-C*->
- **GCF_000278565.1; Pseudomonas sp. Ag1; counts= 1; multicellularity status: FALSE**
  - Calcineurin+GAPEAD1->GAP2->ZnR+DO-GTPase2.1->DO-GTPase1->GAP1-N1+GAP1-M+GAP1-C*->
- **GCF_000720725.1; Streptomyces rimosus; counts= 1; multicellularity status: TRUE**
  - TPR_containing_alpha_helical+Fibronectin-like_repeats->ZnR+ZnR+DO-GTPase2.2->GAP1-N2+GAP1-M+GAP1-C*->TM+TM+TM+TM+ZnR+ZnR+DO-GTPase2.2+TM->vWA(canonical)->PP2C->Pkinase+ZnR+beta-rich->
- **GCA_002356035.1; Nocardia seriolae; counts= 1; multicellularity status: TRUE**
  - Pkinase+TM->ZnR+DO-GTPase2.1->GAP1-N2+GAP1-M+GAP1-C*->
- **GCA_000702925.2; Methylomonas sp. LW13; counts= 1; multicellularity status: FALSE**
  - PNP_UDP_1+EAD1->GAP2->ZnR+DO-GTPase2.1->DO-GTPase1->GAP1-N1+GAP1-M+GAP1-C+EAD1*->
- **GCF_000716685.1; Streptomyces virginiae; counts= 1; multicellularity status: TRUE**
  - TPR_containing_alpha_helical+Fibronectin-like_repeats->ZnR+ZnR+DO-GTPase2.2->GAP1-N2+GAP1-M+GAP1-C*->PSE->vWA(canonical)->PP2C->Pkinase+ZnR+beta-rich->
- **GCF_000717375.1; Streptomyces griseus; counts= 1; multicellularity status: TRUE**

- GAP1-N2+GAP1-M+GAP1-C*->

■ **GCF__000716605.1; Streptomyces sp. NRRL F-525; counts= 1; multicellularity status: TRUE**

- GAP1-N2+GAP1-M+GAP1-C*->

■ **GCF__000717025.1; Streptomyces atroolivaceus; counts= 1; multicellularity status: TRUE**

- GAP1-N2+GAP1-M+GAP1-C*->

■ **GCF__000716805.1; Streptomyces yerevanensis; counts= 1; multicellularity status: TRUE**

- GAP1-N2+GAP1-M+GAP1-C*->vWA(canonical)->PP2C->Pkinase+ZnR+beta-rich->||<-TPR_containing_alpha_helical+Fibronectin-like_repeats

■ **GCF__000716715.1; Dactylosporangium aurantiacum; counts= 1; multicellularity status: TRUE**

- GAP1-N2+GAP1-M+GAP1-C*->||<-HSP70_domain_1

■ **GCF__000716595.1; Saccharothrix sp. NRRL B-16314; counts= 1; multicellularity status: TRUE**

- GAP1-N2+GAP1-M+GAP1-C*->

■ **GCF__000698985.1; Bordetella bronchiseptica; counts= 1; multicellularity status: FALSE**

- Calcineurin+GAPEAD1->GAP2->ZnR+DO-GTPase2.1->DO-GTPase1->GAP1-N1+GAP1-M+GAP1-C*->

■ **GCF__000797385.1; Streptomyces sp. CNQ431; counts= 1; multicellularity status: TRUE**

- GAP1-N2+GAP1-M+GAP1-C*->

■ **GCA__000758745.1; Actinomyces urogenitalis; counts= 1; multicellularity status: TRUE**

- polar_leader+TM+TM+TM->TM+vWA(canonical)+Fibronectin-like_repeats+TM+beta-rich+lc_tail->Tubulin+alpha_helical+??+??->GAP__unknown1+TM+TM+GAP__unknown2->HRDC-like?+Fibronectin-like_repeats->GAP1-N2+GAP1-M+GAP1-C*->?->??+DO-GTPase2.2->

■ **GCA__000522425.1; Candidatus Entotheonella palauensis; counts= 1; multicellularity status: TRUE**

- HSP70+vWA(canonical)->DnaJ+DnaJ+ZnR+ZnR+coiled_coil+Fibronectin-like_repeats->ZnR+ZnR+DO-GTPase2.2->GAP1-N2+GAP1-M+GAP1-C*->

■ **GCF__000702965.1; Butyrivibrio sp. AE2032; counts= 1; multicellularity status: FALSE**

- TM+vWA(canonical)+YtKA-like+IG-like+TM+??->Tubulin+coiled_coil+??->?->?->ZnR+ZnR+DO-GTPase2.2+ZnR+ZnR->GAP1-N2+GAP1-M+GAP1-C*->?->?->?->Tubulin+coiled_coil+??->Tubulin+coiled_coil+??->

■ **GCF__000741785.1; Bifidobacterium stellenboschense; counts= 1; multicellularity status: FALSE**

- TM+TM+TM+TM+ZnR+ZnR+DO-GTPase2.2->GrpE->HSP70->HSP70+vWA(canonical)->DnaJ+DnaJ+ZnR+ZnR+coiled_coil+Fibronectin-like_repeats->ZnR+ZnR+DO-GTPase2.2->GAP1-N2+GAP1-M+GAP1-C*->

■ **GCA__000767255.1; Corynebacterium auriscanis; counts= 1; multicellularity status: FALSE**

- Tubulin+alpha_helical+??+??->?->GAP__unknown1+TM+TM+GAP__unknown2->HRDC-like?+Fibronectin-like_repeats->HRDC-like?+Fibronectin-like_repeats->GAP1-N2+GAP1-M+GAP1-C*->?->?->??+DO-GTPase2.2->

■ **GCA__000601455.1; Fervidicella metallireducens; counts= 1; multicellularity status: FALSE**

- GAP1-N2+GAP1-M+GAP1-C*->

■ **GCA__000576325.1; Bacillus sp. JCM 19045; counts= 1; multicellularity status: NA**

- GAP1-N2+GAP1-M+GAP1-C*->

■ **GCA__000576345.1; Bacillus sp. JCM 19046; counts= 1; multicellularity status: NA**

- GAP1-N2+GAP1-M+GAP1-C*->

■ **GCA_000737695.1; Flavobacterium hydatis; counts= 1; multicellularity status: FALSE**

- GAP2->ZnR+DO-GTPase2.1->DO-GTPase1->GAP1-N1+GAP1-M+GAP1-C+EAD1*->??+EAD1->EAD1+Trypsin->

■ **GCF_000744785.1; Kitasatospora azatica; counts= 1; multicellularity status: TRUE**

- TPR_containing_alpha_helical+Fibronectin-like_repeats->ZnR+ZnR+DO-GTPase2.2->GAP1-N2+GAP1-M+GAP1-C*->TM+TM+TM+TM+ZnR+ZnR+DO-GTPase2.2+TM->vWA(canonical)->PP2C->Pkinase+ZnR+beta-rich->

■ **GCA_000761485.1; Modestobacter caceresii; counts= 1; multicellularity status: TRUE**

- Pkinase+TM->ZnR+ZnR+DO-GTPase2.2->GAP1-N2+GAP1-M+GAP1-C*->

■ **GCA_000723385.1; Mycolicibacterium farcinogenes; counts= 1; multicellularity status: FALSE**

- HRDC-like?+Fibronectin-like_repeats->GAP1-N2+GAP1-M+GAP1-C*->??+DO-GTPase2.2->

■ **GCA_000612825.1; Mycolicibacterium mageritense; counts= 1; multicellularity status: FALSE**

- TM+DUF4190+TM->polar_leader+TM+TM+TM->PSE->Tubulin+alpha_helical+??+??->GAP_unknown1+TM+TM+GAP_unknown2->HRDC-like?+Fibronectin-like_repeats->GAP1-N2+GAP1-M+GAP1-C*->beta-rich+vWA(canonical)->??+DO-GTPase2.2->TM+DUF4190+TM+PknH_C->TM+DUF4190+TM+PknH_C->

■ **GCA_000612885.1; Mycolicibacterium; counts= 1; multicellularity status: FALSE**

- HRDC-like?+Fibronectin-like_repeats->GAP1-N2+GAP1-M+GAP1-C*->??+DO-GTPase2.2->

■ **GCA_000583715.2; Nocardia seriolae; counts= 1; multicellularity status: TRUE**

- Pkinase+TM->ZnR+DO-GTPase2.1->GAP1-N2+GAP1-M+GAP1-C*->

■ **GCA_000517945.1; Paenibacillus sp. FSL H8-457; counts= 1; multicellularity status: TRUE**

- TM+vWA(canonical)+YtKA-like+TM+??->Tubulin+coiled_coil+??->Tubulin+coiled_coil+HTH?+??->TM+TM+TM+vWA(canonical)+TM+TM+TM+polar_tail->Fibronectin-like_repeats+??->ZnR+ZnR+DO-GTPase2.2+ZnR+ZnR->GAP1-N2+GAP1-M+GAP1-C+polar_tail+TM+beta-rich*->

■ **GCA_000517845.1; Paenibacillus; counts= 1; multicellularity status: TRUE**

- TM+vWA(canonical)+YtKA-like+TM+??->Tubulin+coiled_coil+??->Tubulin+coiled_coil+HTH?+??->TM+TM+TM+vWA(canonical)+TM+TM+TM+polar_tail->Fibronectin-like_repeats+??->ZnR+ZnR+DO-GTPase2.2+ZnR+ZnR->GAP1-N2+GAP1-M+GAP1-C+polar_tail+TM+beta-rich*->

■ **GCA_000722545.1; Paenibacillus tyrfis; counts= 1; multicellularity status: TRUE**

- TM+vWA(canonical)+YtKA-like+TM+??->Tubulin+coiled_coil+??->Tubulin+coiled_coil+HTH?+??->?->TM+TM+TM+vWA(canonical)+TM+TM+TM+polar_tail->Fibronectin-like_repeats+??->ZnR+ZnR+DO-GTPase2.2+ZnR+ZnR->GAP1-N2+GAP1-M+GAP1-C+polar_tail+TM+Cadherin-like*->

■ **GCA_000695255.1; Photobacterium galatheae; counts= 1; multicellularity status: FALSE**

- PNP_UDP_1+EAD1->GAP2->ZnR(fragment)+DO-GTPase2.1->DO-GTPase1->GAP1-N1+GAP1-M+GAP1-C+EAD1*->SLATT->

■ **GCA_003150695.1; Rhizobium; counts= 1; multicellularity status: TRUE**

- Calcineurin+GAPEAD1->GAP2->DO-GTPase2.1->DO-GTPase1->GAP1-N1+GAP1-M+GAP1-C+??*->

■ **GCF_000585395.1; Saccharibacillus sacchari; counts= 1; multicellularity status: TRUE**

- Fibronectin-like_repeats+??->ZnR+ZnR+DO-GTPase2.2->GAP1-N2+GAP1-M+GAP1-C+polar_tail*->

■ **GCF_000716535.1; Streptomyces; counts= 1; multicellularity status: TRUE**

- TPR_containing_alpha_helical+Fibronectin-like_repeats->ZnR+ZnR+DO-GTPase2.2->GAP1-N2+GAP1-M+GAP1-C*->PSE->vWA(canonical)->PP2C->Pkinase+ZnR+beta-rich->

■ **GCF_000414115.1; Streptomyces aurantiacus; counts= 1; multicellularity status: TRUE**

  - GAP1-N2+GAP1-M+GAP1-C*->vWA(canonical)->

- **GCA_002803845.1; Streptomyces lavendulae; counts= 1; multicellularity status: TRUE**
  - GAP1-N2+GAP1-M+GAP1-C*->
- **GCF_000745675.1; Streptomyces atratus; counts= 1; multicellularity status: TRUE**
  - GAP1-N2+GAP1-M+GAP1-C*->
- **GCF_000745715.1; Streptomyces mirabilis; counts= 1; multicellularity status: TRUE**
  - TPR_containing_alpha_helical+Fibronectin-like_repeats->ZnR+ZnR+DO-GTPase2.2->GAP1-N2+GAP1-M+GAP1-C*->TM+TM+TM+TM+ZnR+ZnR+DO-GTPase2.2+TM->vWA(canonical)->PP2C->Pkinase+ZnR+beta-rich->
- **GCF_000746395.1; Streptomyces mirabilis; counts= 1; multicellularity status: TRUE**
  - TPR_containing_alpha_helical+Fibronectin-like_repeats->ZnR+ZnR+DO-GTPase2.2->GAP1-N2+GAP1-M+GAP1-C*->TM+TM+TM+TM+ZnR+ZnR+DO-GTPase2.2+TM->vWA(canonical)->PP2C->Pkinase+ZnR+beta-rich->
- **GCA_004217635.1; Streptomyces sp. CNQ-525; counts= 1; multicellularity status: TRUE**
  - PP2C->Pkinase->?->?->?->HSP70_domain_2+low_complexity+domain_betaX->ZnR+ZnR+DO-GTPase2.2->GAP1-N2+GAP1-M+GAP1-C*->TM+TM+TM+TM+ZnR+ZnR+DO-GTPase2.2->
- **GCF_000721595.1; Streptomyces sp. NRRL S-325; counts= 1; multicellularity status: TRUE**
  - GAP1-N2+GAP1-M+GAP1-C*->
- **GCF_000721455.1; Streptomyces sp. NRRL F-6628; counts= 1; multicellularity status: TRUE**
  - GAP1-N2+GAP1-M+GAP1-C*->
- **GCA_000698495.1; Streptomyces sp. NTK 937; counts= 1; multicellularity status: TRUE**
  - GAP1-N2+GAP1-M+GAP1-C*->
- **GCF_000717895.1; Streptomyces sp. NRRL WC-3744; counts= 1; multicellularity status: TRUE**
  - TPR_containing_alpha_helical+Fibronectin-like_repeats->ZnR+ZnR+DO-GTPase2.2->GAP1-N2+GAP1-M+GAP1-C*->PSE->vWA(canonical)->PP2C->Pkinase+ZnR+beta-rich->
- **GCF_900091485.1; Streptomyces; counts= 1; multicellularity status: TRUE**
  - GAP1-N2+GAP1-M+GAP1-C*->
- **GCA_000788395.1; Pseudoalteromonas piratica; counts= 1; multicellularity status: FALSE**
  - PNP_UDP_1+EAD1->GAP2->ZnR(fragment)+DO-GTPase2.1->DO-GTPase1->GAP1-N1+GAP1-M+GAP1-C+EAD1*->SLATT->
- **GCA_000747525.1; Sphingobacterium sp. ML3W; counts= 1; multicellularity status: FALSE**
  - GAP2->ZnR+DO-GTPase2.1->DO-GTPase1->GAP1-N1+GAP1-M+GAP1-C+EAD1*->?->??+EAD1->EAD1+Trypsin->
- **GCA_000773415.1; Burkholderia pseudomallei; counts= 1; multicellularity status: FALSE**
  - GAP2->DO-GTPase2.1->DO-GTPase1->GAP1-N1+GAP1-M+GAP1-C+EAD1*->CASPASE+EAD1->
- **GCA_000812465.1; Cupriavidus sp. IDO; counts= 1; multicellularity status: FALSE**
  - GAP2->DO-GTPase2.1->DO-GTPase1->GAP1-N1+GAP1-M+GAP1-C+EAD1*->
- **GCF_000813415.1; Plesiomonas shigelloides; counts= 1; multicellularity status: FALSE**

- PNP_UDP_1+EAD1->GAP2->ZnR(fragment)+DO-GTPase2.1->DO-GTPase1->GAP1-N1+GAP1-M+GAP1-C+EAD1*->SLATT->

■ **GCA_000714835.1; Paenibacillus polymyxa; counts= 1; multicellularity status: TRUE**

- TM+vWA(canonical)+YtKA-like+TM+??->Tubulin+coiled_coil+??->Tubulin+coiled_coil+HTH?+??->TM+TM+TM+vWA(canonical)+TM+TM+TM+polar_tail->Fibronectin-like_repeats+??->ZnR+ZnR+DO-GTPase2.2+ZnR+ZnR->GAP1-N2+GAP1-M+GAP1-C+polar_tail+TM+beta-rich*->

■ **GCA_000805375.1; Mycolicibacterium setense; counts= 1; multicellularity status: FALSE**

- HRDC-like?+Fibronectin-like_repeats->GAP1-N2+GAP1-M+GAP1-C*->??+DO-GTPase2.2->

■ **GCA_000805385.1; Mycolicibacterium setense; counts= 1; multicellularity status: FALSE**

- HRDC-like?+Fibronectin-like_repeats->GAP1-N2+GAP1-M+GAP1-C*->??+DO-GTPase2.2->

■ **GCA_000749915.1; Pectobacterium polaris; counts= 1; multicellularity status: FALSE**

- Calcineurin+GAPEAD1->GAP2->ZnR+DO-GTPase2.1->DO-GTPase1->GAP1-N1+GAP1-M+GAP1-C*->

■ **GCA_000746665.1; Vibrio vulnificus; counts= 1; multicellularity status: FALSE**

- PNP_UDP_1+EAD1->GAP2->ZnR(fragment)+DO-GTPase2.1->DO-GTPase1->GAP1-N1+GAP1-M+GAP1-C+EAD1*->SLATT->

■ **GCA_000817905.1; Pseudomonas fluorescens; counts= 1; multicellularity status: FALSE**

- Calcineurin+GAPEAD1->GAP2->ZnR+DO-GTPase2.1->DO-GTPase1->GAP1-N1+GAP1-M+GAP1-C*->

■ **GCF_000180155.1; Actinomyces oris; counts= 1; multicellularity status: FALSE**

- Tubulin+alpha_helical+??+??->GAP_unknown1+TM+TM+GAP_unknown2->HRDC-like?+Fibronectin-like_repeats->GAP1-N2+GAP1-M+GAP1-C*->?->??+DO-GTPase2.2->

■ **GCA_000758625.1; Paenibacillus sp. FSL R7-0273; counts= 1; multicellularity status: TRUE**

- TM+vWA(canonical)+YtKA-like+TM+??->Tubulin+coiled_coil+??->Tubulin+coiled_coil+HTH?+??->||<-?||TM+TM+TM+vWA(canonical)+TM+TM+TM+polar_tail->Fibronectin-like_repeats+??->ZnR+ZnR+DO-GTPase2.2+ZnR+ZnR->GAP1-N2+GAP1-M+GAP1-C+polar_tail+TM+beta-rich*->

■ **GCF_000364445.1; Methylobacterium mesophilicum; counts= 1; multicellularity status: TRUE**

- CASPASE+GAPEAD1->GAP2->ZnR+DO-GTPase2.1->DO-GTPase1->GAP1-N1+GAP1-M+GAP1-C*->

■ **GCA_002762195.1; Acetobacteraceae; counts= 1; multicellularity status: FALSE**

- Calcineurin+GAPEAD1->GAP2->ZnR+DO-GTPase2.1->DO-GTPase1->GAP1-N1+GAP1-M+GAP1-C*->

■ **GCA_000819665.1; Paenibacillus polymyxa; counts= 1; multicellularity status: TRUE**

- Tubulin+coiled_coil+??->Tubulin+coiled_coil+HTH?+??->TM+TM+TM+vWA+TM+TM+TM+polar_tail->Fibronectin-like_repeats+??->ZnR+ZnR+DO-GTPase2.2->GAP1-N2+GAP1-M+GAP1-C+polar_tail*->

■ **GCF_000820765.1; Candidatus Soleaferrea massiliensis; counts= 1; multicellularity status: FALSE**

- ZnR+ZnR+DO-GTPase2.2->GAP1-N2+GAP1-M+TM+GAP1-C+TM*->

■ **GCF_900143195.1; Butyricicoccus pullicaecorum; counts= 1; multicellularity status: FALSE**

- GAP1-N2+GAP1-M+TM+GAP1-C*->||<-Pkinase

■ **GCF_000173655.1; Corynebacterium amycolatum; counts= 1; multicellularity status: FALSE**

- polar_leader+TM+TM+TM->TM+vWA(canonical)+Fibronectin-like_repeats+TM+beta-rich+lc_tail->Tubulin+alpha_helical+??+??->GAP_unknown1+TM+TM+GAP_unknown2->HRDC-like?+Fibronectin-like_repeats->GAP1-N2+GAP1-M+GAP1-C*->beta-rich+vWA(canonical)->??+DO-GTPase2.2->

■ **GCF_000305495.1; Bacillus psychrosaccharolyticus; counts= 1; multicellularity status: FALSE**

- TM+vWA(canonical)+YtKA-like+TM+??->PSE->Tubulin+coiled_coil+HTH?+??->TM+TM+TM+vWA(canonical)+TM+TM+TM+polar_tail->Fibronectin-like_repeats+??->ZnR+ZnR+DO-GTPase2.2+ZnR+ZnR->GAP1-N2+GAP1-M+GAP1-C+polar_tail+TM+beta-rich*->

■ **GCF_000271305.1; Hydrocarboniphaga effusa; counts= 1; multicellularity status: FALSE**

- Calcineurin+GAPEAD1->GAP2->DO-GTPase2.1->DO-GTPase1->GAP1-N1+GAP1-M+GAP1-C*->

■ **GCF_000308695.1; Nocardia takedensis; counts= 1; multicellularity status: TRUE**

- GrpE->HSP70->TPR_containing_alpha_helical+Fibronectin-like_repeats->ZnR+ZnR+DO-GTPase2.2->GAP1-N2+GAP1-M+GAP1-C*->TM+TM+TM+TM+ZnR+ZnR+DO-GTPase2.2->?->HSP70->vWA(canonical)->

■ **GCF_000308815.1; Nocardia concava; counts= 1; multicellularity status: TRUE**

- Pkinase+TM->ZnR+DO-GTPase2.1->GAP1-N2+GAP1-M+GAP1-C*->

■ **GCF_000308655.1; Nocardia niigatensis; counts= 1; multicellularity status: TRUE**

- Pkinase+TM->ZnR+DO-GTPase2.1->GAP1-N2+GAP1-M+GAP1-C*->

■ **GCF_000025345.1; Geodermatophilus obscurus; counts= 1; multicellularity status: TRUE**

- Pkinase+TM->ZnR+ZnR+DO-GTPase2.2->GAP1-N2+GAP1-M+GAP1-C*->||<-?<-?||?->Pkinase+low_complexity->

■ **GCF_000190575.1; Bacteroides salanitronis; counts= 1; multicellularity status: FALSE**

- Calcineurin+GAPEAD1->?->DO-GTPase2.1->DO-GTPase1->GAP1-N1+GAP1-M+GAP1-C*->

■ **GCA_003028645.1; Paraburkholderia caribensis; counts= 1; multicellularity status: FALSE**

- Calcineurin+GAPEAD1->GAP2->ZnR+DO-GTPase2.1->DO-GTPase1->GAP1-N1+GAP1-M+GAP1-C*->

■ **GCF_000818175.1; Streptomyces sp. AcH 505; counts= 1; multicellularity status: TRUE**

- GAP1-N2+GAP1-M+GAP1-C*->

■ **GCA_004117095.1; Paenibacillus chitinolyticus; counts= 1; multicellularity status: TRUE**

- TM+vWA(canonical)+YtKA-like+TM+??->Tubulin+coiled_coil+??->Tubulin+coiled_coil+HTH?+??->TM+TM+TM+vWA(canonical)+TM+TM+TM+polar_tail->Fibronectin-like_repeats+??->ZnR+ZnR+DO-GTPase2.2+ZnR+ZnR->GAP1-N2+GAP1-M+GAP1-C+polar_tail+TM+Cadherin-like*->

■ **GCF_000710955.1; Nocardiopsis alba; counts= 1; multicellularity status: TRUE**

- TPR_containing_alpha_helical+Fibronectin-like_repeats->ZnR+ZnR+DO-GTPase2.2->GAP1-N2+GAP1-M+GAP1-C*->TM+TM+TM+TM+ZnR+ZnR+DO-GTPase2.2->

■ **GCA_000759795.1; Pseudescherichia vulneris; counts= 1; multicellularity status: FALSE**

- Calcineurin+GAPEAD1->GAP2->ZnR+DO-GTPase2.1->DO-GTPase1->GAP1-N1+GAP1-M+GAP1-C*->

■ **GCF_000720295.1; Streptomyces sp. NRRL WC-3725; counts= 1; multicellularity status: TRUE**

- TPR_containing_alpha_helical+Fibronectin-like_repeats->ZnR+ZnR+DO-GTPase2.2->GAP1-N2+GAP1-M+GAP1-C*->PSE->vWA(canonical)->PP2C->Pkinase+ZnR+beta-rich->

■ **GCF_000721515.1; Streptomyces sp. NRRL F-5193; counts= 1; multicellularity status: TRUE**

- TPR_containing_alpha_helical+Fibronectin-like_repeats->ZnR+ZnR+DO-GTPase2.2->GAP1-N2+GAP1-M+GAP1-C*->TM+TM+TM+TM+ZnR+ZnR+DO-GTPase2.2+TM->?->vWA(canonical)->PP2C->Pkinase+ZnR+beta-rich->

■ **GCA_000733295.1; Archangium violaceum; counts= 1; multicellularity status: TRUE**

- GAP2->ZnR+DO-GTPase2.1->DO-GTPase1->GAP1-N1+GAP1-M+GAP1-C*->

■ **GCF_000092385.1; Streptomyces bingchenggensis; counts= 1; multicellularity status: TRUE**

  - GAP1-N2+GAP1-M+GAP1-C*->

- **GCA_000785985.1; Actinoplanes utahensis; counts= 1; multicellularity status: TRUE**
  - DO-GTPase1->GAP1-N2+GAP1-M+TM+GAP1-C*->?->?->TM+vWA(canonical)+TM+low_complexity->CASPASE+low_complexity->TPR_region+CASPASE->
- **GCF_000410615.1; Novosphingobium sp. B-7; counts= 1; multicellularity status: FALSE**
  - Calcineurin+GAPEAD1->GAP2->ZnR+DO-GTPase2.1->DO-GTPase1->GAP1-N1+GAP1-M+GAP1-C*->
- **GCA_001643285.1; Aeromonas salmonicida; counts= 1; multicellularity status: FALSE**
  - Calcineurin+GAPEAD1->GAP2->DO-GTPase2.1->DO-GTPase1->GAP1-N1+GAP1-M+GAP1-C*->
- **GCF_000613125.1; Bacillus sp. EB01; counts= 1; multicellularity status: NA**
  - TM+vWA(canonical)+YtKA-like+TM+??->Tubulin+coiled_coil+??->Tubulin+coiled_coil+HTH?+??->TM+TM+TM+vWA(canonical)+TM+TM+TM+polar_tail->Fibronectin-like_repeats+??->ZnR+ZnR+DO-GTPase2.2+ZnR+ZnR->GAP1-N2+GAP1-M+GAP1-C+polar_tail+TM+beta-rich*->
- **GCA_000935305.1; Pseudomonas oryzihabitans; counts= 1; multicellularity status: FALSE**
  - GAP2->DO-GTPase2.1->DO-GTPase1->GAP1-N1+GAP1-M+GAP1-C+EAD1*->CASPASE+EAD1->
- **GCA_000661085.1; Mycobacterium colombiense; counts= 1; multicellularity status: FALSE**
  - TM+DUF4190+TM->TM+DUF4190+TM->polar_leader+TM+TM+TM->TM+vWA(canonical)+Fibronectin-like_repeats+TM+beta-rich+lc_tail->Tubulin+alpha_helical+??+??->GAP_unknown1+TM+TM+GAP_unknown2->HRDC-like?+Fibronectin-like_repeats->GAP1-N2+GAP1-M+GAP1-C*->beta-rich+vWA(canonical)->??+DO-GTPase2.2->TM+DUF4190+TM+PknH_C->TM+DUF4190+TM+PknH_C->TM+DUF4190+TM+PknH_C->TM+DUF4190+TM+PknH_C->TM+DUF4190+TM+PknH_C->
- **GCF_000455325.1; Mycolicibacterium septicum; counts= 1; multicellularity status: FALSE**
  - HRDC-like?+Fibronectin-like_repeats->GAP1-N2+GAP1-M+GAP1-C*->??+DO-GTPase2.2->
- **GCA_000943545.1; Paenibacillus terrae; counts= 1; multicellularity status: TRUE**
  - GAP1-N2+GAP1-M+GAP1-C+polar_tail+TM+beta-rich*->
- **GCA_000943535.1; Paenibacillus polymyxa; counts= 1; multicellularity status: TRUE**
  - TM+vWA(canonical)+YtKA-like+TM+??->Tubulin+coiled_coil+??->Tubulin+coiled_coil+HTH?+??->TM+TM+TM+vWA(canonical)+TM+TM+TM+polar_tail->Fibronectin-like_repeats+??->ZnR+ZnR+DO-GTPase2.2+ZnR+ZnR->GAP1-N2+GAP1-M+GAP1-C+polar_tail+TM+beta-rich*->
- **GCA_000955955.1; Lechevalieria aerocolonigenes; counts= 1; multicellularity status: TRUE**
  - vWA(canonical)+beta-rich+low_complexity->Tubulin+alpha_helical+??+??->??+TM+TM+TM+TM+??->Pkinase+TM->ZnR+ZnR+DO-GTPase2.2->GAP1-N2+GAP1-M+SLATT+SLATT*->
- **GCF_000818335.1; Vibrio campbellii; counts= 1; multicellularity status: FALSE**
  - Calcineurin+GAPEAD1->GAP2->ZnR+DO-GTPase2.1->DO-GTPase1->GAP1-N1+GAP1-M+GAP1-C*->
- **GCF_000818715.1; Vibrio jasicida; counts= 1; multicellularity status: FALSE**
  - ZnR(fragment)+DO-GTPase2.1->DO-GTPase1->GAP1-N1+GAP1-M+GAP1-C+EAD1*->SLATT->
- **GCF_000818475.1; Vibrio hyugaensis; counts= 1; multicellularity status: FALSE**
  - PNP_UDP_1+EAD1->GAP2->ZnR(fragment)+DO-GTPase2.1->DO-GTPase1->GAP1-N1+GAP1-M+GAP1-C+EAD1*->SLATT->
- **GCA_000960765.1; Desulfosporosinus sp. I2; counts= 1; multicellularity status: FALSE**
  - GAP1-N1+GAP1-M+GAP1-C*->
- **GCF_000959835.1; Vibrio vulnificus; counts= 1; multicellularity status: FALSE**

- Calcineurin+GAPEAD1->GAP2->ZnR+DO-GTPase2.1->DO-GTPase1->GAP1-N1+GAP1-M+GAP1-C*->

■ **GCF_000962825.1; Actinoplanes rectilineatus; counts= 1; multicellularity status: TRUE**

- TPR_containing_alpha_helical+Fibronectin-like_repeats->ZnR+ZnR+DO-GTPase2.2->GAP1-N2+GAP1-M+GAP1-C*->

■ **GCA_000938735.2; Sphingobacterium sp. PM2-P1-29; counts= 1; multicellularity status: FALSE**

- DO-GTPase1->GAP1-N1+GAP1-M+GAP1-C+EAD1*->??+EAD1->EAD1+Trypsin->

■ **GCA_000967665.1; Marinomonas sp. S3726; counts= 1; multicellularity status: FALSE**

- Calcineurin+GAPEAD1->GAP2->ZnR+DO-GTPase2.1->DO-GTPase1->GAP1-N1+GAP1-M+GAP1-C*->

■ **GCA_000968575.1; Pseudomonas kilonensis; counts= 1; multicellularity status: FALSE**

- GAP2->DO-GTPase2.1->DO-GTPase1->GAP1-N1+GAP1-M+GAP1-C+EAD1*->CASPASE+EAD1->

■ **GCA_000968555.1; Sinorhizobium meliloti; counts= 1; multicellularity status: TRUE**

- Calcineurin+GAPEAD1->GAP2->ZnR(fragment)+DO-GTPase2.1->DO-GTPase1->GAP1-N1+GAP1-M+GAP1-C*->

■ **GCA_002871755.1; Corynebacterium kroppenstedtii; counts= 1; multicellularity status: FALSE**

- polar_leader+TM+TM+TM->TM+vWA(canonical)+Fibronectin-like_repeats+TM+beta-rich+lc_tail->?->?->GAP_unknown1+TM+TM+GAP_unknown2->HRDC-like?+Fibronectin-like_repeats->GAP1-N2+GAP1-M+GAP1-C*->beta-rich+vWA(canonical)->??+DO-GTPase2.2->

■ **GCF_000971855.1; Corynebacterium kroppenstedtii; counts= 1; multicellularity status: FALSE**

- polar_leader+TM+TM+TM->TM+vWA(canonical)+Fibronectin-like_repeats+TM+beta-rich+lc_tail->Tubulin+alpha_helical+??+??->GAP_unknown1+TM+TM+GAP_unknown2->HRDC-like?+Fibronectin-like_repeats->GAP1-N2+GAP1-M+GAP1-C*->beta-rich+vWA(canonical)->??+DO-GTPase2.2->

■ **GCF_000971965.1; Paenibacillus wulumuqiensis; counts= 1; multicellularity status: TRUE**

- TM+vWA(canonical)+YtKA-like+TM+??->Tubulin+coiled_coil+??->Tubulin+coiled_coil+HTH?+??->TM+TM+TM+vWA(canonical)+TM+TM+TM+polar_tail->Fibronectin-like_repeats+??->ZnR+ZnR+DO-GTPase2.2+ZnR+ZnR->GAP1-N2+GAP1-M+GAP1-C+polar_tail+TM+beta-rich*->

■ **GCF_000971985.1; Paenibacillus dauci; counts= 1; multicellularity status: TRUE**

- TM+vWA(canonical)+YtKA-like+TM+??->Tubulin+coiled_coil+??->Tubulin+coiled_coil+HTH?+??->TM+TM+TM+vWA(canonical)+TM+TM+TM+polar_tail->Fibronectin-like_repeats+??->ZnR+ZnR+DO-GTPase2.2+ZnR+ZnR->GAP1-N2+GAP1-M+GAP1-C+polar_tail+TM+beta-rich*->

■ **GCA_000972385.1; Streptomyces sp. WM6386; counts= 1; multicellularity status: TRUE**

- TPR_containing_alpha_helical+Fibronectin-like_repeats->ZnR+ZnR+DO-GTPase2.2->GAP1-N2+GAP1-M+GAP1-C*->TM+TM+TM+TM+ZnR+ZnR+DO-GTPase2.2+TM->vWA(canonical)->PP2C->Pkinase+ZnR+beta-rich->

■ **GCA_000980905.1; Pseudomonas syringae group genomosp. 3; counts= 1; multicellularity status: FALSE**

- Calcineurin+GAPEAD1->GAP2->ZnR+DO-GTPase2.1->DO-GTPase1->GAP1-N1+GAP1-M+GAP1-C*->

■ **GCA_000988105.2; Leptospira santarosai; counts= 1; multicellularity status: FALSE**

- Calcineurin+GAPEAD1->GAP2->ZnR+DO-GTPase2.1->DO-GTPase1->GAP1-N1+GAP1-M+GAP1-C*->

■ **GCF_000988945.1; Streptomyces europaeiscabiei; counts= 1; multicellularity status: TRUE**

- GAP1-N2+GAP1-M+GAP1-C*->vWA(canonical)->PP2C->Pkinase+ZnR+beta-rich->

■ **GCA_001981455.1; Leptospira santarosai; counts= 1; multicellularity status: FALSE**

- Calcineurin+GAPEAD1->GAP2->ZnR+DO-GTPase2.1->DO-GTPase1->DO-GTPase1->GAP1-N1+GAP1-M+GAP1-C*->

■ **GCA_001012795.1; Mycolicibacterium; counts= 1; multicellularity status: FALSE**

- HRDC-like?+Fibronectin-like_repeats->GAP1-N2+GAP1-M+GAP1-C*->??+DO-GTPase2.2->

■ **GCA_006540185.1; Brevibacillus formosus; counts= 1; multicellularity status: FALSE**

- TM+vWA(canonical)+YtKA-like+TM+??->Tubulin+coiled_coil+??->Tubulin+coiled_coil+HTH?+??->TM+TM+TM+vWA(canonical)+TM+TM+TM+polar_tail->Fibronectin-like_repeats+??->ZnR+ZnR+DO-GTPase2.2+ZnR+ZnR->GAP1-N2+GAP1-M+GAP1-C+polar_tail+TM+beta-rich*->

■ **GCA_001013325.1; Vibrio vulnificus; counts= 1; multicellularity status: FALSE**

- PNP_UDP_1+EAD1->GAP2->ZnR+DO-GTPase2.1->DO-GTPase1->GAP1-N1+GAP1-M+GAP1-C+EAD1*->SLATT->

■ **GCA_001014595.1; Streptomyces sp. KE1; counts= 1; multicellularity status: TRUE**

- GAP1-N2+GAP1-M+GAP1-C*->

■ **GCF_000800535.1; Streptomyces sp. M10; counts= 1; multicellularity status: TRUE**

- GAP1-N2+GAP1-M+GAP1-C*->

■ **GCF_000798835.1; Shewanella sp. ZOR0012; counts= 1; multicellularity status: FALSE**

- GAP2->ZnR+DO-GTPase2.1->DO-GTPase1->GAP1-N1+GAP1-M+GAP1-C+EAD1*->CASPASE+EAD1->

■ **GCA_001029205.1; Paenibacillus sp. VT-400; counts= 1; multicellularity status: TRUE**

- TM+vWA(canonical)+YtKA-like+TM+??->Tubulin+coiled_coil+??->Tubulin+coiled_coil+HTH?+??->TM+TM+TM+vWA(canonical)+TM+TM+TM+polar_tail->Fibronectin-like_repeats+??->ZnR+ZnR+DO-GTPase2.2+ZnR+ZnR->GAP1-N2+GAP1-M+GAP1-C+polar_tail+TM+beta-rich*->

■ **GCF_001029525.1; Nocardiopsis sp. RV163; counts= 1; multicellularity status: TRUE**

- TPR_containing_alpha_helical+Fibronectin-like_repeats->ZnR+ZnR+DO-GTPase2.2->GAP1-N2+GAP1-M+GAP1-C*->

■ **GCA_001029855.1; Bacillus circulans; counts= 1; multicellularity status: FALSE**

- GAP1-N2+GAP1-M+GAP1-C+polar_tail+TM+beta-rich*->

■ **GCA_001039275.2; Brevibacillus brevis; counts= 1; multicellularity status: FALSE**

- TM+vWA(canonical)+YtKA-like+TM+??->Tubulin+coiled_coil+??->Tubulin+coiled_coil+HTH?+??->TM+TM+TM+vWA(canonical)+TM+TM+TM+polar_tail->Fibronectin-like_repeats+??->ZnR+ZnR+DO-GTPase2.2+ZnR+ZnR->GAP1-N2+GAP1-M+GAP1-C+polar_tail+TM+beta-rich*->

■ **GCA_000978965.1; Methanosarcina mazei; counts= 1; multicellularity status: TRUE**

- TM+TM+TM+TM+ZnR+ZnR+DO-GTPase2.2->GrpE->HSP70->HSP70+vWA(canonical)->DnaJ+DnaJ+ZnR+ZnR+coiled_coil+Fibronectin-like_repeats->ZnR+ZnR+DO-GTPase2.2->GAP1-N2+GAP1-M+GAP1-C*->

■ **GCA_000979335.1; Methanosarcina mazei; counts= 1; multicellularity status: TRUE**

- TM+TM+TM+TM+ZnR+ZnR+DO-GTPase2.2->GrpE->HSP70->HSP70+vWA(canonical)->DnaJ+DnaJ+ZnR+ZnR+coiled_coil+Fibronectin-like_repeats->ZnR+ZnR+DO-GTPase2.2->GAP1-N2+GAP1-M+GAP1-C*->

■ **GCA_001043915.1; Methylobacterium aquaticum; counts= 1; multicellularity status: TRUE**

- GAP1-N2+GAP1-M+TM+GAP1-C*->

■ **GCA_001045445.1; Chryseobacterium sp. BLS98; counts= 1; multicellularity status: FALSE**

- GAP2->ZnR+DO-GTPase2.1->DO-GTPase1->GAP1-N1+GAP1-M+GAP1-C+EAD1*->??+EAD1->EAD1+Trypsin->

■ **GCF_001047325.1; Streptomyces viridochromogenes; counts= 1; multicellularity status: TRUE**

- GAP2->DO-GTPase2.1->DO-GTPase1->GAP1-N1+GAP1-M+GAP1-C+EAD1*->CASPASE+EAD1->

■ **GCF_001054325.1; Corynebacterium vitaeruminis; counts= 1; multicellularity status: FALSE**

  - polar_leader+TM+TM+TM->TM+vWA(canonical)+Fibronectin-like_repeats+TM+beta-rich+lc_tail->Tubulin+alpha_helical+??+??->GAP_unknown1+TM+TM+GAP_unknown2->HRDC-like?+Fibronectin-like_repeats->GAP1-N2+GAP1-M+GAP1-C*->beta-rich+vWA(canonical)->??+DO-GTPase2.2->

- **GCF_001059615.1; Corynebacterium aurimucosum; counts= 1; multicellularity status: FALSE**
  - polar_leader+TM+TM+TM->TM+vWA(canonical)+Fibronectin-like_repeats+TM+beta-rich+lc_tail->Tubulin+alpha_helical+??+??->GAP_unknown1+TM+TM+GAP_unknown2->HRDC-like?+Fibronectin-like_repeats->GAP1-N2+GAP1-M+GAP1-C*->beta-rich+vWA(canonical)->??+DO-GTPase2.2->polar_leader+TM+TM+TM->
- **GCF_001152565.1; Yersinia pekkanenii; counts= 1; multicellularity status: FALSE**
  - Calcineurin+GAPEAD1->GAP2->DO-GTPase2.1->DO-GTPase1->GAP1-N1+GAP1-M+GAP1-C*->
- **GCF_001187435.1; Streptomyces caatingaensis; counts= 1; multicellularity status: TRUE**
  - GAP1-N2+GAP1-M+GAP1-C*->
- **GCF_006540225.1; Brevibacillus reuszeri; counts= 1; multicellularity status: FALSE**
  - TM+vWA(canonical)+YtKA-like+TM+??->Tubulin+coiled_coil+??->Tubulin+coiled_coil+HTH?+??->TM+TM+TM+vWA(canonical)+TM+TM+TM+polar_tail->Fibronectin-like_repeats+??->ZnR+ZnR+DO-GTPase2.2+ZnR+ZnR->GAP1-N2+GAP1-M+GAP1-C+polar_tail+TM+beta-rich*->
- **GCA_001187505.1; Mycolicibacterium goodii; counts= 1; multicellularity status: FALSE**
  - HRDC-like?+Fibronectin-like_repeats->GAP1-N2+GAP1-M+GAP1-C*->??+DO-GTPase2.2->
- **GCF_000455265.1; Paenibacillus antibioticophila; counts= 1; multicellularity status: TRUE**
  - Tubulin+coiled_coil+??->Tubulin+coiled_coil+HTH?+??->TM+TM+TM+vWA+TM+TM+TM+polar_tail->Fibronectin-like_repeats+??->ZnR+ZnR+DO-GTPase2.2->GAP1-N2+GAP1-M+GAP1-C+polar_tail*->
- **GCA_000698945.1; Streptomyces wadayamensis; counts= 1; multicellularity status: TRUE**
  - GAP1-N2+GAP1-M+GAP1-C*->
- **GCF_000739635.1; Verrucomicrobium sp. BvORR034; counts= 1; multicellularity status: FALSE**
  - GrpE->HSP70->||<-?||HSP70+vWA(canonical)->DnaJ+DnaJ+ZnR+ZnR+coiled_coil+Fibronectin-like_repeats->ZnR+ZnR+DO-GTPase2.2->GAP1-N2+GAP1-M+GAP1-C*->?->TM+TM+ZnR+ZnR+DO-GTPase2.2+TM+PDZ+PDZ->
- **GCA_001046895.1; Pseudarthrobacter siccitolerans; counts= 1; multicellularity status: FALSE**
  - GrpE->HSP70->TPR_containing_alpha_helical+Fibronectin-like_repeats->ZnR+ZnR+DO-GTPase2.2->GAP1-N2+GAP1-M+GAP1-C*->TM+TM+TM+TM+ZnR+ZnR+DO-GTPase2.2->??->HSP70->vWA(canonical)->
- **GCF_001140765.1; Yersinia; counts= 1; multicellularity status: FALSE**
  - Calcineurin+GAPEAD1->GAP2->ZnR+DO-GTPase2.1->DO-GTPase1->GAP1-N1+GAP1-M+GAP1-C*->
- **GCA_001136465.1; Yersinia pseudotuberculosis; counts= 1; multicellularity status: FALSE**
  - GAP2->ZnR+DO-GTPase2.1->DO-GTPase1->GAP1-N1+GAP1-M+GAP1-C*->
- **GCA_001189515.2; Olsenella sp. oral taxon 807; counts= 1; multicellularity status: FALSE**
  - TM+vWA(canonical)+YtKA-like+IG-like+TM+??->Tubulin+coiled_coil+??->?->?->ZnR+ZnR+DO-GTPase2.2+ZnR+ZnR->GAP1-N2+GAP1-M+GAP1-C*->??+Pkinase->?->Tubulin+coiled_coil+??->Tubulin+coiled_coil+??->
- **GCA_001189025.1; Streptomyces europaeiscabiei; counts= 1; multicellularity status: TRUE**
  - GAP1-N2+GAP1-M+GAP1-C*->
- **GCA_001319605.1; Yersinia mollaretii; counts= 1; multicellularity status: FALSE**
  - Calcineurin+GAPEAD1->GAP2->DO-GTPase2.1->DO-GTPase1->GAP1-N1+GAP1-M+GAP1-C*->
- **GCF_000452605.1; Pseudomonas syringae; counts= 1; multicellularity status: FALSE**

- GAP2->ZnR+DO-GTPase2.1->DO-GTPase1->GAP1-N1+GAP1-M+GAP1-C+EAD1*->CASPASE+EAD1->

■ **GCF_000498935.1; Streptomyces sp. HCCB10043; counts= 1; multicellularity status: TRUE**

- GAP1-N2+GAP1-M+GAP1-C*->

■ **GCF_000502855.1; Mesorhizobium sp. L2C067A000; counts= 1; multicellularity status: TRUE**

- Calcineurin+GAPEAD1->GAP2->ZnR+DO-GTPase2.1->DO-GTPase1->GAP1-N1+GAP1-M+GAP1-C*->

■ **GCF_001244405.1; Eubacterium sp. SB2; counts= 1; multicellularity status: FALSE**

- Calcineurin+GAPEAD1->GAP2->DO-GTPase2.1->DO-GTPase1->GAP1-N1+GAP1-M+GAP1-C*->

■ **GCF_000245555.1; Paenibacillus dendritiformis; counts= 1; multicellularity status: TRUE**

- TM+TM+TM+vWA+TM+TM+TM+polar_tail->Fibronectin-like_repeats+??->ZnR+ZnR+DO-GTPase2.2->GAP1-N2+GAP1-M+GAP1-C+polar_tail*->

■ **GCF_000316035.1; Paenibacillus sp. PAMC 26794; counts= 1; multicellularity status: TRUE**

- TM+vWA(canonical)+YtKA-like+TM+??->Tubulin+coiled_coil+??->Tubulin+coiled_coil+HTH?+??->TM+TM+TM+vWA(canonical)+TM+TM+TM+polar_tail->Fibronectin-like_repeats+??->ZnR+ZnR+DO-GTPase2.2+ZnR+ZnR->GAP1-N2+GAP1-M+GAP1-C+polar_tail+TM+beta-rich*->

■ **GCF_000423085.1; Granulicoccus phenolivorans; counts= 1; multicellularity status: TRUE**

- TM+vWA(canonical)+Fibronectin-like_repeats+TM+beta-rich+lc_tail->||<-?||Tubulin+alpha_helical+??+??->GAP_unknown1+TM+TM+GAP_unknown2->HRDC-like?+Fibronectin-like_repeats->GAP1-N2+GAP1-M+GAP1-C*->beta-rich+vWA(canonical)->??+DO-GTPase2.2->

■ **GCF_000422445.1; Paenibacillus assamensis; counts= 1; multicellularity status: TRUE**

- TM+vWA(canonical)+YtKA-like+TM+??->Tubulin+coiled_coil+??->Tubulin+coiled_coil+HTH?+??->TM+TM+TM+vWA(canonical)+TM+TM+TM+polar_tail->?->Fibronectin-like_repeats+??->ZnR+ZnR+DO-GTPase2.2+ZnR+ZnR->GAP1-N2+GAP1-M+GAP1-C+polar_tail+TM+beta-rich*->

■ **GCF_000422505.1; Paenibacillus pinihumi; counts= 1; multicellularity status: TRUE**

- TM+vWA(canonical)+YtKA-like+TM+??->Tubulin+coiled_coil+??->Tubulin+coiled_coil+HTH?+??->TM+TM+TM+vWA(canonical)+TM+TM+TM+polar_tail->Fibronectin-like_repeats+??->ZnR+ZnR+DO-GTPase2.2+ZnR+ZnR->GAP1-N2+GAP1-M+GAP1-C+polar_tail+TM+beta-rich*->

■ **GCF_000426545.1; Paenibacillus panacisoli; counts= 1; multicellularity status: TRUE**

- TM+vWA(canonical)+YtKA-like+TM+??->Tubulin+coiled_coil+??->Tubulin+coiled_coil+HTH?+??->TM+TM+TM+vWA(canonical)+TM+TM+TM+polar_tail->Fibronectin-like_repeats+??->ZnR+ZnR+DO-GTPase2.2+ZnR+ZnR->GAP1-N2+GAP1-M+GAP1-C+polar_tail+TM+beta-rich*->

■ **GCF_000497205.1; Saccharopolyspora rectivirgula; counts= 1; multicellularity status: TRUE**

- GAP1-N2+GAP1-M+GAP1-C*->

■ **GCA_000517665.1; Rhodococcus rhodochrous; counts= 1; multicellularity status: TRUE**

- TM+vWA(canonical)+Fibronectin-like_repeats+TM+beta-rich+lc_tail->Tubulin+alpha_helical+??+??->GAP_unknown1+TM+TM+GAP_unknown2->HRDC-like?+Fibronectin-like_repeats->GAP1-N2+GAP1-M+GAP1-C*->beta-rich+vWA(canonical)->??+DO-GTPase2.2->

■ **GCF_000517905.1; Paenibacillus sp. FSL H7-689; counts= 1; multicellularity status: TRUE**

- Tubulin+coiled_coil+??->Tubulin+coiled_coil+HTH?+??->TM+TM+TM+vWA+TM+TM+TM+polar_tail->Fibronectin-like_repeats+??->ZnR+ZnR+DO-GTPase2.2->GAP1-N2+GAP1-M+GAP1-C+polar_tail*->

■ **GCA_001632305.1; Paenibacillus; counts= 1; multicellularity status: TRUE**

- TM+vWA(canonical)+YtKA-like+TM+??->Tubulin+coiled_coil+??->Tubulin+coiled_coil+HTH?+??->TM+TM+TM+vWA(canonical)+TM+TM+TM+polar_tail->Fibronectin-like_repeats+??->ZnR+ZnR+DO-GTPase2.2+ZnR+ZnR->GAP1-N2+GAP1-M+GAP1-C+polar_tail+TM+beta-rich*->

■ **GCF_000517965.1; Paenibacillus sp. FSL R7-277; counts= 1; multicellularity status: TRUE**

- Tubulin+coiled_coil+??->Tubulin+coiled_coil+HTH?+??->?->?->TM+TM+TM+vWA(canonical)+TM+TM+TM+polar_tail->Fibronectin-like_repeats+??->ZnR+ZnR+DO-GTPase2.2+ZnR+ZnR->GAP1-N2+GAP1-M+GAP1-C+polar_tail+TM+beta-rich*->

■ **GCF_000524095.1; Mycobacterium kansasii; counts= 1; multicellularity status: FALSE**

- TM+DUF4190+TM->TM+DUF4190+TM->polar_leader+TM+TM+TM->PSE->PSE->GAP_unknown1+TM+TM+GAP_unknown2->GAP_unknown1+TM+TM+GAP_unknown2->PSE->GAP1-N2+GAP1-M+GAP1-C->GAP1-N2+GAP1-M+GAP1-C*->?->beta-rich+vWA(canonical)->

■ **GCA_000517925.1; Paenibacillus; counts= 1; multicellularity status: TRUE**

- TM+vWA(canonical)+YtKA-like+TM+??->Tubulin+coiled_coil+??->Tubulin+coiled_coil+HTH?+??->TM+TM+TM+vWA(canonical)+TM+TM+TM+polar_tail->Fibronectin-like_repeats+??->ZnR+ZnR+DO-GTPase2.2+ZnR+ZnR->GAP1-N2+GAP1-M+GAP1-C+polar_tail+TM+beta-rich*->

■ **GCF_000517865.1; Paenibacillus sp. FSL R7-269; counts= 1; multicellularity status: TRUE**

- TM+vWA(canonical)+YtKA-like+TM+??->Tubulin+coiled_coil+??->Tubulin+coiled_coil+HTH?+??->?->TM+TM+TM+vWA(canonical)+TM+TM+TM+polar_tail->Fibronectin-like_repeats+??->ZnR+ZnR+DO-GTPase2.2+ZnR+ZnR->GAP1-N2+GAP1-M+GAP1-C+polar_tail+TM+beta-rich*->

■ **GCF_000686845.1; Paenibacillus sp. UNC451MF; counts= 1; multicellularity status: TRUE**

- TM+vWA(canonical)+YtKA-like+TM+??->Tubulin+coiled_coil+??->Tubulin+coiled_coil+HTH?+??->TM+TM+TM+vWA(canonical)+TM+TM+TM+polar_tail->Fibronectin-like_repeats+??->ZnR+ZnR+DO-GTPase2.2+ZnR+ZnR->GAP1-N2+GAP1-M+GAP1-C+polar_tail+TM+Cadherin-like*->

■ **GCF_000710755.1; Saccharopolyspora rectivirgula; counts= 1; multicellularity status: TRUE**

- GAP1-N2+GAP1-M+GAP1-C*->

■ **GCF_000719555.1; Streptomyces sp. NRRL F-5727; counts= 1; multicellularity status: TRUE**

- ZnR+ZnR+DO-GTPase2.2->GAP1-N2+GAP1-M+GAP1-C*->TM+TM+TM+TM+ZnR+ZnR+DO-GTPase2.2+TM->?->vWA(canonical)->PP2C->Pkinase+ZnR+beta-rich->

■ **GCA_000949305.1; Rhodococcus; counts= 1; multicellularity status: TRUE**

- polar_leader+TM+TM+TM->TM+vWA(canonical)+Fibronectin-like_repeats+TM+beta-rich+lc_tail->Tubulin+alpha_helical+??+??->GAP_unknown1+TM+TM+GAP_unknown2->HRDC-like?+Fibronectin-like_repeats->GAP1-N2+GAP1-M+GAP1-C*->beta-rich+vWA(canonical)->??+DO-GTPase2.2->

■ **GCF_000719505.1; Streptomyces sp. NRRL B-3229; counts= 1; multicellularity status: TRUE**

- PP2C->Pkinase->?->?->?->HSP70_domain_2+low_complexity+domain_betaX->ZnR+ZnR+DO-GTPase2.2->GAP1-N2+GAP1-M+GAP1-C*->PSE->||<-TPR_region+CASPASE

■ **GCA_000695625.1; Amycolatopsis rifamycinica; counts= 1; multicellularity status: TRUE**

- GrpE->HSP70->TPR_containing_alpha_helical+Fibronectin-like_repeats->ZnR+ZnR+DO-GTPase2.2->GAP1-N2+GAP1-M+GAP1-C*->TM+TM+TM+TM+ZnR+ZnR+DO-GTPase2.2->?->HSP70->vWA(canonical)->?->||<-HSP70<-unknown+CASPASE

■ **GCF_000716435.1; Streptomyces xylophagus; counts= 1; multicellularity status: TRUE**

- GAP1-N2+GAP1-M+GAP1-C*->

■ **GCA_000746875.1; Paenibacillus macerans; counts= 1; multicellularity status: TRUE**

- TM+vWA(canonical)+YtKA-like+TM+??->Tubulin+coiled_coil+??->Tubulin+coiled_coil+HTH?+??->TM+TM+TM+vWA(canonical)+TM+TM+TM+polar_tail->Fibronectin-like_repeats+??->ZnR+ZnR+DO-GTPase2.2+ZnR+ZnR->GAP1-N2+GAP1-M+GAP1-C+polar_tail+TM+beta-rich*->

■ **GCA_000576305.1; Paenibacillus pini; counts= 1; multicellularity status: TRUE**

- Tubulin+coiled_coil+??->Tubulin+coiled_coil+HTH?+??->TM+TM+TM+vWA(canonical)+TM+TM+TM+polar_tail->Fibronectin-like_repeats+??->PSE->GAP1-N2+GAP1-M*->

■ **GCF_000758775.1; Actinomyces sp. S4-C9; counts= 1; multicellularity status: NA**

- GrpE->HSP70->TPR_containing_alpha_helical+Fibronectin-like_repeats->ZnR+ZnR+DO-GTPase2.2->GAP1-N2+GAP1-M+GAP1-C*->TM+TM+TM+TM+ZnR+ZnR+DO-GTPase2.2->?->?->?->HSP70->vWA(canonical)->

■ **GCA_002259145.1; Rhodococcus; counts= 1; multicellularity status: TRUE**

- polar_leader+TM+TM+TM->TM+vWA(canonical)+Fibronectin-like_repeats+TM+beta-rich+lc_tail->Tubulin+alpha_helical+??+??->GAP_unknown1+TM+TM+GAP_unknown2->HRDC-like?+Fibronectin-like_repeats->GAP1-N2+GAP1-M+GAP1-C*->beta-rich+vWA(canonical)->??+DO-GTPase2.2->

■ **GCF_000758965.1; Corynebacterium freneyi; counts= 1; multicellularity status: FALSE**

- polar_leader+TM+TM+TM->TM+vWA(canonical)+Fibronectin-like_repeats+TM+beta-rich+lc_tail->Tubulin+alpha_helical+??+??->GAP_unknown1+TM+TM+GAP_unknown2->||<-?||HRDC-like?+Fibronectin-like_repeats->GAP1-N2+GAP1-M+GAP1-C*->beta-rich+vWA(canonical)->??+DO-GTPase2.2->polar_leader+TM+TM+TM->

■ **GCF_000760815.1; Rhodococcus fascians; counts= 1; multicellularity status: TRUE**

- polar_leader+TM+TM+TM->TM+vWA(canonical)+Fibronectin-like_repeats+TM+beta-rich+lc_tail->Tubulin+alpha_helical+??+??->GAP_unknown1+TM+TM+GAP_unknown2->HRDC-like?+Fibronectin-like_repeats->GAP1-N2+GAP1-M+GAP1-C*->beta-rich+vWA(canonical)->??+DO-GTPase2.2->

- **GCF_000757885.1; Paenibacillus wynnii; counts= 1; multicellularity status: TRUE**
  - TM+vWA(canonical)+YtKA-like+TM+??->Tubulin+coiled_coil+??->Tubulin+coiled_coil+HTH?+??->TM+TM+TM+vWA(canonical)+TM+TM+TM+polar_tail->Fibronectin-like_repeats+??->ZnR+ZnR+DO-GTPase2.2+ZnR+ZnR->GAP1-N2+GAP1-M+GAP1-C+polar_tail+TM+beta-rich*->
- **GCF_000758525.1; Paenibacillus sp. FSL H7-0357; counts= 1; multicellularity status: TRUE**
  - TM+vWA(canonical)+YtKA-like+TM+??->Tubulin+coiled_coil+??->Tubulin+coiled_coil+HTH?+??->TM+TM+TM+vWA(canonical)+TM+TM+TM+polar_tail->Fibronectin-like_repeats+??->ZnR+ZnR+DO-GTPase2.2+ZnR+ZnR->GAP1-N2+GAP1-M+GAP1-C+polar_tail+TM+beta-rich*->
- **GCF_000758725.1; Paenibacillus odorifer; counts= 1; multicellularity status: TRUE**
  - TM+vWA(canonical)+YtKA-like+TM+??->Tubulin+coiled_coil+??->Tubulin+coiled_coil+HTH?+??->TM+TM+TM+vWA(canonical)+TM+TM+TM+polar_tail->Fibronectin-like_repeats+??->ZnR+ZnR+DO-GTPase2.2+ZnR+ZnR->GAP1-N2+GAP1-M+GAP1-C+polar_tail+TM+beta-rich*->
- **GCF_000758685.1; Paenibacillus stellifer; counts= 1; multicellularity status: TRUE**
  - TM+vWA(canonical)+YtKA-like+TM+??->Tubulin+coiled_coil+??->Tubulin+coiled_coil+HTH?+??->TM+TM+TM+vWA(canonical)+TM+TM+TM+polar_tail->Fibronectin-like_repeats+??->ZnR+ZnR+DO-GTPase2.2+ZnR+ZnR->GAP1-N2+GAP1-M+GAP1-C+polar_tail+TM+beta-rich*->
- **GCA_000787385.1; Paenibacillus sp. P1XP2; counts= 1; multicellularity status: TRUE**
  - TM+vWA(canonical)+YtKA-like+TM+??->Tubulin+coiled_coil+??->PSE->PSE->Fibronectin-like_repeats+??->ZnR+ZnR+DO-GTPase2.2+ZnR+ZnR->GAP1-N2+GAP1-M+GAP1-C+polar_tail+TM+beta-rich*->
- **GCA_001263755.1; Corynebacterium riegelii; counts= 1; multicellularity status: FALSE**
  - polar_leader+TM+TM+TM->TM+vWA(canonical)+Fibronectin-like_repeats+TM+beta-rich+lc_tail->Tubulin+alpha_helical+??+??->GAP_unknown1+TM+TM+GAP_unknown2->HRDC-like?+Fibronectin-like_repeats->GAP1-N2+GAP1-M+GAP1-C*->?->??+DO-GTPase2.2->?->||<-?<-?<-HSP70
- **GCF_000812585.1; Chromobacterium piscinae; counts= 1; multicellularity status: FALSE**
  - Calcineurin+GAPEAD1->GAP2->ZnR+DO-GTPase2.1->DO-GTPase1->GAP1-N1+GAP1-M+GAP1-C*->
- **GCA_900478045.1; Corynebacterium minutissimum; counts= 1; multicellularity status: FALSE**
  - polar_leader+TM+TM+TM->TM+vWA(canonical)+Fibronectin-like_repeats+TM+beta-rich+lc_tail->Tubulin+alpha_helical+??+??->GAP_unknown1+TM+TM+GAP_unknown2->HRDC-like?+Fibronectin-like_repeats->GAP1-N2+GAP1-M+GAP1-C*->beta-rich+vWA(canonical)->??+DO-GTPase2.2->polar_leader+TM+TM+TM->
- **GCF_000455485.1; Gorillibacterium massiliense; counts= 1; multicellularity status: FALSE**
  - TM+vWA(canonical)+YtKA-like+TM+??->Tubulin+coiled_coil+??->Tubulin+coiled_coil+HTH?+??->TM+TM+TM+vWA(canonical)+TM+TM+TM+polar_tail->Fibronectin-like_repeats+??->ZnR+ZnR+DO-GTPase2.2+ZnR+ZnR->GAP1-N2+GAP1-M+GAP1-C+polar_tail+TM+beta-rich*->
- **GCF_000612665.1; Bacillus dielmoensis; counts= 1; multicellularity status: FALSE**
  - TM+vWA(canonical)+YtKA-like+TM+??->Tubulin+coiled_coil+??->Tubulin+coiled_coil+HTH?+??->TM+TM+TM+vWA(canonical)+TM+TM+TM+polar_tail->Fibronectin-like_repeats+??->ZnR+ZnR+DO-GTPase2.2+ZnR+ZnR->GAP1-N2+GAP1-M+GAP1-C+polar_tail+TM+beta-rich*->
- **GCF_000612625.1; Bacillus; counts= 1; multicellularity status: NA**
  - TM+vWA(canonical)+YtKA-like+TM+??->Tubulin+coiled_coil+??->Tubulin+coiled_coil+HTH?+??->Fibronectin-like_repeats+??->ZnR+ZnR+DO-GTPase2.2+ZnR+ZnR->GAP1-N2+GAP1-M+GAP1-C+polar_tail+TM+beta-rich*->
- **GCF_000723885.1; Paenibacillus camerounensis; counts= 1; multicellularity status: TRUE**
  - TM+vWA(canonical)+YtKA-like+TM+??->Tubulin+coiled_coil+??->Tubulin+coiled_coil+HTH?+??->||<-?||TM+TM+TM+vWA(canonical)+TM+TM+TM+polar_tail->Fibronectin-like_repeats+??->ZnR+ZnR+DO-GTPase2.2+ZnR+ZnR->GAP1-N2+GAP1-M+GAP1-C+polar_tail+TM+beta-rich*->
- **GCF_000758545.1; Paenibacillus sp. FSL H7-0737; counts= 1; multicellularity status: TRUE**
  - TM+vWA(canonical)+YtKA-like+TM+??->Tubulin+coiled_coil+??->Tubulin+coiled_coil+HTH?+??->TM+TM+TM+vWA(canonical)+TM+TM+TM+polar_tail->Fibronectin-like_repeats+??->ZnR+ZnR+DO-GTPase2.2+ZnR+ZnR->GAP1-N2+GAP1-M+GAP1-C+polar_tail+TM+beta-rich*->
- **GCF_000756615.1; Paenibacillus durus; counts= 1; multicellularity status: TRUE**
  - TM+vWA(canonical)+YtKA-like+TM+??->Tubulin+coiled_coil+??->Tubulin+coiled_coil+HTH?+??->TM+TM+TM+vWA(canonical)+TM+TM+TM+polar_tail->Fibronectin-like_repeats+??->ZnR+ZnR+DO-GTPase2.2+ZnR+ZnR->GAP1-N2+GAP1-M+GAP1-C+polar_tail+TM+beta-rich*->
- **GCF_000758585.1; Paenibacillus sp. FSL R5-0345; counts= 1; multicellularity status: TRUE**

- TM+vWA(canonical)+YtKA-like+TM+??->Tubulin+coiled_coil+??->Tubulin+coiled_coil+HTH?+??->TM+TM+TM+vWA(canonical)+TM+TM+TM+polar_tail->Fibronectin-like_repeats+??->ZnR+ZnR+DO-GTPase2.2+ZnR+ZnR->GAP1-N2+GAP1-M+GAP1-C+polar_tail+TM+beta-rich*->

■ **GCF_000758705.1; Paenibacillus graminis; counts= 1; multicellularity status: TRUE**

- TM+vWA(canonical)+YtKA-like+TM+??->Tubulin+coiled_coil+??->Tubulin+coiled_coil+HTH?+??->TM+TM+TM+vWA(canonical)+TM+TM+TM+polar_tail->Fibronectin-like_repeats+??->ZnR+ZnR+DO-GTPase2.2+ZnR+ZnR->GAP1-N2+GAP1-M+GAP1-C+polar_tail+TM+beta-rich*->

■ **GCF_000758605.1; Paenibacillus sp. FSL R5-0912; counts= 1; multicellularity status: TRUE**

- TM+vWA(canonical)+YtKA-like+TM+??->Tubulin+coiled_coil+??->Tubulin+coiled_coil+HTH?+??->TM+TM+TM+vWA(canonical)+TM+TM+TM+polar_tail->Fibronectin-like_repeats+??->ZnR+ZnR+DO-GTPase2.2+ZnR+ZnR->GAP1-N2+GAP1-M+GAP1-C+polar_tail+TM+beta-rich*->

■ **GCF_000758645.1; Paenibacillus sp. FSL R7-0331; counts= 1; multicellularity status: TRUE**

- TM+vWA(canonical)+YtKA-like+TM+??->Tubulin+coiled_coil+??->Tubulin+coiled_coil+HTH?+??->TM+TM+TM+vWA(canonical)+TM+TM+TM+polar_tail->Fibronectin-like_repeats+??->ZnR+ZnR+DO-GTPase2.2+ZnR+ZnR->GAP1-N2+GAP1-M+GAP1-C+polar_tail+TM+beta-rich*->

■ **GCF_000758565.1; Paenibacillus sp. FSL P4-0081; counts= 1; multicellularity status: TRUE**

- TM+vWA(canonical)+YtKA-like+TM+??->Tubulin+coiled_coil+??->Tubulin+coiled_coil+HTH?+??->TM+TM+TM+vWA(canonical)+TM+TM+TM+polar_tail->Fibronectin-like_repeats+??->ZnR+ZnR+DO-GTPase2.2+ZnR+ZnR->GAP1-N2+GAP1-M+GAP1-C+polar_tail+TM+beta-rich*->

■ **GCF_000758665.1; Paenibacillus borealis; counts= 1; multicellularity status: TRUE**

- TM+vWA(canonical)+YtKA-like+TM+??->Tubulin+coiled_coil+??->Tubulin+coiled_coil+HTH?+??->TM+TM+TM+vWA(canonical)+TM+TM+TM+polar_tail->Fibronectin-like_repeats+??->ZnR+ZnR+DO-GTPase2.2+ZnR+ZnR->GAP1-N2+GAP1-M+GAP1-C+polar_tail+TM+beta-rich*->

■ **GCF_000787775.1; Streptacidiphilus carbonis; counts= 1; multicellularity status: TRUE**

- TPR_containing_alpha_helical+Fibronectin-like_repeats->ZnR+ZnR+DO-GTPase2.2->GAP1-N2+GAP1-M+GAP1-C*->TM+TM+DO-GTPase2->vWA(canonical)->PP2C->Pkinase+ZnR+beta-rich->

■ **GCA_000819445.1; Corynebacterium humireducens; counts= 1; multicellularity status: FALSE**

- polar_leader+TM+TM+TM->TM+vWA(canonical)+Fibronectin-like_repeats+TM+beta-rich+lc_tail->Tubulin+alpha_helical+??+??->GAP_unknown1+TM+TM+GAP_unknown2->HRDC-like?+Fibronectin-like_repeats->GAP1-N2+GAP1-M+GAP1-C*->beta-rich+vWA(canonical)->??+DO-GTPase2.2->polar_leader+TM+TM+TM->

■ **GCF_000818195.1; Streptomyces sp. 150FB; counts= 1; multicellularity status: TRUE**

- GAP1-N2+GAP1-M+GAP1-C*->

■ **GCF_000876225.1; Micromonospora carbonacea; counts= 1; multicellularity status: TRUE**

- GrpE->HSP70->TPR_containing_alpha_helical+Fibronectin-like_repeats->ZnR+ZnR+DO-GTPase2.2->GAP1-N2+GAP1-M+GAP1-C*->TM+TM+TM+TM+ZnR+ZnR+DO-GTPase2.2->

■ **GCA_002221455.1; Methylobacterium radiotolerans; counts= 1; multicellularity status: TRUE**

- Calcineurin+GAPEAD1->GAP2->ZnR+DO-GTPase2.1->DO-GTPase1->GAP1-N1+GAP1-M+GAP1-C*->

■ **GCA_001610855.1; Gemmata sp. SH-PL17; counts= 1; multicellularity status: FALSE**

- GAP1-N2+GAP1-M+TM*->

■ **GCF_000193415.1; Paenibacillus vortex; counts= 1; multicellularity status: TRUE**

- TM+vWA(canonical)+YtKA-like+TM+??->Tubulin+coiled_coil+??->Tubulin+coiled_coil+HTH?+??->TM+TM+TM+vWA(canonical)+TM+TM+TM+polar_tail->Fibronectin-like_repeats+??->ZnR+ZnR+DO-GTPase2.2+ZnR+ZnR->GAP1-N2+GAP1-M+GAP1-C+polar_tail+TM+beta-rich*->

■ **GCF_000949425.1; Paenibacillus sp. IHBB 10380; counts= 1; multicellularity status: TRUE**

- TM+vWA(canonical)+YtKA-like+TM+??->Tubulin+coiled_coil+??->Tubulin+coiled_coil+HTH?+??->TM+TM+TM+vWA(canonical)+TM+TM+TM+polar_tail->Fibronectin-like_repeats+??->ZnR+ZnR+DO-GTPase2.2+ZnR+ZnR->GAP1-N2+GAP1-M+GAP1-C+polar_tail+TM+beta-rich*->

■ **GCA_900112925.1; Paenibacillus algorifonticola; counts= 1; multicellularity status: TRUE**

- TM+vWA(canonical)+YtKA-like+TM+??->Tubulin+coiled_coil+??->Tubulin+coiled_coil+HTH?+??->TM+TM+TM+vWA(canonical)+TM+TM+TM+polar_tail->Fibronectin-like_repeats+??->ZnR+ZnR+DO-GTPase2.2+ZnR+ZnR->GAP1-N2+GAP1-M+GAP1-C+polar_tail+TM+beta-rich*->

■ **GCA_000981585.1; Paenibacillus riograndensis; counts= 1; multicellularity status: TRUE**

- TM+vWA(canonical)+YtKA-like+TM+??->Tubulin+coiled_coil+??->Tubulin+coiled_coil+HTH?+??->TM+TM+TM+vWA(canonical)+TM+TM+TM+polar_tail->Fibronectin-like_repeats+??->ZnR+ZnR+DO-GTPase2.2+ZnR+ZnR->GAP1-N2+GAP1-M+GAP1-C+polar_tail+TM+beta-rich*->

■ **GCF_000993825.1; Paenibacillus durus; counts= 1; multicellularity status: TRUE**

- TM+vWA(canonical)+YtKA-like+TM+??->Tubulin+coiled_coil+??->Tubulin+coiled_coil+HTH?+??->TM+TM+TM+vWA(canonical)+TM+TM+TM+polar_tail->Fibronectin-like_repeats+??->ZnR+ZnR+DO-GTPase2.2+ZnR+ZnR->GAP1-N2+GAP1-M+GAP1-C+polar_tail+TM+beta-rich*->

■ **GCF_000802655.2; Paenibacillus sp. IHB B 3415; counts= 1; multicellularity status: TRUE**

- TM+vWA(canonical)+YtKA-like+TM+??->Tubulin+coiled_coil+??->Tubulin+coiled_coil+HTH?+??->TM+TM+TM+vWA(canonical)+TM+TM+TM+polar_tail->Fibronectin-like_repeats+??->?->ZnR+ZnR+DO-GTPase2.2+ZnR+ZnR->GAP1-N2+GAP1-M+GAP1-C+polar_tail+TM+beta-rich*->

■ **GCA_001263295.1; Agrobacterium sp. SUL3; counts= 1; multicellularity status: FALSE**

- Calcineurin+GAPEAD1->GAP2->ZnR+DO-GTPase2.1->DO-GTPase1->GAP1-N1+GAP1-M+GAP1-C*->

■ **GCA_001020985.1; Corynebacterium mustelae; counts= 1; multicellularity status: FALSE**

- polar_leader+TM+TM+TM->TM+vWA(canonical)+Fibronectin-like_repeats+TM+beta-rich+lc_tail->Tubulin+alpha_helical+??+??->GAP_unknown1+TM+TM+GAP_unknown2->HRDC-like?+Fibronectin-like_repeats->GAP1-N2+GAP1-M+low_complexity+TM+TM*->beta-rich+vWA(canonical)->??+DO-GTPase2.2->polar_leader+TM+TM+TM->

■ **GCF_000702505.1; Thermogemmatispora carboxidivorans; counts= 1; multicellularity status: TRUE**

- ZnR+ZnR+DO-GTPase2.2->GAP1-N2+GAP1-M+GAP1-C*->

■ **GCF_001015055.1; Aneurinibacillus tyrosinisolvens; counts= 1; multicellularity status: FALSE**

- TM+vWA(canonical)+YtKA-like+TM+??->Tubulin+coiled_coil+??->Tubulin+coiled_coil+HTH?+??->TM+TM+TM+vWA(canonical)+TM+TM+TM+polar_tail(frag)->Fibronectin-like_repeats+??->ZnR+ZnR+DO-GTPase2.2+ZnR+ZnR->GAP1-N2+GAP1-M+GAP1-C+polar_tail+TM+beta-rich*->

■ **GCF_000988225.1; Corynebacterium striatum; counts= 1; multicellularity status: FALSE**

- polar_leader+TM+TM+TM->TM+vWA(canonical)+Fibronectin-like_repeats+TM+beta-rich+lc_tail->PSE->GAP_unknown1+TM+TM+GAP_unknown2->HRDC-like?+Fibronectin-like_repeats->GAP1-N2+GAP1-M+GAP1-C*->beta-rich+vWA(canonical)->??+DO-GTPase2.2->polar_leader+TM+TM+TM->

■ **GCA_002803965.1; Corynebacterium striatum; counts= 1; multicellularity status: FALSE**

- polar_leader+TM+TM+TM->TM+vWA(canonical)+Fibronectin-like_repeats+TM+beta-rich+lc_tail->Tubulin+alpha_helical+??+??->GAP_unknown1+TM+TM+GAP_unknown2->HRDC-like?+Fibronectin-like_repeats->GAP1-N2+GAP1-M+GAP1-C*->beta-rich+vWA(canonical)->??+DO-GTPase2.2->polar_leader+TM+TM+TM->

■ **GCF_001059955.1; Corynebacterium striatum; counts= 1; multicellularity status: FALSE**

- GAP1-N2+GAP1-M+GAP1-C*->beta-rich+vWA(canonical)->??+DO-GTPase2.2->polar_leader+TM+TM+TM->

■ **GCF_001058455.1; Corynebacterium striatum; counts= 1; multicellularity status: FALSE**

- polar_leader+TM+TM+TM->TM+vWA(canonical)+Fibronectin-like_repeats+TM+beta-rich+lc_tail->Tubulin+alpha_helical+??+??->GAP_unknown1+TM+TM+GAP_unknown2->HRDC-like?+Fibronectin-like_repeats->GAP1-N2+GAP1-M+GAP1-C*->beta-rich+vWA(canonical)->??+DO-GTPase2.2->polar_leader+TM+TM+TM->

■ **GCA_001270565.1; Streptomyces virginiae; counts= 1; multicellularity status: TRUE**

- TPR_containing_alpha_helical+Fibronectin-like_repeats->PSE->GAP1-N2+GAP1-M+GAP1-C*->PSE->vWA(canonical)->PP2C->Pkinase+ZnR+beta-rich->

■ **GCA_001270635.1; Streptomyces antibioticus; counts= 1; multicellularity status: TRUE**

- TPR_containing_alpha_helical+Fibronectin-like_repeats->ZnR+ZnR+DO-GTPase2.2->GAP1-N2+GAP1-M+GAP1-C*->PSE->vWA(canonical)->PP2C->Pkinase+ZnR+beta-rich->

■ **GCF_001270375.1; Clostridium sp. DMHC 10; counts= 1; multicellularity status: NA**

- TM+TM+TM+TM+ZnR+ZnR+DO-GTPase2.2->TM+vWA(canonical)+YtKA-like+TM+??->PSE->?->Tubulin+coiled_coil+HTH?+??->TM+TM+TM+vWA(canonical)+TM+TM+TM+polar_tail->Fibronectin-like_repeats+??->ZnR+ZnR+DO-GTPase2.2+ZnR+ZnR->GAP1-N2+GAP1-M*->

■ **GCA_001272655.2; Paenibacillus peoriae; counts= 1; multicellularity status: TRUE**

- TM+vWA(canonical)+YtKA-like+TM+??->Tubulin+coiled_coil+??->Tubulin+coiled_coil+HTH?+??->TM+TM+TM+vWA(canonical)+TM+TM+TM+polar_tail->Fibronectin-like_repeats+??->ZnR+ZnR+DO-GTPase2.2+ZnR+ZnR->GAP1-N2+GAP1-M+GAP1-C+polar_tail+TM+beta-rich*->

■ **GCF_001273715.1; Vibrio alginolyticus; counts= 1; multicellularity status: FALSE**

- PNP_UDP_1+EAD1->GAP2->ZnR(fragment)+DO-GTPase2.1->DO-GTPase1->GAP1-N1+GAP1-M+GAP1-C+EAD1*->SLATT->

■ **GCF_001273905.1; Paenibacillus sp. FJAT-27812; counts= 1; multicellularity status: TRUE**

- TM+vWA(canonical)+YtKA-like+TM+??->Tubulin+coiled_coil+??->Tubulin+coiled_coil+HTH?+??->TM+TM+TM+vWA(canonical)+TM+TM+TM+polar_tail->Fibronectin-like_repeats+??->ZnR+ZnR+DO-GTPase2.2+ZnR+ZnR->GAP1-N2+GAP1-M+GAP1-C+polar_tail+TM+beta-rich*->

■ **GCA_001275655.1; Bacillus sp. FJAT-21945; counts= 1; multicellularity status: NA**

- TM+vWA(canonical)+YtKA-like+TM+??->Tubulin+coiled_coil+??->Tubulin+coiled_coil+HTH?+??->TM+TM+TM+vWA(canonical)+TM+TM+TM+polar_tail->Fibronectin-like_repeats+??->ZnR+ZnR+DO-GTPase2.2+ZnR+ZnR->GAP1-N2+GAP1-M+GAP1-C+polar_tail+TM+beta-rich*->

■ **GCA_001275735.1; Pseudomonas coronafaciens; counts= 1; multicellularity status: FALSE**

- GAP2->DO-GTPase2.1->DO-GTPase1->GAP1-N1+GAP1-M+GAP1-C+EAD1*->CASPASE+EAD1->

■ **GCF_001275645.1; Bacillus sp. FJAT-18019; counts= 1; multicellularity status: NA**

- Tubulin+coiled_coil+??->Tubulin+coiled_coil+HTH?+??->TM+TM+TM+vWA+TM+TM+TM+polar_tail->Fibronectin-like_repeats+??->ZnR+ZnR+DO-GTPase2.2->GAP1-N2+GAP1-M+GAP1-C+polar_tail*->

■ **GCF_001278535.1; Caballeronia cordobensis; counts= 1; multicellularity status: FALSE**

- Calcineurin+GAPEAD1->GAP2->ZnR+DO-GTPase2.1->DO-GTPase1->GAP1-N1+GAP1-M+GAP1-C*->

■ **GCA_001279585.1; Nocardiopsis sp. NRRL B-16309; counts= 1; multicellularity status: TRUE**

- GAP1-N2+GAP1-M+GAP1-C*->

■ **GCF_000347295.2; Gordonia sp. NB4-1Y; counts= 1; multicellularity status: FALSE**

- Tubulin+alpha_helical+??+??->GAP_unknown1+TM+TM+GAP_unknown2->HRDC-like?+Fibronectin-like_repeats->HRDC-like?+Fibronectin-like_repeats->GAP1-N2+GAP1-M+GAP1-C(frag)*->?->beta-rich+vWA(canonical)->??+DO-GTPase2.2->

■ **GCF_001280595.1; Paenibacillus sp. A59; counts= 1; multicellularity status: TRUE**

- TM+TM+TM+vWA+TM+TM+TM+polar_tail->Fibronectin-like_repeats+??->ZnR+ZnR+DO-GTPase2.2->GAP1-N2+GAP1-M+GAP1-C+polar_tail*->

■ **GCA_900099955.1; Halomonas; counts= 1; multicellularity status: FALSE**

- GAP2->DO-GTPase2.1->PSE->GAP1-N1+GAP1-M+GAP1-C+EAD1*->CASPASE+EAD1->

■ **GCF_001280845.1; Bacillus sp. FJAT-28004; counts= 1; multicellularity status: NA**

- TM+vWA(canonical)+YtKA-like+TM+??->Tubulin+coiled_coil+??->Tubulin+coiled_coil+HTH?+??->TM+TM+TM+vWA(canonical)+TM+TM+TM+polar_tail->Fibronectin-like_repeats+??->ZnR+ZnR+DO-GTPase2.2+ZnR+ZnR->GAP1-N2+GAP1-M+GAP1-C+polar_tail+TM+beta-rich*->

■ **GCF_001292845.1; Rhodococcus jostii; counts= 1; multicellularity status: TRUE**

- polar_leader+TM+TM+TM->TM+vWA(canonical)+Fibronectin-like_repeats+TM+beta-rich+lc_tail->Tubulin+alpha_helical+??+??->GAP_unknown1+TM+TM+GAP_unknown2->HRDC-like?+Fibronectin-like_repeats->GAP1-N2+GAP1-M+GAP1-C*->beta-rich+vWA(canonical)->??+DO-GTPase2.2->

■ **GCA_001302585.1; Kibdelosporangium phytohabitans; counts= 1; multicellularity status: TRUE**

- Pkinase+TM->ZnR+ZnR+DO-GTPase2.2->GAP1-N2+GAP1-M*->

■ **GCF_001277345.1; Paenibacillus solani; counts= 1; multicellularity status: TRUE**

- TM+vWA(canonical)+YtKA-like+TM+??->Tubulin+coiled_coil+??->Tubulin+coiled_coil+HTH?+??->TM+TM+TM+vWA(canonical)+TM+TM+TM+polar_tail->Fibronectin-like_repeats+??->ZnR+ZnR+DO-GTPase2.2+ZnR+ZnR->GAP1-N2+GAP1-M+GAP1-C+polar_tail+TM+beta-rich*->

■ **GCA_001306465.1; Frankia sp. R43; counts= 1; multicellularity status: TRUE**

- GAP1-N2+GAP1-M+GAP1-C+ZnR+DO-GTPase2*->

■ **GCF_001312305.1; Paenibacillus pinihumi; counts= 1; multicellularity status: TRUE**

- Tubulin+coiled_coil+HTH?+??->TM+TM+TM+vWA+TM+TM+TM+polar_tail->Fibronectin-like_repeats+??->ZnR+ZnR+DO-GTPase2.2->GAP1-N2+GAP1-M+GAP1-C*->

- **GCF_001310955.1; Marinifilum fragile; counts= 1; multicellularity status: TRUE**
  - GrpE->HSP70->DnaJ+DnaJ+ZnR+ZnR+coiled_coil+Fibronectin-like_repeats->ZnR+ZnR+DO-GTPase2.2->GAP1-N2+GAP1-M+GAP1-C*->?->TM+TM+ZnR+ZnR+DO-GTPase2.2+TM+SH3->FGS-fold_domain->
- **GCF_001310595.1; Bacillus sp. JCM 19035; counts= 1; multicellularity status: NA**
  - GAP1-N2+GAP1-M+GAP1-C(frag)*->
- **GCA_001399685.1; Paenibacillus sp. A3; counts= 1; multicellularity status: TRUE**
  - TM+vWA(canonical)+YtKA-like+TM+??->Tubulin+coiled_coil+??->Tubulin+coiled_coil+HTH?+??->?->TM+TM+TM+vWA(canonical)+TM+TM+TM+polar_tail->Fibronectin-like_repeats+??->ZnR+ZnR+DO-GTPase2.2+ZnR+ZnR->GAP1-N2+GAP1-M+GAP1-C+polar_tail+TM+Cadherin-like*->
- **GCF_001403875.1; Paenibacillus ihumii; counts= 1; multicellularity status: TRUE**
  - TM+vWA(canonical)+YtKA-like+TM+??->Tubulin+coiled_coil+??->Tubulin+coiled_coil+HTH?+??->TM+TM+TM+vWA(canonical)+TM+TM+TM+polar_tail->Fibronectin-like_repeats+??->ZnR+ZnR+DO-GTPase2.2+ZnR+ZnR->GAP1-N2+GAP1-M+GAP1-C+polar_tail+TM+beta-rich*->
- **GCF_001373335.1; Ralstonia solanacearum; counts= 1; multicellularity status: FALSE**
  - Calcineurin+GAPEAD1->GAP2->ZnR+DO-GTPase2.1->DO-GTPase1->GAP1-N1+GAP1-M+GAP1-C*->
- **GCF_001418465.1; Streptomyces kanamyceticus; counts= 1; multicellularity status: TRUE**
  - GAP1-N2+GAP1-M+GAP1-C*->vWA(canonical)->PP2C->Pkinase+ZnR+beta-rich->||<-TPR_containing_alpha_helical+Fibronectin-like_repeats
- **GCF_001419745.1; Streptomyces silaceus; counts= 1; multicellularity status: TRUE**
  - GAP1-N2+GAP1-M+GAP1-C*->vWA(canonical)->PP2C->Pkinase+ZnR+beta-rich->
- **GCA_001420695.1; Brevibacillus choshinensis; counts= 1; multicellularity status: FALSE**
  - TM+vWA(canonical)+YtKA-like+TM+??->Tubulin+coiled_coil+??->Tubulin+coiled_coil+HTH?+??->TM+TM+TM+vWA(canonical)+TM+TM+TM+polar_tail->Fibronectin-like_repeats+??->ZnR+ZnR+DO-GTPase2.2+ZnR+ZnR->GAP1-N2+GAP1-M+GAP1-C+polar_tail+TM+beta-rich*->
- **GCA_001422685.1; Paenibacillus sp. Leaf72; counts= 1; multicellularity status: TRUE**
  - Tubulin+coiled_coil+??->Tubulin+coiled_coil+HTH?+??->TM+TM+TM+vWA+TM+TM+TM+polar_tail->Fibronectin-like_repeats+??->ZnR+ZnR+DO-GTPase2.2->GAP1-N2+GAP1-M+GAP1-C+polar_tail*->
- **GCF_001426045.1; Methylobacterium sp. Leaf94; counts= 1; multicellularity status: TRUE**
  - GAP1-N1+GAP1-M+GAP1-C*->
- **GCA_001422775.1; Methylobacterium sp. Leaf87; counts= 1; multicellularity status: TRUE**
  - GAP1-N1+GAP1-M+GAP1-C+EAD1*->CASPASE+EAD1->
- **GCA_001428625.1; Hydrogenophaga sp. Root209; counts= 1; multicellularity status: FALSE**
  - Calcineurin+GAPEAD1->GAP2->ZnR+DO-GTPase2.1->PSE->GAP1-N1+GAP1-M+GAP1-C*->
- **GCA_001423865.2; Brevibacillus sp. Leaf182; counts= 1; multicellularity status: FALSE**
  - TM+vWA(canonical)+YtKA-like+TM+??->Tubulin+coiled_coil+??->Tubulin+coiled_coil+HTH?+??->TM+TM+TM+vWA(canonical)+TM+TM+TM+polar_tail->Fibronectin-like_repeats+??->ZnR+ZnR+DO-GTPase2.2+ZnR+ZnR->GAP1-N2+GAP1-M+GAP1-C+polar_tail+TM+beta-rich*->
- **GCA_001428245.1; Paenibacillus sp. Soil522; counts= 1; multicellularity status: TRUE**
  - TM+vWA(canonical)+YtKA-like+TM+??->Tubulin+coiled_coil+??->Tubulin+coiled_coil+HTH?+??->HRDC-like?+Fibronectin-like_repeats->Fibronectin-like_repeats+??->ZnR+ZnR+DO-GTPase2.2+ZnR+ZnR->GAP1-N2+GAP1-M+GAP1-C+polar_tail+TM+beta-rich*->
- **GCA_001420595.1; Bacillus solani; counts= 1; multicellularity status: FALSE**

- TM+vWA(canonical)+YtKA-like+TM+??->Tubulin+coiled_coil+??->Tubulin+coiled_coil+HTH?+??->TM+TM+TM+vWA(canonical)+TM+TM+TM+polar_tail->Fibronectin-like_repeats+??->ZnR+ZnR+DO-GTPase2.2+ZnR+ZnR->GAP1-N2+GAP1-M+GAP1-C+polar_tail+TM+beta-rich*->

■ **GCA_001421665.1; Sphingobium sp. Leaf26; counts= 1; multicellularity status: FALSE**

- GAP2->ZnR+DO-GTPase2.1->DO-GTPase1->GAP1-N1+GAP1-M+GAP1-C+EAD1*->CASPASE+EAD1->

■ **GCA_001426325.1; Streptomyces; counts= 1; multicellularity status: TRUE**

- GAP1-N2+GAP1-M+GAP1-C*->

■ **GCA_001426865.1; Paenibacillus sp. Root52; counts= 1; multicellularity status: TRUE**

- TM+vWA(canonical)+YtKA-like+TM+??->Tubulin+coiled_coil+??->Tubulin+coiled_coil+HTH?+??->TM+TM+TM+vWA(canonical)+TM+TM+TM+polar_tail->Fibronectin-like_repeats+??->ZnR+ZnR+DO-GTPase2.2+ZnR+ZnR->GAP1-N2+GAP1-M+GAP1-C+polar_tail+TM+beta-rich*->

■ **GCA_001428565.1; Nocardioides sp. Root190; counts= 1; multicellularity status: TRUE**

- polar_leader+TM+TM+TM->TM+vWA(canonical)+Fibronectin-like_repeats+TM+beta-rich+lc_tail->Tubulin+alpha_helical+??+??->GAP_unknown1+TM+TM+GAP_unknown2->?->HRDC-like?+Fibronectin-like_repeats->GAP1-N2+GAP1-M+GAP1-C*->?->??+DO-GTPase2.2->

■ **GCA_001426615.1; Streptomyces; counts= 1; multicellularity status: TRUE**

- GAP1-N2+GAP1-M+GAP1-C*->

■ **GCA_001429505.1; Bosea sp. Root483D1; counts= 1; multicellularity status: NA**

- EAD1-M1+GAP1-C*->

■ **GCF_001425705.1; Pelomonas sp. Root1217; counts= 1; multicellularity status: FALSE**

- Calcineurin+GAPEAD1->GAP2->ZnR+DO-GTPase2.1->DO-GTPase1->GAP1-N1+GAP1-M+GAP1-C*->

■ **GCA_001401405.1; Pseudomonas syringae group genomosp. 3; counts= 1; multicellularity status: FALSE**

- GAP2->DO-GTPase2.1->DO-GTPase1->GAP1-N1+GAP1-M+GAP1-C+EAD1*->CASPASE+EAD1->

■ **GCA_001401265.1; Pseudomonas amygdali; counts= 1; multicellularity status: FALSE**

- Calcineurin+GAPEAD1->GAP2->ZnR+DO-GTPase2.1->DO-GTPase1->GAP1-N1+GAP1-M+GAP1-C*->

■ **GCA_001431425.1; Stenotrophomonas nitritireducens; counts= 1; multicellularity status: FALSE**

- Calcineurin+GAPEAD1->GAP2->DO-GTPase2.1->DO-GTPase1->GAP1-N1+GAP1-M+GAP1-C*->

■ **GCA_001434355.1; Streptomyces anulatus; counts= 1; multicellularity status: TRUE**

- GAP1-N2+GAP1-M+GAP1-C*->

■ **GCF_001439605.1; Bacillus praedii; counts= 1; multicellularity status: FALSE**

- TM+vWA(canonical)+YtKA-like+TM+??->Tubulin+coiled_coil+??->Tubulin+coiled_coil+HTH?+??->TM+TM+TM+vWA(canonical)+TM+TM+TM+polar_tail->Fibronectin-like_repeats+??->ZnR+ZnR+DO-GTPase2.2+ZnR+ZnR->GAP1-N2+GAP1-M+GAP1-C+polar_tail+TM+beta-rich*->

■ **GCA_001444405.1; Pseudoalteromonas phenolica; counts= 1; multicellularity status: FALSE**

- PNP_UDP_1+EAD1->GAP2->ZnR(fragment)+DO-GTPase2.1->DO-GTPase1->GAP1-N1+GAP1-M+GAP1-C+EAD1*->SLATT->

■ **GCA_001457595.1; Mycolicibacterium smegmatis; counts= 1; multicellularity status: FALSE**

- HRDC-like?+Fibronectin-like_repeats->GAP1-N2+GAP1-M+GAP1-C*->??+DO-GTPase2.2->

■ **GCA_001458375.1; Tropicibacter naphthalenivorans; counts= 1; multicellularity status: FALSE**

- GAP2->ZnR+DO-GTPase2.1->DO-GTPase1->GAP1-N1+GAP1-M+GAP1-C+EAD1*->CASPASE+EAD1->

■ **GCF_001457415.1; Gorillibacterium timonense; counts= 1; multicellularity status: FALSE**

- TM+vWA(canonical)+YtKA-like+TM+??->Tubulin+coiled_coil+??->Tubulin+coiled_coil+HTH?+??->TM+TM+TM+vWA(canonical)+TM+TM+TM+polar_tail->Fibronectin-like_repeats+??->ZnR+ZnR+DO-GTPase2.2+ZnR+ZnR->GAP1-N2+GAP1-M+GAP1-C+polar_tail+TM+beta-rich*->

■ **GCA_001467145.1; Pseudomonas; counts= 1; multicellularity status: FALSE**

- Calcineurin+GAPEAD1->GAP2->ZnR+DO-GTPase2.1->DO-GTPase1->GAP1-N1+GAP1-M+GAP1-C*->

■ **GCA_001467105.1; Pseudomonas syringae; counts= 1; multicellularity status: FALSE**

- GAP2->DO-GTPase2.1->DO-GTPase1->GAP1-N1+GAP1-M+GAP1-C+EAD1*->CASPASE+EAD1->

■ **GCF_001469165.1; Thiohalocapsa sp. ML1; counts= 1; multicellularity status: TRUE**

- GrpE->HSP70->HSP70+vWA(canonical)->?->ZnR+ZnR+DO-GTPase2.2->GAP1-N2+GAP1-M+GAP1-C*->?->TIR->TM+ZnR+ZnR+DO-GTPase2.2+PEGA+PEGA->

■ **GCA_001476655.1; Microbacterium testaceum; counts= 1; multicellularity status: FALSE**

- GrpE->HSP70->TPR_containing_alpha_helical+Fibronectin-like_repeats->ZnR+ZnR+DO-GTPase2.2->GAP1-N2+GAP1-M+GAP1-C*->TM+TM+TM+TM+ZnR+ZnR+DO-GTPase2.2->

■ **GCA_002872435.1; Paenibacillus; counts= 1; multicellularity status: TRUE**

- TM+vWA(canonical)+YtKA-like+TM+??->Tubulin+coiled_coil+??->Tubulin+coiled_coil+HTH?+??->TM+TM+TM+vWA(canonical)+TM+TM+TM+polar_tail->Fibronectin-like_repeats+??->ZnR+ZnR+DO-GTPase2.2+ZnR+ZnR->GAP1-N2+GAP1-M+GAP1-C+polar_tail+TM+beta-rich*->

■ **GCA_004219785.1; Salmonella enterica; counts= 1; multicellularity status: FALSE**

- Calcineurin+GAPEAD1->GAP2->ZnR+DO-GTPase2.1->DO-GTPase1->GAP1-N1+GAP1-M+GAP1-C*->

■ **GCF_001049835.1; Paenibacillus; counts= 1; multicellularity status: TRUE**

- TM+vWA(canonical)+YtKA-like+TM+??->Tubulin+coiled_coil+??->Tubulin+coiled_coil+HTH?+??->TM+TM+TM+vWA(canonical)+TM+TM+TM+polar_tail->Fibronectin-like_repeats+??->ZnR+ZnR+DO-GTPase2.2+ZnR+ZnR->GAP1-N2+GAP1-M+GAP1-C+polar_tail+TM+beta-rich*->

■ **GCF_001493375.1; Streptomyces specialis; counts= 1; multicellularity status: TRUE**

- GAP1-N2+GAP1-M+GAP1-C*->

■ **GCF_001485495.1; Gordonia desulfuricans; counts= 1; multicellularity status: FALSE**

- Tubulin+alpha_helical+??+??->GAP_unknown1+TM+TM+GAP_unknown2->HRDC-like?+Fibronectin-like_repeats->GAP1-N2+GAP1-M+GAP1-C*->beta-rich+vWA(canonical)->??+DO-GTPase2.2->

■ **GCA_001482305.1; Leucobacter sp. G161; counts= 1; multicellularity status: TRUE**

- GrpE->HSP70->TPR_containing_alpha_helical+Fibronectin-like_repeats->ZnR+ZnR+DO-GTPase2.2->GAP1-N2+GAP1-M+GAP1-C*->TM+TM+TM+TM+ZnR+ZnR+DO-GTPase2.2->?->?->HSP70->vWA(canonical)->

■ **GCA_001499965.1; Mycobacterium sp. GA-2829; counts= 1; multicellularity status: FALSE**

- HRDC-like?+Fibronectin-like_repeats->GAP1-N2+GAP1-M+GAP1-C*->??+DO-GTPase2.2->

■ **GCA_001500125.1; Mycobacterium sp. IS-1742; counts= 1; multicellularity status: FALSE**

- HRDC-like?+Fibronectin-like_repeats->GAP1-N2+GAP1-M+GAP1-C*->??+DO-GTPase2.2->

■ **GCA_001499995.1; Mycobacterium sp. IS-1496; counts= 1; multicellularity status: FALSE**

- HRDC-like?+Fibronectin-like_repeats->GAP1-N2+GAP1-M+GAP1-C*->??+DO-GTPase2.2->

■ **GCA_001509565.1; Streptomyces griseus; counts= 1; multicellularity status: TRUE**

- GAP1-N2+GAP1-M+GAP1-C*->

■ **GCA_001511815.1; Streptomyces reticuli; counts= 1; multicellularity status: TRUE**

- TPR_containing_alpha_helical+Fibronectin-like_repeats->ZnR+ZnR+DO-GTPase2.2->GAP1-N2+GAP1-M+GAP1-C*->TM+TM+TM+TM+ZnR+ZnR+DO-GTPase2.2+TM->vWA(canonical)->PP2C->Pkinase+ZnR+beta-rich->

■ **GCA_001522865.1; Burkholderia sp. TSV86; counts= 1; multicellularity status: FALSE**

- Calcineurin+GAPEAD1->GAP2->DO-GTPase2.1->DO-GTPase1->GAP1-N1+GAP1-M+GAP1-C*->

■ **GCA_001522035.1; Burkholderia ubonensis; counts= 1; multicellularity status: FALSE**

- Calcineurin+GAPEAD1->GAP2->ZnR+DO-GTPase2.1->DO-GTPase1->GAP1-N1+GAP1-M+GAP1-C*->

■ **GCA_001526205.1; Burkholderia cepacia; counts= 1; multicellularity status: FALSE**

- Calcineurin+GAPEAD1->GAP2->ZnR+DO-GTPase2.1->DO-GTPase1->GAP1-N1+GAP1-M+GAP1-C*->

■ **GCA_001528175.1; Burkholderia ubonensis; counts= 1; multicellularity status: FALSE**

- Calcineurin+GAPEAD1->GAP2->ZnR+DO-GTPase2.1->DO-GTPase1->GAP1-N1+GAP1-M+GAP1-C*->

■ **GCF_001528875.1; Burkholderia ubonensis; counts= 1; multicellularity status: FALSE**

- Calcineurin+GAPEAD1->GAP2->ZnR+DO-GTPase2.1->DO-GTPase1->GAP1-N1+GAP1-M+GAP1-C*->

■ **GCA_001527825.1; Burkholderia stagnalis; counts= 1; multicellularity status: FALSE**

- Calcineurin+GAPEAD1->GAP2->ZnR+DO-GTPase2.1->DO-GTPase1->GAP1-N1+GAP1-M+GAP1-C*->

■ **GCA_001529865.1; Burkholderia ubonensis; counts= 1; multicellularity status: FALSE**

- Calcineurin+GAPEAD1->||<-?||DO-GTPase2.1->DO-GTPase1->GAP1-N1+GAP1-M+GAP1-C*->

■ **GCA_001533325.1; Burkholderia ubonensis; counts= 1; multicellularity status: FALSE**

- GAP2->DO-GTPase2.1->DO-GTPase1->GAP1-N1+GAP1-M+GAP1-C+EAD1*->CASPASE+EAD1->

■ **GCA_001532865.1; Burkholderia ubonensis; counts= 1; multicellularity status: FALSE**

- Calcineurin+GAPEAD1->GAP2->ZnR+DO-GTPase2.1->DO-GTPase1->GAP1-N1+GAP1-M+GAP1-C*->

■ **GCA_001535945.1; Pseudomonas syringae; counts= 1; multicellularity status: FALSE**

- Calcineurin+GAPEAD1->GAP2->ZnR+DO-GTPase2.1->DO-GTPase1->GAP1-N1+GAP1-M+GAP1-C*->

■ **GCA_001421015.2; Paenibacillus bovis; counts= 1; multicellularity status: TRUE**

- Tubulin+coiled_coil+??->Tubulin+coiled_coil+HTH?+??->||<-?||TM+TM+TM+vWA(canonical)+TM+TM+TM+polar_tail->?->Fibronectin-like_repeats+??->ZnR+ZnR+DO-GTPase2.2+ZnR+ZnR->GAP1-N2+GAP1-M*->

■ **GCA_001012825.2; Paenibacillus etheri; counts= 1; multicellularity status: TRUE**

- TM+vWA(canonical)+YtKA-like+TM+??->Tubulin+coiled_coil+??->Tubulin+coiled_coil+HTH?+??->TM+TM+TM+vWA(canonical)+TM+TM+TM+polar_tail->Fibronectin-like_repeats+??->ZnR+ZnR+DO-GTPase2.2+ZnR+ZnR->GAP1-N2+GAP1-M+GAP1-C+polar_tail+TM+beta-rich*->

■ **GCF_001465325.1; Rhodococcus pyridinivorans; counts= 1; multicellularity status: TRUE**

- polar_leader+TM+TM+TM->TM+vWA(canonical)+Fibronectin-like_repeats+TM+beta-rich+lc_tail->Tubulin+alpha_helical+??+??->GAP_unknown1+TM+TM+GAP_unknown2->HRDC-like?+Fibronectin-like_repeats->GAP1-N2+GAP1-M+GAP1-C*->beta-rich+vWA(canonical)->??+DO-GTPase2.2->

■ **GCA_001541145.1; Streptomyces albus; counts= 1; multicellularity status: TRUE**

- TPR_containing_alpha_helical+Fibronectin-like_repeats->ZnR+ZnR+DO-GTPase2.2->GAP1-N2+GAP1-M+GAP1-C*->TM+TM+TM+TM+ZnR+ZnR+DO-GTPase2.2+TM->vWA(canonical)->PP2C->Pkinase+ZnR+beta-rich->

■ **GCA_001544575.2; Caballeronia cordobensis; counts= 1; multicellularity status: FALSE**

- Calcineurin+GAPEAD1->GAP2->ZnR+DO-GTPase2.1->DO-GTPase1->GAP1-N1+GAP1-M+GAP1-C*->

■ **GCF_001546065.1; Paenibacillus riograndensis; counts= 1; multicellularity status: TRUE**

- TM+vWA(canonical)+YtKA-like+TM+??->Tubulin+coiled_coil+??->Tubulin+coiled_coil+HTH?+??->TM+TM+TM+vWA(canonical)+TM+TM+TM+polar_tail->Fibronectin-like_repeats+??->ZnR+ZnR+DO-GTPase2.2+ZnR+ZnR->GAP1-N2+GAP1-M+GAP1-C+polar_tail+TM+beta-rich*->

■ **GCF_001550375.1; Streptomyces europaeiscabiei; counts= 1; multicellularity status: TRUE**

- GAP1-N2+GAP1-M+GAP1-C*->vWA(canonical)->PP2C->Pkinase+ZnR+beta-rich->

■ **GCA_001552955.1; Corynebacterium kroppenstedtii; counts= 1; multicellularity status: FALSE**

- polar_leader+TM+TM+TM->TM+vWA(canonical)+Fibronectin-like_repeats+TM+beta-rich+lc_tail->Tubulin+alpha_helical+??+??->GAP_unknown1+TM+TM+GAP_unknown2->HRDC-like?+Fibronectin-like_repeats->GAP1-N2+GAP1-M+GAP1-C*->beta-rich+vWA(canonical)->??+DO-GTPase2.2->

■ **GCA_001553935.1; Actinomyces oris; counts= 1; multicellularity status: FALSE**

- polar_leader+TM+TM+TM->TM+vWA(canonical)+Fibronectin-like_repeats+TM+beta-rich+lc_tail->Tubulin+alpha_helical+??+??->GAP_unknown1+TM+TM+GAP_unknown2->HRDC-like?+Fibronectin-like_repeats->GAP1-N2+GAP1-M+GAP1-C*->?->??+DO-GTPase2.2->

■ **GCA_001555475.1; Rhodococcus sp. SC4; counts= 1; multicellularity status: TRUE**

- polar_leader+TM+TM+TM->TM+vWA(canonical)+Fibronectin-like_repeats+TM+beta-rich+lc_tail->Tubulin+alpha_helical+??+??->GAP_unknown1+TM+TM+GAP_unknown2->HRDC-like?+Fibronectin-like_repeats->GAP1-N2+GAP1-M+GAP1-C*->beta-rich+vWA(canonical)->??+DO-GTPase2.2->

■ **GCF_001568445.1; Leptospira borgpetersenii; counts= 1; multicellularity status: FALSE**

- Calcineurin+GAPEAD1->GAP2->ZnR+DO-GTPase2.1->DO-GTPase1->GAP1-N1+GAP1-M+GAP1-C*->

■ **GCF_001569365.1; Leptospira weilii; counts= 1; multicellularity status: FALSE**

- Calcineurin+GAPEAD1->GAP2->ZnR+DO-GTPase2.1->DO-GTPase1->GAP1-N1+GAP1-M+GAP1-C*->

■ **GCA_001577385.1; Streptomyces albus; counts= 1; multicellularity status: TRUE**

- GAP1-N2+GAP1-M+GAP1-C*->

■ **GCA_001583455.1; Rhodococcus sp. LB1; counts= 1; multicellularity status: TRUE**

- TM+vWA(canonical)+Fibronectin-like_repeats+TM+beta-rich+lc_tail->Tubulin+alpha_helical+??+??->GAP_unknown1+TM+TM+GAP_unknown2->HRDC-like?+Fibronectin-like_repeats->GAP1-N2+GAP1-M+GAP1-C*->beta-rich+vWA(canonical)->??+DO-GTPase2.2->

■ **GCA_900445325.1; Bacillus circulans; counts= 1; multicellularity status: FALSE**

- GAP1-N2+GAP1-M+GAP1-C+polar_tail+TM+beta-rich*->

■ **GCA_001593085.1; Paenibacillus polymyxa; counts= 1; multicellularity status: TRUE**

- Tubulin+coiled_coil+??->Tubulin+coiled_coil+HTH?+??->TM+TM+TM+vWA+TM+TM+TM+polar_tail->Fibronectin-like_repeats+??->ZnR+ZnR+DO-GTPase2.2->GAP1-N2+GAP1-M+GAP1-C+polar_tail*->

■ **GCA_001425445.1; Devosia sp. Leaf420; counts= 1; multicellularity status: FALSE**

- GAP2->ZnR+DO-GTPase2.1->DO-GTPase1->GAP1-N1+GAP1-M+GAP1-C+EAD1*->CASPASE+EAD1->CASPASE+??->

■ **GCA_001579275.1; Corynebacterium; counts= 1; multicellularity status: FALSE**

- polar_leader+TM+TM+TM->TM+vWA(canonical)+Fibronectin-like_repeats+TM+beta-rich+lc_tail->Tubulin+alpha_helical+??+??->GAP_unknown1+TM+TM+GAP_unknown2->HRDC-like?+Fibronectin-like_repeats->GAP1-N2+GAP1-M+GAP1-C*->beta-rich+vWA(canonical)->??+DO-GTPase2.2->polar_leader+TM+TM+TM->

■ **GCA_001586215.1; Corynebacterium simulans; counts= 1; multicellularity status: FALSE**

- polar_leader+TM+TM+TM->TM+vWA(canonical)+Fibronectin-like_repeats+TM+beta-rich+lc_tail->Tubulin+alpha_helical+??+??->GAP_unknown1+TM+TM+GAP_unknown2->HRDC-like?+Fibronectin-like_repeats->GAP1-N2+GAP1-M+GAP1-C*->beta-rich+vWA(canonical)->??+DO-GTPase2.2->polar_leader+TM+TM+TM->

■ **GCA_001586235.1; Corynebacterium simulans; counts= 1; multicellularity status: FALSE**

- polar_leader+TM+TM+TM->TM+vWA(canonical)+Fibronectin-like_repeats+TM+beta-rich+lc_tail->Tubulin+alpha_helical+??+??->GAP_unknown1+TM+TM+GAP_unknown2->HRDC-like?+Fibronectin-like_repeats->GAP1-N2+GAP1-M+GAP1-C*->beta-rich+vWA(canonical)->??+DO-GTPase2.2->polar_leader+TM+TM+TM->

■ **GCA_001513975.1; Streptomyces curacoi; counts= 1; multicellularity status: TRUE**

- TPR_containing_alpha_helical+Fibronectin-like_repeats->ZnR+ZnR+DO-GTPase2.2->GAP1-N2+GAP1-M+GAP1-C*->TM+TM+TM+TM+ZnR+ZnR+DO-GTPase2.2+TM->vWA(canonical)->PP2C->Pkinase+ZnR+beta-rich->

■ **GCF_001514495.1; Paenibacillus pabuli; counts= 1; multicellularity status: TRUE**

- TM+vWA(canonical)+YtKA-like+TM+??->Tubulin+coiled_coil+??->Tubulin+coiled_coil+HTH?+??->TM+TM+TM+vWA(canonical)+TM+TM+TM+polar_tail->Fibronectin-like_repeats+??->ZnR+ZnR+DO-GTPase2.2+ZnR+ZnR->GAP1-N2+GAP1-M+GAP1-C+polar_tail+TM+beta-rich*->

■ **GCF_001546055.1; Paenibacillus jilunlii; counts= 1; multicellularity status: TRUE**

- TM+vWA(canonical)+YtKA-like+TM+??->Tubulin+coiled_coil+??->Tubulin+coiled_coil+HTH?+??->?->TM+TM+TM+vWA(canonical)+TM+TM+TM+polar_tail->Fibronectin-like_repeats+??->ZnR+ZnR+DO-GTPase2.2+ZnR+ZnR->GAP1-N2+GAP1-M+GAP1-C+polar_tail+TM+beta-rich*->

■ **GCF_000971455.1; Lysinimicrobium rhizosphaerae; counts= 1; multicellularity status: FALSE**

- polar_leader+TM+TM+TM->TM+vWA(canonical)+Fibronectin-like_repeats+TM+beta-rich+lc_tail->Tubulin+alpha_helical+??+??->GAP_unknown1+TM+TM+GAP_unknown2->HRDC-like?+Fibronectin-like_repeats->GAP1-N2+GAP1-M+GAP1-C*->beta-rich+vWA(canonical)->??+DO-GTPase2.2->

■ **GCA_001509505.1; Streptomyces sp. NRRL S-1521; counts= 1; multicellularity status: TRUE**

- GAP1-N2+GAP1-M+GAP1-C*->vWA(canonical)->PP2C->Pkinase+ZnR+beta-rich->||<-TPR_containing_alpha_helical+Fibronectin-like_repeats

■ **GCA_003634525.1; Williamsia muralis; counts= 1; multicellularity status: TRUE**

- polar_leader+TM+TM+TM->TM+vWA(canonical)+Fibronectin-like_repeats+TM+beta-rich+lc_tail->Tubulin+alpha_helical+??+??->GAP_unknown1+TM+TM+GAP_unknown2->HRDC-like?+Fibronectin-like_repeats->GAP1-N2+GAP1-M+GAP1-C*->beta-rich+vWA(canonical)->??+DO-GTPase2.2->

■ **GCA_001570425.1; Mycolicibacterium brisbanense; counts= 1; multicellularity status: FALSE**

- TM+DUF4190+TM->polar_leader+TM+TM+TM->PSE->Tubulin+alpha_helical+??+??->GAP_unknown1+TM+TM+GAP_unknown2->HRDC-like?+Fibronectin-like_repeats->GAP1-N2+GAP1-M+GAP1-C*->beta-rich+vWA(canonical)->??+DO-GTPase2.2->TM+DUF4190+TM+PknH_C->TM+DUF4190+TM+PknH_C->TM+DUF4190+TM+PknH_C->

■ **GCF_001570725.1; Paenibacillus amylolyticus; counts= 1; multicellularity status: TRUE**

- TM+vWA(canonical)+YtKA-like+TM+??->Tubulin+coiled_coil+??->Tubulin+coiled_coil+HTH?+??->TM+TM+TM+vWA(canonical)+TM+TM+TM+polar_tail->Fibronectin-like_repeats+??->ZnR+ZnR+DO-GTPase2.2+ZnR+ZnR->GAP1-N2+GAP1-M+GAP1-C+polar_tail+TM+beta-rich*->

■ **GCA_003326775.1; Thalassospira xiamenensis; counts= 1; multicellularity status: FALSE**

- Calcineurin+GAPEAD1->GAP2->DO-GTPase2.1(fragment)->DO-GTPase1->GAP1-N1+GAP1-M+GAP1-C*->

■ **GCF_900075065.1; Enterobacteriaceae; counts= 1; multicellularity status: FALSE**

- Calcineurin+GAPEAD1->GAP2->ZnR+DO-GTPase2.1->DO-GTPase1->GAP1-N1+GAP1-M+GAP1-C*->

■ **GCA_001619725.1; Paenibacillus elgii; counts= 1; multicellularity status: TRUE**

- TM+vWA(canonical)+YtKA-like+TM+??->Tubulin+coiled_coil+??->Tubulin+coiled_coil+HTH?+??->?->TM+TM+TM+vWA(canonical)+TM+TM+TM+polar_tail->Fibronectin-like_repeats+??->ZnR+ZnR+DO-GTPase2.2+ZnR+ZnR->GAP1-N2+GAP1-M+GAP1-C+polar_tail+TM+Cadherin-like*->

■ **GCA_001619755.1; Paenibacillus jamilae; counts= 1; multicellularity status: TRUE**

- TM+vWA(canonical)+YtKA-like+TM+??->Tubulin+coiled_coil+??->Tubulin+coiled_coil+HTH?+??->TM+TM+TM+vWA(canonical)+TM+TM+TM+polar_tail->Fibronectin-like_repeats+??->ZnR+ZnR+DO-GTPase2.2+ZnR+ZnR->GAP1-N2+GAP1-M+GAP1-C+polar_tail+TM+beta-rich*->

■ **GCA_001619605.1; Brevibacillus parabrevis; counts= 1; multicellularity status: FALSE**

- TM+vWA(canonical)+YtKA-like+TM+??->Tubulin+coiled_coil+??->Tubulin+coiled_coil+HTH?+??->TM+TM+TM+vWA(canonical)+TM+TM+TM+polar_tail->Fibronectin-like_repeats+??->ZnR+ZnR+DO-GTPase2.2+ZnR+ZnR->GAP1-N2+GAP1-M+GAP1-C+polar_tail+TM+beta-rich*->

■ **GCA_001633025.1; Paenibacillus glucanolyticus; counts= 1; multicellularity status: TRUE**

- TM+vWA(canonical)+YtKA-like+TM+??->Tubulin+coiled_coil+??->Tubulin+coiled_coil+HTH?+??->TM+TM+TM+vWA(canonical)+TM+TM+TM+polar_tail->Fibronectin-like_repeats+??->ZnR+ZnR+DO-GTPase2.2+ZnR+ZnR->GAP1-N2+GAP1-M+GAP1-C+polar_tail+TM+beta-rich*->

■ **GCF_001636635.1; Paenibacillus sp. O199; counts= 1; multicellularity status: TRUE**

- Tubulin+coiled_coil+HTH?+??->TM+TM+TM+vWA(canonical)+TM+TM+TM+polar_tail->Fibronectin-like_repeats+??->ZnR+ZnR+DO-GTPase2.2+ZnR+ZnR->GAP1-N2+GAP1-M+GAP1-C+polar_tail+TM+beta-rich*->

■ **GCF_001650215.1; Streptomyces sp. RTd22; counts= 1; multicellularity status: TRUE**

- TPR_containing_alpha_helical+Fibronectin-like_repeats->?->ZnR+ZnR+DO-GTPase2.2->GAP1-N2+GAP1-M+GAP1-C*->TM+TM+TM+TM+ZnR+ZnR+DO-GTPase2.2+TM->vWA(canonical)->PP2C->Pkinase+ZnR+beta-rich->

■ **GCF_001418335.1; Streptomyces aurantiacus; counts= 1; multicellularity status: TRUE**

- TPR_containing_alpha_helical+Fibronectin-like_repeats->ZnR+ZnR+DO-GTPase2.2->GAP1-N2+GAP1-M+GAP1-C*->TM+TM+TM+TM+ZnR+ZnR+DO-GTPase2.2+TM->vWA(canonical)->PP2C->Pkinase+ZnR+beta-rich->

■ **GCA_001639025.1; Corynebacterium afermentans; counts= 1; multicellularity status: FALSE**

- polar_leader+TM+TM+TM->TM+vWA(canonical)+Fibronectin-like_repeats+TM+beta-rich+lc_tail->Tubulin+alpha_helical+??+??->GAP_unknown1+TM+TM+GAP_unknown2->HRDC-like?+Fibronectin-like_repeats->GAP1-N2+GAP1-M+GAP1-C*->beta-rich+vWA(canonical)->??+DO-GTPase2.2->?->||<-?<-?<-HSP70<-GrpE

■ **GCA_001643175.1; Dietzia cinnamea; counts= 1; multicellularity status: FALSE**

- GrpE->HSP70->TPR_containing_alpha_helical+Fibronectin-like_repeats->ZnR+ZnR+DO-GTPase2.2->GAP1-N2+GAP1-M+GAP1-C*->TM+TM+TM+TM+ZnR+ZnR+DO-GTPase2.2->?->HSP70->vWA(canonical)->

■ **GCA_001645205.1; Brevibacillus sp. SKDU10; counts= 1; multicellularity status: FALSE**

- TM+vWA(canonical)+YtKA-like+TM+??->Tubulin+coiled_coil+??->Tubulin+coiled_coil+HTH?+??->TM+TM+TM+vWA(canonical)+TM+TM+TM+polar_tail->Fibronectin-like_repeats+??->ZnR+ZnR+DO-GTPase2.2+ZnR+ZnR->GAP1-N2+GAP1-M+GAP1-C+polar_tail+TM+beta-rich*->

■ **GCA_900455725.1; Rhodococcus gordoniae; counts= 1; multicellularity status: TRUE**

- polar_leader+TM+TM+TM->TM+vWA(canonical)+Fibronectin-like_repeats+TM+beta-rich+lc_tail->Tubulin+alpha_helical+??+??->GAP_unknown1+TM+TM+GAP_unknown2->HRDC-like?+Fibronectin-like_repeats->GAP1-N2+GAP1-M+GAP1-C*->beta-rich+vWA(canonical)->??+DO-GTPase2.2->

■ **GCA_002152295.1; Klebsiella; counts= 1; multicellularity status: FALSE**

- Calcineurin+GAPEAD1->GAP2->ZnR+DO-GTPase2.1->DO-GTPase1->GAP1-N1+GAP1-M+GAP1-C*->

■ **GCF_001649505.1; Brevibacillus brevis; counts= 1; multicellularity status: FALSE**

- TM+vWA(canonical)+YtKA-like+TM+??->Tubulin+coiled_coil+??->Tubulin+coiled_coil+HTH?+??->TM+TM+TM+vWA(canonical)+TM+TM+TM+polar_tail->Fibronectin-like_repeats+??->ZnR+ZnR+DO-GTPase2.2+ZnR+ZnR->GAP1-N2+GAP1-M+GAP1-C+polar_tail+TM+beta-rich*->

■ **GCF_001546485.1; Gardnerella vaginalis; counts= 1; multicellularity status: FALSE**

- TM+TM+TM+TM+ZnR+ZnR+DO-GTPase2.2->?->HSP70->HSP70+vWA(canonical)->DnaJ+DnaJ+ZnR+ZnR+coiled_coil+Fibronectin-like_repeats->ZnR+ZnR+DO-GTPase2.2->GAP1-N2+GAP1-M+GAP1-C*->

■ **GCA_001659845.1; Paenibacillus sp. AD87; counts= 1; multicellularity status: TRUE**

- TM+TM+TM+vWA+TM+TM+TM+polar_tail->Fibronectin-like_repeats+??->ZnR+ZnR+DO-GTPase2.2->GAP1-N2+GAP1-M+GAP1-C+polar_tail*->

■ **GCA_001663525.1; Enterobacter asburiae; counts= 1; multicellularity status: FALSE**

- Calcineurin+GAPEAD1->GAP2->ZnR+DO-GTPase2.1->DO-GTPase1->GAP1-N1+GAP1-M+GAP1-C*->

■ **GCA_001663585.1; Paenibacillus polymyxa; counts= 1; multicellularity status: TRUE**

- Tubulin+coiled_coil+??->Tubulin+coiled_coil+HTH?+??->TM+TM+TM+vWA+TM+TM+TM+polar_tail->Fibronectin-like_repeats+??->ZnR+ZnR+DO-GTPase2.2->GAP1-N2+GAP1-M+GAP1-C+polar_tail*->

■ **GCA_001665445.1; Mycolicibacterium setense; counts= 1; multicellularity status: FALSE**

- HRDC-like?+Fibronectin-like_repeats->GAP1-N2+GAP1-M+GAP1-C*->??+DO-GTPase2.2->

■ **GCA_001665825.1; Mycobacterium sp. 852013-50142_SCH4511227; counts= 1; multicellularity status: FALSE**

- HRDC-like?+Fibronectin-like_repeats->GAP1-N2+GAP1-M+GAP1-C*->??+DO-GTPase2.2->

■ **GCA_001665335.1; Mycolicibacterium conceptionense; counts= 1; multicellularity status: FALSE**

- HRDC-like?+Fibronectin-like_repeats->GAP1-N2+GAP1-M+GAP1-C*->??+DO-GTPase2.2->

■ **GCA_001666795.1; Mycolicibacterium fortuitum; counts= 1; multicellularity status: FALSE**

- HRDC-like?+Fibronectin-like_repeats->GAP1-N2+GAP1-M+GAP1-C*->??+DO-GTPase2.2->

■ **GCA_001667465.1; Mycobacterium sp. 852002-10379_SCH5584320; counts= 1; multicellularity status: FALSE**

- HRDC-like?+Fibronectin-like_repeats->GAP1-N2+GAP1-M+GAP1-C*->??+DO-GTPase2.2->

■ **GCA_001666985.1; Mycolicibacterium fortuitum; counts= 1; multicellularity status: FALSE**

- HRDC-like?+Fibronectin-like_repeats->GAP1-N2+GAP1-M+GAP1-C*->??+DO-GTPase2.2->

■ **GCA_001665685.1; Mycobacterium sp. 852013-50091_SCH5140682; counts= 1; multicellularity status: FALSE**

- TM+DUF4190+TM->polar_leader+TM+TM+TM->PSE->Tubulin+alpha_helical+??+??->GAP_unknown1+TM+TM+GAP_unknown2->HRDC-like?+Fibronectin-like_repeats->GAP1-N2+GAP1-M+GAP1-C*->beta-rich+vWA(canonical)->??+DO-GTPase2.2->TM+DUF4190+TM+PknH_C->TM+DUF4190+TM+PknH_C->TM+DUF4190+TM+PknH_C->

■ **GCA_001662815.1; Paenibacillus polymyxa; counts= 1; multicellularity status: TRUE**

- TM+vWA(canonical)+YtKA-like+TM+??->Tubulin+coiled_coil+??->Tubulin+coiled_coil+HTH?+??->TM+TM+TM+vWA(canonical)+TM+TM+TM+polar_tail->Fibronectin-like_repeats+??->ZnR+ZnR+DO-GTPase2.2+ZnR+ZnR->GAP1-N2+GAP1-M+GAP1-C+polar_tail+TM+beta-rich*->

■ **GCA_001672365.1; Mesorhizobium loti; counts= 1; multicellularity status: TRUE**

- Calcineurin+GAPEAD1->GAP2->ZnR+DO-GTPase2.1->DO-GTPase1->GAP1-N1+GAP1-M+GAP1-C*->

■ **GCA_001672855.1; Mycolicibacterium fortuitum; counts= 1; multicellularity status: FALSE**

- HRDC-like?+Fibronectin-like_repeats->GAP1-N2+GAP1-M+GAP1-C*->??+DO-GTPase2.2->

■ **GCA_001672755.1; Mycobacterium colombiense; counts= 1; multicellularity status: FALSE**

- TM+DUF4190+TM->TM+DUF4190+TM->polar_leader+TM+TM+TM->TM+vWA(canonical)+Fibronectin-like_repeats+TM+beta-rich+lc_tail->Tubulin+alpha_helical+??+??->GAP_unknown1+TM+TM+GAP_unknown2->HRDC-like?+Fibronectin-like_repeats->GAP1-N2+GAP1-M+GAP1-C*->beta-rich+vWA(canonical)->??+DO-GTPase2.2->TM+DUF4190+TM+PknH_C->TM+DUF4190+TM+PknH_C->TM+DUF4190+TM+PknH_C->TM+DUF4190+TM+PknH_C->

■ **GCF_900079515.1; Shewanella sp. Alg231_23; counts= 1; multicellularity status: FALSE**

- PNP_UDP_1+EAD1->GAP2->ZnR(fragment)+DO-GTPase2.1->DO-GTPase1->GAP1-N1+GAP1-M+GAP1-C+EAD1*->SLATT->

■ **GCA_001680695.1; Paenibacillus sp. KS1; counts= 1; multicellularity status: TRUE**

- TM+vWA(canonical)+YtKA-like+TM+??->Tubulin+coiled_coil+??->Tubulin+coiled_coil+HTH?+??->TM+TM+TM+vWA(canonical)+TM+TM+TM+polar_tail->Fibronectin-like_repeats+??->PSE->GAP1-N2+GAP1-M+GAP1-C+polar_tail+TM+beta-rich*->

■ **GCA_001682715.1; Actinomyces oris; counts= 1; multicellularity status: FALSE**

- polar_leader+TM+TM+TM->TM+vWA(canonical)+Fibronectin-like_repeats+TM+beta-rich+lc_tail->Tubulin+alpha_helical+??+??->GAP_unknown1+TM+TM+GAP_unknown2->HRDC-like?+Fibronectin-like_repeats->GAP1-N2+GAP1-M+GAP1-C*->?->??+DO-GTPase2.2->

■ **GCA_001682865.1; Bacillus sp. FJAT-26390; counts= 1; multicellularity status: NA**

- TM+vWA(canonical)+YtKA-like+TM+??->Tubulin+coiled_coil+??->Tubulin+coiled_coil+HTH?+??->TM+TM+TM+vWA(canonical)+TM+TM+TM+polar_tail->Fibronectin-like_repeats+??->ZnR+ZnR+DO-GTPase2.2+ZnR+ZnR->GAP1-N2+GAP1-M+GAP1-C+polar_tail+TM+beta-rich*->

■ **GCA_001686835.1; Mycolicibacterium vulneris; counts= 1; multicellularity status: FALSE**

- HRDC-like?+Fibronectin-like_repeats->GAP1-N2+GAP1-M+GAP1-C*->??+DO-GTPase2.2->

■ **GCA_001686755.1; Mycolicibacterium vulneris; counts= 1; multicellularity status: FALSE**

- HRDC-like?+Fibronectin-like_repeats->GAP1-N2+GAP1-M+GAP1-C*->??+DO-GTPase2.2->

■ **GCA_001686675.1; Mycolicibacterium vulneris; counts= 1; multicellularity status: FALSE**

- HRDC-like?+Fibronectin-like_repeats->GAP1-N2+GAP1-M+GAP1-C*->??+DO-GTPase2.2->

■ **GCA_001698815.1; Pseudomonas syringae; counts= 1; multicellularity status: FALSE**

- GAP2->DO-GTPase2.1->DO-GTPase1->GAP1-N1+GAP1-M+GAP1-C+EAD1*->CASPASE+EAD1->

■ **GCF_001700505.1; Streptomyces mutomycini; counts= 1; multicellularity status: TRUE**

- GAP1-N2+GAP1-M+GAP1-C*->

■ **GCA_001701025.1; Lentzea guizhouensis; counts= 1; multicellularity status: TRUE**

- GrpE->HSP70->TPR_containing_alpha_helical+Fibronectin-like_repeats->TPR_containing_alpha_helical+Fibronectin-like_repeats->ZnR+ZnR+DO-GTPase2.2->GAP1-N2+GAP1-M+GAP1-C*->?->TM+TM+TM+TM+ZnR+ZnR+DO-GTPase2.2->HSP70->?->?->?->||<-PSE<-unknown+CASPASE

■ **GCF_001591665.1; Bacillus soli; counts= 1; multicellularity status: FALSE**

- TM+vWA(canonical)+YtKA-like+TM+??->Tubulin+coiled_coil+??->||<-?||Tubulin+coiled_coil+HTH?+??->TM+TM+TM+vWA(canonical)+TM+TM+TM+polar_tail->Fibronectin-like_repeats+??->ZnR+ZnR+DO-GTPase2.2+ZnR+ZnR->GAP1-N2+GAP1-M+GAP1-C+polar_tail+TM+beta-rich*->

■ **GCF_001591805.1; Bacillus novalis; counts= 1; multicellularity status: FALSE**

- TM+vWA(canonical)+YtKA-like+TM+??->Tubulin+coiled_coil+??->Tubulin+coiled_coil+HTH?+??->TM+TM+TM+vWA(canonical)+TM+TM+TM+polar_tail->Fibronectin-like_repeats+??->ZnR+ZnR+DO-GTPase2.2+ZnR+ZnR->GAP1-N2+GAP1-M+GAP1-C+polar_tail+TM+beta-rich*->

■ **GCA_900078705.1; Bordetella ansorpii; counts= 1; multicellularity status: FALSE**

- Calcineurin+GAPEAD1->GAP2->ZnR+DO-GTPase2.1->DO-GTPase1->GAP1-N1+GAP1-M+GAP1-C*->

■ **GCA_001685025.1; Bacillus sp. FJAT-27225; counts= 1; multicellularity status: NA**

- TM+vWA(canonical)+YtKA-like+TM+??->Tubulin+coiled_coil+??->Tubulin+coiled_coil+HTH?+??->TM+TM+TM+vWA(canonical)+TM+TM+TM+polar_tail->Fibronectin-like_repeats+??->ZnR+ZnR+DO-GTPase2.2+ZnR+ZnR->GAP1-N2+GAP1-M+GAP1-C+polar_tail+TM+beta-rich*->

■ **GCF_001556295.1; Blastomonas sp. CCH5-A3; counts= 1; multicellularity status: FALSE**

- Calcineurin+GAPEAD1->GAP2->ZnR+DO-GTPase2.1->DO-GTPase1->GAP1-N1+GAP1-M+GAP1-C*->

■ **GCF_001685485.1; Formosa haliotis; counts= 1; multicellularity status: FALSE**

- GAP2->PSE->PSE->GAP1-N1+GAP1-M+GAP1-C*->EAD1->?->??+EAD1->EAD1+Trypsin->

■ **GCF_001571225.1; Hydrogenophaga palleronii; counts= 1; multicellularity status: FALSE**

- GAP2->DO-GTPase2.1->DO-GTPase1->GAP1-N1+GAP1-M+GAP1-C+EAD1*->CASPASE+EAD1->

■ **GCA_001529295.1; Flavobacterium sp. TAB 87; counts= 1; multicellularity status: FALSE**

- PNP_UDP_1+GAPEAD1->GAP2->ZnR+DO-GTPase2.1->DO-GTPase1->GAP1-N1+GAP1-M+GAP1-C*->

■ **GCF_001510715.1; Bacillus sp. FJAT-29814; counts= 1; multicellularity status: NA**

- TM+vWA(canonical)+YtKA-like+TM+??->Tubulin+coiled_coil+??->Tubulin+coiled_coil+HTH?+??->TM+TM+TM+vWA(canonical)+TM+TM+TM+polar_tail->Fibronectin-like_repeats+??->ZnR+ZnR+DO-GTPase2.2+ZnR+ZnR->GAP1-N2+GAP1-M+GAP1-C+polar_tail+TM+beta-rich*->

■ **GCA_001682855.1; Bacillus sp. FJAT-27264; counts= 1; multicellularity status: NA**

- TM+vWA(canonical)+YtKA-like+TM+??->Tubulin+coiled_coil+??->Tubulin+coiled_coil+HTH?+??->TM+TM+TM+vWA(canonical)+TM+TM+TM+polar_tail->Fibronectin-like_repeats+??->ZnR+ZnR+DO-GTPase2.2+ZnR+ZnR->GAP1-N2+GAP1-M+GAP1-C+polar_tail+TM+beta-rich*->

■ **GCF_001661965.1; Croceicoccus mobilis; counts= 1; multicellularity status: FALSE**

- Calcineurin+GAPEAD1->GAP2->ZnR+DO-GTPase2.1->DO-GTPase1->GAP1-N1+GAP1-M+GAP1-C*->

■ **GCA__001665575.1; Mycobacterium sp. 852013-51886__SCH5428379; counts= 1; multicellularity status: FALSE**

- HRDC-like?+Fibronectin-like__repeats->GAP1-N2+GAP1-M+GAP1-C*->??+DO-GTPase2.2->

■ **GCA__001672995.1; Mycobacterium sp. 1482292.6; counts= 1; multicellularity status: FALSE**

- GAP__unknown1+TM+TM+GAP__unknown2->HRDC-like?+Fibronectin-like__repeats->GAP1-N2+GAP1-M+GAP1-C*->beta-rich+vWA(canonical)->??+DO-GTPase2.2->TM+DUF4190+TM+PknH__C->TM+DUF4190+TM+PknH__C->TM+DUF4190+TM+PknH__C->TM+DUF4190+TM+PknH__C->TM+DUF4190+TM+PknH__C->

■ **GCA__001673055.1; Mycobacterium sp. 1245801.1; counts= 1; multicellularity status: FALSE**

- GAP__unknown1+TM+TM+GAP__unknown2->HRDC-like?+Fibronectin-like__repeats->GAP1-N2+GAP1-M+GAP1-C*->beta-rich+vWA(canonical)->??+DO-GTPase2.2->TM+DUF4190+TM+PknH__C->TM+DUF4190+TM->

■ **GCF__001700515.1; Streptomyces lushanensis; counts= 1; multicellularity status: TRUE**

- GAP1-N2+GAP1-M+GAP1-C*->

■ **GCA__001513965.1; Streptomyces cellostaticus; counts= 1; multicellularity status: TRUE**

- TPR__containing__alpha__helical+Fibronectin-like__repeats->ZnR+ZnR+DO-GTPase2.2->GAP1-N2+GAP1-M+GAP1-C*->TM+TM+TM+TM+ZnR+ZnR+DO-GTPase2.2+TM->vWA(canonical)->PP2C->Pkinase+ZnR+beta-rich->

■ **GCA__001673415.1; Mycobacterium; counts= 1; multicellularity status: FALSE**

- GAP__unknown1+TM+TM+GAP__unknown2->HRDC-like?+Fibronectin-like__repeats->GAP1-N2+GAP1-M+GAP1-C*->beta-rich+vWA(canonical)->??+DO-GTPase2.2->TM+DUF4190+TM+PknH__C->TM+DUF4190+TM+PknH__C->TM+DUF4190+TM+PknH__C->PSE->?->TM+DUF4190+TM+PknH__C->

■ **GCA__001636945.1; Streptomyces sp. FXJ1.172; counts= 1; multicellularity status: TRUE**

- GrpE->HSP70->||<-PSE||ZnR+ZnR+DO-GTPase2.2->GAP1-N2+GAP1-M+GAP1-C*->TM+TM+TM+TM+ZnR+ZnR+DO-GTPase2.2+Jelly__roll+beta-sandwich->||<-STAND__NTPase+TM+TM+TM+TM+TM+TM+TM

■ **GCA__001665875.1; Mycobacterium sp. 852002-40037__SCH5390672; counts= 1; multicellularity status: FALSE**

- TM+DUF4190+TM->TM+DUF4190+TM->polar__leader+TM+TM+TM->TM+vWA(canonical)+Fibronectin-like__repeats+TM+beta-rich+lc__tail->Tubulin+alpha__helical+??+??->GAP__unknown1+TM+TM+GAP__unknown2->HRDC-like?+Fibronectin-like__repeats->GAP1-N2+GAP1-M+GAP1-C*->beta-rich+vWA(canonical)->??+DO-GTPase2.2->TM+DUF4190+TM+PknH__C->TM+DUF4190+TM+PknH__C->TM+DUF4190+TM+PknH__C->TM+DUF4190+TM+PknH__C->

■ **GCA__001667145.1; Mycobacterium sp. 852002-51971__SCH5477799-a; counts= 1; multicellularity status: FALSE**

- TM+DUF4190+TM->TM+DUF4190+TM->polar__leader+TM+TM+TM->TM+vWA(canonical)+Fibronectin-like__repeats+TM+beta-rich+lc__tail->Tubulin+alpha__helical+??+??->GAP__unknown1+TM+TM+GAP__unknown2->HRDC-like?+Fibronectin-like__repeats->GAP1-N2+GAP1-M+GAP1-C*->beta-rich+vWA(canonical)->??+DO-GTPase2.2->TM+DUF4190+TM+PknH__C->TM+DUF4190+TM+PknH__C->TM+DUF4190+TM+PknH__C->

■ **GCA__001669335.1; Mycobacterium sp. 1245852.3; counts= 1; multicellularity status: FALSE**

- GAP1-N2+GAP1-M+GAP1-C*->beta-rich+vWA(canonical)->??+DO-GTPase2.2->TM+DUF4190+TM+PknH__C->

■ **GCA__001673555.1; Mycobacterium sp. 1165178.9; counts= 1; multicellularity status: FALSE**

- polar__leader+TM+TM+TM->TM+vWA(canonical)+Fibronectin-like__repeats+TM+beta-rich+lc__tail->Tubulin+alpha__helical+??+??->GAP__unknown1+TM+TM+GAP__unknown2->HRDC-like?+Fibronectin-like__repeats->GAP1-N2+GAP1-M+GAP1-C*->beta-rich+vWA(canonical)->??+DO-GTPase2.2->TM+DUF4190+TM+PknH__C->TM+DUF4190+TM+PknH__C->TM+DUF4190+TM+PknH__C->

■ **GCA__004195765.1; Streptomyces sampsonii; counts= 1; multicellularity status: TRUE**

- GAP1-N2+GAP1-M+GAP1-C*->

■ **GCF__001552215.1; Actinomadura macra; counts= 1; multicellularity status: TRUE**

- GrpE->HSP70->TPR__containing__alpha__helical+Fibronectin-like__repeats->ZnR+ZnR+DO-GTPase2.2->GAP1-N2+GAP1-M+GAP1-C*->TM+TM+TM+TM+ZnR+ZnR+DO-GTPase2.2+TM->

■ **GCF__001552635.1; Actinomadura hibisca; counts= 1; multicellularity status: TRUE**

- GrpE->HSP70->TPR__containing__alpha__helical+Fibronectin-like__repeats->ZnR+ZnR+DO-GTPase2.2->GAP1-N2+GAP1-M+GAP1-C*->TM+TM+TM+TM+ZnR+ZnR+DO-GTPase2.2+TM->

■ **GCF_001613405.1; Nocardia crassostreae; counts= 1; multicellularity status: TRUE**

- Pkinase+TM->ZnR+DO-GTPase2.1->GAP1-N2+GAP1-M+GAP1-C*->

■ **GCF_001625085.1; Nocardia acidivorans; counts= 1; multicellularity status: TRUE**

- Pkinase+TM->ZnR+DO-GTPase2.1->GAP1-N2+GAP1-M+GAP1-C*->

■ **GCF_001570765.1; Nocardiopsis listeri; counts= 1; multicellularity status: TRUE**

- TPR_containing_alpha_helical+Fibronectin-like_repeats->ZnR+ZnR+DO-GTPase2.2->GAP1-N2+GAP1-M+GAP1-C*->TM+TM+TM+TM+ZnR+ZnR+DO-GTPase2.2->

■ **GCF_001552195.1; Actinomadura latina; counts= 1; multicellularity status: TRUE**

- GrpE->HSP70->TPR_containing_alpha_helical+Fibronectin-like_repeats->ZnR+ZnR+DO-GTPase2.2->GAP1-N2+GAP1-M+GAP1-C*->TM+TM+TM+TM+ZnR+ZnR+DO-GTPase2.2+TM->

■ **GCF_001612885.1; Nocardia harenae; counts= 1; multicellularity status: TRUE**

- GrpE->HSP70->TPR_containing_alpha_helical+Fibronectin-like_repeats->ZnR+ZnR+DO-GTPase2.2->GAP1-N2+GAP1-M+GAP1-C*->TM+TM+TM+TM+ZnR+ZnR+DO-GTPase2.2->?->HSP70->vWA(canonical)->

■ **GCF_001613145.1; Nocardia jejuensis; counts= 1; multicellularity status: TRUE**

- Pkinase+TM->ZnR+DO-GTPase2.1->GAP1-N2+GAP1-M+GAP1-C*->

■ **GCA_001683795.1; Akkermansia glycaniphila; counts= 1; multicellularity status: FALSE**

- FHA-like?+ZnR+ZnR+DO-GTPase2.2->GAP1-N2+GAP1-M+GAP1-C*->

■ **GCF_001552155.1; Actinomadura formosensis; counts= 1; multicellularity status: TRUE**

- GrpE->HSP70->TPR_containing_alpha_helical+Fibronectin-like_repeats->ZnR+ZnR+DO-GTPase2.2->GAP1-N2+GAP1-M+GAP1-C*->TM+TM+TM+TM+ZnR+ZnR+DO-GTPase2.2+TM->

■ **GCA_001667505.1; Mycobacterium sp. ACS1612; counts= 1; multicellularity status: FALSE**

- TM+DUF4190+TM->TM+DUF4190+TM->polar_leader+TM+TM+TM->PSE->Tubulin+alpha_helical+??+??->GAP_unknown1+TM+TM+GAP_unknown2->HRDC-like?+Fibronectin-like_repeats->GAP1-N2+GAP1-M+GAP1-C*->beta-rich+vWA(canonical)->??+DO-GTPase2.2->?->TM+DUF4190+TM+PknH_C->

■ **GCF_001552175.1; Actinomadura kijaniata; counts= 1; multicellularity status: TRUE**

- GrpE->HSP70->TPR_containing_alpha_helical+Fibronectin-like_repeats->ZnR+ZnR+DO-GTPase2.2->GAP1-N2+GAP1-M+GAP1-C*->TM+TM+TM+TM+ZnR+ZnR+DO-GTPase2.2+TM->

■ **GCA_001667835.1; Mycobacterium sp. E3078; counts= 1; multicellularity status: FALSE**

- TM+DUF4190+TM->TM+DUF4190+TM->polar_leader+TM+TM+TM->TM+vWA(canonical)+Fibronectin-like_repeats+TM+beta-rich+lc_tail->Tubulin+alpha_helical+??+??->GAP_unknown1+TM+TM+GAP_unknown2->HRDC-like?+Fibronectin-like_repeats->GAP1-N2+GAP1-M+GAP1-C*->beta-rich+vWA(canonical)->??+DO-GTPase2.2->TM+DUF4190+TM+PknH_C->TM+DUF4190+TM+PknH_C->TM+DUF4190+TM+PknH_C->TM+DUF4190+TM+PknH_C->

■ **GCA_001545135.1; Mycolicibacterium wolinskyi; counts= 1; multicellularity status: FALSE**

- HRDC-like?+Fibronectin-like_repeats->GAP1-N2+GAP1-M+GAP1-C*->??+DO-GTPase2.2->

■ **GCA_001667865.1; Mycobacterium; counts= 1; multicellularity status: FALSE**

- TM+DUF4190+TM->TM+DUF4190+TM->polar_leader+TM+TM+TM->TM+vWA(canonical)+Fibronectin-like_repeats+TM+beta-rich+lc_tail->Tubulin+alpha_helical+??+??->GAP_unknown1+TM+TM+GAP_unknown2->HRDC-like?+Fibronectin-like_repeats->GAP1-N2+GAP1-M+GAP1-C*->beta-rich+vWA(canonical)->??+DO-GTPase2.2->TM+DUF4190+TM+PknH_C->TM+DUF4190+TM+PknH_C->TM+DUF4190+TM+PknH_C->TM+DUF4190+TM+PknH_C->

■ **GCA_001666755.1; Mycobacterium; counts= 1; multicellularity status: FALSE**

- Tubulin+alpha_helical+??+??->GAP_unknown1+TM+TM+GAP_unknown2->HRDC-like?+Fibronectin-like_repeats->GAP1-N2+GAP1-M+GAP1-C*->beta-rich+vWA(canonical)->

■ **GCF_001642915.1; Roseimaritima ulvae; counts= 1; multicellularity status: TRUE**

- FGS-fold_domain->ZnR+DO-GTPase2.1->GAP1-N2+GAP1-M*->

■ **GCA_001667265.1; Mycobacterium sp. ACS4331; counts= 1; multicellularity status: FALSE**

- Tubulin+alpha_helical+??+??->GAP_unknown1+TM+TM+GAP_unknown2->HRDC-like?+Fibronectin-like_repeats->GAP1-N2+GAP1-M+GAP1-C*->beta-rich+vWA(canonical)->??+DO-GTPase2.2->TM+DUF4190+TM+PknH_C->

■ **GCA_001667995.1; Mycobacterium sp. E2660; counts= 1; multicellularity status: FALSE**

- TM+DUF4190+TM->TM+DUF4190+TM->polar_leader+TM+TM+TM->TM+vWA(canonical)+Fibronectin-like_repeats+TM+beta-rich+lc_tail->Tubulin+alpha_helical+??+??->GAP_unknown1+TM+TM+GAP_unknown2->HRDC-like?+Fibronectin-like_repeats->GAP1-N2+GAP1-M+GAP1-C*->beta-rich+vWA(canonical)->??+DO-GTPase2.2->TM+DUF4190+TM+PknH_C->TM+DUF4190+TM+PknH_C->TM+DUF4190+TM+PknH_C->TM+DUF4190+TM+PknH_C->

■ **GCA_001672685.1; Mycobacterium sp. E2462; counts= 1; multicellularity status: FALSE**

- Tubulin+alpha_helical+??+??->GAP_unknown1+TM+TM+GAP_unknown2->HRDC-like?+Fibronectin-like_repeats->GAP1-N2+GAP1-M+GAP1-C*->beta-rich+vWA(canonical)->??+DO-GTPase2.2->TM+DUF4190+TM+PknH_C->TM+DUF4190+TM+PknH_C->TM+DUF4190+TM+PknH_C->TM+DUF4190+TM+PknH_C->

■ **GCA_001610895.1; Planctomyces sp. SH-PL62; counts= 1; multicellularity status: NA**

- GAP1-N2+GAP1-M*->

■ **GCF_001705305.1; Paenibacillus kribbensis; counts= 1; multicellularity status: TRUE**

- TM+vWA(canonical)+YtKA-like+TM+??->Tubulin+coiled_coil+??->Tubulin+coiled_coil+HTH?+??->TM+TM+TM+vWA(canonical)+TM+TM+TM+polar_tail->Fibronectin-like_repeats+??->ZnR+ZnR+DO-GTPase2.2+ZnR+ZnR->GAP1-N2+GAP1-M+GAP1-C+polar_tail+TM+beta-rich*->

■ **GCA_001637205.1; Paenibacillus glacialis; counts= 1; multicellularity status: TRUE**

- Tubulin+coiled_coil+HTH?+??->TM+TM+TM+vWA+TM+TM+TM+polar_tail->Fibronectin-like_repeats+??->?->ZnR+ZnR+DO-GTPase2.2->GAP1-N2+GAP1-M+GAP1-C+polar_tail*->

■ **GCA_001637165.1; Paenibacillus macquariensis; counts= 1; multicellularity status: TRUE**

- TM+vWA(canonical)+YtKA-like+TM+??->Tubulin+coiled_coil+??->Tubulin+coiled_coil+HTH?+??->TM+TM+TM+vWA(canonical)+TM+TM+TM+polar_tail->Fibronectin-like_repeats+??->ZnR+ZnR+DO-GTPase2.2+ZnR+ZnR->GAP1-N2+GAP1-M+GAP1-C+polar_tail+TM+beta-rich*->

■ **GCA_001637215.1; Paenibacillus macquariensis; counts= 1; multicellularity status: TRUE**

- TM+vWA(canonical)+YtKA-like+TM+??->Tubulin+coiled_coil+??->Tubulin+coiled_coil+HTH?+??->TM+TM+TM+vWA(canonical)+TM+TM+TM+polar_tail->Fibronectin-like_repeats+??->ZnR+ZnR+DO-GTPase2.2+ZnR+ZnR->GAP1-N2+GAP1-M+GAP1-C+polar_tail+TM+beta-rich*->

■ **GCF_900086655.1; Paenibacillus tuaregi; counts= 1; multicellularity status: TRUE**

- Fibronectin-like_repeats+??->ZnR+ZnR+DO-GTPase2.2->GAP1-N2+GAP1-M+GAP1-C+polar_tail*->

■ **GCA_001637225.1; Paenibacillus antarcticus; counts= 1; multicellularity status: TRUE**

- TM+vWA(canonical)+YtKA-like+TM+??->Tubulin+coiled_coil+??->Tubulin+coiled_coil+HTH?+??->TM+TM+TM+vWA(canonical)+TM+TM+TM+polar_tail->Fibronectin-like_repeats+??->ZnR+ZnR+DO-GTPase2.2+ZnR+ZnR->GAP1-N2+GAP1-M+GAP1-C+polar_tail+TM+beta-rich*->

■ **GCA_001857945.1; Paenibacillus crassostreae; counts= 1; multicellularity status: TRUE**

- TM+vWA(canonical)+YtKA-like+TM+??->Tubulin+coiled_coil+??->Tubulin+coiled_coil+HTH?+??->TM+TM+TM+vWA(canonical)+TM+TM+TM+polar_tail->Fibronectin-like_repeats+??->ZnR+ZnR+DO-GTPase2.2+ZnR+ZnR->GAP1-N2+GAP1-M+GAP1-C+polar_tail+TM+beta-rich*->

■ **GCA_001675045.1; Paenibacillus oryzae; counts= 1; multicellularity status: TRUE**

- TM+vWA(canonical)+YtKA-like+TM+??->Tubulin+coiled_coil+??->Tubulin+coiled_coil+HTH?+??->TM+TM+TM+vWA(canonical)+TM+TM+TM+polar_tail->Fibronectin-like_repeats+??->ZnR+ZnR+DO-GTPase2.2+ZnR+ZnR->GAP1-N2+GAP1-M+GAP1-C+polar_tail+TM+beta-rich*->

■ **GCA_001517085.1; Paenibacillus sp. DMB5; counts= 1; multicellularity status: TRUE**

- TM+TM+TM+vWA(canonical)+TM+TM+TM+polar_tail->Fibronectin-like_repeats+??->ZnR+ZnR+DO-GTPase2.2+ZnR+ZnR->GAP1-N2+GAP1-M+GAP1-C+polar_tail+TM+beta-rich*->

■ **GCF_900069005.1; Paenibacillus sp. GM2; counts= 1; multicellularity status: TRUE**

- TM+vWA(canonical)+YtKA-like+TM+??->Tubulin+coiled_coil+??->Tubulin+coiled_coil+HTH?+??->TM+TM+TM+vWA(canonical)+TM+TM+TM+polar_tail->Fibronectin-like_repeats+??->ZnR+ZnR+DO-GTPase2.2+ZnR+ZnR->GAP1-N2+GAP1-M+GAP1-C+polar_tail+TM+beta-rich*->

■ **GCF_001696705.1; Brevibacillus laterosporus; counts= 1; multicellularity status: FALSE**

- TM+vWA(canonical)+YtKA-like+TM+??->Tubulin+coiled_coil+??->Tubulin+coiled_coil+HTH?+??->TM+TM+TM+vWA(canonical)+TM+TM+TM+polar_tail->Fibronectin-like_repeats+??->ZnR+ZnR+DO-GTPase2.2+ZnR+ZnR->GAP1-N2+GAP1-M+GAP1-C+polar_tail+TM+beta-rich*->

- **GCA_001707685.1; Paenibacillus; counts= 1; multicellularity status: TRUE**
  - TM+vWA(canonical)+YtKA-like+TM+??->Tubulin+coiled_coil+??->Tubulin+coiled_coil+HTH?+??->TM+TM+TM+vWA(canonical)+TM+TM+TM+polar_tail->Fibronectin-like_repeats+??->ZnR+ZnR+DO-GTPase2.2+ZnR+ZnR->GAP1-N2+GAP1-M+GAP1-C+polar_tail+TM+beta-rich*->
- **GCA_001709135.1; Paenibacillus polymyxa; counts= 1; multicellularity status: TRUE**
  - TM+vWA(canonical)+YtKA-like+TM+??->Tubulin+coiled_coil+??->Tubulin+coiled_coil+HTH?+??->TM+TM+TM+vWA(canonical)+TM+TM+TM+polar_tail->Fibronectin-like_repeats+??->ZnR+ZnR+DO-GTPase2.2+ZnR+ZnR->GAP1-N2+GAP1-M+GAP1-C+polar_tail+TM+beta-rich*->
- **GCA_001708695.1; Mesorhizobium sp. SEMIA 3007; counts= 1; multicellularity status: TRUE**
  - GAP2->ZnR+DO-GTPase2.1->DO-GTPase1->GAP1-N1+GAP1-M+GAP1-C*->
- **GCF_001715295.1; Streptomyces griseus; counts= 1; multicellularity status: TRUE**
  - GAP1-N2+GAP1-M+GAP1-C*->
- **GCA_001719045.1; Paenibacillus polymyxa; counts= 1; multicellularity status: TRUE**
  - Tubulin+coiled_coil+??->Tubulin+coiled_coil+HTH?+??->TM+TM+TM+vWA+TM+TM+TM+polar_tail->Fibronectin-like_repeats+??->ZnR+ZnR+DO-GTPase2.2->GAP1-N2+GAP1-M+GAP1-C+polar_tail*->
- **GCA_001721045.1; Paenibacillus sp. TI45-13ar; counts= 1; multicellularity status: TRUE**
  - TM+vWA(canonical)+YtKA-like+TM+??->Tubulin+coiled_coil+??->Tubulin+coiled_coil+HTH?+??->TM+TM+TM+vWA(canonical)+TM+TM+TM+polar_tail->Fibronectin-like_repeats+??->ZnR+ZnR+DO-GTPase2.2+ZnR+ZnR->GAP1-N2+GAP1-M+GAP1-C+polar_tail+TM+beta-rich*->
- **GCA_001722515.1; Dietzia alimentaria; counts= 1; multicellularity status: FALSE**
  - GrpE->HSP70->TPR_containing_alpha_helical+Fibronectin-like_repeats->ZnR+ZnR+DO-GTPase2.2->GAP1-N2+GAP1-M+GAP1-C*->TM+TM+TM+TM+ZnR+ZnR+DO-GTPase2.2->?->HSP70_domain_1->HSP70->vWA(canonical)->
- **GCA_001727475.1; Vibrio parahaemolyticus; counts= 1; multicellularity status: FALSE**
  - PNP_UDP_1+EAD1->GAP2->ZnR+DO-GTPase2.1->DO-GTPase1->GAP1-N1+GAP1-M+GAP1-C+EAD1*->SLATT->
- **GCA_001729525.1; Brevibacterium linens; counts= 1; multicellularity status: FALSE**
  - TM+vWA(canonical)+Fibronectin-like_repeats+TM+beta-rich+lc_tail->Tubulin+alpha_helical+??+??->GAP_unknown1+TM+TM+GAP_unknown2->HRDC-like?+Fibronectin-like_repeats->GAP1-N2+GAP1-M+GAP1-C*->beta-rich+vWA(canonical)->??+DO-GTPase2.2->
- **GCF_001723055.1; Streptomyces niveus; counts= 1; multicellularity status: TRUE**
  - GAP1-N2+GAP1-M+GAP1-C*->vWA(canonical)->PP2C->Pkinase+ZnR+beta-rich->||<-TPR_containing_alpha_helical+Fibronectin-like_repeats
- **GCF_001746285.1; Streptomyces sp. EN16; counts= 1; multicellularity status: TRUE**
  - GAP1-N2+GAP1-M+GAP1-C*->
- **GCF_001748185.1; Brevibacillus brevis; counts= 1; multicellularity status: FALSE**
  - TM+vWA(canonical)+YtKA-like+TM+??->Tubulin+coiled_coil+??->Tubulin+coiled_coil+HTH?+??->TM+TM+TM+vWA(canonical)+TM+TM+TM+polar_tail->Fibronectin-like_repeats+??->ZnR+ZnR+DO-GTPase2.2+ZnR+ZnR->GAP1-N2+GAP1-M+GAP1-C+polar_tail+TM+beta-rich*->
- **GCF_001748305.1; Streptomyces luteocolor; counts= 1; multicellularity status: TRUE**
  - GAP1-N2+GAP1-M+GAP1-C*->vWA(canonical)->PP2C->Pkinase+ZnR+beta-rich->||<-TPR_containing_alpha_helical+Fibronectin-like_repeats
- **GCA_001751375.1; Streptomyces; counts= 1; multicellularity status: TRUE**
  - PP2C->Pkinase->?->?->?->HSP70_domain_2+low_complexity+domain_betaX->ZnR+ZnR+DO-GTPase2.2->GAP1-N2+GAP1-M+GAP1-C*->TM+TM+TM+TM+ZnR+ZnR+DO-GTPase2.2->
- **GCA_900079405.3; Streptomyces sp. F-1; counts= 1; multicellularity status: TRUE**

- PP2C->Pkinase->?->?->?->HSP70_domain_2+low_complexity+domain_betaX->ZnR+ZnR+DO-GTPase2.2->GAP1-N2+GAP1-M+GAP1-C*->TM+TM+TM+TM+ZnR+ZnR+DO-GTPase2.2->

■ **GCA_001756795.1; Mycolicibacterium sp. (ex Dasyatis americana); counts= 1; multicellularity status: FALSE**

- HRDC-like?+Fibronectin-like_repeats->GAP1-N2+GAP1-M+GAP1-C*->??+DO-GTPase2.2->

■ **GCA_001758225.1; Humibacillus sp. DSM 29435; counts= 1; multicellularity status: FALSE**

- TM+vWA(canonical)+Fibronectin-like_repeats+TM+beta-rich+lc_tail->Tubulin+alpha_helical+??+??->GAP_unknown1+TM+TM+GAP_unknown2->?->HRDC-like?+Fibronectin-like_repeats->GAP1-N2+GAP1-M+GAP1-C*->?->??+DO-GTPase2.2->

■ **GCA_001751305.1; Streptomyces nanshensis; counts= 1; multicellularity status: TRUE**

- GAP1-N2+GAP1-M+GAP1-C*->

■ **GCA_001807295.1; Corynebacterium sp. HMSC06C06; counts= 1; multicellularity status: FALSE**

- polar_leader+TM+TM+TM->TM+vWA(canonical)+Fibronectin-like_repeats+TM+beta-rich+lc_tail->Tubulin+alpha_helical+??+??->GAP_unknown1+TM+TM+GAP_unknown2->HRDC-like?+Fibronectin-like_repeats->GAP1-N2+GAP1-M+GAP1-C*->beta-rich+vWA(canonical)->??+DO-GTPase2.2->polar_leader+TM+TM+TM->

■ **GCA_001807315.1; Corynebacterium sp. HMSC06D04; counts= 1; multicellularity status: FALSE**

- polar_leader+TM+TM+TM->TM+vWA(canonical)+Fibronectin-like_repeats+TM+beta-rich+lc_tail->Tubulin+alpha_helical+??+??->GAP_unknown1+TM+TM+GAP_unknown2->HRDC-like?+Fibronectin-like_repeats->GAP1-N2+GAP1-M+GAP1-C*->beta-rich+vWA(canonical)->??+DO-GTPase2.2->polar_leader+TM+TM+TM->

■ **GCA_001807205.1; Corynebacterium sp. HMSC05D03; counts= 1; multicellularity status: FALSE**

- polar_leader+TM+TM+TM->TM+vWA(canonical)+Fibronectin-like_repeats+TM+beta-rich+lc_tail->Tubulin+alpha_helical+??+??->GAP_unknown1+TM+TM+GAP_unknown2->HRDC-like?+Fibronectin-like_repeats->GAP1-N2+GAP1-M+GAP1-C*->beta-rich+vWA(canonical)->??+DO-GTPase2.2->polar_leader+TM+TM+TM->

■ **GCA_001810285.1; Corynebacterium; counts= 1; multicellularity status: FALSE**

- polar_leader+TM+TM+TM->TM+vWA(canonical)+Fibronectin-like_repeats+TM+beta-rich+lc_tail->Tubulin+alpha_helical+??+??->PSE->HRDC-like?+Fibronectin-like_repeats->GAP1-N2+GAP1-M+GAP1-C*->beta-rich+vWA(canonical)->??+DO-GTPase2.2->polar_leader+TM+TM+TM->

■ **GCA_001806945.1; Corynebacterium sp. HMSC30G07; counts= 1; multicellularity status: FALSE**

- polar_leader+TM+TM+TM->TM+vWA(canonical)+Fibronectin-like_repeats+TM+beta-rich+lc_tail->Tubulin+alpha_helical+??+??->GAP_unknown1+TM+TM+GAP_unknown2->HRDC-like?+Fibronectin-like_repeats->GAP1-N2+GAP1-M+GAP1-C*->?->??+DO-GTPase2.2->?->||<-?<-?<-HSP70

■ **GCA_001807365.1; Corynebacterium sp. HMSC06G04; counts= 1; multicellularity status: FALSE**

- polar_leader+TM+TM+TM->TM+vWA(canonical)+Fibronectin-like_repeats+TM+beta-rich+lc_tail->Tubulin+alpha_helical+??+??->GAP_unknown1+TM+TM+GAP_unknown2->HRDC-like?+Fibronectin-like_repeats->GAP1-N2+GAP1-M+GAP1-C*->beta-rich+vWA(canonical)->??+DO-GTPase2.2->polar_leader+TM+TM+TM->

■ **GCA_001807215.1; Corynebacterium sp. HMSC05D08; counts= 1; multicellularity status: FALSE**

- polar_leader+TM+TM+TM->TM+vWA(canonical)+Fibronectin-like_repeats+TM+beta-rich+lc_tail->Tubulin+alpha_helical+??+??->GAP_unknown1+TM+TM+GAP_unknown2->HRDC-like?+Fibronectin-like_repeats->GAP1-N2+GAP1-M+GAP1-C*->beta-rich+vWA(canonical)->??+DO-GTPase2.2->polar_leader+TM+TM+TM->

■ **GCA_001811545.1; Corynebacteriaceae; counts= 1; multicellularity status: FALSE**

- polar_leader+TM+TM+TM->TM+vWA(canonical)+Fibronectin-like_repeats+TM+beta-rich+lc_tail->Tubulin+alpha_helical+??+??->GAP_unknown1+TM+TM+GAP_unknown2->HRDC-like?+Fibronectin-like_repeats->GAP1-N2+GAP1-M+GAP1-C*->beta-rich+vWA(canonical)->??+DO-GTPase2.2->polar_leader+TM+TM+TM->

■ **GCA_001811515.1; Turicella sp. HMSC076G08; counts= 1; multicellularity status: FALSE**

- polar_leader+TM+TM+TM->TM+vWA(canonical)+Fibronectin-like_repeats+TM+beta-rich+lc_tail->Tubulin+alpha_helical+??+??->GAP_unknown1+TM+TM+GAP_unknown2->HRDC-like?+Fibronectin-like_repeats->GAP1-N2+GAP1-M+GAP1-C*->beta-rich+vWA(canonical)->??+DO-GTPase2.2->polar_leader+TM+TM+TM->

■ **GCF_001807025.1; Corynebacterium sp. HMSC11E11; counts= 1; multicellularity status: FALSE**

- polar_leader+TM+TM+TM->TM+vWA(canonical)+Fibronectin-like_repeats+TM+beta-rich+lc_tail->Tubulin+alpha_helical+??+??->GAP_unknown1+TM+TM+GAP_unknown2->HRDC-like?+Fibronectin-like_repeats->GAP1-N2+GAP1-M+GAP1-C*->beta-rich+vWA(canonical)->??+DO-GTPase2.2->polar_leader+TM+TM+TM->

■ **GCA_001811845.1; Corynebacteriaceae; counts= 1; multicellularity status: FALSE**

- polar_leader+TM+TM+TM->TM+vWA(canonical)+Fibronectin-like_repeats+TM+beta-rich+lc_tail->Tubulin+alpha_helical+??+??->GAP_unknown1+TM+TM+GAP_unknown2->HRDC-like?+Fibronectin-like_repeats->GAP1-N2+GAP1-M+GAP1-C*->beta-rich+vWA(canonical)->??+DO-GTPase2.2->polar_leader+TM+TM+TM->

■ **GCA_001808885.1; Corynebacterium sp. HMSC074E01; counts= 1; multicellularity status: FALSE**

- polar_leader+TM+TM+TM->TM+vWA(canonical)+Fibronectin-like_repeats+TM+beta-rich+lc_tail->Tubulin+alpha_helical+??+??->GAP_unknown1+TM+TM+GAP_unknown2->HRDC-like?+Fibronectin-like_repeats->GAP1-N2+GAP1-M+GAP1-C*->beta-rich+vWA(canonical)->??+DO-GTPase2.2->polar_leader+TM+TM+TM->

- **GCA_001811255.1; Corynebacteriaceae; counts= 1; multicellularity status: FALSE**
  - polar_leader+TM+TM+TM->TM+vWA(canonical)+Fibronectin-like_repeats+TM+beta-rich+lc_tail->Tubulin+alpha_helical+??+??->GAP_unknown1+TM+TM+GAP_unknown2->HRDC-like?+Fibronectin-like_repeats->GAP1-N2+GAP1-M+GAP1-C*->beta-rich+vWA(canonical)->??+DO-GTPase2.2->polar_leader+TM+TM+TM->
- **GCA_001808895.1; Corynebacterium sp. HMSC070E08; counts= 1; multicellularity status: FALSE**
  - polar_leader+TM+TM+TM->TM+vWA(canonical)+Fibronectin-like_repeats+TM+beta-rich+lc_tail->Tubulin+alpha_helical+??+??->GAP_unknown1+TM+TM+GAP_unknown2->HRDC-like?+Fibronectin-like_repeats->GAP1-N2+GAP1-M+GAP1-C*->beta-rich+vWA(canonical)->??+DO-GTPase2.2->polar_leader+TM+TM+TM->
- **GCA_001810085.1; Corynebacterium sp. HMSC072A02; counts= 1; multicellularity status: FALSE**
  - polar_leader+TM+TM+TM->TM+vWA(canonical)+Fibronectin-like_repeats+TM+beta-rich+lc_tail->Tubulin+alpha_helical+??+??->GAP_unknown1+TM+TM+GAP_unknown2->HRDC-like?+Fibronectin-like_repeats->GAP1-N2+GAP1-M+GAP1-C*->beta-rich+vWA(canonical)->??+DO-GTPase2.2->polar_leader+TM+TM+TM->
- **GCA_001811565.1; Corynebacterium; counts= 1; multicellularity status: FALSE**
  - polar_leader+TM+TM+TM->TM+vWA(canonical)+Fibronectin-like_repeats+TM+beta-rich+lc_tail->Tubulin+alpha_helical+??+??->GAP_unknown1+TM+TM+GAP_unknown2->HRDC-like?+Fibronectin-like_repeats->GAP1-N2+GAP1-M+GAP1-C*->beta-rich+vWA(canonical)->??+DO-GTPase2.2->polar_leader+TM+TM+TM->
- **GCA_001812965.1; Actinomyces; counts= 1; multicellularity status: TRUE**
  - polar_leader+TM+TM+TM->TM+vWA(canonical)+Fibronectin-like_repeats+TM+beta-rich+lc_tail->Tubulin+alpha_helical+??+??->GAP_unknown1+TM+TM+GAP_unknown2->HRDC-like?+Fibronectin-like_repeats->GAP1-N2+GAP1-M+GAP1-C*->?->??+DO-GTPase2.2->
- **GCA_001812805.1; Corynebacterium sp. HMSC078H07; counts= 1; multicellularity status: FALSE**
  - polar_leader+TM+TM+TM->TM+vWA(canonical)+Fibronectin-like_repeats+TM+beta-rich+lc_tail->Tubulin+alpha_helical+??+??->GAP_unknown1+TM+TM+GAP_unknown2->HRDC-like?+Fibronectin-like_repeats->GAP1-N2+GAP1-M+GAP1-C*->beta-rich+vWA(canonical)->??+DO-GTPase2.2->polar_leader+TM+TM+TM->
- **GCA_001815175.1; Turicella sp. HMSC034H07; counts= 1; multicellularity status: FALSE**
  - polar_leader+TM+TM+TM->TM+vWA(canonical)+Fibronectin-like_repeats+TM+beta-rich+lc_tail->Tubulin+alpha_helical+??+??->GAP_unknown1+TM+TM+GAP_unknown2->HRDC-like?+Fibronectin-like_repeats->GAP1-N2+GAP1-M+GAP1-C*->beta-rich+vWA(canonical)->??+DO-GTPase2.2->polar_leader+TM+TM+TM->
- **GCA_001813425.1; Corynebacterium sp. HMSC056E09; counts= 1; multicellularity status: FALSE**
  - polar_leader+TM+TM+TM->TM+vWA(canonical)+Fibronectin-like_repeats+TM+beta-rich+lc_tail->Tubulin+alpha_helical+??+??->GAP_unknown1+TM+TM+GAP_unknown2->HRDC-like?+Fibronectin-like_repeats->GAP1-N2+GAP1-M+GAP1-C*->beta-rich+vWA(canonical)->??+DO-GTPase2.2->polar_leader+TM+TM+TM->
- **GCA_001814395.1; Turicella sp. HMSC059E07; counts= 1; multicellularity status: FALSE**
  - polar_leader+TM+TM+TM->TM+vWA(canonical)+Fibronectin-like_repeats+TM+beta-rich+lc_tail->Tubulin+alpha_helical+??+??->GAP_unknown1+TM+TM+GAP_unknown2->HRDC-like?+Fibronectin-like_repeats->GAP1-N2+GAP1-M+GAP1-C*->beta-rich+vWA(canonical)->??+DO-GTPase2.2->polar_leader+TM+TM+TM->
- **GCA_001814885.1; Corynebacterium sp. HMSC068G04; counts= 1; multicellularity status: FALSE**
  - polar_leader+TM+TM+TM->TM+vWA(canonical)+Fibronectin-like_repeats+TM+beta-rich+lc_tail->Tubulin+alpha_helical+??+??->GAP_unknown1+TM+TM+GAP_unknown2->HRDC-like?+Fibronectin-like_repeats->GAP1-N2+GAP1-M+GAP1-C*->beta-rich+vWA(canonical)->??+DO-GTPase2.2->polar_leader+TM+TM+TM->
- **GCA_001814945.1; Corynebacterium sp. HMSC066C02; counts= 1; multicellularity status: FALSE**
  - polar_leader+TM+TM+TM->TM+vWA(canonical)+Fibronectin-like_repeats+TM+beta-rich+lc_tail->Tubulin+alpha_helical+??+??->GAP_unknown1+TM+TM+GAP_unknown2->HRDC-like?+Fibronectin-like_repeats->GAP1-N2+GAP1-M+GAP1-C*->beta-rich+vWA(canonical)->??+DO-GTPase2.2->polar_leader+TM+TM+TM->
- **GCA_001813815.1; Corynebacterium sp. HMSC076D02; counts= 1; multicellularity status: FALSE**
  - polar_leader+TM+TM+TM->PSE->PSE->Tubulin+alpha_helical+??+??->GAP_unknown1+TM+TM+GAP_unknown2->HRDC-like?+Fibronectin-like_repeats->GAP1-N2+GAP1-M+GAP1-C*->beta-rich+vWA(canonical)->??+DO-GTPase2.2->polar_leader+TM+TM+TM->
- **GCA_001836165.1; Corynebacterium sp. HMSC036D02; counts= 1; multicellularity status: FALSE**
  - polar_leader+TM+TM+TM->TM+vWA(canonical)+Fibronectin-like_repeats+TM+beta-rich+lc_tail->Tubulin+alpha_helical+??+??->GAP_unknown1+TM+TM+GAP_unknown2->HRDC-like?+Fibronectin-like_repeats->GAP1-N2+GAP1-M+GAP1-C*->beta-rich+vWA(canonical)->??+DO-GTPase2.2->polar_leader+TM+TM+TM->
- **GCA_001836185.1; Corynebacterium sp. HMSC036D03; counts= 1; multicellularity status: FALSE**
  - polar_leader+TM+TM+TM->TM+vWA(canonical)+Fibronectin-like_repeats+TM+beta-rich+lc_tail->Tubulin+alpha_helical+??+??->GAP_unknown1+TM+TM+GAP_unknown2->HRDC-like?+Fibronectin-like_repeats->GAP1-N2+GAP1-M+GAP1-C*->beta-rich+vWA(canonical)->??+DO-GTPase2.2->polar_leader+TM+TM+TM->
- **GCA_001853525.1; Mycobacterium sp. 24999; counts= 1; multicellularity status: FALSE**

- HRDC-like?+Fibronectin-like__repeats->GAP1-N2+GAP1-M+GAP1-C*->??+DO-GTPase2.2->

■ **GCA__001854695.1; Frankia sp. NRRL B-16219; counts= 1; multicellularity status: TRUE**

- HSP70->TPR__containing__alpha__helical+Fibronectin-like__repeats->ZnR+ZnR+DO-GTPase2.2->GAP1-N2+GAP1-M+GAP1-C*->TM+TM+TM+TM+ZnR+ZnR+DO-GTPase2.2->

■ **GCA__001860525.1; Paenibacillus sp. LC231; counts= 1; multicellularity status: TRUE**

- TM+vWA(canonical)+YtKA-like+TM+??->Tubulin+coiled__coil+??->Tubulin+coiled__coil+HTH?+??->TM+TM+TM+vWA(canonical)+TM+TM+TM+polar__tail->Fibronectin-like__repeats+??->ZnR+ZnR+DO-GTPase2.2+ZnR+ZnR->GAP1-N2+GAP1-M+GAP1-C+polar__tail+TM+beta-rich*->

■ **GCF__001865165.1; Mycolicibacterium llatzerense; counts= 1; multicellularity status: FALSE**

- GrpE->HSP70->TPR__containing__alpha__helical+Fibronectin-like__repeats->ZnR+ZnR+DO-GTPase2.2->GAP1-N2+GAP1-M+GAP1-C*->TM+TM+TM+TM+ZnR+ZnR+DO-GTPase2.2->??->HSP70->vWA(canonical)->

■ **GCF__001865795.1; Streptomyces albidoflavus; counts= 1; multicellularity status: TRUE**

- GAP1-N2+GAP1-M+GAP1-C*->

■ **GCA__001866665.1; Streptomyces sp. MUSC 1; counts= 1; multicellularity status: TRUE**

- ZnR+ZnR+DO-GTPase2.2->GAP1-N2+GAP1-M+GAP1-C*->TM+TM+TM+TM+ZnR+ZnR+DO-GTPase2.2->

■ **GCA__001813755.1; Corynebacterium sp. HMSC074H12; counts= 1; multicellularity status: FALSE**

- polar__leader+TM+TM+TM->TM+vWA(canonical)+Fibronectin-like__repeats+TM+beta-rich+lc_tail->Tubulin+alpha__helical+??+??->GAP__unknown1+TM+TM+GAP__unknown2->HRDC-like?+Fibronectin-like__repeats->GAP1-N2+GAP1-M+GAP1-C*->beta-rich+vWA(canonical)->??+DO-GTPase2.2->polar__leader+TM+TM+TM->

■ **GCA__001877055.1; Nocardiopsis dassonvillei; counts= 1; multicellularity status: TRUE**

- TPR__containing__alpha__helical+Fibronectin-like__repeats+extended__insert+Fibronectin-like__repeats->ZnR+ZnR+DO-GTPase2.2->GAP1-N2+GAP1-M+GAP1-C*->TM+TM+TM+TM+ZnR+ZnR+DO-GTPase2.2->

■ **GCF__001885705.1; Streptomyces cinnamoneus; counts= 1; multicellularity status: TRUE**

- GAP1-N2+GAP1-M+GAP1-C*->

■ **GCA__900101595.1; Paenibacillus; counts= 1; multicellularity status: TRUE**

- TM+vWA(canonical)+YtKA-like+TM+??->Tubulin+coiled__coil+??->Tubulin+coiled__coil+HTH?+??->TM+TM+TM+vWA(canonical)+TM+TM+TM+polar__tail->Fibronectin-like__repeats+??->ZnR+ZnR+DO-GTPase2.2+ZnR+ZnR->GAP1-N2+GAP1-M+GAP1-C+polar__tail+TM+Cadherin-like*->

■ **GCA__900114875.1; Chryseobacterium limigenitum; counts= 1; multicellularity status: FALSE**

- GAP2->ZnR+DO-GTPase2.1->DO-GTPase1->GAP1-N1+GAP1-M+GAP1-C+EAD1*->PNP_UDP_1+EAD1->

■ **GCA__900103235.1; Ruminococcus; counts= 1; multicellularity status: TRUE**

- GrpE->HSP70->ZnR+ZnR+DO-GTPase2.2->?->?->GAP1-N2+GAP1-M+GAP1-C*->

■ **GCA__900119365.1; Streptomyces atratus; counts= 1; multicellularity status: TRUE**

- GAP1-N2+GAP1-M+GAP1-C*->

■ **GCF__900128415.1; Anaerococcus mediterraneensis; counts= 1; multicellularity status: TRUE**

- TM+TM+TM+TM+ZnR+ZnR+DO-GTPase2.2->GrpE->HSP70->HSP70+vWA(canonical)->DnaJ+DnaJ+ZnR+ZnR+coiled__coil+Fibronectin-like__repeats->ZnR+ZnR+DO-GTPase2.2->GAP1-N2+GAP1-M+GAP1-C*->TM+TM+TM+TM+ZnR+ZnR+DO-GTPase2.2->

■ **GCF__001894745.1; Paenibacillus sp. NAIST15-1; counts= 1; multicellularity status: TRUE**

- TM+vWA(canonical)+YtKA-like+TM+??->Tubulin+coiled__coil+??->Tubulin+coiled__coil+HTH?+??->TM+TM+TM+vWA(canonical)+TM+TM+TM+polar__tail->Fibronectin-like__repeats+??->ZnR+ZnR+DO-GTPase2.2+ZnR+ZnR->GAP1-N2+GAP1-M+GAP1-C+polar__tail+TM+beta-rich*->

■ **GCA__900143165.1; Paenibacillus sp. ov031; counts= 1; multicellularity status: TRUE**

- TM+vWA(canonical)+YtKA-like+TM+??->Tubulin+coiled_coil+??->Tubulin+coiled_coil+HTH?+??->TM+TM+TM+vWA(canonical)+TM+TM+TM+polar_tail->Fibronectin-like_repeats+??->ZnR+ZnR+DO-GTPase2.2+ZnR+ZnR->GAP1-N2+GAP1-M+GAP1-C+polar_tail+TM+beta-rich*->

■ **GCF_001895005.1; Rhodococcus yunnanensis; counts= 1; multicellularity status: TRUE**

- polar_leader+TM+TM+TM->TM+vWA(canonical)+Fibronectin-like_repeats+TM+beta-rich+lc_tail->Tubulin+alpha_helical+??+??->GAP_unknown1+TM+TM+GAP_unknown2->HRDC-like?+Fibronectin-like_repeats->GAP1-N2+GAP1-M+GAP1-C*->beta-rich+vWA(canonical)->??+DO-GTPase2.2->

■ **GCA_900101735.1; Rhodococcus tukisamuensis; counts= 1; multicellularity status: TRUE**

- Tubulin+alpha_helical+??+??->GAP_unknown1+TM+TM+GAP_unknown2->HRDC-like?+Fibronectin-like_repeats->GAP1-N2+GAP1-M+GAP1-C*->beta-rich+vWA(canonical)->??+DO-GTPase2.2->

■ **GCA_900105905.1; Rhodococcus koreensis; counts= 1; multicellularity status: TRUE**

- polar_leader+TM+TM+TM->TM+vWA(canonical)+Fibronectin-like_repeats+TM+beta-rich+lc_tail->Tubulin+alpha_helical+??+??->GAP_unknown1+TM+TM+GAP_unknown2->HRDC-like?+Fibronectin-like_repeats->GAP1-N2+GAP1-M+GAP1-C*->beta-rich+vWA(canonical)->??+DO-GTPase2.2->

■ **GCA_900143025.1; Ruminococcus flavefaciens; counts= 1; multicellularity status: TRUE**

- HSP70->HSP70+vWA(canonical)->?->?->DnaJ+DnaJ+ZnR+ZnR+coiled_coil+Fibronectin-like_repeats->ZnR+ZnR+DO-GTPase2.2->ZnR+ZnR+DO-GTPase2.2->GAP1-N2+GAP1-M+GAP1-C*->

■ **GCA_900141915.1; Tessaracoccus bendigoensis; counts= 1; multicellularity status: TRUE**

- polar_leader+TM+TM+TM->TM+vWA(canonical)+Fibronectin-like_repeats+TM+beta-rich+lc_tail->Tubulin+alpha_helical+??+??->GAP_unknown1+TM+TM+GAP_unknown2->?->HRDC-like?+Fibronectin-like_repeats->GAP1-N2+GAP1-M+GAP1-C*->?->??+DO-GTPase2.2->

■ **GCF_001895105.1; Streptomyces sp. NBRC 110465; counts= 1; multicellularity status: TRUE**

- GAP1-N2+GAP1-M+GAP1-C*->

■ **GCA_900141985.1; Nocardiopsis flavescens; counts= 1; multicellularity status: TRUE**

- GrpE->HSP70->TPR_containing_alpha_helical+Fibronectin-like_repeats+extended_insert+Fibronectin-like_repeats->ZnR+ZnR+DO-GTPase2.2->GAP1-N2+GAP1-M+GAP1-C*->TM+TM+TM+TM+ZnR+ZnR+DO-GTPase2.2->

■ **GCA_001902295.1; Clostridium kluyveri; counts= 1; multicellularity status: FALSE**

- Fibronectin-like_repeats+??->ZnR+ZnR+DO-GTPase2.2->GAP1-N2+GAP1-M+GAP1-C*->TM+TM+TM+TM+ZnR+ZnR+DO-GTPase2.2->TM+vWA(canonical)+YtKA-like+TM+??->Tubulin+coiled_coil+??->Tubulin+coiled_coil+HTH?+??->Fibronectin-like_repeats+??->ZnR+ZnR+DO-GTPase2.2+ZnR+ZnR->

■ **GCA_001905145.1; Nocardiopsis sp. TSRI0078; counts= 1; multicellularity status: TRUE**

- TPR_containing_alpha_helical+Fibronectin-like_repeats+extended_insert+Fibronectin-like_repeats->ZnR+ZnR+DO-GTPase2.2->GAP1-N2+GAP1-M+GAP1-C*->TM+TM+TM+TM+ZnR+ZnR+DO-GTPase2.2->

■ **GCA_001905125.1; Streptomyces sp. TSRI0281; counts= 1; multicellularity status: TRUE**

- GAP1-N2+GAP1-M+GAP1-C*->

■ **GCA_001905365.1; Streptomyces sp. CB02488; counts= 1; multicellularity status: TRUE**

- GAP1-N2+GAP1-M+GAP1-C*->

■ **GCA_001905645.1; Streptomyces sp. CB02115; counts= 1; multicellularity status: TRUE**

- GAP1-N2+GAP1-M+GAP1-C*->

■ **GCA_001905505.1; Streptomyces sp. CB02058; counts= 1; multicellularity status: TRUE**

- GAP1-N2+GAP1-M+GAP1-C*->

■ **GCA_001905005.1; Streptomyces sp. CB02923; counts= 1; multicellularity status: TRUE**

- TPR_containing_alpha_helical+Fibronectin-like_repeats->ZnR+ZnR+DO-GTPase2.2->GAP1-N2+GAP1-M+GAP1-C*->TM+TM+TM+TM+ZnR+ZnR+DO-GTPase2.2+TM->vWA(canonical)->PP2C->Pkinase+ZnR+beta-rich->

■ **GCA_001905405.1; Streptomyces sp. TSRI0445; counts= 1; multicellularity status: TRUE**

- GAP1-N2+GAP1-M+GAP1-C*->

■ **GCA_001905795.1; Streptomyces sp. CB01580; counts= 1; multicellularity status: TRUE**

- GAP1-N2+GAP1-M+GAP1-C*->

■ **GCA_001905785.1; Streptomyces sp. CB02130; counts= 1; multicellularity status: TRUE**

- GAP1-N2+GAP1-M+GAP1-C*->

■ **GCA_001905755.1; Amycolatopsis sp. CB00013; counts= 1; multicellularity status: TRUE**

- vWA(canonical)+beta-rich+low_complexity->Tubulin+alpha_helical+??+??->??+TM+TM+TM+TM+??->Pkinase+TM->ZnR+ZnR+DO-GTPase2.2->GAP1-N2+GAP1-M+GAP1-C+TM*->

■ **GCA_001905735.1; Streptomyces sp. CB01249; counts= 1; multicellularity status: TRUE**

- GAP1-N2+GAP1-M+GAP1-C*->

■ **GCA_900015285.1; Corynebacterium variabile; counts= 1; multicellularity status: FALSE**

- polar_leader+TM+TM+TM->TM+vWA(canonical)+Fibronectin-like_repeats+TM+beta-rich+lc_tail->Tubulin+alpha_helical+??+??->GAP_unknown1+TM+TM+GAP_unknown2->HRDC-like?+Fibronectin-like_repeats->GAP1-N2+GAP1-M+GAP1-C*->beta-rich+vWA(canonical)->??+DO-GTPase2.2->

■ **GCA_001905825.1; Streptomyces sp. CB02009; counts= 1; multicellularity status: TRUE**

- TPR_containing_alpha_helical+Fibronectin-like_repeats->ZnR+ZnR+DO-GTPase2.2->GAP1-N2+GAP1-M+GAP1-C*->TM+TM+TM+TM+ZnR+ZnR+DO-GTPase2.2+TM->vWA(canonical)->PSE->Pkinase+ZnR+beta-rich->

■ **GCA_001907615.1; Mycobacterium sp. SWH-M3; counts= 1; multicellularity status: FALSE**

- GAP_unknown1+TM+TM+GAP_unknown2->HRDC-like?+Fibronectin-like_repeats->GAP1-N2+GAP1-M+GAP1-C*->beta-rich+vWA(canonical)->??+DO-GTPase2.2->TM+DUF4190+TM+PknH_C->TM+DUF4190+TM+PknH_C->

■ **GCA_001905705.1; Streptomyces sp. CB02460; counts= 1; multicellularity status: TRUE**

- GAP1-N2+GAP1-M+GAP1-C*->

■ **GCA_001909085.1; Paenibacillus sp. P32E; counts= 1; multicellularity status: TRUE**

- TM+vWA(canonical)+YtKA-like+TM+??->Tubulin+coiled_coil+??->Tubulin+coiled_coil+HTH?+??->TM+TM+TM+vWA(canonical)+TM+TM+TM+polar_tail->Fibronectin-like_repeats+??->ZnR+ZnR+DO-GTPase2.2+ZnR+ZnR->GAP1-N2+GAP1-M+GAP1-C+polar_tail+TM+beta-rich*->

■ **GCA_001908275.1; Paenibacillus xylanexedens; counts= 1; multicellularity status: TRUE**

- Tubulin+coiled_coil+??->Tubulin+coiled_coil+HTH?+??->TM+TM+TM+vWA+TM+TM+TM+polar_tail->Fibronectin-like_repeats+??->ZnR+ZnR+DO-GTPase2.2->GAP1-N2+GAP1-M+GAP1-C+polar_tail*->

■ **GCA_001909045.1; Paenibacillus sp. P3E; counts= 1; multicellularity status: TRUE**

- TM+vWA(canonical)+YtKA-like+TM+??->Tubulin+coiled_coil+??->Tubulin+coiled_coil+HTH?+??->TM+TM+TM+vWA(canonical)+TM+TM+TM+polar_tail->Fibronectin-like_repeats+??->ZnR+ZnR+DO-GTPase2.2+ZnR+ZnR->GAP1-N2+GAP1-M+GAP1-C+polar_tail+TM+beta-rich*->

■ **GCA_001909055.1; Paenibacillus sp. P26E; counts= 1; multicellularity status: TRUE**

- TM+vWA(canonical)+YtKA-like+TM+??->Tubulin+coiled_coil+??->Tubulin+coiled_coil+HTH?+??->TM+TM+TM+vWA(canonical)+TM+TM+TM+polar_tail->Fibronectin-like_repeats+??->ZnR+ZnR+DO-GTPase2.2+ZnR+ZnR->GAP1-N2+GAP1-M+GAP1-C+polar_tail+TM+beta-rich*->

■ **GCA_001909095.1; Paenibacillus sp. P46E; counts= 1; multicellularity status: TRUE**

- TM+vWA(canonical)+YtKA-like+TM+??->Tubulin+coiled_coil+??->Tubulin+coiled_coil+HTH?+??->TM+TM+TM+vWA(canonical)+TM+TM+TM+polar_tail->Fibronectin-like_repeats+??->ZnR+ZnR+DO-GTPase2.2+ZnR+ZnR->GAP1-N2+GAP1-M+GAP1-C+polar_tail+TM+beta-rich*->

■ **GCF_900129635.1; Singulisphaera sp. GP187; counts= 1; multicellularity status: TRUE**

- DO-GTPase2.1+TM->GAP1-N2+GAP1-M+GAP1-C*->

■ **GCF_000010225.1; Corynebacterium; counts= 1; multicellularity status: FALSE**

- polar_leader+TM+TM+TM->TM+vWA(canonical)+Fibronectin-like_repeats+TM+beta-rich+lc_tail->Tubulin+alpha_helical+??+??->GAP_unknown1+TM+TM+GAP_unknown2->HRDC-like?+Fibronectin-like_repeats->GAP1-N2+GAP1-M+GAP1-C*->beta-rich+vWA(canonical)->??+DO-GTPase2.2->

■ **GCF_001912725.1; Corynebacterium glutamicum; counts= 1; multicellularity status: FALSE**

- polar_leader+TM+TM+TM->TM+vWA(canonical)+Fibronectin-like_repeats+TM+beta-rich+lc_tail->Tubulin+alpha_helical+??+??->GAP_unknown1+TM+TM+GAP_unknown2->HRDC-like?+Fibronectin-like_repeats->GAP1-N2+GAP1-M+GAP1-C*->beta-rich+vWA(canonical)->??+DO-GTPase2.2->

■ **GCF_001912755.2; Corynebacterium glutamicum; counts= 1; multicellularity status: FALSE**

- polar_leader+TM+TM+TM->TM+vWA(canonical)+Fibronectin-like_repeats+TM+beta-rich+lc_tail->Tubulin+alpha_helical+??+??->GAP_unknown1+TM+TM+GAP_unknown2->HRDC-like?+Fibronectin-like_repeats->GAP1-N2+GAP1-M+GAP1-C*->beta-rich+vWA(canonical)->??+DO-GTPase2.2->

■ **GCF_000828015.1; Corynebacterium glutamicum; counts= 1; multicellularity status: FALSE**

- polar_leader+TM+TM+TM->TM+vWA(canonical)+Fibronectin-like_repeats+TM+beta-rich+lc_tail->Tubulin+alpha_helical+??+??->GAP_unknown1+TM+TM+GAP_unknown2->HRDC-like?+Fibronectin-like_repeats->GAP1-N2+GAP1-M+GAP1-C*->beta-rich+vWA(canonical)->??+DO-GTPase2.2->

■ **GCA_900102965.1; Paenibacillus jilunlii; counts= 1; multicellularity status: TRUE**

- TM+vWA(canonical)+YtKA-like+TM+??->Tubulin+coiled_coil+??->Tubulin+coiled_coil+HTH?+??->TM+TM+TM+vWA(canonical)+TM+TM+TM+polar_tail->Fibronectin-like_repeats+??->ZnR+ZnR+DO-GTPase2.2+ZnR+ZnR->GAP1-N2+GAP1-M+GAP1-C+polar_tail+TM+beta-rich*->

■ **GCA_002217275.1; Flavobacterium; counts= 1; multicellularity status: FALSE**

- GAP2->ZnR+DO-GTPase2.1->DO-GTPase1->GAP1-N1+GAP1-M+GAP1-C+EAD1*->PNP_UDP_1+EAD1->

■ **GCA_900115275.1; Pseudomonas syringae; counts= 1; multicellularity status: FALSE**

- GAP2->ZnR+DO-GTPase2.1->DO-GTPase1->GAP1-N1+GAP1-M+GAP1-C+EAD1*->CASPASE+EAD1->

■ **GCA_900104815.1; Streptomyces misionensis; counts= 1; multicellularity status: TRUE**

- PP2C->Pkinase->?->?->?->HSP70_domain_2+low_complexity+domain_betaX->ZnR+ZnR+DO-GTPase2.2->GAP1-N2+GAP1-M+GAP1-C*->TM+TM+TM+TM+ZnR+ZnR+DO-GTPase2.2->

■ **GCA_900111245.1; Streptomyces qinglanensis; counts= 1; multicellularity status: TRUE**

- PP2C->Pkinase->?->?->?->HSP70_domain_2+low_complexity+domain_betaX->ZnR+ZnR+DO-GTPase2.2->GAP1-N2+GAP1-M+GAP1-C*->TM+TM+TM+TM+ZnR+ZnR+DO-GTPase2.2->

■ **GCA_001922145.1; Paenibacillus polymyxa; counts= 1; multicellularity status: TRUE**

- TM+vWA(canonical)+YtKA-like+TM+??->Tubulin+coiled_coil+??->Tubulin+coiled_coil+HTH?+??->TM+TM+TM+vWA(canonical)+TM+TM+TM+polar_tail->Fibronectin-like_repeats+??->ZnR+ZnR+DO-GTPase2.2+ZnR+ZnR->GAP1-N2+GAP1-M+GAP1-C+polar_tail+TM+beta-rich*->

■ **GCA_001929375.1; Actinomyces oris; counts= 1; multicellularity status: FALSE**

- polar_leader+TM+TM+TM->TM+vWA(canonical)+Fibronectin-like_repeats+TM+beta-rich+lc_tail->Tubulin+alpha_helical+??+??->GAP_unknown1+TM+TM+GAP_unknown2->HRDC-like?+Fibronectin-like_repeats->GAP1-N2+GAP1-M+GAP1-C*->?->??+DO-GTPase2.2->

■ **GCF_001936195.1; Corynebacterium glutamicum; counts= 1; multicellularity status: FALSE**

- polar_leader+TM+TM+TM->TM+vWA(canonical)+Fibronectin-like_repeats+TM+beta-rich+lc_tail->Tubulin+alpha_helical+??+??->GAP_unknown1+TM+TM+GAP_unknown2->HRDC-like?+Fibronectin-like_repeats->GAP1-N2+GAP1-M+GAP1-C*->beta-rich+vWA(canonical)->??+DO-GTPase2.2->

■ **GCA_001937485.1; Actinomyces oris; counts= 1; multicellularity status: FALSE**

- polar_leader+TM+TM+TM->TM+vWA(canonical)+Fibronectin-like_repeats+TM+beta-rich+lc_tail->Tubulin+alpha_helical+??+??->GAP_unknown1+TM+TM+GAP_unknown2->HRDC-like?+Fibronectin-like_repeats->GAP1-N2+GAP1-M+GAP1-C*->?->??+DO-GTPase2.2->

■ **GCA_001937415.1; Actinomyces oris; counts= 1; multicellularity status: FALSE**

- polar_leader+TM+TM+TM->TM+vWA(canonical)+Fibronectin-like_repeats+TM+beta-rich+lc_tail->Tubulin+alpha_helical+??+??->GAP_unknown1+TM+TM+GAP_unknown2->HRDC-like?+Fibronectin-like_repeats->GAP1-N2+GAP1-M+GAP1-C*->?->??+DO-GTPase2.2->

■ **GCA_001937725.1; Actinomyces oris; counts= 1; multicellularity status: FALSE**

- polar_leader+TM+TM+TM->TM+vWA(canonical)+Fibronectin-like_repeats+TM+beta-rich+lc_tail->Tubulin+alpha_helical+??+??->GAP_unknown1+TM+TM+GAP_unknown2->HRDC-like?+Fibronectin-like_repeats->GAP1-N2+GAP1-M+GAP1-C*->?->??+DO-GTPase2.2->

■ **GCA_001937425.1; Actinomyces oris; counts= 1; multicellularity status: FALSE**

- polar_leader+TM+TM+TM->TM+vWA(canonical)+Fibronectin-like_repeats+TM+beta-rich+lc_tail->Tubulin+alpha_helical+??+??->||<-?||GAP_unknown1+TM+TM+GAP_unknown2->HRDC-like?+Fibronectin-like_repeats->GAP1-N2+GAP1-M+GAP1-C*->?->??+DO-GTPase2.2->

■ **GCA_001632895.1; Mycobacterium kansasii; counts= 1; multicellularity status: FALSE**

- TM+DUF4190+TM->TM+DUF4190+TM->polar_leader+TM+TM+TM->TM+vWA(canonical)+Fibronectin-like_repeats+TM+beta-rich+lc_tail->Tubulin+alpha_helical+??+??->GAP_unknown1+TM+TM+GAP_unknown2->HRDC-like?+Fibronectin-like_repeats->GAP1-N2+GAP1-M+GAP1-C*->beta-rich+vWA(canonical)->??+DO-GTPase2.2->

■ **GCA_001632905.1; Mycobacterium kansasii; counts= 1; multicellularity status: FALSE**

- TM+DUF4190+TM->TM+DUF4190+TM->polar_leader+TM+TM+TM->TM+vWA(canonical)+Fibronectin-like_repeats+TM+beta-rich+lc_tail->Tubulin+alpha_helical+??+??->GAP_unknown1+TM+TM+GAP_unknown2->HRDC-like?+Fibronectin-like_repeats->GAP1-N2+GAP1-M+GAP1-C*->beta-rich+vWA(canonical)->??+DO-GTPase2.2->TM+DUF4190+TM+PknH_C->

■ **GCA_001632975.1; Mycobacterium kansasii; counts= 1; multicellularity status: FALSE**

- TM+DUF4190+TM->TM+DUF4190+TM->polar_leader+TM+TM+TM->TM+vWA(canonical)+Fibronectin-like_repeats+TM+beta-rich+lc_tail->Tubulin+alpha_helical+??+??->GAP_unknown1+TM+TM+GAP_unknown2->HRDC-like?+Fibronectin-like_repeats->GAP1-N2+GAP1-M+GAP1-C*->beta-rich+vWA(canonical)->??+DO-GTPase2.2->

■ **GCA_001940785.1; Bacillus sp. MRMR6; counts= 1; multicellularity status: NA**

- SP+vWA+YtKA-like+TM+??->Tubulin+coiled_coil+??->Tubulin+coiled_coil+HTH?+??->Fibronectin-like_repeats+??->ZnR+ZnR+DO-GTPase2.2->GAP1-N2+GAP1-M+GAP1-C*->

■ **GCA_001941505.1; Corynebacterium sphenisci; counts= 1; multicellularity status: FALSE**

- polar_leader+TM+TM+TM->TM+vWA(canonical)+Fibronectin-like_repeats+TM+beta-rich+lc_tail->Tubulin+alpha_helical+??+??->GAP_unknown1+TM+TM+GAP_unknown2->HRDC-like?+Fibronectin-like_repeats->GAP1-N2+GAP1-M+GAP1-C*->beta-rich+vWA(canonical)->??+DO-GTPase2.2->

■ **GCF_001941445.1; Corynebacterium aquilae; counts= 1; multicellularity status: FALSE**

- polar_leader+TM+TM+TM->TM+vWA(canonical)+Fibronectin-like_repeats+TM+beta-rich+lc_tail->Tubulin+alpha_helical+??+??->GAP_unknown1+TM+TM+GAP_unknown2->HRDC-like?+Fibronectin-like_repeats->GAP1-N2+GAP1-M+GAP1-C*->beta-rich+vWA(canonical)->??+DO-GTPase2.2->

■ **GCA_006539465.1; Corynebacterium flavescens; counts= 1; multicellularity status: FALSE**

- polar_leader+TM+TM+TM->TM+vWA(canonical)+Fibronectin-like_repeats+TM+beta-rich+lc_tail->Tubulin+alpha_helical+??+??->GAP_unknown1+TM+TM+GAP_unknown2->HRDC-like?+Fibronectin-like_repeats->GAP1-N2+GAP1-M+GAP1-C*->beta-rich+vWA(canonical)->??+DO-GTPase2.2->polar_leader+TM+TM+TM->

■ **GCA_001683055.1; Corynebacterium; counts= 1; multicellularity status: FALSE**

- polar_leader+TM+TM+TM->TM+vWA(canonical)+Fibronectin-like_repeats+TM+beta-rich+lc_tail->Tubulin+alpha_helical+??+??->GAP_unknown1+TM+TM+GAP_unknown2->HRDC-like?+Fibronectin-like_repeats->GAP1-N2+GAP1-M+GAP1-C*->beta-rich+vWA(canonical)->??+DO-GTPase2.2->

■ **GCA_001942465.1; Actinomadura sp. CNU-125; counts= 1; multicellularity status: TRUE**

- GAP2->DO-GTPase2.1->DO-GTPase1->GAP1-N1+GAP1-M+GAP1-C+EAD1*->CASPASE+EAD1->

■ **GCA_001942625.1; Mycobacterium sp. 27335; counts= 1; multicellularity status: FALSE**

- HRDC-like?+Fibronectin-like_repeats->GAP1-N2+GAP1-M+GAP1-C*->??+DO-GTPase2.2->

■ **GCA_001942045.1; Mycolicibacterium porcinum; counts= 1; multicellularity status: FALSE**

- HRDC-like?+Fibronectin-like_repeats->GAP1-N2+GAP1-M+GAP1-C*->??+DO-GTPase2.2->

■ **GCA_001952675.1; Leptospira santarosai; counts= 1; multicellularity status: FALSE**

- Calcineurin+GAPEAD1->GAP2->ZnR+DO-GTPase2.1->DO-GTPase1->DO-GTPase1->GAP1-N1+GAP1-M+GAP1-C*->

■ **GCA_001431765.1; Streptomyces sp. FR-008; counts= 1; multicellularity status: TRUE**

- GAP1-N2+GAP1-M+GAP1-C*->

■ **GCA_001955915.1; Paenibacillus sp. FSL R7-0333; counts= 1; multicellularity status: TRUE**

- TM+vWA(canonical)+YtKA-like+TM+??->Tubulin+coiled_coil+??->Tubulin+coiled_coil+HTH?+??->TM+TM+TM+vWA(canonical)+TM+TM+TM+polar_tail->Fibronectin-like_repeats+??->ZnR+ZnR+DO-GTPase2.2+ZnR+ZnR->GAP1-N2+GAP1-M+GAP1-C+polar_tail+TM+beta-rich*->

■ **GCA_001953885.1; Streptomyces sp. IMTB 2501; counts= 1; multicellularity status: TRUE**

- GrpE->HSP70->TPR_containing_alpha_helical+Fibronectin-like_repeats->ZnR+ZnR+DO-GTPase2.2->GAP1-N2+GAP1-M+GAP1-C*->TM+TM+TM+TM+ZnR+ZnR+DO-GTPase2.2+Jelly_roll+beta-sandwich->||<-STAND_NTPase+TM+TM+TM+TM+TM+TM+TM

■ **GCA_001954285.1; Paenibacillus odorifer; counts= 1; multicellularity status: TRUE**

- TM+vWA(canonical)+YtKA-like+TM+??->Tubulin+coiled_coil+??->Tubulin+coiled_coil+HTH?+??->TM+TM+TM+vWA(canonical)+TM+TM+TM+polar_tail->Fibronectin-like_repeats+??->ZnR+ZnR+DO-GTPase2.2+ZnR+ZnR->GAP1-N2+GAP1-M+GAP1-C+polar_tail+TM+beta-rich*->

■ **GCA_001954345.1; Paenibacillus odorifer; counts= 1; multicellularity status: TRUE**

- TM+vWA(canonical)+YtKA-like+TM+??->Tubulin+coiled_coil+??->Tubulin+coiled_coil+HTH?+??->TM+TM+TM+vWA(canonical)+TM+TM+TM+polar_tail->Fibronectin-like_repeats+??->ZnR+ZnR+DO-GTPase2.2+ZnR+ZnR->GAP1-N2+GAP1-M+GAP1-C+polar_tail+TM+beta-rich*->

■ **GCA_001954415.1; Paenibacillus borealis; counts= 1; multicellularity status: TRUE**

- TM+vWA(canonical)+YtKA-like+TM+??->Tubulin+coiled_coil+??->Tubulin+coiled_coil+HTH?+??->TM+TM+TM+vWA(canonical)+TM+TM+TM+polar_tail->Fibronectin-like_repeats+??->?->ZnR+ZnR+DO-GTPase2.2+ZnR+ZnR->GAP1-N2+GAP1-M+GAP1-C+polar_tail+TM+beta-rich*->

■ **GCA_001954425.1; Paenibacillus odorifer; counts= 1; multicellularity status: TRUE**

- TM+vWA(canonical)+YtKA-like+TM+??->Tubulin+coiled_coil+??->Tubulin+coiled_coil+HTH?+??->TM+TM+TM+vWA(canonical)+TM+TM+TM+polar_tail->Fibronectin-like_repeats+??->ZnR+ZnR+DO-GTPase2.2+ZnR+ZnR->GAP1-N2+GAP1-M+GAP1-C+polar_tail+TM+beta-rich*->

■ **GCA_001954575.1; Paenibacillus odorifer; counts= 1; multicellularity status: TRUE**

- TM+vWA(canonical)+YtKA-like+TM+??->Tubulin+coiled_coil+??->Tubulin+coiled_coil+HTH?+??->TM+TM+TM+vWA(canonical)+TM+TM+TM+polar_tail->Fibronectin-like_repeats+??->ZnR+ZnR+DO-GTPase2.2+ZnR+ZnR->GAP1-N2+GAP1-M+GAP1-C+polar_tail+TM+beta-rich*->

■ **GCA_003255855.1; Paenibacillus odorifer; counts= 1; multicellularity status: TRUE**

- TM+vWA(canonical)+YtKA-like+TM+??->Tubulin+coiled_coil+??->Tubulin+coiled_coil+HTH?+??->TM+TM+TM+vWA(canonical)+TM+TM+TM+polar_tail->Fibronectin-like_repeats+??->ZnR+ZnR+DO-GTPase2.2+ZnR+ZnR->GAP1-N2+GAP1-M+GAP1-C+polar_tail+TM+beta-rich*->

■ **GCA_001955765.1; Paenibacillus odorifer; counts= 1; multicellularity status: TRUE**

- TM+vWA(canonical)+YtKA-like+TM+??->Tubulin+coiled_coil+??->Tubulin+coiled_coil+HTH?+??->TM+TM+TM+vWA(canonical)+TM+TM+TM+polar_tail->Fibronectin-like_repeats+??->ZnR+ZnR+DO-GTPase2.2+ZnR+ZnR->GAP1-N2+GAP1-M+GAP1-C+polar_tail+TM+beta-rich*->

■ **GCA_001955785.1; Paenibacillus odorifer; counts= 1; multicellularity status: TRUE**

- TM+vWA(canonical)+YtKA-like+TM+??->Tubulin+coiled_coil+??->Tubulin+coiled_coil+HTH?+??->TM+TM+TM+vWA(canonical)+TM+TM+TM+polar_tail->Fibronectin-like_repeats+??->ZnR+ZnR+DO-GTPase2.2+ZnR+ZnR->GAP1-N2+GAP1-M+GAP1-C+polar_tail+TM+beta-rich*->

■ **GCA_001955865.1; Paenibacillus glucanolyticus; counts= 1; multicellularity status: TRUE**

- TM+vWA(canonical)+YtKA-like+TM+??->Tubulin+coiled_coil+??->Tubulin+coiled_coil+HTH?+??->TM+TM+TM+vWA(canonical)+TM+TM+TM+polar_tail->Fibronectin-like_repeats+??->ZnR+ZnR+DO-GTPase2.2+ZnR+ZnR->GAP1-N2+GAP1-M+GAP1-C+polar_tail+TM+beta-rich*->

■ **GCA_001955925.1; Paenibacillus sp. FSL R7-0337; counts= 1; multicellularity status: TRUE**

- TM+TM+TM+vWA+TM+TM+TM+polar_tail->Fibronectin-like_repeats+??->ZnR+ZnR+DO-GTPase2.2->GAP1-N2+GAP1-M+GAP1-C+polar_tail*->

■ **GCA_001953865.1; Amycolatopsis coloradensis; counts= 1; multicellularity status: TRUE**

- GrpE->HSP70->TPR_containing_alpha_helical+Fibronectin-like_repeats->ZnR+ZnR+DO-GTPase2.2->GAP1-N2+GAP1-M+GAP1-C*->PSE->?->HSP70->vWA(canonical)->||<-?<-?<-HSP70<-unknown+CASPASE

■ **GCA_001956115.1; Paenibacillus peoriae; counts= 1; multicellularity status: TRUE**

- TM+vWA(canonical)+YtKA-like+TM+??->Tubulin+coiled_coil+??->Tubulin+coiled_coil+HTH?+??->TM+TM+TM+vWA(canonical)+TM+TM+TM+polar_tail->Fibronectin-like_repeats+??->ZnR+ZnR+DO-GTPase2.2+ZnR+ZnR->GAP1-N2+GAP1-M+GAP1-C+polar_tail+TM+beta-rich*->

■ **GCA_001956185.1; Paenibacillus rhizosphaerae; counts= 1; multicellularity status: TRUE**

- TM+vWA(canonical)+YtKA-like+TM+??->Tubulin+coiled_coil+??->Tubulin+coiled_coil+HTH?+??->TM+TM+TM+vWA(canonical)+TM+TM+TM+polar_tail->Fibronectin-like_repeats+??->ZnR+ZnR+DO-GTPase2.2+ZnR+ZnR->GAP1-N2+GAP1-M+GAP1-C+polar_tail+TM+beta-rich*->

■ **GCA_001954455.1; Paenibacillus odorifer; counts= 1; multicellularity status: TRUE**

- TM+vWA(canonical)+YtKA-like+TM+??->Tubulin+coiled_coil+??->Tubulin+coiled_coil+HTH?+??->TM+TM+TM+vWA(canonical)+TM+TM+TM+polar_tail->Fibronectin-like_repeats+??->ZnR+ZnR+DO-GTPase2.2+ZnR+ZnR->GAP1-N2+GAP1-M+GAP1-C+polar_tail+TM+beta-rich*->

■ **GCA_001954515.1; Paenibacillus odorifer; counts= 1; multicellularity status: TRUE**

- TM+vWA(canonical)+YtKA-like+TM+??->Tubulin+coiled_coil+??->Tubulin+coiled_coil+HTH?+??->TM+TM+TM+vWA(canonical)+TM+TM+TM+polar_tail->Fibronectin-like_repeats+??->ZnR+ZnR+DO-GTPase2.2+ZnR+ZnR->GAP1-N2+GAP1-M+GAP1-C+polar_tail+TM+beta-rich*->

■ **GCA_001954135.1; Mycobacterium sp. GA-1841; counts= 1; multicellularity status: FALSE**

- HRDC-like?+Fibronectin-like_repeats->GAP1-N2+GAP1-M+GAP1-C*->??+DO-GTPase2.2->

■ **GCA_001954145.1; Mycolicibacterium fortuitum; counts= 1; multicellularity status: FALSE**

- HRDC-like?+Fibronectin-like_repeats->GAP1-N2+GAP1-M+GAP1-C*->??+DO-GTPase2.2->

■ **GCA_001954595.1; Paenibacillus odorifer; counts= 1; multicellularity status: TRUE**

- TM+vWA(canonical)+YtKA-like+TM+??->Tubulin+coiled_coil+??->Tubulin+coiled_coil+HTH?+??->TM+TM+TM+vWA(canonical)+TM+TM+TM+polar_tail->Fibronectin-like_repeats+??->ZnR+ZnR+DO-GTPase2.2+ZnR+ZnR->GAP1-N2+GAP1-M+GAP1-C+polar_tail+TM+beta-rich*->

■ **GCA_001955855.1; Paenibacillus sp. FSL R5-0765; counts= 1; multicellularity status: TRUE**

- TM+vWA(canonical)+YtKA-like+TM+??->Tubulin+coiled_coil+??->Tubulin+coiled_coil+HTH?+??->TM+TM+TM+vWA(canonical)+TM+TM+TM+polar_tail->Fibronectin-like_repeats+??->ZnR+ZnR+DO-GTPase2.2+ZnR+ZnR->GAP1-N2+GAP1-M+GAP1-C+polar_tail+TM+beta-rich*->

■ **GCA_001954205.1; Mycolicibacterium conceptionense; counts= 1; multicellularity status: FALSE**

- HRDC-like?+Fibronectin-like_repeats->GAP1-N2+GAP1-M+GAP1-C*->??+DO-GTPase2.2->

■ **GCA_001954655.1; Paenibacillus odorifer; counts= 1; multicellularity status: TRUE**

- TM+vWA(canonical)+YtKA-like+TM+??->Tubulin+coiled_coil+??->Tubulin+coiled_coil+HTH?+??->TM+TM+TM+vWA(canonical)+TM+TM+TM+polar_tail->Fibronectin-like_repeats+??->ZnR+ZnR+DO-GTPase2.2+ZnR+ZnR->GAP1-N2+GAP1-M+GAP1-C+polar_tail+TM+beta-rich*->

■ **GCA_001955595.1; Paenibacillus odorifer; counts= 1; multicellularity status: TRUE**

- Tubulin+coiled_coil+??->Tubulin+coiled_coil+HTH?+??->TM+TM+TM+vWA(canonical)+TM+TM+TM+polar_tail->Fibronectin-like_repeats+??->ZnR+ZnR+DO-GTPase2.2+ZnR+ZnR->GAP1-N2+GAP1-M+GAP1-C+polar_tail+TM+beta-rich*->

■ **GCA_001955775.1; Paenibacillus odorifer; counts= 1; multicellularity status: TRUE**

- TM+vWA(canonical)+YtKA-like+TM+??->Tubulin+coiled_coil+??->Tubulin+coiled_coil+HTH?+??->TM+TM+TM+vWA(canonical)+TM+TM+TM+polar_tail->Fibronectin-like_repeats+??->ZnR+ZnR+DO-GTPase2.2+ZnR+ZnR->GAP1-N2+GAP1-M+GAP1-C+polar_tail+TM+beta-rich*->

■ **GCA_001956045.1; Paenibacillus sp. FSL H7-0331; counts= 1; multicellularity status: TRUE**

- TM+vWA(canonical)+YtKA-like+TM+??->Tubulin+coiled_coil+??->Tubulin+coiled_coil+HTH?+??->TM+TM+TM+vWA(canonical)+TM+TM+TM+polar_tail->Fibronectin-like_repeats+??->ZnR+ZnR+DO-GTPase2.2+ZnR+ZnR->GAP1-N2+GAP1-M+GAP1-C+polar_tail+TM+Cadherin-like*->

■ **GCA_001953975.1; Mycobacterium sp. NS-7484; counts= 1; multicellularity status: FALSE**

- HRDC-like?+Fibronectin-like_repeats->GAP1-N2+GAP1-M+GAP1-C*->??+DO-GTPase2.2->

■ **GCA_001956105.1; Paenibacillus sp. FSL H8-0259; counts= 1; multicellularity status: TRUE**

- TM+vWA(canonical)+YtKA-like+TM+??->Tubulin+coiled_coil+??->Tubulin+coiled_coil+HTH?+??->TM+TM+TM+vWA(canonical)+TM+TM+TM+polar_tail->Fibronectin-like_repeats+??->ZnR+ZnR+DO-GTPase2.2+ZnR+ZnR->GAP1-N2+GAP1-M+GAP1-C+polar_tail+TM+beta-rich*->

■ **GCA_001956175.1; Paenibacillus; counts= 1; multicellularity status: TRUE**

- TM+vWA(canonical)+YtKA-like+TM+??->Tubulin+coiled_coil+??->Tubulin+coiled_coil+HTH?+??->TM+TM+TM+vWA(canonical)+TM+TM+TM+polar_tail->Fibronectin-like_repeats+??->ZnR+ZnR+DO-GTPase2.2+ZnR+ZnR->GAP1-N2+GAP1-M+GAP1-C+polar_tail+TM+beta-rich*->

■ **GCA_001956225.1; Paenibacillus peoriae; counts= 1; multicellularity status: TRUE**

- TM+vWA(canonical)+YtKA-like+TM+??->Tubulin+coiled_coil+??->Tubulin+coiled_coil+HTH?+??->TM+TM+TM+vWA(canonical)+TM+TM+TM+polar_tail->Fibronectin-like_repeats+??->ZnR+ZnR+DO-GTPase2.2+ZnR+ZnR->GAP1-N2+GAP1-M+GAP1-C+polar_tail+TM+beta-rich*->

■ **GCA_001956295.1; Paenibacillus sp. FSL A5-0031; counts= 1; multicellularity status: TRUE**

- TM+vWA(canonical)+YtKA-like+TM+??->Tubulin+coiled_coil+??->Tubulin+coiled_coil+HTH?+??->TM+TM+TM+vWA(canonical)+TM+TM+TM+polar_tail->Fibronectin-like_repeats+??->ZnR+ZnR+DO-GTPase2.2+ZnR+ZnR->GAP1-N2+GAP1-M+GAP1-C+polar_tail+TM+beta-rich*->

■ **GCA_001954335.1; Paenibacillus odorifer; counts= 1; multicellularity status: TRUE**

- TM+vWA(canonical)+YtKA-like+TM+??->Tubulin+coiled_coil+??->Tubulin+coiled_coil+HTH?+??->TM+TM+TM+vWA(canonical)+TM+TM+TM+polar_tail->Fibronectin-like_repeats+??->ZnR+ZnR+DO-GTPase2.2+ZnR+ZnR->GAP1-N2+GAP1-M+GAP1-C+polar_tail+TM+beta-rich*->

■ **GCA_001954535.1; Paenibacillus odorifer; counts= 1; multicellularity status: TRUE**

- TM+vWA(canonical)+YtKA-like+TM+??->Tubulin+coiled_coil+??->Tubulin+coiled_coil+HTH?+??->TM+TM+TM+vWA(canonical)+TM+TM+TM+polar_tail->Fibronectin-like_repeats+??->ZnR+ZnR+DO-GTPase2.2+ZnR+ZnR->GAP1-N2+GAP1-M+GAP1-C+polar_tail+TM+beta-rich*->

■ **GCA_001954675.1; Paenibacillus odorifer; counts= 1; multicellularity status: TRUE**

- TM+vWA(canonical)+YtKA-like+TM+??->Tubulin+coiled_coil+??->Tubulin+coiled_coil+HTH?+??->TM+TM+TM+vWA(canonical)+TM+TM+TM+polar_tail->Fibronectin-like_repeats+??->ZnR+ZnR+DO-GTPase2.2+ZnR+ZnR->GAP1-N2+GAP1-M+GAP1-C+polar_tail+TM+beta-rich*->

■ **GCA_001955555.1; Paenibacillus odorifer; counts= 1; multicellularity status: TRUE**

- TM+vWA(canonical)+YtKA-like+TM+??->Tubulin+coiled_coil+??->Tubulin+coiled_coil+HTH?+??->TM+TM+TM+vWA(canonical)+TM+TM+TM+polar_tail->Fibronectin-like_repeats+??->ZnR+ZnR+DO-GTPase2.2+ZnR+ZnR->GAP1-N2+GAP1-M+GAP1-C+polar_tail+TM+beta-rich*->

■ **GCA_001955545.1; Paenibacillus odorifer; counts= 1; multicellularity status: TRUE**

- TM+vWA(canonical)+YtKA-like+TM+??->Tubulin+coiled_coil+??->Tubulin+coiled_coil+HTH?+??->TM+TM+TM+vWA(canonical)+TM+TM+TM+polar_tail->Fibronectin-like_repeats+??->ZnR+ZnR+DO-GTPase2.2+ZnR+ZnR->GAP1-N2+GAP1-M+GAP1-C+polar_tail+TM+beta-rich*->

■ **GCF_001955985.1; Paenibacillus pabuli; counts= 1; multicellularity status: TRUE**

- TM+TM+TM+vWA+TM+TM+TM+polar_tail->Fibronectin-like_repeats+??->ZnR+ZnR+DO-GTPase2.2->GAP1-N2+GAP1-M+GAP1-C+polar_tail*->

■ **GCA_001956155.1; Paenibacillus peoriae; counts= 1; multicellularity status: TRUE**

- TM+vWA(canonical)+YtKA-like+TM+??->Tubulin+coiled_coil+??->Tubulin+coiled_coil+HTH?+??->TM+TM+TM+vWA(canonical)+TM+TM+TM+polar_tail->Fibronectin-like_repeats+??->ZnR+ZnR+DO-GTPase2.2+ZnR+ZnR->GAP1-N2+GAP1-M+GAP1-C+polar_tail+TM+beta-rich*->

■ **GCA_001956235.1; Paenibacillus peoriae; counts= 1; multicellularity status: TRUE**

- TM+vWA(canonical)+YtKA-like+TM+??->Tubulin+coiled_coil+??->Tubulin+coiled_coil+HTH?+??->TM+TM+TM+vWA(canonical)+TM+TM+TM+polar_tail->Fibronectin-like_repeats+??->ZnR+ZnR+DO-GTPase2.2+ZnR+ZnR->GAP1-N2+GAP1-M+GAP1-C+polar_tail+TM+beta-rich*->

■ **GCA_001954375.1; Paenibacillus odorifer; counts= 1; multicellularity status: TRUE**

- TM+vWA(canonical)+YtKA-like+TM+??->Tubulin+coiled_coil+??->Tubulin+coiled_coil+HTH?+??->TM+TM+TM+vWA(canonical)+TM+TM+TM+polar_tail->Fibronectin-like_repeats+??->ZnR+ZnR+DO-GTPase2.2+ZnR+ZnR->GAP1-N2+GAP1-M+GAP1-C+polar_tail+TM+beta-rich*->

■ **GCA_001954445.1; Paenibacillus odorifer; counts= 1; multicellularity status: TRUE**

- TM+vWA(canonical)+YtKA-like+TM+??->Tubulin+coiled_coil+??->Tubulin+coiled_coil+HTH?+??->TM+TM+TM+vWA(canonical)+TM+TM+TM+polar_tail->Fibronectin-like_repeats+??->ZnR+ZnR+DO-GTPase2.2+ZnR+ZnR->GAP1-N2+GAP1-M+GAP1-C+polar_tail+TM+beta-rich*->

■ **GCA_001954495.1; Paenibacillus odorifer; counts= 1; multicellularity status: TRUE**

- TM+vWA(canonical)+YtKA-like+TM+??->Tubulin+coiled_coil+??->Tubulin+coiled_coil+HTH?+??->TM+TM+TM+vWA(canonical)+TM+TM+TM+polar_tail->Fibronectin-like_repeats+??->ZnR+ZnR+DO-GTPase2.2+ZnR+ZnR->GAP1-N2+GAP1-M+GAP1-C+polar_tail+TM+beta-rich*->

■ **GCA_001954505.1; Paenibacillus odorifer; counts= 1; multicellularity status: TRUE**

- TM+vWA(canonical)+YtKA-like+TM+??->Tubulin+coiled_coil+??->Tubulin+coiled_coil+HTH?+??->TM+TM+TM+vWA(canonical)+TM+TM+TM+polar_tail->Fibronectin-like_repeats+??->ZnR+ZnR+DO-GTPase2.2+ZnR+ZnR->GAP1-N2+GAP1-M+GAP1-C+polar_tail+TM+beta-rich*->

■ **GCA_001954585.1; Paenibacillus odorifer; counts= 1; multicellularity status: TRUE**

- TM+vWA(canonical)+YtKA-like+TM+??->Tubulin+coiled_coil+??->Tubulin+coiled_coil+HTH?+??->TM+TM+TM+vWA(canonical)+TM+TM+TM+polar_tail->Fibronectin-like_repeats+??->ZnR+ZnR+DO-GTPase2.2+ZnR+ZnR->GAP1-N2+GAP1-M+GAP1-C+polar_tail+TM+beta-rich*->

■ **GCA_001954665.1; Paenibacillus odorifer; counts= 1; multicellularity status: TRUE**

- TM+vWA(canonical)+YtKA-like+TM+??->Tubulin+coiled_coil+??->Tubulin+coiled_coil+HTH?+??->TM+TM+TM+vWA(canonical)+TM+TM+TM+polar_tail->Fibronectin-like_repeats+??->ZnR+ZnR+DO-GTPase2.2+ZnR+ZnR->GAP1-N2+GAP1-M+GAP1-C+polar_tail+TM+beta-rich*->

■ **GCA_001955495.1; Paenibacillus odorifer; counts= 1; multicellularity status: TRUE**

- TM+vWA(canonical)+YtKA-like+TM+??->Tubulin+coiled_coil+??->Tubulin+coiled_coil+HTH?+??->TM+TM+TM+vWA(canonical)+TM+TM+TM+polar_tail->Fibronectin-like_repeats+??->ZnR+ZnR+DO-GTPase2.2+ZnR+ZnR->GAP1-N2+GAP1-M+GAP1-C+polar_tail+TM+beta-rich*->

■ **GCA_001955835.1; Paenibacillus odorifer; counts= 1; multicellularity status: TRUE**

- TM+vWA(canonical)+YtKA-like+TM+??->Tubulin+coiled_coil+??->Tubulin+coiled_coil+HTH?+??->TM+TM+TM+vWA(canonical)+TM+TM+TM+polar_tail->Fibronectin-like_repeats+??->ZnR+ZnR+DO-GTPase2.2+ZnR+ZnR->GAP1-N2+GAP1-M+GAP1-C+polar_tail+TM+beta-rich*->

■ **GCA_001955995.1; Paenibacillus amylolyticus; counts= 1; multicellularity status: TRUE**

- Tubulin+coiled_coil+??->Tubulin+coiled_coil+HTH?+??->TM+TM+TM+vWA+TM+TM+TM+polar_tail->Fibronectin-like_repeats+??->ZnR+ZnR+DO-GTPase2.2->GAP1-N2+GAP1-M+GAP1-C+polar_tail*->

■ **GCA_001955975.1; Paenibacillus lautus; counts= 1; multicellularity status: TRUE**

- TM+vWA(canonical)+YtKA-like+TM+??->?->Tubulin+coiled_coil+??->Tubulin+coiled_coil+HTH?+??->TM+TM+TM+vWA(canonical)+TM+TM+TM+polar_tail->Fibronectin-like_repeats+??->ZnR+ZnR+DO-GTPase2.2+ZnR+ZnR->GAP1-N2+GAP1-M+GAP1-C+polar_tail+TM+beta-rich*->

■ **GCA_001956055.1; Paenibacillus amylolyticus; counts= 1; multicellularity status: TRUE**

- TM+vWA(canonical)+YtKA-like+TM+??->Tubulin+coiled_coil+??->Tubulin+coiled_coil+HTH?+??->TM+TM+TM+vWA(canonical)+TM+TM+TM+polar_tail->Fibronectin-like_repeats+??->ZnR+ZnR+DO-GTPase2.2+ZnR+ZnR->GAP1-N2+GAP1-M+GAP1-C+polar_tail+TM+beta-rich*->

■ **GCA_001956095.1; Paenibacillus sp. FSL H8-0548; counts= 1; multicellularity status: TRUE**

- TM+vWA(canonical)+YtKA-like+TM+??->Tubulin+coiled_coil+??->Tubulin+coiled_coil+HTH?+??->TM+TM+TM+vWA(canonical)+TM+TM+TM+polar_tail->Fibronectin-like_repeats+??->ZnR+ZnR+DO-GTPase2.2+ZnR+ZnR->GAP1-N2+GAP1-M+GAP1-C+polar_tail+TM+beta-rich*->

■ **GCF_900156185.1; Pseudomonas putida; counts= 1; multicellularity status: FALSE**

- Calcineurin+GAPEAD1->GAP2->ZnR+DO-GTPase2.1->DO-GTPase1->GAP1-N1+GAP1-M+GAP1-C*->

■ **GCA_001981425.1; Leptospira santarosai; counts= 1; multicellularity status: FALSE**

- Calcineurin+GAPEAD1->GAP2->ZnR+DO-GTPase2.1->DO-GTPase1->DO-GTPase1->GAP1-N1+GAP1-M+GAP1-C*->

■ **GCA_001975025.1; Streptomyces alfalfae; counts= 1; multicellularity status: TRUE**

- GAP1-N2+GAP1-M+GAP1-C*->vWA(canonical)->PP2C->Pkinase+ZnR+beta-rich->

■ **GCF_900120345.1; Lachnoclostridium phocaeense; counts= 1; multicellularity status: FALSE**

- PP2C->?->DO-GTPase2.1->GAP1-N2+GAP1-M+TM+TM*->

■ **GCA_001983105.1; Frankia asymbiotica; counts= 1; multicellularity status: TRUE**

- GrpE->HSP70->TPR_containing_alpha_helical+Fibronectin-like_repeats->ZnR+ZnR+DO-GTPase2.2->GAP1-N2+GAP1-M+GAP1-C*->TM+TM+TM+TM+ZnR+ZnR+DO-GTPase2.2->

■ **GCA_001983215.1; Frankia sp. CcI49; counts= 1; multicellularity status: TRUE**

- GAP1-N2+GAP1-M+GAP1-C+ZnR+DO-GTPase2*->

■ **GCA_001984175.1; Saccharothrix sp. ALI-22-I; counts= 1; multicellularity status: TRUE**

- vWA(canonical)+beta-rich+low_complexity->?->?->Tubulin+alpha_helical+??+??->??+TM+TM+TM+TM+??->Pkinase+TM->ZnR+ZnR+DO-GTPase2.2->GAP1-N2+GAP1-M+GAP1-C+TM+beta-rich*->

■ **GCA_001983935.1; Fuerstia marisgermanicae; counts= 1; multicellularity status: FALSE**

- ZnR+ZnR+DO-GTPase2.2->GAP1-N2+GAP1-M+TM*->

■ **GCA_001984575.1; Streptomyces sp. MP131-18; counts= 1; multicellularity status: TRUE**

- GAP1-N2+GAP1-M+GAP1-C*->vWA(canonical)->PP2C->Pkinase+ZnR+beta-rich->||<-TPR_containing_alpha_helical+Fibronectin-like_repeats

■ **GCA_900090055.1; Cuniculiplasma divulgatum; counts= 1; multicellularity status: TRUE**

- TM+TM+TM+TM+TM+TM+DO-GTPase2.1->GAP1-N2+GAP1-M+GAP1-C*->??->TM_region+DO-GTPase1->

■ **GCA_000742735.1; Corynebacterium glutamicum; counts= 1; multicellularity status: FALSE**

- polar_leader+TM+TM+TM->TM+vWA(canonical)+Fibronectin-like_repeats+TM+beta-rich+lc_tail->Tubulin+alpha_helical+??+??->GAP_unknown1+TM+TM+GAP_unknown2->HRDC-like?+Fibronectin-like_repeats->GAP1-N2+GAP1-M+GAP1-C*->beta-rich+vWA(canonical)->??+DO-GTPase2.2->

■ **GCA_001997295.1; Tessaracoccus flavus; counts= 1; multicellularity status: FALSE**

- polar_leader+TM+TM+TM->TM+vWA(canonical)+Fibronectin-like_repeats+TM+beta-rich+lc_tail->Tubulin+alpha_helical+??+??->GAP_unknown1+TM+TM+GAP_unknown2->HRDC-like?+Fibronectin-like_repeats->GAP1-N2+GAP1-M+GAP1-C*->?->??+DO-GTPase2.2->

■ **GCA_001998865.1; Tessaracoccus flavescens; counts= 1; multicellularity status: FALSE**

- polar_leader+TM+TM+TM->TM+vWA(canonical)+Fibronectin-like_repeats+TM+beta-rich+lc_tail->Tubulin+alpha_helical+??+??->GAP_unknown1+TM+TM+GAP_unknown2->?->HRDC-like?+Fibronectin-like_repeats->GAP1-N2+GAP1-M+GAP1-C*->?->??+DO-GTPase2.2->

■ **GCA_001997345.1; Tessaracoccus aquimaris; counts= 1; multicellularity status: TRUE**

- ??+TM+vWA(canonical)+Fibronectin-like_repeats+TM+beta-rich+lc_tail->Tubulin+alpha_helical+??+??->GAP_unknown1+TM+TM+GAP_unknown2->?->?->HRDC-like?+Fibronectin-like_repeats->GAP1-N2+GAP1-M+GAP1-C*->?->??+DO-GTPase2.2->

■ **GCA_001998325.1; Nocardiopsis sinuspersici; counts= 1; multicellularity status: TRUE**

- TPR_containing_alpha_helical+Fibronectin-like_repeats+extended_insert+Fibronectin-like_repeats->ZnR+ZnR+DO-GTPase2.2->GAP1-N2+GAP1-M+GAP1-C*->

■ **GCA_002553535.1; Mycolicibacterium boenickei; counts= 1; multicellularity status: FALSE**

- HRDC-like?+Fibronectin-like_repeats->GAP1-N2+GAP1-M+GAP1-C*->??+DO-GTPase2.2->

■ **GCA_002006205.1; Clostridium beijerinckii; counts= 1; multicellularity status: FALSE**

- Calcineurin+GAPEAD1->GAP2->DO-GTPase2.1->DO-GTPase1->GAP1-N1+GAP1-M+GAP1-C*->

■ **GCA_002006875.1; Micromonospora sp. Rc5; counts= 1; multicellularity status: TRUE**

- GrpE->HSP70->TPR_containing_alpha_helical+Fibronectin-like_repeats->ZnR+ZnR+DO-GTPase2.2->GAP1-N2+GAP1-M+GAP1-C*->TM+TM+TM+TM+ZnR+ZnR+DO-GTPase2.2->

■ **GCA_002007105.1; Hymenobacter sp. CRA2; counts= 1; multicellularity status: FALSE**

- GAP2->DO-GTPase2.1->DO-GTPase1->DO-GTPase1->GAP1-N1+GAP1-M+GAP1-C+PNP_UDP_1+EAD1*->

■ **GCA_002009175.1; Streptomyces niveus; counts= 1; multicellularity status: TRUE**

- GAP1-N2+GAP1-M+GAP1-C*->vWA(canonical)->PP2C->Pkinase+ZnR+beta-rich->||<-TPR_containing_alpha_helical+Fibronectin-like_repeats

■ **GCF_002009845.1; Leptospira alexanderi; counts= 1; multicellularity status: FALSE**

- GAP2->ZnR+DO-GTPase2.1->DO-GTPase1->GAP1-N1+GAP1-M+GAP1-C+EAD1*->PNP_UDP_1+EAD1->

■ **GCA_002021565.1; Paenibacillus selenitireducens; counts= 1; multicellularity status: TRUE**

- TM+vWA(canonical)+YtKA-like+TM+??->Tubulin+coiled_coil+??->Tubulin+coiled_coil+HTH?+??->TM+TM+TM+vWA(canonical)+TM+TM+TM+polar_tail->Fibronectin-like_repeats+??->ZnR+ZnR+DO-GTPase2.2+ZnR+ZnR->GAP1-N2+GAP1-M+GAP1-C+polar_tail+TM+beta-rich*->

■ **GCF_000373405.1; Streptomyces sp. ScaeMP-e10; counts= 1; multicellularity status: TRUE**

- TM+TM+TM+TM+ZnR+ZnR+DO-GTPase2.2+TM->vWA(canonical)->PP2C->Pkinase+ZnR+beta-rich->||<-?<-?<-?||GAP1-N2+GAP1-M+GAP1-C*->

■ **GCF_002019645.1; Bacillus alkalitelluris; counts= 1; multicellularity status: FALSE**

- GrpE->HSP70->HSP70+vWA->DnaJ+DnaJ+ZnR+ZnR+coiled_coil+Fibronectin-like_repeats->ZnR+ZnR+DO-GTPase2.2->GAP1-N2+GAP1-M+GAP1-C*->TM+TM+TM+TM+TM+TM+TM+TM+ZnR+ZnR+DO-GTPase2.2->

■ **GCF_000702365.1; Streptomyces sp. CNS654; counts= 1; multicellularity status: TRUE**

- GAP1-N2+GAP1-M+GAP1-C*->

■ **GCF_000717015.1; Streptomyces; counts= 1; multicellularity status: TRUE**

- GAP1-N2+GAP1-M+GAP1-C*->

■ **GCF_000717105.1; Streptomyces anulatus; counts= 1; multicellularity status: TRUE**

- GAP1-N2+GAP1-M+GAP1-C*->

■ **GCF_000717665.1; Streptomyces floridae; counts= 1; multicellularity status: TRUE**

- GAP1-N2+GAP1-M+GAP1-C*->

■ **GCF_000716025.1; Streptomyces baarnensis; counts= 1; multicellularity status: TRUE**

- GAP1-N2+GAP1-M+GAP1-C*->

■ **GCF_000717215.1; Streptomyces vinaceus; counts= 1; multicellularity status: TRUE**

- GAP1-N2+GAP1-M+GAP1-C*->

■ **GCF_000377965.1; Streptomyces sp. CNB091; counts= 1; multicellularity status: TRUE**

- GAP1-N2+GAP1-M+GAP1-C*->

■ **GCF_000718915.1; Streptomyces; counts= 1; multicellularity status: TRUE**

- GAP1-N2+GAP1-M+GAP1-C*->

■ **GCF_000718455.1; Streptomyces globisporus; counts= 1; multicellularity status: TRUE**

- GAP1-N2+GAP1-M+GAP1-C*->

■ **GCF_000716945.1; Streptomyces albus; counts= 1; multicellularity status: TRUE**

- GAP1-N2+GAP1-M+GAP1-C*->

■ **GCF_002633395.1; Streptomyces; counts= 1; multicellularity status: TRUE**

- GAP1-N2+GAP1-M+GAP1-C*->

■ **GCA_900167605.1; Caloramator quimbayensis; counts= 1; multicellularity status: TRUE**

- PNPase->?->GAP1-N2+GAP1-M+GAP1-C*->

■ **GCA_002835505.1; Enterovibrio nigricans; counts= 1; multicellularity status: FALSE**

- GAP1-N1+GAP1-M+GAP1-C*->

■ **GCA_900167465.1; Geobacter thiogenes; counts= 1; multicellularity status: FALSE**

- ??+PNP_UDP_1+EAD1->GAP2->ZnR+DO-GTPase2.1->DO-GTPase1->GAP1-N1+GAP1-M+GAP1-C+EAD1*->

■ **GCF_000720915.1; Streptomyces albus; counts= 1; multicellularity status: TRUE**

- GAP1-N2+GAP1-M+GAP1-C*->

■ **GCF_000719925.1; Streptomyces sp. NRRL F-2202; counts= 1; multicellularity status: TRUE**

- GAP1-N2+GAP1-M+GAP1-C*->

■ **GCF_000718695.1; Streptomyces griseus group; counts= 1; multicellularity status: TRUE**

- GAP1-N2+GAP1-M+GAP1-C*->

- **GCF_000721415.1; Streptomyces sp. NRRL F-5702; counts= 1; multicellularity status: TRUE**
  - GAP1-N2+GAP1-M+GAP1-C*->
- **GCF_000719585.1; Streptomyces sp. NRRL F-5681; counts= 1; multicellularity status: TRUE**
  - GAP1-N2+GAP1-M+GAP1-C*->
- **GCF_000720065.1; Streptomyces; counts= 1; multicellularity status: TRUE**
  - GAP1-N2+GAP1-M+GAP1-C*->
- **GCF_000719655.1; Streptomyces sp. NRRL B-1381; counts= 1; multicellularity status: TRUE**
  - GAP1-N2+GAP1-M+GAP1-C*->
- **GCF_000725705.1; Streptomyces sp. NRRL S-623; counts= 1; multicellularity status: TRUE**
  - GAP1-N2+GAP1-M+GAP1-C*->
- **GCF_001279425.1; Streptomyces sp. NRRL F-2295; counts= 1; multicellularity status: TRUE**
  - GAP1-N2+GAP1-M+GAP1-C*->
- **GCF_000718235.1; Streptomyces griseus; counts= 1; multicellularity status: TRUE**
  - GAP1-N2+GAP1-M+GAP1-C*->
- **GCA_003147545.1; Streptomyces griseus group; counts= 1; multicellularity status: TRUE**
  - GAP1-N2+GAP1-M+GAP1-C*->
- **GCF_001279735.1; Streptomyces sp. MMG1522; counts= 1; multicellularity status: TRUE**
  - GAP1-N2+GAP1-M+GAP1-C*->
- **GCF_001418625.1; Streptomyces luridiscabiei; counts= 1; multicellularity status: TRUE**
  - GAP1-N2+GAP1-M+GAP1-C*->
- **GCF_001746305.1; Streptomyces sp. EN23; counts= 1; multicellularity status: TRUE**
  - GAP1-N2+GAP1-M+GAP1-C*->
- **GCA_001687325.1; Streptomyces sp. PTY087I2; counts= 1; multicellularity status: TRUE**
  - GAP1-N2+GAP1-M+GAP1-C*->
- **GCF_001746315.1; Streptomyces sp. EN27; counts= 1; multicellularity status: TRUE**
  - GAP1-N2+GAP1-M+GAP1-C*->
- **GCF_001905595.1; Streptomyces sp. CB00316; counts= 1; multicellularity status: TRUE**
  - TM+TM+TM+TM+ZnR+ZnR+DO-GTPase2.2+TM->vWA(canonical)->PP2C->Pkinase+ZnR+beta-rich->||<-?<-?<-?||GAP1-N2+GAP1-M+GAP1-C*->
- **GCF_001931635.1; Streptomyces sp. Tue 6075; counts= 1; multicellularity status: TRUE**

  - GAP1-N2+GAP1-M+GAP1-C*->

- **GCF_001905525.1; Streptomyces sp. TSRI0395; counts= 1; multicellularity status: TRUE**
  - GAP1-N2+GAP1-M+GAP1-C*->
- **GCF_001905905.1; Streptomyces sp. CB02366; counts= 1; multicellularity status: TRUE**
  - GAP1-N2+GAP1-M+GAP1-C*->
- **GCA_003367645.1; Streptomyces sp. IB2014 011-1; counts= 1; multicellularity status: TRUE**
  - TM+TM+TM+TM+ZnR+ZnR+DO-GTPase2.2+TM->vWA(canonical)->PP2C->Pkinase+ZnR+beta-rich->||<-?<-?<-?||GAP1-N2+GAP1-M+GAP1-C*->
- **GCA_001905485.1; Streptomyces sp. TSRI0261; counts= 1; multicellularity status: TRUE**
  - GAP1-N2+GAP1-M+GAP1-C*->
- **GCA_002027255.1; Paenibacillus sp. VT-16-81; counts= 1; multicellularity status: TRUE**
  - TM+vWA(canonical)+YtKA-like+TM+??->Tubulin+coiled_coil+??->PSE->TM+TM+TM+vWA(canonical)+TM+TM+TM+polar_tail->Fibronectin-like_repeats+??->ZnR+ZnR+DO-GTPase2.2+ZnR+ZnR->GAP1-N2+GAP1-M+GAP1-C+polar_tail+TM+beta-rich*->
- **GCA_002326205.1; Pseudomonas; counts= 1; multicellularity status: FALSE**
  - GAP2->DO-GTPase2.1->DO-GTPase1->GAP1-N1+GAP1-M+GAP1-C+EAD1*->CASPASE+EAD1->
- **GCA_003814735.1; Streptomyces katrae; counts= 1; multicellularity status: TRUE**
  - GAP1-N2+GAP1-M+GAP1-C*->
- **GCA_900168305.1; Paenibacillus; counts= 1; multicellularity status: TRUE**
  - TM+vWA(canonical)+YtKA-like+TM+??->Tubulin+coiled_coil+??->Tubulin+coiled_coil+HTH?+??->TM+TM+TM+vWA(canonical)+TM+TM+TM+polar_tail->Fibronectin-like_repeats+??->ZnR+ZnR+DO-GTPase2.2+ZnR+ZnR->GAP1-N2+GAP1-M+GAP1-C+polar_tail+TM+beta-rich*->
- **GCF_002042965.1; Paenibacillus sp. 32352; counts= 1; multicellularity status: TRUE**
  - TM+vWA(canonical)+YtKA-like+TM+??->Tubulin+coiled_coil+??->Tubulin+coiled_coil+HTH?+??->TM+TM+TM+vWA(canonical)+TM+TM+TM+polar_tail->Fibronectin-like_repeats+??->ZnR+ZnR+DO-GTPase2.2+ZnR+ZnR->GAP1-N2+GAP1-M+GAP1-C+polar_tail+TM+Cadherin-like*->
- **GCA_004271805.1; Salmonella enterica; counts= 1; multicellularity status: FALSE**
  - Calcineurin+GAPEAD1->GAP2->ZnR+DO-GTPase2.1->DO-GTPase1->GAP1-N1+GAP1-M+GAP1-C*->
- **GCA_003606245.1; Burkholderia pseudomallei; counts= 1; multicellularity status: FALSE**
  - Calcineurin+GAPEAD1->GAP2->ZnR+DO-GTPase2.1->DO-GTPase1->GAP1-N1+GAP1-M+GAP1-C*->
- **GCA_002071875.1; Burkholderia cenocepacia; counts= 1; multicellularity status: FALSE**
  - Calcineurin+GAPEAD1->GAP2->ZnR+DO-GTPase2.1->DO-GTPase1->GAP1-N1+GAP1-M+GAP1-C*->
- **GCA_002071905.1; Burkholderia cenocepacia; counts= 1; multicellularity status: FALSE**
  - GAP2->DO-GTPase2.1->DO-GTPase1->GAP1-N1+GAP1-M+GAP1-C+EAD1*->CASPASE+EAD1->
- **GCA_001293665.1; Pseudomonas syringae; counts= 1; multicellularity status: FALSE**
  - Calcineurin+GAPEAD1->GAP2->DO-GTPase2.1->DO-GTPase1->GAP1-N1+GAP1-M+GAP1-C*->
- **GCF_001526225.1; Burkholderia ubonensis; counts= 1; multicellularity status: FALSE**

- Calcineurin+GAPEAD1->GAP2->ZnR+DO-GTPase2.1->DO-GTPase1->GAP1-N1+GAP1-M+GAP1-C*->

■ **GCF_000221965.1; Xanthomonas campestris; counts= 1; multicellularity status: FALSE**

- GAP2->DO-GTPase2.1->DO-GTPase1->GAP1-N1+GAP1-M+GAP1-C+EAD1*->CASPASE+EAD1->

■ **GCF_900169525.1; Corynebacterium sp. Marseille-P2417; counts= 1; multicellularity status: FALSE**

- TM+TM+TM->TM+vWA(canonical)+Fibronectin-like_repeats+TM+beta-rich+lc_tail->Tubulin+alpha_helical+??+??->GAP_unknown1+TM+TM+GAP_unknown2->HRDC-like?+Fibronectin-like_repeats->GAP1-N2+GAP1-M+GAP1-C*->beta-rich+vWA(canonical)->??+DO-GTPase2.2->polar_leader+TM+TM+TM->

■ **GCA_900170035.1; Desulfamplus magnetovallimortis; counts= 1; multicellularity status: FALSE**

- GAP1-N2+GAP1-M+GAP1-C*->??->

■ **GCF_000834545.1; Pseudomonas fluorescens; counts= 1; multicellularity status: FALSE**

- GAP2->DO-GTPase2.1->DO-GTPase1->GAP1-N1+GAP1-M+GAP1-C+EAD1*->CASPASE+EAD1->

■ **GCF_001518935.2; Corynebacterium glutamicum; counts= 1; multicellularity status: FALSE**

- polar_leader+TM+TM+TM->TM+vWA(canonical)+Fibronectin-like_repeats+TM+beta-rich+lc_tail->Tubulin+alpha_helical+??+??->GAP_unknown1+TM+TM+GAP_unknown2->HRDC-like?+Fibronectin-like_repeats->GAP1-N2+GAP1-M+GAP1-C*->beta-rich+vWA(canonical)->??+DO-GTPase2.2->

■ **GCF_001718955.1; Burkholderia stagnalis; counts= 1; multicellularity status: FALSE**

- GAP2->||<-?||GAP1-N1+GAP1-M+GAP1-C+CASPASE*->

■ **GCA_003001535.1; Pseudomonas; counts= 1; multicellularity status: FALSE**

- Calcineurin+GAPEAD1->GAP2->ZnR+DO-GTPase2.1->DO-GTPase1->GAP1-N1+GAP1-M+GAP1-C*->

■ **GCA_002079265.1; Rhodococcus sp. BH4; counts= 1; multicellularity status: TRUE**

- polar_leader+TM+TM+TM->TM+vWA(canonical)+Fibronectin-like_repeats+TM+beta-rich+lc_tail->Tubulin+alpha_helical+??+??->GAP_unknown1+TM+TM+GAP_unknown2->HRDC-like?+Fibronectin-like_repeats->GAP1-N2+GAP1-M+GAP1-C*->beta-rich+vWA(canonical)->??+DO-GTPase2.2->

■ **GCF_002003625.1; Mycobacterium kansasii; counts= 1; multicellularity status: FALSE**

- GAP_unknown1+TM+TM+GAP_unknown2->HRDC-like?+Fibronectin-like_repeats->GAP1-N2+GAP1-M+GAP1-C->GAP1-M*->GAP1-N2+GAP1-M+GAP1-C->beta-rich+vWA(canonical)->??+DO-GTPase2.2->

■ **GCF_001643015.1; Corynebacterium crudilactis; counts= 1; multicellularity status: FALSE**

- TM+TM+TM->PSE->Tubulin+alpha_helical+??+??->GAP_unknown1+TM+TM+GAP_unknown2->HRDC-like?+Fibronectin-like_repeats->GAP1-N2+GAP1-M+GAP1-C*->beta-rich+vWA(canonical)->

■ **GCA_002086255.1; Mycolicibacterium insubricum; counts= 1; multicellularity status: FALSE**

- TM+DUF4190+TM->TM+DUF4190+TM->polar_leader+TM+TM+TM->PSE->Tubulin+alpha_helical+??+??->||<-PSE||PSE->GAP1-N2+GAP1-M+GAP1-C*->beta-rich+vWA(canonical)->??+DO-GTPase2.2->

■ **GCA_002086335.1; Mycobacterium mantenii; counts= 1; multicellularity status: FALSE**

- TM+DUF4190+TM->TM+DUF4190+TM->polar_leader+TM+TM+TM->TM+vWA(canonical)+Fibronectin-like_repeats+TM+beta-rich+lc_tail->Tubulin+alpha_helical+??+??->GAP_unknown1+TM+TM+GAP_unknown2->HRDC-like?+Fibronectin-like_repeats->GAP1-N2+GAP1-M+GAP1-C*->beta-rich+vWA(canonical)->??+DO-GTPase2.2->TM+DUF4190+TM+PknH_C->TM+DUF4190+TM+PknH_C->TM+DUF4190+TM+PknH_C->TM+DUF4190+TM+PknH_C->

■ **GCA_002086675.1; Mycobacterium persicum; counts= 1; multicellularity status: FALSE**

- GAP1-N2+GAP1-M+GAP1-C*->beta-rich+vWA(canonical)->??+DO-GTPase2.2->

■ **GCA_002086485.1; Mycobacterium aquaticum; counts= 1; multicellularity status: FALSE**

- TM+DUF4190+TM->polar_leader+TM+TM+TM->TM+vWA(canonical)+Fibronectin-like_repeats+TM+beta-rich+lc_tail->Tubulin+alpha_helical+??+??->GAP_unknown1+TM+TM+GAP_unknown2->HRDC-like?+Fibronectin-like_repeats->GAP1-N2+GAP1-M+GAP1-C*->beta-rich+vWA(canonical)->??+DO-GTPase2.2->TM+DUF4190+TM+PknH_C->TM+DUF4190+TM+PknH_C->TM+DUF4190+TM+PknH_C->

■ **GCA_002082175.1; Streptomyces violaceoruber; counts= 1; multicellularity status: TRUE**

- GAP1-N2+GAP1-M+GAP1-C*->

■ **GCF_001854655.1; Frankia sp. Cc1.17; counts= 1; multicellularity status: TRUE**

- GAP1-N2+GAP1-M+GAP1-C+ZnR+DO-GTPase2*->

■ **GCA_900176515.1; Kibdelosporangium aridum; counts= 1; multicellularity status: TRUE**

- GrpE->HSP70->TPR_containing_alpha_helical+Fibronectin-like_repeats->ZnR+ZnR+DO-GTPase2.2->GAP1-N2+GAP1-M+GAP1-C*->TM+TM+TM+TM+ZnR+ZnR+DO-GTPase2.2->?->HSP70->vWA(canonical)->unknown+CASPASE->HSP70->

■ **GCF_001651875.1; Sinorhizobium saheli; counts= 1; multicellularity status: TRUE**

- GAP2->ZnR+DO-GTPase2.1->DO-GTPase1->GAP1-N1+GAP1-M+GAP1-C+EAD1*->CASPASE+EAD1->

■ **GCA_002082585.1; Kitasatospora albolonga; counts= 1; multicellularity status: TRUE**

- GAP1-N2+GAP1-M+GAP1-C*->

■ **GCA_003013675.1; Rhodococcus hoagii; counts= 1; multicellularity status: TRUE**

- polar_leader+TM+TM+TM->TM+vWA(canonical)+Fibronectin-like_repeats+TM+beta-rich+lc_tail->Tubulin+alpha_helical+??+??->GAP_unknown1+TM+TM+GAP_unknown2->HRDC-like?+Fibronectin-like_repeats->GAP1-N2+GAP1-M+GAP1-C*->beta-rich+vWA(canonical)->??+DO-GTPase2.2->

■ **GCA_002096055.1; Cnuibacter physcomitrellae; counts= 1; multicellularity status: FALSE**

- ??->PP2C->Pkinase+helical_region+TM_region->ZnR+ZnR+DO-GTPase2.2->GAP1-N2+GAP1-M+low_complexity+TM+TM*->

■ **GCA_900177515.1; Azospirillum oryzae; counts= 1; multicellularity status: FALSE**

- HSP70->HSP70+vWA(canonical)->?->DO-GTPase2.2(frag)->GAP1-N2+GAP1-M+GAP1-C*->?->TM+TM+TM+TM+ZnR+ZnR+DO-GTPase2.2->

■ **GCA_002101995.1; Mycolicibacterium fallax; counts= 1; multicellularity status: FALSE**

- TM+DUF4190+TM->TM+DUF4190+TM->polar_leader+TM+TM+TM->TM+vWA(canonical)+Fibronectin-like_repeats+TM+beta-rich+lc_tail->Tubulin+alpha_helical+??+??->GAP_unknown1+TM+TM+GAP_unknown2->HRDC-like?+Fibronectin-like_repeats->GAP1-N2+GAP1-M+GAP1-C*->beta-rich+vWA(canonical)->??+DO-GTPase2.2->

■ **GCA_001052995.1; Mycolicibacterium conceptionense; counts= 1; multicellularity status: FALSE**

- HRDC-like?+Fibronectin-like_repeats->GAP1-N2+GAP1-M+GAP1-C*->??+DO-GTPase2.2->

■ **GCA_002101965.1; Mycolicibacterium wolinskyi; counts= 1; multicellularity status: FALSE**

- HRDC-like?+Fibronectin-like_repeats->GAP1-N2+GAP1-M+GAP1-C*->??+DO-GTPase2.2->

■ **GCA_002102105.1; Mycolicibacterium confluentis; counts= 1; multicellularity status: FALSE**

- Tubulin+alpha_helical+??+??->GAP_unknown1+TM+TM+GAP_unknown2->HRDC-like?+Fibronectin-like_repeats->GAP1-N2+GAP1-M+GAP1-C*->beta-rich+vWA(canonical)->ZnR+ZnR+DO-GTPase2.2->

■ **GCA_002102115.1; Mycolicibacterium doricum; counts= 1; multicellularity status: FALSE**

- HRDC-like?+Fibronectin-like_repeats->GAP1-N2+GAP1-M+GAP1-C*->??+DO-GTPase2.2->

■ **GCA_900155475.1; Methylomagnum ishizawai; counts= 1; multicellularity status: FALSE**

- PNP_UDP_1+EAD1->||<-?||GAP2->ZnR+DO-GTPase2.1->DO-GTPase1->GAP1-N1+GAP1-M+GAP1-C+EAD1*->

■ **GCA_900177415.1; Rhizobium oryzae; counts= 1; multicellularity status: TRUE**

- Calcineurin+GAPEAD1->GAP2->DO-GTPase2.1->DO-GTPase1->GAP1-N1+GAP1-M+GAP1-C*->

■ **GCA_900177695.1; Rhodococcus rhodochrous; counts= 1; multicellularity status: TRUE**

- polar_leader+TM+TM+TM->TM+vWA(canonical)+Fibronectin-like_repeats+TM+beta-rich+lc_tail->Tubulin+alpha_helical+??+??->GAP_unknown1+TM+TM+GAP_unknown2->HRDC-like?+Fibronectin-like_repeats->GAP1-N2+GAP1-M+GAP1-C*->beta-rich+vWA(canonical)->??+DO-GTPase2.2->

■ **GCA_004196125.1; Streptomyces albidoflavus; counts= 1; multicellularity status: TRUE**

- GAP1-N2+GAP1-M+GAP1-C*->

■ **GCA_900177815.1; Paenibacillus aquistagni; counts= 1; multicellularity status: TRUE**

- TM+vWA(canonical)+YtKA-like+TM+??->Tubulin+coiled_coil+??->Tubulin+coiled_coil+HTH?+??->TM+TM+TM+vWA(canonical)+TM+TM+TM+polar_tail->Fibronectin-like_repeats+??->ZnR+ZnR+DO-GTPase2.2+ZnR+ZnR->GAP1-N2+GAP1-M+GAP1-C+polar_tail+TM+beta-rich*->

■ **GCA_900177745.1; Corynebacterium pollutisoli; counts= 1; multicellularity status: FALSE**

- TM+vWA(canonical)+Fibronectin-like_repeats+TM+beta-rich+lc_tail->Tubulin+alpha_helical+??+??->GAP_unknown1+TM+TM+GAP_unknown2->HRDC-like?+Fibronectin-like_repeats->GAP1-N2+GAP1-M+GAP1-C*->beta-rich+vWA(canonical)->??+DO-GTPase2.2->

■ **GCA_002119405.1; Pseudoruegeria sp. SK021; counts= 1; multicellularity status: FALSE**

- Calcineurin+GAPEAD1->GAP2->DO-GTPase2.1(fragment)->DO-GTPase1->GAP1-N1+GAP1-M+GAP1-C*->

■ **GCA_000172695.2; Rhizobium sp. Kim5; counts= 1; multicellularity status: TRUE**

- Calcineurin+GAPEAD1->GAP2->DO-GTPase2.1->DO-GTPase1->GAP1-N1+GAP1-M+GAP1-C*->

■ **GCA_002128305.1; Streptomyces sp. SCSIO 03032; counts= 1; multicellularity status: TRUE**

- GAP1-N2+GAP1-M+GAP1-C*->PSE->PP2C->Pkinase+ZnR+beta-rich->?->?->||<-TPR_containing_alpha_helical+Fibronectin-like_repeats

■ **GCA_002143185.1; Acinetobacter baumannii; counts= 1; multicellularity status: FALSE**

- PNP_UDP_1+EAD1->GAP2->ZnR+DO-GTPase2.1->DO-GTPase1->GAP1-N1+GAP1-M+GAP1-C+EAD1*->

■ **GCF_002150765.1; Lentzea kentuckyensis; counts= 1; multicellularity status: TRUE**

- vWA(canonical)+beta-rich+low_complexity->Tubulin+alpha_helical+??+??->??+TM+TM+TM+TM+??->Pkinase+TM->ZnR+ZnR+DO-GTPase2.2->GAP1-N2+GAP1-M+SLATT+SLATT*->TIR+??->?->?->STAND_NTPase+TPR_region->

■ **GCF_002154385.1; Streptomyces albovinaceus; counts= 1; multicellularity status: TRUE**

- GAP1-N2+GAP1-M+GAP1-C*->

■ **GCF_002154345.1; Streptomyces fimicarius; counts= 1; multicellularity status: TRUE**

- GAP1-N2+GAP1-M+GAP1-C*->

■ **GCA_006539165.1; Streptomyces cacaoi; counts= 1; multicellularity status: TRUE**

- GAP1-N2+GAP1-M+GAP1-C*->PSE->?->||<-TPR_containing_alpha_helical+Fibronectin-like_repeats

■ **GCF_002155975.1; Amycolatopsis kentuckyensis; counts= 1; multicellularity status: TRUE**

- vWA(canonical)+beta-rich+low_complexity->?->?->Tubulin+alpha_helical+??+??->??+TM+TM+TM+TM+??->Pkinase+TM->ZnR+ZnR+DO-GTPase2.2->GAP1-N2+GAP1-M+GAP1-C+TM+beta-rich*->

■ **GCF_002154605.1; Streptomyces milbemycinicus; counts= 1; multicellularity status: TRUE**

- GAP1-N2+GAP1-M+GAP1-C*->

■ **GCA_002156805.1; Corynebacterium striatum; counts= 1; multicellularity status: FALSE**

- polar_leader+TM+TM+TM->TM+vWA(canonical)+Fibronectin-like_repeats+TM+beta-rich+lc_tail->Tubulin+alpha_helical+??+??->GAP_unknown1+TM+TM+GAP_unknown2->HRDC-like?+Fibronectin-like_repeats->GAP1-N2+GAP1-M+GAP1-C*->beta-rich+vWA(canonical)->??+DO-GTPase2.2->polar_leader+TM+TM+TM->

■ **GCF_900163685.1; Corynebacterium glutamicum; counts= 1; multicellularity status: FALSE**

- polar_leader+TM+TM+TM->TM+vWA(canonical)+Fibronectin-like_repeats+TM+beta-rich+lc_tail->Tubulin+alpha_helical+??+??->GAP_unknown1+TM+TM+GAP_unknown2->HRDC-like?+Fibronectin-like_repeats->GAP1-N2+GAP1-M+GAP1-C*->beta-rich+vWA(canonical)->??+DO-GTPase2.2->

■ **GCA_002157835.1; Mycobacterium dioxanotrophicus; counts= 1; multicellularity status: FALSE**

- TM+DUF4190+TM->polar_leader+TM+TM+TM->PSE->Tubulin+alpha_helical+??+??->GAP_unknown1+TM+TM+GAP_unknown2->HRDC-like?+Fibronectin-like_repeats->GAP1-N2+GAP1-M+GAP1-C*->beta-rich+vWA(canonical)->??+DO-GTPase2.2->TM+DUF4190+TM+PknH_C->TM+DUF4190+TM+PknH_C->TM+DUF4190+TM+PknH_C->

■ **GCF_900143625.1; Nocardiopsis sp. JB363; counts= 1; multicellularity status: TRUE**

- TPR_containing_alpha_helical+Fibronectin-like_repeats->ZnR+ZnR+DO-GTPase2.2->GAP1-N2+GAP1-M+GAP1-C*->TM+TM+TM+TM+ZnR+ZnR+DO-GTPase2.2->

■ **GCF_900155535.1; Corynebacterium urinapleomorphum; counts= 1; multicellularity status: FALSE**

- Tubulin+alpha_helical+??+??->GAP_unknown1+TM+TM+GAP_unknown2->HRDC-like?+Fibronectin-like_repeats->GAP1-N2+GAP1-M+GAP1-C*->beta-rich+vWA(canonical)->??+DO-GTPase2.2->

■ **GCA_002160935.1; Drancourtella sp. An12; counts= 1; multicellularity status: FALSE**

- TM+vWA(canonical)+YtKA-like+IG-like+TM+??->Tubulin+coiled_coil+??->?->?->ZnR+insert+ZnR+DO-GTPase2.2+ZnR->GAP1-N2+GAP1-M+GAP1-C*->Pkinase->

■ **GCA_002160755.1; Lachnoclostridium sp. An14; counts= 1; multicellularity status: NA**

- Tubulin+coiled_coil+??->Tubulin+coiled_coil+??->?->??->ZnR+ZnR+DO-GTPase2.2->GAP1-N2+GAP1-M+TM+GAP1-C+TM+TM+TM*->TM+TM+TM+vWA(canonical)+TM+TM+TM+polar_tail->

■ **GCA_002159835.1; Blautia sp. An249; counts= 1; multicellularity status: FALSE**

- Tubulin+coiled_coil+??->Tubulin+coiled_coil+??->?->?->ZnR+insert+ZnR+DO-GTPase2.2+??->GAP1-N2+GAP1-M+GAP1-C*->?->?->Pkinase->

■ **GCA_002160435.1; Pseudoflavonifractor sp. An176; counts= 1; multicellularity status: FALSE**

- GAP1-N2+GAP1-M+GAP1-C*->

■ **GCA_002161835.1; Brevibacillus brevis; counts= 1; multicellularity status: FALSE**

- TM+vWA(canonical)+YtKA-like+TM+??->Tubulin+coiled_coil+??->Tubulin+coiled_coil+HTH?+??->TM+TM+TM+vWA(canonical)+TM+TM+TM+polar_tail->Fibronectin-like_repeats+??->ZnR+ZnR+DO-GTPase2.2+ZnR+ZnR->GAP1-N2+GAP1-M+GAP1-C+polar_tail+TM+beta-rich*->

■ **GCF_002161865.1; Paenibacillus apiarius; counts= 1; multicellularity status: TRUE**

- TM+vWA(canonical)+YtKA-like+TM+??->Tubulin+coiled_coil+??->Tubulin+coiled_coil+HTH?+??->TM+TM+TM+vWA(canonical)+TM+TM+TM+polar_tail->Fibronectin-like_repeats+??->ZnR+ZnR+DO-GTPase2.2+ZnR+ZnR->GAP1-N2+GAP1-M+GAP1-C+polar_tail+TM+beta-rich*->

■ **GCF_004001005.1; Paenibacillus thiaminolyticus; counts= 1; multicellularity status: TRUE**

- TM+vWA(canonical)+YtKA-like+TM+??->Tubulin+coiled_coil+??->Tubulin+coiled_coil+HTH?+??->TM+TM+TM+vWA(canonical)+TM+TM+TM+polar_tail->Fibronectin-like_repeats+??->ZnR+ZnR+DO-GTPase2.2+ZnR+ZnR->GAP1-N2+GAP1-M+GAP1-C+polar_tail+TM+beta-rich*->

■ **GCA_002165735.1; Rhodococcus sp. NCIMB 12038; counts= 1; multicellularity status: TRUE**

- GAP1-N2+GAP1-M+GAP1-C*->beta-rich+vWA(canonical)->??+DO-GTPase2.2->

■ **GCA_002188345.1; Streptomyces sp. CS057; counts= 1; multicellularity status: TRUE**

- GAP1-N2+GAP1-M+GAP1-C*->

■ **GCA_002188355.1; Streptomyces sp. CS227; counts= 1; multicellularity status: TRUE**

- GAP1-N2+GAP1-M+GAP1-C*->

■ **GCA_002189105.1; Saccharibacillus sp. O16; counts= 1; multicellularity status: TRUE**

- TM+vWA(canonical)+YtKA-like+TM+??->Tubulin+coiled_coil+??->?->TM+TM+TM+vWA(canonical)+TM+TM+TM+polar_tail->?->Fibronectin-like_repeats+??->ZnR+ZnR+DO-GTPase2.2+ZnR+ZnR->GAP1-N2+GAP1-M+GAP1-C+polar_tail+TM+beta-rich*->

■ **GCA_002192415.1; Paenibacillus donghaensis; counts= 1; multicellularity status: TRUE**

- TM+vWA(canonical)+YtKA-like+TM+??->Tubulin+coiled_coil+??->Tubulin+coiled_coil+HTH?+??->TM+TM+TM+vWA(canonical)+TM+TM+TM+polar_tail->Fibronectin-like_repeats+??->ZnR+ZnR+DO-GTPase2.2+ZnR+ZnR->GAP1-N2+GAP1-M+GAP1-C+polar_tail+TM+beta-rich*->

■ **GCA_900177675.1; Bacillus sp. OV166; counts= 1; multicellularity status: NA**

- TM+vWA(canonical)+YtKA-like+TM+??->Tubulin+coiled_coil+??->Tubulin+coiled_coil+HTH?+??->TM+TM+TM+vWA(canonical)+TM+TM+TM+polar_tail->Fibronectin-like_repeats+??->ZnR+ZnR+DO-GTPase2.2+ZnR+ZnR->GAP1-N2+GAP1-M+GAP1-C+polar_tail+TM+beta-rich*->

■ **GCF_900143175.1; Xanthomonas retroflexus; counts= 1; multicellularity status: FALSE**

- GAP2->DO-GTPase2.1->DO-GTPase1->GAP1-N1+GAP1-M+GAP1-C+EAD1*->CASPASE+EAD1->

■ **GCA_002198105.1; Mycolicibacterium peregrinum; counts= 1; multicellularity status: FALSE**

- polar_leader+TM+TM+TM->TM+vWA(canonical)+Fibronectin-like_repeats+TM+beta-rich+lc_tail->Tubulin+alpha_helical+??+??->GAP_unknown1+TM+TM+GAP_unknown2->HRDC-like?+Fibronectin-like_repeats->GAP1-N2+GAP1-M+GAP1-C*->beta-rich+vWA(canonical)->??+DO-GTPase2.2->TM+DUF4190+TM+PknH_C->TM+DUF4190+TM+PknH_C->

■ **GCA_002205895.1; Saccharibacillus sp. O23; counts= 1; multicellularity status: TRUE**

- TM+vWA(canonical)+YtKA-like+TM+??->Tubulin+coiled_coil+??->Tubulin+coiled_coil+HTH?+??->TM+TM+TM+vWA(canonical)+TM+TM+TM+polar_tail->?->Fibronectin-like_repeats+??->ZnR+ZnR+DO-GTPase2.2+ZnR+ZnR->GAP1-N2+GAP1-M+GAP1-C+polar_tail+TM+beta-rich*->

■ **GCF_900188525.1; Paenibacillus sp. St-s; counts= 1; multicellularity status: TRUE**

- TM+vWA(canonical)+YtKA-like+TM+??->Tubulin+coiled_coil+??->Tubulin+coiled_coil+HTH?+??->TM+TM+TM+vWA(canonical)+TM+TM+TM+polar_tail->Fibronectin-like_repeats+??->ZnR+ZnR+DO-GTPase2.2+ZnR+ZnR->GAP1-N2+GAP1-M+GAP1-C+polar_tail+TM+beta-rich*->

■ **GCA_900187925.1; Streptomyces sp. PgraA7; counts= 1; multicellularity status: TRUE**

- GAP1-N2+GAP1-M+GAP1-C*->

■ **GCF_900188505.1; Paenibacillus elgii; counts= 1; multicellularity status: TRUE**

- TM+vWA(canonical)+YtKA-like+TM+??->Tubulin+coiled_coil+??->Tubulin+coiled_coil+HTH?+??->?->TM+TM+TM+vWA(canonical)+TM+TM+TM+polar_tail->Fibronectin-like_repeats+??->ZnR+ZnR+DO-GTPase2.2+ZnR+ZnR->GAP1-N2+GAP1-M+GAP1-C+polar_tail+TM+Cadherin-like*->

■ **GCA_002215235.1; Rhodococcus sp. BUPNP1; counts= 1; multicellularity status: TRUE**

- polar_leader+TM+TM+TM->TM+vWA(canonical)+Fibronectin-like_repeats+TM+beta-rich+lc_tail->Tubulin+alpha_helical+??+??->GAP_unknown1+TM+TM+GAP_unknown2->HRDC-like?+Fibronectin-like_repeats->GAP1-N2+GAP1-M+GAP1-C*->beta-rich+vWA(canonical)->??+DO-GTPase2.2->

■ **GCA_002215075.1; Brevibacillus formosus; counts= 1; multicellularity status: FALSE**

- TM+vWA(canonical)+YtKA-like+TM+??->Tubulin+coiled_coil+??->Tubulin+coiled_coil+HTH?+??->TM+TM+TM+vWA(canonical)+TM+TM+TM+polar_tail->Fibronectin-like_repeats+??->ZnR+ZnR+DO-GTPase2.2+ZnR+ZnR->GAP1-N2+GAP1-M+GAP1-C+polar_tail+TM+beta-rich*->

■ **GCA_002091955.1; Rhodococcus sp. 1168; counts= 1; multicellularity status: TRUE**

- GrpE->HSP70->TPR_containing_alpha_helical+Fibronectin-like_repeats->ZnR+ZnR+DO-GTPase2.2->GAP1-N2+GAP1-M+GAP1-C*->TM+TM+TM+TM+ZnR+ZnR+DO-GTPase2.2->??->HSP70->

■ **GCA_900091445.1; Micromonospora echinofusca; counts= 1; multicellularity status: TRUE**

- TPR_containing_alpha_helical+Fibronectin-like_repeats->ZnR+ZnR+DO-GTPase2.2->GAP1-N2+GAP1-M+GAP1-C*->TM+TM+TM+TM+ZnR+ZnR+DO-GTPase2.2->

■ **GCF_002217715.1; Streptomyces sp. SS07; counts= 1; multicellularity status: TRUE**

- GAP1-N2+GAP1-M+GAP1-C*->

■ **GCA_900188005.1; Actinoplanes regularis; counts= 1; multicellularity status: TRUE**

- HSP70->TPR_containing_alpha_helical+Fibronectin-like_repeats->ZnR+ZnR+DO-GTPase2.2->GAP1-N2+GAP1-M+GAP1-C*->TM+TM+TM+TM+ZnR+ZnR+DO-GTPase2.2->TPR_region+CASPASE->?->||<-CASPASE+low_complexity

■ **GCF_900188375.1; Geodermatophilus pulveris; counts= 1; multicellularity status: TRUE**

- Pkinase+TM->ZnR+ZnR+DO-GTPase2.2->GAP1-N2+GAP1-M+GAP1-C*->Pkinase+low_complexity->

■ **GCA_900188105.1; Actinomadura mexicana; counts= 1; multicellularity status: TRUE**

- GrpE->HSP70->TPR_containing_alpha_helical+Fibronectin-like_repeats->ZnR+ZnR+DO-GTPase2.2->GAP1-N2+GAP1-M+GAP1-C*->TM+TM+TM+TM+ZnR+ZnR+DO-GTPase2.2+TM->

■ **GCA_900188445.1; Actinomadura meyerae; counts= 1; multicellularity status: TRUE**

- GrpE->HSP70->TPR_containing_alpha_helical+Fibronectin-like_repeats->ZnR+ZnR+DO-GTPase2.2->GAP1-N2+GAP1-M+GAP1-C*->TM+TM+TM+TM+ZnR+ZnR+DO-GTPase2.2+TM->

■ **GCA_002224835.1; Paenibacillus sp. SSG-1; counts= 1; multicellularity status: TRUE**

- TM+vWA(canonical)+YtKA-like+TM+??->Tubulin+coiled_coil+??->Tubulin+coiled_coil+HTH?+??->TM+TM+TM+vWA(canonical)+TM+TM+TM+polar_tail->Fibronectin-like_repeats+??->ZnR+ZnR+DO-GTPase2.2+ZnR+ZnR->GAP1-N2+GAP1-M+GAP1-C+polar_tail+TM+beta-rich*->

■ **GCA_900107795.1; Chitinophaga terrae Kim and Jung 2007; counts= 1; multicellularity status: TRUE**

- TIR+GAPEAD1->GAP2->ZnR+DO-GTPase2.1->DO-GTPase1->GAP1-N1+GAP1-M+GAP1-C*->

■ **GCA_900067065.1; Eubacteriaceae bacterium CHKCI005; counts= 1; multicellularity status: NA**

- TM+TM+TM+TM+ZnR+ZnR+DO-GTPase2.2->?->HSP70->HSP70+vWA(canonical)->DnaJ+DnaJ+ZnR+ZnR+coiled_coil+Fibronectin-like_repeats->ZnR+ZnR+DO-GTPase2.2->GAP1-N2+GAP1-M+GAP1-C*->

■ **GCA_900109175.1; Pseudomonas linyingensis; counts= 1; multicellularity status: FALSE**

- GAP2->DO-GTPase2.1->DO-GTPase1->GAP1-N1+GAP1-M+GAP1-C+EAD1*->CASPASE+EAD1->

■ **GCA_900110825.1; Mycobacterium; counts= 1; multicellularity status: FALSE**

- HRDC-like?+Fibronectin-like_repeats->GAP1-N2+GAP1-M+GAP1-C*->??+DO-GTPase2.2->

■ **GCA_001245615.1; Mycolicibacterium neworleansense; counts= 1; multicellularity status: FALSE**

- HRDC-like?+Fibronectin-like_repeats->GAP1-N2+GAP1-M+GAP1-C*->??+DO-GTPase2.2->

■ **GCA_900100345.1; Paenibacillus tianmuensis; counts= 1; multicellularity status: TRUE**

- TM+vWA(canonical)+YtKA-like+TM+??->Tubulin+coiled_coil+??->Tubulin+coiled_coil+HTH?+??->?->TM+TM+TM+vWA(canonical)+TM+TM+TM+polar_tail->Fibronectin-like_repeats+??->ZnR+ZnR+DO-GTPase2.2+ZnR+ZnR->GAP1-N2+GAP1-M+GAP1-C+polar_tail+TM+Cadherin-like*->

■ **GCA_900099765.1; Paenibacillus typhae; counts= 1; multicellularity status: TRUE**

- ZnR+ZnR+DO-GTPase2.2+ZnR+ZnR->GAP1-N2+GAP1-M+GAP1-C+polar_tail+TM+beta-rich*->

■ **GCA_900103525.1; Bacillus sp. OK048; counts= 1; multicellularity status: NA**

- TM+vWA(canonical)+YtKA-like+TM+??->Tubulin+coiled_coil+??->Tubulin+coiled_coil+HTH?+??->TM+TM+TM+vWA(canonical)+TM+TM+TM+polar_tail->Fibronectin-like_repeats+??->ZnR+ZnR+DO-GTPase2.2+ZnR+ZnR->GAP1-N2+GAP1-M+GAP1-C+polar_tail+TM+beta-rich*->

■ **GCA_900107875.1; Paenibacillus sp. 276b; counts= 1; multicellularity status: TRUE**

- TM+TM+TM+vWA+TM+TM+TM+polar_tail->Fibronectin-like_repeats+??->ZnR+ZnR+DO-GTPase2.2->GAP1-N2+GAP1-M+GAP1-C+polar_tail*->

■ **GCA_900110315.1; Paenibacillus sophorae; counts= 1; multicellularity status: TRUE**

- TM+vWA(canonical)+YtKA-like+TM+??->Tubulin+coiled_coil+??->Tubulin+coiled_coil+HTH?+??->TM+TM+TM+vWA(canonical)+TM+TM+TM+polar_tail->Fibronectin-like_repeats+??->ZnR+ZnR+DO-GTPase2.2+ZnR+ZnR->GAP1-N2+GAP1-M+GAP1-C+polar_tail+TM+beta-rich*->

■ **GCA_900109125.1; Paenibacillus sp. 181mfcol5.1; counts= 1; multicellularity status: TRUE**

- Tubulin+coiled_coil+??->Tubulin+coiled_coil+HTH?+??->TM+TM+TM+vWA+TM+TM+TM+polar_tail->Fibronectin-like_repeats+??->ZnR+ZnR+DO-GTPase2.2->GAP1-N2+GAP1-M+GAP1-C+polar_tail*->

■ **GCF_900110055.1; Paenibacillus sp. OK076; counts= 1; multicellularity status: TRUE**

- TM+TM+TM+vWA+TM+TM+TM+polar_tail->Fibronectin-like_repeats+??->ZnR+ZnR+DO-GTPase2.2->GAP1-N2+GAP1-M+GAP1-C+polar_tail*->

■ **GCA_900109305.1; Paenibacillus sp. cl141a; counts= 1; multicellularity status: TRUE**

- TM+vWA(canonical)+YtKA-like+TM+??->Tubulin+coiled_coil+??->Tubulin+coiled_coil+HTH?+??->TM+TM+TM+vWA(canonical)+TM+TM+TM+polar_tail->Fibronectin-like_repeats+??->ZnR+ZnR+DO-GTPase2.2+ZnR+ZnR->GAP1-N2+GAP1-M+GAP1-C+polar_tail+TM+beta-rich*->

■ **GCA_900102085.1; Paenibacillus polysaccharolyticus; counts= 1; multicellularity status: TRUE**

- TM+vWA(canonical)+YtKA-like+TM+??->Tubulin+coiled_coil+??->Tubulin+coiled_coil+HTH?+??->TM+TM+TM+vWA(canonical)+TM+TM+TM+polar_tail->Fibronectin-like_repeats+??->ZnR+ZnR+DO-GTPase2.2+ZnR+ZnR->GAP1-N2+GAP1-M+GAP1-C+polar_tail+TM+beta-rich*->

■ **GCA_900114615.1; Pelosinus propionicus; counts= 1; multicellularity status: FALSE**

- Calcineurin+GAPEAD1->GAP2->DO-GTPase2.1->DO-GTPase1->GAP1-N1+GAP1-M+GAP1-C*->

■ **GCA_900101205.1; Paenibacillus sp. OK060; counts= 1; multicellularity status: TRUE**

- TM+vWA(canonical)+YtKA-like+TM+??->Tubulin+coiled_coil+??->Tubulin+coiled_coil+HTH?+??->TM+TM+TM+vWA(canonical)+TM+TM+TM+polar_tail->Fibronectin-like_repeats+??->ZnR+ZnR+DO-GTPase2.2+ZnR+ZnR->GAP1-N2+GAP1-M+GAP1-C+polar_tail+TM+beta-rich*->

■ **GCA_900113065.1; Aureimonas phyllosphaerae; counts= 1; multicellularity status: FALSE**

- GAP1-N1+GAP1-M+GAP1-C+EAD1*->CASPASE+EAD1->

■ **GCF_900109515.1; Paenibacillus sp. OK003; counts= 1; multicellularity status: TRUE**

- TM+TM+TM+vWA+TM+TM+TM+polar_tail->Fibronectin-like_repeats+??->ZnR+ZnR+DO-GTPase2.2->GAP1-N2+GAP1-M+GAP1-C+polar_tail*->

■ **GCF_900095775.1; Paenibacillus amylolyticus; counts= 1; multicellularity status: TRUE**

- Tubulin+coiled_coil+??->Tubulin+coiled_coil+HTH?+??->TM+TM+TM+vWA+TM+TM+TM+polar_tail->Fibronectin-like_repeats+??->ZnR+ZnR+DO-GTPase2.2->GAP1-N2+GAP1-M+GAP1-C+polar_tail*->

■ **GCF_900101225.1; Paenibacillus sp. CF095; counts= 1; multicellularity status: TRUE**

- Tubulin+coiled_coil+??->Tubulin+coiled_coil+HTH?+??->TM+TM+TM+vWA+TM+TM+TM+polar_tail->Fibronectin-like_repeats+??->ZnR+ZnR+DO-GTPase2.2->GAP1-N2+GAP1-M+GAP1-C+polar_tail*->

■ **GCA_900114475.1; Paenibacillus sp. 1_12; counts= 1; multicellularity status: TRUE**

- TM+vWA(canonical)+YtKA-like+TM+??->Tubulin+coiled_coil+??->Tubulin+coiled_coil+HTH?+??->TM+TM+TM+vWA(canonical)+TM+TM+TM+polar_tail->Fibronectin-like_repeats+??->ZnR+ZnR+DO-GTPase2.2+ZnR+ZnR->GAP1-N2+GAP1-M+GAP1-C+polar_tail+TM+Cadherin-like*->

■ **GCA_900102215.1; Fontibacillus panacisegetis; counts= 1; multicellularity status: FALSE**

- TM+vWA(canonical)+YtKA-like+TM+??->Tubulin+coiled_coil+??->Tubulin+coiled_coil+HTH?+??->TM+TM+TM+vWA(canonical)+TM+TM+TM+polar_tail->Fibronectin-like_repeats+??->ZnR+ZnR+DO-GTPase2.2+ZnR+ZnR->GAP1-N2+GAP1-M+GAP1-C+polar_tail+TM+beta-rich*->

■ **GCA_001536285.1; Frankia sp. G2; counts= 1; multicellularity status: TRUE**

- GAP1-N2+GAP1-M+GAP1-C+ZnR+DO-GTPase2*->

■ **GCA_900091595.1; Micromonospora haikouensis; counts= 1; multicellularity status: TRUE**

- GrpE->HSP70->TPR_containing_alpha_helical+Fibronectin-like_repeats->ZnR+ZnR+DO-GTPase2.2->GAP1-N2+GAP1-M+GAP1-C*->TM+TM+TM+TM+ZnR+ZnR+DO-GTPase2.2->

■ **GCF_900114385.1; Geodermatophilus ruber; counts= 1; multicellularity status: TRUE**

- Pkinase+TM->ZnR+ZnR+DO-GTPase2.2->GAP1-N2+GAP1-M+GAP1-C*->

■ **GCF_900104025.1; Geodermatophilus sp. DSM 45219; counts= 1; multicellularity status: TRUE**

- Pkinase+TM->ZnR+ZnR+DO-GTPase2.2->GAP1-N2+GAP1-M+GAP1-C*->||<-?<-?||?->Pkinase+low_complexity->

■ **GCA_900101685.1; Alloactinosynnema iranicum; counts= 1; multicellularity status: TRUE**

- GrpE->HSP70->TPR_containing_alpha_helical+Fibronectin-like_repeats->ZnR+ZnR+DO-GTPase2.2->GAP1-N2+GAP1-M+GAP1-C*->TM+TM+TM+TM+ZnR+ZnR+DO-GTPase2.2->?->||<-?<-?<-HSP70

■ **GCF_900111115.1; Giesbergeria anulus; counts= 1; multicellularity status: FALSE**

- GAP2->DO-GTPase2.1->DO-GTPase1->GAP1-N1+GAP1-M+GAP1-C+EAD1*->CASPASE+EAD1->

■ **GCA_900105385.1; Microlunatus soli; counts= 1; multicellularity status: FALSE**

- polar_leader+TM+TM+TM->TM+vWA(canonical)+Fibronectin-like_repeats+TM+beta-rich+lc_tail->Tubulin+alpha_helical+??+??->GAP_unknown1+TM+TM+GAP_unknown2->HRDC-like?+Fibronectin-like_repeats->GAP1-N2+GAP1-M+GAP1-C*->beta-rich+vWA(canonical)->??+DO-GTPase2.2->HSP70_domain_1->

■ **GCA_900116615.1; Algoriphagus locisalis; counts= 1; multicellularity status: FALSE**

- GAP2->ZnR+DO-GTPase2.1->DO-GTPase1->GAP1-N1+GAP1-M+GAP1-C+EAD1*->EAD1+Trypsin->?->??+EAD1->

■ **GCA_900103445.1; Methylobacterium phyllostachyos; counts= 1; multicellularity status: TRUE**

- Calcineurin+GAPEAD1->GAP2->ZnR+DO-GTPase2.1->DO-GTPase1->GAP1-N1+GAP1-M+GAP1-C*->

■ **GCA_900113225.1; Mitsuaria sp. PDC51; counts= 1; multicellularity status: FALSE**

- Calcineurin+GAPEAD1->GAP2->ZnR+DO-GTPase2.1->DO-GTPase1->GAP1-N1+GAP1-M+GAP1-C*->

■ **GCA_900113665.1; Planctomicrobium piriforme; counts= 1; multicellularity status: TRUE**

- ZnR+ZnR+DO-GTPase2.2->GAP1-N2+GAP1-M+TM+GAP1-C*->??+TM+??+beta-rich??->?->GYF+GYF+ZnR+TM+TM+TM->

■ **GCF_900101405.1; Bradyrhizobium sp. R5; counts= 1; multicellularity status: TRUE**

- Calcineurin+GAPEAD1->GAP2->ZnR+DO-GTPase2.1->DO-GTPase1->GAP1-N1+GAP1-M+GAP1-C*->

■ **GCA_900114075.1; Brevibacillus sp. OK042; counts= 1; multicellularity status: FALSE**

- TM+vWA(canonical)+YtKA-like+TM+??->Tubulin+coiled_coil+??->Tubulin+coiled_coil+HTH?+??->TM+TM+TM+vWA(canonical)+TM+TM+TM+polar_tail->Fibronectin-like_repeats+??->ZnR+ZnR+DO-GTPase2.2+ZnR+ZnR->GAP1-N2+GAP1-M+GAP1-C+polar_tail+TM+beta-rich*->

■ **GCA_900111995.1; Collimonas sp. OK607; counts= 1; multicellularity status: FALSE**

- GAP2->ZnR+DO-GTPase2.1->DO-GTPase1->GAP1-N1+GAP1-M+GAP1-C+EAD1*->CASPASE+EAD1->

■ **GCA_900108985.1; Achromobacter sp. NFACC18-2; counts= 1; multicellularity status: FALSE**

- Calcineurin+GAPEAD1->GAP2->ZnR+DO-GTPase2.1->DO-GTPase1->GAP1-N1+GAP1-M+GAP1-C*->

■ **GCA_900092135.1; Raineyella antarctica; counts= 1; multicellularity status: TRUE**

- ZnR+ZnR+DO-GTPase2.2->GAP1-N2+GAP1-M+low_complexity*->

■ **GCA_900114325.1; Rhodanobacter glycinis; counts= 1; multicellularity status: FALSE**

- Calcineurin+GAPEAD1->GAP2->DO-GTPase2.1->DO-GTPase1->GAP1-N1+GAP1-M+GAP1-C*->

■ **GCF_900111175.1; Actinokineospora terrae; counts= 1; multicellularity status: TRUE**

- CASPASE+TPR_region->?->GAP1-N2+GAP1-M*->

■ **GCA_900099715.1; Ruminococcaceae bacterium YRB3002; counts= 1; multicellularity status: NA**

- HSP70->HSP70+??->DnaJ+DnaJ+ZnR+ZnR+coiled_coil+Fibronectin-like_repeats->ZnR+ZnR+DO-GTPase2.2->GAP1-N2+GAP1-M+GAP1-C*->TM+TM+TM+TM+ZnR+ZnR+DO-GTPase2.2->

■ **GCF_900101545.1; Variovorax sp. CF079; counts= 1; multicellularity status: FALSE**

- Calcineurin+GAPEAD1->GAP2->ZnR+DO-GTPase2.1->DO-GTPase1->GAP1-N1+GAP1-M+GAP1-C*->

■ **GCA_003635025.1; Saccharopolyspora antimicrobica; counts= 1; multicellularity status: TRUE**

- GrpE->HSP70->TPR_containing_alpha_helical+Fibronectin-like_repeats->ZnR+ZnR+DO-GTPase2.2->GAP1-N2+GAP1-M+GAP1-C*->PSE->?->?->||<-?<-?<-HSP70<-unknown+CASPASE

■ **GCA_900106995.1; Saccharopolyspora shandongensis; counts= 1; multicellularity status: TRUE**

- GrpE->HSP70->TPR_containing_alpha_helical+Fibronectin-like_repeats->ZnR+ZnR+DO-GTPase2.2->GAP1-N2+GAP1-M+GAP1-C*->TM+TM+TM+TM+ZnR+ZnR+DO-GTPase2.2->HSP70->vWA(canonical)->

■ **GCA_003053965.1; Saccharopolyspora; counts= 1; multicellularity status: TRUE**

- GrpE->HSP70->TPR_containing_alpha_helical+Fibronectin-like_repeats->ZnR+ZnR+DO-GTPase2.2->GAP1-N2+GAP1-M+GAP1-C*->PSE->?->?->||<-?<-?<-HSP70<-unknown+CASPASE

■ **GCF_900101695.1; Pseudomonas sp. UC 17F4; counts= 1; multicellularity status: FALSE**

- Calcineurin+GAPEAD1->GAP2->ZnR+DO-GTPase2.1->DO-GTPase1->GAP1-N1+GAP1-M+GAP1-C*->

■ **GCA_900115295.1; Sphingomonas sp. OK281; counts= 1; multicellularity status: FALSE**

- GAP1-N1+GAP1-M+GAP1-C*->

■ **GCA_900092005.1; Streptomyces sp. Cmuel-A718b; counts= 1; multicellularity status: TRUE**

- GAP1-N2+GAP1-M+GAP1-C*->

■ **GCA_002797835.1; Streptomyces; counts= 1; multicellularity status: TRUE**

- TPR_containing_alpha_helical+Fibronectin-like_repeats->ZnR+ZnR+DO-GTPase2.2->GAP1-N2+GAP1-M+GAP1-C*->PSE->vWA(canonical)->PP2C->Pkinase+ZnR+beta-rich->

■ **GCA_900091765.1; Streptomyces; counts= 1; multicellularity status: TRUE**

- GAP1-N2+GAP1-M+GAP1-C*->

■ **GCA_900091725.1; Streptomyces sp. DvalAA-43; counts= 1; multicellularity status: TRUE**

- GAP1-N2+GAP1-M+GAP1-C*->

■ **GCA_900091905.1; Streptomyces sp. OspMP-M43; counts= 1; multicellularity status: TRUE**

- HSP70_domain_2+low_complexity+domain_betaX->ZnR+ZnR+DO-GTPase2.2->GAP1-N2+GAP1-M+GAP1-C*->

■ **GCA_900091995.1; Streptomyces sp. MnatMP-M17; counts= 1; multicellularity status: TRUE**

- GAP1-N2+GAP1-M+GAP1-C*->

■ **GCA_900090065.1; Streptomyces sp. OspMP-M45; counts= 1; multicellularity status: TRUE**

- GAP1-N2+GAP1-M+GAP1-C*->

■ **GCA_900090075.1; Streptomyces sp. MnatMP-M77; counts= 1; multicellularity status: TRUE**

- GAP1-N2+GAP1-M+GAP1-C*->

■ **GCA_900091775.1; Streptomyces sp. ScaeMP-e83; counts= 1; multicellularity status: TRUE**

- GAP1-N2+GAP1-M+GAP1-C*->

■ **GCA_900105515.1; Streptomyces sp. 2131.1; counts= 1; multicellularity status: TRUE**

- GAP1-N2+GAP1-M+GAP1-C*->

■ **GCA_900101975.1; Streptomyces sp. 136MFCol5.1; counts= 1; multicellularity status: TRUE**

- GAP1-N2+GAP1-M+GAP1-C*->

■ **GCA_900090085.1; Streptomyces sp. Ncost-T6T-1; counts= 1; multicellularity status: TRUE**

- GAP1-N2+GAP1-M+GAP1-C*->

■ **GCA_900091845.1; Streptomyces sp. SolWspMP-5a-2; counts= 1; multicellularity status: TRUE**

- GAP1-N2+GAP1-M+GAP1-C*->vWA(canonical)->PP2C->Pkinase+ZnR+beta-rich->

■ **GCA_900091885.1; Streptomyces sp. ScaeMP-6W; counts= 1; multicellularity status: TRUE**

- GAP1-N2+GAP1-M+GAP1-C*->

■ **GCA_900091955.1; Streptomyces sp. Ncost-T10-10d; counts= 1; multicellularity status: TRUE**

- GAP1-N2+GAP1-M+GAP1-C*->

■ **GCA_900090135.1; Streptomyces sp. LaPpAH-199; counts= 1; multicellularity status: TRUE**

- GAP1-N2+GAP1-M+GAP1-C*->

■ **GCA_900103585.1; Streptomyces sp. cf386; counts= 1; multicellularity status: TRUE**

- TPR_containing_alpha_helical+Fibronectin-like_repeats->ZnR+ZnR+DO-GTPase2.2->GAP1-N2+GAP1-M+GAP1-C*->TM+TM+TM+TM+ZnR+ZnR+DO-GTPase2.2+TM->vWA(canonical)->PP2C->Pkinase+ZnR+beta-rich->

■ **GCA_900116325.1; Streptomyces sp. ok210; counts= 1; multicellularity status: TRUE**

- GAP1-N2+GAP1-M+GAP1-C*->

■ **GCA_002234615.1; Paenibacillus rigui; counts= 1; multicellularity status: TRUE**

- TM+vWA(canonical)+YtKA-like+TM+??->Tubulin+coiled_coil+??->Tubulin+coiled_coil+HTH?+??->TM+TM+TM+vWA(canonical)+TM+TM+TM+polar_tail->Fibronectin-like_repeats+??->ZnR+ZnR+DO-GTPase2.2+ZnR+ZnR->GAP1-N2+GAP1-M+GAP1-C+polar_tail+TM+Cadherin-like*->

■ **GCA_002240055.1; Leptotrichia sp. oral taxon 498; counts= 1; multicellularity status: TRUE**

- TM+TM+TM+TM+ZnR+ZnR+DO-GTPase2.2->?->HSP70->HSP70+vWA(canonical)->DnaJ+DnaJ+ZnR+ZnR+coiled_coil+Fibronectin-like_repeats->ZnR+ZnR+DO-GTPase2.2->GAP1-N2+GAP1-M+GAP1-C*->

■ **GCA_002240415.1; Paenibacillus kribbensis; counts= 1; multicellularity status: TRUE**

- TM+vWA(canonical)+YtKA-like+TM+??->Tubulin+coiled_coil+??->Tubulin+coiled_coil+HTH?+??->TM+TM+TM+vWA(canonical)+TM+TM+TM+polar_tail->Fibronectin-like_repeats+??->ZnR+ZnR+DO-GTPase2.2+ZnR+ZnR->GAP1-N2+GAP1-M+GAP1-C+polar_tail+TM+beta-rich*->

■ **GCF_002242735.1; Streptomyces sp. 2R; counts= 1; multicellularity status: TRUE**

- GAP1-N2+GAP1-M+GAP1-C*->

■ **GCA_002245895.1; Rhodococcus sp. OK302; counts= 1; multicellularity status: TRUE**

- polar_leader+TM+TM+TM->TM+vWA(canonical)+Fibronectin-like_repeats+TM+beta-rich+lc_tail->Tubulin+alpha_helical+??+??->GAP_unknown1+TM+TM+GAP_unknown2->HRDC-like?+Fibronectin-like_repeats->GAP1-N2+GAP1-M+GAP1-C*->beta-rich+vWA(canonical)->??+DO-GTPase2.2->

■ **GCA_002250525.1; Propionibacteriaceae bacterium NML 150272; counts= 1; multicellularity status: NA**

- GrpE->HSP70->TPR_containing_alpha_helical+Fibronectin-like_repeats->ZnR+ZnR+DO-GTPase2.2->GAP1-N2+GAP1-M+GAP1-C*->TM+TM+TM+TM+ZnR+ZnR+DO-GTPase2.2->??->?->?->?->?->HSP70->

■ **GCA_002250625.1; Propionibacteriaceae bacterium NML 130396; counts= 1; multicellularity status: NA**

- TM+vWA(canonical)+Fibronectin-like_repeats+TM+beta-rich+lc_tail->Tubulin+alpha_helical+??+??->GAP_unknown1+TM+TM+GAP_unknown2->HRDC-like?+Fibronectin-like_repeats->GAP1-N2+GAP1-M+GAP1-C*->beta-rich+vWA(canonical)->??+DO-GTPase2.2->

■ **GCA_002251155.1; Streptomyces sp. FBKL.4005; counts= 1; multicellularity status: TRUE**

- TPR_containing_alpha_helical+Fibronectin-like_repeats->ZnR+ZnR+DO-GTPase2.2->GAP1-N2+GAP1-M+GAP1-C*->TM+TM+TM+TM+ZnR+ZnR+DO-GTPase2.2+TM->vWA(canonical)->PSE->||<-PSE||Pkinase+ZnR+beta-rich->

■ **GCA_002250475.1; Propionibacteriaceae bacterium NML 160184; counts= 1; multicellularity status: NA**

- polar_leader+TM+TM+TM->TM+vWA(canonical)+Fibronectin-like_repeats+TM+beta-rich+lc_tail->Tubulin+alpha_helical+??+??->GAP_unknown1+TM+TM+GAP_unknown2->HRDC-like?+Fibronectin-like_repeats->GAP1-N2+GAP1-M+GAP1-C*->beta-rich+vWA(canonical)->??+DO-GTPase2.2->

■ **GCA_002250565.1; Propionibacteriaceae bacterium NML 150081; counts= 1; multicellularity status: NA**

- polar_leader+TM+TM+TM->TM+vWA(canonical)+Fibronectin-like_repeats+TM+beta-rich+lc_tail->Tubulin+alpha_helical+??+??->GAP_unknown1+TM+TM+GAP_unknown2->HRDC-like?+Fibronectin-like_repeats->GAP1-N2+GAP1-M+GAP1-C*->beta-rich+vWA(canonical)->??+DO-GTPase2.2->

■ **GCA_002257645.1; Paenibacillus sp. XY044; counts= 1; multicellularity status: TRUE**

- TM+vWA(canonical)+YtKA-like+TM+??->Tubulin+coiled_coil+??->Tubulin+coiled_coil+HTH?+??->TM+TM+TM+vWA(canonical)+TM+TM+TM+polar_tail->Fibronectin-like_repeats+??->?->ZnR+ZnR+DO-GTPase2.2+ZnR+ZnR->GAP1-N2+GAP1-M+GAP1-C+polar_tail+TM+beta-rich*->

■ **GCA_002259035.1; Rhodococcus; counts= 1; multicellularity status: TRUE**

- polar_leader+TM+TM+TM->TM+vWA(canonical)+Fibronectin-like_repeats+TM+beta-rich+lc_tail->Tubulin+alpha_helical+??+??->GAP_unknown1+TM+TM+GAP_unknown2->HRDC-like?+Fibronectin-like_repeats->GAP1-N2+GAP1-M+GAP1-C*->beta-rich+vWA(canonical)->??+DO-GTPase2.2->

■ **GCA_002259605.1; Pseudoscardovia radai; counts= 1; multicellularity status: FALSE**

- TM+TM+TM+TM+ZnR+ZnR+DO-GTPase2.2->GrpE->HSP70->DnaJ+DnaJ+ZnR+ZnR+coiled_coil+Fibronectin-like_repeats->ZnR+ZnR+DO-GTPase2.2->GAP1-N2+GAP1-M+GAP1-C*->

■ **GCA_002259645.1; Bifidobacterium tissieri; counts= 1; multicellularity status: FALSE**

- TM+TM+TM+TM+ZnR+ZnR+DO-GTPase2.2->GrpE->HSP70->HSP70+vWA(canonical)->DnaJ+DnaJ+ZnR+ZnR+coiled_coil+Fibronectin-like_repeats->ZnR+ZnR+DO-GTPase2.2->GAP1-N2+GAP1-M+GAP1-C*->

■ **GCF_002259955.1; Brevibacillus laterosporus; counts= 1; multicellularity status: FALSE**

- TM+vWA(canonical)+YtKA-like+TM+??->Tubulin+coiled_coil+??->Tubulin+coiled_coil+HTH?+??->TM+TM+TM+vWA(canonical)+TM+TM+TM+polar_tail->Fibronectin-like_repeats+??->ZnR+ZnR+DO-GTPase2.2+ZnR+ZnR->GAP1-N2+GAP1-M+GAP1-C+polar_tail+TM+beta-rich*->

■ **GCA_002260685.1; Nocardiopsis dassonvillei; counts= 1; multicellularity status: TRUE**

- TPR_containing_alpha_helical+Fibronectin-like_repeats+extended_insert+Fibronectin-like_repeats->ZnR+ZnR+DO-GTPase2.2->GAP1-N2+GAP1-M+GAP1-C*->TM+TM+TM+TM+ZnR+ZnR+DO-GTPase2.2->

■ **GCA_002264385.1; Paenibacillus sp. VTT E-133280; counts= 1; multicellularity status: TRUE**

- TM+vWA(canonical)+YtKA-like+TM+??->Tubulin+coiled_coil+??->Tubulin+coiled_coil+HTH?+??->TM+TM+TM+vWA(canonical)+TM+TM+TM+polar_tail->Fibronectin-like_repeats+??->ZnR+ZnR+DO-GTPase2.2+ZnR+ZnR->GAP1-N2+GAP1-M+GAP1-C+polar_tail+TM+beta-rich*->

■ **GCA_002264395.1; Paenibacillus sp. VTT E-133291; counts= 1; multicellularity status: TRUE**

- TM+vWA(canonical)+YtKA-like+TM+??->Tubulin+coiled_coil+??->Tubulin+coiled_coil+HTH?+??->TM+TM+TM+vWA(canonical)+TM+TM+TM+polar_tail->Fibronectin-like_repeats+??->ZnR+ZnR+DO-GTPase2.2+ZnR+ZnR->GAP1-N2+GAP1-M+GAP1-C+polar_tail+TM+beta-rich*->

■ **GCA_002264345.1; Paenibacillus odorifer; counts= 1; multicellularity status: TRUE**

- TM+vWA(canonical)+YtKA-like+TM+??->Tubulin+coiled_coil+??->Tubulin+coiled_coil+HTH?+??->TM+TM+TM+vWA(canonical)+TM+TM+TM+polar_tail->Fibronectin-like_repeats+??->ZnR+ZnR+DO-GTPase2.2+ZnR+ZnR->GAP1-N2+GAP1-M+GAP1-C+polar_tail+TM+beta-rich*->

■ **GCA_002262935.1; Bacillus sp. SA5d-4; counts= 1; multicellularity status: NA**

- PIN+WG_repeats+Fibronectin-like_repeats->ZnR+ZnR+DO-GTPase2.2->GAP1-N2+GAP1-M+GAP1-C*->TM+TM+TM+TM+ZnR+ZnR+DO-GTPase2.2->

■ **GCF_002264305.1; Paenibacillus taichungensis; counts= 1; multicellularity status: TRUE**

- ZnR+ZnR+DO-GTPase2.2+ZnR+ZnR->GAP1-N2+GAP1-M+GAP1-C+polar_tail+TM+beta-rich*->

■ **GCA_002271635.1; Bacillus circulans; counts= 1; multicellularity status: FALSE**

- GAP1-N2+GAP1-M+GAP1-C+polar_tail+TM+beta-rich*->

■ **GCA_002272715.1; Paenibacillus sp. 7516; counts= 1; multicellularity status: TRUE**

- Tubulin+coiled_coil+??->Tubulin+coiled_coil+HTH?+??->TM+TM+TM+vWA+TM+TM+TM+polar_tail->Fibronectin-like_repeats+??->ZnR+ZnR+DO-GTPase2.2->GAP1-N2+GAP1-M+GAP1-C+polar_tail*->

■ **GCA_002271985.1; Bacillus circulans; counts= 1; multicellularity status: FALSE**

- GAP1-N2+GAP1-M+GAP1-C+polar_tail+TM+beta-rich*->

■ **GCA_002276415.1; Paenibacillus sp. 7523-1; counts= 1; multicellularity status: TRUE**

- Tubulin+coiled_coil+??->Tubulin+coiled_coil+HTH?+??->TM+TM+TM+vWA+TM+TM+TM+polar_tail->Fibronectin-like_repeats+??->ZnR+ZnR+DO-GTPase2.2->GAP1-N2+GAP1-M+GAP1-C+polar_tail*->

■ **GCA_002277955.1; Thermogutta terrifontis; counts= 1; multicellularity status: TRUE**

- ZnR+ZnR+DO-GTPase2.2->GAP1-N2+GAP1-M+GAP1-C*->??+TM+??+beta-rich??->GYF+GYF+TM+TM+TM->

■ **GCF_002286935.1; Bifidobacterium criceti; counts= 1; multicellularity status: FALSE**

- TM+TM+TM+TM+ZnR+ZnR+DO-GTPase2.2->GrpE->HSP70->HSP70+vWA(canonical)->DnaJ+DnaJ+ZnR+ZnR+coiled_coil+Fibronectin-like_repeats->ZnR+ZnR+DO-GTPase2.2->GAP1-N2+GAP1-M+GAP1-C*->

■ **GCA_002286725.1; Pseudomonas indica; counts= 1; multicellularity status: FALSE**

- Calcineurin+GAPEAD1->GAP2->ZnR+DO-GTPase2.1->DO-GTPase1->GAP1-N1+GAP1-M+GAP1-C*->

■ **GCA_002288075.1; Streptomyces sp. CLI2509; counts= 1; multicellularity status: TRUE**

- GAP1-N2+GAP1-M+GAP1-C*->

■ **GCA_002289305.1; Streptomyces albidoflavus; counts= 1; multicellularity status: TRUE**

- GAP1-N2+GAP1-M+GAP1-C*->

■ **GCA_002292625.1; Mycobacterium chimaera; counts= 1; multicellularity status: FALSE**

- TM+DUF4190+TM->TM+DUF4190+TM->polar_leader+TM+TM+TM->TM+vWA(canonical)+Fibronectin-like_repeats+TM+beta-rich+lc_tail->Tubulin+alpha_helical+??+??->GAP_unknown1+TM+TM+GAP_unknown2->HRDC-like?+Fibronectin-like_repeats->GAP1-N2+GAP1-M+GAP1-C(frag)*->beta-rich+vWA(canonical)->??+DO-GTPase2.2->

■ **GCA_002303875.1; Rhodococcus erythropolis; counts= 1; multicellularity status: TRUE**

- GrpE->HSP70->TPR_containing_alpha_helical+Fibronectin-like_repeats->ZnR+ZnR+DO-GTPase2.2->GAP1-N2+GAP1-M+GAP1-C*->TM+TM+TM+TM+ZnR+ZnR+DO-GTPase2.2->??->HSP70->vWA(canonical)->

■ **GCA_002317035.1; Streptomyces albidoflavus; counts= 1; multicellularity status: TRUE**

- GAP1-N2+GAP1-M+GAP1-C*->

■ **GCA_002317005.1; Streptomyces albidoflavus; counts= 1; multicellularity status: TRUE**

- GAP1-N2+GAP1-M+GAP1-C*->

■ **GCA_002318995.1; Shewanella algae; counts= 1; multicellularity status: FALSE**

- GAP1-N1+GAP1-M+GAP1-C+EAD1*->SLATT->

■ **GCA_002332445.1; Brevibacterium linens; counts= 1; multicellularity status: FALSE**

- TM+vWA(canonical)+Fibronectin-like_repeats+TM+beta-rich+lc_tail->Tubulin+alpha_helical+??+??->GAP_unknown1+TM+TM+GAP_unknown2->HRDC-like?+Fibronectin-like_repeats->GAP1-N2+GAP1-M+GAP1-C*->beta-rich+vWA(canonical)->??+DO-GTPase2.2->

■ **GCA_002332325.1; Brevibacterium; counts= 1; multicellularity status: FALSE**

- GrpE->HSP70->TPR_containing_alpha_helical+Fibronectin-like_repeats->ZnR+ZnR+DO-GTPase2.2->GAP1-N2+GAP1-M+GAP1-C*->TM+TM+TM+TM+ZnR+ZnR+DO-GTPase2.2->

■ **GCA_900221035.1; Streptomyces sp. 2323.1; counts= 1; multicellularity status: TRUE**

- TPR_containing_alpha_helical+Fibronectin-like_repeats->ZnR+ZnR+DO-GTPase2.2->GAP1-N2+GAP1-M+GAP1-C*->PSE->vWA(canonical)->PP2C->Pkinase+ZnR+beta-rich->

■ **GCA_002351405.1; Corynebacterium accolens; counts= 1; multicellularity status: FALSE**

- polar_leader+TM+TM+TM->TM+vWA(canonical)+Fibronectin-like_repeats+TM+beta-rich+lc_tail->Tubulin+alpha_helical+??+??->GAP_unknown1+TM+TM+GAP_unknown2->HRDC-like?+Fibronectin-like_repeats->GAP1-N2+GAP1-M+GAP1-C*->beta-rich+vWA(canonical)->??+DO-GTPase2.2->polar_leader+TM+TM+TM->

■ **GCA_002356095.1; Pseudomonas putida; counts= 1; multicellularity status: FALSE**

- GAP2->DO-GTPase2.1->DO-GTPase1->GAP1-N1+GAP1-M+GAP1-C+EAD1*->CASPASE+EAD1->

■ **GCF_002355155.1; Corynebacterium glutamicum; counts= 1; multicellularity status: FALSE**

- polar_leader+TM+TM+TM->TM+vWA(canonical)+Fibronectin-like_repeats+TM+beta-rich+lc_tail->Tubulin+alpha_helical+??+??->GAP_unknown1+TM+TM+GAP_unknown2->HRDC-like?+Fibronectin-like_repeats->GAP1-N2+GAP1-M+GAP1-C*->beta-rich+vWA(canonical)->??+DO-GTPase2.2->

■ **GCA_002382885.1; Streptomyces sp. WZ.A104; counts= 1; multicellularity status: TRUE**

- GAP1-N2+GAP1-M+GAP1-C*->

■ **GCA_002407025.1; Paenibacillus lautus; counts= 1; multicellularity status: TRUE**

- TM+vWA(canonical)+YtKA-like+TM+??->Tubulin+coiled_coil+??->Tubulin+coiled_coil+HTH?+??->TM+TM+TM+vWA(canonical)+TM+TM+TM+polar_tail->Fibronectin-like_repeats+??->ZnR+ZnR+DO-GTPase2.2+ZnR+ZnR->GAP1-N2+GAP1-M+GAP1-C+polar_tail+TM+beta-rich*->

■ **GCA_002412145.1; Brevibacillus laterosporus; counts= 1; multicellularity status: FALSE**

- TM+vWA(canonical)+YtKA-like+TM+??->Tubulin+coiled_coil+??->Tubulin+coiled_coil+HTH?+??->TM+TM+TM+vWA(canonical)+TM+TM+TM+polar_tail->Fibronectin-like_repeats+??->ZnR+ZnR+DO-GTPase2.2+ZnR+ZnR->GAP1-N2+GAP1-M+GAP1-C+polar_tail+TM+beta-rich*->

■ **GCA_900230175.1; Salinibacterium xinjiangense; counts= 1; multicellularity status: FALSE**

- polar_leader+TM+TM+TM->TM+vWA(canonical)+Fibronectin-like_repeats+TM+beta-rich+lc_tail->Tubulin+alpha_helical+??+??->GAP_unknown1+TM+TM+GAP_unknown2->HRDC-like?+Fibronectin-like_repeats->GAP1-N2+GAP1-M+GAP1-C*->beta-rich+vWA(canonical)->??+DO-GTPase2.2->

■ **GCF_900221045.1; Paenibacillus sp. RU26A; counts= 1; multicellularity status: TRUE**

- TM+vWA(canonical)+YtKA-like+TM+??->Tubulin+coiled_coil+??->Tubulin+coiled_coil+HTH?+??->TM+TM+TM+vWA(canonical)+TM+TM+TM+polar_tail->Fibronectin-like_repeats+??->ZnR+ZnR+DO-GTPase2.2+ZnR+ZnR->GAP1-N2+GAP1-M+GAP1-C+polar_tail+TM+beta-rich*->

■ **GCA_900215145.1; Geodermatophilus sabuli; counts= 1; multicellularity status: TRUE**

- Pkinase+TM->ZnR+ZnR+DO-GTPase2.2->GAP1-N2+GAP1-M+GAP1-C*->?->||<-?<-?||?->Pkinase+low_complexity->

■ **GCA_003268965.1; Streptomyces microflavus; counts= 1; multicellularity status: TRUE**

- GAP1-N2+GAP1-M+GAP1-C*->

■ **GCA_002531555.1; Sinorhizobium sp. BJ1; counts= 1; multicellularity status: TRUE**

- Calcineurin+GAPEAD1->GAP2->DO-GTPase2.1->DO-GTPase1->GAP1-N1+GAP1-M+GAP1-C*->

■ **GCA_002531955.1; Rhizobium sp. M10; counts= 1; multicellularity status: TRUE**

- Calcineurin+GAPEAD1->GAP2->DO-GTPase2.1->PSE->GAP1-N1+GAP1-M+GAP1-C*->

■ **GCA_002529455.1; Glycomyces fuscus; counts= 1; multicellularity status: TRUE**

- TPR_containing_alpha_helical+Fibronectin-like_repeats+extended_insert+Fibronectin-like_repeats->ZnR+ZnR+DO-GTPase2.2->GAP1-N2+GAP1-M+GAP1-C*->TM+TM+ZnR+ZnR+DO-GTPase2.2->

■ **GCF_002551245.1; Streptomyces sp. rh34; counts= 1; multicellularity status: TRUE**

- GAP1-N2+GAP1-M+GAP1-C*->

■ **GCF_002551335.1; Streptomyces sp. ms184; counts= 1; multicellularity status: TRUE**

- GAP1-N2+GAP1-M+GAP1-C*->

■ **GCF_002551135.1; Streptomyces sp. st140; counts= 1; multicellularity status: TRUE**

- GAP1-N2+GAP1-M+GAP1-C*->

■ **GCF_002551105.1; Streptomyces sp. st170; counts= 1; multicellularity status: TRUE**

- GAP1-N2+GAP1-M+GAP1-C*->

■ **GCF_002551045.1; Streptomyces sp. wa1063; counts= 1; multicellularity status: TRUE**

- TPR_containing_alpha_helical+Fibronectin-like_repeats->ZnR+ZnR+DO-GTPase2.2->GAP1-N2+GAP1-M+GAP1-C*->TM+TM+TM+TM+ZnR+ZnR+DO-GTPase2.2+TM->vWA(canonical)->PP2C->Pkinase+ZnR+beta-rich->

■ **GCF_002551215.1; Streptomyces sp. rh207; counts= 1; multicellularity status: TRUE**

- GAP1-N2+GAP1-M+GAP1-C*->

■ **GCF_002551345.1; Streptomyces sp. ms115; counts= 1; multicellularity status: TRUE**

- GAP1-N2+GAP1-M+GAP1-C*->

■ **GCF_002551015.1; Streptomyces; counts= 1; multicellularity status: TRUE**

- ZnR+ZnR+DO-GTPase2.2->GAP1-N2+GAP1-M+GAP1-C*->TM+TM+TM+TM+ZnR+ZnR+DO-GTPase2.2+TM->vWA(canonical)->PP2C->Pkinase+ZnR+beta-rich->

■ **GCF_002551305.1; Streptomyces sp. or20; counts= 1; multicellularity status: TRUE**

- GAP1-N2+GAP1-M+GAP1-C*->

■ **GCF_002551375.1; Streptomyces sp. gb14; counts= 1; multicellularity status: TRUE**

- GAP1-N2+GAP1-M+GAP1-C*->

■ **GCF_002551435.1; Streptomyces sp. f150; counts= 1; multicellularity status: TRUE**

- GAP1-N2+GAP1-M+GAP1-C*->

■ **GCF_002551415.1; Streptomyces sp. f51; counts= 1; multicellularity status: TRUE**

- GAP1-N2+GAP1-M+GAP1-C*->

■ **GCF_002551185.1; Streptomyces sp. sk226; counts= 1; multicellularity status: TRUE**

- GAP1-N2+GAP1-M+GAP1-C*->

■ **GCF_002551235.1; Streptomyces sp. rh195; counts= 1; multicellularity status: TRUE**

- GAP1-N2+GAP1-M+GAP1-C*->

■ **GCF_002551475.1; Streptomyces sp. b62; counts= 1; multicellularity status: TRUE**

- GAP1-N2+GAP1-M+GAP1-C*->

■ **GCF_002551145.1; Streptomyces sp. st115; counts= 1; multicellularity status: TRUE**

- GAP1-N2+GAP1-M+GAP1-C*->

■ **GCA_002556545.1; Streptomyces formicae; counts= 1; multicellularity status: TRUE**

- GAP1-N2+GAP1-M+GAP1-C*->vWA(canonical)->PP2C->Pkinase+ZnR+beta-rich->||<-TPR_containing_alpha_helical+Fibronectin-like_repeats

■ **GCA_002565135.1; Bacillus sp. AFS076308; counts= 1; multicellularity status: NA**

- TM+vWA(canonical)+YtKA-like+TM+??->Tubulin+coiled_coil+??->Tubulin+coiled_coil+HTH?+??->Fibronectin-like_repeats+??->ZnR+ZnR+DO-GTPase2.2+ZnR+ZnR->GAP1-N2+GAP1-M+GAP1-C*->

■ **GCF_002573715.1; Paenibacillus sp. EZ-K15; counts= 1; multicellularity status: TRUE**

- TM+vWA(canonical)+YtKA-like+TM+??->?->Tubulin+coiled_coil+??->Tubulin+coiled_coil+HTH?+??->TM+TM+TM+vWA(canonical)+TM+TM+TM+polar_tail->Fibronectin-like_repeats+??->ZnR+ZnR+DO-GTPase2.2+ZnR+ZnR->GAP1-N2+GAP1-M+GAP1-C+polar_tail+TM+beta-rich*->

■ **GCF_002551295.1; Streptomyces sp. or3; counts= 1; multicellularity status: TRUE**

- GAP1-N2+GAP1-M+GAP1-C*->

■ **GCF_002551065.1; Streptomyces sp. t99; counts= 1; multicellularity status: TRUE**

- GAP1-N2+GAP1-M+GAP1-C*->

■ **GCF_002551175.1; Streptomyces sp. st77; counts= 1; multicellularity status: TRUE**

- GAP1-N2+GAP1-M+GAP1-C*->

■ **GCA_002584635.1; Bacillus sp. AFS031507; counts= 1; multicellularity status: NA**

- TM+vWA(canonical)+YtKA-like+TM+??->Tubulin+coiled_coil+??->Tubulin+coiled_coil+HTH?+??->TM+TM+TM+vWA(canonical)+TM+TM+TM+polar_tail->Fibronectin-like_repeats+??->ZnR+ZnR+DO-GTPase2.2+ZnR+ZnR->GAP1-N2+GAP1-M+GAP1-C+polar_tail+TM+beta-rich*->

■ **GCA_002585305.1; Bacillus sp. AFS037270; counts= 1; multicellularity status: NA**

- TM+vWA(canonical)+YtKA-like+TM+??->Tubulin+coiled_coil+??->Tubulin+coiled_coil+HTH?+??->Fibronectin-like_repeats+??->ZnR+ZnR+DO-GTPase2.2+ZnR+ZnR->GAP1-N2+GAP1-M+GAP1-C*->

■ **GCA_900090175.1; Streptomyces sp. LamerLS-316; counts= 1; multicellularity status: TRUE**

- GAP1-N2+GAP1-M+GAP1-C*->

■ **GCA_002727115.1; Streptomyces cinnamoneus; counts= 1; multicellularity status: TRUE**

- GAP1-N2+GAP1-M+GAP1-C*->

■ **GCF_002688625.1; Xanthomonas citri; counts= 1; multicellularity status: FALSE**

- Calcineurin+GAPEAD1->GAP2->GAP2->GAP2->ZnR+DO-GTPase2.1->DO-GTPase1->GAP1-N1+GAP1-M+GAP1-C*->

■ **GCA_900090185.1; Streptomyces sp. AmelKG-D3; counts= 1; multicellularity status: TRUE**

- GAP1-N2+GAP1-M+GAP1-C*->

■ **GCA_002223215.2; Burkholderia sp. AU18528; counts= 1; multicellularity status: FALSE**

- Calcineurin+GAPEAD1->GAP2->DO-GTPase2.1(fragment)->DO-GTPase1->GAP1-N1+GAP1-M+GAP1-C*->

■ **GCF_002705925.1; Mycobacterium kansasii; counts= 1; multicellularity status: FALSE**

- TM+DUF4190+TM->polar_leader+TM+TM+TM->TM+vWA(canonical)+Fibronectin-like_repeats+TM+beta-rich+lc_tail->Tubulin+alpha_helical+??+??->GAP_unknown1+TM+TM+GAP_unknown2->HRDC-like?+Fibronectin-like_repeats->GAP1-N2+GAP1-M+GAP1-C*->beta-rich+vWA(canonical)->??+DO-GTPase2.2->

■ **GCF_900232885.1; Anaeromassilibacillus sp. Marseille-P4683; counts= 1; multicellularity status: FALSE**

- TM+TM+TM+TM+ZnR+ZnR+DO-GTPase2.2->?->HSP70->HSP70+vWA(canonical)->DnaJ+DnaJ+ZnR+ZnR+coiled_coil+Fibronectin-like_repeats->ZnR+ZnR+DO-GTPase2.2->GAP1-N2+GAP1-M+GAP1-C*->

■ **GCF_002735705.1; Janthinobacterium sp. BJB426; counts= 1; multicellularity status: FALSE**

- GAP1-N1+GAP1-M+GAP1-C+EAD1*->CASPASE+EAD1->

■ **GCF_002735695.1; Chitinimonas sp. BJB300; counts= 1; multicellularity status: FALSE**

- Calcineurin+GAPEAD1->GAP2->ZnR+DO-GTPase2.1->DO-GTPase1->GAP1-N1+GAP1-M+GAP1-C*->

■ **GCA_002736145.1; Collinsella aerofaciens; counts= 1; multicellularity status: FALSE**

- TM+TM+TM+TM+ZnR+ZnR+DO-GTPase2.2->GrpE->HSP70->HSP70+vWA(canonical)->DnaJ+DnaJ+ZnR+Fibronectin-like_repeats->ZnR+ZnR+DO-GTPase2.2->GAP1-N2+GAP1-M+GAP1-C*->

■ **GCA_002741035.1; Paenibacillus sp. BIHB4019; counts= 1; multicellularity status: TRUE**

- TM+vWA(canonical)+YtKA-like+TM+??->Tubulin+coiled_coil+??->Tubulin+coiled_coil+HTH?+??->TM+TM+TM+vWA(canonical)+TM+TM+TM+polar_tail->Fibronectin-like_repeats+??->ZnR+ZnR+DO-GTPase2.2+ZnR+ZnR->GAP1-N2+GAP1-M+GAP1-C+polar_tail+TM+beta-rich*->

■ **GCA_002754245.1; Chryseobacterium sp. 52; counts= 1; multicellularity status: FALSE**

- GAP2->ZnR+DO-GTPase2.1->DO-GTPase1->GAP1-N1+GAP1-M+GAP1-C+EAD1*->??+EAD1->EAD1+Trypsin->

■ **GCF_002750415.1; Paenibacillus polymyxa; counts= 1; multicellularity status: TRUE**

- TM+vWA(canonical)+YtKA-like+TM+??->Tubulin+coiled_coil+??->Tubulin+coiled_coil+HTH?+??->TM+TM+TM+vWA(canonical)+TM+TM+TM+polar_tail->Fibronectin-like_repeats+??->ZnR+ZnR+DO-GTPase2.2+ZnR+ZnR->GAP1-N2+GAP1-M+GAP1-C+polar_tail+TM+beta-rich*->

■ **GCA_002775065.1; Corynebacterium striatum; counts= 1; multicellularity status: FALSE**

- polar_leader+TM+TM+TM->TM+vWA(canonical)+Fibronectin-like_repeats+TM+beta-rich+lc_tail->Tubulin+alpha_helical+??+??->GAP_unknown1+TM+TM+GAP_unknown2->HRDC-like?+Fibronectin-like_repeats->GAP1-N2+GAP1-M+GAP1-C*->beta-rich+vWA(canonical)->??+DO-GTPase2.2->polar_leader+TM+TM+TM->

■ **GCA_002794355.1; Pseudorhodobacter sp. MZDSW-24AT; counts= 1; multicellularity status: FALSE**

- AB_hydrolase+GAPEAD1->GAP2->ZnR+DO-GTPase2.1->DO-GTPase1->GAP1-N1+GAP1-M+GAP1-C*->

■ **GCA_002795805.1; Mariprofundus aestuarium; counts= 1; multicellularity status: TRUE**

- GAP2->ZnR(fragment)+DO-GTPase2.1->DO-GTPase1->GAP1-N1+GAP1-M+GAP1-C+EAD1*->PNP_UDP_1+EAD1->

■ **GCA_002797655.1; Streptomyces sp. CNZ306; counts= 1; multicellularity status: TRUE**

- PP2C->Pkinase->?->?->?->HSP70_domain_2+low_complexity+domain_betaX->ZnR+ZnR+DO-GTPase2.2->GAP1-N2+GAP1-M+GAP1-C*->TM+TM+TM+TM+ZnR+ZnR+DO-GTPase2.2->

■ **GCA_002802945.1; Streptomyces sp. TSRI0384-2; counts= 1; multicellularity status: TRUE**

- GAP1-N2+GAP1-M+GAP1-C*->

■ **GCA_002798385.1; Mycolicibacterium goodii; counts= 1; multicellularity status: FALSE**

- HRDC-like?+Fibronectin-like_repeats->GAP1-N2+GAP1-M+GAP1-C*->??+DO-GTPase2.2->

■ **GCA_002803315.1; Paenibacillus sp. GM1FR; counts= 1; multicellularity status: TRUE**

- Tubulin+coiled_coil+??->Tubulin+coiled_coil+HTH?+??->TM+TM+TM+vWA+TM+TM+TM+polar_tail->Fibronectin-like_repeats+??->ZnR+ZnR+DO-GTPase2.2->GAP1-N2+GAP1-M+GAP1-C+polar_tail*->

■ **GCA_002803325.1; Paenibacillus sp. GM2FR; counts= 1; multicellularity status: TRUE**

- TM+vWA(canonical)+YtKA-like+TM+??->Tubulin+coiled_coil+??->Tubulin+coiled_coil+HTH?+??->TM+TM+TM+vWA(canonical)+TM+TM+TM+polar_tail->Fibronectin-like_repeats+??->ZnR+ZnR+DO-GTPase2.2+ZnR+ZnR->GAP1-N2+GAP1-M+GAP1-C+polar_tail+TM+beta-rich*->

■ **GCA_002256345.1; Bradyrhizobium sp. UASWS1016; counts= 1; multicellularity status: TRUE**

- DO-GTPase1->GAP1-N1+GAP1-M+GAP1-C*->

■ **GCA_002803175.1; Streptomyces sp. CB02613; counts= 1; multicellularity status: TRUE**

- GAP1-N2+GAP1-M+GAP1-C*->

- **GCA_002804085.1; Corynebacterium striatum; counts= 1; multicellularity status: FALSE**
  - polar_leader+TM+TM+TM->TM+vWA(canonical)+Fibronectin-like_repeats+TM+beta-rich+lc_tail->Tubulin+alpha_helical+??+??->GAP_unknown1+TM+TM+GAP_unknown2->HRDC-like?+Fibronectin-like_repeats->GAP1-N2+GAP1-M+GAP1-C*->beta-rich+vWA(canonical)->??+DO-GTPase2.2->polar_leader+TM+TM+TM->
- **GCF_002813225.1; Streptomyces sp. Ag109_G2-1; counts= 1; multicellularity status: TRUE**
  - GAP1-N2+GAP1-M+GAP1-C*->
- **GCA_002844215.1; Streptomyces sp. EAG2; counts= 1; multicellularity status: TRUE**
  - GAP1-N2+GAP1-M+GAP1-C*->
- **GCA_002846895.1; Bifidobacterium asteroides; counts= 1; multicellularity status: FALSE**
  - TM+TM+TM+TM+ZnR+ZnR+DO-GTPase2.2->?->HSP70->HSP70+vWA(canonical)->DnaJ+DnaJ+ZnR+ZnR+coiled_coil+Fibronectin-like_repeats->ZnR+ZnR+DO-GTPase2.2->GAP1-N2+GAP1-M+GAP1-C*->
- **GCA_003335365.1; Brevibacterium aurantiacum; counts= 1; multicellularity status: FALSE**
  - TM+vWA(canonical)+Fibronectin-like_repeats+TM+beta-rich+lc_tail->Tubulin+alpha_helical+??+??->GAP_unknown1+TM+TM+GAP_unknown2->HRDC-like?+Fibronectin-like_repeats->GAP1-N2+GAP1-M+GAP1-C*->beta-rich+vWA(canonical)->??+DO-GTPase2.2->
- **GCA_900169375.1; Brevibacterium sp. Mu109; counts= 1; multicellularity status: FALSE**
  - GrpE->HSP70->TPR_containing_alpha_helical+Fibronectin-like_repeats->ZnR+ZnR+DO-GTPase2.2->GAP1-N2+GAP1-M+GAP1-C*->TM+TM+TM+TM+ZnR+ZnR+DO-GTPase2.2->
- **GCF_002860365.1; Bifidobacterium parmae; counts= 1; multicellularity status: FALSE**
  - TM+TM+TM+TM+ZnR+ZnR+DO-GTPase2.2->GrpE->HSP70->HSP70+vWA(canonical)->DnaJ+DnaJ+ZnR+ZnR+coiled_coil+Fibronectin-like_repeats->ZnR+ZnR+DO-GTPase2.2->GAP1-N2+GAP1-M+GAP1-C*->
- **GCA_002860255.1; Bacillus cucumis; counts= 1; multicellularity status: FALSE**
  - TM+vWA(canonical)+YtKA-like+TM+??->Tubulin+coiled_coil+??->Tubulin+coiled_coil+HTH?+??->Fibronectin-like_repeats+??->ZnR+ZnR+DO-GTPase2.2+ZnR+ZnR->GAP1-N2+GAP1-M+GAP1-C*->
- **GCA_002865525.1; Brevibacillus laterosporus; counts= 1; multicellularity status: FALSE**
  - TM+vWA(canonical)+YtKA-like+TM+??->Tubulin+coiled_coil+??->Tubulin+coiled_coil+HTH?+??->TM+TM+TM+vWA(canonical)+TM+TM+TM+polar_tail->Fibronectin-like_repeats+??->ZnR+ZnR+DO-GTPase2.2+ZnR+ZnR->GAP1-N2+GAP1-M+GAP1-C+polar_tail+TM+beta-rich*->
- **GCF_900199695.1; Clostridiales bacterium mt7; counts= 1; multicellularity status: FALSE**
  - GAP1-N2+GAP1-M+GAP1-C+polar_tail+TM+beta-rich*->
- **GCA_002861325.1; Corynebacterium riegelii; counts= 1; multicellularity status: FALSE**
  - polar_leader+TM+TM+TM->TM+vWA(canonical)+Fibronectin-like_repeats+TM+beta-rich+lc_tail->Tubulin+alpha_helical+??+??->GAP_unknown1+TM+TM+GAP_unknown2->HRDC-like?+Fibronectin-like_repeats->GAP1-N2+GAP1-M+GAP1-C*->?->??+DO-GTPase2.2->?->||<-?<-?<-HSP70
- **GCA_002861385.1; Corynebacterium aurimucosum; counts= 1; multicellularity status: FALSE**
  - polar_leader+TM+TM+TM->TM+vWA(canonical)+Fibronectin-like_repeats+TM+beta-rich+lc_tail->Tubulin+alpha_helical+??+??->GAP_unknown1+TM+TM+GAP_unknown2->HRDC-like?+Fibronectin-like_repeats->GAP1-N2+GAP1-M+GAP1-C*->beta-rich+vWA(canonical)->??+DO-GTPase2.2->polar_leader+TM+TM+TM->
- **GCA_001742065.1; Psychrobacter sp. Choline-02u-9; counts= 1; multicellularity status: FALSE**
  - PNP_UDP_1+EAD1->GAP2->ZnR+DO-GTPase2.1->DO-GTPase1->GAP1-N1+GAP1-M+GAP1-C+EAD1*->
- **GCA_002871735.1; Corynebacterium aurimucosum; counts= 1; multicellularity status: FALSE**
  - polar_leader+TM+TM+TM->TM+vWA(canonical)+Fibronectin-like_repeats+TM+beta-rich+lc_tail->Tubulin+alpha_helical+??+??->GAP_unknown1+TM+TM+GAP_unknown2->HRDC-like?+Fibronectin-like_repeats->GAP1-N2+GAP1-M+GAP1-C*->beta-rich+vWA(canonical)->??+DO-GTPase2.2->polar_leader+TM+TM+TM->
- **GCA_002871875.1; Brevibacterium paucivorans; counts= 1; multicellularity status: FALSE**

- polar_leader+TM+TM+TM->TM+vWA(canonical)+Fibronectin-like_repeats+TM+beta-rich+lc_tail->Tubulin+alpha_helical+??+??->GAP_unknown1+TM+TM+GAP_unknown2->HRDC-like?+Fibronectin-like_repeats->GAP1-N2+GAP1-M+GAP1-C*->?->??+DO-GTPase2.2->?->||<-?<-?<-TM+TM+vWA(canonical)+TM<-TM+TM+vWA(canonical)+TM

■ **GCA_002871975.1; Actinomyces graevenitzii; counts= 1; multicellularity status: TRUE**

- GrpE->HSP70->TPR_containing_alpha_helical+Fibronectin-like_repeats->ZnR+ZnR+DO-GTPase2.2->GAP1-N2+GAP1-M+GAP1-C*->TM+TM+TM+TM+ZnR+ZnR+DO-GTPase2.2->

■ **GCA_002890895.1; Pseudomonas stutzeri; counts= 1; multicellularity status: FALSE**

- Calcineurin+GAPEAD1->GAP2->ZnR+DO-GTPase2.1->DO-GTPase1->GAP1-N1+GAP1-M+GAP1-C*->

■ **GCA_002892225.1; Phaeobacter inhibens; counts= 1; multicellularity status: FALSE**

- AB_hydrolase+GAPEAD1->GAP2->ZnR+DO-GTPase2.1->DO-GTPase1->GAP1-N1+GAP1-M+GAP1-C*->

■ **GCA_002892085.1; Phaeobacter inhibens; counts= 1; multicellularity status: FALSE**

- AB_hydrolase+GAPEAD1->GAP2->ZnR+DO-GTPase2.1->DO-GTPase1->GAP1-N1+GAP1-M+GAP1-C*->

■ **GCF_002893865.1; Vibrio campbellii; counts= 1; multicellularity status: FALSE**

- Calcineurin+GAPEAD1->GAP2->ZnR+DO-GTPase2.1->DO-GTPase1->GAP1-N1+GAP1-M+GAP1-C*->

■ **GCA_002904025.1; Pectobacterium carotovorum; counts= 1; multicellularity status: FALSE**

- GAP2->ZnR+DO-GTPase2.1->DO-GTPase1->GAP1-N1+GAP1-M+GAP1-C*->

■ **GCA_002925765.1; Aeromonas jandaei; counts= 1; multicellularity status: FALSE**

- Calcineurin+GAPEAD1->GAP2->ZnR+DO-GTPase2.1->DO-GTPase1->GAP1-N1+GAP1-M+GAP1-C*->

■ **GCA_002943465.1; Rhodoferax sp. TS-BS-61-7; counts= 1; multicellularity status: FALSE**

- GAP2->DO-GTPase2.1->DO-GTPase1->GAP1-N1+GAP1-M+GAP1-C+EAD1*->CASPASE+EAD1->

■ **GCA_002981255.1; Burkholderia multivorans; counts= 1; multicellularity status: FALSE**

- Calcineurin+GAPEAD1->GAP2->ZnR+DO-GTPase2.1->DO-GTPase1->GAP1-N1+GAP1-M+GAP1-C*->

■ **GCF_003002825.1; Spirosoma oryzae; counts= 1; multicellularity status: TRUE**

- GAP2->ZnR+DO-GTPase2.1->DO-GTPase1->GAP1-N1+GAP1-M+GAP1-C+EAD1*->PNP_UDP_1+EAD1->

■ **GCA_003008595.1; Simplicispira sp. SC1-8; counts= 1; multicellularity status: NA**

- Calcineurin+GAPEAD1->GAP2->ZnR+DO-GTPase2.1->PSE->GAP1-N1+GAP1-M+GAP1-C*->

■ **GCF_001008325.2; Leptospira santarosai; counts= 1; multicellularity status: FALSE**

- Calcineurin+GAPEAD1->PSE->PSE->ZnR+DO-GTPase2.1->DO-GTPase1->DO-GTPase1->GAP1-N1+GAP1-M+GAP1-C*->

■ **GCA_003049865.1; Vibrio splendidus; counts= 1; multicellularity status: FALSE**

- Calcineurin+GAPEAD1->GAP2->ZnR+DO-GTPase2.1->DO-GTPase1->GAP1-N1+GAP1-M+GAP1-C*->

■ **GCA_003063455.1; Limnohabitans parvus; counts= 1; multicellularity status: FALSE**

- Calcineurin+GAPEAD1->GAP2->ZnR+DO-GTPase2.1->PSE->GAP1-N1+GAP1-M+GAP1-C*->

■ **GCF_003057255.1; Klebsiella aerogenes; counts= 1; multicellularity status: FALSE**

- Calcineurin+GAPEAD1->GAP2->ZnR+DO-GTPase2.1->DO-GTPase1->GAP1-N1+GAP1-M+GAP1-C*->

■ **GCF_002168275.1; Herrania umbratica; counts= 1; multicellularity status: NA**

- GAP1-N2+GAP1-M+GAP1-C(frag)*->

NucA systems

- **GCF_000503075.1; Mesorhizobium sp. LSHC422A00; counts= 2; multicellularity status: TRUE**
  - EAD1+Trypsin+TM+NPLC_P60*->
  - EAD1+Trypsin+TM+TM+disordered*->
- **GCF_000503035.1; Mesorhizobium sp. LSHC412B00; counts= 2; multicellularity status: TRUE**
  - EAD1+Trypsin+TM+NPLC_P60*->
  - Trypsin+NucA->EAD1+Trypsin*->EADunk+helical_domain->low_complexity+TM+NucA(inactive)+iSvmX2->
- **GCF_000344805.1; Bradyrhizobium oligotrophicum; counts= 2; multicellularity status: TRUE**
  - calcineurin(low_complexity_insert)->Trypsin+EAD1+coiled_coil*->wHTH+TM+PPRs+TPRs->
  - EAD1+Trypsin->*EAD7+helical_domain*->STAND+TM+TPRs->
- **GCF_900113985.1; Phyllobacterium sp. CL33Tsu; counts= 2; multicellularity status: TRUE**
  - EAD1+Trypsin*->||<-?<-?<-Ploop_NTPase+TM+??
  - IG-like+EAD1*->
- **GCF_000686005.1; Phyllobacterium sp. UNC302MFCol5.2; counts= 2; multicellularity status: TRUE**
  - EAD1+Trypsin*->||<-?<-?<-Ploop_NTPase+TM+??
  - IG-like+EAD1*->
- **GCF_000970465.2; Devosia insulae; counts= 2; multicellularity status: FALSE**
  - EAD1+Trypsin*->
  - Trypsin->EAD1+lysozyme->EAD1+Trypsin->*EAD1+helical_domain*->low_complexity+TM+NucA(inactive)+iSvmX2->Trypsin+NucA->
- **GCF_000502815.1; Mesorhizobium sp. LSJC269B00; counts= 2; multicellularity status: TRUE**
  - EAD1+Trypsin*->?->STAND+wHTH+TPRs->
  - Trypsin+NucA->EAD1+Trypsin*->EADunk+helical_domain->low_complexity+TM+NucA(inactive)+iSvmX2->
- **GCF_001043915.1; Methylobacterium aquaticum; counts= 1; multicellularity status: TRUE**
  - EAD1+Trypsin+EAD1+STAND*->low_complexity+TM+NucA(inactive)+iSvmX2->||<-?<-CASPASE+??||Trypsin->
- **GCA_000503035.1; Mesorhizobium sp. LSHC412B00; counts= 1; multicellularity status: TRUE**
  - EAD1+Trypsin+TM+NPLC_P60*->
- **GCF_900112185.1; Bosea sp. DSM 18164; counts= 1; multicellularity status: TRUE**
  - <-Trypsin_2+NucA+Calcineurin<-?<-?||EAD1+Trypsin*->?->??+TM+??+??+iSvmX2->

- **GCF_000022085.1; Methylobacterium nodulans; counts= 1; multicellularity status: TRUE**
  - lysozyme+peptidase_MD->||<-Trypsin+NucA||EAD1+Trypsin*->?->??+TM+SH3+iSvmX2->
- **GCF_000015165.1; Bradyrhizobium sp. BTAi1; counts= 1; multicellularity status: TRUE**
  - EAD1+Trypsin*->?->Ploop_NTPase+TM+??->
- **GCF_001296055.1; Novosphingobium sp. AAP93; counts= 1; multicellularity status: FALSE**
  - EAD1+Trypsin*->?->AAA_ATPase(inactive)+TM+SH3+iSvmX2->||<-PSE||?->Trypsin+NucA->
- **GCF_900117425.1; Novosphingobium sp. NDB2Meth1; counts= 1; multicellularity status: FALSE**
  - EAD1+Trypsin*->?->AAA_ATPase(inactive)+TM+SH3+iSvmX2->||<-PSE||?->Trypsin+NucA->
- **GCF_000985995.1; Neorhizobium galegae; counts= 1; multicellularity status: FALSE**
  - <-Trypsin+NucA||lysozyme+PGBD->EAD1+Trypsin*->EADunk+helical_domain->low_complexity+TM+NucA(inactive)+iSvmX2->GDSL->
- **GCF_001426195.1; Sphingomonas sp. Leaf339; counts= 1; multicellularity status: FALSE**
  - Trypsin+NucA->EAD1+Trypsin*->?->STAND+TM+iSvmX2->
- **GCF_001676615.1; Labrys sp. WJW; counts= 1; multicellularity status: NA**
  - EAD1+Trypsin*->EADunk+helical_domain->??+TM+??->
- **GCA_002858925.1; Caulobacter zeae; counts= 1; multicellularity status: FALSE**
  - EAD1+Trypsin*->?->||<-?<-?||Trypsin->
- **GCF_001624695.1; Labrenzia sp. OB1; counts= 1; multicellularity status: FALSE**
  - Trypsin_2+NucA+Subtilisin->Trypsin->bDLD2+Trypsin+STAND->STAND+wHTH+TPRs->EAD1+Trypsin*->?->low_complexity+Pentapeptide_repeats+TPRs->
- **GCF_000178815.2; Methylosinus; counts= 1; multicellularity status: TRUE**
  - subtilase->EAD1+Trypsin*->STAND+TM+TPRs->||<-PLD+PLD
- **GCF_002407205.1; Rhodobacter sp. CZR27; counts= 1; multicellularity status: FALSE**
  - <-GDSL<-lysozyme+PGBD||??+Trypsin->EAD1+Trypsin*->EADunk+helical_domain->low_complexity+TM+NucA(inactive)+iSvmX2->
- **GCF_002844445.1; Rhodobacter azotoformans; counts= 1; multicellularity status: FALSE**
  - <-GDSL<-lysozyme+PGBD||??+Trypsin->EAD1+Trypsin*->EADunk+helical_domain->low_complexity+TM+NucA(inactive)+iSvmX2->
- **GCF_000016405.1; Rhodobacter sphaeroides; counts= 1; multicellularity status: FALSE**
  - <-GDSL<-lysozyme+PGBD||??+Trypsin->EAD1+Trypsin*->EADunk+helical_domain->low_complexity+TM+NucA(inactive)+iSvmX2->
- **GCF_900104165.1; Phyllobacterium sp. YR620; counts= 1; multicellularity status: TRUE**
  - EAD1+Trypsin*->||<-?<-?<-Ploop_NTPase+TM+??
- **GCF_000376105.1; Asticcacaulis benevestitus; counts= 1; multicellularity status: FALSE**
  - Trypsin+NucA->?->EAD1+Trypsin->*EAD1+Trypsin*->?->AAA_ATPase(inactive)+TM+SH3+iSvmX2->

- **GCA_900106665.1; Paracoccus sanguinis; counts= 1; multicellularity status: NA**
  - <-Trypsin+NucA||EAD1+Trypsin*->?->??+TM+SH3+iSvmX2->GDSL->
- **GCF_001672445.1; Mesorhizobium sp. WSM3873; counts= 1; multicellularity status: TRUE**
  - <-Trypsin<-subtilase<-?<-?<-??+GDSL<-PSE||EAD1+Trypsin*->?->STAND+wHTH+TPRs->
- **GCF_000763825.1; Paracoccus sanguinis; counts= 1; multicellularity status: NA**
  - <-Trypsin+NucA||EAD1+Trypsin*->
- **GCF_002091475.1; Sphingomonas azotifigens; counts= 1; multicellularity status: FALSE**
  - Trypsin+NucA->?->?->AAA_ATPase(inactive)+TM+SH3+iSvmX2->?->EAD1+Trypsin*->
- **GCA_000239775.2; Bradyrhizobium sp. ORS 375; counts= 1; multicellularity status: TRUE**
  - EAD1+Trypsin->*EAD1+??+Ploop_NTPase+??*->
- **GCF_000261805.1; Bradyrhizobium yuanmingense; counts= 1; multicellularity status: TRUE**
  - EAD1+Trypsin*->||<-EAD5+Trypsin<-STAND+wHTH+TPRs<-?<-PLD+PLD<-Trypsin
- **GCF_000233995.1; Mesorhizobium amorphae; counts= 1; multicellularity status: TRUE**
  - Trypsin+NucA->EAD1+Trypsin*->EADunk+helical_domain->
- **GCF_000502255.1; Mesorhizobium sp. L103C120A0; counts= 1; multicellularity status: TRUE**
  - Trypsin+NucA->EAD1+Trypsin*->EADunk+helical_domain->low_complexity+TM+NucA(inactive)+iSvmX2->
- **GCF_000502295.1; Mesorhizobium sp. L103C565B0; counts= 1; multicellularity status: TRUE**
  - Trypsin+NucA->EAD1+Trypsin*->PSE->EADunk+helical_domain->low_complexity+TM+NucA(inactive)+iSvmX2->
- **GCF_000502615.1; Mesorhizobium sp. LNJC405B00; counts= 1; multicellularity status: TRUE**
  - Trypsin+NucA->EAD1+Trypsin*->EADunk+helical_domain->low_complexity+TM+NucA(inactive)+iSvmX2->
- **GCF_000502755.1; Mesorhizobium sp. LSJC264A00; counts= 1; multicellularity status: TRUE**
  - Trypsin+NucA->EAD1+Trypsin*->EADunk+helical_domain->low_complexity+TM+NucA(inactive)+iSvmX2->
- **GCF_000502775.1; Mesorhizobium sp. LSJC265A00; counts= 1; multicellularity status: TRUE**
  - Trypsin+NucA->EAD1+Trypsin*->EADunk+helical_domain->low_complexity+TM+NucA(inactive)+iSvmX2->
- **GCF_000427585.1; Sinorhizobium meliloti; counts= 1; multicellularity status: TRUE**
  - GDSL+CASPASE->subtilase->?->EAD1+Trypsin*->
- **GCF_000502475.1; unclassified Mesorhizobium; counts= 1; multicellularity status: NA**
  - Trypsin+NucA->EAD1+Trypsin*->EADunk+helical_domain->low_complexity+TM+NucA(inactive)+iSvmX2->
- **GCF_000502555.1; unclassified Mesorhizobium; counts= 1; multicellularity status: NA**
  - Trypsin+NucA->EAD1+Trypsin*->EADunk+helical_domain->low_complexity+TM+NucA(inactive)+iSvmX2->

- **GCF_000502695.1; unclassified Mesorhizobium; counts= 1; multicellularity status: NA**
  - Trypsin+NucA->EAD1+Trypsin*->EADunk+helical_domain->low_complexity+TM+NucA(inactive)+iSvmX2->
- **GCF_000502795.1; unclassified Mesorhizobium; counts= 1; multicellularity status: NA**
  - NucA->EAD1+Trypsin*->EADunk+helical_domain->low_complexity+TM+NucA(inactive)+iSvmX2->
- **GCF_000502995.1; unclassified Mesorhizobium; counts= 1; multicellularity status: NA**
  - Trypsin+NucA->EAD1+Trypsin*->EADunk+helical_domain->low_complexity+TM+NucA(inactive)+iSvmX2->
- **GCF_000503155.1; unclassified Mesorhizobium; counts= 1; multicellularity status: NA**
  - Trypsin+NucA->EAD1+Trypsin*->EADunk+helical_domain->low_complexity+TM+NucA(inactive)+iSvmX2->
- **GCF_000502235.1; Mesorhizobium sp. L103C119B0; counts= 1; multicellularity status: TRUE**
  - Trypsin+NucA->EAD1+Trypsin*->EADunk+helical_domain->low_complexity+TM+NucA(inactive)+iSvmX2->
- **GCF_000284375.1; Bradyrhizobium japonicum; counts= 1; multicellularity status: TRUE**
  - calcineurin(low_complexity_insert)->Trypsin+EAD1+coiled_coil*->wHTH+TM+PPRs+TPRs->
- **GCF_000472985.1; Bradyrhizobium japonicum; counts= 1; multicellularity status: TRUE**
  - calcineurin(low_complexity_insert)->Trypsin+EAD1+coiled_coil*->wHTH+TM+PPRs+TPRs->
- **GCF_000773865.1; Bradyrhizobium japonicum; counts= 1; multicellularity status: TRUE**
  - calcineurin(low_complexity_insert)->Trypsin+EAD1+coiled_coil*->wHTH+TM+PPRs+TPRs->
- **GCF_001693485.1; Bradyrhizobium sp. LMTR 3; counts= 1; multicellularity status: TRUE**
  - calcineurin(low_complexity_insert)->Trypsin+EAD1+coiled_coil*->wHTH+TM+PPRs+TPRs->
- **GCF_001641335.1; Bradyrhizobium stylosanthis; counts= 1; multicellularity status: TRUE**
  - calcineurin(low_complexity_insert)->Trypsin+EAD1+coiled_coil*->wHTH+TM+PPRs+TPRs->
- **GCA_000344805.1; Bradyrhizobium oligotrophicum S58; counts= 1; multicellularity status: TRUE**
  - EAD1+Trypsin->EAD7+Lysozyme*->STAND+TM+TPRs->
- **GCF_000011365.1; Bradyrhizobium; counts= 1; multicellularity status: TRUE**
  - HNH+PNPase+EAD7*->
- **GCA_002200945.1; Mesorhizobium amorphae CCBAU 01583; counts= 1; multicellularity status: TRUE**
  - Trypsin->Trypsin->NucA->EAD1*->Trypsin->EAD1->EADunk+helical_domain->??+TM+??->NucA->
- **GCF_900188295.1; Paracoccus sediminis; counts= 1; multicellularity status: NA**
  - Trypsin->||<-?||EAD1+Trypsin-*>EAD1+STAND*->STAND+wHTH+TPRs->

EACC1 systems

- **GCF_000373585.1; Streptomyces sp. MspMP-M5; counts= 5; multicellularity status: TRUE**
  - ??->EACC1*->CASPASE+TPRs->
  - EACC1*->CASPASE+??+TM+TM->
  - EACC1*->CASPASE+TM+??->
  - EACC1*->CASPASE+TM+Beta_rich->
  - EACC1*->CASPASE+TM+BetaPropeller->
- **GCF_000504285.1; Actinospica robiniae; counts= 5; multicellularity status: TRUE**
  - EACC1*->
  - EACC1*->CASPASE+??->
  - EACC1*->CASPASE+Pkinase+TM?+??->
  - EACC1*->CASPASE+TPRs->
  - polar_lead?+TM+TM+TM+polar_tail?+low-complexity->??->EACC1*->CASPASE+TPRs->
- **GCA_001729485.1; Streptomyces lydicus; counts= 4; multicellularity status: TRUE**
  - EACC1*->CASPASE+??->
  - EACC1*->CASPASE+FGS_fold-domain->??->??->TM+TM+BaxI_1->?->NUDIX->
  - Glyoxalase->EACC1*->CASPASE+DnaJ_ZnR+DnaJ_ZnR+DnaJ_C->
  - polar_lead?+TM+TM+TM+polar_tail?+low-complexity->??->EACC1*->CASPASE+TPRs->
- **GCA_000698495.1; Streptomyces sp. NTK 937; counts= 4; multicellularity status: TRUE**
  - EACC1*->CASPASE+STAND+PBPB->
  - EACC1*->CASPASE+STAND+PBPB->
  - EACC1*->CASPASE+STAND+polar_region+TM+BetaPropeller->
  - EACC1*->CASPASE+TPRs->
- **GCA_002846295.1; Streptomyces sp. 59; counts= 4; multicellularity status: TRUE**
  - EACC1*->?->CASPASE+STAND+polar_region+TM+BetaPropeller->TM+Acid_phosphat_B->
  - EACC1*->CASPASE+??+TM+TM+TM+TPRs->
  - EACC1*->CASPASE+BetaPropeller->
  - Pkinase+??->?->?->||<-?<-?||HATPase_c_2->EACC1*->CASPASE+??+SF1-helicase+Vsr(PDDEXK)?->
- **GCA_900107045.1; Amycolatopsis xylanica; counts= 4; multicellularity status: TRUE**
  - ??+TM+TM+??+TM->PBPB->EACC1*->CASPASE+TPRs->PknG_rubred+Pkinase+PknG_TPR->ZnR+PP2C->vWA+alpha-helical->
  - EACC1*->CASPASE+DnaJ_ZnR+DnaJ_ZnR+DnaJ_C->

- EACC1*->CASPASE+STAND+polar_region+TM+BetaPropeller->
- GATase->ABC-ATPase+DUF4162->cNMP_cyclase+TM+TM+TM+TM+TM+TM->EACC1*->

■ **GCA_000349325.1; Streptomyces davaonensis; counts= 4; multicellularity status: TRUE**

- EACC1*->
- EACC1*->CASPASE+TM?+DUF3558+DUF3558->
- EACC1*->CASPASE+TM+TM+bPH_domain+TM+TM+bPH_domain+TM+TM->
- EAD2+VMAP-M0+CASPASE->EACC1*->

■ **GCF_000720135.1; Streptomyces sp. NRRL S-1022; counts= 4; multicellularity status: TRUE**

- EACC1*->
- EACC1*->CASPASE+low-complexity+BetaPropeller->
- EACC1*->CASPASE+STAND+polar_region+TM+BetaPropeller->
- EACC1*->CASPASE+TM+TM+bPH_domain?->

■ **GCF_001613485.1; Nocardia rhamnosiphila; counts= 4; multicellularity status: TRUE**

- EACC1*->CASPASE+??+SF1-helicase+Vsr(PDDEXK)?->
- EACC1*->CASPASE+TPRs->
- EACC1*->CASPASE+TPRs->
- EACC1*->CASPASE+TPRs->

■ **GCF_000746395.1; Streptomyces mirabilis; counts= 4; multicellularity status: TRUE**

- ??->EACC1*->CASPASE+TM+TM+PBPB+vWA->
- EACC1*->CASPASE+Cpn60_TCP1->
- EACC1*->CASPASE+STAND+TPRs->
- polar_lead?+TM+TM+TM+polar_tail?+low-complexity->??->EACC1*->CASPASE+TPRs->

■ **GCA_001514065.1; Streptomyces antibioticus; counts= 4; multicellularity status: TRUE**

- EACC1*->CASPASE+STAND+BetaPropeller->
- EACC1*->CASPASE+STAND+polar_region+TM+BetaPropeller->
- EACC1*->CASPASE+TM+TM+TM+TM+TM+TM->
- EACC1*->CASPASE+TPRs->

■ **GCF_002155975.1; Amycolatopsis kentuckyensis; counts= 4; multicellularity status: TRUE**

- EACC1*->
- EACC1*->CASPASE+TPRs->
- GATase->ABC-ATPase+DUF4162->cNMP_cyclase+TM+TM+TM+TM+TM+TM->EACC1*->
- PBPB->?->?->Aminotran_5+PAS_6+HTH_22->EACC1*->

■ **GCA_003173275.1; Streptomyces; counts= 4; multicellularity status: TRUE**

- EACC1*->
- EACC1*->CASPASE+??+SF1-helicase+Vsr(PDDEXK)?->
- EACC1*->CASPASE+Beta_rich_repeat->

- EACC1*->CASPASE+FGS_fold-domain->??->??->TM+TM+BaxI_1->?->NUDIX->

■ **GCA_002573955.1; Streptomyces sp. Ru87; counts= 4; multicellularity status: TRUE**

- EACC1*->
- EACC1*->CASPASE+TM?+Phytase-like->||<-DUF664
- low-complexity+HD+Tox-RelA+TGS+ACT->Pkinase+TPRs->EACC1*->CASPASE+TPRs->
- polar_lead?+TM+TM+TM+polar_tail?+low-complexity->??->EACC1*->CASPASE+TPRs->

■ **GCA_000494755.1; Actinoplanes friuliensis DSM 7358; counts= 3; multicellularity status: TRUE**

- EACC1*->
- EACC1*->CASPASE+??->
- EACC1*->CASPASE+DUF179->SIR2+??->??->

■ **GCA_002591335.1; Streptomyces malaysiensis; counts= 3; multicellularity status: TRUE**

- EACC1*->CASPASE+HisKA+HATPase_c->
- EACC1*->CASPASE+TM+??->
- TetR_N+TetR_C_6->EACC1*->CASPASE+low-complexity+TM+PBPB->

■ **GCA_000938975.1; Streptomyces iranensis; counts= 3; multicellularity status: TRUE**

- EACC1*->
- EACC1*->CASPASE+low-complexity+TM+PBPB->
- EACC1*->CASPASE+TM+??->

■ **GCA_001511815.1; Streptomyces reticuli; counts= 3; multicellularity status: TRUE**

- ??->??->EACC1*->CASPASE+??->
- AMP-binding+AMP-binding_C->AMP-binding+AMP-binding_C->EACC1*->CASPASE+STAND+polar_region+TM+BetaPropeller->
- EACC1*->CASPASE+TM?+DUF3558+DUF3558->

■ **GCA_000388135.1; Amycolatopsis vancoresmycina DSM 44592; counts= 3; multicellularity status: TRUE**

- EACC1*->CASPASE+Cpn60_TCP1->
- GATase->ABC-ATPase+DUF4162->cNMP_cyclase+TM+TM+TM+TM+TM+TM->EACC1*->
- PBPB->?->?->Aminotran_5+PAS_6+HTH_22->EACC1*->CASPASE+??+TM+TM->||<-Glyco_trans_4_4+UDPGT

■ **GCA_000611795.1; Streptomyces sp. Tu 6176; counts= 3; multicellularity status: TRUE**

- EACC1*->
- EACC1*->
- TPRs->EACC1*->CASPASE+TPRs->

■ **GCA_001507455.1; Streptomyces albus subsp. albus; counts= 3; multicellularity status: TRUE**

- EACC1*->CASPASE+??->
- EACC1*->CASPASE+PBPB+vWA->
- EACC1*->CASPASE+TPRs->

■ **GCA_001866665.1; Streptomyces sp. MUSC 1; counts= 3; multicellularity status: TRUE**

- EACC1*->
- EACC1*->CASPASE+STAND+polar_region+TM+BetaPropeller->
- Pkinase+TPRs->EACC1*->CASPASE+TPRs->

■ **GCA_001905885.1; Streptomyces sp. CB03234; counts= 3; multicellularity status: TRUE**

- EACC1*->
- EACC1*->
- FAD_binding_4+??+BBE->EACC1*->CASPASE+STAND+polar_region+TM+BetaPropeller->

■ **GCA_002007125.1; Streptomyces tsukubensis; counts= 3; multicellularity status: TRUE**

- EACC1*->CASPASE+??+SF1-helicase+Vsr(PDDEXK)?->
- EACC1*->CASPASE+TPRs->Pkinase+??->
- low-complexity+HD+Tox-RelA+TGS+ACT->Pkinase+TPRs->EACC1*->CASPASE+TPRs->

■ **GCA_002754555.1; Streptomyces sp. 94; counts= 3; multicellularity status: TRUE**

- EACC1*->CASPASE+TM+??->
- EACC1*->CASPASE+TM+PBPI->
- EACC1*->CASPASE+TPRs+LysM+PG-binding->

■ **GCA_002846355.1; Streptomyces sp. GP55; counts= 3; multicellularity status: TRUE**

- EACC1*->CASPASE+??+STAND+??->
- EACC1*->CASPASE+DnaJ_ZnR+DnaJ_C+DnaJ_C+TM+TM->
- polar_lead?+TM+TM+TM+polar_tail?+low-complexity->??->EACC1*->CASPASE+TPRs->

■ **GCA_002846415.1; Streptomyces sp. TLI_146; counts= 3; multicellularity status: TRUE**

- EACC1*->
- EACC1*->CASPASE+low-complexity+BetaPropeller->
- low-complexity+HD+Tox-RelA+TGS+ACT->Pkinase+TPRs->EACC1*->CASPASE+TPRs->

■ **GCA_002934265.1; Actinokineospora auranticolor; counts= 3; multicellularity status: TRUE**

- EACC1*->CASPASE+TM+TM->TM+PBPB->
- EACC1*->CASPASE+TPRs->
- EACC1+CASPASE*->?->Phytase-like->

■ **GCA_900090095.1; Streptomyces sp. SceaMP-e96; counts= 3; multicellularity status: TRUE**

- EACC1*->CASPASE+Beta_rich_repeat->
- EACC1*->CASPASE+FGS_fold-domain->??->??->TM+TM+BaxI_1->?->NUDIX->
- EACC1*->TM+TM->

■ **GCA_900105945.1; Amycolatopsis tolypomycina; counts= 3; multicellularity status: TRUE**

- EACC1*->
- GATase->ABC-ATPase+DUF4162->cNMP_cyclase+TM+TM+TM+TM+TM+TM->EACC1*->
- PBPB->?->?->?->EACC1*->CASPASE+TM+TM->

- **GCA_900119165.1; Amycolatopsis australiensis; counts= 3; multicellularity status: TRUE**
  - EACC1*->CASPASE+TPRs->
  - MFS_1->||<-?||GATase->ABC-ATPase+DUF4162->cNMP_cyclase+TM+TM+TM+TM+TM+TM->EACC1->CASPASE+TM+TM+TM+TM->EACC1*->
  - MFS_1->||<-?||GATase->ABC-ATPase+DUF4162->cNMP_cyclase+TM+TM+TM+TM+TM+TM->EACC1*->
- **GCA_900142595.1; Streptomyces yunnanensis; counts= 3; multicellularity status: TRUE**
  - EACC1*->CASPASE+TM+MFS_1+TM+TM+TM+TM+TM+TM+TM+TM->
  - low-complexity+HD+Tox-RelA+TGS+ACT->Pkinase+TPRs->EACC1*->CASPASE+TPRs->
  - polar_lead?+TM+TM+TM+polar_tail?+low-complexity->??->EACC1*->CASPASE+TPRs->
- **GCA_900188105.1; Actinomadura mexicana; counts= 3; multicellularity status: TRUE**
  - ??+EACC1*->
  - EACC1*->CASPASE+??+TM+TM+TM->
  - EACC1*->CASPASE+TM+TM->
- **GCA_900230195.1; Streptomyces zhaozhouensis; counts= 3; multicellularity status: TRUE**
  - EACC1*->CASPASE+JAB->
  - EACC1*->CASPASE+STAND+polar_region+TM+BetaPropeller->
  - Pkinase+TPRs->EACC1*->CASPASE+TPRs->
- **GCA_900230245.1; Burkholderia sp. OK806; counts= 3; multicellularity status: FALSE**
  - EACC1->Tnp_DNA_bind+DDE_Tnp_1+Dimer_Tnp_Tn5->CASPASE+TPRs->EACC1->CASPASE(fragment)->EACC1*->CASPASE+CASPASE+TM+TPRs->CASPASE(fragment)->
  - EACC1->Tnp_DNA_bind+DDE_Tnp_1+Dimer_Tnp_Tn5->CASPASE+TPRs->EACC1*->CASPASE(fragment)->EACC1->CASPASE+CASPASE+TM+TPRs->CASPASE(fragment)->
  - EACC1*->Tnp_DNA_bind+DDE_Tnp_1+Dimer_Tnp_Tn5->CASPASE+TPRs->
- **GCA_000196835.1; Amycolatopsis mediterranei; counts= 3; multicellularity status: TRUE**
  - EACC1*->CASPASE+??->
  - GATase->ABC-ATPase+DUF4162->cNMP_cyclase+TM+TM+TM+TM+TM+TM->EACC1*->
  - PBPB->?->?->Aminotran_5+PAS_6+HTH_22->EACC1*->||<-Glyco_trans_4_4+UDPGT
- **GCA_000240165.1; Streptomyces cattleya; counts= 3; multicellularity status: TRUE**
  - EACC1*->CASPASE+??+SF1-helicase+Vsr(PDDEXK)?->
  - EACC1->CASPASE+low-complexity+BetaPropeller___*_##->
  - EACC1*->CASPASE+PBPB+vWA->
- **GCF_000375625.1; Streptomyces sp. FxanaC1; counts= 3; multicellularity status: TRUE**
  - EACC1*->CASPASE+Beta_rich_repeat->
  - EACC1*->CASPASE+DnaJ_ZnR+DnaJ_ZnR+DnaJ_C->
  - EACC1*->CASPASE+FGS_fold-domain->??->??->TM+TM+BaxI_1->?->NUDIX->
- **GCF_000381925.1; Nocardia sp. BMG111209; counts= 3; multicellularity status: TRUE**
  - EACC1*->
  - EACC1*->CASPASE+TM+Phytase-like->CASPASE+TM+TM+Phytase-like->CASPASE+TM+Phytase-like->

- EACC1*->CASPASE+TPRs->

■ **GCF_000514995.1; Streptomyces sp. CNT371; counts= 3; multicellularity status: TRUE**

- EACC1*->CASPASE->
- EACC1*->CASPASE+Beta_rich_repeat->
- EACC1*->CASPASE+TPRs->

■ **GCF_000717745.1; Streptomyces violens; counts= 3; multicellularity status: TRUE**

- EACC1*->CASPASE+STAND+polar_region+TM+BetaPropeller->
- EACC1*->CASPASE+TM?+Phytase-like->
- EACC1*->CASPASE+TPRs->

■ **GCF_000719865.1; Streptomyces albus; counts= 3; multicellularity status: TRUE**

- EACC1*->CASPASE+PBPB+vWA->
- EACC1*->CASPASE+STAND+polar_region+TM+BetaPropeller->
- EACC1*->CASPASE+TPRs->??->??->

■ **GCF_000719605.1; Streptomyces sp. NRRL S-1813; counts= 3; multicellularity status: TRUE**

- EACC1*->CASPASE+DnaJ_ZnR+DnaJ_ZnR+DnaJ_C->
- EACC1*->CASPASE+FGS_fold-domain->??->??->
- EACC1*->CASPASE+STAND+polar_region+TM+BetaPropeller->

■ **GCF_000721935.1; Streptomyces sp. NRRL WC-3549; counts= 3; multicellularity status: TRUE**

- EACC1*->
- EACC1*->
- EACC1*->CASPASE+TPRs->TPRs->

■ **GCF_000725465.1; Streptomyces bicolor; counts= 3; multicellularity status: TRUE**

- EACC1*->CASPASE+STAND+polar_region+TM+BetaPropeller->
- EACC1*->CASPASE+TM?+Phytase-like->MDMPI_N+MDMPI_C->
- EACC1*->CASPASE+TM+PBPI->

■ **GCF_000518265.1; Actinomadura oligospora; counts= 3; multicellularity status: TRUE**

- EACC1*->CASPASE+BetaPropeller->
- EACC1*->CASPASE+low-complexity+BetaPropeller->
- EACC1*->CASPASE+SAD+HNH->?->||<-METHYLASE

■ **GCF_000308835.1; Nocardia vinacea; counts= 3; multicellularity status: TRUE**

- EACC1*->CASPASE+??->
- EACC1*->CASPASE+EAL->
- EACC1*->CASPASE+TM+Phytase-like->

■ **GCF_000725485.1; Nonomuraea candida; counts= 3; multicellularity status: TRUE**

- EACC1*->CASPASE+low-complexity+BetaPropeller->

- EACC1*->CASPASE+SAD+HNH->
- EACC1*->CASPASE+TM+TM+??->

■ **GCF_000721075.1; Streptosporangium amethystogenes; counts= 3; multicellularity status: TRUE**

- EACC1*->BetaPropeller(fragment)->
- EACC1*->CASPASE+STAND+polar_region+TM+BetaPropeller->
- EACC1*->CASPASE+STAND+polar_region+TM+BetaPropeller->

■ **GCA_900105465.1; Streptomyces sp. PAN_FS17; counts= 3; multicellularity status: TRUE**

- EACC1*->
- EACC1*->CASPASE+??->
- EACC1*->CASPASE+TPRs->

■ **GCF_001417735.1; Streptomyces sp. NBRC 110028; counts= 3; multicellularity status: TRUE**

- EACC1*->CASPASE+STAND+polar_region+TM+BetaPropeller->
- EACC1*->CASPASE+TPRs->Clp_N+Clp_N+Clp_N+Clp_N+ClpABN-AAA->
- HATPase_c_2->EACC1*->

■ **GCA_001424875.1; Kitasatospora sp. Root107; counts= 3; multicellularity status: TRUE**

- EACC1*->CASPASE+TPRs->
- EACC1*->CASPASE+TPRs->
- polar_lead?+TM+TM+TM+polar_tail?+low-complexity->??->EACC1*->CASPASE+TPRs->

■ **GCA_001514055.1; Streptomyces corchorusii; counts= 3; multicellularity status: TRUE**

- EACC1*->CASPASE+low-complexity+TM+PBPB->
- EACC1*->CASPASE+TM?+Phytase-like->MDMPI_N+MDMPI_C->META->
- EACC1*->CASPASE+TPRs->

■ **GCF_001613225.1; Nocardia pseudovaccinii; counts= 3; multicellularity status: TRUE**

- EACC1*->CASPASE+STAND+polar_region+TM+BetaPropeller->
- EACC1*->CASPASE+TPRs->
- EACC1*->CASPASE+TPRs->

■ **GCA_001542625.2; Streptomyces griseochromogenes; counts= 3; multicellularity status: TRUE**

- EACC1*->CASPASE+low-complexity+BetaPropeller->
- EACC1*->CASPASE+wHTH+BetaPropeller->
- low-complexity+HD+Tox-RelA+TGS+ACT->Pkinase+TPRs->EACC1*->CASPASE+TPRs->

■ **GCA_900104815.1; Streptomyces; counts= 3; multicellularity status: TRUE**

- EACC1*->CASPASE+??+ZnR+ZnR->
- EACC1*->CASPASE+STAND+polar_region+TM+BetaPropeller->
- EACC1*->CASPASE+STAND+polar_region+TM+BetaPropeller->

■ **GCA_001905485.1; Streptomyces sp. TSRI0261; counts= 3; multicellularity status: TRUE**

- Beta_rich?->EACC1*->CASPASE+TPRs->??->??->
- EACC1*->CASPASE+??+SF1-helicase+Vsr(PDDEXK)?->
- EACC1*->CASPASE+Beta_rich_repeat->??+SUKH->

■ **GCF_002154375.1; Streptomyces glaucescens; counts= 3; multicellularity status: TRUE**

- EACC1*->CASPASE+??+BsuPI->
- EACC1*->CASPASE+STAND+polar_region+TM+BetaPropeller->
- EACC1*->PSE->AB_hydrolase->

■ **GCA_002188345.1; Streptomyces sp. CS057; counts= 3; multicellularity status: TRUE**

- EACC1*->
- EACC1*->CASPASE+Beta_rich_repeat->
- EACC1*->CASPASE+STAND+??+TPRs+??->

■ **GCA_002234595.1; Amycolatopsis sp. H5; counts= 3; multicellularity status: TRUE**

- GATase->ABC-ATPase+DUF4162->cNMP_cyclase+TM+TM+TM+TM+TM+TM->EACC1->CASPASE+EAD2+TM+TM+TM+TM->EACC1*->
- GATase->ABC-ATPase+DUF4162->cNMP_cyclase+TM+TM+TM+TM+TM+TM->EACC1*->CASPASE+EAD2+TM+TM+TM+TM->EACC1->
- PBPB->?->?->Aminotran_5+PAS_6+HTH_22->EACC1*->CASPASE+??+TM+TM->

■ **GCF_002904335.1; Amycolatopsis sp. CA-126428; counts= 3; multicellularity status: TRUE**

- EACC1*->CASPASE+HSP70->
- GATase->ABC-ATPase+DUF4162->cNMP_cyclase+TM+TM+TM+TM+TM+TM->EACC1*->
- PBPB->?->?->Aminotran_5+PAS_6+HTH_22->EACC1*->||<-Glyco_trans_4_4+UDPGT

■ **GCA_000147815.3; Streptomyces violaceusniger Tu 4113; counts= 2; multicellularity status: TRUE**

- EACC1*->CASPASE+TM+TM->
- TetR_N+TetR_C_6->EACC1*->CASPASE+low-complexity+TM+PBPB->

■ **GCA_000418455.1; Streptomyces rapamycinicus NRRL 5491; counts= 2; multicellularity status: TRUE**

- EACC1*->
- EACC1*->CASPASE+low-complexity+TM+PBPB->

■ **GCA_000523235.1; Nocardia nova SH22a; counts= 2; multicellularity status: TRUE**

- DUF2510(W3)+BicD+DUF4041+T5orf172->EACC1*->CASPASE+TPRs->
- EACC1*->TM+TM->

■ **GCA_000695235.1; Streptomyces albulus; counts= 2; multicellularity status: TRUE**

- EACC1*->
- EACC1*->CASPASE+TM+TM+bPH_domain?->

■ **GCA_000827005.1; Streptomyces albus; counts= 2; multicellularity status: TRUE**

- EACC1*->CASPASE+HisKA+HATPase_c->
- low-complexity+HD+Tox-RelA+TGS+ACT->Pkinase+TPRs->EACC1*->CASPASE+TPRs->

- **GCA_000931445.1; Streptomyces cyaneogriseus subsp. noncyanogenus; counts= 2; multicellularity status: TRUE**
  - EACC1*->
  - EACC1*->CASPASE+TPRs->Clp_N+Clp_N+ClpABN-AAA->
- **GCA_000952035.2; Streptomyces lydicus; counts= 2; multicellularity status: TRUE**
  - EACC1*->
  - EACC1*->CASPASE+low-complexity+TM+PBPB->
- **GCA_000993785.2; Streptomyces xiamenensis; counts= 2; multicellularity status: TRUE**
  - EACC1*->CASPASE+TPRs->
  - TM+TM+TM+PP2C->EACC1*->CASPASE+PBPB+vWA->
- **GCA_001040905.1; Streptomyces sp. PBH53; counts= 2; multicellularity status: TRUE**
  - ??->??->EACC1*->CASPASE+??->
  - EACC1*->CASPASE+STAND+polar_region+TM+BetaPropeller->
- **GCA_001735805.1; Streptomyces puniciscabiei; counts= 2; multicellularity status: TRUE**
  - EACC1*->CASPASE+Pkinase+TM?+??->
  - EACC1*->CASPASE+STAND+polar_region+TM+BetaPropeller->
- **GCA_001886715.1; Nocardia sp. Y48; counts= 2; multicellularity status: TRUE**
  - EACC1*->
  - EACC1*->CASPASE+TPRs->
- **GCA_002021875.1; Streptomyces hygroscopicus; counts= 2; multicellularity status: TRUE**
  - EACC1*->CASPASE+STAND+polar_region+TM+BetaPropeller->
  - TetR_N+TetR_C_6->EACC1*->CASPASE+low-complexity+TM+PBPB->
- **GCA_003595545.1; Streptomyces sp. 11-1-2; counts= 2; multicellularity status: TRUE**
  - EACC1*->CASPASE+STAND+polar_region+TM+BetaPropeller->
  - Pkinase+TPRs->EACC1*->CASPASE+TPRs->
- **GCA_002812405.1; Streptomyces sp. M56; counts= 2; multicellularity status: TRUE**
  - EACC1*->CASPASE+TM+??->
  - TetR_N+TetR_C_6->EACC1*->CASPASE+low-complexity+TM+PBPB->
- **GCA_002943525.1; Kitasatospora sp. MMS16-BH015; counts= 2; multicellularity status: TRUE**
  - EACC1*->
  - EACC1*->CASPASE+STAND+polar_region+TM+BetaPropeller->
- **GCA_002849735.1; Amycolatopsis sp. BJA-103; counts= 2; multicellularity status: TRUE**
  - EACC1*->CASPASE+STAND+polar_region+TM+BetaPropeller->
  - EACC1*->TM+ZU5+??->

■ **GCA_000284295.1; Actinoplanes missouriensis 431; counts= 2; multicellularity status: TRUE**

- EACC1*->CASPASE+TPRs->
- EACC1*->Clp_N+Clp_N+ClpABN-AAA+CASPASE->

■ **GCA_000158915.1; Streptomyces himastatinicus ATCC 53653; counts= 2; multicellularity status: TRUE**

- EACC1*->CASPASE+TPRs->Clp_N+Clp_N+Clp_N+Clp_N+ClpABN-AAA->
- HATPase_c_2->EACC1*->

■ **GCA_000414115.1; Streptomyces aurantiacus JA 4570; counts= 2; multicellularity status: TRUE**

- EACC1*->
- EACC1*->CASPASE+STAND+polar_region+TM+BetaPropeller->

■ **GCA_000497425.1; Streptomyces niveus NCIMB 11891; counts= 2; multicellularity status: TRUE**

- EACC1*->CASPASE+??+SF1-helicase+Vsr(PDDEXK)?->
- EACC1*->CASPASE+Beta_rich_repeat->

■ **GCA_001653075.1; Planomonospora sphaerica; counts= 2; multicellularity status: TRUE**

- EACC1*->CASPASE+SAD+HNH->
- EACC1*->CASPASE+STAND+polar_region+TM+BetaPropeller->

■ **GCA_001653115.1; Streptomyces sp. F-3; counts= 2; multicellularity status: TRUE**

- EACC1*->CASPASE+??->
- EACC1*->CASPASE+TM+TM+PBPB+vWA->

■ **GCA_000696115.1; Streptomyces olindensis; counts= 2; multicellularity status: TRUE**

- EACC1*->CASPASE+STAND+polar_region+TM+BetaPropeller->
- EACC1*->CASPASE+TM?+Phytase-like->

■ **GCA_001047335.1; Streptomyces regensis; counts= 2; multicellularity status: TRUE**

- EACC1*->
- EACC1*->CASPASE+TM?+Phytase-like->MDMPI_N+MDMPI_C->META->

■ **GCA_001189015.1; Streptomyces acidiscabies; counts= 2; multicellularity status: TRUE**

- EACC1*->CASPASE+low-complexity+BetaPropeller->
- EACC1*->CASPASE+TM+TM+PBPB+vWA->

■ **GCA_001189025.1; Streptomyces europaeiscabiei; counts= 2; multicellularity status: TRUE**

- EACC1*->CASPASE+Beta_rich_repeat->??+SUKH->
- EACC1*->CASPASE+STAND+??+TPRs+??->

■ **GCA_001279985.1; Streptomyces sp. AS58; counts= 2; multicellularity status: TRUE**

- EACC1*->
- EACC1*->CASPASE+TM+TM+bPH_domain+TM+TM+bPH_domain+TM+TM->

- **GCA_001279545.1; Streptomyces sp. NRRL B-3648; counts= 2; multicellularity status: TRUE**
  - EACC1*->
  - EACC1*->CASPASE+STAND+polar_region+TM+BetaPropeller->
- **GCA_001298565.1; Actinobacteria bacterium OK006; counts= 2; multicellularity status: NA**
  - EACC1*->CASPASE+STAND+polar_region+TM+BetaPropeller->
  - EACC1*->CASPASE+TPRs->
- **GCA_001298575.1; Actinobacteria bacterium OK074; counts= 2; multicellularity status: NA**
  - EACC1*->CASPASE+low-complexity+BetaPropeller->
  - EACC1*->CASPASE+STAND+polar_region+TM+BetaPropeller->
- **GCA_001509475.1; Streptomyces regalis; counts= 2; multicellularity status: TRUE**
  - EACC1*->CASPASE+STAND+polar_region+TM+BetaPropeller->
  - TPRs->EACC1*->CASPASE+TPRs->
- **GCA_001513965.1; Streptomyces cellostaticus; counts= 2; multicellularity status: TRUE**
  - EACC1*->CASPASE+low-complexity+BetaPropeller->
  - EACC1*->CASPASE+PBPB->
- **GCA_001613935.1; Amycolatopsis regifaucium; counts= 2; multicellularity status: TRUE**
  - EACC1*->
  - EACC1*->
- **GCA_001687325.1; Streptomyces sp. PTY087I2; counts= 2; multicellularity status: TRUE**
  - EACC1*->CASPASE+??+SF1-helicase+Vsr(PDDEXK)?->??->
  - EACC1*->CASPASE+TPRs->
- **GCA_000974985.2; Streptomyces mangrovisoli; counts= 2; multicellularity status: TRUE**
  - EACC1*->CASPASE+alpha-helical+TM+TM+??+TM+TM->
  - Pkinase+TPRs->EACC1*->CASPASE+TPRs->
- **GCA_001917385.1; Actinobacteria bacterium 13_2_20CM_2_71_6; counts= 2; multicellularity status: NA**
  - EACC1*->
  - EACC1*->CASPASE+FTSK+FTSK+FTSK+FTSK->
- **GCA_001920275.1; Actinobacteria bacterium 13_1_20CM_3_71_11; counts= 2; multicellularity status: NA**
  - EACC1*->CASPASE+DUF179->SIR2+??->
  - EACC1+CASPASE+TPR_repeats+low_complexity+TM_region*->
- **GCA_001984575.1; Streptomyces sp. MP131-18; counts= 2; multicellularity status: TRUE**
  - EACC1*->CASPASE+JAB->?->MFS_1->
  - EACC1*->CASPASE+TPRs->

- **GCA_000968685.2; Streptomyces antioxidans; counts= 2; multicellularity status: TRUE**
  - EACC1*->CASPASE+Peripla_BP_5->
  - EACC1*->CASPASE+TM+??->
- **GCA_002119195.1; Streptomyces platensis; counts= 2; multicellularity status: TRUE**
  - EACC1*->CASPASE->TPRs->
  - EACC1*->CASPASE+Beta_rich_repeat->
- **GCA_002224125.1; Streptomyces sp. NBS 14/10; counts= 2; multicellularity status: TRUE**
  - EACC1*->CASPASE+TM+TM+??+TM+TM+??+TM+TM+??->
  - low-complexity+HD+Tox-RelA+TGS+ACT->Pkinase+TPRs->EACC1*->CASPASE+TPRs->
- **GCA_002242805.1; Streptomyces diastatochromogenes; counts= 2; multicellularity status: TRUE**
  - EACC1*->CASPASE+low-complexity+TM+TM+TM+TM+TM+TM+TM+TM+TM+TM+BetaPropeller->
  - EACC1*->CASPASE+STAND+polar_region+TM+BetaPropeller->TM+Acid_phosphat_B->
- **GCA_002300485.1; Streptomyces sp. Ag82_O1-15; counts= 2; multicellularity status: TRUE**
  - EACC1*->CASPASE+BetaPropeller->
  - EACC1*->CASPASE+TM+PBPI->
- **GCA_002754415.1; Micromonospora sp. CNZ299; counts= 2; multicellularity status: TRUE**
  - EACC1*->
  - TM+TM+TM+Peptidase_M48+TM+DUF4864+??->EACC1*->
- **GCA_002813405.1; Streptomyces sp. 69; counts= 2; multicellularity status: TRUE**
  - EACC1*->CASPASE+STAND->TM+BetaPropeller->TM+Acid_phosphat_B->
  - EACC1*->CASPASE+STAND+TPRs->
- **GCA_002907485.1; Streptomyces sp. ZL-24; counts= 2; multicellularity status: TRUE**
  - Beta_rich?->EACC1*->CASPASE+TPRs->??->??->
  - Pkinase+TPRs->EACC1*->CASPASE+TPRs->
- **GCA_002941025.1; Streptomyces sp. QL37; counts= 2; multicellularity status: TRUE**
  - EACC1*->CASPASE+STAND+polar_region+TM+BetaPropeller->
  - low-complexity+HD+Tox-RelA+TGS+ACT->Pkinase+TPRs->EACC1*->CASPASE+TPRs->
- **GCA_003001815.1; Actinoplanes italicus; counts= 2; multicellularity status: TRUE**
  - EACC1+CASPASE+TPR_repeats+low_complexity+TM_region*->STAND+TPRs->
  - Glyco_trans_4_4+UDPGT->EACC1*->CASPASE+PBPB+vWA->TM+PBPB->
- **GCA_003041235.1; Streptomyces sp. A244; counts= 2; multicellularity status: TRUE**
  - EACC1*->CASPASE+??->
  - EACC1*->CASPASE+TM+TM+PBPB+vWA->

■ **GCA_003148685.1; Actinoplanes xinjiangensis; counts= 2; multicellularity status: TRUE**

- EACC1*->CASPASE+PBPB+vWA->TM+PBPB->
- EACC1*->CASPASE+TM+PASTA+PASTA+PASTA->

■ **GCA_003182135.1; Nocardia neocaledoniensis; counts= 2; multicellularity status: TRUE**

- DUF742->EACC1*->CASPASE+??->
- EACC1*->CASPASE+TPRs->

■ **GCA_900091855.1; Streptomyces sp. DvalAA-14; counts= 2; multicellularity status: TRUE**

- EACC1*->CASPASE+??->
- EACC1*->CASPASE+TPRs->

■ **GCA_900091535.1; Micromonospora carbonacea; counts= 2; multicellularity status: TRUE**

- EACC1*->CASPASE+STAND+polar_region+TM+BetaPropeller->
- TM+TM+TM+Peptidase_M48+TM+DUF4864+??->EACC1*->

■ **GCA_900091505.1; Micromonospora chokoriensis; counts= 2; multicellularity status: TRUE**

- EACC1*->
- TM+TM+TM+Peptidase_M48+TM+DUF4864+??->EACC1*->

■ **GCA_900091955.1; Streptomyces sp. Ncost-T10-10d; counts= 2; multicellularity status: TRUE**

- EACC1*->CASPASE+??->
- EACC1*->CASPASE+STAND+polar_region+TM+BetaPropeller->

■ **GCA_900101585.1; Streptomyces prasinopilosus; counts= 2; multicellularity status: TRUE**

- ??+CASPASE->EACC1*->
- <-CASPASE<-?<-?||HATPase_c_2->EACC1+CASPASE(frag)*->

■ **GCA_900105395.1; Streptomyces sp. TLI_053; counts= 2; multicellularity status: TRUE**

- EACC1*->CASPASE+HSP70->
- FAD_binding_4+FAD-oxidase_C->DAGK_cat+DAGK_acc->EACC1*->CASPASE+TPRs->Clp_N+Clp_N+ClpABN-AAA->

■ **GCA_900105695.1; Streptomyces melanosporofaciens; counts= 2; multicellularity status: TRUE**

- EACC1*->CASPASE+STAND+polar_region+TM+BetaPropeller->
- TetR_N+TetR_C_6->EACC1*->CASPASE+low-complexity+TM+PBPB->

■ **GCA_900108335.1; Nonomuraea solani; counts= 2; multicellularity status: TRUE**

- ARSR-HTH+RHOD-CDC25->??->??->EACC1*->
- EACC1*->CASPASE+BetaPropeller->

■ **GCA_900111175.1; Actinokineospora terrae; counts= 2; multicellularity status: TRUE**

- EACC1*->
- EACC1*->CASPASE+??+TM+TM+TM+TM+TM+TM+TM+TM+TM->CASPASE+??+TM+TM+TM+TM+TM+TM+TM+TM->

■ **GCA_900111685.1; Nonomuraea wenchangensis; counts= 2; multicellularity status: TRUE**

- EACC1*->CASPASE+STAND+polar_region+TM+BetaPropeller->
- TM+TM+vWA+TM->TM+TM+vWA+TM->EACC1*->CASPASE+TPRs->

■ **GCA_900112845.1; Streptomyces alni; counts= 2; multicellularity status: TRUE**

- EACC1*->
- EACC1*->CASPASE+low-complexity+BetaPropeller->

■ **GCA_900187925.1; Streptomyces sp. PgraA7; counts= 2; multicellularity status: TRUE**

- EACC1*->
- EACC1*->CASPASE+STAND+polar_region+TM+BetaPropeller->

■ **GCA_900188005.1; Actinoplanes regularis; counts= 2; multicellularity status: TRUE**

- EACC1*->CASPASE+BetaPropeller->
- EACC1*->CASPASE+TPRs->

■ **GCA_900188345.1; Streptosporangium subroseum; counts= 2; multicellularity status: TRUE**

- EACC1*->
- EACC1*->CASPASE+??->

■ **GCA_000177175.2; Streptomyces sp. ACT-1; counts= 2; multicellularity status: TRUE**

- Beta_rich?->EACC1*->CASPASE+TPRs->??->??->
- EACC1*->CASPASE+TPRs->

■ **GCA_000340415.1; Amycolatopsis azurea; counts= 2; multicellularity status: TRUE**

- CASPASE+TPRs->?->EACC1*->
- EACC1*->CASPASE+TPRs->

■ **GCA_002846475.1; Saccharopolyspora spinosa; counts= 2; multicellularity status: TRUE**

- EACC1*->CASPASE+??+SF1-helicase+Vsr(PDDEXK)?->
- EACC1*->CASPASE+BetaPropeller->

■ **GCA_000024545.1; Stackebrandtia nassauensis; counts= 2; multicellularity status: TRUE**

- Clp_N+ClpABN-AAA+CASPASE->EACC1*->
- EACC1*->

■ **GCA_000245355.1; Streptomyces hygroscopicus; counts= 2; multicellularity status: TRUE**

- EACC1*->CASPASE+low-complexity+TM+PBPB->
- EACC1*->CASPASE+TM?+Phytase-like->MDMPI_N+MDMPI_C->META->

■ **GCA_000328705.1; Saccharothrix espanaensis; counts= 2; multicellularity status: TRUE**

- EACC1*->CASPASE+TM+TM->TM+PBPB->
- TM+TM+TM->CASPASE+STAND+polar_region+TM+BetaPropeller->EACC1*->

- **GCA_000400635.2; Amycolatopsis orientalis; counts= 2; multicellularity status: TRUE**
  - EACC1*->
  - EACC1*->CASPASE+DnaJ_ZnR+DnaJ_ZnR+DnaJ_C->
- **GCA_000411315.1; Streptomyces sp. HGB0020; counts= 2; multicellularity status: TRUE**
  - EACC1*->CASPASE+??+TM+??->
  - EACC1*->CASPASE+TPRs->
- **GCF_000373685.1; Streptomyces sp. PsTaAH-124; counts= 2; multicellularity status: TRUE**
  - EACC1*->
  - TPRs->EACC1*->CASPASE+TPRs->
- **GCF_000259895.1; Streptomyces sp. TOR3209; counts= 2; multicellularity status: TRUE**
  - EACC1*->CASPASE+TPRs->
  - Trypsin+HDxxH+VMAP-M0+CASPASE->EACC1*->
- **GCA_002234385.1; Amycolatopsis alba; counts= 2; multicellularity status: TRUE**
  - EACC1*->
  - EACC1*->CASPASE+TPRs->
- **GCA_003947255.1; Amycolatopsis balhimycina; counts= 2; multicellularity status: TRUE**
  - GATase->ABC-ATPase+DUF4162->cNMP_cyclase+TM+TM+TM+TM+TM+TM->EACC1*->
  - PBPB->?->?->Aminotran_5+PAS_6+HTH_22->EACC1*->
- **GCA_001270575.1; Streptomyces decoyicus; counts= 2; multicellularity status: TRUE**
  - EACC1*->CASPASE+DnaJ_ZnR+DnaJ_ZnR+DnaJ_C->
  - EACC1*->CASPASE+FGS_fold-domain->??->??->TM+TM+BaxI_1->?->NUDIX->
- **GCF_000718515.1; Micromonospora parva; counts= 2; multicellularity status: TRUE**
  - EACC1*->
  - TM+TM+TM+Peptidase_M48+TM+DUF4864+??->EACC1*->
- **GCF_000718025.1; Kitasatospora purpeofusca; counts= 2; multicellularity status: TRUE**
  - EACC1*->CASPASE+HSP70->
  - FAD_binding_4+FAD-oxidase_C->DAGK_cat+DAGK_acc->EACC1*->CASPASE+TPRs->Clp_N+Clp_N+ClpABN-AAA->
- **GCF_000719365.1; Streptomyces sp. NRRL S-1448; counts= 2; multicellularity status: TRUE**
  - EACC1*->CASPASE+TM+Beta_rich->
  - EACC1*->CASPASE+TM+PBPB+vWA->
- **GCF_000718555.1; Micromonospora chokoriensis; counts= 2; multicellularity status: TRUE**
  - EACC1*->
  - TM+TM+TM+Peptidase_M48+TM+DUF4864+??->EACC1*->

■ **GCF_000718675.1; Streptomyces rimosus; counts= 2; multicellularity status: TRUE**

- EACC1*->CASPASE+??+SF1-helicase+Vsr(PDDEXK)?->??->
- EACC1*->CASPASE+TM?+Phytase-like->

■ **GCF_000718625.1; Streptomyces lavenduligriseus; counts= 2; multicellularity status: TRUE**

- EACC1*->CASPASE+??+SF1-helicase+Vsr(PDDEXK)?->
- EACC1*->CASPASE+TM?+BetaPropeller->

■ **GCF_000720175.1; Streptomyces sp. NRRL S-337; counts= 2; multicellularity status: TRUE**

- EACC1*->CASPASE+TM?+BetaPropeller->
- EACC1*->CASPASE+TM+Beta_rich->

■ **GCF_000721625.1; Streptomyces sp. NRRL S-384; counts= 2; multicellularity status: TRUE**

- EACC1*->CASPASE+alpha-helical+TM+TM+??+TM+TM->
- polar_lead?+TM+TM+TM+polar_tail?+low-complexity->??->EACC1*->CASPASE+TPRs->

■ **GCF_000719555.1; Streptomyces sp. NRRL F-5727; counts= 2; multicellularity status: TRUE**

- EACC1*->CASPASE+STAND+??+TPRs+??->
- HATPase_c_2->EACC1*->CASPASE+??+SF1-helicase+Vsr(PDDEXK)?->

■ **GCF_000725475.1; Streptomyces durhamensis; counts= 2; multicellularity status: TRUE**

- EACC1*->CASPASE+??+SF1-helicase+Vsr(PDDEXK)?->
- EACC1*->CASPASE+TM+TM+bPH_domain?->

■ **GCF_000717335.1; Amycolatopsis jejuensis; counts= 2; multicellularity status: TRUE**

- EACC1->CASPASE+TPRs->?->EACC1*->
- EACC1*->CASPASE+TPRs->?->EACC1->

■ **GCF_000716885.1; Kitasatospora aureofaciens; counts= 2; multicellularity status: TRUE**

- EACC1*->
- EACC1*->CASPASE+STAND+polar_region+TM+BetaPropeller->

■ **GCF_000720255.1; Streptomyces griseus; counts= 2; multicellularity status: TRUE**

- ??->EACC1*->CASPASE+TM+TM+PBPB+vWA->
- EACC1*->CASPASE+Trypsin+STAND+??+TM->

■ **GCA_000590515.1; Streptomyces sp. PRh5; counts= 2; multicellularity status: TRUE**

- EACC1*->
- EACC1*->CASPASE+STAND+TPRs->

■ **GCA_000732925.1; Amycolatopsis japonica; counts= 2; multicellularity status: TRUE**

- EACC1*->CASPASE+??->
- EACC1*->CASPASE+TPRs->

■ **GCF_000494755.1; Actinoplanes friuliensis; counts= 2; multicellularity status: TRUE**

- EACC1*->
- EACC1*->CASPASE+DUF179->SIR2+??->??->

■ **GCA_000829715.2; Streptomyces sp. NBRC 110027; counts= 2; multicellularity status: TRUE**

- EACC1*->CASPASE->TPRs->
- EACC1*->CASPASE+Beta_rich_repeat->

■ **GCF_000308415.1; Nocardia asiatica; counts= 2; multicellularity status: TRUE**

- EACC1*->CASPASE+STAND->BetaPropeller->
- EACC1*->CASPASE+STAND+BetaPropeller+BetaPropeller->

■ **GCA_000695625.1; Amycolatopsis rifamycinica; counts= 2; multicellularity status: TRUE**

- GATase->ABC-ATPase+DUF4162->cNMP_cyclase+TM+TM+TM+TM+TM+TM->EACC1*->
- PBPB->?->?->Aminotran_5+PAS_6+HTH_22->EACC1*->

■ **GCA_900103775.1; Allokutzneria albata; counts= 2; multicellularity status: TRUE**

- EACC1*->
- EACC1*->CASPASE+DnaJ_ZnR+DnaJ_ZnR+DnaJ_C->

■ **GCF_000331005.1; Streptomyces; counts= 2; multicellularity status: TRUE**

- EACC1*->CASPASE+TM+TM+bPH_domain+TM+TM+bPH_domain+TM+TM->
- HTH+CASPASE->EACC1*->

■ **GCF_000962825.1; Actinoplanes rectilineatus; counts= 2; multicellularity status: TRUE**

- EACC1*->||<-CASPASE+STAND+TM+polar_region+TM+BetaPropeller
- EACC1*->CASPASE+TPRs->

■ **GCA_000982955.1; Micromonospora sp. HK10; counts= 2; multicellularity status: TRUE**

- EACC1*->
- TM+TM+TM+Peptidase_M48+TM+DUF4864+??->EACC1*->

■ **GCF_001083785.1; Nonomuraea sp. SBT364; counts= 2; multicellularity status: TRUE**

- EACC1*->
- EACC1*->CASPASE+low-complexity+BetaPropeller->

■ **GCA_001187435.1; Streptomyces caatingaensis; counts= 2; multicellularity status: TRUE**

- EACC1*->CASPASE+STAND+polar_region+TM+BetaPropeller->
- EACC1*->CASPASE+TPRs->

■ **GCF_001650215.1; Streptomyces himastatinicus; counts= 2; multicellularity status: TRUE**

- EACC1*->CASPASE+low-complexity+TM+PBPB->
- EACC1*->CASPASE+TPRs->Clp_N+Clp_N+Clp_N+Clp_N+ClpABN-AAA->

- **GCF_000620785.1; Pseudonocardia acaciae; counts= 2; multicellularity status: TRUE**
  - EACC1*->CASPASE+STAND+polar_region+TM+BetaPropeller->
  - EACC1*->CASPASE+TM+TM+TM+TM->
- **GCF_000719415.1; Streptomyces sp. NRRL B-5680; counts= 2; multicellularity status: TRUE**
  - EACC1*->
  - EACC1*->CASPASE+??->
- **GCF_000935125.1; Streptomyces natalensis; counts= 2; multicellularity status: TRUE**
  - EACC1*->CASPASE+low-complexity+BetaPropeller->
  - PAS+GAF+PP2C+HATPase_c_2->EACC1*->CASPASE+TM+Beta_rich->
- **GCF_000744785.1; Kitasatospora azatica; counts= 2; multicellularity status: TRUE**
  - cNMP_cyclase+EACC1*->CASPASE+TPRs->
  - EACC1*->CASPASE+DnaJ_ZnR+DnaJ_C+DnaJ_C+TM+TM->
- **GCA_000816025.1; Streptomyces sp. 769; counts= 2; multicellularity status: TRUE**
  - low-complexity+HD+Tox-RelA+TGS+ACT->Pkinase+TPRs->EACC1*->CASPASE+TPRs->
  - polar_lead?+TM+TM+TM+polar_tail?+low-complexity->??->EACC1*->CASPASE+TPRs->
- **GCF_000721495.1; Streptomyces sp. NRRL F-5123; counts= 2; multicellularity status: TRUE**
  - EACC1*->CASPASE+BetaPropeller->
  - EACC1*->CASPASE+STAND+TPRs->
- **GCF_000935185.3; Streptomyces albulus; counts= 2; multicellularity status: TRUE**
  - EACC1*->CASPASE+PBPB->
  - EACC1*->CASPASE+TM+TM+bPH_domain?->
- **GCA_001280065.1; Streptomyces sp. NRRL B-1140; counts= 2; multicellularity status: TRUE**
  - ??->EACC1*->CASPASE+TM+TM+PBPB+vWA->
  - EACC1*->CASPASE->TPRs->
- **GCA_001279655.1; Streptomyces sp. WM6372; counts= 2; multicellularity status: TRUE**
  - EACC1*->CASPASE+??->
  - low-complexity+HD+Tox-RelA+TGS+ACT->Pkinase+TPRs->EACC1*->CASPASE+TPRs->
- **GCF_001279545.1; Streptomyces sp. NRRL B-3648; counts= 2; multicellularity status: TRUE**
  - EACC1*->CASPASE+low-complexity+BetaPropeller->
  - EACC1*->CASPASE+STAND+polar_region+TM+BetaPropeller->
- **GCA_001294335.1; Streptomyces chattanoogensis; counts= 2; multicellularity status: TRUE**
  - EACC1*->CASPASE+TM?+BetaPropeller->
  - TPRs->EACC1*->CASPASE+TPRs->

■ **GCF_001298575.1; Actinobacteria bacterium OK074; counts= 2; multicellularity status: NA**

- EACC1*->
- EACC1*->CASPASE+low-complexity+BetaPropeller->

■ **GCF_001418475.1; Streptomyces ossamyceticus; counts= 2; multicellularity status: TRUE**

- EACC1*->
- EACC1*->CASPASE->TPRs->

■ **GCF_001419705.1; Streptomyces prasinus; counts= 2; multicellularity status: TRUE**

- ??->EACC1*->CASPASE+TPRs->
- EACC1*->CASPASE+??->

■ **GCA_001447075.1; Streptomyces hygroscopicus; counts= 2; multicellularity status: TRUE**

- EACC1*->CASPASE+low-complexity+TM+PBPB->
- EACC1*->CASPASE+TM?+Phytase-like->MDMPI_N+MDMPI_C->META->

■ **GCA_001509775.1; Streptomyces; counts= 2; multicellularity status: TRUE**

- EACC1*->
- TetR_N+TetR_C_6->EACC1*->

■ **GCA_002251155.1; Streptomyces; counts= 2; multicellularity status: TRUE**

- ??->??->EACC1*->CASPASE+??->
- AMP-binding+AMP-binding_C->AMP-binding+AMP-binding_C->EACC1*->CASPASE+STAND+polar_region+TM+BetaPropeller->

■ **GCA_001611795.1; Streptomyces sp. S10(2016); counts= 2; multicellularity status: TRUE**

- EACC1*->CASPASE+RE_Eco29kI+??->
- TM+TM+TM+PP2C->??->EACC1*->CASPASE+TM+TM+PBPB+vWA->

■ **GCA_001590865.1; Streptomyces sp. NBRC 110611; counts= 2; multicellularity status: TRUE**

- *
- EACC1*->CASPASE+TPRs->

■ **GCF_001700515.1; Streptomyces lushanensis; counts= 2; multicellularity status: TRUE**

- EACC1*->
- Pkinase+TPRs->EACC1*->CASPASE+TPRs->

■ **GCA_001653515.1; Streptomyces sp. ERV7; counts= 2; multicellularity status: TRUE**

- EACC1*->CASPASE+BetaPropeller->||<-DUF664
- low-complexity+HD+Tox-RelA+TGS+ACT->Pkinase+TPRs->EACC1*->CASPASE+TPRs->

■ **GCA_001542325.1; Micromonospora rifamycinica; counts= 2; multicellularity status: TRUE**

- EACC1*->CASPASE+TM+TM->PBPB->
- TM+TM+TM+Peptidase_M48+TM+DUF4864+??->EACC1*->

■ **GCF_001613065.1; Nocardia miyunensis; counts= 2; multicellularity status: TRUE**

- EACC1*->
- EACC1*->CASPASE+STAND+polar_region+TM+BetaPropeller->

■ **GCF_001613145.1; Nocardia jejuensis; counts= 2; multicellularity status: TRUE**

- EACC1*->CASPASE+STAND+BetaPropeller->
- EACC1*->CASPASE+TPRs->

■ **GCF_001612785.1; Nocardia beijingensis; counts= 2; multicellularity status: TRUE**

- EACC1*->CASPASE+STAND+polar_region+TM+BetaPropeller->
- Pkinase+TM+LppX_LprAFG->CASPASE+TPRs->EACC1*->

■ **GCF_001653075.1; Planomonospora sphaerica; counts= 2; multicellularity status: TRUE**

- EACC1*->CASPASE+SAD+HNH->
- EACC1*->CASPASE+STAND+polar_region+TM+BetaPropeller->

■ **GCF_001696485.1; Planobispora rosea; counts= 2; multicellularity status: TRUE**

- EACC1*->
- EACC1*->CASPASE+TM+TM+TM+TM+TM+TM+BetaPropeller->

■ **GCF_001746285.1; Streptomyces sp. EN16; counts= 2; multicellularity status: TRUE**

- EACC1*->CASPASE+Beta_rich_repeat->??+SUKH->
- EACC1*->CASPASE+STAND+??+TPRs+??->

■ **GCF_001746365.1; Streptomyces sp. LUP30; counts= 2; multicellularity status: TRUE**

- EACC1*->CASPASE+??+SF1-helicase+Vsr(PDDEXK)?->
- EACC1*->CASPASE+TPRs->

■ **GCA_900079405.3; Streptomyces sp. F-1; counts= 2; multicellularity status: TRUE**

- EACC1*->CASPASE+STAND+polar_region+TM+BetaPropeller->
- EACC1*->CASPASE+STAND+polar_region+TM+BetaPropeller->

■ **GCF_900142575.1; Streptomyces paucisporeus; counts= 2; multicellularity status: TRUE**

- EACC1*->CASPASE+HSP70->
- EACC1*->CASPASE+STAND+polar_region+TM+BetaPropeller->

■ **GCA_001905505.1; Streptomyces sp. CB02058; counts= 2; multicellularity status: TRUE**

- EACC1*->CASPASE+??->
- EACC1*->CASPASE+STAND+??+TPRs->

■ **GCA_001905445.1; Streptomyces sp. CB00455; counts= 2; multicellularity status: TRUE**

- HATPase_c_2->EACC1*->CASPASE+??+SF1-helicase+Vsr(PDDEXK)?->
- low-complexity+HD+Tox-RelA+TGS+ACT->Pkinase+TPRs->EACC1*->CASPASE+TPRs->

- **GCF_001906585.1; Streptomyces kebangsaanensis; counts= 2; multicellularity status: TRUE**
  - ??->EACC1*->CASPASE+TM+TM+PBPB+vWA->
  - <-CASPASE+HATPase_c_4+STAND+TPRs||EACC1*->
- **GCA_001921215.1; Actinophytocola xinjiangensis; counts= 2; multicellularity status: TRUE**
  - Beta_rich?->EACC1*->CASPASE+TPRs->??->??->
  - EACC1*->CASPASE+TM+TM->TM+PBPB->
- **GCF_001941275.1; Streptomyces acidiscabies; counts= 2; multicellularity status: TRUE**
  - EACC1*->CASPASE+TM+TM+PBPB+vWA->
  - EACC1*->CASPASE+TPRs->
- **GCA_001984155.1; Actinosynnema sp. ALI-1.44; counts= 2; multicellularity status: TRUE**
  - EACC1*->
  - PALP->Trp_syntA->TM+LGT->EACC1*->
- **GCF_001866665.1; Streptomyces sp. MUSC 1; counts= 2; multicellularity status: TRUE**
  - EACC1*->
  - EACC1*->CASPASE+STAND+polar_region+TM+BetaPropeller->
- **GCA_001983975.1; Streptomyces autolyticus; counts= 2; multicellularity status: TRUE**
  - EACC1*->CASPASE+TM+??->
  - TetR_N+TetR_C_6->EACC1*->CASPASE+low-complexity+TM+PBPB->
- **GCF_001298565.1; Actinobacteria bacterium OK006; counts= 2; multicellularity status: NA**
  - EACC1*->CASPASE+TPRs->
  - low_complexity+EACC1*->CASPASE+STAND+polar_region+TM+BetaPropeller->
- **GCA_002082195.1; Streptomyces gilvosporeus; counts= 2; multicellularity status: TRUE**
  - EACC1*->CASPASE+low-complexity+TM+PBPB->
  - PAS+GAF+PP2C+HATPase_c_2->EACC1*->CASPASE+TM+Beta_rich->
- **GCA_002090335.1; Streptomyces sp. MOE7; counts= 2; multicellularity status: TRUE**
  - Glyoxalase->EACC1*->CASPASE+DnaJ_ZnR+DnaJ_ZnR+DnaJ_C->
  - polar_lead?+TM+TM+TM+polar_tail?+low-complexity->??->EACC1*->CASPASE+TPRs->
- **GCA_002114215.1; Streptomyces sp. 13-12-16; counts= 2; multicellularity status: TRUE**
  - ??->EACC1*->CASPASE+TPRs->
  - EACC1*->CASPASE+TPRs->
- **GCA_002128465.1; Streptomyces pharetrae; counts= 2; multicellularity status: TRUE**
  - EACC1*->?->AB_hydrolase->
  - EACC1*->CASPASE(fragment)->CASPASE+STAND+polar_region+TM+BetaPropeller->

- **GCF_002155905.1; Streptomyces tricolor; counts= 2; multicellularity status: TRUE**
  - ??->??->EACC1*->CASPASE+??->
  - AMP-binding+AMP-binding_C->AMP-binding+AMP-binding_C->EACC1*->
- **GCF_002154505.1; Streptomyces carpinensis; counts= 2; multicellularity status: TRUE**
  - ??->EACC1*->
  - EACC1*->CASPASE+TM+TM+bPH_domain?->
- **GCF_002155965.1; Allokutzneria sp. NRRL B-24872; counts= 2; multicellularity status: TRUE**
  - EACC1*->
  - EACC1*->CASPASE+TPRs->
- **GCA_002210435.1; Micromonospora wenchangensis; counts= 2; multicellularity status: TRUE**
  - EACC1*->CASPASE+STAND+TPRs->||<-TM+TM+TM
  - TM+TM+TM+Peptidase_M48+TM+DUF4864+??->EACC1*->
- **GCA_002300315.1; Streptomyces; counts= 2; multicellularity status: TRUE**
  - EACC1*->
  - EACC1*->CASPASE+TM+PBPB+vWA->
- **GCA_900090275.1; Micromonospora zamorensis; counts= 2; multicellularity status: TRUE**
  - EACC1*->
  - TM+TM+TM+Peptidase_M48+TM+DUF4864+??->EACC1*->
- **GCA_900111005.1; Lentzea albida; counts= 2; multicellularity status: TRUE**
  - polar_lead?+TM+TM+TM+polar_tail?+low-complexity->??->EACC1*->CASPASE+TPRs->
  - TM+TM+BPD_transp_1->OppC_N+TM+TM+BPD_transp_1->ABC-ATPase+ABC-ATPase->PBPB->TM+Beta_rich+TM+Beta_rich+TM+TM->EACC1+CASPASE+TPR_repeats+low_complexity+TM_region*->
- **GCA_900107455.1; Asanoa ishikariensis; counts= 2; multicellularity status: TRUE**
  - EACC1*->CASPASE+PRTase->
  - TPRs+CASPASE->?->?->EACC1*->?->CASPASE+TPRs->
- **GCF_900111685.1; Nonomuraea wenchangensis; counts= 2; multicellularity status: TRUE**
  - EACC1*->CASPASE+STAND+polar_region+TM+BetaPropeller->
  - TM+TM+vWA+TM->TM+TM+vWA+TM->EACC1*->CASPASE+TPRs->
- **GCA_900091525.1; Micromonospora matsumotoense; counts= 2; multicellularity status: TRUE**
  - EACC1*->CASPASE+STAND+TPRs->||<-?<-?<-?<-TM+TM+TM
  - TM+TM+TM+Peptidase_M48+TM+DUF4864+??->EACC1*->
- **GCA_900091575.1; Micromonospora saelicesensis; counts= 2; multicellularity status: TRUE**
  - EACC1*->
  - TM+TM+TM+Peptidase_M48+TM+DUF4864+??->EACC1*->

- **GCA_900101685.1; Alloactinosynnema iranicum; counts= 2; multicellularity status: TRUE**
  - EACC1*->
  - EACC1*->CASPASE+TPRs->
- **GCA_003053965.1; Saccharopolyspora; counts= 2; multicellularity status: TRUE**
  - EACC1*->CASPASE+BetaPropeller->
  - EACC1*->CASPASE+FTSK+FTSK+FTSK+FTSK->
- **GCA_900115955.1; Lentzea waywayandensis; counts= 2; multicellularity status: TRUE**
  - EACC1*->CASPASE+STAND+polar_region+TM+BetaPropeller->
  - TM+TM+BPD_transp_1->OppC_N+TM+TM+BPD_transp_1->ABC-ATPase+ABC-ATPase->PBPB->TM+Beta_rich+TM+Beta_rich+TM+TM->EACC1+CASPASE+TPR_repeats+low_complexity+TM_region*->
- **GCA_900091995.1; Streptomyces sp. MnatMP-M17; counts= 2; multicellularity status: TRUE**
  - EACC1*->
  - EACC1*->CASPASE+TM+TM+bPH_domain?->
- **GCF_900090095.1; Streptomyces sp. SceaMP-e96; counts= 2; multicellularity status: TRUE**
  - EACC1*->CASPASE+Beta_rich_repeat->
  - Phage_int_SAM_3+Phage_integrase->EACC1*->CASPASE+TPRs->
- **GCF_002242805.1; Streptomyces diastatochromogenes; counts= 2; multicellularity status: TRUE**
  - EACC1*->CASPASE+low-complexity+TM+TM+TM+TM+TM+TM+TM+TM+TM+TM+BetaPropeller->
  - EACC1*->CASPASE+STAND+polar_region+TM+BetaPropeller->TM+Acid_phosphat_B->
- **GCA_900215205.1; Actinoplanes atraurantiacus; counts= 2; multicellularity status: TRUE**
  - EACC1*->CASPASE+Pentapeptide_repeats->
  - EACC1*->CASPASE+TPRs->
- **GCA_003173635.1; Streptomyces; counts= 2; multicellularity status: TRUE**
  - EACC1*->CASPASE+??+SF1-helicase+Vsr(PDDEXK)?->
  - EACC1*->CASPASE+TPRs??+GHKL_ATPase+TPRs??->
- **GCA_002754435.1; Streptomyces sp. 76; counts= 2; multicellularity status: TRUE**
  - EACC1*->CASPASE+??->
  - Glyoxalase->EACC1*->CASPASE+DnaJ_ZnR+DnaJ_ZnR+DnaJ_C->
- **GCF_002754535.1; Streptomyces sp. 61; counts= 2; multicellularity status: TRUE**
  - EACC1*->CASPASE+GAF+PAS+GGDEF->
  - low-complexity+HD+Tox-RelA+TGS+ACT->Pkinase+TPRs->EACC1*->CASPASE+TPRs->
- **GCA_002754615.1; Streptomyces sp. 1; counts= 2; multicellularity status: TRUE**
  - Pkinase+TPRs->EACC1*->CASPASE+TPRs->
  - polar_lead?+TM+TM+TM+polar_tail?+low-complexity->??->EACC1*->CASPASE+TPRs->

■ **GCF_002754555.1; Streptomyces sp. 94; counts= 2; multicellularity status: TRUE**

- EACC1*->CASPASE+TM+??->
- EACC1*->CASPASE+TPRs+LysM+PG-binding->

■ **GCA_002803155.1; Streptomyces sp. CB02959; counts= 2; multicellularity status: TRUE**

- low-complexity+HD+Tox-RelA+TGS+ACT->Pkinase+TPRs->EACC1*->CASPASE+TPRs->
- low_complexity+EACC1*->CASPASE+TM+TM+bPH_domain?->

■ **GCF_002910875.1; Streptomyces sp. SM13; counts= 2; multicellularity status: TRUE**

- EACC1*->CASPASE+??+SF1-helicase+Vsr(PDDEXK)?->
- EACC1*->CASPASE+STAND+polar_region+TM+BetaPropeller->

■ **GCF_002954775.1; Streptomyces geranii; counts= 2; multicellularity status: TRUE**

- EACC1*->CASPASE+STAND+??+TPRs->
- TPR_region+EACC1*->

■ **GCA_003002065.1; Pseudosporangium ferrugineum; counts= 2; multicellularity status: TRUE**

- EACC1*->CASPASE+DUF179->SIR2+??->??->
- EACC1*->CASPASE+TPRs->

■ **GCF_003001815.1; Actinoplanes italicus; counts= 2; multicellularity status: TRUE**

- EACC1+CASPASE+TPR_repeats+low_complexity+TM_region*->STAND+TPRs->
- Glyco_trans_4_4+UDPGT->EACC1*->CASPASE+PBPB+vWA->TM+PBPB->

■ **GCA_003172955.1; Micromonospora sp. 5R2A7; counts= 2; multicellularity status: TRUE**

- EACC1*->
- TM+TM+TM+Peptidase_M48+TM+DUF4864+??->EACC1*->

■ **GCA_000237145.1; Actinoplanes sp. SE50/110; counts= 1; multicellularity status: TRUE**

- EACC1*->CASPASE+JAB->

■ **GCA_000525635.1; Kutzneria albida DSM 43870; counts= 1; multicellularity status: TRUE**

- polar_lead?+TM+TM+TM+polar_tail?+low-complexity->??->EACC1*->CASPASE+TPRs->?->CASPASE+??+TPRs?->

■ **GCA_001027185.1; Streptomyces incarnatus; counts= 1; multicellularity status: TRUE**

- EACC1*->CASPASE+TPRs->

■ **GCA_001747425.1; Actinoalloteichus hymeniacidonis; counts= 1; multicellularity status: TRUE**

- EACC1*->CASPASE+??+SF1-helicase+Vsr(PDDEXK)?->

■ **GCA_001865855.1; Nocardia seriolae; counts= 1; multicellularity status: TRUE**

- EACC1*->CASPASE+STAND+TPRs->

■ **GCA_001941845.1; Streptomyces sp. TN58; counts= 1; multicellularity status: TRUE**

  - EACC1*->CASPASE+RE_Eco29kI+??->

- **GCA_002009175.1; Streptomyces niveus; counts= 1; multicellularity status: TRUE**
  - EACC1*->CASPASE+??->
- **GCA_000058485.1; Frankia alni ACN14a; counts= 1; multicellularity status: TRUE**
  - FHA+??+low-complexity+FHA+ABC-ATPase+TM->||<-?||EACC1+EAD1*->||<-CASPASE+STAND+polar_region+TM+BetaPropeller
- **GCA_000284035.1; Nocardia cyriacigeorgica GUH-2; counts= 1; multicellularity status: TRUE**
  - RNR_N+Ribonuc_red_lgN+Ribonuc_red_lgC->EACC1*->CASPASE+FTSK+FTSK+FTSK+FTSK->
- **GCA_000826545.1; Kibdelosporangium sp. MJ126-NF4; counts= 1; multicellularity status: TRUE**
  - PALP->Trp_syntA->TM+LGT->EACC1*->
- **GCA_000170715.1; Beggiatoa sp. PS; counts= 1; multicellularity status: TRUE**
  - EACC1*->
- **GCA_000154945.1; Streptomyces pristinaespiralis ATCC 25486; counts= 1; multicellularity status: TRUE**
  - low-complexity+HD+Tox-RelA+TGS+ACT->Pkinase+TPRs->EACC1*->CASPASE+TPRs->
- **GCA_000158815.1; Micromonospora sp. ATCC 39149; counts= 1; multicellularity status: TRUE**
  - TM+TM+TM+Peptidase_M48+TM+DUF4864+??->EACC1*->
- **GCA_000156455.1; Streptomyces roseosporus NRRL 15998; counts= 1; multicellularity status: TRUE**
  - EACC1*->CASPASE+Beta_rich_repeat->
- **GCA_000162775.1; Streptomyces sp. e14; counts= 1; multicellularity status: TRUE**
  - TPRs->EACC1*->CASPASE+TPRs->
- **GCA_000158975.1; Streptomyces griseoflavus Tu4000; counts= 1; multicellularity status: TRUE**
  - MglB->?->sGTP-MglA->?->Beta_rich?->EACC1*->CASPASE+TPRs->??->??->
- **GCA_000239075.2; Streptomyces sp. W007; counts= 1; multicellularity status: TRUE**
  - EACC1*->CASPASE+PBPB->
- **GCA_000244955.1; Saccharomonospora marina XMU15; counts= 1; multicellularity status: TRUE**
  - EACC1*->CASPASE+TM+TM+TM+TM->
- **GCA_000340335.2; Streptomyces bottropensis ATCC 25435; counts= 1; multicellularity status: TRUE**
  - EACC1*->CASPASE+TPRs->
- **GCA_000497445.1; Streptomyces roseochromogenus subsp. oscitans DS 12.976; counts= 1; multicellularity status: TRUE**
  - EACC1*->CASPASE+BetaPropeller->||<-?<-?||HATPase_c_2->
- **GCA_000498935.1; Streptomyces sp. HCCB10043; counts= 1; multicellularity status: TRUE**

- EACC1*->CASPASE+Beta_rich_repeat->

■ **GCA_000564855.1; Actinokineospora spheciospongiae; counts= 1; multicellularity status: TRUE**

- EACC1*->CASPASE+??+ART->

■ **GCA_000707925.1; Streptomyces rimosus R6-500; counts= 1; multicellularity status: TRUE**

- EACC1*->CASPASE+??+SF1-helicase+Vsr(PDDEXK)?->REase+STAND->

■ **GCA_000811985.1; Nocardia vulneris; counts= 1; multicellularity status: TRUE**

- DUF2510(W3)+BicD+DUF4041+T5orf172->EACC1*->

■ **GCA_000817655.1; Streptomyces sp. RSD-27; counts= 1; multicellularity status: TRUE**

- EACC1*->CASPASE+STAND(fragment)->AAA(fragment-helical-region)+wHTH+TM+BetaPropeller->

■ **GCA_000932205.1; Streptomyces sp. MBRL 601; counts= 1; multicellularity status: TRUE**

- EACC1*->CASPASE+STAND+TM+polar_region+TM+BetaPropeller->

■ **GCA_000966955.1; Streptomyces sp. NRRL S-495; counts= 1; multicellularity status: TRUE**

- FAD_binding_4+FAD-oxidase_C->DAGK_cat+DAGK_acc->EACC1*->

■ **GCA_000967025.1; Streptomyces sp. NRRL B-1568; counts= 1; multicellularity status: TRUE**

- EACC1*->CASPASE+STAND+polar_region+TM+BetaPropeller->

■ **GCA_001279885.1; Streptomyces sp. XY593; counts= 1; multicellularity status: TRUE**

- EACC1*->CASPASE+??+SF1-helicase+Vsr(PDDEXK)?->

■ **GCA_001279805.1; Streptomyces sp. XY431; counts= 1; multicellularity status: TRUE**

- EACC1*->

■ **GCA_001280005.1; Streptomyces sp. NRRL WC-3618; counts= 1; multicellularity status: TRUE**

- EACC1*->

■ **GCA_001426245.1; Phycicoccus sp. Root101; counts= 1; multicellularity status: NA**

- EACC1*->CASPASE+??+TM+TM+TM+TM+TM+TM+TM+TM+TM+TM+TM->

■ **GCA_001426405.1; Streptomyces sp. Root1304; counts= 1; multicellularity status: TRUE**

- EACC1*->CASPASE+STAND+polar_region+TM+BetaPropeller->||<-MFS_1

■ **GCA_001456965.1; Rhodococcus enclensis; counts= 1; multicellularity status: TRUE**

- EACC1*->CASPASE+??->

■ **GCA_001482415.1; Streptomyces silvensis; counts= 1; multicellularity status: TRUE**

- EACC1*->CASPASE+Type_III_YscG+VHL->

■ **GCA_001509495.1; Actinoplanes awajinensis subsp. mycoplanecinus; counts= 1; multicellularity status: TRUE**

- EACC1*->CASPASE+??->

■ **GCA_001541145.1; Streptomyces albus subsp. albus; counts= 1; multicellularity status: TRUE**

- EACC1*->

■ **GCA_001666935.1; Mycobacterium sp. E188; counts= 1; multicellularity status: FALSE**

- EACC1*->TM+TM->

■ **GCA_001746415.1; Streptomyces agglomeratus; counts= 1; multicellularity status: TRUE**

- EACC1*->CASPASE+BetaPropeller->

■ **GCA_001188955.3; Kitasatospora aureofaciens; counts= 1; multicellularity status: TRUE**

- EACC1*->

■ **GCA_001866675.1; Streptomyces sp. MUSC 14; counts= 1; multicellularity status: TRUE**

- EACC1*->CASPASE+STAND+polar_region+TM+BetaPropeller->

■ **GCA_001905645.1; Streptomyces sp. CB02115; counts= 1; multicellularity status: TRUE**

- EACC1*->CASPASE+Beta_rich_repeat->??+SUKH->

■ **GCA_001905845.1; Streptomyces sp. TSRI0107; counts= 1; multicellularity status: TRUE**

- EACC1*->

■ **GCA_001905365.1; Streptomyces sp. CB02488; counts= 1; multicellularity status: TRUE**

- MglB->DUF742->sGTP-MglA->?->?->EACC1*->

■ **GCA_001920315.1; Catenulispora sp. 13_1_20CM_3_70_7; counts= 1; multicellularity status: TRUE**

- EACC1*->CASPASE+PBPB->

■ **GCA_001942255.1; Nocardiopsis sp. CNR-923; counts= 1; multicellularity status: TRUE**

- EACC1*->

■ **GCA_002027195.1; Streptomyces sp. GKU 895; counts= 1; multicellularity status: TRUE**

- EACC1*->

■ **GCA_002104855.1; Streptomyces sp. 4F; counts= 1; multicellularity status: TRUE**

- low-complexity+HD+Tox-RelA+TGS+ACT->Pkinase+TPRs->EACC1*->CASPASE+TPRs->

■ **GCA_002276245.1; Streptomyces sp. alain-838; counts= 1; multicellularity status: TRUE**

- low-complexity+HD+Tox-RelA+TGS+ACT->Pkinase+TPRs->EACC1*->CASPASE+TPRs->

■ **GCA_002564045.1; Amycolatopsis sulphurea; counts= 1; multicellularity status: TRUE**

- GATase->ABC-ATPase+DUF4162->cNMP_cyclase+TM+TM+TM+TM+TM+TM->EACC1*->

■ **GCA_002754455.1; Streptomyces sp. 2233.5; counts= 1; multicellularity status: TRUE**

- Phage_int_SAM_3+Phage_integrase->EACC1*->CASPASE+TPRs->

■ **GCA_002754595.1; Streptomyces sp. 93; counts= 1; multicellularity status: TRUE**

- EACC1*->CASPASE+STAND+polar_region+TM+BetaPropeller->

■ **GCA_002754535.1; Streptomyces sp. 61; counts= 1; multicellularity status: TRUE**

- low-complexity+HD+Tox-RelA+TGS+ACT->Pkinase+TPRs->EACC1*->CASPASE+TPRs->

■ **GCA_002784125.1; Flexibacter sp. CG_4_10_14_3_um_filter_32_15; counts= 1; multicellularity status: TRUE**

- EACC1*->CASPASE+??->

■ **GCA_001896795.2; Thermomonospora sp. CIF 1; counts= 1; multicellularity status: TRUE**

- EACC1*->CASPASE+??+NUDIX->

■ **GCA_002911015.1; Streptomyces populi; counts= 1; multicellularity status: TRUE**

- EACC1*->CASPASE(fragment)->

■ **GCA_002846315.1; Streptomyces sp. 73; counts= 1; multicellularity status: TRUE**

- EACC1*->CASPASE+STAND+polar_region+TM+BetaPropeller->

■ **GCA_002846625.1; Streptomyces sp. OK885; counts= 1; multicellularity status: TRUE**

- EACC1*->CASPASE+TPRs->Pkinase+??->

■ **GCA_002855875.1; Klebsiella pneumoniae; counts= 1; multicellularity status: FALSE**

- EACC1*->

■ **GCA_002920575.1; Streptomyces sp. Ru73; counts= 1; multicellularity status: TRUE**

- EACC1*->

■ **GCA_002920535.1; Streptomyces sp. Ru71; counts= 1; multicellularity status: TRUE**

- EACC1*->CASPASE+TPRs->Clp_N+Clp_N+ClpABN-AAA->

■ **GCA_003001935.1; Nonomuraea fuscirosea; counts= 1; multicellularity status: TRUE**

- EACC1*->CASPASE+PBPB->

■ **GCA_003002955.1; Glycomyces artemisiae; counts= 1; multicellularity status: TRUE**

- EACC1*->CASPASE+TPRs->

■ **GCA_900067225.1; Candidatus Frankia californiensis; counts= 1; multicellularity status: TRUE**

- EACC1*->CASPASE+SF1-helicase->

■ **GCA_900091795.1; Streptomyces sp. Termitarium-T10T-6; counts= 1; multicellularity status: TRUE**

- low_complexity+EACC1*->CASPASE+GAF+PAS->

■ **GCA_900091835.1; Streptomyces sp. PpalLS-921; counts= 1; multicellularity status: TRUE**

- EACC1*->CASPASE+STAND+PBPB->

■ **GCA_900091965.1; Streptomyces sp. Ncost-T6T-2b; counts= 1; multicellularity status: TRUE**

- EACC1*->

■ **GCA_900092015.1; Streptomyces sp. MnatMP-M27; counts= 1; multicellularity status: TRUE**

- TetR_N+TetR_C_6->EACC1*->CASPASE+TPRs->

■ **GCA_900090165.1; Streptomyces sp. AmelKG-E11A; counts= 1; multicellularity status: TRUE**

- EACC1*->CASPASE+TPRs->

■ **GCA_900101745.1; Glycomyces harbinensis; counts= 1; multicellularity status: TRUE**

- EACC1*->CASPASE+TPRs->

■ **GCA_900100325.1; Frankineae bacterium MT45; counts= 1; multicellularity status: TRUE**

- EACC1*->CASPASE+low-complexity+DUF4190(has2TM)->

■ **GCA_900103585.1; Streptomyces sp. cf386; counts= 1; multicellularity status: TRUE**

- EACC1*->CASPASE+TPRs->

■ **GCA_900099985.1; Thermostaphylospora chromogena; counts= 1; multicellularity status: TRUE**

- EACC1*->CASPASE+Astro_capsid_N->

■ **GCA_900106995.1; Saccharopolyspora shandongensis; counts= 1; multicellularity status: TRUE**

- EACC1*->CASPASE+STAND+polar_region+TM+BetaPropeller->

■ **GCA_900108315.1; Saccharopolyspora hirsuta; counts= 1; multicellularity status: TRUE**

- EACC1*->CASPASE+BetaPropeller->BetaPropeller(fragment)->

■ **GCA_900107965.1; Streptomyces yanglinensis; counts= 1; multicellularity status: TRUE**

- low-complexity+HD+Tox-RelA+TGS+ACT->Pkinase+TPRs->EACC1*->CASPASE+TPRs->

■ **GCA_900109115.1; Xiangella phaseoli; counts= 1; multicellularity status: TRUE**

- EACC1*->CASPASE+Pkinase->

■ **GCA_900115345.1; Amycolatopsis rubida; counts= 1; multicellularity status: TRUE**

- EACC1*->CASPASE+??+SF1-helicase+Vsr(PDDEXK)?->vWA->

■ **GCA_900115095.1; Actinomadura madurae; counts= 1; multicellularity status: TRUE**

- EACC1*->CASPASE+??+TM+TM+TM->

■ **GCA_900119365.1; Streptomyces atratus; counts= 1; multicellularity status: TRUE**

- EACC1*->

■ **GCA_900142575.1; Streptomyces paucisporeus; counts= 1; multicellularity status: TRUE**

- EACC1*->CASPASE+STAND+polar_region+TM+BetaPropeller->

■ **GCA_900143515.1; Micromonospora cremea; counts= 1; multicellularity status: TRUE**

- EACC1*->

■ **GCA_900188025.1; Blastococcus sp. DSM 44272; counts= 1; multicellularity status: TRUE**

- EACC1*->CASPASE+TM+TM+TM+TM+TM+TM+TM+TM->Aminotran_5->

■ **GCA_900188205.1; Geodermatophilus saharensis; counts= 1; multicellularity status: TRUE**

- EACC1*->CASPASE+??+TM+TM->

■ **GCA_900230215.1; Jatrophihabitans sp. GAS493; counts= 1; multicellularity status: TRUE**

- EACC1*->CASPASE+low-complexity+DUF4190(has2TM)->?->Glyoxalase->

■ **GCA_900215565.1; Streptomyces sp. Ag109_G2-15; counts= 1; multicellularity status: TRUE**

- EACC1*->CASPASE+STAND+polar_region+TM+BetaPropeller->TM+Acid_phosphat_B->

■ **GCA_900230275.1; Streptomyces sp. OV198; counts= 1; multicellularity status: TRUE**

- EACC1*->CASPASE+TM+PBPI->

■ **GCA_900236475.1; Streptomyces chartreusis NRRL 3882; counts= 1; multicellularity status: TRUE**

- EACC1*->CASPASE+STAND+polar_region+TM+BetaPropeller->

■ **GCA_001693675.1; Streptomyces clavuligerus; counts= 1; multicellularity status: TRUE**

- EACC1*->CASPASE+??+SF1-helicase+Vsr(PDDEXK)?->

■ **GCA_000331185.1; Streptomycetaceae; counts= 1; multicellularity status: TRUE**

- EACC1*->CASPASE+TM?+Phytase-like->

■ **GCA_000342125.1; Streptomyces mobaraensis; counts= 1; multicellularity status: TRUE**

- EACC1*->

■ **GCA_000342345.1; Streptomyces; counts= 1; multicellularity status: TRUE**

- TM+TM+TM+PP2C->??->EACC1*->CASPASE+TM+TM+PBPB+vWA->

■ **GCA_000331005.1; Streptomyces turgidiscabies; counts= 1; multicellularity status: TRUE**

- EACC1*->CASPASE+TM+TM+bPH_domain+TM+TM+bPH_domain+TM+TM->

■ **GCF_000231035.2; Saccharomonospora paurometabolica; counts= 1; multicellularity status: TRUE**

- CASPASE+??->EACC1*->

■ **GCA_000342005.1; Amycolatopsis decaplanina; counts= 1; multicellularity status: TRUE**

- MFS_1->?->||<-?||EACC1*->

■ **GCA_000154965.1; Streptomyces sviceus; counts= 1; multicellularity status: TRUE**

- EACC1*->CASPASE+??+SF1-helicase+Vsr(PDDEXK)?->

■ **GCA_000297395.2; Micromonospora lupini; counts= 1; multicellularity status: TRUE**

- EACC1*->

■ **GCA_000225525.2; Streptomyces zinciresistens; counts= 1; multicellularity status: TRUE**

- HATPase_c_2->EACC1*->CASPASE+??+SF1-helicase+Vsr(PDDEXK)?->

■ **GCA_000178855.1; Ktedonobacter racemifer; counts= 1; multicellularity status: TRUE**

- EACC1*->CASPASE+STAND+??->

■ **GCA_000154885.1; Streptomyces sp. Mg1; counts= 1; multicellularity status: TRUE**

- EACC1*->CASPASE+STAND+polar_region+TM+BetaPropeller->

■ **GCA_002796545.1; Actinobacteria; counts= 1; multicellularity status: TRUE**

- EACC1*->

■ **GCA_000448385.1; Saccharopolyspora erythraea; counts= 1; multicellularity status: TRUE**

- EACC1*->CASPASE+??+SF1-helicase+Vsr(PDDEXK)?->

■ **GCF_000226455.1; Streptomyces chartreusis; counts= 1; multicellularity status: TRUE**

- EACC1*->CASPASE+STAND+polar_region+TM+BetaPropeller->

■ **GCA_001485105.1; Streptomyces acidiscabies; counts= 1; multicellularity status: TRUE**

- EACC1*->CASPASE+TM+TM+PBPB+vWA->

■ **GCF_000203835.1; Streptomyces coelicolor; counts= 1; multicellularity status: TRUE**

- *

■ **GCA_000010605.1; Streptomyces griseus; counts= 1; multicellularity status: TRUE**

- Beta_rich?->EACC1*->CASPASE+TPRs->??->??->

■ **GCA_000024865.1; Streptosporangium roseum; counts= 1; multicellularity status: TRUE**

- EACC1+??*->

■ **GCA_000092985.1; Nocardiopsis dassonvillei; counts= 1; multicellularity status: TRUE**

- EACC1*->CASPASE+TM?+Phytase-like->

■ **GCA_000166135.1; Frankia inefficax; counts= 1; multicellularity status: TRUE**

- EACC1*->CASPASE+TIR->

■ **GCA_000092385.1; Streptomyces; counts= 1; multicellularity status: TRUE**

- EACC1*->CASPASE+TM+TM+??+TM+TM+??+TM+TM+??->

■ **GCA_000265505.1; Bernardetia litoralis; counts= 1; multicellularity status: TRUE**

- EACC1*->CASPASE+??->

■ **GCA_000311765.1; Gluconobacter oxydans; counts= 1; multicellularity status: FALSE**

- EACC1*->CASPASE+GAPEAD1->

■ **GCA_000024025.1; Catenulispora acidiphila; counts= 1; multicellularity status: TRUE**

- EACC1*->CASPASE+low-complexity+BetaPropeller->CASPASE+low-complexity+BetaPropeller->

■ **GCA_000023245.1; Actinosynnema mirum; counts= 1; multicellularity status: TRUE**

- EACC1*->

■ **GCA_000963515.1; Streptomyces albulus; counts= 1; multicellularity status: TRUE**

- EACC1*->

■ **GCF_000309385.1; Nodosilinea nodulosa; counts= 1; multicellularity status: TRUE**

- EACC1*->CASPASE+DUF4062+??+HATPase_c_4+STAND->

■ **GCF_000341105.1; Nocardiopsis potens; counts= 1; multicellularity status: TRUE**

- EACC1*->CASPASE+TM?+Phytase-like->

■ **GCF_000341025.1; Nocardiopsis salina; counts= 1; multicellularity status: TRUE**

- EACC1*->

■ **GCA_000739085.1; Amycolatopsis methanolica; counts= 1; multicellularity status: TRUE**

- EACC1*->CASPASE+STAND+polar_region+TM+BetaPropeller->

■ **GCF_000379825.1; Longispora albida; counts= 1; multicellularity status: TRUE**

- EACC1*->CASPASE+STAND+polar_region+TM+BetaPropeller->

■ **GCA_001445835.1; Streptomyces vitaminophilus; counts= 1; multicellularity status: TRUE**

- EACC1*->CASPASE+TPRs->

■ **GCF_000373305.1; Streptomyces sp. CcalMP-8W; counts= 1; multicellularity status: TRUE**

- EACC1*->CASPASE+TPRs??+GHKL_ATPase+TPRs??->

■ **GCF_000373665.1; Streptomyces sp. BoleA5; counts= 1; multicellularity status: TRUE**

- EACC1*->CASPASE+PBPB+vWA->

■ **GCF_000374305.1; Actinomadura flavalba; counts= 1; multicellularity status: TRUE**

- EACC1*->CASPASE+SAD+HNH->

■ **GCF_000374445.1; Actinokineospora enzanensis; counts= 1; multicellularity status: TRUE**

- EACC1*->CASPASE+TPRs->

■ **GCF_000377165.1; Streptomyces sp. CNY243; counts= 1; multicellularity status: TRUE**

- EACC1*->CASPASE+??->

- **GCF_000377545.1; Streptomyces sp. CNY228; counts= 1; multicellularity status: TRUE**
  - TM+REase->EACC1*->CASPASE+??+SF1-helicase+Vsr(PDDEXK)?->
- **GCF_000377965.1; Streptomyces sp. CNB091; counts= 1; multicellularity status: TRUE**
  - Beta_rich?->EACC1*->CASPASE+TPRs->??->??->
- **GCF_000367365.1; Streptomyces prunicolor; counts= 1; multicellularity status: TRUE**
  - polar_lead?+TM+TM+TM+polar_tail?+low-complexity->??->EACC1*->CASPASE+TPRs->
- **GCF_000376565.1; Streptomyces hokutonensis; counts= 1; multicellularity status: TRUE**
  - EACC1*->CASPASE+STAND+BetaPropeller->
- **GCA_002260685.1; Nocardiopsis; counts= 1; multicellularity status: TRUE**
  - EACC1*->CASPASE+TM?+Phytase-like->
- **GCF_000469645.1; Rhodococcus; counts= 1; multicellularity status: TRUE**
  - EACC1*->CASPASE+??->
- **GCF_000384175.1; Streptomyces purpureus; counts= 1; multicellularity status: TRUE**
  - EACC1*->CASPASE+STAND+polar_region+TM+BetaPropeller->
- **GCF_000383935.1; Streptomyces sp. Amel2xE9; counts= 1; multicellularity status: TRUE**
  - TPRs->EACC1*->CASPASE+TPRs->
- **GCF_000340335.1; Streptomyces bottropensis; counts= 1; multicellularity status: TRUE**
  - EACC1*->CASPASE+TPRs->
- **GCF_000383615.1; Streptomyces canus; counts= 1; multicellularity status: TRUE**
  - low-complexity+HD+Tox-RelA+TGS+ACT->Pkinase+TPRs->EACC1*->CASPASE+TPRs->
- **GCF_000383655.1; Streptomyces sp. 351MFTsu5.1; counts= 1; multicellularity status: TRUE**
  - EACC1*->CASPASE+TPRs->
- **GCF_000383675.1; Terracoccus sp. 273MFTsu3.1; counts= 1; multicellularity status: TRUE**
  - EACC1*->CASPASE+??+TM+TM+TM+TM+TM+TM+TM+TM->
- **GCF_000231075.2; Amycolatopsis sp. ATCC 39116; counts= 1; multicellularity status: TRUE**
  - Pkinase+TM+TM+DUF3298+PSBP->EACC1*->CASPASE+STAND+polar_region+TM+BetaPropeller->||<-?||?->||<-PSE<-?<-?<-FAD_binding_4+??+BBE
- **GCA_000018005.1; Frankia sp. EAN1pec; counts= 1; multicellularity status: TRUE**
  - ??+TM+BetaPropeller->TIR+EACC1+EAD1*->
- **GCF_000379645.1; Actinoplanes globisporus; counts= 1; multicellularity status: TRUE**

- EACC1*->CASPASE+STAND+polar_region+TM+BetaPropeller->

■ **GCF_000383915.1; Amycolatopsis benzoatilytica; counts= 1; multicellularity status: TRUE**

- GATase->ABC-ATPase+DUF4162->cNMP_cyclase+TM+TM+TM+TM+TM+TM->EACC1*->

■ **GCF_000384315.1; Amycolatopsis nigrescens; counts= 1; multicellularity status: TRUE**

- ??+TM+TM+??+TM->PBPB->EACC1*->CASPASE+TPRs->PknG_rubred+Pkinase+PknG_TPR->ZnR+PP2C->vWA+alpha-helical->

■ **GCF_000526215.1; Nocardia sp. BMG51109; counts= 1; multicellularity status: TRUE**

- EACC1*->CASPASE+??->

■ **GCF_000468475.2; Actinomadura madurae; counts= 1; multicellularity status: TRUE**

- EACC1*->CASPASE+??+TM+TM+TM->

■ **GCF_000512185.1; Aeromonas hydrophila; counts= 1; multicellularity status: FALSE**

- EACC1*->

■ **GCF_000525635.1; Kutzneria albida; counts= 1; multicellularity status: TRUE**

- polar_lead?+TM+TM+TM+polar_tail?+low-complexity->??->EACC1*->CASPASE+TPRs->?->CASPASE+??+TPRs?->

■ **GCA_900116145.1; Streptomyces; counts= 1; multicellularity status: TRUE**

- EACC1*->CASPASE+??+SF1-helicase+Vsr(PDDEXK)?->

■ **GCF_000620185.1; Geodermatophilaceae bacterium URHB0062; counts= 1; multicellularity status: TRUE**

- EACC1*->CASPASE+TM+TM+TM+TM+TM+TM+TM+TM->

■ **GCF_000427885.1; Glycomyces tenuis; counts= 1; multicellularity status: TRUE**

- EACC1*->

■ **GCF_000422585.1; Hugenholtzia roseola; counts= 1; multicellularity status: TRUE**

- EACC1*->CASPASE+??->

■ **GCF_000482585.1; Streptomyces sp. CNQ329; counts= 1; multicellularity status: TRUE**

- EACC1*->CASPASE+STAND+TPRs->

■ **GCF_000473265.1; Amycolatopsis thermoflava; counts= 1; multicellularity status: TRUE**

- Pkinase+TM+TM+DUF3298+PSBP->EACC1*->CASPASE+STAND+polar_region+TM+BetaPropeller->

■ **GCF_000647875.1; Streptomyces sp. SolWspMP-sol2th; counts= 1; multicellularity status: TRUE**

- EACC1*->CASPASE+TPRs??+GHKL_ATPase+TPRs??->

■ **GCF_000426165.1; Streptomyces sp. TAA486; counts= 1; multicellularity status: TRUE**

- EACC1*->CASPASE+TPRs->

■ **GCF_000422825.1; Nocardioides insulae; counts= 1; multicellularity status: TRUE**

  - EACC1*->

- **GCA_000710755.1; Saccharopolyspora rectivirgula; counts= 1; multicellularity status: TRUE**
  - EACC1*->CASPASE+??+SF1-helicase+Vsr(PDDEXK)?->
- **GCF_000717015.1; Streptomyces; counts= 1; multicellularity status: TRUE**
  - low-complexity+HD+Tox-RelA+TGS+ACT->Pkinase+TPRs->EACC1*->CASPASE+TPRs->
- **GCF_000717245.1; [Kitasatospora] papulosa; counts= 1; multicellularity status: TRUE**
  - EACC1*->
- **GCF_000717915.1; Streptomyces sp. NRRL S-244; counts= 1; multicellularity status: TRUE**
  - EACC1*->CASPASE+??+SF1-helicase+Vsr(PDDEXK)?->
- **GCA_004364985.1; Glycomyces sp. NRRL B-16210; counts= 1; multicellularity status: TRUE**
  - EACC1*->CASPASE+TPRs->
- **GCF_000718945.1; Streptomyces sp. NRRL S-813; counts= 1; multicellularity status: TRUE**
  - ??->EACC1*->
- **GCA_900090135.1; Streptomyces; counts= 1; multicellularity status: TRUE**
  - EACC1*->CASPASE+Concanavalin?->
- **GCF_000719335.1; Streptomyces sp. NRRL S-87; counts= 1; multicellularity status: TRUE**
  - low-complexity+HD+Tox-RelA+TGS+ACT->Pkinase+TPRs->EACC1*->CASPASE+TPRs->
- **GCA_002082175.1; Streptomyces; counts= 1; multicellularity status: TRUE**
  - low-complexity+HD+Tox-RelA+TGS+ACT->Pkinase+TPRs->EACC1*->CASPASE+TPRs->
- **GCF_000718785.1; Streptomyces sp. NRRL WC-3626; counts= 1; multicellularity status: TRUE**
  - EACC1*->CASPASE+STAND+polar_region+TM+BetaPropeller->HTH+N6_Mtase+Methylase_S->
- **GCF_000717875.1; Streptomyces; counts= 1; multicellularity status: TRUE**
  - EACC1*->CASPASE+STAND+??+TPRs+??->
- **GCA_001279425.1; Streptomyces; counts= 1; multicellularity status: TRUE**
  - EACC1*->CASPASE+Concanavalin?->
- **GCA_000707945.1; Streptomycetaceae; counts= 1; multicellularity status: TRUE**
  - EACC1*->CASPASE+??+SF1-helicase+Vsr(PDDEXK)?->REase+STAND->
- **GCF_000717665.1; Streptomyces floridae; counts= 1; multicellularity status: TRUE**
  - EACC1*->CASPASE+Concanavalin?->
- **GCF_000719065.1; Herbidospora cretacea; counts= 1; multicellularity status: TRUE**

- EACC1*->

■ **GCF_000720835.1; Streptomyces achromogenes; counts= 1; multicellularity status: TRUE**

- EACC1*->CASPASE+??+SF1-helicase+Vsr(PDDEXK)?->

■ **GCA_900090065.1; Streptomyces; counts= 1; multicellularity status: TRUE**

- EACC1*->CASPASE+??+SF1-helicase+Vsr(PDDEXK)?->

■ **GCF_000720405.1; Streptomyces sp. NRRL B-24051; counts= 1; multicellularity status: TRUE**

- EACC1*->

■ **GCF_000721105.1; Streptomyces cellulosae; counts= 1; multicellularity status: TRUE**

- EACC1*->CASPASE+TPRs->

■ **GCA_900090075.1; Streptomyces; counts= 1; multicellularity status: TRUE**

- Beta_rich?->EACC1*->CASPASE+TPRs->??->??->

■ **GCF_000720115.1; Streptomyces sp. NRRL F-2580; counts= 1; multicellularity status: TRUE**

- EACC1*->CASPASE+??+SF1-helicase+Vsr(PDDEXK)?->

■ **GCF_000719915.1; Streptomyces sp. NRRL F-2890; counts= 1; multicellularity status: TRUE**

- EACC1*->CASPASE+TPRs->??->??->

■ **GCF_000718235.1; Streptomyces griseus; counts= 1; multicellularity status: TRUE**

- EACC1*->CASPASE+TPRs->

■ **GCA_000966965.1; Streptomyces; counts= 1; multicellularity status: TRUE**

- EACC1*->CASPASE+??+SF1-helicase+Vsr(PDDEXK)?->

■ **GCA_001279005.1; Streptomyces; counts= 1; multicellularity status: TRUE**

- EACC1*->CASPASE+STAND+??+TPRs->

■ **GCF_000719855.1; Streptomyces sp. NRRL F-4474; counts= 1; multicellularity status: TRUE**

- EACC1*->CASPASE+TPRs->

■ **GCA_001270595.1; Streptomyces varsoviensis; counts= 1; multicellularity status: TRUE**

- EACC1*->CASPASE+Pkinase+TM?+??->

■ **GCF_000721305.1; Streptomyces sp. NRRL S-1868; counts= 1; multicellularity status: TRUE**

- EACC1*->CASPASE+TPRs->

■ **GCF_000721445.1; Streptomyces sp. NRRL F-5053; counts= 1; multicellularity status: TRUE**

- EACC1*->CASPASE+TPRs->

■ **GCF_000721715.1; Streptomyces sp. NRRL B-24720; counts= 1; multicellularity status: TRUE**

- EACC1*->

■ **GCF_000719575.1; Streptomyces sp. NRRL S-1824; counts= 1; multicellularity status: TRUE**

- DUF262+DUF1524+DNA_binding_1+??->?->polar_lead?+TM+TM+TM+polar_tail?+low-complexity->??->EACC1*->

■ **GCF_000725555.1; Streptomyces erythrochromogenes; counts= 1; multicellularity status: TRUE**

- EACC1*->CASPASE+??+SF1-helicase+Vsr(PDDEXK)?->

■ **GCA_004219385.1; Enterobacteriaceae; counts= 1; multicellularity status: FALSE**

- EACC1*->

■ **GCF_000156455.1; Streptomyces filamentosus; counts= 1; multicellularity status: TRUE**

- EACC1*->

■ **GCA_003947585.1; Streptomyces rimosus; counts= 1; multicellularity status: TRUE**

- EACC1*->CASPASE+??+SF1-helicase+Vsr(PDDEXK)?->??->

■ **GCA_000583715.2; Nocardia seriolae; counts= 1; multicellularity status: TRUE**

- EACC1*->CASPASE+STAND+TPRs->

■ **GCF_000388135.1; Amycolatopsis vancoresmycina; counts= 1; multicellularity status: TRUE**

- PBPB->?->?->Aminotran_5+PAS_6+HTH_22->EACC1*->CASPASE+??+TM+TM->||<-Glyco_trans_4_4+UDPGT

■ **GCA_002189675.1; Streptomyces; counts= 1; multicellularity status: TRUE**

- EACC1*->CASPASE+TPRs->

■ **GCF_001984445.1; Streptomyces lydicus; counts= 1; multicellularity status: TRUE**

- Glyoxalase->EACC1*->CASPASE+DnaJ_ZnR+DnaJ_ZnR+DnaJ_C->

■ **GCF_000717055.1; Streptomyces iakyrus; counts= 1; multicellularity status: TRUE**

- EACC1*->CASPASE+TM+TM+PBPB+vWA->

■ **GCF_000797385.1; Streptomyces sp. CNQ431; counts= 1; multicellularity status: TRUE**

- EACC1*->CASPASE+??->

■ **GCF_000787755.1; Streptacidiphilus albus; counts= 1; multicellularity status: TRUE**

- EACC1*->CASPASE+TPRs->Pkinase+??->

■ **GCA_003182415.1; Actinokineospora spheciospongiae; counts= 1; multicellularity status: TRUE**

- EACC1*->CASPASE+??+ART->

■ **GCF_000482865.1; Actinokineospora inagensis; counts= 1; multicellularity status: TRUE**

- EACC1*->

■ **GCF_000158815.1; Micromonospora sp. ATCC 39149; counts= 1; multicellularity status: TRUE**

- TM+TM+TM+Peptidase_M48+TM+DUF4864+??->EACC1*->

■ **GCA_005863225.1; Nocardia cyriacigeorgica; counts= 1; multicellularity status: TRUE**

- RNR_N+Ribonuc_red_lgN+Ribonuc_red_lgC->EACC1*->

■ **GCF_000478275.1; Amycolatopsis orientalis; counts= 1; multicellularity status: TRUE**

- EACC1*->

■ **GCA_000754115.1; Amycolatopsis sp. MJM2582; counts= 1; multicellularity status: TRUE**

- EACC1*->

■ **GCF_000411975.1; Amycolatopsis orientalis; counts= 1; multicellularity status: TRUE**

- EACC1*->

■ **GCF_000744815.1; Streptacidiphilus oryzae; counts= 1; multicellularity status: TRUE**

- EACC1*->CASPASE+TPRs->

■ **GCF_000373645.1; Streptomyces sp. ATexAB-D23; counts= 1; multicellularity status: TRUE**

- EACC1*->CASPASE+STAND+PBPB->

■ **GCA_003073355.1; Streptomyces scopuliridis; counts= 1; multicellularity status: TRUE**

- EACC1*->CASPASE+STAND+polar_region+TM+BetaPropeller->

■ **GCF_000424805.1; Streptomyces sp. CNQ865; counts= 1; multicellularity status: TRUE**

- EACC1*->CASPASE+Beta_rich_repeat->

■ **GCA_004217635.1; Streptomyces sp. CNQ-525; counts= 1; multicellularity status: TRUE**

- EACC1*->CASPASE+TPRs->

■ **GCF_000701285.1; Streptomyces sp. CNQ-525; counts= 1; multicellularity status: TRUE**

- EACC1*->CASPASE+Beta_rich_repeat->

■ **GCF_000426325.1; Streptomyces sp. CNS606; counts= 1; multicellularity status: TRUE**

- EACC1*->CASPASE+??->

■ **GCF_000720075.1; Streptomyces sp. NRRL F-3213; counts= 1; multicellularity status: TRUE**

- GATase->ABC-ATPase+DUF4162->cNMP_cyclase+TM+TM+TM+TM+TM+TM->EACC1*->

■ **GCF_000698495.1; Streptomyces sp. NTK 937; counts= 1; multicellularity status: TRUE**

- EACC1*->CASPASE+STAND+PBPB->

■ **GCA_000802245.2; Streptomyces; counts= 1; multicellularity status: TRUE**

- HATPase_c_2->EACC1*->CASPASE+??+SF1-helicase+Vsr(PDDEXK)?->

■ **GCF_000308435.1; Nocardia araoensis; counts= 1; multicellularity status: TRUE**

- EACC1*->CASPASE+TM+TM->

■ **GCF_000308695.1; Nocardia takedensis; counts= 1; multicellularity status: TRUE**

- EACC1*->

■ **GCF_000308875.1; Nocardia transvalensis; counts= 1; multicellularity status: TRUE**

- EACC1*->CASPASE+TPRs->

■ **GCF_000308815.1; Nocardia concava; counts= 1; multicellularity status: TRUE**

- EACC1*->CASPASE+BetaPropeller->

■ **GCF_000308615.1; Nocardia jiangxiensis; counts= 1; multicellularity status: TRUE**

- DUF262+DUF1524+DNA_binding_1+??->?->EACC1*->CASPASE+STAND+TPRs+OMPdecase->

■ **GCF_000242715.1; Streptomyces acidiscabies; counts= 1; multicellularity status: TRUE**

- EACC1*->CASPASE+TM+TM+PBPB+vWA->

■ **GCF_000308655.1; Nocardia niigatensis; counts= 1; multicellularity status: TRUE**

- EACC1*->CASPASE+STAND+TPRs->

■ **GCA_000308475.2; Nocardia brasiliensis; counts= 1; multicellularity status: TRUE**

- EACC1*->CASPASE+??->

■ **GCF_000162775.1; Streptomyces sp. e14; counts= 1; multicellularity status: TRUE**

- TPRs->EACC1*->CASPASE+TPRs->

■ **GCA_000819545.1; Streptomyces nodosus; counts= 1; multicellularity status: TRUE**

- EACC1*->CASPASE+STAND+polar_region+TM+BetaPropeller->

■ **GCF_000718165.1; Streptomyces fulvoviolaceus; counts= 1; multicellularity status: TRUE**

- EACC1*->CASPASE+TPRs->

■ **GCA_001190165.1; Streptomyces; counts= 1; multicellularity status: TRUE**

- EACC1*->CASPASE+PBPB+vWA->

■ **GCF_000716435.1; Streptomyces xylophagus; counts= 1; multicellularity status: TRUE**

- EACC1*->CASPASE+TPRs->

■ **GCF_000411995.1; Amycolatopsis orientalis; counts= 1; multicellularity status: TRUE**

- GATase->ABC-ATPase+DUF4162->cNMP_cyclase+TM+TM+TM+TM+TM+TM->EACC1*->

■ **GCA_000876225.1; Micromonospora carbonacea; counts= 1; multicellularity status: TRUE**

- ??+TM+TM+TM+Peptidase_M48+TM+DUF4864+??->EACC1*->

■ **GCF_000931445.1; Streptomyces cyaneogriseus; counts= 1; multicellularity status: TRUE**

- EACC1*->CASPASE+Pkinase->

■ **GCA_000943515.2; Amycolatopsis orientalis; counts= 1; multicellularity status: TRUE**

- EACC1*->

■ **GCA_000955955.1; Lechevalieria aerocolonigenes; counts= 1; multicellularity status: TRUE**

- EACC1*->

■ **GCF_000958545.1; Streptomyces sp. FxanaA7; counts= 1; multicellularity status: TRUE**

- EACC1*->CASPASE+TM+TM+bPH_domain+TM+TM+bPH_domain+TM+TM->

■ **GCA_003687835.1; Enterobacteriaceae; counts= 1; multicellularity status: FALSE**

- EACC1*->

■ **GCF_000718635.1; Streptomyces varsoviensis; counts= 1; multicellularity status: TRUE**

- EACC1*->CASPASE+Pkinase+TM?+??->

■ **GCA_001280045.1; Streptomyces; counts= 1; multicellularity status: TRUE**

- EACC1*->PSE->MDMPI_N+MDMPI_C->META->

■ **GCF_900092705.1; Escherichia coli; counts= 1; multicellularity status: FALSE**

- EACC1*->

■ **GCF_000719175.1; Kitasatospora aureofaciens; counts= 1; multicellularity status: TRUE**

- EACC1*->

■ **GCF_001189015.1; Streptomyces acidiscabies; counts= 1; multicellularity status: TRUE**

- EACC1*->CASPASE+TM+TM+PBPB+vWA->

■ **GCF_000715845.1; Streptomyces monomycini; counts= 1; multicellularity status: TRUE**

- EACC1*->

■ **GCF_000716515.1; Streptomyces rimosus; counts= 1; multicellularity status: TRUE**

- EACC1*->CASPASE+TM?+Phytase-like->

■ **GCF_000514555.1; Salinispora arenicola; counts= 1; multicellularity status: TRUE**

- EACC1*->CASPASE+STAND+polar_region+TM+BetaPropeller->

■ **GCF_000158975.1; Streptomyces griseoflavus; counts= 1; multicellularity status: TRUE**

- MglB->DUF742->sGTP-MglA->?->Beta_rich?->EACC1*->CASPASE+TPRs->??->??->

■ **GCF_000154945.1; Streptomyces pristinaespiralis; counts= 1; multicellularity status: TRUE**

- low-complexity+HD+Tox-RelA+TGS+ACT->Pkinase+TPRs->EACC1*->CASPASE+TPRs->

■ **GCF_000058485.1; Frankia alni; counts= 1; multicellularity status: TRUE**

  - FHA+??+low-complexity+FHA+ABC-ATPase+TM->EACC1+EAD1*->||<-CASPASE+STAND+polar_region+TM+BetaPropeller
- **GCF_000308755.1; Nocardia pneumoniae; counts= 1; multicellularity status: TRUE**
  - EACC1*->CASPASE+STAND+polar_region+TM+BetaPropeller->
- **GCF_000284295.1; Actinoplanes missouriensis; counts= 1; multicellularity status: TRUE**
  - EACC1*->CASPASE+TPRs->
- **GCF_000316095.1; Streptomyces viridosporus; counts= 1; multicellularity status: TRUE**
  - EACC1*->CASPASE+TPRs->??->??->
- **GCF_000308515.1; Nocardia carnea; counts= 1; multicellularity status: TRUE**
  - EACC1*->
- **GCF_000308455.1; Nocardia abscessus; counts= 1; multicellularity status: TRUE**
  - EACC1*->CASPASE+STAND+polar_region+TM+BetaPropeller->
- **GCF_000308575.1; Nocardia exalbida; counts= 1; multicellularity status: TRUE**
  - Pkinase+TM+LppX_LprAFG->CASPASE+TPRs->EACC1*->
- **GCF_000426185.1; Cellulomonas sp. URHD0024; counts= 1; multicellularity status: FALSE**
  - EACC1*->CASPASE+STAND+polar_region+TM+BetaPropeller->
- **GCF_000429085.1; Streptomyces flavidovirens; counts= 1; multicellularity status: TRUE**
  - EACC1*->CASPASE+TM?+Phytase-like->
- **GCA_003752125.1; Amycolatopsis thermoflava; counts= 1; multicellularity status: TRUE**
  - EACC1*->CASPASE+TM+TM+TM+TM->
- **GCF_000428945.1; Hamadaea tsunoensis; counts= 1; multicellularity status: TRUE**
  - PBPB->?->?->?->?->?->EACC1*->
- **GCA_000696185.1; Kitasatospora cheerisanensis; counts= 1; multicellularity status: TRUE**
  - EACC1*->CASPASE+??+ZnR+ZnR->
- **GCF_000695235.1; Streptomyces albulus; counts= 1; multicellularity status: TRUE**
  - EACC1*->CASPASE+TM+TM+bPH_domain?->
- **GCF_000701325.1; Streptomyces sp. URHA0041; counts= 1; multicellularity status: TRUE**
  - Pkinase+TPRs->EACC1*->CASPASE+TPRs->
- **GCA_001294325.1; Streptomyces sp. NRRL F-5917; counts= 1; multicellularity status: TRUE**
  - EACC1*->CASPASE+TPRs->
- **GCF_000719095.1; Streptacidiphilus jeojiense; counts= 1; multicellularity status: TRUE**

- EACC1*->CASPASE+TPRs->Pkinase+??->

■ **GCF_000719495.1; Streptomyces griseus; counts= 1; multicellularity status: TRUE**

- EACC1*->CASPASE+??->

■ **GCF_000717175.1; Pseudonocardia autotrophica; counts= 1; multicellularity status: TRUE**

- EACC1*->CASPASE+STAND+polar_region+TM+BetaPropeller->

■ **GCF_000710405.2; Kitasatospora sp. MBT66; counts= 1; multicellularity status: TRUE**

- FAD_binding_4+FAD-oxidase_C->DAGK_cat+DAGK_acc->EACC1*->CASPASE+TPRs->?->Clp_N+Clp_N+ClpABN-AAA->

■ **GCA_900176525.1; Lentzea albidocapillata; counts= 1; multicellularity status: TRUE**

- TM+TM+BPD_transp_1->OppC_N+TM+TM+BPD_transp_1->ABC-ATPase+ABC-ATPase->PBPB->TM+Beta_rich+TM+Beta_rich+TM+TM->EACC1+CASPASE+TPR_repeats+low_complexity+TM_region*->

■ **GCF_000719695.1; Streptomyces sp. NRRL F-6131; counts= 1; multicellularity status: TRUE**

- FAD_binding_4+FAD-oxidase_C->DAGK_cat+DAGK_acc->EACC1*->CASPASE+TPRs->Clp_N+Clp_N+ClpABN-AAA->

■ **GCF_000717135.1; Catenuloplanes japonicus; counts= 1; multicellularity status: TRUE**

- METHYLASE->EACC1*->CASPASE+TM+TM+TM+TM->

■ **GCF_000721345.1; Kitasatospora sp. NRRL B-11411; counts= 1; multicellularity status: TRUE**

- EACC1*->CASPASE+alpha-helical+TM+TM+??+TM+TM->

■ **GCF_000717945.1; Streptomyces sp. NRRL S-920; counts= 1; multicellularity status: TRUE**

- EACC1*->CASPASE+GAF+PAS+GGDEF->

■ **GCF_000717595.1; Streptomyces flavochromogenes; counts= 1; multicellularity status: TRUE**

- EACC1*->CASPASE+TPRs+LysM+PG-binding->

■ **GCF_000720805.1; Streptomyces violaceoruber; counts= 1; multicellularity status: TRUE**

- EACC1*->CASPASE+TM+TM+bPH_domain?->

■ **GCA_001513955.1; Streptomyces; counts= 1; multicellularity status: TRUE**

- EACC1*->CASPASE+GAF+PAS->

■ **GCF_000720765.1; Streptomyces sp. NRRL S-646; counts= 1; multicellularity status: TRUE**

- EACC1*->CASPASE+??+TM+??->

■ **GCF_000717655.1; Streptomyces katrae; counts= 1; multicellularity status: TRUE**

- EACC1*->CASPASE+??->

■ **GCF_000720635.1; Streptomyces sp. NRRL WC-3742; counts= 1; multicellularity status: TRUE**

- EACC1*->CASPASE+??+TPRs->

■ **GCF_000725705.1; Streptomyces sp. NRRL S-623; counts= 1; multicellularity status: TRUE**

  - EACC1*->CASPASE+TPRs->

- **GCF_000718375.1; Streptomyces; counts= 1; multicellularity status: TRUE**
  - EACC1*->CASPASE+STAND+polar_region+TM+BetaPropeller->
- **GCF_000718315.1; Streptomyces griseofuscus; counts= 1; multicellularity status: TRUE**
  - EACC1*->
- **GCA_003933295.1; Streptomyces griseofuscus; counts= 1; multicellularity status: TRUE**
  - EACC1*->CASPASE+GAF+PAS->
- **GCF_000720475.1; Streptomyces aureocirculatus; counts= 1; multicellularity status: TRUE**
  - EACC1*->CASPASE+PBPB->
- **GCA_003846255.1; Streptomyces anulatus; counts= 1; multicellularity status: TRUE**
  - Beta_rich?->EACC1*->CASPASE+TPRs->??->??->
- **GCF_000725545.1; Streptomyces exfoliatus; counts= 1; multicellularity status: TRUE**
  - EACC1*->CASPASE+TPRs->DUF664->
- **GCF_000745345.1; Streptomyces yeochonensis; counts= 1; multicellularity status: TRUE**
  - EACC1*->CASPASE+TPRs->
- **GCF_000744705.1; Streptacidiphilus albus; counts= 1; multicellularity status: TRUE**
  - EACC1*->||<-?<-?||Pkinase+??->
- **GCF_000785985.1; Actinoplanes utahensis; counts= 1; multicellularity status: TRUE**
  - EACC1*->CASPASE+??->
- **GCF_001613425.1; Nocardia vulneris; counts= 1; multicellularity status: TRUE**
  - DUF2510(W3)+BicD+DUF4041+T5orf172->EACC1*->CASPASE+TPRs->
- **GCF_000519205.1; Amycolatopsis taiwanensis; counts= 1; multicellularity status: TRUE**
  - EACC1*->CASPASE+CLP_protease->
- **GCF_000739045.1; Streptomyces luteus; counts= 1; multicellularity status: TRUE**
  - low-complexity+HD+Tox-RelA+TGS+ACT->Pkinase+TPRs->EACC1*->CASPASE+TPRs->
- **GCA_000761215.1; Streptomyces glaucescens; counts= 1; multicellularity status: TRUE**
  - EACC1*->AB_hydrolase->
- **GCF_000826545.1; Kibdelosporangium sp. MJ126-NF4; counts= 1; multicellularity status: TRUE**
  - PALP->Trp_syntA->TM+LGT->EACC1*->
- **GCF_000938975.1; Streptomyces iranensis; counts= 1; multicellularity status: TRUE**

- EACC1*->

■ **GCF_000967025.1; Streptomyces sp. NRRL B-1568; counts= 1; multicellularity status: TRUE**

- EACC1*->CASPASE+STAND+polar_region+TM+BetaPropeller->

■ **GCF_000966955.1; Streptomyces sp. NRRL S-495; counts= 1; multicellularity status: TRUE**

- EACC1*->CASPASE+TPRs->Clp_N+Clp_N+ClpABN-AAA->

■ **GCF_000720235.1; Streptomyces; counts= 1; multicellularity status: TRUE**

- EACC1*->

■ **GCA_005490965.1; Streptomyces sp. MBT28; counts= 1; multicellularity status: TRUE**

- low-complexity+HD+Tox-RelA+TGS+ACT->Pkinase+TPRs->EACC1*->CASPASE+TPRs->

■ **GCF_000974985.2; Streptomyces mangrovisoli; counts= 1; multicellularity status: TRUE**

- Pkinase+TPRs->EACC1*->CASPASE+TPRs->

■ **GCF_001011035.1; Streptomyces sp. CNQ-509; counts= 1; multicellularity status: TRUE**

- EACC1+CASPASE+Phytase-like*->Phytase-like->

■ **GCF_000718775.1; Streptomyces sp. NRRL S-31; counts= 1; multicellularity status: TRUE**

- EACC1*->CASPASE+TM+??->

■ **GCF_001044425.1; Streptomyces roseus; counts= 1; multicellularity status: TRUE**

- low-complexity+HD+Tox-RelA+TGS+ACT->Pkinase+TPRs->EACC1*->CASPASE+TPRs->

■ **GCA_002249075.1; Enterobacteriaceae; counts= 1; multicellularity status: FALSE**

- EACC1*->

■ **GCA_001279445.1; Streptomyces; counts= 1; multicellularity status: TRUE**

- EACC1*->CASPASE+TPRs->

■ **GCA_001279855.1; Streptomyces; counts= 1; multicellularity status: TRUE**

- EACC1*->CASPASE+TPRs->

■ **GCF_001279805.1; Streptomyces sp. XY431; counts= 1; multicellularity status: TRUE**

- EACC1*->CASPASE+TPRs->Clp_N+Clp_N+ClpABN-AAA->

■ **GCA_001279555.1; Micromonospora sp. NRRL B-16802; counts= 1; multicellularity status: TRUE**

- EACC1*->

■ **GCF_001279725.1; Streptomyces sp. WM4235; counts= 1; multicellularity status: TRUE**

- EACC1*->CASPASE+??+SF1-helicase+Vsr(PDDEXK)?->

■ **GCA_001279725.1; Streptomyces sp. WM4235; counts= 1; multicellularity status: TRUE**

- EACC1*->CASPASE+TM?+BetaPropeller->

■ **GCF_001279885.1; Streptomyces sp. XY593; counts= 1; multicellularity status: TRUE**

- EACC1*->CASPASE+??+SF1-helicase+Vsr(PDDEXK)?->

■ **GCF_001279655.1; Streptomyces sp. WM6372; counts= 1; multicellularity status: TRUE**

- EACC1*->CASPASE+??->

■ **GCA_001279525.1; Nocardia sp. NRRL S-836; counts= 1; multicellularity status: TRUE**

- EACC1*->CASPASE+Cpn60_TCP1->

■ **GCA_001279775.1; Streptomyces sp. MMG1533; counts= 1; multicellularity status: TRUE**

- EACC1*->

■ **GCF_001279775.1; Streptomyces sp. MMG1533; counts= 1; multicellularity status: TRUE**

- EACC1*->CASPASE+STAND+TPRs->

■ **GCF_001280885.1; Streptomyces sp. XY332; counts= 1; multicellularity status: TRUE**

- EACC1*->CASPASE+??->

■ **GCF_001279345.1; Streptomyces rimosus; counts= 1; multicellularity status: TRUE**

- EACC1*->

■ **GCF_001282115.1; Streptomyces sp. NRRL B-24085; counts= 1; multicellularity status: TRUE**

- EACC1*->CASPASE+BetaPropeller->

■ **GCF_001294335.1; Streptomyces chattanoogensis; counts= 1; multicellularity status: TRUE**

- PAS+GAF+PP2C+HATPase_c_2->EACC1*->

■ **GCA_001292625.1; Shimia sp. SK013; counts= 1; multicellularity status: TRUE**

- EACC1*->CASPASE+TM+??->

■ **GCA_900070365.1; Alloactinosynnema sp. L-07; counts= 1; multicellularity status: TRUE**

- EACC1*->CASPASE+STAND+TPRs->

■ **GCA_002056725.1; Comamonas kerstersii; counts= 1; multicellularity status: FALSE**

- EACC1*->

■ **GCA_001302585.1; Kibdelosporangium phytohabitans; counts= 1; multicellularity status: TRUE**

- PALP->Trp_syntA->TM+LGT->EACC1*->

■ **GCF_001618405.1; Nocardia arizonensis; counts= 1; multicellularity status: TRUE**

- EACC1*->

■ **GCA_001414035.1; Frankia sp. ACN1ag; counts= 1; multicellularity status: TRUE**

- <-CASPASE+STAND+polar_region+TM+BetaPropeller||EACC1+EAD1*->

■ **GCA_002005225.1; Streptomyces pactum; counts= 1; multicellularity status: TRUE**

- polar_lead?+TM+TM+TM+polar_tail?+low-complexity->??->EACC1*->CASPASE+TPRs->

■ **GCF_001418495.1; Streptomyces pathocidini; counts= 1; multicellularity status: TRUE**

- EACC1*->CASPASE+Peripla_BP_5->

■ **GCF_001418565.1; Streptomyces graminilatus; counts= 1; multicellularity status: TRUE**

- EACC1*->CASPASE+PBPB->

■ **GCF_001418325.1; Streptomyces atriruber; counts= 1; multicellularity status: TRUE**

- EACC1*->CASPASE+TPRs->

■ **GCF_001418575.1; Streptomyces griseoplanus; counts= 1; multicellularity status: TRUE**

- ??->EACC1*->CASPASE+TPRs->

■ **GCF_001418415.1; Streptomyces hirsutus; counts= 1; multicellularity status: TRUE**

- EACC1*->CASPASE+STAND+polar_region+TM+BetaPropeller->

■ **GCF_001418165.1; Streptomyces prasinus; counts= 1; multicellularity status: TRUE**

- TIR+CASPASE->EACC1*->

■ **GCF_001418175.1; Streptomyces hirsutus; counts= 1; multicellularity status: TRUE**

- EACC1*->CASPASE+TPRs->

■ **GCF_001419695.1; Streptomyces prasinopilosus; counts= 1; multicellularity status: TRUE**

- ??+CASPASE->EACC1*->

■ **GCF_001748305.1; Streptomyces; counts= 1; multicellularity status: TRUE**

- low-complexity+HD+Tox-RelA+TGS+ACT->Pkinase+TPRs->EACC1*->CASPASE+TPRs->

■ **GCA_003129505.1; Streptomyces; counts= 1; multicellularity status: TRUE**

- EACC1*->CASPASE+TM+TM+PBPB+vWA->

■ **GCF_001420875.1; Frankia sp. AvcI1; counts= 1; multicellularity status: TRUE**

- FHA+??+low-complexity+FHA+ABC-ATPase+TM->EACC1+EAD1*->||<-CASPASE+STAND+polar_region+TM+BetaPropeller

■ **GCA_001426325.1; Streptomyces; counts= 1; multicellularity status: TRUE**

- EACC1*->CASPASE+STAND+??+TPRs+??->

■ **GCA_001429655.1; Terrabacter sp. Soil811; counts= 1; multicellularity status: TRUE**

- EACC1*->CASPASE+??+TM+TM+TM+TM+TM+TM+TM+TM->

■ **GCA_001426615.1; Streptomyces; counts= 1; multicellularity status: TRUE**

- ??+CASPASE->EACC1*->

■ **GCA_001427245.1; Streptomyces sp. Root55; counts= 1; multicellularity status: TRUE**

- EACC1*->CASPASE+STAND+polar_region+TM+BetaPropeller->

■ **GCA_001429185.1; Terrabacter sp. Root85; counts= 1; multicellularity status: TRUE**

- EACC1*->CASPASE+??+TM+TM+TM+TM+TM+TM+TM+TM->

■ **GCA_900094765.1; Rhodococcus enclensis; counts= 1; multicellularity status: TRUE**

- EACC1*->CASPASE+??->

■ **GCA_002903025.1; Klebsiella pneumoniae; counts= 1; multicellularity status: FALSE**

- EACC1*->

■ **GCA_900114955.1; Streptomyces; counts= 1; multicellularity status: TRUE**

- EACC1*->CASPASE+TM+TM+bPH_domain+TM+TM+bPH_domain+TM+TM->

■ **GCF_001550235.1; Streptomyces scabiei; counts= 1; multicellularity status: TRUE**

- EACC1*->CASPASE+TPRs->

■ **GCA_000772045.1; Streptomyces sp. CCM_MD2014; counts= 1; multicellularity status: TRUE**

- EACC1*->CASPASE+TPRs->

■ **GCA_001595515.1; Streptomyces sp. CC71; counts= 1; multicellularity status: TRUE**

- EACC1*->CASPASE(fragment)->

■ **GCF_000974485.1; Streptomyces sp. NBRC 109706; counts= 1; multicellularity status: TRUE**

- TM+TM+TM+PP2C->EACC1*->CASPASE+PBPB+vWA->

■ **GCF_001570525.1; Herbidospora sakaeratensis; counts= 1; multicellularity status: TRUE**

- EACC1*->

■ **GCA_001514235.1; Streptomyces caeruleatus; counts= 1; multicellularity status: TRUE**

- *

■ **GCA_002943895.1; Streptomyces; counts= 1; multicellularity status: TRUE**

- EACC1*->CASPASE+STAND+polar_region+TM+BetaPropeller->

■ **GCF_001613385.1; Nocardia flavorosea; counts= 1; multicellularity status: TRUE**

- EACC1*->CASPASE+TPRs->

■ **GCF_001612725.1; Nocardia anaemiae; counts= 1; multicellularity status: TRUE**

- EACC1*->CASPASE+TPRs->

■ **GCF_001612865.1; Nocardia cummidelens; counts= 1; multicellularity status: TRUE**

  - DUF2510(W3)+BicD+DUF4041+T5orf172->EACC1->CASPASE+TPRs->EACC1*->CASPASE+EAL->

- **GCF_001613445.1; Nocardia grenadensis; counts= 1; multicellularity status: TRUE**
  - EACC1*->CASPASE+TPRs->
- **GCF_001613205.1; Nocardia sienata; counts= 1; multicellularity status: TRUE**
  - EACC1*->CASPASE+??+SF1-helicase+Vsr(PDDEXK)?->
- **GCA_001632865.1; Streptomyces ambofaciens; counts= 1; multicellularity status: TRUE**
  - EACC1*->CASPASE+GTP_EFTU+GTP_EFTU_D2+GTP_EFTU_D3->
- **GCF_001418335.1; Streptomyces aurantiacus; counts= 1; multicellularity status: TRUE**
  - EACC1*->
- **GCA_001646665.1; Streptomyces albulus; counts= 1; multicellularity status: TRUE**
  - HATPase_c_2->EACC1*->CASPASE+??+SF1-helicase+Vsr(PDDEXK)?->
- **GCA_001654495.1; Streptomyces sp. SAT1; counts= 1; multicellularity status: TRUE**
  - ??->EACC1*->CASPASE+TM+TM+PBPB+vWA->
- **GCA_001672315.1; Streptomyces sp. H-KF8; counts= 1; multicellularity status: TRUE**
  - ??->EACC1*->CASPASE+TPRs->
- **GCF_001687325.1; Streptomyces sp. PTY087I2; counts= 1; multicellularity status: TRUE**
  - EACC1*->CASPASE+TPRs->
- **GCA_002844785.1; Enterobacteriaceae; counts= 1; multicellularity status: FALSE**
  - EACC1*->
- **GCF_001570545.1; Herbidospora mongoliensis; counts= 1; multicellularity status: TRUE**
  - EACC1*->
- **GCA_001509485.1; Streptomyces sp. NRRL F-4489; counts= 1; multicellularity status: TRUE**
  - low-complexity+HD+Tox-RelA+TGS+ACT->Pkinase+TPRs->EACC1*->CASPASE+TPRs->
- **GCA_001514035.1; Streptomyces yokosukanensis; counts= 1; multicellularity status: TRUE**
  - EACC1*->CASPASE+TPRs->
- **GCA_001514125.1; Streptomyces longwoodensis; counts= 1; multicellularity status: TRUE**
  - EACC1*->CASPASE+STAND+??->
- **GCA_001514285.1; Streptomyces sp. DSM 15324; counts= 1; multicellularity status: TRUE**
  - EACC1*->CASPASE+low-complexity+BetaPropeller->
- **GCA_001704275.1; Streptomyces noursei; counts= 1; multicellularity status: TRUE**

- low-complexity+HD+Tox-RelA+TGS+ACT->Pkinase+TPRs->EACC1*->CASPASE+TPRs->

■ **GCF_001612745.1; Nocardia amamiensis; counts= 1; multicellularity status: TRUE**

- low_complexity+EACC1*->

■ **GCA_004362495.1; Nocardia ignorata; counts= 1; multicellularity status: TRUE**

- EACC1*->CASPASE+EAL->

■ **GCF_001553785.1; Actinoplanes sp. TFC3; counts= 1; multicellularity status: TRUE**

- EACC1*->CASPASE+SF1-helicase->

■ **GCF_001613345.1; Nocardia uniformis; counts= 1; multicellularity status: TRUE**

- EACC1*->CASPASE+??->

■ **GCA_001651785.1; Amycolatopsis; counts= 1; multicellularity status: TRUE**

- GATase->ABC-ATPase+DUF4162->cNMP_cyclase+TM+TM+TM+TM+TM+TM->EACC1*->

■ **GCF_001612615.1; Nocardia amikacinitolerans; counts= 1; multicellularity status: TRUE**

- EACC1*->CASPASE+TPRs->

■ **GCF_001612945.1; Nocardia inohanensis; counts= 1; multicellularity status: TRUE**

- EACC1*->CASPASE+??->

■ **GCF_001552235.1; Actinomadura; counts= 1; multicellularity status: TRUE**

- EACC1*->CASPASE+??+NUDIX->

■ **GCF_001612825.1; Nocardia caishijiensis; counts= 1; multicellularity status: TRUE**

- EACC1*->CASPASE+TM+TM+TM+TM+TM+TM+TM+TM->

■ **GCF_001613165.1; Nocardia mexicana; counts= 1; multicellularity status: TRUE**

- EACC1*->

■ **GCA_004563805.1; Streptomyces niveus; counts= 1; multicellularity status: TRUE**

- EACC1*->?->PALP->

■ **GCA_003353555.1; Streptomyces sp. EN27; counts= 1; multicellularity status: TRUE**

- EACC1*->CASPASE+TPRs->

■ **GCA_001751255.1; Streptomyces nanshensis; counts= 1; multicellularity status: TRUE**

- EACC1*->CASPASE+STAND+??+TPRs->

■ **GCA_001751305.1; Streptomyces; counts= 1; multicellularity status: TRUE**

- EACC1*->CASPASE+TPRs->

■ **GCA_001858135.1; Enterobacter cloacae complex ‘Hoffmann cluster IV’; counts= 1; multicellularity status: FALSE**

- EACC1*->

■ **GCA_001866645.1; Streptomyces sp. MUSC 93; counts= 1; multicellularity status: TRUE**

- EACC1*->CASPASE+??+TM+TM+??->

■ **GCA_001879105.1; Streptomyces gilvigriseus; counts= 1; multicellularity status: TRUE**

- DUF742->?->?->||<-?<-?||EACC1*->?->CASPASE+PBPB+vWA->?->||<-?||MFS_1->

■ **GCA_001884705.1; Couchioplanes caeruleus; counts= 1; multicellularity status: TRUE**

- EACC1*->CASPASE+DUF179->SIR2+??->??->

■ **GCF_001885705.1; Streptomyces cinnamoneus; counts= 1; multicellularity status: TRUE**

- EACC1*->CASPASE+TM+TM+PBPB+vWA->

■ **GCF_900119165.1; Amycolatopsis australiensis; counts= 1; multicellularity status: TRUE**

- MFS_1->||<-?||GATase->ABC-ATPase+DUF4162->cNMP_cyclase+TM+TM+TM+TM+TM+TM->EACC1*->CASPASE+TM+TM+TM+TM->EACC1->

■ **GCA_001888885.1; Streptomyces viridifaciens; counts= 1; multicellularity status: TRUE**

- EACC1*->

■ **GCF_001895105.1; Streptomyces sp. NBRC 110465; counts= 1; multicellularity status: TRUE**

- Beta_rich?->EACC1*->CASPASE+TPRs->??->??->

■ **GCA_900141985.1; Nocardiopsis flavescens; counts= 1; multicellularity status: TRUE**

- EACC1*->CASPASE+STAND+polar_region+TM+BetaPropeller->

■ **GCA_001905125.1; Streptomyces sp. TSRI0281; counts= 1; multicellularity status: TRUE**

- EACC1*->CASPASE+??+SF1-helicase+Vsr(PDDEXK)?->

■ **GCA_001905385.1; Streptomyces sp. CB02414; counts= 1; multicellularity status: TRUE**

- EACC1*->CASPASE+TPRs->

■ **GCF_001905385.1; Streptomyces sp. CB02414; counts= 1; multicellularity status: TRUE**

- EACC1*->CASPASE+STAND+polar_region+TM+BetaPropeller->

■ **GCA_001905005.1; Streptomyces sp. CB02923; counts= 1; multicellularity status: TRUE**

- SF2-helicase+RecQ_Zn_bind+RQC+HRDC->EACC1*->

■ **GCA_001905425.1; Streptomyces sp. MJM1172; counts= 1; multicellularity status: TRUE**

- EACC1*->CASPASE+STAND+polar_region+TM+BetaPropeller->

■ **GCA_001905025.1; Streptomyces; counts= 1; multicellularity status: TRUE**

- EACC1*->CASPASE+STAND+polar_region+TM+BetaPropeller->

■ **GCA_001905465.1; Kitasatospora sp. CB01950; counts= 1; multicellularity status: TRUE**

- EACC1*->CASPASE+TM+TM+TM+TM->

■ **GCA_001905755.1; Amycolatopsis sp. CB00013; counts= 1; multicellularity status: TRUE**

- EACC1*->

■ **GCA_001905735.1; Streptomyces sp. CB01249; counts= 1; multicellularity status: TRUE**

- EACC1*->CASPASE+TPRs->

■ **GCA_001905575.1; Streptomyces sp. CB00072; counts= 1; multicellularity status: TRUE**

- EACC1*->

■ **GCF_001905725.1; Streptomyces sp. CB02400; counts= 1; multicellularity status: TRUE**

- EACC1*->CASPASE+STAND+polar_region+TM+BetaPropeller->HTH+N6_Mtase+Methylase_S->

■ **GCA_900141745.1; Paraburkholderia phenazinium; counts= 1; multicellularity status: FALSE**

- EACC1*->CASPASE+TPRs->

■ **GCF_900143515.1; Micromonospora cremea; counts= 1; multicellularity status: TRUE**

- EACC1*->

■ **GCA_001941625.1; Actinoalloteichus; counts= 1; multicellularity status: TRUE**

- EACC1*->CASPASE+??->

■ **GCA_001940455.1; Actinokineospora bangkokensis; counts= 1; multicellularity status: TRUE**

- EACC1*->CASPASE+GTP_EFTU+GTP_EFTU_D2->

■ **GCA_001953885.1; Streptomyces sp. IMTB 2501; counts= 1; multicellularity status: TRUE**

- EACC1*->CASPASE+TM+TM+PBPB+vWA->

■ **GCA_001953865.1; Amycolatopsis coloradensis; counts= 1; multicellularity status: TRUE**

- EACC1*->

■ **GCA_001983105.1; Frankia asymbiotica; counts= 1; multicellularity status: TRUE**

- EACC1*->CASPASE+STAND+polar_region+TM+BetaPropeller->

■ **GCA_001970135.1; Streptomyces sp. M1013; counts= 1; multicellularity status: TRUE**

- EACC1*->CASPASE+TPRs->

■ **GCA_002006875.1; Micromonospora sp. Rc5; counts= 1; multicellularity status: TRUE**

- ??+TM+TM+TM+Peptidase_M48+TM+DUF4864+??->?->EACC1*->

■ **GCF_000497445.1; Streptomyces roseochromogenus; counts= 1; multicellularity status: TRUE**

- EACC1*->CASPASE+TPRs->

■ **GCF_000720185.1; Streptomyces sp. NRRL S-340; counts= 1; multicellularity status: TRUE**

- EACC1*->CASPASE+TM+TM+PBPB+vWA->

■ **GCA_002024165.1; Streptomyces scabrisporus; counts= 1; multicellularity status: TRUE**

- EACC1*->

■ **GCF_001572115.1; Streptomyces; counts= 1; multicellularity status: TRUE**

- EACC1*->CASPASE+TPRs->

■ **GCF_001735805.1; Streptomyces puniciscabiei; counts= 1; multicellularity status: TRUE**

- EACC1*->CASPASE+STAND+polar_region+TM+BetaPropeller->

■ **GCF_001005085.2; Streptomyces humi; counts= 1; multicellularity status: TRUE**

- EACC1*->CASPASE+TPRs->

■ **GCF_900142595.1; Streptomyces yunnanensis; counts= 1; multicellularity status: TRUE**

- EACC1*->CASPASE+TM+MFS_1+TM+TM+TM+TM+TM+TM+TM+TM->

■ **GCF_001905365.1; Streptomyces sp. CB02488; counts= 1; multicellularity status: TRUE**

- MglB->DUF742->sGTP-MglA->?->?->EACC1*->CASPASE+TPRs->||<-?||?->?->DUF664->

■ **GCF_001941845.1; Streptomyces sp. TN58; counts= 1; multicellularity status: TRUE**

- EACC1*->CASPASE+RE_Eco29kI+??->

■ **GCF_001905885.1; Streptomyces sp. CB03234; counts= 1; multicellularity status: TRUE**

- FAD_binding_4+??+BBE->EACC1*->CASPASE+STAND+polar_region+TM+BetaPropeller->

■ **GCF_002027195.1; Streptomyces sp. GKU 895; counts= 1; multicellularity status: TRUE**

- EACC1*->

■ **GCA_002027145.1; Microbispora sp. GKU 823; counts= 1; multicellularity status: TRUE**

- EACC1+CASPASE+TPR_region*->

■ **GCA_900129505.1; Bradyrhizobium erythrophlei; counts= 1; multicellularity status: TRUE**

- EACC1*->CASPASE+??+TM->

■ **GCA_002754515.1; Streptomyces; counts= 1; multicellularity status: TRUE**

- EACC1*->CASPASE+??->

■ **GCA_002057455.1; Nonomuraea sp. ATCC 55076; counts= 1; multicellularity status: TRUE**

- ARSR-HTH+RHOD-CDC25->??->??->EACC1*->

■ **GCF_002078235.1; Streptomyces viridosporus; counts= 1; multicellularity status: TRUE**

- EACC1*->CASPASE+TPRs->??->??->

■ **GCA_001298555.1; Actinobacteria bacterium OV450; counts= 1; multicellularity status: NA**

- HATPase_c_2->EACC1*->CASPASE+??+SF1-helicase+Vsr(PDDEXK)?->

■ **GCF_001426245.1; Phycicoccus; counts= 1; multicellularity status: NA**

- EACC1*->CASPASE+??+TM+TM+TM+TM+TM+TM+TM+TM+TM+TM+TM->

■ **GCA_900113515.1; Amycolatopsis regifaucium; counts= 1; multicellularity status: TRUE**

- EACC1*->

■ **GCF_001509495.1; Actinoplanes awajinensis; counts= 1; multicellularity status: TRUE**

- EACC1*->CASPASE+??->

■ **GCA_001567585.1; Micromonospora rosaria; counts= 1; multicellularity status: TRUE**

- EACC1*->CASPASE+TM+TM+TM+TM+TM+TM->

■ **GCA_002082585.1; Kitasatospora albolonga; counts= 1; multicellularity status: TRUE**

- EACC1*->CASPASE+??+SF1-helicase+Vsr(PDDEXK)?->

■ **GCF_001886715.1; Nocardia sp. Y48; counts= 1; multicellularity status: TRUE**

- EACC1*->

■ **GCA_002099185.1; Streptomyces sp. CB03238; counts= 1; multicellularity status: TRUE**

- EACC1*->CASPASE->

■ **GCA_002114065.1; Streptomyces griseofuscus; counts= 1; multicellularity status: TRUE**

- EACC1*->CASPASE+GAF+PAS->

■ **GCA_002128305.1; Streptomyces sp. SCSIO 03032; counts= 1; multicellularity status: TRUE**

- EACC1*->CASPASE+STAND+polar_region+TM+BetaPropeller->

■ **GCA_002149035.1; Streptosporangium minutum; counts= 1; multicellularity status: TRUE**

- EACC1+??*->

■ **GCF_002150765.1; Lentzea kentuckyensis; counts= 1; multicellularity status: TRUE**

- TM+TM+BPD_transp_1->OppC_N+TM+TM+BPD_transp_1->ABC-ATPase+ABC-ATPase->PBPB->TM+Beta_rich+TM+Beta_rich+TM+TM->EACC1+CASPASE+TPR_repeats+low_complexity+TM_region*->

■ **GCA_900107925.1; Amycolatopsis pretoriensis; counts= 1; multicellularity status: TRUE**

- GATase->ABC-ATPase+DUF4162->cNMP_cyclase+TM+TM+TM+TM+TM+TM->EACC1*->

■ **GCF_002154415.1; Streptomyces angustmyceticus; counts= 1; multicellularity status: TRUE**

- EACC1*->CASPASE+TPRs->

■ **GCF_002156075.1; Streptomyces scabiei; counts= 1; multicellularity status: TRUE**

- EACC1*->CASPASE+??->

■ **GCF_002154575.1; Streptomyces bobili; counts= 1; multicellularity status: TRUE**

- low-complexity+HD+Tox-RelA+TGS+ACT->Pkinase+TPRs->EACC1*->CASPASE+TPRs->

■ **GCF_002155725.1; Streptomyces scabiei; counts= 1; multicellularity status: TRUE**

- EACC1*->CASPASE+TPRs->

■ **GCA_006539165.1; Streptomyces cacaoi; counts= 1; multicellularity status: TRUE**

- EACC1*->CASPASE+TPRs->

■ **GCA_003937945.1; Amycolatopsis lexingtonensis; counts= 1; multicellularity status: TRUE**

- GATase->ABC-ATPase+DUF4162->cNMP_cyclase+TM+TM+TM+TM+TM+TM->EACC1*->

■ **GCF_002155895.1; Streptomyces rochei; counts= 1; multicellularity status: TRUE**

- low-complexity+HD+Tox-RelA+TGS+ACT->Pkinase+TPRs->EACC1*->CASPASE+TPRs->

■ **GCF_002155885.1; Streptomyces rhizosphaericus; counts= 1; multicellularity status: TRUE**

- EACC1*->CASPASE+STAND+TPRs->

■ **GCA_002188365.1; Streptomyces sp. CS113; counts= 1; multicellularity status: TRUE**

- EACC1*->CASPASE+TPRs->

■ **GCA_900091515.1; Micromonospora purpureochromogenes; counts= 1; multicellularity status: TRUE**

- TM+TM+TM+Peptidase_M48+TM+DUF4864+??->EACC1*->

■ **GCA_002266845.1; Micromonospora echinospora; counts= 1; multicellularity status: TRUE**

- EACC1*->CASPASE+TM+TM+TM+TM+TM+TM->

■ **GCF_900091505.1; Micromonospora chokoriensis; counts= 1; multicellularity status: TRUE**

- EACC1*->

■ **GCA_900090235.1; Micromonospora echinaurantiaca; counts= 1; multicellularity status: TRUE**

- TM+TM+TM+Peptidase_M48+TM+DUF4864+??->EACC1*->TPRs+CASPASE->

■ **GCA_900091455.1; Micromonospora coriariae; counts= 1; multicellularity status: TRUE**

- EACC1*->

■ **GCF_002140155.1; Micromonospora sp. NBS 11-29; counts= 1; multicellularity status: TRUE**

- EACC1*->CASPASE+STAND+polar_region+TM+BetaPropeller->

■ **GCF_900188345.1; Streptosporangium subroseum; counts= 1; multicellularity status: TRUE**

- EACC1*->

■ **GCA_900188405.1; Streptomyces glauciniger; counts= 1; multicellularity status: TRUE**

- EACC1*->CASPASE+PBPB+vWA->

■ **GCF_900188005.1; Actinoplanes regularis; counts= 1; multicellularity status: TRUE**

- EACC1*->CASPASE+TPRs->

■ **GCF_900188105.1; Actinomadura mexicana; counts= 1; multicellularity status: TRUE**

- EACC1*->CASPASE+TM+TM->

■ **GCF_900188025.1; Blastococcus sp. DSM 44272; counts= 1; multicellularity status: TRUE**

- EACC1*->CASPASE+TM+TM+TM+TM+TM+TM+TM+TM->Aminotran_5->

■ **GCA_000522445.1; Candidatus Entotheonella palauensis; counts= 1; multicellularity status: TRUE**

- RVT_1->23S_rRNA_IVP->EACC1*->

■ **GCA_900110955.1; Lechevalieria xinjiangensis; counts= 1; multicellularity status: TRUE**

- TM+TM+BPD_transp_1->OppC_N+TM+TM+BPD_transp_1->ABC-ATPase+ABC-ATPase->PBPB->TM+Beta_rich+TM+Beta_rich+TM+TM->EACC1+CASPASE+TPR_repeats+low_complexity+TM_region*->

■ **GCA_900100275.1; Lentzea violacea; counts= 1; multicellularity status: TRUE**

- TM+TM+BPD_transp_1->OppC_N+TM+TM+BPD_transp_1->ABC-ATPase+ABC-ATPase->PBPB->TM+Beta_rich+TM+Beta_rich+TM+TM->EACC1+CASPASE+TPR_repeats+low_complexity+TM_region*->

■ **GCA_900100955.1; Lechevalieria fradiae; counts= 1; multicellularity status: TRUE**

- TM+TM+BPD_transp_1->OppC_N+TM+TM+BPD_transp_1->ABC-ATPase+ABC-ATPase->PBPB->TM+Beta_rich+TM+Beta_rich+TM+TM->EACC1+CASPASE+TPR_repeats+low_complexity+TM_region*->

■ **GCA_003350445.1; Lentzea flaviverrucosa; counts= 1; multicellularity status: TRUE**

- TM+TM+BPD_transp_1->OppC_N+TM+TM+BPD_transp_1->ABC-ATPase+ABC-ATPase->PBPB->TM+Beta_rich+TM+Beta_rich+TM+TM->EACC1+CASPASE+TPR_repeats+low_complexity+TM_region*->

■ **GCA_900104175.1; Lentzea jiangxiensis; counts= 1; multicellularity status: TRUE**

- TM+TM+BPD_transp_1->OppC_N+TM+TM+BPD_transp_1->ABC-ATPase+ABC-ATPase->PSE->TM+Beta_rich+TM+Beta_rich+TM+TM->EACC1+CASPASE+TPR_repeats+low_complexity+TM_region*->

■ **GCF_900101745.1; Glycomyces harbinensis; counts= 1; multicellularity status: TRUE**

- EACC1*->CASPASE+TPRs->

■ **GCA_900102815.1; Glycomyces sambucus; counts= 1; multicellularity status: TRUE**

- EACC1*->

■ **GCA_001536285.1; Frankia sp. G2; counts= 1; multicellularity status: TRUE**

- EACC1+EAD1*->CASPASE+STAND+polar_region+TM+BetaPropeller->

■ **GCF_900107045.1; Amycolatopsis xylanica; counts= 1; multicellularity status: TRUE**

- EACC1*->CASPASE+STAND+polar_region+TM+BetaPropeller->

■ **GCF_900100325.1; Frankineae bacterium MT45; counts= 1; multicellularity status: TRUE**

- EACC1*->CASPASE+low-complexity+DUF4190(has2TM)->

■ **GCA_900091615.1; Micromonospora yangpuensis; counts= 1; multicellularity status: TRUE**

- EACC1*->CASPASE+SF1-helicase->

■ **GCA_900114035.1; Amycolatopsis sacchari; counts= 1; multicellularity status: TRUE**

- EACC1*->CASPASE+TM+TM+TM+TM->

■ **GCA_000749465.3; Amycolatopsis lurida; counts= 1; multicellularity status: TRUE**

- EACC1*->

■ **GCA_900110575.1; Amycolatopsis saalfeldensis; counts= 1; multicellularity status: TRUE**

- EACC1*->

■ **GCF_003253475.1; Xiangella phaseoli; counts= 1; multicellularity status: TRUE**

- EACC1*->CASPASE+Pkinase->

■ **GCA_900104725.1; Actinoplanes derwentensis; counts= 1; multicellularity status: TRUE**

- ??+STAND+??->??+STAND+EACC1*->

■ **GCF_900114325.1; Rhodanobacter glycinis; counts= 1; multicellularity status: FALSE**

- EACC1*->

■ **GCF_900111175.1; Actinokineospora terrae; counts= 1; multicellularity status: TRUE**

- EACC1*->CASPASE+??+TM+TM+TM+TM+TM+TM+TM+TM+TM->CASPASE+??+TM+TM+TM+TM+TM+TM+TM+TM->

■ **GCA_003635025.1; Saccharopolyspora antimicrobica; counts= 1; multicellularity status: TRUE**

- EACC1*->CASPASE+FTSK+FTSK+FTSK+FTSK->

■ **GCF_900112555.1; Saccharopolyspora; counts= 1; multicellularity status: TRUE**

- EACC1*->CASPASE+BetaPropeller->

■ **GCA_900112325.1; Verrucosispora sediminis; counts= 1; multicellularity status: TRUE**

- EACC1*->CASPASE+TPRs->

■ **GCF_900105695.1; Streptomyces melanosporofaciens; counts= 1; multicellularity status: TRUE**

- EACC1*->CASPASE+STAND+polar_region+TM+BetaPropeller->

■ **GCA_002797835.1; Streptomyces; counts= 1; multicellularity status: TRUE**

- EACC1*->CASPASE+??->

■ **GCA_003610145.1; Streptomyces sp. 2112.3; counts= 1; multicellularity status: TRUE**

- EACC1*->

■ **GCA_900105755.1; Streptomyces sp. Ag109_O5-10; counts= 1; multicellularity status: TRUE**

- EACC1*->CASPASE+TM+TM+BetaPropeller->

■ **GCA_900113015.1; Actinoplanes philippinensis; counts= 1; multicellularity status: TRUE**

- EACC1*->CASPASE+PBPB+vWA->TM+PBPB->

■ **GCA_900105515.1; Streptomyces sp. 2131.1; counts= 1; multicellularity status: TRUE**

- EACC1*->CASPASE+STAND+polar_region+TM+BetaPropeller->

■ **GCF_900112845.1; Streptomyces alni; counts= 1; multicellularity status: TRUE**

- EACC1*->

■ **GCF_900091855.1; Streptomyces sp. DvalAA-14; counts= 1; multicellularity status: TRUE**

- EACC1*->

■ **GCA_900091875.1; Streptomyces sp. BpilaLS-43; counts= 1; multicellularity status: TRUE**

- EACC1*->CASPASE+??+SF1-helicase+Vsr(PDDEXK)?->

■ **GCF_900079415.1; Streptomyces sp. F-7; counts= 1; multicellularity status: TRUE**

- TM+TM+TM+PP2C->??->EACC1*->CASPASE+TM+TM+PBPB+vWA->

■ **GCA_900103985.1; Streptomyces guanduensis; counts= 1; multicellularity status: TRUE**

- EACC1*->CASPASE+TPRs->

■ **GCF_001049855.1; Streptomyces sp. Wb2n-11; counts= 1; multicellularity status: TRUE**

- EACC1*->CASPASE+TM?+Phytase-like->

■ **GCF_900105395.1; Streptomyces sp. TLI_053; counts= 1; multicellularity status: TRUE**

- FAD_binding_4+FAD-oxidase_C->DAGK_cat+DAGK_acc->EACC1*->CASPASE+TPRs->Clp_N+Clp_N+ClpABN-AAA->

■ **GCF_900103585.1; Streptomyces sp. cf386; counts= 1; multicellularity status: TRUE**

- EACC1*->CASPASE+TPRs->

■ **GCA_002234405.1; Amycolatopsis thailandensis; counts= 1; multicellularity status: TRUE**

- EACC1*->

■ **GCA_002234605.1; Amycolatopsis sp. KNN50.9b; counts= 1; multicellularity status: TRUE**

- EACC1*->CASPASE+low-complexity+BetaPropeller->

■ **GCA_001896135.2; Streptomyces phaeoluteigriseus; counts= 1; multicellularity status: TRUE**

- low-complexity+HD+Tox-RelA+TGS+ACT->Pkinase+TPRs->EACC1*->CASPASE+TPRs->

■ **GCA_002242735.1; Streptomyces sp. 2R; counts= 1; multicellularity status: TRUE**

- EACC1*->CASPASE+TPRs+LysM+PG-binding->

■ **GCA_002234475.1; Salmonella enterica; counts= 1; multicellularity status: FALSE**

- EACC1*->

■ **GCA_003428925.1; Streptomyces; counts= 1; multicellularity status: TRUE**

- EACC1*->CASPASE+Trypsin+TPRs->

■ **GCA_002291145.1; Streptomyces sp. SA15; counts= 1; multicellularity status: TRUE**

- EACC1*->CASPASE+STAND+polar_region+TM+BetaPropeller->

■ **GCF_000220705.2; Streptomyces xinghaiensis; counts= 1; multicellularity status: TRUE**

- EACC1*->CASPASE+PBPB+vWA->

■ **GCA_003633975.1; Streptomyces sp. Ag82_O1-15; counts= 1; multicellularity status: TRUE**

- EACC1*->CASPASE+BetaPropeller->

■ **GCF_001543895.1; Streptomyces thermoautotrophicus; counts= 1; multicellularity status: TRUE**

- EACC1*->CASPASE+STAND+polar_region+TM+BetaPropeller->

■ **GCF_900230215.1; Jatrophihabitans sp. GAS493; counts= 1; multicellularity status: TRUE**

- EACC1*->CASPASE+low-complexity+DUF4190(has2TM)->?->Glyoxalase->

■ **GCA_002354875.1; Actinosynnema pretiosum; counts= 1; multicellularity status: TRUE**

- EACC1*->

■ **GCA_002411735.1; Klebsiella pneumoniae; counts= 1; multicellularity status: FALSE**

- EACC1*->

■ **GCF_900230245.1; Burkholderia sp. OK806; counts= 1; multicellularity status: FALSE**

- EACC1*->Tnp_DNA_bind+DDE_Tnp_1+Dimer_Tnp_Tn5->CASPASE+TPRs->EACC1->CASPASE(fragment)->EACC1->CASPASE+CASPASE+TM+TPRs->CASPASE(fragment)->

■ **GCA_900215615.1; Streptomyces sp. Ag82_G6-1; counts= 1; multicellularity status: TRUE**

- EACC1*->CASPASE+??+TM+TM+TM->

■ **GCA_900215415.1; Nocardia amikacinitolerans; counts= 1; multicellularity status: TRUE**

- EACC1*->CASPASE+TPRs->

■ **GCF_900215565.1; Streptomyces sp. Ag109_G2-15; counts= 1; multicellularity status: TRUE**

- EACC1*->CASPASE+STAND+polar_region+TM+BetaPropeller->TM+Acid_phosphat_B->

■ **GCF_002518865.1; Escherichia coli; counts= 1; multicellularity status: FALSE**

- EACC1*->

■ **GCF_002551245.1; Streptomyces sp. rh34; counts= 1; multicellularity status: TRUE**

- EACC1*->CASPASE+??+SF1-helicase+Vsr(PDDEXK)?->

■ **GCF_002551335.1; Streptomyces sp. ms184; counts= 1; multicellularity status: TRUE**

- EACC1*->CASPASE+TPRs->

■ **GCF_002551135.1; Streptomyces sp. st140; counts= 1; multicellularity status: TRUE**

- EACC1*->CASPASE+TPRs->

■ **GCF_002551215.1; Streptomyces; counts= 1; multicellularity status: TRUE**

- EACC1*->CASPASE+STAND+??+TPRs+??->

■ **GCF_002551345.1; Streptomyces sp. ms115; counts= 1; multicellularity status: TRUE**

- EACC1*->CASPASE+TPRs->

■ **GCF_002551305.1; Streptomyces sp. or20; counts= 1; multicellularity status: TRUE**

- HATPase_c_2->EACC1*->CASPASE+Beta_rich_repeat->

■ **GCF_002551435.1; Streptomyces sp. f150; counts= 1; multicellularity status: TRUE**

- EACC1*->CASPASE+TPRs->

■ **GCF_002551475.1; Streptomyces sp. b62; counts= 1; multicellularity status: TRUE**

- EACC1*->CASPASE+TPRs->

■ **GCA_002554515.1; Nocardia sp. FDAARGOS_372; counts= 1; multicellularity status: TRUE**

- DUF2510(W3)+BicD+DUF4041+T5orf172->EACC1*->CASPASE+TPRs->

■ **GCA_003600245.1; Amycolatopsis sulphurea; counts= 1; multicellularity status: TRUE**

- GATase->ABC-ATPase+DUF4162->cNMP_cyclase+TM+TM+TM+TM+TM+TM->EACC1*->

■ **GCF_002551295.1; Streptomyces sp. or3; counts= 1; multicellularity status: TRUE**

- EACC1*->CASPASE+TPRs->

■ **GCF_002551175.1; Streptomyces sp. st77; counts= 1; multicellularity status: TRUE**

- EACC1*->CASPASE+STAND+polar_region+TM+BetaPropeller->

■ **GCF_002591335.1; Streptomyces malaysiensis; counts= 1; multicellularity status: TRUE**

- TetR_N+TetR_C_6->EACC1*->CASPASE+low-complexity+TM+PBPB->

■ **GCA_900090155.1; Streptomyces sp. ScaeMP-e48; counts= 1; multicellularity status: TRUE**

- EACC1->CASPASE+low-complexity+BetaPropeller___*_##->

■ **GCA_900090175.1; Streptomyces sp. LamerLS-316; counts= 1; multicellularity status: TRUE**

- polar_lead?+TM+TM+TM+polar_tail?+low-complexity->??->EACC1*->CASPASE+TPRs->

■ **GCA_002727115.1; Streptomyces cinnamoneus; counts= 1; multicellularity status: TRUE**

- EACC1*->CASPASE+TM+TM+PBPB+vWA->

■ **GCF_002705975.1; Streptomyces sp. AmelKG-E11A; counts= 1; multicellularity status: TRUE**

- EACC1*->

■ **GCA_002946835.1; Streptomyces; counts= 1; multicellularity status: TRUE**

- EACC1*->CASPASE+TPRs->

■ **GCA_002754655.1; Verrucosispora sp. CNZ293; counts= 1; multicellularity status: TRUE**

- TM+TM+TM+Peptidase_M48+TM+DUF4864+??->EACC1*->

■ **GCF_002754415.1; Micromonospora sp. CNZ299; counts= 1; multicellularity status: TRUE**

- EACC1*->

■ **GCF_002754595.1; Streptomyces sp. 93; counts= 1; multicellularity status: TRUE**

- EACC1*->

■ **GCA_002277855.1; Streptomyces sp. Alain-F2R5; counts= 1; multicellularity status: TRUE**

- low-complexity+HD+Tox-RelA+TGS+ACT->Pkinase+TPRs->EACC1*->CASPASE+TPRs->

■ **GCA_002803075.1; Streptomyces sp. CB01373; counts= 1; multicellularity status: TRUE**

- EACC1*->CASPASE+TM+TM+PBPB+vWA->

■ **GCA_002803085.1; Streptomyces sp. CB01201; counts= 1; multicellularity status: TRUE**

- EACC1*->CASPASE+STAND+polar_region+TM+BetaPropeller->

■ **GCA_002803145.1; Kitasatospora sp. CB02891; counts= 1; multicellularity status: TRUE**

- EACC1*->CASPASE+PBPB+vWA->

■ **GCF_002812405.1; Streptomyces sp. M56; counts= 1; multicellularity status: TRUE**

- TetR_N+TetR_C_6->EACC1*->CASPASE+low-complexity+TM+PBPB->

■ **GCF_002813405.1; Streptomyces sp. 69; counts= 1; multicellularity status: TRUE**

- EACC1*->CASPASE+STAND->TM+BetaPropeller->TM+Acid_phosphat_B->

■ **GCA_002813165.1; Streptomyces sp. 4121.5; counts= 1; multicellularity status: TRUE**

- EACC1*->CASPASE+TM+TM+??+TM+TM+??+TM+TM+??->

■ **GCA_002844215.1; Streptomyces sp. EAG2; counts= 1; multicellularity status: TRUE**

- EACC1*->CASPASE+TPRs->

■ **GCF_002846295.1; Streptomyces sp. 59; counts= 1; multicellularity status: TRUE**

- EACC1*->CASPASE+STAND+polar_region+TM+BetaPropeller->TM+Acid_phosphat_B->

■ **GCF_002846355.1; Streptomyces sp. GP55; counts= 1; multicellularity status: TRUE**

- EACC1*->CASPASE+??+STAND+??->

■ **GCF_002846415.1; Streptomyces sp. TLI_146; counts= 1; multicellularity status: TRUE**

- EACC1*->CASPASE+low-complexity+BetaPropeller->

■ **GCF_002846315.1; Streptomyces sp. 73; counts= 1; multicellularity status: TRUE**

- EACC1*->CASPASE+STAND+polar_region+TM+BetaPropeller->

■ **GCA_002847285.1; Streptomyces sp. CMB-StM0423; counts= 1; multicellularity status: TRUE**

- EACC1+CASPASE+Phytase-like*->Phytase-like->||<-METHYLASE

■ **GCA_002846615.1; Amycolatopsis niigatensis; counts= 1; multicellularity status: TRUE**

- EACC1*->

■ **GCA_002846365.1; Nocardia fluminea; counts= 1; multicellularity status: TRUE**

- EACC1*->CASPASE+FTSK+FTSK+FTSK->

■ **GCF_002849735.1; Amycolatopsis sp. BJA-103; counts= 1; multicellularity status: TRUE**

- EACC1*->TM+ZU5+??->

■ **GCA_002853675.1; Klebsiella quasipneumoniae; counts= 1; multicellularity status: FALSE**

- EACC1*->

■ **GCA_002891315.1; Streptomyces noursei; counts= 1; multicellularity status: TRUE**

- low-complexity+HD+Tox-RelA+TGS+ACT->Pkinase+TPRs->EACC1*->CASPASE+TPRs->

■ **GCA_002891865.1; Streptomyces malaysiensis; counts= 1; multicellularity status: TRUE**

- TetR_N+TetR_C_6->EACC1*->CASPASE+low-complexity+TM+PBPB->

■ **GCF_002904295.1; Amycolatopsis sp. CA-128772; counts= 1; multicellularity status: TRUE**

- GATase->ABC-ATPase+DUF4162->cNMP_cyclase+TM+TM+TM+TM+TM+TM->EACC1*->

■ **GCA_002910775.2; Streptomyces; counts= 1; multicellularity status: TRUE**

- EACC1*->CASPASE+TPRs->

■ **GCA_001577385.1; Streptomyces; counts= 1; multicellularity status: TRUE**

- TM+REase->EACC1*->CASPASE+??+SF1-helicase+Vsr(PDDEXK)?->

■ **GCF_002920535.1; Streptomyces sp. Ru71; counts= 1; multicellularity status: TRUE**

- EACC1*->CASPASE+TPRs->Clp_N+Clp_N+ClpABN-AAA->

■ **GCA_002920615.1; Streptomyces sp. Ru62; counts= 1; multicellularity status: TRUE**

- EACC1*->CASPASE+TM?+Phytase-like->MDMPI_N+MDMPI_C->META->

■ **GCF_002920575.1; Streptomyces sp. Ru73; counts= 1; multicellularity status: TRUE**

- EACC1*->

■ **GCA_900099115.2; Thiotrichales bacterium HS_08; counts= 1; multicellularity status: TRUE**

- RVT_1->?->EACC1*->

■ **GCA_002933535.1; Nocardia nova; counts= 1; multicellularity status: TRUE**

- EACC1*->CASPASE+TPRs->

■ **GCF_002934265.1; Actinokineospora auranticolor; counts= 1; multicellularity status: TRUE**

- EACC1*->CASPASE+TM+TM->TM+PBPB->

■ **GCA_003002815.1; Umezawaea tangerina; counts= 1; multicellularity status: TRUE**

- EACC1*->CASPASE+TM+TM+TM+TM->

■ **GCF_003001935.1; Nonomuraea fuscirosea; counts= 1; multicellularity status: TRUE**

- EACC1*->CASPASE+PBPB->

■ **GCA_003011965.1; Streptosporangium nondiastaticum; counts= 1; multicellularity status: TRUE**

- EACC1*->CASPASE+STAND+polar_region+TM+BetaPropeller->

■ **GCA_003023875.1; Nocardia sp. MDA0666; counts= 1; multicellularity status: TRUE**

- EACC1*->CASPASE+TM+TM+TM+TM->

■ **GCF_000156695.2; Streptomyces; counts= 1; multicellularity status: TRUE**

- EACC1*->

■ **GCA_003013775.1; Micromonospora sp. MH33; counts= 1; multicellularity status: TRUE**

- EACC1*->

■ **GCF_000412265.2; Streptomyces sp. Mg1; counts= 1; multicellularity status: TRUE**

- EACC1*->CASPASE+STAND+polar_region+TM+BetaPropeller->

■ **GCF_001279685.1; Streptomyces sp. WM6373; counts= 1; multicellularity status: TRUE**

- EACC1*->CASPASE+??+SF1-helicase+Vsr(PDDEXK)?->

■ **GCF_001426405.1; Streptomyces; counts= 1; multicellularity status: TRUE**

- low_complexity+EACC1*->CASPASE+STAND+polar_region+TM+BetaPropeller->||<-MFS_1

■ **GCA_003030385.1; Plantactinospora sp. BB1; counts= 1; multicellularity status: TRUE**

- EACC1*->CASPASE+TPRs->

■ **GCA_003030345.1; Plantactinospora sp. BC1; counts= 1; multicellularity status: TRUE**

- EACC1*->CASPASE+TPRs->

■ **GCF_001590865.1; Streptomyces sp. NBRC 110611; counts= 1; multicellularity status: TRUE**

- EACC1*->

■ **GCF_003041235.1; Streptomyces sp. A244; counts= 1; multicellularity status: TRUE**

- EACC1*->CASPASE+??->

■ **GCF_002251155.1; Streptomyces sp. FBKL.4005; counts= 1; multicellularity status: TRUE**

- EACC1*->CASPASE+TM?+DUF3558+DUF3558->

■ **GCF_900269665.1; Nocardia suismassiliense; counts= 1; multicellularity status: TRUE**

- EACC1*->CASPASE+STAND+BetaPropeller+BetaPropeller->

■ **GCA_003097515.1; Streptomyces sp. HNM0039; counts= 1; multicellularity status: TRUE**

- EACC1*->CASPASE+STAND+??->

■ **GCA_003116775.1; Streptomyces sp. CS147; counts= 1; multicellularity status: TRUE**

- EACC1*->CASPASE+STAND+polar_region+TM+BetaPropeller->

■ **GCA_003116715.1; Streptomyces sp. CS081A; counts= 1; multicellularity status: TRUE**

- EACC1*->CASPASE+STAND+polar_region+TM+BetaPropeller->

■ **GCF_003121295.1; Streptomyces orinoci; counts= 1; multicellularity status: TRUE**

- EACC1*->CASPASE+STAND+polar_region+TM+BetaPropeller->

■ **GCF_001756925.1; Streptomyces xinghaiensis; counts= 1; multicellularity status: TRUE**

- EACC1*->CASPASE+PBPB+vWA->

■ **GCA_003143855.1; Streptomyces sp. NWU339; counts= 1; multicellularity status: TRUE**

- EACC1*->CASPASE+STAND+polar_region+TM+BetaPropeller->

■ **GCA_003143935.1; Streptomyces sp. Act143; counts= 1; multicellularity status: TRUE**

- EACC1*->CASPASE+TPRs->

■ **GCF_003121585.1; Nocardioides sp. YIM ART13; counts= 1; multicellularity status: TRUE**

- EACC1*->

■ **GCF_003123685.1; Nocardia sp. SYSU K10002; counts= 1; multicellularity status: TRUE**

- EACC1+CASPASE*->

■ **GCA_003148865.1; Lechevalieria deserti; counts= 1; multicellularity status: TRUE**

- TM+TM+BPD_transp_1->OppC_N+TM+TM+BPD_transp_1->ABC-ATPase+ABC-ATPase->PBPB->TM+Beta_rich+TM+Beta_rich+TM+TM->EACC1+CASPASE+TPR_repeats+low_complexity+TM_region*->

■ **GCA_006471505.1; Escherichia coli; counts= 1; multicellularity status: FALSE**

- EACC1*->

■ **GCA_003176715.1; Micromonospora sp. S4605; counts= 1; multicellularity status: TRUE**

- TM+TM+TM+Peptidase_M48+TM+DUF4864+??->EACC1*->TPRs+CASPASE->

■ **GCF_003182135.1; Nocardia neocaledoniensis; counts= 1; multicellularity status: TRUE**

- EACC1*->

II. Source data - domain architectures, genome identifiers, gene neighborhoods

VMAP-ternary systems

<table>
<thead>
<tr><th>acc</th><th>architecture</th><th>operon</th><th>len</th><th>species</th><th>gca</th><th>taxend</th></tr>
</thead>
<tbody>
<tr><td>AAZ54441.1</td><td>vWA+APATPase+wHTH+TPRs</td><td>Trypsin+EAD2→ EAD2+VMAP-M0+VMAP-C→ MoxR→ vWA+APATPase+wHTH+TPRs*→ Trypco1→</td><td>1523</td><td>Thermobifida fusca YX</td><td>GCA_000012405.1</td><td>actinobacteria</td></tr>
<tr><td>ABG51370.1</td><td>vWA+HTH+HTH+HTH+HTH+HTH+HTH+HTH+HTH+HTH+FGS-fold_domain</td><td>CASPASE+VMAP-C→ MoxR→ ?→ vWA(partial)→ vWA+HTH+HTH+HTH+HTH+HTH+HTH+HTH+HTH+HTH+FGS-fold_domain*→</td><td>1911</td><td>Trichodesmium erythraeum IMS101</td><td>GCA_000014265.1</td><td>cyanobacteria</td></tr>
<tr><td>ABG51457.1</td><td>vWA+FGS-fold_domain+FGS-fold_domain</td><td>EAD6+VMAP-M12+VMAP-C→ MoxR→ vWA+FGS-fold_domain+FGS-fold_domain*→</td><td>1196</td><td>Trichodesmium erythraeum IMS101</td><td>GCA_000014265.1</td><td>cyanobacteria</td></tr>
<tr><td>ABG53299.1</td><td>vWA+FGS-fold_domain</td><td>EAD1+Trypsin→ ?→ EAD1+VMAP-M0+VMAP-C→ MoxR→ ?→ ||← ?||vWA+FGS-fold_domain*→</td><td>862</td><td>Trichodesmium erythraeum IMS101</td><td>GCA_000014265.1</td><td>cyanobacteria</td></tr>
<tr><td>ABW16290.1</td><td>vWA+APATPase+wHTH+TPRs</td><td>EAD2+EAD1→ EAD2+VMAP-M0+VMAP-C→ MoxR→ vWA+APATPase+wHTH+TPRs*→ ||← METALLOPROTEASE+TPRs</td><td>1500</td><td>Frankia sp. EAN1pec</td><td>GCA_000018005.1</td><td>actinobacteria</td></tr>
<tr><td>ABW33201.1</td><td>vWA+FGS-fold_domain</td><td>Trypco1→ Trypsin+VMAP-M1+VMAP-C→ MoxR→ vWA+FGS-fold_domain*→ FGS-fold_domain→ FGS-fold_domain→</td><td>955</td><td>Acaryochloris marina MBIC11017</td><td>GCA_000018105.1</td><td>cyanobacteria</td></tr>
<tr><td>ACC85234.1</td><td>vWA+Calcineurin-like+SAVED</td><td>VMAP-M1+VMAP-C→ MoxR→ vWA+Calcineurin-like+SAVED*→</td><td>1237</td><td>Nostoc punctiforme PCC 73102</td><td>GCA_000020025.1</td><td>cyanobacteria</td></tr>
<tr><td>ACL45435.1</td><td>vWA</td><td>FGS-fold_domain← ?← ?← ?||VMAP-M1+VMAP-C→ MoxR→ vWA*→ ||← zf-IS66+DDE_Tnp_IS66← TIR+EAD9← ?← Trypsin← Trypco1</td><td>745</td><td>Cyanothece sp. PCC 7425</td><td>GCA_000022045.1</td><td>cyanobacteria</td></tr>
<tr><td>ACY97176.1</td><td>vWA+APATPase+wHTH+TPRs</td><td>Radical_SAM→ METALLOPROTEASE+TPRs→ ||← TIR+APATPase+TPRs||EAD2+VMAP-M0+VMAP-C→ MoxR→ vWA+APATPase+wHTH+TPRs*→</td><td>1383</td><td>Thermomonospora curvata DSM 43183</td><td>GCA_000024385.1</td><td>actinobacteria</td></tr>
<tr><td>ADH69904.1</td><td>vWA+APATPase+wHTH+TPRs</td><td>Trypsin+EAD2→ EAD2+VMAP-M0+VMAP-C→ MoxR→ vWA+APATPase+wHTH+TPRs*→ ||← DUF397</td><td>1509</td><td>Nocardiopsis dassonvillei subsp. dassonvillei DSM 43111</td><td>GCA_000092985.1</td><td>actinobacteria</td></tr>
<tr><td>ADI03786.1</td><td>vWA+APATPase+wHTH+TPRs</td><td>EAD2+VMAP-M0+VMAP-C→ MoxR→ vWA+APATPase+wHTH+TPRs*→ ||← METALLOPROTEASE+TPRs← FXSXX-COOH-like← ?← ?← Na_H_Exchanger</td><td>1376</td><td>Streptomyces bingchenggensis BCW-1</td><td>GCA_000092385.1</td><td>actinobacteria</td></tr>
<tr><td>ADO19142.1</td><td>vWA</td><td>vWA*→ ?→ ?→ FGS-fold_domain→</td><td>609</td><td>Nostoc flagelliforme str. Sunitezuoqi</td><td>-</td><td>cyanobacteria</td></tr>
<tr><td>AEV84216.1</td><td>vWA+TPRs</td><td>EAD1+VMAP-M0+VMAP-C→ MoxR→ vWA+TPRs*→</td><td>915</td><td>Actinoplanes sp. SE50/110</td><td>GCA_000237145.1</td><td>actinobacteria</td></tr>
<tr><td>AFY58464.1</td><td>vWA+FGS-fold_domain</td><td>EAD1+VMAP-M1+VMAP-C→ MoxR→ ?→ vWA+FGS-fold_domain*→ FGS-fold_domain→</td><td>879</td><td>Rivularia sp. PCC 7116</td><td>GCA_000316665.1</td><td>cyanobacteria</td></tr>
<tr><td>AFZ01290.1</td><td>vWA+AP-GTPase+COR</td><td>vWA+MNS+CASPASE+MNS← MoxR← EAD10+VMAP-M1+VMAP-C||?→ CASPASE→ EAD10+VMAP-M1+VMAP-C→ MoxR→ vWA+AP-GTPase+COR*→</td><td>1196</td><td>Calothrix sp. PCC 6303</td><td>GCA_000317435.1</td><td>cyanobacteria</td></tr>
<tr><td>AGI87916.1</td><td>vWA+TPRs</td><td>TCAD15||Trypco1→ Trypsin+HDxxH+VMAP-M0+VMAP-C→ MoxR→ vWA+TPRs*→ FXSXX-COOH→ Radical_SAM→ METALLOPROTEASE+TPRs→ TIR+APATPase+TPRs→</td><td>1108</td><td>Streptomyces albus J1074</td><td>GCA_000359525.1</td><td>actinobacteria</td></tr>
<tr><td>AGM04021.1</td><td>vWA+APATPase+wHTH+TPRs</td><td>VMAP-M0+VMAP-C→ MoxR→ vWA+APATPase+wHTH+TPRs*→ ||← METALLOPROTEASE+TPRs← FXSXX-COOH-like← cNMP_binding+HTH_Crp_2</td><td>1449</td><td>Amycolatopsis orientalis HCCB10007</td><td>GCA_000400635.2</td><td>actinobacteria</td></tr>
<tr><td>AGP53016.1</td><td>vWA+APATPase+wHTH+TPRs</td><td>PNP_UDP_1+EAD2→ EAD2+VMAP-M0+VMAP-C→ MoxR→ vWA+APATPase+wHTH+TPRs*→ ||← METALLOPROTEASE+TPRs← FXSXX-COOH-like</td><td>1194</td><td>Streptomyces rapamycinicus NRRL 5491</td><td>GCA_000418455.1</td><td>actinobacteria</td></tr>
<tr><td>AGS71776.1</td><td>vWA+TPRs</td><td>TCAD15→ Trypco1→ Trypsin+HDxxH+VMAP-M0+VMAP-C→ MoxR→ vWA+TPRs*→ FXSXX-COOH-like→ METALLOPROTEASE+TPRs→ TIR+APATPase+TPRs→</td><td>1323</td><td>Streptomyces collinus Tu 365</td><td>GCA_000444875.1</td><td>actinobacteria</td></tr>
<tr><td>AHH96337.1</td><td>vWA+TIR+BetaPropeller</td><td>EAD2+VMAP-M0+VMAP-C→ MoxR→ ?→ vWA+TIR+BetaPropeller*→ ?→ ?→ ||← cNMP_cyclase+EAD2||cNMP_binding+HTH_Crp_2→ ?→ SIR2→</td><td>1185</td><td>Kutzneria albida DSM 43870</td><td>GCA_000525635.1</td><td>actinobacteria</td></tr>
<tr><td>AHJ30118.1</td><td>vWA+beta-rich-region+HTH+FGS-fold_domain</td><td>TIR+EAD6→ EAD6+VMAP-M5→ VMAP-C→ MoxR→ vWA+beta-rich-region+HTH+FGS-fold_domain*→ four_helix_bundle_protein→ RVT_1→</td><td>1076</td><td>Nodularia spumigena CCY9414</td><td>GCA_000340565.3</td><td>cyanobacteria</td></tr>
<tr><td>AIG79293.1</td><td>vWA+APATPase+wHTH+TPRs</td><td>cNMP_cyclase+VMAP-M0+VMAP-C→ MoxR→ vWA+APATPase+wHTH+TPRs*→ ||← METALLOPROTEASE+TPRs← FXSXX-COOH-like← cNMP_binding+HTH_Crp_2</td><td>1458</td><td>Amycolatopsis japonica</td><td>GCA_000732925.1</td><td>actinobacteria</td></tr>
<tr><td>AIJ13394.1</td><td>vWA+TPRs</td><td>TCAD15||Trypco1→ Trypsin+HDxxH+VMAP-M0+VMAP-C→ MoxR→ vWA+TPRs*→ FXSXX-COOH→ Radical_SAM→ METALLOPROTEASE+TPRs→ TIR+APATPase+TPRs→ Trypco1→</td><td>1147</td><td>Streptomyces lividans TK24</td><td>GCA_000739105.1</td><td>actinobacteria</td></tr>
<tr><td>AIS01610.1</td><td>vWA+TIR+APATPase+wHTH+TPRs</td><td>DRHyd+VMAP-M0+VMAP-C→ MoxR→ vWA+TIR+APATPase+wHTH+TPRs*→</td><td>1416</td><td>Streptomyces glaucescens</td><td>GCA_000761215.1</td><td>actinobacteria</td></tr>
<tr><td>AIV38673.1</td><td>vWA+TPRs</td><td>TCAD15||Trypco1→ Trypsin+HDxxH+VMAP-M0+VMAP-C→ MoxR→ vWA+TPRs*→ FXSXX-COOH→ Radical_SAM→ METALLOPROTEASE+TPRs→ TIR+APATPase+TPRs→ Trypco1→</td><td>1102</td><td>Streptomyces sp. CCM_MD2014</td><td>GCA_000772045.1</td><td>actinobacteria</td></tr>
<tr><td>AJK55727.1</td><td>vWA+APATPase+wHTH+TPRs</td><td>cNMP_cyclase+VMAP-M0+VMAP-C→ MoxR→ vWA+APATPase+wHTH+TPRs*→ ||← ?||?→ ||← FXSXX-COOH-like← cNMP_binding+HTH_Crp_2</td><td>1463</td><td>Amycolatopsis lurida NRRL 2430</td><td>GCA_000749465.2</td><td>actinobacteria</td></tr>
<tr><td>AJP03761.1</td><td>vWA+TPRs</td><td>TCAD15||Trypco1→ Trypsin+HDxxH+VMAP-M0+VMAP-C→ MoxR→ vWA+TPRs*→ FXSXX-COOH→ METALLOPROTEASE+TPRs→ TIR+APATPase+TPRs→</td><td>1118</td><td>Streptomyces cyaneogriseus subsp. noncyanogenus</td><td>GCA_000931445.1</td><td>actinobacteria</td></tr>
<tr><td>AKI98917.1</td><td>vWA+SLOG+SLOG+TPRs</td><td>EAD1+VMAP-M1+VMAP-C→ MoxR→ vWA+SLOG+SLOG+TPRs*→ EAD1+Trypsin→</td><td>1959</td><td>Archangium gephyra</td><td>GCA_001027285.1</td><td>deltaproteobacteria</td></tr>
<tr><td>AKJ08455.1</td><td>vWA+SLOG</td><td>EAD1+VMAP-M1+VMAP-C→ vWA+SLOG*→</td><td>1054</td><td>Archangium gephyra</td><td>GCA_001027285.1</td><td>deltaproteobacteria</td></tr>
<tr><td>AKJ10474.1</td><td>vWA+APATPase+wHTH+TPRs</td><td>Trypco2→ EAD2+VMAP-M0+VMAP-C→ vWA+APATPase+wHTH+TPRs*→ ||← FXSXX-COOH-like← Trypsin+EAD2||?→ ?→ ?→ ?→ DUF397→</td><td>1201</td><td>Streptomyces incarnatus</td><td>GCA_001027185.1</td><td>actinobacteria</td></tr>
</tbody>
</table>

<table>
<tr><th>acc</th><th>architecture</th><th>operon</th><th>len</th><th>species</th><th>gca</th><th>taxend</th></tr>
<tr><td>AKN68644.1</td><td>vWA+TPRs</td><td>TCAD15→ Trypco1→ Trypsin+HDxxH+VMAP-M0+VMAP-C→ MoxR→ vWA+TPRs*→ FXSXX-COOH-like→</td><td>1261</td><td>Streptomyces sp. PBH53</td><td>GCA_001040905.1</td><td>actinobacteria</td></tr>
<tr><td>AKN69695.1</td><td>vWA+wHTH+cNMP_cyclase+Band7+HAD</td><td>Trypco1→ Trypsin+VMAP-M0+VMAP-C→ MoxR→ vWA+wHTH+cNMP_cyclase+Band7+HAD*→</td><td>1303</td><td>Streptomyces sp. PBH53</td><td>GCA_001040905.1</td><td>actinobacteria</td></tr>
<tr><td>AKV66222.1</td><td>vWA+FGS-fold_domain</td><td>AB_hydrolase→ CASPASE+VMAP-M14+VMAP-C→ MoxR→ vWA+FGS-fold_domain*→</td><td>842</td><td>Microcystis panniformis FACHB-1757</td><td>GCA_001264245.1</td><td>cyanobacteria</td></tr>
<tr><td>AKV67733.1</td><td>vWA+FGS-fold_domain</td><td>HTH+VMAP-M9+VMAP-C→ MoxR→ vWA+FGS-fold_domain*→ ?→ ||← ?||FGS-fold_domain→</td><td>961</td><td>Microcystis panniformis FACHB-1757</td><td>GCA_001264245.1</td><td>cyanobacteria</td></tr>
<tr><td>AKV70250.1</td><td>vWA+FGS-fold_domain</td><td>Trypco1→ Trypsin+VMAP-M1+VMAP-C→ MoxR→ vWA+FGS-fold_domain*→ ?→ ||← ?← ?||FGS-fold_domain→</td><td>884</td><td>Microcystis panniformis FACHB-1757</td><td>GCA_001264245.1</td><td>cyanobacteria</td></tr>
<tr><td>ALB40811.1</td><td>vWA+FGS-fold_domain</td><td>EAD6+VMAP-C→ MoxR→ vWA+FGS-fold_domain*→ FGS-fold_domain→</td><td>880</td><td>Anabaena sp. WA102</td><td>GCA_001277295.1</td><td>cyanobacteria</td></tr>
<tr><td>ALC19574.1</td><td>vWA+Tubulin</td><td>Trypco1→ Trypsin+VMAP-M7+VMAP-C→ MoxR→ vWA+Tubulin*→</td><td>1629</td><td>Streptomyces pristinaespiralis</td><td>GCA_001278075.1</td><td>actinobacteria</td></tr>
<tr><td>ALC31298.1</td><td>vWA+REase+SWACOS+TPRs+cNMP_cyclase</td><td>CASPASE+VMAP-M2+VMAP-C→ MoxR→ vWA+REase+SWACOS+TPRs+cNMP_cyclase*→</td><td>1713</td><td>Streptomyces sp. CFMR 7</td><td>GCA_001278095.1</td><td>actinobacteria</td></tr>
<tr><td>ALG12738.1</td><td>vWA+APATPase+wHTH+TPRs</td><td>EAD2+VMAP-M0+VMAP-C→ MoxR→ vWA+APATPase+wHTH+TPRs→ ||← METALLOPROTEASE+TPRs← FXSXX-COOH-like||EAD2+VMAP-M0+VMAP-C→ MoxR→ vWA+APATPase+wHTH+TPRs*→ ||← METALLOPROTEASE+TPRs← FXSXX-COOH-like</td><td>1395</td><td>Kibdelosporangium phytohabitans</td><td>GCA_001302585.1</td><td>actinobacteria</td></tr>
<tr><td>ALM38630.1</td><td>vWA+TPRs</td><td>low-complexity+HDxxH+VMAP-M0→ VMAP-C→ MoxR→ vWA+TPRs*→ FXSXX-COOH→ ?→ METALLOPROTEASE+TPRs→ TIR+APATPase+TPRs→</td><td>1137</td><td>Streptomyces sp. FR-008</td><td>GCA_001431765.1</td><td>actinobacteria</td></tr>
<tr><td>ALO96113.1</td><td>vWA+TPRs</td><td>TCAD15→ Trypco1→ Trypsin+HDxxH+VMAP-M0+VMAP-C→ MoxR→ vWA+TPRs*→ FXSXX-COOH-like→ METALLOPROTEASE+TPRs→ TIR+APATPase+TPRs→</td><td>1160</td><td>Streptomyces hygroscopicus subsp. limoneus</td><td>GCA_001447075.1</td><td>actinobacteria</td></tr>
<tr><td>ALV33603.1</td><td>vWA+TPRs</td><td>TCAD15||Trypco1→ Trypsin+HDxxH+VMAP-M0+VMAP-C→ MoxR→ vWA+TPRs*→ FXSXX-COOH→ Radical_SAM→ METALLOPROTEASE+TPRs→ TIR+APATPase+TPRs→</td><td>1125</td><td>Streptomyces sp. CdTB01</td><td>GCA_001484565.1</td><td>actinobacteria</td></tr>
<tr><td>AMM08319.1</td><td>vWA+TPRs</td><td>TCAD15||Trypco1→ VMAP-C→ MoxR→ vWA+TPRs*→ FXSXX-COOH→ Radical_SAM→ METALLOPROTEASE+TPRs→ TIR+APATPase+TPRs→</td><td>1039</td><td>Streptomyces albus</td><td>GCA_001577385.1</td><td>actinobacteria</td></tr>
<tr><td>AMV25224.1</td><td>EAD1+HD+vWA+NEFF1</td><td>EAD1+HD← EAD1+Trypsin||VMAP-M21+VMAP-C→ MoxR→ EAD1+HD+vWA+NEFF1*→</td><td>1336</td><td>Gemmata sp. SH-PL17</td><td>GCA_001610855.1</td><td>planctomycetes</td></tr>
<tr><td>AMW10311.1</td><td>vWA+Pkinase+wHTH+TPRs+cNMP_cyclase+Band7+HAD</td><td>Trypco1→ Trypsin+VMAP-M0+VMAP-C→ MoxR→ vWA+Pkinase+wHTH+TPRs+cNMP_cyclase+Band7+HAD*→</td><td>1423</td><td>Streptomyces sp. S10(2016)</td><td>GCA_001611795.1</td><td>actinobacteria</td></tr>
<tr><td>AMW13454.1</td><td>vWA+APATPase+wHTH+TPRs</td><td>EAD2+VMAP-M0+VMAP-C→ MoxR→ vWA+APATPase+wHTH+TPRs*→ AB_hydrolase→ CASPASE+EAD2→ FXSXX-COOH-like→ METALLOPROTEASE+TPRs→</td><td>1500</td><td>Streptomyces sp. S10(2016)</td><td>GCA_001611795.1</td><td>actinobacteria</td></tr>
<tr><td>ANJ11717.1</td><td>vWA+APATPase+wHTH+TPRs</td><td>Trypco2→ EAD2+VMAP-M0+VMAP-C→ vWA+APATPase+wHTH+TPRs*→ ||← METALLOPROTEASE+TPRs← FXSXX-COOH-like← Trypsin+EAD2</td><td>1201</td><td>Streptomyces parvulus</td><td>GCA_001660045.1</td><td>actinobacteria</td></tr>
<tr><td>ANL87877.1</td><td>vWA+REC+SLOG+CASPASE</td><td>EAD5+Trypsin→ EAD5+VMAP-M4+VMAP-C→ MoxR→ vWA+REC+SLOG+CASPASE*→</td><td>1509</td><td>Rhizobium phaseoli</td><td>GCA_001664405.1</td><td>alphaproteobacteria</td></tr>
<tr><td>ANN22215.1</td><td>vWA+APATPase+wHTH+TPRs</td><td>cNMP_cyclase+VMAP-M0+VMAP-C→ MoxR→ vWA+APATPase+wHTH+TPRs*→ ||← METALLOPROTEASE+TPRs← FXSXX-COOH-like← cNMP_binding+HTH_Crp_2</td><td>1202</td><td>Amycolatopsis orientalis</td><td>GCA_000943515.2</td><td>actinobacteria</td></tr>
<tr><td>ANP56390.1</td><td>vWA+APATPase+wHTH+TPRs</td><td>Trypco2→ EAD2+VMAP-M0+VMAP-C→ vWA+APATPase+wHTH+TPRs*→ ||← METALLOPROTEASE+TPRs← Trypsin+EAD2||?→ Trypco2→</td><td>1201</td><td>Streptomyces griseochromogenes</td><td>GCA_001542625.2</td><td>actinobacteria</td></tr>
<tr><td>ANS63696.1</td><td>vWA+APATPase+wHTH+TPRs</td><td>EAD2+VMAP-M0+VMAP-C→ MoxR→ vWA+APATPase+wHTH+TPRs*→ ||← CASPASE+EAD2</td><td>1315</td><td>Streptomyces lincolnensis</td><td>GCA_001685355.1</td><td>actinobacteria</td></tr>
<tr><td>ANS68576.1</td><td>vWA+APATPase+wHTH+TPRs</td><td>EAD2+VMAP-M0+VMAP-C→ MoxR→ vWA+APATPase+wHTH+TPRs*→ ||← Na_H_Exchanger||AB_hydrolase→ CASPASE+EAD2→ FXSXX-COOH-like→ METALLOPROTEASE+TPRs→</td><td>1508</td><td>Streptomyces lincolnensis</td><td>GCA_001685355.1</td><td>actinobacteria</td></tr>
<tr><td>ANS69559.1</td><td>vWA+SFII-helicase+Z1</td><td>AB_hydrolase→ CASPASE+VMAP-M2+VMAP-C→ MoxR→ vWA+SFII-helicase+Z1*→</td><td>1269</td><td>Streptomyces lincolnensis</td><td>GCA_001685355.1</td><td>actinobacteria</td></tr>
<tr><td>ANS70744.1</td><td>vWA+STAND_NTPase+Trypsin+TIR+CASPASE+EAD2</td><td>AB_hydrolase→ CASPASE+VMAP-M0+VMAP-C→ MoxR→ vWA+STAND_NTPase+Trypsin+TIR+CASPASE+EAD2*→</td><td>1564</td><td>Streptomyces lincolnensis</td><td>GCA_001685355.1</td><td>actinobacteria</td></tr>
<tr><td>AOR30905.1</td><td>vWA+APATPase+wHTH+TPRs</td><td>Trypsin+EAD2+VMAP-M0+VMAP-C→ vWA+APATPase+wHTH+TPRs*→</td><td>779</td><td>Streptomyces puniciscabiei</td><td>GCA_001735805.1</td><td>actinobacteria</td></tr>
<tr><td>AOR34174.1</td><td>vWA+Pkinase+ParA-Soj-PloopNTPase(SIMIBI)</td><td>Trypco1→ Trypsin+EAD2+VMAP-M0+VMAP-C→ MoxR→ vWA+Pkinase+ParA-Soj-PloopNTPase(SIMIBI)*→</td><td>1190</td><td>Streptomyces puniciscabiei</td><td>GCA_001735805.1</td><td>actinobacteria</td></tr>
<tr><td>AOT62241.1</td><td>vWA+APATPase+wHTH+TPRs+cNMP_cyclase+NaeI</td><td>Trypco1→ ?→ ?→ AB_hydrolase→ CASPASE+VMAP-M2+VMAP-C→ MoxR→ vWA+APATPase+wHTH+TPRs+cNMP_cyclase+NaeI*→</td><td>1810</td><td>Streptomyces rubrolavendulae</td><td>GCA_001750785.1</td><td>actinobacteria</td></tr>
<tr><td>APC37890.1</td><td>vWA+APATPase+wHTH+TPRs</td><td>Trypsin+EAD2→ EAD2+VMAP-M0+VMAP-C→ MoxR→ vWA+APATPase+wHTH+TPRs*→ ||← DUF397</td><td>1509</td><td>Nocardiopsis dassonvillei</td><td>GCA_001877055.1</td><td>actinobacteria</td></tr>
<tr><td>APU44607.1</td><td>vWA+TPRs</td><td>TCAD15||Trypsin+HDxxH+VMAP-M0+VMAP-C→ MoxR→ vWA+TPRs*→ FXSXX-COOH→ Radical_SAM→ METALLOPROTEASE+TPRs→ TIR+APATPase+TPRs→</td><td>1088</td><td>Streptomyces sp. TN58</td><td>GCA_001941845.1</td><td>actinobacteria</td></tr>
<tr><td>AQA11755.1</td><td>vWA+TPRs</td><td>AB_hydrolase→ CASPASE+EAD2+VMAP-M0+VMAP-C→ MoxR→ vWA+TPRs*→</td><td>1171</td><td>Streptomyces autolyticus</td><td>GCA_001983975.1</td><td>actinobacteria</td></tr>
<tr><td>AQT76777.1</td><td>vWA+TPRs</td><td>TCAD15||Trypsin+HDxxH+VMAP-M0+VMAP-C→ MoxR→ vWA+TPRs*→ FXSXX-COOH→ Radical_SAM→ METALLOPROTEASE+TPRs→ TIR+APATPase+TPRs→</td><td>1093</td><td>Streptomyces sp. fd1-xmd</td><td>GCA_002007685.1</td><td>actinobacteria</td></tr>
<tr><td>ARF57533.1</td><td>vWA+APATPase+wHTH+TPRs</td><td>Trypco1→ Trypsin+EAD2+VMAP-M0+VMAP-C→ MoxR→ vWA+APATPase+wHTH+TPRs*→</td><td>1334</td><td>Streptomyces gilvosporeus</td><td>GCA_002082195.1</td><td>actinobacteria</td></tr>
<tr><td>ARH93170.1</td><td>vWA+TPRs</td><td>TCAD15||Trypco1→ PSE→ MoxR→ vWA+TPRs*→ FXSXX-COOH→ Radical_SAM→ TIR+APATPase+TPRs→</td><td>1134</td><td>Streptomyces sp. MOE7</td><td>GCA_002090335.1</td><td>actinobacteria</td></tr>
<tr><td>ARH94837.1</td><td>vWA</td><td>EAD2+VMAP-M0+VMAP-C→ vWA(partial)→ vWA*→</td><td>335</td><td>Streptomyces sp. MOE7</td><td>GCA_002090335.1</td><td>actinobacteria</td></tr>
<tr><td>ARQ68581.1</td><td>vWA+TPRs</td><td>TCAD15||PSE→ Trypsin+HDxxH+VMAP-M0+VMAP-C→ MoxR→ vWA+TPRs*→ FXSXX-COOH→ Radical_SAM→ METALLOPROTEASE+TPRs→ TIR+APATPase+TPRs→</td><td>1094</td><td>Streptomyces sp. SCSIO 03032</td><td>GCA_002128305.1</td><td>actinobacteria</td></tr>
<tr><td>BAE95415.1</td><td>vWA+APATPase+wHTH+TPRs</td><td>Trypco1→ Trypsin+VMAP-M8+VMAP-C→ MoxR→ vWA+APATPase+wHTH+TPRs*→</td><td>1111</td><td>Streptomyces kanamyceticus</td><td>-</td><td>actinobacteria</td></tr>
<tr><td>BAG18883.1</td><td>vWA+TIR+FxsC_Cter</td><td>AB_hydrolase→ CASPASE+VMAP-M2+VMAP-C→ MoxR→ vWA+TIR+FxsC_Cter*→</td><td>1001</td><td>Streptomyces griseus subsp. griseus NBRC 13350</td><td>GCA_000010605.1</td><td>actinobacteria</td></tr>
<tr><td>BAS55188.1</td><td>vWA+FGS-fold_domain</td><td>TIR+EAD9→ EAD9+VMAP-M1+VMAP-C→ MoxR→ vWA+FGS-fold_domain*→</td><td>1083</td><td>Leptolyngbya boryana IAM M-101</td><td>GCA_002142475.1</td><td>cyanobacteria</td></tr>
<tr><td>BAU85996.1</td><td>vWA+TPRs</td><td>TCAD15||Trypco1→ Trypsin+HDxxH+VMAP-M0+VMAP-C→ MoxR→ vWA+TPRs*→ FXSXX-COOH→ METALLOPROTEASE+TPRs→ TIR+APATPase+TPRs→</td><td>1091</td><td>Streptomyces laurentii</td><td>GCA_002355495.1</td><td>actinobacteria</td></tr>
<tr><td>BAU87440.1</td><td>vWA+APATPase+wHTH+TPRs+cNMP_cyclase+NaeI</td><td>AB_hydrolase→ CASPASE+VMAP-M2+VMAP-C→ MoxR→ vWA+APATPase+wHTH+TPRs+cNMP_cyclase+NaeI*→</td><td>1575</td><td>Streptomyces laurentii</td><td>GCA_002355495.1</td><td>actinobacteria</td></tr>
<tr><td>BAY12967.1</td><td>vWA+MNS</td><td>EAD8+VMAP-M1+VMAP-C→ MoxR→ ?→ vWA+MNS*→</td><td>1139</td><td>Calothrix sp. NIES-2098</td><td>GCA_002368175.1</td><td>cyanobacteria</td></tr>
<tr><td>BAZ13460.1</td><td>vWA+GUN4+FGS-fold_domain</td><td>EAD1+Trypsin→ EAD1+VMAP-M1+VMAP-C→ MoxR→ vWA+GUN4+FGS-fold_domain*→</td><td>1035</td><td>Calothrix sp. NIES-4071</td><td>GCA_002368455.1</td><td>cyanobacteria</td></tr>
<tr><td>BAZ33904.1</td><td>vWA+Glyco_trans_4_4+Glycos_transf_1</td><td>cNMP_cyclase+VMAP-M1+VMAP-C→ MoxR→ vWA+Glyco_trans_4_4+Glycos_transf_1*→ FGS-fold_domain→</td><td>1018</td><td>Cylindrospermum sp. NIES-4074</td><td>GCA_003994795.1</td><td>cyanobacteria</td></tr>
</table>

<table>
<tr><th>acc</th><th>architecture</th><th>operon</th><th>len</th><th>species</th><th>gca</th><th>taxend</th></tr>
<tr><td>CAF33302.1</td><td>vWA+APATPase+wHTH+TPRs+cNMP_cyclase+NaeI</td><td>CASPASE+VMAP-M2+VMAP-C→ MoxR→ vWA+APATPase+wHTH+TPRs+cNMP_cyclase+NaeI*→</td><td>1793</td><td>Streptomyces fradiae</td><td>-</td><td>actinobacteria</td></tr>
<tr><td>CAH05112.1</td><td>vWA+APATPase+wHTH+TPRs+cNMP_cyclase+NaeI</td><td>AB_hydrolase→ CASPASE+VMAP-M2+VMAP-C→ MoxR→ vWA+APATPase+wHTH+TPRs+cNMP_cyclase+NaeI*→</td><td>1796</td><td>Streptomyces fradiae</td><td>-</td><td>actinobacteria</td></tr>
<tr><td>CAO86961.1</td><td>vWA+FGS-fold_domain</td><td>HTH+VMAP-M9+VMAP-C→ MoxR→ vWA+FGS-fold_domain*→ ||← ?||?→ ||← ?← ?||?→ FGS-fold_domain→</td><td>964</td><td>Microcystis aeruginosa PCC 7806</td><td>-</td><td>cyanobacteria</td></tr>
<tr><td>CBG74991.1</td><td>vWA+APATPase+wHTH+TPRs</td><td>TCAD9+Pkinase+NACHT+HEAT_repeats→ EAD2+VMAP-M0+VMAP-C→ vWA→ MoxR→ vWA+APATPase+TPRs*→</td><td>1029</td><td>Streptomyces scabiei 87.22</td><td>GCA_000091305.1</td><td>actinobacteria</td></tr>
<tr><td>CBG74993.1</td><td>vWA</td><td>TCAD9+Pkinase+NACHT+HEAT_repeats→ EAD2+VMAP-M0+VMAP-C→ vWA*→ MoxR→ vWA+APATPase+TPRs→</td><td>460</td><td>Streptomyces scabiei 87.22</td><td>GCA_000091305.1</td><td>actinobacteria</td></tr>
<tr><td>CCH29662.1</td><td>vWA+APATPase+wHTH+TPRs</td><td>EAD2+VMAP-M0+VMAP-C→ MoxR→ vWA+APATPase+wHTH+TPRs*→ ||← METALLOPROTEASE+TPRs← FXSXX-COOH-like← cNMP_cyclase+EAD2</td><td>1326</td><td>Saccharothrix espanaensis DSM 44229</td><td>GCA_000328705.1</td><td>actinobacteria</td></tr>
<tr><td>CCH33419.1</td><td>vWA+APATPase+wHTH+TPRs</td><td>cNMP_cyclase+EAD2→ EAD2+VMAP-M0+VMAP-C→ MoxR→ vWA+APATPase+wHTH+TPRs*→ ||← METALLOPROTEASE+TPRs</td><td>1447</td><td>Saccharothrix espanaensis DSM 44229</td><td>GCA_000328705.1</td><td>actinobacteria</td></tr>
<tr><td>CCH34758.1</td><td>vWA+APATPase+wHTH+TPRs</td><td>EAD2+VMAP-M0+VMAP-C→ MoxR→ vWA+APATPase+wHTH+TPRs*→ ||← ?← ?← TCAD15</td><td>1335</td><td>Saccharothrix espanaensis DSM 44229</td><td>GCA_000328705.1</td><td>actinobacteria</td></tr>
<tr><td>CCI02683.1</td><td>vWA</td><td>Trypsin+VMAP-M1+VMAP-C→ MoxR→ vWA*→</td><td>583</td><td>Microcystis aeruginosa PCC 9443</td><td>GCA_000312185.1</td><td>cyanobacteria</td></tr>
<tr><td>CCI03549.1</td><td>vWA+FGS-fold_domain</td><td>HTH+VMAP-M9+VMAP-C→ MoxR→ vWA+FGS-fold_domain*→ ?→ ?→ FGS-fold_domain→</td><td>930</td><td>Microcystis aeruginosa PCC 9443</td><td>GCA_000312185.1</td><td>cyanobacteria</td></tr>
<tr><td>CCK25370.1</td><td>vWA+HTH</td><td>CASPASE+VMAP-M0+VMAP-C→ vWA+HTH*→</td><td>762</td><td>Streptomyces davawensis JCM 4913</td><td>GCA_000349325.1</td><td>actinobacteria</td></tr>
<tr><td>CCK29233.1</td><td>vWA+APATPase+wHTH+TPRs</td><td>EAD2+VMAP-M0+VMAP-C→ vWA+APATPase+wHTH+TPRs*→ ||← METALLOPROTEASE+TPRs← FXSXX-COOH-like← Trypsin+EAD2||FXSXX-COOH-like→ METALLOPROTEASE+TPRs→</td><td>1276</td><td>Streptomyces davawensis JCM 4913</td><td>GCA_000349325.1</td><td>actinobacteria</td></tr>
<tr><td>CEL17030.1</td><td>vWA+APATPase+wHTH+TPRs</td><td>EAD2+VMAP-M0+VMAP-C→ MoxR→ vWA+APATPase+wHTH+TPRs*→ ||← METALLOPROTEASE+TPRs← FXSXX-COOH-like||?→ ||← ?||EAD2+VMAP-M0+VMAP-C→ MoxR→ vWA+APATPase+wHTH+TPRs→</td><td>1441</td><td>Kibdelosporangium sp. MJ126-NF4</td><td>GCA_000826545.1</td><td>actinobacteria</td></tr>
<tr><td>CEL17037.1</td><td>vWA+APATPase+wHTH+TPRs</td><td>vWA+APATPase+wHTH+TPRs→ ||← METALLOPROTEASE+TPRs← FXSXX-COOH-like||?→ ||← ?||EAD2+VMAP-M0+VMAP-C→ MoxR→ vWA+APATPase+wHTH+TPRs*→ ||← METALLOPROTEASE+TPRs</td><td>1403</td><td>Kibdelosporangium sp. MJ126-NF4</td><td>GCA_000826545.1</td><td>actinobacteria</td></tr>
<tr><td>CNF91710.1</td><td>vWA+FtsK+FtsK+CASPASE+FtsK</td><td>Trypco1→ Trypsin+VMAP-M8+VMAP-C→ MoxR→ vWA+FtsK+FtsK+CASPASE+FtsK*→</td><td>1677</td><td>Mycobacterium tuberculosis</td><td>GCA_001160225.1</td><td>actinobacteria</td></tr>
<tr><td>CQR61924.1</td><td>vWA+TPRs</td><td>← TCAD15||Trypco1→ Trypsin+HDxxH+VMAP-M0+VMAP-C→ MoxR→ vWA+TPRs*→ FXSXX-COOH→ Radical_SAM→ METALLOPROTEASE+TPRs→ TIR+APATPase+TPRs→</td><td>1118</td><td>Streptomyces leeuwenhoekii</td><td>GCA_001013905.1</td><td>actinobacteria</td></tr>
<tr><td>CQR62799.1</td><td>vWA+Pkinase+NUDIX</td><td>VMAP-M2+VMAP-C→ MoxR→ vWA+Pkinase+NUDIX*→</td><td>1271</td><td>Streptomyces leeuwenhoekii</td><td>GCA_001013905.1</td><td>actinobacteria</td></tr>
<tr><td>CUM41332.1</td><td>vWA+TPRs</td><td>AB_hydrolase→ CASPASE+EAD2+VMAP-M0+VMAP-C→ MoxR→ vWA+TPRs*→</td><td>1193</td><td>Streptomyces venezuelae</td><td>GCA_001406115.1</td><td>actinobacteria</td></tr>
<tr><td>CUM41527.1</td><td>vWA+APATPase+wHTH+TPRs</td><td>EAD2+VMAP-M0+VMAP-C→ MoxR→ vWA+APATPase+wHTH+TPRs*→ ||← Na_H_Exchanger||AB_hydrolase→ CASPASE+EAD2→ FXSXX-COOH-like→ METALLOPROTEASE+TPRs→</td><td>1526</td><td>Streptomyces venezuelae</td><td>GCA_001406115.1</td><td>actinobacteria</td></tr>
<tr><td>CUU60227.1</td><td>vWA+APATPase+wHTH+TPRs</td><td>EAD2+EAD1→ EAD2+VMAP-M0+VMAP-C→ MoxR→ vWA+APATPase+wHTH+TPRs*→ ||← METALLOPROTEASE+TPRs← FXSXX-COOH-like</td><td>1582</td><td>Frankia sp. G2</td><td>GCA_001536285.1</td><td>actinobacteria</td></tr>
<tr><td>CUW28293.1</td><td>vWA+wHTH+cNMP_cyclase+Band7+HAD</td><td>Trypco1→ Trypsin+VMAP-M0+VMAP-C→ MoxR→ vWA+wHTH+cNMP_cyclase+Band7+HAD*→</td><td>1288</td><td>Streptomyces reticuli</td><td>GCA_001511815.1</td><td>actinobacteria</td></tr>
<tr><td>CUW31051.1</td><td>vWA+TPRs</td><td>TCAD15→ Trypco1→ Trypsin+HDxxH+VMAP-M0+VMAP-C→ MoxR→ vWA+TPRs*→ FXSXX-COOH-like→ METALLOPROTEASE+TPRs→ TIR+APATPase+TPRs→</td><td>1217</td><td>Streptomyces reticuli</td><td>GCA_001511815.1</td><td>actinobacteria</td></tr>
<tr><td>EAW35097.1</td><td>vWA+FGS-fold_domain</td><td>EAD4+VMAP-M3+VMAP-C→ MoxR→ vWA+FGS-fold_domain*→ FGS-fold_domain→</td><td>894</td><td>Lyngbya sp. PCC 8106</td><td>GCA_000169095.1</td><td>cyanobacteria</td></tr>
<tr><td>EDX26496.1</td><td>vWA</td><td>← cNMP_binding+HTH_Crp_2← ?← ?||EAD2+VMAP-M0+VMAP-C→ MoxR→ vWA*→</td><td>537</td><td>Streptomyces sp. Mg1</td><td>GCA_000154885.1</td><td>actinobacteria</td></tr>
<tr><td>EFC84333.1</td><td>vWA+DO-GTPase2</td><td>TIR+VMAP-M2+VMAP-C→ MoxR→ vWA+DO-GTPase2*→</td><td>1017</td><td>Frankia sp. EUN1f</td><td>GCA_000177675.1</td><td>actinobacteria</td></tr>
<tr><td>EFD69442.1</td><td>vWA+Pkinase+wHTH+TPRs+cNMP_cyclase</td><td>Trypco1→ Trypsin+VMAP-M0+VMAP-C→ MoxR→ vWA→ vWA+Pkinase+HTH+TPRs+cNMP_cyclase*→</td><td>1038</td><td>Streptomyces lividans TK24</td><td>GCA_000158935.1</td><td>actinobacteria</td></tr>
<tr><td>EFD69443.1</td><td>vWA_protein</td><td>Trypco1→ Trypsin+VMAP-M0+VMAP-C→ MoxR→ vWA*→ vWA+Pkinase+HTH+TPRs+cNMP_cyclase→</td><td>296</td><td>Streptomyces lividans TK24</td><td>GCA_000158935.1</td><td>actinobacteria</td></tr>
<tr><td>EFE78827.1</td><td>vWA+APATPase+wHTH+TPRs</td><td>EAD2+VMAP-M0+VMAP-C→ MoxR→ vWA+APATPase+wHTH+TPRs*→ AB_hydrolase→ CASPASE+EAD2→ FXSXX-COOH-like→ METALLOPROTEASE+TPRs→</td><td>1347</td><td>Streptomyces roseosporus NRRL 15998</td><td>GCA_000156455.1</td><td>actinobacteria</td></tr>
<tr><td>EFH31709.1</td><td>vWA+TIR+FxsC_Cter</td><td>AB_hydrolase→ CASPASE+VMAP-M2+VMAP-C→ MoxR→ vWA+TIR+FxsC_Cter*→ ||← DUF397</td><td>967</td><td>Streptomyces pristinaespiralis ATCC 25486</td><td>GCA_000154945.1</td><td>actinobacteria</td></tr>
<tr><td>EFL17576.1</td><td>vWA</td><td>← TCAD15||Trypsin+HDxxH+VMAP-M0+VMAP-C→ MoxR→ vWA*→ FXSXX-COOH→ Radical_SAM→ METALLOPROTEASE+TPRs→ TIR+APATPase+TPRs→</td><td>583</td><td>Streptomyces sp. C</td><td>GCA_000158895.1</td><td>actinobacteria</td></tr>
<tr><td>EFL20927.1</td><td>vWA+APATPase+wHTH+TPRs</td><td>EAD2+VMAP-M0+VMAP-C→ MoxR→ vWA+APATPase+wHTH+TPRs*→ ||← METALLOPROTEASE+TPRs← FXSXX-COOH-like</td><td>1469</td><td>Streptomyces himastatinicus ATCC 53653</td><td>GCA_000158915.1</td><td>actinobacteria</td></tr>
<tr><td>EFL31903.1</td><td>vWA</td><td>Trypco1→ Trypsin+VMAP-M0+VMAP-C→ MoxR→ vWA*→</td><td>570</td><td>Streptomyces viridochromogenes DSM 40736</td><td>GCA_000158955.1</td><td>actinobacteria</td></tr>
<tr><td>EFL34810.1</td><td>vWA+TPRs</td><td>← TCAD15||Trypco1→ Trypsin+HDxxH+VMAP-M0+VMAP-C→ MoxR→ vWA+TPRs*→</td><td>1000</td><td>Streptomyces viridochromogenes DSM 40736</td><td>GCA_000158955.1</td><td>actinobacteria</td></tr>
<tr><td>EFL35514.1</td><td>vWA</td><td>EAD2+VMAP-M0+VMAP-C→ MoxR→ vWA*→ TIR+APATPase+TPRs→ ?→ CASPASE+EAD2→ METALLOPROTEASE+TPRs→</td><td>464</td><td>Streptomyces viridochromogenes DSM 40736</td><td>GCA_000158955.1</td><td>actinobacteria</td></tr>
<tr><td>EFO80365.1</td><td>vWA+TPRs</td><td>← CASPASE+TCAD1+TCAD2||CASPASE+TCAD1+DUF4404→ VMAP-M13+VMAP-C→ MoxR→ vWA+TPRs*→</td><td>1062</td><td>Oscillochloris trichoides DG-6</td><td>GCA_000152145.1</td><td>chloroflexi</td></tr>
<tr><td>EGE41651.1</td><td>vWA+TIR+FxsC_Cter</td><td>AB_hydrolase→ CASPASE+VMAP-M2+VMAP-C→ MoxR→ vWA+TIR+FxsC_Cter*→</td><td>1001</td><td>Streptomyces sp. ACT-1</td><td>GCA_000177175.2</td><td>actinobacteria</td></tr>
</table>

<table>
<tr><th>acc</th><th>architecture</th><th>operon</th><th>len</th><th>species</th><th>gca</th><th>taxend</th></tr>
<tr><td>EHM30204.1</td><td>vWA+APATPase+wHTH+TPRs</td><td>EAD2+VMAP-M0+VMAP-C→ MoxR→ vWA+APATPase+wHTH+TPRs*→ AB_hydrolase→ CASPASE+EAD2→ METALLOPROTEASE+TPRs→</td><td>1329</td><td>Streptomyces sp. W007</td><td>GCA_000239075.2</td><td>actinobacteria</td></tr>
<tr><td>EHR73674.1</td><td>vWA+HTH</td><td>AB_hydrolase→ CASPASE+VMAP-M15+VMAP-C→ MoxR→ vWA+HTH*→</td><td>881</td><td>Burkholderiales bacterium JOSHI_001</td><td>GCA_000244995.1</td><td>betaproteobacteria</td></tr>
<tr><td>EIF91212.1</td><td>vWA+Pkinase</td><td>vWA+Pkinase*→</td><td>686</td><td>Streptomyces tsukubensis NRRL18488</td><td>GCA_000297155.2</td><td>actinobacteria</td></tr>
<tr><td>EIP98807.1</td><td>vWA+REC+HTH+SLOG</td><td>TCAD5+AB_hydrolase+TCAD7+macroglobulin-like+CASPASE→ CASPASE+VMAP-C→ MoxR→ vWA+REC+HTH+SLOG*→</td><td>1192</td><td>Opitutaceae bacterium TAV1</td><td>GCA_000243495.3</td><td>verrucomicrobia</td></tr>
<tr><td>EIV90731.1</td><td>vWA+APATPase+wHTH+TPRs</td><td>EAD2+VMAP-M0+VMAP-C→ MoxR→ vWA+APATPase+wHTH+TPRs*→ ||← METALLOPROTEASE+TPRs← ?← EAD5+TIR</td><td>1480</td><td>Frankia sp. QA3</td><td>GCA_000262465.1</td><td>actinobacteria</td></tr>
<tr><td>EKC91743.1</td><td>vWA+TPRs</td><td>VMAP-C(partial)→ MoxR→ vWA+TPRs*→</td><td>899</td><td>Streptomyces sp. SM8</td><td>-</td><td>actinobacteria</td></tr>
<tr><td>EKU96832.1</td><td>vWA+FGS-fold_domain</td><td>AB_hydrolase→ CASPASE+EAD9→ EAD9+VMAP-M1+VMAP-C→ MoxR→ vWA+FGS-fold_domain*→ FGS-fold_domain→ four_helix_bundle_protein→ RVT_1→ FGS-fold_domain→</td><td>926</td><td>Leptolyngbya sp. PCC 7375</td><td>GCA_000316115.1</td><td>cyanobacteria</td></tr>
<tr><td>EKU96985.1</td><td>vWA+BetaPropeller+FGS-fold_domain</td><td>FGS-fold_domain→ EAD1+Trypsin→ ?→ EAD1+VMAP-M1+VMAP-C→ MoxR→ vWA+BetaPropeller+FGS-fold_domain*→ FGS-fold_domain→ ||← RVT_1← four_helix_bundle_protein</td><td>1247</td><td>Leptolyngbya sp. PCC 7375</td><td>GCA_000316115.1</td><td>cyanobacteria</td></tr>
<tr><td>ELQ77549.1</td><td>vWA</td><td>← TCAD15||Trypco1→ Trypsin+HDxxH+VMAP-M0+VMAP-C→ MoxR→ vWA*→</td><td>345</td><td>Streptomyces rimosus subsp. rimosus ATCC 10970</td><td>GCA_000331185.1</td><td>actinobacteria</td></tr>
<tr><td>EME53021.1</td><td>vWA+APATPase+wHTH+TPRs</td><td>cNMP_cyclase+VMAP-M0+VMAP-C→ MoxR→ vWA+APATPase+wHTH+TPRs*→ ||← METALLOPROTEASE+TPRs← FXSXX-COOH-like← cNMP_binding+HTH_Crp_2</td><td>1468</td><td>Amycolatopsis decaplanina DSM 44594</td><td>GCA_000342005.1</td><td>actinobacteria</td></tr>
<tr><td>EMF02657.1</td><td>vWA+TPRs</td><td>← TCAD15||Trypco1→ Trypsin+HDxxH+VMAP-M0+VMAP-C→ MoxR→ vWA+TPRs*→ FXSXX-COOH-like→ METALLOPROTEASE+TPRs→ TIR+APATPase+TPRs→</td><td>1056</td><td>Streptomyces mobaraensis NBRC 13819 = DSM 40847</td><td>GCA_000342125.1</td><td>actinobacteria</td></tr>
<tr><td>EOY47501.1</td><td>vWA+Pkinase</td><td>Trypco1→ Trypsin+VMAP-M0+VMAP-C→ MoxR→ vWA+Pkinase*→</td><td>449</td><td>Streptomyces lividans 1326</td><td>GCA_000403665.1</td><td>actinobacteria</td></tr>
<tr><td>EOY50271.1</td><td>vWA</td><td>← TCAD15||Trypco1→ Trypsin+HDxxH+VMAP-M0+VMAP-C→ MoxR→ vWA*→ ?→ ?→ FXSXX-COOH→ Radical_SAM→ METALLOPROTEASE+TPRs→ TIR+APATPase+TPRs→ Trypco1→</td><td>356</td><td>Streptomyces lividans 1326</td><td>GCA_000403665.1</td><td>actinobacteria</td></tr>
<tr><td>EPD55277.1</td><td>vWA+TPRs</td><td>← TCAD15||Trypco1→ Trypsin+HDxxH+VMAP-M0+VMAP-C→ MoxR→ vWA+TPRs*→ FXSXX-COOH→ Radical_SAM→ METALLOPROTEASE+TPRs→ TIR+APATPase+TPRs→</td><td>1151</td><td>Streptomyces sp. HGB0020</td><td>GCA_000411315.1</td><td>actinobacteria</td></tr>
<tr><td>EPF21804.1</td><td>vWA+FGS-fold_domain</td><td>AB_hydrolase→ CASPASE+VMAP-M14+VMAP-C→ MoxR→ vWA(partial)→ vWA+FGS-fold_domain*→</td><td>706</td><td>Microcystis aeruginosa SPC777</td><td>GCA_000412595.1</td><td>cyanobacteria</td></tr>
<tr><td>EPH45106.1</td><td>vWA+Pkinase+ParA-Soj-PloopNTPase(SIMIBI)</td><td>Trypco1→ Trypsin+EAD2+VMAP-M0+VMAP-C→ MoxR→ ?→ vWA+Pkinase+ParA-Soj-PloopNTPase(SIMIBI)*→</td><td>1092</td><td>Streptomyces aurantiacus JA 4570</td><td>GCA_000414115.1</td><td>actinobacteria</td></tr>
<tr><td>EPH45390.1</td><td>vWA+APATPase+wHTH+TPRs</td><td>vWA+APATPase+wHTH+TPRs*→ ||← METALLOPROTEASE+TPRs← FXSXX-COOH-like← CASPASE+EAD2</td><td>1294</td><td>Streptomyces aurantiacus JA 4570</td><td>GCA_000414115.1</td><td>actinobacteria</td></tr>
<tr><td>EPJ42712.1</td><td>vWA+EAD2+CASPASE+EAD2</td><td>EAD2+VMAP-M0+VMAP-C→ MoxR→ vWA+EAD2+CASPASE+EAD2*→</td><td>997</td><td>Streptomyces afghaniensis 772</td><td>GCA_000415505.1</td><td>actinobacteria</td></tr>
<tr><td>ESP99762.1</td><td>vWA+TPRs</td><td>← TCAD15||Trypco1→ Trypsin+HDxxH+VMAP-M0+VMAP-C→ MoxR→ vWA+TPRs*→ FXSXX-COOH→ Radical_SAM→ METALLOPROTEASE+TPRs→ TIR+APATPase+TPRs→</td><td>1106</td><td>Streptomyces sp. GBA 94-10</td><td>GCA_000495635.1</td><td>actinobacteria</td></tr>
<tr><td>ESQ05820.1</td><td>vWA+TPRs</td><td>← TCAD15||Trypco1→ Trypsin+HDxxH+VMAP-M0+VMAP-C→ MoxR→ vWA+TPRs*→ FXSXX-COOH→ Radical_SAM→ METALLOPROTEASE+TPRs→ TIR+APATPase+TPRs→</td><td>1106</td><td>Streptomyces sp. PVA 94-07</td><td>GCA_000495755.1</td><td>actinobacteria</td></tr>
<tr><td>ESQ11649.1</td><td>vWA_protein</td><td>Trypco1→ Trypsin+VMAP-M0+VMAP-C→ MoxR→ vWA_protein*→</td><td>586</td><td>uncultured Thiohalocapsa sp. PB-PSB1</td><td>GCA_000495615.1</td><td>gammaproteobacteria</td></tr>
<tr><td>EST31053.1</td><td>vWA+Pkinase+TPRs</td><td>Trypco2→ Trypsin+EAD2+VMAP-M0+VMAP-C→ MoxR→ vWA+Pkinase+TPRs*→</td><td>1121</td><td>Streptomyces niveus NCIMB 11891</td><td>GCA_000497425.1</td><td>actinobacteria</td></tr>
<tr><td>EST34701.1</td><td>vWA+APATPase+wHTH+TPRs</td><td>EAD2+VMAP-M0+VMAP-C→ MoxR→ vWA+APATPase+wHTH+TPRs*→ ||← METALLOPROTEASE+TPRs← FXSXX-COOH-like</td><td>1484</td><td>Streptomyces roseochromogenus subsp. oscitans DS 12.976</td><td>GCA_000497445.1</td><td>actinobacteria</td></tr>
<tr><td>EWM11539.1</td><td>vWA+TIR+BetaPropeller</td><td>EAD2+VMAP-M0+VMAP-C→ vWA+TIR+BetaPropeller*→ ?→ ?→ cNMP_binding+HTH_Crp_2→</td><td>1096</td><td>Kutzneria sp. 744</td><td>GCA_000568255.1</td><td>actinobacteria</td></tr>
<tr><td>EXU70110.1</td><td>vWA+TPRs</td><td>AB_hydrolase→ CASPASE+EAD2+VMAP-M0+VMAP-C→ MoxR→ vWA+TPRs*→</td><td>1172</td><td>Streptomyces sp. PRh5</td><td>GCA_000590515.1</td><td>actinobacteria</td></tr>
<tr><td>EYU68527.1</td><td>vWA+TIR+FxsC_Cter</td><td>AB_hydrolase→ CASPASE+VMAP-M2+VMAP-C→ MoxR→ vWA+TIR+FxsC_Cter*→</td><td>839</td><td>Streptomyces sp. PCS3-D2</td><td>GCA_000612545.1</td><td>actinobacteria</td></tr>
<tr><td>EYU71427.1</td><td>vWA+Trypsin+cNMP_cyclase+CCCH(TrebleClef)+Pentapeptide_repeats</td><td>AB_hydrolase→ CASPASE+VMAP-M0+VMAP-C→ MoxR→ vWA+Trypsin+cNMP_cyclase+CCCH(TrebleClef)+Pentapeptide_repeats*→</td><td>1196</td><td>Streptomyces sp. PCS3-D2</td><td>GCA_000612545.1</td><td>actinobacteria</td></tr>
<tr><td>GAO06359.1</td><td>vWA+TPRs</td><td>← TCAD15||Trypco1→ Trypsin+HDxxH+VMAP-M0+VMAP-C→ MoxR→ vWA+TPRs*→ FXSXX-COOH→ Radical_SAM→ TIR+APATPase+TPRs→</td><td>1127</td><td>Streptomyces sp. NBRC 110027</td><td>GCA_000829715.2</td><td>actinobacteria</td></tr>
<tr><td>GAO06532.1</td><td>vWA+wHTH+TPRs+APATPase+wHTH+TPRs</td><td>Trypco1→ Trypsin+EAD2+VMAP-M0+VMAP-C→ MoxR→ vWA+wHTH+TPRs+APATPase+wHTH+TPRs*→ ||← ?||?→ ||← AB_hydrolase</td><td>1588</td><td>Streptomyces sp. NBRC 110027</td><td>GCA_000829715.2</td><td>actinobacteria</td></tr>
<tr><td>GAP48484.1</td><td>vWA+APATPase+wHTH+TPRs</td><td>EAD2+VMAP-M0+VMAP-C→ MoxR→ vWA+APATPase+wHTH+TPRs*→ AB_hydrolase→ CASPASE+EAD2→ FXSXX-COOH-like→ METALLOPROTEASE+TPRs→</td><td>1477</td><td>Streptomyces azureus</td><td>GCA_001270025.1</td><td>actinobacteria</td></tr>
<tr><td>GAP50094.1</td><td>vWA+Pkinase+wHTH+TPRs+cNMP_cyclase+Band7+HAD</td><td>Trypco1→ Trypsin+VMAP-M0+VMAP-C→ MoxR→ vWA+Pkinase+wHTH+TPRs+cNMP_cyclase+Band7+HAD*→</td><td>1448</td><td>Streptomyces azureus</td><td>GCA_001270025.1</td><td>actinobacteria</td></tr>
<tr><td>GAQ55305.1</td><td>vWA+Pkinase+wHTH+cNMP_cyclase+Band7+HAD</td><td>vWA+Pkinase+wHTH+cNMP_cyclase+Band7+HAD*→</td><td>1190</td><td>Streptomyces acidiscabies</td><td>GCA_001485105.1</td><td>actinobacteria</td></tr>
<tr><td>GAQ56299.1</td><td>vWA+TPRs</td><td>vWA+TPRs*→ FXSXX-COOH→ Radical_SAM→ METALLOPROTEASE+TPRs→ TIR+APATPase+TPRs→</td><td>1047</td><td>Streptomyces acidiscabies</td><td>GCA_001485105.1</td><td>actinobacteria</td></tr>
<tr><td>GAQ67487.1</td><td>vWA+SFII-helicase+Trypsin+cNMP_cyclase+Coiled-coil+CASPASE</td><td>AB_hydrolase→ CASPASE+VMAP-M2+VMAP-C→ MoxR→ vWA+SFII-helicase+Trypsin+cNMP_cyclase+Coiled-coil+CASPASE*→ ||← ?← ?← TIR+APATPase+TPRs</td><td>1797</td><td>Streptomyces scabiei</td><td>GCA_001485125.1</td><td>actinobacteria</td></tr>
<tr><td>GAT82291.1</td><td>vWA+APATPase+wHTH+TPRs</td><td>Trypsin+EAD2→ EAD2+VMAP-M0+VMAP-C→ MoxR→ vWA+APATPase+wHTH+TPRs*→ ||← METALLOPROTEASE+TPRs||?→ Trypco1→</td><td>1628</td><td>Streptomyces sp. F-3</td><td>GCA_001653115.1</td><td>actinobacteria</td></tr>
<tr><td>GAV40520.1</td><td>vWA+Pkinase+wHTH+cNMP_cyclase+Band7+HAD</td><td>vWA+Pkinase+wHTH+cNMP_cyclase+Band7+HAD*→</td><td>1258</td><td>Streptomyces acidiscabies</td><td>GCA_001949645.1</td><td>actinobacteria</td></tr>
<tr><td>GAV42170.1</td><td>vWA+TPRs</td><td>vWA+TPRs*→ FXSXX-COOH→ Radical_SAM→ METALLOPROTEASE+TPRs→ TIR+APATPase+TPRs→</td><td>1017</td><td>Streptomyces acidiscabies</td><td>GCA_001949645.1</td><td>actinobacteria</td></tr>
<tr><td>GAV44387.1</td><td>vWA+TIR+FxsC_Cter(partial)</td><td>vWA+TIR+FxsC_Cter(partial)*→</td><td>765</td><td>Streptomyces acidiscabies</td><td>GCA_001949645.1</td><td>actinobacteria</td></tr>
</table>

acc	architecture	operon	len	species	gca	taxend
KDR57872.1	vWA	VMAP-C_protein→ MoxR→ vWA*→	569	Arthrospira platensis str. Paraca	GCA_000175415.3	cyanobacteria
KDS87355.1	vWA+Pkinase+ParA-Soj-PloopNTPase(SIMIBI)	NUDIX→ Trypco1→ Trypsin+EAD2+VMAP-M0+VMAP-C→ MoxR→ vWA+Pkinase+ParA-Soj-PloopNTPase(SIMIBI)*→ SIR2→	1158	Streptomyces fradiae	-	actinobacteria
KES08164.1	vWA+Pkinase+ParA-Soj-PloopNTPase(SIMIBI)	Trypco1→ Trypsin+EAD2+VMAP-M0+VMAP-C→ MoxR→ vWA+Pkinase+ParA-Soj-PloopNTPase(SIMIBI)*→	1111	Streptomyces toyocaensis	GCA_000725125.1	actinobacteria
KIF16909.1	vWA+FGS-fold_domain	Trypco1→ Trypsin+VMAP-M1+VMAP-C→ MoxR→ vWA+FGS-fold_domain*→ FGS-fold_domain→	878	Aphanocapsa montana BDHKU210001	GCA_000817745.1	cyanobacteria
KIF79231.1	vWA+TPRs	← TCAD15||Trypsin+HDxxH+VMAP-M0+VMAP-C→ MoxR→ vWA+TPRs*→ FXSXX-COOH→ METALLOPROTEASE+TPRs→ TIR+APATPase+TPRs→	1108	Streptomyces sp. 150FB	GCA_000818195.1	actinobacteria
KIH98248.1	vWA+APATPase+wHTH+TPRs	Trypco1→ Trypsin+EAD2+VMAP-M0+VMAP-C→ MoxR→ vWA+APATPase+wHTH+TPRs*→ ||← Radical_SAM← FXSXX-COOH-like	1426	Streptomonospora alba	GCA_000826685.1	actinobacteria
KIR65538.1	vWA+TPRs	Trypsin+HDxxH+VMAP-M0+VMAP-C→ MoxR→ vWA+TPRs*→ ?→ TIR+APATPase+TPRs→	1129	Micromonospora carbonacea	GCA_000876225.1	actinobacteria
KIX48996.1	vWA+APATPase+wHTH+TPRs	EAD2+VMAP-M0+VMAP-C→ MoxR→ vWA+APATPase+wHTH+TPRs*→ AB_hydrolase→ CASPASE+EAD2→ FXSXX-COOH-like→ METALLOPROTEASE+TPRs→	1463	Streptomyces griseus	-	actinobacteria
KJK39591.1	vWA+APATPase+wHTH+TPRs	EAD2+VMAP-M0+VMAP-C→ MoxR→ vWA+APATPase+wHTH+TPRs*→ AB_hydrolase→ CASPASE+EAD2→ FXSXX-COOH-like→ METALLOPROTEASE+TPRs→	1508	Streptomyces variegatus	GCA_000955965.1	actinobacteria
KJK41459.1	vWA+Pkinase+wHTH+TPRs+cNMP_cyclase+Band7+HAD	Trypco1→ Trypsin+VMAP-M0+VMAP-C→ MoxR→ vWA+Pkinase+wHTH+TPRs+cNMP_cyclase+Band7+HAD*→	1423	Streptomyces variegatus	GCA_000955965.1	actinobacteria
KJK51066.1	vWA+APATPase+wHTH+TPRs	← cNMP_binding+HTH_Crp_2||MoxR→ vWA+APATPase+wHTH+TPRs*→ ||← METALLOPROTEASE+TPRs← FXSXX-COOH-like	1204	Lechevalieria aerocolonigenes	GCA_000955955.1	actinobacteria
KJY16212.1	vWA+TPRs	← TCAD15||Trypsin+HDxxH+VMAP-M0+VMAP-C→ MoxR→ vWA+TPRs*→ FXSXX-COOH→	1077	Streptomyces sp. NRRL S-104	GCA_000966965.1	actinobacteria
KJY21448.1	vWA+Trypsin+cNMP_cyclase+CCCH(TrebleClef)+Pentapeptide_repeats	VMAP-M0+VMAP-C→ MoxR→ vWA+Trypsin+cNMP_cyclase+CCCH(TrebleClef)+Pentapeptide_repeats*→	1198	Streptomyces sp. NRRL S-104	GCA_000966965.1	actinobacteria
KJY37233.1	vWA	vWA*→	346	Streptomyces katrae	GCA_000966975.1	actinobacteria
KJY41391.1	vWA	← TCAD15||Trypsin+HDxxH+VMAP-M0+VMAP-C→ MoxR→ vWA*→	467	Streptomyces sp. NRRL S-444	GCA_000966985.1	actinobacteria
KMO98800.1	vWA+TPRs	← TCAD15||Trypsin+HDxxH+VMAP-M0+VMAP-C→ MoxR→ vWA+TPRs*→ METALLOPROTEASE+TPRs→ TIR+APATPase+TPRs→	1071	Streptomyces roseus	GCA_001044425.1	actinobacteria
KMS71131.1	vWA+APATPase+wHTH+TPRs	Trypco2→ EAD2+VMAP-M0+VMAP-C→ vWA+APATPase+wHTH+TPRs*→ ||← FXSXX-COOH-like← Trypsin+EAD2	1201	Streptomyces viridochromogenes	GCA_001047325.1	actinobacteria
KMS80645.1	vWA+TPRs	← TCAD15||Trypco1→ Trypsin+HDxxH+VMAP-M0+VMAP-C→ MoxR→ vWA+TPRs*→ FXSXX-COOH→ METALLOPROTEASE+TPRs→ TIR+APATPase+TPRs→	1118	Streptomyces leeuwenhoekii	GCA_001047315.1	actinobacteria
KMS83272.1	vWA+TPRs	Trypco1→ Trypsin+HDxxH+VMAP-M0+VMAP-C→ MoxR→ vWA+TPRs*→ FXSXX-COOH-like→ METALLOPROTEASE+TPRs→ TIR+APATPase+TPRs→	1238	Streptomyces regensis	GCA_001047335.1	actinobacteria
KMS84544.1	vWA+Pkinase+cNMP_cyclase+Band7+HAD	Trypco1→ Trypsin+VMAP-M0+VMAP-C→ MoxR→ vWA+Pkinase+cNMP_cyclase+Band7+HAD*→	1313	Streptomyces regensis	GCA_001047335.1	actinobacteria
KMW70431.1	vWA+FGS-fold_domain	FGS-fold_domain→ ||← ?← ?||EAD4+VMAP-M3+VMAP-C→ MoxR→ vWA+FGS-fold_domain*→ ?→ FGS-fold_domain→	884	Limnoraphis robusta CS-951	GCA_000972705.2	cyanobacteria
KMW70584.1	vWA_protein	FGS-fold_domain→ ||← ?← ?||EAD4+VMAP-M3+VMAP-C→ MoxR→ vWA_protein*→	354	Limnoraphis robusta CS-951	GCA_000972705.2	cyanobacteria
KND29573.1	vWA+Pkinase+wHTH+cNMP_cyclase+Band7+HAD	Trypco1→ Trypsin+VMAP-M0+VMAP-C→ MoxR→ ?→ vWA+Pkinase+wHTH+cNMP_cyclase+Band7+HAD*→	1217	Streptomyces acidiscabies	GCA_001189015.1	actinobacteria
KND32413.1	vWA+TPRs	← TCAD15||Trypsin+HDxxH+VMAP-M0+VMAP-C→ MoxR→ vWA+TPRs*→ Radical_SAM→ METALLOPROTEASE+TPRs→ TIR+APATPase+TPRs→	1100	Streptomyces acidiscabies	GCA_001189015.1	actinobacteria
KND33426.1	vWA+APATPase+wHTH+TPRs	EAD2+VMAP-M0+VMAP-C→ MoxR→ vWA+APATPase+wHTH+TPRs*→ AB_hydrolase→ CASPASE+EAD2→ FXSXX-COOH-like→ METALLOPROTEASE+TPRs→	1329	Streptomyces europaeiscabiei	GCA_001189025.1	actinobacteria
KOG25205.1	vWA_protein	Trypco2→ Trypsin+EAD2+VMAP-M0+VMAP-C→ MoxR→ vWA_protein*→	277	Streptomyces viridochromogenes	GCA_001270515.1	actinobacteria
KOG25691.1	vWA+APATPase+wHTH+TPRs	EAD2+VMAP-M0+VMAP-C→ MoxR→ vWA+APATPase+wHTH+TPRs*→ AB_hydrolase→ CASPASE+EAD2→ FXSXX-COOH-like→ METALLOPROTEASE+TPRs→	1355	Streptomyces viridochromogenes	GCA_001270515.1	actinobacteria
KOG44801.1	vWA+Trypsin+cNMP_cyclase+CCCH(TrebleClef)+Pentapeptide_repeats	vWA+Trypsin+cNMP_cyclase+CCCH(TrebleClef)+Pentapeptide_repeats*→	1144	Streptomyces virginiae	GCA_001270565.1	actinobacteria
KOG57815.1	vWA+TPRs	← TCAD15||Trypsin+HDxxH+VMAP-M0+VMAP-C→ MoxR→ vWA+TPRs*→ FXSXX-COOH→ Radical_SAM→ METALLOPROTEASE+TPRs→ TIR+APATPase+TPRs→	1098	Streptomyces virginiae	GCA_001270565.1	actinobacteria
KOG73838.1	vWA+TPRs	TCAD15→ Trypco1→ Trypsin+HDxxH+VMAP-M0+VMAP-C→ MoxR→ vWA+TPRs*→ FXSXX-COOH-like→ METALLOPROTEASE+TPRs→ TIR+APATPase+TPRs→	1235	Streptomyces antibioticus	GCA_001270635.1	actinobacteria
KOT34693.1	vWA+wHTH+TPRs+wHTH+TPRs+APATPase+wHTH+TPRs	Trypco1→ Trypsin+EAD2+VMAP-M0+VMAP-C→ MoxR→ vWA+wHTH+TPRs+wHTH+TPRs+APATPase+wHTH+TPRs*→ ||← AB_hydrolase	1732	Streptomyces sp. NRRL WC-3701	GCA_001279025.1	actinobacteria
KOT44382.1	vWA+TPRs	← TCAD15||Trypco1→ Trypsin+HDxxH+VMAP-M0+VMAP-C→ MoxR→ vWA+TPRs*→ Radical_SAM→ TIR+APATPase+TPRs→	1085	Streptomyces rimosus subsp. rimosus	GCA_001279015.1	actinobacteria
KOT47672.1	vWA+TPRs	← TCAD15||Trypco1→ Trypsin+HDxxH+VMAP-M0+VMAP-C→ MoxR→ vWA+TPRs*→ Radical_SAM→ TIR+APATPase+TPRs→	1085	Streptomyces rimosus subsp. rimosus	GCA_001279065.1	actinobacteria
KOT61113.1	vWA+wHTH+TPRs+wHTH+TPRs+APATPase+wHTH+TPRs	Trypco1→ Trypsin+EAD2+VMAP-M0+VMAP-C→ MoxR→ vWA+wHTH+TPRs+wHTH+TPRs+APATPase+wHTH+TPRs*→ ||← AB_hydrolase	1742	Streptomyces rimosus subsp. rimosus	GCA_001279065.1	actinobacteria
KOT93823.1	vWA+wHTH+TPRs+wHTH+TPRs+APATPase+wHTH+TPRs	Trypco1→ Trypsin+EAD2+VMAP-M0+VMAP-C→ MoxR→ vWA+wHTH+TPRs+wHTH+TPRs+APATPase+wHTH+TPRs*→ ||← AB_hydrolase	1733	Streptomyces sp. NRRL F-5755	GCA_001279365.1	actinobacteria
KOT96760.1	vWA+TPRs	← TCAD15||Trypco1→ Trypsin+HDxxH+VMAP-M0+VMAP-C→ MoxR→ vWA+TPRs*→ Radical_SAM→ TIR+APATPase+TPRs→	1085	Streptomyces rimosus subsp. pseudoverticillatus	GCA_001279345.1	actinobacteria
KOT99550.1	vWA+wHTH+TPRs+wHTH+TPRs+APATPase+wHTH+TPRs	Trypco1→ Trypsin+EAD2+VMAP-M0+VMAP-C→ MoxR→ vWA+wHTH+TPRs+wHTH+TPRs+APATPase+wHTH+TPRs*→ ||← AB_hydrolase	1732	Streptomyces rimosus subsp. pseudoverticillatus	GCA_001279345.1	actinobacteria
KOU00046.1	vWA+TPRs	← TCAD15||Trypco1→ Trypsin+HDxxH+VMAP-M0+VMAP-C→ MoxR→ vWA+TPRs*→ Radical_SAM→ TIR+APATPase+TPRs→ TIR+APATPase+TPRs→	1094	Streptomyces sp. NRRL F-5755	GCA_001279365.1	actinobacteria
KOU06764.1	vWA+TPRs	← TCAD15||Trypco1→ Trypsin+HDxxH+VMAP-M0+VMAP-C→ MoxR→ vWA+TPRs*→ FXSXX-COOH→ Radical_SAM→ METALLOPROTEASE+TPRs→ TIR+APATPase+TPRs→ Trypco1→	1125	Streptomyces sp. NRRL F-4711	GCA_001279355.1	actinobacteria

<table>
<thead>
<tr><th>acc</th><th>architecture</th><th>operon</th><th>len</th><th>species</th><th>gca</th><th>taxend</th></tr>
</thead>
<tbody>
<tr><td>KOU20535.1</td><td>vWA+TPRs</td><td>TCAD15||Trypsin+HDxxH+VMAP-M0+VMAP-C→ MoxR→ vWA+TPRs*→ FXSXX-COOH→</td><td>1098</td><td>Streptomyces sp. WM6368</td><td>GCA_001279625.1</td><td>actinobacteria</td></tr>
<tr><td>KOU20844.1</td><td>vWA+TPRs</td><td>TCAD15||Trypsin+HDxxH+VMAP-M0+VMAP-C→ MoxR→ vWA+TPRs*→ FXSXX-COOH→</td><td>1076</td><td>Streptomyces sp. WM6349</td><td>GCA_001279445.1</td><td>actinobacteria</td></tr>
<tr><td>KOU33656.1</td><td>vWA+CASPASE+TCAD12</td><td>AB_hydrolase→ CASPASE+VMAP-M2+VMAP-C→ MoxR→ vWA+CASPASE+TCAD12*→</td><td>1107</td><td>Streptomyces sp. WM6373</td><td>GCA_001279685.1</td><td>actinobacteria</td></tr>
<tr><td>KOU34863.1</td><td>vWA+TPRs</td><td>TCAD15||Trypsin+HDxxH+VMAP-M0+VMAP-C→ MoxR→ vWA+TPRs*→ FXSXX-COOH→</td><td>1077</td><td>Streptomyces sp. WM6373</td><td>GCA_001279685.1</td><td>actinobacteria</td></tr>
<tr><td>KOU55156.1</td><td>vWA+APATPase+Trypsin+TIR+FxsC_Cter(partial)</td><td>vWA+APATPase+Trypsin+TIR+FxsC_Cter(partial)*→ ||← DUF397</td><td>1147</td><td>Streptomyces sp. MMG1533</td><td>GCA_001279775.1</td><td>actinobacteria</td></tr>
<tr><td>KOU62395.1</td><td>vWA+APATPase+wHTH+TPRs</td><td>EAD2+VMAP-M0+VMAP-C→ MoxR→ vWA+APATPase+wHTH+TPRs*→ ||← Na_H_Exchanger||AB_hydrolase→ CASPASE+EAD2→ FXSXX-COOH-like→ METALLOPROTEASE+TPRs→</td><td>1532</td><td>Streptomyces sp. MMG1533</td><td>GCA_001279775.1</td><td>actinobacteria</td></tr>
<tr><td>KOU66101.1</td><td>vWA+TPRs</td><td>TCAD15||Trypsin+HDxxH+VMAP-M0+VMAP-C→ vWA+TPRs*→ Radical_SAM→ METALLOPROTEASE+TPRs→ TIR+APATPase+TPRs→</td><td>1041</td><td>Streptomyces sp. WM4235</td><td>GCA_001279725.1</td><td>actinobacteria</td></tr>
<tr><td>KOU66771.1</td><td>vWA+TPRs</td><td>TCAD15||Trypsin+HDxxH+VMAP-M0+VMAP-C→ MoxR→ vWA+TPRs*→ FXSXX-COOH→</td><td>1077</td><td>Streptomyces sp. IGB124</td><td>GCA_001279935.1</td><td>actinobacteria</td></tr>
<tr><td>KOU75547.1</td><td>vWA+TPRs</td><td>TCAD15||Trypsin+HDxxH+VMAP-M0+VMAP-C→ MoxR→ vWA+TPRs*→ FXSXX-COOH→</td><td>1077</td><td>Streptomyces sp. XY66</td><td>GCA_001279905.1</td><td>actinobacteria</td></tr>
<tr><td>KOU88266.1</td><td>vWA+TPRs</td><td>TCAD15||Trypsin+HDxxH+VMAP-M0+VMAP-C→ MoxR→ vWA+TPRs*→ FXSXX-COOH→</td><td>1077</td><td>Streptomyces sp. XY58</td><td>GCA_001279855.1</td><td>actinobacteria</td></tr>
<tr><td>KOU97671.1</td><td>vWA+TPRs</td><td>TCAD15||Trypsin+HDxxH+VMAP-M0+VMAP-C→ MoxR→ vWA+TPRs*→ FXSXX-COOH→</td><td>1076</td><td>Streptomyces sp. XY511</td><td>GCA_001279485.1</td><td>actinobacteria</td></tr>
<tr><td>KOV10493.1</td><td>vWA+TPRs</td><td>AB_hydrolase→ CASPASE+EAD2+VMAP-C→ MoxR→ vWA+TPRs*→</td><td>2147</td><td>Streptomyces sp. XY431</td><td>GCA_001279805.1</td><td>actinobacteria</td></tr>
<tr><td>KOV19775.1</td><td>vWA+TPRs</td><td>TCAD15||Trypsin+HDxxH+VMAP-M0+VMAP-C→ MoxR→ vWA+TPRs*→ FXSXX-COOH→</td><td>1077</td><td>Streptomyces sp. XY413</td><td>GCA_001279765.1</td><td>actinobacteria</td></tr>
<tr><td>KOV37678.1</td><td>vWA+TPRs</td><td>TCAD15||Trypsin+HDxxH+VMAP-M0+VMAP-C→ MoxR→ vWA+TPRs*→ FXSXX-COOH→ METALLOPROTEASE+TPRs→ TIR+APATPase+TPRs→</td><td>1077</td><td>Streptomyces sp. H021</td><td>GCA_001279495.1</td><td>actinobacteria</td></tr>
<tr><td>KOV64928.1</td><td>vWA+APATPase+wHTH+TPRs</td><td>EAD2+VMAP-M0+VMAP-C→ vWA+APATPase+wHTH+TPRs*→ ||← METALLOPROTEASE+TPRs← FXSXX-COOH-like← Trypsin+EAD2← Trypco2||METALLOPROTEASE+TPRs→</td><td>1186</td><td>Streptomyces sp. AS58</td><td>GCA_001279985.1</td><td>actinobacteria</td></tr>
<tr><td>KOV86253.1</td><td>vWA+TPRs</td><td>MoxR→ vWA+TPRs*→ Radical_SAM→ TIR+APATPase+TPRs→ Radical_SAM→ ?→ STAND_NTPase→ TIR+APATPase+TPRs→ ||← DUF397</td><td>1078</td><td>Streptomyces sp. NRRL WC-3618</td><td>GCA_001280005.1</td><td>actinobacteria</td></tr>
<tr><td>KOV97699.1</td><td>vWA+Pkinase+cNMP_cyclase+Band7+HAD</td><td>Trypco1→ Trypsin+VMAP-M0+VMAP-C→ MoxR→ vWA+Pkinase+cNMP_cyclase+Band7+HAD*→</td><td>1313</td><td>Streptomyces sp. NRRL WC-3723</td><td>GCA_001280045.1</td><td>actinobacteria</td></tr>
<tr><td>KOV97767.1</td><td>vWA+TPRs</td><td>TCAD15→ Trypco1→ Trypsin+HDxxH+VMAP-M0+VMAP-C→ MoxR→ vWA+TPRs*→ FXSXX-COOH-like→ METALLOPROTEASE+TPRs→ TIR+APATPase+TPRs→</td><td>1235</td><td>Streptomyces sp. NRRL WC-3723</td><td>GCA_001280045.1</td><td>actinobacteria</td></tr>
<tr><td>KOX08458.1</td><td>vWA+APATPase+wHTH+TPRs</td><td>EAD2+VMAP-M0+VMAP-C→ vWA+APATPase+wHTH+TPRs*→ ||← METALLOPROTEASE+TPRs</td><td>1254</td><td>Micromonospora sp. NRRL B-16802</td><td>GCA_001279555.1</td><td>actinobacteria</td></tr>
<tr><td>KOX39399.1</td><td>vWA+TPRs</td><td>TCAD15||Trypco1→ Trypsin+HDxxH+VMAP-M0+VMAP-C→ MoxR→ vWA+TPRs*→ FXSXX-COOH→ Radical_SAM→ METALLOPROTEASE+TPRs→ TIR+APATPase+TPRs→ ?→ ?→ CASPASE→</td><td>1125</td><td>Streptomyces sp. NRRL F-4707</td><td>GCA_001279605.1</td><td>actinobacteria</td></tr>
<tr><td>KOX52870.1</td><td>vWA+TPRs</td><td>TCAD15||Trypco1→ Trypsin+HDxxH+VMAP-M0+VMAP-C→ MoxR→ vWA+TPRs*→ FXSXX-COOH→ Radical_SAM→ METALLOPROTEASE+TPRs→ TIR+APATPase+TPRs→ ?→ CASPASE→ TIR+APATPase+TPRs→</td><td>1125</td><td>Streptomyces sp. NRRL F-7442</td><td>GCA_001279615.1</td><td>actinobacteria</td></tr>
<tr><td>KPC67231.1</td><td>vWA+TPRs</td><td>TCAD15||Trypco1→ Trypsin+HDxxH+VMAP-M0+VMAP-C→ MoxR→ vWA+TPRs*→ FXSXX-COOH→ Radical_SAM→ TIR+APATPase+TPRs→</td><td>1077</td><td>Streptomyces chattanoogensis</td><td>GCA_001294335.1</td><td>actinobacteria</td></tr>
<tr><td>KPC72055.1</td><td>vWA+APATPase+wHTH+TPRs</td><td>EAD2+VMAP-M0+VMAP-C→ MoxR→ vWA+APATPase+wHTH+TPRs*→ ||← Na_H_Exchanger||AB_hydrolase→ CASPASE+EAD2→ FXSXX-COOH-like→ METALLOPROTEASE+TPRs→</td><td>1503</td><td>Streptomyces sp. NRRL WC-3753</td><td>GCA_001294395.1</td><td>actinobacteria</td></tr>
<tr><td>KPC90543.1</td><td>vWA+TPRs</td><td>TCAD15→ ?→ ?→ Trypco1→ Trypsin+HDxxH+VMAP-M0+VMAP-C→ MoxR→ vWA+TPRs*→ METALLOPROTEASE+TPRs→ TIR+APATPase+TPRs→</td><td>1054</td><td>Streptomyces sp. NRRL F-6602</td><td>GCA_001294325.1</td><td>actinobacteria</td></tr>
<tr><td>KPH99459.1</td><td>vWA+BetaPropeller</td><td>AB_hydrolase→ CASPASE+VMAP-M2+VMAP-C→ MoxR→ vWA+BetaPropeller*→</td><td>852</td><td>Actinobacteria bacterium OK006</td><td>GCA_001298565.1</td><td>actinobacteria</td></tr>
<tr><td>KPH99925.1</td><td>vWA+APATPase+wHTH+TPRs</td><td>EAD2+VMAP-M0+VMAP-C→ MoxR→ vWA+APATPase+wHTH+TPRs*→ ||← Na_H_Exchanger||AB_hydrolase→ CASPASE+EAD2→ METALLOPROTEASE+TPRs→</td><td>1498</td><td>Actinobacteria bacterium OV450</td><td>GCA_001298555.1</td><td>actinobacteria</td></tr>
<tr><td>KPI09079.1</td><td>vWA+APATPase+wHTH+TPRs</td><td>EAD2+TIR→ EAD2+VMAP-M0+VMAP-C→ MoxR→ vWA+APATPase+wHTH+TPRs*→ ||← METALLOPROTEASE+TPRs</td><td>1290</td><td>Actinobacteria bacterium OK006</td><td>GCA_001298565.1</td><td>actinobacteria</td></tr>
<tr><td>KPI10108.1</td><td>vWA+APATPase+wHTH+TPRs</td><td>EAD2+VMAP-M0+VMAP-C→ vWA+APATPase+wHTH+TPRs*→ ||← METALLOPROTEASE+TPRs← FXSXX-COOH-like← Trypsin+EAD2← Trypco2||FXSXX-COOH-like→ METALLOPROTEASE+TPRs→</td><td>1317</td><td>Actinobacteria bacterium OK006</td><td>GCA_001298565.1</td><td>actinobacteria</td></tr>
<tr><td>KPI17053.1</td><td>vWA+Pkinase+ParA-Soj-PloopNTPase(SIMIBI)</td><td>NUDIX→ Trypco1→ Trypsin+EAD2+VMAP-M0+VMAP-C→ MoxR→ vWA+Pkinase+ParA-Soj-PloopNTPase(SIMIBI)*→ SIR2→</td><td>1168</td><td>Actinobacteria bacterium OK074</td><td>GCA_001298575.1</td><td>actinobacteria</td></tr>
<tr><td>KPI25952.1</td><td>vWA+TPRs</td><td>TCAD15||Trypco1→ Trypsin+HDxxH+VMAP-M0+VMAP-C→ MoxR→ vWA+TPRs*→ FXSXX-COOH→ Radical_SAM→ METALLOPROTEASE+TPRs→ TIR+APATPase+TPRs→</td><td>1165</td><td>Actinobacteria bacterium OV320</td><td>GCA_001298545.1</td><td>actinobacteria</td></tr>
<tr><td>KPI32272.1</td><td>vWA+cNMP_cyclase+Band7+HAD</td><td>Trypco1→ Trypsin+VMAP-M0+VMAP-C→ MoxR→ vWA+cNMP_cyclase+Band7+HAD*→</td><td>1122</td><td>Actinobacteria bacterium OV320</td><td>GCA_001298545.1</td><td>actinobacteria</td></tr>
<tr><td>KPQ32127.1</td><td>vWA+FGS-fold_domain</td><td>Trypco1→ Trypsin+VMAP-M1+VMAP-C→ MoxR→ vWA+FGS-fold_domain*→ FGS-fold_domain→ ||← RVT_1← four_helix_bundle_protein</td><td>920</td><td>Phormidesmis priestleyi Ana</td><td>GCA_001314865.1</td><td>cyanobacteria</td></tr>
<tr><td>KPQ33019.1</td><td>vWA+FGS-fold_domain</td><td>EAD1+Trypsin→ ?→ EAD1+VMAP-M1+VMAP-C→ MoxR→ vWA+FGS-fold_domain*→ FGS-fold_domain→ FGS-fold_domain→</td><td>1004</td><td>Phormidesmis priestleyi Ana</td><td>GCA_001314865.1</td><td>cyanobacteria</td></tr>
<tr><td>KQC35451.1</td><td>vWA+APATPase+wHTH+TPRs</td><td>EAD2+EAD1→ EAD2+VMAP-M0+VMAP-C→ MoxR→ vWA+APATPase+wHTH+TPRs*→ ||← FXSXX-COOH-like</td><td>1199</td><td>Frankia sp. ACN1ag</td><td>GCA_001414035.1</td><td>actinobacteria</td></tr>
<tr><td>KQM03129.1</td><td>vWA+APATPase+wHTH+TPRs</td><td>EAD2+EAD1→ EAD2+VMAP-M0+VMAP-C→ MoxR→ vWA+APATPase+wHTH+TPRs*→ ||← METALLOPROTEASE+TPRs← FXSXX-COOH-like</td><td>1380</td><td>Frankia sp. CpI1-P</td><td>GCA_001421075.1</td><td>actinobacteria</td></tr>
<tr><td>KRD17930.1</td><td>vWA+APATPase+wHTH+TPRs</td><td>DUF397→ MoxR→ vWA+APATPase+wHTH+TPRs*→ ||← vWA← EAD2+VMAP-M0+VMAP-C</td><td>870</td><td>Streptomyces sp. Root264</td><td>GCA_001428885.1</td><td>actinobacteria</td></tr>
<tr><td>KST64955.1</td><td>vWA+Ntox3+MACRODOMAIN+FGS-fold_domain</td><td>EAD1+VMAP-M1+VMAP-C→ MoxR→ vWA+Ntox3+MACRODOMAIN+FGS-fold_domain*→</td><td>1071</td><td>Mastigocoleus testarum BC008</td><td>GCA_001456025.1</td><td>cyanobacteria</td></tr>
<tr><td>KUF20465.1</td><td>vWA+TPRs</td><td>Trypsin+HDxxH+VMAP-M0+VMAP-C→ MoxR→ vWA+TPRs*→</td><td>1213</td><td>Streptomyces silvensis</td><td>GCA_001482415.1</td><td>actinobacteria</td></tr>
</tbody>
</table>

acc	architecture	operon	len	species	gca	taxend
KUH39613.1	vWA_protein	AB_hydrolase→ CASPASE+VMAP-M2+VMAP-C→ MoxR→ vWA_protein*→ vWA(partial)+APATPase+wHTH+cNMP_cyclase+NaeI→	502	Streptomyces kanasensis	GCA_001484625.1	actinobacteria
KUJ68393.1	vWA+APATPase+wHTH+TPRs	PNP_UDP_1+EAD2→ EAD2+VMAP-M0+VMAP-C→ MoxR→ vWA+APATPase+wHTH+TPRs*→ ||← PSE← FXSXX-COOH-like	1473	Streptomyces albus subsp. albus	GCA_001507455.1	actinobacteria
KUL22000.1	vWA+APATPase+wHTH+TPRs	FXSXX-COOH-like→ METALLOPROTEASE+TPRs→ PNP_UDP_1+VMAP-M0+VMAP-C→ vWA+APATPase+wHTH+TPRs*→	1255	Actinoplanes awajinensis subsp. mycoplanecinus	GCA_001509495.1	actinobacteria
KUL67220.1	vWA+Pkinase+ZNR+Pkinase+ParA-Soj-PloopNTPase(SIMIBI)	SIR2→ ||← ?← ?||Trypco1→ Trypsin+EAD2+VMAP-M0+VMAP-C→ MoxR→ vWA+Pkinase+ZNR+Pkinase+ParA-Soj-PloopNTPase(SIMIBI)*→ Porphobil_deam+Porphobil_deamC→	1367	Streptomyces violaceusniger	GCA_001509775.1	actinobacteria
KUN02935.1	vWA+Pkinase+ParA-Soj-PloopNTPase(SIMIBI)	Trypco1→ Trypsin+EAD2+VMAP-M0+VMAP-C→ MoxR→ vWA+Pkinase+ParA-Soj-PloopNTPase(SIMIBI)*→	1115	Streptomyces yokosukanensis	GCA_001514035.1	actinobacteria
KUN19774.1	vWA+TIR+APATPase+wHTH+TPRs	DRHyd+VMAP-M0+VMAP-C→ MoxR→ vWA+TIR+APATPase+wHTH+TPRs*→	1340	Streptomyces antibioticus	GCA_001514065.1	actinobacteria
KUN28796.1	vWA+Pkinase+wHTH+TPRs+cNMP_cyclase+Band7+HAD	Trypco1→ PSE→ MoxR→ vWA+Pkinase+wHTH+TPRs+cNMP_cyclase+Band7+HAD*→	1412	Streptomyces antibioticus	GCA_001514065.1	actinobacteria
KUN39593.1	vWA+Pkinase+wHTH+TPRs+cNMP_cyclase+Band7+HAD	Trypco1→ Trypsin+VMAP-M0+VMAP-C→ MoxR→ vWA+Pkinase+wHTH+TPRs+cNMP_cyclase+Band7+HAD*→	1526	Streptomyces longwoodensis	GCA_001514125.1	actinobacteria
KUN44905.1	vWA+APATPase+wHTH+TPRs	EAD2+VMAP-M0+VMAP-C→ vWA+APATPase+wHTH+TPRs*→ ||← METALLOPROTEASE+TPRs← Trypsin+EAD2← Trypco2||METALLOPROTEASE+TPRs→	1296	Streptomyces olivochromogenes	GCA_001514115.1	actinobacteria
KUN49123.1	vWA+APATPase+wHTH+TPRs+cNMP_cyclase+NaeI	AB_hydrolase→ CASPASE+VMAP-M2+VMAP-C→ MoxR→ vWA+APATPase+wHTH+TPRs+cNMP_cyclase+NaeI*→	1667	Streptomyces avermitilis	GCA_001514135.1	actinobacteria
KUN87228.1	vWA+Pkinase+wHTH+TPRs+cNMP_cyclase+Band7+HAD	Trypco1→ Trypsin+VMAP-M0+VMAP-C→ MoxR→ vWA+Pkinase+wHTH+TPRs+cNMP_cyclase+Band7+HAD*→	1443	Streptomyces griseoruber	GCA_001514205.1	actinobacteria
KUN89326.1	vWA+APATPase+wHTH+TPRs	Trypsin+EAD2+VMAP-M0+VMAP-C→ vWA+APATPase+wHTH+TPRs*→ KAP_NTPase→	806	Streptomyces griseoruber	GCA_001514205.1	actinobacteria
KUN98985.1	vWA+Pkinase+ParA-Soj-PloopNTPase(SIMIBI)	Trypco1→ Trypsin+EAD2+VMAP-M0+VMAP-C→ MoxR→ vWA+Pkinase+ParA-Soj-PloopNTPase(SIMIBI)*→	1105	Streptomyces caeruleatus	GCA_001514235.1	actinobacteria
KUO22454.1	vWA+Pkinase+wHTH+TPRs+cNMP_cyclase+Band7+HAD	Trypco1→ Trypsin+VMAP-M0+VMAP-C→ MoxR→ vWA+Pkinase+wHTH+TPRs+cNMP_cyclase+Band7+HAD*→	1396	Streptomyces sp. RV15	GCA_001514305.1	actinobacteria
KYK10151.1	vWA+Pkinase+wHTH+TPRs+cNMP_cyclase	Trypco1→ MoxR→ vWA+Pkinase+wHTH+TPRs+cNMP_cyclase*→	991	Streptomyces sp. CC71	GCA_001595515.1	actinobacteria
KYK14919.1	vWA+APATPase+wHTH+TPRs	vWA+APATPase+wHTH+TPRs*→ ||← Na_H_Exchanger	1391	Streptomyces sp. CC71	GCA_001595515.1	actinobacteria
OBQ03799.1	vWA+FGS-fold_domain	Trypco1→ Trypsin+VMAP-M1+VMAP-C→ MoxR→ vWA+FGS-fold_domain*→	870	Anabaena sp. AL09	GCA_001672255.1	cyanobacteria
OBQ11326.1	vWA+FGS-fold_domain	Trypco1→ Trypsin+VMAP-M1+VMAP-C→ MoxR→ vWA+FGS-fold_domain*→	871	Anabaena sp. LE011-02	GCA_001672225.1	cyanobacteria
OBQ20858.1	vWA+FGS-fold_domain	vWA+FGS-fold_domain*→	880	Anabaena sp. AL93	GCA_001672085.1	cyanobacteria
OBQ35051.1	vWA+FGS-fold_domain	Trypco1→ VMAP-M1+EAD6+VMAP-C→ MoxR→ vWA+FGS-fold_domain*→	789	Anabaena sp. CRKS33	GCA_001672075.1	cyanobacteria
OBQ40142.1	vWA+FGS-fold_domain	Trypco1→ MoxR→ vWA+FGS-fold_domain*→	870	Anabaena sp. MDT14b	GCA_001672195.1	cyanobacteria
OBQ42247.1	vWA+beta-rich-region+HTH+FGS-fold_domain	cNMP_cyclase+EAD1→ EAD1+VMAP-M24+VMAP-C→ MoxR→ vWA+beta-rich-region+HTH+FGS-fold_domain*→ ?→ ||← RVT_1← four_helix_bundle_protein← FGS-fold_domain	1075	Aphanizomenon flos-aquae WA102	GCA_001672105.1	cyanobacteria
OEV11574.1	vWA	TCAD15||Trypco1→ Trypsin+HDxxH+VMAP-M0+VMAP-C→ MoxR→ vWA*→	299	Streptomyces nanshensis	GCA_001751255.1	actinobacteria
OEV17914.1	vWA	AB_hydrolase→ CASPASE+VMAP-M2+VMAP-C→ MoxR→ vWA→ vWA*→	369	Streptomyces nanshensis	GCA_001751305.1	actinobacteria
OEV17915.1	vWA	AB_hydrolase→ CASPASE+VMAP-M2+VMAP-C→ MoxR→ vWA*→ vWA→	354	Streptomyces nanshensis	GCA_001751305.1	actinobacteria
OEV28767.1	vWA	Trypsin+HDxxH+VMAP-M0→ Trypsin+HDxxH+VMAP-M0+VMAP-C→ MoxR→ vWA*→ ?→ TIR+APATPase+TPRs→	606	Streptomyces nanshensis	GCA_001751265.1	actinobacteria
OHV36996.1	vWA+APATPase+wHTH+TPRs	EAD2+VMAP-M0+VMAP-C→ MoxR→ vWA+APATPase+wHTH+TPRs*→ ||← ?||EAD2+VMAP-M0+VMAP-C→ MoxR→ vWA+APATPase+wHTH+TPRs→ ||← METALLOPROTEASE+TPRs	1397	Frankia sp. EUN1h	GCA_001854645.1	actinobacteria
OHV37160.1	vWA+APATPase+wHTH+TPRs	EAD2+VMAP-M0+VMAP-C→ MoxR→ vWA+APATPase+wHTH+TPRs→ ||← ?||EAD2+VMAP-M0+VMAP-C→ MoxR→ vWA+APATPase+wHTH+TPRs*→ ||← METALLOPROTEASE+TPRs	1281	Frankia sp. EUN1h	GCA_001854645.1	actinobacteria
OIJ95017.1	vWA+SFII-helicase+DUF3427	AB_hydrolase→ CASPASE+VMAP-M2+VMAP-C→ MoxR→ vWA+SFII-helicase+DUF3427*→	1325	Streptomyces sp. MUSC 93	GCA_001866645.1	actinobacteria
OIK25119.1	vWA+APATPase+wHTH+TPRs	Trypco2→ EAD2+VMAP-M0+VMAP-C→ vWA+APATPase+wHTH+TPRs*→ ||← PSE← FXSXX-COOH-like← Trypsin+EAD2	1218	Streptomyces malaysiense	GCA_000980885.2	actinobacteria
OKH35591.1	vWA+FGS-fold_domain	cNMP_cyclase+VMAP-M1+VMAP-C→ MoxR→ vWA+FGS-fold_domain*→ FGS-fold_domain→	863	Phormidium ambiguum IAM M-71	GCA_001904725.1	cyanobacteria
OKI00871.1	vWA+Pkinase+ParA-Soj-PloopNTPase(SIMIBI)	Trypco1→ Trypsin+EAD2+VMAP-M0+VMAP-C→ MoxR→ vWA+Pkinase+ParA-Soj-PloopNTPase(SIMIBI)*→ ?→ NUDIX→	1137	Streptomyces sp. CB02923	GCA_001905005.1	actinobacteria
OKI13910.1	vWA+APATPase+wHTH+TPRs	EAD2+VMAP-M0+VMAP-C→ MoxR→ vWA+APATPase+wHTH+TPRs*→ ||← METALLOPROTEASE+TPRs← FXSXX-COOH-like	1444	Saccharothrix sp. CB00851	GCA_001905065.1	actinobacteria
OKI29456.1	vWA+TPRs	TCAD15||Trypsin+HDxxH+VMAP-M0+VMAP-C→ MoxR→ vWA+TPRs*→ FXSXX-COOH→ Radical_SAM→ METALLOPROTEASE+TPRs→ TIR+APATPase+TPRs→	1076	Streptomyces sp. CB03578	GCA_001905025.1	actinobacteria
OKI29918.1	vWA+APATPase+wHTH+TPRs	EAD2+VMAP-M0+VMAP-C→ vWA+APATPase+wHTH+TPRs*→ ||← METALLOPROTEASE+TPRs← cNMP_cyclase+EAD2	1294	Saccharothrix sp. CB00851	GCA_001905065.1	actinobacteria
OKI39951.1	vWA+APATPase+wHTH+TPRs	cNMP_binding+HTH_Crp_2← ?||EAD2+VMAP-M0+VMAP-C→ MoxR→ vWA+APATPase+wHTH+TPRs*→ ||← Na_H_Exchanger||AB_hydrolase→ CASPASE+EAD2→ FXSXX-COOH-like→ METALLOPROTEASE+TPRs→	1508	Streptomyces sp. CB03578	GCA_001905025.1	actinobacteria
OKI55839.1	vWA+APATPase+wHTH+TPRs	EAD2+VMAP-M0+VMAP-C→ MoxR→ vWA+APATPase+wHTH+TPRs*→ AB_hydrolase→ CASPASE+EAD2→ METALLOPROTEASE+TPRs→	1328	Streptomyces sp. CB00072	GCA_001905575.1	actinobacteria
OKI62008.1	vWA+APATPase+wHTH+TPRs	EAD2+VMAP-M0+VMAP-C→ MoxR→ vWA+APATPase+wHTH+TPRs*→ ||← Na_H_Exchanger||AB_hydrolase→ CASPASE+EAD2→ METALLOPROTEASE+TPRs→	1522	Streptomyces sp. MJM1172	GCA_001905425.1	actinobacteria
OKI68699.1	vWA+APATPase+wHTH+TPRs	EAD2+VMAP-M0+VMAP-C→ MoxR→ vWA+APATPase+wHTH+TPRs*→ AB_hydrolase→ CASPASE+EAD2→ FXSXX-COOH-like→ METALLOPROTEASE+TPRs→	1347	Streptomyces sp. TSRI0445	GCA_001905405.1	actinobacteria
OKI79079.1	vWA+APATPase+wHTH+TPRs	EAD2+VMAP-M0+VMAP-C→ MoxR→ vWA+APATPase+wHTH+TPRs*→ AB_hydrolase→ CASPASE+EAD2→ FXSXX-COOH-like→ METALLOPROTEASE+TPRs→	1329	Streptomyces sp. TSRI0395	GCA_001905525.1	actinobacteria
OKJ43967.1	vWA+TPRs	TCAD15||Trypco1→ Trypsin+HDxxH+VMAP-M0+VMAP-C→ MoxR→ vWA+TPRs*→ FXSXX-COOH→ Radical_SAM→ METALLOPROTEASE+TPRs→ TIR+APATPase+TPRs→	1132	Streptomyces sp. CB01580	GCA_001905795.1	actinobacteria
OKJ54712.1	vWA+APATPase+wHTH+TPRs	EAD2+VMAP-M0+VMAP-C→ MoxR→ vWA+APATPase+wHTH+TPRs*→ AB_hydrolase→ CASPASE+EAD2→ FXSXX-COOH-like→ METALLOPROTEASE+TPRs→	1329	Streptomyces sp. CB02115	GCA_001905645.1	actinobacteria
OKJ83696.1	vWA+APATPase+wHTH+TPRs	EAD2+VMAP-M0+VMAP-C→ MoxR→ vWA+APATPase+wHTH+TPRs*→ AB_hydrolase→ CASPASE+EAD2→ FXSXX-COOH-like→ METALLOPROTEASE+TPRs→	1358	Streptomyces sp. CB01883	GCA_001905855.1	actinobacteria
OKJ89683.1	vWA+APATPase+wHTH+TPRs	EAD2+VMAP-M0+VMAP-C→ MoxR→ vWA+APATPase+wHTH+TPRs*→ ||← Na_H_Exchanger||AB_hydrolase→ CASPASE+EAD2→ FXSXX-COOH-like→ METALLOPROTEASE+TPRs→	1489	Streptomyces sp. TSRI0107	GCA_001905845.1	actinobacteria
OKK02915.1	vWA+APATPase+wHTH+TPRs	AB_hydrolase→ EAD2+VMAP-M0+VMAP-C→ MoxR→ vWA+APATPase+wHTH+TPRs*→ ||← METALLOPROTEASE+TPRs← FXSXX-COOH-like	1279	Streptomyces sp. CB03234	GCA_001905885.1	actinobacteria
OKK13505.1	vWA+APATPase+wHTH+TPRs	EAD2+VMAP-M0+VMAP-C→ MoxR→ vWA+APATPase+wHTH+TPRs*→ FXSXX-COOH-like→ METALLOPROTEASE+TPRs→	1426	Streptomyces sp. CB02400	GCA_001905725.1	actinobacteria
OKK20627.1	vWA+TPRs	TCAD15||Trypsin+HDxxH+VMAP-M0+VMAP-C→ MoxR→ vWA+TPRs*→ FXSXX-COOH→ Radical_SAM→ METALLOPROTEASE+TPRs→ TIR+APATPase+TPRs→	1122	Streptomyces sp. CB00455	GCA_001905445.1	actinobacteria

acc	architecture	operon	len	species	gca	taxend
OLC62284.1	vWA+Glyco_transf_20	MoxR→ vWA+Glyco_transf_20*→	1123	Ktedonobacter sp. 13_1_40CM_4_52_4	GCA_001918255.1	chloroflexi
OLO30728.1	vWA+TIR+FxsC_Cter	← PNP_UDP_1+EAD2||AB_hydrolase→ CASPASE+VMAP-M2+VMAP-C→ MoxR→ vWA+TIR+FxsC_Cter*→	1009	Streptomyces sp. MNU77	GCA_000935135.2	actinobacteria
OLZ67587.1	vWA+APATPase+wHTH+TPRs	EAD2+VMAP-M0+VMAP-C→ MoxR→ vWA+APATPase+wHTH+TPRs*→ ||← METALLOPROTEASE+TPRs← FXSXX-COOH-like	1282	Streptomyces sp. IMTB 2501	GCA_001953885.1	actinobacteria
OLZ74128.1	vWA+TPRs	← TCAD15||Trypsin+HDxxH+VMAP-M0+VMAP-C→ MoxR→ vWA+TPRs*→ FXSXX-COOH→ Radical_SAM→ METALLOPROTEASE+TPRs→ TIR+APATPase+TPRs→	1094	Streptomyces amritsarensis	GCA_001953875.1	actinobacteria
OMI34266.1	vWA+APATPase+wHTH+TPRs	PNP_UDP_1+EAD2→ EAD2+VMAP-M0+VMAP-C→ MoxR→ vWA+APATPase+wHTH+TPRs*→ ||← METALLOPROTEASE+TPRs← FXSXX-COOH-like	1440	Streptomyces sparsogenes DSM 40356	GCA_001969965.1	actinobacteria
OMI41127.1	vWA+Pkinase+Pkinase	vWA+Pkinase+Pkinase*→ Porphobil_deam+Porphobil_deamC→	836	Streptomyces sparsogenes DSM 40356	GCA_001969965.1	actinobacteria
ONH30898.1	vWA+APATPase+wHTH+TPRs	EAD2+VMAP-M0+VMAP-C→ MoxR→ vWA+APATPase+wHTH+TPRs*→ TIR+APATPase+TPRs→ ||← METALLOPROTEASE+TPRs	1329	Frankia sp. M16386	GCA_001983105.1	actinobacteria
OOC02455.1	vWA+APATPase+wHTH+TPRs	cNMP_cyclase+VMAP-M0+VMAP-C→ MoxR→ vWA+APATPase+wHTH+TPRs*→ ||← METALLOPROTEASE+TPRs← FXSXX-COOH-like← cNMP_binding+HTH_Crp_2	1202	Amycolatopsis azurea DSM 43854	GCA_001995215.1	actinobacteria
OOQ46879.1	vWA+APATPase+wHTH+TPRs	Trypsin+EAD2+VMAP-C→ vWA+APATPase+wHTH+TPRs*→ KAP_NTPase→	805	Streptomyces antibioticus	GCA_002019855.1	actinobacteria
OOQ51257.1	vWA+APATPase+wHTH+TPRs	vWA+APATPase+wHTH+TPRs*→ ||← METALLOPROTEASE+TPRs← FXSXX-COOH-like← Trypsin+EAD2||METALLOPROTEASE+TPRs→	1250	Streptomyces antibioticus	GCA_002019855.1	actinobacteria
OOQ52337.1	vWA+Pkinase+wHTH+TPRs+cNMP_cyclase+Band7+HAD	Trypco1→ Trypsin+VMAP-M0+VMAP-C→ MoxR→ vWA+Pkinase+wHTH+TPRs+cNMP_cyclase+Band7+HAD*→	1161	Streptomyces antibioticus	GCA_002019855.1	actinobacteria
OQY51160.1	vWA_protein	VMAP-C(partial)→ VMAP-C(partial)→ ?→ ?→ ?→ MoxR→ vWA_protein*→	588	Beggiatoa sp. 4572_84	GCA_002085445.1	gammaproteobacteria
OSC70555.1	vWA+TPRs	AB_hydrolase→ CASPASE+EAD2→ VMAP-M0+VMAP-C→ MoxR→ vWA+TPRs*→	1178	Streptomyces sp. 4F	GCA_002104855.1	actinobacteria
OSC74050.1	vWA(partial)+REase+STAND_NTPase+TPRs+cNMP_cyclase	vWA(partial)+REase+STAND_NTPase+TPRs+cNMP_cyclase*→	1256	Streptomyces sp. BF-3	GCA_002104865.1	actinobacteria
OSZ59308.1	vWA+Pkinase+ParA-Soj-PloopNTPase(SIMIBI)	Trypco1→ Trypsin+EAD2+VMAP-M0+VMAP-C→ MoxR→ vWA+Pkinase+ParA-Soj-PloopNTPase(SIMIBI)*→	1106	Streptomyces pharetrae CZA14	GCA_002128465.1	actinobacteria
OSZ60893.1	vWA+TIR+APATPase+Trypsin+HDxxH+VMAP-M0+VMAP-C	← TCAD15||PSE→ Trypsin+HDxxH+VMAP-M0+VMAP-C→ MoxR→ vWA+TIR+APATPase+Trypsin+HDxxH+VMAP-M0+VMAP-C*→	1351	Streptomyces pharetrae CZA14	GCA_002128465.1	actinobacteria
OUC12065.1	vWA_protein	Trypsin+VMAP-M1+VMAP-C→ MoxR→ vWA_protein*→ FGS-fold_domain→ FGS-fold_domain→	410	Alkalinema sp. CACIAM 70d	GCA_002148405.1	cyanobacteria
PBC92565.1	vWA+cNMP_binding+HTH_Crp_2+TerD	AB_hydrolase→ CASPASE+VMAP-M0+VMAP-C→ MoxR→ vWA+cNMP_binding+HTH_Crp_2+TerD*→	1494	Streptomyces sp. Ag82_O1-15	GCA_002300485.1	actinobacteria
PDW02245.1	vWA+Pkinase+REC+TCAD9+APHkinase	vWA+Pkinase+REC+TCAD9+APHkinase*→	1297	Chloroflexi bacterium Kir15-3F	GCA_002532535.1	chloroflexi
PJN27976.1	vWA+BetaPropeller+Pkinase+TIR	Trypco1→ Trypsin+EAD2+VMAP-M0+VMAP-C→ MoxR→ vWA+BetaPropeller+Pkinase+TIR*→	1356	Kitasatospora sp. CB02891	GCA_002803145.1	actinobacteria
SBT88418.1	vWA+Pkinase+wHTH+TPRs+cNMP_cyclase+Band7+HAD	Trypco1→ Trypsin+VMAP-M0+VMAP-C→ MoxR→ vWA+Pkinase+wHTH+TPRs+cNMP_cyclase+Band7+HAD*→	1567	Streptomyces sp. DI166	GCA_900091315.1	actinobacteria
SBT93465.1	vWA+HTH	CASPASE+VMAP-M0+VMAP-C→ vWA+HTH*→	729	Streptomyces sp. DI166	GCA_900091315.1	actinobacteria
SBT93723.1	vWA_protein	AB_hydrolase→ CASPASE+VMAP-M0+VMAP-C→ MoxR→ vWA_protein*→ Trypsin+CASPASE+S1COLD→	608	Streptomyces sp. DI166	GCA_900091315.1	actinobacteria
SBT94501.1	vWA+APATPase+wHTH+TPRs	EAD2+VMAP-M0+VMAP-C→ vWA+APATPase+wHTH+TPRs*→	1320	Streptomyces sp. DI166	GCA_900091315.1	actinobacteria
SBU89710.1	vWA+TIR+FxsC_Cter	AB_hydrolase→ CASPASE+VMAP-M2+VMAP-C→ MoxR→ vWA+TIR+FxsC_Cter*→	1001	Streptomyces sp. MnatMP-M77	GCA_900090075.1	actinobacteria
SBU92090.1	vWA+Pkinase+ParA-Soj-PloopNTPase(SIMIBI)	Trypco1→ Trypsin+EAD2+VMAP-M0+VMAP-C→ MoxR→ vWA+Pkinase+ParA-Soj-PloopNTPase(SIMIBI)*→	1070	Streptomyces sp. MnatMP-M77	GCA_900090075.1	actinobacteria
SBU92604.1	vWA+APATPase+wHTH+TPRs	EAD2+VMAP-M0+VMAP-C→ MoxR→ vWA+APATPase+wHTH+TPRs*→ AB_hydrolase→ CASPASE+EAD2→ FXSXX-COOH-like→ METALLOPROTEASE+TPRs→	1457	Streptomyces sp. OspMP-M45	GCA_900090065.1	actinobacteria
SBV04727.1	vWA+REase+STAND_NTPase+SFI-helicase	DUF397→ Trypco1→ Trypsin+EAD2+VMAP-M0+VMAP-C→ MoxR→ vWA+REase+STAND_NTPase+SFI-helicase*→	1640	Streptomyces sp. Ncost-T6T-1	GCA_900090085.1	actinobacteria
SBV07778.1	vWA+TPRs+TIR+FxsC_Cter(partial)	AB_hydrolase→ CASPASE+VMAP-M2+VMAP-C→ MoxR→ vWA+TPRs+TIR+FxsC_Cter(partial)*→	1422	Streptomyces sp. MnatMP-M77	GCA_900090075.1	actinobacteria
SCD36331.1	vWA+APATPase+wHTH+TPRs	EAD2+VMAP-M0+VMAP-C→ MoxR→ vWA+APATPase+wHTH+TPRs*→ AB_hydrolase→ CASPASE+EAD2→ FXSXX-COOH-like→ METALLOPROTEASE+TPRs→	1457	Streptomyces sp. PalvLS-984	GCA_900091765.1	actinobacteria
SCD66673.1	vWA+APATPase+wHTH+TPRs	EAD2+VMAP-M0+VMAP-C→ MoxR→ vWA+APATPase+wHTH+TPRs*→ AB_hydrolase→ CASPASE+EAD2→ FXSXX-COOH-like→ METALLOPROTEASE+TPRs→	1457	Streptomyces sp. PpalLS-921	GCA_900091835.1	actinobacteria
SCD67020.1	vWA+APATPase+wHTH+TPRs	EAD2+VMAP-M0+VMAP-C→ MoxR→ ?→ vWA+APATPase+wHTH+TPRs*→ AB_hydrolase→	1364	Streptomyces sp. BpilaLS-43	GCA_900091875.1	actinobacteria
SCD89054.1	vWA+REase+SWACOS+TPRs+cNMP_cyclase	AB_hydrolase→ CASPASE+VMAP-M2+VMAP-C→ MoxR→ vWA+REase+SWACOS+TPRs+cNMP_cyclase*→	1705	Streptomyces sp. DvalAA-19	GCA_900091865.1	actinobacteria
SCD99771.1	vWA_protein	VMAP-C→ MoxR→ vWA_protein*→	835	Streptomyces sp. TverLS-915	GCA_900091755.1	actinobacteria
SCE03681.1	vWA+TPRs	vWA+TPRs*→ FXSXX-COOH→ Radical_SAM→	1026	Streptomyces sp. BvitLS-983	GCA_900091785.1	actinobacteria
SCE24206.1	vWA+TPRs	← TCAD15||Trypco1→ Trypsin+HDxxH+VMAP-M0+VMAP-C→ MoxR→ vWA+TPRs*→ FXSXX-COOH→	1137	Streptomyces sp. ScaeMP-6W	GCA_900091885.1	actinobacteria
SCE36538.1	vWA+APATPase+wHTH+TPRs	vWA+APATPase+wHTH+TPRs*→ ||← METALLOPROTEASE+TPRs← FXSXX-COOH-like||Trypco1→	1363	Streptomyces sp. SolWspMP-5a-2	GCA_900091845.1	actinobacteria
SCE53113.1	vWA+TPRs	vWA+TPRs*→	1050	Streptomyces sp. DvalAA-14	GCA_900091855.1	actinobacteria
SCE55987.1	vWA+TPRs	AB_hydrolase→ CASPASE+VMAP-M2+VMAP-C→ MoxR→ vWA+TPRs*→	666	Streptomyces sp. OspMP-M43	GCA_900091905.1	actinobacteria
SCE56241.1	vWA+TPRs	← TCAD15||Trypco1→ Trypsin+HDxxH+VMAP-M0+VMAP-C→ MoxR→ vWA+TPRs*→ FXSXX-COOH→ Radical_SAM→ METALLOPROTEASE+TPRs→ TIR+APATPase+TPRs→ Trypco1→	1134	Streptomyces sp. DvalAA-43	GCA_900091725.1	actinobacteria
SCE64837.1	vWA+TPRs	Trypsin+HDxxH+VMAP-M0+VMAP-C→ MoxR→ vWA+TPRs*→ ?→ Radical_SAM→ TIR+APATPase+TPRs→ ||← Voltage_CLC	1129	Micromonospora haikouensis	GCA_900091595.1	actinobacteria
SCF42109.1	vWA_protein	CASPASE(partial)+VMAP-M2+VMAP-C→ MoxR→ vWA_protein*→	1041	Micromonospora matsumotoense	GCA_900091525.1	actinobacteria
SCF49061.1	vWA+TPRs	VMAP-C→ MoxR→ ?→ vWA+TPRs*→ FXSXX-COOH→ Radical_SAM→ METALLOPROTEASE+TPRs→ TIR+APATPase+TPRs→ Trypco1→	1042	Streptomyces sp. LcepLS	GCA_900091485.1	actinobacteria
SCF56449.1	vWA+STAND_NTPase+BetaPropeller+REase(Mrr_cat-PDDEXK)	AB_hydrolase→ CASPASE+VMAP-M0+VMAP-C→ MoxR→ vWA+STAND_NTPase+BetaPropeller+REase(Mrr_cat-PDDEXK)*→	1067	Streptomyces sp. Ncost-T10-10d	GCA_900091955.1	actinobacteria
SCF58287.1	vWA+Pkinase+cNMP_cyclase+Band7+HAD	VMAP-C(partial)→ MoxR→ vWA+Pkinase+cNMP_cyclase+Band7+HAD*→	1314	Streptomyces sp. LamerLS-31b	GCA_900091975.1	actinobacteria

<table>
<tr><th>acc</th><th>architecture</th><th>operon</th><th>len</th><th>species</th><th>gca</th><th>taxend</th></tr>
<tr><td>SCF72582.1</td><td>vWA+TPRs</td><td>vWA+TPRs*→ FXSXX-COOH→ Radical_SAM→ METALLOPROTEASE+TPRs→ TIR+APATPase+TPRs→</td><td>980</td><td>Streptomyces sp. Ncost-T10-10d</td><td>GCA_900091955.1</td><td>actinobacteria</td></tr>
<tr><td>SCF77036.1</td><td>vWA+TIR+FxsC_Cter</td><td>AB_hydrolase→ CASPASE+VMAP-M2+VMAP-C→ MoxR→ vWA+TIR+FxsC_Cter*→</td><td>1071</td><td>Streptomyces sp. MnatMP-M17</td><td>GCA_900091995.1</td><td>actinobacteria</td></tr>
<tr><td>SCF89167.1</td><td>vWA+APATPase+wHTH+TPRs</td><td>Trypco2→ EAD2+VMAP-M0+VMAP-C→ vWA+APATPase+wHTH+TPRs*→ ||← METALLOPROTEASE+TPRs← FXSXX-COOH-like← Trypsin+EAD2← HTH_31</td><td>1321</td><td>Streptomyces sp. LamerLS-31b</td><td>GCA_900091975.1</td><td>actinobacteria</td></tr>
<tr><td>SCF93414.1</td><td>vWA+REase+SWACOS+TPRs+cNMP_cyclase</td><td>AB_hydrolase→ CASPASE+VMAP-M2+VMAP-C→ MoxR→ vWA+REase+SWACOS+TPRs+cNMP_cyclase*→</td><td>1650</td><td>Streptomyces sp. Cmuel-A718b</td><td>GCA_900092005.1</td><td>actinobacteria</td></tr>
<tr><td>SCF93674.1</td><td>vWA+Pkinase+cNMP_cyclase+Band7</td><td>vWA+Pkinase+cNMP_cyclase+Band7*→</td><td>1181</td><td>Streptomyces sp. DconLS</td><td>GCA_900091985.1</td><td>actinobacteria</td></tr>
<tr><td>SCF98831.1</td><td>vWA+REase+SFI-helicase+DUF4729</td><td>Trypsin+EAD2+VMAP-M0+VMAP-C→ MoxR→ vWA+REase+SFI-helicase+DUF4729*→</td><td>1616</td><td>Streptomyces sp. Ncost-T10-10d</td><td>GCA_900091955.1</td><td>actinobacteria</td></tr>
<tr><td>SCG16381.1</td><td>vWA+SWACOS+TPRs</td><td>Trypco1→ Trypsin+VMAP-M8+VMAP-C→ MoxR→ vWA+SWACOS+TPRs*→</td><td>1403</td><td>Micromonospora echinofusca</td><td>GCA_900091445.1</td><td>actinobacteria</td></tr>
<tr><td>SCG36816.1</td><td>vWA+Trypsin+HDxxH+VMAP-M0+VMAP-C</td><td>Trypco1→ Trypsin+HDxxH+VMAP-M0+VMAP-C→ MoxR→ vWA+Trypsin+HDxxH+VMAP-M0+VMAP-C*→ ?→ ||← ?← ?||?→ DUF397→</td><td>1064</td><td>Micromonospora humi</td><td>GCA_900090105.1</td><td>actinobacteria</td></tr>
<tr><td>SCG40329.1</td><td>vWA+APATPase+wHTH+TPRs</td><td>EAD2+VMAP-M0+VMAP-C→ MoxR→ vWA+APATPase+wHTH+TPRs*→ ||← METALLOPROTEASE+TPRs</td><td>1315</td><td>Micromonospora humi</td><td>GCA_900090105.1</td><td>actinobacteria</td></tr>
<tr><td>SCK15830.1</td><td>vWA+Pkinase+ParA-Soj-PloopNTPase(SIMIBI)</td><td>Trypco1→ Trypsin+EAD2+VMAP-M0+VMAP-C→ MoxR→ vWA+Pkinase+ParA-Soj-PloopNTPase(SIMIBI)*→</td><td>1138</td><td>Streptomyces sp. SceaMP-e96</td><td>GCA_900090095.1</td><td>actinobacteria</td></tr>
<tr><td>SCK31273.1</td><td>vWA+TPRs</td><td>← TCAD15||Trypco1→ Trypsin+HDxxH+VMAP-M0+VMAP-C→ MoxR→ vWA+TPRs*→ FXSXX-COOH→ Radical_SAM→ TIR+APATPase+TPRs→</td><td>1129</td><td>Streptomyces sp. SceaMP-e96</td><td>GCA_900090095.1</td><td>actinobacteria</td></tr>
<tr><td>SCK31299.1</td><td>vWA+TPRs</td><td>← TCAD15||Trypco1→ Trypsin+HDxxH+VMAP-M0+VMAP-C→ MoxR→ vWA+TPRs*→ FXSXX-COOH→ METALLOPROTEASE+TPRs→ TIR+APATPase+TPRs→</td><td>1018</td><td>Streptomyces sp. WMMB 322</td><td>GCA_900090145.1</td><td>actinobacteria</td></tr>
<tr><td>SCK33554.1</td><td>vWA+Pkinase+wHTH+TPRs+cNMP_cyclase</td><td>Trypco1→ Trypsin+VMAP-M0+VMAP-C→ MoxR→ vWA+Pkinase+wHTH+TPRs+cNMP_cyclase*→</td><td>1277</td><td>Streptomyces</td><td>GCA_900090165.1</td><td>actinobacteria</td></tr>
<tr><td>SCK52956.1</td><td>vWA+wHTH+TPRs+APATPase+wHTH+TPRs</td><td>Trypco1→ Trypsin+EAD2+VMAP-M0+VMAP-C→ MoxR→ vWA+wHTH+TPRs+APATPase+wHTH+TPRs*→ ?→ ?→ ||← AB_hydrolase</td><td>1607</td><td>Streptomyces sp. SceaMP-e96</td><td>GCA_900090095.1</td><td>actinobacteria</td></tr>
<tr><td>SCL25845.1</td><td>vWA+Calcineurin-like+NACHT</td><td>Trypco1→ Trypsin+HDxxH+VMAP-M0+VMAP-C→ MoxR→ vWA+Calcineurin-like+NACHT*→</td><td>1535</td><td>Micromonospora inyonensis</td><td>GCA_900091415.1</td><td>actinobacteria</td></tr>
<tr><td>SCL28499.1</td><td>vWA+APATPase+wHTH+TPRs</td><td>EAD2+VMAP-M0+VMAP-C→ MoxR→ vWA+APATPase+wHTH+TPRs*→ ||← METALLOPROTEASE+TPRs← FXSXX-COOH-like</td><td>1353</td><td>Micromonospora nigra</td><td>GCA_900091585.1</td><td>actinobacteria</td></tr>
<tr><td>SCL86372.1</td><td>vWA+APATPase+wHTH+TPRs</td><td>EAD2+VMAP-M0+VMAP-C→ MoxR→ vWA+APATPase+wHTH+TPRs*→ AB_hydrolase→</td><td>1445</td><td>Streptomyces sp. DpondAA-F4</td><td>GCA_900095575.1</td><td>actinobacteria</td></tr>
<tr><td>SCY50757.1</td><td>vWA+TPRs</td><td>← TCAD15||Trypco1→ Trypsin+HDxxH+VMAP-M0+VMAP-C→ MoxR→ vWA+TPRs*→ FXSXX-COOH→ Radical_SAM→ METALLOPROTEASE+TPRs→ TIR+APATPase+TPRs→</td><td>1157</td><td>Streptomyces sp. 136MFCol5.1</td><td>GCA_900101975.1</td><td>actinobacteria</td></tr>
<tr><td>SDA57601.1</td><td>vWA+Calcineurin-like+APATPase+SFII-helicase</td><td>AB_hydrolase→ CASPASE+VMAP-M4+VMAP-C→ MoxR→ vWA+Calcineurin-like+APATPase+SFII-helicase*→</td><td>1397</td><td>Janthinobacterium sp. 551a</td><td>GCA_900103675.1</td><td>betaproteobacteria</td></tr>
<tr><td>SDE27042.1</td><td>vWA+SFII-helicase+Trypsin+cNMP_cyclase</td><td>AB_hydrolase→ CASPASE+VMAP-M2+VMAP-C→ MoxR→ vWA+SFII-helicase+Trypsin+cNMP_cyclase*→</td><td>1461</td><td>Streptomyces emeiensis</td><td>GCA_900101585.1</td><td>actinobacteria</td></tr>
<tr><td>SDF68672.1</td><td>vWA+APATPase+wHTH+TPRs</td><td>cNMP_cyclase+VMAP-M0+VMAP-C→ MoxR→ vWA+APATPase+wHTH+TPRs*→ ||← METALLOPROTEASE+TPRs← FXSXX-COOH-like</td><td>1458</td><td>Lechevalieria fradiae</td><td>GCA_900100955.1</td><td>actinobacteria</td></tr>
<tr><td>SDG68818.1</td><td>vWA+STAND_NTPase+Trypsin+CASPASE+S1COLD</td><td>AB_hydrolase→ CASPASE+VMAP-M0+VMAP-C→ MoxR→ vWA+STAND_NTPase+Trypsin+CASPASE+S1COLD*→</td><td>1349</td><td>Streptomyces jietaisiensis</td><td>GCA_900102095.1</td><td>actinobacteria</td></tr>
<tr><td>SDK59304.1</td><td>vWA+Pkinase+wHTH+TPRs+cNMP_cyclase+Band7+HAD</td><td>Trypco1→ Trypsin+VMAP-M0+VMAP-C→ MoxR→ vWA+Pkinase+wHTH+TPRs+cNMP_cyclase+Band7+HAD*→</td><td>1689</td><td>Streptomyces indicus</td><td>GCA_900100315.1</td><td>actinobacteria</td></tr>
<tr><td>SDL96695.1</td><td>vWA+TPRs</td><td>← TCAD15||Trypco1→ Trypsin+HDxxH+VMAP-M0+VMAP-C→ MoxR→ vWA+TPRs*→ FXSXX-COOH→ Radical_SAM→ METALLOPROTEASE+TPRs→ TIR+APATPase+TPRs→</td><td>1111</td><td>Streptomyces wuyuanensis</td><td>GCA_900103455.1</td><td>actinobacteria</td></tr>
<tr><td>SDM15376.1</td><td>vWA+APATPase+wHTH+TPRs</td><td>← cNMP_binding+HTH_Crp_2||cNMP_cyclase+EAD2+VMAP-M0+VMAP-C→ MoxR→ vWA+APATPase+wHTH+TPRs*→ ||← METALLOPROTEASE+TPRs← FXSXX-COOH-like||?→ ?→ TIR+TPRs→</td><td>1411</td><td>Lentzea violacea</td><td>GCA_900100275.1</td><td>actinobacteria</td></tr>
<tr><td>SDM66838.1</td><td>vWA+APATPase+wHTH+TPRs</td><td>EAD2+VMAP-M0+VMAP-C→ vWA+APATPase+wHTH+TPRs*→ ||← METALLOPROTEASE+TPRs← FXSXX-COOH-like← Trypsin+EAD2← Trypco2||FXSXX-COOH-like→ METALLOPROTEASE+TPRs→</td><td>1318</td><td>Streptomyces sp. cf386</td><td>GCA_900103585.1</td><td>actinobacteria</td></tr>
<tr><td>SDN22428.1</td><td>vWA+APATPase+wHTH+TPRs</td><td>PNP_UDP_1+EAD2→ EAD2+VMAP-M0+VMAP-C→ MoxR→ vWA+APATPase+wHTH+TPRs*→ ||← METALLOPROTEASE+TPRs← FXSXX-COOH-like</td><td>1504</td><td>Streptomyces guanduensis</td><td>GCA_900103985.1</td><td>actinobacteria</td></tr>
<tr><td>SDN24910.1</td><td>vWA+Pkinase+FtsK+TPRs</td><td>ADP_ribosyl_GH→ Trypco1→ Trypsin+EAD2+VMAP-M0+VMAP-C→ MoxR→ vWA+Pkinase+FtsK+TPRs*→ NUDIX→ SIR2→</td><td>1325</td><td>Streptomyces wuyuanensis</td><td>GCA_900103455.1</td><td>actinobacteria</td></tr>
<tr><td>SDO92256.1</td><td>vWA+Pkinase+wHTH+TPRs+cNMP_cyclase+Band7+HAD</td><td>Trypsin+VMAP-M0+VMAP-C→ MoxR→ vWA+Pkinase+wHTH+TPRs+cNMP_cyclase+Band7+HAD*→</td><td>1558</td><td>Streptomyces sp. cf386</td><td>GCA_900103585.1</td><td>actinobacteria</td></tr>
<tr><td>SDR23156.1</td><td>vWA+TPRs</td><td>← TCAD15||Trypco1→ Trypsin+HDxxH+VMAP-M0+VMAP-C→ MoxR→ vWA+TPRs*→ FXSXX-COOH→ Radical_SAM→ TIR+APATPase+TPRs→</td><td>1134</td><td>Streptomyces sp. KS_16</td><td>GCA_900102245.1</td><td>actinobacteria</td></tr>
<tr><td>SDT07870.1</td><td>vWA+APATPase+SWACOS+wHTH+TPRs+Beta-rich-tail</td><td>Trypco1→ Trypsin+EAD2→ EAD2+VMAP-M10+VMAP-C→ MoxR→ vWA+APATPase+SWACOS+wHTH+TPRs+Beta-rich-tail*→</td><td>1562</td><td>Streptomyces sp. TLI_053</td><td>GCA_900105395.1</td><td>actinobacteria</td></tr>
<tr><td>SDT36692.1</td><td>vWA+Pkinase+wHTH+TPRs+cNMP_cyclase</td><td>Trypco1→ Trypsin+VMAP-M0+VMAP-C→ MoxR→ vWA+Pkinase+wHTH+TPRs+cNMP_cyclase*→</td><td>1352</td><td>Streptomyces sp. 2114.2</td><td>GCA_900104935.1</td><td>actinobacteria</td></tr>
<tr><td>SDT74622.1</td><td>vWA+TPRs</td><td>← TCAD15||Trypco1→ Trypsin+HDxxH+VMAP-M0+VMAP-C→ MoxR→ vWA+TPRs*→ FXSXX-COOH→ Radical_SAM→ METALLOPROTEASE+TPRs→ TIR+APATPase+TPRs→ Trypco1→</td><td>1144</td><td>Streptomyces sp. 2114.2</td><td>GCA_900104935.1</td><td>actinobacteria</td></tr>
<tr><td>SDU45427.1</td><td>vWA+APATPase+wHTH+TPRs</td><td>cNMP_cyclase+VMAP-M0+VMAP-C→ MoxR→ vWA+APATPase+wHTH+TPRs*→ ||← METALLOPROTEASE+TPRs← FXSXX-COOH-like← cNMP_binding+HTH_Crp_2</td><td>1449</td><td>Amycolatopsis keratiniphila</td><td>GCA_900105855.1</td><td>actinobacteria</td></tr>
<tr><td>SDX38190.1</td><td>vWA+FGS-fold_domain+FGS-fold_domain+FGS-fold_domain</td><td>AB_hydrolase→ CASPASE+VMAP-M4+VMAP-C→ MoxR→ vWA+FGS-fold_domain+FGS-fold_domain+FGS-fold_domain*→</td><td>1552</td><td>Thiocapsa roseopersicina</td><td>GCA_900106925.1</td><td>gammaproteobacteria</td></tr>
<tr><td>SDY41558.1</td><td>vWA+APATPase+wHTH+TPRs</td><td>← cNMP_binding+HTH_Crp_2||cNMP_cyclase+EAD2→ EAD2+VMAP-M0+VMAP-C→ MoxR→ vWA+APATPase+wHTH+TPRs*→ ||← METALLOPROTEASE+TPRs</td><td>1388</td><td>Amycolatopsis xylanica</td><td>GCA_900107045.1</td><td>actinobacteria</td></tr>
<tr><td>SDY89948.1</td><td>vWA+APATPase+wHTH+Pkinase</td><td>CASPASE+VMAP-M2+VMAP-C→ MoxR→ vWA+APATPase+wHTH+Pkinase*→</td><td>1024</td><td>Amycolatopsis xylanica</td><td>GCA_900107045.1</td><td>actinobacteria</td></tr>
<tr><td>SEB55912.1</td><td>vWA+TPRs</td><td>← TCAD15||Trypco1→ Trypsin+HDxxH+VMAP-M0+VMAP-C→ MoxR→ vWA+TPRs*→ FXSXX-COOH→ Radical_SAM→ TIR+APATPase+TPRs→</td><td>1134</td><td>Streptomyces sp. 2224.1</td><td>GCA_900105915.1</td><td>actinobacteria</td></tr>
<tr><td>SEB60365.1</td><td>vWA_protein</td><td>AB_hydrolase→ CASPASE+EAD2+VMAP-M0+VMAP-C→ MoxR→ vWA_protein*→ vWA+TIR+FxsC_Cter→</td><td>306</td><td>Streptomyces sp. PAN_FS17</td><td>GCA_900105465.1</td><td>actinobacteria</td></tr>
<tr><td>SEB64482.1</td><td>vWA+CASPASE+NACHT+PIN</td><td>Trypco1→ Trypsin+HDxxH+VMAP-M0+VMAP-C→ MoxR→ vWA+CASPASE+NACHT+PIN*→</td><td>1157</td><td>Streptomyces sp. 2131.1</td><td>GCA_900105515.1</td><td>actinobacteria</td></tr>
<tr><td>SEB86929.1</td><td>vWA+TPRs</td><td>AB_hydrolase→ CASPASE+EAD2+VMAP-M0+VMAP-C→ MoxR→ vWA+TPRs*→</td><td>1003</td><td>Streptomyces melanosporofaciens</td><td>GCA_900105695.1</td><td>actinobacteria</td></tr>
<tr><td>SEC39357.1</td><td>vWA+Pkinase+wHTH+TPRs+cNMP_cyclase+Band7+HAD</td><td>Trypsin+VMAP-M0+VMAP-C→ MoxR→ vWA+Pkinase+wHTH+TPRs+cNMP_cyclase+Band7+HAD*→</td><td>1578</td><td>Streptomyces sp. PAN_FS17</td><td>GCA_900105465.1</td><td>actinobacteria</td></tr>
<tr><td>SEC48111.1</td><td>vWA+APATPase+wHTH</td><td>HTH+EAD2+VMAP-M0+VMAP-C→ MoxR→ vWA+APATPase+wHTH*→</td><td>1170</td><td>Amycolatopsis tolypomycina</td><td>GCA_900105945.1</td><td>actinobacteria</td></tr>
<tr><td>SEC51035.1</td><td>vWA+TPRs</td><td>← TCAD15||Trypco1→ Trypsin+HDxxH+VMAP-M0+VMAP-C→ MoxR→ vWA+TPRs*→ FXSXX-COOH→ METALLOPROTEASE+TPRs→ TIR+APATPase+TPRs→</td><td>1044</td><td>Streptomyces sp. TLI_105</td><td>GCA_900105415.1</td><td>actinobacteria</td></tr>
<tr><td>SEC56283.1</td><td>vWA+TPRs</td><td>← TCAD15||Trypco1→ Trypsin+HDxxH+VMAP-M0+VMAP-C→ MoxR→ vWA+TPRs*→ FXSXX-COOH→ Radical_SAM→ METALLOPROTEASE+TPRs→ TIR+APATPase+TPRs→</td><td>1155</td><td>Streptomyces sp. 2131.1</td><td>GCA_900105515.1</td><td>actinobacteria</td></tr>
<tr><td>SEC79446.1</td><td>vWA+Pkinase+ZNR+Pkinase+ParA-Soj-PloopNTPase(SIMIBI)</td><td>SIR2→ ||← ?← ?||Trypco1→ Trypsin+EAD2+VMAP-M0+VMAP-C→ MoxR→ vWA+Pkinase+ZNR+Pkinase+ParA-Soj-PloopNTPase(SIMIBI)*→ Porphobil_deam+Porphobil_deamC→</td><td>1501</td><td>Streptomyces melanosporofaciens</td><td>GCA_900105695.1</td><td>actinobacteria</td></tr>
</table>

acc	architecture	operon	len	species	gca	taxend
SEC83562.1	vWA+APATPase+wHTH+TPRs	EAD2+VMAP-M0+VMAP-C→ MoxR→ vWA+APATPase+wHTH+TPRs*→ ||← Na_H_Exchanger||AB_hydrolase→ CASPASE+EAD2→ FXSXX-COOH-like→ METALLOPROTEASE+TPRs→	1512	Streptomyces sp. 1222.5	GCA_900105245.1	actinobacteria
SEC83886.1	vWA+wHTH+cNMP_cyclase+Band7+HAD	Trypco1→ Trypsin+VMAP-M0+VMAP-C→ MoxR→ vWA+wHTH+cNMP_cyclase+Band7+HAD*→	1271	Streptomyces sp. 1222.5	GCA_900105245.1	actinobacteria
SEC94800.1	vWA+wHTH+cNMP_cyclase+Band7+HAD	Trypco1→ Trypsin+VMAP-M0+VMAP-C→ MoxR→ vWA+wHTH+cNMP_cyclase+Band7+HAD*→	1268	Streptomyces sp. 2231.1	GCA_900105605.1	actinobacteria
SEC95242.1	vWA+APATPase+wHTH+TPRs	EAD2+VMAP-M0+VMAP-C→ MoxR→ vWA+APATPase+wHTH+TPRs*→ ||← Na_H_Exchanger||AB_hydrolase→ CASPASE+EAD2→ FXSXX-COOH-like→ METALLOPROTEASE+TPRs→	1512	Streptomyces sp. 2231.1	GCA_900105605.1	actinobacteria
SED06705.1	vWA+APATPase+wHTH+TPRs	PNP_UDP_1+EAD2→ MoxR→ vWA+APATPase+wHTH+TPRs*→ ||← METALLOPROTEASE+TPRs← FXSXX-COOH-like	1452	Streptomyces melanosporofaciens	GCA_900105695.1	actinobacteria
SED37029.1	vWA+TIR+FxsC_Cter	AB_hydrolase→ CASPASE+VMAP-M2+VMAP-C→ MoxR→ vWA+TIR+FxsC_Cter*→	1001	Streptomyces griseus	GCA_900105705.1	actinobacteria
SED44913.1	vWA+Pkinase+wHTH+TPRs+cNMP_cyclase+Band7+HAD	Trypsin+VMAP-M0+VMAP-C→ MoxR→ vWA+Pkinase+wHTH+TPRs+cNMP_cyclase+Band7+HAD*→	1578	Streptomyces sp. KS_5	GCA_900105275.1	actinobacteria
SED54965.1	vWA+APATPase+wHTH+TPRs	cNMP_cyclase+EAD2→ EAD2+VMAP-M0+VMAP-C→ MoxR→ vWA+APATPase+wHTH+TPRs*→ ||← METALLOPROTEASE+TPRs← FXSXX-COOH-like	1341	Amycolatopsis tolypomycina	GCA_900105945.1	actinobacteria
SED97882.1	vWA+APATPase+wHTH+TPRs	Trypco2→ EAD2+VMAP-M0+VMAP-C→ vWA+APATPase+wHTH+TPRs*→ ||← METALLOPROTEASE+TPRs← FXSXX-COOH-like← Trypsin+EAD2	1309	Streptomyces misionensis	GCA_900104815.1	actinobacteria
SEE32562.1	vWA+TPRs	TCAD15||Trypco1→ Trypsin+HDxxH+VMAP-M0+VMAP-C→ MoxR→ vWA+TPRs*→ FXSXX-COOH→ Radical_SAM→ TIR+APATPase+TPRs→	1134	Streptomyces sp. 2112.3	GCA_900105775.1	actinobacteria
SEE51089.1	vWA+TPRs	TCAD15||Trypco1→ Trypsin+HDxxH+VMAP-M0+VMAP-C→ MoxR→ vWA+TPRs*→ FXSXX-COOH→ Radical_SAM→ TIR+APATPase+TPRs→	1146	Streptomyces sp. 2314.4	GCA_900105525.1	actinobacteria
SEE56281.1	vWA+TPRs	Trypco1→ Trypsin+EAD2+VMAP-M0+VMAP-C→ MoxR→ vWA+TPRs*→	1201	Streptomyces sp. 2314.4	GCA_900105525.1	actinobacteria
SEE67995.1	vWA+APATPase+wHTH+TPRs	vWA+APATPase+wHTH+TPRs*→ ?→ ||← ?← ?← S1COLD	1167	Streptomyces sp. KS_5	GCA_900105275.1	actinobacteria
SEF23786.1	vWA+APATPase+wHTH+TPRs	EAD2+VMAP-M0+VMAP-C→ MoxR→ vWA+APATPase+wHTH+TPRs*→ ||← METALLOPROTEASE+TPRs← FXSXX-COOH-like	1401	Amycolatopsis pretoriensis	GCA_900107925.1	actinobacteria
SEF26728.1	vWA+APATPase+wHTH+TPRs	cNMP_cyclase+EAD2→ EAD2+VMAP-M0+VMAP-C→ vWA+APATPase+wHTH+TPRs→ ||← METALLOPROTEASE+TPRs← FXSXX-COOH-like||EAD2+VMAP-M0+VMAP-C→ MoxR→ vWA+APATPase+wHTH+TPRs*→ ||← METALLOPROTEASE+TPRs← FXSXX-COOH-like	1419	Amycolatopsis pretoriensis	GCA_900107925.1	actinobacteria
SEF26739.1	vWA+APATPase+wHTH+TPRs	cNMP_cyclase+EAD2→ EAD2+VMAP-M0+VMAP-C→ vWA+APATPase+wHTH+TPRs*→ ||← METALLOPROTEASE+TPRs← FXSXX-COOH-like||EAD2+VMAP-M0+VMAP-C→ MoxR→ vWA+APATPase+wHTH+TPRs→ ||← METALLOPROTEASE+TPRs← FXSXX-COOH-like	1320	Amycolatopsis pretoriensis	GCA_900107925.1	actinobacteria
SEF86779.1	vWA+TPRs	TCAD15||Trypsin+HDxxH+VMAP-M0+VMAP-C→ MoxR→ vWA+TPRs*→ FXSXX-COOH→ Radical_SAM→ METALLOPROTEASE+TPRs→ TIR+APATPase+TPRs→ Trypco1→	1150	Streptomyces yanglinensis	GCA_900107965.1	actinobacteria
SEF89170.1	vWA+APATPase+wHTH+TPRs	PNP_UDP_1+EAD2→ EAD2+VMAP-M0+VMAP-C→ MoxR→ vWA+APATPase+wHTH+TPRs*→ ||← METALLOPROTEASE+TPRs← FXSXX-COOH-like	1507	Streptomyces yanglinensis	GCA_900107965.1	actinobacteria
SEG16301.1	vWA+APATPase+wHTH+TPRs	Radical_SAM→ METALLOPROTEASE+TPRs→ ||← TIR+APATPase+TPRs||FXSXX-COOH-like→ ?→ EAD2+VMAP-M0+VMAP-C→ MoxR→ vWA+APATPase+wHTH+TPRs*→ || METALLOPROTEASE+TPRs← Radical_SAM← FXSXX-COOH	1381	Actinomadura echinospora	GCA_900108175.1	actinobacteria
SEG54017.1	vWA+BetaPropeller+SWACOS+STAND_NTPase	Trypco1→ Trypsin+VMAP-M8+VMAP-C→ MoxR→ vWA+BetaPropeller+SWACOS+STAND_NTPase*→	1835	Actinomadura echinospora	GCA_900108175.1	actinobacteria
SEG88246.1	vWA+APATPase+wHTH+TPRs	Trypsin+EAD2→ EAD2+VMAP-M0+VMAP-C→ MoxR→ vWA+APATPase+wHTH+TPRs*→ ||← METALLOPROTEASE+TPRs← FXSXX-COOH-like	1411	Nonomuraea solani	GCA_900108335.1	actinobacteria
SEG93346.1	vWA+Pkinase+ParA-Soj-PloopNTPase(SIMIBI)	Trypco1→ Trypsin+EAD2+VMAP-M0+VMAP-C→ MoxR→ vWA+Pkinase+ParA-Soj-PloopNTPase(SIMIBI)*→	1119	Streptomyces yanglinensis	GCA_900107965.1	actinobacteria
SEN16512.1	vWA+APATPase+wHTH+TPRs	EAD2+VMAP-M0+VMAP-C→ vWA+APATPase+wHTH+TPRs*→ ||← METALLOPROTEASE+TPRs← FXSXX-COOH-like← Trypsin+EAD2← Trypco2	1316	Streptomyces rubidus	GCA_900110255.1	actinobacteria
SEQ80125.1	vWA+APATPase+wHTH+TPRs	cNMP_binding+HTH_Crp_2||cNMP_cyclase+VMAP-M0+VMAP-C→ MoxR→ vWA+APATPase+wHTH+TPRs*→ ||← METALLOPROTEASE+TPRs← FXSXX-COOH-like	1446	Lechevalieria xinjiangensis	GCA_900110955.1	actinobacteria
SER48031.1	vWA+APATPase+wHTH+TPRs	cNMP_binding+HTH_Crp_2||cNMP_cyclase+VMAP-M0+VMAP-C→ MoxR→ vWA+APATPase+wHTH+TPRs*→ ||← METALLOPROTEASE+TPRs← FXSXX-COOH-like	1445	Lentzea albida	GCA_900111005.1	actinobacteria
SER87460.1	vWA+wHTH+cNMP_cyclase+Band7+HAD	Trypco1→ Trypsin+VMAP-M0+VMAP-C→ MoxR→ vWA+wHTH+cNMP_cyclase+Band7+HAD*→	1328	Streptomyces sp. yr375	GCA_900110755.1	actinobacteria
SES32171.1	vWA+Trypsin+TCAD11+TPRs	CASPASE+VMAP-M2+VMAP-C→ MoxR→ vWA+Trypsin+TCAD11+TPRs*→	1417	Actinokineospora terrae	GCA_900111175.1	actinobacteria
SES36659.1	vWA+TPRs	TCAD15||Trypco1→ Trypsin+HDxxH+VMAP-M0+VMAP-C→ MoxR→ vWA+TPRs*→ FXSXX-COOH→ Radical_SAM→	1146	Streptomyces sp. yr375	GCA_900110755.1	actinobacteria
SES49412.1	vWA+APATPase+wHTH+TPRs	cNMP_binding+HTH_Crp_2||cNMP_cyclase+VMAP-M0+VMAP-C→ MoxR→ vWA+APATPase+wHTH+TPRs*→ ||← METALLOPROTEASE+TPRs← FXSXX-COOH-like	1443	Lentzea flaviverrucosa	GCA_900110985.1	actinobacteria
SFE91364.1	vWA+APATPase+wHTH+TPRs	EAD2+VMAP-M0+VMAP-C→ vWA+APATPase+wHTH+TPRs*→ ||← METALLOPROTEASE+TPRs← FXSXX-COOH-like	1265	Actinoplanes philippinensis	GCA_900113015.1	actinobacteria
SFF36468.1	vWA+TPRs	TCAD15||Trypsin+HDxxH+VMAP-M0+VMAP-C→ MoxR→ vWA+TPRs*→ FXSXX-COOH→ Radical_SAM→ METALLOPROTEASE+TPRs→ TIR+APATPase+TPRs→	1157	Streptomyces alni	GCA_900112845.1	actinobacteria
SFF65888.1	vWA+SFII-helicase+Z1	AB_hydrolase→ CASPASE+VMAP-M2+VMAP-C→ VMAP-C(partial)→ MoxR→ vWA+SFII-helicase+Z1*→	1391	Streptomyces mirabilis	GCA_900112965.1	actinobacteria
SFM91649.1	vWA+METALLOPROTEASE+TCAD8	AB_hydrolase→ CASPASE+VMAP-M4+VMAP-C→ MoxR→ vWA+METALLOPROTEASE+TCAD8*→	1077	Rugamonas rubra	GCA_900114705.1	gammaproteobacteria
SFN86739.1	vWA+APATPase+wHTH+TPRs	Radical_SAM→ ||← TIR+APATPase+TPRs||?→ SWACOS→ ?→ EAD2+VMAP-M0+VMAP-C→ MoxR→ vWA+APATPase+wHTH+TPRs*→ ||← Radical_SAM← ?||cNMP_cyclase+EAD2→	1390	Actinomadura madurae	GCA_900115095.1	actinobacteria
SFO06617.1	vWA+APATPase+wHTH+TPRs	Trypco1→ Trypsin+EAD2+VMAP-M0+VMAP-C→ MoxR→ vWA+APATPase+GGDEF+EAD2+VMAP-M0→ EAD2+VMAP-M0+VMAP-C→ MoxR→ vWA+APATPase+wHTH+TPRs*→	1414	Streptomyces sp. cf124	GCA_900114955.1	actinobacteria
SFO06655.1	vWA+APATPase+GGDEF+EAD2+VMAP-M0	Trypco1→ Trypsin+EAD2+VMAP-M0+VMAP-C→ MoxR→ vWA+APATPase+GGDEF+EAD2+VMAP-M0*→ EAD2+VMAP-M0+VMAP-C→ MoxR→ vWA+APATPase+wHTH+TPRs→	1163	Streptomyces sp. cf124	GCA_900114955.1	actinobacteria
SFQ99524.1	vWA+APATPase+wHTH+TPRs	cNMP_binding+HTH_Crp_2||cNMP_cyclase+VMAP-M0+VMAP-C→ MoxR→ vWA+APATPase+wHTH+TPRs*→ ||← METALLOPROTEASE+TPRs← FXSXX-COOH-like	1381	Lentzea waywayandensis	GCA_900115955.1	actinobacteria
SFR29853.1	vWA+CASPASE+NACHT+TPRs	AB_hydrolase→ BTLCP+VMAP-M2+VMAP-C→ MoxR→ vWA+CASPASE+NACHT+TPRs*→	1828	Lentzea waywayandensis	GCA_900115955.1	actinobacteria
SFS61774.1	vWA+TPRs	TCAD15||Trypco1→ Trypsin+HDxxH+VMAP-M0+VMAP-C→ MoxR→ vWA+TPRs*→ FXSXX-COOH→ Radical_SAM→ METALLOPROTEASE+TPRs→ TIR+APATPase+TPRs→	1157	Streptomyces sp. ok210	GCA_900116325.1	actinobacteria
SFS77547.1	vWA_protein	EAD2+VMAP-M0+VMAP-C→ MoxR→ vWA_protein*→ TIR+APATPase+TPRs→ ||← METALLOPROTEASE+TPRs← FXSXX-COOH-like← TIR+EAD2	391	Saccharopolyspora flava	GCA_900116135.1	actinobacteria
SFS99759.1	vWA+TPRs	TCAD15||Trypco1→ Trypsin+HDxxH+VMAP-M0+VMAP-C→ MoxR→ vWA+TPRs*→ FXSXX-COOH→ Radical_SAM→ METALLOPROTEASE+TPRs→ TIR+APATPase+TPRs→	1110	Streptomyces harbinensis	GCA_900116145.1	actinobacteria
SFV19391.1	vWA+CASPASE+TPRs	AB_hydrolase→ CASPASE+VMAP-M4+VMAP-C→ MoxR→ vWA+CASPASE+TPRs*→	1254	Bradyrhizobium arachidis	GCA_900116675.1	alphaproteobacteria
SFW79240.1	vWA+APATPase+wHTH+TPRs	EAD2+VMAP-M0+VMAP-C→ MoxR→ vWA+APATPase+wHTH+TPRs*→ ||← METALLOPROTEASE+TPRs← FXSXX-COOH-like	1400	Amycolatopsis australiensis	GCA_900119165.1	actinobacteria
SFX27404.1	vWA+TPRs	Trypco1→ Trypsin+HDxxH+VMAP-M0+VMAP-C→ MoxR→ vWA+TPRs*→ FXSXX-COOH→ Radical_SAM→ METALLOPROTEASE+TPRs→ TIR+APATPase+TPRs→ Trypco1→	1133	Streptomyces atratus	GCA_900119365.1	actinobacteria
SFY49287.1	vWA+APATPase+wHTH+TPRs	Trypco2→ tRNA→ vWA+APATPase+wHTH+TPRs*→ ||← METALLOPROTEASE+TPRs← FXSXX-COOH-like← Trypsin+EAD2	1309	Streptomyces sp. F-1	GCA_900079405.3	actinobacteria
SHH97075.1	vWA+TPRs	TCAD15||Trypco1→ Trypsin+HDxxH+VMAP-M0+VMAP-C→ MoxR→ vWA+TPRs*→ FXSXX-COOH→ Radical_SAM→ METALLOPROTEASE+TPRs→ TIR+APATPase+TPRs→	1124	Streptomyces sp. 3124.6	GCA_900129855.1	actinobacteria
SHI04221.1	vWA+Pkinase+ParA-Soj-PloopNTPase(SIMIBI)	Trypco1→ Trypsin+EAD2+VMAP-M0+VMAP-C→ MoxR→ vWA+Pkinase+ParA-Soj-PloopNTPase(SIMIBI)*→	1130	Streptomyces sp. 3124.6	GCA_900129855.1	actinobacteria
SHI52217.1	vWA+SFII-helicase+Z1	AB_hydrolase→ CASPASE+VMAP-M2+VMAP-C→ MoxR→ vWA+SFII-helicase+Z1*→	1348	Streptomyces sp. 3124.6	GCA_900129855.1	actinobacteria

<table>
<tr><th>acc</th><th>architecture</th><th>operon</th><th>len</th><th>species</th><th>gca</th><th>taxend</th></tr>
<tr><td>SHK77186.1</td><td>vWA+TPRs</td><td>TCAD15||Trypsin+HDxxH+VMAP-M0+VMAP-C→ MoxR→ vWA+TPRs*→ FXSXX-COOH→ Radical_SAM→ METALLOPROTEASE+TPRs→ TIR+APATPase+TPRs→</td><td>1112</td><td>Streptomyces paucisporeus</td><td>GCA_900142575.1</td><td>actinobacteria</td></tr>
<tr><td>SMD27174.1</td><td>vWA+APATPase+wHTH+TPRs</td><td>cNMP_cyclase+VMAP-M0+VMAP-C→ MoxR→ vWA+APATPase+wHTH+TPRs*→ ||← METALLOPROTEASE+TPRs</td><td>1452</td><td>Kibdelosporangium aridum</td><td>GCA_900176515.1</td><td>actinobacteria</td></tr>
<tr><td>SME88395.1</td><td>vWA</td><td>TCAD9+Pkinase+NACHT+HEAT_repeats→ EAD2+VMAP-M0+VMAP-C→ vWA*→ MoxR→ vWA+APATPase+TPRs→</td><td>459</td><td>Streptomyces sp. Amel2xC10</td><td>GCA_900177255.1</td><td>actinobacteria</td></tr>
<tr><td>SOF02547.1</td><td>vWA+cNMP_binding+HTH_Crp_2+TerD</td><td>AB_hydrolase→ CASPASE+VMAP-M0+VMAP-C→ MoxR→ vWA+cNMP_binding+HTH_Crp_2+TerD*→</td><td>1494</td><td>Streptomyces sp. OV198</td><td>GCA_900230275.1</td><td>actinobacteria</td></tr>
<tr><td>WP_002735856.1</td><td>vWA+FGS-fold_domain</td><td>Trypco1→ Trypsin+VMAP-M1+VMAP-C→ MoxR→ vWA+FGS-fold_domain*→</td><td>884</td><td>Microcystis aeruginosa</td><td>GCA_000330925.1</td><td>cyanobacteria</td></tr>
<tr><td>WP_002740175.1</td><td>vWA+FGS-fold_domain</td><td>HTH+VMAP-M9+VMAP-C→ MoxR→ vWA+FGS-fold_domain*→ ||← ?||?→ ?→ PSE→ FGS-fold_domain→</td><td>962</td><td>Microcystis aeruginosa</td><td>GCA_000330925.1</td><td>cyanobacteria</td></tr>
<tr><td>WP_002743980.1</td><td>vWA+FGS-fold_domain</td><td>HTH+VMAP-M9+VMAP-C→ MoxR→ vWA+FGS-fold_domain*→</td><td>955</td><td>Microcystis aeruginosa</td><td>GCA_000332585.1</td><td>cyanobacteria</td></tr>
<tr><td>WP_002750294.1</td><td>vWA+FGS-fold_domain</td><td>Trypco1→ Trypsin+VMAP-M1+VMAP-C→ MoxR→ vWA+FGS-fold_domain*→ ?→ PSE→ ||← PSE||PSE→ FGS-fold_domain→</td><td>928</td><td>Microcystis aeruginosa</td><td>GCA_000307995.2</td><td>cyanobacteria</td></tr>
<tr><td>WP_002752611.1</td><td>vWA+FGS-fold_domain</td><td>AB_hydrolase→ CASPASE+VMAP-M14+VMAP-C→ MoxR→ vWA+FGS-fold_domain*→</td><td>842</td><td>Microcystis aeruginosa</td><td>GCA_000307995.2</td><td>cyanobacteria</td></tr>
<tr><td>WP_002755866.1</td><td>vWA+FGS-fold_domain</td><td>HTH+VMAP-M9+VMAP-C→ MoxR→ vWA+FGS-fold_domain*→ ?→ FGS-fold_domain→</td><td>962</td><td>Microcystis aeruginosa</td><td>GCA_000307995.2</td><td>cyanobacteria</td></tr>
<tr><td>WP_002756922.1</td><td>vWA+FGS-fold_domain</td><td>AB_hydrolase→ CASPASE+EAD2→ EAD2+VMAP-M11+VMAP-C→ MoxR→ vWA+FGS-fold_domain*→</td><td>705</td><td>Microcystis aeruginosa</td><td>GCA_000312165.1</td><td>cyanobacteria</td></tr>
<tr><td>WP_002757190.1</td><td>vWA+FGS-fold_domain</td><td>HTH+VMAP-M9+VMAP-C→ MoxR→ vWA+FGS-fold_domain*→ ?→ ?→ ?→ PSE→ FGS-fold_domain→</td><td>962</td><td>Microcystis aeruginosa</td><td>GCA_000312165.1</td><td>cyanobacteria</td></tr>
<tr><td>WP_002762808.1</td><td>vWA+FGS-fold_domain</td><td>AB_hydrolase→ CASPASE+VMAP-M14+VMAP-C→ MoxR→ vWA+FGS-fold_domain*→</td><td>842</td><td>Microcystis aeruginosa</td><td>GCA_000312165.1</td><td>cyanobacteria</td></tr>
<tr><td>WP_002771100.1</td><td>vWA+FGS-fold_domain</td><td>CASPASE+VMAP-M14+VMAP-C→ MoxR→ vWA+FGS-fold_domain*→</td><td>841</td><td>Microcystis aeruginosa</td><td>GCA_000312185.1</td><td>cyanobacteria</td></tr>
<tr><td>WP_002775631.1</td><td>vWA+FGS-fold_domain</td><td>AB_hydrolase→ CASPASE+VMAP-M14+VMAP-C→ MoxR→ vWA+FGS-fold_domain*→</td><td>842</td><td>Microcystis aeruginosa</td><td>GCA_000312205.1</td><td>cyanobacteria</td></tr>
<tr><td>WP_002782653.1</td><td>vWA+FGS-fold_domain</td><td>AB_hydrolase→ CASPASE+VMAP-M14+VMAP-C→ MoxR→ vWA+FGS-fold_domain*→</td><td>843</td><td>Microcystis aeruginosa</td><td>GCA_000312725.1</td><td>cyanobacteria</td></tr>
<tr><td>WP_002783570.1</td><td>vWA+FGS-fold_domain</td><td>vWA+FGS-fold_domain*→</td><td>838</td><td>Microcystis aeruginosa</td><td>GCA_000312725.1</td><td>cyanobacteria</td></tr>
<tr><td>WP_002786766.1</td><td>vWA+FGS-fold_domain</td><td>HTH+VMAP-M9+VMAP-C→ MoxR→ vWA+FGS-fold_domain*→</td><td>964</td><td>Microcystis aeruginosa</td><td>GCA_000312225.1</td><td>cyanobacteria</td></tr>
<tr><td>WP_002787893.1</td><td>vWA+FGS-fold_domain</td><td>AB_hydrolase→ CASPASE+VMAP-M14+VMAP-C→ MoxR→ vWA+FGS-fold_domain*→</td><td>848</td><td>Microcystis aeruginosa</td><td>GCA_000312225.1</td><td>cyanobacteria</td></tr>
<tr><td>WP_002788676.1</td><td>vWA+FGS-fold_domain</td><td>Trypsin+VMAP-M1+VMAP-C→ MoxR→ vWA+FGS-fold_domain*→ FGS-fold_domain→</td><td>883</td><td>Microcystis aeruginosa</td><td>GCA_000312225.1</td><td>cyanobacteria</td></tr>
<tr><td>WP_002791729.1</td><td>vWA+FGS-fold_domain</td><td>Trypsin+VMAP-M1+VMAP-C→ MoxR→ vWA+FGS-fold_domain*→</td><td>885</td><td>Microcystis aeruginosa</td><td>GCA_000312245.1</td><td>cyanobacteria</td></tr>
<tr><td>WP_002792348.1</td><td>vWA+FGS-fold_domain</td><td>AB_hydrolase→ PSE→ MoxR→ vWA+FGS-fold_domain*→</td><td>842</td><td>Microcystis aeruginosa</td><td>GCA_000312245.1</td><td>cyanobacteria</td></tr>
<tr><td>WP_002795442.1</td><td>vWA+FGS-fold_domain</td><td>CASPASE+EAD2→ EAD2+VMAP-M11+VMAP-C→ MoxR→ vWA+FGS-fold_domain*→</td><td>916</td><td>Microcystis aeruginosa</td><td>GCA_000312265.1</td><td>cyanobacteria</td></tr>
<tr><td>WP_003103440.1</td><td>vWA+APATPase+wHTH+TPRs</td><td>cNMP_cyclase+EAD2→ EAD2+VMAP-M0+VMAP-C→ MoxR→ vWA+APATPase+wHTH+TPRs*→ ||← METALLOPROTEASE+TPRs</td><td>1475</td><td>Amycolatopsis vancoresmycina</td><td>GCA_000388135.1</td><td>actinobacteria</td></tr>
<tr><td>WP_003961788.1</td><td>vWA+TPRs</td><td>TCAD15||Trypco1→ Trypsin+HDxxH+VMAP-M0+VMAP-C→ MoxR→ ?→ vWA+TPRs*→</td><td>1091</td><td>Streptomyces clavuligerus</td><td>GCA_000163875.1</td><td>actinobacteria</td></tr>
<tr><td>WP_003963216.1</td><td>vWA+wHTH+TPRs+wHTH+TPRs+APATPase+wHTH+TPRs</td><td>Trypco1→ Trypsin+EAD2+VMAP-M0+VMAP-C→ MoxR→ vWA+wHTH+TPRs+wHTH+TPRs+APATPase+wHTH+TPRs*→</td><td>1828</td><td>Streptomyces clavuligerus</td><td>GCA_000163875.1</td><td>actinobacteria</td></tr>
<tr><td>WP_003969340.1</td><td>vWA+TPRs+TIR+FxsC_Cter(partial)</td><td>AB_hydrolase→ CASPASE+VMAP-M2+VMAP-C→ MoxR→ vWA+TPRs+TIR+FxsC_Cter(partial)*→</td><td>1499</td><td>Streptomyces sp. ACT-1</td><td>GCA_000177175.2</td><td>actinobacteria</td></tr>
<tr><td>WP_003973698.1</td><td>vWA+TPRs</td><td>TCAD15||Trypco1→ Trypsin+HDxxH+VMAP-M0+VMAP-C→ MoxR→ vWA+TPRs*→ FXSXX-COOH→ Radical_SAM→ METALLOPROTEASE+TPRs→ TIR+APATPase+TPRs→ Trypco1→</td><td>1096</td><td>Streptomyces</td><td>GCA_000158935.1</td><td>actinobacteria</td></tr>
<tr><td>WP_003987317.1</td><td>vWA+wHTH+TPRs+wHTH+TPRs+APATPase+wHTH+TPRs</td><td>vWA+wHTH+TPRs+wHTH+TPRs+APATPase+wHTH+TPRs*→</td><td>1342</td><td>Streptomyces rimosus</td><td>GCA_000331185.1</td><td>actinobacteria</td></tr>
<tr><td>WP_004001495.1</td><td>vWA+Pkinase+wHTH+TPRs+cNMP_cyclase+Band7+HAD</td><td>vWA+Pkinase+wHTH+TPRs+cNMP_cyclase+Band7+HAD*→</td><td>1415</td><td>Streptomyces viridochromogenes</td><td>GCA_000332625.1</td><td>actinobacteria</td></tr>
<tr><td>WP_004003790.1</td><td>vWA+APATPase+wHTH+TPRs</td><td>Trypsin+EAD2→ EAD2+VMAP-M0+VMAP-C→ MoxR→ vWA+APATPase+wHTH+TPRs*→ ||← METALLOPROTEASE+TPRs← FXSXX-COOH-like</td><td>1449</td><td>Streptomyces viridochromogenes</td><td>GCA_000332625.1</td><td>actinobacteria</td></tr>
<tr><td>WP_004162655.1</td><td>vWA+FGS-fold_domain</td><td>Trypco1→ Trypsin+VMAP-M1+VMAP-C→ MoxR→ vWA+FGS-fold_domain*→</td><td>871</td><td>Microcystis aeruginosa</td><td>GCA_000312265.1</td><td>cyanobacteria</td></tr>
<tr><td>WP_004162811.1</td><td>vWA+FGS-fold_domain</td><td>HTH+VMAP-M9+VMAP-C→ MoxR→ vWA+FGS-fold_domain*→ ?→ ?→ FGS-fold_domain→</td><td>962</td><td>Microcystis aeruginosa</td><td>GCA_000312265.1</td><td>cyanobacteria</td></tr>
<tr><td>WP_004268279.1</td><td>vWA+FGS-fold_domain</td><td>HTH+VMAP-M9+VMAP-C→ MoxR→ vWA+FGS-fold_domain*→ PSE→ FGS-fold_domain→</td><td>962</td><td>Microcystis aeruginosa</td><td>GCA_000312285.1</td><td>cyanobacteria</td></tr>
<tr><td>WP_004269016.1</td><td>vWA+FGS-fold_domain</td><td>AB_hydrolase→ CASPASE+VMAP-M14+VMAP-C→ MoxR→ vWA+FGS-fold_domain*→</td><td>842</td><td>Microcystis aeruginosa</td><td>GCA_000312285.1</td><td>cyanobacteria</td></tr>
<tr><td>WP_005157860.1</td><td>vWA+APATPase+wHTH+TPRs</td><td>cNMP_cyclase+VMAP-M0+VMAP-C→ MoxR→ vWA+APATPase+wHTH+TPRs*→ ||← METALLOPROTEASE+TPRs← FXSXX-COOH-like← cNMP_binding+HTH_Crp_2</td><td>1466</td><td>Amycolatopsis azurea</td><td>GCA_000340415.1</td><td>actinobacteria</td></tr>
<tr><td>WP_005308092.1</td><td>vWA+Trypsin+cNMP_cyclase+CCCH(TrebleClef)+Pentapeptide_repeats</td><td>VMAP-C→ MoxR→ vWA+Trypsin+cNMP_cyclase+CCCH(TrebleClef)+Pentapeptide_repeats*→</td><td>1306</td><td>Streptomyces pristinaespiralis</td><td>GCA_001278075.1</td><td>actinobacteria</td></tr>
<tr><td>WP_006100461.1</td><td>vWA+FGS-fold_domain</td><td>EAD9+EAD1+VMAP-C→ ?→ MoxR→ ||← ?||?→ ?→ vWA+FGS-fold_domain*→ FGS-fold_domain→ ||← ?← ?||FGS-fold_domain→ FGS-fold_domain→ ?→ FGS-fold_domain→</td><td>894</td><td>Coleofasciculus chthonoplastes</td><td>GCA_000155555.1</td><td>cyanobacteria</td></tr>
<tr><td>WP_006346673.1</td><td>vWA+TPRs</td><td>vWA+TPRs*→</td><td>959</td><td>Streptomyces tsukubensis</td><td>GCA_000297155.2</td><td>actinobacteria</td></tr>
<tr><td>WP_006514987.1</td><td>vWA+Cas_csx3</td><td>FGS-fold_domain→ ||← ?||PNP_UDP_1+VMAP-M1+VMAP-C→ MoxR→ vWA+Cas_csx3*→</td><td>729</td><td>Leptolyngbya sp. PCC 7375</td><td>GCA_000316115.1</td><td>cyanobacteria</td></tr>
<tr><td>WP_006541138.1</td><td>vWA+APATPase+wHTH+TPRs</td><td>EAD2+EAD1→ EAD2+VMAP-M0+VMAP-C→ MoxR→ vWA+APATPase+wHTH+TPRs*→ ||← METALLOPROTEASE+TPRs</td><td>1577</td><td>Frankia sp. EUN1f</td><td>GCA_000177675.1</td><td>actinobacteria</td></tr>
<tr><td>WP_006541355.1</td><td>vWA+APATPase+wHTH+TPRs</td><td>vWA+APATPase+wHTH+TPRs*→ ||← METALLOPROTEASE+TPRs← FXSXX-COOH-like</td><td>1270</td><td>Frankia sp. EUN1f</td><td>GCA_000177675.1</td><td>actinobacteria</td></tr>
<tr><td>WP_006602968.1</td><td>vWA+TPRs</td><td>Trypco1→ Trypsin+EAD2+VMAP-M0+VMAP-C→ ?→ PSE→ vWA+TPRs*→ ||← AB_hydrolase</td><td>1207</td><td>Streptomyces auratus</td><td>GCA_000280865.1</td><td>actinobacteria</td></tr>
<tr><td>WP_006603150.1</td><td>vWA+TPRs</td><td>TCAD15||Trypco1→ Trypsin+HDxxH+VMAP-M0+VMAP-C→ MoxR→ vWA+TPRs*→ FXSXX-COOH→ Radical_SAM→ TIR+APATPase+TPRs→</td><td>1147</td><td>Streptomyces auratus</td><td>GCA_000280865.1</td><td>actinobacteria</td></tr>
<tr><td>WP_006626227.1</td><td>vWA+TPRs</td><td>VMAP-C_protein→ MoxR→ vWA+TPRs*→</td><td>770</td><td>Arthrospira platensis</td><td>GCA_000307915.1</td><td>cyanobacteria</td></tr>
<tr><td>WP_006670063.1</td><td>vWA+TPRs+CASPASE</td><td>VMAP-C_protein→ MoxR→ vWA+TPRs+CASPASE*→</td><td>1436</td><td>Arthrospira maxima</td><td>GCA_000173555.1</td><td>cyanobacteria</td></tr>
<tr><td>WP_007445178.1</td><td>vWA+APATPase+wHTH+TPRs</td><td>EAD2+VMAP-M0+VMAP-C→ MoxR→ vWA+APATPase+wHTH+TPRs*→ ||← Na_H_Exchanger</td><td>1547</td><td>Streptomyces coelicoflavus</td><td>GCA_000241835.2</td><td>actinobacteria</td></tr>
<tr><td>WP_007447796.1</td><td>vWA+TPRs</td><td>TCAD15||Trypco1→ Trypsin+HDxxH+VMAP-M0+VMAP-C→ MoxR→ vWA+TPRs*→ Radical_SAM→ TIR+APATPase+TPRs→</td><td>1002</td><td>Streptomyces sp. W007</td><td>GCA_000239075.2</td><td>actinobacteria</td></tr>
</table>

acc	architecture	operon	len	species	gca	taxend
WP_007516491.1	vWA+APATPase+wHTH+TPRs	EAD2+VMAP-M0+VMAP-C→ MoxR→ vWA+APATPase+wHTH+TPRs→ ||← METALLOPROTEASE+TPRs||EAD2+VMAP-M0+VMAP-C→ MoxR→ vWA+APATPase+wHTH+TPRs*→ ||← METALLOPROTEASE+TPRs	1518	Frankia sp. CN3	GCF_000235425.2	actinobacteria
WP_007822168.1	vWA+TPRs	TCAD15||Trypsin+HDxxH+VMAP-M0+VMAP-C→ MoxR→ vWA+TPRs*→ FXSXX-COOH→ Radical_SAM→ METALLOPROTEASE+TPRs→ TIR+APATPase+TPRs→ Trypco1→	1303	Streptomyces sp. Tu6071	GCA_000213055.1	actinobacteria
WP_008050073.1	vWA+TPRs+CASPASE	VMAP-C_protein→ MoxR→ vWA+TPRs+CASPASE*→	2054	Arthrospira sp. PCC 8005	GCA_000176895.2	cyanobacteria
WP_008185551.1	vWA+FGS-fold_domain	AB_hydrolase→ CASPASE+VMAP-M14+VMAP-C→ MoxR→ vWA+FGS-fold_domain*→ FGS-fold_domain→ four_helix_bundle_protein→ FGS-fold_domain→ four_helix_bundle_protein→	873	Moorea producens	GCA_000211815.1	cyanobacteria
WP_008196903.1	vWA+FGS-fold_domain	AB_hydrolase→ CASPASE+VMAP-M14+VMAP-C→ MoxR→ vWA+FGS-fold_domain*→	841	Microcystis sp. T1-4	GCA_000297435.1	cyanobacteria
WP_008307738.1	vWA+FGS-fold_domain	FGS-fold_domain→ ||← ?||?→ ?→ PNP_UDP_1+VMAP-M1+VMAP-C→ MoxR→ vWA+FGS-fold_domain*→ ||← PSE||?→ ||← ?||?→ FGS-fold_domain→ FGS-fold_domain→	984	Leptolyngbya sp. PCC 6406	GCF_000332095.2	cyanobacteria
WP_009149137.1	vWA+TPRs+CASPASE	EAD1+Trypsin→ EAD1+VMAP-M1+Calcineurin-like→ EAD1+VMAP-M1+VMAP-C→ MoxR→ vWA+TPRs+CASPASE*→	1817	Moorea producens	GCA_000211815.1	cyanobacteria
WP_009714190.1	vWA+Pkinase+Pkinase+ParA-Soj-PloopNTPase(SIMIBI)	SIR2→ ||← ?← ?← ?← PSE||Trypco1→ PSE→ MoxR→ vWA+Pkinase+Pkinase+ParA-Soj-PloopNTPase(SIMIBI)*→ Porphobil_deam+Porphobil_deamC→	1213	Streptomyces himastatinicus	GCA_000158915.1	actinobacteria
WP_009784648.1	vWA+CASPASE	vWA+CASPASE*→	1193	Lyngbya sp. PCC 8106	GCA_000169095.1	cyanobacteria
WP_009997502.1	vWA+TPRs	TCAD15||Trypco1→ Trypsin+HDxxH+VMAP-M0+VMAP-C→ MoxR→ vWA+TPRs*→	1155	Streptomyces clavuligerus	-	actinobacteria
WP_010033823.1	vWA+APATPase+wHTH+TPRs	EAD2+VMAP-M0+VMAP-C→ MoxR→ vWA+APATPase+wHTH+TPRs*→ ||← Na_H_Exchanger||AB_hydrolase→ CASPASE+EAD2→ FXSXX-COOH-like→ METALLOPROTEASE+TPRs→	1517	Streptomyces chartreusis	GCA_900236475.1	actinobacteria
WP_010042268.1	vWA+cNMP_cyclase+Band7+HAD	Trypco1→ Trypsin+VMAP-M0+VMAP-C→ MoxR→ vWA+cNMP_cyclase+Band7+HAD*→	1083	Streptomyces chartreusis	GCA_900236475.1	actinobacteria
WP_010271951.1	vWA+TPRs	TCAD15||Trypsin+HDxxH+VMAP-M0+VMAP-C→ MoxR→ vWA+TPRs*→ FXSXX-COOH→ Radical_SAM→ METALLOPROTEASE+TPRs→ TIR+APATPase+TPRs→ Trypco1→	1303	Streptomyces sp. SA3_actG	GCA_900091815.1	actinobacteria
WP_010310059.1	vWA+TPRs	S1COLD→ PSE→ ?→ PSE→ ||← ?||?→ Trypco1→ Trypsin+HDxxH+VMAP-M0+VMAP-C→ MoxR→ vWA+TPRs*→ Voltage_CLC→ FXSXX-COOH-like→ Radical_SAM→ METALLOPROTEASE+TPRs→ TIR+APATPase+TPRs→	1149	Saccharopolyspora spinosa	GCA_002846475.1	actinobacteria
WP_010353495.1	vWA+TIR+FxsC_Cter(partial)	AB_hydrolase→ CASPASE+EAD2+VMAP-M0+VMAP-C→ MoxR→ vWA+TIR+FxsC_Cter(partial)*→	809	Streptomyces acidiscabies	GCF_000242715.1	actinobacteria
WP_010353577.1	vWA+TPRs	TCAD15||PSE→ Trypsin+HDxxH+VMAP-M0+VMAP-C→ MoxR→ vWA+TPRs*→ FXSXX-COOH→ Radical_SAM→ METALLOPROTEASE+TPRs→ TIR+APATPase+TPRs→	1197	Streptomyces acidiscabies	-	actinobacteria
WP_010354765.1	vWA+Pkinase+wHTH+cNMP_cyclase+Band7+HAD	Trypco1→ Trypsin+VMAP-M0+VMAP-C→ MoxR→ vWA+Pkinase+wHTH+cNMP_cyclase+Band7+HAD*→	1366	Streptomyces acidiscabies	GCF_000242715.1	actinobacteria
WP_010356078.1	vWA+Pkinase+TPRs	Trypco2→ Trypsin+EAD2+VMAP-M0+VMAP-C→ MoxR→ vWA+Pkinase+TPRs*→	1028	Streptomyces acidiscabies	GCA_001485105.1	actinobacteria
WP_010470739.1	vWA+APATPase+wHTH+TPRs+cNMP_cyclase+NaeI	Trypco1→ AB_hydrolase→ CASPASE+VMAP-M2+VMAP-C→ MoxR→ vWA+APATPase+wHTH+TPRs+cNMP_cyclase+NaeI*→	1708	Streptomyces somaliensis	GCF_000258595.1	actinobacteria
WP_010642339.1	vWA+TPRs	TCAD15||Trypco1→ Trypsin+HDxxH+VMAP-M0+VMAP-C→ MoxR→ vWA+TPRs*→ FXSXX-COOH→ Radical_SAM→ PSE→ METALLOPROTEASE+TPRs→ TIR+APATPase+TPRs→	1137	Streptomyces sp. S4	-	actinobacteria
WP_010983774.1	vWA+APATPase+wHTH+TPRs+cNMP_cyclase+NaeI	AB_hydrolase→ CASPASE+VMAP-M2+VMAP-C→ MoxR→ vWA+APATPase+wHTH+TPRs+cNMP_cyclase+NaeI*→	1667	Streptomyces avermitilis	GCA_000009765.2	actinobacteria
WP_011028360.1	vWA+Pkinase+wHTH+TPRs+cNMP_cyclase	Trypco1→ Trypsin+VMAP-M0+VMAP-C→ MoxR→ vWA+Pkinase+wHTH+TPRs+cNMP_cyclase*→	1349	Streptomyces coelicolor	GCF_000203835.1	actinobacteria
WP_011030142.1	vWA+TPRs	TCAD15||Trypco1→ Trypsin+HDxxH+VMAP-M0+VMAP-C→ MoxR→ vWA+TPRs*→ FXSXX-COOH→ Radical_SAM→ METALLOPROTEASE+TPRs→ TIR+APATPase+TPRs→ Trypco1→	1096	Streptomyces coelicolor	GCF_000203835.1	actinobacteria
WP_011601499.1	vWA+APATPase+wHTH+TPRs	EAD2→ EAD2+VMAP-M0+VMAP-C→ MoxR→ vWA+APATPase+wHTH+TPRs*→ ||← METALLOPROTEASE+TPRs	1346	Frankia alni	GCA_000058485.1	actinobacteria
WP_011611368.1	vWA+FGS-fold_domain	Trypco1→ Trypsin+VMAP-M1+VMAP-C→ MoxR→ vWA+FGS-fold_domain*→ four_helix_bundle_protein→ PSE→ FGS-fold_domain→	877	Trichodesmium erythraeum	GCA_000014265.1	cyanobacteria
WP_011611683.1	vWA+FGS-fold_domain	EAD7+VMAP-M1+VMAP-C→ MoxR→ ?→ ?→ vWA+FGS-fold_domain*→ FGS-fold_domain→	892	Trichodesmium erythraeum	GCA_000014265.1	cyanobacteria
WP_012167133.1	vWA+VMAP-C+FGS-fold_domain	FGS-fold_domain→ ||← ?||PNP_UDP_1+VMAP-M1+VMAP-C→ MoxR→ vWA+VMAP-C+FGS-fold_domain*→ FGS-fold_domain→ ||← PSE||FGS-fold_domain→	1201	Acaryochloris marina	GCA_000018105.1	cyanobacteria
WP_012266972.1	vWA+FGS-fold_domain	HTH+VMAP-M9+VMAP-C→ VMAP-C(partial)→ MoxR→ vWA+FGS-fold_domain*→ ?→ ?→ ?→ FGS-fold_domain→	961	Microcystis aeruginosa	GCA_000010625.1	cyanobacteria
WP_012267113.1	vWA+FGS-fold_domain	Trypco1→ ||← ?||Trypsin+VMAP-M1+VMAP-C→ PSE→ ?→ vWA+FGS-fold_domain*→	871	Microcystis aeruginosa	GCA_000010625.1	cyanobacteria
WP_012377981.1	vWA+Pkinase+ParA-Soj-PloopNTPase(SIMIBI)	Trypco1→ Trypsin+EAD2+VMAP-M0+VMAP-C→ MoxR→ vWA+Pkinase+ParA-Soj-PloopNTPase(SIMIBI)*→ ||← ?← ?← PSE||?→ ||← ?← PSE← ?← Trypco1	1070	Streptomyces griseus	GCA_000010605.1	actinobacteria
WP_012381064.1	vWA+TPRs+TIR+FxsC_Cter(partial)	AB_hydrolase→ CASPASE+VMAP-M2+VMAP-C→ MoxR→ vWA+TPRs+TIR+FxsC_Cter(partial)*→	1512	Streptomyces griseus	GCA_000010605.1	actinobacteria
WP_012784685.1	vWA+TPRs	Trypsin+HDxxH+VMAP-M0+VMAP-C→ MoxR→ vWA+TPRs*→ ?→ Radical_SAM→	822	Catenulispora acidiphila	GCA_000024025.1	actinobacteria
WP_013155599.1	vWA+APATPase+wHTH+TPRs	Trypsin+VMAP-M0+VMAP-C→ vWA+APATPase+wHTH+TPRs*→ ||← DUF397||Trypco1→	1261	Nocardiopsis dassonvillei	GCA_000092985.1	actinobacteria
WP_013229639.1	vWA+APATPase+wHTH+TPRs	cNMP_cyclase+EAD2→ EAD2+VMAP-M0+VMAP-C→ MoxR→ vWA+APATPase+wHTH+TPRs*→ ||← METALLOPROTEASE+TPRs← FXSXX-COOH-like	1226	Amycolatopsis mediterranei	GCA_000196835.1	actinobacteria
WP_013424069.1	vWA+APATPase+wHTH+TPRs	EAD2+VMAP-M0+VMAP-C→ MoxR→ vWA+APATPase+wHTH+TPRs*→ EAD2+EAD1→	1387	Frankia inefficax	GCA_000166135.1	actinobacteria
WP_014043988.1	vWA+SFII-helicase+Z1	AB_hydrolase→ CASPASE+VMAP-M2+VMAP-C→ MoxR→ vWA+SFII-helicase+Z1*→	1390	Streptomyces	GCA_000177195.2	actinobacteria
WP_014049540.1	vWA+APATPase+wHTH+TPRs	EAD2+VMAP-M0+VMAP-C→ MoxR→ vWA+APATPase+wHTH+TPRs*→ AB_hydrolase→ CASPASE+EAD2→ FXSXX-COOH-like→ METALLOPROTEASE+TPRs→	1445	Streptomyces	GCA_000177195.2	actinobacteria
WP_014055711.1	vWA+Pkinase+ZNR+Pkinase+ParA-Soj-PloopNTPase(SIMIBI)	ADP_ribosyl_GH→ SIR2→ ||← ?← ?||Trypco1→ Trypsin+EAD2+VMAP-M0+VMAP-C→ MoxR→ vWA+Pkinase+ZNR+Pkinase+ParA-Soj-PloopNTPase(SIMIBI)*→ Porphobil_deam+Porphobil_deamC→	1619	Streptomyces violaceusniger	GCA_000147815.3	actinobacteria
WP_014056867.1	vWA+APATPase+wHTH+TPRs	PNP_UDP_1+EAD2→ EAD2+VMAP-M0+VMAP-C→ MoxR→ vWA+APATPase+wHTH+TPRs*→ ||← METALLOPROTEASE+TPRs	1452	Streptomyces violaceusniger	GCA_000147815.3	actinobacteria
WP_014133537.1	vWA+CASPASE+TCAD14+PIN+CCCH(ZNR)+FHA	Trypco1→ Trypsin+HDxxH+VMAP-M0+VMAP-C→ MoxR→ vWA+CASPASE+TCAD14+PIN+CCCH(ZNR)+FHA*→	1228	Kitasatospora setae	GCA_000269985.1	actinobacteria
WP_014142316.1	vWA+TPRs	AB_hydrolase→ CASPASE+EAD2+VMAP-M0+VMAP-C→ MoxR→ vWA+TPRs*→	1153	Streptomyces cattleya	GCA_000240165.1	actinobacteria
WP_014144833.1	vWA+TPRs	TCAD15||Trypco1→ Trypsin+HDxxH+VMAP-M0+VMAP-C→ MoxR→ vWA+TPRs*→ FXSXX-COOH→ Radical_SAM→ TIR+APATPase+TPRs→	1087	Streptomyces cattleya	GCA_000240165.1	actinobacteria
WP_014145553.1	vWA+Pkinase+ParA-Soj-PloopNTPase(SIMIBI)	Trypco1→ Trypsin+EAD2+VMAP-M0+VMAP-C→ MoxR→ vWA+Pkinase+ParA-Soj-PloopNTPase(SIMIBI)*→	1098	Streptomyces cattleya	GCA_000240165.1	actinobacteria
WP_014174718.1	vWA_protein	PNP_UDP_1+EAD2→ ?→ vWA*→ ?→ ?→ EAD2+VMAP-M0+VMAP-C→ MoxR→ vWA+APATPase+wHTH+TPRs→ ||← METALLOPROTEASE+TPRs	258	Streptomyces bingchenggensis	GCA_000092385.1	actinobacteria
WP_014174725.1	vWA+APATPase+wHTH+TPRs	PNP_UDP_1+EAD2→ ?→ vWA→ ?→ ?→ EAD2+VMAP-M0+VMAP-C→ MoxR→ vWA+APATPase+wHTH+TPRs*→ ||← METALLOPROTEASE+TPRs	1460	Streptomyces bingchenggensis	GCA_000092385.1	actinobacteria
WP_014175451.1	vWA+Pkinase+Pkinase+ParA-Soj-PloopNTPase(SIMIBI)	Trypco1→ Trypsin+EAD2+VMAP-M0+VMAP-C→ MoxR→ vWA+Pkinase+Pkinase+ParA-Soj-PloopNTPase(SIMIBI)*→ Porphobil_deam+Porphobil_deamC→	1410	Streptomyces bingchenggensis	GCA_000092385.1	actinobacteria
WP_014275764.1	vWA+TPRs+CASPASE	VMAP-C_protein→ MoxR→ vWA+TPRs+CASPASE*→	1693	Arthrospira platensis	GCA_000210375.1	cyanobacteria
WP_014672525.1	vWA+Pkinase+cNMP_cyclase+Band7+HAD	Trypco1→ Trypsin+VMAP-M0+VMAP-C→ MoxR→ vWA+Pkinase+cNMP_cyclase+Band7+HAD*→	1308	Streptomyces hygroscopicus	GCA_000245355.1	actinobacteria
WP_014674945.1	vWA+TPRs	TCAD15→ Trypco1→ Trypsin+HDxxH+VMAP-M0+VMAP-C→ MoxR→ vWA+TPRs*→ FXSXX-COOH-like→ METALLOPROTEASE+TPRs→ TIR+APATPase+TPRs→	1157	Streptomyces hygroscopicus	GCA_000245355.1	actinobacteria

<table>
<tr><th>acc</th><th>architecture</th><th>operon</th><th>len</th><th>species</th><th>gca</th><th>taxend</th></tr>
<tr><td>WP_015036147.1</td><td>vWA+TPRs</td><td>TCAD15||Trypco1→ Trypsin+HDxxH+VMAP-M0+VMAP-C→ MoxR→ vWA+TPRs*→ FXSXX-COOH→ METALLOPROTEASE+TPRs→ TIR+APATPase+TPRs→</td><td>1090</td><td>Streptomyces venezuelae</td><td>GCA_001886595.1</td><td>actinobacteria</td></tr>
<tr><td>WP_015038303.1</td><td>vWA+wHTH+TPRs+wHTH+TPRs+APATPase+wHTH+TPRs</td><td>Trypsin+EAD2+VMAP-M0+VMAP-C→ MoxR→ vWA+wHTH+TPRs+wHTH+TPRs+APATPase+wHTH+TPRs*→</td><td>1802</td><td>Streptomyces venezuelae</td><td>GCA_001886595.1</td><td>actinobacteria</td></tr>
<tr><td>WP_015079684.1</td><td>vWA+FGS-fold_domain</td><td>vWA+FGS-fold_domain*→</td><td>731</td><td>Anabaena sp. 90</td><td>GCA_000312705.1</td><td>cyanobacteria</td></tr>
<tr><td>WP_015081254.1</td><td>vWA+TPRs</td><td>CASPASE← ?||?→ ?→ EAD10+VMAP-M1+VMAP-C→ MoxR→ vWA+TPRs*→</td><td>725</td><td>Anabaena sp. 90</td><td>GCA_000312705.1</td><td>cyanobacteria</td></tr>
<tr><td>WP_015083216.1</td><td>vWA</td><td>vWA*→</td><td>603</td><td>Anabaena sp. 90</td><td>GCA_000312705.1</td><td>cyanobacteria</td></tr>
<tr><td>WP_015119023.1</td><td>vWA+FGS-fold_domain</td><td>EAD1+Trypsin→ EAD1+VMAP-M1+VMAP-C→ MoxR→ vWA+FGS-fold_domain*→ FGS-fold_domain→</td><td>886</td><td>Rivularia sp. PCC 7116</td><td>GCA_000316665.1</td><td>cyanobacteria</td></tr>
<tr><td>WP_015135700.1</td><td>vWA+GAF+FGS-fold_domain</td><td>FGS-fold_domain→ ||← RVT_1← four_helix_bundle_protein||FGS-fold_domain→ PSE→ Trypsin+VMAP-M1+VMAP-C→ MoxR→ vWA+GAF+FGS-fold_domain*→</td><td>1106</td><td>Leptolyngbya sp. PCC 7376</td><td>GCA_000316605.1</td><td>cyanobacteria</td></tr>
<tr><td>WP_015197943.1</td><td>vWA+MNS+CASPASE+MNS</td><td>MoxR← EAD10+VMAP-M1+VMAP-C← CASPASE← ?||EAD10+VMAP-M1+VMAP-C→ MoxR→ vWA+MNS+CASPASE+MNS*→</td><td>1286</td><td>Calothrix parietina</td><td>GCA_000317435.1</td><td>cyanobacteria</td></tr>
<tr><td>WP_015620661.1</td><td>vWA+TCAD13+NaeI+TPRs</td><td>AB_hydrolase→ CASPASE+VMAP-M2+VMAP-C→ MoxR→ vWA+TCAD13+NaeI+TPRs*→</td><td>1332</td><td>Actinoplanes sp. N902-109</td><td>GCA_000389965.1</td><td>actinobacteria</td></tr>
<tr><td>WP_015655512.1</td><td>vWA+EAD2+VMAP-M0+VMAP-C</td><td>Trypco1→ Trypsin+VMAP-M7+VMAP-C→ MoxR→ vWA+EAD2+VMAP-M0+VMAP-C*→</td><td>1007</td><td>Streptomyces davawensis</td><td>GCA_000349325.1</td><td>actinobacteria</td></tr>
<tr><td>WP_015655648.1</td><td>vWA+STAND_NTPase+Trypsin+CASPASE+S1COLD</td><td>AB_hydrolase→ CASPASE+VMAP-M0+VMAP-C→ MoxR→ vWA+STAND_NTPase+Trypsin+CASPASE+S1COLD*→</td><td>1319</td><td>Streptomyces davawensis</td><td>GCA_000349325.1</td><td>actinobacteria</td></tr>
<tr><td>WP_015660488.1</td><td>vWA+APATPase+wHTH+TPRs</td><td>AB_hydrolase→ EAD2+VMAP-M0+VMAP-C→ MoxR→ vWA+APATPase+wHTH+TPRs*→ ||← CASPASE+EAD2</td><td>1416</td><td>Streptomyces davawensis</td><td>GCA_000349325.1</td><td>actinobacteria</td></tr>
<tr><td>WP_015660598.1</td><td>vWA+Pkinase+wHTH+TPRs+cNMP_cyclase+Band7+HAD</td><td>Trypco1→ Trypsin+VMAP-M0+VMAP-C→ MoxR→ vWA+Pkinase+wHTH+TPRs+cNMP_cyclase+Band7+HAD*→</td><td>1583</td><td>Streptomyces davawensis</td><td>GCA_000349325.1</td><td>actinobacteria</td></tr>
<tr><td>WP_015792498.1</td><td>vWA+TPRs+CASPASE</td><td>AB_hydrolase→ CASPASE+VMAP-M0+VMAP-C→ MoxR→ vWA+TPRs+CASPASE*→</td><td>1199</td><td>Catenulispora acidiphila</td><td>GCA_000024025.1</td><td>actinobacteria</td></tr>
<tr><td>WP_016188158.1</td><td>vWA+APATPase+wHTH+TPRs</td><td>vWA+APATPase+wHTH+TPRs*→ ||← DUF397||Trypco1→</td><td>1422</td><td>Thermobifida fusca</td><td>GCA_000401915.1</td><td>actinobacteria</td></tr>
<tr><td>WP_016334896.1</td><td>vWA+GCV_H+HTH+cNMP_cyclase+cNMP_cyclase+TIR+CAS-PASE</td><td>AB_hydrolase→ CASPASE+VMAP-M0+VMAP-C→ MoxR→ vWA+GCV_H+HTH+cNMP_cyclase+cNMP_cyclase+TIR+CASPASE*→</td><td>1568</td><td>Amycolatopsis orientalis</td><td>GCA_000400635.2</td><td>actinobacteria</td></tr>
<tr><td>WP_016435421.1</td><td>vWA+cNMP_cyclase+Band7+HAD</td><td>Trypco1→ Trypsin+VMAP-M0+VMAP-C→ MoxR→ vWA+cNMP_cyclase+Band7+HAD*→</td><td>1093</td><td>Streptomyces sp. HGB0020</td><td>GCA_000411315.1</td><td>actinobacteria</td></tr>
<tr><td>WP_016469758.1</td><td>vWA+TPRs</td><td>TCAD15→ ?→ ?→ Trypco1→ Trypsin+HDxxH+VMAP-M0+VMAP-C→ MoxR→ vWA+TPRs*→ FXSXX-COOH-like→ METALLOPROTEASE+TPRs→ TIR+APATPase+TPRs→</td><td>1054</td><td>Streptomyces</td><td>GCA_000411495.1</td><td>actinobacteria</td></tr>
<tr><td>WP_016515783.1</td><td>vWA+FGS-fold_domain</td><td>MoxR→ vWA(partial)→ vWA+FGS-fold_domain*→</td><td>838</td><td>Microcystis aeruginosa</td><td>GCA_000412595.1</td><td>cyanobacteria</td></tr>
<tr><td>WP_016516064.1</td><td>vWA+FGS-fold_domain</td><td>HTH+VMAP-M9+VMAP-C→ MoxR→ vWA+FGS-fold_domain*→</td><td>961</td><td>Microcystis aeruginosa</td><td>GCA_000412595.1</td><td>cyanobacteria</td></tr>
<tr><td>WP_016642618.1</td><td>vWA+Pkinase+wHTH+TPRs+cNMP_cyclase+HAD</td><td>Trypco1→ Trypsin+VMAP-M0+VMAP-C→ MoxR→ ||← ?||vWA+Pkinase+wHTH+TPRs+cNMP_cyclase+HAD*→</td><td>1140</td><td>Streptomyces aurantiacus</td><td>GCA_000414115.1</td><td>actinobacteria</td></tr>
<tr><td>WP_016907460.1</td><td>vWA+TPRs</td><td>Trypco1||Trypsin+HDxxH+VMAP-M0+VMAP-C→ MoxR→ vWA+TPRs*→ FXSXX-COOH→ METALLOPROTEASE+TPRs→ TIR+APATPase+TPRs→</td><td>1054</td><td>Streptomyces xiaopingdaonensis</td><td>GCF_000262345.1</td><td>actinobacteria</td></tr>
<tr><td>WP_017530615.1</td><td>vWA+BetaPropeller+TCAD10+REC</td><td>AB_hydrolase→ CASPASE+VMAP-M16+VMAP-C→ MoxR→ vWA+BetaPropeller+TCAD10+REC*→</td><td>1202</td><td>Pseudomonas fluorescens</td><td>GCF_000308175.1</td><td>gammaproteobacteria</td></tr>
<tr><td>WP_017562502.1</td><td>vWA+APATPase+wHTH+TPRs</td><td>Trypsin+VMAP-M0+VMAP-C→ vWA+APATPase+wHTH+TPRs*→ ||← DUF397||Trypco1→</td><td>1259</td><td>Nocardiopsis synnemataformans</td><td>GCF_000340945.1</td><td>actinobacteria</td></tr>
<tr><td>WP_017563873.1</td><td>vWA+APATPase+wHTH+TPRs</td><td>Trypsin+EAD2→ EAD2+VMAP-M0+VMAP-C→ MoxR→ vWA+APATPase+wHTH+TPRs*→ ||← DUF397</td><td>1516</td><td>Nocardiopsis synnemataformans</td><td>GCF_000340945.1</td><td>actinobacteria</td></tr>
<tr><td>WP_017571137.1</td><td>vWA+APATPase+wHTH+TPRs</td><td>Trypsin+EAD2→ EAD2+VMAP-M0+VMAP-C→ MoxR→ vWA+APATPase+wHTH+TPRs*→ ||← DUF397</td><td>1494</td><td>Nocardiopsis halotolerans</td><td>GCF_000341065.1</td><td>actinobacteria</td></tr>
<tr><td>WP_017577293.1</td><td>vWA+APATPase+wHTH+TPRs</td><td>Trypsin+VMAP-M0+VMAP-C→ vWA+APATPase+wHTH+TPRs*→ ||← DUF397||Trypco1→</td><td>1283</td><td>Nocardiopsis kunsanensis</td><td>GCF_000340965.1</td><td>actinobacteria</td></tr>
<tr><td>WP_017587752.1</td><td>vWA+APATPase+wHTH+TPRs</td><td>Trypsin+EAD2→ EAD2+VMAP-M0+VMAP-C→ MoxR→ vWA+APATPase+wHTH+TPRs*→ ||← DUF397</td><td>1518</td><td>Nocardiopsis ganjiahuensis</td><td>GCF_000341085.1</td><td>actinobacteria</td></tr>
<tr><td>WP_017591222.1</td><td>vWA+APATPase+wHTH+TPRs</td><td>Trypsin+VMAP-M0+VMAP-C→ MoxR→ vWA+APATPase+wHTH+TPRs*→ ||← DUF397||Trypco1→</td><td>1508</td><td>Nocardiopsis potens</td><td>GCF_000341105.1</td><td>actinobacteria</td></tr>
<tr><td>WP_017595227.1</td><td>vWA+APATPase+wHTH+TPRs</td><td>Trypsin+EAD2+VMAP-M0+VMAP-C→ MoxR→ vWA+APATPase+wHTH+TPRs*→ ||← DUF397</td><td>1532</td><td>Nocardiopsis potens</td><td>GCF_000341105.1</td><td>actinobacteria</td></tr>
<tr><td>WP_017600471.1</td><td>vWA+APATPase+wHTH+TPRs</td><td>vWA+APATPase+wHTH+TPRs*→ ||← DUF397||Trypco1→</td><td>1252</td><td>Nocardiopsis lucentensis</td><td>GCF_000341125.1</td><td>actinobacteria</td></tr>
<tr><td>WP_017607480.1</td><td>vWA+APATPase+wHTH+TPRs</td><td>Trypsin+VMAP-M0+VMAP-C→ vWA+APATPase+wHTH+TPRs*→ ||← DUF397||Trypco1→</td><td>1282</td><td>Nocardiopsis xinjiangensis</td><td>GCF_000341145.1</td><td>actinobacteria</td></tr>
<tr><td>WP_017615244.1</td><td>vWA+APATPase+wHTH+TPRs</td><td>Trypsin+VMAP-M0+VMAP-C→ vWA+APATPase+wHTH+TPRs*→ ||← DUF397||Trypco1→</td><td>1276</td><td>Nocardiopsis salina</td><td>GCF_000341025.1</td><td>actinobacteria</td></tr>
<tr><td>WP_017622184.1</td><td>vWA+APATPase+wHTH+TPRs</td><td>Trypsin+VMAP-M0+VMAP-C→ MoxR→ vWA+APATPase+wHTH+TPRs*→ ||← DUF397||Trypco1→</td><td>1528</td><td>Nocardiopsis chromatogenes</td><td>GCF_000341185.1</td><td>actinobacteria</td></tr>
<tr><td>WP_017661634.1</td><td>vWA+FGS-fold_domain</td><td>-</td><td>871</td><td>Geitlerinema sp. PCC 7105</td><td>-</td><td>cyanobacteria</td></tr>
<tr><td>WP_017742480.1</td><td>vWA+FGS-fold_domain</td><td>EAD1+VMAP-M1+VMAP-C→ MoxR→ vWA+FGS-fold_domain*→ four_helix_bundle_protein→ RVT_1→</td><td>871</td><td>Scytonema hofmannii</td><td>GCA_000346485.2</td><td>cyanobacteria</td></tr>
<tr><td>WP_017742621.1</td><td>vWA+CASPASE+APATPase</td><td>low-complexity+VMAP-M26+VMAP-C→ MoxR→ vWA+CASPASE+APATPase*→</td><td>1326</td><td>Scytonema hofmannii</td><td>GCA_000346485.2</td><td>cyanobacteria</td></tr>
<tr><td>WP_017746610.1</td><td>vWA_protein</td><td>vWA_protein*→</td><td>259</td><td>Scytonema hofmannii</td><td>GCA_000346485.2</td><td>cyanobacteria</td></tr>
<tr><td>WP_017748256.1</td><td>vWA+FGS-fold_domain</td><td>TCAD3+EAD1+EAD9→ EAD9+EAD1+VMAP-C→ MoxR→ vWA+FGS-fold_domain*→ FGS-fold_domain→ four_helix_bundle_protein→ RVT_1→</td><td>882</td><td>Scytonema hofmannii</td><td>GCA_000346485.2</td><td>cyanobacteria</td></tr>
<tr><td>WP_018087270.1</td><td>vWA+wHTH+TPRs+APATPase+wHTH+TPRs</td><td>Trypco1→ Trypsin+EAD2+VMAP-M0+VMAP-C→ MoxR→ vWA+wHTH+TPRs+APATPase+wHTH+TPRs*→ ?→ ?→ ||← AB_hydrolase</td><td>1612</td><td>Streptomyces sp. FxanaC1</td><td>GCF_000375625.1</td><td>actinobacteria</td></tr>
<tr><td>WP_018087461.1</td><td>vWA+TPRs</td><td>TCAD15||Trypco1→ Trypsin+HDxxH+VMAP-M0+VMAP-C→ MoxR→ vWA+TPRs*→ FXSXX-COOH→ Radical_SAM→ TIR+APATPase+TPRs→</td><td>1132</td><td>Streptomyces sp. FxanaC1</td><td>-</td><td>actinobacteria</td></tr>
<tr><td>WP_018102708.1</td><td>vWA+TPRs</td><td>TCAD15||Trypco1→ Trypsin+HDxxH+VMAP-M0+VMAP-C→ MoxR→ vWA+TPRs*→ FXSXX-COOH→ Radical_SAM→ METALLOPROTEASE+TPRs→ TIR+APATPase+TPRs→ Trypco1→</td><td>1139</td><td>Streptomyces</td><td>GCF_000375725.1</td><td>actinobacteria</td></tr>
<tr><td>WP_018383820.1</td><td>vWA+APATPase+wHTH+TPRs</td><td>AB_hydrolase→ EAD2+VMAP-M0+VMAP-C→ MoxR→ vWA+APATPase+wHTH+TPRs*→ ||← METALLOPROTEASE+TPRs← CASPASE+EAD2</td><td>1526</td><td>Streptomyces vitaminophilus</td><td>GCA_001445835.1</td><td>actinobacteria</td></tr>
<tr><td>WP_018470761.1</td><td>vWA+TPRs</td><td>TCAD15||Trypco1→ Trypsin+HDxxH+VMAP-M0+VMAP-C→ MoxR→ vWA+TPRs*→ FXSXX-COOH→ Radical_SAM→ METALLOPROTEASE+TPRs→ TIR+APATPase+TPRs→</td><td>1137</td><td>Streptomyces sp. LaPpAH-202</td><td>GCA_003688775.1</td><td>actinobacteria</td></tr>
<tr><td>WP_018535458.1</td><td>vWA+APATPase+wHTH+TPRs</td><td>EAD2+VMAP-M0+VMAP-C→ MoxR→ vWA+APATPase+wHTH+TPRs*→ AB_hydrolase→ CASPASE+EAD2→ FXSXX-COOH-like→ METALLOPROTEASE+TPRs→</td><td>1440</td><td>Streptomyces sp. HmicA12</td><td>GCF_000373565.1</td><td>actinobacteria</td></tr>
<tr><td>WP_018543767.1</td><td>vWA+APATPase+wHTH+TPRs</td><td>Trypsin+EAD2→ EAD2+VMAP-M0+VMAP-C→ MoxR→ vWA+APATPase+wHTH+TPRs*→ ||← METALLOPROTEASE+TPRs← FXSXX-COOH-like||Trypco1→</td><td>1537</td><td>Streptomyces sp. LaPpAH-108</td><td>GCF_000373625.1</td><td>actinobacteria</td></tr>
<tr><td>WP_018558006.1</td><td>vWA+APATPase+wHTH+TPRs</td><td>Trypco1→ Trypsin+EAD2→ EAD2+VMAP-M0+VMAP-C→ MoxR→ vWA+APATPase+wHTH+TPRs*→ ||← METALLOPROTEASE+TPRs← FXSXX-COOH-like</td><td>1483</td><td>Streptomyces sp. BoleA5</td><td>GCF_000373665.1</td><td>actinobacteria</td></tr>
<tr><td>WP_018835112.1</td><td>vWA+TPRs</td><td>TCAD15||Trypco1→ Trypsin+HDxxH+VMAP-M0+VMAP-C→ MoxR→ vWA+TPRs*→ FXSXX-COOH→ Radical_SAM→ TIR+APATPase+TPRs→</td><td>1072</td><td>Streptomyces sp. CNQ766</td><td>GCF_000377105.1</td><td>actinobacteria</td></tr>
<tr><td>WP_018853257.1</td><td>vWA+TPRs</td><td>TCAD15||Trypco1→ Trypsin+HDxxH+VMAP-M0+VMAP-C→ MoxR→ vWA+TPRs*→ FXSXX-COOH→ Radical_SAM→ TIR+APATPase+TPRs→</td><td>1072</td><td>Streptomyces sp. CNY243</td><td>GCF_000377165.1</td><td>actinobacteria</td></tr>
<tr><td>WP_018893565.1</td><td>vWA+TPRs</td><td>TCAD15||Trypco1→ Trypsin+HDxxH+VMAP-M0+VMAP-C→ MoxR→ vWA+TPRs*→ FXSXX-COOH→ Radical_SAM→ METALLOPROTEASE+TPRs→ TIR+APATPase+TPRs→</td><td>1137</td><td>Streptomyces sp. CNY228</td><td>GCF_000377545.1</td><td>actinobacteria</td></tr>
<tr><td>WP_018955297.1</td><td>vWA+APATPase+wHTH+TPRs</td><td>EAD2+VMAP-M0+VMAP-C→ MoxR→ vWA+APATPase+wHTH+TPRs*→ AB_hydrolase→ CASPASE+EAD2→ FXSXX-COOH-like→ METALLOPROTEASE+TPRs→</td><td>1484</td><td>Streptomyces sp. CNB091</td><td>GCF_000377965.1</td><td>actinobacteria</td></tr>
</table>

<table>
<tr><th>acc</th><th>architecture</th><th>operon</th><th>len</th><th>species</th><th>gca</th><th>taxend</th></tr>
<tr><td>WP_019325899.1</td><td>vWA+APATPase+wHTH+TPRs</td><td>EAD2+VMAP-M0+VMAP-C→ MoxR→ vWA+APATPase+wHTH+TPRs*→ ||← Na_H_Exchanger</td><td>1548</td><td>Streptomyces sp. TOR3209</td><td>GCA_003984925.1</td><td>actinobacteria</td></tr>
<tr><td>WP_019354554.1</td><td>vWA+TPRs</td><td>Trypco1→ Trypsin+HDxxH+VMAP-M0+VMAP-C→ MoxR→ vWA+TPRs*→ FXSXX-COOH-like→ METALLOPROTEASE+TPRs→ TIR+APATPase+TPRs→</td><td>1069</td><td>Streptomyces sp. AA1529</td><td>GCF_000280905.1</td><td>actinobacteria</td></tr>
<tr><td>WP_019433358.1</td><td>vWA+TPRs</td><td>TCAD15||Trypco1→ Trypsin+HDxxH+VMAP-M0+VMAP-C→ MoxR→ vWA+TPRs*→ FXSXX-COOH→ Radical_SAM→</td><td>1110</td><td>Streptomyces sp. AA0539</td><td>GCF_000297635.1</td><td>actinobacteria</td></tr>
<tr><td>WP_019496021.1</td><td>vWA+Glyco_trans_4_4+Glycos_transf_1</td><td>EAD1+VMAP-M23+VMAP-C→ MoxR→ vWA+Glyco_trans_4_4+Glycos_transf_1*→</td><td>977</td><td>Calothrix sp. PCC 7103</td><td>GCF_000331305.1</td><td>cyanobacteria</td></tr>
<tr><td>WP_019496294.1</td><td>vWA+FGS-fold_domain</td><td>EAD9+VMAP-M1+VMAP-C→ MoxR→ vWA+FGS-fold_domain*→ FGS-fold_domain→ EAD1+APATPase+TPRs→</td><td>875</td><td>Calothrix sp. PCC 7103</td><td>GCF_000331305.1</td><td>cyanobacteria</td></tr>
<tr><td>WP_019496819.1</td><td>vWA+MNS+CASPASE+MNS</td><td>VMAP-M1+VMAP-C→ MoxR→ vWA+MNS+CASPASE+MNS*→</td><td>1293</td><td>Calothrix sp. PCC 7103</td><td>GCF_000331305.1</td><td>cyanobacteria</td></tr>
<tr><td>WP_019545070.1</td><td>vWA+TPRs</td><td>TCAD15← ?||?→ ||← Trypco1||Trypsin+HDxxH+VMAP-M0+VMAP-C→ MoxR→ vWA+TPRs*→ FXSXX-COOH→ METALLOPROTEASE+TPRs→ TIR+APATPase+TPRs→</td><td>1054</td><td>Streptomyces sulphureus</td><td>GCF_000381025.1</td><td>actinobacteria</td></tr>
<tr><td>WP_019607628.1</td><td>vWA+APATPase+wHTH+TPRs</td><td>Trypsin+EAD2→ EAD2+VMAP-M0+VMAP-C→ MoxR→ vWA+APATPase+wHTH+TPRs*→ ||← DUF397</td><td>1509</td><td>Nocardiopsis sp. CNS-639</td><td>GCF_000381685.1</td><td>actinobacteria</td></tr>
<tr><td>WP_019608801.1</td><td>vWA+APATPase+wHTH+TPRs</td><td>Trypsin+VMAP-M0+VMAP-C→ vWA+APATPase+wHTH+TPRs*→ ||← DUF397||Trypco1→</td><td>1261</td><td>Nocardiopsis sp. CNS-639</td><td>GCF_000381685.1</td><td>actinobacteria</td></tr>
<tr><td>WP_019889387.1</td><td>vWA+TPRs</td><td>TCAD15||Trypco1→ Trypsin+HDxxH+VMAP-M0+VMAP-C→ MoxR→ vWA+TPRs*→ Radical_SAM→ TIR+APATPase+TPRs→</td><td>1062</td><td>Streptomyces purpureus</td><td>GCF_000384175.1</td><td>actinobacteria</td></tr>
<tr><td>WP_020126655.1</td><td>vWA+APATPase+wHTH+TPRs</td><td>Trypco1→ Trypsin+EAD2+VMAP-M0+VMAP-C→ MoxR→ vWA+APATPase+GGDEF+EAD2+EAD2+VMAP-M0→ EAD2+VMAP-M0+VMAP-C→ MoxR→ vWA+APATPase+wHTH+TPRs*→</td><td>1428</td><td>Streptomyces sp. 303MFCol5.2</td><td>-</td><td>actinobacteria</td></tr>
<tr><td>WP_020275244.1</td><td>vWA+cNMP_cyclase+Band7</td><td>Trypco1→ Trypsin+VMAP-M0+VMAP-C→ MoxR→ ?→ vWA+cNMP_cyclase+Band7*→</td><td>851</td><td>Streptomyces afghaniensis</td><td>GCA_000415505.1</td><td>actinobacteria</td></tr>
<tr><td>WP_020277090.1</td><td>vWA+TPRs</td><td>TCAD15||Trypco1→ Trypsin+HDxxH+VMAP-M0+VMAP-C→ MoxR→ vWA+TPRs*→</td><td>1107</td><td>Streptomyces afghaniensis</td><td>GCA_000415505.1</td><td>actinobacteria</td></tr>
<tr><td>WP_020507876.1</td><td>vWA+FGS-fold_domain+FGS-fold_domain+PilP+FGS-fold_domain</td><td>CASPASE+VMAP-M27+VMAP-C→ MoxR→ vWA+FGS-fold_domain+FGS-fold_domain+PilP+FGS-fold_domain*→</td><td>1617</td><td>Lamprocystis purpurea</td><td>GCF_000379525.1</td><td>gammaproteobacteria</td></tr>
<tr><td>WP_020634031.1</td><td>vWA+APATPase+wHTH+TPRs</td><td>cNMP_cyclase+VMAP-M0+VMAP-C→ PSE→ vWA+APATPase+wHTH+TPRs*→ ||← METALLOPROTEASE+TPRs← FXSXX-COOH-like← cNMP_binding+HTH_Crp_2</td><td>1471</td><td>Amycolatopsis alba</td><td>GCA_002234385.1</td><td>actinobacteria</td></tr>
<tr><td>WP_020641866.1</td><td>vWA+APATPase+wHTH+TPRs</td><td>Trypco1← ?||EAD2+VMAP-M0+VMAP-C→ MoxR→ vWA+APATPase+wHTH+TPRs*→ ||← METALLOPROTEASE+TPRs</td><td>1394</td><td>Amycolatopsis balhimycina</td><td>GCF_000384295.1</td><td>actinobacteria</td></tr>
<tr><td>WP_021593015.1</td><td>vWA+APATPase+wHTH+TPRs</td><td>Radical_SAM→ ||← TIR+APATPase+TPRs||SWACOS→ ?→ EAD2+VMAP-M0+VMAP-C→ MoxR→ vWA+APATPase+wHTH+TPRs*→ ||← Radical_SAM← ?||cNMP_cyclase+EAD2→ ||← ? ?||cNMP_binding+HTH_Crp_2→</td><td>1392</td><td>Actinomadura madurae</td><td>GCF_000468475.2</td><td>actinobacteria</td></tr>
<tr><td>WP_023065259.1</td><td>vWA+FGS-fold_domain</td><td>Trypco1→ Trypsin+VMAP-M1+VMAP-C→ MoxR→ vWA+FGS-fold_domain*→ ||← ?||FGS-fold_domain→</td><td>868</td><td>Lyngbya aestuarii</td><td>GCA_000478195.2</td><td>cyanobacteria</td></tr>
<tr><td>WP_023072613.1</td><td>vWA+FGS-fold_domain</td><td>FGS-fold_domain→ ||← ?||PNP_UDP_1+VMAP-M1+VMAP-C→ MoxR→ vWA+FGS-fold_domain*→ FGS-fold_domain→</td><td>879</td><td>Leptolyngbya sp. Heron Island J</td><td>GCA_000482245.1</td><td>cyanobacteria</td></tr>
<tr><td>WP_023538706.1</td><td>vWA+TPRs</td><td>TCAD15||PSE→ Trypsin+HDxxH+VMAP-M0+VMAP-C→ MoxR→ vWA+TPRs*→ FXSXX-COOH→ METALLOPROTEASE+TPRs→ TIR+APATPase+TPRs→</td><td>982</td><td>Streptomyces niveus</td><td>GCA_000497425.1</td><td>actinobacteria</td></tr>
<tr><td>WP_023546824.1</td><td>vWA+TPRs</td><td>TCAD15→ Trypco1→ Trypsin+HDxxH+VMAP-M0+VMAP-C→ MoxR→ vWA+TPRs*→ PSE→ PSE→ TIR+APATPase+TPRs→</td><td>1092</td><td>Streptomyces roseochromogenus</td><td>GCA_000497445.1</td><td>actinobacteria</td></tr>
<tr><td>WP_023590986.1</td><td>vWA+APATPase+wHTH+TPRs+cNMP_cyclase+NaeI</td><td>Trypco1→ ?→ ?→ AB_hydrolase→ CASPASE+VMAP-M2+VMAP-C→ MoxR→ vWA+APATPase+wHTH+TPRs+cNMP_cyclase+NaeI*→</td><td>1704</td><td>Streptomyces thermolilacinus</td><td>GCA_000478605.2</td><td>actinobacteria</td></tr>
<tr><td>WP_024075602.1</td><td>vWA+BetaPropeller+TCAD10+REC</td><td>AB_hydrolase→ CASPASE+VMAP-M16+VMAP-C→ MoxR→ vWA+BetaPropeller+TCAD10+REC*→</td><td>1202</td><td>Pseudomonas</td><td>GCA_000508205.1</td><td>gammaproteobacteria</td></tr>
<tr><td>WP_024491665.1</td><td>vWA+TPRs</td><td>AB_hydrolase→ CASPASE+EAD2+VMAP-M0+VMAP-C→ MoxR→ vWA+TPRs*→</td><td>1191</td><td>Streptomyces sp. AW19M42</td><td>GCF_000470535.1</td><td>actinobacteria</td></tr>
<tr><td>WP_024758050.1</td><td>vWA+APATPase+wHTH+TPRs</td><td>Trypsin+EAD2→ EAD2+VMAP-M0+VMAP-C→ MoxR→ vWA+APATPase+wHTH+TPRs*→ ||← METALLOPROTEASE+TPRs</td><td>1550</td><td>Streptomyces exfoliatus</td><td>GCF_000518045.1</td><td>actinobacteria</td></tr>
<tr><td>WP_024761808.1</td><td>vWA+TPRs</td><td>TCAD15||Trypco1→ Trypsin+HDxxH+VMAP-M0+VMAP-C→ MoxR→ vWA+TPRs*→ METALLOPROTEASE+TPRs→ TIR+APATPase+TPRs→</td><td>1073</td><td>Streptomyces exfoliatus</td><td>-</td><td>actinobacteria</td></tr>
<tr><td>WP_024934114.1</td><td>vWA+FtsK+FtsK+FtsK</td><td>Trypco1→ Trypsin+VMAP-M8+VMAP-C→ MoxR→ vWA+FtsK+FtsK+FtsK*→</td><td>1506</td><td>Actinomadura madurae</td><td>GCF_000468475.2</td><td>actinobacteria</td></tr>
<tr><td>WP_024968775.1</td><td>vWA+FGS-fold_domain</td><td>MoxR→ vWA+FGS-fold_domain*→</td><td>884</td><td>Microcystis aeruginosa</td><td>GCF_000599945.1</td><td>cyanobacteria</td></tr>
<tr><td>WP_024969247.1</td><td>vWA+FGS-fold_domain</td><td>vWA+FGS-fold_domain*→</td><td>876</td><td>Microcystis aeruginosa</td><td>GCF_000599945.1</td><td>cyanobacteria</td></tr>
<tr><td>WP_024970302.1</td><td>vWA+FGS-fold_domain</td><td>AB_hydrolase→ CASPASE+VMAP-M14+VMAP-C→ MoxR→ vWA+FGS-fold_domain*→</td><td>842</td><td>Microcystis aeruginosa</td><td>GCF_000599945.1</td><td>cyanobacteria</td></tr>
<tr><td>WP_026065859.1</td><td>vWA+TPRs</td><td>Trypco1→ Trypsin+HDxxH+VMAP-M0+VMAP-C→ MoxR→ vWA+TPRs*→ FXSXX-COOH-like→</td><td>874</td><td>Actinoalloteichus spitiensis</td><td>GCF_000239155.1</td><td>actinobacteria</td></tr>
<tr><td>WP_026118556.1</td><td>vWA</td><td>EAD2+VMAP-M0+VMAP-C→ MoxR→ vWA*→ Trypco1→</td><td>546</td><td>Nocardiopsis salina</td><td>GCF_000341025.1</td><td>actinobacteria</td></tr>
<tr><td>WP_026122567.1</td><td>vWA+APATPase+wHTH+TPRs</td><td>Trypsin+VMAP-M0+VMAP-C→ vWA+APATPase+wHTH+TPRs*→ ||← DUF397||Trypco1→</td><td>1269</td><td>Nocardiopsis halotolerans</td><td>GCF_000341065.1</td><td>actinobacteria</td></tr>
<tr><td>WP_026124370.1</td><td>vWA+APATPase+wHTH+TPRs</td><td>Trypsin+VMAP-M0+VMAP-C→ MoxR→ vWA+APATPase+wHTH+TPRs*→ ||← DUF397||Trypco1→</td><td>1504</td><td>Nocardiopsis baichengensis</td><td>GCF_000341205.1</td><td>actinobacteria</td></tr>
<tr><td>WP_026178210.1</td><td>vWA+APATPase+Trypsin+TIR+FxsC_Cter(partial)</td><td>TIR+EAD2→ EAD2+VMAP-M10+VMAP-C→ MoxR→ vWA+APATPase+Trypsin+TIR+FxsC_Cter(partial)*→</td><td>1265</td><td>Streptomyces hokutonensis</td><td>GCF_000376565.1</td><td>actinobacteria</td></tr>
<tr><td>WP_026206227.1</td><td>vWA+CASPASE</td><td>AB_hydrolase→ CASPASE+VMAP-M2+VMAP-C→ MoxR→ vWA+CASPASE*→</td><td>923</td><td>Actinoplanes globisporus</td><td>GCF_000379645.1</td><td>actinobacteria</td></tr>
<tr><td>WP_026211906.1</td><td>vWA+TPRs+CASPASE</td><td>Trypsin+HDxxH+VMAP-M0+VMAP-C→ MoxR→ vWA+TPRs+CASPASE*→</td><td>1257</td><td>Longispora albida</td><td>GCF_000379825.1</td><td>actinobacteria</td></tr>
<tr><td>WP_026217851.1</td><td>vWA+BetaPropeller</td><td>CASPASE+VMAP-M19→ VMAP-C→ MoxR→ vWA+BetaPropeller*→</td><td>924</td><td>Streptomyces scabrisporus</td><td>GCF_000372745.1</td><td>actinobacteria</td></tr>
<tr><td>WP_026249297.1</td><td>vWA+TPRs</td><td>TCAD15||Trypco1→ Trypsin+HDxxH+VMAP-M0+VMAP-C→ MoxR→ vWA+TPRs*→ FXSXX-COOH→ Radical_SAM→ METALLOPROTEASE+TPRs→ TIR+APATPase+TPRs→ Trypco1→</td><td>1137</td><td>Streptomyces sp. ATexAB-D23</td><td>GCF_000373645.1</td><td>actinobacteria</td></tr>
<tr><td>WP_026276898.1</td><td>vWA+TPRs</td><td>TCAD15||Trypco1→ Trypsin+HDxxH+VMAP-M0+VMAP-C→ MoxR→ vWA+TPRs*→ FXSXX-COOH→ Radical_SAM→ TIR+APATPase+TPRs→</td><td>1072</td><td>Streptomyces sp. CNS335</td><td>GCF_000377125.1</td><td>actinobacteria</td></tr>
<tr><td>WP_026401298.1</td><td>vWA+APATPase+wHTH+TPRs</td><td>Radical_SAM→ METALLOPROTEASE+TPRs→ ||← TIR+APATPase+TPRs||?→ ?→ EAD2+VMAP-M0+VMAP-C→ MoxR→ vWA+APATPase+wHTH+TPRs*→ || FXSXX-COOH-like||cNMP_cyclase+EAD2→</td><td>1399</td><td>Actinomadura rifamycini</td><td>GCF_000425065.1</td><td>actinobacteria</td></tr>
<tr><td>WP_026401380.1</td><td>vWA+FtsK+FtsK+cNMP_cyclase+FtsK</td><td>Trypco1→ Trypsin+VMAP-M8+VMAP-C→ MoxR→ vWA+FtsK+FtsK+cNMP_cyclase+FtsK*→</td><td>1694</td><td>Actinomadura rifamycini</td><td>GCF_000425065.1</td><td>actinobacteria</td></tr>
<tr><td>WP_026418515.1</td><td>vWA+TPRs</td><td>Trypco1→ Trypsin+HDxxH+VMAP-M0+VMAP-C→ MoxR→ vWA+TPRs*→ FXSXX-COOH-like→ Radical_SAM→ METALLOPROTEASE+TPRs→ TIR+APATPase+TPRs→</td><td>874</td><td>Actinoalloteichus cyanogriseus</td><td>GCA_002895405.1</td><td>actinobacteria</td></tr>
<tr><td>WP_026423710.1</td><td>vWA+Trypsin+TCAD11</td><td>CASPASE+VMAP-M2+VMAP-C→ MoxR→ vWA+Trypsin+TCAD11*→</td><td>907</td><td>Actinokineospora inagensis</td><td>GCF_000482865.1</td><td>actinobacteria</td></tr>
<tr><td>WP_026468136.1</td><td>vWA+APATPase+wHTH</td><td>vWA+APATPase+wHTH*→</td><td>859</td><td>Amycolatopsis balhimycina</td><td>GCA_003947255.1</td><td>actinobacteria</td></tr>
<tr><td>WP_026468526.1</td><td>vWA+APATPase+wHTH+TPRs</td><td>vWA+APATPase+wHTH+TPRs→ ||← METALLOPROTEASE+TPRs||cNMP_cyclase+EAD2→ EAD2+VMAP-M0+VMAP-C→ MoxR→ vWA+APATPase+wHTH+TPRs*→ || METALLOPROTEASE+TPRs</td><td>1428</td><td>Amycolatopsis balhimycina</td><td>GCA_003947255.1</td><td>actinobacteria</td></tr>
<tr><td>WP_026468531.1</td><td>vWA+APATPase+wHTH+TPRs</td><td>-</td><td>1231</td><td>Amycolatopsis balhimycina</td><td>-</td><td>actinobacteria</td></tr>
</table>

<table>
<tr><th>acc</th><th>architecture</th><th>operon</th><th>len</th><th>species</th><th>gca</th><th>taxend</th></tr>
<tr><td>WP_027140710.1</td><td>vWA+APATPase+wHTH+TPRs</td><td>-</td><td>1281</td><td>Frankia sp. CN3</td><td>-</td><td>actinobacteria</td></tr>
<tr><td>WP_027344130.1</td><td>vWA+Trypsin+APATPase+wHTH+TPRs</td><td>Trypco1→ Trypsin+VMAP-M8+VMAP-C→ MoxR→ vWA+Trypsin+APATPase+wHTH+TPRs*→</td><td>1497</td><td>Hamadaea tsunoensis</td><td>GCF_000428945.1</td><td>actinobacteria</td></tr>
<tr><td>WP_027562298.1</td><td>vWA+CASPASE+TPRs</td><td>AB_hydrolase→ CASPASE+VMAP-M4+VMAP-C→ MoxR→ vWA+CASPASE+TPRs*→</td><td>1256</td><td>Bradyrhizobium</td><td>GCF_000472685.1</td><td>alphaproteobacteria</td></tr>
<tr><td>WP_027742287.1</td><td>vWA+TPRs</td><td>TCAD15||Trypco1→ Trypsin+HDxxH+VMAP-M0+VMAP-C→ MoxR→ vWA+TPRs*→ FXSXX-COOH→ Radical_SAM→ TIR+APATPase+TPRs→</td><td>1072</td><td>Streptomyces sp. CNT371</td><td>GCF_000514995.1</td><td>actinobacteria</td></tr>
<tr><td>WP_027753922.1</td><td>vWA+TPRs</td><td>TCAD15||Trypco1→ Trypsin+HDxxH+VMAP-M0+VMAP-C→ MoxR→ vWA+TPRs*→ FXSXX-COOH→ Radical_SAM→ TIR+APATPase+TPRs→</td><td>1105</td><td>Streptomyces sp. CNH099</td><td>GCF_000514715.1</td><td>actinobacteria</td></tr>
<tr><td>WP_027759022.1</td><td>vWA+TPRs</td><td>Trypco1→ Trypsin+HDxxH+VMAP-M0+VMAP-C→ MoxR→ vWA+TPRs*→ FXSXX-COOH-like→ METALLOPROTEASE+TPRs→ TIR+APATPase+TPRs→</td><td>1069</td><td>Streptomyces sp. CNT318</td><td>GCF_000424765.1</td><td>actinobacteria</td></tr>
<tr><td>WP_027765362.1</td><td>vWA+TPRs</td><td>AB_hydrolase→ CASPASE+EAD2+VMAP-M0+VMAP-C→ MoxR→ vWA+TPRs*→</td><td>1182</td><td>Streptomyces sp. CNS606</td><td>GCF_000426325.1</td><td>actinobacteria</td></tr>
<tr><td>WP_027766480.1</td><td>vWA+TPRs</td><td>TCAD15||Trypco1→ Trypsin+HDxxH+VMAP-M0+VMAP-C→ MoxR→ vWA+TPRs*→ FXSXX-COOH→ Radical_SAM→ TIR+APATPase+TPRs→</td><td>1072</td><td>Streptomyces sp. CNQ865</td><td>GCF_000424805.1</td><td>actinobacteria</td></tr>
<tr><td>WP_027772933.1</td><td>vWA+TPRs</td><td>TCAD15||Trypco1→ Trypsin+HDxxH+VMAP-M0+VMAP-C→ MoxR→ vWA+TPRs*→ FXSXX-COOH→ Radical_SAM→ TIR+APATPase+TPRs→</td><td>1073</td><td>Streptomyces sp. CNQ329</td><td>GCF_000482585.1</td><td>actinobacteria</td></tr>
<tr><td>WP_028442480.1</td><td>vWA+APATPase+wHTH+TPRs</td><td>EAD2+VMAP-M0+VMAP-C→ MoxR→ vWA+APATPase+wHTH+TPRs*→ AB_hydrolase→ CASPASE+EAD2→ FXSXX-COOH-like→ METALLOPROTEASE+TPRs→</td><td>1457</td><td>Streptomyces sp. DpondAA-B6</td><td>GCA_900091745.1</td><td>actinobacteria</td></tr>
<tr><td>WP_028806360.1</td><td>vWA+cNMP_cyclase+Band7+HAD</td><td>Trypco1→ Trypsin+VMAP-M0+VMAP-C→ MoxR→ vWA+cNMP_cyclase+Band7+HAD*→</td><td>1217</td><td>Streptomyces sp. 303MFCol5.2</td><td>GCF_000383635.1</td><td>actinobacteria</td></tr>
<tr><td>WP_028806989.1</td><td>vWA+TPRs</td><td>TCAD15||Trypco1→ Trypsin+HDxxH+VMAP-M0+VMAP-C→ MoxR→ vWA+TPRs*→ FXSXX-COOH→ Radical_SAM→ METALLOPROTEASE+TPRs→ TIR+APATPase+TPRs→</td><td>1123</td><td>Streptomyces sp. 303MFCol5.2</td><td>GCF_000383635.1</td><td>actinobacteria</td></tr>
<tr><td>WP_028807049.1</td><td>vWA+Pkinase+ParA-Soj-PloopNTPase(SIMIBI)</td><td>DUF397→ ||← ?||Trypsin+EAD2+VMAP-M0+VMAP-C→ MoxR→ vWA+Pkinase+ParA-Soj-PloopNTPase(SIMIBI)*→</td><td>1117</td><td>Streptomyces sp. 303MFCol5.2</td><td>GCF_000383635.1</td><td>actinobacteria</td></tr>
<tr><td>WP_028808563.1</td><td>vWA+TPRs</td><td>Trypco1→ Trypsin+EAD2+VMAP-M0+VMAP-C→ MoxR→ vWA+TPRs*→</td><td>880</td><td>Streptomyces canus</td><td>GCF_000383615.1</td><td>actinobacteria</td></tr>
<tr><td>WP_028811696.1</td><td>vWA+TPRs</td><td>TCAD15||Trypco1→ Trypsin+HDxxH+VMAP-M0+VMAP-C→ MoxR→ vWA+TPRs*→ FXSXX-COOH→ METALLOPROTEASE+TPRs→ TIR+APATPase+TPRs→</td><td>1059</td><td>Streptomyces flavidovirens</td><td>GCF_000429085.1</td><td>actinobacteria</td></tr>
<tr><td>WP_030017200.1</td><td>vWA+TPRs</td><td>TCAD15||Trypco1→ Trypsin+HDxxH+VMAP-M0+VMAP-C→ MoxR→ vWA+TPRs*→ FXSXX-COOH→ Radical_SAM→ TIR+APATPase+TPRs→</td><td>1102</td><td>Streptomyces monomycini</td><td>GCF_000715845.1</td><td>actinobacteria</td></tr>
<tr><td>WP_030018283.1</td><td>vWA+wHTH+TPRs+wHTH</td><td>Trypco1→ Trypsin+EAD2+VMAP-M0+VMAP-C→ MoxR→ vWA+wHTH+TPRs+wHTH*→ ?→ ||← AB_hydrolase</td><td>1089</td><td>Streptomyces monomycini</td><td>-</td><td>actinobacteria</td></tr>
<tr><td>WP_030076156.1</td><td>vWA+APATPase+wHTH+TPRs</td><td>EAD2+VMAP-M0+VMAP-C→ MoxR→ vWA+APATPase+wHTH+TPRs*→ AB_hydrolase→ CASPASE+EAD2→ PSE→ METALLOPROTEASE+TPRs→</td><td>1480</td><td>Streptomyces baarnensis</td><td>GCF_000716025.1</td><td>actinobacteria</td></tr>
<tr><td>WP_030165950.1</td><td>vWA+APATPase+wHTH+TPRs</td><td>Trypsin+EAD2→ EAD2+VMAP-M0+VMAP-C→ MoxR→ vWA+APATPase+wHTH+TPRs*→ ||← METALLOPROTEASE+TPRs← FXSXX-COOH-like</td><td>1461</td><td>Streptomyces sp. NRRL S-813</td><td>GCF_000718945.1</td><td>actinobacteria</td></tr>
<tr><td>WP_030179051.1</td><td>vWA+wHTH+TPRs+wHTH+TPRs+APATPase+wHTH+TPRs</td><td>Trypco1→ Trypsin+EAD2+VMAP-M0+VMAP-C→ MoxR→ vWA+wHTH+TPRs+wHTH+TPRs+APATPase+wHTH+TPRs*→ ||← AB_hydrolase</td><td>1732</td><td>Streptomyces</td><td>GCA_000707945.1</td><td>actinobacteria</td></tr>
<tr><td>WP_030182699.1</td><td>vWA+TPRs</td><td>TCAD15||Trypco1→ Trypsin+HDxxH+VMAP-M0+VMAP-C→ MoxR→ vWA+TPRs*→ FXSXX-COOH→ Radical_SAM→ TIR+APATPase+TPRs→</td><td>1102</td><td>Streptomyces</td><td>GCA_001270665.1</td><td>actinobacteria</td></tr>
<tr><td>WP_030190028.1</td><td>vWA+TPRs</td><td>TCAD15→ Trypco1→ Trypsin+HDxxH+VMAP-M0+VMAP-C→ MoxR→ vWA+TPRs*→ FXSXX-COOH-like→ METALLOPROTEASE+TPRs→ TIR+APATPase+TPRs→</td><td>1097</td><td>Streptomyces violaceorubidus</td><td>GCF_000717995.1</td><td>actinobacteria</td></tr>
<tr><td>WP_030203313.1</td><td>vWA+Trypsin+cNMP_cyclase+TPRs</td><td>AB_hydrolase→ CASPASE+VMAP-M0+VMAP-C→ MoxR→ vWA+Trypsin+cNMP_cyclase+TPRs*→</td><td>1279</td><td>Streptomyces sp. NRRL S-87</td><td>GCF_000719335.1</td><td>actinobacteria</td></tr>
<tr><td>WP_030212926.1</td><td>vWA+APATPase+wHTH+TPRs</td><td>EAD2+VMAP-M0+VMAP-C→ MoxR→ vWA+APATPase+wHTH+TPRs*→ FXSXX-COOH-like→ METALLOPROTEASE+TPRs→</td><td>1423</td><td>Streptomyces sp. NRRL WC-3626</td><td>GCF_000718785.1</td><td>actinobacteria</td></tr>
<tr><td>WP_030244400.1</td><td>vWA+APATPase+wHTH+TPRs</td><td>EAD2+VMAP-M0+VMAP-C→ MoxR→ vWA+APATPase+wHTH+TPRs*→ ||← Na_H_Exchanger||AB_hydrolase→ CASPASE+EAD2→ FXSXX-COOH-like→ METALLOPROTEASE+TPRs→</td><td>1519</td><td>Streptomyces sp. NRRL S-455</td><td>GCF_000717875.1</td><td>actinobacteria</td></tr>
<tr><td>WP_030306272.1</td><td>vWA+TPRs</td><td>MoxR→ vWA+TPRs*→ FXSXX-COOH→ Radical_SAM→ METALLOPROTEASE+TPRs→ TIR+APATPase+TPRs→</td><td>1137</td><td>Streptomyces</td><td>GCA_001509565.1</td><td>actinobacteria</td></tr>
<tr><td>WP_030328546.1</td><td>vWA+APATPase+wHTH+TPRs</td><td>CASPASE+TPRs→ EAD2+VMAP-M0+VMAP-C→ MoxR→ vWA+APATPase+wHTH+TPRs*→ ||← METALLOPROTEASE+TPRs← Radical_SAM← FXSXX-COOH-like</td><td>1417</td><td>Micromonospora parva</td><td>GCF_000718515.1</td><td>actinobacteria</td></tr>
<tr><td>WP_030355655.1</td><td>vWA+TPRs</td><td>TCAD15||Trypco1→ Trypsin+HDxxH+VMAP-M0+VMAP-C→ MoxR→ vWA+TPRs*→ FXSXX-COOH→ Radical_SAM→ METALLOPROTEASE+TPRs→ TIR+APATPase+TPRs→</td><td>1131</td><td>Streptomyces scopuliridis</td><td>GCA_003073355.1</td><td>actinobacteria</td></tr>
<tr><td>WP_030373066.1</td><td>vWA+wHTH+TPRs+wHTH+TPRs+APATPase+wHTH+TPRs</td><td>Trypco1→ Trypsin+EAD2+VMAP-M0+VMAP-C→ MoxR→ vWA+wHTH+TPRs+wHTH+TPRs+APATPase+wHTH+TPRs*→ ||← AB_hydrolase</td><td>1750</td><td>Streptomyces rimosus</td><td>GCF_000717815.1</td><td>actinobacteria</td></tr>
<tr><td>WP_030375332.1</td><td>vWA+TPRs</td><td>FXSXX-COOH-like||Trypco1→ Trypsin+HDxxH+VMAP-M0+VMAP-C→ MoxR→ vWA+TPRs*→ FXSXX-COOH→ Radical_SAM→ TIR+APATPase+TPRs→</td><td>1102</td><td>Streptomyces rimosus</td><td>GCF_000717815.1</td><td>actinobacteria</td></tr>
<tr><td>WP_030389769.1</td><td>vWA+Trypsin+cNMP_cyclase+CCCH(TrebleClef)+Pentapeptide_repeats</td><td>AB_hydrolase→ CASPASE+VMAP-M0+VMAP-C→ MoxR→ vWA+Trypsin+cNMP_cyclase+CCCH(TrebleClef)+Pentapeptide_repeats*→</td><td>1196</td><td>Streptomyces sp. NRRL S-241</td><td>GCF_000717925.1</td><td>actinobacteria</td></tr>
<tr><td>WP_030393995.1</td><td>vWA+APATPase+SWACOS+wHTH+TPRs+Beta-rich-tail</td><td>Trypsin+EAD2→ EAD2+VMAP-M10+VMAP-C→ MoxR→ vWA+APATPase+SWACOS+wHTH+TPRs+Beta-rich-tail*→</td><td>1580</td><td>Streptomyces purpeofuscus</td><td>GCF_000718025.1</td><td>actinobacteria</td></tr>
<tr><td>WP_030464470.1</td><td>vWA+CASPASE+TCAD14+PIN+CCCH(ZNR)+FHA</td><td>MoxR→ vWA+CASPASE+TCAD14+PIN+CCCH(ZNR)+FHA*→</td><td>1330</td><td>Kitasatospora sp. NRRL B-11411</td><td>GCF_000721345.1</td><td>actinobacteria</td></tr>
<tr><td>WP_030470827.1</td><td>vWA+APATPase+wHTH+TPRs</td><td>cNMP_cyclase+VMAP-M0+VMAP-C→ MoxR→ vWA+APATPase+wHTH+TPRs*→ ||← METALLOPROTEASE+TPRs← FXSXX-COOH-like||TIR+APATPase+TPRs→ ||← DUF397</td><td>1157</td><td>Lechevalieria aerocolonigenes</td><td>GCF_000719205.1</td><td>actinobacteria</td></tr>
<tr><td>WP_030553426.1</td><td>vWA+TPRs</td><td>TCAD15||Trypco1→ Trypsin+HDxxH+VMAP-M0+VMAP-C→ MoxR→ vWA+TPRs*→ FXSXX-COOH→ METALLOPROTEASE+TPRs→ TIR+APATPase+TPRs→</td><td>1076</td><td>Streptomyces exfoliatus</td><td>GCF_000725545.1</td><td>actinobacteria</td></tr>
<tr><td>WP_030574529.1</td><td>vWA+TPRs</td><td>TCAD15→ Trypco1→ Trypsin+HDxxH+VMAP-M0+VMAP-C→ MoxR→ vWA+TPRs*→ FXSXX-COOH-like→ METALLOPROTEASE+TPRs→ TIR+APATPase+TPRs→</td><td>1068</td><td>Streptomyces sclerotialus</td><td>-</td><td>actinobacteria</td></tr>
<tr><td>WP_030592893.1</td><td>vWA+wHTH+TPRs+wHTH+TPRs+APATPase+wHTH+TPRs</td><td>Trypco1→ Trypsin+EAD2+VMAP-M0+VMAP-C→ MoxR→ vWA+wHTH+TPRs+wHTH+TPRs+APATPase+wHTH+TPRs*→ ||← AB_hydrolase</td><td>1732</td><td>Streptomyces</td><td>GCA_001270585.1</td><td>actinobacteria</td></tr>
<tr><td>WP_030604157.1</td><td>vWA+TPRs</td><td>TCAD15||Trypco1→ Trypsin+HDxxH+VMAP-M0+VMAP-C→ MoxR→ vWA+TPRs*→ FXSXX-COOH→ Radical_SAM→ TIR+APATPase+TPRs→</td><td>1102</td><td>Streptomyces</td><td>GCA_001270585.1</td><td>actinobacteria</td></tr>
<tr><td>WP_030623484.1</td><td>vWA+APATPase+wHTH+TPRs</td><td>EAD2+VMAP-M0+VMAP-C→ MoxR→ vWA+APATPase+wHTH+TPRs*→ AB_hydrolase→ CASPASE+EAD2→ PSE→ METALLOPROTEASE+TPRs→</td><td>1478</td><td>Streptomyces albus</td><td>GCF_000720915.1</td><td>actinobacteria</td></tr>
<tr><td>WP_030628104.1</td><td>vWA+TPRs</td><td>TCAD15||Trypco1→ Trypsin+HDxxH+VMAP-M0+VMAP-C→ MoxR→ vWA+TPRs*→ FXSXX-COOH→ Radical_SAM→ TIR+APATPase+TPRs→</td><td>1102</td><td>Streptomyces</td><td>GCF_000716455.1</td><td>actinobacteria</td></tr>
<tr><td>WP_030630372.1</td><td>vWA+wHTH+TPRs+wHTH+TPRs+APATPase+wHTH+TPRs</td><td>Trypco1→ Trypsin+EAD2+VMAP-M0+VMAP-C→ MoxR→ vWA+wHTH+TPRs+wHTH+TPRs+APATPase+wHTH+TPRs*→ ||← AB_hydrolase</td><td>1750</td><td>Streptomyces rimosus</td><td>GCF_000720595.1</td><td>actinobacteria</td></tr>
<tr><td>WP_030632509.1</td><td>vWA+APATPase+wHTH+TPRs</td><td>EAD2+VMAP-M0+VMAP-C→ MoxR→ vWA+APATPase+wHTH+TPRs*→ AB_hydrolase→ CASPASE+EAD2→ FXSXX-COOH-like→ METALLOPROTEASE+TPRs→</td><td>1457</td><td>Streptomyces</td><td>GCF_000720535.1</td><td>actinobacteria</td></tr>
<tr><td>WP_030647823.1</td><td>vWA+wHTH+TPRs+wHTH+TPRs+APATPase+wHTH+TPRs</td><td>Trypco1→ Trypsin+EAD2+VMAP-M0+VMAP-C→ MoxR→ vWA+wHTH+TPRs+wHTH+TPRs+APATPase+wHTH+TPRs*→ ||← AB_hydrolase</td><td>1734</td><td>Streptomyces rimosus</td><td>GCF_000718675.1</td><td>actinobacteria</td></tr>
<tr><td>WP_030652329.1</td><td>vWA+Pkinase+cNMP_cyclase+Band7+HAD</td><td>Trypco1→ Trypsin+VMAP-M0+VMAP-C→ MoxR→ vWA+Pkinase+cNMP_cyclase+Band7+HAD*→</td><td>1313</td><td>Streptomyces</td><td>GCF_000716535.1</td><td>actinobacteria</td></tr>
<tr><td>WP_030654968.1</td><td>vWA+TPRs</td><td>TCAD15||Trypco1→ Trypsin+HDxxH+VMAP-M0+VMAP-C→ MoxR→ vWA+TPRs*→ FXSXX-COOH→ Radical_SAM→ TIR+APATPase+TPRs→</td><td>1102</td><td>Streptomyces rimosus</td><td>GCF_000718675.1</td><td>actinobacteria</td></tr>
<tr><td>WP_030656871.1</td><td>vWA+Trypsin+cNMP_cyclase+CCCH(TrebleClef)+Pentapeptide_repeats</td><td>AB_hydrolase→ CASPASE+VMAP-M0+VMAP-C→ MoxR→ vWA+Trypsin+cNMP_cyclase+CCCH(TrebleClef)+Pentapeptide_repeats*→</td><td>1198</td><td>Streptomyces</td><td>GCF_000718705.1</td><td>actinobacteria</td></tr>
<tr><td>WP_030659385.1</td><td>vWA+CASPASE+TCAD12</td><td>AB_hydrolase→ CASPASE+VMAP-M2+VMAP-C→ MoxR→ vWA+CASPASE+TCAD12*→</td><td>1107</td><td>Streptomyces</td><td>-</td><td>actinobacteria</td></tr>
<tr><td>WP_030660077.1</td><td>vWA+Pkinase+ParA-Soj-PloopNTPase(SIMIBI)</td><td>Trypco1→ Trypsin+EAD2+VMAP-M0+VMAP-C→ MoxR→ vWA+Pkinase+ParA-Soj-PloopNTPase(SIMIBI)*→ ?→ NUDIX→</td><td>1127</td><td>Streptomyces rimosus</td><td>GCF_000720725.1</td><td>actinobacteria</td></tr>
</table>

<table>
<tr><th>acc</th><th>architecture</th><th>operon</th><th>len</th><th>species</th><th>gca</th><th>taxend</th></tr>
<tr><td>WP_030663654.1</td><td>vWA+APATPase+wHTH+TPRs</td><td>EAD2+VMAP-M0+VMAP-C→ MoxR→ vWA+APATPase+wHTH+TPRs*→ ?→ ||← Na_H_Exchanger||AB_hydrolase→ CASPASE+EAD2→ FXSXX-COOH-like→</td><td>1542</td><td>Streptomyces cellulosae</td><td>GCF_000721105.1</td><td>actinobacteria</td></tr>
<tr><td>WP_030667973.1</td><td>vWA+wHTH+TPRs+wHTH+TPRs+APATPase+wHTH+TPRs</td><td>vWA+wHTH+TPRs+wHTH+TPRs+APATPase+wHTH+TPRs*→ ||← AB_hydrolase</td><td>1616</td><td>Streptomyces rimosus</td><td>-</td><td>actinobacteria</td></tr>
<tr><td>WP_030672371.1</td><td>vWA+TPRs</td><td>TCAD15||Trypco1→ Trypsin+HDxxH+VMAP-M0+VMAP-C→ MoxR→ vWA+TPRs*→ FXSXX-COOH→ Radical_SAM→ TIR+APATPase+TPRs→</td><td>1102</td><td>Streptomyces rimosus</td><td>GCF_000720725.1</td><td>actinobacteria</td></tr>
<tr><td>WP_030695576.1</td><td>vWA+TPRs</td><td>TCAD15||Trypco1→ Trypsin+HDxxH+VMAP-M0+VMAP-C→ MoxR→ vWA+TPRs*→ FXSXX-COOH→ ||← ?||Radical_SAM→ METALLOPROTEASE+TPRs→ TIR+APATPase+TPRs→</td><td>1138</td><td>Streptomyces griseus</td><td>GCF_000719495.1</td><td>actinobacteria</td></tr>
<tr><td>WP_030705577.1</td><td>vWA_protein</td><td>AB_hydrolase→ CASPASE+VMAP-M2+VMAP-C→ MoxR→ vWA_protein*→ TIR+FxsC_Cter→</td><td>535</td><td>Streptomyces griseus</td><td>GCF_000717795.1</td><td>actinobacteria</td></tr>
<tr><td>WP_030711849.1</td><td>vWA+APATPase+wHTH+TPRs</td><td>EAD2+VMAP-M0+VMAP-C→ MoxR→ vWA+APATPase+wHTH+TPRs*→ AB_hydrolase→ CASPASE+EAD2→ FXSXX-COOH-like→ METALLOPROTEASE+TPRs→</td><td>1463</td><td>Streptomyces griseus</td><td>GCF_000719355.1</td><td>actinobacteria</td></tr>
<tr><td>WP_030715041.1</td><td>vWA+Trypsin+cNMP_cyclase+CCCH(TrebleClef)+Pentapeptide_repeats</td><td>AB_hydrolase→ CASPASE+VMAP-M0+VMAP-C→ MoxR→ vWA+Trypsin+cNMP_cyclase+CCCH(TrebleClef)+Pentapeptide_repeats*→</td><td>1196</td><td>Streptomyces sp. NRRL S-237</td><td>GCF_000718935.1</td><td>actinobacteria</td></tr>
<tr><td>WP_030715500.1</td><td>vWA+Trypsin+cNMP_cyclase+CCCH(TrebleClef)+Pentapeptide_repeats</td><td>AB_hydrolase→ CASPASE+VMAP-M0+VMAP-C→ MoxR→ vWA+Trypsin+cNMP_cyclase+CCCH(TrebleClef)+Pentapeptide_repeats*→</td><td>1217</td><td>Streptomyces sp. NRRL F-2580</td><td>GCF_000720115.1</td><td>actinobacteria</td></tr>
<tr><td>WP_030754069.1</td><td>vWA+TPRs</td><td>TCAD15||Trypco1→ Trypsin+HDxxH+VMAP-M0+VMAP-C→ ?→ MoxR→ vWA+TPRs*→ FXSXX-COOH→ Radical_SAM→ METALLOPROTEASE+TPRs→ TIR+APATPase+TPRs→</td><td>1146</td><td>Streptomyces sp. NRRL F-5135</td><td>GCF_000719775.1</td><td>actinobacteria</td></tr>
<tr><td>WP_030765391.1</td><td>vWA+TPRs</td><td>TCAD15||Trypco1→ Trypsin+HDxxH+VMAP-M0+VMAP-C→ MoxR→ vWA+TPRs*→ FXSXX-COOH→ Radical_SAM→ METALLOPROTEASE+TPRs→ TIR+APATPase+TPRs→</td><td>1137</td><td>Streptomyces griseus</td><td>GCF_000719435.1</td><td>actinobacteria</td></tr>
<tr><td>WP_030766032.1</td><td>vWA+Trypsin+cNMP_cyclase+CCCH(TrebleClef)+Pentapeptide_repeats</td><td>AB_hydrolase→ CASPASE+VMAP-M0+VMAP-C→ MoxR→ vWA+Trypsin+cNMP_cyclase+CCCH(TrebleClef)+Pentapeptide_repeats*→</td><td>1192</td><td>Streptomyces sp. NRRL F-2664</td><td>GCF_000719345.1</td><td>actinobacteria</td></tr>
<tr><td>WP_030766469.1</td><td>vWA+Trypsin+cNMP_cyclase+CCCH(TrebleClef)+Pentapeptide_repeats</td><td>AB_hydrolase→ CASPASE+VMAP-M0+VMAP-C→ MoxR→ vWA+Trypsin+cNMP_cyclase+CCCH(TrebleClef)+Pentapeptide_repeats*→</td><td>1198</td><td>Streptomyces</td><td>GCF_000720795.1</td><td>actinobacteria</td></tr>
<tr><td>WP_030799596.1</td><td>vWA+wHTH+TPRs+wHTH+TPRs</td><td>Trypsin+EAD2+VMAP-M0+VMAP-C→ MoxR→ vWA+wHTH+TPRs+wHTH+TPRs*→ ||← AB_hydrolase||?→ ||← PSE||?→ ||← ?← PSE||?→ ?→ ||← DUF397</td><td>1377</td><td>Streptomyces sp. NRRL S-337</td><td>-</td><td>actinobacteria</td></tr>
<tr><td>WP_030816755.1</td><td>vWA+TPRs</td><td>Trypsin+HDxxH+VMAP-M0+VMAP-C→ MoxR→ vWA+TPRs*→ FXSXX-COOH→ Radical_SAM→ METALLOPROTEASE+TPRs→ TIR+APATPase+TPRs→</td><td>1096</td><td>Streptomyces sp. NRRL F-2799</td><td>GCF_000719895.1</td><td>actinobacteria</td></tr>
<tr><td>WP_030821754.1</td><td>vWA+CASPASE+TCAD12</td><td>AB_hydrolase→ CASPASE+VMAP-M2+VMAP-C→ MoxR→ vWA+CASPASE+TCAD12*→</td><td>1107</td><td>Streptomyces</td><td>GCA_000966965.1</td><td>actinobacteria</td></tr>
<tr><td>WP_030825272.1</td><td>vWA+APATPase+wHTH+TPRs</td><td>PNP_UDP_1+EAD2→ EAD2+VMAP-M0+VMAP-C→ MoxR→ vWA+APATPase+wHTH+TPRs*→ ||← METALLOPROTEASE+TPRs</td><td>1468</td><td>Streptomyces hygroscopicus</td><td>GCF_000721535.1</td><td>actinobacteria</td></tr>
<tr><td>WP_030831121.1</td><td>vWA+APATPase+wHTH+TPRs</td><td>Trypsin+EAD2→ EAD2+VMAP-M0+VMAP-C→ MoxR→ vWA+APATPase+wHTH+TPRs*→ ||← METALLOPROTEASE+TPRs← Radical_SAM← FXSXX-COOH||Voltage_CLC→</td><td>1517</td><td>Streptomyces</td><td>GCA_001279005.1</td><td>actinobacteria</td></tr>
<tr><td>WP_030836701.1</td><td>vWA+cNMP_cyclase+Band7+HAD</td><td>Trypco1→ Trypsin+VMAP-M0+VMAP-C→ MoxR→ vWA+cNMP_cyclase+Band7+HAD*→</td><td>1089</td><td>Streptomyces sp. NRRL S-475</td><td>GCF_000720215.1</td><td>actinobacteria</td></tr>
<tr><td>WP_030853861.1</td><td>vWA+Trypsin+cNMP_cyclase+CCCH(TrebleClef)+Pentapeptide_repeats</td><td>AB_hydrolase→ CASPASE+VMAP-M0+VMAP-C→ MoxR→ vWA+Trypsin+cNMP_cyclase+CCCH(TrebleClef)+Pentapeptide_repeats*→</td><td>1193</td><td>Streptomyces sp. NRRL F-4474</td><td>GCF_000719855.1</td><td>actinobacteria</td></tr>
<tr><td>WP_030857533.1</td><td>vWA+APATPase+wHTH+TPRs</td><td>EAD2+VMAP-M0+VMAP-C→ MoxR→ vWA+APATPase+wHTH+TPRs*→ ||← Na_H_Exchanger← ?||METALLOPROTEASE+TPRs→</td><td>1537</td><td>Streptomyces sp. NRRL S-37</td><td>GCF_000721605.1</td><td>actinobacteria</td></tr>
<tr><td>WP_030863838.1</td><td>vWA+Pkinase+wHTH+TPRs+cNMP_cyclase</td><td>Trypco1→ Trypsin+VMAP-M0+VMAP-C→ MoxR→ vWA+Pkinase+wHTH+TPRs+cNMP_cyclase*→</td><td>1352</td><td>Streptomyces violaceoruber</td><td>GCF_000717845.1</td><td>actinobacteria</td></tr>
<tr><td>WP_030873012.1</td><td>vWA+TPRs</td><td>TCAD15||Trypco1→ Trypsin+HDxxH+VMAP-M0+VMAP-C→ MoxR→ vWA+TPRs*→ FXSXX-COOH→ Radical_SAM→ METALLOPROTEASE+TPRs→ TIR+APATPase+TPRs→ Trypco1→</td><td>1096</td><td>Streptomyces violaceoruber</td><td>GCF_000717845.1</td><td>actinobacteria</td></tr>
<tr><td>WP_030892008.1</td><td>vWA+TPRs</td><td>TCAD15→ ?→ ?→ PSE→ ?→ MoxR→ vWA+Trypsin+HDxxH+VMAP-M0+VMAP-C→ PSE→ MoxR→ vWA+TPRs*→ FXSXX-COOH→ METALLOPROTEASE+TPRs→ TIR+APATPase+TPRs→</td><td>1189</td><td>Streptomyces sp. NRRL F-5053</td><td>GCF_000721445.1</td><td>actinobacteria</td></tr>
<tr><td>WP_030895433.1</td><td>vWA+APATPase+wHTH+TPRs</td><td>EAD2+VMAP-M0+VMAP-C→ MoxR→ vWA+APATPase+wHTH+TPRs*→ ||← Na_H_Exchanger||AB_hydrolase→ CASPASE+EAD2→ FXSXX-COOH-like→ METALLOPROTEASE+TPRs→</td><td>1519</td><td>Streptomyces sp. NRRL S-474</td><td>GCF_000722015.1</td><td>actinobacteria</td></tr>
<tr><td>WP_030898785.1</td><td>vWA+Trypsin+cNMP_cyclase+CCCH(TrebleClef)+Pentapeptide_repeats</td><td>AB_hydrolase→ CASPASE+VMAP-M0+VMAP-C→ MoxR→ vWA+Trypsin+cNMP_cyclase+CCCH(TrebleClef)+Pentapeptide_repeats*→</td><td>1198</td><td>Streptomyces virginiae</td><td>GCF_000720455.1</td><td>actinobacteria</td></tr>
<tr><td>WP_030899222.1</td><td>vWA+CASPASE+TCAD12</td><td>AB_hydrolase→ CASPASE+VMAP-M2+VMAP-C→ MoxR→ vWA+CASPASE+TCAD12*→</td><td>1107</td><td>Streptomyces virginiae</td><td>-</td><td>actinobacteria</td></tr>
<tr><td>WP_030899562.1</td><td>vWA+TPRs</td><td>TCAD15||Trypco1→ Trypsin+HDxxH+VMAP-M0+VMAP-C→ MoxR→ vWA+TPRs*→ FXSXX-COOH→ Radical_SAM→ METALLOPROTEASE+TPRs→ TIR+APATPase+TPRs→</td><td>1136</td><td>Streptomyces sp. NRRL F-5126</td><td>GCF_000721265.1</td><td>actinobacteria</td></tr>
<tr><td>WP_030940437.1</td><td>vWA+Trypsin+cNMP_cyclase+CCCH(TrebleClef)+Pentapeptide_repeats+HAD</td><td>AB_hydrolase→ CASPASE+VMAP-M0+VMAP-C→ MoxR→ vWA+Trypsin+cNMP_cyclase+CCCH(TrebleClef)+Pentapeptide_repeats+HAD*→</td><td>1589</td><td>Streptomyces violaceoruber</td><td>GCF_000720805.1</td><td>actinobacteria</td></tr>
<tr><td>WP_030944915.1</td><td>vWA+APATPase+wHTH+TPRs</td><td>EAD2+VMAP-M0+VMAP-C→ MoxR→ vWA+APATPase+wHTH+TPRs*→</td><td>1542</td><td>Streptomyces violaceoruber</td><td>GCF_000720805.1</td><td>actinobacteria</td></tr>
<tr><td>WP_030949105.1</td><td>vWA+APATPase+wHTH+TPRs</td><td>vWA+APATPase+wHTH+TPRs*→ KAP_NTPase→</td><td>877</td><td>Streptomyces sp. NRRL S-646</td><td>GCF_000720765.1</td><td>actinobacteria</td></tr>
<tr><td>WP_030969877.1</td><td>vWA+TPRs</td><td>TCAD15||Trypco1→ Trypsin+HDxxH+VMAP-M0+VMAP-C→ MoxR→ vWA+TPRs*→ FXSXX-COOH→ Radical_SAM→ METALLOPROTEASE+TPRs→ TIR+APATPase+TPRs→</td><td>1143</td><td>Streptomyces sp. NRRL S-1824</td><td>-</td><td>actinobacteria</td></tr>
<tr><td>WP_030981804.1</td><td>vWA+TPRs</td><td>TCAD15||Trypco1→ Trypsin+HDxxH+VMAP-M0+VMAP-C→ MoxR→ vWA+TPRs*→ FXSXX-COOH→ Radical_SAM→ TIR+APATPase+TPRs→</td><td>1124</td><td>Streptomyces sp. NRRL S-1813</td><td>GCF_000719605.1</td><td>actinobacteria</td></tr>
<tr><td>WP_030993624.1</td><td>vWA+Pkinase+cNMP_cyclase+Band7+HAD</td><td>Trypco1→ Trypsin+VMAP-M0+VMAP-C→ MoxR→ vWA+Pkinase+cNMP_cyclase+Band7+HAD*→</td><td>1314</td><td>Streptomyces sp. NRRL WC-3744</td><td>GCF_000717895.1</td><td>actinobacteria</td></tr>
<tr><td>WP_030995021.1</td><td>vWA+TPRs</td><td>TCAD15||Trypsin+HDxxH+VMAP-M0+VMAP-C→ MoxR→ vWA+TPRs*→ FXSXX-COOH→ Radical_SAM→ METALLOPROTEASE+TPRs→ TIR+APATPase+TPRs→ Trypco1→</td><td>1265</td><td>Streptomyces sp. NRRL F-5630</td><td>GCF_000719475.1</td><td>actinobacteria</td></tr>
<tr><td>WP_031015549.1</td><td>vWA+APATPase+wHTH+TPRs</td><td>EAD2+VMAP-M0+VMAP-C→ MoxR→ vWA+APATPase+wHTH+TPRs*→ ||← Na_H_Exchanger||AB_hydrolase→ CASPASE+EAD2→ FXSXX-COOH-like→ METALLOPROTEASE+TPRs→</td><td>1548</td><td>Streptomyces sp. NRRL WC-3795</td><td>GCF_000720675.1</td><td>actinobacteria</td></tr>
<tr><td>WP_031025253.1</td><td>vWA+Pkinase+cNMP_cyclase+Band7+HAD</td><td>Trypco1→ Trypsin+VMAP-M0+VMAP-C→ MoxR→ vWA+Pkinase+cNMP_cyclase+Band7+HAD*→</td><td>1313</td><td>Streptomyces sp. NRRL WC-3725</td><td>GCF_000720295.1</td><td>actinobacteria</td></tr>
<tr><td>WP_031039674.1</td><td>vWA+TPRs</td><td>TCAD15||Trypco1→ Trypsin+HDxxH+VMAP-M0+VMAP-C→ MoxR→ vWA+TPRs*→ FXSXX-COOH→ Radical_SAM→ METALLOPROTEASE+TPRs→ TIR+APATPase+TPRs→</td><td>1194</td><td>Streptomyces olivaceus</td><td>-</td><td>actinobacteria</td></tr>
<tr><td>WP_031044453.1</td><td>vWA+TPRs</td><td>TCAD15||Trypco1→ Trypsin+HDxxH+VMAP-M0+VMAP-C→ MoxR→ vWA+TPRs*→ FXSXX-COOH→ Radical_SAM→ METALLOPROTEASE+TPRs→ TIR+APATPase+TPRs→ Trypco1→</td><td>1099</td><td>Streptomyces sp. NRRL F-5650</td><td>GCF_000721365.1</td><td>actinobacteria</td></tr>
<tr><td>WP_031085536.1</td><td>vWA+APATPase+wHTH+TPRs</td><td>EAD2+VMAP-M0+VMAP-C→ MoxR→ vWA+APATPase+wHTH+TPRs*→ PSE→ PSE→ CASPASE+EAD2→ FXSXX-COOH-like→ METALLOPROTEASE+TPRs→</td><td>1451</td><td>Streptomyces sp. NRRL WC-3549</td><td>GCF_000721935.1</td><td>actinobacteria</td></tr>
<tr><td>WP_031096228.1</td><td>vWA+APATPase+wHTH+TPRs</td><td>EAD2+VMAP-M0+VMAP-C→ MoxR→ vWA+APATPase+wHTH+TPRs*→ AB_hydrolase→ CASPASE+EAD2→ FXSXX-COOH-like→ METALLOPROTEASE+TPRs→</td><td>1443</td><td>Streptomyces</td><td>GCF_000722185.1</td><td>actinobacteria</td></tr>
</table>

<table>
<tr><th>acc</th><th>architecture</th><th>operon</th><th>len</th><th>species</th><th>gca</th><th>taxend</th></tr>
<tr><td>WP_031152033.1</td><td>vWA+Trypsin+cNMP_cyclase+CCCH(TrebleClef)+Pentapeptide_repeats</td><td>AB_hydrolase→ CASPASE+VMAP-M0+VMAP-C→ MoxR→ vWA+Trypsin+cNMP_cyclase+CCCH(TrebleClef)+Pentapeptide_repeats*→</td><td>1196</td><td>Streptomyces erythrochromogenes</td><td>GCF_000725555.1</td><td>actinobacteria</td></tr>
<tr><td>WP_031153630.1</td><td>vWA+Trypsin+cNMP_cyclase+CCCH(TrebleClef)+Pentapeptide_repeats</td><td>AB_hydrolase→ CASPASE+VMAP-M0+VMAP-C→ MoxR→ vWA+Trypsin+cNMP_cyclase+CCCH(TrebleClef)+Pentapeptide_repeats*→</td><td>1258</td><td>Streptomyces xanthophaeus</td><td>GCF_000725725.1</td><td>actinobacteria</td></tr>
<tr><td>WP_031177221.1</td><td>vWA+TPRs</td><td>TCAD15||Trypco1→ Trypsin+HDxxH+VMAP-M0+VMAP-C→ MoxR→ vWA+TPRs*→ FXSXX-COOH→ Radical_SAM→ METALLOPROTEASE+TPRs→ TIR+APATPase+TPRs→</td><td>1137</td><td>Streptomyces sp. NRRL B-3253</td><td>GCF_000725635.1</td><td>actinobacteria</td></tr>
<tr><td>WP_031181175.1</td><td>vWA+TPRs</td><td>TCAD15||PSE→ Trypsin+HDxxH+VMAP-M0+VMAP-C→ MoxR→ vWA+TPRs*→ FXSXX-COOH→ Radical_SAM→ METALLOPROTEASE+TPRs→ TIR+APATPase+TPRs→</td><td>1096</td><td>Streptomyces seoulensis</td><td>GCA_004328625.1</td><td>actinobacteria</td></tr>
<tr><td>WP_031184764.1</td><td>vWA+TPRs</td><td>TCAD15||Trypco1→ Trypsin+HDxxH+VMAP-M0+VMAP-C→ MoxR→ vWA+TPRs*→ FXSXX-COOH→ Radical_SAM→ METALLOPROTEASE+TPRs→ TIR+APATPase+TPRs→ Trypco1→</td><td>1098</td><td>Streptomyces sp. NRRL F-5635</td><td>GCF_000725645.1</td><td>actinobacteria</td></tr>
<tr><td>WP_031522422.1</td><td>vWA+TPRs</td><td>TCAD15||Trypsin+HDxxH+VMAP-M0+VMAP-C→ MoxR→ vWA+TPRs*→ FXSXX-COOH→ Radical_SAM→ METALLOPROTEASE+TPRs→ TIR+APATPase+TPRs→</td><td>1114</td><td>Streptomyces sp. NRRL F-5123</td><td>-</td><td>actinobacteria</td></tr>
<tr><td>WP_032771199.1</td><td>vWA+APATPase+wHTH+TPRs</td><td>EAD2+VMAP-M0+VMAP-C→ MoxR→ vWA+APATPase+wHTH+TPRs*→ PSE→ CASPASE+EAD2→ FXSXX-COOH-like→ METALLOPROTEASE+TPRs→</td><td>1463</td><td>Streptomyces filamentosus</td><td>GCF_000156695.2</td><td>actinobacteria</td></tr>
<tr><td>WP_032780816.1</td><td>vWA+Pkinase+ParA-Soj-PloopNTPase(SIMIBI)</td><td>Trypco1→ Trypsin+EAD2+VMAP-M0+VMAP-C→ MoxR→ vWA+Pkinase+ParA-Soj-PloopNTPase(SIMIBI)*→</td><td>1140</td><td>Streptomyces sp. CcalMP-8W</td><td>GCF_000373305.1</td><td>actinobacteria</td></tr>
<tr><td>WP_032798208.1</td><td>vWA+APATPase+wHTH+TPRs</td><td>EAD2+VMAP-M0+VMAP-C→ MoxR→ vWA+APATPase+wHTH+TPRs*→ PSE→ PSE→ CASPASE+EAD2→ FXSXX-COOH-like→</td><td>1463</td><td>Streptomyces filamentosus</td><td>GCF_000156455.1</td><td>actinobacteria</td></tr>
<tr><td>WP_033034317.1</td><td>vWA+wHTH+TPRs+wHTH+TPRs+APATPase+wHTH+TPRs</td><td>Trypco1→ Trypsin+EAD2+VMAP-M0+VMAP-C→ MoxR→ vWA+wHTH+TPRs+wHTH+TPRs+APATPase+wHTH+TPRs*→ ||← AB_hydrolase</td><td>1732</td><td>Streptomyces rimosus</td><td>GCF_000716515.1</td><td>actinobacteria</td></tr>
<tr><td>WP_033209913.1</td><td>vWA+TPRs</td><td>TCAD15||PSE→ Trypsin+HDxxH+VMAP-M0+VMAP-C→ MoxR→ vWA+TPRs*→ FXSXX-COOH→</td><td>1055</td><td>Streptomyces bikiniensis</td><td>-</td><td>actinobacteria</td></tr>
<tr><td>WP_033218020.1</td><td>vWA+Trypsin+cNMP_cyclase+CCCH(TrebleClef)+Pentapeptide_repeats</td><td>AB_hydrolase→ CASPASE+VMAP-M0+VMAP-C→ MoxR→ vWA+Trypsin+cNMP_cyclase+CCCH(TrebleClef)+Pentapeptide_repeats*→</td><td>1192</td><td>Streptomyces virginiae</td><td>-</td><td>actinobacteria</td></tr>
<tr><td>WP_033238117.1</td><td>vWA+TPRs</td><td>TCAD15||Trypco1→ Trypsin+HDxxH+VMAP-M0+VMAP-C→ MoxR→ vWA+TPRs*→ FXSXX-COOH→ Radical_SAM→ METALLOPROTEASE+TPRs→ TIR+APATPase+TPRs→</td><td>1137</td><td>Streptomyces griseus</td><td>GCF_000717375.1</td><td>actinobacteria</td></tr>
<tr><td>WP_033266673.1</td><td>vWA+TPRs</td><td>TCAD15||Trypco1→ Trypsin+HDxxH+VMAP-M0+VMAP-C→ MoxR→ vWA+TPRs*→ FXSXX-COOH→ Radical_SAM→ TIR+APATPase+TPRs→</td><td>1136</td><td>Streptomyces lydicus</td><td>GCF_000717345.1</td><td>actinobacteria</td></tr>
<tr><td>WP_033267593.1</td><td>vWA</td><td>EAD2+VMAP-M0+VMAP-C→ vWA*→</td><td>491</td><td>Streptomyces lydicus</td><td>GCF_001984445.1</td><td>actinobacteria</td></tr>
<tr><td>WP_033303679.1</td><td>vWA+APATPase+wHTH+TPRs</td><td>EAD2+VMAP-M0+VMAP-C→ MoxR→ vWA+APATPase+wHTH+TPRs*→ AB_hydrolase→ CASPASE+EAD2→ FXSXX-COOH-like→ METALLOPROTEASE+TPRs→</td><td>1442</td><td>Streptomyces atroolivaceus</td><td>GCF_000717025.1</td><td>actinobacteria</td></tr>
<tr><td>WP_033306176.1</td><td>vWA+Pkinase+wHTH+TPRs+cNMP_cyclase+Band7+HAD</td><td>Trypco1→ Trypsin+VMAP-M0+VMAP-C→ MoxR→ vWA+Pkinase+wHTH+TPRs+cNMP_cyclase+Band7+HAD*→</td><td>1423</td><td>Streptomyces iakyrus</td><td>GCF_000717055.1</td><td>actinobacteria</td></tr>
<tr><td>WP_033433374.1</td><td>vWA+APATPase+wHTH+TPRs</td><td>vWA+APATPase+wHTH+TPRs→ ||← METALLOPROTEASE+TPRs← FXSXX-COOH-like||?→ ||← ?||EAD2+VMAP-M0+VMAP-C→ MoxR→ vWA+APATPase+wHTH+TPRs*→ || METALLOPROTEASE+TPRs</td><td>1343</td><td>Saccharothrix syringae</td><td>GCF_000716755.1</td><td>actinobacteria</td></tr>
<tr><td>WP_033433400.1</td><td>vWA+APATPase+wHTH+TPRs</td><td>-</td><td>1397</td><td>Saccharothrix syringae</td><td>-</td><td>actinobacteria</td></tr>
<tr><td>WP_033435813.1</td><td>vWA+APATPase+wHTH+TPRs</td><td>EAD2+VMAP-M0+VMAP-C→ MoxR→ vWA+APATPase+wHTH+TPRs*→ ||← TCAD15</td><td>1224</td><td>Saccharothrix syringae</td><td>GCF_000716755.1</td><td>actinobacteria</td></tr>
<tr><td>WP_033951030.1</td><td>vWA+TPRs</td><td>TCAD15||Trypco1→ Trypsin+HDxxH+VMAP-M0+VMAP-C→ MoxR→ vWA+TPRs*→ FXSXX-COOH→ Radical_SAM→ METALLOPROTEASE+TPRs→ TIR+APATPase+TPRs→</td><td>1135</td><td>Streptomyces sp. CNQ431</td><td>GCF_000797385.1</td><td>actinobacteria</td></tr>
<tr><td>WP_034272253.1</td><td>vWA+APATPase+wHTH+TPRs</td><td>PNP_UDP_1+VMAP-M0+VMAP-C→ MoxR→ vWA+APATPase+wHTH+TPRs*→ ||← METALLOPROTEASE+TPRs||METALLOPROTEASE+TPRs→ ||← PNP_UDP_1+EAD2 EAD2+VMAP-M0+APATPase+TPRs</td><td>1393</td><td>Actinospica robiniae</td><td>GCF_000504285.1</td><td>actinobacteria</td></tr>
<tr><td>WP_035845007.1</td><td>vWA+APATPase+BetaPropeller+cNMP_cyclase+NaeI</td><td>Trypco1→ Trypsin+EAD2→ EAD2+VMAP-M10+VMAP-C→ MoxR→ vWA+APATPase+BetaPropeller+cNMP_cyclase+NaeI*→</td><td>1519</td><td>Kitasatospora azatica</td><td>GCF_000744785.1</td><td>actinobacteria</td></tr>
<tr><td>WP_035869417.1</td><td>vWA_protein</td><td>AB_hydrolase→ CASPASE+VMAP-M2+VMAP-C→ MoxR→ vWA_protein*→</td><td>521</td><td>Kitasatospora cheerisanensis</td><td>GCA_000696185.1</td><td>actinobacteria</td></tr>
<tr><td>WP_036013968.1</td><td>vWA+APATPase+wHTH+TPRs</td><td>cNMP_binding+HTH_Crp_2||cNMP_cyclase+EAD2+VMAP-M0+VMAP-C→ MoxR→ vWA+APATPase+wHTH+TPRs*→ ||← METALLOPROTEASE+TPRs FXSXX-COOH-like||TIR+TPRs→</td><td>1412</td><td>Lentzea albidocapillata</td><td>GCA_900176525.1</td><td>actinobacteria</td></tr>
<tr><td>WP_036266990.1</td><td>vWA_protein</td><td>EAD1+VMAP-M1+VMAP-C→ MoxR→ MoxR→ vWA_protein*→ vWA(partial)→ FGS-fold_domain→ FGS-fold_domain→</td><td>433</td><td>Mastigocoleus testarum</td><td>GCA_001456025.1</td><td>cyanobacteria</td></tr>
<tr><td>WP_037064623.1</td><td>vWA+APATPase+wHTH+TPRs</td><td>-</td><td>1224</td><td>Pseudonocardia acaciae</td><td>-</td><td>actinobacteria</td></tr>
<tr><td>WP_037614039.1</td><td>vWA+TPRs</td><td>TCAD15→ ?→ ?→ Trypco1→ Trypsin+HDxxH+VMAP-M0+VMAP-C→ MoxR→ vWA+TPRs*→ FXSXX-COOH-like→ METALLOPROTEASE+TPRs→ TIR+APATPase+TPRs→</td><td>1054</td><td>Streptomyces albus</td><td>GCA_004768505.1</td><td>actinobacteria</td></tr>
<tr><td>WP_037620923.1</td><td>vWA+TPRs</td><td>TCAD15||Trypco1→ Trypsin+HDxxH+VMAP-M0+VMAP-C→ MoxR→ vWA+TPRs*→ FXSXX-COOH→ Radical_SAM→ METALLOPROTEASE+TPRs→ TIR+APATPase+TPRs→</td><td>1104</td><td>Streptomyces aureus</td><td>GCF_000725495.1</td><td>actinobacteria</td></tr>
<tr><td>WP_037623874.1</td><td>vWA+DO-GTPase2</td><td>AB_hydrolase→ CASPASE+VMAP-M18+VMAP-C→ MoxR→ vWA+DO-GTPase2*→</td><td>971</td><td>Streptomyces aureus</td><td>GCF_000725495.1</td><td>actinobacteria</td></tr>
<tr><td>WP_037655222.1</td><td>vWA+Pkinase+cNMP_cyclase+Band7+HAD</td><td>Trypco1→ Trypsin+VMAP-M0+VMAP-C→ MoxR→ vWA+Pkinase+cNMP_cyclase+Band7+HAD*→</td><td>1320</td><td>Streptomyces griseofuscus</td><td>GCF_000718315.1</td><td>actinobacteria</td></tr>
<tr><td>WP_037669722.1</td><td>vWA+APATPase+wHTH+TPRs</td><td>EAD2+VMAP-M0+VMAP-C→ PSE→ vWA+APATPase+wHTH+TPRs*→</td><td>1191</td><td>Streptomyces afghaniensis</td><td>GCF_000415505.1</td><td>actinobacteria</td></tr>
<tr><td>WP_037672148.1</td><td>vWA+cNMP_cyclase+Band7+HAD</td><td>Trypco1→ Trypsin+VMAP-M0+VMAP-C→ MoxR→ vWA+cNMP_cyclase+Band7+HAD*→</td><td>1108</td><td>Streptomyces griseus</td><td>GCF_000720255.1</td><td>actinobacteria</td></tr>
<tr><td>WP_037673438.1</td><td>vWA+TPRs</td><td>TCAD15||Trypco1→ Trypsin+HDxxH+VMAP-M0+VMAP-C→ MoxR→ vWA+TPRs*→ FXSXX-COOH→ METALLOPROTEASE+TPRs→ TIR+APATPase+TPRs→</td><td>1039</td><td>Streptomyces globisporus</td><td>-</td><td>actinobacteria</td></tr>
<tr><td>WP_037685307.1</td><td>vWA+TPRs</td><td>TCAD15||Trypco1→ Trypsin+HDxxH+VMAP-M0+VMAP-C→ MoxR→ vWA+TPRs*→ FXSXX-COOH→ Radical_SAM→ METALLOPROTEASE+TPRs→ TIR+APATPase+TPRs→</td><td>1112</td><td>Streptomyces durhamensis</td><td>-</td><td>actinobacteria</td></tr>
<tr><td>WP_037689151.1</td><td>vWA+TPRs</td><td>TCAD15||Trypco1→ Trypsin+HDxxH+VMAP-M0+VMAP-C→ MoxR→ vWA+TPRs*→ FXSXX-COOH→ Radical_SAM→ METALLOPROTEASE+TPRs→ TIR+APATPase+TPRs→</td><td>1150</td><td>Streptomyces atratus</td><td>GCF_000745675.1</td><td>actinobacteria</td></tr>
<tr><td>WP_037691437.1</td><td>vWA+APATPase+wHTH+TPRs</td><td>EAD2+VMAP-M0+VMAP-C→ MoxR→ vWA+APATPase+wHTH+TPRs*→ AB_hydrolase→ CASPASE+EAD2→ FXSXX-COOH-like→ METALLOPROTEASE+TPRs→</td><td>1440</td><td>Streptomyces atratus</td><td>GCF_000745675.1</td><td>actinobacteria</td></tr>
<tr><td>WP_037703811.1</td><td>vWA_protein</td><td>vWA_protein*→</td><td>251</td><td>Streptomyces scabiei</td><td>GCA_000738695.1</td><td>actinobacteria</td></tr>
<tr><td>WP_037722884.1</td><td>vWA</td><td>TCAD9+Pkinase+NACHT+HEAT_repeats→ EAD2+VMAP-M0+VMAP-C→ vWA*→ MoxR→ vWA+APATPase+TPRs→</td><td>481</td><td>Streptomyces scabiei</td><td>GCA_000738715.1</td><td>actinobacteria</td></tr>
<tr><td>WP_037743264.1</td><td>vWA+APATPase+wHTH+TPRs</td><td>EAD2+VMAP-M0+VMAP-C→ MoxR→ vWA+APATPase+wHTH+TPRs*→ ||← Na_H_Exchanger||AB_hydrolase→ CASPASE+EAD2→ FXSXX-COOH-like→ METALLOPROTEASE+TPRs→</td><td>1519</td><td>Streptomyces mirabilis</td><td>GCF_000746395.1</td><td>actinobacteria</td></tr>
<tr><td>WP_037751402.1</td><td>vWA+TPRs</td><td>TCAD15||Trypco1→ Trypsin+HDxxH+VMAP-M0+VMAP-C→ MoxR→ vWA+TPRs*→ FXSXX-COOH→ Radical_SAM→ TIR+APATPase+TPRs→</td><td>1071</td><td>Streptomyces sp. CNQ-525</td><td>GCF_000701285.1</td><td>actinobacteria</td></tr>
<tr><td>WP_037761316.1</td><td>vWA+TPRs</td><td>TCAD15||Trypco1→ Trypsin+HDxxH+VMAP-M0+VMAP-C→ MoxR→ vWA+TPRs*→ FXSXX-COOH→ Radical_SAM→ METALLOPROTEASE+TPRs→</td><td>1003</td><td>Streptomyces sp. FXJ7.023</td><td>GCF_000404005.1</td><td>actinobacteria</td></tr>
<tr><td>WP_037767255.1</td><td>vWA+APATPase+wHTH+TPRs</td><td>EAD2+VMAP-M0+VMAP-C→ MoxR→ vWA+APATPase+wHTH+TPRs*→ AB_hydrolase→ CASPASE+EAD2→ FXSXX-COOH-like→ METALLOPROTEASE+TPRs→</td><td>1497</td><td>Streptomyces olindensis</td><td>GCA_000696115.1</td><td>actinobacteria</td></tr>
<tr><td>WP_037783701.1</td><td>vWA+TPRs</td><td>TCAD15||Trypco1→ Trypsin+HDxxH+VMAP-M0+VMAP-C→ MoxR→ vWA+TPRs*→ FXSXX-COOH→ Radical_SAM→ METALLOPROTEASE+TPRs→ TIR+APATPase+TPRs→</td><td>1089</td><td>Streptomyces sp. HGB0020</td><td>-</td><td>actinobacteria</td></tr>
<tr><td>WP_037787089.1</td><td>vWA+TPRs</td><td>TCAD15||Trypco1→ Trypsin+HDxxH+VMAP-M0+VMAP-C→ MoxR→ vWA+TPRs*→ FXSXX-COOH→ Radical_SAM→ METALLOPROTEASE+TPRs→ TIR+APATPase+TPRs→</td><td>1135</td><td>Streptomyces sp. GBA 94-10</td><td>GCF_000495635.1</td><td>actinobacteria</td></tr>
</table>

acc	architecture	operon	len	species	gca	taxend
WP_037798431.1	vWA+APATPase+wHTH+TPRs	cNMP_binding+HTH_Crp_2← ?||EAD2+VMAP-M0+VMAP-C→ MoxR→ vWA+APATPase+wHTH+TPRs*→ ||← Na_H_Exchanger||AB_hydrolase→ CASPASE+EAD2→ FXSXX-COOH-like→ METALLOPROTEASE+TPRs→	1525	Streptomyces sp. Mg1	GCA_000412265.2	actinobacteria
WP_037821823.1	vWA+TPRs	TCAD15||Trypco1→ Trypsin+HDxxH+VMAP-M0+VMAP-C→ MoxR→ vWA+TPRs*→ FXSXX-COOH→ Radical_SAM→ METALLOPROTEASE+TPRs→ TIR+APATPase+TPRs→	1120	Streptomyces sp. NRRL B-24720	-	actinobacteria
WP_037824673.1	vWA+TPRs	TCAD15||Trypco1→ Trypsin+HDxxH+VMAP-M0+VMAP-C→ MoxR→ vWA+TPRs*→ FXSXX-COOH→ Radical_SAM→ METALLOPROTEASE+TPRs→ TIR+APATPase+TPRs→ Trypco1→	1098	Streptomyces	GCF_000719665.1	actinobacteria
WP_037832270.1	vWA+TPRs	TCAD15||Trypco1→ Trypsin+HDxxH+VMAP-M0+VMAP-C→ MoxR→ vWA+TPRs*→ FXSXX-COOH→ Radical_SAM→ METALLOPROTEASE+TPRs→ TIR+APATPase+TPRs→ Trypco1→	1098	Streptomyces sp. NRRL S-1831	GCF_000721335.1	actinobacteria
WP_037839805.1	vWA+TPRs	TCAD15||PSE→ Trypsin+HDxxH+VMAP-M0+VMAP-C→ MoxR→ vWA+TPRs*→ FXSXX-COOH→ Radical_SAM→ TIR+APATPase+TPRs→	1226	Streptomyces sp. NRRL S-337	GCF_000720175.1	actinobacteria
WP_037842882.1	vWA+TPRs	TCAD15||Trypco1→ Trypsin+HDxxH+VMAP-M0+VMAP-C→ MoxR→ vWA+TPRs*→ FXSXX-COOH→ Radical_SAM→ METALLOPROTEASE+TPRs→ TIR+APATPase+TPRs→	1137	Streptomyces sp. NRRL F-6628	GCF_000721455.1	actinobacteria
WP_037880970.1	vWA+APATPase+wHTH+TPRs	EAD2+VMAP-M0+VMAP-C→ MoxR→ vWA+APATPase+wHTH+TPRs*→ AB_hydrolase→ CASPASE+EAD2→ FXSXX-COOH-like→ METALLOPROTEASE+TPRs→	1457	Streptomyces sp. NTK 937	GCA_000698495.1	actinobacteria
WP_037895842.1	vWA+Trypsin+cNMP_cyclase+CCCH(TrebleClef)+Pentapeptide_repeats	AB_hydrolase→ CASPASE+VMAP-M0+VMAP-C→ MoxR→ vWA+Trypsin+cNMP_cyclase+CCCH(TrebleClef)+Pentapeptide_repeats*→	1258	Streptomyces xanthophaeus	GCF_000725805.1	actinobacteria
WP_037930286.1	vWA+APATPase+wHTH+TPRs	EAD2+VMAP-M0+VMAP-C→ MoxR→ vWA+APATPase+wHTH+TPRs*→ ||← Na_H_Exchanger||FXSXX-COOH-like→ METALLOPROTEASE+TPRs→	1517	Streptomyces toyocaensis	GCA_000725125.1	actinobacteria
WP_037952430.1	vWA+APATPase+wHTH+TPRs	PNP_UDP_1+EAD2→ EAD2+VMAP-M0+VMAP-C→ MoxR→ vWA+APATPase+wHTH+TPRs*→ ||← METALLOPROTEASE+TPRs← FXSXX-COOH-like	1455	Streptomyces sp. PRh5	GCA_000590515.1	actinobacteria
WP_037969441.1	vWA+TPRs	TCAD15||Trypco1→ Trypsin+HDxxH+VMAP-M0+VMAP-C→ MoxR→ vWA+TPRs*→ FXSXX-COOH→ Radical_SAM→ METALLOPROTEASE+TPRs→ TIR+APATPase+TPRs→	1135	Streptomyces sp. PVA 94-07	GCF_000495755.1	actinobacteria
WP_038524289.1	vWA+APATPase+wHTH+TPRs	-	1197	Amycolatopsis japonica	-	actinobacteria
WP_038534869.1	vWA+Pkinase+wHTH+TPRs+cNMP_cyclase	Trypco1→ Trypsin+VMAP-M0+VMAP-C→ MoxR→ vWA+Pkinase+wHTH+TPRs+cNMP_cyclase*→	1349	Streptomyces	GCA_000739105.1	actinobacteria
WP_039651442.1	vWA+TPRs	TCAD15→ ?→ Trypco1→ Trypsin+HDxxH+VMAP-M0+VMAP-C→ MoxR→ vWA+TPRs*→ FXSXX-COOH-like→ METALLOPROTEASE+TPRs→ TIR+APATPase+TPRs→	1136	Streptomyces	GCA_000802245.2	actinobacteria
WP_039727590.1	vWA+FGS-fold_domain	Trypco1→ Trypsin+VMAP-M1+VMAP-C→ MoxR→ vWA+FGS-fold_domain*→ ?→ FGS-fold_domain→	893	Lyngbya confervoides	GCA_004212215.1	cyanobacteria
WP_039795711.1	vWA+APATPase+wHTH+TPRs	-	1202	Amycolatopsis alba	-	actinobacteria
WP_039896832.1	vWA+FGS-fold_domain	-	883	Lyngbya sp. PCC 8106	-	cyanobacteria
WP_039923843.1	vWA+APATPase+wHTH+TPRs	-	1199	Amycolatopsis decaplanina	-	actinobacteria
WP_039936444.1	vWA+APATPase+wHTH+TPRs	EAD2+VMAP-M0+VMAP-C→ MoxR→ vWA+APATPase+wHTH+TPRs*→ ||← METALLOPROTEASE+TPRs← FXSXX-COOH-like	1451	Streptomyces himastatinicus	GCF_000158915.1	actinobacteria
WP_039938052.1	vWA+APATPase+wHTH+TPRs	vWA+APATPase+wHTH+TPRs*→ ||← METALLOPROTEASE+TPRs← Radical_SAM← FXSXX-COOH||?→ ?→ ||← Trypco1	1131	Streptomyces himastatinicus	GCF_000158915.1	actinobacteria
WP_040793520.1	vWA+TPRs	Trypco1→ Trypsin+HDxxH+VMAP-M0+VMAP-C→ MoxR→ vWA+TPRs*→ Voltage_CLC→ METALLOPROTEASE→ TIR+APATPase+wHTH+TPRs→	794	Nocardia paucivorans	GCF_000308675.1	actinobacteria
WP_040841206.1	vWA+TPRs	Trypsin+HDxxH+VMAP-M0+VMAP-C→ MoxR→ vWA+TPRs*→ Voltage_CLC→ METALLOPROTEASE→ TIR+APATPase+wHTH+TPRs→	794	Nocardia brevicatena	GCF_000308495.1	actinobacteria
WP_041256884.1	vWA+APATPase+wHTH+TPRs	-	1324	Frankia sp. EAN1pec	-	actinobacteria
WP_041316524.1	vWA+APATPase+wHTH+TPRs	-	1280	Saccharothrix espanaensis	-	actinobacteria
WP_041318577.1	vWA+APATPase+wHTH+TPRs	-	1434	Saccharothrix espanaensis	-	actinobacteria
WP_041439415.1	vWA+APATPase+wHTH+TPRs	-	1272	Thermomonospora curvata	-	actinobacteria
WP_041826591.1	vWA+TPRs	TCAD15||Trypco1→ Trypsin+HDxxH+VMAP-M0+VMAP-C→ MoxR→ vWA+TPRs*→ FXSXX-COOH→ Radical_SAM→ METALLOPROTEASE+TPRs→ TIR+APATPase+TPRs→	1137	Streptomyces albus	GCA_003182435.1	actinobacteria
WP_041992912.1	vWA+APATPase+wHTH+TPRs	EAD2+VMAP-M0+VMAP-C→ MoxR→ vWA+APATPase+wHTH+TPRs*→ ||← Na_H_Exchanger||AB_hydrolase→ CASPASE+EAD2→ FXSXX-COOH-like→	1445	Streptomyces sp. AcH 505	GCA_000818175.1	actinobacteria
WP_042152731.1	vWA+Pkinase+ParA-Soj-PloopNTPase(SIMIBI)	Trypco1→ Trypsin+EAD2+VMAP-M0+VMAP-C→ MoxR→ vWA+Pkinase+ParA-Soj-PloopNTPase(SIMIBI)*→	1136	Streptomyces sp. NBRC 110027	GCA_000829715.2	actinobacteria
WP_042196291.1	vWA+EAD2+VMAP-M0+VMAP-C	AB_hydrolase→ CASPASE+VMAP-M2+VMAP-C→ MoxR→ vWA+EAD2+VMAP-M0+VMAP-C*→	1031	Kibdelosporangium sp. MJ126-NF4	GCA_000826545.1	actinobacteria
WP_042456080.1	vWA+TPRs	TCAD15||Trypco1→ Trypsin+HDxxH+VMAP-M0+VMAP-C→ MoxR→ vWA+TPRs*→ FXSXX-COOH→ Radical_SAM→ METALLOPROTEASE+TPRs→ TIR+APATPase+TPRs→	1053	Streptacidiphilus jiangxiensis	GCA_900109465.1	actinobacteria
WP_042829953.1	vWA+wHTH+TPRs+wHTH+TPRs+APATPase+wHTH+TPRs	Trypco1→ PSE→ MoxR→ vWA+wHTH+TPRs+wHTH+TPRs+APATPase+wHTH+TPRs*→ ||← AB_hydrolase	1732	Streptomyces rimosus	GCA_000707965.1	actinobacteria
WP_043177770.1	vWA+TPRs	TCAD15||Trypco1→ Trypsin+HDxxH+VMAP-M0+VMAP-C→ MoxR→ vWA+TPRs*→ FXSXX-COOH→ Radical_SAM→ METALLOPROTEASE+TPRs→ TIR+APATPase+TPRs→	1114	Streptomyces sp. NRRL F-2890	GCF_000719915.1	actinobacteria
WP_043270727.1	vWA+TPRs	TCAD15||Trypco1→ Trypsin+HDxxH+VMAP-M0+VMAP-C→ MoxR→ vWA+TPRs*→ FXSXX-COOH→ Radical_SAM→	1153	Streptomyces sp. CT34	GCF_000805335.1	actinobacteria
WP_043437220.1	vWA+APATPase+wHTH+TPRs	Trypsin+EAD2→ EAD2+VMAP-M0+VMAP-C→ MoxR→ vWA+APATPase+wHTH+TPRs*→ ||← METALLOPROTEASE+TPRs← Radical_SAM← FXSXX-COOH||Voltage_CLC→ ||← Trypco1	1493	Streptomyces nodosus	GCF_000819545.1	actinobacteria
WP_043464408.1	vWA+APATPase+SWACOS+wHTH+TPRs+Beta-rich-tail	Trypsin+EAD2→ EAD2+VMAP-M10+VMAP-C→ MoxR→ ||← ?||vWA+APATPase+SWACOS+wHTH+TPRs+Beta-rich-tail*→	1327	Kitasatospora sp. MBT66	GCF_000710405.2	actinobacteria
WP_043498196.1	vWA+APATPase+wHTH+TPRs	EAD2+VMAP-M0+VMAP-C→ MoxR→ vWA+APATPase+wHTH+TPRs*→ ||← METALLOPROTEASE+TPRs← FXSXX-COOH-like	1466	Streptomyces glaucescens	GCA_000761215.1	actinobacteria
WP_043678220.1	vWA+APATPase+wHTH+TPRs	EAD2+VMAP-M0+VMAP-C→ MoxR→ vWA+APATPase+wHTH+TPRs*→ AB_hydrolase→ CASPASE+EAD2→ FXSXX-COOH-like→ METALLOPROTEASE+TPRs→	1475	Streptomyces xylophagus	GCF_000716435.1	actinobacteria
WP_043777243.1	vWA+APATPase+wHTH+TPRs	cNMP_cyclase+EAD2→ EAD2+VMAP-M0+VMAP-C→ MoxR→ vWA+APATPase+wHTH+TPRs*→ ||← METALLOPROTEASE+TPRs← FXSXX-COOH-like	1201	Amycolatopsis rifamycinica	GCA_000695625.1	actinobacteria
WP_043978690.1	vWA+TPRs	TCAD15||Trypco1→ Trypsin+HDxxH+VMAP-M0+VMAP-C→ MoxR→ vWA+TPRs*→ FXSXX-COOH→ Radical_SAM→ TIR+APATPase+TPRs→	1102	Streptomyces rimosus	GCA_000707945.1	actinobacteria
WP_043996830.1	vWA+FGS-fold_domain	HTH+VMAP-M9+VMAP-C→ MoxR→ ?→ vWA+FGS-fold_domain*→ ?→ PSE→ FGS-fold_domain→	839	Microcystis aeruginosa	GCF_000312185.1	cyanobacteria
WP_044367918.1	vWA+APATPase+wHTH+TPRs	Trypco1→ Trypsin+EAD2+VMAP-M0+VMAP-C→ MoxR→ vWA+APATPase+wHTH+TPRs*→	1346	Streptomyces natalensis	GCA_000935125.1	actinobacteria
WP_044370540.1	vWA+APATPase+wHTH+TPRs	EAD2+VMAP-M0+VMAP-C→ MoxR→ vWA+APATPase+wHTH+TPRs*→ AB_hydrolase→ CASPASE+EAD2→ FXSXX-COOH-like→ METALLOPROTEASE+TPRs→	1463	Streptomyces griseus	GCA_003147545.1	actinobacteria
WP_044567980.1	vWA+APATPase+wHTH+TPRs	PNP_UDP_1+EAD2→ EAD2+VMAP-M0+VMAP-C→ MoxR→ vWA+APATPase+wHTH+TPRs*→ ||← METALLOPROTEASE+TPRs	1454	Streptomyces iranensis	GCA_000938975.1	actinobacteria
WP_044575624.1	vWA+TPRs	AB_hydrolase→ CASPASE+EAD2+VMAP-M0+VMAP-C→ MoxR→ vWA+TPRs*→	1166	Streptomyces iranensis	GCA_000938975.1	actinobacteria
WP_045051690.1	vWA+REC+TCAD8	AB_hydrolase→ CASPASE+VMAP-M4+VMAP-C→ MoxR→ vWA+REC+TCAD8*→	1336	Chromobacterium violaceum	GCA_000952105.1	betaproteobacteria
WP_045304982.1	vWA+Pkinase+ParA-Soj-PloopNTPase(SIMIBI)	Trypco1→ Trypsin+EAD2+VMAP-M0+VMAP-C→ MoxR→ vWA+Pkinase+ParA-Soj-PloopNTPase(SIMIBI)*→	1125	Saccharothrix sp. ST-888	GCA_000955975.1	actinobacteria
WP_046085906.1	vWA+APATPase+wHTH+TPRs	PNP_UDP_1+EAD2→ EAD2+VMAP-M0+VMAP-C→ MoxR→ vWA+APATPase+wHTH+TPRs*→ ||← METALLOPROTEASE+TPRs	1454	Streptomyces antioxidans	-	actinobacteria

<table>
<tr><th>acc</th><th>architecture</th><th>operon</th><th>len</th><th>species</th><th>gca</th><th>taxend</th></tr>
<tr><td>WP_046086747.1</td><td>vWA+TPRs</td><td>AB_hydrolase→ CASPASE+EAD2+VMAP-M0+VMAP-C→ MoxR→ vWA+TPRs*→</td><td>1177</td><td>Streptomyces antioxidans</td><td>-</td><td>actinobacteria</td></tr>
<tr><td>WP_046114163.1</td><td>vWA+BetaPropeller+REC</td><td>AB_hydrolase→ CASPASE+VMAP-M0+VMAP-C→ MoxR→ vWA+BetaPropeller+REC*→</td><td>1536</td><td>Aquincola tertiaricarbonis</td><td>GCF_000969605.1</td><td>betaproteobacteria</td></tr>
<tr><td>WP_046321251.1</td><td>vWA+TPRs+CASPASE</td><td>VMAP-C_protein→ MoxR→ vWA+TPRs+CASPASE*→</td><td>2052</td><td>Arthrospira sp. TJSD091</td><td>GCF_003060805.1</td><td>cyanobacteria</td></tr>
<tr><td>WP_046471648.1</td><td>vWA+APATPase+wHTH+TPRs</td><td>Trypsin+VMAP-M0+VMAP-C→ MoxR→ vWA+APATPase+wHTH+TPRs*→ ||← DUF397||Trypco1→</td><td>1516</td><td>Allosalinactinospora lopnorensis</td><td>GCF_000981225.1</td><td>actinobacteria</td></tr>
<tr><td>WP_046507861.1</td><td>vWA+TPRs</td><td>vWA+TPRs*→ FXSXX-COOH→ Radical_SAM→ METALLOPROTEASE+TPRs→ TIR+APATPase+TPRs→</td><td>1010</td><td>Streptomyces sp. NRRL B-24891</td><td>GCF_000981895.1</td><td>actinobacteria</td></tr>
<tr><td>WP_046591908.1</td><td>vWA+TPRs</td><td>vWA+CASPASE+NACHT+PIN← MoxR← Trypsin+HDxxH+VMAP-M0+VMAP-C← Trypco1||Trypco1→ Trypsin+HDxxH+VMAP-M0+VMAP-C→ MoxR→ vWA+TPRs*→ FXSXX-COOH→ Radical_SAM→ TIR+APATPase+TPRs→</td><td>1116</td><td>Streptomyces mangrovisoli</td><td>GCA_000974985.2</td><td>actinobacteria</td></tr>
<tr><td>WP_046724800.1</td><td>vWA+TPRs</td><td>TCAD15||Trypco1→ Trypsin+HDxxH+VMAP-M0+VMAP-C→ MoxR→ vWA+TPRs*→ FXSXX-COOH→ Radical_SAM→ METALLOPROTEASE+TPRs→ TIR+APATPase+TPRs→</td><td>1114</td><td>Streptomyces xiamenensis</td><td>GCA_000993785.2</td><td>actinobacteria</td></tr>
<tr><td>WP_046729107.1</td><td>vWA+APATPase+wHTH+TPRs</td><td>Trypsin+EAD2+VMAP-M0+VMAP-C→ vWA+APATPase+wHTH+TPRs*→ KAP_NTPase→</td><td>803</td><td>Streptomyces humi</td><td>GCF_001005085.2</td><td>actinobacteria</td></tr>
<tr><td>WP_046779758.1</td><td>vWA+Trypsin+cNMP_cyclase+CCCH(TrebleClef)+Pentapeptide_repeats</td><td>AB_hydrolase→ CASPASE+VMAP-M0+VMAP-C→ MoxR→ vWA+Trypsin+cNMP_cyclase+CCCH(TrebleClef)+Pentapeptide_repeats*→</td><td>1198</td><td>Streptomyces yangpuensis</td><td>GCF_001005295.1</td><td>actinobacteria</td></tr>
<tr><td>WP_047020366.1</td><td>vWA+TPRs</td><td>TCAD15||Trypco1→ Trypsin+HDxxH+VMAP-M0+VMAP-C→ MoxR→ vWA+TPRs*→ FXSXX-COOH→ Radical_SAM→ TIR+APATPase+TPRs→</td><td>1072</td><td>Streptomyces sp. CNQ-509</td><td>GCA_001011035.1</td><td>actinobacteria</td></tr>
<tr><td>WP_047141005.1</td><td>vWA+TPRs</td><td>TCAD15||Trypco1→ Trypsin+HDxxH+VMAP-M0+VMAP-C→ MoxR→ vWA+TPRs*→ FXSXX-COOH→ Radical_SAM→</td><td>1138</td><td>Streptomyces sp. KE1</td><td>GCA_001014595.1</td><td>actinobacteria</td></tr>
<tr><td>WP_047155762.1</td><td>vWA+FGS-fold_domain</td><td>-</td><td>878</td><td>Trichodesmium erythraeum</td><td>-</td><td>cyanobacteria</td></tr>
<tr><td>WP_047155868.1</td><td>vWA_protein</td><td>-</td><td>567</td><td>Trichodesmium erythraeum</td><td>-</td><td>cyanobacteria</td></tr>
<tr><td>WP_047467284.1</td><td>vWA+TPRs</td><td>TCAD15||Trypco1→ Trypsin+HDxxH+VMAP-M0+VMAP-C→ MoxR→ vWA+TPRs*→ FXSXX-COOH→ Radical_SAM→ METALLOPROTEASE+TPRs→ TIR+APATPase+TPRs→</td><td>1138</td><td>Streptomyces sp. M10</td><td>GCF_000800535.1</td><td>actinobacteria</td></tr>
<tr><td>WP_047861214.1</td><td>vWA+SLOG</td><td>EAD1+VMAP-M1+VMAP-C→ vWA+SLOG*→</td><td>1012</td><td>Archangium gephyra</td><td>GCF_001027285.1</td><td>deltaproteobacteria</td></tr>
<tr><td>WP_048244483.1</td><td>vWA+APATPase+wHTH</td><td>AB_hydrolase→ CASPASE+VMAP-M4+VMAP-C→ MoxR→ vWA+APATPase+wHTH*→</td><td>1373</td><td>Burkholderia cepacia</td><td>GCA_001039005.1</td><td>betaproteobacteria</td></tr>
<tr><td>WP_048584307.1</td><td>vWA+APATPase+wHTH+TPRs</td><td>Trypco2→ EAD2+VMAP-M0+VMAP-C→ vWA+APATPase+wHTH+TPRs*→ ||← METALLOPROTEASE+TPRs← FXSXX-COOH-like← Trypsin+EAD2</td><td>1301</td><td>Streptomyces viridochromogenes</td><td>GCF_001047325.1</td><td>actinobacteria</td></tr>
<tr><td>WP_048586084.1</td><td>vWA+TPRs</td><td>TCAD15→ Trypco1→ Trypsin+HDxxH+VMAP-M0+VMAP-C→ MoxR→ vWA+TPRs*→ Radical_SAM→ TIR+APATPase+TPRs→</td><td>1089</td><td>Streptomyces viridochromogenes</td><td>GCF_001047325.1</td><td>actinobacteria</td></tr>
<tr><td>WP_048832216.1</td><td>vWA+TPRs</td><td>TCAD15||Trypco1→ Trypsin+HDxxH+VMAP-M0+VMAP-C→ MoxR→ vWA+TPRs*→ FXSXX-COOH→ Radical_SAM→ METALLOPROTEASE+TPRs→ TIR+APATPase+TPRs→</td><td>1096</td><td>Streptomyces varsoviensis</td><td>GCF_000718635.1</td><td>actinobacteria</td></tr>
<tr><td>WP_049580601.1</td><td>vWA+TPRs</td><td>vWA+TPRs*→ FXSXX-COOH→</td><td>938</td><td>Streptomyces sp. SBT349</td><td>GCF_001083795.1</td><td>actinobacteria</td></tr>
<tr><td>WP_049716945.1</td><td>vWA+TPRs</td><td>TCAD15||Trypsin+HDxxH+VMAP-M0+VMAP-C→ MoxR→ vWA+TPRs*→ FXSXX-COOH→ METALLOPROTEASE+TPRs→ TIR+APATPase+TPRs→</td><td>1159</td><td>Streptomyces caatingaensis</td><td>GCA_001187435.1</td><td>actinobacteria</td></tr>
<tr><td>WP_049770996.1</td><td>vWA</td><td>FGS-fold_domain← ?← ?← ?||?→ VMAP-M1+VMAP-C→ MoxR→ vWA*→ ||← zf-IS66+DDE_Tnp_IS66← TIR+EAD9← PSE← Trypsin← Trypco1</td><td>640</td><td>Cyanothece sp. PCC 7425</td><td>GCF_000022045.1</td><td>cyanobacteria</td></tr>
<tr><td>WP_049977087.1</td><td>vWA+TPRs</td><td>TCAD15← ?||Trypco1→ Trypsin+HDxxH+VMAP-M0+VMAP-C→ MoxR→ vWA+TPRs*→ FXSXX-COOH→ Radical_SAM→ METALLOPROTEASE+TPRs→ TIR+APATPase+TPRs→</td><td>1137</td><td>Streptomyces wadayamensis</td><td>GCA_000698945.1</td><td>actinobacteria</td></tr>
<tr><td>WP_050025079.1</td><td>vWA+AP-GTPase+COR+EAD1+Trypsin</td><td>VMAP-M22+VMAP-C→ MoxR→ vWA+AP-GTPase+COR+EAD1+Trypsin*→</td><td>1458</td><td>Verrucomicrobium sp. BvORR034</td><td>GCF_000739635.1</td><td>verrucomicrobia</td></tr>
<tr><td>WP_050358421.1</td><td>vWA+Pkinase+ParA-Soj-PloopNTPase(SIMIBI)</td><td>Trypco1→ Trypsin+EAD2+VMAP-M0+VMAP-C→ MoxR→ vWA+Pkinase+ParA-Soj-PloopNTPase(SIMIBI)*→</td><td>1245</td><td>Streptomyces europaeiscabiei</td><td>GCF_001189025.1</td><td>actinobacteria</td></tr>
<tr><td>WP_050360439.1</td><td>vWA+TPRs</td><td>TCAD15||Trypco1→ Trypsin+HDxxH+VMAP-M0+VMAP-C→ MoxR→ vWA+TPRs*→ Radical_SAM→ TIR+APATPase+TPRs→</td><td>936</td><td>Streptomyces europaeiscabiei</td><td>GCF_001189025.1</td><td>actinobacteria</td></tr>
<tr><td>WP_050361396.1</td><td>vWA+TPRs</td><td>AB_hydrolase→ CASPASE+EAD2+VMAP-M0+VMAP-C→ MoxR→ vWA+TPRs*→</td><td>1170</td><td>Streptomyces europaeiscabiei</td><td>GCF_001189025.1</td><td>actinobacteria</td></tr>
<tr><td>WP_050371750.1</td><td>vWA+Pkinase+TPRs</td><td>Trypco2→ Trypsin+EAD2+VMAP-M0+VMAP-C→ MoxR→ vWA+Pkinase+TPRs*→</td><td>1028</td><td>Streptomyces acidiscabies</td><td>GCF_001189015.1</td><td>actinobacteria</td></tr>
<tr><td>WP_050373512.1</td><td>vWA+Pkinase+wHTH+cNMP_cyclase+Band7+HAD</td><td>Trypco1→ Trypsin+VMAP-M0+VMAP-C→ MoxR→ vWA+Pkinase+wHTH+cNMP_cyclase+Band7+HAD*→</td><td>1190</td><td>Streptomyces acidiscabies</td><td>GCF_001189015.1</td><td>actinobacteria</td></tr>
<tr><td>WP_050500594.1</td><td>vWA+Pkinase+ParA-Soj-PloopNTPase(SIMIBI)</td><td>Trypco1→ Trypsin+EAD2+VMAP-M0+VMAP-C→ MoxR→ vWA+Pkinase+ParA-Soj-PloopNTPase(SIMIBI)*→ ?→ NUDIX→</td><td>1128</td><td>Streptomyces rimosus</td><td>GCF_000720595.1</td><td>actinobacteria</td></tr>
<tr><td>WP_050503919.1</td><td>vWA+Pkinase+ParA-Soj-PloopNTPase(SIMIBI)</td><td>Trypco1→ Trypsin+EAD2+VMAP-M0+VMAP-C→ MoxR→ vWA+Pkinase+ParA-Soj-PloopNTPase(SIMIBI)*→ ?→ NUDIX→</td><td>1127</td><td>Streptomyces rimosus</td><td>GCF_000718675.1</td><td>actinobacteria</td></tr>
<tr><td>WP_050505663.1</td><td>vWA+Pkinase+TPRs</td><td>Trypco2→ Trypsin+EAD2+VMAP-M0+VMAP-C→ MoxR→ vWA+Pkinase+TPRs*→</td><td>1129</td><td>Streptomyces albus</td><td>GCF_000716945.1</td><td>actinobacteria</td></tr>
<tr><td>WP_050507882.1</td><td>vWA+Pkinase+ParA-Soj-PloopNTPase(SIMIBI)</td><td>Trypco1→ Trypsin+EAD2+VMAP-M0+VMAP-C→ MoxR→ vWA+Pkinase+ParA-Soj-PloopNTPase(SIMIBI)*→ ?→ NUDIX→</td><td>1127</td><td>Streptomyces</td><td>GCF_000716455.1</td><td>actinobacteria</td></tr>
<tr><td>WP_050509157.1</td><td>vWA+TPRs</td><td>TCAD15||Trypco1→ Trypsin+HDxxH+VMAP-M0+VMAP-C→ MoxR→ vWA+TPRs*→ FXSXX-COOH→ Radical_SAM→ TIR+APATPase+TPRs→</td><td>1102</td><td>Streptomyces rimosus</td><td>GCF_000716515.1</td><td>actinobacteria</td></tr>
<tr><td>WP_050512457.1</td><td>vWA+Pkinase+ParA-Soj-PloopNTPase(SIMIBI)</td><td>Trypco1→ Trypsin+EAD2+VMAP-M0+VMAP-C→ MoxR→ vWA+Pkinase+ParA-Soj-PloopNTPase(SIMIBI)*→ ?→ NUDIX→</td><td>1127</td><td>Streptomyces rimosus</td><td>GCF_000717815.1</td><td>actinobacteria</td></tr>
<tr><td>WP_050987396.1</td><td>vWA+Pkinase+ParA-Soj-PloopNTPase(SIMIBI)</td><td>Trypco1→ Trypsin+EAD2+VMAP-M0+VMAP-C→ MoxR→ vWA+Pkinase+ParA-Soj-PloopNTPase(SIMIBI)*→</td><td>1108</td><td>Streptomyces acidiscabies</td><td>GCF_000242715.1</td><td>actinobacteria</td></tr>
<tr><td>WP_050990728.1</td><td>vWA+Pkinase+wHTH+TPRs+cNMP_cyclase</td><td>Trypco1→ Trypsin+VMAP-M0+VMAP-C→ MoxR→ vWA+Pkinase+wHTH+TPRs+cNMP_cyclase*→</td><td>1270</td><td>Streptomyces sp. TOR3209</td><td>GCF_000259895.1</td><td>actinobacteria</td></tr>
<tr><td>WP_051034042.1</td><td>vWA+TPRs</td><td>Trypco2→ Trypsin+EAD2+VMAP-M0+VMAP-C→ MoxR→ vWA+TPRs*→ AB_hydrolase→</td><td>1420</td><td>Streptomyces sp. SS</td><td>GCF_000302615.1</td><td>actinobacteria</td></tr>
<tr><td>WP_051034131.1</td><td>vWA+APATPase+wHTH+TPRs</td><td>EAD2+VMAP-M0+VMAP-C→ MoxR→ vWA+APATPase+wHTH+TPRs*→ ||← Na_H_Exchanger||AB_hydrolase→ CASPASE+EAD2→ FXSXX-COOH-like→ METALLOPROTEASE+TPRs→</td><td>1552</td><td>Streptomyces sp. SS</td><td>GCF_000302615.1</td><td>actinobacteria</td></tr>
<tr><td>WP_051042918.1</td><td>vWA</td><td>Trypsin+EAD2→ EAD2+VMAP-M0+VMAP-C→ MoxR→ vWA*→ Trypco1→</td><td>548</td><td>Nocardiopsis xinjiangensis</td><td>GCF_000341145.1</td><td>actinobacteria</td></tr>
<tr><td>WP_051087510.1</td><td>vWA+Pkinase+ParA-Soj-PloopNTPase(SIMIBI)</td><td>Trypco1→ Trypsin+EAD2+VMAP-M0+VMAP-C→ MoxR→ vWA+Pkinase+ParA-Soj-PloopNTPase(SIMIBI)*→</td><td>1055</td><td>Streptomyces vitaminophilus</td><td>GCA_001445835.1</td><td>actinobacteria</td></tr>
<tr><td>WP_051093954.1</td><td>vWA+Pkinase+ParA-Soj-PloopNTPase(SIMIBI)</td><td>-</td><td>1179</td><td>Streptomyces sp. MspMP-M5</td><td>-</td><td>actinobacteria</td></tr>
<tr><td>WP_051095183.1</td><td>vWA+Pkinase+ParA-Soj-PloopNTPase(SIMIBI)</td><td>Trypco1→ Trypsin+EAD2+VMAP-M0+VMAP-C→ MoxR→ vWA+Pkinase+ParA-Soj-PloopNTPase(SIMIBI)*→</td><td>1116</td><td>Streptomyces sp. LaPpAH-108</td><td>GCF_000373625.1</td><td>actinobacteria</td></tr>
<tr><td>WP_051101697.1</td><td>vWA+Pkinase+wHTH+TPRs+cNMP_cyclase+Band7</td><td>Trypco1→ Trypsin+VMAP-M0+VMAP-C→ MoxR→ vWA+Pkinase+wHTH+TPRs+cNMP_cyclase+Band7*→</td><td>1343</td><td>Streptomyces sp. FxanaD5</td><td>GCF_000373465.1</td><td>actinobacteria</td></tr>
<tr><td>WP_051102942.1</td><td>vWA+Pkinase+ParA-Soj-PloopNTPase(SIMIBI)</td><td>Trypco1→ Trypsin+EAD2+VMAP-M0+VMAP-C→ MoxR→ vWA+Pkinase+ParA-Soj-PloopNTPase(SIMIBI)*→</td><td>1114</td><td>Streptomyces sp. CNY243</td><td>GCF_000377165.1</td><td>actinobacteria</td></tr>
<tr><td>WP_051103089.1</td><td>vWA+Pkinase+ParA-Soj-PloopNTPase(SIMIBI)</td><td>Trypco1→ Trypsin+EAD2+VMAP-M0+VMAP-C→ MoxR→ vWA+Pkinase+ParA-Soj-PloopNTPase(SIMIBI)*→</td><td>1114</td><td>Streptomyces sp. CNQ766</td><td>-</td><td>actinobacteria</td></tr>
</table>

<table>
<tr><th>acc</th><th>architecture</th><th>operon</th><th>len</th><th>species</th><th>gca</th><th>taxend</th></tr>
<tr><td>WP_051105858.1</td><td>vWA+Pkinase+ParA-Soj-PloopNTPase(SIMIBI)</td><td>Trypco1→ Trypsin+EAD2+VMAP-M0+VMAP-C→ MoxR→ vWA+Pkinase+ParA-Soj-PloopNTPase(SIMIBI)*→</td><td>1110</td><td>Streptomyces hokutonensis</td><td>GCF_000376565.1</td><td>actinobacteria</td></tr>
<tr><td>WP_051107757.1</td><td>vWA+cNMP_cyclase+cNMP_cyclase+FtsK+TPRs</td><td>Trypco1→ Trypsin+VMAP-M8+VMAP-C→ MoxR→ vWA+cNMP_cyclase+cNMP_cyclase+FtsK+TPRs*→ ?→ ?→ ||← DUF397← DUF397</td><td>1751</td><td>Actinomadura atramentaria</td><td>GCF_000381885.1</td><td>actinobacteria</td></tr>
<tr><td>WP_051113540.1</td><td>vWA+APATPase+wHTH+TPRs</td><td>EAD2+VMAP-M0+VMAP-C→ vWA+APATPase+wHTH+TPRs*→ ||← METALLOPROTEASE+TPRs← FXSXX-COOH-like← Trypsin+EAD2||METALLOPROTEASE+TPRs→</td><td>1290</td><td>Streptomyces davawensis</td><td>GCF_000349325.1</td><td>actinobacteria</td></tr>
<tr><td>WP_051114495.1</td><td>vWA+Pkinase+ParA-Soj-PloopNTPase(SIMIBI)</td><td>Trypco1→ Trypsin+EAD2+VMAP-M0+VMAP-C→ MoxR→ vWA+Pkinase+ParA-Soj-PloopNTPase(SIMIBI)*→</td><td>1114</td><td>Streptomyces sp. CNS335</td><td>GCF_000377125.1</td><td>actinobacteria</td></tr>
<tr><td>WP_051114526.1</td><td>vWA+Pkinase+ParA-Soj-PloopNTPase(SIMIBI)</td><td>Trypco1→ Trypsin+EAD2+VMAP-M0+VMAP-C→ MoxR→ vWA+Pkinase+ParA-Soj-PloopNTPase(SIMIBI)*→</td><td>1077</td><td>Streptomyces sp. CNB091</td><td>GCF_000377965.1</td><td>actinobacteria</td></tr>
<tr><td>WP_051127194.1</td><td>vWA+Pkinase+wHTH+TPRs+cNMP_cyclase+EAD2+Band7+HAD</td><td>Trypco1→ Trypsin+VMAP-M0+VMAP-C→ MoxR→ vWA+Pkinase+wHTH+TPRs+cNMP_cyclase+EAD2+Band7+HAD*→</td><td>1459</td><td>Streptomyces sp. HmicA12</td><td>GCF_000373565.1</td><td>actinobacteria</td></tr>
<tr><td>WP_051138277.1</td><td>vWA+Pkinase+wHTH+TPRs+cNMP_cyclase+Band7</td><td>Trypco1→ Trypsin+VMAP-M0+VMAP-C→ MoxR→ vWA+Pkinase+wHTH+TPRs+cNMP_cyclase+Band7*→</td><td>1313</td><td>Streptomyces canus</td><td>-</td><td>actinobacteria</td></tr>
<tr><td>WP_051159397.1</td><td>vWA+Pkinase+ParA-Soj-PloopNTPase(SIMIBI)</td><td>Trypco1→ Trypsin+EAD2+VMAP-M0+VMAP-C→ MoxR→ vWA+Pkinase+ParA-Soj-PloopNTPase(SIMIBI)*→</td><td>1150</td><td>Streptomyces sp. FXJ7.023</td><td>GCF_000404005.1</td><td>actinobacteria</td></tr>
<tr><td>WP_051159736.1</td><td>vWA+TPRs</td><td>AB_hydrolase→ CASPASE+EAD2+VMAP-M0+VMAP-C→ MoxR→ ||← ?||vWA+TPRs*→ ||← DUF397</td><td>1059</td><td>Streptomyces sp. FXJ7.023</td><td>GCF_000404005.1</td><td>actinobacteria</td></tr>
<tr><td>WP_051262220.1</td><td>vWA+TPRs</td><td>TCAD15→ ?→ ||← ?||Trypco1→ Trypsin+HDxxH+VMAP-M0+VMAP-C→ MoxR→ vWA+TPRs*→ FXSXX-COOH→ METALLOPROTEASE+TPRs→ TIR+APATPase+TPRs→</td><td>1043</td><td>Streptomyces sp. CNH287</td><td>GCF_000424825.1</td><td>actinobacteria</td></tr>
<tr><td>WP_051312162.1</td><td>vWA+Pkinase+ParA-Soj-PloopNTPase(SIMIBI)</td><td>Trypco1→ Trypsin+EAD2+VMAP-M0+VMAP-C→ MoxR→ vWA+Pkinase+ParA-Soj-PloopNTPase(SIMIBI)*→</td><td>1114</td><td>Streptomyces sp. CNQ865</td><td>GCF_000424805.1</td><td>actinobacteria</td></tr>
<tr><td>WP_051370568.1</td><td>vWA+Pkinase+wHTH+TPRs+cNMP_cyclase+Band7+HAD</td><td>Trypco1→ Trypsin+VMAP-M0+VMAP-C→ MoxR→ vWA+Pkinase+wHTH+TPRs+cNMP_cyclase+Band7+HAD*→ ?→ ?→ ?→ Radical_SAM→ Antibiotic_NAT→ TIR+FxsC_Cter→</td><td>1618</td><td>Streptomyces sp. 142MFCol3.1</td><td>GCF_000424945.1</td><td>actinobacteria</td></tr>
<tr><td>WP_051370807.1</td><td>vWA+TPRs</td><td>TCAD15||Trypco1→ Trypsin+HDxxH+VMAP-M0+VMAP-C→ MoxR→ vWA+TPRs*→ FXSXX-COOH→ Radical_SAM→ METALLOPROTEASE+TPRs→ TIR+APATPase+TPRs→</td><td>1096</td><td>Streptomyces sp. 142MFCol3.1</td><td>GCF_000424945.1</td><td>actinobacteria</td></tr>
<tr><td>WP_051387006.1</td><td>vWA+TPRs</td><td>MoxR→ vWA+TPRs*→</td><td>1096</td><td>Streptomyces sp. AW19M42</td><td>-</td><td>actinobacteria</td></tr>
<tr><td>WP_051433611.1</td><td>vWA+Pkinase+TPRs</td><td>Trypco2→ Trypsin+EAD2+VMAP-M0+VMAP-C→ MoxR→ vWA+Pkinase+TPRs*→</td><td>1134</td><td>Streptomyces niveus</td><td>GCF_000497425.1</td><td>actinobacteria</td></tr>
<tr><td>WP_051572814.1</td><td>vWA+CASPASE+TCAD13</td><td>AB_hydrolase→ CASPASE+VMAP-M2+VMAP-C→ MoxR→ vWA+CASPASE+TCAD13*→</td><td>1093</td><td>Streptomyces sp. PRh5</td><td>GCA_000590515.1</td><td>actinobacteria</td></tr>
<tr><td>WP_051647873.1</td><td>vWA+Pkinase+ParA-Soj-PloopNTPase(SIMIBI)</td><td>NUDIX→ Trypco1→ Trypsin+EAD2+VMAP-M0+VMAP-C→ MoxR→ vWA+Pkinase+ParA-Soj-PloopNTPase(SIMIBI)*→ SIR2→</td><td>1003</td><td>Streptomyces fradiae</td><td>GCA_001756925.1</td><td>actinobacteria</td></tr>
<tr><td>WP_051652644.1</td><td>vWA_protein</td><td>Trypsin+EAD2+VMAP-M0+VMAP-C→ PSE→ vWA_protein*→ BetaPropeller→</td><td>350</td><td>Kitasatospora cheerisanensis</td><td>GCF_000696185.1</td><td>actinobacteria</td></tr>
<tr><td>WP_051674211.1</td><td>vWA+TPRs</td><td>TCAD15||Trypsin+HDxxH+VMAP-M0+VMAP-C→ MoxR→ vWA+TPRs*→ FXSXX-COOH→ Radical_SAM→ METALLOPROTEASE+TPRs→ TIR+APATPase+TPRs→</td><td>1105</td><td>Streptomyces sp. URHA0041</td><td>GCF_000701325.1</td><td>actinobacteria</td></tr>
<tr><td>WP_051695577.1</td><td>vWA+TPRs</td><td>TCAD15||Trypsin+HDxxH+VMAP-M0+VMAP-C→ MoxR→ vWA+TPRs*→ FXSXX-COOH→ PSE→ METALLOPROTEASE+TPRs→ TIR+APATPase+TPRs→</td><td>1079</td><td>Streptomyces sp. NRRL S-244</td><td>GCF_000717915.1</td><td>actinobacteria</td></tr>
<tr><td>WP_051695727.1</td><td>vWA+Pkinase+ParA-Soj-PloopNTPase(SIMIBI)</td><td>Trypco1→ Trypsin+EAD2+VMAP-M0+VMAP-C→ MoxR→ vWA+Pkinase+ParA-Soj-PloopNTPase(SIMIBI)*→</td><td>1092</td><td>Streptomyces sp. NRRL S-244</td><td>GCF_000717915.1</td><td>actinobacteria</td></tr>
<tr><td>WP_051700487.1</td><td>vWA+TPRs</td><td>TCAD15||Trypsin+HDxxH+VMAP-M0+VMAP-C→ MoxR→ vWA+TPRs*→ FXSXX-COOH→</td><td>1104</td><td>Streptomyces sp. NRRL F-4474</td><td>GCF_000719855.1</td><td>actinobacteria</td></tr>
<tr><td>WP_051715921.1</td><td>vWA+TPRs</td><td>TCAD15||Trypco1→ Trypsin+HDxxH+VMAP-M0+VMAP-C→ MoxR→ vWA+TPRs*→ Radical_SAM→ TIR+APATPase+TPRs→</td><td>1106</td><td>Streptomyces sp. NRRL S-475</td><td>GCF_000720215.1</td><td>actinobacteria</td></tr>
<tr><td>WP_051716612.1</td><td>vWA+APATPase+wHTH+TPRs</td><td>EAD2+VMAP-M0+VMAP-C→ MoxR→ vWA+APATPase+wHTH+TPRs*→ ||← Na_H_Exchanger||AB_hydrolase→ CASPASE+EAD2→ FXSXX-COOH-like→ METALLOPROTEASE+TPRs→</td><td>1487</td><td>Streptomyces sp. NRRL F-5727</td><td>GCF_000719555.1</td><td>actinobacteria</td></tr>
<tr><td>WP_051716748.1</td><td>vWA+TPRs</td><td>Trypco1→ Trypsin+HDxxH+VMAP-M0+VMAP-C→ MoxR→ vWA+TPRs*→ METALLOPROTEASE+TPRs→ TIR+APATPase+TPRs→</td><td>1003</td><td>Streptomyces megasporus</td><td>GCF_000718985.1</td><td>actinobacteria</td></tr>
<tr><td>WP_051731479.1</td><td>vWA+TPRs</td><td>TCAD15||Trypco1→ Trypsin+HDxxH+VMAP-M0+VMAP-C→ MoxR→ vWA+TPRs*→ FXSXX-COOH→ Radical_SAM→ TIR+APATPase+TPRs→</td><td>1102</td><td>Streptomyces rimosus</td><td>GCA_000707925.1</td><td>actinobacteria</td></tr>
<tr><td>WP_051738500.1</td><td>vWA+TPRs</td><td>TCAD15||Trypco1→ Trypsin+HDxxH+VMAP-M0+VMAP-C→ MoxR→ vWA+TPRs*→ FXSXX-COOH→ Radical_SAM→ TIR+APATPase+TPRs→</td><td>1102</td><td>Streptomyces rimosus</td><td>GCA_000707965.1</td><td>actinobacteria</td></tr>
<tr><td>WP_051740295.1</td><td>vWA+CASPASE+TCAD6+cNMP_cyclase+Antibiotic_NAT</td><td>AB_hydrolase→ CASPASE+VMAP-M2+VMAP-C→ MoxR→ vWA+CASPASE+TCAD6+cNMP_cyclase+Antibiotic_NAT*→</td><td>1375</td><td>Kitasatospora sp. MBT66</td><td>GCF_000710405.2</td><td>actinobacteria</td></tr>
<tr><td>WP_051744676.1</td><td>vWA+Pkinase+wHTH+TPRs+cNMP_cyclase+Band7+HAD</td><td>Trypco1→ PSE→ MoxR→ vWA+Pkinase+wHTH+TPRs+cNMP_cyclase+Band7+HAD*→</td><td>1574</td><td>Streptomyces yerevanensis</td><td>GCF_000716805.1</td><td>actinobacteria</td></tr>
<tr><td>WP_051744857.1</td><td>vWA+APATPase+wHTH+TPRs</td><td>EAD2+VMAP-M0+VMAP-C→ vWA+APATPase+wHTH+TPRs*→ ||← METALLOPROTEASE+TPRs← Trypsin+EAD2← Trypco2← ?||?→ DUF397→</td><td>1317</td><td>Streptomyces yerevanensis</td><td>-</td><td>actinobacteria</td></tr>
<tr><td>WP_051756012.1</td><td>vWA+CASPASE+TCAD6+cNMP_cyclase+Antibiotic_NAT</td><td>AB_hydrolase→ ?→ ||← ?||MoxR→ vWA+CASPASE+TCAD6+cNMP_cyclase+Antibiotic_NAT*→</td><td>1381</td><td>Streptomyces purpeofuscus</td><td>GCF_000718025.1</td><td>actinobacteria</td></tr>
<tr><td>WP_051761502.1</td><td>vWA+TIR+FxsC_Cter</td><td>Trypco1→ Trypsin+VMAP-M8+VMAP-C→ MoxR→ vWA+TIR+FxsC_Cter*→</td><td>939</td><td>Microbispora rosea</td><td>GCF_000718475.1</td><td>actinobacteria</td></tr>
<tr><td>WP_051763106.1</td><td>vWA+TPRs</td><td>TCAD15||Trypsin+HDxxH+VMAP-M0+VMAP-C→ MoxR→ vWA+TPRs*→ FXSXX-COOH→ Radical_SAM→ METALLOPROTEASE+TPRs→ TIR+APATPase+TPRs→</td><td>1083</td><td>Streptomyces virginiae</td><td>GCF_000716685.1</td><td>actinobacteria</td></tr>
<tr><td>WP_051766461.1</td><td>vWA+APATPase+wHTH+TPRs</td><td>EAD2+VMAP-M0+VMAP-C→ MoxR→ vWA+APATPase+wHTH+TPRs*→ ||← METALLOPROTEASE+TPRs← FXSXX-COOH-like||?→ ||← ?||EAD2+VMAP-M0+VMAP-C→ MoxR→ vWA+APATPase+wHTH+TPRs→</td><td>1409</td><td>Saccharothrix syringae</td><td>GCF_000716755.1</td><td>actinobacteria</td></tr>
<tr><td>WP_051770902.1</td><td>vWA+APATPase+SWACOS+wHTH+TPRs+Beta-rich-tail</td><td>Trypco1→ Trypsin+EAD2→ EAD2+VMAP-M10+VMAP-C→ MoxR→ vWA+APATPase+SWACOS+wHTH+TPRs+Beta-rich-tail*→</td><td>1569</td><td>Streptomyces sp. NRRL F-6131</td><td>GCF_000719695.1</td><td>actinobacteria</td></tr>
<tr><td>WP_051772444.1</td><td>vWA+APATPase+wHTH+TPRs</td><td>EAD2+VMAP-M0+VMAP-C→ vWA+APATPase+wHTH+TPRs*→ ||← METALLOPROTEASE+TPRs← cNMP_cyclase+EAD2</td><td>1242</td><td>Saccharothrix sp. NRRL B-16314</td><td>GCF_000716595.1</td><td>actinobacteria</td></tr>
<tr><td>WP_051772626.1</td><td>vWA+APATPase+wHTH+TPRs</td><td>EAD2+VMAP-M0+VMAP-C→ MoxR→ vWA+APATPase+wHTH+TPRs*→ ?→ ||← METALLOPROTEASE+TPRs</td><td>1445</td><td>Saccharothrix sp. NRRL B-16314</td><td>GCF_000716595.1</td><td>actinobacteria</td></tr>
<tr><td>WP_051774580.1</td><td>vWA+TPRs</td><td>TCAD15||Trypsin+HDxxH+VMAP-M0+VMAP-C→ MoxR→ vWA+TPRs*→ FXSXX-COOH→ Radical_SAM→ METALLOPROTEASE+TPRs→ TIR+APATPase+TPRs→</td><td>1082</td><td>Streptomyces sp. NRRL S-237</td><td>GCF_000718935.1</td><td>actinobacteria</td></tr>
<tr><td>WP_051777157.1</td><td>vWA+Pkinase+ParA-Soj-PloopNTPase(SIMIBI)</td><td>Trypco1→ Trypsin+EAD2+VMAP-M0+VMAP-C→ MoxR→ vWA+Pkinase+ParA-Soj-PloopNTPase(SIMIBI)*→</td><td>1111</td><td>Streptomyces violaceorubidus</td><td>GCF_000717995.1</td><td>actinobacteria</td></tr>
<tr><td>WP_051777805.1</td><td>vWA+CASPASE+TCAD14+PIN+CCCH(ZNR)+FHA</td><td>Trypco1→ Trypsin+HDxxH+VMAP-M0+VMAP-C→ MoxR→ vWA+CASPASE+TCAD14+PIN+CCCH(ZNR)+FHA*→</td><td>1267</td><td>Kitasatospora phosalacinea</td><td>GCF_000717185.1</td><td>actinobacteria</td></tr>
<tr><td>WP_051778639.1</td><td>vWA+Coiled-coil+TIR+FxsC_Cter(partial)</td><td>Trypco1→ Trypsin+EAD2+VMAP-M0+VMAP-C→ MoxR→ vWA+Coiled-coil+TIR+FxsC_Cter(partial)*→</td><td>815</td><td>Kitasatospora phosalacinea</td><td>GCF_000717185.1</td><td>actinobacteria</td></tr>
<tr><td>WP_051779191.1</td><td>vWA+TPRs</td><td>TCAD15||Trypsin+HDxxH+VMAP-M0+VMAP-C→ MoxR→ vWA+TPRs*→ FXSXX-COOH→ ?→ Radical_SAM→</td><td>1132</td><td>Streptomyces sp. NRRL S-241</td><td>GCF_000717925.1</td><td>actinobacteria</td></tr>
<tr><td>WP_051781725.1</td><td>vWA+TPRs</td><td>TCAD15||Trypsin+HDxxH+VMAP-M0+VMAP-C→ MoxR→ vWA+TPRs*→ FXSXX-COOH→ Radical_SAM→ METALLOPROTEASE+TPRs→ TIR+APATPase+TPRs→</td><td>1083</td><td>Streptomyces</td><td>GCF_000720795.1</td><td>actinobacteria</td></tr>
<tr><td>WP_051783241.1</td><td>vWA+Pkinase+ParA-Soj-PloopNTPase(SIMIBI)</td><td>Trypco1→ Trypsin+EAD2+VMAP-M0+VMAP-C→ MoxR→ vWA+Pkinase+ParA-Soj-PloopNTPase(SIMIBI)*→</td><td>1116</td><td>Streptomyces</td><td>GCF_001417675.1</td><td>actinobacteria</td></tr>
<tr><td>WP_051785085.1</td><td>vWA+TPRs</td><td>TCAD15||Trypsin+HDxxH+VMAP-M0+VMAP-C→ MoxR→ vWA+TPRs*→ FXSXX-COOH→ Radical_SAM→ METALLOPROTEASE+TPRs→ TIR+APATPase+TPRs→</td><td>1120</td><td>Streptomyces virginiae</td><td>GCF_000720455.1</td><td>actinobacteria</td></tr>
<tr><td>WP_051787118.1</td><td>vWA+APATPase+wHTH+TPRs</td><td>EAD2+VMAP-M0+VMAP-C→ MoxR→ vWA+APATPase+wHTH+TPRs*→ ||← Na_H_Exchanger||AB_hydrolase→ CASPASE+EAD2→ FXSXX-COOH-like→ METALLOPROTEASE+TPRs→</td><td>1500</td><td>Streptomyces</td><td>GCF_000716445.1</td><td>actinobacteria</td></tr>
<tr><td>WP_051787936.1</td><td>vWA+APATPase+wHTH+TPRs</td><td>EAD2+VMAP-M0+VMAP-C→ MoxR→ vWA+APATPase+wHTH+TPRs*→</td><td>1470</td><td>Microtetraspora glauca</td><td>-</td><td>actinobacteria</td></tr>
<tr><td>WP_051790540.1</td><td>vWA+Pkinase+wHTH+TPRs</td><td>Trypco1→ Trypsin+VMAP-M0+VMAP-C→ MoxR→ vWA+Pkinase+wHTH+TPRs*→</td><td>1081</td><td>Streptomyces sp. NRRL S-1022</td><td>GCF_000720135.1</td><td>actinobacteria</td></tr>
</table>

<table>
<tr><th>acc</th><th>architecture</th><th>operon</th><th>len</th><th>species</th><th>gca</th><th>taxend</th></tr>
<tr><td>WP_051796644.1</td><td>vWA+APATPase+wHTH+TPRs</td><td>-</td><td>1187</td><td>Rhodococcus rhodnii</td><td>-</td><td>actinobacteria</td></tr>
<tr><td>WP_051796738.1</td><td>vWA+TPRs</td><td>TCAD15||Trypco1→ Trypsin+HDxxH+VMAP-M0+VMAP-C→ MoxR→ vWA+TPRs*→ FXSXX-COOH→ Radical_SAM→ METALLOPROTEASE+TPRs→ TIR+APATPase+TPRs→</td><td>1111</td><td>Streptomyces sp. NRRL S-87</td><td>-</td><td>actinobacteria</td></tr>
<tr><td>WP_051797194.1</td><td>vWA+APATPase+wHTH+TPRs</td><td>METALLOPROTEASE+TPRs→ EAD2+VMAP-M0+VMAP-C→ vWA+APATPase+wHTH+TPRs*→</td><td>1241</td><td>Catenuloplanes japonicus</td><td>GCF_000717135.1</td><td>actinobacteria</td></tr>
<tr><td>WP_051804663.1</td><td>vWA+APATPase+wHTH+TPRs</td><td>EAD2+VMAP-M0+VMAP-C→ MoxR→ vWA+APATPase+wHTH+TPRs*→ ||← Na_H_Exchanger||AB_hydrolase→ CASPASE+EAD2→ FXSXX-COOH-like→ METALLOPROTEASE+TPRs→</td><td>1476</td><td>Streptomyces griseus</td><td>GCF_000718525.1</td><td>actinobacteria</td></tr>
<tr><td>WP_051805087.1</td><td>vWA+TPRs</td><td>TCAD15||Trypsin+HDxxH+VMAP-M0+VMAP-C→ MoxR→ vWA+TPRs*→ FXSXX-COOH→ Radical_SAM→ METALLOPROTEASE+TPRs→ TIR+APATPase+TPRs→</td><td>1075</td><td>Streptomyces sp. NRRL F-2747</td><td>GCF_000720145.1</td><td>actinobacteria</td></tr>
<tr><td>WP_051807270.1</td><td>vWA+APATPase+wHTH+TPRs</td><td>PNP_UDP_1+VMAP-M0+VMAP-C→ MoxR→ vWA+APATPase+wHTH+TPRs*→ ||← METALLOPROTEASE+TPRs</td><td>1428</td><td>Actinoplanes subtropicus</td><td>GCF_000721705.1</td><td>actinobacteria</td></tr>
<tr><td>WP_051807479.1</td><td>vWA+TPRs</td><td>TCAD15||Trypsin+HDxxH+VMAP-M0+VMAP-C→ MoxR→ vWA+TPRs*→</td><td>1122</td><td>Streptomyces sp. NRRL F-2664</td><td>GCF_000719345.1</td><td>actinobacteria</td></tr>
<tr><td>WP_051810361.1</td><td>vWA+TPRs</td><td>vWA+TPRs*→ FXSXX-COOH→</td><td>1134</td><td>Streptomyces sp. NRRL S-378</td><td>-</td><td>actinobacteria</td></tr>
<tr><td>WP_051810959.1</td><td>vWA+Pkinase+FtsK+TPRs</td><td>ADP_ribosyl_GH→ Trypco1→ Trypsin+EAD2+VMAP-M0+VMAP-C→ PSE→ PSE→ vWA+Pkinase+FtsK+TPRs*→ NUDIX→</td><td>1401</td><td>Streptomyces</td><td>GCF_000716675.1</td><td>actinobacteria</td></tr>
<tr><td>WP_051811665.1</td><td>vWA+Pkinase+wHTH+TPRs+cNMP_cyclase+HAD</td><td>MoxR→ vWA+Pkinase+wHTH+TPRs+cNMP_cyclase+HAD*→</td><td>1496</td><td>Streptomyces</td><td>GCF_000716675.1</td><td>actinobacteria</td></tr>
<tr><td>WP_051814235.1</td><td>vWA+APATPase+wHTH+TPRs</td><td>Trypsin+EAD2→ EAD2+VMAP-M0+VMAP-C→ MoxR→ vWA+APATPase+wHTH+TPRs*→ ||← METALLOPROTEASE+TPRs← Trypco1</td><td>1374</td><td>Streptomyces iakyrus</td><td>-</td><td>actinobacteria</td></tr>
<tr><td>WP_051820561.1</td><td>vWA+TPRs</td><td>TCAD15||Trypco1→ Trypsin+HDxxH+VMAP-M0+VMAP-C→ MoxR→ vWA+TPRs*→ FXSXX-COOH→ METALLOPROTEASE+TPRs→ TIR+APATPase+TPRs→</td><td>1085</td><td>Streptomyces flavochromogenes</td><td>GCF_000717595.1</td><td>actinobacteria</td></tr>
<tr><td>WP_051820772.1</td><td>vWA+Pkinase+ParA-Soj-PloopNTPase(SIMIBI)</td><td>Trypco1→ Trypsin+EAD2+VMAP-M0+VMAP-C→ MoxR→ vWA+Pkinase+ParA-Soj-PloopNTPase(SIMIBI)*→</td><td>1104</td><td>Streptomyces flavochromogenes</td><td>GCF_000717595.1</td><td>actinobacteria</td></tr>
<tr><td>WP_051823228.1</td><td>vWA+wHTH+TPRs+wHTH+TPRs</td><td>Trypsin+EAD2+VMAP-M0+VMAP-C→ MoxR→ vWA+wHTH+TPRs+wHTH+TPRs*→ ||← AB_hydrolase||?→ ||← ?||?→ ||← PSE← ?← DUF397</td><td>1364</td><td>Streptomyces sp. NRRL S-1448</td><td>-</td><td>actinobacteria</td></tr>
<tr><td>WP_051828049.1</td><td>vWA+Pkinase+ParA-Soj-PloopNTPase(SIMIBI)</td><td>Trypco1→ Trypsin+EAD2+VMAP-M0+VMAP-C→ MoxR→ vWA+Pkinase+ParA-Soj-PloopNTPase(SIMIBI)*→</td><td>1132</td><td>Streptomyces bicolor</td><td>GCF_000725465.1</td><td>actinobacteria</td></tr>
<tr><td>WP_051830823.1</td><td>vWA+SWACOS+wHTH</td><td>Trypco1→ Trypsin+EAD2+VMAP-M0+VMAP-C→ MoxR→ vWA+SWACOS+wHTH*→</td><td>1279</td><td>Streptomyces violens</td><td>GCF_000717745.1</td><td>actinobacteria</td></tr>
<tr><td>WP_051831367.1</td><td>vWA+TPRs</td><td>TCAD15← ?||Trypsin+HDxxH+VMAP-M0+VMAP-C→ MoxR→ vWA+TPRs*→ FXSXX-COOH→ Radical_SAM→ METALLOPROTEASE+TPRs→ TIR+APATPase+TPRs→ TIR+APATPase+TPRs→</td><td>1093</td><td>Streptomyces violens</td><td>GCF_000717745.1</td><td>actinobacteria</td></tr>
<tr><td>WP_051831996.1</td><td>vWA+TPRs</td><td>TCAD15||Trypsin+HDxxH+VMAP-M0+VMAP-C→ MoxR→ vWA+TPRs*→ FXSXX-COOH→ Radical_SAM→ METALLOPROTEASE+TPRs→ TIR+APATPase+TPRs→</td><td>1081</td><td>Streptomyces katrae</td><td>GCF_000717655.1</td><td>actinobacteria</td></tr>
<tr><td>WP_051833218.1</td><td>vWA+TIR+APATPase+Trypsin+HDxxH+VMAP-M0+VMAP-C</td><td>TCAD15||Trypco1→ Trypsin+HDxxH+VMAP-M0+VMAP-C→ MoxR→ vWA+TIR+APATPase+Trypsin+HDxxH+VMAP-M0+VMAP-C*→</td><td>1434</td><td>Streptomyces sp. NRRL S-646</td><td>GCF_000720765.1</td><td>actinobacteria</td></tr>
<tr><td>WP_051836728.1</td><td>vWA+TPRs</td><td>TCAD15||Trypsin+HDxxH+VMAP-M0+VMAP-C→ MoxR→ vWA+TPRs*→ FXSXX-COOH→ Radical_SAM→ METALLOPROTEASE+TPRs→ TIR+APATPase+TPRs→</td><td>1148</td><td>Streptomyces sp. NRRL F-2580</td><td>GCF_000720115.1</td><td>actinobacteria</td></tr>
<tr><td>WP_051837519.1</td><td>vWA+TIR+FxsC_Cter</td><td>Trypco1→ Trypsin+VMAP-M0+VMAP-C→ vWA+TIR+FxsC_Cter*→</td><td>809</td><td>Streptomyces sp. NRRL WC-3742</td><td>GCF_000720635.1</td><td>actinobacteria</td></tr>
<tr><td>WP_051840310.1</td><td>vWA+TPRs</td><td>TCAD15||Trypsin+HDxxH+VMAP-M0+VMAP-C→ MoxR→ vWA+TPRs*→ FXSXX-COOH→ Radical_SAM→ METALLOPROTEASE+TPRs→ TIR+APATPase+TPRs→</td><td>1117</td><td>Streptomyces lavendulae</td><td>GCA_002803845.1</td><td>actinobacteria</td></tr>
<tr><td>WP_051842349.1</td><td>vWA+APATPase+wHTH+TPRs</td><td>EAD2+VMAP-M0+VMAP-C→ MoxR→ vWA+APATPase+wHTH+TPRs*→ ||← Na_H_Exchanger||AB_hydrolase→ CASPASE+EAD2→ FXSXX-COOH-like→ METALLOPROTEASE+TPRs→</td><td>1495</td><td>Streptomyces sp. NRRL F-5193</td><td>GCF_000721515.1</td><td>actinobacteria</td></tr>
<tr><td>WP_051842718.1</td><td>vWA+Pkinase+wHTH+TPRs+cNMP_cyclase+Band7+HAD</td><td>Trypco1→ Trypsin+VMAP-M0+VMAP-C→ MoxR→ vWA+Pkinase+wHTH+TPRs+cNMP_cyclase+Band7+HAD*→</td><td>1659</td><td>Streptomyces</td><td>-</td><td>actinobacteria</td></tr>
<tr><td>WP_051846592.1</td><td>vWA+Trypsin+HDxxH+VMAP-M0+VMAP-C</td><td>TCAD15→ ?→ ?→ PSE→ ?→ MoxR→ vWA+Trypsin+HDxxH+VMAP-M0+VMAP-C*→ PSE→ MoxR→ vWA+TPRs→ FXSXX-COOH→ METALLOPROTEASE+TPRs→ TIR+APATPase+TPRs→</td><td>1251</td><td>Streptomyces sp. NRRL F-5053</td><td>GCF_000721445.1</td><td>actinobacteria</td></tr>
<tr><td>WP_051847209.1</td><td>vWA+Pkinase+ParA-Soj-PloopNTPase(SIMIBI)</td><td>Trypco1→ PSE→ vWA+Pkinase+ParA-Soj-PloopNTPase(SIMIBI)*→</td><td>1174</td><td>Streptomyces sp. NRRL F-5053</td><td>GCF_000721445.1</td><td>actinobacteria</td></tr>
<tr><td>WP_051850261.1</td><td>vWA+APATPase+wHTH+TPRs</td><td>Trypco2→ EAD2+VMAP-M0+VMAP-C→ vWA+APATPase+wHTH+TPRs*→ ||← METALLOPROTEASE+TPRs← Trypsin+EAD2← HTH_31</td><td>1321</td><td>Streptomyces griseofuscus</td><td>GCF_000718315.1</td><td>actinobacteria</td></tr>
<tr><td>WP_051850820.1</td><td>vWA+Pkinase+wHTH+TPRs+cNMP_cyclase</td><td>Trypco1→ Trypsin+VMAP-M0+VMAP-C→ MoxR→ vWA+Pkinase+wHTH+TPRs+cNMP_cyclase*→</td><td>1272</td><td>Streptomyces sp. NRRL WC-3795</td><td>GCF_000720675.1</td><td>actinobacteria</td></tr>
<tr><td>WP_051850937.1</td><td>vWA+Pkinase+ParA-Soj-PloopNTPase(SIMIBI)</td><td>Trypco1→ Trypsin+EAD2+VMAP-M0+VMAP-C→ MoxR→ vWA+Pkinase+ParA-Soj-PloopNTPase(SIMIBI)*→</td><td>1113</td><td>Streptomyces sp. NRRL WC-3795</td><td>GCF_000720675.1</td><td>actinobacteria</td></tr>
<tr><td>WP_051851738.1</td><td>vWA+Pkinase+ParA-Soj-PloopNTPase(SIMIBI)</td><td>Trypco1→ Trypsin+EAD2+VMAP-M0+VMAP-C→ MoxR→ vWA+Pkinase+ParA-Soj-PloopNTPase(SIMIBI)*→</td><td>1116</td><td>Streptomyces sp. NRRL F-5650</td><td>GCF_000721365.1</td><td>actinobacteria</td></tr>
<tr><td>WP_051854418.1</td><td>vWA+APATPase+wHTH+TPRs</td><td>Trypco1→ Trypsin+VMAP-M8+VMAP-C→ MoxR→ vWA+APATPase+wHTH+TPRs*→</td><td>1472</td><td>Streptomyces sp. NRRL B-1347</td><td>GCF_000720325.1</td><td>actinobacteria</td></tr>
<tr><td>WP_051854856.1</td><td>vWA+Pkinase+ParA-Soj-PloopNTPase(SIMIBI)</td><td>DUF397→ ||← ?||Trypco1→ Trypsin+EAD2+VMAP-M0+VMAP-C→ MoxR→ vWA+Pkinase+ParA-Soj-PloopNTPase(SIMIBI)*→</td><td>1129</td><td>Streptomyces sp. NRRL B-1347</td><td>GCF_000720325.1</td><td>actinobacteria</td></tr>
<tr><td>WP_051855892.1</td><td>vWA+Pkinase+wHTH+TPRs+cNMP_cyclase+HAD</td><td>Trypco1→ Trypsin+VMAP-M0+VMAP-C→ MoxR→ vWA+Pkinase+wHTH+TPRs+cNMP_cyclase+HAD*→</td><td>1325</td><td>Streptomyces sp. NRRL B-1347</td><td>GCF_000720325.1</td><td>actinobacteria</td></tr>
<tr><td>WP_051857016.1</td><td>vWA+Pkinase+ParA-Soj-PloopNTPase(SIMIBI)</td><td>Trypco1→ Trypsin+EAD2+VMAP-M0+VMAP-C→ MoxR→ vWA+Pkinase+ParA-Soj-PloopNTPase(SIMIBI)*→</td><td>1174</td><td>Streptomyces sp. NRRL S-1868</td><td>GCF_000721305.1</td><td>actinobacteria</td></tr>
<tr><td>WP_051857985.1</td><td>vWA+Pkinase+ParA-Soj-PloopNTPase(SIMIBI)</td><td>Trypco1→ Trypsin+EAD2+VMAP-M0+VMAP-C→ MoxR→ vWA+Pkinase+ParA-Soj-PloopNTPase(SIMIBI)*→</td><td>1109</td><td>Streptomyces toyocaensis</td><td>GCF_000725125.1</td><td>actinobacteria</td></tr>
<tr><td>WP_051858348.1</td><td>vWA+Pkinase+wHTH+TPRs+cNMP_cyclase+Band7+HAD</td><td>Trypco1→ Trypsin+VMAP-M0+VMAP-C→ MoxR→ vWA+Pkinase+wHTH+TPRs+cNMP_cyclase+Band7+HAD*→</td><td>1632</td><td>Streptomyces toyocaensis</td><td>GCA_000725125.1</td><td>actinobacteria</td></tr>
<tr><td>WP_051859650.1</td><td>vWA+TPRs</td><td>TCAD15||Trypsin+HDxxH+VMAP-M0+VMAP-C→ MoxR→ vWA+TPRs*→ FXSXX-COOH→ Radical_SAM→ METALLOPROTEASE+TPRs→ TIR+APATPase+TPRs→</td><td>1214</td><td>Streptomyces xanthophaeus</td><td>GCF_000725725.1</td><td>actinobacteria</td></tr>
<tr><td>WP_051861419.1</td><td>vWA+TPRs</td><td>TCAD15||PSE→ Trypsin+HDxxH+VMAP-M0+VMAP-C→ MoxR→ vWA+TPRs*→ FXSXX-COOH→ Radical_SAM→ METALLOPROTEASE+TPRs→ TIR+APATPase+TPRs→ TIR+APATPase+TPRs→</td><td>1092</td><td>Streptomyces ochraceiscleroticus</td><td>-</td><td>actinobacteria</td></tr>
<tr><td>WP_051862305.1</td><td>vWA+SWACOS+wHTH</td><td>Trypco1→ Trypsin+EAD2+VMAP-M0+VMAP-C→ MoxR→ vWA+SWACOS+wHTH*→</td><td>1268</td><td>Streptomyces ochraceiscleroticus</td><td>GCF_000720485.1</td><td>actinobacteria</td></tr>
<tr><td>WP_051865813.1</td><td>vWA+APATPase+wHTH+TPRs</td><td>Trypco2→ ?→ EAD2+VMAP-M0+VMAP-C→ vWA+APATPase+wHTH+TPRs*→ ||← METALLOPROTEASE+TPRs← Trypsin+EAD2</td><td>1330</td><td>Streptomyces griseus</td><td>GCF_000720255.1</td><td>actinobacteria</td></tr>
<tr><td>WP_051872174.1</td><td>vWA+TPRs</td><td>TCAD15||PSE→ Trypsin+HDxxH+VMAP-M0+VMAP-C→ MoxR→ vWA+TPRs*→ FXSXX-COOH→ Radical_SAM→ METALLOPROTEASE+TPRs→ TIR+APATPase+TPRs→ TIR+APATPase+TPRs→</td><td>1096</td><td>Streptomyces sclerotialus</td><td>-</td><td>actinobacteria</td></tr>
<tr><td>WP_051885844.1</td><td>vWA+TPRs</td><td>AB_hydrolase→ CASPASE+EAD2+VMAP-M0+VMAP-C→ MoxR→ vWA+TPRs*→</td><td>1335</td><td>Streptomyces hygroscopicus</td><td>GCF_000721535.1</td><td>actinobacteria</td></tr>
<tr><td>WP_051893194.1</td><td>vWA+TPRs</td><td>TCAD15||Trypsin+HDxxH+VMAP-M0+VMAP-C→ MoxR→ vWA+TPRs*→ FXSXX-COOH→ ?→ Radical_SAM→ METALLOPROTEASE+TPRs→ TIR+APATPase+TPRs→</td><td>1113</td><td>Streptomyces erythrochromogenes</td><td>GCF_000725555.1</td><td>actinobacteria</td></tr>
</table>

<table>
<tr><th>acc</th><th>architecture</th><th>operon</th><th>len</th><th>species</th><th>gca</th><th>taxend</th></tr>
<tr><td>WP_051902870.1</td><td>vWA+TPRs</td><td>TCAD15||Trypsin+HDxxH+VMAP-M0+VMAP-C→ MoxR→ vWA+TPRs*→ FXSXX-COOH→ Radical_SAM→ METALLOPROTEASE+TPRs→ TIR+APATPase+TPRs→</td><td>1308</td><td>Streptomyces xanthophaeus</td><td>GCF_000725805.1</td><td>actinobacteria</td></tr>
<tr><td>WP_051905926.1</td><td>vWA+REase+cNMP_cyclase+CASPASE</td><td>SIR2→ ?→ ||← ?||AB_hydrolase→ CASPASE+EAD2+VMAP-M0+VMAP-C→ MoxR→ vWA+REase+cNMP_cyclase+CASPASE*→</td><td>1176</td><td>Streptomyces</td><td>GCF_000725785.1</td><td>actinobacteria</td></tr>
<tr><td>WP_051926349.1</td><td>vWA+Pkinase+wHTH+TPRs+cNMP_cyclase+Band7+HAD</td><td>Trypco1→ Trypsin+VMAP-M0+VMAP-C→ MoxR→ vWA+Pkinase+wHTH+TPRs+cNMP_cyclase+Band7+HAD*→</td><td>1613</td><td>Streptomyces durhamensis</td><td>GCF_000725475.1</td><td>actinobacteria</td></tr>
<tr><td>WP_051927217.1</td><td>vWA+Pkinase+TIR+APATPase+wHTH+TPRs</td><td>Trypco1→ Trypsin+EAD2+VMAP-M0+VMAP-C→ MoxR→ vWA+Pkinase+TIR+APATPase+wHTH+TPRs*→</td><td>1512</td><td>Streptomyces durhamensis</td><td>GCF_000725475.1</td><td>actinobacteria</td></tr>
<tr><td>WP_051958240.1</td><td>vWA+PNP_UDP_1+APATPase+wHTH+TPRs</td><td>AB_hydrolase→ CASPASE+EAD2+VMAP-M0+VMAP-C→ MoxR→ vWA+PNP_UDP_1+APATPase+wHTH+TPRs*→</td><td>1506</td><td>Streptomyces mirabilis</td><td>GCF_000745715.1</td><td>actinobacteria</td></tr>
<tr><td>WP_052030314.1</td><td>vWA+TPRs</td><td>TCAD15||Trypsin+HDxxH+VMAP-M0+VMAP-C→ MoxR→ vWA+TPRs*→ FXSXX-COOH→ Radical_SAM→ METALLOPROTEASE+TPRs→ TIR+APATPase+TPRs→</td><td>1104</td><td>Streptomyces sp. PCS3-D2</td><td>GCF_000612545.1</td><td>actinobacteria</td></tr>
<tr><td>WP_052146298.1</td><td>vWA+Pkinase+wHTH+TPRs+cNMP_cyclase</td><td>Trypco1→ Trypsin+VMAP-M0+VMAP-C→ MoxR→ vWA+Pkinase+wHTH+TPRs+cNMP_cyclase*→ ?→ ?→ ||← ?||Radical_SAM→ Antibiotic_NAT→ TIR+FxsC_Cter→</td><td>979</td><td>Streptomyces galbus</td><td>GCF_000772895.1</td><td>actinobacteria</td></tr>
<tr><td>WP_052164025.1</td><td>vWA+CASPASE(partial)</td><td>AB_hydrolase→ CASPASE+EAD2→ VMAP-M0+VMAP-C→ MoxR→ vWA+CASPASE(partial)*→</td><td>791</td><td>Actinoplanes utahensis</td><td>GCF_000785985.1</td><td>actinobacteria</td></tr>
<tr><td>WP_052164720.1</td><td>vWA+cNMP_cyclase+HTH+HTH+TPRs</td><td>EAD2+VMAP-M0+VMAP-C→ MoxR→ vWA+cNMP_cyclase+HTH+HTH+TPRs*→</td><td>1283</td><td>Actinoplanes utahensis</td><td>GCF_000785985.1</td><td>actinobacteria</td></tr>
<tr><td>WP_052183279.1</td><td>vWA+Pkinase+wHTH+TPRs+cNMP_cyclase+Band7</td><td>Trypco1→ Trypsin+VMAP-M0+VMAP-C→ MoxR→ vWA+Pkinase+wHTH+TPRs+cNMP_cyclase+Band7*→</td><td>1343</td><td>Streptomyces sp. UNC401CLCol</td><td>GCF_000686765.1</td><td>actinobacteria</td></tr>
<tr><td>WP_052261118.1</td><td>vWA+BetaPropeller+TCAD10+REC</td><td>AB_hydrolase→ CASPASE+VMAP-M16+VMAP-C→ MoxR→ vWA+BetaPropeller+TCAD10+REC*→</td><td>1211</td><td>Pseudomonas sp. C5pp</td><td>GCF_000814065.1</td><td>gammaproteobacteria</td></tr>
<tr><td>WP_052310350.1</td><td>vWA+TPRs</td><td>TCAD15||Trypco1→ Trypsin+HDxxH+VMAP-M0+VMAP-C→ MoxR→ vWA+TPRs*→</td><td>997</td><td>Streptomyces viridochromogenes</td><td>GCF_000158955.1</td><td>actinobacteria</td></tr>
<tr><td>WP_052330862.1</td><td>vWA+FGS-fold_domain</td><td>-</td><td>888</td><td>Rivularia sp. PCC 7116</td><td>-</td><td>cyanobacteria</td></tr>
<tr><td>WP_052360456.1</td><td>vWA+TIR+BetaPropeller</td><td>EAD2+VMAP-M0+VMAP-C→ MoxR→ ?→ vWA+TIR+BetaPropeller*→ ?→ ?→ ||← cNMP_cyclase+EAD2||cNMP_binding+HTH_Crp_2→ ?→ SIR2→</td><td>1194</td><td>Kutzneria albida</td><td>GCF_000525635.1</td><td>actinobacteria</td></tr>
<tr><td>WP_052390345.1</td><td>vWA+CASPASE+TIR+FxsC_Cter(partial)</td><td>Trypco1→ Trypsin+VMAP-M0+VMAP-C→ MoxR→ vWA+CASPASE+TIR+FxsC_Cter(partial)*→</td><td>1010</td><td>Streptomyces sp. NRRL B-24484</td><td>-</td><td>actinobacteria</td></tr>
<tr><td>WP_052390618.1</td><td>vWA+TPRs</td><td>Trypco1→ Trypsin+EAD2+VMAP-M0+VMAP-C→ MoxR→ vWA+TPRs*→</td><td>881</td><td>Streptomyces sp. NRRL B-24484</td><td>GCF_000717715.1</td><td>actinobacteria</td></tr>
<tr><td>WP_052398034.1</td><td>vWA+APATPase+wHTH+BetaPropeller</td><td>Trypsin+EAD2+VMAP-M0+VMAP-C→ MoxR→ vWA+APATPase+wHTH+BetaPropeller*→</td><td>1462</td><td>Streptomyces sp. NRRL F-5123</td><td>GCF_000721495.1</td><td>actinobacteria</td></tr>
<tr><td>WP_052410846.1</td><td>vWA+Pkinase+ParA-Soj-PloopNTPase(SIMIBI)</td><td>Trypco1→ Trypsin+EAD2+VMAP-M0+VMAP-C→ MoxR→ vWA+Pkinase+ParA-Soj-PloopNTPase(SIMIBI)*→</td><td>1150</td><td>Streptomyces olivaceus</td><td>GCF_000721235.1</td><td>actinobacteria</td></tr>
<tr><td>WP_052411496.1</td><td>vWA+NACHT+CASPASE+BetaPropeller</td><td>Trypco1→ MoxR→ vWA+NACHT+CASPASE+BetaPropeller*→</td><td>1448</td><td>Streptomyces sp. NRRL S-118</td><td>GCF_000716335.1</td><td>actinobacteria</td></tr>
<tr><td>WP_052411905.1</td><td>vWA+Pkinase+ParA-Soj-PloopNTPase(SIMIBI)</td><td>Trypsin+EAD2+VMAP-M0+VMAP-C→ MoxR→ vWA+Pkinase+ParA-Soj-PloopNTPase(SIMIBI)*→</td><td>974</td><td>Streptomyces sp. NRRL S-118</td><td>GCF_000716335.1</td><td>actinobacteria</td></tr>
<tr><td>WP_052413856.1</td><td>vWA+TIR+APATPase+Trypsin+HDxxH+VMAP-M0+VMAP-C</td><td>TCAD15||PSE→ Trypsin+HDxxH+VMAP-M0+VMAP-C→ MoxR→ vWA+TIR+APATPase+Trypsin+HDxxH+VMAP-M0+VMAP-C*→</td><td>1343</td><td>Streptomyces glaucescens</td><td>GCA_000761215.1</td><td>actinobacteria</td></tr>
<tr><td>WP_052424449.1</td><td>vWA+Pkinase+STAND_NTPase+STAND_NTPase</td><td>Trypco1→ Trypsin+HDxxH+VMAP-M0+VMAP-C→ MoxR→ vWA+Pkinase+STAND_NTPase+STAND_NTPase*→</td><td>1335</td><td>Streptomyces fulvoviolaceus</td><td>GCF_000718165.1</td><td>actinobacteria</td></tr>
<tr><td>WP_052479908.1</td><td>vWA+APATPase+wHTH+TPRs</td><td>EAD2+VMAP-M0+VMAP-C→ vWA+APATPase+wHTH+TPRs*→ ||← METALLOPROTEASE+TPRs← Trypsin+EAD2← Trypco2||METALLOPROTEASE+TPRs→</td><td>1318</td><td>Streptomyces sp. NBRC 110035</td><td>GCF_000829695.1</td><td>actinobacteria</td></tr>
<tr><td>WP_052499299.1</td><td>vWA+TPRs</td><td>TCAD15||Trypco1→ Trypsin+HDxxH+VMAP-M0+VMAP-C→ MoxR→ vWA+TPRs*→ FXSXX-COOH→ METALLOPROTEASE+TPRs→ TIR+APATPase+TPRs→</td><td>1038</td><td>Streptomyces vietnamensis</td><td>GCF_000830005.1</td><td>actinobacteria</td></tr>
<tr><td>WP_052499534.1</td><td>vWA+REase+SWACOS+TPRs+cNMP_cyclase</td><td>AB_hydrolase→ CASPASE+VMAP-M2+VMAP-C→ MoxR→ vWA+REase+SWACOS+TPRs+cNMP_cyclase*→</td><td>1715</td><td>Streptomyces vietnamensis</td><td>GCF_000830005.1</td><td>actinobacteria</td></tr>
<tr><td>WP_052518892.1</td><td>vWA+SLOG+EAD1+TIR</td><td>EAD1+Trypsin→ EAD1+VMAP-M1+VMAP-C→ MoxR→ vWA+SLOG+EAD1+TIR*→</td><td>1400</td><td>Archangium violaceum</td><td>GCA_000733295.1</td><td>deltaproteobacteria</td></tr>
<tr><td>WP_052579849.1</td><td>vWA+Pkinase+wHTH+TPRs+cNMP_cyclase+Band7+HAD</td><td>Trypsin+VMAP-M0+VMAP-C→ MoxR→ vWA+Pkinase+wHTH+TPRs+cNMP_cyclase+Band7+HAD*→ ||← ?||?→ ?→ ?→ ?→ ||← ?||PSE→ TIR+FxsC_Cter→</td><td>1648</td><td>Actinosporangium sp. NRRL B-3428</td><td>-</td><td>actinobacteria</td></tr>
<tr><td>WP_052658798.1</td><td>vWA+TPRs</td><td>AB_hydrolase→ CASPASE+EAD2+VMAP-M0+VMAP-C→ MoxR→ vWA+TPRs*→</td><td>1168</td><td>Streptomyces sp. MNU77</td><td>GCA_000935135.2</td><td>actinobacteria</td></tr>
<tr><td>WP_052680467.1</td><td>vWA+Pkinase+TPRs</td><td>Trypco2→ Trypsin+EAD2+VMAP-M0+VMAP-C→ MoxR→ vWA+Pkinase+TPRs*→</td><td>1155</td><td>Streptomyces sp. NRRL F-4428</td><td>GCF_000956015.1</td><td>actinobacteria</td></tr>
<tr><td>WP_052680669.1</td><td>vWA+Trypsin+cNMP_cyclase+CCCH(TrebleClef)+Pentapeptide_repeats</td><td>AB_hydrolase→ CASPASE+VMAP-M0+VMAP-C→ MoxR→ vWA+Trypsin+cNMP_cyclase+CCCH(TrebleClef)+Pentapeptide_repeats*→</td><td>1199</td><td>Streptomyces sp. NRRL F-4428</td><td>GCF_000956015.1</td><td>actinobacteria</td></tr>
<tr><td>WP_052680675.1</td><td>vWA+TPRs</td><td>TCAD15||Trypsin+HDxxH+VMAP-M0+VMAP-C→ MoxR→ vWA+TPRs*→ FXSXX-COOH→</td><td>1093</td><td>Streptomyces sp. NRRL F-4428</td><td>-</td><td>actinobacteria</td></tr>
<tr><td>WP_052682031.1</td><td>vWA+CASPASE+TIR+FxsC_Cter(partial)</td><td>Trypco1→ Trypsin+VMAP-M0+VMAP-C→ MoxR→ vWA+CASPASE+TIR+FxsC_Cter(partial)*→</td><td>1000</td><td>Saccharothrix sp. ST-888</td><td>GCF_000955975.1</td><td>actinobacteria</td></tr>
<tr><td>WP_052687083.1</td><td>vWA+APATPase+wHTH+TPRs</td><td>Trypco1→ Trypsin+EAD2+VMAP-M0+VMAP-C→ MoxR→ vWA+APATPase+wHTH+TPRs*→ ||← AB_hydrolase</td><td>1324</td><td>Streptomyces lydicus</td><td>GCA_000952035.2</td><td>actinobacteria</td></tr>
<tr><td>WP_052687086.1</td><td>vWA+TPRs</td><td>TCAD15||Trypco1→ Trypsin+HDxxH+VMAP-M0+VMAP-C→ MoxR→ vWA+TPRs*→ FXSXX-COOH→ Radical_SAM→ TIR+APATPase+TPRs→</td><td>1106</td><td>Streptomyces lydicus</td><td>GCA_000952035.2</td><td>actinobacteria</td></tr>
<tr><td>WP_052701300.1</td><td>vWA+ZNR+Pkinase+ParA-Soj-PloopNTPase(SIMIBI)</td><td>SIR2→ ||← ?← ?||Trypco1→ Trypsin+EAD2+VMAP-M0+VMAP-C→ MoxR→ vWA+ZNR+Pkinase+ParA-Soj-PloopNTPase(SIMIBI)*→ Porphobil_deam+Porphobil_deamC→</td><td>1201</td><td>Streptomyces iranensis</td><td>GCA_000938975.1</td><td>actinobacteria</td></tr>
<tr><td>WP_052709503.1</td><td>vWA+APATPase+SWACOS+wHTH+TPRs+Beta-rich-tail</td><td>Trypsin+EAD2→ EAD2+VMAP-M10+VMAP-C→ MoxR→ vWA+APATPase+SWACOS+wHTH+TPRs+Beta-rich-tail*→</td><td>1558</td><td>Streptomyces sp. NRRL S-495</td><td>GCF_000966955.1</td><td>actinobacteria</td></tr>
<tr><td>WP_052709964.1</td><td>vWA+APATPase+wHTH+TPRs</td><td>AB_hydrolase→ CASPASE+VMAP-M4+VMAP-C→ MoxR→ vWA+APATPase+wHTH+TPRs*→</td><td>1575</td><td>Azospirillum thiophilum</td><td>GCF_001305595.1</td><td>alphaproteobacteria</td></tr>
<tr><td>WP_052714800.1</td><td>vWA+TPRs</td><td>TCAD15||Trypsin+HDxxH+VMAP-M0+VMAP-C→ MoxR→ vWA+TPRs*→ FXSXX-COOH→</td><td>1120</td><td>Streptomyces</td><td>GCF_000720235.1</td><td>actinobacteria</td></tr>
<tr><td>WP_052718810.1</td><td>vWA+TPRs</td><td>TCAD15||Trypco1→ Trypsin+HDxxH+VMAP-M0+VMAP-C→ MoxR→ vWA+TPRs*→ FXSXX-COOH→ Radical_SAM→ TIR+APATPase+TPRs→</td><td>1130</td><td>Streptomyces sp. NBRC 110027</td><td>GCF_000829715.2</td><td>actinobacteria</td></tr>
<tr><td>WP_052720904.1</td><td>vWA+SWACOS+TPRs</td><td>Trypco1→ Trypsin+VMAP-M8+VMAP-C→ MoxR→ vWA+SWACOS+TPRs*→</td><td>1320</td><td>Actinoplanes rectilineatus</td><td>GCF_000962825.1</td><td>actinobacteria</td></tr>
<tr><td>WP_052721099.1</td><td>vWA+APATPase+wHTH+TPRs</td><td>TIR+EAD2→ METALLOPROTEASE+TPRs→ EAD2+VMAP-M0+VMAP-C→ vWA+APATPase+wHTH+TPRs*→</td><td>1274</td><td>Actinoplanes rectilineatus</td><td>GCF_000962825.1</td><td>actinobacteria</td></tr>
<tr><td>WP_052731472.1</td><td>vWA+TIR+APATPase+Trypsin+HDxxH+VMAP-M0+VMAP-C</td><td>TCAD15||Trypco1→ Trypsin+HDxxH+VMAP-M0+VMAP-C→ MoxR→ vWA+TIR+APATPase+Trypsin+HDxxH+VMAP-M0+VMAP-C*→</td><td>1422</td><td>Streptomyces sp. MBT28</td><td>GCF_000972435.1</td><td>actinobacteria</td></tr>
<tr><td>WP_052743202.1</td><td>vWA+CASPASE+NACHT+PIN</td><td>vWA+TPRs← MoxR← Trypsin+HDxxH+VMAP-M0+VMAP-C← Trypco1||Trypco1→ Trypsin+HDxxH+VMAP-M0+VMAP-C→ MoxR→ vWA+CASPASE+NACHT+PIN*→</td><td>1151</td><td>Streptomyces mangrovisoli</td><td>GCA_000974985.2</td><td>actinobacteria</td></tr>
<tr><td>WP_052745063.1</td><td>vWA+APATPase+wHTH+TPRs</td><td>EAD2+VMAP-M0+VMAP-C→ vWA+APATPase+wHTH+TPRs*→ ||← METALLOPROTEASE+TPRs← FXSXX-COOH-like← CASPASE+EAD2</td><td>1295</td><td>Micromonospora sp. HK10</td><td>GCF_000982955.1</td><td>actinobacteria</td></tr>
<tr><td>WP_052745900.1</td><td>vWA+APATPase+wHTH+TPRs</td><td>Trypco1||Trypsin+EAD2→ EAD2+VMAP-M0+VMAP-C→ MoxR→ vWA+APATPase+wHTH+TPRs*→ ||← DUF397</td><td>1497</td><td>Allosalinactinospora lopnorensis</td><td>GCF_000981225.1</td><td>actinobacteria</td></tr>
<tr><td>WP_052757695.1</td><td>vWA+TPRs</td><td>TCAD15||Trypsin+HDxxH+VMAP-M0+VMAP-C→ MoxR→ vWA+TPRs*→ FXSXX-COOH→ PSE→ PSE→ METALLOPROTEASE+TPRs→ TIR+APATPase+TPRs→</td><td>1132</td><td>Streptomyces yangpuensis</td><td>GCF_001005295.1</td><td>actinobacteria</td></tr>
</table>

acc	architecture	operon	len	species	gca	taxend
WP_052771536.1	vWA+APATPase+wHTH+TPRs	MoxR→ vWA+APATPase+wHTH+TPRs*→	1041	Streptomyces scabiei	GCF_001005405.1	actinobacteria
WP_052840000.1	vWA+APATPase+wHTH+TPRs	EAD2+VMAP-M0+VMAP-C→ MoxR→ vWA+APATPase+wHTH+TPRs*→ ||← PSE||AB_hydrolase→ CASPASE+EAD2→ FXSXX-COOH-like→ METALLOPROTEASE+TPRs→	1556	Streptomyces aureofaciens	-	actinobacteria
WP_052842417.1	vWA+Pkinase+wHTH+TPRs+cNMP_cyclase	Trypco1→ Trypsin+VMAP-M0+VMAP-C→ MoxR→ vWA+Pkinase+wHTH+TPRs+cNMP_cyclase*→	1345	Streptomyces aureofaciens	-	actinobacteria
WP_052845518.1	vWA+TPRs	vWA+TPRs*→ FXSXX-COOH→ Radical_SAM→ TIR+APATPase+TPRs→ FXSXX-COOH→ Radical_SAM→	982	Streptomyces sp. NRRL S-31	GCF_000718775.1	actinobacteria
WP_052848444.1	vWA+TPRs	TCAD15||Trypsin+HDxxH+VMAP-M0+VMAP-C→ MoxR→ vWA+TPRs*→ FXSXX-COOH→ Radical_SAM→ METALLOPROTEASE+TPRs→ TIR+APATPase+TPRs→	1100	Streptomyces avicenniae	-	actinobacteria
WP_052856488.1	vWA+TPRs	TCAD15||Trypco1→ Trypsin+HDxxH+VMAP-M0+VMAP-C→ MoxR→ vWA+TPRs*→ FXSXX-COOH→ Radical_SAM→ TIR+APATPase+TPRs→	1126	Streptomyces cellulolflavus	GCF_000720995.1	actinobacteria
WP_052867817.1	vWA+TPRs	TCAD15||Trypco1→ Trypsin+HDxxH+VMAP-M0+VMAP-C→ MoxR→ vWA+TPRs*→ FXSXX-COOH→ Radical_SAM→ METALLOPROTEASE+TPRs→ TIR+APATPase+TPRs→ Trypco1→	1110	Streptomyces niger	GCF_000718305.1	actinobacteria
WP_052868496.1	vWA+SWACOS+wHTH	Trypco1→ Trypsin+EAD2+VMAP-M0+VMAP-C→ MoxR→ vWA+SWACOS+wHTH*→	1275	Streptomyces niger	GCF_000718305.1	actinobacteria
WP_052871930.1	vWA+Trypsin+cNMP_cyclase+CCCH(TrebleClef)+Pentapeptide_repeats	AB_hydrolase→ CASPASE+VMAP-M0+VMAP-C→ MoxR→ vWA+Trypsin+cNMP_cyclase+CCCH(TrebleClef)+Pentapeptide_repeats*→	1199	Streptomyces sp. NRRL F-4335	-	actinobacteria
WP_052873786.1	vWA+TPRs	TCAD15||Trypsin+HDxxH+VMAP-M0+VMAP-C→ MoxR→ vWA+TPRs*→ PSE→ METALLOPROTEASE+TPRs→ TIR+APATPase+TPRs→	1138	Streptomyces sp. NRRL F-4335	-	actinobacteria
WP_053048758.1	vWA+Pkinase+ZNR+Pkinase+ParA-Soj-PloopNTPase(SIMIBI)	Trypco1→ Trypsin+EAD2+VMAP-M0+VMAP-C→ MoxR→ vWA+Pkinase+ZNR+Pkinase+ParA-Soj-PloopNTPase(SIMIBI)*→ Porphobil_deam+Porphobil_deamC→	1341	Streptomyces antioxidans	-	actinobacteria
WP_053055495.1	vWA+APATPase+wHTH+TPRs	Trypco2→ EAD2+VMAP-M0+VMAP-C→ vWA+APATPase+wHTH+TPRs*→ ||← METALLOPROTEASE+TPRs← FXSXX-COOH-like← Trypsin+EAD2	1323	Streptomyces malaysiense	GCF_000980885.2	actinobacteria
WP_053065798.1	vWA+TPRs	TCAD15||Trypsin+HDxxH+VMAP-M0+VMAP-C→ MoxR→ vWA+TPRs*→ FXSXX-COOH→ Radical_SAM→ METALLOPROTEASE+TPRs→ TIR+APATPase+TPRs→	1076	Streptomyces sp. Mg1	GCF_000412265.2	actinobacteria
WP_053066357.1	vWA+CASPASE+SLOG+TPRs	EAD1+VMAP-M1+VMAP-C→ MoxR→ vWA+CASPASE+SLOG+TPRs*→	1460	Archangium gephyra	GCA_001027285.1	deltaproteobacteria
WP_053080709.1	vWA+TPRs	TCAD15||Trypsin+HDxxH+VMAP-M0+VMAP-C→ MoxR→ vWA+TPRs*→ FXSXX-COOH→ PSE→ METALLOPROTEASE+TPRs→ TIR+APATPase+TPRs→	1104	Streptomyces roseus	GCF_001044425.1	actinobacteria
WP_053138638.1	vWA+APATPase+wHTH+TPRs	EAD2+VMAP-M0+VMAP-C→ MoxR→ vWA+APATPase+wHTH+TPRs*→ ||← Na_H_Exchanger||AB_hydrolase→ CASPASE+EAD2→ FXSXX-COOH-like→ PSE→ METALLOPROTEASE+TPRs→	1562	Streptomyces ambofaciens	GCA_001267885.1	actinobacteria
WP_053167756.1	vWA+TPRs	TCAD15||Trypsin+HDxxH+VMAP-M0+VMAP-C→ MoxR→ vWA+TPRs*→ FXSXX-COOH→ Radical_SAM→ METALLOPROTEASE+TPRs→ TIR+APATPase+TPRs→	1139	Streptomyces virginiae	GCF_001270565.1	actinobacteria
WP_053200553.1	vWA+APATPase+wHTH+TPRs	Trypco2→ EAD2+VMAP-M0+VMAP-C→ vWA+APATPase+wHTH+TPRs*→ ||← METALLOPROTEASE+TPRs← FXSXX-COOH-like← Trypsin+EAD2	1303	Streptomyces viridochromogenes	GCF_001270485.1	actinobacteria
WP_053211137.1	vWA+Pkinase+cNMP_cyclase+Band7+HAD	Trypco1→ Trypsin+VMAP-M0+VMAP-C→ MoxR→ vWA+Pkinase+cNMP_cyclase+Band7+HAD*→	1308	Streptomyces antibioticus	GCA_001270635.1	actinobacteria
WP_053612266.1	vWA+Trypsin+cNMP_cyclase+CCCH(TrebleClef)+Pentapeptide_repeats	AB_hydrolase→ CASPASE+VMAP-M0+VMAP-C→ MoxR→ vWA+Trypsin+cNMP_cyclase+CCCH(TrebleClef)+Pentapeptide_repeats*→	1198	Streptomyces sp. XY533	GCA_001279845.1	actinobacteria
WP_053612976.1	vWA+CASPASE+TCAD12	AB_hydrolase→ CASPASE+VMAP-M2+VMAP-C→ MoxR→ vWA+CASPASE+TCAD12*→	1107	Streptomyces	GCA_001279445.1	actinobacteria
WP_053613993.1	vWA+TPRs	TCAD15||Trypsin+HDxxH+VMAP-M0+VMAP-C→ MoxR→ vWA+TPRs*→ FXSXX-COOH→	1114	Streptomyces	GCF_001279445.1	actinobacteria
WP_053625154.1	vWA+Trypsin+cNMP_cyclase+CCCH(TrebleClef)+Pentapeptide_repeats	AB_hydrolase→ CASPASE+VMAP-M0+VMAP-C→ MoxR→ vWA+Trypsin+cNMP_cyclase+CCCH(TrebleClef)+Pentapeptide_repeats*→	1198	Streptomyces	GCA_001279445.1	actinobacteria
WP_053627348.1	vWA+Trypsin+cNMP_cyclase+CCCH(TrebleClef)+Pentapeptide_repeats	AB_hydrolase→ CASPASE+VMAP-M0+VMAP-C→ MoxR→ vWA+Trypsin+cNMP_cyclase+CCCH(TrebleClef)+Pentapeptide_repeats*→	1198	Streptomyces sp. XY511	GCA_001279485.1	actinobacteria
WP_053627414.1	vWA+CASPASE+TCAD12	AB_hydrolase→ CASPASE+VMAP-M2+VMAP-C→ MoxR→ vWA+CASPASE+TCAD12*→	1107	Streptomyces sp. XY511	GCA_001279485.1	actinobacteria
WP_053628752.1	vWA+TPRs	TCAD15||Trypsin+HDxxH+VMAP-M0+VMAP-C→ MoxR→ vWA+TPRs*→ FXSXX-COOH→ Radical_SAM→	1114	Streptomyces sp. XY511	GCF_001279485.1	actinobacteria
WP_053629375.1	vWA+CASPASE+TCAD12	AB_hydrolase→ CASPASE+VMAP-M2+VMAP-C→ MoxR→ vWA+CASPASE+TCAD12*→	1107	Streptomyces sp. H021	GCA_001279495.1	actinobacteria
WP_053629734.1	vWA+Trypsin+cNMP_cyclase+CCCH(TrebleClef)+Pentapeptide_repeats	AB_hydrolase→ CASPASE+VMAP-M0+VMAP-C→ MoxR→ vWA+Trypsin+cNMP_cyclase+CCCH(TrebleClef)+Pentapeptide_repeats*→	1198	Streptomyces sp. H021	GCA_001279495.1	actinobacteria
WP_053630092.1	vWA+TPRs	TCAD15||Trypsin+HDxxH+VMAP-M0+VMAP-C→ MoxR→ vWA+TPRs*→ FXSXX-COOH→ Radical_SAM→ METALLOPROTEASE+TPRs→ TIR+APATPase+TPRs→	1120	Streptomyces sp. H021	GCF_001279495.1	actinobacteria
WP_053633026.1	vWA+Trypsin+cNMP_cyclase+CCCH(TrebleClef)+Pentapeptide_repeats	AB_hydrolase→ CASPASE+VMAP-M0+VMAP-C→ MoxR→ vWA+Trypsin+cNMP_cyclase+CCCH(TrebleClef)+Pentapeptide_repeats*→	1198	Streptomyces	GCA_001279685.1	actinobacteria
WP_053633406.1	vWA+TPRs	TCAD15||Trypsin+HDxxH+VMAP-M0+VMAP-C→ MoxR→ vWA+TPRs*→ FXSXX-COOH→ Radical_SAM→	1120	Streptomyces	GCF_001279855.1	actinobacteria
WP_053633441.1	vWA+CASPASE+TCAD12	AB_hydrolase→ CASPASE+VMAP-M2+VMAP-C→ MoxR→ vWA+CASPASE+TCAD12*→	1107	Streptomyces	GCA_001279855.1	actinobacteria
WP_053635218.1	vWA+Pkinase+wHTH+TPRs+cNMP_cyclase+Band7+HAD	Trypco1→ Trypsin+VMAP-M0+VMAP-C→ MoxR→ vWA+Pkinase+wHTH+TPRs+cNMP_cyclase+Band7+HAD*→	1659	Streptomyces sp. XY152	GCF_001279455.1	actinobacteria
WP_053637571.1	vWA+TPRs	TCAD15||Trypco1→ Trypsin+HDxxH+VMAP-M0+VMAP-C→ MoxR→ vWA+TPRs*→ FXSXX-COOH→ Radical_SAM→ METALLOPROTEASE+TPRs→ TIR+APATPase+TPRs→ Trypco1→	1098	Streptomyces sp. NRRL F-4707	GCF_001279605.1	actinobacteria
WP_053640772.1	vWA+TPRs	vWA+TPRs*→ FXSXX-COOH→ METALLOPROTEASE+TPRs→ TIR+APATPase+TPRs→	884	Streptomyces sp. NRRL F-6492	GCF_001280125.1	actinobacteria
WP_053647130.1	vWA+APATPase+SWACOS+wHTH+TPRs+Beta-rich-tail	vWA+APATPase+SWACOS+wHTH+TPRs+Beta-rich-tail*→	1497	Streptomyces sp. XY431	GCF_001279805.1	actinobacteria
WP_053651319.1	vWA+TPRs	AB_hydrolase→ CASPASE+EAD2+VMAP-C→ MoxR→ vWA+TPRs*→	2139	Streptomyces sp. XY431	GCF_001279805.1	actinobacteria
WP_053653628.1	vWA+APATPase+wHTH+TPRs	CASPASE+TPRs→ EAD2+VMAP-M0+VMAP-C→ MoxR→ vWA+APATPase+wHTH+TPRs*→ ||← METALLOPROTEASE+TPRs← Radical_SAM← FXSXX-COOH-like	1417	Micromonospora sp. NRRL B-16802	GCA_001279555.1	actinobacteria
WP_053655870.1	vWA+APATPase+wHTH+TPRs	EAD2+VMAP-M0+VMAP-C→ vWA+APATPase+wHTH+TPRs*→ ||← METALLOPROTEASE+TPRs	1268	Micromonospora sp. NRRL B-16802	GCF_001279555.1	actinobacteria
WP_053659581.1	vWA+Pkinase+ParA-Soj-PloopNTPase(SIMIBI)	Trypco1→ Trypsin+EAD2+VMAP-M0+VMAP-C→ MoxR→ vWA+Pkinase+ParA-Soj-PloopNTPase(SIMIBI)*→	1112	Streptomyces sp. MMG1121	GCF_001280015.1	actinobacteria
WP_053661282.1	vWA+TPRs	TCAD15→ Trypco1→ Trypsin+HDxxH+VMAP-M0+VMAP-C→ MoxR→ vWA+TPRs*→ FXSXX-COOH-like→ METALLOPROTEASE+TPRs→ TIR+APATPase+TPRs→	1089	Streptomyces sp. MMG1121	GCA_001280015.1	actinobacteria

<table>
<tr><th>acc</th><th>architecture</th><th>operon</th><th>len</th><th>species</th><th>gca</th><th>taxend</th></tr>
<tr><td>WP_053662415.1</td><td>vWA+TPRs</td><td>TCAD15||Trypco1→ Trypsin+HDxxH+VMAP-M0+VMAP-C→ MoxR→ vWA+TPRs*→ FXSXX-COOH→ Radical_SAM→ METALLOPROTEASE+TPRs→ TIR+APATPase+TPRs→ Trypco1→ PSE→ ?→ CASPASE→</td><td>1098</td><td>Streptomyces sp. NRRL F-7442</td><td>GCF_001279615.1</td><td>actinobacteria</td></tr>
<tr><td>WP_053662834.1</td><td>vWA+Pkinase+ParA-Soj-PloopNTPase(SIMIBI)</td><td>Trypco1→ Trypsin+EAD2+VMAP-M0+VMAP-C→ MoxR→ vWA+Pkinase+ParA-Soj-PloopNTPase(SIMIBI)*→</td><td>1116</td><td>Streptomyces sp. NRRL F-7442</td><td>GCF_001279615.1</td><td>actinobacteria</td></tr>
<tr><td>WP_053669153.1</td><td>vWA+TPRs</td><td>TCAD15||PSE→ Trypsin+HDxxH+VMAP-M0+VMAP-C→ MoxR→ vWA+TPRs*→ FXSXX-COOH→ METALLOPROTEASE+TPRs→ TIR+APATPase+TPRs→</td><td>1058</td><td>Streptomyces sp. NRRL F-6491</td><td>GCF_001280095.1</td><td>actinobacteria</td></tr>
<tr><td>WP_053676461.1</td><td>vWA+TPRs</td><td>TCAD15||Trypsin+HDxxH+VMAP-M0+VMAP-C→ MoxR→ vWA+TPRs*→ FXSXX-COOH→ Radical_SAM→ METALLOPROTEASE+TPRs→ TIR+APATPase+TPRs→</td><td>1056</td><td>Streptomyces sp. WM4235</td><td>-</td><td>actinobacteria</td></tr>
<tr><td>WP_053679072.1</td><td>vWA+TPRs</td><td>TCAD15||Trypsin+HDxxH+VMAP-M0+VMAP-C→ MoxR→ vWA+TPRs*→ FXSXX-COOH→ Radical_SAM→</td><td>1120</td><td>Streptomyces sp. XY66</td><td>GCF_001279905.1</td><td>actinobacteria</td></tr>
<tr><td>WP_053679427.1</td><td>vWA+CASPASE+TCAD12</td><td>AB_hydrolase→ CASPASE+VMAP-M2+VMAP-C→ MoxR→ vWA+CASPASE+TCAD12*→</td><td>1107</td><td>Streptomyces sp. XY66</td><td>GCA_001279905.1</td><td>actinobacteria</td></tr>
<tr><td>WP_053681301.1</td><td>vWA+Trypsin+cNMP_cyclase+CCCH(TrebleClef)+Pentapeptide_repeats</td><td>AB_hydrolase→ CASPASE+VMAP-M0+VMAP-C→ MoxR→ vWA+Trypsin+cNMP_cyclase+CCCH(TrebleClef)+Pentapeptide_repeats*→</td><td>1198</td><td>Streptomyces sp. XY66</td><td>GCA_001279905.1</td><td>actinobacteria</td></tr>
<tr><td>WP_053683716.1</td><td>vWA+CASPASE+TCAD12</td><td>AB_hydrolase→ CASPASE+VMAP-M2+VMAP-C→ MoxR→ vWA+CASPASE+TCAD12*→</td><td>1111</td><td>Streptomyces sp. XY593</td><td>GCA_001279885.1</td><td>actinobacteria</td></tr>
<tr><td>WP_053685064.1</td><td>vWA+Trypsin+cNMP_cyclase+CCCH(TrebleClef)+Pentapeptide_repeats</td><td>AB_hydrolase→ CASPASE+VMAP-M0+VMAP-C→ MoxR→ vWA+Trypsin+cNMP_cyclase+CCCH(TrebleClef)+Pentapeptide_repeats*→</td><td>1198</td><td>Streptomyces sp. XY593</td><td>GCA_001279885.1</td><td>actinobacteria</td></tr>
<tr><td>WP_053685938.1</td><td>vWA+Trypsin+cNMP_cyclase+CCCH(TrebleClef)+Pentapeptide_repeats</td><td>AB_hydrolase→ CASPASE+VMAP-M0+VMAP-C→ MoxR→ vWA+Trypsin+cNMP_cyclase+CCCH(TrebleClef)+Pentapeptide_repeats*→</td><td>1198</td><td>Streptomyces sp. IGB124</td><td>GCA_001279935.1</td><td>actinobacteria</td></tr>
<tr><td>WP_053686250.1</td><td>vWA+TPRs</td><td>TCAD15||Trypsin+HDxxH+VMAP-M0+VMAP-C→ MoxR→ vWA+TPRs*→ FXSXX-COOH→</td><td>1120</td><td>Streptomyces sp. IGB124</td><td>GCF_001279935.1</td><td>actinobacteria</td></tr>
<tr><td>WP_053687101.1</td><td>vWA+CASPASE+TCAD12</td><td>AB_hydrolase→ CASPASE+VMAP-M2+VMAP-C→ MoxR→ vWA+CASPASE+TCAD12*→</td><td>1107</td><td>Streptomyces sp. IGB124</td><td>-</td><td>actinobacteria</td></tr>
<tr><td>WP_053691781.1</td><td>vWA+TPRs</td><td>TCAD15||Trypsin+HDxxH+VMAP-M0+VMAP-C→ MoxR→ vWA+TPRs*→ FXSXX-COOH→ Radical_SAM→ METALLOPROTEASE+TPRs→ TIR+APATPase+TPRs→</td><td>1075</td><td>Streptomyces sp. WM6372</td><td>-</td><td>actinobacteria</td></tr>
<tr><td>WP_053697007.1</td><td>vWA+TPRs</td><td>TCAD15||Trypco1→ Trypsin+HDxxH+VMAP-M0+VMAP-C→ MoxR→ vWA+TPRs*→ FXSXX-COOH→ Radical_SAM→ TIR+APATPase+TPRs→ TIR+APATPase+TPRs→</td><td>1111</td><td>Streptomyces sp. NRRL F-5755</td><td>GCF_001279365.1</td><td>actinobacteria</td></tr>
<tr><td>WP_053703978.1</td><td>vWA+TPRs</td><td>TCAD15||Trypsin+HDxxH+VMAP-M0+VMAP-C→ MoxR→ vWA+TPRs*→ FXSXX-COOH→</td><td>1137</td><td>Streptomyces sp. WM6368</td><td>GCF_001279625.1</td><td>actinobacteria</td></tr>
<tr><td>WP_053704399.1</td><td>vWA+Trypsin+cNMP_cyclase+CCCH(TrebleClef)+Pentapeptide_repeats</td><td>AB_hydrolase→ CASPASE+VMAP-M0+VMAP-C→ MoxR→ vWA+Trypsin+cNMP_cyclase+CCCH(TrebleClef)+Pentapeptide_repeats*→</td><td>1196</td><td>Streptomyces sp. WM6368</td><td>GCA_001279625.1</td><td>actinobacteria</td></tr>
<tr><td>WP_053705991.1</td><td>vWA+TPRs</td><td>TCAD15||Trypsin+HDxxH+VMAP-M0+VMAP-C→ MoxR→ vWA+TPRs*→ FXSXX-COOH→ Radical_SAM→</td><td>1120</td><td>Streptomyces sp. WM6373</td><td>GCF_001279685.1</td><td>actinobacteria</td></tr>
<tr><td>WP_053712681.1</td><td>vWA+Trypsin+cNMP_cyclase+CCCH(TrebleClef)+Pentapeptide_repeats</td><td>AB_hydrolase→ CASPASE+VMAP-M0+VMAP-C→ MoxR→ vWA+Trypsin+cNMP_cyclase+CCCH(TrebleClef)+Pentapeptide_repeats*→</td><td>1198</td><td>Streptomyces sp. XY413</td><td>GCA_001279765.1</td><td>actinobacteria</td></tr>
<tr><td>WP_053713048.1</td><td>vWA+CASPASE+TCAD12</td><td>AB_hydrolase→ CASPASE+VMAP-M2+VMAP-C→ MoxR→ vWA+CASPASE+TCAD12*→</td><td>1107</td><td>Streptomyces sp. XY413</td><td>GCA_001279765.1</td><td>actinobacteria</td></tr>
<tr><td>WP_053713228.1</td><td>vWA+TPRs</td><td>TCAD15||Trypsin+HDxxH+VMAP-M0+VMAP-C→ MoxR→ vWA+TPRs*→ FXSXX-COOH→</td><td>1120</td><td>Streptomyces sp. XY413</td><td>GCF_001279765.1</td><td>actinobacteria</td></tr>
<tr><td>WP_053736427.1</td><td>vWA+CASPASE+APATPase+TPRs</td><td>AB_hydrolase→ BTLCP+VMAP-M2+VMAP-C→ MoxR→ vWA+CASPASE+APATPase+TPRs*→</td><td>1091</td><td>Nocardia sp. NRRL S-836</td><td>GCA_001279525.1</td><td>actinobacteria</td></tr>
<tr><td>WP_053741039.1</td><td>vWA+TPRs</td><td>MoxR→ vWA+TPRs*→ Radical_SAM→ TIR+APATPase+TPRs→ Radical_SAM→ ?→ STAND_NTPase→ vWA→ TIR+APATPase+TPRs→</td><td>1088</td><td>Streptomyces sp. NRRL WC-3618</td><td>GCF_001280005.1</td><td>actinobacteria</td></tr>
<tr><td>WP_053759729.1</td><td>vWA+APATPase+wHTH+TPRs</td><td>EAD2+VMAP-M0+VMAP-C→ vWA+APATPase+wHTH+TPRs*→ ||← METALLOPROTEASE+TPRs← FXSXX-COOH-like← Trypsin+EAD2← Trypco2||METALLOPROTEASE+TPRs→</td><td>1290</td><td>Streptomyces sp. AS58</td><td>GCF_001279985.1</td><td>actinobacteria</td></tr>
<tr><td>WP_053784648.1</td><td>vWA+TPRs</td><td>TCAD15||Trypsin+HDxxH+VMAP-M0+VMAP-C→ MoxR→ vWA+TPRs*→ FXSXX-COOH→ Radical_SAM→ METALLOPROTEASE+TPRs→ TIR+APATPase+TPRs→</td><td>1081</td><td>Streptomyces sp. XY332</td><td>GCF_001280885.1</td><td>actinobacteria</td></tr>
<tr><td>WP_053800883.1</td><td>vWA+TPRs</td><td>TCAD15||Trypco1→ Trypsin+HDxxH+VMAP-M0+VMAP-C→ MoxR→ vWA+TPRs*→ FXSXX-COOH→ Radical_SAM→ TIR+APATPase+TPRs→</td><td>1102</td><td>Streptomyces rimosus</td><td>GCF_001279345.1</td><td>actinobacteria</td></tr>
<tr><td>WP_053922005.1</td><td>vWA+TPRs</td><td>TCAD15||Trypco1→ Trypsin+HDxxH+VMAP-M0+VMAP-C→ MoxR→ vWA+TPRs*→ FXSXX-COOH→ Radical_SAM→ TIR+APATPase+TPRs→</td><td>1105</td><td>Streptomyces chattanoogensis</td><td>GCF_001294335.1</td><td>actinobacteria</td></tr>
<tr><td>WP_053930842.1</td><td>vWA+APATPase+wHTH+TPRs</td><td>EAD2+VMAP-M0+VMAP-C→ MoxR→ vWA+APATPase+wHTH+TPRs*→ AB_hydrolase→ CASPASE+EAD2→ FXSXX-COOH-like→ METALLOPROTEASE+TPRs→</td><td>1443</td><td>Streptomyces sp. NRRL S-4</td><td>GCA_001294345.1</td><td>actinobacteria</td></tr>
<tr><td>WP_054099049.1</td><td>vWA+TPRs</td><td>-</td><td>1127</td><td>Streptomyces sp. NRRL WC-3753</td><td>-</td><td>actinobacteria</td></tr>
<tr><td>WP_054102283.1</td><td>vWA+APATPase+wHTH+TPRs</td><td>-</td><td>1547</td><td>Streptomyces sp. NRRL WC-3753</td><td>-</td><td>actinobacteria</td></tr>
<tr><td>WP_054214454.1</td><td>vWA+Pkinase+ParA-Soj-PloopNTPase(SIMIBI)</td><td>NUDIX→ Trypco1→ Trypsin+EAD2+VMAP-M0+VMAP-C→ MoxR→ vWA+Pkinase+ParA-Soj-PloopNTPase(SIMIBI)*→ SIR2→</td><td>1131</td><td>Actinobacteria bacterium OK074</td><td>GCF_001298575.1</td><td>actinobacteria</td></tr>
<tr><td>WP_054220968.1</td><td>vWA+TPRs</td><td>TCAD15||Trypsin+HDxxH+VMAP-M0+VMAP-C→ MoxR→ vWA+TPRs*→ FXSXX-COOH→ Radical_SAM→ METALLOPROTEASE+TPRs→ TIR+APATPase+TPRs→</td><td>1067</td><td>Actinobacteria bacterium OV450</td><td>GCA_001298555.1</td><td>actinobacteria</td></tr>
<tr><td>WP_054230306.1</td><td>vWA+Pkinase+ParA-Soj-PloopNTPase(SIMIBI)</td><td>ADP_ribosyl_GH→ NUDIX→ Trypco1→ Trypsin+EAD2+VMAP-M0+VMAP-C→ MoxR→ vWA+Pkinase+ParA-Soj-PloopNTPase(SIMIBI)*→</td><td>1156</td><td>Actinobacteria bacterium OK006</td><td>GCA_001298565.1</td><td>actinobacteria</td></tr>
<tr><td>WP_054232446.1</td><td>vWA+APATPase+wHTH+TPRs</td><td>EAD2+VMAP-M0+VMAP-C→ vWA+APATPase+wHTH+TPRs*→ ||← METALLOPROTEASE+TPRs← FXSXX-COOH-like← Trypsin+EAD2← Trypco2||METALLOPROTEASE+TPRs→</td><td>1296</td><td>Actinobacteria bacterium OK006</td><td>-</td><td>actinobacteria</td></tr>
<tr><td>WP_054236610.1</td><td>vWA+BetaPropeller</td><td>AB_hydrolase→ CASPASE+VMAP-M2+VMAP-C→ MoxR→ vWA+BetaPropeller*→</td><td>803</td><td>Actinobacteria bacterium OK006</td><td>GCF_001298565.1</td><td>actinobacteria</td></tr>
<tr><td>WP_054238173.1</td><td>vWA+cNMP_cyclase+Band7+HAD</td><td>Trypco1→ Trypsin+VMAP-M0+VMAP-C→ MoxR→ vWA+cNMP_cyclase+Band7+HAD*→</td><td>1132</td><td>Actinobacteria bacterium OV320</td><td>-</td><td>actinobacteria</td></tr>
<tr><td>WP_054242429.1</td><td>vWA+Pkinase+ParA-Soj-PloopNTPase(SIMIBI)</td><td>DUF397→ Trypco1→ Trypsin+EAD2+VMAP-M0+VMAP-C→ MoxR→ vWA+Pkinase+ParA-Soj-PloopNTPase(SIMIBI)*→</td><td>1116</td><td>Actinobacteria bacterium OV320</td><td>GCA_001298545.1</td><td>actinobacteria</td></tr>
</table>

<table>
<tr><th>acc</th><th>architecture</th><th>operon</th><th>len</th><th>species</th><th>gca</th><th>taxend</th></tr>
<tr><td>WP_054242795.1</td><td>vWA+TPRs</td><td>TCAD15||Trypco1→ Trypsin+HDxxH+VMAP-M0+VMAP-C→ MoxR→ vWA+TPRs*→ FXSXX-COOH→ Radical_SAM→ METALLOPROTEASE+TPRs→ TIR+APATPase+TPRs→</td><td>1126</td><td>Actinobacteria bacterium OV320</td><td>GCF_001298545.1</td><td>actinobacteria</td></tr>
<tr><td>WP_054289253.1</td><td>vWA+SWACOS</td><td>AB_hydrolase→ CASPASE+VMAP-M2+VMAP-C→ MoxR→ vWA+SWACOS*→</td><td>1294</td><td>Kibdelosporangium phytohabitans</td><td>GCA_001302585.1</td><td>actinobacteria</td></tr>
<tr><td>WP_054294635.1</td><td>vWA+APATPase+wHTH+TPRs</td><td>EAD2+VMAP-M0+VMAP-C→ MoxR→ vWA+APATPase+wHTH+TPRs*→ ||← METALLOPROTEASE+TPRs||EAD2+VMAP-M0+VMAP-C→ MoxR→ vWA+APATPase+wHTH+TPRs→ || METALLOPROTEASE+TPRs← FXSXX-COOH-like</td><td>1442</td><td>Kibdelosporangium phytohabitans</td><td>GCA_001302585.1</td><td>actinobacteria</td></tr>
<tr><td>WP_054568190.1</td><td>vWA+APATPase+wHTH+TPRs</td><td>EAD2+EAD1→ EAD2+VMAP-M0+VMAP-C→ MoxR→ vWA+APATPase+wHTH+TPRs*→ ||← METALLOPROTEASE+TPRs</td><td>1592</td><td>Frankia sp. R43</td><td>GCA_001306465.1</td><td>actinobacteria</td></tr>
<tr><td>WP_055419746.1</td><td>vWA+APATPase+wHTH+TPRs</td><td>EAD2+VMAP-M0+VMAP-C→ MoxR→ vWA+APATPase+wHTH+TPRs*→ ||← Na_H_Exchanger||AB_hydrolase→ CASPASE+EAD2→ FXSXX-COOH-like→ METALLOPROTEASE+TPRs→</td><td>1544</td><td>Streptomyces pactum</td><td>GCA_002005225.1</td><td>actinobacteria</td></tr>
<tr><td>WP_055420298.1</td><td>vWA+APATPase+wHTH+TPRs</td><td>EAD2+VMAP-M0+VMAP-C→ MoxR→ vWA+APATPase+wHTH+TPRs*→</td><td>1015</td><td>Streptomyces pactum</td><td>GCA_002005225.1</td><td>actinobacteria</td></tr>
<tr><td>WP_055467560.1</td><td>vWA+TPRs</td><td>TCAD15||Trypco1→ Trypsin+HDxxH+VMAP-M0+VMAP-C→ MoxR→ vWA+TPRs*→ FXSXX-COOH→ Radical_SAM→ METALLOPROTEASE+TPRs→ TIR+APATPase+TPRs→ Trypco1→</td><td>1098</td><td>Streptomyces sp. NBRC 110030</td><td>GCF_001417675.1</td><td>actinobacteria</td></tr>
<tr><td>WP_055491167.1</td><td>vWA+APATPase+wHTH+TPRs</td><td>Trypsin+EAD2→ EAD2+VMAP-M0+VMAP-C→ MoxR→ vWA+APATPase+wHTH+TPRs*→ ||← METALLOPROTEASE+TPRs← Radical_SAM← FXSXX-COOH||Voltage_CLC→</td><td>1477</td><td>Streptomyces sp. TP-A0356</td><td>GCF_001417775.1</td><td>actinobacteria</td></tr>
<tr><td>WP_055491865.1</td><td>vWA+APATPase+wHTH+TPRs</td><td>Trypsin+VMAP-M6+VMAP-C→ MoxR→ vWA+APATPase+wHTH+TPRs*→ ||← METALLOPROTEASE+TPRs← Radical_SAM← FXSXX-COOH-like← Trypco1</td><td>1470</td><td>Streptomyces sp. TP-A0356</td><td>GCF_001417775.1</td><td>actinobacteria</td></tr>
<tr><td>WP_055499354.1</td><td>vWA+TPRs</td><td>vWA+TPRs*→ FXSXX-COOH-like→ METALLOPROTEASE+TPRs→ TIR+APATPase+TPRs→</td><td>1015</td><td>Streptomyces flocculus</td><td>GCF_001418505.1</td><td>actinobacteria</td></tr>
<tr><td>WP_055512459.1</td><td>vWA+Pkinase+ParA-Soj-PloopNTPase(SIMIBI)</td><td>Trypco1→ Trypsin+EAD2+VMAP-M0+VMAP-C→ MoxR→ vWA+Pkinase+ParA-Soj-PloopNTPase(SIMIBI)*→</td><td>1119</td><td>Streptomyces aurantiacus</td><td>GCF_001418335.1</td><td>actinobacteria</td></tr>
<tr><td>WP_055516094.1</td><td>vWA+APATPase+wHTH+TPRs</td><td>EAD2+VMAP-M0+VMAP-C→ MoxR→ vWA+APATPase+wHTH+TPRs*→ FXSXX-COOH-like→ METALLOPROTEASE+TPRs→ AB_hydrolase→ CASPASE+EAD2→</td><td>1441</td><td>Streptomyces aurantiacus</td><td>GCF_001418335.1</td><td>actinobacteria</td></tr>
<tr><td>WP_055518044.1</td><td>vWA+APATPase+wHTH+TPRs</td><td>Trypsin+EAD2→ EAD2+VMAP-M0+VMAP-C→ MoxR→ vWA+APATPase+wHTH+TPRs*→ ||← METALLOPROTEASE+TPRs||Trypco1→</td><td>1490</td><td>Streptomyces aurantiacus</td><td>GCF_001418335.1</td><td>actinobacteria</td></tr>
<tr><td>WP_055550146.1</td><td>vWA+TPRs</td><td>AB_hydrolase→ CASPASE+EAD2+VMAP-M0+VMAP-C→ MoxR→ vWA+TPRs*→</td><td>1160</td><td>Streptomyces sp. NBRC 110028</td><td>GCF_001417735.1</td><td>actinobacteria</td></tr>
<tr><td>WP_055551712.1</td><td>vWA+APATPase+wHTH+TPRs</td><td>PNP_UDP_1+EAD2→ EAD2+VMAP-M0+VMAP-C→ MoxR→ vWA+APATPase+wHTH+TPRs*→ ||← METALLOPROTEASE+TPRs</td><td>1452</td><td>Streptomyces sp. NBRC 110028</td><td>GCF_001417735.1</td><td>actinobacteria</td></tr>
<tr><td>WP_055556059.1</td><td>vWA+Pkinase+FtsK+TPRs</td><td>vWA+Pkinase+FtsK+TPRs*→</td><td>1068</td><td>Streptomyces kanamyceticus</td><td>GCF_001418465.1</td><td>actinobacteria</td></tr>
<tr><td>WP_055569004.1</td><td>vWA+Pkinase+wHTH+TPRs+cNMP_cyclase+HAD</td><td>vWA+Pkinase+wHTH+TPRs+cNMP_cyclase+HAD*→</td><td>1205</td><td>Streptomyces atriruber</td><td>GCF_001418325.1</td><td>actinobacteria</td></tr>
<tr><td>WP_055602658.1</td><td>vWA+TPRs</td><td>vWA+TPRs*→ FXSXX-COOH→ METALLOPROTEASE+TPRs→</td><td>847</td><td>Streptomyces aureus</td><td>GCF_001418135.1</td><td>actinobacteria</td></tr>
<tr><td>WP_055613191.1</td><td>vWA+APATPase+wHTH+TPRs</td><td>Trypsin+EAD2→ EAD2+VMAP-M0+VMAP-C→ MoxR→ vWA+APATPase+wHTH+TPRs*→ ||← METALLOPROTEASE+TPRs← FXSXX-COOH-like||Trypco1→</td><td>1494</td><td>Streptomyces phaeochromogenes</td><td>GCF_001418655.1</td><td>actinobacteria</td></tr>
<tr><td>WP_055618099.1</td><td>vWA+Pkinase+wHTH+TPRs+cNMP_cyclase+Band7+HAD</td><td>vWA+Pkinase+wHTH+TPRs+cNMP_cyclase+Band7+HAD*→</td><td>1436</td><td>Streptomyces phaeochromogenes</td><td>GCF_001418655.1</td><td>actinobacteria</td></tr>
<tr><td>WP_055631399.1</td><td>vWA+REase+cNMP_cyclase+CASPASE</td><td>AB_hydrolase→ CASPASE+EAD2+VMAP-M0+VMAP-C→ MoxR→ vWA+REase+cNMP_cyclase+CASPASE*→</td><td>1144</td><td>Streptomyces griseoruber</td><td>GCA_001514205.1</td><td>actinobacteria</td></tr>
<tr><td>WP_055643571.1</td><td>vWA+TPRs</td><td>AB_hydrolase→ CASPASE+EAD2+VMAP-M0+VMAP-C→ MoxR→ vWA+TPRs*→</td><td>1193</td><td>Streptomyces venezuelae</td><td>GCA_001443625.1</td><td>actinobacteria</td></tr>
<tr><td>WP_055643712.1</td><td>vWA+APATPase+wHTH+TPRs</td><td>EAD2+VMAP-M0+VMAP-C→ MoxR→ vWA+APATPase+wHTH+TPRs*→ ||← Na_H_Exchanger||AB_hydrolase→ CASPASE+EAD2→ FXSXX-COOH-like→ METALLOPROTEASE+TPRs→</td><td>1526</td><td>Streptomyces venezuelae</td><td>GCA_001443625.1</td><td>actinobacteria</td></tr>
<tr><td>WP_055702450.1</td><td>vWA+Pkinase+wHTH+TPRs+cNMP_cyclase+HAD</td><td>vWA+Pkinase+wHTH+TPRs+cNMP_cyclase+HAD*→</td><td>1252</td><td>Streptomyces silaceus</td><td>-</td><td>actinobacteria</td></tr>
<tr><td>WP_055705732.1</td><td>vWA+APATPase+wHTH+TPRs</td><td>Trypco2→ EAD2+VMAP-M0+VMAP-C→ vWA+APATPase+wHTH+TPRs*→ ||← METALLOPROTEASE+TPRs← Trypsin+EAD2</td><td>1284</td><td>Streptomyces puniciscabiei</td><td>GCF_001419685.1</td><td>actinobacteria</td></tr>
<tr><td>WP_055706346.1</td><td>vWA+DNA-primase-like_ZnR+S1+KH+KH</td><td>Trypco1→ Trypsin+EAD2+VMAP-M0+VMAP-C→ MoxR→ vWA+DNA-primase-like_ZnR+S1+KH+KH*→</td><td>1101</td><td>Streptomyces puniciscabiei</td><td>GCF_001419685.1</td><td>actinobacteria</td></tr>
<tr><td>WP_055715312.1</td><td>vWA+APATPase+wHTH+TPRs</td><td>Trypsin+EAD2+VMAP-M0+VMAP-C→ vWA+APATPase+wHTH+TPRs*→</td><td>850</td><td>Streptomyces torulosus</td><td>GCF_001419765.1</td><td>actinobacteria</td></tr>
<tr><td>WP_055750281.1</td><td>vWA+APATPase+wHTH+TPRs</td><td>EAD2→ EAD2+VMAP-M0+VMAP-C→ MoxR→ vWA+APATPase+wHTH+TPRs*→ ||← METALLOPROTEASE+TPRs← FXSXX-COOH-like</td><td>1346</td><td>Frankia sp. AvcI1</td><td>GCF_001420875.1</td><td>actinobacteria</td></tr>
<tr><td>WP_056567333.1</td><td>vWA+TPRs</td><td>TCAD15||PSE→ Trypsin+HDxxH+VMAP-M0+VMAP-C→ MoxR→ vWA+TPRs*→ FXSXX-COOH→ METALLOPROTEASE+TPRs→ TIR+APATPase+TPRs→</td><td>1063</td><td>Streptomyces</td><td>GCA_001426405.1</td><td>actinobacteria</td></tr>
<tr><td>WP_056644000.1</td><td>vWA+TPRs</td><td>TCAD15||Trypco1→ Trypsin+HDxxH+VMAP-M0+VMAP-C→ MoxR→ vWA+TPRs*→ FXSXX-COOH→ METALLOPROTEASE+TPRs→ TIR+APATPase+TPRs→</td><td>1070</td><td>Streptomyces sp. Root431</td><td>GCA_001426585.1</td><td>actinobacteria</td></tr>
<tr><td>WP_056701957.1</td><td>vWA+Pkinase+ParA-Soj-PloopNTPase(SIMIBI)</td><td>Trypco1→ Trypsin+EAD2+VMAP-M0+VMAP-C→ MoxR→ vWA+Pkinase+ParA-Soj-PloopNTPase(SIMIBI)*→</td><td>1245</td><td>Streptomyces</td><td>GCA_001426325.1</td><td>actinobacteria</td></tr>
<tr><td>WP_056706812.1</td><td>vWA+TPRs</td><td>AB_hydrolase→ CASPASE+EAD2+VMAP-M0+VMAP-C→ MoxR→ vWA+TPRs*→</td><td>1170</td><td>Streptomyces</td><td>GCA_001426325.1</td><td>actinobacteria</td></tr>
<tr><td>WP_056794874.1</td><td>vWA+Pkinase+ParA-Soj-PloopNTPase(SIMIBI)</td><td>Trypco1→ Trypsin+EAD2+VMAP-M0+VMAP-C→ MoxR→ vWA+Pkinase+ParA-Soj-PloopNTPase(SIMIBI)*→</td><td>1135</td><td>Streptomyces</td><td>GCA_001426615.1</td><td>actinobacteria</td></tr>
<tr><td>WP_057232849.1</td><td>vWA+APATPase+BetaPropeller+Pkinase+wHTH+TPRs+wHTH+TPRs+FtsK</td><td>Trypco1→ Trypsin+EAD2→ EAD2+VMAP-M10+VMAP-C→ MoxR→ vWA+APATPase+BetaPropeller+Pkinase+wHTH+TPRs+wHTH+TPRs+FtsK*→</td><td>2079</td><td>Kitasatospora sp. Root107</td><td>GCA_001424875.1</td><td>actinobacteria</td></tr>
<tr><td>WP_057238953.1</td><td>vWA+CASPASE+TIR+FxsC_Cter(partial)</td><td>Trypco1→ Trypsin+VMAP-M0+VMAP-C→ MoxR→ vWA+CASPASE+TIR+FxsC_Cter(partial)*→</td><td>841</td><td>Kitasatospora</td><td>GCA_001424875.1</td><td>actinobacteria</td></tr>
<tr><td>WP_057241032.1</td><td>vWA+APATPase+BetaPropeller+Pkinase+wHTH+TPRs+wHTH+TPRs+FtsK</td><td>Trypco1→ Trypsin+EAD2→ EAD2+VMAP-M10+VMAP-C→ MoxR→ vWA+APATPase+BetaPropeller+Pkinase+wHTH+TPRs+wHTH+TPRs+FtsK*→</td><td>2142</td><td>Kitasatospora sp. Root187</td><td>GCA_001429805.1</td><td>actinobacteria</td></tr>
<tr><td>WP_057578299.1</td><td>vWA</td><td>EAD2+VMAP-M0+VMAP-C→ vWA*→ ||← vWA+APATPase+wHTH+TPRs← MoxR← DUF397</td><td>383</td><td>Streptomyces sp. Root264</td><td>GCA_001428885.1</td><td>actinobacteria</td></tr>
<tr><td>WP_057578301.1</td><td>vWA+APATPase+wHTH+TPRs</td><td>DUF397→ MoxR→ vWA+APATPase+wHTH+TPRs*→ ||← vWA← EAD2+VMAP-M0+VMAP-C</td><td>886</td><td>Streptomyces sp. Root264</td><td>GCF_001428885.1</td><td>actinobacteria</td></tr>
<tr><td>WP_057584733.1</td><td>vWA+TPRs</td><td>TCAD15||Trypco1→ Trypsin+HDxxH+VMAP-M0+VMAP-C→ MoxR→ vWA+TPRs*→ FXSXX-COOH→ Radical_SAM→ METALLOPROTEASE+TPRs→ TIR+APATPase+TPRs→</td><td>1102</td><td>Streptomyces sp. Root264</td><td>GCA_001428885.1</td><td>actinobacteria</td></tr>
<tr><td>WP_057599902.1</td><td>vWA+cNMP_cyclase+Band7+HAD</td><td>Trypco1→ Trypsin+VMAP-M0+VMAP-C→ MoxR→ vWA+cNMP_cyclase+Band7+HAD*→</td><td>1137</td><td>Streptomyces sp. Root1310</td><td>GCA_001426425.1</td><td>actinobacteria</td></tr>
<tr><td>WP_057602960.1</td><td>vWA+Pkinase+ParA-Soj-PloopNTPase(SIMIBI)</td><td>Trypco1→ Trypsin+EAD2+VMAP-M0+VMAP-C→ MoxR→ vWA+Pkinase+ParA-Soj-PloopNTPase(SIMIBI)*→</td><td>1114</td><td>Streptomyces sp. Root1310</td><td>GCA_001426425.1</td><td>actinobacteria</td></tr>
<tr><td>WP_057603580.1</td><td>vWA+TPRs</td><td>TCAD15||Trypco1→ Trypsin+HDxxH+VMAP-M0+VMAP-C→ MoxR→ vWA+TPRs*→ FXSXX-COOH→ Radical_SAM→ METALLOPROTEASE+TPRs→ TIR+APATPase+TPRs→</td><td>1118</td><td>Streptomyces sp. Root1310</td><td>GCA_001426425.1</td><td>actinobacteria</td></tr>
<tr><td>WP_057605974.1</td><td>vWA+APATPase+wHTH+TPRs</td><td>EAD2+VMAP-M0+VMAP-C→ vWA+APATPase+wHTH+TPRs*→ ||← METALLOPROTEASE+TPRs← Trypsin+EAD2||Trypco2→</td><td>1274</td><td>Streptomyces sp. Root1310</td><td>GCA_001426425.1</td><td>actinobacteria</td></tr>
<tr><td>WP_057662565.1</td><td>vWA+TPRs</td><td>AB_hydrolase→ CASPASE+EAD2+VMAP-M0+VMAP-C→ MoxR→ vWA+TPRs*→</td><td>1170</td><td>Streptomyces anulatus</td><td>GCF_001434355.1</td><td>actinobacteria</td></tr>
<tr><td>WP_057664687.1</td><td>vWA+Pkinase+ParA-Soj-PloopNTPase(SIMIBI)</td><td>Trypco1→ Trypsin+EAD2+VMAP-M0+VMAP-C→ MoxR→ vWA+Pkinase+ParA-Soj-PloopNTPase(SIMIBI)*→</td><td>1245</td><td>Streptomyces anulatus</td><td>GCF_001434355.1</td><td>actinobacteria</td></tr>
</table>

<table>
<tr><th>acc</th><th>architecture</th><th>operon</th><th>len</th><th>species</th><th>gca</th><th>taxend</th></tr>
<tr><td>WP_058080417.1</td><td>vWA+Pkinase+cNMP_cyclase+Band7+HAD</td><td>Trypco1→ Trypsin+VMAP-M0+VMAP-C→ MoxR→ vWA+Pkinase+cNMP_cyclase+Band7+HAD*→</td><td>1308</td><td>Streptomyces hygroscopicus</td><td>GCA_001447075.1</td><td>actinobacteria</td></tr>
<tr><td>WP_058184023.1</td><td>vWA+CASPASE+FGS-fold_domain</td><td>CASPASE+AB_hydrolase→ CASPASE+VMAP-M28+VMAP-C→ MoxR→ vWA+CASPASE+FGS-fold_domain*→</td><td>1553</td><td>Mastigocoleus testarum</td><td>GCA_001456025.1</td><td>cyanobacteria</td></tr>
<tr><td>WP_058915122.1</td><td>vWA</td><td>EAD2+VMAP-M0+VMAP-C→ vWA*→</td><td>386</td><td>Streptomyces sp. 4F</td><td>GCA_001484705.1</td><td>actinobacteria</td></tr>
<tr><td>WP_058915198.1</td><td>vWA</td><td>EAD2+VMAP-M0+VMAP-C→ vWA*→</td><td>385</td><td>Streptomyces sp. 4F</td><td>GCA_001484705.1</td><td>actinobacteria</td></tr>
<tr><td>WP_058925471.1</td><td>vWA+cNMP_cyclase+Band7+HAD</td><td>Trypco1→ Trypsin+VMAP-M0+VMAP-C→ MoxR→ vWA+cNMP_cyclase+Band7+HAD*→</td><td>1094</td><td>Streptomyces sp. CdTB01</td><td>GCA_001484565.1</td><td>actinobacteria</td></tr>
<tr><td>WP_058930817.1</td><td>vWA+BetaPropeller+CASPASE+STAND_NTPase</td><td>AB_hydrolase→ CASPASE+VMAP-M17+VMAP-C→ MoxR→ vWA+BetaPropeller+CASPASE+STAND_NTPase*→</td><td>1717</td><td>Pseudarthrobacter sulfonivorans</td><td>GCA_001484605.1</td><td>actinobacteria</td></tr>
<tr><td>WP_059012194.1</td><td>vWA+TPRs</td><td>TCAD15||Trypco1→ Trypsin+HDxxH+VMAP-M0+VMAP-C→ MoxR→ vWA+TPRs*→ FXSXX-COOH→ PSE→ ?→ ?→ TIR+APATPase+TPRs→</td><td>1108</td><td>Streptomyces specialis</td><td>GCF_001493375.1</td><td>actinobacteria</td></tr>
<tr><td>WP_059042125.1</td><td>vWA+Pkinase+ParA-Soj-PloopNTPase(SIMIBI)</td><td>Trypco1→ Trypsin+EAD2+VMAP-M0+VMAP-C→ MoxR→ vWA+Pkinase+ParA-Soj-PloopNTPase(SIMIBI)*→</td><td>1110</td><td>Streptomyces acidiscabies</td><td>GCA_001485105.1</td><td>actinobacteria</td></tr>
<tr><td>WP_059047321.1</td><td>vWA+TPRs</td><td>vWA+TPRs*→ FXSXX-COOH→ Radical_SAM→ METALLOPROTEASE+TPRs→ TIR+APATPase+TPRs→</td><td>1062</td><td>Streptomyces acidiscabies</td><td>GCF_001485105.1</td><td>actinobacteria</td></tr>
<tr><td>WP_059128852.1</td><td>vWA+TPRs</td><td>TCAD15||Trypco1→ Trypsin+HDxxH+VMAP-M0+VMAP-C→ MoxR→ vWA+TPRs*→ FXSXX-COOH→ Radical_SAM→ METALLOPROTEASE+TPRs→ TIR+APATPase+TPRs→</td><td>1126</td><td>Streptomyces sp. NRRL F-5122</td><td>GCA_001507435.1</td><td>actinobacteria</td></tr>
<tr><td>WP_059146065.1</td><td>vWA+APATPase+wHTH+TPRs</td><td>PNP_UDP_1+EAD2→ EAD2+VMAP-M0+VMAP-C→ MoxR→ vWA+APATPase+wHTH+TPRs*→ ||← METALLOPROTEASE+TPRs</td><td>1452</td><td>Streptomyces</td><td>GCA_001509775.1</td><td>actinobacteria</td></tr>
<tr><td>WP_059146322.1</td><td>vWA+TPRs</td><td>AB_hydrolase→ CASPASE+EAD2+VMAP-M0+VMAP-C→ MoxR→ vWA+TPRs*→</td><td>1165</td><td>Streptomyces violaceusniger</td><td>GCA_001509775.1</td><td>actinobacteria</td></tr>
<tr><td>WP_059252564.1</td><td>vWA+TPRs</td><td>TCAD15→ Trypco1→ Trypsin+HDxxH+VMAP-M0+VMAP-C→ MoxR→ vWA+TPRs*→ FXSXX-COOH-like→ METALLOPROTEASE+TPRs→ TIR+APATPase+TPRs→</td><td>1220</td><td>Streptomyces reticuli</td><td>GCF_001511815.1</td><td>actinobacteria</td></tr>
<tr><td>WP_059261798.1</td><td>vWA+Pkinase+cNMP_cyclase+Band7+HAD</td><td>Trypco1→ Trypsin+VMAP-M0+VMAP-C→ MoxR→ vWA+Pkinase+cNMP_cyclase+Band7+HAD*→</td><td>1311</td><td>Streptomyces corchorusii</td><td>GCA_001514055.1</td><td>actinobacteria</td></tr>
<tr><td>WP_059264232.1</td><td>vWA+TPRs</td><td>TCAD15→ Trypco1→ Trypsin+HDxxH+VMAP-M0+VMAP-C→ MoxR→ vWA+TPRs*→ FXSXX-COOH-like→ METALLOPROTEASE+TPRs→ TIR+APATPase+TPRs→</td><td>1157</td><td>Streptomyces corchorusii</td><td>GCA_001514055.1</td><td>actinobacteria</td></tr>
<tr><td>WP_059362765.1</td><td>vWA+APATPase+wHTH+TPRs</td><td>cNMP_cyclase+VMAP-M0+VMAP-C→ MoxR→ vWA+APATPase+wHTH+TPRs*→ ||← METALLOPROTEASE+TPRs← FXSXX-COOH-like||TIR+APATPase+TPRs→ ||← DUF397</td><td>1201</td><td>Lechevalieria aerocolonigenes</td><td>GCF_000974445.1</td><td>actinobacteria</td></tr>
<tr><td>WP_059417485.1</td><td>vWA+Pkinase+ParA-Soj-PloopNTPase(SIMIBI)</td><td>MoxR→ vWA+Pkinase+ParA-Soj-PloopNTPase(SIMIBI)*→</td><td>1118</td><td>Streptomyces azureus</td><td>GCA_001270025.1</td><td>actinobacteria</td></tr>
<tr><td>WP_060176762.1</td><td>vWA+TPRs</td><td>TCAD15||Trypsin+HDxxH+VMAP-M0+VMAP-C→ MoxR→ vWA+TPRs*→ FXSXX-COOH→ Radical_SAM→ METALLOPROTEASE+TPRs→ TIR+APATPase+TPRs→</td><td>1099</td><td>Streptomyces sp. IMTB 1903</td><td>GCF_001531145.1</td><td>actinobacteria</td></tr>
<tr><td>WP_060730269.1</td><td>vWA+wHTH+TPRs+wHTH+TPRs+APATPase+wHTH+TPRs</td><td>Trypco1→ Trypsin+EAD2+VMAP-M0+VMAP-C→ MoxR→ vWA+wHTH+TPRs+wHTH+TPRs+APATPase+wHTH+TPRs*→ ||← AB_hydrolase</td><td>1733</td><td>Streptomyces albus</td><td>GCA_001541145.1</td><td>actinobacteria</td></tr>
<tr><td>WP_060733247.1</td><td>vWA+TPRs</td><td>TCAD15||Trypco1→ Trypsin+HDxxH+VMAP-M0+VMAP-C→ MoxR→ vWA+TPRs*→ FXSXX-COOH→ Radical_SAM→ TIR+APATPase+TPRs→</td><td>1102</td><td>Streptomyces albus</td><td>GCA_001541145.1</td><td>actinobacteria</td></tr>
<tr><td>WP_060879576.1</td><td>vWA+APATPase+wHTH+TPRs</td><td>MoxR→ vWA+APATPase+wHTH+TPRs*→</td><td>1041</td><td>Streptomyces scabiei</td><td>GCF_001550225.1</td><td>actinobacteria</td></tr>
<tr><td>WP_060888221.1</td><td>vWA+Pkinase+wHTH+TPRs+cNMP_cyclase</td><td>Trypco1→ Trypsin+VMAP-M0+VMAP-C→ MoxR→ vWA+Pkinase+wHTH+TPRs+cNMP_cyclase*→</td><td>1019</td><td>Streptomyces</td><td>GCF_001550315.1</td><td>actinobacteria</td></tr>
<tr><td>WP_060888708.1</td><td>vWA</td><td>vWA*→</td><td>377</td><td>Streptomyces scabiei</td><td>GCF_001550215.1</td><td>actinobacteria</td></tr>
<tr><td>WP_060895935.1</td><td>vWA+Pkinase+ParA-Soj-PloopNTPase(SIMIBI)</td><td>vWA+Pkinase+ParA-Soj-PloopNTPase(SIMIBI)*→</td><td>1032</td><td>Streptomyces diastatochromogenes</td><td>-</td><td>actinobacteria</td></tr>
<tr><td>WP_060907571.1</td><td>vWA+APATPase+wHTH+TPRs</td><td>MoxR→ vWA+APATPase+wHTH+TPRs*→</td><td>1029</td><td>Streptomyces scabiei</td><td>GCF_001572115.1</td><td>actinobacteria</td></tr>
<tr><td>WP_060944903.1</td><td>vWA+APATPase+wHTH+TPRs</td><td>vWA+APATPase+wHTH+TPRs*→ ||← METALLOPROTEASE+TPRs</td><td>1323</td><td>Streptomyces hygroscopicus</td><td>GCF_001553455.1</td><td>actinobacteria</td></tr>
<tr><td>WP_060947196.1</td><td>vWA+TPRs</td><td>vWA+TPRs*→</td><td>1061</td><td>Streptomyces hygroscopicus</td><td>GCF_001553455.1</td><td>actinobacteria</td></tr>
<tr><td>WP_060948653.1</td><td>vWA+APATPase+wHTH+TPRs</td><td>PNP_UDP_1+EAD2→ EAD2+VMAP-M0+VMAP-C→ MoxR→ vWA+APATPase+wHTH+TPRs*→ ||← METALLOPROTEASE+TPRs</td><td>1462</td><td>Streptomyces hygroscopicus</td><td>GCF_001553435.1</td><td>actinobacteria</td></tr>
<tr><td>WP_060950690.1</td><td>vWA+TPRs</td><td>AB_hydrolase→ CASPASE+EAD2+VMAP-M0+VMAP-C→ MoxR→ vWA+TPRs*→</td><td>1308</td><td>Streptomyces hygroscopicus</td><td>GCF_001553435.1</td><td>actinobacteria</td></tr>
<tr><td>WP_061080206.1</td><td>vWA+APATPase+wHTH</td><td>Trypsin+VMAP-M8+VMAP-C→ MoxR→ vWA+APATPase+wHTH*→</td><td>1065</td><td>Nocardiopsis dassonvillei</td><td>-</td><td>actinobacteria</td></tr>
<tr><td>WP_061081053.1</td><td>vWA+APATPase+wHTH+TPRs</td><td>Trypsin+EAD2→ EAD2+VMAP-M0+VMAP-C→ MoxR→ vWA+APATPase+wHTH+TPRs*→ ||← DUF397</td><td>1514</td><td>Nocardiopsis dassonvillei</td><td>-</td><td>actinobacteria</td></tr>
<tr><td>WP_061083272.1</td><td>vWA+APATPase+wHTH+TPRs</td><td>Trypsin+VMAP-M0+VMAP-C→ vWA+APATPase+wHTH+TPRs*→</td><td>1261</td><td>Nocardiopsis dassonvillei</td><td>-</td><td>actinobacteria</td></tr>
<tr><td>WP_061430554.1</td><td>vWA+FGS-fold_domain</td><td>AB_hydrolase→ CASPASE+VMAP-M14+VMAP-C→ MoxR→ vWA+FGS-fold_domain*→</td><td>842</td><td>Microcystis aeruginosa</td><td>GCA_001578075.1</td><td>cyanobacteria</td></tr>
<tr><td>WP_061432000.1</td><td>vWA+FGS-fold_domain</td><td>HTH+VMAP-M9+VMAP-C→ MoxR→ vWA+FGS-fold_domain*→ ?→ ?→ FGS-fold_domain→</td><td>961</td><td>Microcystis aeruginosa</td><td>GCA_001578075.1</td><td>cyanobacteria</td></tr>
<tr><td>WP_061432248.1</td><td>vWA+FGS-fold_domain</td><td>Trypco1→ Trypsin+VMAP-M1+VMAP-C→ MoxR→ vWA+FGS-fold_domain*→</td><td>870</td><td>Microcystis aeruginosa</td><td>GCA_001578075.1</td><td>cyanobacteria</td></tr>
<tr><td>WP_061441116.1</td><td>vWA</td><td>EAD2+VMAP-M0+VMAP-C→ vWA*→</td><td>505</td><td>Streptomyces sp. CCM_MD2014</td><td>GCA_000772045.1</td><td>actinobacteria</td></tr>
<tr><td>WP_061442995.1</td><td>vWA+Pkinase+ParA-Soj-PloopNTPase(SIMIBI)</td><td>Trypco1→ Trypsin+EAD2+VMAP-M0+VMAP-C→ MoxR→ vWA+Pkinase+ParA-Soj-PloopNTPase(SIMIBI)*→</td><td>1116</td><td>Streptomyces sp. CCM_MD2014</td><td>GCF_000772045.1</td><td>actinobacteria</td></tr>
<tr><td>WP_061446900.1</td><td>vWA+TPRs</td><td>TCAD15||Trypco1→ Trypsin+HDxxH+VMAP-M0+VMAP-C→ MoxR→ vWA+TPRs*→ FXSXX-COOH→ Radical_SAM→ METALLOPROTEASE+TPRs→ TIR+APATPase+TPRs→ Trypco1→</td><td>1077</td><td>Streptomyces sp. CCM_MD2014</td><td>GCF_000772045.1</td><td>actinobacteria</td></tr>
<tr><td>WP_061783879.1</td><td>vWA+APATPase+wHTH+TPRs</td><td>Trypsin+EAD2→ EAD2+VMAP-M0+VMAP-C→ MoxR→ vWA+APATPase+wHTH+TPRs*→ ||← DUF397||Trypco1→</td><td>1479</td><td>Thermobifida fusca</td><td>GCF_001592045.1</td><td>actinobacteria</td></tr>
<tr><td>WP_061790674.1</td><td>vWA+DRHyd+TCAD8</td><td>AB_hydrolase→ CASPASE+VMAP-M20+VMAP-C→ MoxR→ vWA+DRHyd+TCAD8*→</td><td>1395</td><td>Herbaspirillum rubrisubalbicans</td><td>GCA_003719195.1</td><td>betaproteobacteria</td></tr>
<tr><td>WP_061927345.1</td><td>vWA+TPRs</td><td>TCAD15||PSE→ Trypsin+HDxxH+VMAP-M0+VMAP-C→ MoxR→ vWA+TPRs*→ FXSXX-COOH→ Radical_SAM→ METALLOPROTEASE+TPRs→ TIR+APATPase+TPRs→ Trypco1→</td><td>1114</td><td>Streptomyces bungoensis</td><td>GCA_001514215.1</td><td>actinobacteria</td></tr>
<tr><td>WP_061981586.1</td><td>vWA+APATPase+wHTH+TPRs</td><td>cNMP_cyclase+VMAP-M0+VMAP-C→ MoxR→ vWA+APATPase+wHTH+TPRs*→ ||← METALLOPROTEASE+TPRs← FXSXX-COOH-like← cNMP_binding+HTH_Crp_2</td><td>1217</td><td>Amycolatopsis regifaucium</td><td>GCA_001613935.1</td><td>actinobacteria</td></tr>
<tr><td>WP_062009594.1</td><td>vWA+TPRs</td><td>AB_hydrolase→ CASPASE+EAD2+VMAP-M0+VMAP-C→ MoxR→ vWA+TPRs*→</td><td>1317</td><td>Streptomyces sporocinereus</td><td>GCF_001570645.1</td><td>actinobacteria</td></tr>
<tr><td>WP_062152500.1</td><td>vWA+APATPase+wHTH+TPRs</td><td>Trypsin+EAD2→ EAD2+VMAP-M0+VMAP-C→ MoxR→ vWA+APATPase+wHTH+TPRs*→ ||← METALLOPROTEASE+TPRs← FXSXX-COOH-like</td><td>1449</td><td>Streptomyces curacoi</td><td>GCA_001513975.1</td><td>actinobacteria</td></tr>
<tr><td>WP_062192328.1</td><td>vWA+APATPase+wHTH+TPRs</td><td>EAD2+VMAP-M0+VMAP-C→ vWA+APATPase+wHTH+TPRs*→ ||← METALLOPROTEASE+TPRs← Trypsin+EAD2</td><td>1318</td><td>Streptomyces sp. AVP053U2</td><td>GCA_001469455.2</td><td>actinobacteria</td></tr>
<tr><td>WP_062206696.1</td><td>vWA+TPRs</td><td>TCAD15||Trypco1→ Trypsin+HDxxH+VMAP-M0+VMAP-C→ MoxR→ vWA+TPRs*→ ?→ FXSXX-COOH→ Radical_SAM→ METALLOPROTEASE+TPRs→ TIR+APATPase+TPRs→</td><td>1094</td><td>Streptomyces sp. NBRC 109706</td><td>GCF_000974485.1</td><td>actinobacteria</td></tr>
<tr><td>WP_062640490.1</td><td>vWA+Pkinase+ParA-Soj-PloopNTPase(SIMIBI)</td><td>DUF397→ Trypco1→ Trypsin+EAD2+VMAP-M0+VMAP-C→ MoxR→ vWA+Pkinase+ParA-Soj-PloopNTPase(SIMIBI)*→</td><td>1100</td><td>Streptomyces sp. NBRC 110468</td><td>GCF_001184025.1</td><td>actinobacteria</td></tr>
<tr><td>WP_062642514.1</td><td>vWA+TPRs</td><td>TCAD15||Trypco1→ Trypsin+HDxxH+VMAP-M0+VMAP-C→ MoxR→ vWA+TPRs*→ FXSXX-COOH→ Radical_SAM→ METALLOPROTEASE+TPRs→ TIR+APATPase+TPRs→ Trypco1→</td><td>1110</td><td>Streptomyces sp. NBRC 110468</td><td>GCF_001184025.1</td><td>actinobacteria</td></tr>
</table>

acc	architecture	operon	len	species	gca	taxend
WP_062649304.1	vWA+cNMP_cyclase+Band7+HAD	Trypco1→ Trypsin+VMAP-M0+VMAP-C→ MoxR→ vWA+cNMP_cyclase+Band7+HAD*→	1170	Streptomyces sp. NBRC 110468	-	actinobacteria
WP_062706177.1	vWA+TPRs	Trypco1→ Trypsin+HDxxH+VMAP-M0+VMAP-C→ MoxR→ vWA+TPRs*→ Radical_SAM→ TIR+APATPase+TPRs→ ||← TCAD15	1094	Streptomyces regalis	GCA_001509475.1	actinobacteria
WP_062717917.1	vWA+TPRs	TCAD15→ Trypco1→ Trypsin+HDxxH+VMAP-M0+VMAP-C→ MoxR→ vWA+TPRs*→ Radical_SAM→ TIR+APATPase+TPRs→	1087	Streptomyces caeruleatus	GCA_001514235.1	actinobacteria
WP_062720228.1	vWA+TIR+FxsC_Cter(partial)	AB_hydrolase→ CASPASE+EAD2+VMAP-M0+VMAP-C→ MoxR→ vWA+TIR+FxsC_Cter(partial)*→	865	Streptomyces caeruleatus	GCA_001514235.1	actinobacteria
WP_062721860.1	vWA+Pkinase+ParA-Soj-PloopNTPase(SIMIBI)	Trypsin+VMAP-M0+VMAP-C→ MoxR→ vWA+Pkinase+ParA-Soj-PloopNTPase(SIMIBI)*→	1122	Streptomyces caeruleatus	GCF_001514235.1	actinobacteria
WP_063274055.1	vWA+APATPase+wHTH+TPRs	cNMP_cyclase+VMAP-M0+VMAP-C→ MoxR→ vWA+APATPase+wHTH+TPRs*→ ||← METALLOPROTEASE+TPRs← FXSXX-COOH-like← cNMP_binding+HTH_Crp_2	1197	Amycolatopsis keratiniphila	-	actinobacteria
WP_063483361.1	vWA+APATPase+wHTH+TPRs	EAD2+VMAP-M0+VMAP-C→ MoxR→ vWA+APATPase+wHTH+TPRs*→ ||← Na_H_Exchanger||AB_hydrolase→ CASPASE+EAD2→ FXSXX-COOH-like→ METALLOPROTEASE+TPRs→	1562	Streptomyces ambofaciens	GCA_001632865.1	actinobacteria
WP_063628645.1	vWA+Pkinase+wHTH+TPRs+cNMP_cyclase	Trypco1→ Trypsin+VMAP-M0+VMAP-C→ MoxR→ PSE→ vWA+Pkinase+wHTH+TPRs+cNMP_cyclase*→	1038	Streptomyces lividans	GCF_000158935.1	actinobacteria
WP_063731010.1	vWA+Pkinase+Pkinase+ParA-Soj-PloopNTPase(SIMIBI)	Trypco1→ Trypsin+EAD2+VMAP-M0+VMAP-C→ MoxR→ vWA+Pkinase+Pkinase+ParA-Soj-PloopNTPase(SIMIBI)*→ Porphobil_deam+Porphobil_deamC→	1197	Streptomyces sp. RTd22	GCF_001650215.1	actinobacteria
WP_063732781.1	vWA+BetaPropeller+CASPASE+APAT-Pase+wHTH+TPRs+AB_hydrolase	EAD1+Trypsin→ EAD2+VMAP-M0+VMAP-C→ MoxR→ vWA+BetaPropeller+CASPASE+APATPase+wHTH+TPRs+AB_hydrolase*→	1525	Streptomyces sp. RTd22	GCF_001640565.1	actinobacteria
WP_063734284.1	vWA+TPRs	AB_hydrolase→ CASPASE+EAD2+VMAP-M0+VMAP-C→ MoxR→ vWA+TPRs*→	1161	Streptomyces sp. RTd22	GCF_001650215.1	actinobacteria
WP_063736496.1	vWA+APATPase+wHTH+TPRs	PNP_UDP_1+EAD2→ EAD2+VMAP-M0+VMAP-C→ MoxR→ vWA+APATPase+wHTH+TPRs*→ ||← METALLOPROTEASE+TPRs	1451	Streptomyces sp. RTd22	GCF_001650215.1	actinobacteria
WP_063736878.1	vWA+APATPase+wHTH+TPRs	vWA+APATPase+wHTH+TPRs*→ ||← METALLOPROTEASE+TPRs← Radical_SAM← FXSXX-COOH||Voltage_CLC→ PSE→ ||← Trypco1	1444	Streptomyces sp. RTd22	GCF_001640565.1	actinobacteria
WP_063781306.1	vWA+TPRs	TCAD15||Trypco1→ Trypsin+HDxxH+VMAP-M0+VMAP-C→ MoxR→ vWA+TPRs*→ FXSXX-COOH→ Radical_SAM→ TIR+APATPase+TPRs→	1121	Streptomyces decoyicus	GCA_001270575.1	actinobacteria
WP_063784888.1	vWA+Pkinase+wHTH+TPRs+cNMP_cyclase+Band7+HAD	Trypco1→ Trypsin+VMAP-M0+VMAP-C→ MoxR→ vWA+Pkinase+wHTH+TPRs+cNMP_cyclase+Band7+HAD*→	1705	Streptomyces viridochromogenes	GCF_001270495.1	actinobacteria
WP_063798505.1	vWA+APATPase+wHTH+TPRs	cNMP_binding+HTH_Crp_2||cNMP_cyclase+EAD2+VMAP-M0+VMAP-C→ MoxR→ vWA+APATPase+wHTH+TPRs*→ ||← METALLOPROTEASE+TPRs← FXSXX-COOH-like	1397	Nocardia sp. NRRL S-836	GCA_001279525.1	actinobacteria
WP_063820828.1	vWA+APATPase+wHTH+TPRs	-	1433	Frankia sp. QA3	-	actinobacteria
WP_063826594.1	vWA+APATPase+wHTH+TPRs	EAD2+EAD1→ EAD2+VMAP-M0+VMAP-C→ MoxR→ vWA+APATPase+wHTH+TPRs*→ ||← METALLOPROTEASE+TPRs	1554	Frankia discariae	GCF_000373365.1	actinobacteria
WP_063870491.1	vWA+APATPase+wHTH+TPRs	EAD2+EAD1→ EAD2+VMAP-M0+VMAP-C→ MoxR→ vWA+APATPase+wHTH+TPRs*→ ||← METALLOPROTEASE+TPRs← FXSXX-COOH-like	1199	Frankia sp. CpI1-P	-	actinobacteria
WP_064273537.1	vWA+APATPase+wHTH+TPRs	EAD2+VMAP-M0+VMAP-C→ MoxR→ vWA+APATPase+wHTH+TPRs*→ ||← METALLOPROTEASE+TPRs← Radical_SAM← FXSXX-COOH||Voltage_CLC→ ||← Trypco1	1475	Streptomyces sp. RTd22	GCF_001650215.1	actinobacteria
WP_064273706.1	vWA+BetaPropeller+CASPASE+NACHT	EAD1+Trypsin→ EAD2+VMAP-M0+VMAP-C→ MoxR→ vWA+BetaPropeller+CASPASE+NACHT*→	1398	Streptomyces sp. RTd22	-	actinobacteria
WP_064455400.1	vWA+APATPase+wHTH+TPRs	PNP_UDP_1+EAD2→ EAD2+VMAP-M0+VMAP-C→ MoxR→ vWA+APATPase+wHTH+TPRs*→ ||← METALLOPROTEASE+TPRs	1460	Streptomyces sp. NBRC 109436	GCF_001652895.1	actinobacteria
WP_064456135.1	vWA+TPRs	CASPASE+EAD2+VMAP-M0+VMAP-C→ MoxR→ vWA+TPRs*→	1174	Streptomyces sp. NBRC 109436	GCF_001652895.1	actinobacteria
WP_065477020.1	vWA+APATPase+wHTH+TPRs	EAD2+VMAP-M0+VMAP-C→ MoxR→ vWA+APATPase+wHTH+TPRs*→ AB_hydrolase→ CASPASE+EAD2→ FXSXX-COOH-like→ METALLOPROTEASE+TPRs→	1487	Streptomyces sp. PTY087I2	GCA_001687325.1	actinobacteria
WP_065757097.1	vWA+TPRs	TCAD15||Trypco1→ Trypsin+HDxxH+VMAP-M0+VMAP-C→ MoxR→ vWA+TPRs*→	1155	Streptomyces clavuligerus	GCA_001693675.1	actinobacteria
WP_065913170.1	vWA+APATPase+wHTH+TPRs	cNMP_binding+HTH_Crp_2||cNMP_cyclase+EAD2+VMAP-M0+VMAP-C→ MoxR→ vWA+APATPase+wHTH+TPRs*→ ||← METALLOPROTEASE+TPRs← FXSXX-COOH-like	1418	Lentzea guizhouensis	GCA_001701025.1	actinobacteria
WP_065917436.1	vWA+CASPASE+NACHT+TPRs	AB_hydrolase→ BTLCP+VMAP-M2+VMAP-C→ MoxR→ vWA+CASPASE+NACHT+TPRs*→	1833	Lentzea guizhouensis	GCA_001701025.1	actinobacteria
WP_065959104.1	vWA+TPRs	AB_hydrolase→ CASPASE+EAD2+VMAP-M0+VMAP-C→ MoxR→ vWA+TPRs*→	1166	Streptomyces sparsogenes	GCA_001969965.1	actinobacteria
WP_065965731.1	vWA+Pkinase+Pkinase	Trypco1→ Trypsin+EAD2+VMAP-M0+VMAP-C→ MoxR→ vWA+Pkinase+Pkinase*→ Porphobil_deam+Porphobil_deamC→	1144	Streptomyces sparsogenes	GCF_001704635.1	actinobacteria
WP_065966463.1	vWA+APATPase+wHTH+TPRs	PNP_UDP_1+EAD2→ EAD2+VMAP-M0+VMAP-C→ MoxR→ vWA+APATPase+wHTH+TPRs*→ ||← METALLOPROTEASE+TPRs	1466	Streptomyces sparsogenes	GCF_001704635.1	actinobacteria
WP_066027130.1	vWA+TPRs	AB_hydrolase→ CASPASE+EAD2+VMAP-M0+VMAP-C→ MoxR→ vWA+TPRs*→	1308	Streptomyces sporocinereus	GCF_001705785.1	actinobacteria
WP_066027899.1	vWA+APATPase+wHTH+TPRs	PNP_UDP_1+EAD2→ ||← ?||EAD2+VMAP-M0+VMAP-C→ MoxR→ vWA+APATPase+wHTH+TPRs*→ ||← METALLOPROTEASE+TPRs	1462	Streptomyces sporocinereus	GCF_001705785.1	actinobacteria
WP_066926886.1	vWA+wHTH+TPRs+APATPase+wHTH+TPRs	Trypsin+EAD2+VMAP-M0+VMAP-C→ MoxR→ ?→ vWA+wHTH+TPRs+APATPase+wHTH+TPRs*→	1358	Streptomyces sp. NBRC 110611	GCF_001590865.1	actinobacteria
WP_067008717.1	vWA+APATPase+wHTH+TPRs	Trypco2→ EAD2+VMAP-M0+VMAP-C→ vWA+APATPase+wHTH+TPRs*→ ||← METALLOPROTEASE+TPRs← Trypsin+EAD2	1257	Streptomyces cellostaticus	GCA_001513965.1	actinobacteria
WP_067010480.1	vWA+Pkinase+wHTH+TPRs+cNMP_cyclase+Band7+HAD	DUF397→ ?→ ?→ ?→ Trypco1→ Trypsin+VMAP-M0+VMAP-C→ MoxR→ vWA+Pkinase+wHTH+TPRs+cNMP_cyclase+Band7+HAD*→	1580	Streptomyces cellostaticus	GCA_001513965.1	actinobacteria
WP_067017920.1	vWA+Pkinase+ParA-Soj-PloopNTPase(SIMIBI)	Trypco1→ Trypsin+EAD2+VMAP-M0+VMAP-C→ MoxR→ vWA+Pkinase+ParA-Soj-PloopNTPase(SIMIBI)*→	1135	Streptomyces sp. RV15	GCA_001514305.1	actinobacteria
WP_067024564.1	vWA+APATPase+Trypsin+TIR+FxsC_Cter(partial)	TIR+EAD2→ EAD2+VMAP-M10+VMAP-C→ MoxR→ vWA+APATPase+Trypsin+TIR+FxsC_Cter(partial)*→ ||← DUF397	1226	Streptomyces sp. RV15	GCA_001514305.1	actinobacteria
WP_067031497.1	vWA+APATPase+wHTH+TPRs	EAD2+VMAP-M0+VMAP-C→ MoxR→ vWA+APATPase+wHTH+TPRs*→	983	Streptomyces sp. RV15	GCA_001514305.1	actinobacteria
WP_067043278.1	vWA+Pkinase+ParA-Soj-PloopNTPase(SIMIBI)	Trypco1→ Trypsin+EAD2+VMAP-M0+VMAP-C→ MoxR→ vWA+Pkinase+ParA-Soj-PloopNTPase(SIMIBI)*→	1116	Streptomyces sp. FXJ1.172	GCA_001636945.1	actinobacteria
WP_067055649.1	vWA+TPRs	TCAD15→ Trypco1→ Trypsin+HDxxH+VMAP-M0+VMAP-C→ MoxR→ vWA+TPRs*→ FXSXX-COOH→ METALLOPROTEASE+TPRs→ TIR+APATPase+TPRs→	1163	Streptomyces sp. FXJ1.172	GCA_001636945.1	actinobacteria
WP_067075421.1	vWA+APATPase+wHTH+TPRs	PNP_UDP_1+EAD2→ EAD2+VMAP-M0+VMAP-C→ MoxR→ vWA+APATPase+wHTH+TPRs*→ ||← METALLOPROTEASE+TPRs	1462	Streptomyces endus	GCF_001553475.1	actinobacteria
WP_067079480.1	vWA+TPRs	AB_hydrolase→ CASPASE+EAD2+VMAP-M0+VMAP-C→ MoxR→ vWA+TPRs*→	1191	Streptomyces endus	GCF_001553475.1	actinobacteria
WP_067128388.1	vWA+Pkinase+ParA-Soj-PloopNTPase(SIMIBI)	Trypco1→ Trypsin+EAD2+VMAP-M0+VMAP-C→ MoxR→ vWA+Pkinase+ParA-Soj-PloopNTPase(SIMIBI)*→	1106	Streptomyces yokosukanensis	GCF_001514035.1	actinobacteria
WP_067129121.1	vWA+TPRs	TCAD15→ Trypco1→ Trypsin+HDxxH+VMAP-M0+VMAP-C→ MoxR→ vWA+TPRs*→ FXSXX-COOH→ METALLOPROTEASE+TPRs→ TIR+APATPase+TPRs→	1073	Streptomyces yokosukanensis	GCA_001514035.1	actinobacteria
WP_067166187.1	vWA+Pkinase+ParA-Soj-PloopNTPase(SIMIBI)	NUDIX→ Trypco1→ Trypsin+EAD2+VMAP-M0+VMAP-C→ MoxR→ vWA+Pkinase+ParA-Soj-PloopNTPase(SIMIBI)*→	1133	Streptomyces sp. ERV7	GCA_001653515.1	actinobacteria
WP_067167742.1	vWA+TPRs	vWA+TPRs*→ Radical_SAM→ TIR+APATPase+TPRs→	986	Streptomyces sp. ERV7	GCA_001653515.1	actinobacteria
WP_067237319.1	vWA+TPRs	TCAD15||Trypco1→ Trypsin+HDxxH+VMAP-M0+VMAP-C→ MoxR→ vWA+TPRs*→ FXSXX-COOH→ Radical_SAM→ METALLOPROTEASE+TPRs→ TIR+APATPase+TPRs→	1084	Streptomyces longwoodensis	GCA_001514125.1	actinobacteria
WP_067244441.1	vWA+REase+cNMP_cyclase+CASPASE	AB_hydrolase→ CASPASE+EAD2+VMAP-M0+VMAP-C→ MoxR→ vWA+REase+cNMP_cyclase+CASPASE*→	1144	Streptomyces sp. DSM 15324	GCA_001514285.1	actinobacteria
WP_067362281.1	vWA+Pkinase+ParA-Soj-PloopNTPase(SIMIBI)	ADP_ribosyl_GH→ NUDIX→ Trypco1→ Trypsin+EAD2+VMAP-M0+VMAP-C→ MoxR→ vWA+Pkinase+ParA-Soj-PloopNTPase(SIMIBI)*→	1156	Streptomyces olivochromogenes	GCA_002335465.1	actinobacteria
WP_067395191.1	vWA+APATPase+wHTH+TPRs	Trypsin+EAD2→ EAD2+VMAP-M0+VMAP-C→ MoxR→ vWA+APATPase+wHTH+TPRs*→ ||← METALLOPROTEASE+TPRs← FXSXX-COOH-like||Trypco1→	1596	Streptomyces sp. F-3	GCF_001653115.1	actinobacteria

acc	architecture	operon	len	species	gca	taxend
WP_067415632.1	vWA+TPRs	TCAD15||Trypco1→ Trypsin+HDxxH+VMAP-M0+VMAP-C→ MoxR→ vWA+TPRs*→ FXSXX-COOH→ Radical_SAM→ METALLOPROTEASE+TPRs→ TIR+APATPase+TPRs→	1137	Streptomyces sampsonii	GCF_001704195.1	actinobacteria
WP_067455624.1	vWA+FtsK+FtsK+FtsK	Trypsin+VMAP-M8+VMAP-C→ MoxR→ vWA+FtsK+FtsK+FtsK*→	1335	Actinomadura macra	GCF_001552215.1	actinobacteria
WP_067632860.1	vWA+FtsK+FtsK+CASPASE+FtsK	Trypco1→ Trypsin+VMAP-M8+VMAP-C→ MoxR→ vWA+FtsK+FtsK+CASPASE+FtsK*→	1695	Actinomadura latina	GCF_001552195.1	actinobacteria
WP_067637708.1	vWA+APATPase+wHTH+TPRs	Radical_SAM→ ||← TIR+APATPase+TPRs||SWACOS→ PSE→ ?→ EAD2+VMAP-M0+VMAP-C→ MoxR→ vWA+APATPase+wHTH+TPRs*→ ||← Radical_SAM← ?||cNMP_cyclase+EAD2→	1384	Actinomadura latina	GCF_001552195.1	actinobacteria
WP_067794950.1	vWA+FtsK+FtsK+FtsK	Trypco1→ Trypsin+VMAP-M8+VMAP-C→ MoxR→ vWA+FtsK+FtsK+FtsK*→	1515	Actinomadura formosensis	GCF_001552155.1	actinobacteria
WP_067817617.1	vWA+FtsK+FtsK+FtsK+FtsK+FtsK+FtsK	Trypsin+VMAP-M8+VMAP-C→ MoxR→ vWA+FtsK+FtsK+FtsK+FtsK+FtsK+FtsK*→	2289	Actinomadura kijaniata	GCF_001552175.1	actinobacteria
WP_067892555.1	vWA+APATPase+wHTH	Trypsin+EAD2+VMAP-M0+VMAP-C→ MoxR→ vWA+APATPase+wHTH*→ ?→ ||← ?← ?← ?||?→ ?→ DUF397→	934	Actinomadura chibensis	GCF_001552135.1	actinobacteria
WP_067896613.1	vWA+APATPase+wHTH+TPRs	Radical_SAM→ METALLOPROTEASE+TPRs→ ||← TIR+APATPase+TPRs||FXSXX-COOH→ ?→ EAD2+VMAP-M0+VMAP-C→ MoxR→ vWA+APATPase+wHTH+TPRs*→ cNMP_cyclase+EAD2→	1385	Actinomadura chibensis	GCF_001552135.1	actinobacteria
WP_067901341.1	vWA+Integrin_beta(vWA)	AB_hydrolase→ BTLCP+VMAP-M0+VMAP-C→ MoxR→ vWA+Integrin_beta(vWA)*→	1071	Actinomadura chibensis	GCF_001552135.1	actinobacteria
WP_067973520.1	vWA+APATPase+wHTH+TPRs	Trypsin+VMAP-M0+VMAP-C→ MoxR→ vWA+APATPase+wHTH+TPRs*→ ||← DUF397	1532	Nocardiopsis trehalosi	GCF_001552555.1	actinobacteria
WP_068013580.1	vWA+TPRs	Trypco1→ Trypsin+HDxxH+VMAP-M0+VMAP-C→ MoxR→ vWA+TPRs*→ Voltage_CLC→ METALLOPROTEASE→ TIR+APATPase+wHTH+TPRs→ Radical_SAM→	813	Nocardia mexicana	GCA_003350525.1	actinobacteria
WP_068753449.1	vWA+APATPase+wHTH+TPRs	Trypsin+EAD2→ EAD2+VMAP-M0+VMAP-C→ MoxR→ vWA+APATPase+wHTH+TPRs*→ ||← DUF397||Trypco1→	1477	Thermobifida cellulosilytica	GCA_001517975.1	actinobacteria
WP_068790131.1	vWA+FGS-fold_domain	VMAP-C→ MoxR→ vWA+FGS-fold_domain*→ FGS-fold_domain→	867	Phormidium willei	GCA_001637315.1	cyanobacteria
WP_069169727.1	vWA+APATPase+wHTH+TPRs	EAD2+VMAP-M0+VMAP-C→ MoxR→ vWA+APATPase+wHTH+TPRs*→ AB_hydrolase→ CASPASE+EAD2→ FXSXX-COOH-like→ METALLOPROTEASE+TPRs→	1444	Streptomyces griseus	GCF_001715295.1	actinobacteria
WP_069466096.1	vWA+APATPase+wHTH+TPRs	EAD2+VMAP-M0+VMAP-C→ vWA+APATPase+wHTH+TPRs*→ ||← METALLOPROTEASE+TPRs← Trypsin+EAD2← Trypco2	1212	Streptomyces rubidus	GCF_001723065.1	actinobacteria
WP_069570250.1	vWA	EAD2+VMAP-M0+VMAP-C→ vWA*→	491	Streptomyces lydicus	GCA_001729485.1	actinobacteria
WP_069571998.1	vWA+TPRs	TCAD15||Trypco1→ Trypsin+HDxxH+VMAP-M0+VMAP-C→ MoxR→ vWA+TPRs*→ FXSXX-COOH→ Radical_SAM→	1147	Streptomyces lydicus	GCA_001729485.1	actinobacteria
WP_069622119.1	vWA+Pkinase	Trypco2→ Trypsin+EAD2+VMAP-M0+VMAP-C→ MoxR→ vWA+Pkinase*→	890	Streptomyces niveus	GCF_001723075.1	actinobacteria
WP_069622478.1	vWA+TPRs	vWA+TPRs*→ FXSXX-COOH→ METALLOPROTEASE+TPRs→	806	Streptomyces niveus	GCF_001723075.1	actinobacteria
WP_069628672.1	vWA+Pkinase+TPRs	Trypco2→ Trypsin+EAD2+VMAP-M0+VMAP-C→ MoxR→ vWA+Pkinase+TPRs*→	1121	Streptomyces niveus	GCF_001723055.1	actinobacteria
WP_069737637.1	vWA+Pkinase+ParA-Soj-PloopNTPase(SIMIBI)	Trypco1→ Trypsin+EAD2+VMAP-M0+VMAP-C→ MoxR→ vWA+Pkinase+ParA-Soj-PloopNTPase(SIMIBI)*→	1256	Streptomyces sp. EN27	GCF_001746315.1	actinobacteria
WP_069740765.1	vWA+Pkinase+ParA-Soj-PloopNTPase(SIMIBI)	Trypco1→ Trypsin+EAD2+VMAP-M0+VMAP-C→ MoxR→ vWA+Pkinase+ParA-Soj-PloopNTPase(SIMIBI)*→	1256	Streptomyces sp. EN23	GCF_001746305.1	actinobacteria
WP_069761741.1	vWA+APATPase+wHTH+TPRs	Trypsin+EAD2→ EAD2+VMAP-M0+VMAP-C→ MoxR→ vWA+APATPase+wHTH+TPRs*→ ||← METALLOPROTEASE+TPRs← FXSXX-COOH-like||Trypco1→	1469	Streptomyces sp. LUP47B	GCF_001746295.1	actinobacteria
WP_069782621.1	vWA+APATPase+wHTH+TPRs	Trypsin+EAD2→ EAD2+VMAP-M0+VMAP-C→ MoxR→ vWA+APATPase+wHTH+TPRs*→ ||← METALLOPROTEASE+TPRs← FXSXX-COOH-like||Trypco1→	1534	Streptomyces puniciscabiei	GCA_001735805.1	actinobacteria
WP_069860373.1	vWA+APATPase+wHTH+TPRs	PNP_UDP_1+EAD2→ EAD2+VMAP-M0+VMAP-C→ MoxR→ vWA+APATPase+wHTH+TPRs*→ ||← METALLOPROTEASE+TPRs← FXSXX-COOH-like	1453	Streptomyces sp. SPMA113	GCF_001748165.1	actinobacteria
WP_069866024.1	vWA+TPRs	AB_hydrolase→ CASPASE+EAD2+VMAP-M0+VMAP-C→ MoxR→ vWA+TPRs*→	1171	Streptomyces sp. SPMA113	GCF_001748165.1	actinobacteria
WP_069886105.1	vWA+Pkinase+FtsK+TPRs	ADP_ribosyl_GH→ Trypsin+EAD2+VMAP-M0+VMAP-C→ MoxR→ vWA+Pkinase+FtsK+TPRs*→ NUDIX→	1260	Streptomyces luteocolor	GCF_001748305.1	actinobacteria
WP_069886112.1	vWA+Pkinase+wHTH+TPRs+cNMP_cyclase+HAD	Trypco1→ Trypsin+VMAP-M0+VMAP-C→ MoxR→ vWA+Pkinase+wHTH+TPRs+cNMP_cyclase+HAD*→	1252	Streptomyces luteocolor	-	actinobacteria
WP_069921916.1	vWA+TPRs	TCAD15||Trypsin+HDxxH+VMAP-M0+VMAP-C→ MoxR→ vWA+TPRs*→ FXSXX-COOH→ Radical_SAM→ METALLOPROTEASE+TPRs→ TIR+APATPase+TPRs→	1208	Streptomyces subrutilus	GCA_001746425.1	actinobacteria
WP_069926978.1	vWA+TPRs	TCAD15||PSE→ Trypsin+HDxxH+VMAP-M0+VMAP-C→ MoxR→ vWA+TPRs*→ FXSXX-COOH→ METALLOPROTEASE+TPRs→ TIR+APATPase+TPRs→	1075	Streptomyces agglomeratus	GCA_001746465.1	actinobacteria
WP_069933003.1	vWA+TPRs	TCAD15||PSE→ Trypsin+HDxxH+VMAP-M0+VMAP-C→ MoxR→ vWA+TPRs*→ FXSXX-COOH→ METALLOPROTEASE+TPRs→ TIR+APATPase+TPRs→	1075	Streptomyces agglomeratus	GCA_001746415.1	actinobacteria
WP_069992837.1	vWA+TPRs	Trypco1→ Trypsin+HDxxH+VMAP-M0+VMAP-C→ MoxR→ vWA+TPRs*→ FXSXX-COOH-like→ METALLOPROTEASE+TPRs→ TIR+APATPase+TPRs→	1069	Streptomyces	GCA_001751375.1	actinobacteria
WP_070025225.1	vWA+Pkinase+cNMP_cyclase+Band7+HAD	Trypco1→ Trypsin+VMAP-M0+VMAP-C→ MoxR→ vWA+Pkinase+cNMP_cyclase+Band7+HAD*→	1268	Streptomyces sp. F-1	GCA_900079405.3	actinobacteria
WP_070202374.1	vWA+TIR+FxsC_Cter	AB_hydrolase→ CASPASE+VMAP-M2+VMAP-C→ MoxR→ vWA+TIR+FxsC_Cter*→	941	Streptomyces nanshensis	-	actinobacteria
WP_070202821.1	vWA(partial)+REase+STAND_NTPase+TPRs+cNMP_cyclase	CASPASE+VMAP-M2+VMAP-C→ MoxR→ PSE→ PSE→ vWA(partial)+REase+STAND_NTPase+TPRs+cNMP_cyclase*→	1317	Streptomyces nanshensis	GCA_001751305.1	actinobacteria
WP_070344004.1	vWA+TPRs	TCAD15||PSE→ Trypsin+HDxxH+VMAP-M0+VMAP-C→ MoxR→ vWA+TPRs*→ FXSXX-COOH→ METALLOPROTEASE+TPRs→ TIR+APATPase+TPRs→	1075	Streptomyces agglomeratus	GCA_001746485.1	actinobacteria
WP_070388963.1	vWA+Pkinase+ParA-Soj-PloopNTPase(SIMIBI)	vWA+Pkinase+ParA-Soj-PloopNTPase(SIMIBI)*→	1081	Streptomyces pactum	GCA_001767375.1	actinobacteria
WP_070390415.1	vWA+TPRs	TCAD15||PSE→ Trypsin+HDxxH+VMAP-M0+VMAP-C→ MoxR→ vWA+TPRs*→ PSE→ Radical_SAM→ METALLOPROTEASE+TPRs→ TIR+APATPase+TPRs→	1161	Streptomyces pactum	GCA_001767375.1	actinobacteria
WP_070391592.1	vWA+FGS-fold_domain	EAD1+Trypsin→ ||← ?||PSE→ EAD1+VMAP-M1+VMAP-C→ MoxR→ vWA+FGS-fold_domain*→	939	Moorea producens	GCA_001767235.1	cyanobacteria
WP_071102798.1	vWA+FGS-fold_domain	EAD1+Trypsin→ EAD1+VMAP-M1+Calcineurin-like→ EAD1+VMAP-M1+VMAP-C→ MoxR→ vWA+FGS-fold_domain*→	902	Moorea producens	GCA_001854205.1	cyanobacteria
WP_071106909.1	vWA+FGS-fold_domain	AB_hydrolase→ CASPASE+VMAP-M14+VMAP-C→ MoxR→ vWA+FGS-fold_domain*→ FGS-fold_domain→ four_helix_bundle_protein→	869	Moorea producens	GCA_001854205.1	cyanobacteria
WP_071330512.1	vWA+TPRs	TCAD15||Trypco1→ Trypsin+HDxxH+VMAP-M0+VMAP-C→ MoxR→ vWA+TPRs*→ FXSXX-COOH→ Radical_SAM→ METALLOPROTEASE+TPRs→ TIR+APATPase+TPRs→	1137	Streptomyces sampsonii	GCF_001865315.1	actinobacteria
WP_071336898.1	vWA+TPRs	TCAD15||Trypco1→ Trypsin+HDxxH+VMAP-M0+VMAP-C→ MoxR→ vWA+TPRs*→ FXSXX-COOH→ Radical_SAM→ METALLOPROTEASE+TPRs→ TIR+APATPase+TPRs→	1137	Streptomyces albidoflavus	GCF_001865795.1	actinobacteria
WP_071370429.1	vWA+cNMP_cyclase+Band7+HAD	Trypco1→ Trypsin+VMAP-M0+VMAP-C→ MoxR→ vWA+cNMP_cyclase+Band7+HAD*→	1115	Streptomyces sp. MUSC 14	GCA_001866675.1	actinobacteria
WP_071376575.1	vWA+TPRs	TCAD15||Trypco1→ Trypsin+HDxxH+VMAP-M0+VMAP-C→ MoxR→ vWA+TPRs*→ FXSXX-COOH→ Radical_SAM→ METALLOPROTEASE+TPRs→ TIR+APATPase+TPRs→	1105	Streptomyces sp. MUSC 14	GCA_001866675.1	actinobacteria
WP_071381763.1	vWA+cNMP_cyclase+Band7+HAD	Trypco1→ Trypsin+VMAP-M0+VMAP-C→ MoxR→ vWA+cNMP_cyclase+Band7+HAD*→	1122	Streptomyces sp. MUSC 1	GCA_001866665.1	actinobacteria
WP_071384258.1	vWA+TPRs	TCAD15||Trypco1→ Trypsin+HDxxH+VMAP-M0+VMAP-C→ MoxR→ vWA+TPRs*→ FXSXX-COOH→ Radical_SAM→ METALLOPROTEASE+TPRs→ TIR+APATPase+TPRs→	1105	Streptomyces sp. MUSC 1	GCA_001866665.1	actinobacteria
WP_071387451.1	vWA+Pkinase+cNMP_cyclase+Band7+HAD	Trypco1→ Trypsin+VMAP-M0+VMAP-C→ MoxR→ vWA+Pkinase+cNMP_cyclase+Band7+HAD*→	1272	Streptomyces malaysiense	GCA_000980885.2	actinobacteria
WP_071515078.1	vWA+FGS-fold_domain	-	847	Geitlerinema sp. PCC 9228	-	cyanobacteria
WP_071623019.1	vWA+APATPase+wHTH+TPRs	Trypsin+VMAP-M0+VMAP-C→ vWA+APATPase+wHTH+TPRs*→ ||← DUF397||Trypco1→	1261	Nocardiopsis dassonvillei	GCA_001877055.1	actinobacteria
WP_072031082.1	vWA+APATPase+wHTH+TPRs	cNMP_cyclase+VMAP-M0+VMAP-C→ MoxR→ vWA+APATPase+wHTH+TPRs*→ ||← METALLOPROTEASE+TPRs← FXSXX-COOH-like← cNMP_binding+HTH_Crp_2	1197	Amycolatopsis keratiniphila	GCA_001953855.1	actinobacteria

acc	architecture	operon	len	species	gca	taxend
WP_072319532.1	vWA+FGS-fold_domain	AB_hydrolase→ CASPASE+EAD2→ EAD2+VMAP-M11+VMAP-C→ MoxR→ vWA+FGS-fold_domain*→	701	Microcystis aeruginosa	GCF_001578075.1	cyanobacteria
WP_072926264.1	vWA+FGS-fold_domain	HTH+VMAP-M9+VMAP-C→ MoxR→ vWA+FGS-fold_domain*→	961	Microcystis aeruginosa	GCF_001895325.1	cyanobacteria
WP_072926889.1	vWA+FGS-fold_domain	AB_hydrolase→ CASPASE+VMAP-M14+VMAP-C→ MoxR→ vWA+FGS-fold_domain*→	842	Microcystis aeruginosa	GCF_001895325.1	cyanobacteria
WP_072927156.1	vWA+FGS-fold_domain	Trypco1→ Trypsin+VMAP-M1+VMAP-C→ MoxR→ vWA+FGS-fold_domain*→	883	Microcystis aeruginosa	GCF_001895325.1	cyanobacteria
WP_073223271.1	vWA+Pkinase+ParA-Soj-PloopNTPase(SIMIBI)	Trypco1→ Trypsin+EAD2+VMAP-M0+VMAP-C→ MoxR→ vWA+Pkinase+ParA-Soj-PloopNTPase(SIMIBI)*→	1242	Streptomyces sp. NBRC 110465	GCF_001895105.1	actinobacteria
WP_073380260.1	vWA+APATPase+wHTH+TPRs	Trypsin+EAD2→ EAD2+VMAP-M0+VMAP-C→ MoxR→ vWA+APATPase+wHTH+TPRs*→ ||← DUF397||Trypco1→	1504	Nocardiopsis flavescens	GCA_900141985.1	actinobacteria
WP_073595266.1	vWA+FGS-fold_domain	-	864	Phormidium ambiguum	-	cyanobacteria
WP_073631276.1	vWA+FGS-fold_domain	Trypco2→ TCAD3+TCAD4+CASPASE+EAD1→ EAD1+VMAP-M1+VMAP-C→ MoxR→ vWA+FGS-fold_domain*→ FGS-fold_domain→	947	Scytonema sp. HK-05	GCA_002368235.1	cyanobacteria
WP_073631997.1	vWA+FGS-fold_domain	Trypco1→ Trypsin+EAD9→ EAD9+EAD1+VMAP-C→ MoxR→ vWA+FGS-fold_domain*→ ||← ?||FGS-fold_domain→	958	Scytonema sp. HK-05	GCA_002368235.1	cyanobacteria
WP_073700917.1	vWA+APATPase+wHTH+TPRs	Trypsin+VMAP-M0+VMAP-C→ vWA+APATPase+wHTH+TPRs*→ ||← DUF397||Trypco1→	1263	Nocardiopsis sp. TSRI0078	GCA_001905145.1	actinobacteria
WP_073717857.1	vWA+TPRs	TCAD15||Trypco1→ Trypsin+HDxxH+VMAP-M0+VMAP-C→ MoxR→ vWA+TPRs*→ Radical_SAM→ TIR+APATPase+TPRs→	1138	Streptomyces sp. TSRI0281	GCA_001905125.1	actinobacteria
WP_073719856.1	vWA+APATPase+wHTH+TPRs	EAD2+VMAP-M0+VMAP-C→ MoxR→ vWA+APATPase+wHTH+TPRs*→ AB_hydrolase→ CASPASE+EAD2→ FXSXX-COOH-like→ METALLOPROTEASE+TPRs→	1440	Streptomyces sp. TSRI0281	GCA_001905125.1	actinobacteria
WP_073721464.1	vWA+wHTH+TPRs+wHTH+TPRs+APATPase+wHTH+TPRs	Trypco1→ Trypsin+EAD2+VMAP-M0+VMAP-C→ MoxR→ vWA+wHTH+TPRs+wHTH+TPRs+APATPase+wHTH+TPRs*→	1783	Streptomyces sp. TSRI0281	GCF_001905125.1	actinobacteria
WP_073722820.1	vWA+Pkinase+wHTH+TPRs+cNMP_cyclase	Trypco1→ Trypsin+VMAP-M0+VMAP-C→ MoxR→ vWA+Pkinase+wHTH+TPRs+cNMP_cyclase*→	1311	Streptomyces sp. CB02414	GCA_001905385.1	actinobacteria
WP_073732208.1	vWA+APATPase+wHTH+TPRs	EAD2+VMAP-M0+VMAP-C→ MoxR→ vWA+APATPase+wHTH+TPRs*→ ||← Na_H_Exchanger||AB_hydrolase→ CASPASE+EAD2→ FXSXX-COOH-like→ PSE→ METALLOPROTEASE+TPRs→	1539	Streptomyces sp. CB02414	GCA_001905385.1	actinobacteria
WP_073735943.1	vWA+TPRs	AB_hydrolase→ CASPASE+EAD2+VMAP-M0+VMAP-C→ MoxR→ vWA+TPRs*→	1183	Streptomyces sp. CB02488	-	actinobacteria
WP_073736958.1	vWA+TPRs	TCAD15||Trypco1→ Trypsin+HDxxH+VMAP-M0+VMAP-C→ MoxR→ vWA+TPRs*→ FXSXX-COOH→ Radical_SAM→ METALLOPROTEASE+TPRs→ TIR+APATPase+TPRs→ Trypco1→	1163	Streptomyces sp. CB02488	GCA_001905365.1	actinobacteria
WP_073741611.1	vWA+TPRs	AB_hydrolase→ CASPASE+EAD2+VMAP-M0+VMAP-C→ MoxR→ vWA+TPRs*→	1170	Streptomyces sp. CB02115	-	actinobacteria
WP_073742765.1	vWA+APATPase+wHTH+TPRs	EAD2+VMAP-M0+VMAP-C→ MoxR→ vWA+APATPase+wHTH+TPRs*→ AB_hydrolase→ CASPASE+EAD2→ FXSXX-COOH-like→ METALLOPROTEASE+TPRs→	1484	Streptomyces sp. CB02115	GCF_001905645.1	actinobacteria
WP_073755836.1	vWA+wHTH+TPRs+wHTH+TPRs+APATPase+wHTH+TPRs	Trypsin+EAD2+VMAP-M0+VMAP-C→ MoxR→ vWA+wHTH+TPRs+wHTH+TPRs+APATPase+wHTH+TPRs*→	1790	Streptomyces sp. CB03234	GCF_001905885.1	actinobacteria
WP_073757570.1	vWA+wHTH+TPRs+wHTH+TPRs+APATPase+wHTH+TPRs	Trypco1→ Trypsin+EAD2+VMAP-M0+VMAP-C→ MoxR→ vWA+wHTH+TPRs+wHTH+TPRs+APATPase+wHTH+TPRs*→ ||← AB_hydrolase	1767	Streptomyces sp. CB02923	GCA_001905005.1	actinobacteria
WP_073772252.1	vWA+STAND_NTPase+Trypsin+CASPASE+S1COLD	AB_hydrolase→ CASPASE+VMAP-M0+VMAP-C→ MoxR→ vWA+STAND_NTPase+Trypsin+CASPASE+S1COLD*→	1339	Streptomyces sp. CB02366	GCF_001905905.1	actinobacteria
WP_073777497.1	vWA+APATPase+wHTH+TPRs	EAD2+VMAP-M0+VMAP-C→ MoxR→ vWA+APATPase+wHTH+TPRs*→ AB_hydrolase→ CASPASE+EAD2→ FXSXX-COOH-like→ METALLOPROTEASE+TPRs→	1463	Streptomyces sp. TSRI0445	GCF_001905405.1	actinobacteria
WP_073791958.1	vWA+cNMP_cyclase+TCAD13	AB_hydrolase→ CASPASE+VMAP-M2+VMAP-C→ MoxR→ vWA+cNMP_cyclase+TCAD13*→	1097	Streptomyces sp. CB01580	GCF_001905795.1	actinobacteria
WP_073793562.1	vWA+Pkinase+wHTH+TPRs+cNMP_cyclase	Trypco1→ Trypsin+VMAP-M0+VMAP-C→ MoxR→ vWA+Pkinase+wHTH+TPRs+cNMP_cyclase*→	1289	Streptomyces uncialis	GCF_001905345.1	actinobacteria
WP_073801500.1	vWA+TPRs	AB_hydrolase→ CASPASE+EAD2+VMAP-M0+VMAP-C→ MoxR→ vWA+TPRs*→	1170	Streptomyces sp. TSRI0261	GCF_001905485.1	actinobacteria
WP_073805364.1	vWA+APATPase+wHTH+TPRs	EAD2+VMAP-M0+VMAP-C→ MoxR→ vWA+APATPase+wHTH+TPRs*→ AB_hydrolase→ CASPASE+EAD2→ FXSXX-COOH-like→ METALLOPROTEASE+TPRs→	1489	Streptomyces sp. TSRI0261	GCA_001905485.1	actinobacteria
WP_073848019.1	vWA+APATPase+wHTH+TPRs	cNMP_cyclase+VMAP-M0+VMAP-C→ MoxR→ vWA+APATPase+wHTH+TPRs*→ ||← METALLOPROTEASE+TPRs← FXSXX-COOH-like← cNMP_binding+HTH_Crp_2	1197	Amycolatopsis sp. CB00013	GCA_001905755.1	actinobacteria
WP_073862966.1	vWA+TPRs	TCAD15||Trypco1→ Trypsin+HDxxH+VMAP-M0+VMAP-C→ MoxR→ vWA+TPRs*→ FXSXX-COOH→ Radical_SAM→ METALLOPROTEASE+TPRs→ TIR+APATPase+TPRs→ Trypco1→	1139	Streptomyces sp. CB01249	GCA_001905735.1	actinobacteria
WP_073863467.1	vWA+TPRs	AB_hydrolase→ CASPASE+EAD2+VMAP-M0+VMAP-C→ MoxR→ vWA+TPRs*→	1170	Streptomyces sp. CB01249	GCF_001905735.1	actinobacteria
WP_073866272.1	vWA+TPRs	TCAD15||Trypco1→ Trypsin+HDxxH+VMAP-M0+VMAP-C→ MoxR→ vWA+TPRs*→ Radical_SAM→ TIR+APATPase+TPRs→	936	Streptomyces sp. CB00072	GCF_001905575.1	actinobacteria
WP_073868984.1	vWA+APATPase+wHTH+TPRs	EAD2+VMAP-M0+VMAP-C→ MoxR→ vWA+APATPase+wHTH+TPRs*→ AB_hydrolase→ CASPASE+EAD2→ FXSXX-COOH-like→ METALLOPROTEASE+TPRs→	1483	Streptomyces sp. CB00072	GCF_001905575.1	actinobacteria
WP_073881342.1	vWA+Pkinase+ParA-Soj-PloopNTPase(SIMIBI)	Trypco1→ Trypsin+EAD2+VMAP-M0+VMAP-C→ MoxR→ vWA+Pkinase+ParA-Soj-PloopNTPase(SIMIBI)*→	1142	Streptomyces sp. CB00316	-	actinobacteria
WP_073892180.1	vWA+APATPase+wHTH+TPRs	-	1264	Saccharothrix sp. CB00851	-	actinobacteria
WP_073900317.1	vWA+APATPase+wHTH+TPRs	-	1427	Saccharothrix sp. CB00851	-	actinobacteria
WP_073912307.1	vWA+TPRs	TCAD15||Trypco1→ Trypsin+HDxxH+VMAP-M0+VMAP-C→ MoxR→ vWA+TPRs*→ FXSXX-COOH→ METALLOPROTEASE+TPRs→ TIR+APATPase+TPRs→	1083	Streptomyces sp. CB02009	GCF_001905825.1	actinobacteria
WP_073936870.1	vWA+APATPase+wHTH+TPRs	Trypsin+EAD2→ EAD2+VMAP-M0+VMAP-C→ MoxR→ vWA+APATPase+wHTH+TPRs*→ ||← METALLOPROTEASE+TPRs← Radical_SAM← FXSXX-COOH||Voltage_CLC→	1534	Streptomyces sp. CB02400	GCA_001905725.1	actinobacteria
WP_073938688.1	vWA+Pkinase+wHTH+TPRs+cNMP_cyclase+Band7+HAD	Trypco1→ Trypsin+VMAP-M0+VMAP-C→ MoxR→ vWA+Pkinase+wHTH+TPRs+cNMP_cyclase+Band7+HAD*→	1560	Streptomyces sp. TSRI0107	GCF_001905845.1	actinobacteria
WP_073939274.1	vWA+APATPase+wHTH+TPRs	EAD2+VMAP-M0+VMAP-C→ MoxR→ vWA+APATPase+wHTH+TPRs*→ ||← Na_H_Exchanger||CASPASE+EAD2→ FXSXX-COOH-like→ METALLOPROTEASE+TPRs→	1529	Streptomyces sp. TSRI0107	GCF_001905845.1	actinobacteria
WP_073958207.1	vWA+Pkinase+ParA-Soj-PloopNTPase(SIMIBI)	Trypco1→ Trypsin+EAD2+VMAP-M0+VMAP-C→ MoxR→ vWA+Pkinase+ParA-Soj-PloopNTPase(SIMIBI)*→	1245	Streptomyces sp. TSRI0395	GCF_001905525.1	actinobacteria
WP_073958768.1	vWA+TPRs	AB_hydrolase→ CASPASE+EAD2+VMAP-M0+VMAP-C→ MoxR→ vWA+TPRs*→	1170	Streptomyces sp. TSRI0395	GCF_001905525.1	actinobacteria
WP_073959593.1	vWA+APATPase+wHTH+TPRs	EAD2+VMAP-M0+VMAP-C→ MoxR→ vWA+APATPase+wHTH+TPRs*→ AB_hydrolase→ CASPASE+EAD2→ FXSXX-COOH-like→ METALLOPROTEASE+TPRs→	1484	Streptomyces sp. TSRI0395	GCF_001905525.1	actinobacteria
WP_073963981.1	vWA+TPRs	TCAD15||Trypco1→ Trypsin+HDxxH+VMAP-M0+VMAP-C→ MoxR→ vWA+TPRs*→ FXSXX-COOH→ Radical_SAM→ METALLOPROTEASE+TPRs→ TIR+APATPase+TPRs→	1135	Streptomyces sp. CB02460	GCA_001905705.1	actinobacteria
WP_074992219.1	vWA+Pkinase+cNMP_cyclase+Band7+HAD	Trypco1→ Trypsin+VMAP-M0+VMAP-C→ MoxR→ vWA+Pkinase+cNMP_cyclase+Band7+HAD*→	1268	Streptomyces misionensis	GCA_900104815.1	actinobacteria
WP_075001997.1	vWA+TPRs	Trypco1→ Trypsin+HDxxH+VMAP-M0+VMAP-C→ MoxR→ vWA+TPRs*→ FXSXX-COOH-like→ METALLOPROTEASE+TPRs→ TIR+APATPase+TPRs→	1069	Streptomyces qinglanensis	GCA_900111245.1	actinobacteria
WP_075020915.1	vWA+FtsK+FtsK+FtsK	Trypco1→ Trypsin+VMAP-M8+VMAP-C→ MoxR→ vWA+FtsK+FtsK+FtsK*→	1506	Actinomadura madurae	GCA_900115095.1	actinobacteria
WP_075029687.1	vWA+Pkinase+ParA-Soj-PloopNTPase(SIMIBI)	ADP_ribosyl_GH→ NUDIX→ Trypco1→ Trypsin+EAD2+VMAP-M0+VMAP-C→ MoxR→ vWA+Pkinase+ParA-Soj-PloopNTPase(SIMIBI)*→ SIR2→	1152	Streptomyces mirabilis	GCA_900112965.1	actinobacteria
WP_075128572.1	vWA+APATPase+wHTH+TPRs	cNMP_cyclase+VMAP-M0+VMAP-C→ MoxR→ vWA+APATPase+wHTH+TPRs*→ ||← METALLOPROTEASE+TPRs← FXSXX-COOH-like← cNMP_binding+HTH_Crp_2	1204	Actinophytocola xanthii	GCA_001921205.1	actinobacteria
WP_075137510.1	vWA+APATPase+wHTH+TPRs	cNMP_cyclase+VMAP-M0+VMAP-C→ MoxR→ vWA+APATPase+wHTH+TPRs*→ ||← METALLOPROTEASE+TPRs← FXSXX-COOH-like← cNMP_binding+HTH_Crp_2	1197	Actinophytocola xinjiangensis	GCA_001921215.1	actinobacteria
WP_075347515.1	vWA+APATPase+wHTH+TPRs	EAD2+VMAP-M0+VMAP-C→ MoxR→ vWA+APATPase+wHTH+TPRs*→ AB_hydrolase→ CASPASE+EAD2→ FXSXX-COOH-like→ METALLOPROTEASE+TPRs→	1484	Streptomyces sp. MNU77	GCA_000935135.2	actinobacteria

acc	architecture	operon	len	species	gca	taxend
WP_075598587.1	vWA+FGS-fold_domain	-	862	Oscillatoriales cyanobacterium 'hensonii'	-	cyanobacteria
WP_075601459.1	vWA+FGS-fold_domain	EAD1+Trypsin→ ?→ EAD1+VMAP-M1+VMAP-C→ MoxR→ vWA+FGS-fold_domain*→ four_helix_bundle_protein→ RVT_1→ FGS-fold_domain→	860	Oscillatoriales cyanobacterium 'hensonii'	GCA_001939115.1	cyanobacteria
WP_075660888.1	vWA+TIR+FxsC_Cter(partial)	vWA+TIR+FxsC_Cter(partial)*→	730	Streptomyces acidiscabies	GCF_001941265.1	actinobacteria
WP_075661654.1	vWA+Pkinase+wHTH+cNMP_cyclase+Band7+HAD	vWA+Pkinase+wHTH+cNMP_cyclase+Band7+HAD*→	1037	Streptomyces acidiscabies	GCF_001941265.1	actinobacteria
WP_075692473.1	vWA+TIR+FxsC_Cter(partial)	vWA+TIR+FxsC_Cter(partial)*→	726	Streptomyces acidiscabies	GCF_001941285.1	actinobacteria
WP_075693252.1	vWA+Pkinase+wHTH+cNMP_cyclase+Band7+HAD	vWA+Pkinase+wHTH+cNMP_cyclase+Band7+HAD*→	1230	Streptomyces acidiscabies	GCF_001941285.1	actinobacteria
WP_075735350.1	vWA+Pkinase+wHTH+cNMP_cyclase+Band7+HAD	vWA+Pkinase+wHTH+cNMP_cyclase+Band7+HAD*→	1243	Streptomyces acidiscabies	GCF_001941275.1	actinobacteria
WP_075779362.1	vWA+TIR+FxsC_Cter(partial)	vWA+TIR+FxsC_Cter(partial)*→	726	Streptomyces acidiscabies	GCF_001941325.1	actinobacteria
WP_075779491.1	vWA+Pkinase+wHTH+cNMP_cyclase+Band7+HAD	vWA+Pkinase+wHTH+cNMP_cyclase+Band7+HAD*→	1227	Streptomyces acidiscabies	GCF_001941325.1	actinobacteria
WP_075779623.1	vWA+Pkinase+TPRs	Trypco2→ Trypsin+EAD2+VMAP-M0+VMAP-C→ MoxR→ vWA+Pkinase+TPRs*→	1028	Streptomyces acidiscabies	GCF_001941325.1	actinobacteria
WP_075779895.1	vWA+Pkinase+ParA-Soj-PloopNTPase(SIMIBI)	Trypco1→ Trypsin+EAD2+VMAP-M0+VMAP-C→ MoxR→ vWA+Pkinase+ParA-Soj-PloopNTPase(SIMIBI)*→	1108	Streptomyces acidiscabies	GCF_001941325.1	actinobacteria
WP_075895039.1	vWA+APATPase+wHTH+TPRs	Radical_SAM→ PSE→ ||← PSE||FXSXX-COOH-like→ ?→ EAD2+VMAP-M0+VMAP-C→ MoxR→ vWA+APATPase+wHTH+TPRs*→ ||← FXSXX-COOH-like||cNMP_cyclase+EAD2→	1298	Actinomadura sp. CNU-125	GCA_001942465.1	actinobacteria
WP_075895313.1	vWA_protein	AB_hydrolase→ BTLCP+VMAP-M2+VMAP-C→ MoxR→ vWA_protein*→	821	Actinomadura sp. CNU-125	GCA_001942465.1	actinobacteria
WP_075896676.1	vWA+FGS-fold_domain	AB_hydrolase→ CASPASE+VMAP-M14+VMAP-C→ MoxR→ vWA+FGS-fold_domain*→ FGS-fold_domain→ four_helix_bundle_protein→	900	Moorea bouillonii	GCA_001942495.1	cyanobacteria
WP_075897253.1	vWA+FGS-fold_domain	EAD1+Trypsin→ EAD1+VMAP-M1+Calcineurin-like→ EAD1+VMAP-M1+VMAP-C→ MoxR→ vWA+FGS-fold_domain*→	862	Moorea bouillonii	GCA_001942495.1	cyanobacteria
WP_075925848.1	vWA_protein	Trypsin+EAD2→ VMAP-C→ MoxR→ ?→ vWA_protein*→ TIR+APATPase+TPRs→ ||← DUF397||Trypco1→ ||← PSE← ?← PSE← PSE← DUF397	455	Nocardiopsis sp. CNR-923	GCA_001942255.1	actinobacteria
WP_075927196.1	vWA+APATPase+wHTH	Trypsin+VMAP-M8→ VMAP-C→ MoxR→ vWA+APATPase+wHTH*→	1058	Nocardiopsis sp. CNR-923	GCA_001942255.1	actinobacteria
WP_075971297.1	vWA+Trypsin+cNMP_cyclase+CCCH(TrebleClef)+Pentapeptide_repeats	AB_hydrolase→ CASPASE+VMAP-M0+VMAP-C→ MoxR→ vWA+Trypsin+cNMP_cyclase+CCCH(TrebleClef)+Pentapeptide_repeats*→	1195	Streptomyces sp. TN58	GCA_001941845.1	actinobacteria
WP_076044739.1	vWA+Trypsin+cNMP_cyclase+CCCH(TrebleClef)+Pentapeptide_repeats	AB_hydrolase→ CASPASE+VMAP-M0+VMAP-C→ MoxR→ vWA+Trypsin+cNMP_cyclase+CCCH(TrebleClef)+Pentapeptide_repeats*→	1193	Streptomyces amritsarensis	GCA_001953875.1	actinobacteria
WP_076096475.1	vWA+TPRs	TCAD15→ Trypco1→ Trypsin+HDxxH+VMAP-M0+VMAP-C→ MoxR→ vWA+TPRs*→ Radical_SAM→ TIR+APATPase+TPRs→	1092	Streptomyces sp. IMTB 2501	GCA_001953885.1	actinobacteria
WP_076155767.1	vWA+TPRs	AB_hydrolase→ CASPASE+VMAP-M2+VMAP-C→ MoxR→ vWA+TPRs*→	805	Amycolatopsis coloradensis	GCA_001953865.1	actinobacteria
WP_076161090.1	vWA+APATPase+wHTH+TPRs	cNMP_cyclase+VMAP-M0+VMAP-C→ MoxR→ vWA+APATPase+wHTH+TPRs*→ ||← METALLOPROTEASE+TPRs← FXSXX-COOH-like← cNMP_binding+HTH_Crp_2	1198	Amycolatopsis coloradensis	GCA_001953865.1	actinobacteria
WP_076819102.1	vWA+TPRs	Trypco1→ Trypsin+HDxxH+VMAP-M0+VMAP-C→ MoxR→ vWA+TPRs*→ Voltage_CLC→ FXSXX-COOH-like→ METALLOPROTEASE+TPRs→ TIR+APATPase+TPRs→	929	Frankia sp. M16386	GCA_001983105.1	actinobacteria
WP_076821064.1	vWA+APATPase+wHTH+TPRs	EAD2+EAD1→ EAD2+VMAP-M0+VMAP-C→ MoxR→ vWA+APATPase+wHTH+TPRs*→ ||← METALLOPROTEASE+TPRs	1517	Frankia sp. M16386	GCA_001983105.1	actinobacteria
WP_076831948.1	vWA+APATPase+wHTH+TPRs	EAD2+EAD1→ EAD2+VMAP-M0+VMAP-C→ MoxR→ vWA+APATPase+wHTH+TPRs*→ ||← METALLOPROTEASE+TPRs	1598	Frankia sp. CcI49	GCA_001983215.1	actinobacteria
WP_076913890.1	vWA+REC	AB_hydrolase→ CASPASE+VMAP-M4+VMAP-C→ MoxR→ vWA+REC*→	1172	Burkholderia pseudomallei	GCA_001977425.1	betaproteobacteria
WP_076973926.1	vWA+APATPase+wHTH+TPRs	Trypsin+VMAP-M6+VMAP-C→ MoxR→ vWA+APATPase+wHTH+TPRs*→ ||← METALLOPROTEASE+TPRs← Radical_SAM← Trypco1	1478	Streptomyces sp. M1013	GCA_001970135.1	actinobacteria
WP_076976234.1	vWA+TPRs	TCAD15→ Trypco1→ Trypsin+HDxxH+VMAP-M0+VMAP-C→ MoxR→ vWA+TPRs*→ FXSXX-COOH-like→ METALLOPROTEASE+TPRs→ TIR+APATPase+TPRs→	1097	Streptomyces sp. M1013	GCA_001970135.1	actinobacteria
WP_076994244.1	vWA+APATPase+wHTH+TPRs	EAD2+VMAP-M0+VMAP-C→ MoxR→ vWA+APATPase+wHTH+TPRs*→ ||← METALLOPROTEASE+TPRs← FXSXX-COOH-like||EAD2+VMAP-M0+VMAP-C→ MoxR→ vWA+APATPase+wHTH+TPRs→ ||← METALLOPROTEASE+TPRs← FXSXX-COOH-like	1432	Actinosynnema sp. ALI-1.44	GCA_001984155.1	actinobacteria
WP_076994249.1	vWA+APATPase+wHTH+TPRs	EAD2+VMAP-M0+VMAP-C→ MoxR→ vWA+APATPase+wHTH+TPRs→ ||← METALLOPROTEASE+TPRs← FXSXX-COOH-like||EAD2+VMAP-M0+VMAP-C→ MoxR→ vWA+APATPase+wHTH+TPRs*→ ||← METALLOPROTEASE+TPRs← FXSXX-COOH-like	1394	Actinosynnema sp. ALI-1.44	GCA_001984155.1	actinobacteria
WP_077011609.1	vWA+APATPase+wHTH+TPRs	EAD2+VMAP-M0+VMAP-C→ MoxR→ vWA+APATPase+wHTH+TPRs*→ ||← METALLOPROTEASE+TPRs← cNMP_cyclase+EAD2	1322	Saccharothrix sp. ALI-22-I	GCA_001984175.1	actinobacteria
WP_077058987.1	vWA+TPRs	TCAD15||Trypco1→ Trypsin+HDxxH+VMAP-M0+VMAP-C→ MoxR→ vWA+TPRs*→ FXSXX-COOH→ Radical_SAM→ METALLOPROTEASE+TPRs→ TIR+APATPase+TPRs→	1097	Streptomyces sp. MP131-18	GCF_001984575.1	actinobacteria
WP_077060243.1	vWA+Pkinase+ParA-Soj-PloopNTPase(SIMIBI)	Trypco1→ Trypsin+EAD2+VMAP-M0+VMAP-C→ MoxR→ vWA+Pkinase+ParA-Soj-PloopNTPase(SIMIBI)*→	1126	Streptomyces sp. MP131-18	GCA_001984575.1	actinobacteria
WP_077116335.1	vWA+TPRs	Trypco1→ Trypsin+HDxxH+VMAP-M0+VMAP-C→ MoxR→ vWA+TPRs*→ Voltage_CLC→ METALLOPROTEASE→ TIR+APATPase+wHTH+TPRs→	801	Nocardia donostiensis	GCA_001989395.1	actinobacteria
WP_077193459.1	vWA+TPRs	TCAD15||Trypco1→ Trypsin+HDxxH+VMAP-M0+VMAP-C→ MoxR→ vWA+TPRs*→ FXSXX-COOH→ Radical_SAM→ TIR+APATPase+TPRs→	1136	Streptomyces lydicus	GCF_001984445.1	actinobacteria
WP_077692791.1	vWA+APATPase+wHTH+TPRs	Trypsin+VMAP-M0+VMAP-C→ vWA+APATPase+wHTH+TPRs*→ ||← DUF397||Trypco1→	1264	Nocardiopsis sinuspersici	GCA_001998325.1	actinobacteria
WP_077796725.1	vWA+Pkinase+ParA-Soj-PloopNTPase(SIMIBI)	Trypco1→ Trypsin+EAD2+VMAP-M0+VMAP-C→ MoxR→ vWA+Pkinase+ParA-Soj-PloopNTPase(SIMIBI)*→	1114	Streptomyces sp. JHA26	GCF_002005565.1	actinobacteria
WP_078067203.1	vWA+TPRs	TCAD15||PSE→ Trypsin+HDxxH+VMAP-M0+VMAP-C→ MoxR→ vWA+TPRs*→ FXSXX-COOH→ Radical_SAM→ METALLOPROTEASE+TPRs→ TIR+APATPase+TPRs→	1145	Streptomyces jeddahensis	GCA_001642995.1	actinobacteria
WP_078075117.1	vWA+TPRs	TCAD15||PSE→ Trypsin+HDxxH+VMAP-M0+VMAP-C→ MoxR→ vWA+TPRs*→ FXSXX-COOH→ METALLOPROTEASE+TPRs→ TIR+APATPase+TPRs→	1035	Streptomyces niveus	-	actinobacteria
WP_078095066.1	vWA+TPRs	TCAD15||Trypsin+HDxxH+VMAP-M0+VMAP-C→ MoxR→ vWA+TPRs*→ FXSXX-COOH→ Radical_SAM→ METALLOPROTEASE+TPRs→ TIR+APATPase+TPRs→	1099	Streptomyces sp. fd1-xmd	GCF_002007685.1	actinobacteria
WP_078095390.1	vWA+Trypsin+cNMP_cyclase+CCCH(TrebleClef)+Pentapeptide_repeats	AB_hydrolase→ CASPASE+VMAP-M0+VMAP-C→ MoxR→ vWA+Trypsin+cNMP_cyclase+CCCH(TrebleClef)+Pentapeptide_repeats*→	1192	Streptomyces sp. fd1-xmd	GCA_002007685.1	actinobacteria
WP_078490521.1	vWA+Pkinase+wHTH+TPRs	Trypco1→ Trypsin+VMAP-M0+VMAP-C→ MoxR→ ||← ?||vWA+Pkinase+wHTH+TPRs*→	1072	Streptomyces scopuliridis	-	actinobacteria
WP_078496221.1	vWA+APATPase+wHTH+TPRs	EAD2+VMAP-M0+VMAP-C→ MoxR→ vWA+APATPase+wHTH+TPRs*→ AB_hydrolase→ CASPASE+EAD2→ FXSXX-COOH-like→ METALLOPROTEASE+TPRs→	1479	Streptomyces sp. W007	GCF_000239075.1	actinobacteria
WP_078506948.1	vWA	EAD2+VMAP-M0+VMAP-C→ MoxR→ vWA*→ TIR+APATPase+TPRs→ PSE→ CASPASE+EAD2→ FXSXX-COOH-like→ METALLOPROTEASE+TPRs→	441	Streptomyces viridochromogenes	-	actinobacteria

<table>
<tr><th>acc</th><th>architecture</th><th>operon</th><th>len</th><th>species</th><th>gca</th><th>taxend</th></tr>
<tr><td>WP_078559684.1</td><td>vWA+wHTH+TPRs+wHTH+TPRs+BetaPropeller</td><td>Trypco1→ Trypsin+EAD2+VMAP-M0+VMAP-C→ MoxR→ vWA+wHTH+TPRs+wHTH+TPRs+BetaPropeller*→</td><td>1391</td><td>Streptomyces sp. PRh5</td><td>-</td><td>actinobacteria</td></tr>
<tr><td>WP_078610845.1</td><td>vWA+APATPase+wHTH+TPRs</td><td>EAD2+VMAP-M0+VMAP-C→ MoxR→ vWA+APATPase+wHTH+TPRs*→ ||← METALLOPROTEASE+TPRs← FXSXX-COOH-like← ?← ?← Na_H_Exchanger</td><td>1444</td><td>Streptomyces</td><td>-</td><td>actinobacteria</td></tr>
<tr><td>WP_078621013.1</td><td>vWA+APATPase+wHTH+TPRs</td><td>vWA+APATPase+wHTH+TPRs*→ ||← METALLOPROTEASE+TPRs← CASPASE+EAD2</td><td>1336</td><td>Streptomyces aurantiacus</td><td>-</td><td>actinobacteria</td></tr>
<tr><td>WP_078621095.1</td><td>vWA+Pkinase+wHTH+TPRs+cNMP_cyclase+Band7+HAD</td><td>Trypco1→ Trypsin+VMAP-M0+VMAP-C→ MoxR→ vWA+Pkinase+wHTH+TPRs+cNMP_cyclase+Band7+HAD*→</td><td>1658</td><td>Streptomyces sp. NRRL WC-3626</td><td>GCF_000718785.1</td><td>actinobacteria</td></tr>
<tr><td>WP_078621929.1</td><td>vWA+APATPase+wHTH+TPRs+NaeI</td><td>AB_hydrolase→ CASPASE+VMAP-M2+VMAP-C→ MoxR→ vWA+APATPase+wHTH+TPRs+NaeI*→</td><td>1286</td><td>Streptomyces sp. NRRL S-244</td><td>-</td><td>actinobacteria</td></tr>
<tr><td>WP_078625713.1</td><td>vWA+HTH+HTH+ParA-Soj-PloopNTPase(SIMIBI)</td><td>CASPASE+VMAP-M2+VMAP-C→ MoxR→ vWA+HTH+HTH+ParA-Soj-PloopNTPase(SIMIBI)*→ PSE→ PSE→ ||← ?||?→ ||← ?||?→ DUF397→</td><td>956</td><td>Streptomyces sp. CNB091</td><td>GCF_000377965.1</td><td>actinobacteria</td></tr>
<tr><td>WP_078630591.1</td><td>vWA+APATPase+wHTH+TPRs</td><td>EAD2+VMAP-M0+VMAP-C→ MoxR→ vWA+APATPase+wHTH+TPRs*→ ||← METALLOPROTEASE+TPRs← FXSXX-COOH-like</td><td>1355</td><td>Streptomyces roseochromogenus</td><td>GCF_000497445.1</td><td>actinobacteria</td></tr>
<tr><td>WP_078633428.1</td><td>vWA+REase+cNMP_cyclase+CASPASE</td><td>AB_hydrolase→ CASPASE+EAD2+VMAP-M0+VMAP-C→ MoxR→ vWA+REase+cNMP_cyclase+CASPASE*→</td><td>1160</td><td>Streptomyces antibioticus</td><td>GCF_002019855.1</td><td>actinobacteria</td></tr>
<tr><td>WP_078633957.1</td><td>vWA+APATPase+wHTH+TPRs</td><td>EAD2+VMAP-M0+VMAP-C→ vWA+APATPase+wHTH+TPRs*→ ||← METALLOPROTEASE+TPRs← FXSXX-COOH-like← PSE||METALLOPROTEASE+TPRs→</td><td>1358</td><td>Streptomyces antibioticus</td><td>GCF_002019855.1</td><td>actinobacteria</td></tr>
<tr><td>WP_078636541.1</td><td>vWA+APATPase+wHTH+TPRs</td><td>TCAD9+Pkinase+NACHT+HEAT_repeats→ EAD2+VMAP-M0+VMAP-C→ vWA→ MoxR→ vWA+APATPase+TPRs*→</td><td>991</td><td>Streptomyces antibioticus</td><td>GCF_002019855.1</td><td>actinobacteria</td></tr>
<tr><td>WP_078636543.1</td><td>vWA</td><td>TCAD9+Pkinase+NACHT+HEAT_repeats→ EAD2+VMAP-M0+VMAP-C→ vWA*→ MoxR→ vWA+APATPase+TPRs→</td><td>433</td><td>Streptomyces antibioticus</td><td>GCA_002019855.1</td><td>actinobacteria</td></tr>
<tr><td>WP_078639145.1</td><td>vWA+APATPase+wHTH+TPRs</td><td>PNP_UDP_1+EAD2→ EAD2+VMAP-M0+VMAP-C→ MoxR→ vWA+APATPase+wHTH+TPRs*→ ||← METALLOPROTEASE+TPRs</td><td>1452</td><td>Streptomyces hygroscopicus</td><td>GCA_002021875.1</td><td>actinobacteria</td></tr>
<tr><td>WP_078640352.1</td><td>vWA+Pkinase+ZNR+Pkinase+ParA-Soj-PloopNTPase(SIMIBI)</td><td>SIR2→ ||← ?← ?||Trypco1→ Trypsin+EAD2+VMAP-M0+VMAP-C→ MoxR→ vWA+Pkinase+ZNR+Pkinase+ParA-Soj-PloopNTPase(SIMIBI)*→ Porphobil_deam+Porphobil_deamC→</td><td>1524</td><td>Streptomyces hygroscopicus</td><td>GCA_002021875.1</td><td>actinobacteria</td></tr>
<tr><td>WP_078641309.1</td><td>vWA+TPRs</td><td>AB_hydrolase→ CASPASE+EAD2+VMAP-M0+VMAP-C→ MoxR→ vWA+TPRs*→</td><td>1171</td><td>Streptomyces hygroscopicus</td><td>GCA_002021875.1</td><td>actinobacteria</td></tr>
<tr><td>WP_078759921.1</td><td>vWA+APATPase+wHTH+TPRs</td><td>Trypsin+VMAP-M0+VMAP-C→ MoxR→ vWA+APATPase+wHTH+TPRs*→ ||← DUF397||Trypco1→</td><td>1508</td><td>Marinactinospora thermotolerans</td><td>GCA_900167435.1</td><td>actinobacteria</td></tr>
<tr><td>WP_078841454.1</td><td>vWA+TPRs</td><td>TCAD15||PSE→ Trypsin+HDxxH+VMAP-M0+VMAP-C→ MoxR→ vWA+TPRs*→ FXSXX-COOH→ Radical_SAM→ METALLOPROTEASE+TPRs→ TIR+APATPase+TPRs→</td><td>1080</td><td>Streptomyces acidiscabies</td><td>GCF_001189015.1</td><td>actinobacteria</td></tr>
<tr><td>WP_078855504.1</td><td>vWA+Pkinase+wHTH+TPRs+cNMP_cyclase</td><td>vWA+Pkinase+wHTH+TPRs+cNMP_cyclase*→</td><td>1130</td><td>Streptomyces sp. MBT28</td><td>-</td><td>actinobacteria</td></tr>
<tr><td>WP_078866458.1</td><td>vWA+TPRs</td><td>TCAD15||PSE→ Trypsin+HDxxH+VMAP-M0+VMAP-C→ MoxR→ vWA+TPRs*→ FXSXX-COOH→ Radical_SAM→ TIR+APATPase+TPRs→</td><td>1198</td><td>Streptomyces sp. NRRL S-1448</td><td>-</td><td>actinobacteria</td></tr>
<tr><td>WP_078869289.1</td><td>vWA+APATPase+wHTH+TPRs</td><td>Trypsin+EAD2→ EAD2+VMAP-M0+VMAP-C→ MoxR→ vWA+APATPase+wHTH+TPRs*→ ||← METALLOPROTEASE+TPRs← FXSXX-COOH-like</td><td>1320</td><td>Streptomyces sp. NRRL B-1347</td><td>-</td><td>actinobacteria</td></tr>
<tr><td>WP_078872795.1</td><td>vWA+TPRs</td><td>MoxR→ vWA+Trypsin+HDxxH+VMAP-M0+VMAP-C→ ||← ?||PSE→ MoxR→ ||← ?||vWA+TPRs*→ FXSXX-COOH→ METALLOPROTEASE+TPRs→ TIR+APATPase+TPRs→</td><td>1045</td><td>Streptomyces sp. NRRL S-1868</td><td>-</td><td>actinobacteria</td></tr>
<tr><td>WP_078872798.1</td><td>vWA+Trypsin+HDxxH+VMAP-M0+VMAP-C</td><td>TCAD15→ ?→ ?→ PSE→ ?→ MoxR→ vWA+Trypsin+HDxxH+VMAP-M0+VMAP-C*→ ||← ?||PSE→ MoxR→ ||← ?||vWA+TPRs→ FXSXX-COOH→ METALLOPROTEASE+TPRs→ TIR+APATPase+TPRs→</td><td>1090</td><td>Streptomyces sp. NRRL S-1868</td><td>-</td><td>actinobacteria</td></tr>
<tr><td>WP_078877222.1</td><td>vWA+TPRs</td><td>TCAD15||Trypco1→ Trypsin+HDxxH+VMAP-M0+VMAP-C→ MoxR→ vWA+TPRs*→ FXSXX-COOH→ Radical_SAM→ METALLOPROTEASE+TPRs→ TIR+APATPase+TPRs→</td><td>1142</td><td>Streptomyces sp. 150FB</td><td>-</td><td>actinobacteria</td></tr>
<tr><td>WP_078884155.1</td><td>vWA+APATPase+wHTH+TPRs</td><td>Trypsin+EAD2→ EAD2+VMAP-M0+VMAP-C→ MoxR→ ?→ vWA+APATPase+wHTH+TPRs*→ ||← METALLOPROTEASE+TPRs← Radical_SAM← FXSXX-COOH-like||Voltage_CLC→</td><td>1363</td><td>Streptomyces sp. NRRL S-340</td><td>-</td><td>actinobacteria</td></tr>
<tr><td>WP_078885533.1</td><td>vWA+wHTH+TPRs+APATPase+wHTH+TPRs</td><td>Trypco1→ Trypsin+EAD2+VMAP-M0+VMAP-C→ MoxR→ vWA+wHTH+TPRs+APATPase+wHTH+TPRs*→ ||← ?||?→ ||← AB_hydrolase</td><td>1438</td><td>Streptomyces sp. NBRC 110027</td><td>-</td><td>actinobacteria</td></tr>
<tr><td>WP_078889789.1</td><td>vWA+APATPase+wHTH+TPRs+SFI-helicase</td><td>AB_hydrolase→ CASPASE+VMAP-M2+VMAP-C→ MoxR→ vWA+APATPase+wHTH+TPRs+SFI-helicase*→</td><td>1581</td><td>Streptomyces sp. NRRL S-1813</td><td>GCF_000719605.1</td><td>actinobacteria</td></tr>
<tr><td>WP_078892246.1</td><td>vWA+Pkinase+wHTH+TPRs+cNMP_cyclase+HAD</td><td>Trypsin+VMAP-M0+VMAP-C→ MoxR→ vWA+Pkinase+wHTH+TPRs+cNMP_cyclase+HAD*→</td><td>1619</td><td>Streptomyces olivaceus</td><td>-</td><td>actinobacteria</td></tr>
<tr><td>WP_078893851.1</td><td>vWA+PNP_UDP_1+APATPase+wHTH+TPRs</td><td>AB_hydrolase→ CASPASE+EAD2+VMAP-M0+VMAP-C→ MoxR→ vWA+PNP_UDP_1+APATPase+wHTH+TPRs*→</td><td>1472</td><td>Streptomyces sp. NRRL S-1022</td><td>GCF_000720135.1</td><td>actinobacteria</td></tr>
<tr><td>WP_078906829.1</td><td>vWA+Trypsin+cNMP_cyclase+CCCH(TrebleClef)+Pentapeptide_repeats</td><td>AB_hydrolase→ CASPASE+VMAP-M0+VMAP-C→ MoxR→ ||← ?||vWA+Trypsin+cNMP_cyclase+CCCH(TrebleClef)+Pentapeptide_repeats*→</td><td>1158</td><td>Streptomyces sp. NRRL S-378</td><td>-</td><td>actinobacteria</td></tr>
<tr><td>WP_078914741.1</td><td>vWA+CASPASE+TCAD14+PIN+CCCH(ZNR)+FHA</td><td>Trypco1→ Trypsin+HDxxH+VMAP-M0+VMAP-C→ MoxR→ ||← ?||vWA+CASPASE+TCAD14+PIN+CCCH(ZNR)+FHA*→</td><td>1145</td><td>Streptomyces sp. NRRL S-384</td><td>-</td><td>actinobacteria</td></tr>
<tr><td>WP_078916501.1</td><td>vWA+Pkinase+wHTH+TPRs+cNMP_cyclase+Band7+HAD</td><td>vWA+Pkinase+wHTH+TPRs+cNMP_cyclase+Band7+HAD*→</td><td>1579</td><td>Streptomyces viridochromogenes</td><td>-</td><td>actinobacteria</td></tr>
<tr><td>WP_078935568.1</td><td>vWA+Pkinase+wHTH+TPRs+cNMP_cyclase</td><td>Trypco1→ Trypsin+VMAP-M0+VMAP-C→ MoxR→ vWA+Pkinase+wHTH+TPRs+cNMP_cyclase*→</td><td>1183</td><td>Streptomyces europaeiscabiei</td><td>-</td><td>actinobacteria</td></tr>
<tr><td>WP_078942262.1</td><td>vWA+APATPase+wHTH+TPRs</td><td>EAD2+VMAP-M0+VMAP-C→ MoxR→ vWA+APATPase+wHTH+TPRs*→ AB_hydrolase→ CASPASE+EAD2→ FXSXX-COOH-like→ METALLOPROTEASE+TPRs→</td><td>1463</td><td>Streptomyces mediolani</td><td>GCF_000721685.1</td><td>actinobacteria</td></tr>
<tr><td>WP_078942771.1</td><td>vWA+Pkinase+wHTH+TPRs+cNMP_cyclase+Band7+HAD</td><td>vWA+Pkinase+wHTH+TPRs+cNMP_cyclase+Band7+HAD*→</td><td>1583</td><td>Streptomyces viridochromogenes</td><td>-</td><td>actinobacteria</td></tr>
<tr><td>WP_078943028.1</td><td>vWA+APATPase+wHTH+TPRs</td><td>VMAP-M25+VMAP-M0+VMAP-C→ MoxR→ vWA+APATPase+wHTH+TPRs*→ AB_hydrolase→ CASPASE+EAD2→ METALLOPROTEASE+TPRs→</td><td>1298</td><td>Streptomyces aureus</td><td>-</td><td>actinobacteria</td></tr>
<tr><td>WP_078946634.1</td><td>vWA+Pkinase+wHTH+TPRs</td><td>Trypco1→ Trypsin+VMAP-M0+VMAP-C→ MoxR→ vWA+Pkinase+wHTH+TPRs*→</td><td>1081</td><td>Streptomyces sp. NRRL B-3648</td><td>GCF_001279545.1</td><td>actinobacteria</td></tr>
<tr><td>WP_078953405.1</td><td>vWA+APATPase+wHTH+TPRs+SFI-helicase</td><td>AB_hydrolase→ CASPASE+VMAP-M2+VMAP-C→ MoxR→ vWA+APATPase+wHTH+TPRs+SFI-helicase*→</td><td>1581</td><td>Streptomyces decoyicus</td><td>GCF_001270575.1</td><td>actinobacteria</td></tr>
<tr><td>WP_078957678.1</td><td>vWA+Pkinase+wHTH+TPRs+cNMP_cyclase+Band7+HAD</td><td>Trypco1→ Trypsin+VMAP-M0+VMAP-C→ MoxR→ vWA+Pkinase+wHTH+TPRs+cNMP_cyclase+Band7+HAD*→</td><td>1547</td><td>Streptomyces glaucescens</td><td>GCA_000761215.1</td><td>actinobacteria</td></tr>
<tr><td>WP_078961270.1</td><td>vWA+APATPase+wHTH+TPRs</td><td>EAD2+VMAP-M0+VMAP-C→ MoxR→ vWA+APATPase+wHTH+TPRs*→ AB_hydrolase→ CASPASE+EAD2→ FXSXX-COOH-like→ METALLOPROTEASE+TPRs→</td><td>1484</td><td>Streptomyces</td><td>GCF_001189025.1</td><td>actinobacteria</td></tr>
<tr><td>WP_078968245.1</td><td>vWA+SFII-helicase+Trypsin+cNMP_cyclase+Coiled-coil+CASPASE</td><td>AB_hydrolase→ CASPASE+VMAP-M2+VMAP-C→ MoxR→ vWA+SFII-helicase+Trypsin+cNMP_cyclase+Coiled-coil+CASPASE*→</td><td>1796</td><td>Streptomyces</td><td>GCF_001280095.1</td><td>actinobacteria</td></tr>
<tr><td>WP_078969206.1</td><td>vWA+Pkinase+NUDIX</td><td>VMAP-M2+VMAP-C→ MoxR→ vWA+Pkinase+NUDIX*→</td><td>1259</td><td>Streptomyces cyaneogriseus</td><td>-</td><td>actinobacteria</td></tr>
<tr><td>WP_078969534.1</td><td>vWA+TPRs</td><td>TCAD15||Trypco1→ Trypsin+HDxxH+VMAP-M0+VMAP-C→ MoxR→ vWA+TPRs*→ FXSXX-COOH→ Radical_SAM→ METALLOPROTEASE+TPRs→ TIR+APATPase+TPRs→</td><td>1095</td><td>Streptomyces cyaneogriseus</td><td>GCF_000931445.1</td><td>actinobacteria</td></tr>
<tr><td>WP_078970667.1</td><td>vWA+APATPase+wHTH+TPRs</td><td>Trypco1→ Trypsin+EAD2+VMAP-M0+VMAP-C→ MoxR→ vWA+APATPase+wHTH+TPRs*→ ||← AB_hydrolase</td><td>1187</td><td>Streptomyces chattanoogensis</td><td>-</td><td>actinobacteria</td></tr>
<tr><td>WP_078978683.1</td><td>vWA+BetaPropeller</td><td>CASPASE+VMAP-M19+VMAP-C→ MoxR→ vWA+BetaPropeller*→</td><td>897</td><td>Streptomyces scabrisporus</td><td>GCA_002024165.1</td><td>actinobacteria</td></tr>
<tr><td>WP_079023028.1</td><td>vWA+Pkinase+wHTH+TPRs+cNMP_cyclase</td><td>Trypco1→ Trypsin+VMAP-M0+VMAP-C→ MoxR→ vWA+Pkinase+wHTH+TPRs+cNMP_cyclase*→</td><td>1155</td><td>Streptomyces sp. NRRL B-24891</td><td>-</td><td>actinobacteria</td></tr>
</table>

<table>
<tr><th>acc</th><th>architecture</th><th>operon</th><th>len</th><th>species</th><th>gca</th><th>taxend</th></tr>
<tr><td>WP_079024941.1</td><td>vWA+TPRs</td><td>TCAD15||Trypco1→ Trypsin+HDxxH+VMAP-M0+VMAP-C→ MoxR→ vWA+TPRs*→ FXSXX-COOH→ Radical_SAM→ METALLOPROTEASE+TPRs→ TIR+APATPase+TPRs→</td><td>1095</td><td>Streptomyces leeuwenhoekii</td><td>GCF_001047315.1</td><td>actinobacteria</td></tr>
<tr><td>WP_079030148.1</td><td>vWA+Pkinase+wHTH+TPRs+cNMP_cyclase+HAD</td><td>Trypco1→ Trypsin+VMAP-M0+VMAP-C→ ?→ MoxR→ vWA+Pkinase+wHTH+TPRs+cNMP_cyclase+HAD*→</td><td>1542</td><td>Streptomyces ambofaciens</td><td>GCA_001267885.1</td><td>actinobacteria</td></tr>
<tr><td>WP_079041858.1</td><td>vWA+Pkinase+TPRs</td><td>vWA+Pkinase+TPRs*→</td><td>894</td><td>Streptomyces aureus</td><td>GCF_001418135.1</td><td>actinobacteria</td></tr>
<tr><td>WP_079044303.1</td><td>vWA+APATPase+wHTH+TPRs</td><td>EAD2+VMAP-M0+VMAP-C→ MoxR→ vWA+APATPase+wHTH+TPRs*→ AB_hydrolase→ CASPASE+EAD2→ FXSXX-COOH-like→ METALLOPROTEASE+TPRs→</td><td>1484</td><td>Streptomyces anulatus</td><td>GCF_001434355.1</td><td>actinobacteria</td></tr>
<tr><td>WP_079051130.1</td><td>vWA+Pkinase+wHTH+TPRs+cNMP_cyclase+Band7+HAD</td><td>Trypsin+VMAP-M0+VMAP-C→ MoxR→ vWA+Pkinase+wHTH+TPRs+cNMP_cyclase+Band7+HAD*→</td><td>1558</td><td>Streptomyces curacoi</td><td>-</td><td>actinobacteria</td></tr>
<tr><td>WP_079054844.1</td><td>vWA+Pkinase+ParA-Soj-PloopNTPase(SIMIBI)</td><td>Trypco1→ Trypsin+EAD2+VMAP-M0+VMAP-C→ MoxR→ vWA+Pkinase+ParA-Soj-PloopNTPase(SIMIBI)*→</td><td>958</td><td>Streptomyces neyagawaensis</td><td>GCF_001418645.1</td><td>actinobacteria</td></tr>
<tr><td>WP_079059666.1</td><td>vWA+TIR+cNMP_cyclase+S1COLD+EAD2</td><td>AB_hydrolase→ CASPASE+EAD2+VMAP-M0+VMAP-C→ MoxR→ vWA+TIR+cNMP_cyclase+S1COLD+EAD2*→</td><td>1102</td><td>Streptomyces violaceusniger</td><td>GCF_001509775.1</td><td>actinobacteria</td></tr>
<tr><td>WP_079065161.1</td><td>vWA+APATPase+wHTH+TPRs</td><td>EAD2+VMAP-M0+VMAP-C→ vWA+APATPase+wHTH+TPRs*→ ||← METALLOPROTEASE+TPRs← FXSXX-COOH-like← Trypsin+EAD2← Trypco2||METALLOPROTEASE+TPRs→</td><td>1317</td><td>Streptomyces olivochromogenes</td><td>-</td><td>actinobacteria</td></tr>
<tr><td>WP_079065729.1</td><td>vWA+CASPASE+TIR+NUDIX</td><td>Trypco1→ Trypsin+HDxxH+VMAP-M0+VMAP-C→ MoxR→ vWA+CASPASE+TIR+NUDIX*→</td><td>1286</td><td>Streptomyces olivochromogenes</td><td>GCA_002335465.1</td><td>actinobacteria</td></tr>
<tr><td>WP_079078451.1</td><td>vWA+TPRs</td><td>TCAD15||Trypco1→ Trypsin+HDxxH+VMAP-M0+VMAP-C→ MoxR→ vWA+TPRs*→ FXSXX-COOH→ Radical_SAM→ METALLOPROTEASE+TPRs→ TIR+APATPase+TPRs→</td><td>1090</td><td>Streptomyces sp. CdTB01</td><td>-</td><td>actinobacteria</td></tr>
<tr><td>WP_079082984.1</td><td>vWA+Pkinase+wHTH+TPRs+cNMP_cyclase+Band7+HAD</td><td>Trypco1→ Trypsin+VMAP-M0+VMAP-C→ MoxR→ vWA+Pkinase+wHTH+TPRs+cNMP_cyclase+Band7+HAD*→</td><td>1536</td><td>Streptomyces antibioticus</td><td>GCF_001514065.1</td><td>actinobacteria</td></tr>
<tr><td>WP_079084658.1</td><td>vWA+Pkinase+wHTH+TPRs+cNMP_cyclase+Band7+HAD</td><td>Trypco1→ Trypsin+VMAP-M0+VMAP-C→ MoxR→ vWA+Pkinase+wHTH+TPRs+cNMP_cyclase+Band7+HAD*→</td><td>1646</td><td>Streptomyces sp. RV15</td><td>GCF_001514305.1</td><td>actinobacteria</td></tr>
<tr><td>WP_079104732.1</td><td>vWA+Pkinase+wHTH+TPRs+cNMP_cyclase</td><td>Trypco1→ Trypsin+VMAP-M0+VMAP-C→ MoxR→ vWA+Pkinase+wHTH+TPRs+cNMP_cyclase*→</td><td>1153</td><td>Streptomyces europaeiscabiei</td><td>-</td><td>actinobacteria</td></tr>
<tr><td>WP_079123647.1</td><td>vWA+TPRs</td><td>TCAD15||Trypco1→ Trypsin+HDxxH+VMAP-M0+VMAP-C→ MoxR→ vWA+TPRs*→ FXSXX-COOH→ Radical_SAM→ TIR+APATPase+TPRs→</td><td>1212</td><td>Streptomyces sp. NBRC 110611</td><td>GCF_001590865.1</td><td>actinobacteria</td></tr>
<tr><td>WP_079125114.1</td><td>vWA+TPRs</td><td>TCAD15||Trypco1→ Trypsin+HDxxH+VMAP-M0+VMAP-C→ MoxR→ vWA+TPRs*→ FXSXX-COOH→ Radical_SAM→ METALLOPROTEASE+TPRs→ TIR+APATPase+TPRs→</td><td>1105</td><td>Streptomyces lushanensis</td><td>GCF_001700515.1</td><td>actinobacteria</td></tr>
<tr><td>WP_079127044.1</td><td>vWA+Pkinase+wHTH+TPRs+cNMP_cyclase</td><td>Trypco1→ PSE→ MoxR→ vWA+Pkinase+wHTH+TPRs+cNMP_cyclase*→</td><td>1217</td><td>Streptomyces sp. CC71</td><td>GCF_001595515.1</td><td>actinobacteria</td></tr>
<tr><td>WP_079127936.1</td><td>vWA+STAND_NTPase+BetaPropeller+REase(Mrr_cat-PDDEXK)</td><td>AB_hydrolase→ CASPASE+VMAP-M0+VMAP-C→ MoxR→ vWA+STAND_NTPase+BetaPropeller+REase(Mrr_cat-PDDEXK)*→</td><td>1081</td><td>Streptomyces niveus</td><td>GCF_001723055.1</td><td>actinobacteria</td></tr>
<tr><td>WP_079134774.1</td><td>vWA+APATPase+wHTH+TPRs</td><td>EAD2+VMAP-M0+VMAP-C→ MoxR→ vWA+APATPase+wHTH+TPRs*→</td><td>1479</td><td>Streptomyces sp. EN16</td><td>GCF_001746285.1</td><td>actinobacteria</td></tr>
<tr><td>WP_079138844.1</td><td>vWA+APATPase+wHTH+TPRs</td><td>vWA+APATPase+wHTH+TPRs*→ ||← METALLOPROTEASE+TPRs← Trypsin+EAD2← Trypco2</td><td>1266</td><td>Streptomyces rubidus</td><td>GCF_001723065.1</td><td>actinobacteria</td></tr>
<tr><td>WP_079141625.1</td><td>vWA+cNMP_cyclase+Band7+HAD</td><td>vWA+cNMP_cyclase+Band7+HAD*→</td><td>949</td><td>Streptomyces sp. LUP30</td><td>-</td><td>actinobacteria</td></tr>
<tr><td>WP_079141871.1</td><td>vWA+TPRs</td><td>Trypsin+HDxxH+VMAP-M0+VMAP-C→ MoxR→ vWA+TPRs*→ FXSXX-COOH→ Radical_SAM→ METALLOPROTEASE+TPRs→ TIR+APATPase+TPRs→</td><td>1109</td><td>Streptomyces sp. LUP30</td><td>-</td><td>actinobacteria</td></tr>
<tr><td>WP_079146592.1</td><td>vWA+APATPase+wHTH+TPRs</td><td>Trypco2→ EAD2+VMAP-M0+VMAP-C→ vWA+APATPase+wHTH+TPRs*→ ||← METALLOPROTEASE+TPRs← Trypsin+EAD2||?→ Trypco2→</td><td>1259</td><td>Streptomyces griseochromogenes</td><td>-</td><td>actinobacteria</td></tr>
<tr><td>WP_079147211.1</td><td>vWA+Pkinase+wHTH+TPRs+cNMP_cyclase+Band7</td><td>Trypco1→ Trypsin+VMAP-M0+VMAP-C→ MoxR→ vWA+Pkinase+wHTH+TPRs+cNMP_cyclase+Band7*→</td><td>1401</td><td>Streptomyces griseochromogenes</td><td>GCF_001542625.1</td><td>actinobacteria</td></tr>
<tr><td>WP_079154905.1</td><td>vWA+Pkinase+wHTH+TPRs+cNMP_cyclase+HAD</td><td>Trypsin+VMAP-M0+VMAP-C→ MoxR→ vWA+Pkinase+wHTH+TPRs+cNMP_cyclase+HAD*→</td><td>1616</td><td>Streptomyces pactum</td><td>-</td><td>actinobacteria</td></tr>
<tr><td>WP_079155447.1</td><td>vWA+Pkinase+wHTH+TPRs+cNMP_cyclase+HAD</td><td>Trypco1→ Trypsin+VMAP-M0+VMAP-C→ MoxR→ vWA+Pkinase+wHTH+TPRs+cNMP_cyclase+HAD*→</td><td>1542</td><td>Streptomyces ambofaciens</td><td>GCA_001632865.1</td><td>actinobacteria</td></tr>
<tr><td>WP_079163728.1</td><td>vWA+APATPase+wHTH+TPRs</td><td>Trypco2→ EAD2+VMAP-M0+VMAP-C→ vWA+APATPase+wHTH+TPRs*→ ||← METALLOPROTEASE+TPRs← FXSXX-COOH-like← Trypsin+EAD2</td><td>1266</td><td>Streptomyces parvulus</td><td>GCF_001660045.1</td><td>actinobacteria</td></tr>
<tr><td>WP_079164118.1</td><td>vWA+TPRs</td><td>TCAD15||Trypco1→ Trypsin+HDxxH+VMAP-M0+VMAP-C→ MoxR→ vWA+TPRs*→ FXSXX-COOH→ Radical_SAM→ METALLOPROTEASE+TPRs→ TIR+APATPase+TPRs→</td><td>1095</td><td>Streptomyces leeuwenhoekii</td><td>GCF_001013905.1</td><td>actinobacteria</td></tr>
<tr><td>WP_079164533.1</td><td>vWA+Pkinase+wHTH+TPRs+cNMP_cyclase+Band7+HAD</td><td>Trypco1→ Trypsin+VMAP-M0+VMAP-C→ MoxR→ vWA+Pkinase+wHTH+TPRs+cNMP_cyclase+Band7+HAD*→ ?→ ?→ ?→ ||← ?||Radical_SAM→ Antibiotic_NAT→ TIR+FxsC_Cter→</td><td>1525</td><td>Streptomyces lincolnensis</td><td>GCA_001685355.1</td><td>actinobacteria</td></tr>
<tr><td>WP_079165222.1</td><td>vWA+SFII-helicase+Z1</td><td>AB_hydrolase→ CASPASE+VMAP-M2+VMAP-C→ MoxR→ vWA+SFII-helicase+Z1*→</td><td>1254</td><td>Streptomyces lincolnensis</td><td>-</td><td>actinobacteria</td></tr>
<tr><td>WP_079173956.1</td><td>vWA+APATPase+wHTH+TPRs</td><td>EAD2+VMAP-M0+VMAP-C→ vWA+APATPase+wHTH+TPRs*→ ||← METALLOPROTEASE+TPRs← Trypsin+EAD2← Trypco2||METALLOPROTEASE+TPRs→</td><td>1300</td><td>Streptomyces mirabilis</td><td>GCA_900112965.1</td><td>actinobacteria</td></tr>
<tr><td>WP_079176518.1</td><td>vWA+Pkinase+Pkinase+NaeI</td><td>Trypco2→ Trypsin+EAD2+VMAP-M0+VMAP-C→ MoxR→ vWA+Pkinase+Pkinase+NaeI*→</td><td>1494</td><td>Streptomyces sp. MUSC 14</td><td>GCF_001866675.1</td><td>actinobacteria</td></tr>
<tr><td>WP_079178870.1</td><td>vWA+TPRs</td><td>TCAD15||PSE→ MoxR→ vWA+TPRs*→ FXSXX-COOH→ PSE→ METALLOPROTEASE+TPRs→ TIR+APATPase+TPRs→</td><td>1107</td><td>Streptomyces humi</td><td>-</td><td>actinobacteria</td></tr>
<tr><td>WP_079180720.1</td><td>vWA+Pkinase+wHTH+TPRs+cNMP_cyclase</td><td>Trypco1→ Trypsin+VMAP-M0+VMAP-C→ MoxR→ vWA+Pkinase+wHTH+TPRs+cNMP_cyclase*→</td><td>1177</td><td>Streptomyces sp. CB02058</td><td>-</td><td>actinobacteria</td></tr>
<tr><td>WP_079182682.1</td><td>vWA+Pkinase+NaeI</td><td>Trypco1→ Trypsin+VMAP-M0+VMAP-C→ MoxR→ vWA+Pkinase+NaeI*→</td><td>1103</td><td>Streptomyces sp. TSRI0281</td><td>GCF_001905125.1</td><td>actinobacteria</td></tr>
<tr><td>WP_079185913.1</td><td>vWA+TPRs</td><td>TCAD15||Trypco1→ Trypsin+HDxxH+VMAP-M0+VMAP-C→ MoxR→ vWA+TPRs*→ FXSXX-COOH→ Radical_SAM→ METALLOPROTEASE+TPRs→ TIR+APATPase+TPRs→</td><td>1243</td><td>Streptomyces sp. CB01580</td><td>-</td><td>actinobacteria</td></tr>
<tr><td>WP_079192457.1</td><td>vWA+wHTH+cNMP_cyclase+Band7+HAD</td><td>Trypco1→ Trypsin+VMAP-M0+VMAP-C→ MoxR→ vWA+wHTH+cNMP_cyclase+Band7+HAD*→</td><td>1273</td><td>Streptomyces sp. CB01883</td><td>GCA_001905855.1</td><td>actinobacteria</td></tr>
<tr><td>WP_079192460.1</td><td>vWA+APATPase+wHTH+TPRs</td><td>EAD2+VMAP-M0+VMAP-C→ MoxR→ vWA+APATPase+wHTH+TPRs*→ ||← Na_H_Exchanger||AB_hydrolase→ CASPASE+EAD2→ FXSXX-COOH-like→ METALLOPROTEASE+TPRs→</td><td>1510</td><td>Streptomyces sp. CB01883</td><td>GCF_001905855.1</td><td>actinobacteria</td></tr>
<tr><td>WP_079193740.1</td><td>vWA+TPRs</td><td>TCAD15||Trypco1→ Trypsin+HDxxH+VMAP-M0+VMAP-C→ MoxR→ vWA+TPRs*→ FXSXX-COOH→ Radical_SAM→ TIR+APATPase+TPRs→</td><td>1103</td><td>Streptomyces sp. CB02923</td><td>GCA_001905005.1</td><td>actinobacteria</td></tr>
<tr><td>WP_079195175.1</td><td>vWA+SWACOS+Trypsin+TIR+HTH</td><td>CASPASE+VMAP-M2+VMAP-C→ MoxR→ ||← ?||vWA+SWACOS+Trypsin+TIR+HTH*→</td><td>1121</td><td>Streptomyces sp. CB02400</td><td>GCF_001905725.1</td><td>actinobacteria</td></tr>
<tr><td>WP_079208172.1</td><td>vWA+FGS-fold_domain</td><td>AB_hydrolase→ PSE→ ||← PSE||EAD2+VMAP-M11+VMAP-C→ MoxR→ vWA+FGS-fold_domain*→</td><td>700</td><td>Microcystis aeruginosa</td><td>GCA_002025445.1</td><td>cyanobacteria</td></tr>
<tr><td>WP_079209937.1</td><td>vWA+FGS-fold_domain</td><td>Trypco1→ ||← ?||Trypsin+VMAP-M1+VMAP-C→ MoxR→ vWA+FGS-fold_domain*→</td><td>872</td><td>Microcystis aeruginosa</td><td>GCA_002025445.1</td><td>cyanobacteria</td></tr>
<tr><td>WP_079248443.1</td><td>vWA+TPRs</td><td>TCAD15||Trypsin+HDxxH+VMAP-M0+VMAP-C→ MoxR→ ||← ?||vWA+TPRs*→ FXSXX-COOH→ Radical_SAM→ METALLOPROTEASE+TPRs→ TIR+APATPase+TPRs→</td><td>970</td><td>Streptomyces sp. MJM1172</td><td>-</td><td>actinobacteria</td></tr>
<tr><td>WP_079249393.1</td><td>vWA+APATPase+wHTH+TPRs</td><td>EAD2+VMAP-M0+VMAP-C→ MoxR→ vWA+APATPase+wHTH+TPRs*→ ||← METALLOPROTEASE+TPRs← FXSXX-COOH-like</td><td>1355</td><td>Streptomyces sp. IMTB 2501</td><td>GCF_001953885.1</td><td>actinobacteria</td></tr>
<tr><td>WP_079250460.1</td><td>vWA+TPRs</td><td>TCAD15||Trypsin+HDxxH+VMAP-M0+VMAP-C→ MoxR→ vWA+TPRs*→ FXSXX-COOH→ Radical_SAM→ METALLOPROTEASE+TPRs→ TIR+APATPase+TPRs→</td><td>1100</td><td>Streptomyces amritsarensis</td><td>GCF_001953875.1</td><td>actinobacteria</td></tr>
<tr><td>WP_079251228.1</td><td>vWA+TIR+FxsC_Cter(partial)</td><td>vWA+TIR+FxsC_Cter(partial)*→</td><td>631</td><td>Streptomyces acidiscabies</td><td>-</td><td>actinobacteria</td></tr>
<tr><td>WP_079259908.1</td><td>vWA+APATPase+wHTH+TPRs</td><td>PNP_UDP_1+EAD2→ ?→ EAD2+VMAP-M0+VMAP-C→ MoxR→ vWA+APATPase+wHTH+TPRs*→ ||← METALLOPROTEASE+TPRs← FXSXX-COOH-like</td><td>1453</td><td>Streptomyces autolyticus</td><td>GCA_001983975.1</td><td>actinobacteria</td></tr>
<tr><td>WP_079273265.1</td><td>vWA+TPRs</td><td>TCAD15||Trypsin+HDxxH+VMAP-M0+VMAP-C→ MoxR→ vWA+TPRs*→ FXSXX-COOH→ Radical_SAM→ METALLOPROTEASE+TPRs→ TIR+APATPase+TPRs→</td><td>1093</td><td>Streptomyces sp. TN58</td><td>GCF_001941845.1</td><td>actinobacteria</td></tr>
<tr><td>WP_079277810.1</td><td>vWA+APATPase+wHTH+TPRs</td><td>AB_hydrolase→ EAD2+VMAP-M0+VMAP-C→ MoxR→ vWA+APATPase+wHTH+TPRs*→ ||← METALLOPROTEASE+TPRs← FXSXX-COOH-like← CASPASE+EAD2</td><td>1351</td><td>Streptomyces sp. CB03234</td><td>GCF_001905885.1</td><td>actinobacteria</td></tr>
<tr><td>WP_079309890.1</td><td>vWA_protein</td><td>MoxR→ vWA_protein*→</td><td>501</td><td>Streptomyces sp. GKU 895</td><td>GCA_002027195.1</td><td>actinobacteria</td></tr>
<tr><td>WP_079406085.1</td><td>vWA+TPRs</td><td>TCAD15||Trypsin+HDxxH+VMAP-M0+VMAP-C→ MoxR→ vWA+TPRs*→ FXSXX-COOH→ Radical_SAM→ METALLOPROTEASE+TPRs→ TIR+APATPase+TPRs→</td><td>1103</td><td>Streptomyces sp. 3211</td><td>GCF_002028385.1</td><td>actinobacteria</td></tr>
</table>

<table>
<tr><th>acc</th><th>architecture</th><th>operon</th><th>len</th><th>species</th><th>gca</th><th>taxend</th></tr>
<tr><td>WP_079406523.1</td><td>vWA+Trypsin+cNMP_cyclase+CCCH(TrebleClef)+Pentapeptide_repeats</td><td>AB_hydrolase→ CASPASE+VMAP-M0+VMAP-C→ MoxR→ vWA+Trypsin+cNMP_cyclase+CCCH(TrebleClef)+Pentapeptide_repeats*→</td><td>1196</td><td>Streptomyces sp. 3211</td><td>GCF_002028385.1</td><td>actinobacteria</td></tr>
<tr><td>WP_079429737.1</td><td>vWA+TPRs</td><td>TCAD15||Trypsin+HDxxH+VMAP-M0+VMAP-C→ MoxR→ vWA+TPRs*→ ?→ FXSXX-COOH→ Radical_SAM→ METALLOPROTEASE+TPRs→ TIR+APATPase+TPRs→</td><td>1347</td><td>Streptomyces katrae</td><td>GCF_002028425.1</td><td>actinobacteria</td></tr>
<tr><td>WP_079430380.1</td><td>vWA+Trypsin+cNMP_cyclase+CCCH(TrebleClef)+Pentapeptide_repeats</td><td>AB_hydrolase→ ||← ?||MoxR→ vWA+Trypsin+cNMP_cyclase+CCCH(TrebleClef)+Pentapeptide_repeats*→</td><td>1369</td><td>Streptomyces katrae</td><td>GCA_003814735.1</td><td>actinobacteria</td></tr>
<tr><td>WP_079660314.1</td><td>vWA+Pkinase+ParA-Soj-PloopNTPase(SIMIBI)</td><td>Trypco1→ Trypsin+EAD2+VMAP-M0+VMAP-C→ MoxR→ vWA+Pkinase+ParA-Soj-PloopNTPase(SIMIBI)*→</td><td>1108</td><td>Streptomyces sp. 3124.6</td><td>GCF_900129855.1</td><td>actinobacteria</td></tr>
<tr><td>WP_079662554.1</td><td>vWA+cNMP_cyclase+Band7+HAD</td><td>Trypco1→ Trypsin+VMAP-M0+VMAP-C→ MoxR→ vWA+cNMP_cyclase+Band7+HAD*→</td><td>1118</td><td>Streptomyces sp. 3124.6</td><td>GCA_900129855.1</td><td>actinobacteria</td></tr>
<tr><td>WP_079663892.1</td><td>vWA+SFII-helicase+Z1</td><td>AB_hydrolase→ CASPASE+VMAP-M2+VMAP-C→ MoxR→ vWA+SFII-helicase+Z1*→</td><td>1363</td><td>Streptomyces sp. 3124.6</td><td>-</td><td>actinobacteria</td></tr>
<tr><td>WP_079664780.1</td><td>vWA+TPRs</td><td>TCAD15||Trypco1→ Trypsin+HDxxH+VMAP-M0+VMAP-C→ MoxR→ vWA+TPRs*→ FXSXX-COOH→ Radical_SAM→ METALLOPROTEASE+TPRs→ TIR+APATPase+TPRs→</td><td>1087</td><td>Streptomyces sp. 3124.6</td><td>GCF_900129855.1</td><td>actinobacteria</td></tr>
<tr><td>WP_081104772.1</td><td>vWA+HD+RelA+TGS</td><td>AB_hydrolase→ CASPASE+VMAP-M2+VMAP-C→ MoxR→ vWA+HD+RelA+TGS*→</td><td>1124</td><td>Mycobacterium fortuitum</td><td>GCF_001570465.1</td><td>actinobacteria</td></tr>
<tr><td>WP_081219385.1</td><td>vWA+TPRs</td><td>TCAD15||Trypco1→ Trypsin+HDxxH+VMAP-M0+VMAP-C→ MoxR→ vWA+TPRs*→ FXSXX-COOH→ Radical_SAM→ METALLOPROTEASE+TPRs→ TIR+APATPase+TPRs→</td><td>1101</td><td>Streptomyces sp. M41(2017)</td><td>GCA_002078175.1</td><td>actinobacteria</td></tr>
<tr><td>WP_081281385.1</td><td>vWA+HD+RelA+TGS</td><td>AB_hydrolase→ CASPASE+VMAP-M2+VMAP-C→ MoxR→ vWA+HD+RelA+TGS*→</td><td>973</td><td>Mycobacterium fortuitum</td><td>-</td><td>actinobacteria</td></tr>
<tr><td>WP_081438249.1</td><td>vWA+APATPase+wHTH+TPRs</td><td>EAD2+VMAP-M0+VMAP-C→ MoxR→ vWA+APATPase+wHTH+TPRs*→ TIR+APATPase+TPRs→ ||← METALLOPROTEASE+TPRs</td><td>1496</td><td>Frankia sp. M16386</td><td>GCF_001983105.1</td><td>actinobacteria</td></tr>
<tr><td>WP_081511557.1</td><td>vWA+TPRs</td><td>Trypco1→ Trypsin+HDxxH+VMAP-M0+VMAP-C→ MoxR→ vWA+TPRs*→ Voltage_CLC→ METALLOPROTEASE→ TIR+APATPase+wHTH+TPRs→</td><td>824</td><td>Nocardia donostiensis</td><td>-</td><td>actinobacteria</td></tr>
<tr><td>WP_081522098.1</td><td>vWA+Trypsin+cNMP_cyclase+CCCH(TrebleClef)+Pentapeptide_repeats</td><td>AB_hydrolase→ CASPASE+VMAP-M0+VMAP-C→ MoxR→ vWA+Trypsin+cNMP_cyclase+CCCH(TrebleClef)+Pentapeptide_repeats*→</td><td>1196</td><td>Streptomyces sp. Sge12</td><td>GCA_002080455.1</td><td>actinobacteria</td></tr>
<tr><td>WP_081522474.1</td><td>vWA+TPRs</td><td>TCAD15||Trypsin+HDxxH+VMAP-M0+VMAP-C→ MoxR→ vWA+TPRs*→ FXSXX-COOH→ Radical_SAM→ METALLOPROTEASE+TPRs→ TIR+APATPase+TPRs→</td><td>1132</td><td>Streptomyces sp. Sge12</td><td>GCA_002080455.1</td><td>actinobacteria</td></tr>
<tr><td>WP_081551503.1</td><td>vWA+Pkinase+ParA-Soj-PloopNTPase(SIMIBI)</td><td>DUF397→ Trypco1→ Trypsin+EAD2+VMAP-M0+VMAP-C→ MoxR→ vWA+Pkinase+ParA-Soj-PloopNTPase(SIMIBI)*→</td><td>1116</td><td>Streptomyces sp. B9173</td><td>-</td><td>actinobacteria</td></tr>
<tr><td>WP_081552946.1</td><td>vWA+TPRs</td><td>TCAD15||PSE→ Trypsin+HDxxH+VMAP-M0+VMAP-C→ MoxR→ vWA+TPRs*→ FXSXX-COOH→ Radical_SAM→ METALLOPROTEASE+TPRs→ TIR+APATPase+TPRs→</td><td>1107</td><td>Streptomyces sp. B9173</td><td>-</td><td>actinobacteria</td></tr>
<tr><td>WP_081656248.1</td><td>vWA+FGS-fold_domain</td><td>Trypco1→ Trypsin+EAD9→ EAD9+EAD1+VMAP-C→ MoxR→ vWA+FGS-fold_domain*→ FGS-fold_domain→ four_helix_bundle_protein→ RVT_1→</td><td>972</td><td>Fischerella sp. PCC 9431</td><td>GCF_000447295.1</td><td>cyanobacteria</td></tr>
<tr><td>WP_081692359.1</td><td>vWA+MNS+MNS</td><td>CASPASE+VMAP-M1+VMAP-C→ MoxR→ vWA+MNS+MNS*→</td><td>1255</td><td>Planktothrix agardhii</td><td>GCF_000464825.1</td><td>cyanobacteria</td></tr>
<tr><td>WP_081736412.1</td><td>vWA+APATPase+wHTH+TPRs</td><td>cNMP_cyclase+VMAP-M0+VMAP-C→ MoxR→ vWA+APATPase+wHTH+TPRs*→ ||← METALLOPROTEASE+TPRs← FXSXX-COOH-like← cNMP_binding+HTH_Crp_2</td><td>1473</td><td>Amycolatopsis orientalis</td><td>GCF_000478275.1</td><td>actinobacteria</td></tr>
<tr><td>WP_081748187.1</td><td>vWA+FGS-fold_domain</td><td>EAD1+Trypsin→ ?→ EAD1+VMAP-C→ MoxR→ vWA+FGS-fold_domain*→ four_helix_bundle_protein→ PSE→ ?→ RVT_1→ FGS-fold_domain→</td><td>694</td><td>Leptolyngbya sp. PCC 6406</td><td>GCF_000332095.2</td><td>cyanobacteria</td></tr>
<tr><td>WP_081974155.1</td><td>vWA+CASPASE+TCAD6+cNMP_cyclase+Antibiotic_NAT</td><td>AB_hydrolase→ CASPASE+VMAP-M2+VMAP-C→ MoxR→ vWA+CASPASE+TCAD6+cNMP_cyclase+Antibiotic_NAT*→</td><td>1198</td><td>Kitasatospora phosalacinea</td><td>GCF_000717185.1</td><td>actinobacteria</td></tr>
<tr><td>WP_081976007.1</td><td>vWA+APATPase+wHTH+TPRs</td><td>cNMP_cyclase+VMAP-M0+VMAP-C→ MoxR→ vWA+APATPase+wHTH+TPRs*→ ||← METALLOPROTEASE+TPRs← FXSXX-COOH-like← cNMP_binding+HTH_Crp_2</td><td>1458</td><td>Amycolatopsis sp. MJM2582</td><td>GCA_000754115.1</td><td>actinobacteria</td></tr>
<tr><td>WP_082114514.1</td><td>vWA+APATPase+wHTH+TPRs</td><td>cNMP_binding+HTH_Crp_2||cNMP_cyclase+VMAP-M0+VMAP-C→ MoxR→ vWA+APATPase+wHTH+TPRs*→ ||← METALLOPROTEASE+TPRs← FXSXX-COOH-like</td><td>1424</td><td>Lechevalieria aerocolonigenes</td><td>GCF_000955955.1</td><td>actinobacteria</td></tr>
<tr><td>WP_082122179.1</td><td>vWA+APATPase+wHTH+TPRs</td><td>EAD2+EAD1→ EAD2+VMAP-M0+VMAP-C→ MoxR→ vWA+APATPase+wHTH+TPRs*→ ||← METALLOPROTEASE+TPRs← FXSXX-COOH-like</td><td>1380</td><td>Frankia sp. CpI1-S</td><td>GCA_000948395.1</td><td>actinobacteria</td></tr>
<tr><td>WP_082172447.1</td><td>vWA+FGS-fold_domain</td><td>FGS-fold_domain→ ||← ?← ?||EAD4+VMAP-M3+VMAP-C→ MoxR→ vWA+FGS-fold_domain*→ ?→ FGS-fold_domain→</td><td>600</td><td>Limnoraphis robusta</td><td>GCF_000972705.2</td><td>cyanobacteria</td></tr>
<tr><td>WP_082824563.1</td><td>vWA+TPRs</td><td>VMAP-C_protein→ MoxR→ vWA+TPRs*→</td><td>696</td><td>Arthrospira platensis</td><td>-</td><td>cyanobacteria</td></tr>
<tr><td>WP_082857577.1</td><td>vWA+APATPase+wHTH+TPRs</td><td>cNMP_cyclase+VMAP-M0+VMAP-C→ MoxR→ vWA+APATPase+wHTH+TPRs*→ ||← METALLOPROTEASE+TPRs← FXSXX-COOH-like← cNMP_binding+HTH_Crp_2</td><td>1457</td><td>Amycolatopsis regifaucium</td><td>GCA_900113515.1</td><td>actinobacteria</td></tr>
<tr><td>WP_083103842.1</td><td>vWA</td><td>EAD2+VMAP-M0+VMAP-C→ vWA*→</td><td>490</td><td>Streptomyces gilvosporeus</td><td>GCA_002082195.1</td><td>actinobacteria</td></tr>
<tr><td>WP_083255164.1</td><td>vWA+APATPase+wHTH+TPRs</td><td>cNMP_cyclase+VMAP-M0+VMAP-C→ MoxR→ vWA+APATPase+wHTH+TPRs*→ ||← METALLOPROTEASE+TPRs← FXSXX-COOH-like← cNMP_binding+HTH_Crp_2</td><td>1470</td><td>Amycolatopsis orientalis</td><td>GCF_000943515.2</td><td>actinobacteria</td></tr>
<tr><td>WP_083401471.1</td><td>vWA+APATPase+wHTH+TPRs</td><td>EAD2+VMAP-M0+VMAP-C→ MoxR→ vWA+APATPase+wHTH+TPRs→ ||← METALLOPROTEASE+TPRs||EAD2+VMAP-M0+VMAP-C→ MoxR→ vWA+APATPase+wHTH+TPRs*→ || METALLOPROTEASE+TPRs</td><td>1518</td><td>Frankia sp. EUN1h</td><td>GCF_001854645.1</td><td>actinobacteria</td></tr>
<tr><td>WP_083466592.1</td><td>vWA+APATPase+wHTH+TPRs</td><td>EAD2+VMAP-M0+VMAP-C→ MoxR→ vWA+APATPase+wHTH+TPRs*→ ||← METALLOPROTEASE+TPRs← FXSXX-COOH-like← ?||EAD2+VMAP-M0+VMAP-C→ MoxR→ vWA+APATPase+wHTH+TPRs→ ||← METALLOPROTEASE+TPRs</td><td>1425</td><td>Kibdelosporangium sp. MJ126-NF4</td><td>GCF_000826545.1</td><td>actinobacteria</td></tr>
<tr><td>WP_083507376.1</td><td>vWA+APATPase+wHTH+TPRs</td><td>EAD2+EAD1→ EAD2+VMAP-M0+VMAP-C→ vWA+APATPase+wHTH+TPRs*→ ||← METALLOPROTEASE+TPRs← FXSXX-COOH-like</td><td>1390</td><td>Frankia sp. ACN1ag</td><td>GCF_001414035.1</td><td>actinobacteria</td></tr>
<tr><td>WP_083619629.1</td><td>vWA+MACRODOMAIN+GUN4+DO-GTPase2</td><td>vWA+MACRODOMAIN+GUN4+DO-GTPase2*→</td><td>1224</td><td>Planktothrix serta</td><td>GCA_900010725.1</td><td>cyanobacteria</td></tr>
<tr><td>WP_083650068.1</td><td>vWA+APATPase+wHTH+TPRs</td><td>cNMP_cyclase+VMAP-M0+VMAP-C→ MoxR→ vWA+APATPase+wHTH+TPRs*→ ||← METALLOPROTEASE+TPRs← FXSXX-COOH-like← cNMP_binding+HTH_Crp_2</td><td>1458</td><td>Amycolatopsis sp. CB00013</td><td>GCF_001905755.1</td><td>actinobacteria</td></tr>
<tr><td>WP_084210794.1</td><td>vWA+APATPase+wHTH+TPRs</td><td>cNMP_binding+HTH_Crp_2→ ?→ cNMP_cyclase+VMAP-M0+VMAP-C→ MoxR→ vWA+APATPase+wHTH+TPRs*→ ||← METALLOPROTEASE+TPRs</td><td>1487</td><td>Pseudonocardia acaciae</td><td>GCF_000620785.1</td><td>actinobacteria</td></tr>
<tr><td>WP_084265700.1</td><td>vWA+Pkinase+DO-GTPase2</td><td>Trypsin+VMAP-M8+VMAP-C→ MoxR→ vWA+Pkinase+DO-GTPase2*→</td><td>1099</td><td>Actinomadura macra</td><td>GCF_001552215.1</td><td>actinobacteria</td></tr>
<tr><td>WP_084555188.1</td><td>vWA+FGS-fold_domain</td><td>cNMP_cyclase+VMAP-M1+VMAP-C→ MoxR→ vWA+FGS-fold_domain*→ FGS-fold_domain→</td><td>826</td><td>Phormidium ambiguum</td><td>GCF_001904725.1</td><td>cyanobacteria</td></tr>
<tr><td>WP_084641579.1</td><td>vWA+APATPase+wHTH+TPRs</td><td>cNMP_cyclase+EAD2→ EAD2+VMAP-M0+VMAP-C→ MoxR→ vWA+APATPase+wHTH+TPRs*→ ||← METALLOPROTEASE+TPRs||cNMP_cyclase+EAD2→ EAD2+VMAP-M0+VMAP-C→ MoxR→ TIR+APATPase+TPRs→ ||← METALLOPROTEASE+TPRs</td><td>1486</td><td>Amycolatopsis balhimycina</td><td>GCA_003947255.1</td><td>actinobacteria</td></tr>
<tr><td>WP_084749695.1</td><td>vWA+APATPase+wHTH+TPRs</td><td>cNMP_cyclase+VMAP-M0+VMAP-C→ MoxR→ vWA+APATPase+wHTH+TPRs*→ ||← METALLOPROTEASE+TPRs← FXSXX-COOH-like← cNMP_binding+HTH_Crp_2</td><td>1462</td><td>Amycolatopsis coloradensis</td><td>GCF_001953865.1</td><td>actinobacteria</td></tr>
<tr><td>WP_084763081.1</td><td>vWA+MNS+GUN4+EAD10+BetaPropeller</td><td>EAD10+VMAP-M1+VMAP-C→ MoxR→ vWA+MNS+GUN4+EAD10+BetaPropeller*→</td><td>1529</td><td>[Scytonema hofmanni] UTEX B 1581</td><td>GCF_000582685.1</td><td>cyanobacteria</td></tr>
<tr><td>WP_084896381.1</td><td>vWA+wHTH+TPRs+wHTH+TPRs+APATPase+wHTH+TPRs</td><td>Trypco1→ Trypsin+EAD2+VMAP-M0+VMAP-C→ MoxR→ vWA+wHTH+TPRs+wHTH+TPRs+APATPase+wHTH+TPRs*→</td><td>1788</td><td>Streptomyces sp. CB03238</td><td>GCA_002099185.1</td><td>actinobacteria</td></tr>
<tr><td>WP_084989906.1</td><td>vWA+FGS-fold_domain</td><td>HTH+VMAP-M9+VMAP-C→ MoxR→ vWA+FGS-fold_domain*→ ||← ?← ?← ?← PSE||?→ FGS-fold_domain→</td><td>964</td><td>Microcystis aeruginosa</td><td>GCA_002095975.1</td><td>cyanobacteria</td></tr>
<tr><td>WP_085207283.1</td><td>vWA+REase+cNMP_cyclase+CASPASE</td><td>AB_hydrolase→ CASPASE+EAD2+VMAP-M0+VMAP-C→ MoxR→ vWA+REase+cNMP_cyclase+CASPASE*→</td><td>1172</td><td>Streptomyces sp. Amel2xC10</td><td>GCA_900177255.1</td><td>actinobacteria</td></tr>
<tr><td>WP_085214149.1</td><td>vWA+Pkinase+wHTH+TPRs+cNMP_cyclase+Band7+HAD</td><td>Trypco1→ Trypsin+VMAP-M0+VMAP-C→ MoxR→ vWA+Pkinase+wHTH+TPRs+cNMP_cyclase+Band7+HAD*→</td><td>1501</td><td>Streptomyces sp. Amel2xC10</td><td>GCA_900177255.1</td><td>actinobacteria</td></tr>
<tr><td>WP_085564588.1</td><td>vWA+Pkinase+cNMP_cyclase+Band7+HAD</td><td>Trypco1→ Trypsin+VMAP-M0+VMAP-C→ MoxR→ vWA+Pkinase+cNMP_cyclase+Band7+HAD*→</td><td>1308</td><td>Streptomyces griseofuscus</td><td>GCA_002114065.1</td><td>actinobacteria</td></tr>
</table>

<table>
<tr><th>acc</th><th>architecture</th><th>operon</th><th>len</th><th>species</th><th>gca</th><th>taxend</th></tr>
<tr><td>WP_085923529.1</td><td>vWA+Pkinase+ParA-Soj-PloopNTPase(SIMIBI)</td><td>Trypco1→ Trypsin+EAD2+VMAP-M0+VMAP-C→ MoxR→ vWA+Pkinase+ParA-Soj-PloopNTPase(SIMIBI)*→</td><td>1127</td><td>Streptomyces platensis</td><td>-</td><td>actinobacteria</td></tr>
<tr><td>WP_085983492.1</td><td>vWA+APATPase+wHTH+TPRs</td><td>EAD2+VMAP-M0+VMAP-C→ MoxR→ vWA+APATPase+wHTH+TPRs*→ ||← METALLOPROTEASE+TPRs← cNMP_cyclase+EAD2</td><td>1336</td><td>Saccharothrix espanaensis</td><td>GCF_000328705.1</td><td>actinobacteria</td></tr>
<tr><td>WP_086560182.1</td><td>vWA+APATPase+GGDEF+EAD2+VMAP-M0+VMAP-C</td><td>Trypsin+EAD2+VMAP-M0+VMAP-C→ MoxR→ vWA+APATPase+GGDEF+EAD2+VMAP-M0+VMAP-C*→</td><td>1327</td><td>Streptomyces africanus</td><td>GCF_002150735.1</td><td>actinobacteria</td></tr>
<tr><td>WP_086706666.1</td><td>vWA+TIR+cNMP_cyclase+S1COLD+EAD2</td><td>AB_hydrolase→ CASPASE+EAD2+VMAP-M0+VMAP-C→ MoxR→ vWA+TIR+cNMP_cyclase+S1COLD+EAD2*→</td><td>1102</td><td>Streptomyces castelarensis</td><td>GCF_002154275.1</td><td>actinobacteria</td></tr>
<tr><td>WP_089154134.1</td><td>vWA+Trypsin+Coiled-coil</td><td>Trypco1+Trypsin+VMAP-M0+VMAP-C→ MoxR→ vWA+Trypsin+Coiled-coil*→ Glyco_hydro_16+Ricin_B_lectin→ ||← ?||?→ DUF397→</td><td>1004</td><td>Micromonospora sp. NBS 11-29</td><td>GCF_002140155.1</td><td>actinobacteria</td></tr>
<tr><td>WP_096617002.1</td><td>vWA+REC+CASPASE+FGS-fold_domain</td><td>EAD1+Trypsin→ ?→ EAD1+VMAP-M1+VMAP-C→ ||← ?||VMAP-C(partial)→ MoxR→ vWA+REC+CASPASE+FGS-fold_domain*→</td><td>1263</td><td>Calothrix sp. NIES-4101</td><td>GCA_004296455.1</td><td>cyanobacteria</td></tr>
<tr><td>WP_097965258.1</td><td>vWA+Pkinase+APATPase+TPRs</td><td>Trypsin+EAD2+VMAP-M0+VMAP-C→ MoxR→ vWA+Pkinase+APATPase+TPRs*→</td><td>1310</td><td>Streptomyces sp. or20</td><td>GCF_002551305.1</td><td>actinobacteria</td></tr>
<tr><td>WP_098754349.1</td><td>vWA+Pkinase+Pkinase+TPRs</td><td>vWA+Pkinase+Pkinase+TPRs*→</td><td>1734</td><td>Streptomyces sp. Ru87</td><td>GCA_002573955.1</td><td>actinobacteria</td></tr>
<tr><td>WP_100179620.1</td><td>vWA+REC+REC+GAF+GAF+GAF+Acylphosphatase+CASPASE</td><td>AB_hydrolase→ CASPASE+VMAP-M4+VMAP-C→ MoxR→ vWA+REC+REC+GAF+GAF+GAF+Acylphosphatase+CASPASE*→</td><td>1802</td><td>Bradyrhizobium sp. TSA1</td><td>GCA_002776695.1</td><td>alphaproteobacteria</td></tr>
</table>

iSTAND ternary systems

<table>
<tr><th>acc</th><th>architecture</th><th>operon</th><th>len</th><th>species</th><th>gca</th><th>taxend</th></tr>
<tr><td>ABG51300.1</td><td>iSTAND</td><td>iSTAND*→ MoxR→ TM+TM+vWA+TM+TM+TM+TM+TM+TM→</td><td>378</td><td>Trichodesmium erythraeum IMS101</td><td>GCA_000014265.1</td><td>cyanobacteria</td></tr>
<tr><td>ACC83829.1</td><td>EAD9+iSTAND</td><td>CASPASE+EAD9→ EAD9+iSTAND*→ STAND_NTPase→ MoxR(fragment)→ helical_region(leucine-richLWxKmotif)+vWA→??+REC→ GUN4→</td><td>326</td><td>Nostoc punctiforme PCC 73102</td><td>GCA_000020025.1</td><td>cyanobacteria</td></tr>
<tr><td>ACK70921.1</td><td>HTH+iSTAND</td><td>TM||HTH+iSTAND*→ MoxR→ helical_region(leucine-richLWxKmotif)+vWA→</td><td>416</td><td>Gloeothece citriformis PCC 7424</td><td>GCA_000021825.1</td><td>cyanobacteria</td></tr>
<tr><td>AEE50346.1</td><td>EAD11+iSTAND</td><td>TM+TM+TM+TM+STAND_NTPase+TM+TM+TM+TM→||←STAND_NTPase+REase←TM||HTH→ PIN+DUF3368→ EAD11+iSTAND*→ MoxR→||←vWA←??+beta-sandwich(Ig-like?)</td><td>356</td><td>Haliscomenobacter hydrossis DSM 1100</td><td>GCA_000212735.1</td><td>bacteroidetes</td></tr>
<tr><td>AEE54384.1</td><td>CASPASE+iSTAND</td><td>CASPASE+iSTAND*→ MoxR→ vWA+??+FGS-fold_domain+FGS-fold_domain+FGS-fold_domain+FGS-fold_domain→</td><td>548</td><td>Haliscomenobacter hydrossis DSM 1100</td><td>GCA_000212735.1</td><td>bacteroidetes</td></tr>
<tr><td>AEJ01436.1</td><td>TIR+iSTAND</td><td>TIR+iSTAND*→ MoxR→ TM+TM+vWA+TM+FGS-fold_domain→</td><td>436</td><td>Nitrosomonas sp. Is79A3</td><td>GCA_000219585.1</td><td>betaproteobacteria</td></tr>
<tr><td>AFY52644.1</td><td>Trypsin+iSTAND</td><td>Trypco1→ Trypsin+iSTAND*→ MoxR→ helical_region(leucine-richLWxKmotif)+vWA→ vWA+FGS-fold_domain→</td><td>504</td><td>Rivularia sp. PCC 7116</td><td>GCA_000316665.1</td><td>cyanobacteria</td></tr>
<tr><td>AFZ01471.1</td><td>CASPASE+iSTAND</td><td>CASPASE+iSTAND*→ STAND_NTPase(fragment)→</td><td>527</td><td>Calothrix sp. PCC 6303</td><td>GCA_000317435.1</td><td>cyanobacteria</td></tr>
<tr><td>AFZ27597.1</td><td>Coiled-coil+iSTAND</td><td>CASPASE+EAD9→ Coiled-coil+iSTAND*→</td><td>335</td><td>Cylindrospermum stagnale PCC 7417</td><td>GCA_000317535.1</td><td>cyanobacteria</td></tr>
<tr><td>AOY79837.1</td><td>Coiled-coil+iSTAND</td><td>Coiled-coil+iSTAND*→ MoxR→ helical_region(leucine-richLWxKmotif)+TM+Calx-beta?+TM+vWA→</td><td>342</td><td>Moorea producens JHB</td><td>GCA_001854205.1</td><td>cyanobacteria</td></tr>
<tr><td>ATY32529.1</td><td>Trypsin+iSTAND</td><td>rve_3→ Trypsin+iSTAND*→ MoxR→</td><td>494</td><td>Sphingomonas sp. Cra20</td><td>GCA_002796605.1</td><td>alphaproteobacteria</td></tr>
<tr><td>AUB37286.1</td><td>EAD9+iSTAND</td><td>EAD9+iSTAND*→ MoxR→ helical_region(leucine-richLWxKmotif)+vWA→??+TM→ CASPASE(fragment)→</td><td>328</td><td>Nostoc flagelliforme CCNUN1</td><td>GCA_002813575.1</td><td>cyanobacteria</td></tr>
<tr><td>BAI89278.1</td><td>Coiled-coil+iSTAND</td><td>SLH+OprB→||←TM||RadC-like_JAB+sGTPase→ Coiled-coil+iSTAND*→ MoxR→ helical_region(leucine-richLWxKmotif)+vWA→ TA_antitoxin(DUF1902)→ Ntox45→ HEPN→</td><td>630</td><td>Arthrospira platensis NIES-39</td><td>GCA_000210375.1</td><td>cyanobacteria</td></tr>
<tr><td>BAY12344.1</td><td>EAD9+iSTAND</td><td>EAD9+iSTAND*→ MoxR→ helical_region(leucine-richLWxKmotif)+vWA→ Cation_ATPase→ Calcineurin→</td><td>322</td><td>Calothrix sp. NIES-2098</td><td>GCA_002368175.1</td><td>cyanobacteria</td></tr>
<tr><td>BAY13982.1</td><td>Coiled-coil+iSTAND</td><td>Coiled-coil+iSTAND*→ MoxR→ helical_region(leucine-richLWxKmotif)+TM+Calx-beta?+TM+vWA→ vWA+FGS-fold_domain→</td><td>341</td><td>Calothrix sp. NIES-2098</td><td>GCA_002368175.1</td><td>cyanobacteria</td></tr>
<tr><td>BAY41492.1</td><td>iSTAND</td><td>Pentapeptide_repeats+TM+TM+TM+TM+TM+TM+TM+Pentapeptide_repeats→ Pentapeptide_repeats→ STNAD_NTPase+TM+TM+TM+TM+TM+TM+TM+TM→ iSTAND*→ MoxR→ TM+TM+vWA→||←DDE_endonuclease←PAIREDC-HTH||beta-rich_jelly-roll?→</td><td>359</td><td>Nostoc sp. NIES-2111</td><td>GCA_002368215.1</td><td>cyanobacteria</td></tr>
<tr><td>BAY43400.1</td><td>iSTAND</td><td>iSTAND*→ MoxR→ helical_region(leucine-richLWxKmotif)+vWA→ TM+Pentapeptide_repeats+TM+TM+TM+TM+TM+TM+TM+Pentapeptide_repeats→||←TM+TM+TM+TM+TM</td><td>312</td><td>Scytonema sp. HK-05</td><td>GCA_002368235.1</td><td>cyanobacteria</td></tr>
<tr><td>BAZ19856.1</td><td>EAD8+iSTAND</td><td>EAD8+Trypsin→ EAD8+iSTAND*→ MoxR→ helical_region(leucine-richLWxKmotif)+vWA→ REase→ vWA+GTPase-AIG→</td><td>361</td><td>Scytonema sp. NIES-4073</td><td>GCA_002368435.1</td><td>cyanobacteria</td></tr>
<tr><td>BAZ48964.1</td><td>HTH+iSTAND</td><td>HTH+iSTAND*→ MoxR→ helical_region(leucine-richLWxKmotif)+vWA→</td><td>383</td><td>Nostoc sp. NIES-4103</td><td>GCA_002368335.1</td><td>cyanobacteria</td></tr>
<tr><td>BBD55736.1</td><td>??+HTH+iSTAND</td><td>??+HTH+iSTAND*→ MoxR→ helical_region(leucine-richLWxKmotif)+vWA→</td><td>431</td><td>Planktothrix agardhii NIES-204</td><td>GCA_003609755.1</td><td>cyanobacteria</td></tr>
<tr><td>CAL77009.1</td><td>Trypsin+iSTAND</td><td>Trypco2→ Trypsin+iSTAND*→ MoxR→2TM+vWA+22TM→??+TM→??→</td><td>499</td><td>Bradyrhizobium sp. ORS 278</td><td>GCA_000026145.1</td><td>alphaproteobacteria</td></tr>
<tr><td>CBS89362.1</td><td>Trypsin+iSTAND</td><td>Trypco2→ Trypsin+iSTAND*→ MoxR→2TM+vWA+22TM→</td><td>501</td><td>Azospirillum lipoferum 4B</td><td>GCA_000283655.1</td><td>alphaproteobacteria</td></tr>
<tr><td>CUR12819.1</td><td>Death-like.bact-prob+iSTAND</td><td>Death-like.bact-prob+iSTAND*→ MoxR→ helical_region(leucine-richLWxKmotif)+vWA→ vWA+TPRs+CASPASE→??→</td><td>368</td><td>Planktothrix paucivesiculata PCC 9631</td><td>GCA_900009265.1</td><td>cyanobacteria</td></tr>
<tr><td>CUR34706.1</td><td>HTH+iSTAND</td><td>HTH+iSTAND*→ MoxR→ helical_region(leucine-richLWxKmotif)+vWA→</td><td>342</td><td>Planktothrix tepida PCC 9214</td><td>GCA_900009145.1</td><td>cyanobacteria</td></tr>
<tr><td>CUS33832.1</td><td>TIR+iSTAND</td><td>DUF805(3TM_protein)→??→ DUF805(3TM_protein)→ TM+TM+TM→ TIR+iSTAND*→ MoxR→ TM+TM+vWA+TM+TM→</td><td>463</td><td>Candidatus Nitrospira nitrosa</td><td>GCA_001458735.1</td><td>nitrospirae</td></tr>
<tr><td>EAZ90085.1</td><td>CASPASE+VMAP-M11</td><td>AB_hydrolase→ CASPASE+VMAP-M11*→ MoxR→ helical_region(leucine-richLWxKmotif)+vWA→ vWA+FGS-fold_domain→</td><td>553</td><td>Crocosphaera chwakensis CCY0110</td><td>GCA_000169335.1</td><td>cyanobacteria</td></tr>
<tr><td>EDN69714.1</td><td>iSTAND</td><td>PNP_UDP_1→ PNP_UDP_1(fragment)→ iSTAND*→ HTH→ MoxR→</td><td>199</td><td>Beggiatoa sp. PS</td><td>GCA_000170715.1</td><td>gammaproteobacteria</td></tr>
<tr><td>EDX78652.1</td><td>HTH+iSTAND</td><td>TM||HTH+iSTAND*→ MoxR→</td><td>363</td><td>Coleofasciculus chthonoplastes PCC 7420</td><td>GCA_000155555.1</td><td>cyanobacteria</td></tr>
<tr><td>EKD10616.1</td><td>Coiled-coil+iSTAND</td><td>SLH+OprB→ RadC-like_JAB+sGTPase→ Coiled-coil+iSTAND*→ MoxR→ helical_region(leucine-richLWxKmotif)+vWA→ TA_antitoxin(DUF1902)→ HicA_toxin→ Ntox45→</td><td>622</td><td>Arthrospira platensis C1</td><td>GCA_000307915.1</td><td>cyanobacteria</td></tr>
<tr><td>EKE99441.1</td><td>Coiled-coil+iSTAND</td><td>TM+DUF218→||←??←??||Coiled-coil+iSTAND*→ MoxR→</td><td>327</td><td>Tolypothrix sp. PCC 7601</td><td>GCA_000300115.1</td><td>cyanobacteria</td></tr>
<tr><td>EKF00758.1</td><td>HTH+STAND_NTPase+macroglobulin-like+CASPASE+??</td><td>HTH+STAND_NTPase+macroglobulin-like+CASPASE+??*→ CASPASE→ Pkinase+STAND_NTPase+macroglobulin-like+CASPASE→ CASPASE→ Coiled-coil→</td><td>1058</td><td>Tolypothrix sp. PCC 7601</td><td>GCA_000300115.1</td><td>cyanobacteria</td></tr>
<tr><td>ERN41330.1</td><td>iSTAND</td><td>??→ iSTAND*→ MoxR→ TM+TM+vWA+TM→</td><td>371</td><td>Rubidibacter lacunae KORDI 51-2</td><td>GCA_000473895.1</td><td>cyanobacteria</td></tr>
</table>

acc	architecture	operon	len	species	gca	taxend
ERN41366.1	CASPASE+iSTAND	←Trypco1||CASPASE+iSTAND*→ MoxR→ helical_region(leucine-richLWxKmotif)+vWA→ BetaPropeller+TPRs+FGE-sulfatase→ FGS-fold_domain→	516	Rubidibacter lacunae KORDI 51-2	GCA_000473895.1	cyanobacteria
ETR67316.1	DRHyd+iSTAND	DRHyd+iSTAND*→??→ GspA-ATPase(fragment)→	370	Candidatus Magnetoglobus multicellularis str. Araruama	GCA_000516475.1	deltaproteobacteria
ETR67363.1	DRHyd+iSTAND	DRHyd+iSTAND*→ MoxR→	409	Candidatus Magnetoglobus multicellularis str. Araruama	GCA_000516475.1	deltaproteobacteria
ETR68943.1	iSTAND(fragment)	iSTAND(fragment)*→??→??→	131	Candidatus Magnetoglobus multicellularis str. Araruama	GCA_000516475.1	deltaproteobacteria
ETR70369.1	TIR+iSTAND	TIR+iSTAND*→	434	Candidatus Magnetoglobus multicellularis str. Araruama	GCA_000516475.1	deltaproteobacteria
ETR71372.1	ZnR+ZnR+TIR+iSTAND	STNAD_NTPase+REase7→ ZnR+ZnR+TIR+iSTAND*→ MoxR→	493	Candidatus Magnetoglobus multicellularis str. Araruama	GCA_000516475.1	deltaproteobacteria
EXI79137.1	TIR+iSTAND	TIR+iSTAND*→ MoxR→ TM+TM→ vWA+TIR→ TM+FGS-fold_domain→	471	Candidatus Accumulibacter sp. BA-92	GCA_000585055.1	betaproteobacteria
GAK59821.1	TIR+iSTAND	TIR+iSTAND*→ GspA-ATPase(fragment)→ Ploop_inactive→	455	Candidatus Vecturithrix granuli	GCA_000739535.1	bacteria
GBF81152.1	HTH+iSTAND	HTH+iSTAND*→ MoxR→	411	Aphanothece sacrum FPU1	GCA_003864295.1	cyanobacteria
GCE31429.1	wHTH+TPRs+STAND_NTPase+wHTH+TPRs	wHTH+TPRs+STAND_NTPase+wHTH+TPRs*→	1250	Dictyobacter alpinus	GCA_003967575.1	chloroflexi
KEI68398.1	??+HTH+iSTAND	??+HTH+iSTAND*→ MoxR→ vWA+FGE-sulfatase→	429	Planktothrix agardhii NIVA-CYA 126/8	GCA_000710505.1	cyanobacteria
KFB67381.1	iSTAND	iSTAND*→ MoxR→	283	Candidatus Accumulibacter sp. SK-01	GCA_000584955.2	betaproteobacteria
KHD04797.1	Coiled-coil+iSTAND	Coiled-coil+iSTAND*→	252	Candidatus Thiomargarita nelsonii	-	gammaproteobacteria
KIF23001.1	iSTAND	iSTAND*→ MoxR→??→	255	Aphanocapsa montana BDHKU210001	GCA_000817745.1	cyanobacteria
KIF28928.1	EAD9+iSTAND	EAD9+iSTAND*→ MoxR→??→	330	Hassallia byssoidea VB512170	GCA_000817785.1	cyanobacteria
KIF42870.1	EAD9+iSTAND	TM+TM+TM+TM+TM→ EAD9+iSTAND*→	261	Lyngbya confervoides BDU141951	GCA_000817775.1	cyanobacteria
KIJ79200.1	DRHyd+iSTAND	cyclic_phosphodiesterase→ OppC_N+TM+BPD_transp_1→ DRHyd+iSTAND*→	401	Tolypothrix campylonemoides VB511288	GCA_000828075.2	cyanobacteria
KPA09311.1	TM+TM+iSTAND	TM+TM+iSTAND*→	338	Candidatus Magnetomorum sp. HK-1	GCA_001292585.1	deltaproteobacteria
KPA11637.1	DRHyd+iSTAND	DRHyd+iSTAND*→	345	Candidatus Magnetomorum sp. HK-1	GCA_001292585.1	deltaproteobacteria
KPA12577.1	iSTAND	iSTAND*→ MoxR→??+FGE-sulfatase→	212	Candidatus Magnetomorum sp. HK-1	GCA_001292585.1	deltaproteobacteria
KPA12842.1	TIR+iSTAND	TIR+iSTAND*→ STAND_NTPase→	447	Candidatus Magnetomorum sp. HK-1	GCA_001292585.1	deltaproteobacteria
KPA13794.1	TM+TM+iSTAND	TM+TM+iSTAND*→ MoxR→	338	Candidatus Magnetomorum sp. HK-1	GCA_001292585.1	deltaproteobacteria
KPA15048.1	iSTAND	iSTAND*→ MoxR→||←tRNA_anti-codon+tRNA-synt_2||REC+PAS+NtrC-AAA+FIS-HTH→ REC+cNMP_cyclase→ HAM→	264	Candidatus Magnetomorum sp. HK-1	GCA_001292585.1	deltaproteobacteria
KPA15524.1	iSTAND(fragment)	Nucleoside_deoxyribosyltransferas?→ iSTAND(fragment)*→	160	Candidatus Magnetomorum sp. HK-1	GCA_001292585.1	deltaproteobacteria
KPA17452.1	ZnR+ZnR+TIR+iSTAND	ZnR+ZnR+TIR+iSTAND*→ MoxR→ TM+TM+vWA+TM+FGS-fold_domain→	522	Candidatus Magnetomorum sp. HK-1	GCA_001292585.1	deltaproteobacteria
KPA18269.1	TIR+iSTAND	TIR+iSTAND*→ MoxR→ TM+TM+vWA+TM+PEGA+PEGA+FGS-fold_domain→	466	Candidatus Magnetomorum sp. HK-1	GCA_001292585.1	deltaproteobacteria
KPQ40050.1	Coiled-coil+iSTAND	Coiled-coil+iSTAND*→ MoxR→ helical_region(leucine-richLWxKmotif)+vWA→	570	Phormidium sp. OSCR	GCA_001314905.1	cyanobacteria
KPQ40445.1	CASPASE+iSTAND	←Trypco1||CASPASE+iSTAND*→ MoxR→ helical_region(leucine-richLWxKmotif)+vWA→ vWA+FGS-fold_domain→	524	Phormidium sp. OSCR	GCA_001314905.1	cyanobacteria
KPV52199.1	Coiled-coil+iSTAND	STAND_NTPase→ Coiled-coil+iSTAND*→ STAND_NTPase→	358	Kouleothrix aurantiaca	GCA_001399705.1	chloroflexi
KRB42991.1	Coiled-coil+iSTAND	CASPASE→ Coiled-coil+iSTAND*→ MoxR→ helical_region(leucine-richLWxKmotif)+vWA→ CASPASE+Pkinase→	309	Terrabacter sp. Root181	GCA_001428505.1	actinobacteria

acc	architecture	operon	len	species	gca	taxend
KST64486.1	CASPASE+iSTAND	??+Coiled-coil+TM+TM←??||CASPASE+APATPase+wTHT+TPR→ CASPASE+iSTAND*→ MoxR→ helical_region(leucine-richLWxKmotif)+vWA→	525	Mastigocoleus testarum BC008	GCA_001456025.1	cyanobacteria
KXU90039.1	TIR+iSTAND	DUF805(3TM_protein)→ TM+TM+TM→ TIR+iSTAND*→ MoxR→ TM+TM+vWA+TM+TM→??→??→||←??	457	Paraburkholderia monticola	GCA_001580545.1	betaproteobacteria
KYC36464.1	Coiled-coil+iSTAND	Coiled-coil+iSTAND*→	290	Scytonema hofmannii PCC 7110	GCA_000346485.2	cyanobacteria
KYC38261.1	iSTAND	PNP_UDP_1+EAD9→ iSTAND*→ MoxR→ helical_region(leucine-richLWxKmotif)+vWA+FGS-fold_domain→	304	Scytonema hofmannii PCC 7110	GCA_000346485.2	cyanobacteria
KYC41250.1	EAD9+iSTAND	EAD9+iSTAND*→ MoxR→ helical_region(leucine-richLWxKmotif)+vWA→??+CHASE+TM+TM→	326	Scytonema hofmannii PCC 7110	GCA_000346485.2	cyanobacteria
KYC44054.1	BetaPropeller+iSTAND	BetaPropeller+iSTAND*→	317	Scytonema hofmannii PCC 7110	GCA_000346485.2	cyanobacteria
OCQ92168.1	??+HTH+iSTAND	??→??+HTH+iSTAND*→ MoxR→	433	Oscillatoriales cyanobacterium USR001	GCA_001698425.1	cyanobacteria
OCQ93508.1	Death-like.bact-prob+iSTAND	??+Death-like-bact-prob+Trypsin→??→ Death-like.bact-prob+iSTAND*→ MoxR→ helical_region(leucine-richLWxKmotif)+vWA→	442	Nostoc sp. MBR 210	GCA_001698435.1	cyanobacteria
OCQ97673.1	HTH+iSTAND	HTH+iSTAND*→ MoxR→ helical_region(leucine-richLWxKmotif)+vWA→??+CASPASE→	355	Nostoc sp. MBR 210	GCA_001698435.1	cyanobacteria
OCR02031.1	EAD1+CASPASE+iSTAND	EAD1+CASPASE+iSTAND*→ MoxR→ helical_region(leucine-richLWxKmotif)+vWA→ vWA+EAD1-unk5+CASPASE→	616	Oscillatoriales cyanobacterium USR001	GCA_001698425.1	cyanobacteria
OCR02802.1	HTH+iSTAND	HTH+iSTAND*→ MoxR→	429	Oscillatoriales cyanobacterium USR001	GCA_001698425.1	cyanobacteria
OCR02924.1	CASPASE+iSTAND	CASPASE+iSTAND*→ MoxR→ helical_region(leucine-richLWxKmotif)+vWA→ Ntox45→ HEPN→??→	546	Oscillatoriales cyanobacterium USR001	GCA_001698425.1	cyanobacteria
ODH02967.1	EAD1+VMAP-M1+iSTAND	EAD1+VMAP-M1+iSTAND*→ MoxR→ helical_region(leucine-richLWxKmotif)+vWA→ TM+sGTPase→	471	Nostoc sp. KVJ20	GCA_001712795.1	cyanobacteria
ODN56964.1	HTH+iSTAND	HTH+iSTAND*→ MoxR→ helical_region(leucine-richLWxKmotif)+vWA→ vWA+FGS-fold_domain→	377	Scytonema millei VB511283	GCA_000817735.2	cyanobacteria
OEJ75068.1	iSTAND	iSTAND*→ MoxR→ helical_region(leucine-richLWxKmotif)+vWA+FGS-fold_domain→ Four-helical-protein→ RVT→	333	Desertifilum sp. IPPAS B-1220	GCA_001746915.1	cyanobacteria
OJY86315.1	DRHyd+iSTAND	??||DRHyd+iSTAND*→ MoxR→ TM+TM+beta-sandwich(Ig-like?)+vWA+TM+??+LRRs?→ TM+ATP-synt_A→ TM+TM→ ATP-synt_B→	388	Sphingobacteriales bacterium 40-81	GCA_001899605.1	bacteroidetes
OKH60575.1	??+iSTAND	pJV1-spdB3?→ cNMP_cyclase→??+iSTAND*→ MoxR→ helical_region(leucine-richLWxKmotif)+vWA→ vWA+FGS-fold_domain→	347	Scytonema sp. HK-05	GCA_001904675.1	cyanobacteria
OLT60416.1	Trypsin+iSTAND	Trypsin+iSTAND*→ MoxR→ helical_region(leucine-richLWxKmotif)+vWA→	454	Moorea bouillonii PNG	GCA_001942495.1	cyanobacteria
ONN59423.1	Death-like.bact-prob+iSTAND	Death-like-bact-prob+Trypsin→??→ Death-like.bact-prob+iSTAND*→ MoxR→	369	Mastigocladus laminosus UU774	-	cyanobacteria
OQP57790.1	TIR+HATPase?	TIR+HATPase?*→ MoxR→ TM+beta-sandwich(Ig-like?)+TM+vWA+TM+??→	419	Niastella vici	GCA_002077945.1	bacteroidetes
OQW38836.1	TIR+iSTAND	TIR+iSTAND*→ MoxR→ TM→ TM+vWA+TM+FGS-fold_domain→	421	Proteobacteria bacterium SG_bin4	GCA_002083395.1	proteobacteria
OQX97257.1	iSTAND	TIR→ iSTAND*→ MoxR→ Coiled-coil+??→??+DrHyd+APATPase+wHTH+TPR→ TPRs→	298	Bacteroidetes bacterium 4572_128	GCA_002084355.1	bacteroidetes
OWY22263.1	TIR+iSTAND	TIR+iSTAND*→ MoxR→ TM+beta-sandwich(Ig-like?)+TM+vWA+TM+TM+TPRs+BetaPropeller→ ZnR→||←TM||MazG+MazG→	431	Sphingobacteriales bacterium TSM_CSS	GCA_002212985.1	bacteroidetes
PAX57114.1	EAD9+iSTAND	CASPASE+EAD9→ EAD9+iSTAND*→ MoxR(fragment)→	334	Calothrix elsteri CCALA 953	GCA_002289455.1	cyanobacteria
PHI20256.1	EAD11+iSTAND	EAD11+iSTAND*→ MoxR→ beta-sandwich(Ig-like?)+TM+vWA+TM+OmpA→	394	Lewinellaceae bacterium SD302	GCA_002591745.1	bacteroidetes
PKO80078.1	DRHyd+iSTAND	DRHyd+iSTAND*→ MoxR→ vWA→??+TIR+CASPASE→	450	Betaproteobacteria bacterium HGW-Betaproteobacteria-13	GCA_002842325.1	betaproteobacteria
PNK01636.1	Trypsin+iSTAND	Trypco1→ Trypsin+iSTAND*→ MoxR→	503	Cylindrospermopsis raciborskii S07	GCA_002893205.1	cyanobacteria
PSB54106.1	EAD1+CASPASE+iSTAND	EAD1+CASPASE+iSTAND*→ MoxR→	621	filamentous cyanobacterium Phorm 6	GCA_003003725.1	cyanobacteria
PSM31871.1	Coiled-coil??+iSTAND	Coiled-coil??+iSTAND*→ MoxR→ helical_region(leucine-richLWxKmotif)+vWA→??→ EAD7+EAD7+EAD7+EAD7+VWA→||←??	340	Haliangium sp. UPWRP_2	GCA_002212765.2	deltaproteobacteria
PSN17482.1	iSTAND	iSTAND*→ MoxR→ helical_region(leucine-richLWxKmotif)+vWA→ Coiled-coil→	237	filamentous cyanobacterium CCP5	GCA_003017785.1	cyanobacteria
PSR11591.1	EAD11+iSTAND	EAD11+iSTAND*→ MoxR→ TM+beta-rich_region+TM+vWA→	374	Bacteroidetes bacterium	GCA_003023605.1	bacteroidetes
PZN72517.1	TIR+iSTAND	TIR+iSTAND*→ MoxR→	467	Candidatus Methyloumidiphilus alinensis	GCA_003242955.1	gammaproteobacteria
PZN77012.1	iSTAND	??→ iSTAND*→ MoxR→ TM+TM+vWA+TM+FGE-sulfatase→	325	Candidatus Methyloumidiphilus alinensis	GCA_003242955.1	gammaproteobacteria
PZN82957.1	EAD9+iSTAND	DrHyd+EAD9→ EAD9+iSTAND*→ MoxR→ helical_region(leucine-richLWxKmotif)+vWA→	344	Candidatus Methyloumidiphilus alinensis	GCA_003242955.1	gammaproteobacteria
RAM51670.1	HTH+iSTAND	HTH+iSTAND*→ MoxR→	354	Hapalosiphonaceae cyanobacterium JJU2	GCA_003261315.1	cyanobacteria
RIJ88564.1	Trypsin+iSTAND	Trypco1→ Trypsin+iSTAND*→	335	Candidatus Brocadia sp.	GCA_003577195.1	planctomycetes

<table>
<tr><th>acc</th><th>architecture</th><th>operon</th><th>len</th><th>species</th><th>gca</th><th>taxend</th></tr>
<tr><td>RKW05163.1</td><td>Ploop_NTPase(RecA-like?)</td><td>HTH+DDE_transposase→ STAND_NTPase→ Ploop_NTPase(RecA-like?)*→</td><td>191</td><td>Capnocytophaga sp.</td><td>GCA_003638885.1</td><td>bacteroidetes</td></tr>
<tr><td>RKZ39497.1</td><td>iSTAND</td><td>iSTAND*→</td><td>257</td><td>Gammaproteobacteria bacterium</td><td>GCA_003645185.1</td><td>gammaproteobacteria</td></tr>
<tr><td>RKZ83713.1</td><td>Trypsin+iSTAND</td><td>??→ Trypco1→||←??||Trypsin+iSTAND*→ MoxR→ HTH→</td><td>512</td><td>Gammaproteobacteria bacterium</td><td>GCA_003646175.1</td><td>gammaproteobacteria</td></tr>
<tr><td>RKZ89871.1</td><td>iSTAND</td><td>HTH→||←??||iSTAND*→ DUF4065→??→ MoxR→</td><td>358</td><td>Gammaproteobacteria bacterium</td><td>GCA_003645315.1</td><td>gammaproteobacteria</td></tr>
<tr><td>RKZ91146.1</td><td>TM+TM+iSTAND</td><td>TM+TM+iSTAND*→</td><td>318</td><td>Gammaproteobacteria bacterium</td><td>GCA_003646175.1</td><td>gammaproteobacteria</td></tr>
<tr><td>RKZ93584.1</td><td>CASPASE+iSTAND</td><td>EAD-unk2→ CASPASE+iSTAND*→ HTH→ MoxR→??→??+DUF4351→</td><td>447</td><td>Gammaproteobacteria bacterium</td><td>GCA_003646175.1</td><td>gammaproteobacteria</td></tr>
<tr><td>RMF22456.1</td><td>Coiled-coil+iSTAND(fragment)</td><td>Coiled-coil+iSTAND(fragment)*→</td><td>158</td><td>Bacteroidetes bacterium</td><td>GCA_003695465.1</td><td>bacteroidetes</td></tr>
<tr><td>RMF26839.1</td><td>iSTAND(fragment)</td><td>iSTAND(fragment)*→ MoxR→ TM+beta-sandwich(Ig-like?)→</td><td>239</td><td>Bacteroidetes bacterium</td><td>GCA_003695465.1</td><td>bacteroidetes</td></tr>
<tr><td>RMG26468.1</td><td>CASPASE+iSTAND</td><td>??||CASPASE+iSTAND*→ TM+TM+vWA+??+BetaPropeller→</td><td>491</td><td>Bacteroidetes bacterium</td><td>GCA_003696835.1</td><td>bacteroidetes</td></tr>
<tr><td>RMG30280.1</td><td>CASPASE+iSTAND</td><td>REC+Alkaline_phosphatase-like→ CASPASE+iSTAND*→??→ Radical_SAM→</td><td>495</td><td>Bacteroidetes bacterium</td><td>GCA_003696835.1</td><td>bacteroidetes</td></tr>
<tr><td>RMG84230.1</td><td>EAD11+iSTAND</td><td>EAD11+iSTAND*→ STAND(fragment)→</td><td>354</td><td>Bacteroidetes bacterium</td><td>GCA_003696485.1</td><td>bacteroidetes</td></tr>
<tr><td>SAL85251.1</td><td>Trypsin+iSTAND</td><td>Trypco2→ Trypsin+iSTAND*→ MoxR→ TM+LRRs→</td><td>535</td><td>Caballeronia choica</td><td>GCA_001544535.2</td><td>betaproteobacteria</td></tr>
<tr><td>SEB22698.1</td><td>TIR+iSTAND</td><td>TIR+iSTAND*→ MoxR→ TM+TM+vWA+BetaPropeller+PASTA→</td><td>439</td><td>Variovorax sp. YR216</td><td>GCA_900107745.1</td><td>betaproteobacteria</td></tr>
<tr><td>SEH05005.1</td><td>??+CASPASE+iSTAND</td><td>PseudoU_synth||??→??+CASPASE+iSTAND*→</td><td>718</td><td>Thiotrichales bacterium HS_08</td><td>GCA_900099115.2</td><td>gammaproteobacteria</td></tr>
<tr><td>SEH05860.1</td><td>Trypsin+iSTAND</td><td>Trypsin+iSTAND*→</td><td>341</td><td>Thiotrichales bacterium HS_08</td><td>GCA_900099115.2</td><td>gammaproteobacteria</td></tr>
<tr><td>WP_017306429.1</td><td>??+iSTAND</td><td>??+iSTAND*→ MoxR→ helical_region(leucine-richLWxKmotif)+vWA→ TM→||←Y1_Tnp</td><td>401</td><td>Spirulina subsalsa</td><td>GCF_000314005.1</td><td>cyanobacteria</td></tr>
<tr><td>WP_017318743.1</td><td>EAD9+iSTAND</td><td>EAD9+iSTAND*→ MoxR→ helical_region(leucine-richLWxKmotif)+vWA→</td><td>324</td><td>Mastigocladopsis repens</td><td>GCF_000315565.1</td><td>cyanobacteria</td></tr>
<tr><td>WP_017660035.1</td><td>CASPASE+iSTAND</td><td>NTP_transferase→ BetaPropeller+AB_hydrolase→||←Trypco1||CASPASE+iSTAND*→ MoxR→ helical_region(leucine-richLWxKmotif)+vWA→||←HicB_lk_antitox||vWA+FGS-fold_domain→ FGS-fold_domain→</td><td>520</td><td>Geitlerinema sp. PCC 7105</td><td>GCF_000332355.1</td><td>cyanobacteria</td></tr>
<tr><td>WP_018400346.1</td><td>HTH+iSTAND</td><td>HTH+iSTAND*→ MoxR→ helical_region(leucine-richLWxKmotif)+vWA→</td><td>377</td><td>filamentous cyanobacterium ESFC-1</td><td>GCF_000380225.1</td><td>cyanobacteria</td></tr>
<tr><td>WP_018401004.1</td><td>??+iSTAND</td><td>NfeD||??→??+iSTAND*→ MoxR→</td><td>399</td><td>filamentous cyanobacterium ESFC-1</td><td>GCF_000380225.1</td><td>cyanobacteria</td></tr>
<tr><td>WP_019493547.1</td><td>Coiled-coil+iSTAND</td><td>Coiled-coil+iSTAND*→ MoxR→ helical_region(leucine-richLWxKmotif)+vWA+FGS-fold_domain→</td><td>304</td><td>Calothrix sp. PCC 7103</td><td>GCF_000331305.1</td><td>cyanobacteria</td></tr>
<tr><td>WP_020531248.1</td><td>TIR+iSTAND</td><td>TIR+iSTAND*→ MoxR→</td><td>437</td><td>Flexithrix dorotheae</td><td>GCF_000379765.1</td><td>bacteroidetes</td></tr>
<tr><td>WP_020536784.1</td><td>EAD11+iSTAND</td><td>EAD11+iSTAND*→ MoxR→??+beta-sandwich(Ig-like?)+TM+vWA+TM+FGE-sulfatase→||←TPRs</td><td>363</td><td>Lewinella cohaerens</td><td>GCF_000379805.1</td><td>bacteroidetes</td></tr>
<tr><td>WP_020569053.1</td><td>EAD11+iSTAND</td><td>EAD11+iSTAND*→ MoxR→</td><td>379</td><td>Lewinella persica</td><td>GCF_000373105.1</td><td>bacteroidetes</td></tr>
<tr><td>WP_020569756.1</td><td>Coiled-coil+iSTAND</td><td>Coiled-coil+iSTAND*→ MoxR→ TM+TM+??TM+??+CASPASE→</td><td>361</td><td>Lewinella persica</td><td>GCF_000373105.1</td><td>bacteroidetes</td></tr>
<tr><td>WP_026722524.1</td><td>iSTAND</td><td>TIR→ iSTAND*→ MoxR→ helical_region(leucine-richLWxKmotif)+vWA→</td><td>276</td><td>Fischerella sp. PCC 9431</td><td>GCF_000447295.1</td><td>cyanobacteria</td></tr>
<tr><td>WP_028091549.1</td><td>EAD9+iSTAND</td><td>EAD9+iSTAND*→ MoxR→ TM+TM+??+Amidase→ RVT→</td><td>356</td><td>Dolichospermum circinale</td><td>GCF_000426905.1</td><td>cyanobacteria</td></tr>
<tr><td>WP_028158837.1</td><td>TIR+iSTAND</td><td>TIR+iSTAND*→ MoxR→ TM+TM+vWA+TM+Sel1→??→</td><td>453</td><td>Bradyrhizobium japonicum</td><td>GCF_000472745.1</td><td>alphaproteobacteria</td></tr>
<tr><td>WP_028668053.1</td><td>CASPASE+iSTAND</td><td>CASPASE+iSTAND*→ MoxR→ vWA→ CASPASE+LRRs→ SUI→??→</td><td>647</td><td>Runella zeae</td><td>GCF_000423565.1</td><td>bacteroidetes</td></tr>
<tr><td>WP_038297199.1</td><td>EAD9+iSTAND</td><td>EAD9+iSTAND*→ MoxR→ helical_region(leucine-richLWxKmotif)+vWA→</td><td>327</td><td>[Scytonema hofmanni] UTEX B 1581</td><td>GCF_000582685.1</td><td>cyanobacteria</td></tr>
<tr><td>WP_047155941.1</td><td>Death-like.bact-prob+iSTAND</td><td>-</td><td>369</td><td>Trichodesmium erythraeum</td><td>-</td><td>cyanobacteria</td></tr>
<tr><td>WP_052486108.1</td><td>TIR+iSTAND</td><td>TIR+iSTAND*→ MoxR→</td><td>427</td><td>Scytonema tolypothrichoides</td><td>GCF_000828085.2</td><td>cyanobacteria</td></tr>
<tr><td>WP_061996503.1</td><td>Pkinase+STAND_NT-Pase+wHTH+TPRs+GGDEF</td><td>Pkinase+STAND_NTPase+wHTH+TPRs+GGDEF*→</td><td>1806</td><td>Clostridium sp. ATCC 25772</td><td>GCF_001573875.1</td><td>firmicutes</td></tr>
<tr><td>WP_066426097.1</td><td>??+iSTAND</td><td>??+iSTAND*→ MoxR→ helical_region(leucine-richLWxKmotif)+vWA→</td><td>386</td><td>Anabaena sp. 4-3</td><td>GCF_001597745.1</td><td>cyanobacteria</td></tr>
<tr><td>WP_071187406.1</td><td>EAD9+iSTAND</td><td>Trypco1→ Trypsin+EAD9→ EAD9+iSTAND*→ MoxR→ helical_region(leucine-richLWxKmotif)+vWA→7TMR-HDED+Beta-rich+HTH+FGS-fold_domain→</td><td>339</td><td>Trichormus sp. NMC-1</td><td>GCF_001858025.1</td><td>cyanobacteria</td></tr>
<tr><td>WP_072720293.1</td><td>??+iSTAND</td><td>??+iSTAND*→ MoxR→ vWA→||←Y1_Tnp||HTH+RNase_H+ZnR→ FGS-fold_domain→</td><td>403</td><td>Planktothrix tepida</td><td>-</td><td>cyanobacteria</td></tr>
<tr><td>WP_081755753.1</td><td>DRHyd+iSTAND</td><td>DRHyd+iSTAND*→ MoxR→ vWA→??+TIR+APATPase+TPRs+Pentapeptide_repeats→</td><td>428</td><td>Desulfovibrio magneticus</td><td>GCF_000526455.1</td><td>deltaproteobacteria</td></tr>
<tr><td>WP_081881197.1</td><td>Coiled-coil+Ploop_NTPase</td><td>Coiled-coil+Ploop_NTPase*→ REase→||←SLOG||??→ TM→ DUF262+??+DUF1524(HNH?)→</td><td>644</td><td>Ghiorsea bivora</td><td>GCF_000744415.1</td><td>zetaproteobacteria</td></tr>
<tr><td>WP_081968917.1</td><td>CASPASE+iSTAND</td><td>CASPASE+iSTAND*→ MoxR→ vWA→??+SGNH_hydrolase+CASPASE→</td><td>698</td><td>Phaeodactylibacter xiamenensis</td><td>GCF_000759025.1</td><td>bacteroidetes</td></tr>
<tr><td>WP_084170174.1</td><td>wHTH+STAND_NTPase</td><td>METHYLASE→||←TM+Cupin+AraC-HTH+AraC-HTH||AB_hydrolase→ wHTH+STAND_NTPase*→</td><td>664</td><td>Paraburkholderia ferrariae</td><td>GCF_000685035.1</td><td>betaproteobacteria</td></tr>
<tr><td>WP_088242757.1</td><td>EAD9+iSTAND</td><td>EAD9+iSTAND*→ MoxR→ helical_region(leucine-richLWxKmotif)+vWA→ vWA+FGS-fold_domain→</td><td>327</td><td>Calothrix rhizosoleniae</td><td>GCF_900185595.1</td><td>cyanobacteria</td></tr>
<tr><td>WP_088242861.1</td><td>EAD4+iSTAND</td><td>EAD4+iSTAND*→ MoxR→</td><td>426</td><td>Calothrix rhizosoleniae</td><td>GCF_900185595.1</td><td>cyanobacteria</td></tr>
</table>

acc	architecture	operon	len	species	gca	taxend
WP_088278871.1	TIR+iSTAND	TIR+iSTAND*→ MoxR→ BetaPropeller+FGS-fold_domain→	495	Ideonella sp. A 288	GCF_002198735.1	betaproteobacteria
WP_113510333.1	iSTAND	iSTAND*→ MoxR→ TM+TM+vWA+TM+Sel1→	277	Rhizobiales bacterium	GCF_900469875.1	alphaproteobacteria
WP_124143316.1	??+iSTAND	??+iSTAND*→ MoxR→	395	Okeania hirsuta	GCF_003838225.1	cyanobacteria

FtsH ternary systems

acc	architecture	operon	len	species	gca	taxend
WP_015348943.1	inactive_FtsH+FtsH	Radical_SAM→ X6→ X5→ inactive_FtsH+FtsH*→ X3_analog→ a/b_unk+NEFF1+TPR_region→ MoxR→?→ helical_unk+ivWA→	705	Myxococcus stipitatus	GCF_000331735.1	deltaproteobacteria
PCJ17226.1	inactive_FtsH+FtsH	Radical_SAM→?→ X5→ inactive_FtsH+FtsH*→ X3_analog→??+NEFF1→ PSE→ PSE→ helical_unk+ivWA→	691	Candidatus Cloacimonetes bacterium	GCA_002402105.1	bacteria
WP_013563425.1	inactive_FtsH+FtsH	inactive_FtsH+FtsH*→ X3_analog→??+TPRs+NEFF1→ MoxR→ helical_unk+ivWA→	750	Isosphaera pallida	GCF_000186345.1	planctomycetes
PID39910.1	inactive_FtsH+FtsH	Radical_SAM→ X6→ X5→ inactive_FtsH+FtsH*→ X3_analog→ a/b_unk+NEFF1+TPR_region→ MoxR→ helical_unk+ivWA→	738	Proteobacteria bacterium	GCA_002747355.1	proteobacteria
WP_090492713.1	inactive_FtsH+FtsH	Radical_SAM→ X6→ X5→ inactive_FtsH+FtsH*→ X3_analog→ a/b_unk+NEFF1+TPR_region→ MoxR→ helical_unk+ivWA→	706	Myxococcus virescens	GCF_900101905.1	deltaproteobacteria
WP_095958505.1	inactive_FtsH+FtsH	Radical_SAM→ X6→ X5→ inactive_FtsH+FtsH*→ X3_analog→ a/b_unk+NEFF1+TPR_region→ MoxR→ helical_unk+ivWA→	706	Myxococcus macrosporus	GCF_002305895.1	deltaproteobacteria
WP_140790498.1	inactive_FtsH+FtsH	Radical_SAM→ X6→ X5→ inactive_FtsH+FtsH*→ X3_analog→ a/b_unk+NEFF1+TPR_region→ MoxR→ helical_unk+ivWA→	708	Myxococcus xanthus	GCF_006401215.1	deltaproteobacteria
WP_140798142.1	inactive_FtsH+FtsH	Radical_SAM→ X6→ X5→ inactive_FtsH+FtsH*→ X3_analog→ a/b_unk+NEFF1+TPR_region→ MoxR→ helical_unk+ivWA→	706	Myxococcus xanthus	GCF_006400955.1	deltaproteobacteria
WP_140862181.1	inactive_FtsH+FtsH	Radical_SAM→ X6→ X5→ inactive_FtsH+FtsH*→ X3_analog→ a/b_unk+NEFF1+TPR_region→ MoxR→ helical_unk+ivWA→	706	Myxococcus xanthus	GCF_006401635.1	deltaproteobacteria
WP_141255298.1	inactive_FtsH+FtsH	Radical_SAM→ X6→ X5→ inactive_FtsH+FtsH*→ X3_analog→ a/b_unk+NEFF1+TPR_region→ MoxR→ helical_unk+ivWA→	706	Myxococcus	GCF_006518195.1	deltaproteobacteria
WP_141592591.1	inactive_FtsH+FtsH	Radical_SAM→ X6→ X5→ inactive_FtsH+FtsH*→ X3_analog→ a/b_unk+NEFF1+TPR_region→ MoxR→ helical_unk+ivWA→	706	Myxococcus sp. AB025A	GCF_006547365.1	deltaproteobacteria
WP_141619821.1	inactive_FtsH+FtsH	Radical_SAM→ X6→ X5→ inactive_FtsH+FtsH*→ X3_analog→ a/b_unk+NEFF1+TPR_region→ MoxR→ helical_unk+ivWA→	706	Myxococcus sp. AB024B	GCF_006547355.1	deltaproteobacteria
WP_046713160.1	inactive_FtsH+FtsH	Radical_SAM→ X6→ X5→ inactive_FtsH+FtsH*→ X3_analog→ a/b_unk+NEFF1+TPR_region→ MoxR→||←?||helical_unk+ivWA→	705	Myxococcus fulvus	GCF_000988565.1	deltaproteobacteria
RCK81659.1	inactive_FtsH+FtsH	inactive_FtsH+FtsH*→?→?→ MoxR→ helical_unk+ivWA→ Radical_SAM→ RadSAM_pep→	695	Candidatus Ozemobacter sibiricus	GCA_003327425.1	bacteria
PKL48731.1	inactive_FtsH+FtsH	inactive_FtsH+FtsH*→?→ MoxR→ helical_unk+ivWA→ Radical_SAM→ RadSAM_pep→?→ X5→	698	Candidatus Riflebacteria bacterium HGW-Riflebacteria-2	GCA_002839435.1	bacteria
PCC71843.1	inactive_FtsH+FtsH	Radical_SAM→?→ X5→ inactive_FtsH+FtsH*→ X3_analog→?→ MoxR→ helical_unk+ivWA→	700	Nannocystis exedens	GCA_002343915.1	deltaproteobacteria
WP_096329841.1	inactive_FtsH+FtsH	Radical_SAM→?→ X5→ inactive_FtsH+FtsH*→ X3_analog→?→ MoxR→ helical_unk+ivWA→	704	Nannocystis exedens	GCF_900112715.1	deltaproteobacteria
WP_011552959.1	inactive_FtsH+FtsH	Radical_SAM→ X6→ X5→ inactive_FtsH+FtsH*→ X3_analog→ a/b_unk+NEFF1+TPR_region→ MoxR→?→ helical_unk+ivWA→	706	Myxococcus	GCF_000012685.1	deltaproteobacteria
WP_002635065.1	inactive_FtsH+FtsH	Radical_SAM→ X6→ X5→ inactive_FtsH+FtsH*→ X3_analog→ a/b_unk+NEFF1+TPR_region→ MoxR→ helical_unk+ivWA→	706	Myxococcus hansupus	GCF_000280925.3	deltaproteobacteria
WP_095985479.1	inactive_FtsH+FtsH	Radical_SAM→ X6→ X5→ inactive_FtsH+FtsH*→ X3_analog→ a/b_unk+NEFF1+TPR_region→ MoxR→ helical_unk+ivWA→?→?→||←Radical_SAM	705	Cystobacter fuscus	GCF_002305875.1	deltaproteobacteria
WP_002627974.1	inactive_FtsH+FtsH	Radical_SAM→ X6→?→ X5→ inactive_FtsH+FtsH*→ X3_analog→ a/b_unk+NEFF1+TPR_region→ MoxR→ helical_unk+ivWA→?→?→||←Radical_SAM	705	Cystobacter fuscus	GCF_000335475.2	deltaproteobacteria
WP_013939476.1	inactive_FtsH+FtsH	X6→ X5→ inactive_FtsH+FtsH*→ X3_analog→ a/b_unk+NEFF1+TPR_region→ MoxR→ helical_unk+ivWA→?→?→||←Radical_SAM	706	Myxococcus macrosporus	GCF_000219105.1	deltaproteobacteria
WP_071896885.1	inactive_FtsH+FtsH	Radical_SAM→ X6→ X5→ inactive_FtsH+FtsH*→ X3_analog→ a/b_unk+NEFF1+TPR_region→ MoxR→ helical_unk+ivWA→?→?→||←Radical_SAM	705	Cystobacter ferrugineus	GCF_001887355.1	deltaproteobacteria
PKL44251.1	inactive_FtsH+FtsH	inactive_FtsH+FtsH*→?→ MoxR→ helical_unk+ivWA→ Radical_SAM→ RadSAM_pep→?→ X5→	699	Candidatus Riflebacteria bacterium HGW-Riflebacteria-1	GCA_002839445.1	bacteria
WP_074957489.1	inactive_FtsH+FtsH	Radical_SAM→ X6→ X5→ inactive_FtsH+FtsH*→ X3_analog→ a/b_unk+NEFF1+TPR_region→ MoxR→||←?||helical_unk+vWA→	705	Myxococcus fulvus	GCF_900111765.1	deltaproteobacteria
MBM74108.1	inactive_FtsH+FtsH	Radical_SAM→?→ X5→ inactive_FtsH+FtsH*→ X3_analog→?→ MoxR→??+ivWA→||←?||STAND_NTPase→	715	Proteobacteria bacterium	GCA_002722755.1	proteobacteria
MBK05551.1	inactive_FtsH+FtsH	Radical_SAM→?→ X5→ inactive_FtsH+FtsH*→ X3_analog→?→ MoxR←?||helical_unk+vWA→	737	Deltaproteobacteria bacterium	GCA_002720785.1	deltaproteobacteria
WP_129628747.1	inactive_FtsH+FtsH	TPRs→?→?→??+vWA+FGS-fold_domain→ inactive_FtsH+FtsH*→ REase→ MoxR	600	Oscillochloris sp. Chuk17	GCF_004138135.1	chloroflexi
WP_006562442.1	inactive_FtsH+FtsH	TPRs→?→?→??+vWA+FGS-fold_domain→ inactive_FtsH+FtsH*→ MoxR→?→?→ STAND_NTPase→	598	Oscillochloris trichoides	GCF_000152145.1	chloroflexi
WP_141322754.1	inactive_FtsH+FtsH	Radical_SAM→ X6→ X5→ inactive_FtsH+FtsH*→ X3_analog→ a/b_unk+NEFF1+TPR_region→ MoxR→||←?||helical_unk+ivWA→	705	Myxococcus sp. AB022	GCF_006518215.1	deltaproteobacteria
WP_020473751.1	inactive_FtsH+FtsH	Radical_SAM→?→ Radical_SAM→ X6→ X5→ inactive_FtsH+FtsH*→ X3_analog→?→ a/b_unk+NEFF+TPR_region→	714	Zavarzinella formosa	GCF_000255705.1	planctomycetes
WP_082839483.1	inactive_FtsH+FtsH	Radical_SAM→ X6→ X5→ inactive_FtsH+FtsH*→ X3_analog→ a/b_unk+NEFF1+TPR_region→ a/b_unk+NEFF1+TPR_region→	740	Gemmata sp. SH-PL17	GCF_001610855.1	planctomycetes
VFJ42630.1	inactive_FtsH+FtsH	Radical_SAM.Ammememo→ TIR→ TIR+Pkinase+inactive_Ploop_NTPase+Ploop_NTPase→ Ploop_NTPase→ small_peptide→??→ inactive_FtsH+FtsH*→??→ MoxR→??+ivWA	582	Candidatus Kentron sp. FM	-	gammaproteobacteria
WP_051865898.1	inactive_FtsH+FtsH	EAD1+CASPASE→ RING(inactive)+RING→ E1+UFD→ EAD1+Ubl-Nterm+Ubl+E2→ JAB-N+JAB→ X1→ inactive_FtsH+FtsH*→ MoxR→ X2→ X3→ TPRs→ low_complexity+a/b_unk+TPR_repeat←BetaPropeller+vWA←RadSAM_pep←RADICAL_SAM.Ammemo	615	Streptomyces griseus	GCF_000720255.1	actinobacteria
WP_015197961.1	inactive_FtsH+FtsH	E1+UFD→ EAD8+Trypsin→ TPRs+FGS-fold_domain→ EAD8+EAD1→ EAD1+Ubl-Nterm+Ubl+E2→ JAB-N+JAB→ X1→ inactive_FtsH+FtsH*→ TPRs+C-type_lectin→ MoxR→?→ X3→ TPRs+C-type_lectin→ TPRs+C-type_lectin→?→ Clp_N_repeats+vWA←RADICAL_SAM.Ammemo	663	Calothrix parietina	GCF_000317435.1	cyanobacteria
WP_079310654.1	inactive_FtsH+FtsH	E1+UFD→ E1+UFD→ EAD1+Ubl-Nterm+Ubl+E2→ JAB_N+JAB→ X1→ inactive_FtsH+FtsH*→ MoxR→ X2→ X3→ PSE→?→ beta_propeller(frag?)	624	Streptomyces sp. GKU 895	GCF_002027195.1	actinobacteria
WP_081970790.1	inactive_FtsH+FtsH	E1+UFD→ EAD1+Ubl-Nterm+Ubl+E2→ JAB_N+JAB→ X1→ inactive_FtsH+FtsH*→ MoxR→ X2→ X3→ TPRs→ low_complexity+a/b_unk+TPR_repeat→ BetaPropeller+vWA→||←RadSAM_pep←RADICAL_SAM.Ammemo	561	Dactylosporangium aurantiacum	GCF_000716715.1	actinobacteria
WP_093170141.1	inactive_FtsH+FtsH	E1+UFD→ EAD1+Ubl-Nterm+Ubl+E2→ JAB_N+JAB→ X1→ inactive_FtsH+FtsH*→ MoxR→ X2→ X3→ TPRs→ low_complexity+a/b_unk+TPR_repeat→ BetaPropeller+vWA→||←RadSAM_pep	616	Sinosporangium album	GCF_900100605.1	actinobacteria

acc	architecture	operon	len	species	gca	taxend
WP_052745217.1	inactive_FtsH+FtsH	←DO-GTPase2←Pkinase+TM||EAD1+CASPASE→ RING(inactive)+RING→ E1+UFD→ EAD1+Ubl-Nterm+Ubl+E2→ JAB_N+JAB*→ X1→ inactive_FtsH+FtsH→ MoxR→??→ X2→ X3→ TPRs→ low_complexity+a/b_unk+TPR_repeat→ BetaPropeller+vWA→||←RadSAM_pep←RADICAL_SAM.Ammemo←MPTase←RadSAM.X1	639	Streptomyces sp. WM6386	GCF_000972385.1	actinobacteria
WP_052067792.1	inactive_FtsH+FtsH	←TM+??←TM+coiled-coil←GAP1-N2+GAP1-M+??←DO-GTPase2←Pkinase+TM||EAD1+CASPASE→ RING(inactive)+RING→ E1+UFD→ EAD1+Ubl-Nterm+Ubl+E2→ JAB_N+JAB→ X1→ inactive_FtsH+FtsH*→ MoxR→ X2→ X3→ TPRs→ low_complexity+a/b_unk+TPR_repeat→ BetaPropeller+vWA→||←RadSAM_pep←RADICAL_SAM.Ammemo←MPTase	628	Streptomyces mirabilis	GCF_000746395.1	actinobacteria
WP_067128135.1	inactive_FtsH+FtsH	EAD1+CASPASE→ RING(inactive)+RING→ E1+UFD→ EAD1+Ubl-Nterm+Ubl+E2→ JAB_N+JAB→ X1→ inactive_FtsH+FtsH*→ MoxR→ X2→ X3→ TPRs→?→ BetaPropeller+vWA←RadSAM_pep←RADICAL_SAM.Ammemo←MPTase	637	Streptomyces yokosukanensis	GCF_001514035.1	actinobacteria
TCO52884.1	inactive_FtsH+FtsH	←TM+??←TM+coiled-coil←GAP1-N2+GAP1-M+??←DO-GTPase2←Pkinase+TM||EAD1+CASPASE→ RING(inactive)+RING→ E1+UFD→ EAD1+Ubl-Nterm+Ubl+E2→ JAB_N+JAB→ X1→ inactive_FtsH+FtsH*→ MoxR→ X2→ X3→ TPRs→ low_complexity+a/b_unk+TPR_repeat→ BetaPropeller+vWA→||←RadSAM_pep←RADICAL_SAM.Ammemo	611	Actinocrispum wychmicini	GCA_004345645.1	actinobacteria
WP_020645963.1	inactive_FtsH+FtsH	EAD1+CASPASE→ RING(inactive)+RING→ E1+UFD→ EAD1+Ubl-Nterm+Ubl+E2→ JAB_N+JAB→ X1→ inactive_FtsH+FtsH*→ MoxR→ X2→ X3→ TPRs→ low_complexity+a/b_unk+TPR_repeat→ BetaPropeller+vWA→||←RadSAM_pep←RADICAL_SAM.Ammemo←TM+TM	599	Amycolatopsis balhimycina	GCF_000384295.1	actinobacteria
WP_093947432.1	inactive_FtsH+FtsH	EAD1+CASPASE→ RING(inactive)+RING→ E1+UFD→ EAD1+Ubl-Nterm+Ubl+E2→ JAB_N+JAB→ X1→ inactive_FtsH+FtsH*→ MoxR→ X2→ X3→ TPRs→ low_complexity+a/b_unk+TPR_repeat→ BetaPropeller+vWA→||←RadSAM_pep←RADICAL_SAM.Ammemo←TM+TM	599	Amycolatopsis sp. H5	GCF_002234595.1	actinobacteria
WP_126270580.1	inactive_FtsH+FtsH	EAD1+CASPASE→ RING(inactive)+RING→ E1+UFD→ EAD1+Ubl-Nterm+Ubl+E2→ JAB_N+JAB→ X1→ inactive_FtsH+FtsH*→ MoxR→ X2→ X3→ TPRs→ low_complexity+a/b_unk+TPR_repeat→ BetaPropeller+vWA→||←RadSAM_pep←RADICAL_SAM.Ammemo	624	Streptomyces sp. GGCR-6	GCF_003955715.1	actinobacteria
WP_132124194.1	inactive_FtsH+FtsH	←TM+??←TM+coiled-coil←GAP1-N2+GAP1-M+??←DO-GTPase2←Pkinase+TM||EAD1+CASPASE→ RING(inactive)+RING→ E1+UFD→ EAD1+Ubl-Nterm+Ubl+E2→ JAB_N+JAB→ X1→ inactive_FtsH+FtsH*→ MoxR→ X2→ X3→ TPRs→ low_complexity+a/b_unk+TPR_repeat→ BetaPropeller+vWA→||←RadSAM_pep←RADICAL_SAM.Ammemo	587	Actinocrispum wychmicini	GCF_004345645.1	actinobacteria
WP_132804228.1	inactive_FtsH+FtsH	EAD1+CASPASE→ RING(inactive)+RING→ E1+UFD→ EAD1+Ubl-Nterm+Ubl+E2→ JAB_N+JAB→ X1→ inactive_FtsH+FtsH*→ MoxR→ X2→ X3→ TPRs→ low_complexity+a/b_unk+TPR_repeat→ BetaPropeller+vWA→||←RadSAM_pep←RADICAL_SAM.Ammemo	624	Streptomyces sp. BK042	GCF_004343295.1	actinobacteria
WP_136970956.1	inactive_FtsH+FtsH	←RadSAM_pep←RADICAL_SAM.Ammemo←BetaPropeller+vWA||Prok_Ring(degenerate)+Prok_Ring→ E1+UFD→ EAD1+Ubl-Nterm+Ubl+E2→ JAB-N+JAB→ X1→ inactive_FtsH+FtsH*→ MoxR→	662	Polyangium sorediatum	-	deltaproteobacteria
WP_092557310.1	inactive_FtsH+FtsH	Trypsin+EAD1→ BetaPropeller+vWA→ RADICAL_SAM.Ammemo→ RadSAM_pep→ RING(inactive)+RING→ E1+UFD→ EAD1+Ubl-Nterm+Ubl+E2→ JAB_N+JAB→ X1→ inactive_FtsH+FtsH*→ MoxR→ X2→ X3→ TPRs→ low_complexity+a/b_unk+TPR_repeat→ CASPASE+Ploop_NTPase→ BetaPropeller→ ABhydrolase+TIR→ EAD1+TIR→ BetaPropeller+vWA→	660	Actinoplanes derwentensis	GCF_900104725.1	actinobacteria
WP_051927025.1	inactive_FtsH+FtsH	EAD1+CASPASE→ RING(inactive)+RING→ E1+UFD→ EAD1+Ubl-Nterm+Ubl+E2→ JAB_N+JAB→ X1→ inactive_FtsH+FtsH*→ MoxR→ X2→ X3→ TPRs→ low_complexity+a/b_unk+TPR_repeat→ BetaPropeller+vWA→||←RadSAM_pep←RADICAL_SAM.Ammemo←MPTase←RadSAM.X1	636	Streptomyces durhamensis	GCF_000725475.1	actinobacteria
WP_053662245.1	inactive_FtsH+FtsH	EAD1+CASPASE→ RING(inactive)+RING→ E1+UFD→ EAD1+Ubl-Nterm+Ubl+E2→ JAB_N+JAB→ X1→ inactive_FtsH+FtsH*→ MoxR→ X2→ X3→ TPRs→ low_complexity+a/b_unk+TPR_repeat→ BetaPropeller+vWA→||←RadSAM_pep←RADICAL_SAM.Ammemo←MPTase←RadSAM.X1	639	Streptomyces sp. MMG1121	GCF_001280015.1	actinobacteria
WP_071384075.1	inactive_FtsH+FtsH	EAD1+CASPASE→ RING(inactive)+RING→ E1+UFD→ EAD1+Ubl-Nterm+Ubl+E2→ JAB_N+JAB→ X1→ inactive_FtsH+FtsH*→ MoxR→ X2→ X3→ TPRs→ low_complexity+a/b_unk+TPR_repeat→ BetaPropeller+vWA→||←RadSAM_pep←RADICAL_SAM.Ammemo←MPTase←RadSAM.X1	634	Streptomyces sp. MUSC 1	GCF_001866665.1	actinobacteria
WP_069777255.1	inactive_FtsH+FtsH	←TM+??←TM+coiled-coil←GAP1-N2+GAP1-M+??←DO-GTPase2←Pkinase+TM||EAD1+CASPASE→ RING(inactive)+RING→ E1+UFD→ EAD1+Ubl-Nterm+Ubl+E2→ JAB_N+JAB→ X1→ inactive_FtsH+FtsH*→ MoxR→ X2→ X3→ TPRs→ low_complexity+a/b_unk+TPR_repeat→||←BetaPropeller+vWA←RadSAM_pep←RADICAL_SAM.Ammemo←MPTase	638	Streptomyces puniciscabiei	GCF_001735805.1	actinobacteria
WP_051115179.1	inactive_FtsH+FtsH	Clp_N_repeats+vWA→ Clp_N_repeats+vWA→ Clp_N_repeats+vWA→ Clp_N_repeats+vWA→ Clp_N_repeats+vWA→ Clp_N_repeats+vWA→??→??+4TM→ EAD1+CASPASE→ RADICAL_SAM.Ammemo→ RadSAM_pep→ RING(inactive)+RING→ E1+UFD→ EAD1+Ubl-Nterm+Ubl+E2→ JAB_N+JAB→ X1→ inactive_FtsH+FtsH*→ MoxR→ X2→?→ TPRs→ low_complexity+a/b_unk+TPRs→	689	Actinoplanes globisporus	GCF_000379645.1	actinobacteria
WP_133930094.1	inactive_FtsH+FtsH	EAD1+CASPASE→ RING(inactive)+RING→ E1+UFD→ EAD1+Ubl-Nterm+Ubl+E2→ JAB_N+JAB→ X1→ inactive_FtsH+FtsH*→ MoxR→ X2→ X3→ TPRs→ low_complexity+a/b_unk+TPRs→||←BetaPropeller+vWA←RadSAM_pep←RADICAL_SAM.Ammemo	615	Streptomyces sp. BK161	GCF_004364215.1	actinobacteria
TQS46759.1	inactive_FtsH+FtsH	BetaPropeller+vWA→ BetaPropeller+vWA→ BetaPropeller+vWA→||←TIR+EAD1||BetaPropeller+vWA→ RADICAL_SAM.Ammemo→ RadSAM_pep→ RING(inactive)+RING→ E1+UFD→ EAD1+Ubl-Nterm+Ubl+E2→ JAB_N+JAB→ X1→ inactive_FtsH+FtsH*→ MoxR→ X2→ X3→ TPRs→ low_complexity+a/b_unk+TPRs→	716	Cryptosporangium sp. A-T 5661	GCA_006912135.1	actinobacteria
WP_071371602.1	inactive_FtsH+FtsH	←TM+??←TM+coiled-coil←GAP1-N2+GAP1-M+??←DO-GTPase2←Pkinase+TM||EAD1+CASPASE→ CASPASE(frag)→ CASPASE(frag)→ CASPASE(frag)→ RING(inactive)+RING→ E1+UFD→ EAD1+Ubl-Nterm+Ubl+E2→ JAB_N+JAB→ X1→ inactive_FtsH+FtsH*→ MoxR→ X2→ X3→ TPRs→ low_complexity+a/b_unk+TPR_repeat→||←BetaPropeller+vWA←RadSAM_pep←RADICAL_SAM.Ammemo←MPTase	634	Streptomyces sp. MUSC 14	-	actinobacteria
QBG49801.1	inactive_FtsH+FtsH	E1+UFD→ EAD1+Ubl-Nterm+Ubl+E2→ JAB_N+JAB→ X1→ inactive_FtsH+FtsH*→ MoxR→ TPRs→?→ EAD1+CASPASE→	758	Streptomyces spiramyceticus	-	actinobacteria
WP_136931981.1	inactive_FtsH+FtsH	←RadSAM_pep←Radical_SAM←BetaPropeller+vWA||Prok_Ring(degenerate)+Prok_Ring→ E1+UFD→ EAD1+Ubl-Nterm+Ubl+E2→ JAB-N+JAB→ X1→ inactive_FtsH+FtsH*→ MoxR→?→ TPRs→ low_complexity+a/b_unk+TPRs→	662	Polyangium fumosum	GCF_005144585.1	deltaproteobacteria
WP_130402109.1	inactive_FtsH+FtsH	Clp_N_repeats+vWA→??→??+4TM→ EAD1+CASPASE→ RING(inactive)+RING→ E1+UFD→ EAD1+Ubl-Nterm+Ubl+E2→ JAB_N+JAB→ X1→ inactive_FtsH+FtsH*→ MoxR→ TPRs→ low_complexity+a/b_unk+TPR_repeat→	681	Micromonospora violae	GCF_004217135.1	actinobacteria
KPA11743.1	inactive_FtsH+FtsH	TIR+??→||←?||Ubl+E2→ JAB-N+JAB→ inactive_FtsH+FtsH*→ E1+UFD→ TPRs+FGS-fold_domain→	743	Candidatus Magnetomorum sp. HK-1	GCA_001292585.1	deltaproteobacteria
WP_086686847.1	inactive_FtsH+FtsH	RadSAM_pep→ RING(inactive)+RING→ E1+UFD→ Trypco2→ TCAD4+CASPASE+EAD1→ EAD1+Ubl-Nterm+Ubl+E2→ JAB_N+JAB→ X1→ inactive_FtsH+FtsH*→ TPRs+FGS-fold_domain→ MoxR→?→?→ FGS-fold_domain→||←?||?→||←BetaPropeller+vWA||RADICAL_SAM.Ammemo→	660	Nostoc sp. T09	GCF_002154695.1	cyanobacteria
WP_109500344.1	inactive_FtsH+FtsH	EAD1+CASPASE→ RING(inactive)+RING→ E1+UFD→ EAD1+Ubl-Nterm+Ubl+E2→ JAB_N+JAB→ X1→ inactive_FtsH+FtsH*→ MoxR→ X2→ X3→ TPRs→ low_complexity+a/b_unk+TPR_repeat→||←BetaPropeller+vWA←RadSAM_pep←RADICAL_SAM.Ammemo	626	Streptomyces sp. Act143	GCF_003143935.1	actinobacteria
ETR70572.1	inactive_FtsH+FtsH	inactive_FtsH+FtsH*→ inactive_FtsH+FtsH→ Prok_Ring+Prok_Ring→ E1+UFD→ TIR+??→ Ubl+E2→ JAB_N2→ JAB→	235	Candidatus Magnetoglobus multicellularis str. Araruama	GCA_000516475.1	deltaproteobacteria
SDT45767.1	inactive_FtsH+FtsH	Trypsin+EAD1→ BetaPropeller+vWA→ RADICAL_SAM.Ammemo→ RadSAM_pep→ RING(inactive)+RING→ E1+UFD→ EAD1+Ubl-Nterm+Ubl+E2→ JAB_N+JAB→ X1→ inactive_FtsH+FtsH*→ MoxR→ X2→ X3→ TPRs→ low_complexity+a/b_unk+TPR_repeat→ CASPASE+Ploop_NTPase→ beta_propeller→ ABhydrolase+TIR→ EAD1+TIR→ BetaPropeller+vWA→	668	Actinoplanes derwentensis	GCA_900104725.1	actinobacteria
RMG09056.1	inactive_FtsH+FtsH	MPTase→||←Prok-RING+TM←?←Prok-RING+TM+TM←Band7←Band7||inactive_FtsH+FtsH*→ X3_analog→	793	Planctomycetes bacterium	GCA_003695705.1	planctomycetes
WP_083304944.1	inactive_FtsH+FtsH	RadSAM_pep→ Prok_Ring(degenerate)+Prok_Ring→ X1→ TPRs→ inactive_FtsH+FtsH*→ MoxR→?→?→ TPRs+FGS-fold_domain→→||←?||?→?→||←RADICAL_SAM.Ammemo	622	Moorea producens	GCF_001767235.1	cyanobacteria
WP_009740768.1	inactive_FtsH+FtsH	E1+UFD→ EAD1+Ubl-Nterm+Ubl+E2→ JAB_N+JAB→ X1→ X1→ inactive_FtsH+FtsH*→ MoxR→ Clathrin+DnaJ_CXXCXGXG+DnaJ_C→ low_complexity+a/b_unk+TPR_repeat→||←?←TIR	712	Frankia sp. QA3	GCF_000262465.1	actinobacteria
WP_097261157.1	inactive_FtsH+FtsH	RING(inactive)+RING→ E1+UFD→ EAD1+Ubl-Nterm+Ubl+E2→||←PSE→ X1→ inactive_FtsH+FtsH*→ MoxR→?→?→ TPRs→ low_complexity+a/b_unk+TPR_repeat→ BetaPropeller+vWA→||←RadSAM_pep←RADICAL_SAM.Ammemo	622	Streptomyces sp. TLI_55	GCF_900206255.1	actinobacteria
WP_058554410.1	inactive_FtsH+FtsH	RING(inactive)+RING→ E1+UFD→ helical_X+TIR→ EAD1+Ubl-Nterm+Ubl+E2→||←PSE||?→ inactive_FtsH+FtsH*→ MoxR→ BetaPropeller+vWA→ X2→ X3?→ TPRs→ low_complexity+a/b_unk+TPR_repeat→||←?||BetaPropeller+vWA→ HTH→ RADICAL_SAM.Ammemo→ RadSAM_pep→	677	Thiohalocapsa sp. ML1	GCF_001469165.1	gammaproteobacteria
WP_131767338.1	inactive_FtsH+FtsH	RadSAM_pep→ RING(inactive)+RING→ E1+?→ TIR→||←?←?←?||X1→ inactive_FtsH+FtsH*→ MoxR→	461	Frankia symbiont of Datisca glomerata	GCF_900634715.1	actinobacteria
WP_012825462.1	inactive_FtsH+FtsH	beta_propeller+vWA→ X1→ inactive_FtsH+FtsH*→ MoxR→ X2→ X3→?→ Radical_SAM→ RadSAM_pep→	691	Haliangium ochraceum	GCF_000024805.1	deltaproteobacteria
WP_047223339.1	inactive_FtsH+FtsH	X1→ inactive_FtsH+FtsH*→ MoxR→ X2→	749	Frankia coriariae	GCF_001017755.1	actinobacteria
OGK06825.1	inactive_FtsH+FtsH	inactive_FtsH+FtsH*→?→ MoxR→	699	Candidatus Riflebacteria bacterium GWC2_50_8	GCA_001787855.1	bacteria
WP_131796573.1	inactive_FtsH+FtsH	inactive_FtsH+FtsH*→ MoxR→	452	Frankia symbiont of Datisca glomerata	GCF_900635405.1	actinobacteria

<table>
<tr><th>acc</th><th>architecture</th><th>operon</th><th>len</th><th>species</th><th>gca</th><th>taxend</th></tr>
<tr><td>RKZ47050.1</td><td>inactive_FtsH+FtsH</td><td>inactive_FtsH+FtsH*→?→</td><td>662</td><td>Gammaproteobacteria bacterium</td><td>GCA_003645185.1</td><td>gammaproteobacteria</td></tr>
<tr><td>OIP44723.1</td><td>inactive_FtsH+FtsH</td><td>X5→ inactive_FtsH+FtsH*→</td><td>723</td><td>Deltaproteobacteria bacterium CG2_30_63_29</td><td>GCA_001873925.1</td><td>deltaproteobacteria</td></tr>
<tr><td>PJB47822.1</td><td>inactive_FtsH+FtsH</td><td>X5→ inactive_FtsH+FtsH*→</td><td>723</td><td>Deltaproteobacteria bacterium CG_4_9_14_3_um_filter_63_12</td><td>GCA_002789955.1</td><td>deltaproteobacteria</td></tr>
<tr><td>CEF49305.1</td><td>inactive_FtsH+FtsH</td><td>X6→ X5→ inactive_FtsH+FtsH*→</td><td>700</td><td>uncultured bacterium</td><td>GCA_000821145.1</td><td>bacteria</td></tr>
<tr><td>WP_141643777.1</td><td>inactive_FtsH+FtsH</td><td>Radical_SAM→ X6→ X5→ inactive_FtsH+FtsH*→ X3_analog→ a/b_unk+NEFF1+TPR_region→ MoxR→</td><td>705</td><td>Myxococcus sp. AM401</td><td>GCF_006636215.1</td><td>deltaproteobacteria</td></tr>
<tr><td>WP_071102283.1</td><td>inactive_FtsH+FtsH</td><td>inactive_FtsH+FtsH*→ MoxR→?→||←?←?||X2→</td><td>660</td><td>Moorea producens</td><td>GCF_001854205.1</td><td>cyanobacteria</td></tr>
<tr><td>WP_010040329.1</td><td>inactive_FtsH+FtsH</td><td>Radical_SAM→ X6→ X5→ inactive_FtsH+FtsH*→ X3_analog→ a/b_unk+NEFF1+TPR_region→ a/b_unk+NEFF1+TPR_region→</td><td>748</td><td>Gemmata obscuriglobus</td><td>GCF_000171775.1</td><td>planctomycetes</td></tr>
<tr><td>RLS40983.1</td><td>inactive_FtsH+FtsH</td><td>X5→ inactive_FtsH+FtsH*→ X3_analog→?+NEFF+?→ MoxR→</td><td>720</td><td>Planctomycetes bacterium</td><td>GCA_003669555.1</td><td>planctomycetes</td></tr>
<tr><td>MBH25655.1</td><td>inactive_FtsH+FtsH</td><td>inactive_FtsH+FtsH*→ X3_analog→</td><td>730</td><td>Myxococcales bacterium</td><td>GCA_002716585.1</td><td>deltaproteobacteria</td></tr>
<tr><td>MBK06498.1</td><td>inactive_FtsH+FtsH</td><td>inactive_FtsH+FtsH*→ X3_analog→</td><td>723</td><td>Deltaproteobacteria bacterium</td><td>GCA_002720785.1</td><td>deltaproteobacteria</td></tr>
<tr><td>TNE49083.1</td><td>inactive_FtsH+FtsH</td><td>inactive_FtsH+FtsH*→ X3_analog→</td><td>725</td><td>Deltaproteobacteria bacterium</td><td>GCA_006226375.1</td><td>deltaproteobacteria</td></tr>
<tr><td>WP_088257901.1</td><td>inactive_FtsH+FtsH</td><td>inactive_FtsH+FtsH*→ X3_analog→</td><td>722</td><td>Fimbriiglobus ruber</td><td>GCF_002197845.1</td><td>planctomycetes</td></tr>
<tr><td>TET34120.1</td><td>inactive_FtsH+FtsH</td><td>X5→ inactive_FtsH+FtsH*→?→?→ X3_analog→</td><td>729</td><td>Planctomycetes bacterium</td><td>GCA_004376375.1</td><td>planctomycetes</td></tr>
<tr><td>PZR18634.1</td><td>inactive_FtsH+FtsH</td><td>X5→ inactive_FtsH+FtsH*→ X3_analog→</td><td>704</td><td>Archangium gephyra</td><td>GCA_003243425.1</td><td>deltaproteobacteria</td></tr>
<tr><td>WP_006976996.1</td><td>inactive_FtsH+FtsH</td><td>X5→ inactive_FtsH+FtsH*→ X3_analog→?→ a/b_unk+NEFF+TPRs</td><td>707</td><td>Plesiocystis pacifica</td><td>GCF_000170895.1</td><td>deltaproteobacteria</td></tr>
<tr><td>HBI41239.1</td><td>inactive_FtsH+FtsH</td><td>X6→ X5→ inactive_FtsH+FtsH*→</td><td>716</td><td>Planctomycetales bacterium</td><td>GCA_003508275.1</td><td>planctomycetes</td></tr>
<tr><td>WP_106395424.1</td><td>inactive_FtsH+FtsH</td><td>Radical_SAM→ X6→ X5→ inactive_FtsH+FtsH*→</td><td>706</td><td>Enhygromyxa salina</td><td>GCF_002994615.1</td><td>deltaproteobacteria</td></tr>
<tr><td>WP_131772555.1</td><td>inactive_FtsH+FtsH</td><td>inactive_FtsH+FtsH*→ MoxR→ X2→||←?||TPRs→</td><td>511</td><td>Frankia symbiont of Coriaria nepalensis</td><td>GCF_900635385.1</td><td>actinobacteria</td></tr>
<tr><td>WP_013873919.1</td><td>inactive_FtsH+FtsH</td><td>X1→ inactive_FtsH+FtsH*→ MoxR→ X2→||←?||TPRs→</td><td>735</td><td>Frankia</td><td>GCF_000177615.2</td><td>actinobacteria</td></tr>
<tr><td>WP_076843094.1</td><td>inactive_FtsH+FtsH</td><td>X1→ inactive_FtsH+FtsH*→ MoxR→ X2→?→ TPRs→</td><td>744</td><td>Frankia sp. BMG5.30</td><td>GCF_001983005.1</td><td>actinobacteria</td></tr>
</table>

β-propeller containing ternary systems

acc	architecture	operon	len	species	gca	taxend
OFY83689.1	extended_strand+helical_region+vWA+beta_propeller+beta_rich_region+bpX1+Ribosomal_L12_insert	bpX4→ MoxR→ bpX2→ extended_strand+helical_region+vWA+beta_propeller+beta_rich_region+bpX1+Ribosomal_L12_insert*→ bpX3→	846	Bacteroidetes bacterium RIFCSPLOWO2_12_FULL_35_15	GCA_001769385.1	bacteroidetes
AFM05601.1	extended_strand+helical_region+vWA+beta_propeller+beta_rich_region+bpX1+Ribosomal_L12_insert	bpX4→?→ MoxR→?→ bpX2→ extended_strand+helical_region+vWA+beta_propeller+beta_rich_region+bpX1+Ribosomal_L12_insert*→	873	Bernardetia litoralis DSM 6794	GCA_000265505.1	bacteroidetes
POR24865.1	extended_strand+helical_region+vWA+beta_propeller+beta_rich_region+bpX1+Ribosomal_L12_insert	bpX4→ MoxR→ bpX2→ extended_strand+helical_region+vWA+beta_propeller+beta_rich_region+bpX1+Ribosomal_L12_insert*→ abpX3+2TM+TPR_region→	803	Flavobacterium columnare	GCA_002916795.1	bacteroidetes
OXA84853.1	extended_strand+helical_region+vWA+beta_propeller+beta_rich_region+bpX1+Ribosomal_L12_insert	bpX4→ MoxR→ bpX2→ extended_strand+helical_region+vWA+beta_propeller+beta_rich_region+bpX1+Ribosomal_L12_insert*→	851	Flavobacterium hydatis	GCA_002217335.1	bacteroidetes
SEN93879.1	extended_strand+helical_region+vWA+beta_propeller+beta_rich_region+bpX1+Ribosomal_L12_insert	bpX4→ MoxR→ bpX2→ extended_strand+helical_region+vWA+beta_propeller+beta_rich_region+bpX1+Ribosomal_L12_insert*→ abpX3+2TM+TPR_region→	845	Flavobacterium sp. fv08	GCA_900110335.1	bacteroidetes
KOY86779.1	extended_strand+helical_region+vWA+beta_propeller+beta_rich_region+bpX1	bpX4→ MoxR→ bpX2→ extended_strand+helical_region+vWA+beta_propeller+beta_rich_region+bpX1*→	767	bacterium 336/3	GCA_001281695.1	bacteria
WP_128150033.1	extended_strand+helical_region+vWA+beta_propeller+beta_rich_region+bpX1	bpX4→ MoxR→ bpX2→ extended_strand+helical_region+vWA+beta_propeller+beta_rich_region+bpX1*→ bpX3+4TM→2TM+TPR_region→	779	Apibacter sp. HY041	GCF_004014855.1	bacteroidetes
PWK28201.1	extended_strand+helical_region+vWA+beta_propeller+beta_rich_region+bpX1	bpX4→ MoxR→ bpX2→ extended_strand+helical_region+vWA+beta_propeller+beta_rich_region+bpX1*→ bpX3+TPR_region→	808	Arcicella aurantiaca	GCA_003148625.1	bacteroidetes
WP_052599568.1	extended_strand+helical_region+vWA+beta_propeller+beta_rich_region+bpX1	bpX4→ MoxR→ bpX2→ extended_strand+helical_region+vWA+beta_propeller+beta_rich_region+bpX1*→ bpX3+TPR_region→	776	Aureispira sp. CCB-QB1	GCF_000724545.1	bacteroidetes
PSL46654.1	extended_strand+helical_region+vWA+beta_propeller+beta_rich_region+bpX1	MoxR→ bpX2→ extended_strand+helical_region+vWA+beta_propeller+beta_rich_region+bpX1*→ bpX3+TPR_region+beta_propeller→	789	Chitinophaga niastensis	GCA_003014755.1	bacteroidetes
RBL92136.1	extended_strand+helical_region+vWA+beta_propeller+beta_rich_region+bpX1	bpX4→ MoxR→ bpX2→ extended_strand+helical_region+vWA+beta_propeller+beta_rich_region+bpX1*→ bpX3+TPR_region+beta_propeller→	787	Chitinophaga sp. GDMCC 1.1325	GCA_003308995.1	bacteroidetes
RFM30704.1	extended_strand+helical_region+vWA+beta_propeller+beta_rich_region+bpX1	bpX4→ MoxR→ bpX2→ extended_strand+helical_region+vWA+beta_propeller+beta_rich_region+bpX1*→ bpX3+TPR_region+beta_propeller→ HTH+TetR_C→	775	Chitinophaga sp. K20C18050901	GCA_003412465.1	bacteroidetes
PWV51444.1	extended_strand+helical_region+vWA+beta_propeller+beta_rich_region+bpX1	bpX4→ MoxR→ bpX2→ extended_strand+helical_region+vWA+beta_propeller+beta_rich_region+bpX1*→ bpX3+TPR_region+beta_propeller→ HTH+TetR_C→	817	Chitinophaga sp. S165	GCA_003182155.1	bacteroidetes
SEW36401.1	extended_strand+helical_region+vWA+beta_propeller+beta_rich_region+bpX1	bpX4→ MoxR→ bpX2→ extended_strand+helical_region+vWA+beta_propeller+beta_rich_region+bpX1*→ bpX3+TPR_region+beta_propeller→ HTH+TetR_C→	597	Chitinophaga sp. YR573	GCA_900110995.1	bacteroidetes
SFO49267.1	extended_strand+helical_region+vWA+beta_propeller+beta_rich_region+bpX1	bpX4→ MoxR→ bpX2→ extended_strand+helical_region+vWA+beta_propeller+beta_rich_region+bpX1*→ bpX3+TPR_region+beta_propeller→	829	Chitinophaga sp. YR627	GCA_900114995.1	bacteroidetes
SDQ16602.1	extended_strand+helical_region+vWA+beta_propeller+beta_rich_region+bpX1	MoxR→ bpX2→ extended_strand+helical_region+vWA+beta_propeller+beta_rich_region+bpX1*→ bpX3+4TM→2TM+TPR_region→	802	Chryseobacterium soldanellicola	GCA_900100115.1	bacteroidetes
ABG58961.1	extended_strand+helical_region+vWA+beta_propeller+beta_rich_region+bpX1	bpX4→ MoxR→ bpX2→ extended_strand+helical_region+vWA+beta_propeller+beta_rich_region+bpX1*→ bpX3+TPR_region→	843	Cytophaga hutchinsonii ATCC 33406	GCA_000014145.1	bacteroidetes
WP_108823377.1	extended_strand+helical_region+vWA+beta_propeller+beta_rich_region+bpX1	bpX4→ MoxR→ bpX2→ extended_strand+helical_region+vWA+beta_propeller+beta_rich_region+bpX1*→ bpX3+TPR_region→	770	Dysgonomonas sp. Marseille-P4361	GCF_900343125.1	bacteroidetes
BAV07989.1	extended_strand+helical_region+vWA+beta_propeller+beta_rich_region+bpX1	bpX4→ MoxR→ bpX2→ extended_strand+helical_region+vWA+beta_propeller+beta_rich_region+bpX1→ *extended_strand+helical_region+vWA+beta_propeller+beta_rich_region+bpX1*→ bpX3+TPR_region→	883	Filimonas lacunae	GCA_002355595.1	bacteroidetes
PVY14782.1	extended_strand+helical_region+vWA+beta_propeller+beta_rich_region+bpX1	bpX4→ MoxR→ bpX2→ extended_strand+helical_region+vWA+beta_propeller+beta_rich_region+bpX1*→ bpX3+TPR_region→	874	Filimonas sp. YR581	GCA_003097095.1	bacteroidetes
KIC92612.1	extended_strand+helical_region+vWA+beta_propeller+beta_rich_region+bpX1	bpX4→ MoxR→ bpX2→ extended_strand+helical_region+vWA+beta_propeller+beta_rich_region+bpX1*→ bpX3+TPR_region→	783	Flavihumibacter solisilvae	GCA_000814475.1	bacteroidetes
KYP15344.1	extended_strand+helical_region+vWA+beta_propeller+beta_rich_region+bpX1	bpX4→ MoxR→ bpX2→ extended_strand+helical_region+vWA+beta_propeller+beta_rich_region+bpX1*→ bpX3+TPR_region→	772	Flavihumibacter sp. CACIAM 22H1	GCA_001601815.1	bacteroidetes
ANE49440.1	extended_strand+helical_region+vWA+beta_propeller+beta_rich_region+bpX1	bpX4→ MoxR→ bpX2→ extended_strand+helical_region+vWA+beta_propeller+beta_rich_region+bpX1*→ bpX3→	781	Flavisolibacter tropicus	GCA_001644645.1	bacteroidetes
AWH86615.1	extended_strand+helical_region+vWA+beta_propeller+beta_rich_region+bpX1	bpX4→ MoxR→ bpX2→ extended_strand+helical_region+vWA+beta_propeller+beta_rich_region+bpX1*→ abpX3+2TM+TPR_region→	801	Flavobacterium album	GCA_003096035.1	bacteroidetes
WP_052705351.1	extended_strand+helical_region+vWA+beta_propeller+beta_rich_region+bpX1	bpX4→ MoxR→ bpX2→ extended_strand+helical_region+vWA+beta_propeller+beta_rich_region+bpX1*→ bpX3+4TM→2TM+TPR_region→	845	Flavobacterium sp. 316	GCF_000967805.1	bacteroidetes
AEE53802.1	extended_strand+helical_region+vWA+beta_propeller+beta_rich_region+bpX1	bpX4→ MoxR→ bpX2→ extended_strand+helical_region+vWA+beta_propeller+beta_rich_region+bpX1*→ bpX3+TPR_region+beta_propeller→	819	Haliscomenobacter hydrossis DSM 1100	GCA_000212735.1	bacteroidetes
PJJ59945.1	extended_strand+helical_region+vWA+beta_propeller+beta_rich_region+bpX1	bpX4→ MoxR→ bpX2→ extended_strand+helical_region+vWA+beta_propeller+beta_rich_region+bpX1*→ bpX3+2TM+TPR_region+beta_propeller→	828	Hymenobacter chitinivorans DSM 11115	GCA_002797555.1	bacteroidetes
RTQ48805.1	extended_strand+helical_region+vWA+beta_propeller+beta_rich_region+bpX1	MoxR→ bpX2→ extended_strand+helical_region+vWA+beta_propeller+beta_rich_region+bpX1*→	836	Hymenobacter gummosus	GCA_003970915.1	bacteroidetes
SNR82429.1	extended_strand+helical_region+vWA+beta_propeller+beta_rich_region+bpX1	bpX4→ MoxR→ bpX2→ extended_strand+helical_region+vWA+beta_propeller+beta_rich_region+bpX1*→ bpX3+TPR_region+beta_propeller→	813	Hymenobacter mucosus	GCA_900188255.1	bacteroidetes
WP_022821708.1	extended_strand+helical_region+vWA+beta_propeller+beta_rich_region+bpX1	bpX4→ MoxR→ bpX2→ extended_strand+helical_region+vWA+beta_propeller+beta_rich_region+bpX1*→	823	Hymenobacter norwichensis	GCF_000420705.1	bacteroidetes

acc	architecture	operon	len	species	gca	taxend
RSK36273.1	extended_strand+helical_region+vWA+beta_propeller+beta_rich_region+bpX1	MoxR→ bpX2→ extended_strand+helical_region+vWA+beta_propeller+beta_rich_region+bpX1*→	830	Hymenobacter sp. 9PBR-2	GCA_003944705.1	bacteroidetes
EDP94883.1	extended_strand+helical_region+vWA+beta_propeller+beta_rich_region+bpX1	bpX4→ MoxR→ bpX2→ extended_strand+helical_region+vWA+beta_propeller+beta_rich_region+bpX1*→ bpX3+4TM→2TM+TPR_region→	799	Kordia algicida OT-1	GCA_000154725.1	bacteroidetes
RKR84050.1	extended_strand+helical_region+vWA+beta_propeller+beta_rich_region+bpX1	bpX4→ MoxR→ bpX2→ extended_strand+helical_region+vWA+beta_propeller+beta_rich_region+bpX1*→	788	Mucilaginibacter gracilis	GCA_003633615.1	bacteroidetes
EHQ24573.1	extended_strand+helical_region+vWA+beta_propeller+beta_rich_region+bpX1	extended_strand+helical_region+vWA+beta_propeller+beta_rich_region+bpX1*→ bpX3+TPR_region+beta_propeller→	784	Mucilaginibacter paludis DSM 18603	GCA_000166195.3	bacteroidetes
RVU02898.1	extended_strand+helical_region+vWA+beta_propeller+beta_rich_region+bpX1	bpX4→ MoxR→ bpX2→ extended_strand+helical_region+vWA+beta_propeller+beta_rich_region+bpX1*→ bpX3+2TM+TPR_region+beta_propeller→	791	Mucilaginibacter sp. YBJ-36	GCA_004005815.1	bacteroidetes
AEW02126.1	extended_strand+helical_region+vWA+beta_propeller+beta_rich_region+bpX1	bpX4→ MoxR→ bpX2→ extended_strand+helical_region+vWA+beta_propeller+beta_rich_region+bpX1*→ bpX3+TPR_region+beta_propeller→	836	Niastella koreensis GR20-10	GCA_000246855.1	bacteroidetes
KRT17467.1	extended_strand+helical_region+vWA+beta_propeller+beta_rich_region+bpX1	bpX4→ MoxR→ bpX2→ extended_strand+helical_region+vWA+beta_propeller+beta_rich_region+bpX1*→ bpX3+2TM+TPR_region+beta_propeller→	791	Pedobacter ginsenosidimutans	GCA_001442625.1	bacteroidetes
RQO30278.1	extended_strand+helical_region+vWA+beta_propeller+beta_rich_region+bpX1	bpX4→ MoxR→ bpX2→ extended_strand+helical_region+vWA+beta_propeller+beta_rich_region+bpX1*→ bpX3+4TM→2TM+TPR_region→	787	Taibaiella sp. KBW10	GCA_003852495.1	bacteroidetes
PQO39541.1	extended_strand+helical_region+vWA+beta_propeller+beta_rich_region+bpX1	bpX4→ MoxR→ bpX2→ extended_strand+helical_region+vWA+beta_propeller+beta_rich_region+bpX1*→ bpX3+TPR_region+beta_propeller→	917	Blastopirellula marina	GCA_002967765.1	planctomycetes
OWK46343.1	extended_strand+helical_region+vWA+beta_propeller+beta_rich_region+bpX1	bpX2→ extended_strand+helical_region+vWA+beta_propeller+beta_rich_region+bpX1*→ bpX3+TPR_region+beta_propeller→	969	Fimbriiglobus ruber	GCA_002197845.1	planctomycetes
AMV26576.1	extended_strand+helical_region+vWA+beta_propeller+beta_rich_region+bpX1	bpX4→ MoxR→ bpX2→ extended_strand+helical_region+vWA+beta_propeller+beta_rich_region+bpX1*→ bpX3+TPR_region+beta_propeller→	960	Gemmata sp. SH-PL17	GCA_001610855.1	planctomycetes
RUL85748.1	extended_strand+helical_region+vWA+beta_propeller+beta_rich_region+bpX1	bpX4→ MoxR→ bpX2→ extended_strand+helical_region+vWA+beta_propeller+beta_rich_region+bpX1*→	960	Isosphaeraceae bacterium GM2012	GCA_003977685.1	planctomycetes
SFH92940.1	extended_strand+helical_region+vWA+beta_propeller+beta_rich_region+bpX1	bpX4→ MoxR→ bpX2→ extended_strand+helical_region+vWA+beta_propeller+beta_rich_region+bpX1*→ bpX3+TPR_region+beta_propeller→	904	Planctomicrobium piriforme	GCA_900113665.1	planctomycetes
AMV18939.1	extended_strand+helical_region+vWA+beta_propeller+beta_rich_region+bpX1	bpX4→ MoxR→ bpX2→ extended_strand+helical_region+vWA+beta_propeller+beta_rich_region+bpX1*→ bpX3+TPR_region+beta_propeller→	939	Planctomyces sp. SH-PL14	GCA_001610835.1	planctomycetes
RIK61979.1	extended_strand+helical_region+vWA+beta_propeller+beta_rich_region+bpX1	MoxR→ bpX2→ extended_strand+helical_region+vWA+beta_propeller+beta_rich_region+bpX1*→	969	Planctomycetes bacterium	GCA_003576915.1	planctomycetes
RLS72559.1	extended_strand+helical_region+vWA+beta_propeller+beta_rich_region+bpX1	bpX4→ MoxR→ bpX2→ extended_strand+helical_region+vWA+beta_propeller+beta_rich_region+bpX1*→ bpX3+2TM+TPR_region+??→	920	Planctomycetes bacterium	GCA_003669275.1	planctomycetes
OYP36357.1	extended_strand+helical_region+vWA+beta_propeller+beta_rich_region+bpX1	bpX4→ MoxR→ bpX2→ extended_strand+helical_region+vWA+beta_propeller+beta_rich_region+bpX1*→ bpX3+TPR_region+beta_propeller→	923	Rhodopirellula sp. MGV	GCA_002251265.1	planctomycetes
WP_044252884.1	extended_strand+helical_region+vWA+beta_propeller+beta_rich_region+bpX1	MoxR→ bpX2→ extended_strand+helical_region+vWA+beta_propeller+beta_rich_region+bpX1*→ bpX3+TPR_region+beta_propeller→	934	Rhodopirellula sp. SWK7	-	planctomycetes
WP_068134229.1	extended_strand+helical_region+vWA+beta_propeller+beta_rich_region+bpX1	bpX4→ MoxR→ bpX2→ extended_strand+helical_region+vWA+beta_propeller+beta_rich_region+bpX1*→ bpX3+TPR_region+beta_propeller→	926	Roseimaritima ulvae	GCF_001642915.1	planctomycetes
WP_010582785.1	extended_strand+helical_region+vWA+beta_propeller+beta_rich_region+bpX1	extended_strand+helical_region+vWA+beta_propeller+beta_rich_region+bpX1*→ bpX3+TPR_region+beta_propeller→	912	Schlesneria paludicola	GCF_000255655.1	planctomycetes
SIO62481.1	extended_strand+helical_region+vWA+beta_propeller+beta_rich_region+bpX1	bpX4→ MoxR→ bpX2→ extended_strand+helical_region+vWA+beta_propeller+beta_rich_region+bpX1*→ bpX3+TPR_region+beta_propeller→	930	Singulisphaera sp. GP187	GCA_900129635.1	planctomycetes
WP_020472485.1	extended_strand+helical_region+vWA+beta_propeller+beta_rich_region+bpX1	extended_strand+helical_region+vWA+beta_propeller+beta_rich_region+bpX1*→	910	Zavarzinella formosa	GCF_000255705.1	planctomycetes
WP_124744879.1	extended_strand+helical_region+vWA+beta_propeller+beta_rich_region+bpX1	bpX4→ MoxR→ bpX2→ extended_strand+helical_region+vWA+beta_propeller+beta_rich_region+bpX1*→ bpX3+TPR_region→	770	Glaciecola sp. THG-3.7	GCF_003856375.1	gammaproteobacteria
EDY17134.1	extended_strand+helical_region+vWA+beta_propeller+beta_rich_region+bpX1	bpX4→ MoxR→ bpX2→ extended_strand+helical_region+vWA+beta_propeller+beta_rich_region+bpX1*→ bpX3+TPR_region+beta_propeller→	928	Chthoniobacter flavus Ellin428	GCA_000173075.1	verrucomicrobia
EEF58752.1	extended_strand+helical_region+vWA+beta_propeller+beta_rich_region+bpX1	bpX4→ MoxR→ bpX2→ extended_strand+helical_region+vWA+beta_propeller+beta_rich_region+bpX1*→?→ bpX3+TPR_region+beta_propeller→	799	Pedosphaera parvula Ellin514	GCA_000172555.1	verrucomicrobia
RBP36328.1	extended_strand+helical_region+vWA+beta_propeller+beta_rich_region+bpX1	bpX4→ MoxR→ bpX2→ extended_strand+helical_region+vWA+beta_propeller+beta_rich_region+bpX1*→ bpX3+TPR_region+beta_propeller→	841	Roseimicrobium gellanilyticum	GCA_003315205.1	verrucomicrobia
PTY04067.1	extended_strand+helical_region+vWA+beta_propeller+beta_rich_region+bpX1	extended_strand+helical_region+vWA+beta_propeller+beta_rich_region+bpX1*→	1028	Verrucomicrobia bacterium LW23	GCA_003054695.1	verrucomicrobia
WP_009959261.1	extended_strand+helical_region+vWA+beta_propeller+beta_rich_region+bpX1	bpX4→ MoxR→ bpX2→ extended_strand+helical_region+vWA+beta_propeller+beta_rich_region+bpX1*→ bpX3+TPR_region+beta_propeller→	896	Verrucomicrobium spinosum	GCF_000172155.1	verrucomicrobia
WP_030304647.1	vWA+beta_propeller	MoxR→ bpX5→ vWA+beta_propeller*→ bpX6+TMs+TPRs+Betapropeller→	599	Streptomyces sp. NRRL F-6131	GCF_000719695.1	actinobacteria
PJI85297.1	vWA+beta_propeller	MoxR→?→ vWA+beta_propeller*→	626	Yoonia maricola	GCA_002797915.1	alphaproteobacteria
ODA19159.1	vWA+beta_propeller	MoxR→ bpX5→ vWA+beta_propeller*→ bpX6+TPRs+Betapropeller→	586	Achromobacter xylosoxidans	GCA_001707535.1	betaproteobacteria
KPC53758.1	vWA+beta_propeller	MoxR→ bpX5→ vWA+beta_propeller*→ bpX6+TPRs+Betapropeller→	605	Amantichitinum ursilacus	GCA_001294205.1	betaproteobacteria
SMC19581.1	vWA+beta_propeller	MoxR→ bpX5→ vWA+beta_propeller*→ bpX6+TMs+TPRs+Betapropeller→	597	Andreprevotia lacus DSM 23236	GCA_900176275.1	betaproteobacteria
AAQ60410.1	vWA+beta_propeller	MoxR→ bpX5→ vWA+beta_propeller*→	597	Chromobacterium violaceum ATCC 12472	GCA_000007705.1	betaproteobacteria
RFP17714.1	vWA+beta_propeller	MoxR→ bpX5→ vWA+beta_propeller*→ bpX6+TMs+TPRs+Betapropeller→	583	Duganella sp. BJB489	GCA_003416915.1	betaproteobacteria
KQZ25959.1	vWA+beta_propeller	MoxR→ bpX5→ vWA+beta_propeller*→ bpX6+TMs+TPRs+Betapropeller→	589	Duganella sp. Root1480D1	GCA_001427305.1	betaproteobacteria
WP_051057605.1	vWA+beta_propeller	MoxR→ bpX5→ vWA+beta_propeller*→?→ bpX6+TPRs+Betapropeller→	602	Herbaspirillum sp. B39	GCF_000333495.1	betaproteobacteria
SHH13774.1	vWA+beta_propeller	MoxR→ bpX5→ vWA+beta_propeller*→ bpX6+TMs+TPRs+Betapropeller→	611	Massilia sp. CF038	GCA_900129765.1	betaproteobacteria
ATQ78387.1	vWA+beta_propeller	MoxR→ bpX5→ vWA+beta_propeller→ *vWA+beta_propeller*→ bpX6+TPRs+Betapropeller→	594	Massilia violaceinigra	GCA_002752675.1	betaproteobacteria
RAS17227.1	bpX6+TPRs+Betapropeller←vWA+beta_propeller	MoxR→ bpX5→ vWA+beta_propeller*→ bpX6+TPRs+Betapropeller→||←?←BetaPropeller←BetaPropeller	601	Microvirgula sp. AG722	GCA_003269145.1	betaproteobacteria
ANH68664.1	vWA+beta_propeller	MoxR→ bpX5→ vWA+beta_propeller*→ bpX6+TMs+TPRs+Betapropeller→	652	Mitsuaria sp. 7	GCA_001653795.1	betaproteobacteria
CBJ39465.1	vWA+beta_propeller	MoxR→ bpX5→ vWA+beta_propeller*→ bpX6+TMs+TPRs+Betapropeller→	614	Ralstonia solanacearum CMR15	GCA_000427195.1	betaproteobacteria
ARN22671.1	vWA+beta_propeller	MoxR→ bpX5→ vWA+beta_propeller*→ bpX6+TMs+TPRs+Betapropeller→	599	Rhizobacter gummiphilus	GCA_002116905.1	betaproteobacteria
KQW02656.1	vWA+beta_propeller	MoxR→ bpX5→ vWA+beta_propeller*→ bpX6+TPRs+Betapropeller→	607	Rhizobacter sp. Root1221	GCA_001425785.1	betaproteobacteria
RQP22489.1	vWA+beta_propeller	MoxR→ bpX5→ vWA+beta_propeller*→ bpX6+TMs+TPRs+Betapropeller→	616	Rhizobacter sp. S-16	GCA_003852895.1	betaproteobacteria
SDW66728.1	vWA+beta_propeller	MoxR→ bpX5→ vWA+beta_propeller*→ bpX6+TMs+TPRs+Betapropeller→	619	Variovorax sp. YR634	GCA_900106655.1	betaproteobacteria
KFA87837.1	vWA+beta_propeller	bpX5→ vWA+beta_propeller*→ bpX6+TPRs+Betapropeller→	609	Archangium violaceum Cb vi76	GCA_000733295.1	deltaproteobacteria

<table>
<tr><th>acc</th><th>architecture</th><th>operon</th><th>len</th><th>species</th><th>gca</th><th>taxend</th></tr>
<tr><td>RJS21768.1</td><td>vWA+beta_propeller</td><td>MoxR→ bpX5→ vWA+beta_propeller*→ bpX6+TPRs+Betapropeller→</td><td>606</td><td>Corallococcus sp. H22C18031201</td><td>GCA_003600865.1</td><td>deltaproteobacteria</td></tr>
<tr><td>AKF84122.1</td><td>vWA+beta_propeller</td><td>bpX5→ vWA+beta_propeller*→</td><td>640</td><td>Myxococcus fulvus 124B02</td><td>GCA_000988565.1</td><td>deltaproteobacteria</td></tr>
<tr><td>ATB51059.1</td><td>vWA+beta_propeller</td><td>MoxR→ bpX5→ vWA+beta_propeller*→ bpX6+TPRs+Betapropeller→</td><td>606</td><td>Myxococcus macrosporus DSM 14697</td><td>GCA_002305895.1</td><td>deltaproteobacteria</td></tr>
<tr><td>ABF92814.1</td><td>vWA+beta_propeller</td><td>MoxR→ bpX5→ vWA+beta_propeller*→ bpX6+TPRs+Betapropeller→</td><td>608</td><td>Myxococcus xanthus DK 1622</td><td>GCA_000012685.1</td><td>deltaproteobacteria</td></tr>
<tr><td>OTQ06733.1</td><td>vWA+beta_propeller</td><td>MoxR→ bpX5→ vWA+beta_propeller*→ bpX6+TMs+TPRs+Betapropeller→</td><td>588</td><td>Gilliamella apicola</td><td>GCA_002141575.1</td><td>gammaproteobacteria</td></tr>
<tr><td>ABC29563.1</td><td>vWA+beta_propeller</td><td>MoxR→ bpX5→ vWA+beta_propeller*→ bpX6+TPRs+Betapropeller→</td><td>623</td><td>Hahella chejuensis KCTC 2396</td><td>GCA_000012985.1</td><td>gammaproteobacteria</td></tr>
<tr><td>WP_022951101.1</td><td>vWA+beta_propeller</td><td>MoxR→ bpX5→ vWA+beta_propeller*→ bpX6+TPRs+Betapropeller→</td><td>608</td><td>Leucothrix mucor</td><td>GCF_000419525.1</td><td>gammaproteobacteria</td></tr>
<tr><td>WP_064748987.1</td><td>vWA+beta_propeller</td><td>MoxR→ bpX5→ vWA+beta_propeller*→</td><td>616</td><td>Lysobacter antibioticus</td><td>GCF_000336385.2</td><td>gammaproteobacteria</td></tr>
<tr><td>BAV97131.1</td><td>vWA+beta_propeller</td><td>MoxR→ bpX5→ vWA+beta_propeller*→ bpX6+TPRs+Betapropeller→</td><td>605</td><td>Lysobacter enzymogenes</td><td>GCA_002355975.1</td><td>gammaproteobacteria</td></tr>
<tr><td>KRA21135.1</td><td>vWA+beta_propeller</td><td>MoxR→ bpX5→ vWA+beta_propeller*→ bpX6+TPRs+Betapropeller→</td><td>608</td><td>Lysobacter sp. Root604</td><td>GCA_001427505.1</td><td>gammaproteobacteria</td></tr>
<tr><td>KRB02264.1</td><td>vWA+beta_propeller</td><td>MoxR→ bpX5→ vWA+beta_propeller*→ bpX6+TPRs+Betapropeller→</td><td>620</td><td>Lysobacter sp. Root690</td><td>GCA_001427785.1</td><td>gammaproteobacteria</td></tr>
<tr><td>PNL50802.1</td><td>vWA+beta_propeller</td><td>RADICAL_SAM.Ammemo→?→ MoxR→ bpX5→ vWA+beta_propeller*→ bpX6+TPRs+Betapropeller→</td><td>637</td><td>Proteus mirabilis</td><td>GCA_002206145.2</td><td>gammaproteobacteria</td></tr>
<tr><td>EKT57597.1</td><td>vWA+beta_propeller</td><td>bpX5→ vWA+beta_propeller*→</td><td>628</td><td>Providencia sneebia DSM 19967</td><td>GCA_000314895.2</td><td>gammaproteobacteria</td></tr>
<tr><td>KIH83594.1</td><td>vWA+beta_propeller</td><td>MoxR→ bpX5→ vWA+beta_propeller*→ bpX6+TMs+TPRs+Betapropeller→</td><td>606</td><td>Pseudomonas batumici</td><td>GCA_000820515.1</td><td>gammaproteobacteria</td></tr>
<tr><td>WP_117164965.1</td><td>vWA+beta_propeller</td><td>MoxR→ bpX5→ vWA+beta_propeller*→ bpX6+TMs+TPRs+Betapropeller→</td><td>598</td><td>Pseudomonas cichorii</td><td>GCF_003415775.1</td><td>gammaproteobacteria</td></tr>
<tr><td>WP_029532585.1</td><td>vWA+beta_propeller</td><td>MoxR→ PSE→ vWA+beta_propeller*→?→ TPRs+Betapropeller→</td><td>573</td><td>Pseudomonas fuscovaginae</td><td>GCF_000467005.1</td><td>gammaproteobacteria</td></tr>
<tr><td>RMP14790.1</td><td>vWA+beta_propeller</td><td>MoxR→ bpX5→ vWA+beta_propeller*→ bpX6+TMs+TPRs+Betapropeller→</td><td>610</td><td>Pseudomonas marginalis pv. marginalis</td><td>GCA_003700875.1</td><td>gammaproteobacteria</td></tr>
<tr><td>RMR06475.1</td><td>vWA+beta_propeller</td><td>vWA+beta_propeller*→</td><td>617</td><td>Pseudomonas savastanoi pv. glycinea</td><td>GCA_003701285.1</td><td>gammaproteobacteria</td></tr>
<tr><td>WP_122602356.1</td><td>vWA+beta_propeller</td><td>MoxR→ bpX5→ vWA+beta_propeller*→ bpX6+TMs+TPRs+Betapropeller→</td><td>608</td><td>Pseudomonas sp. 286</td><td>GCF_900582885.1</td><td>gammaproteobacteria</td></tr>
<tr><td>KIV62454.1</td><td>vWA+beta_propeller</td><td>MoxR→ bpX5→ vWA+beta_propeller*→ bpX6+TMs+TPRs+Betapropeller→</td><td>610</td><td>Pseudomonas sp. FeS53a</td><td>GCA_000931385.1</td><td>gammaproteobacteria</td></tr>
<tr><td>WP_082033854.1</td><td>vWA+beta_propeller</td><td>MoxR→ bpX5→ vWA+beta_propeller*→</td><td>614</td><td>Pseudomonas sp. PI1</td><td>GCF_000812405.1</td><td>gammaproteobacteria</td></tr>
<tr><td>BAQ80643.1</td><td>vWA+beta_propeller</td><td>MoxR→ bpX5→ vWA+beta_propeller*→ bpX6+TMs+TPRs+Betapropeller→</td><td>605</td><td>Pseudomonas sp. St29</td><td>GCA_001547915.1</td><td>gammaproteobacteria</td></tr>
<tr><td>SNZ04485.1</td><td>vWA+beta_propeller</td><td>MoxR→ bpX5→ vWA+beta_propeller*→ bpX6+TPRs+Betapropeller→</td><td>614</td><td>Pseudomonas sp. URIL14HWK12:I9</td><td>GCA_900215525.1</td><td>gammaproteobacteria</td></tr>
<tr><td>ODU30905.1</td><td>vWA+beta_propeller</td><td>MoxR→ bpX5→ vWA+beta_propeller*→ bpX6+TPRs+Betapropeller→</td><td>598</td><td>Xanthomonadaceae bacterium SCN 69-320</td><td>GCA_001725155.1</td><td>gammaproteobacteria</td></tr>
<tr><td>PPT63983.1</td><td>vWA+beta_propeller</td><td>MoxR→ bpX5→ vWA+beta_propeller*→ bpX6+TPRs+Betapropeller→</td><td>616</td><td>Xanthomonas arboricola</td><td>GCA_002940525.1</td><td>gammaproteobacteria</td></tr>
<tr><td>KMM74478.1</td><td>vWA+beta_propeller</td><td>MoxR→ bpX5→ vWA+beta_propeller*→ bpX6+TPRs+Betapropeller→</td><td>616</td><td>Xanthomonas sp. NCPPB 1128</td><td>GCA_001043115.1</td><td>gammaproteobacteria</td></tr>
<tr><td>WP_020510239.1</td><td>ClpN_repeats+vWA</td><td>ClpN_repeats+vWA→ ClpN_repeats+vWA→ ClpN_repeats+vWA→ ClpN_repeats+vWA→ ClpN_repeats+vWA→ ClpN_repeats+vWA→?→?→ EAD1+CASPASE→</td><td>1040</td><td>Actinoplanes globisporus</td><td>GCF_000379645.1</td><td>actinobacteria</td></tr>
<tr><td>SDT45587.1</td><td>beta_propeller+helical_linker+vWA</td><td>??→ Trypsin+EAD1→ beta_propeller+helical_linker+vWA*→ RADICAL_SAM.Ammemo→ RadSAM_pep→?→ E1+UFD→ EAD1+Ubl-Nterm+Ubl+E2→</td><td>1145</td><td>Actinoplanes derwentensis</td><td>GCA_900104725.1</td><td>actinobacteria</td></tr>
<tr><td>SDT45954.1</td><td>beta_propeller+helical_linker+vWA</td><td>low_complexity+a/b_unk+TPR_repeat→ CASPASE+Ploop_NTPase→ BetaPropeller→ a/b_hydrolase→ EAD1+TIR→ beta_propeller+helical_linker+vWA*→</td><td>1137</td><td>Actinoplanes derwentensis</td><td>GCA_900104725.1</td><td>actinobacteria</td></tr>
<tr><td>OXM68678.1</td><td>beta_propeller+helical_linker+vWA</td><td>MoxR→?→ X3→ TPRs→ low_complexity+TPRs→ beta_propeller+helical_linker+vWA*→||←RadSAM_pep←RADICAL_SAM.Ammemo</td><td>1038</td><td>Amycolatopsis sp. H5</td><td>GCA_002234595.1</td><td>actinobacteria</td></tr>
<tr><td>WP_033359495.1</td><td>beta_propeller+helical_linker+vWA</td><td>MoxR→?→ X3→ TPRs→ low_complexity+TPRs→ beta_propeller+helical_linker+vWA*→||←RadSAM_pep←RADICAL_SAM.Ammemo</td><td>1005</td><td>Dactylosporangium aurantiacum</td><td>GCF_000716715.1</td><td>actinobacteria</td></tr>
<tr><td>KLL11673.1</td><td>beta_propeller+helical_linker+vWA</td><td>beta_propeller+helical_linker+vWA*→</td><td>858</td><td>Frankia coriariae</td><td>GCA_001017755.1</td><td>actinobacteria</td></tr>
<tr><td>SDG79101.1</td><td>beta_propeller+helical_linker+vWA</td><td>TPRs→ low_complexity+TPRs→ beta_propeller+helical_linker+vWA*→||←RadSAM_pep</td><td>1050</td><td>Sinosporangium album</td><td>GCA_900100605.1</td><td>actinobacteria</td></tr>
<tr><td>AZP16236.1</td><td>beta_propeller+helical_linker+vWA</td><td>beta_propeller+helical_linker+vWA*→||←RadSAM_pep←RADICAL_SAM.Ammemo</td><td>1043</td><td>Streptomyces sp. GGCR-6</td><td>GCA_003955715.1</td><td>actinobacteria</td></tr>
<tr><td>KOV61826.1</td><td>←beta_propeller+helical_linker+vWA</td><td>X3→ TPRs→ low_complexity+TPRs→ beta_propeller+helical_linker+vWA*→||←RadSAM_pep←RADICAL_SAM.Ammemo</td><td>1055</td><td>Streptomyces sp. MMG1121</td><td>GCA_001280015.1</td><td>actinobacteria</td></tr>
<tr><td>OUL34959.1</td><td>←beta_propeller+helical_linker+vWA</td><td>X3→ TPRs+C-type_lectin→||←?||low_complexity+TPRs→ beta_propeller+helical_linker+vWA*→||←RADICAL_SAM.Ammemo</td><td>771</td><td>Nostoc sp. T09</td><td>GCA_002154695.1</td><td>cyanobacteria</td></tr>
<tr><td>WP_053332818.1</td><td>beta_propeller+helical_linker+vWA</td><td>beta_propeller+helical_linker+vWA*→ TPRs→</td><td>870</td><td>Haliangium ochraceum</td><td>GCF_000024805.1</td><td>deltaproteobacteria</td></tr>
<tr><td>WP_082673981.1</td><td>beta_propeller+helical_linker+vWA</td><td>X3→ TPRs→ low_complexity+TPRs→||←?||beta_propeller+helical_linker+vWA*→</td><td>1188</td><td>Thiohalocapsa sp. ML1</td><td>GCF_001469165.1</td><td>gammaproteobacteria</td></tr>
<tr><td>KHD04278.1</td><td>helical_linker+vWA</td><td>helical_linker+vWA*→</td><td>424</td><td>Candidatus Thiomargarita nelsonii</td><td>-</td><td>gammaproteobacteria</td></tr>
<tr><td>RKZ47048.1</td><td>helical_linker+vWA</td><td>helical_linker+vWA*→</td><td>424</td><td>Gammaproteobacteria bacterium</td><td>GCA_003645185.1</td><td>gammaproteobacteria</td></tr>
<tr><td>WP_035921195.1</td><td>vWA</td><td>vWA*→</td><td>273</td><td>Frankia sp. QA3</td><td>GCF_000262465.1</td><td>actinobacteria</td></tr>
<tr><td>ETR67287.1</td><td>vWA</td><td>vWA*→</td><td>221</td><td>Candidatus Magnetoglobus multicellularis str. Araruama</td><td>GCA_000516475.1</td><td>deltaproteobacteria</td></tr>
</table>

acc	architecture	operon	len	species	gca	taxend
ETR65220.1	vWA	vWA*→	229	Candidatus Magnetoglobus multicellularis str. Araruama	GCA_000516475.1	deltaproteobacteria
WP_086758355.1	vWA	vWA*→	152	Streptomyces scabiei	GCF_002156075.1	actinobacteria

GAP1-N1-type GTPase centric systems

acc	architecture	operon	len	species	gca	taxend
ADY35731.1	GAP1-N1+GAP1-M+GAP1-C	Calcineurin+GAPEAD1→?→ DO-GTPase2.1→ DO-GTPase1→ GAP1-N1+GAP1-M+GAP1-C*→	662	Bacteroides salanitronis DSM 18170	GCA_000190575.1	bacteroidetes
AEL07773.1	GAP1-N1+GAP1-M+GAP1-C+EAD1	GAP2→ DO-GTPase1→ GAP1-N1+GAP1-M+GAP1-C+EAD1*→	822	Xanthomonas campestris pv. raphani 756C	GCA_000221965.1	gammaproteobacteria
AGS80673.1	GAP1-N1+GAP1-M+GAP1-C+EAD1	GAP2→ ZnR+DO-GTPase2.1→ DO-GTPase1→ GAP1-N1+GAP1-M+GAP1-C+EAD1*→ PNP_UDP_1+EAD1→	845	Leptospira phage vb_LkmZ_Bejolso9-LE1	GCA_002603685.1	dsdna viruses, no rna stage
AIJ49675.1	GAP1-N1+GAP1-M+GAP1-C+EAD1	GAP2→ DO-GTPase1→ GAP1-N1+GAP1-M+GAP1-C+EAD1*→ CASPASE+EAD1→	830	Comamonas testosteroni TK102	GCA_000739375.1	betaproteobacteria
AJX95795.1	GAP1-N1+GAP1-M+GAP1-C	Calcineurin+GAPEAD1→ GAP2→ ZnR+DO-GTPase2.1→ DO-GTPase1→ GAP1-N1+GAP1-M+GAP1-C*→	704	Burkholderia pseudomallei PB08298010	GCA_000959345.1	betaproteobacteria
AMV35122.1	GAP1-N1+GAP1-M+GAP1-C+EAD1	GAP2→ ZnR+DO-GTPase2.1→ DO-GTPase1→ GAP1-N1+GAP1-M+GAP1-C+EAD1*→||←EAD1+Trypsin	844	Pirellula sp. SH-Sr6A	GCA_001610875.1	planctomycetes
ASL44118.1	GAP1-N1+GAP1-M+GAP1-C	Calcineurin+GAPEAD1→ GAP2→ ZnR+DO-GTPase2.1→ DO-GTPase1→ GAP1-N1+GAP1-M+GAP1-C*→	688	Burkholderia sp. AD24	GCA_002220305.1	betaproteobacteria
ASR53154.1	GAP1-N1+GAP1-M+GAP1-C	Calcineurin+GAPEAD1→ GAP2→ ZnR+DO-GTPase2.1→ DO-GTPase1→ GAP1-N1+GAP1-M+GAP1-C*→	871	Blastomonas fulva	GCA_003431825.1	alphaproteobacteria
AUC22653.1	GAP1-N1+GAP1-M+GAP1-C+PNP_UDP_1	GAP2→ ZnR+DO-GTPase2.1→ DO-GTPase1→ GAP1-N1+GAP1-M+GAP1-C+PNP_UDP_1*→	1031	Polaribacter sejongensis	GCA_002814075.1	bacteroidetes
BBC81760.1	GAP1-N1+GAP1-M+GAP1-C	AB_hydrolase+GAPEAD1→ GAP2→ ZnR+DO-GTPase2.1→ DO-GTPase1→ GAP1-N1+GAP1-M+GAP1-C*→	677	Acetobacter orientalis	GCA_003966365.1	alphaproteobacteria
BBD02433.1	GAP1-N1+GAP1-M+GAP1-C	Calcineurin+GAPEAD1→ GAP2→ ZnR+DO-GTPase2.1→ DO-GTPase1→ GAP1-N1+GAP1-M+GAP1-C*→	881	Sphingobium sp. YG1	GCA_003609795.1	alphaproteobacteria
CDG51235.1	GAP1-N1+GAP1-M+GAP1-C+EAD1	GAP2→ ZnR+DO-GTPase2.1→ DO-GTPase1→ GAP1-N1+GAP1-M+GAP1-C+EAD1*→ CASPASE+EAD1→	820	Halomonas sp. A3H3	GCA_000982975.1	gammaproteobacteria
CNI41896.1	GAP1-N1+GAP1-M+GAP1-C	Calcineurin+GAPEAD1→ GAP2→ DO-GTPase2.1→ DO-GTPase1→ GAP1-N1+GAP1-M+GAP1-C*→	651	Yersinia pekkanenii	GCA_001152565.1	gammaproteobacteria
CRE86890.1	GAP1-N1+GAP1-M+GAP1-C	Calcineurin+GAPEAD1→ GAP2→ ZnR+DO-GTPase2.1→ DO-GTPase1→ GAP1-N1+GAP1-M+GAP1-C*→	838	Yersinia intermedia	GCA_001319925.1	gammaproteobacteria
CUQ34010.1	GAP1-N1	DO-GTPase1→ GAP1-N1*→	43	Prevotella copri	GCA_001405915.1	bacteroidetes
EEQ09204.1	GAP1-N1+GAP1-M+GAP1-C	DO-GTPase2.1(fragment)→ DO-GTPase1→ GAP1-N1+GAP1-M+GAP1-C*→	589	Yersinia mollaretii ATCC 43969	GCA_000167995.1	gammaproteobacteria
EGG75068.1	GAP1-N1+GAP1-M+GAP1-C	Calcineurin+GAPEAD1→ GAP2→ ZnR+DO-GTPase2.1→ DO-GTPase1→ GAP1-N1+GAP1-M+GAP1-C*→	884	Gluconacetobacter sp. SXCC-1	GCA_000208635.2	alphaproteobacteria
EIT67703.1	GAP1-N1+GAP1-M+GAP1-C	Calcineurin+GAPEAD1→ DO-GTPase1→ GAP1-N1+GAP1-M+GAP1-C*→	904	Hydrocarboniphaga effusa AP103	GCA_000271305.1	gammaproteobacteria
EJF69446.1	GAP1-M+GAP1-C	Calcineurin+GAPEAD1→ GAP2→ DO-GTPase1→ GAP1-M+GAP1-C*→	793	Pseudomonas sp. Ag1	GCA_000278565.1	gammaproteobacteria
EJP17918.1	GAP1-N1	GAP2→ ZnR+DO-GTPase2.1→ DO-GTPase1→ GAP1-N1*→	61	Leptospira interrogans str. FPW2026	GCA_000243895.3	spirochaetes
EKO23840.1	GAP1-N1+GAP1-M+GAP1-C	Calcineurin+GAPEAD1→ GAP2→ ZnR+DO-GTPase2.1→ DO-GTPase1→ GAP1-N1+GAP1-M+GAP1-C*→	668	Leptospira interrogans str. UI 12621	GCA_000244275.3	spirochaetes
EKR43466.1	GAP1-N1+GAP1-M+GAP1-C	GAP1-N1+GAP1-M+GAP1-C*→	350	Leptospira interrogans serovar Grippotyphosa str. UI 08368	GCA_000244135.3	spirochaetes
EMG74074.1	GAP1-N1+GAP1-M+GAP1-C	Calcineurin+GAPEAD1→ GAP2→ ZnR+DO-GTPase2.1→ DO-GTPase1→ GAP1-N1+GAP1-M+GAP1-C*→	679	Salmonella enterica subsp. enterica serovar Newport str. SH111077	GCA_000344825.1	gammaproteobacteria
EMS40767.1	GAP1-N1+GAP1-M+GAP1-C	CASPASE+GAPEAD1→ GAP2→ ZnR+DO-GTPase2.1→ DO-GTPase1→ GAP1-N1+GAP1-M+GAP1-C*→	650	Methylobacterium mesophilicum SR1.6/6	GCA_000364445.1	alphaproteobacteria
ENV08026.1	GAP1-N1+GAP1-M+GAP1-C	GAP2→ ZnR+DO-GTPase2.1→ DO-GTPase1→ GAP1-N1+GAP1-M+GAP1-C*→ EAD1→ CASPASE+EAD1→	395	Acinetobacter sp. CIP 56.2	GCA_000368445.1	gammaproteobacteria
ERV84201.1	GAP1-N1+GAP1-M+GAP1-C	Calcineurin+GAPEAD1→ GAP2→ ZnR+DO-GTPase2.1→ DO-GTPase1→ GAP1-N1+GAP1-M+GAP1-C*→	840	Pseudomonas aeruginosa BWHPSA028	GCA_000481145.1	gammaproteobacteria
ESZ35911.1	GAP1-N1+GAP1-M+GAP1-C	Calcineurin+GAPEAD1→ GAP2→ ZnR+DO-GTPase2.1→ DO-GTPase1→ GAP1-N1+GAP1-M+GAP1-C*→	876	Mesorhizobium sp. L2C067A000	GCA_000502855.1	alphaproteobacteria
ETR71189.1	GAP1-N1+GAP1-M+GAP1-C	GrpE→?→?→ GrpE→ DO-GTPase2.1→ GAP1-N1+GAP1-M+GAP1-C*→	276	Candidatus Magnetoglobus multicellularis str. Araruama	GCA_000516475.1	deltaproteobacteria
EWG65005.1	GAP1-N1+GAP1-M+GAP1-C	Calcineurin+GAPEAD1→ GAP2→ DO-GTPase2.1→ DO-GTPase1→ GAP1-N1+GAP1-M+GAP1-C*→	651	Enterobacter sp. DC3	GCA_000568155.1	gammaproteobacteria
GAN50643.1	GAP1-N1+GAP1-M+GAP1-C	Calcineurin+GAPEAD1→ GAP2→ ZnR+DO-GTPase2.1→ DO-GTPase1→ GAP1-N1+GAP1-M+GAP1-C*→	858	Methylobacterium sp. ME121	GCA_000974725.1	alphaproteobacteria
KDD14872.1	GAP1-N1+GAP1-M+GAP1-C	DO-GTPase2.1(fragment)→ DO-GTPase1→ GAP1-N1+GAP1-M+GAP1-C*→	855	Bordetella bronchiseptica MBORD731	GCA_000698985.1	betaproteobacteria
KDL57610.1	GAP1-N1+GAP1-M+GAP1-C	Calcineurin+GAPEAD1→ GAP2→ DO-GTPase2.1→ DO-GTPase1→ GAP1-N1+GAP1-M+GAP1-C*→	651	Klebsiella variicola	GCA_000694595.1	gammaproteobacteria
KEC69694.1	GAP1-N1+GAP1-M+GAP1-C	Calcineurin+GAPEAD1→ GAP2→ DO-GTPase2.1→ GAP1-N1+GAP1-M+GAP1-C*→	907	Rhizobium leguminosarum bv. phaseoli CCGM1	GCA_000705615.1	alphaproteobacteria
KFB70201.1	GAP1-N1+GAP1-M+GAP1-C+EAD1	GAP2→ ZnR+DO-GTPase2.1→ DO-GTPase1→ GAP1-N1+GAP1-M+GAP1-C+EAD1*→ CASPASE+EAD1→	829	Candidatus Accumulibacter sp. SK-01	GCA_000584955.2	betaproteobacteria
KIA82238.1	GAP1-N1+GAP1-M+GAP1-C	Calcineurin+GAPEAD1→ GAP2→ ZnR+DO-GTPase2.1→ GAP1-N1+GAP1-M+GAP1-C*→	873	Chromobacterium piscinae	GCA_000812585.1	betaproteobacteria
KIP94692.1	GAP1-N1+GAP1-M+GAP1-C+EAD1	GAP2→ ZnR+DO-GTPase2.1→ DO-GTPase1→ GAP1-N1+GAP1-M+GAP1-C+EAD1*→ CASPASE+EAD1→	829	Pseudomonas fluorescens	GCA_000834545.1	gammaproteobacteria
KIU30546.1	GAP1-N1+GAP1-M+GAP1-C	Calcineurin+GAPEAD1→ GAP2→ ZnR+DO-GTPase2.1→ DO-GTPase1→ GAP1-N1+GAP1-M+GAP1-C*→	879	Methylobacterium radiotolerans	GCA_000878385.1	alphaproteobacteria
KJS35250.1	GAP1-N1+GAP1-M+GAP1-C+EAD1	GAP2→ ZnR+DO-GTPase2.1→ DO-GTPase1→ GAP1-N1+GAP1-M+GAP1-C+EAD1*→	749	Rhodospirillaceae bacterium BRH_c57	GCA_000961745.1	alphaproteobacteria
KJS38336.1	GAP1-N1+GAP1-M+GAP1-C	Calcineurin+GAPEAD1→ GAP2→ DO-GTPase2.1→ DO-GTPase1→ GAP1-N1+GAP1-M+GAP1-C*→	694	Hyphomonas sp. BRH_c22	GCA_000961795.1	alphaproteobacteria

acc	architecture	operon	len	species	gca	taxend
KMS75234.1	GAP1-N1+GAP1-M+GAP1-C+EAD1	GAP2→ ZnR+DO-GTPase2.1→ DO-GTPase1→ GAP1-N1+GAP1-M+GAP1-C+EAD1*→ CASPASE+EAD1→	824	Streptomyces viridochromogenes	GCA_001047325.1	actinobacteria
KOR33477.1	GAP1-N1+GAP1-M+GAP1-C	GAP1-N1+GAP1-M+GAP1-C*→	1014	Achromatium sp. WMS3	GCA_001276615.1	gammaproteobacteria
KPW87077.1	GAP1-N1+GAP1-M+GAP1-C	Calcineurin+GAPEAD1→ GAP2→ ZnR+DO-GTPase2.1→ GAP1-N1+GAP1-M+GAP1-C*→	880	Pseudomonas congelans	GCA_001400775.1	gammaproteobacteria
KQU31117.1	GAP1-N1+GAP1-M+GAP1-C	GAP1-N1+GAP1-M+GAP1-C*→	494	Methylobacterium sp. Leaf94	GCA_001426045.1	alphaproteobacteria
KQV53300.1	GAP1-N1+GAP1-M+GAP1-C	Calcineurin+GAPEAD1→ GAP2→ ZnR+DO-GTPase2.1→ DO-GTPase1→ GAP1-N1+GAP1-M+GAP1-C*→	820	Pelomonas sp. Root1217	GCA_001425705.1	betaproteobacteria
KVK81003.1	GAP1-N1+GAP1-M+GAP1-C	Calcineurin+GAPEAD1→ GAP2→||←?||DO-GTPase1→ GAP1-N1+GAP1-M+GAP1-C*→	676	Burkholderia ubonensis	GCA_001526225.1	betaproteobacteria
KVM36471.1	GAP1-N1+GAP1-M+GAP1-C	Calcineurin+GAPEAD1→ GAP2→||←?||DO-GTPase1→ GAP1-N1+GAP1-M+GAP1-C*→	704	Burkholderia ubonensis	GCA_001527385.1	betaproteobacteria
KVT48317.1	GAP1-N1+GAP1-M+GAP1-C	Calcineurin+GAPEAD1→ GAP2→ DO-GTPase2.1→ DO-GTPase1→ GAP1-N1+GAP1-M+GAP1-C*→	871	Burkholderia ubonensis	GCA_001528875.1	betaproteobacteria
KXG12688.1	GAP1-N1+GAP1-M+GAP1-C+EAD1	GAP2→ ZnR+DO-GTPase2.1→ DO-GTPase1→ GAP1-N1+GAP1-M+GAP1-C+EAD1*→ CASPASE+EAD1→	827	Pseudomonas aeruginosa	GCA_001560865.1	gammaproteobacteria
KYF95748.1	GAP1-N1+GAP1-M+GAP1-C	GAP1-N1+GAP1-M+GAP1-C*→||←DO-GTPase1←ZnR+DO-GTPase2.1←GAP2	868	Sorangium cellulosum	GCA_001589295.1	deltaproteobacteria
KZB53811.1	GAP1-N1+GAP1-M+GAP1-C	Calcineurin+GAPEAD1→ GAP2→ ZnR(fragment)+DO-GTPase2.1→ DO-GTPase1→ GAP1-N1+GAP1-M+GAP1-C*→	674	Thalassospira xiamenensis	GCA_001613855.1	alphaproteobacteria
OCX33136.1	GAP1-N1+GAP1-M+GAP1-C	DO-GTPase1→ GAP1-N1+GAP1-M+GAP1-C*→	872	Bradyrhizobium sp. UASWS1016	GCA_001705105.1	alphaproteobacteria
OFW09671.1	GAP1-N1+GAP1-M+GAP1-C	DO-GTPase1→ GAP1-N1+GAP1-M+GAP1-C*→	905	Acidobacteria bacterium RIFCSPLOWO2_12_FULL_59_11	GCA_001767115.1	acidobacteria
OGB06618.1	GAP1-N1+GAP1-M+GAP1-C	Calcineurin+GAPEAD1→ GAP2→ ZnR+DO-GTPase2.1→ DO-GTPase1→ GAP1-N1+GAP1-M+GAP1-C*→	687	Burkholderiales bacterium RIFCSPHIGHO2_02_FULL_64_19	GCA_001772075.1	betaproteobacteria
OHC26956.1	GAP1-N1+GAP1-M+GAP1-C	Calcineurin+GAPEAD1→ GAP2→ DO-GTPase2.1→ DO-GTPase1→ GAP1-N1+GAP1-M+GAP1-C*→	581	Pseudomonadales bacterium RIFCSPLOWO2_12_59_9	GCA_001830395.1	gammaproteobacteria
OJW12786.1	GAP1-N1+GAP1-M+GAP1-C	TIR+GAPEAD1→ GAP2→ ZnR+DO-GTPase2.1→ DO-GTPase1→ GAP1-N1+GAP1-M+GAP1-C*→	712	Mucilaginibacter sp. 44-25	GCA_001898335.1	bacteroidetes
OJW81738.1	GAP1-N1+GAP1-M+GAP1-C+EAD1	GAP2→ ZnR+DO-GTPase2.1→ DO-GTPase1→ GAP1-N1+GAP1-M+GAP1-C+EAD1*→ PNP_UDP_1+EAD1→	792	Bacteroidetes bacterium 46-16	GCA_001897835.1	bacteroidetes
OJX35082.1	GAP1-N1+GAP1-M+GAP1-C+EAD1	GAP2→ DO-GTPase2.1→ DO-GTPase1→ GAP1-N1+GAP1-M+GAP1-C+EAD1*→ CASPASE+EAD1→	822	Burkholderiales bacterium 68-20	GCA_001898665.1	betaproteobacteria
OKO35123.1	GAP1-N1+GAP1-M+GAP1-C	GAP2→ GAP2→ ZnR+DO-GTPase2.1→ DO-GTPase1→ DO-GTPase1→ GAP1-N1+GAP1-M+GAP1-C*→ EAD1→	381	Acinetobacter baumannii	GCA_001874765.1	gammaproteobacteria
OKO42867.1	GAP1-N1+GAP1-M+GAP1-C	PNP_UDP_1+EAD1→ GAP2→ ZnR+DO-GTPase2.1→ DO-GTPase1→ GAP1-N1+GAP1-M+GAP1-C*→ EAD1→	582	Acinetobacter baumannii	GCA_001874765.1	gammaproteobacteria
OLF53922.1	GAP1-N1+GAP1-M+GAP1-C+EAD1	GAP2→ ZnR+DO-GTPase2.1→ DO-GTPase1→ GAP1-N1+GAP1-M+GAP1-C+EAD1*→ CASPASE+EAD1→	819	Pseudomonas chlororaphis	GCA_001921865.1	gammaproteobacteria
OQW76544.1	GAP1-N1+GAP1-M+GAP1-C	Calcineurin+GAPEAD1→ GAP2→ ZnR+DO-GTPase2.1→ DO-GTPase1→ GAP1-N1+GAP1-M+GAP1-C*→	880	Proteobacteria bacterium ST_bin14	GCA_002083655.1	proteobacteria
OUM77232.1	GAP1-N1+GAP1-M+GAP1-C	GAP2→ ZnR+DO-GTPase2.1→ DO-GTPase1→ GAP1-N1+GAP1-M+GAP1-C*→	304	Acinetobacter baumannii	GCA_001432265.1	gammaproteobacteria
OUN76202.1	GAP1-N1+GAP1-M+GAP1-C	Calcineurin+GAPEAD1→?→ DO-GTPase2.1→ DO-GTPase1→ GAP1-N1+GAP1-M+GAP1-C*→	657	Alistipes sp. An54	GCA_002159375.1	bacteroidetes
OWK25036.1	GAP1-N1+GAP1-M+GAP1-C	GAPEAD1→ GAP2→ GAP1-N1+GAP1-M+GAP1-C*→?→ Calcineurin→	336	Rhizobium yanglingense	GCA_002200935.1	alphaproteobacteria
OYX25764.1	GAP1-N1+GAP1-M+GAP1-C+EAD1	GAP2→ ZnR+DO-GTPase2.1→ DO-GTPase1→ GAP1-N1+GAP1-M+GAP1-C+EAD1*→??+EAD1→ EAD1+Trypsin→	834	Flavobacteriales bacterium 32-35-8	GCA_002280395.1	bacteroidetes
OYY94018.1	GAP1-N1+GAP1-M+GAP1-C+EAD1	Calcineurin+EAD1→||←?||GAP2→ ZnR+DO-GTPase2.1→ DO-GTPase1→ GAP1-N1+GAP1-M+GAP1-C+EAD1*→	730	Hydrogenophilales bacterium 28-61-23	GCA_002281555.1	proteobacteria
PCI26491.1	GAP1-N1+GAP1-M+GAP1-C+PNP_UDP_1	GAP2→ DO-GTPase2.1→ DO-GTPase1→ GAP1-N1+GAP1-M+GAP1-C+PNP_UDP_1*→	1045	SAR324 cluster bacterium	GCA_002401295.1	deltaproteobacteria
PCI68527.1	GAP1-N1+GAP1-M+GAP1-C+EAD1	GAP2→ ZnR+DO-GTPase2.1→ DO-GTPase1→ GAP1-N1+GAP1-M+GAP1-C+EAD1*→	829	Gammaproteobacteria bacterium	GCA_002401655.1	gammaproteobacteria
PCI83911.1	GAP1-N1+GAP1-M+GAP1-C+CASPASE	GAP2→ ZnR+DO-GTPase2.1→ DO-GTPase1→ GAP1-N1+GAP1-M+GAP1-C+CASPASE*→	930	Ectothiorhodospiraceae bacterium	GCA_002401775.1	gammaproteobacteria
PCI90653.1	GAP1-N1+GAP1-M+GAP1-C+CASPASE	GAP2→ ZnR+DO-GTPase2.1→ DO-GTPase1→ GAP1-N1+GAP1-M+GAP1-C+CASPASE*→	930	Ectothiorhodospiraceae bacterium	GCA_002401775.1	gammaproteobacteria
PHV10311.1	GAP1-N1+GAP1-M+GAP1-C	Calcineurin+GAPEAD1→ GAP2→ ZnR+DO-GTPase2.1→ DO-GTPase1→ GAP1-N1+GAP1-M+GAP1-C*→	689	Chitinimonas sp. BJB300	GCA_002735695.1	betaproteobacteria
PHV25597.1	GAP1-N1+GAP1-M+GAP1-C+EAD1	GAP2→ DO-GTPase2.1→ DO-GTPase1→ GAP1-N1+GAP1-M+GAP1-C+EAD1*→ CASPASE+EAD1→	779	Janthinobacterium sp. BJB426	GCA_002735705.1	betaproteobacteria
PIL47826.1	GAP1-N1+GAP1-M+GAP1-C	PNP_UDP_1+EAD1→ GAP2→ ZnR+DO-GTPase2.1→ DO-GTPase1→ GAP1-N1+GAP1-M+GAP1-C*→	489	Acinetobacter baumannii	GCA_002760695.1	gammaproteobacteria
PIL52425.1	GAP1-N1+GAP1-M+GAP1-C	PNP_UDP_1+EAD1→ GAP2→ ZnR+DO-GTPase2.1→ DO-GTPase1→ GAP1-N1+GAP1-M+GAP1-C*→	469	Acinetobacter baumannii	GCA_002760725.1	gammaproteobacteria
PIL53638.1	GAP1-N1+GAP1-M+GAP1-C	PNP_UDP_1+EAD1→ GAP2→ ZnR+DO-GTPase2.1→ DO-GTPase1→ GAP1-N1+GAP1-M+GAP1-C*→	383	Acinetobacter baumannii	GCA_002760715.1	gammaproteobacteria
PIL63415.1	GAP1-N1+GAP1-M+GAP1-C	PNP_UDP_1+EAD1→ GAP2→ ZnR+DO-GTPase2.1→ DO-GTPase1→ GAP1-N1+GAP1-M+GAP1-C*→	161	Acinetobacter baumannii	GCA_002760785.1	gammaproteobacteria
PIL66775.1	GAP1-N1+GAP1-M+GAP1-C	GAP2→ ZnR+DO-GTPase2.1→ DO-GTPase1→ GAP1-N1+GAP1-M+GAP1-C*→	228	Acinetobacter baumannii	GCA_002760765.1	gammaproteobacteria
PIL69197.1	GAP1-N1+GAP1-M+GAP1-C	ZnR+DO-GTPase2.1→ DO-GTPase1→ GAP1-N1+GAP1-M+GAP1-C*→	212	Acinetobacter baumannii	GCA_002760825.1	gammaproteobacteria
PIL82486.1	GAP1-N1+GAP1-M+GAP1-C	GAP1-N1+GAP1-M+GAP1-C*→	426	Acinetobacter baumannii	GCA_002760895.1	gammaproteobacteria
PKG61900.1	GAP1-N1+GAP1-M+GAP1-C	DO-GTPase1→ GAP1-N1+GAP1-M+GAP1-C*→	645	Psychrobacter sp. Choline-02u-13	GCA_002836235.1	gammaproteobacteria
PKM46863.1	GAP1-N1+GAP1-M+GAP1-C	Calcineurin+GAPEAD1→ GAP2→ DO-GTPase2.1→ DO-GTPase1→ GAP1-N1+GAP1-M+GAP1-C*→	683	Gammaproteobacteria bacterium HGW-Gammaproteobacteria-1	GCA_002840095.1	gammaproteobacteria
PKM75314.1	GAP1-N1+GAP1-M+GAP1-C	DO-GTPase1→ GAP1-N1+GAP1-M+GAP1-C*→	639	Firmicutes bacterium HGW-Firmicutes-17	GCA_002841525.1	firmicutes
PKO39589.1	GAP1-N1+GAP1-M+GAP1-C	Calcineurin+GAPEAD1→ GAP2→ ZnR+DO-GTPase2.1→ PSE→ GAP1-N1+GAP1-M+GAP1-C*→	689	Betaproteobacteria bacterium HGW-Betaproteobacteria-6	GCA_002842145.1	betaproteobacteria
PKP48546.1	GAP1-N1+GAP1-M+GAP1-C+EAD1	GAP2→ ZnR+DO-GTPase2.1→ DO-GTPase1→ GAP1-N1+GAP1-M+GAP1-C+EAD1*→ PNP_UDP_1+EAD1→	829	Bacteroidetes bacterium HGW-Bacteroidetes-11	GCA_002842665.1	bacteroidetes
PLT23558.1	GAP1-N1+GAP1-M+GAP1-C	GAP2→ ZnR+DO-GTPase2.1→ DO-GTPase1→ GAP1-N1+GAP1-M+GAP1-C*→	378	Psychrobacter sp. MES7-P7E	GCA_002863645.1	gammaproteobacteria

acc	architecture	operon	len	species	gca	taxend
PMH59968.1	GAP1-N1+GAP1-M+GAP1-C	Calcineurin+GAPEAD1→ GAP2→ ZnR+DO-GTPase2.1→ DO-GTPase1→ GAP1-N1+GAP1-M+GAP1-C*→	611	Vibrio lentus	GCA_002873265.1	gammaproteobacteria
PMH70067.1	GAP1-N1+GAP1-M+GAP1-C+EAD1	PNP_UDP_1+EAD1→ GAP2→ ZnR+DO-GTPase2.1→ DO-GTPase1→ GAP1-N1+GAP1-M+GAP1-C+EAD1*→ SLATT→	805	Vibrio splendidus	GCA_002878555.1	gammaproteobacteria
PRY19984.1	GAP1-N1+GAP1-M+GAP1-C+EAD1	GAP2→ ZnR+DO-GTPase2.1→ DO-GTPase1→ GAP1-N1+GAP1-M+GAP1-C+EAD1*→ PNP_UDP_1+EAD1→	815	Pseudosporangium ferrugineum	GCA_003002065.1	actinobacteria
PRY35997.1	GAP1-N1+GAP1-M+GAP1-C+EAD1	GAP2→ ZnR+DO-GTPase2.1→ DO-GTPase1→ GAP1-N1+GAP1-M+GAP1-C+EAD1*→ PNP_UDP_1+EAD1→	813	Spirosoma oryzae	GCA_003002825.1	bacteroidetes
PSH02583.1	GAP1-N1+GAP1-M+GAP1-C	DO-GTPase1→ GAP1-N1+GAP1-M+GAP1-C*→	418	Acidobacteria bacterium	GCA_003010405.1	acidobacteria
SAQ17800.1	GAP1-N1+GAP1-M+GAP1-C	Calcineurin+GAPEAD1→ GAP2→ DO-GTPase2.1→ DO-GTPase1→ GAP1-N1+GAP1-M+GAP1-C*→	651	Klebsiella oxytoca	GCA_900084005.1	gammaproteobacteria
SAY47011.1	GAP1-N1+GAP1-M+GAP1-C	Calcineurin+GAPEAD1→ GAP2→ ZnR+DO-GTPase2.1→ DO-GTPase1→ GAP1-N1+GAP1-M+GAP1-C*→	855	Komagataeibacter rhaeticus	GCA_900086575.1	alphaproteobacteria
SDC45789.1	GAP1-N1+GAP1-M+GAP1-C	Calcineurin+GAPEAD1→ GAP2→ ZnR+DO-GTPase2.1→ DO-GTPase1→ GAP1-N1+GAP1-M+GAP1-C*→	872	Variovorax sp. CF079	GCA_900101545.1	betaproteobacteria
SDF29865.1	GAP1-N1+GAP1-M+GAP1-C	Calcineurin+GAPEAD1→ GAP2→ ZnR+DO-GTPase2.1→ DO-GTPase1→ GAP1-N1+GAP1-M+GAP1-C*→	643	Bradyrhizobium sp. R5	GCA_900101405.1	alphaproteobacteria
SDP87527.1	GAP1-N1+GAP1-M+GAP1-C	Calcineurin+GAPEAD1→ GAP2→ ZnR+DO-GTPase2.1→ DO-GTPase1→ GAP1-N1+GAP1-M+GAP1-C*→	873	Pseudomonas congelans	GCA_900103225.1	gammaproteobacteria
SDQ38520.1	GAP1-N1+GAP1-M+GAP1-C	Calcineurin+GAPEAD1→ GAP2→ ZnR+DO-GTPase2.1→ DO-GTPase1→ GAP1-N1+GAP1-M+GAP1-C*→	888	Pseudomonas sp. UC 17F4	GCA_900101695.1	gammaproteobacteria
SDX24507.1	GAP1-M+GAP1-C	Calcineurin+GAPEAD1→ GAP2→ ZnR+DO-GTPase2.1→ DO-GTPase1→ GAP1-M+GAP1-C*→	728	Lysobacter enzymogenes	GCA_900106525.1	gammaproteobacteria
SDY57230.1	GAP1-N1+GAP1-M+GAP1-C	Calcineurin+GAPEAD1→ GAP2→ ZnR+DO-GTPase2.1→ DO-GTPase1→ GAP1-N1+GAP1-M+GAP1-C*→	873	Pseudomonas syringae	GCA_900107345.1	gammaproteobacteria
SEA61952.1	GAP1-N1+GAP1-M+GAP1-C	Calcineurin+GAPEAD1→ GAP2→ ZnR+DO-GTPase2.1→ DO-GTPase1→ GAP1-N1+GAP1-M+GAP1-C*→	653	Acidovorax soli	GCA_900107605.1	betaproteobacteria
SED79523.1	GAP1-N1+GAP1-M+GAP1-C	Calcineurin+GAPEAD1→ GAP2→ ZnR+DO-GTPase2.1→ DO-GTPase1→ GAP1-N1+GAP1-M+GAP1-C*→	874	Paraburkholderia caballeronis	GCA_900104845.1	betaproteobacteria
SEQ94678.1	GAP1-N1+GAP1-M+GAP1-C	Calcineurin+GAPEAD1→ GAP2→ ZnR+DO-GTPase2.1→ DO-GTPase1→ GAP1-N1+GAP1-M+GAP1-C*→	653	Litorimicrobium taeanense	GCA_900110775.1	alphaproteobacteria
SER37675.1	GAP1-N1+GAP1-M+GAP1-C+EAD1	GAP2→ ZnR+DO-GTPase2.1→ DO-GTPase1→ GAP1-N1+GAP1-M+GAP1-C+EAD1*→ CASPASE+EAD1→	828	Giesbergeria anulus	GCA_900111115.1	betaproteobacteria
SFB61072.1	GAP1-N1+GAP1-M+GAP1-C	Calcineurin+GAPEAD1→ GAP2→ ZnR+DO-GTPase2.1→ DO-GTPase1→ GAP1-N1+GAP1-M+GAP1-C*→ SLATT→	689	Azotobacter beijerinckii	GCA_900112015.1	gammaproteobacteria
SFD88056.1	GAP1-N1+GAP1-M+GAP1-C	Calcineurin+GAPEAD1→ GAP2→ DO-GTPase2.1→ DO-GTPase1→ GAP1-N1+GAP1-M+GAP1-C*→	690	Blastococcus sp. DSM 46838	GCA_900112815.1	actinobacteria
SFU95039.1	GAP1-N1+GAP1-M+GAP1-C	Calcineurin+GAPEAD1→ GAP2→ ZnR+DO-GTPase2.1→ DO-GTPase1→ GAP1-N1+GAP1-M+GAP1-C*→	880	Pseudomonas sp. OV546	GCA_900116845.1	gammaproteobacteria
SHI10396.1	GAP1-N1+GAP1-M+GAP1-C	Calcineurin+GAPEAD1→ GAP2→ DO-GTPase2.1→ DO-GTPase1→ GAP1-N1+GAP1-M+GAP1-C*→	653	Sporobacter termitidis DSM 10068	GCA_900130065.1	firmicutes
SIR73769.1	GAP1-N1+GAP1-M+GAP1-C	Calcineurin+GAPEAD1→ GAP2→ ZnR+DO-GTPase2.1→ DO-GTPase1→ GAP1-N1+GAP1-M+GAP1-C*→	888	Pseudomonas putida	GCA_900156185.1	gammaproteobacteria
SLY49252.1	GAP1-N1+GAP1-M+GAP1-C	GAPEAD1→ GAP2→ DO-GTPase1→ GAP1-N1+GAP1-M+GAP1-C*→	651	Klebsiella pneumoniae	GCA_900174015.1	gammaproteobacteria
SOD32715.1	GAP1-N1+GAP1-M+GAP1-C+EAD1	GAP2→ ZnR+DO-GTPase2.1→ DO-GTPase1→ GAP1-N1+GAP1-M+GAP1-C+EAD1*→ CASPASE+EAD1→	825	Serratia sp. JKS296	GCA_900215445.1	gammaproteobacteria
WP_000583717.1	GAP1-N1+GAP1-M+GAP1-C+EAD1	GAP2→ ZnR+DO-GTPase2.1→ DO-GTPase1→ GAP1-N1+GAP1-M+GAP1-C+EAD1*→ PNP_UDP_1+EAD1→	842	Leptospira interrogans	GCA_000216135.3	spirochaetes
WP_002184169.1	GAP1-N1+GAP1-M+GAP1-C+EAD1	GAP2→ ZnR+DO-GTPase2.1→ DO-GTPase1→ GAP1-N1+GAP1-M+GAP1-C+EAD1*→ PNP_UDP_1+EAD1→	842	Leptospira interrogans	GCA_000306495.2	spirochaetes
WP_002551483.1	GAP1-N1+GAP1-M+GAP1-C+EAD1	GAP2→ DO-GTPase2.1→ DO-GTPase1→ GAP1-N1+GAP1-M+GAP1-C+EAD1*→ CASPASE(frag)→ PSE→ CASPASE+EAD1→	834	Pseudomonas savastanoi	GCA_000164015.3	gammaproteobacteria
WP_003585899.1	GAP1-N1+GAP1-M+GAP1-C	Calcineurin+GAPEAD1→ GAP2→ DO-GTPase2.1→ DO-GTPase1→ GAP1-N1+GAP1-M+GAP1-C*→	644	Rhizobium leguminosarum	GCA_000271785.1	alphaproteobacteria
WP_004293833.1	GAP1-N1+GAP1-M+GAP1-C	Calcineurin+GAPEAD1→?→ DO-GTPase2.1→ DO-GTPase1→ GAP1-N1+GAP1-M+GAP1-C*→	663	Bacteroides	GCA_000738065.1	bacteroidetes
WP_004624951.1	GAP1-N1+GAP1-M+GAP1-C	Calcineurin+GAPEAD1→ GAP2→ DO-GTPase2.1→ DO-GTPase1→ GAP1-N1+GAP1-M+GAP1-C*→	655	[Clostridium] termitidis	GCA_000350485.1	firmicutes
WP_004911050.1	GAP1-N1+GAP1-M+GAP1-C+EAD1	GAP2→ ZnR+DO-GTPase2.1→ DO-GTPase1→ GAP1-N1+GAP1-M+GAP1-C+EAD1*→ CASPASE+EAD1→	826	Acinetobacter junii	GCA_000368665.1	gammaproteobacteria
WP_005142132.1	GAP1-N1+GAP1-M+GAP1-C+EAD1	PNP_UDP_1+EAD1→ GAP2→ ZnR+DO-GTPase2.1→ DO-GTPase1→ GAP1-N1+GAP1-M+GAP1-C+EAD1*→	798	Acinetobacter	GCA_001896005.1	gammaproteobacteria
WP_005265695.1	GAP1-N1+GAP1-M+GAP1-C+EAD1	PNP_UDP_1+EAD1→ GAP2→ ZnR+DO-GTPase2.1→ DO-GTPase1→ GAP1-N1+GAP1-M+GAP1-C+EAD1*→	798	Acinetobacter	GCA_003369745.1	gammaproteobacteria
WP_005682844.1	GAP1-N1+GAP1-M+GAP1-C	Calcineurin+GAPEAD1→?→ DO-GTPase2.1→ DO-GTPase1→ GAP1-N1+GAP1-M+GAP1-C*→	663	Bacteroides	GCA_001404375.1	bacteroidetes
WP_005792628.1	GAP1-N1+GAP1-M+GAP1-C	Calcineurin+GAPEAD1→ GAP2→ ZnR+DO-GTPase2.1→ DO-GTPase1→ GAP1-N1+GAP1-M+GAP1-C*→	681	Acidovorax delafieldii	GCA_000175235.1	betaproteobacteria
WP_006889296.1	GAP1-N1+GAP1-M+GAP1-C+EAD1	PNP_UDP_1+EAD1→ GAP2→||←?||GAP2→ ZnR+DO-GTPase2.1→ DO-GTPase1→ GAP1-N1+GAP1-M+GAP1-C+EAD1*→	814	Methylobacter tundripaludum	GCA_000190755.3	gammaproteobacteria
WP_006964510.1	GAP1-N1+GAP1-M+GAP1-C	??+PNP_UDP_1+EAD1→ GAP2→ ZnR+DO-GTPase2.1→ DO-GTPase1→ GAP1-N1+GAP1-M+GAP1-C*→?→ EAD1→	621	Desulfotignum phosphitoxidans	GCA_000350545.1	deltaproteobacteria
WP_007244465.1	GAP1-N1+GAP1-M+GAP1-C	Calcineurin+GAPEAD1→ GAP2→ ZnR+DO-GTPase2.1→ GAP1-N1+GAP1-M+GAP1-C*→	870	Pseudomonas syringae group genomosp. 3	GCA_002966555.1	gammaproteobacteria
WP_007766092.1	GAP1-N1+GAP1-M+GAP1-C+EAD1	GAP2→ PSE→ DO-GTPase1→ GAP1-N1+GAP1-M+GAP1-C+EAD1*→ CASPASE+EAD1→	813	Rhizobium sp. CF080	GCA_000282095.2	alphaproteobacteria
WP_007888757.1	GAP1-N1+GAP1-M+GAP1-C	Calcineurin+GAPEAD1→ GAP2→ DO-GTPase2.1→ DO-GTPase1→ GAP1-N1+GAP1-M+GAP1-C*→	609	Roseburia inulinivorans	GCA_000174195.1	firmicutes
WP_009169750.1	GAP1-N1+GAP1-M+GAP1-C	Calcineurin+GAPEAD1→ GAP2→ DO-GTPase2.1→ DO-GTPase1→ GAP1-N1+GAP1-M+GAP1-C*→	634	Clostridium sp. DL-VIII	GCA_000230835.1	firmicutes
WP_009284468.1	GAP1-N1+GAP1-M+GAP1-C+EAD1	GAP2→ DO-GTPase2.1→ DO-GTPase1→ GAP1-N1+GAP1-M+GAP1-C+EAD1*→ PNP_UDP_1+EAD1→	828	Fibrisoma limi	GCA_000296815.2	bacteroidetes
WP_009518308.1	GAP1-N1+GAP1-M+GAP1-C+EAD1	GAP2→ ZnR+DO-GTPase2.1→ DO-GTPase1→ GAP1-N1+GAP1-M+GAP1-C+EAD1*→ CASPASE+EAD1→	826	Acinetobacter baumannii	GCA_000314635.1	gammaproteobacteria
WP_010208169.1	GAP1-N1+GAP1-M+GAP1-C	Calcineurin+GAPEAD1→ GAP2→ ZnR+DO-GTPase2.1→ GAP1-N1+GAP1-M+GAP1-C*→	866	Pseudomonas syringae group genomosp. 3	GCF_000177455.1	gammaproteobacteria
WP_010335847.1	GAP1-N1+GAP1-M+GAP1-C	Calcineurin+GAPEAD1→ GAP2→ ZnR+DO-GTPase2.1→ DO-GTPase1→ GAP1-N1+GAP1-M+GAP1-C*→	896	Sphingobium yanoikuyae	-	alphaproteobacteria
WP_010801596.1	GAP1-N1+GAP1-M+GAP1-C	Calcineurin+GAPEAD1→?→ DO-GTPase2.1→ DO-GTPase1→ GAP1-N1+GAP1-M+GAP1-C*→	664	Parabacteroides	GCA_000364265.1	bacteroidetes
WP_011103173.1	GAP1-N1+GAP1-M+GAP1-C	DO-GTPase2.1→ DO-GTPase1→ GAP1-N1+GAP1-M+GAP1-C*→	650	Pseudomonas syringae group genomosp. 3	GCF_000007805.1	gammaproteobacteria
WP_011965441.1	GAP1-N1+GAP1-M+GAP1-C	Calcineurin+GAPEAD1→?→ DO-GTPase2.1→ DO-GTPase1→ GAP1-N1+GAP1-M+GAP1-C*→	659	Bacteroides vulgatus	GCA_000012825.1	bacteroidetes
WP_011995908.1	GAP1-N1+GAP1-M+GAP1-C+EAD1	GAP2→ ZnR+DO-GTPase2.1→ DO-GTPase1→ GAP1-N1+GAP1-M+GAP1-C+EAD1*→ CASPASE+EAD1→	824	Xanthobacter autotrophicus	GCA_000017645.1	alphaproteobacteria
WP_012406443.1	GAP1-N1+GAP1-M+GAP1-C	Calcineurin+GAPEAD1→ GAP2→ ZnR+DO-GTPase2.1→ DO-GTPase1→ GAP1-N1+GAP1-M+GAP1-C*→	666	Paraburkholderia phymatum	GCA_000020045.1	betaproteobacteria
WP_012548896.1	GAP1-N1+GAP1-M+GAP1-C	Calcineurin+GAPEAD1→ GAP2→?→ GAP2→ ZnR+DO-GTPase2.1→ DO-GTPase1→ GAP1-N1+GAP1-M+GAP1-C*→	603	Aliivibrio salmonicida	GCA_000196495.1	gammaproteobacteria
WP_012723147.1	GAP1-N1+GAP1-M+GAP1-C	Calcineurin+GAPEAD1→ GAP2→ ZnR+DO-GTPase2.1→ DO-GTPase1→ GAP1-N1+GAP1-M+GAP1-C*→	871	Pseudomonas fluorescens	GCA_000009225.1	gammaproteobacteria

acc	architecture	operon	len	species	gca	taxend
WP_012806743.1	GAP1-N1+GAP1-M+GAP1-C	GAP1-N1+GAP1-M+GAP1-C*→	696	Candidatus Accumulibacter phosphatis	GCA_000024165.1	betaproteobacteria
WP_013593491.1	GAP1-N1+GAP1-M+GAP1-C+EAD1	GAP2→ DO-GTPase2.1→ DO-GTPase1→ GAP1-N1+GAP1-M+GAP1-C+EAD1*→ CASPASE+EAD1→	822	Acidovorax avenae	GCA_000176855.2	betaproteobacteria
WP_014682791.1	GAP1-N1+GAP1-M+GAP1-C	GAP2→ DO-GTPase2.1→ DO-GTPase1→ GAP1-N1+GAP1-M+GAP1-C*→	789	Deinococcus gobiensis	GCA_000252445.1	deinococci
WP_015073721.1	GAP1-N1+GAP1-M+GAP1-C	CASPASE+GAPEAD1→ GAP2→ ZnR+DO-GTPase2.1→ DO-GTPase1→ GAP1-N1+GAP1-M+GAP1-C*→	664	Gluconobacter oxydans	GCA_000311765.1	alphaproteobacteria
WP_015241824.1	GAP1-N1+GAP1-M+GAP1-C+EAD1	GAP2→ ZnR+DO-GTPase2.1→ DO-GTPase1→ GAP1-N1+GAP1-M+GAP1-C+EAD1*→ CASPASE+EAD1→	825	Sinorhizobium meliloti	GCA_000320385.2	alphaproteobacteria
WP_017050961.1	GAP1-N1+GAP1-M+GAP1-C+EAD1	PNP_UDP_1+EAD1→ GAP2→ ZnR(fragment)+DO-GTPase2.1→ DO-GTPase1→ GAP1-N1+GAP1-M+GAP1-C+EAD1*→ SLATT→	809	Vibrio ordalii	GCA_000287135.2	gammaproteobacteria
WP_017258880.1	GAP1-N1+GAP1-M+GAP1-C	Calcineurin+GAPEAD1→ GAP2→ ZnR+DO-GTPase2.1→ DO-GTPase1→ GAP1-N1+GAP1-M+GAP1-C*→	882	Pedobacter arcticus	GCF_000302595.1	bacteroidetes
WP_018632651.1	GAP1-N1+GAP1-M+GAP1-C	Calcineurin+GAPEAD1→ GAP2→ ZnR+DO-GTPase2.1→ DO-GTPase1→ GAP1-N1+GAP1-M+GAP1-C*→	649	Neomegalonema perideroedes	GCF_000374145.1	alphaproteobacteria
WP_019600192.1	GAP1-N1+GAP1-M+GAP1-C+PNP_UDP_1	GAP2→ ZnR+DO-GTPase2.1→ DO-GTPase1→ GAP1-N1+GAP1-M+GAP1-C+PNP_UDP_1*→	1034	Rhodonellum	GCA_000473765.1	bacteroidetes
WP_020123514.1	GAP1-N1+GAP1-M+GAP1-C+EAD1	GAP2→ ZnR+DO-GTPase2.1→ DO-GTPase1→ GAP1-N1+GAP1-M+GAP1-C+EAD1*→ CASPASE+EAD1→	765	Streptomyces canus	GCF_000383615.1	actinobacteria
WP_021205085.1	GAP1-N1+GAP1-M+GAP1-C	Calcineurin+GAPEAD1→ GAP2→ ZnR+DO-GTPase2.1→ DO-GTPase1→ GAP1-N1+GAP1-M+GAP1-C*→	871	Pseudomonas aeruginosa	GCA_000455705.1	gammaproteobacteria
WP_021446629.1	GAP1-N1+GAP1-M+GAP1-C+EAD1	GAP2→ ZnR+DO-GTPase2.1→ DO-GTPase1→ GAP1-N1+GAP1-M+GAP1-C+EAD1*→ CASPASE+EAD1→	828	Alcaligenes sp. EGD-AK7	GCA_000465875.3	betaproteobacteria
WP_022696794.1	GAP1-N1+GAP1-M+GAP1-C+DNA-glycosylase+EAD1	GAP2→ DO-GTPase2.1→ DO-GTPase1→ GAP1-N1+GAP1-M+GAP1-C+DNA-glycosylase+EAD1*→ CASPASE+EAD1→	810	Euryhalocaulis caribicus	GCF_000412185.1	alphaproteobacteria
WP_023775588.1	GAP1-N1+GAP1-M+GAP1-C	GAP1-N1+GAP1-M+GAP1-C*→	511	Mesorhizobium sp. LNHC229A00	GCA_000502415.1	alphaproteobacteria
WP_023783068.1	GAP1-N1+GAP1-M+GAP1-C	Calcineurin+GAPEAD1→ GAP2→ DO-GTPase2.1→ DO-GTPase1→ GAP1-N1+GAP1-M+GAP1-C*→	643	Mesorhizobium sp. LNHC220B00	GCA_000502395.1	alphaproteobacteria
WP_023802006.1	GAP1-N1+GAP1-M+GAP1-C	Calcineurin+GAPEAD1→ GAP2→ ZnR(fragment)+DO-GTPase2.1→ DO-GTPase1→ GAP1-N1+GAP1-M+GAP1-C*→	677	Mesorhizobium sp. L48C026A00	GCA_000502375.1	alphaproteobacteria
WP_023808270.1	GAP1-N1+GAP1-M+GAP1-C	Calcineurin+GAPEAD1→ GAP2→ ZnR+DO-GTPase2.1→ DO-GTPase1→ GAP1-N1+GAP1-M+GAP1-C*→	880	Mesorhizobium sp. L2C085B000	GCA_000502895.1	alphaproteobacteria
WP_024138975.1	GAP1-N1+GAP1-M+GAP1-C	Calcineurin+GAPEAD1→ GAP2→ DO-GTPase2.1(fragment)→ DO-GTPase1→ GAP1-N1+GAP1-M+GAP1-C*→	629	Salmonella enterica	GCF_000344825.1	gammaproteobacteria
WP_024658860.1	GAP1-N1+GAP1-M+GAP1-C	Calcineurin+GAPEAD1→ GAP2→ ZnR+DO-GTPase2.1→ DO-GTPase1→ GAP1-N1+GAP1-M+GAP1-C*→	876	Pseudomonas syringae	GCF_000452545.1	gammaproteobacteria
WP_025177520.1	GAP1-N1+GAP1-M+GAP1-C	Calcineurin+GAPEAD1→ GAP2→ ZnR+DO-GTPase2.1→ DO-GTPase1→ GAP1-N1+GAP1-M+GAP1-C*→	671	Leptospira interrogans	GCA_002074835.1	spirochaetes
WP_025381480.1	GAP1-N1+GAP1-M+GAP1-C+EAD1	GAP2→ ZnR+DO-GTPase2.1→ DO-GTPase1→ GAP1-N1+GAP1-M+GAP1-C+EAD1*→ CASPASE+EAD1→	820	Yersinia similis	GCA_000582515.1	gammaproteobacteria
WP_025626868.1	GAP1-N1+GAP1-M+GAP1-C+EAD1	GAP2→ ZnR+DO-GTPase2.1→ DO-GTPase1→ GAP1-N1+GAP1-M+GAP1-C+EAD1*→	662	Vibrio parahaemolyticus	GCF_000490295.1	gammaproteobacteria
WP_025726211.1	GAP1-N1+GAP1-M+GAP1-C	Calcineurin+GAPEAD1→?→ DO-GTPase2.1→ DO-GTPase1→ GAP1-N1+GAP1-M+GAP1-C*→	664	Bacteroides sp. 14(A)	GCF_000526555.1	bacteroidetes
WP_026768844.1	GAP1-N1+GAP1-M+GAP1-C+EAD1	GAP2→ ZnR+DO-GTPase2.1→ DO-GTPase1→ GAP1-N1+GAP1-M+GAP1-C+EAD1*→ PNP_UDP_1+EAD1→	846	Asinibacterium sp. OR53	GCF_000515315.1	bacteroidetes
WP_027604116.1	GAP1-N1+GAP1-M+GAP1-C	Calcineurin+GAPEAD1→ GAP2→ ZnR+DO-GTPase2.1→ DO-GTPase1→ GAP1-N1+GAP1-M+GAP1-C*→	879	Pseudomonas	GCA_900177315.1	gammaproteobacteria
WP_027988968.1	GAP1-N1+GAP1-M+GAP1-C+EAD1	GAP2→ ZnR+DO-GTPase2.1→ DO-GTPase1→ GAP1-N1+GAP1-M+GAP1-C+EAD1*→ CASPASE+EAD1→	825	Sinorhizobium meliloti	GCA_004001485.1	alphaproteobacteria
WP_028170393.1	GAP1-M+GAP1-C	Calcineurin+GAPEAD1→ GAP2→ ZnR+DO-GTPase2.1→ DO-GTPase1→ GAP1-M+GAP1-C*→	783	Bradyrhizobium japonicum	GCA_000807315.1	alphaproteobacteria
WP_028672472.1	GAP1-N1+GAP1-M+GAP1-C	DO-GTPase1→ GAP1-N1+GAP1-M+GAP1-C*→	867	Saccharospirillum impatiens	GCF_000423605.1	gammaproteobacteria
WP_029095773.1	GAP1-N1+GAP1-M+GAP1-C	Calcineurin+GAPEAD1→ GAP2→ ZnR+DO-GTPase2.1→ DO-GTPase1→ GAP1-N1+GAP1-M+GAP1-C*→	611	Budvicia aquatica	GCA_002591785.1	gammaproteobacteria
WP_032370172.1	GAP1-N1+GAP1-M+GAP1-C+EAD1	GAP2→?→ DO-GTPase1→ GAP1-N1+GAP1-M+GAP1-C+EAD1*→ PNP_UDP_1+EAD1→ PNP_UDP_1→	818	Rhodococcus fascians	GCF_000760995.1	actinobacteria
WP_032389288.1	GAP1-N1+GAP1-M+GAP1-C+EAD1	GAP2→?→ DO-GTPase1→ GAP1-N1+GAP1-M+GAP1-C+EAD1*→ PNP_UDP_1+EAD1→ PNP_UDP_1→	818	Rhodococcus fascians	GCF_000761035.1	actinobacteria
WP_032815283.1	GAP1-N1+GAP1-M+GAP1-C	DO-GTPase2.1(fragment)→ DO-GTPase1→ GAP1-N1+GAP1-M+GAP1-C*→	564	Yersinia mollaretii	GCF_000167995.1	gammaproteobacteria
WP_032899633.1	GAP1-N1+GAP1-M+GAP1-C	Calcineurin+GAPEAD1→ GAP2→ ZnR+DO-GTPase2.1→ DO-GTPase1→ GAP1-N1+GAP1-M+GAP1-C*→	872	Pseudomonas sp. Ag1	GCF_000278565.1	gammaproteobacteria
WP_033159517.1	GAP1-N1+GAP1-M+GAP1-C+EAD1	PNP_UDP_1+EAD1→ GAP2→ ZnR+DO-GTPase2.1→ DO-GTPase1→ GAP1-N1+GAP1-M+GAP1-C+EAD1*→	811	Methylomonas sp. LW13	GCA_000702925.2	gammaproteobacteria
WP_033840772.1	GAP1-N1+GAP1-M+GAP1-C	Calcineurin+GAPEAD1→ GAP2→ ZnR+DO-GTPase2.1→ DO-GTPase1→ GAP1-N1+GAP1-M+GAP1-C*→	877	Bordetella bronchiseptica	GCF_000698985.1	betaproteobacteria
WP_035621591.1	GAP1-N1+GAP1-M+GAP1-C+EAD1	GAP2→ ZnR+DO-GTPase2.1→ DO-GTPase1→ GAP1-N1+GAP1-M+GAP1-C+EAD1*→??+EAD1→ EAD1+Trypsin→	845	Flavobacterium hydatis	GCA_000737695.1	bacteroidetes
WP_036748648.1	GAP1-N1+GAP1-M+GAP1-C+EAD1	PNP_UDP_1+EAD1→ GAP2→ ZnR(fragment)+DO-GTPase2.1→ DO-GTPase1→ GAP1-N1+GAP1-M+GAP1-C+EAD1*→ SLATT→	810	Photobacterium galatheae	GCA_000695255.1	gammaproteobacteria
WP_037146786.1	GAP1-M+GAP1-C+??	Calcineurin+GAPEAD1→ GAP2→ DO-GTPase2.1→ DO-GTPase1→ GAP1-M+GAP1-C+??*→	784	Rhizobium	GCA_003150695.1	alphaproteobacteria
WP_038637402.1	GAP1-N1+GAP1-M+GAP1-C+EAD1	PNP_UDP_1+EAD1→ GAP2→ ZnR(fragment)+DO-GTPase2.1→ DO-GTPase1→ GAP1-N1+GAP1-M+GAP1-C+EAD1*→ SLATT→	809	Pseudoalteromonas piratica	GCA_000788395.1	gammaproteobacteria
WP_038698912.1	GAP1-N1+GAP1-M+GAP1-C+EAD1	GAP2→ ZnR+DO-GTPase2.1→ DO-GTPase1→ GAP1-N1+GAP1-M+GAP1-C+EAD1*→?→??+EAD1→ EAD1+Trypsin→	850	Sphingobacterium sp. ML3W	GCA_000747525.1	bacteroidetes
WP_038799205.1	GAP1-N1+GAP1-M+GAP1-C+EAD1	GAP2→ DO-GTPase2.1→ DO-GTPase1→ GAP1-N1+GAP1-M+GAP1-C+EAD1*→ CASPASE+EAD1→	829	Burkholderia pseudomallei	GCA_000773415.1	betaproteobacteria
WP_039013431.1	GAP1-N1+GAP1-M+GAP1-C+EAD1	GAP2→ DO-GTPase2.1→ DO-GTPase1→ GAP1-N1+GAP1-M+GAP1-C+EAD1*→	828	Cupriavidus sp. IDO	GCA_000812465.1	betaproteobacteria
WP_039045051.1	GAP1-N1+GAP1-M+GAP1-C+EAD1	PNP_UDP_1+EAD1→ GAP2→ ZnR(fragment)+DO-GTPase2.1→ DO-GTPase1→ GAP1-N1+GAP1-M+GAP1-C+EAD1*→ SLATT→	807	Plesiomonas shigelloides	GCF_000813415.1	gammaproteobacteria
WP_039487494.1	GAP1-N1+GAP1-M+GAP1-C	Calcineurin+GAPEAD1→ GAP2→ ZnR+DO-GTPase2.1→ DO-GTPase1→ GAP1-N1+GAP1-M+GAP1-C*→	869	Pectobacterium polaris	GCA_000749915.1	gammaproteobacteria
WP_039554384.1	GAP1-N1+GAP1-M+GAP1-C+EAD1	PNP_UDP_1+EAD1→ GAP2→ ZnR(fragment)+DO-GTPase2.1→ DO-GTPase1→ GAP1-N1+GAP1-M+GAP1-C+EAD1*→ SLATT→	809	Vibrio vulnificus	GCA_000746665.1	gammaproteobacteria
WP_039766187.1	GAP1-N1+GAP1-M+GAP1-C	Calcineurin+GAPEAD1→ GAP2→ ZnR+DO-GTPase2.1→ DO-GTPase1→ GAP1-N1+GAP1-M+GAP1-C*→	870	Pseudomonas fluorescens	GCA_000817905.1	gammaproteobacteria
WP_039894283.1	GAP1-N1+GAP1-M+GAP1-C	CASPASE+GAPEAD1→ GAP2→ ZnR+DO-GTPase2.1→ DO-GTPase1→ GAP1-N1+GAP1-M+GAP1-C*→	657	Methylobacterium mesophilicum	GCF_000364445.1	alphaproteobacteria
WP_039999911.1	GAP1-N1+GAP1-M+GAP1-C	Calcineurin+GAPEAD1→ GAP2→ ZnR+DO-GTPase2.1→ DO-GTPase1→ GAP1-N1+GAP1-M+GAP1-C*→	880	Acetobacteraceae	GCA_002762195.1	alphaproteobacteria
WP_040455186.1	GAP1-N1+GAP1-M+GAP1-C	Calcineurin+GAPEAD1→ GAP2→ DO-GTPase2.1→ DO-GTPase1→ GAP1-N1+GAP1-M+GAP1-C*→	887	Hydrocarboniphaga effusa	GCF_000271305.1	gammaproteobacteria
WP_041583905.1	GAP1-N1+GAP1-M+GAP1-C	Calcineurin+GAPEAD1→?→ DO-GTPase2.1→ DO-GTPase1→ GAP1-N1+GAP1-M+GAP1-C*→	663	Bacteroides salanitronis	GCF_000190575.1	bacteroidetes
WP_041766525.1	GAP1-N1+GAP1-M+GAP1-C	Calcineurin+GAPEAD1→ GAP2→ ZnR+DO-GTPase2.1→ DO-GTPase1→ GAP1-N1+GAP1-M+GAP1-C*→	701	Paraburkholderia caribensis	GCA_003028645.1	betaproteobacteria
WP_042387557.1	GAP1-N1+GAP1-M+GAP1-C	Calcineurin+GAPEAD1→ GAP2→ ZnR+DO-GTPase2.1→ DO-GTPase1→ GAP1-N1+GAP1-M+GAP1-C*→	871	Pseudescherichia vulneris	GCA_000759795.1	gammaproteobacteria

acc	architecture	operon	len	species	gca	taxend
WP_043393687.1	GAP1-N1+GAP1-M+GAP1-C	GAP2→ ZnR+DO-GTPase2.1→ DO-GTPase1→ GAP1-N1+GAP1-M+GAP1-C*→	905	Archangium violaceum	GCA_000733295.1	deltaproteobacteria
WP_043607170.1	GAP1-N1+GAP1-M+GAP1-C	Calcineurin+GAPEAD1→ GAP2→ ZnR+DO-GTPase2.1→ DO-GTPase1→ GAP1-N1+GAP1-M+GAP1-C*→	673	Novosphingobium sp. B-7	GCF_000410615.1	alphaproteobacteria
WP_043816345.1	GAP1-N1+GAP1-M+GAP1-C	Calcineurin+GAPEAD1→ GAP2→ DO-GTPase2.1→ DO-GTPase1→ GAP1-N1+GAP1-M+GAP1-C*→	656	Aeromonas salmonicida	GCA_001643285.1	gammaproteobacteria
WP_044342662.1	GAP1-N1+GAP1-M+GAP1-C+EAD1	GAP2→ DO-GTPase2.1→ DO-GTPase1→ GAP1-N1+GAP1-M+GAP1-C+EAD1*→ CASPASE+EAD1→	828	Pseudomonas oryzihabitans	GCA_000935305.1	gammaproteobacteria
WP_045379206.1	GAP1-N1+GAP1-M+GAP1-C	Calcineurin+GAPEAD1→ GAP2→ ZnR+DO-GTPase2.1→ DO-GTPase1→ GAP1-N1+GAP1-M+GAP1-C*→	430	Vibrio campbellii	GCF_000818335.1	gammaproteobacteria
WP_045419498.1	GAP1-N1+GAP1-M+GAP1-C+EAD1	ZnR(fragment)+DO-GTPase2.1→ DO-GTPase1→ GAP1-N1+GAP1-M+GAP1-C+EAD1*→ SLATT→	809	Vibrio jasicida	GCF_000818715.1	gammaproteobacteria
WP_045492829.1	GAP1-N1+GAP1-M+GAP1-C+EAD1	PNP_UDP_1+EAD1→ GAP2→ ZnR(fragment)+DO-GTPase2.1→ DO-GTPase1→ GAP1-N1+GAP1-M+GAP1-C+EAD1*→ SLATT→	809	Vibrio hyugaensis	GCF_000818475.1	gammaproteobacteria
WP_045576370.1	GAP1-N1+GAP1-M+GAP1-C	GAP1-N1+GAP1-M+GAP1-C*→	668	Desulfosporosinus sp. I2	GCA_000960765.1	firmicutes
WP_045628704.1	GAP1-N1+GAP1-M+GAP1-C	Calcineurin+GAPEAD1→ GAP2→ ZnR+DO-GTPase2.1→ DO-GTPase1→ GAP1-N1+GAP1-M+GAP1-C*→	608	Vibrio vulnificus	GCF_000959835.1	gammaproteobacteria
WP_045752253.1	GAP1-N1+GAP1-M+GAP1-C+EAD1	DO-GTPase1→ GAP1-N1+GAP1-M+GAP1-C+EAD1*→??+EAD1→ EAD1+Trypsin→	847	Sphingobacterium sp. PM2-P1-29	GCA_000938735.2	bacteroidetes
WP_046017835.1	GAP1-N1+GAP1-M+GAP1-C	Calcineurin+GAPEAD1→ GAP2→ ZnR+DO-GTPase2.1→ DO-GTPase1→ GAP1-N1+GAP1-M+GAP1-C*→	656	Marinomonas sp. S3726	GCA_000967665.1	gammaproteobacteria
WP_046064071.1	GAP1-N1+GAP1-M+GAP1-C+EAD1	GAP2→ DO-GTPase2.1→ DO-GTPase1→ GAP1-N1+GAP1-M+GAP1-C+EAD1*→ CASPASE+EAD1→	826	Pseudomonas kilonensis	GCA_000968575.1	gammaproteobacteria
WP_046067188.1	GAP1-N1+GAP1-M+GAP1-C	Calcineurin+GAPEAD1→ GAP2→ ZnR(fragment)+DO-GTPase2.1→ DO-GTPase1→ GAP1-N1+GAP1-M+GAP1-C*→	676	Sinorhizobium meliloti	GCA_000968555.1	alphaproteobacteria
WP_046463994.1	GAP1-N1+GAP1-M+GAP1-C	Calcineurin+GAPEAD1→ GAP2→ ZnR+DO-GTPase2.1→ DO-GTPase1→ GAP1-N1+GAP1-M+GAP1-C*→	872	Pseudomonas syringae group genomosp. 3	GCA_000980905.1	gammaproteobacteria
WP_046689645.1	GAP1-N1+GAP1-M+GAP1-C	Calcineurin+GAPEAD1→ GAP2→ ZnR+DO-GTPase2.1→ DO-GTPase1→ GAP1-N1+GAP1-M+GAP1-C*→	671	Leptospira santarosai	GCA_000988105.2	spirochaetes
WP_046951455.1	GAP1-N1+GAP1-M+GAP1-C	Calcineurin+GAPEAD1→ GAP2→ ZnR+DO-GTPase2.1→ DO-GTPase1→ DO-GTPase1→ GAP1-N1+GAP1-M+GAP1-C*→	671	Leptospira santarosai	GCA_001981455.1	spirochaetes
WP_047110447.1	GAP1-N1+GAP1-M+GAP1-C+EAD1	PNP_UDP_1+EAD1→ GAP2→ ZnR+DO-GTPase2.1→ DO-GTPase1→ GAP1-N1+GAP1-M+GAP1-C+EAD1*→ SLATT→	807	Vibrio vulnificus	GCA_001013325.1	gammaproteobacteria
WP_047535633.1	GAP1-N1+GAP1-M+GAP1-C+EAD1	GAP2→ ZnR+DO-GTPase2.1→ DO-GTPase1→ GAP1-N1+GAP1-M+GAP1-C+EAD1*→ CASPASE+EAD1→	823	Shewanella sp. ZOR0012	GCF_000798835.1	gammaproteobacteria
WP_048399784.1	GAP1-N1+GAP1-M	-	1007	Candidatus Achromatium palustre	-	gammaproteobacteria
WP_048503675.1	GAP1-N1+GAP1-M+GAP1-C+EAD1	GAP2→ ZnR+DO-GTPase2.1→ DO-GTPase1→ GAP1-N1+GAP1-M+GAP1-C+EAD1*→??+EAD1→ EAD1+Trypsin→	843	Chryseobacterium sp. BLS98	GCA_001045445.1	bacteroidetes
WP_048580951.1	GAP1-N1+GAP1-M+GAP1-C+EAD1	GAP2→ DO-GTPase2.1→ DO-GTPase1→ GAP1-N1+GAP1-M+GAP1-C+EAD1*→ CASPASE+EAD1→	765	Streptomyces viridochromogenes	GCF_001047325.1	actinobacteria
WP_049614825.1	GAP1-N1+GAP1-M+GAP1-C	Calcineurin+GAPEAD1→ GAP2→ DO-GTPase2.1→ DO-GTPase1→ GAP1-N1+GAP1-M+GAP1-C*→	658	Yersinia pekkanenii	GCF_001152565.1	gammaproteobacteria
WP_050085962.1	GAP1-N1+GAP1-M+GAP1-C	Calcineurin+GAPEAD1→ GAP2→ ZnR+DO-GTPase2.1→ DO-GTPase1→ GAP1-N1+GAP1-M+GAP1-C*→	869	Yersinia	GCF_001140765.1	gammaproteobacteria
WP_050103946.1	GAP1-N1+GAP1-M+GAP1-C	GAP2→ ZnR+DO-GTPase2.1→ DO-GTPase1→ GAP1-N1+GAP1-M+GAP1-C*→	869	Yersinia pseudotuberculosis	GCA_001136465.1	gammaproteobacteria
WP_050537229.1	GAP1-N1+GAP1-M+GAP1-C	Calcineurin+GAPEAD1→ GAP2→ DO-GTPase2.1→ DO-GTPase1→ GAP1-N1+GAP1-M+GAP1-C*→	669	Yersinia mollaretii	GCA_001319605.1	gammaproteobacteria
WP_050586283.1	GAP1-N1+GAP1-M+GAP1-C+EAD1	GAP2→ ZnR+DO-GTPase2.1→ DO-GTPase1→ GAP1-N1+GAP1-M+GAP1-C+EAD1*→ CASPASE+EAD1→	745	Pseudomonas syringae	GCF_000452605.1	gammaproteobacteria
WP_050594058.1	GAP1-M+GAP1-C	Calcineurin+GAPEAD1→ GAP2→ ZnR+DO-GTPase2.1→ DO-GTPase1→ GAP1-M+GAP1-C*→	739	Mesorhizobium sp. L2C067A000	GCF_000502855.1	alphaproteobacteria
WP_050642156.1	GAP1-N1+GAP1-M+GAP1-C	Calcineurin+GAPEAD1→ GAP2→ DO-GTPase2.1→ DO-GTPase1→ GAP1-N1+GAP1-M+GAP1-C*→	644	Eubacterium sp. SB2	GCF_001244405.1	firmicutes
WP_052246573.1	GAP1-N1+GAP1-M+GAP1-C	Calcineurin+GAPEAD1→ GAP2→ ZnR+DO-GTPase2.1→ DO-GTPase1→ GAP1-N1+GAP1-M+GAP1-C*→	785	Chromobacterium piscinae	GCF_000812585.1	betaproteobacteria
WP_052516912.1	GAP1-N1+GAP1-M+GAP1-C	Calcineurin+GAPEAD1→ GAP2→ ZnR+DO-GTPase2.1→ DO-GTPase1→ GAP1-N1+GAP1-M+GAP1-C*→	890	Methylobacterium radiotolerans	GCA_002221455.1	alphaproteobacteria
WP_052820524.1	GAP1-N1+GAP1-M+GAP1-C	Calcineurin+GAPEAD1→ GAP2→ ZnR+DO-GTPase2.1→ DO-GTPase1→ GAP1-N1+GAP1-M+GAP1-C*→	878	Agrobacterium sp. SUL3	GCA_001263295.1	alphaproteobacteria
WP_053349796.1	GAP1-N1+GAP1-M+GAP1-C+EAD1	PNP_UDP_1+EAD1→ GAP2→ ZnR(fragment)+DO-GTPase2.1→ DO-GTPase1→ GAP1-N1+GAP1-M+GAP1-C+EAD1*→ SLATT→	809	Vibrio alginolyticus	GCF_001273715.1	gammaproteobacteria
WP_053486428.1	GAP1-N1+GAP1-M+GAP1-C+EAD1	GAP2→ DO-GTPase2.1→ DO-GTPase1→ GAP1-N1+GAP1-M+GAP1-C+EAD1*→ CASPASE+EAD1→	826	Pseudomonas coronafaciens	GCA_001275735.1	gammaproteobacteria
WP_053572419.1	GAP1-N1+GAP1-M+GAP1-C	Calcineurin+GAPEAD1→ GAP2→ ZnR+DO-GTPase2.1→ DO-GTPase1→ GAP1-N1+GAP1-M+GAP1-C*→	698	Caballeronia cordobensis	GCF_001278535.1	betaproteobacteria
WP_053856890.1	GAP1-N1+GAP1-M+GAP1-C+EAD1	GAP2→ DO-GTPase2.1→ PSE→ GAP1-N1+GAP1-M+GAP1-C+EAD1*→ CASPASE+EAD1→	809	Halomonas	GCA_900099955.1	gammaproteobacteria
WP_055335602.1	GAP1-N1+GAP1-M+GAP1-C	Calcineurin+GAPEAD1→ GAP2→ ZnR+DO-GTPase2.1→ DO-GTPase1→ GAP1-N1+GAP1-M+GAP1-C*→	871	Ralstonia solanacearum	GCF_001373335.1	betaproteobacteria
WP_056213067.1	GAP1-N1+GAP1-M+GAP1-C	GAP1-N1+GAP1-M+GAP1-C*→	487	Methylobacterium sp. Leaf94	GCF_001426045.1	alphaproteobacteria
WP_056265586.1	GAP1-N1+GAP1-M+GAP1-C+EAD1	GAP1-N1+GAP1-M+GAP1-C+EAD1*→ CASPASE+EAD1→	750	Methylobacterium sp. Leaf87	GCA_001422775.1	alphaproteobacteria
WP_056276317.1	GAP1-N1+GAP1-M+GAP1-C	Calcineurin+GAPEAD1→ GAP2→ ZnR+DO-GTPase2.1→ PSE→ GAP1-N1+GAP1-M+GAP1-C*→	686	Hydrogenophaga sp. Root209	GCA_001428625.1	betaproteobacteria
WP_056690820.1	GAP1-N1+GAP1-M+GAP1-C+EAD1	GAP2→ ZnR+DO-GTPase2.1→ DO-GTPase1→ GAP1-N1+GAP1-M+GAP1-C+EAD1*→ CASPASE+EAD1→	824	Sphingobium sp. Leaf26	GCA_001421665.1	alphaproteobacteria
WP_057298085.1	GAP1-N1+GAP1-M+GAP1-C	Calcineurin+GAPEAD1→ GAP2→ ZnR+DO-GTPase2.1→ DO-GTPase1→ GAP1-N1+GAP1-M+GAP1-C*→	762	Pelomonas sp. Root1217	GCF_001425705.1	betaproteobacteria
WP_057416337.1	GAP1-N1+GAP1-M+GAP1-C+EAD1	GAP2→ DO-GTPase2.1→ DO-GTPase1→ GAP1-N1+GAP1-M+GAP1-C+EAD1*→ CASPASE+EAD1→	826	Pseudomonas syringae group genomosp. 3	GCA_001401405.1	gammaproteobacteria
WP_057443670.1	GAP1-N1+GAP1-M+GAP1-C	Calcineurin+GAPEAD1→ GAP2→ ZnR+DO-GTPase2.1→ DO-GTPase1→ GAP1-N1+GAP1-M+GAP1-C*→	876	Pseudomonas amygdali	GCA_001401265.1	gammaproteobacteria
WP_057505037.1	GAP1-N1+GAP1-M+GAP1-C	Calcineurin+GAPEAD1→ GAP2→ DO-GTPase2.1→ DO-GTPase1→ GAP1-N1+GAP1-M+GAP1-C*→	871	Stenotrophomonas nitritireducens	GCA_001431425.1	gammaproteobacteria
WP_058029521.1	GAP1-N1+GAP1-M+GAP1-C+EAD1	PNP_UDP_1+EAD1→ GAP2→ ZnR(fragment)+DO-GTPase2.1→ DO-GTPase1→ GAP1-N1+GAP1-M+GAP1-C+EAD1*→ SLATT→	809	Pseudoalteromonas phenolica	GCA_001444405.1	gammaproteobacteria
WP_058248883.1	GAP1-N1+GAP1-M+GAP1-C+EAD1	GAP2→ ZnR+DO-GTPase2.1→ DO-GTPase1→ GAP1-N1+GAP1-M+GAP1-C+EAD1*→ CASPASE+EAD1→	835	Tropicibacter naphthalenivorans	GCA_001458375.1	alphaproteobacteria
WP_058410945.1	GAP1-N1+GAP1-M+GAP1-C	Calcineurin+GAPEAD1→ GAP2→ ZnR+DO-GTPase2.1→ DO-GTPase1→ GAP1-N1+GAP1-M+GAP1-C*→	674	Pseudomonas	GCA_001467145.1	gammaproteobacteria
WP_058428192.1	GAP1-N1+GAP1-M+GAP1-C+EAD1	GAP2→ DO-GTPase2.1→ DO-GTPase1→ GAP1-N1+GAP1-M+GAP1-C+EAD1*→ CASPASE+EAD1→	834	Pseudomonas syringae	GCA_001467105.1	gammaproteobacteria
WP_058819802.1	GAP1-N1+GAP1-M+GAP1-C	Calcineurin+GAPEAD1→ GAP2→ ZnR+DO-GTPase2.1→ DO-GTPase1→ GAP1-N1+GAP1-M+GAP1-C*→	629	Salmonella enterica	GCA_004219785.1	gammaproteobacteria
WP_059570022.1	GAP1-N1+GAP1-M+GAP1-C	Calcineurin+GAPEAD1→ GAP2→ DO-GTPase2.1→ DO-GTPase1→ GAP1-N1+GAP1-M+GAP1-C*→	643	Burkholderia sp. TSV86	GCA_001522865.1	betaproteobacteria
WP_059729175.1	GAP1-N1+GAP1-M+GAP1-C	Calcineurin+GAPEAD1→ GAP2→ ZnR+DO-GTPase2.1→ DO-GTPase1→ GAP1-N1+GAP1-M+GAP1-C*→	880	Burkholderia ubonensis	GCA_001522035.1	betaproteobacteria
WP_059733060.1	GAP1-N1+GAP1-M+GAP1-C	Calcineurin+GAPEAD1→ GAP2→ ZnR+DO-GTPase2.1→ DO-GTPase1→ GAP1-N1+GAP1-M+GAP1-C*→	876	Burkholderia cepacia	GCA_001526205.1	betaproteobacteria

acc	architecture	operon	len	species	gca	taxend
WP_059864452.1	GAP1-N1+GAP1-M+GAP1-C	Calcineurin+GAPEAD1→ GAP2→ ZnR+DO-GTPase2.1→ DO-GTPase1→ GAP1-N1+GAP1-M+GAP1-C*→	876	Burkholderia ubonensis	GCA_001528175.1	betaproteobacteria
WP_059931657.1	GAP1-N1+GAP1-M+GAP1-C	Calcineurin+GAPEAD1→ GAP2→ ZnR+DO-GTPase2.1→ DO-GTPase1→ GAP1-N1+GAP1-M+GAP1-C*→	864	Burkholderia ubonensis	GCF_001528875.1	betaproteobacteria
WP_060016453.1	GAP1-N1+GAP1-M+GAP1-C	Calcineurin+GAPEAD1→ GAP2→ ZnR+DO-GTPase2.1→ DO-GTPase1→ GAP1-N1+GAP1-M+GAP1-C*→	876	Burkholderia stagnalis	GCA_001527825.1	betaproteobacteria
WP_060087567.1	GAP1-M+GAP1-C	Calcineurin+GAPEAD1→||←?||DO-GTPase2.1→ DO-GTPase1→ GAP1-M+GAP1-C*→	792	Burkholderia ubonensis	GCA_001529865.1	betaproteobacteria
WP_060227644.1	GAP1-N1+GAP1-M+GAP1-C+EAD1	GAP2→ DO-GTPase2.1→ DO-GTPase1→ GAP1-N1+GAP1-M+GAP1-C+EAD1*→ CASPASE+EAD1→	827	Burkholderia ubonensis	GCA_001533325.1	betaproteobacteria
WP_060275707.1	GAP1-N1+GAP1-M+GAP1-C	Calcineurin+GAPEAD1→ GAP2→ ZnR+DO-GTPase2.1→ DO-GTPase1→ GAP1-N1+GAP1-M+GAP1-C*→	871	Burkholderia ubonensis	GCA_001532865.1	betaproteobacteria
WP_060408983.1	GAP1-M+GAP1-C	Calcineurin+GAPEAD1→ GAP2→ ZnR+DO-GTPase2.1→ DO-GTPase1→ GAP1-M+GAP1-C*→	786	Pseudomonas syringae	GCA_001535945.1	gammaproteobacteria
WP_060854171.1	GAP1-N1+GAP1-M+GAP1-C	Calcineurin+GAPEAD1→ GAP2→ ZnR+DO-GTPase2.1→ DO-GTPase1→ GAP1-N1+GAP1-M+GAP1-C*→	700	Caballeronia cordobensis	GCA_001544575.2	betaproteobacteria
WP_061209303.1	GAP1-N1+GAP1-M+GAP1-C	Calcineurin+GAPEAD1→ GAP2→ ZnR+DO-GTPase2.1→ DO-GTPase1→ GAP1-N1+GAP1-M+GAP1-C*→	672	Leptospira borgpetersenii	GCF_001568445.1	spirochaetes
WP_061218562.1	GAP1-N1+GAP1-M+GAP1-C	Calcineurin+GAPEAD1→ GAP2→ ZnR+DO-GTPase2.1→ DO-GTPase1→ GAP1-N1+GAP1-M+GAP1-C*→	671	Leptospira weilii	GCF_001569365.1	spirochaetes
WP_061908297.1	GAP1-N1+GAP1-M+GAP1-C+EAD1	GAP2→ ZnR+DO-GTPase2.1→ DO-GTPase1→ GAP1-N1+GAP1-M+GAP1-C+EAD1*→ CASPASE+EAD1→ CASPASE+??→	814	Devosia sp. Leaf420	GCA_001425445.1	alphaproteobacteria
WP_062959907.1	GAP1-N1+GAP1-M+GAP1-C	Calcineurin+GAPEAD1→ GAP2→ DO-GTPase2.1(fragment)→ DO-GTPase1→ GAP1-N1+GAP1-M+GAP1-C*→	601	Thalassospira xiamenensis	GCA_003326775.1	alphaproteobacteria
WP_063151208.1	GAP1-N1+GAP1-M+GAP1-C	Calcineurin+GAPEAD1→ GAP2→ ZnR+DO-GTPase2.1→ DO-GTPase1→ GAP1-N1+GAP1-M+GAP1-C*→	871	Enterobacteriaceae	GCF_900075065.1	gammaproteobacteria
WP_064170057.1	GAP1-N1+GAP1-M+GAP1-C	Calcineurin+GAPEAD1→ GAP2→ ZnR+DO-GTPase2.1→ DO-GTPase1→ GAP1-N1+GAP1-M+GAP1-C*→	869	Klebsiella	GCA_002152295.1	gammaproteobacteria
WP_064791174.1	GAP1-N1+GAP1-M+GAP1-C	Calcineurin+GAPEAD1→ GAP2→ ZnR+DO-GTPase2.1→ DO-GTPase1→ GAP1-N1+GAP1-M+GAP1-C*→	871	Enterobacter asburiae	GCA_001663525.1	gammaproteobacteria
WP_064992291.1	GAP1-N1+GAP1-M+GAP1-C	Calcineurin+GAPEAD1→ GAP2→ ZnR+DO-GTPase2.1→ DO-GTPase1→ GAP1-N1+GAP1-M+GAP1-C*→	674	Mesorhizobium loti	GCA_001672365.1	alphaproteobacteria
WP_065204322.1	GAP1-N1+GAP1-M+GAP1-C+EAD1	PNP_UDP_1+EAD1→ GAP2→ ZnR(fragment)+DO-GTPase2.1→ DO-GTPase1→ GAP1-N1+GAP1-M+GAP1-C+EAD1*→ SLATT→	809	Shewanella sp. Alg231_23	GCF_900079515.1	gammaproteobacteria
WP_065832748.1	GAP1-N1+GAP1-M+GAP1-C+EAD1	GAP2→ DO-GTPase2.1→ DO-GTPase1→ GAP1-N1+GAP1-M+GAP1-C+EAD1*→ CASPASE+EAD1→	828	Pseudomonas syringae	GCA_001698815.1	gammaproteobacteria
WP_066124811.1	GAP1-N1+GAP1-M+GAP1-C	Calcineurin+GAPEAD1→ GAP2→ ZnR+DO-GTPase2.1→ DO-GTPase1→ GAP1-N1+GAP1-M+GAP1-C*→	875	Bordetella ansorpii	GCA_900078705.1	betaproteobacteria
WP_066213797.1	GAP1-N1+GAP1-M+GAP1-C	Calcineurin+GAPEAD1→ GAP2→ ZnR+DO-GTPase2.1→ DO-GTPase1→ GAP1-N1+GAP1-M+GAP1-C*→	880	Blastomonas sp. CCH5-A3	GCF_001556295.1	alphaproteobacteria
WP_066223696.1	GAP1-N1+GAP1-M+GAP1-C	GAP2→ PSE→ PSE→ GAP1-N1+GAP1-M+GAP1-C*→ EAD1→?→??+EAD1→ EAD1+Trypsin→	344	Formosa haliotis	GCF_001685485.1	bacteroidetes
WP_066271148.1	GAP1-N1+GAP1-M+GAP1-C+EAD1	GAP2→ DO-GTPase2.1→ DO-GTPase1→ GAP1-N1+GAP1-M+GAP1-C+EAD1*→ CASPASE+EAD1→	800	Hydrogenophaga palleronii	GCF_001571225.1	betaproteobacteria
WP_066306413.1	GAP1-N1+GAP1-M+GAP1-C	PNP_UDP_1+GAPEAD1→ GAP2→ ZnR+DO-GTPase2.1→ DO-GTPase1→ GAP1-N1+GAP1-M+GAP1-C*→	693	Flavobacterium sp. TAB 87	GCA_001529295.1	bacteroidetes
WP_066533121.1	GAP1-N1+GAP1-M+GAP1-C+EAD1	GAP2→ ZnR(fragment)+DO-GTPase2.1→ DO-GTPase1→ GAP1-N1+GAP1-M+GAP1-C+EAD1*→ CASPASE+EAD1→	822	Comamonas terrigena	-	betaproteobacteria
WP_066769098.1	GAP1-N1+GAP1-M+GAP1-C	Calcineurin+GAPEAD1→ GAP2→ ZnR+DO-GTPase2.1→ DO-GTPase1→ GAP1-N1+GAP1-M+GAP1-C*→	887	Croceicoccus mobilis	GCF_001661965.1	alphaproteobacteria
WP_069091270.1	GAP1-N1+GAP1-M+GAP1-C	GAP2→ ZnR+DO-GTPase2.1→ DO-GTPase1→ GAP1-N1+GAP1-M+GAP1-C*→	880	Mesorhizobium sp. SEMIA 3007	GCA_001708695.1	alphaproteobacteria
WP_069212636.1	GAP1-N1+GAP1-M+GAP1-C+EAD1	PNP_UDP_1+EAD1→ GAP2→ ZnR(fragment)+DO-GTPase2.1→ DO-GTPase1→ GAP1-N1+GAP1-M+GAP1-C+EAD1*→ SLATT→	809	Vibrio cholerae	-	gammaproteobacteria
WP_069548605.1	GAP1-N1+GAP1-M+GAP1-C+EAD1	PNP_UDP_1+EAD1→ GAP2→ ZnR+DO-GTPase2.1→ DO-GTPase1→ GAP1-N1+GAP1-M+GAP1-C+EAD1*→ SLATT→	807	Vibrio parahaemolyticus	GCA_001727475.1	gammaproteobacteria
WP_072408563.1	GAP1-N1+GAP1-M+GAP1-C+EAD1	GAP2→ ZnR+DO-GTPase2.1→ DO-GTPase1→ GAP1-N1+GAP1-M+GAP1-C+EAD1*→ PNP_UDP_1+EAD1→	826	Chryseobacterium limigenitum	GCA_900114875.1	bacteroidetes
WP_074276944.1	GAP1-N1+GAP1-M+GAP1-C	Calcineurin+GAPEAD1→ GAP2→ DO-GTPase2.1→ DO-GTPase1→ GAP1-N1+GAP1-M+GAP1-C*→	649	Bradyrhizobium erythrophlei	GCA_900129675.1	alphaproteobacteria
WP_074277581.1	GAP1-M+GAP1-C	GAP1-M+GAP1-C*→	522	Bradyrhizobium erythrophlei	GCA_900129675.1	alphaproteobacteria
WP_074304590.1	GAP1-N1+GAP1-M+GAP1-C	Calcineurin+GAPEAD1→ GAP2→ DO-GTPase2.1→ DO-GTPase1→ GAP1-N1+GAP1-M+GAP1-C*→	917	Singulisphaera sp. GP187	GCA_900129635.1	planctomycetes
WP_074658816.1	GAP1-N1+GAP1-M+GAP1-C+EAD1	GAP2→ ZnR+DO-GTPase2.1→ DO-GTPase1→ GAP1-N1+GAP1-M+GAP1-C+EAD1*→ PNP_UDP_1+EAD1→	829	Flavobacterium	GCA_002217275.1	bacteroidetes
WP_074907605.1	GAP1-N1+GAP1-M+GAP1-C+EAD1	GAP2→ ZnR+DO-GTPase2.1→ DO-GTPase1→ GAP1-N1+GAP1-M+GAP1-C+EAD1*→ CASPASE+EAD1→	829	Pseudomonas syringae	GCA_900115275.1	gammaproteobacteria
WP_075907279.1	GAP1-N1+GAP1-M+GAP1-C+EAD1	GAP2→ DO-GTPase2.1→ DO-GTPase1→ GAP1-N1+GAP1-M+GAP1-C+EAD1*→ CASPASE+EAD1→	824	Actinomadura sp. CNU-125	GCA_001942465.1	actinobacteria
WP_075979084.1	GAP1-N1+GAP1-M+GAP1-C	Calcineurin+GAPEAD1→ GAP2→ ZnR+DO-GTPase2.1→ DO-GTPase1→ DO-GTPase1→ GAP1-N1+GAP1-M+GAP1-C*→	215	Leptospira santarosai	GCA_001952675.1	spirochaetes
WP_076403769.1	GAP1-N1+GAP1-M+GAP1-C	Calcineurin+GAPEAD1→ GAP2→ ZnR+DO-GTPase2.1→ DO-GTPase1→ GAP1-N1+GAP1-M+GAP1-C*→	867	Pseudomonas putida	GCF_900156185.1	gammaproteobacteria
WP_076658804.1	GAP1-N1+GAP1-M+GAP1-C	Calcineurin+GAPEAD1→ GAP2→ ZnR+DO-GTPase2.1→ DO-GTPase1→ DO-GTPase1→ GAP1-N1+GAP1-M+GAP1-C*→	217	Leptospira santarosai	GCA_001981425.1	spirochaetes
WP_077839733.1	GAP1-N1+GAP1-M+GAP1-C	Calcineurin+GAPEAD1→ GAP2→ DO-GTPase2.1→ DO-GTPase1→ GAP1-N1+GAP1-M+GAP1-C*→	637	Clostridium beijerinckii	GCA_002006205.1	firmicutes
WP_078010983.1	GAP1-N1+GAP1-M+GAP1-C+PNP_UDP_1+EAD1	GAP2→ DO-GTPase2.1→ DO-GTPase1→ DO-GTPase1→ GAP1-N1+GAP1-M+GAP1-C+PNP_UDP_1+EAD1*→	1142	Hymenobacter sp. CRA2	GCA_002007105.1	bacteroidetes
WP_078130559.1	GAP1-N1+GAP1-M+GAP1-C+EAD1	GAP2→ ZnR+DO-GTPase2.1→ DO-GTPase1→ GAP1-N1+GAP1-M+GAP1-C+EAD1*→ PNP_UDP_1+EAD1→	845	Leptospira alexanderi	GCF_002009845.1	spirochaetes
WP_078754774.1	GAP1-N1+GAP1-M+GAP1-C	GAP1-N1+GAP1-M+GAP1-C*→	606	Enterovibrio nigricans	GCA_002835505.1	gammaproteobacteria
WP_078789132.1	GAP1-N1+GAP1-M+GAP1-C+EAD1	??+PNP_UDP_1+EAD1→ GAP2→ ZnR+DO-GTPase2.1→ DO-GTPase1→ GAP1-N1+GAP1-M+GAP1-C+EAD1*→	815	Geobacter thiogenes	GCA_900167465.1	deltaproteobacteria
WP_079392583.1	GAP1-N1+GAP1-M+GAP1-C+EAD1	GAP2→ DO-GTPase2.1→ DO-GTPase1→ GAP1-N1+GAP1-M+GAP1-C+EAD1*→ CASPASE+EAD1→	832	Pseudomonas	GCA_002326205.1	gammaproteobacteria
WP_080161065.1	GAP1-N1+GAP1-M+GAP1-C	Calcineurin+GAPEAD1→ GAP2→ ZnR+DO-GTPase2.1→ DO-GTPase1→ GAP1-N1+GAP1-M+GAP1-C*→	629	Salmonella enterica	GCA_004271805.1	gammaproteobacteria
WP_080291534.1	GAP1-N1+GAP1-M+GAP1-C	Calcineurin+GAPEAD1→ GAP2→ ZnR+DO-GTPase2.1→ DO-GTPase1→ GAP1-N1+GAP1-M+GAP1-C*→	667	Burkholderia pseudomallei	GCA_003606245.1	betaproteobacteria
WP_080323218.1	GAP1-N1+GAP1-M+GAP1-C	Calcineurin+GAPEAD1→ GAP2→ ZnR+DO-GTPase2.1→ DO-GTPase1→ GAP1-N1+GAP1-M+GAP1-C*→	872	Burkholderia cenocepacia	GCA_002071875.1	betaproteobacteria
WP_080324388.1	GAP1-N1+GAP1-M+GAP1-C+EAD1	GAP2→ DO-GTPase2.1→ DO-GTPase1→ GAP1-N1+GAP1-M+GAP1-C+EAD1*→ CASPASE+EAD1→	829	Burkholderia cenocepacia	GCA_002071905.1	betaproteobacteria
WP_080377390.1	GAP1-N1+GAP1-M+GAP1-C	Calcineurin+GAPEAD1→ GAP2→ DO-GTPase2.1→ DO-GTPase1→ GAP1-N1+GAP1-M+GAP1-C*→	650	Pseudomonas syringae	GCA_001293665.1	gammaproteobacteria
WP_080406895.1	GAP1-N1+GAP1-M+GAP1-C	Calcineurin+GAPEAD1→ GAP2→ ZnR+DO-GTPase2.1→ DO-GTPase1→ GAP1-N1+GAP1-M+GAP1-C*→	667	Burkholderia ubonensis	GCF_001526225.1	betaproteobacteria
WP_080565882.1	GAP1-N1+GAP1-M+GAP1-C+EAD1	GAP2→ DO-GTPase2.1→ DO-GTPase1→ GAP1-N1+GAP1-M+GAP1-C+EAD1*→ CASPASE+EAD1→	828	Xanthomonas campestris	GCF_000221965.1	gammaproteobacteria
WP_080902476.1	GAP1-N1+GAP1-M+GAP1-C+EAD1	GAP2→ DO-GTPase2.1→ DO-GTPase1→ GAP1-N1+GAP1-M+GAP1-C+EAD1*→ CASPASE+EAD1→	834	Pseudomonas fluorescens	GCF_000834545.1	gammaproteobacteria

acc	architecture	operon	len	species	gca	taxend
WP_081333857.1	GAP1-N1+GAP1-M+GAP1-C+CASPASE	GAP2→||←?||GAP1-N1+GAP1-M+GAP1-C+CASPASE*→	921	Burkholderia stagnalis	GCF_001718955.1	betaproteobacteria
WP_081480398.1	GAP1-N1+GAP1-M+GAP1-C	Calcineurin+GAPEAD1→ GAP2→ ZnR+DO-GTPase2.1→ DO-GTPase1→ GAP1-N1+GAP1-M+GAP1-C*→	879	Pseudomonas	GCA_003001535.1	gammaproteobacteria
WP_084435545.1	GAP1-N1+GAP1-M+GAP1-C+EAD1	GAP2→ ZnR+DO-GTPase2.1→ DO-GTPase1→ GAP1-N1+GAP1-M+GAP1-C+EAD1*→ CASPASE+EAD1→	753	Sinorhizobium saheli	GCF_001651875.1	alphaproteobacteria
WP_085211717.1	GAP1-N1+GAP1-M+GAP1-C+EAD1	PNP_UDP_1+EAD1→||←?||GAP2→ ZnR+DO-GTPase2.1→ DO-GTPase1→ GAP1-N1+GAP1-M+GAP1-C+EAD1*→	825	Methylomagnum ishizawai	GCA_900155475.1	gammaproteobacteria
WP_085423962.1	GAP1-N1+GAP1-M+GAP1-C	Calcineurin+GAPEAD1→ GAP2→ DO-GTPase2.1→ DO-GTPase1→ GAP1-N1+GAP1-M+GAP1-C*→	644	Rhizobium oryzae	GCA_900177415.1	alphaproteobacteria
WP_085946283.1	GAP1-N1+GAP1-M+GAP1-C	Calcineurin+GAPEAD1→ GAP2→ PSE→ DO-GTPase1→ GAP1-N1+GAP1-M+GAP1-C*→	649	Aeromonas salmonicida	-	gammaproteobacteria
WP_086054884.1	GAP1-N1+GAP1-M+GAP1-C	Calcineurin+GAPEAD1→ GAP2→ DO-GTPase2.1(fragment)→ DO-GTPase1→ GAP1-N1+GAP1-M+GAP1-C*→	653	Pseudoruegeria sp. SK021	GCA_002119405.1	alphaproteobacteria
WP_086156276.1	GAP1-N1+GAP1-M+GAP1-C	Calcineurin+GAPEAD1→ GAP2→ DO-GTPase2.1→ DO-GTPase1→ GAP1-N1+GAP1-M+GAP1-C*→	641	Rhizobium sp. Kim5	GCA_000172695.2	alphaproteobacteria
WP_086321643.1	GAP1-N1+GAP1-M+GAP1-C+EAD1	PNP_UDP_1+EAD1→ GAP2→ ZnR+DO-GTPase2.1→ DO-GTPase1→ GAP1-N1+GAP1-M+GAP1-C+EAD1*→	798	Acinetobacter baumannii	GCA_002143185.1	gammaproteobacteria
WP_088100321.1	GAP1-N1+GAP1-M+GAP1-C+EAD1	GAP2→ DO-GTPase2.1→ DO-GTPase1→ GAP1-N1+GAP1-M+GAP1-C+EAD1*→ CASPASE+EAD1→	822	Xanthomonas retroflexus	GCF_900143175.1	gammaproteobacteria
WP_089766267.1	GAP1-N1+GAP1-M+GAP1-C	TIR+GAPEAD1→ GAP2→ ZnR+DO-GTPase2.1→ DO-GTPase1→ GAP1-N1+GAP1-M+GAP1-C*→	720	Chitinophaga terrae Kim and Jung 2007	GCA_900107795.1	bacteroidetes
WP_090271101.1	GAP1-M+GAP1-C	Calcineurin+GAPEAD1→ GAP2→ DO-GTPase2.1(fragment)→ DO-GTPase1→ GAP1-M+GAP1-C*→	542	Litorimicrobium taeanense	-	alphaproteobacteria
WP_090309635.1	GAP1-N1+GAP1-M+GAP1-C+EAD1	GAP2→ DO-GTPase2.1→ DO-GTPase1→ GAP1-N1+GAP1-M+GAP1-C+EAD1*→ CASPASE+EAD1→	829	Pseudomonas linyingensis	GCA_900109175.1	gammaproteobacteria
WP_090943024.1	GAP1-N1+GAP1-M+GAP1-C	Calcineurin+GAPEAD1→ GAP2→ DO-GTPase2.1→ DO-GTPase1→ GAP1-N1+GAP1-M+GAP1-C*→	637	Pelosinus propionicus	GCA_900114615.1	firmicutes
WP_090964306.1	GAP1-N1+GAP1-M+GAP1-C+EAD1	GAP1-N1+GAP1-M+GAP1-C+EAD1*→ CASPASE+EAD1→	748	Aureimonas phyllosphaerae	GCA_900113065.1	alphaproteobacteria
WP_091457793.1	GAP1-N1+GAP1-M+GAP1-C+EAD1	GAP2→ DO-GTPase2.1→ DO-GTPase1→ GAP1-N1+GAP1-M+GAP1-C+EAD1*→ CASPASE+EAD1→	822	Giesbergeria anulus	GCF_900111115.1	betaproteobacteria
WP_091458865.1	GAP1-N1+GAP1-M+GAP1-C	Calcineurin+GAPEAD1→ GAP2→ ZnR+DO-GTPase2.1→ DO-GTPase1→ GAP1-N1+GAP1-M+GAP1-C*→	681	Giesbergeria anulus	GCA_900111115.1	betaproteobacteria
WP_091694811.1	GAP1-N1+GAP1-M+GAP1-C+EAD1	GAP2→ ZnR+DO-GTPase2.1→ DO-GTPase1→ GAP1-N1+GAP1-M+GAP1-C+EAD1*→ EAD1+Trypsin→?→??+EAD1→	844	Algoriphagus locisalis	GCA_900116615.1	bacteroidetes
WP_091713433.1	GAP1-N1+GAP1-M+GAP1-C	Calcineurin+GAPEAD1→ GAP2→ ZnR+DO-GTPase2.1→ DO-GTPase1→ GAP1-N1+GAP1-M+GAP1-C*→	682	Methylobacterium phyllostachyos	GCA_900103445.1	alphaproteobacteria
WP_091720743.1	GAP1-N1+GAP1-M+GAP1-C	Calcineurin+GAPEAD1→ GAP2→ ZnR+DO-GTPase2.1→ DO-GTPase1→ GAP1-N1+GAP1-M+GAP1-C*→	689	Mitsuaria sp. PDC51	GCA_900113225.1	betaproteobacteria
WP_092089286.1	GAP1-N1+GAP1-M+GAP1-C	Calcineurin+GAPEAD1→ GAP2→ ZnR+DO-GTPase2.1→ DO-GTPase1→ GAP1-N1+GAP1-M+GAP1-C*→	564	Bradyrhizobium sp. R5	GCF_900101405.1	alphaproteobacteria
WP_092194891.1	GAP1-N1+GAP1-M+GAP1-C	Calcineurin+GAPEAD1→ GAP2→ DO-GTPase2.1→ DO-GTPase1→ GAP1-N1+GAP1-M+GAP1-C*→	699	Blastococcus sp. DSM 46838	-	actinobacteria
WP_092438108.1	GAP1-N1+GAP1-M+GAP1-C+EAD1	GAP2→ ZnR+DO-GTPase2.1→ DO-GTPase1→ GAP1-N1+GAP1-M+GAP1-C+EAD1*→ CASPASE+EAD1→	823	Collimonas sp. OK607	GCA_900111995.1	betaproteobacteria
WP_092579008.1	GAP1-N1+GAP1-M+GAP1-C	Calcineurin+GAPEAD1→ GAP2→ ZnR+DO-GTPase2.1→ DO-GTPase1→ GAP1-N1+GAP1-M+GAP1-C*→	876	Achromobacter sp. NFACC18-2	GCA_900108985.1	betaproteobacteria
WP_092699236.1	GAP1-N1+GAP1-M+GAP1-C	Calcineurin+GAPEAD1→ GAP2→ ZnR(fragment)+DO-GTPase2.1→ DO-GTPase1→ GAP1-N1+GAP1-M+GAP1-C*→	664	Acidovorax soli	-	betaproteobacteria
WP_092700910.1	GAP1-N1+GAP1-M+GAP1-C	Calcineurin+GAPEAD1→ GAP2→ DO-GTPase2.1→ DO-GTPase1→ GAP1-N1+GAP1-M+GAP1-C*→	871	Rhodanobacter glycinis	GCA_900114325.1	gammaproteobacteria
WP_093103449.1	GAP1-N1+GAP1-M+GAP1-C	Calcineurin+GAPEAD1→ GAP2→ ZnR+DO-GTPase2.1→ DO-GTPase1→ GAP1-N1+GAP1-M+GAP1-C*→	880	Variovorax sp. CF079	GCF_900101545.1	betaproteobacteria
WP_093387274.1	GAP1-N1+GAP1-M+GAP1-C	Calcineurin+GAPEAD1→ GAP2→ ZnR+DO-GTPase2.1→ DO-GTPase1→ GAP1-N1+GAP1-M+GAP1-C*→	867	Pseudomonas sp. UC 17F4	GCF_900101695.1	gammaproteobacteria
WP_093401103.1	GAP1-N1+GAP1-M+GAP1-C	GAP1-N1+GAP1-M+GAP1-C*→	681	Sphingomonas sp. OK281	GCA_900115295.1	alphaproteobacteria
WP_095649279.1	GAP1-N1+GAP1-M+GAP1-C	Calcineurin+GAPEAD1→ GAP2→ ZnR+DO-GTPase2.1→ DO-GTPase1→ GAP1-N1+GAP1-M+GAP1-C*→	871	Pseudomonas indica	GCA_002286725.1	gammaproteobacteria
WP_096141060.1	GAP1-N1+GAP1-M+GAP1-C+EAD1	GAP1-N1+GAP1-M+GAP1-C+EAD1*→ SLATT→	735	Shewanella algae	GCA_002318995.1	gammaproteobacteria
WP_096425621.1	GAP1-N1+GAP1-M+GAP1-C+EAD1	GAP2→ DO-GTPase2.1→ DO-GTPase1→ GAP1-N1+GAP1-M+GAP1-C+EAD1*→ CASPASE+EAD1→	827	Pseudomonas putida	GCA_002356095.1	gammaproteobacteria
WP_097522738.1	GAP1-N1+GAP1-M+GAP1-C	Calcineurin+GAPEAD1→ GAP2→ DO-GTPase2.1→ DO-GTPase1→ GAP1-N1+GAP1-M+GAP1-C*→	674	Sinorhizobium sp. BJ1	GCA_002531555.1	alphaproteobacteria
WP_097608923.1	GAP1-N1+GAP1-M+GAP1-C	Calcineurin+GAPEAD1→ GAP2→ DO-GTPase2.1→ PSE→ GAP1-N1+GAP1-M+GAP1-C*→	682	Rhizobium sp. M10	GCA_002531955.1	alphaproteobacteria
WP_099207057.1	GAP1-N1+GAP1-M+GAP1-C	Calcineurin+GAPEAD1→ GAP2→ GAP2→ GAP2→ ZnR+DO-GTPase2.1→ DO-GTPase1→ GAP1-N1+GAP1-M+GAP1-C*→	681	Xanthomonas citri	GCF_002688625.1	gammaproteobacteria
WP_099315960.1	GAP1-M+GAP1-C	Calcineurin+GAPEAD1→ GAP2→ DO-GTPase2.1(fragment)→ DO-GTPase1→ GAP1-M+GAP1-C*→	521	Burkholderia sp. AU18528	GCA_002223215.2	betaproteobacteria
WP_099380024.1	GAP1-N1+GAP1-M+GAP1-C+EAD1	GAP1-N1+GAP1-M+GAP1-C+EAD1*→ CASPASE+EAD1→	748	Janthinobacterium sp. BJB426	GCF_002735705.1	betaproteobacteria
WP_099407274.1	GAP1-N1+GAP1-M+GAP1-C	Calcineurin+GAPEAD1→ GAP2→ ZnR+DO-GTPase2.1→ DO-GTPase1→ GAP1-N1+GAP1-M+GAP1-C*→	655	Chitinimonas sp. BJB300	GCF_002735695.1	betaproteobacteria
WP_099766932.1	GAP1-N1+GAP1-M+GAP1-C+EAD1	GAP2→ ZnR+DO-GTPase2.1→ DO-GTPase1→ GAP1-N1+GAP1-M+GAP1-C+EAD1*→??+EAD1→ EAD1+Trypsin→	843	Chryseobacterium sp. 52	GCA_002754245.1	bacteroidetes
WP_100195995.1	GAP1-N1+GAP1-M+GAP1-C	AB_hydrolase+GAPEAD1→ GAP2→ ZnR+DO-GTPase2.1→ DO-GTPase1→ GAP1-N1+GAP1-M+GAP1-C*→	681	Pseudorhodobacter sp. MZDSW-24AT	GCA_002794355.1	alphaproteobacteria
WP_100278437.1	GAP1-N1+GAP1-M+GAP1-C+EAD1	GAP2→ ZnR(fragment)+DO-GTPase2.1→ DO-GTPase1→ GAP1-N1+GAP1-M+GAP1-C+EAD1*→ PNP_UDP_1+EAD1→	837	Mariprofundus aestuarium	GCA_002795805.1	zetaproteobacteria
WP_100554785.1	GAP1-N1+GAP1-M+GAP1-C	DO-GTPase1→ GAP1-N1+GAP1-M+GAP1-C*→	787	Bradyrhizobium sp. UASWS1016	GCA_002256345.1	alphaproteobacteria
WP_102083185.1	GAP1-N1+GAP1-M+GAP1-C+EAD1	PNP_UDP_1+EAD1→ GAP2→ ZnR+DO-GTPase2.1→ DO-GTPase1→ GAP1-N1+GAP1-M+GAP1-C+EAD1*→	811	Psychrobacter sp. Choline-02u-9	GCA_001742065.1	gammaproteobacteria
WP_102837409.1	GAP1-N1+GAP1-M+GAP1-C	Calcineurin+GAPEAD1→ GAP2→ ZnR+DO-GTPase2.1→ DO-GTPase1→ GAP1-N1+GAP1-M+GAP1-C*→	871	Pseudomonas stutzeri	GCA_002890895.1	gammaproteobacteria
WP_102873726.1	GAP1-N1+GAP1-M+GAP1-C	AB_hydrolase+GAPEAD1→ GAP2→ ZnR+DO-GTPase2.1→ DO-GTPase1→ GAP1-N1+GAP1-M+GAP1-C*→	681	Phaeobacter inhibens	GCA_002892225.1	alphaproteobacteria
WP_102883896.1	GAP1-N1+GAP1-M+GAP1-C	AB_hydrolase+GAPEAD1→ GAP2→ ZnR+DO-GTPase2.1→ DO-GTPase1→ GAP1-N1+GAP1-M+GAP1-C*→	681	Phaeobacter inhibens	GCA_002892085.1	alphaproteobacteria
WP_103025358.1	GAP1-N1+GAP1-M+GAP1-C	Calcineurin+GAPEAD1→ GAP2→ ZnR+DO-GTPase2.1→ DO-GTPase1→ GAP1-N1+GAP1-M+GAP1-C*→	604	Vibrio campbellii	GCF_002893865.1	gammaproteobacteria
WP_103162222.1	GAP1-N1+GAP1-M+GAP1-C	GAP2→ ZnR+DO-GTPase2.1→ DO-GTPase1→ GAP1-N1+GAP1-M+GAP1-C*→	869	Pectobacterium carotovorum	GCA_002904025.1	gammaproteobacteria
WP_104015000.1	GAP1-N1+GAP1-M+GAP1-C	Calcineurin+GAPEAD1→ GAP2→ ZnR+DO-GTPase2.1→ DO-GTPase1→ GAP1-N1+GAP1-M+GAP1-C*→	813	Aeromonas jandaei	GCA_002925765.1	gammaproteobacteria
WP_105260592.1	GAP1-N1+GAP1-M+GAP1-C+EAD1	GAP2→ DO-GTPase2.1→ DO-GTPase1→ GAP1-N1+GAP1-M+GAP1-C+EAD1*→ CASPASE+EAD1→	827	Rhodoferax sp. TS-BS-61-7	GCA_002943465.1	betaproteobacteria
WP_105807446.1	GAP1-N1+GAP1-M+GAP1-C	Calcineurin+GAPEAD1→ GAP2→ ZnR+DO-GTPase2.1→ DO-GTPase1→ GAP1-N1+GAP1-M+GAP1-C*→	880	Burkholderia multivorans	GCA_002981255.1	betaproteobacteria
WP_106138897.1	GAP1-N1+GAP1-M+GAP1-C+EAD1	GAP2→ ZnR+DO-GTPase2.1→ DO-GTPase1→ GAP1-N1+GAP1-M+GAP1-C+EAD1*→ PNP_UDP_1+EAD1→	786	Spirosoma oryzae	GCF_003002825.1	bacteroidetes

**acc**	**architecture**	**operon**	**len**	**species**	**gca**	**taxend**
WP_106139082.1	GAP1-N1+GAP1-M+GAP1-C+EAD1	ZnR+DO-GTPase2.1→ DO-GTPase1→ GAP1-N1+GAP1-M+GAP1-C+EAD1*→ PNP_UDP_1+EAD1→	830	Spirosoma oryzae	GCA_003002825.1	bacteroidetes
WP_106448472.1	GAP1-N1+GAP1-M+GAP1-C	Calcineurin+GAPEAD1→ GAP2→ ZnR+DO-GTPase2.1→ PSE→ GAP1-N1+GAP1-M+GAP1-C*→	684	Simplicispira sp. SC1-8	GCA_003008595.1	betaproteobacteria
WP_107607145.1	GAP1-N1+GAP1-M+GAP1-C	Calcineurin+GAPEAD1→ PSE→ PSE→ ZnR+DO-GTPase2.1→ DO-GTPase1→ DO-GTPase1→ GAP1-N1+GAP1-M+GAP1-C*→	238	Leptospira santarosai	GCF_001008325.2	spirochaetes
WP_108195516.1	GAP1-N1+GAP1-M+GAP1-C	Calcineurin+GAPEAD1→ GAP2→ ZnR+DO-GTPase2.1→ DO-GTPase1→ GAP1-N1+GAP1-M+GAP1-C*→	611	Vibrio splendidus	GCA_003049865.1	gammaproteobacteria
WP_108311778.1	GAP1-N1+GAP1-M+GAP1-C	Calcineurin+GAPEAD1→ GAP2→ ZnR+DO-GTPase2.1→ PSE→ GAP1-N1+GAP1-M+GAP1-C*→	687	Limnohabitans parvus	GCA_003063455.1	betaproteobacteria
WP_108419298.1	GAP1-N1+GAP1-M+GAP1-C	Calcineurin+GAPEAD1→ GAP2→ ZnR+DO-GTPase2.1→ DO-GTPase1→ GAP1-N1+GAP1-M+GAP1-C*→	871	Klebsiella aerogenes	GCF_003057255.1	gammaproteobacteria

GAP1-N2-type GTPase-centric systems

acc	architecture	operon	len	species	gca	taxend
ADB15798.1	GAP1-N2+GAP1-M+GAP1-C	ZnR+ZnR+DO-GTPase2.2→ GAP1-N2+GAP1-M+GAP1-C*→??+TM+??+beta-rich??→ GYF+GYF+TM+TM+TM→	844	Pirellula staleyi DSM 6068	GCA_000025185.1	planctomycetes
ADB76255.1	GAP1-N2+GAP1-M+GAP1-C	Pkinase+TM→ TM→ ZnR+ZnR+DO-GTPase2.2→ GAP1-N2+GAP1-M+GAP1-C*→?→||←?←?||?→ Pkinase+low_complexity→	552	Geodermatophilus obscurus DSM 43160	GCA_000025345.1	actinobacteria
ADI12266.1	GAP1-N2+GAP1-M+GAP1-C	GAP1-N2+GAP1-M+GAP1-C*→	813	Streptomyces bingchenggensis BCW-1	GCA_000092385.1	actinobacteria
AFP36529.1	GAP1-N2+GAP1-M+GAP1-C	HRDC-like?+Fibronectin-like_repeats→ GAP1-N2+GAP1-M+GAP1-C*→??+DO-GTPase2.2→	866	Mycolicibacterium smegmatis MC2 155	GCA_000283295.1	actinobacteria
AGS49281.1	GAP1-N2+GAP1-M+GAP1-C	ZnR+ZnR+DO-GTPase2.2→ GAP1-N2+GAP1-M+GAP1-C*→ TM+TM+TM+TM+ZnR+ZnR+DO-GTPase2.2→?→ HSP70→ vWA(canonical)→ unknown+CASPASE→ HSP70→	762	uncultured bacterium esnapd2	-	bacteria
AJE68329.1	GAP1-N2+GAP1-M+GAP1-C	polar_leader+TM+TM+TM→ TM+vWA(canonical)+Fibronectin-like_repeats+TM+beta-rich+lc_tail→ Tubulin+alpha_helical+??+??→ GAP_unknown1+TM+TM+GAP_unknown2→ HRDC-like?+Fibronectin-like_repeats→ GAP1-N2+GAP1-M+GAP1-C*→ beta-rich+vWA(canonical)→??+DO-GTPase2.2→	661	Corynebacterium glutamicum	GCA_000828015.1	actinobacteria
AJI77744.1	GAP1-N2+GAP1-M+GAP1-C	polar_leader+TM+TM+TM→ TM+vWA(canonical)+Fibronectin-like_repeats+TM+beta-rich+lc_tail→ Tubulin+alpha_helical+??+??→ GAP_unknown1+TM+TM+GAP_unknown2→ HRDC-like?+Fibronectin-like_repeats→ GAP1-N2+GAP1-M+GAP1-C*→ beta-rich+vWA(canonical)→??+DO-GTPase2.2→ polar_leader+TM+TM+TM→	611	Corynebacterium singulare	GCA_000833575.1	actinobacteria
AKA68369.1	GAP1-N2+GAP1-M+GAP1-C	Fibronectin-like_repeats+??→ ZnR+ZnR+DO-GTPase2.2→ GAP1-N2+GAP1-M+GAP1-C*→ TM+TM+TM+TM+ZnR+ZnR+DO-GTPase2.2→ TM+vWA(canonical)+YtKA-like+TM+??→ Tubulin+coiled_coil+??→ Tubulin+coiled_coil+HTH?+??→ TM+TM+TM+vWA(canonical)+TM+TM+TM+polar_tail→ Fibronectin-like_repeats+??→ ZnR+ZnR+DO-GTPase2.2+ZnR+ZnR→	891	Clostridium scatologenes	GCA_000968375.1	firmicutes
AKN30804.1	GAP1-N2+GAP1-M+GAP1-C	Fibronectin-like_repeats+??→ ZnR+ZnR+DO-GTPase2.2→ GAP1-N2+GAP1-M+GAP1-C*→ TM+TM+TM+TM+ZnR+ZnR+DO-GTPase2.2→ TM+vWA(canonical)+YtKA-like+TM+??→ Tubulin+coiled_coil+??→ Tubulin+coiled_coil+HTH?+??→ TM+TM+TM+vWA(canonical)+TM+TM+TM+polar_tail→ Fibronectin-like_repeats+??→ ZnR+ZnR+DO-GTPase2.2+ZnR+ZnR→	891	Clostridium carboxidivorans P7	GCA_001038625.1	firmicutes
AKN72246.1	GAP1-N2+GAP1-M+GAP1-C	TPR_containing_alpha_helical+Fibronectin-like_repeats→ ZnR+ZnR+DO-GTPase2.2→ GAP1-N2+GAP1-M+GAP1-C*→ TM+TM+TM+TM+ZnR+ZnR+DO-GTPase2.2+TM→ vWA(canonical)→ Pkinase+ZnR+beta-rich→	951	Streptomyces sp. PBH53	GCA_001040905.1	actinobacteria
ALP34837.1	GAP1-N2+GAP1-M+GAP1-C+polar_tail+TM+beta-rich	Tubulin+coiled_coil+??→ Tubulin+coiled_coil+HTH?+??→ TM+TM+TM+vWA(canonical)+TM+TM+TM+polar_tail→ Fibronectin-like_repeats+??→ ZnR+ZnR+DO-GTPase2.2+ZnR+ZnR→ GAP1-N2+GAP1-M+GAP1-C+polar_tail+TM+beta-rich*→	1007	Paenibacillus sp. IHB B 3084	GCA_001447315.1	firmicutes
ALP51108.1	GAP1-N2+GAP1-M+GAP1-C	polar_leader+TM+TM+TM→ TM+vWA(canonical)+Fibronectin-like_repeats+TM+beta-rich+lc_tail→ Tubulin+alpha_helical+??+??→ GAP_unknown1+TM+TM+GAP_unknown2→ HRDC-like?+Fibronectin-like_repeats→ GAP1-N2+GAP1-M+GAP1-C*→ beta-rich+vWA(canonical)→??+DO-GTPase2.2→	661	Corynebacterium glutamicum	GCA_001447865.2	actinobacteria
AMA01136.1	GAP1-N2+GAP1-M+GAP1-C	polar_leader+TM+TM+TM→ TM+vWA(canonical)+Fibronectin-like_repeats+TM+beta-rich+lc_tail→ Tubulin+alpha_helical+??+??→ GAP_unknown1+TM+TM+GAP_unknown2→ HRDC-like?+Fibronectin-like_repeats→ GAP1-N2+GAP1-M+GAP1-C*→ beta-rich+vWA(canonical)→??+DO-GTPase2.2→	661	Corynebacterium glutamicum	GCA_001518935.2	actinobacteria
AMV18226.1	GAP1-N2+GAP1-M+TM+GAP1-C	DO-GTPase2.2→ GAP1-N2+GAP1-M+TM+GAP1-C*→??+TM+??+beta-rich??→ GYF+GYF+Trypsin→	871	Planctomyces sp. SH-PL14	GCA_001610835.1	planctomycetes
AMV18442.1	GAP1-N2+GAP1-M+TM+GAP1-C	ZnR+ZnR+DO-GTPase2.2→ GAP1-N2+GAP1-M+TM+GAP1-C*→??+TM+??+beta-rich??→ GYF+GYF+Trypsin→ Pkinase+low_complexity+TM→	896	Planctomyces sp. SH-PL14	GCA_001610835.1	planctomycetes
ANE05409.1	GAP1-N2+GAP1-M+GAP1-C	polar_leader+TM+TM+TM→ TM+vWA(canonical)+Fibronectin-like_repeats+TM+beta-rich+lc_tail→ Tubulin+alpha_helical+??+??→ GAP_unknown1+TM+TM+GAP_unknown2→ HRDC-like?+Fibronectin-like_repeats→ GAP1-N2+GAP1-M+GAP1-C*→ beta-rich+vWA(canonical)→	647	Corynebacterium crudilactis	GCA_001643015.1	actinobacteria
APA96797.1	GAP1-N2+GAP1-M+GAP1-C	Pkinase+TM→?→ ZnR+DO-GTPase2.1→ GAP1-N2+GAP1-M+GAP1-C*→	742	Nocardia seriolae	GCA_001865855.1	actinobacteria
APB71256.1	GAP1-N2+GAP1-M+GAP1-C+polar_tail+TM+beta-rich	TM+vWA(canonical)+YtKA-like+TM+??→ Tubulin+coiled_coil+??→ Tubulin+coiled_coil+HTH?+??→ TM+TM+TM+vWA(canonical)+TM+TM+TM+polar_tail→ Fibronectin-like_repeats+??→ ZnR+ZnR+DO-GTPase2.2+ZnR+ZnR→ GAP1-N2+GAP1-M+GAP1-C+polar_tail+TM+beta-rich*→	1002	Paenibacillus polymyxa	GCA_001874405.2	firmicutes
APB76202.1	GAP1-N2+GAP1-M+GAP1-C+polar_tail+TM+beta-rich	TM+vWA(canonical)+YtKA-like+TM+??→ PSE→ Tubulin+coiled_coil+HTH?+??→ TM+TM+TM+vWA(canonical)+TM+TM+TM+polar_tail→ Fibronectin-like_repeats+??→ ZnR+ZnR+DO-GTPase2.2+ZnR+ZnR→ GAP1-N2+GAP1-M+GAP1-C+polar_tail+TM+beta-rich*→	1002	Paenibacillus polymyxa	GCA_001874425.2	firmicutes
APS18487.1	GAP1-N2+GAP1-M+GAP1-C	GAP1-N2+GAP1-M+GAP1-C*→	821	Streptomyces sp. Tue 6075	GCA_001931635.1	actinobacteria
APT08387.1	GAP1-N2+GAP1-M+GAP1-C	polar_leader+TM+TM+TM→ TM+vWA(canonical)+Fibronectin-like_repeats+TM+beta-rich+lc_tail→ Tubulin+alpha_helical+??+??→ GAP_unknown1+TM+TM+GAP_unknown2→ HRDC-like?+Fibronectin-like_repeats→ GAP1-N2+GAP1-M+GAP1-C*→ beta-rich+vWA(canonical)→??+DO-GTPase2.2→	661	Corynebacterium glutamicum	GCA_001936195.1	actinobacteria
ARI51644.1	GAP1-N2+GAP1-M+GAP1-C	GAP1-N2+GAP1-M+GAP1-C*→	882	Streptomyces sp. S8	GCA_002094995.1	actinobacteria
ARX88353.1	GAP1-N2+GAP1-M+GAP1-C	Pkinase→?→?→?→?→ TPR_containing_alpha_helical+Fibronectin-like_repeats→ ZnR+ZnR+DO-GTPase2.2→ GAP1-N2+GAP1-M+GAP1-C*→?→ TM+TM+TM+TM+ZnR+ZnR+DO-GTPase2.2→	764	Streptomyces alboflavus	GCA_002189675.1	actinobacteria
AUG76307.1	GAP1-N2+GAP1-M+GAP1-C	PP2C→ Pkinase→?→?→?→ HSP70_domain_2+low_complexity+domain_betaX→ ZnR+ZnR+DO-GTPase2.2→ GAP1-N2+GAP1-M+GAP1-C*→ TM+TM+TM+TM+ZnR+ZnR+DO-GTPase2.2→	807	Kitasatospora sp. MMS16-BH015	GCA_002943525.1	actinobacteria
BAC00114.1	GAP1-N2+GAP1-M+GAP1-C	polar_leader+TM+TM+TM→ TM+vWA(canonical)+Fibronectin-like_repeats+TM+beta-rich+lc_tail→ Tubulin+alpha_helical+??+??→ GAP_unknown1+TM+TM+GAP_unknown2→ HRDC-like?+Fibronectin-like_repeats→ GAP1-N2+GAP1-M+GAP1-C*→ beta-rich+vWA(canonical)→??+DO-GTPase2.2→	662	Corynebacterium glutamicum ATCC 13032	GCA_000011325.1	actinobacteria
BAF55637.1	GAP1-N2+GAP1-M+GAP1-C	polar_leader+TM+TM+TM→ TM+vWA(canonical)+Fibronectin-like_repeats+TM+beta-rich+lc_tail→ Tubulin+alpha_helical+??+??→ GAP_unknown1+TM+TM+GAP_unknown2→ HRDC-like?+Fibronectin-like_repeats→ GAP1-N2+GAP1-M+GAP1-C*→ beta-rich+vWA(canonical)→??+DO-GTPase2.2→	662	Corynebacterium glutamicum R	GCA_000010225.1	actinobacteria
BAG18277.1	GAP1-N2+GAP1-M+GAP1-C	GAP1-N2+GAP1-M+GAP1-C*→	915	Streptomyces griseus subsp. griseus NBRC 13350	GCA_000010605.1	actinobacteria
BAH49624.1	GAP1-N2+GAP1-M+GAP1-C	polar_leader+TM+TM+TM→ TM+vWA(canonical)+Fibronectin-like_repeats+TM+beta-rich+lc_tail→ Tubulin+alpha_helical+??+??→ GAP_unknown1+TM+TM+GAP_unknown2→ HRDC-like?+Fibronectin-like_repeats→ GAP1-N2+GAP1-M+GAP1-C*→ beta-rich+vWA(canonical)→??+DO-GTPase2.2→	851	Rhodococcus opacus B4	GCA_000010805.1	actinobacteria
BAU97076.1	GAP1-N2+GAP1-M+GAP1-C	polar_leader+TM+TM+TM→ TM+vWA(canonical)+Fibronectin-like_repeats+TM+beta-rich+lc_tail→ Tubulin+alpha_helical+??+??→ GAP_unknown1+TM+TM+GAP_unknown2→ HRDC-like?+Fibronectin-like_repeats→ GAP1-N2+GAP1-M+GAP1-C*→ beta-rich+vWA(canonical)→??+DO-GTPase2.2→	654	Corynebacterium glutamicum	GCA_002355155.1	actinobacteria
BAV22155.1	GAP1-N2+GAP1-M+GAP1-C	GrpE→ HSP70→??+Fibronectin-like_repeats→ ZnR+ZnR+DO-GTPase2.2→ GAP1-N2+GAP1-M+GAP1-C*→ TM+TM+TM+TM+ZnR+ZnR+DO-GTPase2.2→	805	Corynebacterium glutamicum	GCA_002355675.1	actinobacteria
BAV24350.1	GAP1-N2+GAP1-M+GAP1-C	polar_leader+TM+TM+TM→ TM+vWA(canonical)+Fibronectin-like_repeats+TM+beta-rich+lc_tail→ Tubulin+alpha_helical+??+??→ GAP_unknown1+TM+TM+GAP_unknown2→ HRDC-like?+Fibronectin-like_repeats→ GAP1-N2+GAP1-M+GAP1-C*→ beta-rich+vWA(canonical)→??+DO-GTPase2.2→	654	Corynebacterium glutamicum	GCA_002355675.1	actinobacteria
BAV84920.1	GAP1-N2+GAP1-M+GAP1-C	HRDC-like?+Fibronectin-like_repeats→ GAP1-N2+GAP1-M+GAP1-C*→	695	Actinomyces naeslundii	GCA_002355915.1	actinobacteria
CAF20743.1	GAP1-N2+GAP1-M+GAP1-C	polar_leader+TM+TM+TM→ TM+vWA(canonical)+Fibronectin-like_repeats+TM+beta-rich+lc_tail→ Tubulin+alpha_helical+??+??→ GAP_unknown1+TM+TM+GAP_unknown2→ HRDC-like?+Fibronectin-like_repeats→ GAP1-N2+GAP1-M+GAP1-C*→ beta-rich+vWA(canonical)→??+DO-GTPase2.2→	654	Corynebacterium glutamicum ATCC 13032	GCA_000196335.1	actinobacteria
CCI52713.1	GAP1-N2+GAP1-M+GAP1-C	polar_leader+TM+TM+TM→ TM+vWA(canonical)+Fibronectin-like_repeats+TM+beta-rich+lc_tail→ Tubulin+alpha_helical+??+??→ GAP_unknown1+TM+TM+GAP_unknown2→ HRDC-like?+Fibronectin-like_repeats→ GAP1-N2+GAP1-M+GAP1-C*→?→??+DO-GTPase2.2→	883	Tetrasphaera jenkinsii Ben 74	GCA_001046875.1	actinobacteria
CDB93702.1	GAP1-N2+GAP1-M+TM+GAP1-C	GAP1-N2+GAP1-M+TM+GAP1-C*→	713	Firmicutes bacterium CAG:41	GCA_000431615.1	firmicutes
CDC62438.1	GAP1-N2+GAP1-M+TM+GAP1-C	GAP1-N2+GAP1-M+TM+GAP1-C*→	699	Clostridium sp. CAG:448	GCA_000433415.1	firmicutes
CDE12586.1	GAP1-N2+GAP1-M+TM+GAP1-C	HSP70(frag)+vWA(canonical)→?→ DO-GTPase2.1→ GAP1-N2+GAP1-M+TM+GAP1-C*→	767	Ruminococcus sp. CAG:330	GCA_000437175.1	firmicutes

acc	architecture	operon	len	species	gca	taxend
CDE84522.1	GAP1-N2+GAP1-M+TM+GAP1-C	FHA-like?+ZnR+ZnR+DO-GTPase2.2→ GAP1-N2+GAP1-M+TM+GAP1-C*→	451	Coraliomargarita sp. CAG:312	GCA_000438015.1	verrucomicrobia
CNG08901.1	GAP1-N2+GAP1-M+GAP1-C	TPR_containing_alpha_helical+Fibronectin-like_repeats→ ZnR+ZnR+DO-GTPase2.2→ GAP1-N2+GAP1-M+GAP1-C*→ TM+TM+TM+TM+ZnR+ZnR+DO-GTPase2.2+TM→	656	Mycobacterium tuberculosis	GCA_001160225.1	actinobacteria
CQD15063.1	GAP1-N2+GAP1-M+GAP1-C	polar_leader+TM+TM+TM→ TM+vWA(canonical)+Fibronectin-like_repeats+TM+beta-rich+lc_tail→ Tubulin+alpha_helical+??+??→ GAP_unknown1+TM+TM+GAP_unknown2→ HRDC-like?+Fibronectin-like_repeats→ GAP1-N2+GAP1-M+GAP1-C*→ beta-rich+vWA(canonical)→??+DO-GTPase2.2→ polar_leader+TM+TM+TM→	532	Corynebacterium striatum	GCA_001261935.1	actinobacteria
EDK33704.1	GAP1-N2+GAP1-M	TM+TM+TM+TM+ZnR+ZnR+DO-GTPase2.2→ TM+vWA(canonical)+YtKA-like+TM+??→ Tubulin+coiled_coil+??→ Tubulin+coiled_coil+HTH?+??→ Fibronectin-like_repeats+??→ ZnR+ZnR+DO-GTPase2.2+ZnR+ZnR→ GAP1-N2+GAP1-M*→	353	Clostridium kluyveri DSM 555	GCA_000016505.1	firmicutes
EEB63587.1	GAP1-N2+GAP1-M+GAP1-C	polar_leader+TM+TM+TM→ TM+vWA(canonical)+Fibronectin-like_repeats+TM+beta-rich+lc_tail→ Tubulin+alpha_helical+??+??→ GAP_unknown1+TM+TM+GAP_unknown2→ HRDC-like?+Fibronectin-like_repeats→ GAP1-N2+GAP1-M+GAP1-C*→ beta-rich+vWA(canonical)→??+DO-GTPase2.2→	868	Corynebacterium amycolatum SK46	GCA_000173655.1	actinobacteria
EET86374.1	GAP1-N2+GAP1-M+GAP1-C	TM+TM+TM+TM+ZnR+ZnR+DO-GTPase2.2→ TM+vWA(canonical)+YtKA-like+TM+??→ Tubulin+coiled_coil+??→ Tubulin+coiled_coil+HTH?+??→ TM+TM+TM+vWA(canonical)+TM+TM+TM+polar_tail→ Fibronectin-like_repeats+??→ ZnR+ZnR+DO-GTPase2.2+ZnR+ZnR→ GAP1-N2+GAP1-M+GAP1-C*→ GAP1-N2+GAP1-M→	144	Clostridium carboxidivorans P7	GCA_000175595.1	firmicutes
EET86375.1	GAP1-M	TM+vWA(canonical)+YtKA-like+TM+??→ Tubulin+coiled_coil+??→ Tubulin+coiled_coil+HTH?+??→ TM+TM+TM+vWA(canonical)+TM+TM+TM+polar_tail→ Fibronectin-like_repeats+??→ ZnR+ZnR+DO-GTPase2.2+ZnR+ZnR→ GAP1-N2+GAP1-M+GAP1-C→ GAP1-M*→	216	Clostridium carboxidivorans P7	GCA_000175595.1	firmicutes
EFE84745.1	GAP1-N2+GAP1-M+GAP1-C	GAP1-N2+GAP1-M+GAP1-C*→	1002	Streptomyces albus J1074	GCA_000156475.1	actinobacteria
EFL03271.1	GAP1-N2+GAP1-M+GAP1-C	GAP1-N2+GAP1-M+GAP1-C*→	785	Streptomyces sp. SPB78	GCA_000158855.1	actinobacteria
EFL18295.1	GAP1-N2+GAP1-M+GAP1-C	GAP1-N2+GAP1-M+GAP1-C*→	293	Streptomyces sp. C	GCA_000158895.1	actinobacteria
EFL28264.1	GAP1-N2+GAP1-M+GAP1-C	TPR_containing_alpha_helical+Fibronectin-like_repeats→ ZnR+ZnR+DO-GTPase2.2→ GAP1-N2+GAP1-M+GAP1-C*→ TM+TM+TM+TM+ZnR+ZnR+DO-GTPase2.2+TM→ vWA(canonical)→ PP2C→ Pkinase+ZnR+beta-rich→	725	Streptomyces himastatinicus ATCC 53653	GCA_000158915.1	actinobacteria
EGV41882.1	GAP1-N2+GAP1-M+GAP1-C	TM+TM+TM→ TM+vWA(canonical)+Fibronectin-like_repeats+TM+beta-rich+lc_tail→ Tubulin+alpha_helical+??+??→ GAP_unknown1+TM+TM+GAP_unknown2→ HRDC-like?+Fibronectin-like_repeats→ GAP1-N2+GAP1-M+GAP1-C*→ beta-rich+vWA(canonical)→??+DO-GTPase2.2→	661	Corynebacterium glutamicum S9114	GCA_000224315.2	actinobacteria
EHM28476.1	GAP1-N2+GAP1-M+GAP1-C	TM+TM+TM+TM+ZnR+ZnR+DO-GTPase2.2+TM→?→ vWA(canonical)→ PP2C→ Pkinase+ZnR+beta-rich→||←?←?||GAP1-N2+GAP1-M+GAP1-C*→	805	Streptomyces sp. W007	GCA_000239075.2	actinobacteria
EHQ63001.1	GAP1-N2+GAP1-M+GAP1-C+polar_tail+TM+beta-rich	TM+vWA(canonical)+YtKA-like+TM+??→ Tubulin+coiled_coil+??→ Tubulin+coiled_coil+HTH?+??→ TM+TM+TM+vWA(canonical)+TM+TM+TM+polar_tail→ Fibronectin-like_repeats+??→ ZnR+ZnR+DO-GTPase2.2+ZnR+ZnR→ GAP1-N2+GAP1-M+GAP1-C+polar_tail+TM+beta-rich*→	1034	Paenibacillus dendritiformis C454	GCA_000245555.2	firmicutes
EJZ04364.1	GAP1-N2+GAP1-M+GAP1-C	HRDC-like?+Fibronectin-like_repeats→ GAP1-N2+GAP1-M+GAP1-C*→	133	Mycolicibacterium fortuitum subsp. fortuitum DSM 46621 = ATCC 6841	GCA_000295855.1	actinobacteria
EPH39666.1	GAP1-N2+GAP1-M+GAP1-C	GAP1-N2+GAP1-M+GAP1-C*→ vWA(canonical)→	870	Streptomyces aurantiacus JA 4570	GCA_000414115.1	actinobacteria
EQB68984.1	GAP1-N2+GAP1-M+GAP1-C	TM+TM+TM+TM+TM+TM+DO-GTPase2.1→ GAP1-N2+GAP1-M+GAP1-C*→??→ TM_region+DO-GTPase1→	556	Thermoplasmatales archaeon Gpl	GCA_001856845.1	euryarchaeota
EQD81319.1	GAP1-N2+GAP1-M+GAP1-C	ZnR+ZnR+DO-GTPase2.2→ GAP1-N2+GAP1-M+GAP1-C*→	434	Saccharopolyspora erythraea D	GCA_000448385.1	actinobacteria
EST36721.1	GAP1-N2+GAP1-M+GAP1-C	GAP1-N2+GAP1-M+GAP1-C*→ TM+TM+TM+TM+ZnR+ZnR+DO-GTPase2.2+Jelly_roll+beta-sandwich→||←STAND_NTPase+TM+TM+TM+TM+TM+TM+TM	371	Streptomyces roseochromogenus subsp. oscitans DS 12.976	GCA_000497445.1	actinobacteria
ETJ06992.1	GAP1-N2+GAP1-M+GAP1-C	GAP1-N2+GAP1-M+GAP1-C(frag)*→	492	Actinomyces urogenitalis DORA_12	GCA_000508425.1	actinobacteria
ETT33608.1	GAP1-N2+GAP1-M+GAP1-C+polar_tail+TM+beta-rich	TM+vWA(canonical)+YtKA-like+TM+??→ Tubulin+coiled_coil+??→ Tubulin+coiled_coil+HTH?+??→?→ TM+TM+TM+vWA(canonical)+TM+TM+TM+polar_tail→ Fibronectin-like_repeats+??→ ZnR+ZnR+DO-GTPase2.2+ZnR+ZnR→ GAP1-N2+GAP1-M+GAP1-C+polar_tail+TM+beta-rich*→	796	Paenibacillus sp. FSL R7-269	GCA_000517865.1	firmicutes
ETT49033.1	GAP1-N2+GAP1-M+GAP1-C+polar_tail+TM+beta-rich	TM+vWA(canonical)+YtKA-like+TM+??→ Tubulin+coiled_coil+??→ Tubulin+coiled_coil+HTH?+??→ TM+TM+TM+vWA(canonical)+TM+TM+TM+polar_tail→ Fibronectin-like_repeats+??→ ZnR+ZnR+DO-GTPase2.2+ZnR+ZnR→ GAP1-N2+GAP1-M+GAP1-C+polar_tail+TM+beta-rich*→	1067	Paenibacillus sp. FSL H7-689	GCA_000517905.1	firmicutes
ETT60645.1	GAP1-N2+GAP1-M+GAP1-C+polar_tail+TM+beta-rich	Tubulin+coiled_coil+??→ Tubulin+coiled_coil+HTH?+??→?→?→ TM+TM+TM+vWA(canonical)+TM+TM+TM+polar_tail→ Fibronectin-like_repeats+??→ ZnR+ZnR+DO-GTPase2.2+ZnR+ZnR→ GAP1-N2+GAP1-M+GAP1-C+polar_tail+TM+beta-rich*→	837	Paenibacillus sp. FSL R7-277	GCA_000517965.1	firmicutes
ETZ98452.1	GAP1-M+GAP1-C	Tubulin+alpha_helical+??+??→ GAP_unknown1+TM+TM+GAP_unknown2→ GAP_unknown1+TM+TM+GAP_unknown2→ HRDC-like?+Fibronectin-like_repeats→ HRDC-like?+Fibronectin-like_repeats→ GAP1-N2+GAP1-M+GAP1-C→ GAP1-M+GAP1-C*→?→ beta-rich+vWA(canonical)→??+DO-GTPase2.2→	598	Mycobacterium kansasii 824	GCA_000524095.1	actinobacteria
EYT47746.1	GAP1-N2+GAP1-M+GAP1-C	GAP1-N2+GAP1-M+GAP1-C*→	325	Dietzia sp. UCD-THP	GCA_000349585.1	actinobacteria
GAK49946.1	GAP1-N2+GAP1-M+low_complexity+TM+TM	DO-GTPase2.1→ GAP1-N2+GAP1-M+low_complexity+TM+TM*→	480	Candidatus Moduliflexus flocculans	GCA_000739515.1	bacteria
GAS84505.1	GAP1-N2+GAP1-M+GAP1-C+polar_tail+TM+beta-rich	TM+vWA(canonical)+YtKA-like+TM+??→ Tubulin+coiled_coil+??→ Tubulin+coiled_coil+HTH?+??→ TM+TM+TM+vWA(canonical)+TM+TM+TM+polar_tail→ Fibronectin-like_repeats+??→ ZnR+ZnR+DO-GTPase2.2+ZnR+ZnR→ GAP1-N2+GAP1-M+GAP1-C+polar_tail+TM+beta-rich*→	1062	Paenibacillus amylolyticus	GCA_001570725.1	firmicutes
GAV11658.1	GAP1-N2+GAP1-M+GAP1-C+polar_tail+TM+beta-rich	TM+vWA(canonical)+YtKA-like+TM+??→ Tubulin+coiled_coil+??→ Tubulin+coiled_coil+HTH?+??→ TM+TM+TM+vWA(canonical)+TM+TM+TM+polar_tail→ Fibronectin-like_repeats+??→ ZnR+ZnR+DO-GTPase2.2+ZnR+ZnR→ GAP1-N2+GAP1-M+GAP1-C+polar_tail+TM+beta-rich*→	947	Paenibacillus sp. NAIST15-1	GCA_001894745.1	firmicutes
GAV98212.1	GAP1-N2+GAP1-M+GAP1-C	polar_leader+TM+TM+TM→ TM+vWA(canonical)+Fibronectin-like_repeats+TM+beta-rich+lc_tail→ Tubulin+alpha_helical+??+??→ GAP_unknown1+TM+TM+GAP_unknown2→ HRDC-like?+Fibronectin-like_repeats→ GAP1-N2+GAP1-M+GAP1-C*→ beta-rich+vWA(canonical)→??+DO-GTPase2.2→	661	Corynebacterium glutamicum	GCA_002003445.1	actinobacteria
KAJ49549.1	GAP1-N2+GAP1-M	ZnR+ZnR+DO-GTPase2.2+ZnR+ZnR→ GAP1-N2+GAP1-M*→	387	Clostridium tetanomorphum DSM 665	GCA_000647895.1	firmicutes
KAJ53559.1	GAP1-N2+GAP1-M	TM+TM+TM+TM+ZnR+ZnR+DO-GTPase2.2→ TM+vWA(canonical)+YtKA-like+TM+??→ Tubulin+coiled_coil+??→ Tubulin+coiled_coil+HTH?+??→ TM+TM+TM+vWA(canonical)+TM+TM+TM+polar_tail→ Fibronectin-like_repeats+??→ ZnR+ZnR+DO-GTPase2.2+ZnR+ZnR→ GAP1-N2+GAP1-M*→	387	Clostridium tetanomorphum DSM 665	GCA_000647895.1	firmicutes
KDQ04749.1	GAP1-M+GAP1-C	GrpE→ TPR_containing_alpha_helical+Fibronectin-like_repeats→ ZnR+ZnR+DO-GTPase2.2→?→ GAP1-M+GAP1-C*→ TM+TM+TM+TM+ZnR+ZnR+DO-GTPase2.2→??→ HSP70→ vWA(canonical)→	701	Rhodococcus qingshengii	GCA_000698455.1	actinobacteria
KFI97917.1	GAP1-N2+GAP1-M+GAP1-C	TM+TM+TM+TM+ZnR+ZnR+DO-GTPase2.2→ GrpE→ HSP70→ HSP70+vWA(canonical)→ DnaJ+DnaJ+ZnR+ZnR+coiled_coil+Fibronectin-like_repeats→ ZnR+ZnR+DO-GTPase2.2→ GAP1-N2+GAP1-M+GAP1-C*→	967	Bifidobacterium stellenboschense	GCA_000741785.1	actinobacteria
KGF17444.1	GAP1-N2+GAP1-M+GAP1-C	TM+vWA(canonical)+Fibronectin-like_repeats+TM+beta-rich+lc_tail→ Tubulin+alpha_helical+??+??→ GAP_unknown1+TM+TM+GAP_unknown2→ HRDC-like?+Fibronectin-like_repeats→ GAP1-N2+GAP1-M+GAP1-C*→??+DO-GTPase2.2→	854	Corynebacterium freneyi DNF00450	GCA_000758965.1	actinobacteria
KHD07856.1	GAP1-N2+GAP1-M+GAP1-C	ZnR+ZnR+DO-GTPase2.2→ GAP1-N2+GAP1-M+GAP1-C*→ TM+TM+TM+TM+ZnR+ZnR+DO-GTPase2.2→	813	Candidatus Thiomargarita nelsonii	GCA_000785145.2	gammaproteobacteria
KHD08233.1	GAP1-N2+GAP1-M+GAP1-C	DO-GTPase1→?→ GAP1-N2+GAP1-M+GAP1-C*→ DO-GTPase2?→	914	Candidatus Thiomargarita nelsonii	GCA_000785145.2	gammaproteobacteria
KHD11963.1	GAP1-N2+GAP1-M+GAP1-C	ZnR+ZnR+DO-GTPase2.2→ GAP1-N2+GAP1-M+GAP1-C*→	668	Candidatus Thiomargarita nelsonii	-	gammaproteobacteria
KHD12726.1	GAP1-N2+GAP1-M	DO-GTPase1→?→ GAP1-N2+GAP1-M*→	187	Candidatus Thiomargarita nelsonii	-	gammaproteobacteria
KHD13072.1	GAP1-N2+GAP1-M+GAP1-C	ZnR+ZnR+DO-GTPase2.2→ GAP1-N2+GAP1-M+GAP1-C*→	662	Candidatus Thiomargarita nelsonii	-	gammaproteobacteria
KIF04207.1	GAP1-N2+GAP1-M+GAP1-C	GAP1-N2+GAP1-M+GAP1-C*→	206	Streptomyces sp. RSD-27	GCA_000817655.1	actinobacteria
KIF72394.1	GAP1-N2+GAP1-M+GAP1-C	GAP1-N2+GAP1-M+GAP1-C*→	812	Streptomyces sp. AcH 505	GCA_000818175.1	actinobacteria

acc	architecture	operon	len	species	gca	taxend
KIH72595.1	GAP1-N2+GAP1-M+GAP1-C	polar_leader+TM+TM+TM→ TM+vWA(canonical)+Fibronectin-like_repeats+TM+beta-rich+lc_tail→ Tubulin+alpha_helical+??+??→ GAP_unknown1+TM+TM+GAP_unknown2→ HRDC-like?+Fibronectin-like_repeats→ GAP1-N2+GAP1-M+GAP1-C*→ beta-rich+vWA(canonical)→??+DO-GTPase2.2→	661	Corynebacterium glutamicum	GCA_000819915.1	actinobacteria
KIX33869.1	GAP1-N2+GAP1-M+GAP1-C	GAP1-N2+GAP1-M+GAP1-C*→	821	Streptomyces griseus	-	actinobacteria
KIX77715.1	GAP1-N2+GAP1-M+GAP1-C	GAP1-N2+GAP1-M+GAP1-C*→	135	Streptomyces sp. MBRL 601	GCA_000932205.1	actinobacteria
KJY25356.1	GAP1-N2+GAP1-M+GAP1-C	GAP1-N2+GAP1-M+GAP1-C*→	204	Streptomyces katrae	GCA_000966975.1	actinobacteria
KJY44650.1	GAP1-N2+GAP1-M+GAP1-C	GAP1-N2+GAP1-M+GAP1-C*→	273	Streptomyces sp. NRRL S-444	GCA_000966985.1	actinobacteria
KMK66000.1	GAP1-N2+GAP1-M+TM+GAP1-C	DO-GTPase2.2→ GAP1-N2+GAP1-M+TM+GAP1-C*→	551	Puniceibacterium sp. IMCC21224	GCA_001038505.1	alphaproteobacteria
KMS85951.1	GAP1-N2+GAP1-M+GAP1-C	TPR_containing_alpha_helical+Fibronectin-like_repeats→ ZnR+ZnR+DO-GTPase2.2→ GAP1-N2+GAP1-M+GAP1-C*→ vWA(canonical)→ Pkinase+ZnR+beta-rich→	879	Streptomyces regensis	GCA_001047335.1	actinobacteria
KNB71604.1	GAP1-N2+GAP1-M+GAP1-C+polar_tail+TM+beta-rich	TM+vWA(canonical)+YtKA-like+TM+??→ Tubulin+coiled_coil+??→ Tubulin+coiled_coil+HTH?+??→ TM+TM+TM+vWA(canonical)+TM+TM+TM+polar_tail→ Fibronectin-like_repeats+??→ ZnR+ZnR+DO-GTPase2.2+ZnR+ZnR→ GAP1-N2+GAP1-M+GAP1-C+polar_tail+TM+beta-rich*→	825	Brevibacillus reuszeri	GCA_001187725.1	firmicutes
KOG83654.1	GAP1-N2+GAP1-M+GAP1-C	GAP1-N2+GAP1-M+GAP1-C*→	840	Streptomyces griseus subsp. rhodochrous	GCA_001270675.1	actinobacteria
KOP69111.1	GAP1-N2+GAP1-M+GAP1-C+polar_tail+TM+beta-rich	TM+vWA(canonical)+YtKA-like+TM+??→ Tubulin+coiled_coil+??→ Tubulin+coiled_coil+HTH?+??→ TM+TM+TM+vWA(canonical)+TM+TM+TM+polar_tail→ Fibronectin-like_repeats+??→ ZnR+ZnR+DO-GTPase2.2+ZnR+ZnR→ GAP1-N2+GAP1-M+GAP1-C+polar_tail+TM+beta-rich*→	1010	Bacillus sp. FJAT-18019	GCA_001275645.1	firmicutes
KOR29103.1	GAP1-N2+GAP1-M+GAP1-C	GAP1-N2+GAP1-M+GAP1-C*→	440	Achromatium sp. WMS3	GCA_001276615.1	gammaproteobacteria
KOR33350.1	GAP1-N2+GAP1-M+GAP1-C	GAP1-N2+GAP1-M+GAP1-C*→	839	Achromatium sp. WMS3	GCA_001276615.1	gammaproteobacteria
KOR82125.1	GAP1-N2+GAP1-M+GAP1-C+polar_tail+TM+beta-rich	TM+vWA(canonical)+YtKA-like+TM+??→ Tubulin+coiled_coil+??→ Tubulin+coiled_coil+HTH?+??→ TM+TM+TM+vWA(canonical)+TM+TM+TM+polar_tail→ Fibronectin-like_repeats+??→ ZnR+ZnR+DO-GTPase2.2+ZnR+ZnR→ GAP1-N2+GAP1-M+GAP1-C+polar_tail+TM+beta-rich*→	1011	Paenibacillus solani	GCA_001277345.1	firmicutes
KOS01186.1	GAP1-N2+GAP1-M+GAP1-C+polar_tail+TM+beta-rich	TM+vWA(canonical)+YtKA-like+TM+??→ Tubulin+coiled_coil+??→ Tubulin+coiled_coil+HTH?+??→ TM+TM+TM+vWA(canonical)+TM+TM+TM+polar_tail→ Fibronectin-like_repeats+??→ ZnR+ZnR+DO-GTPase2.2+ZnR+ZnR→ GAP1-N2+GAP1-M+GAP1-C+polar_tail+TM+beta-rich*→	1006	Paenibacillus polymyxa	GCA_001277855.1	firmicutes
KOU00955.1	GAP1-N2+GAP1-M+GAP1-C	GAP1-N2+GAP1-M+GAP1-C*→	840	Streptomyces sp. NRRL F-2295	GCA_001279425.1	actinobacteria
KOU49584.1	GAP1-N2+GAP1-M+GAP1-C	GAP1-N2+GAP1-M+GAP1-C*→	840	Streptomyces sp. MMG1522	GCA_001279735.1	actinobacteria
KOV83698.1	GAP1-N2+GAP1-M+GAP1-C	TPR_containing_alpha_helical+Fibronectin-like_repeats→ ZnR+ZnR+DO-GTPase2.2→ GAP1-N2+GAP1-M+GAP1-C*→ vWA(canonical)→ Pkinase+ZnR+beta-rich→	780	Streptomyces sp. NRRL WC-3723	GCA_001280045.1	actinobacteria
KOY14601.1	GAP1-N2+GAP1-M+GAP1-C+polar_tail+TM+beta-rich	TM+vWA(canonical)+YtKA-like+TM+??→ Tubulin+coiled_coil+??→ Tubulin+coiled_coil+HTH?+??→ TM+TM+TM+vWA(canonical)+TM+TM+TM+polar_tail→ Fibronectin-like_repeats+??→ ZnR+ZnR+DO-GTPase2.2+ZnR+ZnR→ GAP1-N2+GAP1-M+GAP1-C+polar_tail+TM+beta-rich*→	1078	Paenibacillus sp. A59	GCA_001280595.1	firmicutes
KOY55812.1	GAP1-N2+GAP1-M+GAP1-C	TPR_containing_alpha_helical+Fibronectin-like_repeats→ GAP1-N2+GAP1-M+GAP1-C*→ TM+TM+TM+TM+ZnR+ZnR+DO-GTPase2.2+TM→ vWA(canonical)→ PP2C→ Pkinase+ZnR+beta-rich→	834	Streptomyces sp. XY332	GCA_001280885.1	actinobacteria
KPI13878.1	GAP1-N2+GAP1-M+GAP1-C	Fibronectin-like_repeats→ ZnR+ZnR+DO-GTPase2.2→ GAP1-N2+GAP1-M+GAP1-C*→	344	Actinobacteria bacterium OV450	GCA_001298555.1	actinobacteria
KPL06138.1	GAP1-N2+GAP1-M+TM+GAP1-C	ZnR+DO-GTPase2.1→ GAP1-N2+GAP1-M+TM+GAP1-C*→	393	candidate division BRC1 bacterium SM23_51	GCA_001304075.1	bacteria
KUO19072.1	GAP1-N2+GAP1-M+GAP1-C	TPR_containing_alpha_helical+Fibronectin-like_repeats→ ZnR+ZnR+DO-GTPase2.2→ GAP1-N2+GAP1-M+GAP1-C*→ TM+TM+TM+TM+ZnR+ZnR+DO-GTPase2.2+TM→ vWA(canonical)→ PP2C→ Pkinase+ZnR+beta-rich→	1166	Streptomyces sp. RV15	GCA_001514305.1	actinobacteria
KXA22897.1	GAP1-N2+GAP1-M+GAP1-C	TM+TM+TM+TM+ZnR+ZnR+DO-GTPase2.2→?→ HSP70→ HSP70+vWA(canonical)→ DnaJ+DnaJ+ZnR+ZnR+coiled_coil+Fibronectin-like_repeats→ ZnR+ZnR+DO-GTPase2.2→ GAP1-N2+GAP1-M+GAP1-C*→	884	Gardnerella vaginalis	GCA_001546485.1	actinobacteria
KXK18278.1	GAP1-N2+GAP1-M+GAP1-C	GAP1-N2+GAP1-M+GAP1-C*→	1057	Chloroflexi bacterium OLB15	GCA_001567085.1	chloroflexi
OAA27476.1	GAP1-N2+GAP1-M+GAP1-C+ZnR+DO-GTPase2	GAP1-N2+GAP1-M+GAP1-C+ZnR+DO-GTPase2*→	998	Frankia sp. EI5c	GCA_001636565.1	actinobacteria
OAI50476.1	GAP1-N2+GAP1-M+TM+low_complexity	GAP1-N2+GAP1-M+TM+low_complexity*→	435	Planctomycetaceae bacterium SCGC AG-212-F19	GCA_001644415.1	planctomycetes
ODS34705.1	GAP1-N2+GAP1-M+GAP1-C	DO-GTPase2.1→ GAP1-N2+GAP1-M+GAP1-C*→	595	Candidatus Scalindua rubra	GCA_001723765.1	planctomycetes
OFT57549.1	GAP1-N2+GAP1-M+GAP1-C	polar_leader+TM+TM+TM→ TM+vWA(canonical)+Fibronectin-like_repeats+TM+beta-rich+lc_tail→ Tubulin+alpha_helical+??+??→ GAP_unknown1+TM+TM+GAP_unknown2→ HRDC-like?+Fibronectin-like_repeats→ GAP1-N2+GAP1-M+GAP1-C*→ beta-rich+vWA(canonical)→??+DO-GTPase2.2→ polar_leader+TM+TM+TM→	611	Corynebacterium sp. HMSC05E07	GCA_001807225.1	actinobacteria
OFU52656.1	GAP1-N2+GAP1-M+GAP1-C	polar_leader+TM+TM+TM→ TM+vWA(canonical)+Fibronectin-like_repeats+TM+beta-rich+lc_tail→ Tubulin+alpha_helical+??+??→ GAP_unknown1+TM+TM+GAP_unknown2→ HRDC-like?+Fibronectin-like_repeats→ GAP1-N2+GAP1-M+GAP1-C*→ beta-rich+vWA(canonical)→??+DO-GTPase2.2→ polar_leader+TM+TM+TM→	854	Corynebacterium sp. HMSC11E11	GCA_001807025.1	actinobacteria
OGV44577.1	GAP1-N2+GAP1-M+TM+GAP1-C	FHA-like+??+ZnR+ZnR+DO-GTPase2.2+??→ GAP1-N2+GAP1-M+TM+GAP1-C*→	747	Lentisphaerae bacterium GWF2_57_35	GCA_001804865.1	lentisphaerae
OGV69332.1	GAP1-N2+GAP1-M	GAP1-N2+GAP1-M*→??+TM+??+beta-rich??→	279	Lentisphaerae bacterium RIFOXYA12_64_32	GCA_001803235.1	lentisphaerae
OHB70404.1	GAP1-N2+GAP1-M+GAP1-C	FGS-fold_domain→ ZnR+DO-GTPase2.1→ GAP1-N2+GAP1-M+GAP1-C*→	472	Planctomycetes bacterium RBG_13_63_9	GCA_001828265.1	planctomycetes
OHB78921.1	GAP1-N2+GAP1-M+GAP1-C	FGS-fold_domain→ ZnR+DO-GTPase2.1→ GAP1-N2+GAP1-M+GAP1-C(frag)*→	318	Planctomycetes bacterium RBG_16_64_10	GCA_001828305.1	planctomycetes
OHB82388.1	GAP1-N2+GAP1-M+GAP1-C	ZnR+DO-GTPase2.1→ GAP1-N2+GAP1-M+GAP1-C*→	456	Planctomycetes bacterium RBG_16_64_12	GCA_001828335.1	planctomycetes
OHE80810.1	GAP1-N2+GAP1-M+TM+GAP1-C	GAP1-N2+GAP1-M+TM+GAP1-C*→	912	Verrucomicrobia bacterium GWF2_62_7	GCA_001831115.1	verrucomicrobia
OHV41104.1	GAP1-N2+GAP1-M+GAP1-C+ZnR+DO-GTPase2	GAP1-N2+GAP1-M+GAP1-C+ZnR+DO-GTPase2*→	984	Frankia sp. Cc1.17	GCA_001854655.1	actinobacteria
OJW24960.1	GAP1-N2+GAP1-M+GAP1-C	ZnR+DO-GTPase2.2→ GAP1-N2+GAP1-M+GAP1-C*→	1040	Planctomycetales bacterium 71-10	GCA_001898385.1	planctomycetes
OJW26983.1	GAP1-N2+GAP1-M	GAP1-N2+GAP1-M*→	222	Planctomycetales bacterium 71-10	GCA_001898385.1	planctomycetes
OKH62121.1	GAP1-N2+GAP1-M+GAP1-C	HRDC-like?+Fibronectin-like_repeats→ GAP1-N2+GAP1-M+GAP1-C(frag)*→	396	Mycobacterium sp. SWH-M5	GCA_001905305.1	actinobacteria
OKI26354.1	GAP1-N2+GAP1-M+GAP1-C	HSP70→||←?||GrpE→ HSP70→ TPR_containing_alpha_helical+Fibronectin-like_repeats→ ZnR+ZnR+DO-GTPase2.2→ GAP1-N2+GAP1-M+GAP1-C*→ TM+TM+TM+TM+ZnR+ZnR+DO-GTPase2.2→	781	Saccharothrix sp. CB00851	GCA_001905065.1	actinobacteria
OKI77275.1	GAP1-N2+GAP1-M+GAP1-C	GAP1-N2+GAP1-M+GAP1-C*→	817	Streptomyces sp. TSRI0395	GCA_001905525.1	actinobacteria
OKJ17675.1	GAP1-N2+GAP1-M+GAP1-C	TM+TM+TM+TM+ZnR+ZnR+DO-GTPase2.2+TM→ vWA(canonical)→ PP2C→ Pkinase+ZnR+beta-rich→||←?←?←?||GAP1-N2+GAP1-M+GAP1-C*→	807	Streptomyces sp. CB00316	GCA_001905595.1	actinobacteria
OKJ37923.1	GAP1-N2+GAP1-M+GAP1-C	GAP1-N2+GAP1-M+GAP1-C*→	830	Streptomyces sp. CB02366	GCA_001905905.1	actinobacteria

acc	architecture	operon	len	species	gca	taxend
OKX84744.1	GAP1-N2+GAP1-M+GAP1-C	polar_leader+TM+TM+TM→ TM+vWA(canonical)+Fibronectin-like_repeats+TM+beta-rich+lc_tail→ Tubulin+alpha_helical+??+??→ GAP_unknown1+TM+TM+GAP_unknown2→ HRDC-like?+Fibronectin-like_repeats→ GAP1-N2+GAP1-M+GAP1-C*→ beta-rich+vWA(canonical)→??+DO-GTPase2.2→	661	Corynebacterium glutamicum	GCA_001912755.2	actinobacteria
OKX87375.1	GAP1-N2+GAP1-M+GAP1-C	polar_leader+TM+TM+TM→ TM+vWA(canonical)+Fibronectin-like_repeats+TM+beta-rich+lc_tail→ Tubulin+alpha_helical+??+??→ GAP_unknown1+TM+TM+GAP_unknown2→ HRDC-like?+Fibronectin-like_repeats→ GAP1-N2+GAP1-M+GAP1-C*→ beta-rich+vWA(canonical)→??+DO-GTPase2.2→	661	Corynebacterium glutamicum	GCA_001912725.1	actinobacteria
OLA86335.1	GAP1-N2+GAP1-M+TM+GAP1-C	FHA-like?+ZnR+ZnR+DO-GTPase2.2→ GAP1-N2+GAP1-M+TM+GAP1-C*→	443	Verrucomicrobia bacterium CAG:312_58_20	GCA_001917305.1	verrucomicrobia
OME85740.1	GAP1-N2+GAP1-M+GAP1-C+polar_tail+TM+beta-rich	TM+vWA(canonical)+YtKA-like+TM+??→ Tubulin+coiled_coil+??→ Tubulin+coiled_coil+HTH?+??→ TM+TM+TM+vWA(canonical)+TM+TM+TM+polar_tail→ Fibronectin-like_repeats+??→ ZnR+ZnR+DO-GTPase2.2+ZnR+ZnR→ GAP1-N2+GAP1-M+GAP1-C+polar_tail+TM+beta-rich*→	1046	Paenibacillus pabuli	GCA_001955985.1	firmicutes
OMF04519.1	GAP1-N2+GAP1-M+GAP1-C+polar_tail+TM+beta-rich	TM+vWA(canonical)+YtKA-like+TM+??→ Tubulin+coiled_coil+??→ Tubulin+coiled_coil+HTH?+??→ TM+TM+TM+vWA(canonical)+TM+TM+TM+polar_tail→ Fibronectin-like_repeats+??→ ZnR+ZnR+DO-GTPase2.2+ZnR+ZnR→ GAP1-N2+GAP1-M+GAP1-C+polar_tail+TM+beta-rich*→	1073	Paenibacillus amylolyticus	GCA_001956035.1	firmicutes
OMG48224.1	GAP1-N2+GAP1-M+GAP1-C+polar_tail+TM+beta-rich	TM+TM+TM+vWA(canonical)+TM+TM+TM+polar_tail→ Fibronectin-like_repeats+??→ ZnR+ZnR+DO-GTPase2.2+ZnR+ZnR→ GAP1-N2+GAP1-M+GAP1-C+polar_tail+TM+beta-rich*→	1139	Paenibacillus macerans	GCA_001956835.1	firmicutes
OMO95906.1	TM+TM+TM+vWA+TM+TM+TM+ZnR+GTPase+GAP1-N2+GAP1-M+GAP1-C+ATP_grasp+ATP_grasp+Rossmann-like+thioredoxin_fold	TM+TM+TM+vWA+TM+TM+TM+ZnR+GTPase+GAP1-N2+GAP1-M+GAP1-C+ATP_grasp+ATP_grasp+Rossmann-like+thioredoxin_fold*→	3618	Corchorus olitorius	GCA_001974825.1	viridiplantae
ONI53537.1	GAP1-N2+GAP1-M+GAP1-C	vWA(canonical)→ PP2C→ Pkinase+ZnR+beta-rich→||←?←?←?||GAP1-N2+GAP1-M+GAP1-C*→	807	Streptomyces sp. IB2014 011-1	GCA_001983595.1	actinobacteria
OQY57022.1	GAP1-N2+GAP1-M+GAP1-C	GAP1-N2+GAP1-M+GAP1-C*→ TM+TM+TM+TM+ZnR+ZnR+DO-GTPase2.2→||←HSP70←GrpE	844	Beggiatoa sp. 4572_84	GCA_002085445.1	gammaproteobacteria
ORB87026.1	GAP1-N2+GAP1-M+GAP1-C	TM+DUF4190+TM→ TM+DUF4190+TM→ polar_leader+TM+TM+TM→ TM+vWA(canonical)+Fibronectin-like_repeats+TM+beta-rich+lc_tail→ Tubulin+alpha_helical+??+??→ GAP_unknown1+TM+TM+GAP_unknown2→ HRDC-like?+Fibronectin-like_repeats→ GAP1-N2+GAP1-M+GAP1-C*→ beta-rich+vWA(canonical)→??+DO-GTPase2.2→	818	Mycobacterium kansasii	GCA_002086865.1	actinobacteria
ORB92406.1	GAP1-N2+GAP1-M+GAP1-C	TM+DUF4190+TM→ TM+DUF4190+TM→ polar_leader+TM+TM+TM→ TM+vWA(canonical)+Fibronectin-like_repeats+TM+beta-rich+lc_tail→ Tubulin+alpha_helical+??+??→ GAP_unknown1+TM+TM+GAP_unknown2→ HRDC-like?+Fibronectin-like_repeats→ GAP1-N2+GAP1-M+GAP1-C*→ beta-rich+vWA(canonical)→??+DO-GTPase2.2→	818	Mycobacterium kansasii	GCA_002086855.1	actinobacteria
ORB97806.1	GAP1-N2+GAP1-M+GAP1-C	TM+DUF4190+TM→ TM+DUF4190+TM→ polar_leader+TM+TM+TM→ TM+vWA(canonical)+Fibronectin-like_repeats+TM+beta-rich+lc_tail→ Tubulin+alpha_helical+??+??→ GAP_unknown1+TM+TM+GAP_unknown2→ HRDC-like?+Fibronectin-like_repeats→ GAP1-N2+GAP1-M+GAP1-C*→ beta-rich+vWA(canonical)→??+DO-GTPase2.2→	818	Mycobacterium kansasii	GCA_002086925.1	actinobacteria
ORC04457.1	GAP1-N2+GAP1-M+GAP1-C	TM+DUF4190+TM→ TM+DUF4190+TM→ polar_leader+TM+TM+TM→ TM+vWA(canonical)+Fibronectin-like_repeats+TM+beta-rich+lc_tail→ Tubulin+alpha_helical+??+??→ GAP_unknown1+TM+TM+GAP_unknown2→ HRDC-like?+Fibronectin-like_repeats→ GAP1-N2+GAP1-M+GAP1-C*→ beta-rich+vWA(canonical)→??+DO-GTPase2.2→	734	Mycobacterium kansasii	GCA_002086935.1	actinobacteria
OSC69297.1	GAP1-N2+GAP1-M+GAP1-C	GAP1-N2+GAP1-M+GAP1-C*→	817	Streptomyces sp. BF-3	GCA_002104865.1	actinobacteria
OUT55991.1	GAP1-N2+GAP1-M+TM+GAP1-C	ZnR+ZnR+DO-GTPase2.2→ GAP1-N2+GAP1-M+TM+GAP1-C*→??+TM+??+beta-rich??→ GYF+GYF+TM+TM+TM→	691	Rhodopirellula	GCA_002167455.1	planctomycetes
OUT61367.1	GAP1-N2+GAP1-M	FGS-fold_domain→ ZnR+DO-GTPase2.1→ GAP1-N2+GAP1-M*→	256	Rhodopirellula sp. TMED11	GCA_002167455.1	planctomycetes
OUU82773.1	GAP1-N2+GAP1-M+low_complexity+TM+TM	FHA-like?+ZnR+ZnR+DO-GTPase2.2→ GAP1-N2+GAP1-M+low_complexity+TM+TM*→	524	Verrucomicrobia bacterium TMED71	GCA_002170515.1	verrucomicrobia
OUU88014.1	GAP1-N2+GAP1-M+TM+beta-rich+??	FHA-like?+ZnR+ZnR+DO-GTPase2.2→ GAP1-N2+GAP1-M+TM+beta-rich+??*→??+TM+??→	1016	Verrucomicrobiaceae bacterium TMED76	GCA_002170435.1	verrucomicrobia
OWP54298.1	GAP1-N2+GAP1-M+GAP1-C	TM+TM+TM+TM+TM+TM+DO-GTPase2.1→ GAP1-N2+GAP1-M+GAP1-C*→??→ TM_region+DO-GTPase1→	556	Cuniculiplasma sp. C_DKE	GCA_002204655.1	euryarchaeota
OXY86693.1	GAP1-N2+GAP1-M+GAP1-C	GAP1-N2+GAP1-M+GAP1-C*→	807	Streptomyces sp. 2R	GCA_002242735.1	actinobacteria
OYT35071.1	GAP1-N2+GAP1-M	GAP1-N2+GAP1-M*→	330	archaeon ex4484_74	GCA_002254715.1	archaea
OYV90204.1	??+GAP1-N2+GAP1-M+??+TM+TM	??+GAP1-N2+GAP1-M+??+TM+TM*→	1329	Planctomycetia bacterium 21-64-5	GCA_002279215.1	planctomycetes
OYW70936.1	GAP1-N2+GAP1-M+TM	FHA-like?+ZnR+ZnR+DO-GTPase2.2→ GAP1-N2+GAP1-M+TM*→	441	Verrucomicrobia bacterium 12-59-8	GCA_002279765.1	verrucomicrobia
OYW74544.1	GAP1-N2+GAP1-M+TM+GAP1-C	FHA-like?+ZnR+ZnR+DO-GTPase2.2→ GAP1-N2+GAP1-M+TM+GAP1-C*→	918	Verrucomicrobia bacterium 12-59-8	GCA_002279765.1	verrucomicrobia
OZQ64785.1	GAP1-N2+GAP1-M+GAP1-C+polar_tail+TM+beta-rich	ZnR+ZnR+DO-GTPase2.2+ZnR+ZnR→ GAP1-N2+GAP1-M+GAP1-C+polar_tail+TM+beta-rich*→	1079	Paenibacillus taichungensis	GCA_002264305.1	firmicutes
PAU68390.1	GAP1-N2+GAP1-M+GAP1-C	TM+TM+TM+TM+ZnR+ZnR+DO-GTPase2.2→ GrpE→ HSP70→ HSP70+vWA(canonical)→ DnaJ+DnaJ+ZnR+ZnR+coiled_coil+Fibronectin-like_repeats→ ZnR+ZnR+DO-GTPase2.2→ GAP1-N2+GAP1-M+GAP1-C*→	849	Bifidobacterium criceti	GCA_002286935.1	actinobacteria
PAW85721.1	GAP1-N2+GAP1-M+TM+GAP1-C	ZnR+ZnR+DO-GTPase2.2→ GAP1-N2+GAP1-M+TM+GAP1-C*→	713	Opitutae bacterium Tous-C2FEB	GCA_002304375.1	verrucomicrobia
PAZ01797.1	GAP1-N2+GAP1-M+TM+GAP1-C	FHA-like?+ZnR+ZnR+DO-GTPase2.2→ GAP1-N2+GAP1-M+TM+GAP1-C*→	713	Opitutae bacterium AMD-G3	GCA_002290585.1	verrucomicrobia
PAZ04443.1	GAP1-N2+GAP1-M+TM+GAP1-C	FHA-like?+ZnR+ZnR+DO-GTPase2.2→ GAP1-N2+GAP1-M+TM+GAP1-C*→	709	Opitutae bacterium AMD-G1	GCA_002290595.1	verrucomicrobia
PHS09131.1	GAP1-N2+GAP1-M+GAP1-C	FGS-fold_domain→ ZnR+DO-GTPase2.1→ GAP1-N2+GAP1-M+GAP1-C*→	464	Blastopirellula sp.	GCA_002733575.1	planctomycetes
PHX68902.1	GAP1-N2+GAP1-M+TM+GAP1-C	FHA-like?+ZnR+ZnR+DO-GTPase2.2→ GAP1-N2+GAP1-M+TM+GAP1-C*→	709	Opitutae bacterium	GCA_002737185.1	verrucomicrobia
PHX78035.1	GAP1-N2+GAP1-M+TM	ZnR+ZnR+DO-GTPase2.2→ GAP1-N2+GAP1-M+TM*→	359	Planctomycetaceae bacterium	GCA_002737585.1	planctomycetes
PHX79082.1	GAP1-N2+GAP1-M+TM+GAP1-C	FHA-like?+ZnR+ZnR+DO-GTPase2.2→ GAP1-N2+GAP1-M+TM+GAP1-C*→	709	Opitutae bacterium	GCA_002737235.1	verrucomicrobia
PHX79721.1	GAP1-N2+GAP1-M+TM+GAP1-C	FHA-like?+ZnR+ZnR+DO-GTPase2.2→ GAP1-N2+GAP1-M+TM+GAP1-C*→	711	Opitutae bacterium	GCA_002737235.1	verrucomicrobia
PHX79760.1	GAP1-N2+GAP1-M+TM+GAP1-C	FHA-like?+ZnR+ZnR+DO-GTPase2.2→ GAP1-N2+GAP1-M+TM+GAP1-C*→	710	Opitutae bacterium	GCA_002737235.1	verrucomicrobia
PIE36367.1	GAP1-N2+GAP1-M+GAP1-C	GAP1-N2+GAP1-M+GAP1-C*→	586	candidate division KSB3 bacterium	GCA_002748445.1	bacteria
PIG42998.1	GAP1-N2+GAP1-M+GAP1-C	TPR_containing_alpha_helical+Fibronectin-like_repeats→ ZnR+ZnR+DO-GTPase2.2→ GAP1-N2+GAP1-M+GAP1-C*→ TM+TM+TM+TM+ZnR+ZnR+DO-GTPase2.2+TM→?→ vWA(canonical)→ PP2C→ Pkinase+ZnR+beta-rich→	857	Streptomyces sp. 61	GCA_002754535.1	actinobacteria
PIH58188.1	GAP1-N2+GAP1-M+GAP1-C+polar_tail+TM+beta-rich	TM+vWA(canonical)+YtKA-like+TM+??→ Tubulin+coiled_coil+??→ Tubulin+coiled_coil+HTH?+??→ TM+TM+TM+vWA(canonical)+TM+TM+TM+polar_tail→ Fibronectin-like_repeats+??→ ZnR+ZnR+DO-GTPase2.2+ZnR+ZnR→ GAP1-N2+GAP1-M+GAP1-C+polar_tail+TM+beta-rich*→	1056	Paenibacillus sp. LK1	GCA_002750415.1	firmicutes
PIS64671.1	GAP1-N2+GAP1-M+GAP1-C	polar_leader+TM+TM+TM→ PSE→ PSE→ GAP_unknown1+TM+TM+GAP_unknown2→ HRDC-like?+Fibronectin-like_repeats→ HRDC-like?+Fibronectin-like_repeats→ GAP1-N2+GAP1-M+GAP1-C(frag)*→?→ beta-rich+vWA(canonical)→??+DO-GTPase2.2→ polar_leader+TM+TM+TM→	199	Corynebacterium striatum	GCA_002775095.1	actinobacteria
PIS66423.1	GAP1-N2+GAP1-M+GAP1-C	polar_leader+TM+TM+TM→ PSE→ PSE→ GAP_unknown1+TM+TM+GAP_unknown2→ HRDC-like?+Fibronectin-like_repeats→ GAP1-N2+GAP1-M+GAP1-C*→ beta-rich+vWA(canonical)→??+DO-GTPase2.2→ polar_leader+TM+TM+TM→	496	Corynebacterium striatum	GCA_002775105.1	actinobacteria
PJF21892.1	GAP1-N2+GAP1-M+GAP1-C	GAP1-N2+GAP1-M+GAP1-C*→	794	Chloroflexi bacterium	GCA_002794505.1	chloroflexi
PJF22367.1	GAP1-N2+GAP1-M+TM+GAP1-C	GAP1-N2+GAP1-M+TM+GAP1-C*→	560	Chloroflexi bacterium	GCA_002794505.1	chloroflexi
PJF23276.1	GAP1-N2+GAP1-M+GAP1-C	GAP1-N2+GAP1-M+GAP1-C*→ Pkinase+helical_region+TM_region→	1045	Chloroflexi bacterium	GCA_002794505.1	chloroflexi
PJF38527.1	GAP1-N2+GAP1-M	GAP1-N2+GAP1-M*→	220	Chloroflexi bacterium	GCA_002794585.1	chloroflexi
PJF41071.1	GAP1-N2+GAP1-M+GAP1-C	GAP1-N2+GAP1-M+GAP1-C*→	915	Chloroflexi bacterium	GCA_002794595.1	chloroflexi

acc	architecture	operon	len	species	gca	taxend
PJF45050.1	GAP1-N2+GAP1-M+GAP1-C	GAP1-N2+GAP1-M+GAP1-C*→	819	Chloroflexi bacterium	GCA_002794585.1	chloroflexi
PJT46684.1	GAP1-N2+GAP1-M+GAP1-C	GAP1-N2+GAP1-M+GAP1-C*→	789	Streptomyces albidoflavus	GCA_002808645.1	actinobacteria
PKB09493.1	GAP1-N2+GAP1-M+GAP1-C	GAP1-N2+GAP1-M+GAP1-C*→	622	Streptomyces sp. Ag109_G2-1	GCA_002813225.1	actinobacteria
PKK14792.1	GAP1-N2+GAP1-M+GAP1-C	GrpE→ HSP70→ TPR_containing_alpha_helical+Fibronectin-like_repeats→ ZnR+ZnR+DO-GTPase2.2→ GAP1-N2+GAP1-M+GAP1-C*→ TM+TM+TM+TM+ZnR+ZnR+DO-GTPase2.2+TM→	784	Thermomonospora sp. CIF 1	GCA_001896795.2	actinobacteria
PKQ21887.1	GAP1-N2+GAP1-M+low_complexity	ZnR+ZnR+DO-GTPase2.2→ GAP1-N2+GAP1-M+low_complexity*→	403	Actinobacteria bacterium HGW-Actinobacteria-5	GCA_002843035.1	actinobacteria
PLS26070.1	GAP1-N2+GAP1-M+GAP1-C	TM+TM+TM+TM+ZnR+ZnR+DO-GTPase2.2→ GrpE→ HSP70→ HSP70+vWA(canonical)→ DnaJ+DnaJ+ZnR+ZnR+coiled_coil+Fibronectin-like_repeats→ ZnR+ZnR+DO-GTPase2.2→ GAP1-N2+GAP1-M+GAP1-C*→	968	Bifidobacterium parmae	GCA_002860365.1	actinobacteria
SBT88951.1	GAP1-N2+GAP1-M+GAP1-C	TPR_containing_alpha_helical+Fibronectin-like_repeats→ ZnR+ZnR+DO-GTPase2.2→ GAP1-N2+GAP1-M+GAP1-C*→	305	Streptomyces sp. DI166	GCA_900091315.1	actinobacteria
SCD96045.1	GAP1-N2+GAP1-M+GAP1-C	GAP1-N2+GAP1-M+GAP1-C*→	694	Streptomyces sp. BpilaLS-43	GCA_900091875.1	actinobacteria
SCE10320.1	GAP1-N2+GAP1-M+GAP1-C	GAP1-N2+GAP1-M+GAP1-C*→	239	Streptomyces sp. TverLS-915	GCA_900091755.1	actinobacteria
SCE16867.1	GAP1-M+GAP1-C	GAP1-N2+GAP1-M+GAP1-C→ GAP1-M+GAP1-C*→	707	Streptomyces sp. Termitarium-T10T-6	GCA_900091795.1	actinobacteria
SCE16889.1	GAP1-N2+GAP1-M+GAP1-C	GAP1-N2+GAP1-M+GAP1-C*→ GAP1-N2+GAP1-M+GAP1-C→	126	Streptomyces sp. Termitarium-T10T-6	GCA_900091795.1	actinobacteria
SCE26370.1	GAP1-M+GAP1-C	GAP1-M+GAP1-C*→	412	Streptomyces sp. DpondAA-F4a	GCA_900091715.1	actinobacteria
SCE41839.1	GAP1-N2+GAP1-M+GAP1-C	GAP1-N2+GAP1-M+GAP1-C*→	789	Streptomyces sp. IgraMP-1	GCA_900091895.1	actinobacteria
SCG07756.1	GAP1-N2+GAP1-M+GAP1-C	GAP1-N2+GAP1-M+GAP1-C*→	831	Streptomyces sp. Ncost-T6T-2b	GCA_900091965.1	actinobacteria
SCK36440.1	GAP1-N2+GAP1-M+GAP1-C	GAP1-N2+GAP1-M+GAP1-C*→	807	Streptomyces sp. ScaeMP-e48	GCA_900090155.1	actinobacteria
SDD36788.1	GAP1-N2+GAP1-M+GAP1-C+polar_tail+TM+beta-rich	TM+vWA(canonical)+YtKA-like+TM+??→ Tubulin+coiled_coil+??→ Tubulin+coiled_coil+HTH?+??→ TM+TM+TM+vWA(canonical)+TM+TM+TM+polar_tail→ Fibronectin-like_repeats+??→ ZnR+ZnR+DO-GTPase2.2+ZnR+ZnR→ GAP1-N2+GAP1-M+GAP1-C+polar_tail+TM+beta-rich*→	1062	Paenibacillus sp. CF095	GCA_900101225.1	firmicutes
SDM18502.1	GAP1-N2+GAP1-M+GAP1-C	Pkinase+TM→ TM→ ZnR+ZnR+DO-GTPase2.2→ GAP1-N2+GAP1-M+GAP1-C*→?→||←?←?||?→ Pkinase+low_complexity→	554	Geodermatophilus siccatus	GCA_900103785.1	actinobacteria
SDO47351.1	GAP1-N2+GAP1-M+GAP1-C	Pkinase+TM→ TM→ ZnR+ZnR+DO-GTPase2.2→ GAP1-N2+GAP1-M+GAP1-C*→?→||←?←?||?→ Pkinase+low_complexity→	553	Geodermatophilus sp. DSM 45219	GCA_900104025.1	actinobacteria
SDX59550.1	GAP1-N2+GAP1-M+GAP1-C	Pkinase+TM→ TM→ ZnR+ZnR+DO-GTPase2.2→ GAP1-N2+GAP1-M+GAP1-C*→?→||←?←?||?→ Pkinase+low_complexity→	556	Geodermatophilus africanus	GCA_900107105.1	actinobacteria
SED53293.1	GAP1-N2+GAP1-M+GAP1-C	GAP1-N2+GAP1-M+GAP1-C*→	813	Streptomyces griseus	GCA_900105705.1	actinobacteria
SEE68325.1	GAP1-N2+GAP1-M+GAP1-C	TPR_containing_alpha_helical+Fibronectin-like_repeats→ ZnR+ZnR+DO-GTPase2.2→ GAP1-N2+GAP1-M+GAP1-C*→ TM+TM+TM+TM+ZnR+ZnR+DO-GTPase2.2+TM→?→ vWA(canonical)→ PP2C→ Pkinase+ZnR+beta-rich→	895	Streptomyces sp. 3213.3	GCA_900105265.1	actinobacteria
SEF85085.1	GAP1-N2+GAP1-M+GAP1-C+polar_tail+TM+Cadherin-like	TM+vWA(canonical)+YtKA-like+TM+??→ Tubulin+coiled_coil+??→ Tubulin+coiled_coil+HTH?+??→ TM+TM+TM+vWA(canonical)+TM+TM+TM+polar_tail→ Fibronectin-like_repeats+??→ ZnR+ZnR+DO-GTPase2.2+ZnR+ZnR→ GAP1-N2+GAP1-M+GAP1-C+polar_tail+TM+Cadherin-like*→	965	Paenibacillus sp. UNC499MF	GCA_900107975.1	firmicutes
SEG04795.1	GAP1-N2+GAP1-M+GAP1-C	GrpE→ HSP70→ TPR_containing_alpha_helical+Fibronectin-like_repeats→ ZnR+ZnR+DO-GTPase2.2→ GAP1-N2+GAP1-M+GAP1-C*→ TM+TM+TM+TM+ZnR+ZnR+DO-GTPase2.2+TM→	791	Actinomadura echinospora	GCA_900108175.1	actinobacteria
SEH00144.1	GAP1-N2+GAP1-M+GAP1-C	GrpE→ HSP70→ TPR_containing_alpha_helical+Fibronectin-like_repeats→ ZnR+ZnR+DO-GTPase2.2→ GAP1-N2+GAP1-M+GAP1-C*→ TM+TM+TM+TM+ZnR+ZnR+DO-GTPase2.2+TM→	755	Nonomuraea solani	GCA_900108335.1	actinobacteria
SEK51062.1	GAP1-N2+GAP1-M+GAP1-C+polar_tail+TM+beta-rich	TM+vWA(canonical)+YtKA-like+TM+??→ Tubulin+coiled_coil+??→ Tubulin+coiled_coil+HTH?+??→ TM+TM+TM+vWA(canonical)+TM+TM+TM+polar_tail→ Fibronectin-like_repeats+??→ ZnR+ZnR+DO-GTPase2.2+ZnR+ZnR→ GAP1-N2+GAP1-M+GAP1-C+polar_tail+TM+beta-rich*→	1059	Paenibacillus sp. OK003	GCA_900109515.1	firmicutes
SEO61305.1	GAP1-N2+GAP1-M+GAP1-C+polar_tail+TM+beta-rich	TM+vWA(canonical)+YtKA-like+TM+??→ Tubulin+coiled_coil+??→ Tubulin+coiled_coil+HTH?+??→ TM+TM+TM+vWA(canonical)+TM+TM+TM+polar_tail→ Fibronectin-like_repeats+??→ ZnR+ZnR+DO-GTPase2.2+ZnR+ZnR→ GAP1-N2+GAP1-M+GAP1-C+polar_tail+TM+beta-rich*→	1058	Paenibacillus sp. OK076	GCA_900110055.1	firmicutes
SES36605.1	GAP1-N2+GAP1-M	CASPASE+TPR_region→?→ GAP1-N2+GAP1-M*→	182	Actinokineospora terrae	GCA_900111175.1	actinobacteria
SET23369.1	GAP1-N2+GAP1-M+GAP1-C	Pkinase+TM→ TM→ ZnR+ZnR+DO-GTPase2.2→ GAP1-N2+GAP1-M+GAP1-C*→?→||←?←?||?→ Pkinase+low_complexity→	552	Geodermatophilus poikilotrophus	GCA_900111455.1	actinobacteria
SEU01591.1	GAP1-N2+GAP1-M+GAP1-C+polar_tail+TM+beta-rich	TM+vWA(canonical)+YtKA-like+TM+??→ Tubulin+coiled_coil+??→ Tubulin+coiled_coil+HTH?+??→ TM+TM+TM+vWA(canonical)+TM+TM+TM+polar_tail→ Fibronectin-like_repeats+??→ ZnR+ZnR+DO-GTPase2.2+ZnR+ZnR→ GAP1-N2+GAP1-M+GAP1-C+polar_tail+TM+beta-rich*→	1013	Paenibacillus sp. NFR01	GCA_900111565.1	firmicutes
SFL38747.1	GAP1-N2+GAP1-M+GAP1-C	Pkinase+TM→ TM→ ZnR+ZnR+DO-GTPase2.2→ GAP1-N2+GAP1-M+GAP1-C*→	564	Geodermatophilus ruber	GCA_900114385.1	actinobacteria
SFN59903.1	GAP1-N2+GAP1-M+GAP1-C	GrpE→ HSP70→ TPR_containing_alpha_helical+Fibronectin-like_repeats→ ZnR+ZnR+DO-GTPase2.2→ GAP1-N2+GAP1-M+GAP1-C*→ TM+TM+TM+TM+ZnR+ZnR+DO-GTPase2.2+TM→	778	Actinomadura madurae	GCA_900115095.1	actinobacteria
SHN70262.1	GAP1-N2+GAP1-M+GAP1-C	Pkinase+TM→ TM→ ZnR+ZnR+DO-GTPase2.2→ GAP1-N2+GAP1-M+GAP1-C*→?→||←?←?||?→ Pkinase+low_complexity→	552	Geodermatophilus obscurus	GCA_900143215.1	actinobacteria
SHX49947.1	GAP1-N2+GAP1-M+GAP1-C	HSP70→ TPR_containing_alpha_helical+Fibronectin-like_repeats→ ZnR+ZnR+DO-GTPase2.2→ GAP1-N2+GAP1-M+GAP1-C*→	697	Mycobacteroides abscessus subsp. abscessus	GCA_900134035.1	actinobacteria
SIM65459.1	GAP1-N2+GAP1-M+GAP1-C	TM+TM+TM+TM+TM+TM+DO-GTPase2.1→ GAP1-N2+GAP1-M+GAP1-C*→??→ TM_region+DO-GTPase1→	556	Cuniculiplasma divulgatum	GCA_900083515.1	euryarchaeota
SIO30381.1	GAP1-N2+GAP1-M+GAP1-C	DO-GTPase2.1+TM→ GAP1-N2+GAP1-M+GAP1-C*→	484	Singulisphaera sp. GP187	GCA_900129635.1	planctomycetes
SIO90030.1	GAP1-N2+GAP1-M+GAP1-C	TPR_containing_alpha_helical+Fibronectin-like_repeats→ ZnR+ZnR+DO-GTPase2.2→ GAP1-N2+GAP1-M+GAP1-C*→ TM+TM+TM+TM+ZnR+ZnR+DO-GTPase2.2→	674	Nocardiopsis sp. JB363	GCA_900143625.1	actinobacteria
SJM69463.1	GAP1-N2+GAP1-M+GAP1-C	TM+TM+TM→ TM+vWA(canonical)+Fibronectin-like_repeats+TM+beta-rich+lc_tail→ Tubulin+alpha_helical+??+??→ GAP_unknown1+TM+TM+GAP_unknown2→ HRDC-like?+Fibronectin-like_repeats→ GAP1-N2+GAP1-M+GAP1-C*→ beta-rich+vWA(canonical)→??+DO-GTPase2.2→	654	Corynebacterium glutamicum	GCA_900163685.1	actinobacteria
SLL09269.1	GAP1-N2+GAP1-M+GAP1-C+polar_tail+TM+beta-rich	GAP1-N2+GAP1-M+GAP1-C+polar_tail+TM+beta-rich*→	746	Mycobacteroides abscessus subsp. abscessus	GCA_900132645.1	actinobacteria
SMF90090.1	GAP1-N2+GAP1-M+GAP1-C+polar_tail+TM+beta-rich	TM+vWA(canonical)+YtKA-like+TM+??→ Tubulin+coiled_coil+??→ Tubulin+coiled_coil+HTH?+??→ TM+TM+TM+vWA(canonical)+TM+TM+TM+polar_tail→ Fibronectin-like_repeats+??→ ZnR+ZnR+DO-GTPase2.2+ZnR+ZnR→ GAP1-N2+GAP1-M+GAP1-C+polar_tail+TM+beta-rich*→	1009	Paenibacillus uliginis N3/975	GCA_900177425.1	firmicutes
SNS80072.1	GAP1-N2+GAP1-M+GAP1-C	Pkinase+TM→ TM→ ZnR+ZnR+DO-GTPase2.2→ GAP1-N2+GAP1-M+GAP1-C*→ Pkinase+low_complexity→	553	Geodermatophilus pulveris	GCA_900188375.1	actinobacteria
SOC51163.1	GAP1-N2+GAP1-M+TM+GAP1-C+TM	Pkinase+TM_region→ ZnR+ZnR+DO-GTPase2.2→ GAP1-N2+GAP1-M+TM+GAP1-C+TM*→	652	Ornithinimicrobium pekingense	GCA_900221015.1	actinobacteria
WP_002646734.1	GAP1-N2+GAP1-M+GAP1-C	GrpE→ HSP70→ HSP70+vWA(canonical)→ DnaJ+DnaJ+DZnR+Fibronectin-like_repeats→ ZnR+ZnR+DO-GTPase2.2→ GAP1-N2+GAP1-M+GAP1-C*→?→ TM+TM+TM+TM+ZnR+ZnR+DO-GTPase2.2→	858	Gimesia maris	GCA_000181475.1	planctomycetes
WP_002648417.1	GAP1-N2+GAP1-M+TM+GAP1-C	GAP1-N2+GAP1-M+TM+GAP1-C→ ZnR+ZnR+DO-GTPase2.2→ GAP1-N2+GAP1-M+TM+GAP1-C*→??+TM+??+beta-rich??→ GYF+GYF+TM+TM+TM→	861	Gimesia maris	GCA_000181475.1	planctomycetes
WP_002648419.1	GAP1-N2+GAP1-M+TM+GAP1-C	GAP1-N2+GAP1-M+TM+GAP1-C*→ ZnR+ZnR+DO-GTPase2.2→ GAP1-N2+GAP1-M+TM+GAP1-C→??+TM+??+beta-rich??→ GYF+GYF+TM+TM+TM→	873	Gimesia maris	GCA_000181475.1	planctomycetes
WP_002655127.1	GAP1-N2+GAP1-M+GAP1-C	FGS-fold_domain→ ZnR+DO-GTPase2.1→ GAP1-N2+GAP1-M+GAP1-C*→	459	Blastopirellula marina	GCA_000153105.1	planctomycetes
WP_003334069.1	GAP1-N2+GAP1-M+GAP1-C+polar_tail+TM+beta-rich	TM+vWA(canonical)+YtKA-like+TM+??→ Tubulin+coiled_coil+??→ Tubulin+coiled_coil+HTH?+??→ TM+TM+TM+vWA(canonical)+TM+TM+TM+polar_tail→ Fibronectin-like_repeats+??→ ZnR+ZnR+DO-GTPase2.2+ZnR+ZnR→ GAP1-N2+GAP1-M+GAP1-C+polar_tail+TM+beta-rich*→	833	Brevibacillus laterosporus	GCA_000219535.3	firmicutes
WP_003342911.1	GAP1-N2+GAP1-M+GAP1-C+polar_tail+TM+beta-rich	TM+vWA(canonical)+YtKA-like+TM+??→ Tubulin+coiled_coil+??→ Tubulin+coiled_coil+HTH?+??→ TM+TM+TM+vWA(canonical)+TM+TM+TM+polar_tail→ Fibronectin-like_repeats+??→ ZnR+ZnR+DO-GTPase2.2+ZnR+ZnR→ GAP1-N2+GAP1-M+GAP1-C+polar_tail+TM+beta-rich*→	833	Brevibacillus laterosporus	GCA_000237005.2	firmicutes

acc	architecture	operon	len	species	gca	taxend
WP_003388640.1	GAP1-N2+GAP1-M+GAP1-C+polar_tail+TM+beta-rich	TM+vWA(canonical)+YtKA-like+TM+??→ Tubulin+coiled_coil+??→ Tubulin+coiled_coil+HTH?+??→ TM+TM+TM+vWA(canonical)+TM+TM+TM+polar_tail→ Fibronectin-like_repeats+??→ ZnR+ZnR+DO-GTPase2.2+ZnR+ZnR→ GAP1-N2+GAP1-M+GAP1-C+polar_tail+TM+beta-rich*→	898	Brevibacillus borstelensis	GCA_000353565.1	firmicutes
WP_003862914.1	GAP1-N2+GAP1-M+GAP1-C	polar_leader+TM+TM+TM→ TM+vWA(canonical)+Fibronectin-like_repeats+TM+beta-rich+lc_tail→ Tubulin+alpha_helical+??+??→ GAP_unknown1+TM+TM+GAP_unknown2→ HRDC-like?+Fibronectin-like_repeats→ GAP1-N2+GAP1-M+GAP1-C*→ beta-rich+vWA(canonical)→??+DO-GTPase2.2→	631	Corynebacterium	GCA_002243555.1	actinobacteria
WP_003883300.1	GAP1-N2+GAP1-M+GAP1-C	HRDC-like?+Fibronectin-like_repeats→ GAP1-N2+GAP1-M+GAP1-C*→??+DO-GTPase2.2→	820	Mycobacteriaceae	GCA_000416365.2	actinobacteria
WP_003884602.1	GAP1-M+GAP1-C	polar_leader+TM+TM+TM→ TM+vWA(canonical)+Fibronectin-like_repeats+TM+beta-rich+lc_tail→ Tubulin+alpha_helical+??+??→ GAP_unknown1+TM+TM+GAP_unknown2→ PSE→ PSE→ HRDC-like?+Fibronectin-like_repeats→ PSE→ GAP1-M+GAP1-C*→ beta-rich+vWA(canonical)→??+DO-GTPase2.2→ TM+DUF4190+TM+PknH_C→	699	Mycolicibacterium fortuitum	GCA_000295855.1	actinobacteria
WP_003891373.1	GAP1-N2+GAP1-M+GAP1-C	HRDC-like?+Fibronectin-like_repeats→ GAP1-N2+GAP1-M+GAP1-C*→ ZnR+ZnR+DO-GTPase2.2→	822	Mycolicibacterium smegmatis	-	actinobacteria
WP_003965489.1	GAP1-N2+GAP1-M+GAP1-C	GAP1-N2+GAP1-M+GAP1-C*→	813	Streptomyces sp. ACT-1	GCA_000177175.2	actinobacteria
WP_004074144.1	GAP1-N2+GAP1-M+GAP1-C	HSP70→ HSP70+vWA(canonical)→ DnaJ+DnaJ+ZnR+ZnR+coiled_coil+Fibronectin-like_repeats→ ZnR+ZnR+DO-GTPase2.2→ GAP1-N2+GAP1-M+GAP1-C*→?→?→ TM+TM+TM+TM+ZnR+ZnR+DO-GTPase2.2+TPR_region→	836	Clostridium sp. ASF502	GCA_000364245.1	firmicutes
WP_004942138.1	GAP1-N2+GAP1-M+GAP1-C	GAP1-N2+GAP1-M+GAP1-C*→	838	Streptomyces mobaraensis	GCA_000342125.1	actinobacteria
WP_005191940.1	GAP1-N2+GAP1-M+GAP1-C	polar_leader+TM+TM+TM→ TM+vWA(canonical)+Fibronectin-like_repeats+TM+beta-rich+lc_tail→ Tubulin+alpha_helical+??+??→ GAP_unknown1+TM+TM+GAP_unknown2→ HRDC-like?+Fibronectin-like_repeats→ GAP1-N2+GAP1-M+GAP1-C*→ beta-rich+vWA(canonical)→??+DO-GTPase2.2→	901	Gordonia amarae	GCA_000241345.2	actinobacteria
WP_005252622.1	GAP1-N2+GAP1-M+GAP1-C	polar_leader+TM+TM+TM→ TM+vWA(canonical)+Fibronectin-like_repeats+TM+beta-rich+lc_tail→ Tubulin+alpha_helical+??+??→ GAP_unknown1+TM+TM+GAP_unknown2→ HRDC-like?+Fibronectin-like_repeats→ GAP1-N2+GAP1-M+GAP1-C*→ beta-rich+vWA(canonical)→??+DO-GTPase2.2→	913	Rhodococcus opacus	GCA_000599545.1	actinobacteria
WP_005394499.1	GAP1-N2+GAP1-M+GAP1-C	GrpE→ HSP70→ TPR_containing_alpha_helical+Fibronectin-like_repeats→ ZnR+ZnR+DO-GTPase2.2→ GAP1-N2+GAP1-M+GAP1-C*→ TM+TM+TM+TM+ZnR+ZnR+DO-GTPase2.2→	668	Corynebacterium glucuronolyticum	GCA_000159595.1	actinobacteria
WP_005514168.1	GAP1-N2+GAP1-M+GAP1-C	polar_leader+TM+TM+TM→ TM+vWA(canonical)+Fibronectin-like_repeats+TM+beta-rich+lc_tail→ Tubulin+alpha_helical+??+??→ GAP_unknown1+TM+TM+GAP_unknown2→ HRDC-like?+Fibronectin-like_repeats→ GAP1-N2+GAP1-M+GAP1-C*→ beta-rich+vWA(canonical)→??+DO-GTPase2.2→	842	Rhodococcus hoagii	GCA_000164155.2	actinobacteria
WP_005522822.1	GAP1-N2+GAP1-M+GAP1-C	polar_leader+TM+TM+TM→ TM+vWA(canonical)+Fibronectin-like_repeats+TM+beta-rich+lc_tail→ Tubulin+alpha_helical+??+??→ GAP_unknown1+TM+TM+GAP_unknown2→ HRDC-like?+Fibronectin-like_repeats→ GAP1-N2+GAP1-M+GAP1-C*→ beta-rich+vWA(canonical)→??+DO-GTPase2.2→ polar_leader+TM+TM+TM→	523	Corynebacterium matruchotii	GCA_000158635.1	actinobacteria
WP_005524888.1	GAP1-N2+GAP1-M+GAP1-C	polar_leader+TM+TM+TM→ TM+vWA(canonical)+Fibronectin-like_repeats+TM+beta-rich+lc_tail→ Tubulin+alpha_helical+??+??→ GAP_unknown1+TM+TM+GAP_unknown2→ HRDC-like?+Fibronectin-like_repeats→ GAP1-N2+GAP1-M+GAP1-C*→ beta-rich+vWA(canonical)→??+DO-GTPase2.2→ polar_leader+TM+TM+TM→	523	Corynebacterium matruchotii	GCA_000175375.1	actinobacteria
WP_005530380.1	GAP1-N2+GAP1-M+GAP1-C	polar_leader+TM+TM+TM→ TM+vWA(canonical)+Fibronectin-like_repeats+TM+beta-rich+lc_tail→ Tubulin+alpha_helical+??+??→ GAP_unknown1+TM+TM+GAP_unknown2→ HRDC-like?+Fibronectin-like_repeats→ GAP1-N2+GAP1-M+GAP1-C*→ beta-rich+vWA(canonical)→??+DO-GTPase2.2→ polar_leader+TM+TM+TM→	532	Corynebacterium striatum	GCA_000159135.1	actinobacteria
WP_005830175.1	GAP1-N2+GAP1-M+GAP1-C+polar_tail+TM+beta-rich	Tubulin+coiled_coil+??→ PSE→ TM+TM+TM+vWA(canonical)+TM+TM+TM+polar_tail→ Fibronectin-like_repeats+??→ ZnR+ZnR+DO-GTPase2.2+ZnR+ZnR→ GAP1-N2+GAP1-M+GAP1-C+polar_tail+TM+beta-rich*→	834	Brevibacillus agri	GCA_000328345.1	firmicutes
WP_005883244.1	GAP1-N2+GAP1-M+GAP1-C	TM+vWA(canonical)+Fibronectin-like_repeats+TM+beta-rich+lc_tail→ Tubulin+alpha_helical+??+??→ GAP_unknown1+TM+TM+GAP_unknown2→ HRDC-like?+Fibronectin-like_repeats→ GAP1-N2+GAP1-M+GAP1-C*→?→??+DO-GTPase2.2→?→||←?←?←TM+TM+vWA(canonical)+TM←TM+TM+vWA(canonical)+TM	930	Brevibacterium mcbrellneri	GCA_000178455.1	actinobacteria
WP_005986730.1	GAP1-N2+GAP1-M+GAP1-C	GrpE→ HSP70→ TPR_containing_alpha_helical+Fibronectin-like_repeats→ ZnR+ZnR+DO-GTPase2.2→ GAP1-N2+GAP1-M+GAP1-C*→ TM+TM+TM+TM+ZnR+ZnR+DO-GTPase2.2→	808	Actinomyces graevenitzii	GCA_000239695.1	actinobacteria
WP_006128441.1	GAP1-N2+GAP1-M	-	790	Streptomyces filamentosus	GCA_000156455.1	actinobacteria
WP_006246539.1	GAP1-N2+GAP1-M+GAP1-C	GrpE→ HSP70→ TPR_containing_alpha_helical+Fibronectin-like_repeats→ ZnR+ZnR+DO-GTPase2.2→ GAP1-N2+GAP1-M+GAP1-C*→ TM+TM+TM+TM+ZnR+ZnR+DO-GTPase2.2→?→?→?→?→ HSP70→ vWA(canonical)→	796	Mycolicibacterium tusciae	GCF_000243415.2	actinobacteria
WP_006284418.1	GAP1-N2+GAP1-M+GAP1-C+polar_tail+TM+beta-rich	TM+vWA(canonical)+YtKA-like+TM+??→ Tubulin+coiled_coil+??→ Tubulin+coiled_coil+HTH?+??→ TM+TM+TM+vWA(canonical)+TM+TM+TM+polar_tail→ Fibronectin-like_repeats+??→ ZnR+ZnR+DO-GTPase2.2+ZnR+ZnR→ GAP1-N2+GAP1-M+GAP1-C+polar_tail+TM+beta-rich*→	956	Paenibacillus popilliae	GCA_000315235.1	firmicutes
WP_006350590.1	GAP1-N2+GAP1-M+GAP1-C	ZnR+ZnR+DO-GTPase2.2→ GAP1-N2+GAP1-M+GAP1-C*→ TM+TM+TM+TM+ZnR+ZnR+DO-GTPase2.2→	812	Streptomyces tsukubensis	GCA_000297155.2	actinobacteria
WP_006542074.1	GAP1-N2+GAP1-M+GAP1-C+ZnR+DO-GTPase2	GAP1-N2+GAP1-M+GAP1-C+ZnR+DO-GTPase2*→	970	Frankia sp. EUN1f	GCA_000177675.1	actinobacteria
WP_006548379.1	GAP1-N2+GAP1-M+GAP1-C	polar_leader+TM+TM+TM→ TM+vWA(canonical)+Fibronectin-like_repeats+TM+beta-rich+lc_tail→ Tubulin+alpha_helical+??+??→ GAP_unknown1+TM+TM+GAP_unknown2→ HRDC-like?+Fibronectin-like_repeats→ GAP1-N2+GAP1-M+GAP1-C*→?→??+DO-GTPase2.2→	891	Actinomyces urogenitalis	GCA_000159035.1	actinobacteria
WP_006552801.1	GAP1-N2+GAP1-M+GAP1-C	polar_leader+TM+TM+TM→ TM+vWA(canonical)+Fibronectin-like_repeats+TM+beta-rich+lc_tail→ Tubulin+alpha_helical+??+??→ PSE→ HRDC-like?+Fibronectin-like_repeats→ GAP1-N2+GAP1-M+GAP1-C*→ beta-rich+vWA(canonical)→??+DO-GTPase2.2→	909	Rhodococcus pyridinivorans	GCA_000236965.2	actinobacteria
WP_007062064.1	GAP1-N2+GAP1-M+GAP1-C	Fibronectin-like_repeats+??→ ZnR+ZnR+DO-GTPase2.2→ GAP1-N2+GAP1-M+GAP1-C*→?→ TM+TM+TM+TM+ZnR+ZnR+DO-GTPase2.2→ TM+vWA(canonical)+YtKA-like+TM+??→ Tubulin+coiled_coil+??→ Tubulin+coiled_coil+HTH?+??→ TM+TM+TM+vWA(canonical)+TM+TM+TM+polar_tail→ PSE→ ZnR+ZnR+DO-GTPase2.2+ZnR+ZnR→	794	Clostridium carboxidivorans	GCA_000175595.1	firmicutes
WP_007064688.1	GAP1-N2+GAP1-M	TM+TM+TM+TM+ZnR+ZnR+DO-GTPase2.2→ TM+vWA(canonical)+YtKA-like+TM+??→ Tubulin+coiled_coil+??→ Tubulin+coiled_coil+HTH?+??→ TM+TM+TM+vWA(canonical)+TM+TM+TM+polar_tail→ PSE→ ZnR+ZnR+DO-GTPase2.2+ZnR+ZnR→ GAP1-N2+GAP1-M*→	369	Clostridium carboxidivorans	GCA_001038625.1	firmicutes
WP_007083299.1	GAP1-N2+GAP1-M+GAP1-C+polar_tail+TM+beta-rich	TM+vWA(canonical)+YtKA-like+TM+??→ Tubulin+coiled_coil+??→ Tubulin+coiled_coil+HTH?+??→ TM+TM+TM+vWA(canonical)+TM+TM+TM+polar_tail→ Fibronectin-like_repeats+??→ ZnR+ZnR+DO-GTPase2.2+ZnR+ZnR→ GAP1-N2+GAP1-M+GAP1-C+polar_tail+TM+beta-rich*→	928	Bacillus bataviensis	GCA_000307875.1	firmicutes
WP_007166499.1	GAP1-N2+GAP1-M+GAP1-C	TM+DUF4190+TM→ TM+DUF4190+TM→ polar_leader+TM+TM+TM→ TM+vWA(canonical)+Fibronectin-like_repeats+TM+beta-rich+lc_tail→ Tubulin+alpha_helical+??+??→ GAP_unknown1+TM+TM+GAP_unknown2→ HRDC-like?+Fibronectin-like_repeats→ GAP1-N2+GAP1-M+GAP1-C(frag)*→ beta-rich+vWA(canonical)→??+DO-GTPase2.2→	364	Mycobacterium	GCA_000164135.1	actinobacteria
WP_007319238.1	GAP1-N2+GAP1-M+GAP1-C	polar_leader+TM+TM+TM→ TM+vWA(canonical)+Fibronectin-like_repeats+TM+beta-rich+lc_tail→ Tubulin+alpha_helical+??+??→ GAP_unknown1+TM+TM+GAP_unknown2→ HRDC-like?+Fibronectin-like_repeats→ GAP1-N2+GAP1-M+GAP1-C*→ beta-rich+vWA(canonical)→??+DO-GTPase2.2→	890	Gordonia effusa	GCA_000241305.2	actinobacteria
WP_007320746.1	GAP1-N2+GAP1-M+GAP1-C	polar_leader+TM+TM+TM→ TM+vWA(canonical)+Fibronectin-like_repeats+TM+beta-rich+lc_tail→ Tubulin+alpha_helical+??+??→ GAP_unknown1+TM+TM+GAP_unknown2→ HRDC-like?+Fibronectin-like_repeats→ GAP1-N2+GAP1-M+GAP1-C*→ beta-rich+vWA(canonical)→??+DO-GTPase2.2→	938	Gordonia araii	GCA_000241265.2	actinobacteria
WP_007430267.1	GAP1-N2+GAP1-M+GAP1-C+polar_tail+TM+beta-rich	Tubulin+coiled_coil+??→ PSE→ TM+TM+TM+vWA(canonical)+TM+TM+TM+polar_tail→ Fibronectin-like_repeats+??→ ZnR+ZnR+DO-GTPase2.2+ZnR+ZnR→ GAP1-N2+GAP1-M+GAP1-C+polar_tail+TM+beta-rich*→	1000	Paenibacillus sp. Aloe-11	GCA_000245715.1	firmicutes
WP_007451403.1	GAP1-N2+GAP1-M	-	821	Streptomyces sp. W007	GCA_000239075.2	actinobacteria
WP_007724671.1	GAP1-N2+GAP1-M+GAP1-C+polar_tail+TM+beta-rich	TM+vWA(canonical)+YtKA-like+TM+??→ Tubulin+coiled_coil+??→ Tubulin+coiled_coil+HTH?+??→ TM+TM+TM+vWA(canonical)+TM+TM+TM+polar_tail→ Fibronectin-like_repeats+??→ ZnR+ZnR+DO-GTPase2.2+ZnR+ZnR→ GAP1-N2+GAP1-M+GAP1-C+polar_tail+TM+beta-rich*→	831	Brevibacillus sp. BC25	GCA_000282075.1	firmicutes
WP_007770561.1	GAP1-N2+GAP1-M+GAP1-C	TM+DUF4190+TM→ TM+DUF4190+TM→ polar_leader+TM+TM+TM→ TM+vWA(canonical)+Fibronectin-like_repeats+TM+beta-rich+lc_tail→ Tubulin+alpha_helical+??+??→ PSE→ PSE→ GAP1-N2+GAP1-M+GAP1-C*→ beta-rich+vWA(canonical)→??+DO-GTPase2.2→ TM+DUF4190+TM+PknH_C→ TM+DUF4190+TM+PknH_C→?→?→ TM+DUF4190+TM+PknH_C→	829	Mycobacterium colombiense	GCA_000222105.4	actinobacteria
WP_007778305.1	GAP1-N2+GAP1-M+GAP1-C+polar_tail+TM+beta-rich	TM+vWA(canonical)+YtKA-like+TM+??→ Tubulin+coiled_coil+??→ Tubulin+coiled_coil+HTH?+??→ TM+TM+TM+vWA(canonical)+TM+TM+TM+polar_tail→ Fibronectin-like_repeats+??→ ZnR+ZnR+DO-GTPase2.2+ZnR+ZnR→ GAP1-N2+GAP1-M+GAP1-C+polar_tail+TM+beta-rich*→	834	Brevibacillus sp. CF112	GCA_000282015.1	firmicutes
WP_007820797.1	GAP1-N2+GAP1-M+GAP1-C	GAP1-N2+GAP1-M+GAP1-C*→	772	Streptomyces sp. Tu6071	GCA_000213055.1	actinobacteria
WP_008098874.1	GAP1-N2+GAP1-M+TM+GAP1-C	FHA-like?+ZnR+ZnR+DO-GTPase2.2→ GAP1-N2+GAP1-M+TM+GAP1-C*→	709	Verrucomicrobiae bacterium DG1235	GCA_000155695.1	verrucomicrobia
WP_008195826.1	GAP1-N2+GAP1-M+GAP1-C	ZnR+ZnR+DO-GTPase2.2→ GAP1-N2+GAP1-M+GAP1-C*→ TM+TM+TM+TM+TM+ZnR+ZnR+DO-GTPase2.2+TM+beta-sandwich+NTF-like→ GrpE→	850	Microcystis sp. T1-4	GCA_000297435.1	cyanobacteria
WP_008379378.1	GAP1-N2+GAP1-M+GAP1-C	polar_leader+TM+TM+TM→ TM+vWA(canonical)+Fibronectin-like_repeats+TM+beta-rich+lc_tail→ Tubulin+alpha_helical+??+??→ GAP_unknown1+TM+TM+GAP_unknown2→ HRDC-like?+Fibronectin-like_repeats→ GAP1-N2+GAP1-M+GAP1-C*→ beta-rich+vWA(canonical)→??+DO-GTPase2.2→	866	Gordonia malaquae	GCA_000344135.1	actinobacteria
WP_008410972.1	GAP1-N2+GAP1-M+GAP1-C	GAP1-N2+GAP1-M+GAP1-C*→	789	Streptomyces	GCA_000299175.2	actinobacteria
WP_008714732.1	GAP1-N2+GAP1-M+GAP1-C	polar_leader+TM+TM+TM→ TM+vWA(canonical)+Fibronectin-like_repeats+TM+beta-rich+lc_tail→ Tubulin+alpha_helical+??+??→ GAP_unknown1+TM+TM+GAP_unknown2→ HRDC-like?+Fibronectin-like_repeats→ GAP1-N2+GAP1-M+GAP1-C*→ beta-rich+vWA(canonical)→??+DO-GTPase2.2→	904	Rhodococcus sp. AW25M09	GCA_000333955.1	actinobacteria
WP_008729950.1	GAP1-N2+GAP1-M+GAP1-C	polar_leader+TM+TM+TM→ TM+vWA(canonical)+Fibronectin-like_repeats+TM+beta-rich+lc_tail→ Tubulin+alpha_helical+??+??→ GAP_unknown1+TM+TM+GAP_unknown2→ HRDC-like?+Fibronectin-like_repeats→ GAP1-N2+GAP1-M+GAP1-C*→?→??+DO-GTPase2.2→	909	Actinomyces massiliensis	GCA_000296275.1	actinobacteria
WP_009474379.1	GAP1-N2+GAP1-M+GAP1-C	polar_leader+TM+TM+TM→ TM+vWA(canonical)+Fibronectin-like_repeats+TM+beta-rich+lc_tail→ Tubulin+alpha_helical+??+??→ GAP_unknown1+TM+TM+GAP_unknown2→ HRDC-like?+Fibronectin-like_repeats→ GAP1-N2+GAP1-M+GAP1-C*→ beta-rich+vWA(canonical)→??+DO-GTPase2.2→	913	Rhodococcus sp. JVH1	GCA_000280725.1	actinobacteria
WP_009526733.1	GAP1-N2+GAP1-M+GAP1-C	TM+TM+TM+TM+ZnR+ZnR+DO-GTPase2.2→ GrpE→ HSP70→ HSP70+vWA(canonical)→ DnaJ+DnaJ+ZnR+ZnR+coiled_coil+Fibronectin-like_repeats→ ZnR+ZnR+DO-GTPase2.2→ GAP1-N2+GAP1-M+GAP1-C*→?→?→?→?→ TM+TM+TM+TM+ZnR+ZnR+DO-GTPase2.2→	886	Peptoanaerobacter stomatis	GCA_000238095.2	firmicutes
WP_009530652.1	GAP1-N2+GAP1-M+GAP1-C	HSP70→ HSP70+vWA(canonical)→ DnaJ+DnaJ+ZnR+ZnR+coiled_coil+Fibronectin-like_repeats→ ZnR+ZnR+DO-GTPase2.2→ GAP1-N2+GAP1-M+GAP1-C*→?→?→ TM+TM+TM+TM+ZnR+ZnR+DO-GTPase2.2→	830	Peptoanaerobacter stomatis	GCA_000293035.1	firmicutes
WP_009589527.1	GAP1-N2+GAP1-M+GAP1-C+polar_tail+TM+beta-rich	TM+vWA(canonical)+YtKA-like+TM+??→ Tubulin+coiled_coil+??→ Tubulin+coiled_coil+HTH?+??→ TM+TM+TM+vWA(canonical)+TM+TM+TM+polar_tail→ Fibronectin-like_repeats+??→ ZnR+ZnR+DO-GTPase2.2+ZnR+ZnR→ GAP1-N2+GAP1-M+GAP1-C+polar_tail+TM+beta-rich*→	990	Paenibacillus sp. HGF5	GCA_000204455.2	firmicutes
WP_009649088.1	GAP1-N2+GAP1-M+GAP1-C	GrpE→ HSP70→ TPR_containing_alpha_helical+Fibronectin-like_repeats→ ZnR+ZnR+DO-GTPase2.2→ GAP1-N2+GAP1-M+GAP1-C*→ TM+TM+TM+TM+ZnR+ZnR+DO-GTPase2.2→??→?→?→?→ HSP70→ vWA(canonical)→	818	Actinomyces sp. ICM47	GCA_000278725.1	actinobacteria
WP_009672117.1	GAP1-N2+GAP1-M+GAP1-C+polar_tail+TM+Cadherin-like	TM+vWA(canonical)+YtKA-like+TM+??→ Tubulin+coiled_coil+??→ Tubulin+coiled_coil+HTH?+??→ TM+TM+TM+vWA(canonical)+TM+TM+TM+polar_tail→ Fibronectin-like_repeats+??→ ZnR+ZnR+DO-GTPase2.2+ZnR+ZnR→ GAP1-N2+GAP1-M+GAP1-C+polar_tail+TM+Cadherin-like*→	965	Paenibacillus	GCA_000214295.2	firmicutes
WP_009741053.1	GAP1-N2+GAP1-M+GAP1-C	GrpE→ HSP70→ TPR_containing_alpha_helical+Fibronectin-like_repeats→ ZnR+ZnR+DO-GTPase2.2→ GAP1-N2+GAP1-M+GAP1-C*→ TM+TM+TM+TM+ZnR+ZnR+DO-GTPase2.2→??→ HSP70→	792	Frankia sp. QA3	GCA_000262465.1	actinobacteria

acc	architecture	operon	len	species	gca	taxend
WP_009748877.1	GAP1-N2+GAP1-M+GAP1-C	polar_leader+TM+TM+TM→ TM+vWA(canonical)+Fibronectin-like_repeats+TM+beta-rich+lc_tail→ Tubulin+alpha_helical+??+??→ PSE→ HRDC-like?+Fibronectin-like_repeats→ GAP1-N2+GAP1-M+GAP1-C*→?→??+DO-GTPase2.2→	920	Actinomyces sp. oral taxon 448	GCA_000220835.1	actinobacteria
WP_009945310.1	GAP1-N2+GAP1-M+GAP1-C	GrpE→ HSP70→ TPR_containing_alpha_helical+Fibronectin-like_repeats→ ZnR+ZnR+DO-GTPase2.2→ GAP1-N2+GAP1-M+GAP1-C*→ TM+TM+TM+TM+ZnR+ZnR+DO-GTPase2.2→||←?←?←HSP70←unknown+CASPASE	802	Saccharopolyspora erythraea	GCA_000062885.1	actinobacteria
WP_009947927.1	GAP1-N2+GAP1-M+GAP1-C	GAP1-N2+GAP1-M+GAP1-C*→	787	Saccharopolyspora erythraea	GCA_000062885.1	actinobacteria
WP_010045066.1	GAP1-N2+GAP1-M+TM	GAP1-N2+GAP1-M+TM*→	274	Gemmata obscuriglobus	GCA_003149495.1	planctomycetes
WP_010047932.1	GAP1-N2+GAP1-M+TM+low_complexity+TM	DO-GTPase2.1→?→ GAP1-N2+GAP1-M+TM+low_complexity+TM*→	908	Gemmata obscuriglobus	GCA_003149495.1	planctomycetes
WP_010188424.1	GAP1-N2+GAP1-M+GAP1-C	polar_leader+TM+TM+TM→ TM+vWA(canonical)+Fibronectin-like_repeats+TM+beta-rich+lc_tail→ Tubulin+alpha_helical+??+??→ GAP_unknown1+TM+TM+GAP_unknown2→ HRDC-like?+Fibronectin-like_repeats→ GAP1-N2+GAP1-M+GAP1-C*→ beta-rich+vWA(canonical)→??+DO-GTPase2.2→ polar_leader+TM+TM+TM→	557	Corynebacterium aurimucosum	GCA_000022905.1	actinobacteria
WP_010272083.1	GAP1-N2+GAP1-M+GAP1-C	GAP1-N2+GAP1-M+GAP1-C*→	772	Streptomyces sp. SA3_actG	GCF_000179195.1	actinobacteria
WP_010346131.1	GAP1-N2+GAP1-M+GAP1-C+polar_tail+TM+beta-rich	TM+vWA(canonical)+YtKA-like+TM+??→ Tubulin+coiled_coil+??→ Tubulin+coiled_coil+HTH?+??→ TM+TM+TM+vWA(canonical)+TM+TM+TM+polar_tail→ Fibronectin-like_repeats+??→ ZnR+ZnR+DO-GTPase2.2+ZnR+ZnR→ GAP1-N2+GAP1-M+GAP1-C+polar_tail+TM+beta-rich*→	999	Paenibacillus peoriae	GCF_000236805.1	firmicutes
WP_010493411.1	GAP1-N2+GAP1-M+GAP1-C+polar_tail+TM+Cadherin-like	TM+vWA(canonical)+YtKA-like+TM+??→ Tubulin+coiled_coil+??→ Tubulin+coiled_coil+HTH?+??→?→ TM+TM+TM+vWA(canonical)+TM+TM+TM+polar_tail→ Fibronectin-like_repeats+??→ ZnR+ZnR+DO-GTPase2.2+ZnR+ZnR→ GAP1-N2+GAP1-M+GAP1-C+polar_tail+TM+Cadherin-like*→	936	Paenibacillus elgii	GCF_000213315.1	firmicutes
WP_010585440.1	GAP1-N2+GAP1-M+TM+GAP1-C	ZnR+ZnR+DO-GTPase2.2→ GAP1-N2+GAP1-M+TM+GAP1-C*→??+TM+??+beta-rich??→ GYF+GYF+ZnR+TM+TM+TM→	875	Schlesneria paludicola	GCF_000255655.1	planctomycetes
WP_010641232.1	GAP1-N2+GAP1-M	-	789	Streptomyces sp. S4	-	actinobacteria
WP_010696001.1	GAP1-N2+GAP1-M+GAP1-C	GrpE→ HSP70→ TPR_containing_alpha_helical+Fibronectin-like_repeats→ ZnR+ZnR+DO-GTPase2.2→ GAP1-N2+GAP1-M+GAP1-C*→ TM+TM+TM+TM+ZnR+ZnR+DO-GTPase2.2→ HSP70→ vWA(canonical)→	800	Saccharopolyspora spinosa	GCA_002846475.1	actinobacteria
WP_011015330.1	GAP1-N2+GAP1-M+GAP1-C	polar_leader+TM+TM+TM→ TM+vWA(canonical)+Fibronectin-like_repeats+TM+beta-rich+lc_tail→ Tubulin+alpha_helical+??+??→ GAP_unknown1+TM+TM+GAP_unknown2→ HRDC-like?+Fibronectin-like_repeats→ GAP1-N2+GAP1-M+GAP1-C*→ beta-rich+vWA(canonical)→??+DO-GTPase2.2→	631	Corynebacterium glutamicum	GCF_000011325.1	actinobacteria
WP_011557476.1	GAP1-N2+GAP1-M+GAP1-C	HRDC-like?+Fibronectin-like_repeats→ GAP1-N2+GAP1-M+GAP1-C*→??+DO-GTPase2.2→	829	Mycobacterium	GCA_000014165.1	actinobacteria
WP_011594626.1	GAP1-N2+GAP1-M+GAP1-C	polar_leader+TM+TM+TM→ TM+vWA(canonical)+Fibronectin-like_repeats+TM+beta-rich+lc_tail→ Tubulin+alpha_helical+??+??→ GAP_unknown1+TM+TM+GAP_unknown2→ HRDC-like?+Fibronectin-like_repeats→ GAP1-N2+GAP1-M+GAP1-C*→ beta-rich+vWA(canonical)→??+DO-GTPase2.2→	913	Rhodococcus jostii	GCA_000014565.1	actinobacteria
WP_011726629.1	GAP1-N2+GAP1-M+GAP1-C	HRDC-like?+Fibronectin-like_repeats→ GAP1-N2+GAP1-M+GAP1-C*→??+DO-GTPase2.2→	822	Mycolicibacterium smegmatis	-	actinobacteria
WP_011775814.1	GAP1-N2+GAP1-M+TM+GAP1-C+TM+TM	PP2C→?→ ZnR+ZnR+DO-GTPase2.2→ GAP1-N2+GAP1-M+TM+GAP1-C+TM+TM*→	763	Paenarthrobacter aurescens	GCA_000014925.1	actinobacteria
WP_011854161.1	GAP1-N2+GAP1-M+GAP1-C	HRDC-like?+Fibronectin-like_repeats→ GAP1-N2+GAP1-M+GAP1-C*→??+DO-GTPase2.2→	829	Mycobacteriaceae	GCA_000016005.1	actinobacteria
WP_012102064.1	GAP1-N2+GAP1-M+GAP1-C	Fibronectin-like_repeats+??→ ZnR+ZnR+DO-GTPase2.2→ GAP1-N2+GAP1-M+GAP1-C*→ TM+TM+TM+TM+ZnR+ZnR+DO-GTPase2.2→ TM+vWA(canonical)+YtKA-like+TM+??→ Tubulin+coiled_coil+??→ Tubulin+coiled_coil+HTH?+??→ Fibronectin-like_repeats+??→ ZnR+ZnR+DO-GTPase2.2+ZnR+ZnR→ GAP1-N2+GAP1-M→	886	Clostridium kluyveri	GCA_000010265.1	firmicutes
WP_012620487.1	GAP1-N2+GAP1-M	GAP1-N2+GAP1-M+GAP1-C→ TM+TM+TM+TM+ZnR+ZnR+DO-GTPase2.2→ TM+vWA(canonical)+YtKA-like+TM+??→ Tubulin+coiled_coil+??→ Tubulin+coiled_coil+HTH?+??→ Fibronectin-like_repeats+??→ ZnR+ZnR+DO-GTPase2.2+ZnR+ZnR→ GAP1-N2+GAP1-M*→	359	Clostridium kluyveri	GCA_000010265.1	firmicutes
WP_012851763.1	GAP1-N2+GAP1-M+GAP1-C	GrpE→ HSP70→ TPR_containing_alpha_helical+Fibronectin-like_repeats→ ZnR+ZnR+DO-GTPase2.2→ GAP1-N2+GAP1-M+GAP1-C*→ TM+TM+TM+TM+ZnR+ZnR+DO-GTPase2.2→	792	Thermomonospora curvata	GCA_000024385.1	actinobacteria
WP_012852227.1	GAP1-N2+GAP1-M+GAP1-C	GrpE→ HSP70→ TPR_containing_alpha_helical+Fibronectin-like_repeats→ ZnR+ZnR+DO-GTPase2.2→ GAP1-N2+GAP1-M+GAP1-C*→ TM+TM+TM+TM+ZnR+ZnR+DO-GTPase2.2+TM→	784	Thermomonospora curvata	GCA_000024385.1	actinobacteria
WP_012913508.1	GAP1-N2+GAP1-M	FGS-fold_domain→ ZnR+DO-GTPase2.1→ GAP1-N2+GAP1-M*→	246	Pirellula staleyi	GCA_000025185.1	planctomycetes
WP_013237251.1	GAP1-N2+GAP1-M+GAP1-C	Fibronectin-like_repeats+??→ ZnR+ZnR+DO-GTPase2.2→ GAP1-N2+GAP1-M+GAP1-C*→ TM+vWA(canonical)+YtKA-like+TM+??→ Tubulin+coiled_coil+??→ Tubulin+coiled_coil+HTH?+??→ TM+TM+TM+vWA(canonical)+TM+TM+TM+polar_tail→ Fibronectin-like_repeats+??→	868	Clostridium ljungdahlii	GCA_000143685.1	firmicutes
WP_013237261.1	GAP1-N2+GAP1-M	Tubulin+coiled_coil+HTH?+??→ TM+TM+TM+vWA(canonical)+TM+TM+TM+polar_tail→ Fibronectin-like_repeats+??→?→?→?→ ZnR+ZnR+DO-GTPase2.2+ZnR+ZnR→ GAP1-N2+GAP1-M*→	351	Clostridium ljungdahlii	GCA_000143685.1	firmicutes
WP_013310112.1	GAP1-N2+GAP1-M+GAP1-C+polar_tail+TM+beta-rich	TM+vWA(canonical)+YtKA-like+TM+??→ Tubulin+coiled_coil+??→ Tubulin+coiled_coil+HTH?+??→ TM+TM+TM+vWA(canonical)+TM+TM+TM+polar_tail→ Fibronectin-like_repeats+??→ ZnR+ZnR+DO-GTPase2.2+ZnR+ZnR→ GAP1-N2+GAP1-M+GAP1-C+polar_tail+TM+beta-rich*→	1002	Paenibacillus polymyxa	GCA_000146875.2	firmicutes
WP_013370937.1	GAP1-N2+GAP1-M+GAP1-C+polar_tail+TM+beta-rich	TM+vWA(canonical)+YtKA-like+TM+??→ Tubulin+coiled_coil+??→ Tubulin+coiled_coil+HTH?+??→ TM+TM+TM+vWA(canonical)+TM+TM+TM+polar_tail→ Fibronectin-like_repeats+??→ ZnR+ZnR+DO-GTPase2.2+ZnR+ZnR→ GAP1-N2+GAP1-M+GAP1-C+polar_tail+TM+beta-rich*→	1009	Paenibacillus polymyxa	GCA_000164985.2	firmicutes
WP_013564717.1	GAP1-N2+GAP1-M	FGS-fold_domain→ ZnR+DO-GTPase2.1→ GAP1-N2+GAP1-M*→	231	Isosphaera pallida	GCA_000186345.1	planctomycetes
WP_013655639.1	GAP1-N2+GAP1-M+GAP1-C+TM+polar_tail+beta-rich	TM+vWA(canonical)+YtKA-like+TM+??→ Tubulin+coiled_coil+??→ Tubulin+coiled_coil+HTH?+??→ TM+TM+TM+vWA(canonical)+TM+TM+TM+polar_tail→ Fibronectin-like_repeats+??→ ZnR+ZnR+DO-GTPase2.2+ZnR+ZnR→ GAP1-N2+GAP1-M+GAP1-C+TM+polar_tail+beta-rich*→	870	Cellulosilyticum lentocellum	GCA_000178835.2	firmicutes
WP_013914661.1	GAP1-N2+GAP1-M+GAP1-C+polar_tail+TM+Cadherin-like	TM+vWA(canonical)+YtKA-like+TM+??→ Tubulin+coiled_coil+??→ Tubulin+coiled_coil+HTH?+??→ TM+TM+TM+vWA(canonical)+TM+TM+TM+polar_tail→ Fibronectin-like_repeats+??→ ZnR+ZnR+DO-GTPase2.2+ZnR+ZnR→ GAP1-N2+GAP1-M+GAP1-C+polar_tail+TM+Cadherin-like*→	921	Paenibacillus mucilaginosus	GCA_000218915.1	firmicutes
WP_014011376.1	GAP1-N2+GAP1-M+GAP1-C	polar_leader+TM+TM+TM→ TM+vWA(canonical)+Fibronectin-like_repeats+TM+beta-rich+lc_tail→ Tubulin+alpha_helical+??+??→ GAP_unknown1+TM+TM+GAP_unknown2→ HRDC-like?+Fibronectin-like_repeats→ GAP1-N2+GAP1-M+GAP1-C*→ beta-rich+vWA(canonical)→??+DO-GTPase2.2→	579	Corynebacterium variabile	GCA_000179395.2	actinobacteria
WP_014049056.1	GAP1-N2+GAP1-M+GAP1-C	GAP1-N2+GAP1-M+GAP1-C*→	803	Streptomyces	GCA_000177195.2	actinobacteria
WP_014153312.1	GAP1-N2+GAP1-M+GAP1-C	GAP1-N2+GAP1-M+GAP1-C*→	804	Streptomyces pratensis	GCA_000176115.2	actinobacteria
WP_014173128.1	GAP1-N2+GAP1-M+GAP1-C	ZnR+ZnR+DO-GTPase2.2→ GAP1-N2+GAP1-M+GAP1-C*→ TM+TM+TM+TM+ZnR+ZnR+DO-GTPase2.2→	815	Streptomyces	GCA_000092385.1	actinobacteria
WP_014281329.1	GAP1-N2+GAP1-M+GAP1-C+polar_tail+TM+beta-rich	TM+vWA(canonical)+YtKA-like+TM+??→ Tubulin+coiled_coil+??→ Tubulin+coiled_coil+HTH?+??→ TM+TM+TM+vWA(canonical)+TM+TM+TM+polar_tail→ Fibronectin-like_repeats+??→ ZnR+ZnR+DO-GTPase2.2+ZnR+ZnR→ GAP1-N2+GAP1-M+GAP1-C+polar_tail+TM+beta-rich*→	1005	Paenibacillus terrae	GCA_000235585.1	firmicutes
WP_014368531.1	GAP1-N2+GAP1-M+GAP1-C+polar_tail+TM+Cadherin-like	TM+vWA(canonical)+YtKA-like+TM+??→ PSE→ PSE→ TM+TM+TM+vWA(canonical)+TM+TM+TM+polar_tail→ Fibronectin-like_repeats+??→ ZnR+ZnR+DO-GTPase2.2+ZnR+ZnR→ GAP1-N2+GAP1-M+GAP1-C+polar_tail+TM+Cadherin-like*→	921	Paenibacillus mucilaginosus	GCA_000250655.1	firmicutes
WP_014695139.1	GAP1-N2+GAP1-M+GAP1-C	GrpE→ HSP70→ TPR_containing_alpha_helical+Fibronectin-like_repeats→ ZnR+ZnR+DO-GTPase2.2→ GAP1-N2+GAP1-M+GAP1-C*→ TM+TM+TM+TM+ZnR+ZnR+DO-GTPase2.2→	740	Actinoplanes	GCA_000237145.1	actinobacteria
WP_014910933.1	GAP1-N2+GAP1-M+GAP1-C	TPR_containing_alpha_helical+Fibronectin-like_repeats→ ZnR+ZnR+DO-GTPase2.2→ GAP1-N2+GAP1-M+GAP1-C*→ TM+TM+TM+TM+ZnR+ZnR+DO-GTPase2.2→	868	Nocardiopsis alba	GCA_000294515.1	actinobacteria
WP_014965367.1	GAP1-N2+GAP1-M+TM+low_complexity	GAP1-N2+GAP1-M+TM+low_complexity*→	602	Nitrosopumilus	GCA_000299395.1	thaumarchaeota
WP_015245331.1	GAP1-N2+GAP1-M+GAP1-C	GAP1-N2+GAP1-M+GAP1-C*→	1116	Singulisphaera acidiphila	GCA_000242455.3	planctomycetes
WP_015246829.1	GAP1-N2+GAP1-M	FGS-fold_domain→ ZnR+DO-GTPase2.1→ GAP1-N2+GAP1-M*→	220	Singulisphaera acidiphila	GCA_000242455.3	planctomycetes
WP_015250492.1	GAP1-N2+GAP1-M+GAP1-C	GAP1-N2+GAP1-M+GAP1-C*→	469	Singulisphaera acidiphila	GCA_000242455.3	planctomycetes
WP_015506890.1	GAP1-N2+GAP1-M+GAP1-C	GAP1-N2+GAP1-M+GAP1-C*→	789	Streptomyces	GCA_000359525.1	actinobacteria
WP_015579268.1	GAP1-N2+GAP1-M+GAP1-C	GAP1-N2+GAP1-M+GAP1-C*→	804	Streptomyces sp. PAMC 26508	GCA_000364805.1	actinobacteria
WP_015612204.1	GAP1-N2+GAP1-M+GAP1-C	GAP1-N2+GAP1-M+GAP1-C*→	812	Streptomyces fulvissimus	GCA_000385945.1	actinobacteria
WP_015652149.1	GAP1-N2+GAP1-M+GAP1-C	polar_leader+TM+TM+TM→ TM+vWA(canonical)+Fibronectin-like_repeats+TM+beta-rich+lc_tail→ Tubulin+alpha_helical+??+??→ GAP_unknown1+TM+TM+GAP_unknown2→ HRDC-like?+Fibronectin-like_repeats→ GAP1-N2+GAP1-M+GAP1-C*→	654	Corynebacterium callunae	GCA_000344785.1	actinobacteria
WP_015657008.1	GAP1-N2+GAP1-M+GAP1-C	TPR_containing_alpha_helical+Fibronectin-like_repeats→ ZnR+ZnR+DO-GTPase2.2→ GAP1-N2+GAP1-M+GAP1-C*→ TM+TM+TM+TM+ZnR+ZnR+DO-GTPase2.2+TM→ vWA(canonical)→ PP2C→ Pkinase+ZnR+beta-rich→	832	Streptomyces davaonensis	GCA_000349325.1	actinobacteria
WP_015710909.1	GAP1-N2+GAP1-M+GAP1-C	TM+TM+TM+TM+ZnR+ZnR+DO-GTPase2.2→?→ HSP70→ HSP70+vWA(canonical)→?→ ZnR+ZnR+DO-GTPase2.2→ GAP1-N2+GAP1-M+GAP1-C*→	889	Treponema azotonutricium	GCA_000214355.1	spirochaetes
WP_015737595.1	GAP1-N2+GAP1-M+GAP1-C+polar_tail+TM+beta-rich	TM+vWA(canonical)+YtKA-like+TM+??→?→ Tubulin+coiled_coil+??→ Tubulin+coiled_coil+HTH?+??→ TM+TM+TM+vWA(canonical)+TM+TM+TM+polar_tail→ Fibronectin-like_repeats+??→ ZnR+ZnR+DO-GTPase2.2+ZnR+ZnR→ GAP1-N2+GAP1-M+GAP1-C+polar_tail+TM+beta-rich*→	992	Paenibacillus sp. Y412MC10	GCA_000024685.1	firmicutes
WP_015893872.1	GAP1-N2+GAP1-M+GAP1-C+polar_tail+TM+beta-rich	TM+vWA(canonical)+YtKA-like+TM+??→ Tubulin+coiled_coil+??→ Tubulin+coiled_coil+HTH?+??→ TM+TM+TM+vWA(canonical)+TM+TM+TM+polar_tail→ Fibronectin-like_repeats+??→ ZnR+ZnR+DO-GTPase2.2+ZnR+ZnR→ GAP1-N2+GAP1-M+GAP1-C+polar_tail+TM+beta-rich*→	832	Brevibacillus brevis	GCA_000010165.1	firmicutes
WP_016202160.1	GAP1-N2+GAP1-M+GAP1-C+polar_tail+TM+beta-rich	GAP1-N2+GAP1-M+GAP1-C+polar_tail+TM+beta-rich*→	750	Bacillus nealsonii	GCA_000401235.1	firmicutes
WP_016362300.1	GAP1-N2+GAP1-M+GAP1-C+polar_tail+TM+Cadherin-like	TM+vWA(canonical)+YtKA-like+TM+??→ Tubulin+coiled_coil+??→ Tubulin+coiled_coil+HTH?+??→ TM+TM+TM+vWA(canonical)+TM+TM+TM+polar_tail→ Fibronectin-like_repeats+??→ ZnR+ZnR+DO-GTPase2.2+ZnR+ZnR→ GAP1-N2+GAP1-M+GAP1-C+polar_tail+TM+Cadherin-like*→	921	Paenibacillus mucilaginosus	GCA_000258535.2	firmicutes

acc	architecture	operon	len	species	gca	taxend
WP_016431979.1	GAP1-N2+GAP1-M+GAP1-C	GAP1-N2+GAP1-M+GAP1-C*→ vWA(canonical)→ PP2C→ Pkinase+ZnR+beta-rich→||←TPR_containing_alpha_helical+Fibronectin-like_repeats	785	Streptomyces sp. HGB0020	GCA_000411315.1	actinobacteria
WP_016739560.1	GAP1-N2+GAP1-M+GAP1-C+polar_tail+TM+beta-rich	TM+vWA(canonical)+YtKA-like+TM+??→ Tubulin+coiled_coil+??→ Tubulin+coiled_coil+HTH?+??→ TM+TM+TM+vWA(canonical)+TM+TM+TM+polar_tail→ Fibronectin-like_repeats+??→ ZnR+ZnR+DO-GTPase2.2+ZnR+ZnR→ GAP1-N2+GAP1-M+GAP1-C+polar_tail+TM+beta-rich*→	832	Bacillales	GCA_001184755.1	firmicutes
WP_016821154.1	GAP1-N2+GAP1-M+GAP1-C+polar_tail+TM+beta-rich	Tubulin+coiled_coil+??→ Tubulin+coiled_coil+HTH?+??→ TM+TM+TM+vWA(canonical)+TM+TM+TM+polar_tail→ Fibronectin-like_repeats+??→ ZnR+ZnR+DO-GTPase2.2+ZnR+ZnR→ GAP1-N2+GAP1-M+GAP1-C+polar_tail+TM+beta-rich*→	1009	Paenibacillus polymyxa	GCA_000597985.1	firmicutes
WP_016882979.1	GAP1-N2+GAP1-M+GAP1-C	polar_leader+TM+TM+TM→ TM+vWA(canonical)+Fibronectin-like_repeats+TM+beta-rich+lc_tail→ Tubulin+alpha_helical+??+??→ GAP_unknown1+TM+TM+GAP_unknown2→ HRDC-like?+Fibronectin-like_repeats→ GAP1-N2+GAP1-M+GAP1-C*→ beta-rich+vWA(canonical)→??+DO-GTPase2.2→	913	Rhodococcus sp. DK17	GCF_000263875.1	actinobacteria
WP_017194728.1	GAP1-N2+GAP1-M+GAP1-C	polar_leader+TM+TM+TM→ TM+vWA(canonical)+Fibronectin-like_repeats+TM+beta-rich+lc_tail→ Tubulin+alpha_helical+??+??→ GAP_unknown1+TM+TM+GAP_unknown2→ HRDC-like?+Fibronectin-like_repeats→ GAP1-N2+GAP1-M+GAP1-C*→?→??+DO-GTPase2.2→	909	Actinomyces massiliensis	GCF_000269805.1	actinobacteria
WP_017246635.1	GAP1-N2+GAP1-M+GAP1-C+polar_tail+TM+beta-rich	TM+vWA(canonical)+YtKA-like+TM+??→ Tubulin+coiled_coil+??→ Tubulin+coiled_coil+HTH?+??→ TM+TM+TM+vWA(canonical)+TM+TM+TM+polar_tail→ Fibronectin-like_repeats+??→ ZnR+ZnR+DO-GTPase2.2+ZnR+ZnR→ GAP1-N2+GAP1-M+GAP1-C+polar_tail+TM+beta-rich*→	832	Brevibacillus brevis	GCA_000296715.2	firmicutes
WP_017428408.1	GAP1-N2+GAP1-M+GAP1-C+polar_tail+TM+beta-rich	TM+vWA(canonical)+YtKA-like+TM+??→ Tubulin+coiled_coil+??→ Tubulin+coiled_coil+HTH?+??→ TM+TM+TM+vWA(canonical)+TM+TM+TM+polar_tail→ Fibronectin-like_repeats+??→ ZnR+ZnR+DO-GTPase2.2+ZnR+ZnR→ GAP1-N2+GAP1-M+GAP1-C+polar_tail+TM+beta-rich*→	1009	Paenibacillus polymyxa	GCA_000307675.1	firmicutes
WP_017535628.1	GAP1-N2+GAP1-M+GAP1-C	TPR_containing_alpha_helical+Fibronectin-like_repeats→ ZnR+ZnR+DO-GTPase2.2→ GAP1-N2+GAP1-M+GAP1-C*→ TM+TM+TM+TM+ZnR+ZnR+DO-GTPase2.2→	868	Nocardiopsis alba	GCF_000341225.1	actinobacteria
WP_017542441.1	GAP1-N2+GAP1-M+GAP1-C	GrpE→ HSP70→ TPR_containing_alpha_helical+Fibronectin-like_repeats→ ZnR+ZnR+DO-GTPase2.2→ GAP1-N2+GAP1-M+GAP1-C*→ TM+TM+TM+TM+ZnR+ZnR+DO-GTPase2.2→??→	813	Nocardiopsis prasina	GCF_000341265.1	actinobacteria
WP_017565406.1	GAP1-N2+GAP1-M+GAP1-C	TPR_containing_alpha_helical+Fibronectin-like_repeats+extended_insert+Fibronectin-like_repeats→ ZnR+ZnR+DO-GTPase2.2→ GAP1-N2+GAP1-M+GAP1-C*→ TM+TM+ZnR+ZnR+DO-GTPase2.2→	943	Nocardiopsis synnemataformans	GCF_000340945.1	actinobacteria
WP_017569953.1	GAP1-N2+GAP1-M+GAP1-C	GAP1-N2+GAP1-M+GAP1-C*→	781	Nocardiopsis halotolerans	GCF_000341065.1	actinobacteria
WP_017576279.1	GAP1-N2+GAP1-M+GAP1-C	HSP70→ TPR_containing_alpha_helical+Fibronectin-like_repeats→ ZnR+ZnR+DO-GTPase2.2→ GAP1-N2+GAP1-M+GAP1-C*→ TM+TM+TM+TM+ZnR+ZnR+DO-GTPase2.2→	857	Nocardiopsis kunsanensis	GCF_000340965.1	actinobacteria
WP_017583227.1	GAP1-N2+GAP1-M+GAP1-C	GrpE→ HSP70→ TPR_containing_alpha_helical+Fibronectin-like_repeats→ ZnR+ZnR+DO-GTPase2.2→ GAP1-N2+GAP1-M+GAP1-C*→ TM+TM+TM+TM+ZnR+ZnR+DO-GTPase2.2→??→ HSP70→ vWA(canonical)→	790	Nocardiopsis valliformis	GCF_000340985.1	actinobacteria
WP_017589329.1	GAP1-N2+GAP1-M+GAP1-C	TPR_containing_alpha_helical+Fibronectin-like_repeats→ ZnR+ZnR+DO-GTPase2.2→ GAP1-N2+GAP1-M+GAP1-C*→ TM+TM+TM+TM+ZnR+ZnR+DO-GTPase2.2→	861	Nocardiopsis ganjiahuensis	GCF_000341085.1	actinobacteria
WP_017606786.1	GAP1-N2+GAP1-M+GAP1-C	TPR_containing_alpha_helical+Fibronectin-like_repeats→ ZnR+ZnR+DO-GTPase2.2→ GAP1-N2+GAP1-M+GAP1-C*→	830	Nocardiopsis alkaliphila	GCF_000341005.1	actinobacteria
WP_017609551.1	GAP1-N2+GAP1-M+GAP1-C	HSP70→ PSE→ ZnR+ZnR+DO-GTPase2.2→ GAP1-N2+GAP1-M+GAP1-C*→ TM+TM+TM+TM+ZnR+ZnR+DO-GTPase2.2→	879	Nocardiopsis xinjiangensis	GCF_000341145.1	actinobacteria
WP_017814015.1	GAP1-N2+GAP1-M+GAP1-C+polar_tail+TM+beta-rich	TM+vWA(canonical)+YtKA-like+TM+??→ Tubulin+coiled_coil+??→ Tubulin+coiled_coil+HTH?+??→ TM+TM+TM+vWA(canonical)+TM+TM+TM+polar_tail→ Fibronectin-like_repeats+??→ ZnR+ZnR+DO-GTPase2.2+ZnR+ZnR→ GAP1-N2+GAP1-M+GAP1-C+polar_tail+TM+beta-rich*→	1138	Paenibacillus sp. A9	GCF_000346635.1	firmicutes
WP_018019435.1	GAP1-N2+GAP1-M+GAP1-C	polar_leader+TM+TM+TM→ TM+vWA(canonical)+Fibronectin-like_repeats+TM+beta-rich+lc_tail→ Tubulin+alpha_helical+??+??→ GAP_unknown1+TM+TM+GAP_unknown2→ HRDC-like?+Fibronectin-like_repeats→ GAP1-N2+GAP1-M+GAP1-C*→ beta-rich+vWA(canonical)→??+DO-GTPase2.2→ polar_leader+TM+TM+TM→	583	Corynebacterium ciconiae	GCF_000372385.1	actinobacteria
WP_018023270.1	GAP1-N2+GAP1-M+GAP1-C	polar_leader+TM+TM+TM→ TM+vWA(canonical)+Fibronectin-like_repeats+TM+beta-rich+lc_tail→ Tubulin+alpha_helical+??+??→ GAP_unknown1+TM+TM+GAP_unknown2→ HRDC-like?+Fibronectin-like_repeats→ GAP1-N2+GAP1-M+GAP1-C*→ beta-rich+vWA(canonical)→??+DO-GTPase2.2→?→ TM+TM+TM→	526	Corynebacterium ulceribovis	GCF_000372445.1	actinobacteria
WP_018100677.1	GAP1-N2+GAP1-M+GAP1-C	GAP1-N2+GAP1-M+GAP1-C*→	815	Streptomyces	GCF_000375725.1	actinobacteria
WP_018384902.1	GAP1-N2+GAP1-M+GAP1-C	GAP1-N2+GAP1-M+GAP1-C*→ vWA(canonical)→ PSE→ Pkinase+ZnR+beta-rich→||←TPR_containing_alpha_helical+Fibronectin-like_repeats	792	Streptomyces vitaminophilus	GCA_001445835.1	actinobacteria
WP_018469179.1	GAP1-N2+GAP1-M+GAP1-C	GAP1-N2+GAP1-M+GAP1-C*→	789	Streptomyces sp. LaPpAH-202	GCA_003688775.1	actinobacteria
WP_018491644.1	GAP1-N2+GAP1-M	-	877	Streptomyces sp. CcalMP-8W	-	actinobacteria
WP_018514559.1	GAP1-N2+GAP1-M	-	859	Streptomyces sp. ScaeMP-e10	-	actinobacteria
WP_018554808.1	GAP1-N2+GAP1-M+GAP1-C	GAP1-N2+GAP1-M+GAP1-C*→	820	Streptomyces sp. ATexAB-D23	GCF_000373645.1	actinobacteria
WP_018657913.1	GAP1-N2+GAP1-M+GAP1-C	GrpE→ HSP70→ TPR_containing_alpha_helical+Fibronectin-like_repeats→ ZnR+ZnR+DO-GTPase2.2→ GAP1-N2+GAP1-M+GAP1-C*→ TM+TM+TM+TM+ZnR+ZnR+DO-GTPase2.2+TM→	818	Actinomadura flavalba	GCF_000374305.1	actinobacteria
WP_018671154.1	GAP1-N2+GAP1-M+GAP1-C+polar_tail+TM+beta-rich	TM+vWA(canonical)+YtKA-like+TM+??→ Tubulin+coiled_coil+??→ Tubulin+coiled_coil+HTH?+??→ TM+TM+TM+vWA(canonical)+TM+TM+TM+polar_tail→ Fibronectin-like_repeats+??→ ZnR+ZnR+DO-GTPase2.2+ZnR+ZnR→ GAP1-N2+GAP1-M+GAP1-C+polar_tail+TM+beta-rich*→	833	Brevibacillus laterosporus	GCA_002706795.1	firmicutes
WP_018680664.1	GAP1-N2+GAP1-M+GAP1-C	GrpE→ HSP70→ TPR_containing_alpha_helical+Fibronectin-like_repeats→ ZnR+ZnR+DO-GTPase2.2→ GAP1-N2+GAP1-M+GAP1-C*→ TM+TM+TM+TM+ZnR+ZnR+DO-GTPase2.2→ HSP70→ vWA(canonical)→	847	Actinokineospora enzanensis	GCF_000374445.1	actinobacteria
WP_018758407.1	GAP1-N2+GAP1-M+GAP1-C+polar_tail+TM+beta-rich	TM+vWA(canonical)+YtKA-like+TM+??→ Tubulin+coiled_coil+??→ Tubulin+coiled_coil+HTH?+??→ TM+TM+TM+vWA(canonical)+TM+TM+TM+polar_tail→ Fibronectin-like_repeats+??→ ZnR+ZnR+DO-GTPase2.2+ZnR+ZnR→ GAP1-N2+GAP1-M+GAP1-C+polar_tail+TM+beta-rich*→	956	Paenibacillus terrigena	GCF_000374845.1	firmicutes
WP_018883185.1	GAP1-N2+GAP1-M+GAP1-C+polar_tail+TM+beta-rich	TM+vWA(canonical)+YtKA-like+TM+??→ Tubulin+coiled_coil+??→ Tubulin+coiled_coil+HTH?+??→ TM+TM+TM+vWA(canonical)+TM+TM+TM+polar_tail→ Fibronectin-like_repeats+??→ ZnR+ZnR+DO-GTPase2.2+ZnR+ZnR→ GAP1-N2+GAP1-M+GAP1-C+polar_tail+TM+beta-rich*→	1000	Paenibacillus massiliensis	GCF_000377505.1	firmicutes
WP_018895729.1	GAP1-N2+GAP1-M+GAP1-C	GAP1-N2+GAP1-M+GAP1-C*→	789	Streptomyces sp. CNY228	GCF_000377545.1	actinobacteria
WP_018978026.1	GAP1-N2+GAP1-M+GAP1-C+polar_tail+TM+beta-rich	TM+vWA(canonical)+YtKA-like+TM+??→ Tubulin+coiled_coil+??→ Tubulin+coiled_coil+HTH?+??→ TM+TM+TM+vWA(canonical)+TM+TM+TM+polar_tail→ Fibronectin-like_repeats+??→ ZnR+ZnR+DO-GTPase2.2+ZnR+ZnR→ GAP1-N2+GAP1-M+GAP1-C+polar_tail+TM+beta-rich*→	1089	Saccharibacillus kuerlensis	GCF_000378145.1	firmicutes
WP_019061048.1	GAP1-N2+GAP1-M+GAP1-C	GrpE→ HSP70→ TPR_containing_alpha_helical+Fibronectin-like_repeats→ ZnR+ZnR+DO-GTPase2.2→ GAP1-N2+GAP1-M+GAP1-C*→ TM+TM+TM+TM+ZnR+ZnR+DO-GTPase2.2+Jelly_roll+beta-sandwich→||←STAND_NTPase+TM+TM+TM+TM+TM+TM+TM	512	Streptomyces prunicolor	GCF_000367365.1	actinobacteria
WP_019062737.1	GAP1-N2+GAP1-M	-	1082	Streptomyces prunicolor	-	actinobacteria
WP_019071485.1	GAP1-N2+GAP1-M+GAP1-C	TPR_containing_alpha_helical+Fibronectin-like_repeats→ ZnR+ZnR+DO-GTPase2.2→ GAP1-N2+GAP1-M+GAP1-C*→ TM+TM+TM+TM+ZnR+ZnR+DO-GTPase2.2+TM→ vWA(canonical)→ PP2C→ Pkinase+ZnR+beta-rich→	920	Streptomyces hokutonensis	GCF_000376565.1	actinobacteria
WP_019359896.1	GAP1-N2+GAP1-M	-	861	Streptomyces sp. AA1529	-	actinobacteria
WP_019422337.1	GAP1-N2+GAP1-M+GAP1-C+polar_tail	TM+TM+TM+vWA+TM+TM+TM+polar_tail→ Fibronectin-like_repeats+??→ ZnR+ZnR+DO-GTPase2.2→ GAP1-N2+GAP1-M+GAP1-C+polar_tail*→	1006	Paenibacillus sp. OSY-SE	GCF_000283315.1	firmicutes
WP_019513906.1	GAP1-N2+GAP1-M+GAP1-C	TM+DUF4190+TM→ TM+DUF4190+TM→ polar_leader+TM+TM+TM→ TM+vWA(canonical)+Fibronectin-like_repeats+TM+beta-rich+lc_tail→ Tubulin+alpha_helical+??+??→ GAP_unknown1+TM+TM+GAP_unknown2→ HRDC-like?+Fibronectin-like_repeats→ GAP1-N2+GAP1-M+GAP1-C*→ beta-rich+vWA(canonical)→??+DO-GTPase2.2→ PSE→ TM+DUF4190+TM+PknH_C→ TM+DUF4190+TM+PknH_C→	840	Mycobacteriaceae	GCA_000317305.3	actinobacteria
WP_019606768.1	GAP1-N2+GAP1-M+GAP1-C	TPR_containing_alpha_helical+Fibronectin-like_repeats+extended_insert+Fibronectin-like_repeats→ ZnR+ZnR+DO-GTPase2.2→ GAP1-N2+GAP1-M+GAP1-C*→ TM+TM+TM+TM+ZnR+ZnR+DO-GTPase2.2→	903	Nocardiopsis sp. CNS-639	GCF_000381685.1	actinobacteria
WP_019634286.1	GAP1-N2+GAP1-M+GAP1-C	GrpE→ HSP70→ TPR_containing_alpha_helical+Fibronectin-like_repeats→ ZnR+ZnR+DO-GTPase2.2→ GAP1-N2+GAP1-M+GAP1-C*→ TM+TM+TM+TM+ZnR+ZnR+DO-GTPase2.2+TM→	775	Actinomadura atramentaria	GCF_000381885.1	actinobacteria
WP_019635347.1	GAP1-N2+GAP1-M+GAP1-C+polar_tail+TM+beta-rich	TM+vWA(canonical)+YtKA-like+TM+??→ Tubulin+coiled_coil+??→ Tubulin+coiled_coil+HTH?+??→ TM+TM+TM+vWA(canonical)+TM+TM+TM+polar_tail→ Fibronectin-like_repeats+??→ ZnR+ZnR+DO-GTPase2.2+ZnR+ZnR→ GAP1-N2+GAP1-M+GAP1-C+polar_tail+TM+beta-rich*→	1152	Paenibacillus fonticola	GCF_000381905.1	firmicutes
WP_019687104.1	GAP1-N2+GAP1-M+GAP1-C+polar_tail+TM+beta-rich	TM+vWA(canonical)+YtKA-like+TM+??→ Tubulin+coiled_coil+??→ Tubulin+coiled_coil+HTH?+??→ TM+TM+TM+vWA(canonical)+TM+TM+TM+polar_tail→ Fibronectin-like_repeats+??→ ZnR+ZnR+DO-GTPase2.2+ZnR+ZnR→ GAP1-N2+GAP1-M+GAP1-C+polar_tail+TM+beta-rich*→	1009	Paenibacillus polymyxa	GCA_900454525.1	firmicutes
WP_019913381.1	GAP1-N2+GAP1-M+GAP1-C+polar_tail+TM+beta-rich	TM+vWA(canonical)+YtKA-like+TM+??→ Tubulin+coiled_coil+??→ Tubulin+coiled_coil+HTH?+??→ TM+TM+TM+vWA(canonical)+TM+TM+TM+polar_tail→ Fibronectin-like_repeats+??→ ZnR+ZnR+DO-GTPase2.2+ZnR+ZnR→ GAP1-N2+GAP1-M+GAP1-C+polar_tail+TM+beta-rich*→	1038	Paenibacillus sp. HW567	GCF_000374185.1	firmicutes
WP_020384555.1	GAP1-N2+GAP1-M+GAP1-C	polar_leader+TM+TM+TM→ TM+vWA(canonical)+Fibronectin-like_repeats+TM+beta-rich+lc_tail→ Tubulin+alpha_helical+??+??→ GAP_unknown1+TM+TM+GAP_unknown2→ HRDC-like?+Fibronectin-like_repeats→ GAP1-N2+GAP1-M+GAP1-C*→ beta-rich+vWA(canonical)→??+DO-GTPase2.2→ TM+TM+TM→	526	Corynebacterium doosanense	GCA_000767055.1	actinobacteria
WP_020440145.1	GAP1-N2+GAP1-M+GAP1-C	polar_leader+TM+TM+TM→ TM+vWA(canonical)+Fibronectin-like_repeats+TM+beta-rich+lc_tail→ Tubulin+alpha_helical+??+??→ GAP_unknown1+TM+TM+GAP_unknown2→ HRDC-like?+Fibronectin-like_repeats→ GAP1-N2+GAP1-M+GAP1-C*→ beta-rich+vWA(canonical)→??+DO-GTPase2.2→	613	Corynebacterium terpenotabidum	GCA_000418365.1	actinobacteria
WP_020464398.1	GAP1-N2+GAP1-M+GAP1-C	HSP70→ TPR_containing_alpha_helical+Fibronectin-like_repeats→ ZnR+ZnR+DO-GTPase2.2→ GAP1-N2+GAP1-M+GAP1-C*→ TM+TM+TM+TM+ZnR+ZnR+DO-GTPase2.2→	887	Frankia sp. EAN1pec	GCA_000018005.1	actinobacteria
WP_020471914.1	GAP1-N2+GAP1-M+GAP1-C	GAP1-N2+GAP1-M+GAP1-C*→	831	Zavarzinella formosa	GCF_000255705.1	planctomycetes
WP_020504915.1	GAP1-N2+GAP1-M+GAP1-C	GAP1-N2+GAP1-M+GAP1-C*→	1131	Lamprocystis purpurea	GCF_000379525.1	gammaproteobacteria
WP_020509485.1	GAP1-N2+GAP1-M+GAP1-C	HSP70→||←?||GrpE→ HSP70→ TPR_containing_alpha_helical+Fibronectin-like_repeats→ ZnR+ZnR+DO-GTPase2.2→ GAP1-N2+GAP1-M+GAP1-C*→ TM+TM+TM+TM+ZnR+ZnR+DO-GTPase2.2→	817	Actinoplanes globisporus	GCF_000379645.1	actinobacteria
WP_020610518.1	GAP1-N2+GAP1-M+GAP1-C	PP2C→ Pkinase→?→?→?→ HSP70_domain_2+low_complexity+domain_betaX→ ZnR+ZnR+DO-GTPase2.2→ GAP1-N2+GAP1-M+GAP1-C*→ TM+TM+TM+TM+ZnR+ZnR+DO-GTPase2.2→	816	Streptomyces sp. CNY243	GCF_000377165.1	actinobacteria
WP_020617137.1	GAP1-N2+GAP1-M+GAP1-C+polar_tail+TM+beta-rich	TM+vWA(canonical)+YtKA-like+TM+??→ Tubulin+coiled_coil+??→ Tubulin+coiled_coil+HTH?+??→ TM+TM+TM+vWA(canonical)+TM+TM+TM+polar_tail→ Fibronectin-like_repeats+??→?→ ZnR+ZnR+DO-GTPase2.2+ZnR+ZnR→ GAP1-N2+GAP1-M+GAP1-C+polar_tail+TM+beta-rich*→	1061	Paenibacillus daejeonensis	GCF_000378385.1	firmicutes
WP_020629738.1	GAP1-N2+GAP1-M+GAP1-C	GrpE→ HSP70→ TPR_containing_alpha_helical+Fibronectin-like_repeats→ ZnR+ZnR+DO-GTPase2.2→ GAP1-N2+GAP1-M+GAP1-C*→ TM+TM+TM+TM+ZnR+ZnR+DO-GTPase2.2→?→ HSP70→ vWA(canonical)→||←?←?←HSP70	816	Amycolatopsis alba	GCA_002234385.1	actinobacteria

acc	architecture	operon	len	species	gca	taxend
WP_020679048.1	GAP1-N2+GAP1-M+GAP1-C	PP2C→ Pkinase→?→?→?→ HSP70_domain_2+low_complexity+domain_betaX→ ZnR+ZnR+DO-GTPase2.2→ GAP1-N2+GAP1-M+GAP1-C*→ TM+TM+TM+TM+ZnR+ZnR+DO-GTPase2.2→	816	Streptomyces sp. CNQ766	GCF_000377105.1	actinobacteria
WP_020679138.1	GAP1-N2+GAP1-M+GAP1-C	PP2C→ Pkinase→?→?→?→ HSP70_domain_2+low_complexity+domain_betaX→ ZnR+ZnR+DO-GTPase2.2→ GAP1-N2+GAP1-M+GAP1-C*→ TM+TM+TM+TM+ZnR+ZnR+DO-GTPase2.2→	816	Streptomyces sp. CNS335	GCF_000377125.1	actinobacteria
WP_020933904.1	GAP1-N2+GAP1-M+GAP1-C	polar_leader+TM+TM+TM→ TM+vWA(canonical)+Fibronectin-like_repeats+TM+beta-rich+lc_tail→ Tubulin+alpha_helical+??+??→ GAP_unknown1+TM+TM+GAP_unknown2→ HRDC-like?+Fibronectin-like_repeats→ GAP1-N2+GAP1-M+GAP1-C*→ beta-rich+vWA(canonical)→??+DO-GTPase2.2→ TM+TM+TM→	631	Corynebacterium maris	GCA_000442645.1	actinobacteria
WP_020942943.1	GAP1-N2+GAP1-M	-	967	Streptomyces collinus	GCA_000444875.1	actinobacteria
WP_021836295.1	GAP1-N2+GAP1-M+GAP1-C	DnaJ+DnaJ+ZnR+ZnR+coiled_coil+Fibronectin-like_repeats→ ZnR+ZnR+DO-GTPase2.2(frag)→?→ GAP1-N2+GAP1-M+GAP1-C*→ TM+TM+TM+TM+TM+ZnR+ZnR+DO-GTPase2.2+TM+beta-sandwich+NTF-like→ GrpE→ HSP70→?→?→ HSP70+vWA(canonical)→	849	Crocosphaera watsonii	GCA_001039615.1	cyanobacteria
WP_022586058.1	GAP1-N2+GAP1-M+GAP1-C+polar_tail+TM+beta-rich	TM+vWA(canonical)+YtKA-like+TM+??→ Tubulin+coiled_coil+??→ Tubulin+coiled_coil+HTH?+??→ TM+TM+TM+vWA(canonical)+TM+TM+TM+polar_tail→ Fibronectin-like_repeats+??→ ZnR+ZnR+DO-GTPase2.2+ZnR+ZnR→ GAP1-N2+GAP1-M+GAP1-C+polar_tail+TM+beta-rich*→	833	Brevibacillus laterosporus	GCA_000472325.2	firmicutes
WP_023162773.1	GAP1-N2+GAP1-M+GAP1-C	Fibronectin-like_repeats+??→ ZnR+ZnR+DO-GTPase2.2→ GAP1-N2+GAP1-M+GAP1-C*→ TM+vWA(canonical)+YtKA-like+TM+??→ Tubulin+coiled_coil+??→ PSE→ TM+TM+TM+vWA(canonical)+TM+TM+TM+polar_tail→ Fibronectin-like_repeats+??→	868	Clostridium autoethanogenum	GCA_000484505.1	firmicutes
WP_023162785.1	GAP1-N2+GAP1-M	Tubulin+coiled_coil+??→ PSE→ TM+TM+TM+vWA(canonical)+TM+TM+TM+polar_tail→ Fibronectin-like_repeats+??→?→?→?→ ZnR+ZnR+DO-GTPase2.2+ZnR+ZnR→ GAP1-N2+GAP1-M*→	351	Clostridium autoethanogenum	GCA_000484505.1	firmicutes
WP_023373955.1	GAP1-N2+GAP1-M+GAP1-C	TM+DUF4190+TM→ TM+DUF4190+TM→ polar_leader+TM+TM+TM→ TM+vWA(canonical)+Fibronectin-like_repeats+TM+beta-rich+lc_tail→ Tubulin+alpha_helical+??+??→ GAP_unknown1+TM+TM+GAP_unknown2→ HRDC-like?+Fibronectin-like_repeats→ GAP1-N2+GAP1-M+GAP1-C*→ beta-rich+vWA(canonical)→??+DO-GTPase2.2→	819	Mycobacterium kansasii	GCA_000157895.2	actinobacteria
WP_023416404.1	GAP1-N2+GAP1-M+GAP1-C	GAP1-N2+GAP1-M+GAP1-C*→	789	Streptomyces sp. PVA 94-07	GCA_000495755.1	actinobacteria
WP_023421402.1	GAP1-N2+GAP1-M+GAP1-C	GAP1-N2+GAP1-M+GAP1-C*→	789	Streptomyces sp. GBA 94-10	GCA_000495635.1	actinobacteria
WP_023537168.1	GAP1-N2+GAP1-M+GAP1-C	GAP1-N2+GAP1-M+GAP1-C*→ vWA(canonical)→ PP2C→ Pkinase+ZnR+beta-rich→||←TPR_containing_alpha_helical+Fibronectin-like_repeats	865	Streptomyces niveus	GCA_000497425.1	actinobacteria
WP_023552702.1	GAP1-N2+GAP1-M+GAP1-C	Pkinase→?→?→?→?→ HSP70_domain_2+low_complexity+domain_betaX→ ZnR+ZnR+DO-GTPase2.2→ GAP1-N2+GAP1-M+GAP1-C*→ TM+TM+TM+TM+ZnR+ZnR+DO-GTPase2.2→	815	Streptomyces roseochromogenus	GCA_000497445.1	actinobacteria
WP_023559201.1	GAP1-N2+GAP1-M+GAP1-C+polar_tail+TM+beta-rich	TM+vWA(canonical)+YtKA-like+TM+??→ Tubulin+coiled_coil+??→ Tubulin+coiled_coil+HTH?+??→ TM+TM+TM+vWA(canonical)+TM+TM+TM+polar_tail→ Fibronectin-like_repeats+??→ ZnR+ZnR+DO-GTPase2.2+ZnR+ZnR→ GAP1-N2+GAP1-M+GAP1-C+polar_tail+TM+beta-rich*→	836	Brevibacillus panacihumi	GCA_000503775.1	firmicutes
WP_023921833.1	GAP1-N2+GAP1-M+GAP1-C	??→??+HTH?+GrpE→ HSP70→ DnaJ+DnaJ+ZnR+Fibronectin-like_repeats→ ZnR+ZnR+DO-GTPase2.2→ GAP1-N2+GAP1-M+GAP1-C*→?→ TM+TM+TM+TM+ZnR+ZnR+DO-GTPase2.2→	861	Ruminococcus lactaris	GCA_000507785.1	firmicutes
WP_023964422.1	GAP1-N2+GAP1-M+GAP1-C+polar_tail+TM+beta-rich	Tubulin+coiled_coil+??→ PSE→ TM+TM+TM+vWA(canonical)+TM+TM+TM+polar_tail→ Fibronectin-like_repeats+??→ ZnR+ZnR+DO-GTPase2.2+ZnR+ZnR→ GAP1-N2+GAP1-M+GAP1-C+polar_tail+TM+beta-rich*→	989	Paenibacillus sp. JCM 10914	GCA_000509425.1	firmicutes
WP_023988392.1	GAP1-N2+GAP1-M+GAP1-C+polar_tail+TM+beta-rich	TM+vWA(canonical)+YtKA-like+TM+??→ Tubulin+coiled_coil+??→ Tubulin+coiled_coil+HTH?+??→ TM+TM+TM+vWA(canonical)+TM+TM+TM+polar_tail→ Fibronectin-like_repeats+??→ ZnR+ZnR+DO-GTPase2.2+ZnR+ZnR→ GAP1-N2+GAP1-M+GAP1-C+polar_tail+TM+beta-rich*→	1002	Paenibacillus polymyxa	GCA_000507205.2	firmicutes
WP_024027639.1	GAP1-N2+GAP1-M+GAP1-C+polar_tail+TM+beta-rich	TM+vWA(canonical)+YtKA-like+TM+??→ Tubulin+coiled_coil+??→ Tubulin+coiled_coil+HTH?+??→ TM+TM+TM+vWA(canonical)+TM+TM+TM+polar_tail→ Fibronectin-like_repeats+??→ ZnR+ZnR+DO-GTPase2.2+ZnR+ZnR→ GAP1-N2+GAP1-M+GAP1-C+polar_tail+TM+beta-rich*→	953	Bacillus vireti	GCA_000508325.2	firmicutes
WP_024264121.1	GAP1-N2+GAP1-M+GAP1-C	GAP1-N2+GAP1-M+GAP1-C*→	829	Streptomyces filamentosus	GCA_000156695.2	actinobacteria
WP_024391768.1	GAP1-N2+GAP1-M+GAP1-C	HSP70→?→ ZnR+ZnR+DO-GTPase2.2→ GAP1-N2+GAP1-M+GAP1-C*→	697	Streptococcus suis	GCF_000440555.1	firmicutes
WP_024494939.1	GAP1-N2+GAP1-M+GAP1-C	GAP1-N2+GAP1-M+GAP1-C*→	735	Streptomyces sp. AW19M42	GCF_000470535.1	actinobacteria
WP_024632788.1	GAP1-N2+GAP1-M+GAP1-C+polar_tail	TM+TM+TM+vWA+TM+TM+TM+polar_tail→ Fibronectin-like_repeats+??→ ZnR+ZnR+DO-GTPase2.2→ GAP1-N2+GAP1-M+GAP1-C+polar_tail*→	1079	Paenibacillus	GCA_000499205.1	firmicutes
WP_024984503.1	GAP1-N2+GAP1-M+GAP1-C+polar_tail+TM+beta-rich	TM+vWA(canonical)+YtKA-like+TM+??→ Tubulin+coiled_coil+??→ Tubulin+coiled_coil+HTH?+??→ TM+TM+TM+vWA(canonical)+TM+TM+TM+polar_tail→ Fibronectin-like_repeats+??→ ZnR+ZnR+DO-GTPase2.2+ZnR+ZnR→ GAP1-N2+GAP1-M+GAP1-C+polar_tail+TM+beta-rich*→	920	Brevibacillus borstelensis	GCF_000612185.1	firmicutes
WP_025333415.1	GAP1-N2+GAP1-M+GAP1-C+polar_tail+TM+beta-rich	TM+vWA(canonical)+YtKA-like+TM+??→ Tubulin+coiled_coil+??→ Tubulin+coiled_coil+HTH?+??→ TM+TM+TM+vWA(canonical)+TM+TM+TM+polar_tail→ Fibronectin-like_repeats+??→ ZnR+ZnR+DO-GTPase2.2+ZnR+ZnR→ GAP1-N2+GAP1-M+GAP1-C+polar_tail+TM+beta-rich*→	1025	Paenibacillus sabinae	GCA_000612505.1	firmicutes
WP_025677111.1	GAP1-N2+GAP1-M+GAP1-C+polar_tail+TM+beta-rich	Tubulin+coiled_coil+??→ Tubulin+coiled_coil+HTH?+??→ TM+TM+TM+vWA(canonical)+TM+TM+TM+polar_tail→ Fibronectin-like_repeats+??→ ZnR+ZnR+DO-GTPase2.2+ZnR+ZnR→ GAP1-N2+GAP1-M+GAP1-C+polar_tail+TM+beta-rich*→	1000	Paenibacillus massiliensis	GCF_000520695.1	firmicutes
WP_025679141.1	GAP1-N2+GAP1-M+GAP1-C+polar_tail	Tubulin+coiled_coil+??→ Tubulin+coiled_coil+HTH?+??→ TM+TM+TM+vWA+TM+TM+TM+polar_tail→ Fibronectin-like_repeats+??→ ZnR+ZnR+DO-GTPase2.2→ GAP1-N2+GAP1-M+GAP1-C+polar_tail*→	1009	Paenibacillus polymyxa	GCF_000520795.1	firmicutes
WP_025685528.1	GAP1-N2+GAP1-M+GAP1-C+polar_tail+TM+beta-rich	TM+vWA(canonical)+YtKA-like+TM+??→ Tubulin+coiled_coil+??→ Tubulin+coiled_coil+HTH?+??→ TM+TM+TM+vWA(canonical)+TM+TM+TM+polar_tail→ Fibronectin-like_repeats+??→ ZnR+ZnR+DO-GTPase2.2+ZnR+ZnR→ GAP1-N2+GAP1-M+GAP1-C+polar_tail+TM+beta-rich*→	1006	Paenibacillus sp. 1-49	GCF_000520815.1	firmicutes
WP_025702099.1	GAP1-N2+GAP1-M+GAP1-C+polar_tail+TM+beta-rich	TM+TM+TM+vWA(canonical)+TM+TM+TM+polar_tail→ Fibronectin-like_repeats+??→ ZnR+ZnR+DO-GTPase2.2+ZnR+ZnR→ GAP1-N2+GAP1-M+GAP1-C+polar_tail+TM+beta-rich*→	750	Paenibacillus forsythiae	GCF_000520735.1	firmicutes
WP_025717315.1	GAP1-N2+GAP1-M+GAP1-C+polar_tail+TM+beta-rich	TM+vWA(canonical)+YtKA-like+TM+??→ Tubulin+coiled_coil+??→ Tubulin+coiled_coil+HTH?+??→ TM+TM+TM+vWA(canonical)+TM+TM+TM+polar_tail→ Fibronectin-like_repeats+??→ ZnR+ZnR+DO-GTPase2.2+ZnR+ZnR→ GAP1-N2+GAP1-M+GAP1-C+polar_tail+TM+beta-rich*→	1019	Paenibacillus sp. 1-18	GCF_000520755.1	firmicutes
WP_025724191.1	GAP1-N2+GAP1-M+GAP1-C+polar_tail+TM+beta-rich	TM+vWA(canonical)+YtKA-like+TM+??→ Tubulin+coiled_coil+??→ Tubulin+coiled_coil+HTH?+??→ TM+TM+TM+vWA(canonical)+TM+TM+TM+polar_tail→ Fibronectin-like_repeats+??→ ZnR+ZnR+DO-GTPase2.2+ZnR+ZnR→ GAP1-N2+GAP1-M+GAP1-C+polar_tail+TM+beta-rich*→	1002	Paenibacillus polymyxa	GCF_000520775.1	firmicutes
WP_025848105.1	GAP1-N2+GAP1-M+GAP1-C+polar_tail+TM+beta-rich	TM+vWA(canonical)+YtKA-like+TM+??→ Tubulin+coiled_coil+??→ Tubulin+coiled_coil+HTH?+??→ TM+TM+TM+vWA(canonical)+TM+TM+TM+polar_tail→ Fibronectin-like_repeats+??→ ZnR+ZnR+DO-GTPase2.2+ZnR+ZnR→ GAP1-N2+GAP1-M+GAP1-C+polar_tail+TM+beta-rich*→	834	Brevibacillus agri	GCF_000612125.1	firmicutes
WP_025849411.1	GAP1-N2+GAP1-M+GAP1-C+polar_tail+TM+Cadherin-like	TM+vWA(canonical)+YtKA-like+TM+??→ Tubulin+coiled_coil+??→ Tubulin+coiled_coil+HTH?+??→?→ TM+TM+TM+vWA(canonical)+TM+TM+TM+polar_tail→ Fibronectin-like_repeats+??→ ZnR+ZnR+DO-GTPase2.2+ZnR+ZnR→ GAP1-N2+GAP1-M+GAP1-C+polar_tail+TM+Cadherin-like*→	935	Paenibacillus ehimensis	GCF_000612225.1	firmicutes
WP_026400369.1	GAP1-N2+GAP1-M+GAP1-C	GrpE→ HSP70→ TPR_containing_alpha_helical+Fibronectin-like_repeats→ ZnR+ZnR+DO-GTPase2.2→ GAP1-N2+GAP1-M+GAP1-C*→ TM+TM+TM+TM+ZnR+ZnR+DO-GTPase2.2+TM→	810	Actinomadura rifamycini	GCF_000425065.1	actinobacteria
WP_026408761.1	GAP1-N2+GAP1-M+GAP1-C	GrpE→ HSP70→??+Fibronectin→ ZnR+ZnR+DO-GTPase2.2→ GAP1-N2+GAP1-M+GAP1-C*→ TM+TM+TM+TM+ZnR+ZnR+DO-GTPase2.2→	808	Actinomyces dentalis	GCF_000429225.1	actinobacteria
WP_026414948.1	GAP1-N2+GAP1-M+GAP1-C	GrpE→ HSP70→ TPR_containing_alpha_helical+Fibronectin-like_repeats→ ZnR+ZnR+DO-GTPase2.2→ GAP1-N2+GAP1-M+GAP1-C*→	767	Actinomadura oligospora	GCF_000518265.1	actinobacteria
WP_026423378.1	GAP1-N2+GAP1-M+GAP1-C+TM+beta-rich	vWA(canonical)+beta-rich+low_complexity→?→?→ Tubulin+alpha_helical+??+??→??+TM+TM+TM+TM+??→ Pkinase+TM→ ZnR+ZnR+DO-GTPase2.2→ GAP1-N2+GAP1-M+GAP1-C+TM+beta-rich*→	605	Actinokineospora inagensis	GCF_000482865.1	actinobacteria
WP_026557639.1	GAP1-N2+GAP1-M+GAP1-C+polar_tail+TM+beta-rich	TM+vWA(canonical)+YtKA-like+TM+??→ Tubulin+coiled_coil+??→ Tubulin+coiled_coil+HTH?+??→ TM+TM+TM+vWA(canonical)+TM+TM+TM+polar_tail→ Fibronectin-like_repeats+??→ ZnR+ZnR+DO-GTPase2.2+ZnR+ZnR→ GAP1-N2+GAP1-M+GAP1-C+polar_tail+TM+beta-rich*→	834	Bacillus sp. NSP2.1	GCF_000470975.1	firmicutes
WP_026567693.1	GAP1-N2+GAP1-M+GAP1-C+polar_tail+TM+beta-rich	TM+vWA(canonical)+YtKA-like+TM+??→ Tubulin+coiled_coil+??→ Tubulin+coiled_coil+HTH?+??→ Fibronectin-like_repeats+??→ ZnR+ZnR+DO-GTPase2.2+ZnR+ZnR→ GAP1-N2+GAP1-M+GAP1-C+polar_tail+TM+beta-rich*→	947	Bacillus sp. UNC41MFS5	GCF_000686805.1	firmicutes
WP_027013014.1	GAP1-N2+GAP1-M+GAP1-C	polar_leader+TM+TM+TM→ TM+vWA(canonical)+Fibronectin-like_repeats+TM+beta-rich+lc_tail→ Tubulin+alpha_helical+??+??→ GAP_unknown1+TM+TM+GAP_unknown2→ HRDC-like?+Fibronectin-like_repeats→ GAP1-N2+GAP1-M+GAP1-C*→ beta-rich+vWA(canonical)→??+DO-GTPase2.2→	708	Corynebacterium freiburgense	GCF_000428805.1	actinobacteria
WP_027019792.1	GAP1-N2+GAP1-M+GAP1-C	polar_leader+TM+TM+TM→||←?||TM+vWA(canonical)+Fibronectin-like_repeats+TM+beta-rich+lc_tail→ Tubulin+alpha_helical+??+??→ GAP_unknown1+TM+TM+GAP_unknown2→ HRDC-like?+Fibronectin-like_repeats→ GAP1-N2+GAP1-M+GAP1-C*→ beta-rich+vWA(canonical)→??+DO-GTPase2.2→	816	Corynebacterium sputi	GCF_000427865.1	actinobacteria
WP_027344127.1	GAP1-N2+GAP1-M+GAP1-C	TPR_containing_alpha_helical+Fibronectin-like_repeats→ ZnR+ZnR+DO-GTPase2.2→ GAP1-N2+GAP1-M+GAP1-C*→ TM+TM+TM+TM+ZnR+ZnR+DO-GTPase2.2→	799	Hamadaea tsunoensis	GCF_000428945.1	actinobacteria
WP_027623024.1	GAP1-N2+GAP1-M+GAP1-C	Fibronectin-like_repeats+??→ ZnR+ZnR+DO-GTPase2.2→ GAP1-N2+GAP1-M+GAP1-C*→ TM+TM+TM+TM+ZnR+ZnR+DO-GTPase2.2→ TM+vWA(canonical)+YtKA-like+TM+??→ Tubulin+coiled_coil+??→ Tubulin+coiled_coil+HTH?+??→ TM+TM+TM+vWA(canonical)+TM+TM+TM+polar_tail→ Fibronectin-like_repeats+??→ ZnR+ZnR+DO-GTPase2.2+ZnR+ZnR→	888	Clostridium lundense	GCF_000619945.1	firmicutes
WP_027740791.1	GAP1-N2+GAP1-M+GAP1-C	PP2C→ Pkinase→?→?→?→ HSP70_domain_2+low_complexity+domain_betaX→ ZnR+ZnR+DO-GTPase2.2→ GAP1-N2+GAP1-M+GAP1-C*→ TM+TM+TM+TM+ZnR+ZnR+DO-GTPase2.2→	816	Streptomyces sp. CNT371	GCF_000514995.1	actinobacteria
WP_027752538.1	GAP1-N2+GAP1-M+GAP1-C	PP2C→ Pkinase→?→?→?→ HSP70_domain_2+low_complexity+domain_betaX→ ZnR+ZnR+DO-GTPase2.2→ GAP1-N2+GAP1-M+GAP1-C*→ TM+TM+TM+TM+ZnR+ZnR+DO-GTPase2.2→	814	Streptomyces sp. CNH099	GCF_000514715.1	actinobacteria
WP_027761039.1	GAP1-N2+GAP1-M+GAP1-C	PP2C→ Pkinase→?→?→?→ HSP70_domain_2+low_complexity+domain_betaX→ ZnR+ZnR+DO-GTPase2.2→ GAP1-N2+GAP1-M+GAP1-C*→ TM+TM+TM+TM+ZnR+ZnR+DO-GTPase2.2→	861	Streptomyces sp. CNT318	GCF_000424765.1	actinobacteria
WP_027766703.1	GAP1-N2+GAP1-M+GAP1-C	PP2C→ Pkinase→?→?→?→ HSP70_domain_2+low_complexity+domain_betaX→ ZnR+ZnR+DO-GTPase2.2→ GAP1-N2+GAP1-M+GAP1-C*→ TM+TM+TM+TM+ZnR+ZnR+DO-GTPase2.2→	816	Streptomyces sp. CNQ865	GCF_000424805.1	actinobacteria
WP_027871381.1	GAP1-N2+GAP1-M+low_complexity+TM+TM	TM+vWA(canonical)+YtKA-like+IG-like+TM+??→ Tubulin+coiled_coil+??→?→?→ ZnR+insert+ZnR+DO-GTPase2.2+ZnR→ GAP1-N2+GAP1-M+low_complexity+TM+TM*→	517	[Eubacterium] cellulosolvens	GCF_000621585.1	firmicutes
WP_028128959.1	GAP1-N2+GAP1-M+GAP1-C	TM+TM+TM+TM+ZnR+ZnR+DO-GTPase2.2→ GrpE→ HSP70→ HSP70+vWA(canonical)→?→ ZnR+ZnR+DO-GTPase2.2→ GAP1-N2+GAP1-M+GAP1-C*→	709	Selenomonas sp. AE3005	GCF_000686945.1	firmicutes
WP_028418123.1	GAP1-N2+GAP1-M+GAP1-C	GAP1-N2+GAP1-M+GAP1-C*→	877	Streptomyces sp. SolWspMP-sol2th	GCF_000647875.1	actinobacteria
WP_028442687.1	GAP1-N2+GAP1-M+GAP1-C	GAP1-N2+GAP1-M+GAP1-C*→	803	Streptomyces	GCA_900091745.1	actinobacteria

acc	architecture	operon	len	species	gca	taxend
WP_028507202.1	GAP1-N2+GAP1-M+GAP1-C	TM+vWA(canonical)+YtKA-like+IG-like+TM+??→ Tubulin+coiled_coil+??→?→?→ ZnR+ZnR+DO-GTPase2.2+ZnR+ZnR→ GAP1-N2+GAP1-M+GAP1-C*→?→?→?→ Tubulin+coiled_coil+??→	853	Ruminococcaceae bacterium AB4001	GCF_000621765.1	firmicutes
WP_028510139.1	GAP1-N2+GAP1-M+GAP1-C	HSP70→?→ HSP70(frag)+vWA(canonical)→?→ ZnR+ZnR+Fibronectin-like_repeats→ ZnR+ZnR+DO-GTPase2.2→?→ GAP1-N2+GAP1-M+GAP1-C*→	898	Ruminococcus sp. NK3A76	GCF_000686125.1	firmicutes
WP_028510938.1	GAP1-N2+GAP1-M+GAP1-C	GrpE→ HSP70→ HSP70+vWA(canonical)→?→ ZnR+ZnR+DO-GTPase2.2→ GAP1-N2+GAP1-M+GAP1-C*→	920	Ruminococcus sp. NK3A76	GCF_000686125.1	firmicutes
WP_028542357.1	GAP1-N2+GAP1-M+GAP1-C+polar_tail+TM+beta-rich	TM+vWA(canonical)+YtKA-like+TM+??→ Tubulin+coiled_coil+??→ Tubulin+coiled_coil+HTH?+??→ TM+TM+TM+vWA(canonical)+TM+TM+TM+polar_tail→ Fibronectin-like_repeats+??→ ZnR+ZnR+DO-GTPase2.2+ZnR+ZnR→ GAP1-N2+GAP1-M+GAP1-C+polar_tail+TM+beta-rich*→	1002	Paenibacillus	GCA_900116035.1	firmicutes
WP_028547019.1	GAP1-N2+GAP1-M+GAP1-C+polar_tail+TM+beta-rich	TM+vWA(canonical)+YtKA-like+TM+??→ Tubulin+coiled_coil+??→ Tubulin+coiled_coil+HTH?+??→ TM+TM+TM+vWA(canonical)+TM+TM+TM+polar_tail→ Fibronectin-like_repeats+??→ ZnR+ZnR+DO-GTPase2.2+ZnR+ZnR→ GAP1-N2+GAP1-M+GAP1-C+polar_tail+TM+beta-rich*→	966	Paenibacillus taiwanensis	GCF_000425125.1	firmicutes
WP_028636929.1	GAP1-N2+GAP1-M+GAP1-C	polar_leader+TM+TM+TM→ TM+vWA(canonical)+Fibronectin-like_repeats+TM+beta-rich+lc_tail→ Tubulin+alpha_helical+??+??→ GAP_unknown1+TM+TM+GAP_unknown2→?→ HRDC-like?+Fibronectin-like_repeats→ GAP1-N2+GAP1-M+GAP1-C*→?→??+DO-GTPase2.2→	716	Nocardioides sp. URHA0032	GCF_000620645.1	actinobacteria
WP_029110494.1	GAP1-N2+GAP1-M+GAP1-C	HRDC-like?+Fibronectin-like_repeats→ GAP1-N2+GAP1-M+GAP1-C*→??+DO-GTPase2.2→	819	Mycobacterium sp. URHD0025	GCF_000426065.1	actinobacteria
WP_029162797.1	GAP1-N2+GAP1-M	TM+TM+TM+TM+ZnR+ZnR+DO-GTPase2.2→ TM+vWA(canonical)+YtKA-like+TM+??→ Tubulin+coiled_coil+??→ Tubulin+coiled_coil+HTH?+??→ TM+TM+TM+vWA(canonical)+TM+TM+TM+polar_tail→ Fibronectin-like_repeats+??→ ZnR+ZnR+DO-GTPase2.2+ZnR+ZnR→ GAP1-N2+GAP1-M*→	368	Clostridium scatologenes	GCA_000968375.1	firmicutes
WP_029368928.1	GAP1-N2+GAP1-M+GAP1-C	TM+DUF4190+TM→ polar_leader+TM+TM+TM→ PSE→ Tubulin+alpha_helical+??+??→ GAP_unknown1+TM+TM+GAP_unknown2→ HRDC-like?+Fibronectin-like_repeats→ GAP1-N2+GAP1-M+GAP1-C*→ beta-rich+vWA(canonical)→??+DO-GTPase2.2→ TM+DUF4190+TM+PknH_C→ TM+DUF4190+TM+PknH_C→ TM+DUF4190+TM+PknH_C→	837	Mycobacterium sp. UM_WWY	GCF_000455145.1	actinobacteria
WP_029519151.1	GAP1-N2+GAP1-M+GAP1-C+polar_tail+TM+beta-rich	TM+vWA(canonical)+YtKA-like+TM+??→ Tubulin+coiled_coil+??→ Tubulin+coiled_coil+HTH?+??→ TM+TM+TM+vWA(canonical)+TM+TM+TM+polar_tail→ Fibronectin-like_repeats+??→ ZnR+ZnR+DO-GTPase2.2+ZnR+ZnR→ GAP1-N2+GAP1-M+GAP1-C+polar_tail+TM+beta-rich*→	1005	Paenibacillus polymyxa	GCA_003941845.1	firmicutes
WP_030124720.1	GAP1-N2+GAP1-M+GAP1-C	GAP1-N2+GAP1-M+GAP1-C*→	804	Streptomyces	GCF_000716815.1	actinobacteria
WP_030172525.1	GAP1-N2+GAP1-M+GAP1-C	GrpE→ HSP70→ TPR_containing_alpha_helical+Fibronectin-like_repeats→ ZnR+ZnR+DO-GTPase2.2→ GAP1-N2+GAP1-M+GAP1-C*→ TM+TM+TM+TM+ZnR+ZnR+DO-GTPase2.2+TM→	612	Spirillospora albida	GCF_000718255.1	actinobacteria
WP_030350718.1	GAP1-N2+GAP1-M+GAP1-C	GAP1-N2+GAP1-M+GAP1-C*→	791	Streptomyces scopuliridis	GCA_003073355.1	actinobacteria
WP_030360297.1	GAP1-N2+GAP1-M+GAP1-C	GAP1-N2+GAP1-M+GAP1-C*→	970	Streptomyces	GCF_000716675.1	actinobacteria
WP_030386043.1	GAP1-N2+GAP1-M+GAP1-C	TPR_containing_alpha_helical+Fibronectin-like_repeats→ PSE→ GAP1-N2+GAP1-M+GAP1-C*→ TM+TM+TM+TM+ZnR+ZnR+DO-GTPase2.2+TM→ vWA(canonical)→ PP2C→ Pkinase+ZnR+beta-rich→	810	Streptomyces sp. NRRL S-241	GCF_000717925.1	actinobacteria
WP_030442497.1	GAP1-N2+GAP1-M+TM+GAP1-C	ZnR+ZnR+DO-GTPase2.2→ GAP1-N2+GAP1-M+TM+GAP1-C*→	726	Actinoplanes subtropicus	GCF_000721705.1	actinobacteria
WP_030455001.1	GAP1-N2+GAP1-M+GAP1-C+TM+low_complexity+TM	vWA(canonical)→ DO-GTPase1→ GAP1-N2+GAP1-M+GAP1-C+TM+low_complexity+TM*→	749	Herbidospora cretacea	GCF_000719065.1	actinobacteria
WP_030466420.1	GAP1-N2+GAP1-M+SLATT+SLATT	vWA(canonical)+beta-rich+low_complexity→ Tubulin+alpha_helical+??+??→??+TM+TM+TM+TM+??→ Pkinase+TM→ ZnR+ZnR+DO-GTPase2.2→ GAP1-N2+GAP1-M+SLATT+SLATT*→	653	Lechevalieria aerocolonigenes	GCF_000974445.1	actinobacteria
WP_030466670.1	GAP1-N2+GAP1-M+GAP1-C	TM+vWA(canonical)+TM→ HSP70→??+Fibronectin-like_repeats→ ZnR+ZnR+DO-GTPase2.2→ GAP1-N2+GAP1-M+GAP1-C*→ TM+TM+TM+TM+ZnR+ZnR+DO-GTPase2.2→||←GrpE	736	Lechevalieria aerocolonigenes	GCF_000974445.1	actinobacteria
WP_030562288.1	GAP1-N2+GAP1-M+GAP1-C	TPR_containing_alpha_helical+Fibronectin-like_repeats→ ZnR+ZnR+DO-GTPase2.2→ GAP1-N2+GAP1-M+GAP1-C*→ TM+TM+TM+TM+ZnR+ZnR+DO-GTPase2.2+TM→ vWA(canonical)→ PP2C→ Pkinase+ZnR+beta-rich→	850	Streptomyces cyaneofuscatus	GCF_000718135.1	actinobacteria
WP_030626361.1	GAP1-N2+GAP1-M+GAP1-C	GAP1-N2+GAP1-M+GAP1-C*→	803	Streptomyces	GCF_000720535.1	actinobacteria
WP_030637776.1	GAP1-N2+GAP1-M+GAP1-C	GAP1-N2+GAP1-M+GAP1-C*→	804	Streptomyces flavovirens	GCF_000720395.1	actinobacteria
WP_030670494.1	GAP1-N2+GAP1-M+GAP1-C	GrpE→ HSP70→ TPR_containing_alpha_helical+Fibronectin-like_repeats→ ZnR+ZnR+DO-GTPase2.2→ GAP1-N2+GAP1-M+GAP1-C*→ TM+TM+TM+TM+ZnR+ZnR+DO-GTPase2.2+Jelly_roll+beta-sandwich→||←STAND_NTPase+TM+TM+TM+TM+TM+TM+TM	476	Streptomyces cellulosae	GCF_000721105.1	actinobacteria
WP_030687754.1	GAP1-N2+GAP1-M+GAP1-C	GAP1-N2+GAP1-M+GAP1-C*→ vWA(canonical)→ PP2C→ Pkinase+ZnR+beta-rich→	812	Streptomyces sp. NRRL B-1347	GCF_000720325.1	actinobacteria
WP_030699417.1	GAP1-N2+GAP1-M+GAP1-C	GAP1-N2+GAP1-M+GAP1-C*→	789	Streptomyces griseus	GCF_000719495.1	actinobacteria
WP_030703758.1	GAP1-N2+GAP1-M	-	808	Streptomyces griseus	-	actinobacteria
WP_030709903.1	GAP1-N2+GAP1-M+GAP1-C	GAP1-N2+GAP1-M+GAP1-C*→	826	Streptomyces griseus	GCA_003989035.1	actinobacteria
WP_030719916.1	GAP1-N2+GAP1-M+GAP1-C	TPR_containing_alpha_helical+Fibronectin-like_repeats→ ZnR+ZnR+DO-GTPase2.2→ GAP1-N2+GAP1-M+GAP1-C*→ PSE→ vWA(canonical)→ PP2C→ Pkinase+ZnR+beta-rich→	814	Streptomyces sp. NRRL S-237	GCF_000718935.1	actinobacteria
WP_030760536.1	GAP1-N2+GAP1-M+GAP1-C	GAP1-N2+GAP1-M+GAP1-C*→||←PSE||vWA(canonical)→ PP2C→ Pkinase+ZnR+beta-rich→	814	Streptomyces sp. NRRL S-575	GCF_000718955.1	actinobacteria
WP_030766999.1	GAP1-N2+GAP1-M+GAP1-C	GAP1-N2+GAP1-M+GAP1-C*→	789	Streptomyces griseus	GCF_000719435.1	actinobacteria
WP_030779616.1	GAP1-N2+GAP1-M+GAP1-C	GAP1-N2+GAP1-M+GAP1-C*→ vWA(canonical)→ PP2C→ Pkinase+ZnR+beta-rich→||←TPR_containing_alpha_helical+Fibronectin-like_repeats	840	Streptomyces sp. NRRL S-920	GCF_000717945.1	actinobacteria
WP_030804300.1	GAP1-N2+GAP1-M+GAP1-C	GAP1-N2+GAP1-M+GAP1-C*→	830	Streptomyces mediolani	GCF_000721685.1	actinobacteria
WP_030878351.1	GAP1-N2+GAP1-M+GAP1-C	GAP1-N2+GAP1-M+GAP1-C*→ PSE→?→||←TPR_containing_alpha_helical+Fibronectin-like_repeats	792	Streptomyces sp. NRRL S-1868	GCF_000721305.1	actinobacteria
WP_030879622.1	GAP1-N2+GAP1-M+GAP1-C	GAP1-N2+GAP1-M+GAP1-C*→	804	Streptomyces sp. NRRL B-24051	GCF_000720405.1	actinobacteria
WP_030885101.1	GAP1-N2+GAP1-M+GAP1-C	GAP1-N2+GAP1-M+GAP1-C*→ PSE→?→||←TPR_containing_alpha_helical+Fibronectin-like_repeats	792	Streptomyces sp. NRRL F-5053	GCF_000721445.1	actinobacteria
WP_030900988.1	GAP1-N2+GAP1-M+GAP1-C	GAP1-N2+GAP1-M+GAP1-C*→	833	Streptomyces sp. NRRL F-5126	GCF_000721265.1	actinobacteria
WP_030902783.1	GAP1-N2+GAP1-M+GAP1-C	GAP1-N2+GAP1-M+GAP1-C*→||←PSE||vWA(canonical)→ PP2C→ Pkinase+ZnR+beta-rich→	814	Streptomyces sp. NRRL S-515	GCF_000720795.1	actinobacteria
WP_030917603.1	GAP1-N2+GAP1-M+GAP1-C	GAP1-N2+GAP1-M+GAP1-C*→	802	Streptomyces sp. NRRL B-24720	GCF_000721715.1	actinobacteria
WP_030974850.1	GAP1-N2+GAP1-M+GAP1-C	GAP1-N2+GAP1-M+GAP1-C*→	806	Streptomyces sp. NRRL S-1824	GCF_000719575.1	actinobacteria
WP_030998168.1	GAP1-N2+GAP1-M+GAP1-C	GAP1-N2+GAP1-M+GAP1-C*→	777	Streptomyces sp. NRRL F-5630	GCF_000719475.1	actinobacteria
WP_031086456.1	GAP1-N2+GAP1-M+GAP1-C	GAP1-N2+GAP1-M+GAP1-C*→	803	Streptomyces sp. NRRL WC-3549	GCF_000721935.1	actinobacteria
WP_031095640.1	GAP1-N2+GAP1-M+GAP1-C	GAP1-N2+GAP1-M+GAP1-C*→	804	Streptomyces	GCA_001294345.1	actinobacteria
WP_031176954.1	GAP1-N2+GAP1-M+GAP1-C	GAP1-N2+GAP1-M+GAP1-C*→	789	Streptomyces sp. NRRL B-3253	GCF_000725635.1	actinobacteria
WP_031186232.1	GAP1-N2+GAP1-M+GAP1-C	TPR_containing_alpha_helical+Fibronectin-like_repeats→ ZnR+ZnR+DO-GTPase2.2→ GAP1-N2+GAP1-M+GAP1-C*→ PSE→ vWA(canonical)→ PP2C→ Pkinase+ZnR+beta-rich→	880	Streptomyces	GCF_000725655.1	actinobacteria
WP_031192409.1	GAP1-N2+GAP1-M+GAP1-C	Pkinase→?→?→?→ HSP70_domain_2+low_complexity+domain_betaX→ ZnR+ZnR+DO-GTPase2.2→ GAP1-N2+GAP1-M+GAP1-C*→ TM+TM+TM+TM+ZnR+ZnR+DO-GTPase2.2→	814	Streptomyces	GCF_000716675.1	actinobacteria
WP_031412702.1	GAP1-N2+GAP1-M+GAP1-C+polar_tail+TM+beta-rich	TM+vWA(canonical)+YtKA-like+TM+??→ Tubulin+coiled_coil+??→ Tubulin+coiled_coil+HTH?+??→ TM+TM+TM+vWA(canonical)+TM+TM+TM+polar_tail→ Fibronectin-like_repeats+??→ ZnR+ZnR+DO-GTPase2.2+ZnR+ZnR→ GAP1-N2+GAP1-M+GAP1-C+polar_tail+TM+beta-rich*→	833	Brevibacillus laterosporus	-	firmicutes
WP_031463423.1	GAP1-N2+GAP1-M+GAP1-C+polar_tail+TM+beta-rich	TM+vWA(canonical)+YtKA-like+TM+??→ Tubulin+coiled_coil+??→ Tubulin+coiled_coil+HTH?+??→ TM+TM+TM+vWA(canonical)+TM+TM+TM+polar_tail→ Fibronectin-like_repeats+??→ ZnR+ZnR+DO-GTPase2.2+ZnR+ZnR→ GAP1-N2+GAP1-M+GAP1-C+polar_tail+TM+beta-rich*→	1009	Paenibacillus polymyxa	GCF_000735775.1	firmicutes
WP_031498745.1	GAP1-N2+GAP1-M+GAP1-C	TPR_containing_alpha_helical+Fibronectin-like_repeats→ ZnR+ZnR+DO-GTPase2.2→ GAP1-N2+GAP1-M+GAP1-C*→ TM+TM+TM+TM+ZnR+ZnR+DO-GTPase2.2+TM→ vWA(canonical)→ PP2C→ Pkinase+ZnR+beta-rich→	953	Streptomyces bicolor	GCF_000725465.1	actinobacteria
WP_031540405.1	GAP1-N2+GAP1-M+GAP1-C+polar_tail+TM+beta-rich	GAP1-N2+GAP1-M+GAP1-C+polar_tail+TM+beta-rich*→	746	Bacillus	GCF_001076885.1	firmicutes
WP_031932515.1	GAP1-N2+GAP1-M+GAP1-C+polar_tail+TM+beta-rich	TM+vWA(canonical)+YtKA-like+TM+??→ Tubulin+coiled_coil+??→ Tubulin+coiled_coil+HTH?+??→ TM+TM+TM+vWA(canonical)+TM+TM+TM+polar_tail→ Fibronectin-like_repeats+??→ ZnR+ZnR+DO-GTPase2.2+ZnR+ZnR→ GAP1-N2+GAP1-M+GAP1-C+polar_tail+TM+beta-rich*→	920	Brevibacillus borstelensis	GCA_000738785.1	firmicutes
WP_032079599.1	GAP1-N2+GAP1-M	TM+TM+TM+TM+ZnR+ZnR+DO-GTPase2.2→ TM+vWA(canonical)+YtKA-like+TM+??→ Tubulin+coiled_coil+??→ Tubulin+coiled_coil+HTH?+??→ TM+TM+TM+vWA(canonical)+TM+TM+TM+polar_tail→ Fibronectin-like_repeats+??→ ZnR+ZnR+DO-GTPase2.2+ZnR+ZnR→ GAP1-N2+GAP1-M*→	369	Clostridium drakei	GCA_003096175.1	firmicutes

acc	architecture	operon	len	species	gca	taxend
WP_032748152.1	GAP1-N2+GAP1-M+GAP1-C	GAP1-N2+GAP1-M+GAP1-C*→	830	Streptomyces globisporus	GCA_000261345.2	actinobacteria
WP_032786924.1	GAP1-N2+GAP1-M+GAP1-C	GAP1-N2+GAP1-M+GAP1-C*→	826	Streptomyces sp. JS01	GCA_000743295.1	actinobacteria
WP_032791831.1	GAP1-N2+GAP1-M+GAP1-C	ZnR+ZnR+DO-GTPase2.2→ PSE→ TM+TM+TM+TM+ZnR+ZnR+DO-GTPase2.2+TM→ vWA(canonical)→ PP2C→ Pkinase+ZnR+beta-rich→||←?←?||GAP1-N2+GAP1-M+GAP1-C*→	833	Streptomyces sp. W007	-	actinobacteria
WP_032917154.1	GAP1-N2+GAP1-M+GAP1-C	TPR_containing_alpha_helical+Fibronectin-like_repeats→ ZnR+ZnR+DO-GTPase2.2→ GAP1-N2+GAP1-M+GAP1-C*→ TM+TM+TM+TM+ZnR+ZnR+DO-GTPase2.2+TM→ vWA(canonical)→ PP2C→ Pkinase+ZnR+beta-rich→	820	Streptomyces rimosus	GCF_000720725.1	actinobacteria
WP_033090710.1	GAP1-N2+GAP1-M+GAP1-C	Pkinase+TM→ ZnR+DO-GTPase2.1→ GAP1-N2+GAP1-M+GAP1-C*→	696	Nocardia seriolae	GCA_002356035.1	actinobacteria
WP_033217760.1	GAP1-N2+GAP1-M+GAP1-C	TPR_containing_alpha_helical+Fibronectin-like_repeats→ ZnR+ZnR+DO-GTPase2.2→ GAP1-N2+GAP1-M+GAP1-C*→ PSE→ vWA(canonical)→ PP2C→ Pkinase+ZnR+beta-rich→	814	Streptomyces virginiae	GCF_000716685.1	actinobacteria
WP_033238161.1	GAP1-N2+GAP1-M+GAP1-C	GAP1-N2+GAP1-M+GAP1-C*→	789	Streptomyces griseus	GCF_000717375.1	actinobacteria
WP_033281599.1	GAP1-N2+GAP1-M+GAP1-C	GAP1-N2+GAP1-M+GAP1-C*→	814	Streptomyces sp. NRRL F-525	GCF_000716605.1	actinobacteria
WP_033300149.1	GAP1-N2+GAP1-M+GAP1-C	GAP1-N2+GAP1-M+GAP1-C*→	806	Streptomyces atroolivaceus	GCF_000717025.1	actinobacteria
WP_033322584.1	GAP1-N2+GAP1-M+GAP1-C	GAP1-N2+GAP1-M+GAP1-C*→ vWA(canonical)→ PP2C→ Pkinase+ZnR+beta-rich→||←TPR_containing_alpha_helical+Fibronectin-like_repeats	806	Streptomyces yerevanensis	GCF_000716805.1	actinobacteria
WP_033359526.1	GAP1-N2+GAP1-M+GAP1-C	GAP1-N2+GAP1-M+GAP1-C*→||←HSP70_domain_1	782	Dactylosporangium aurantiacum	GCF_000716715.1	actinobacteria
WP_033439532.1	GAP1-N2+GAP1-M+GAP1-C	GAP1-N2+GAP1-M+GAP1-C*→	751	Saccharothrix sp. NRRL B-16314	GCF_000716595.1	actinobacteria
WP_033947549.1	GAP1-N2+GAP1-M+GAP1-C	GAP1-N2+GAP1-M+GAP1-C*→	789	Streptomyces sp. CNQ431	GCF_000797385.1	actinobacteria
WP_034236272.1	GAP1-N2+GAP1-M+GAP1-C	polar_leader+TM+TM+TM→ TM+vWA(canonical)+Fibronectin-like_repeats+TM+beta-rich+lc_tail→ Tubulin+alpha_helical+??+??→ GAP_unknown1+TM+TM+GAP_unknown2→ HRDC-like?+Fibronectin-like_repeats→ GAP1-N2+GAP1-M+GAP1-C*→?→??+DO-GTPase2.2→	891	Actinomyces urogenitalis	GCA_000758745.1	actinobacteria
WP_034382650.1	GAP1-N2+GAP1-M+GAP1-C	GrpE→ HSP70→ TPR_containing_alpha_helical+Fibronectin-like_repeats→ ZnR+ZnR+DO-GTPase2.2→ GAP1-N2+GAP1-M+GAP1-C*→ TM+TM+TM+TM+ZnR+ZnR+DO-GTPase2.2+TM→	725	Herbidospora cretacea	GCF_000719065.1	actinobacteria
WP_034412972.1	GAP1-N2+GAP1-M+GAP1-C	HSP70+vWA(canonical)→ DnaJ+DnaJ+ZnR+ZnR+coiled_coil+Fibronectin-like_repeats→ ZnR+ZnR+DO-GTPase2.2→ GAP1-N2+GAP1-M+GAP1-C*→	825	Candidatus Entotheonella palauensis	GCA_000522425.1	nitrospinae/tectomicrobia group
WP_034447882.1	GAP1-N2+GAP1-M+GAP1-C	TM+vWA(canonical)+YtKA-like+IG-like+TM+??→ Tubulin+coiled_coil+??→?→?→ ZnR+ZnR+DO-GTPase2.2+ZnR+ZnR→ GAP1-N2+GAP1-M+GAP1-C*→?→?→?→ Tubulin+coiled_coil+??→ Tubulin+coiled_coil+??→	810	Butyrivibrio sp. AE2032	GCF_000702965.1	firmicutes
WP_034527772.1	GAP1-N2+GAP1-M+GAP1-C	TM+TM+TM+TM+ZnR+ZnR+DO-GTPase2.2→ GrpE→ HSP70→ HSP70+vWA(canonical)→ DnaJ+DnaJ+ZnR+ZnR+coiled_coil+Fibronectin-like_repeats→ ZnR+ZnR+DO-GTPase2.2→ GAP1-N2+GAP1-M+GAP1-C*→	968	Bifidobacterium stellenboschense	GCF_000741785.1	actinobacteria
WP_035113596.1	GAP1-N2+GAP1-M+GAP1-C	Tubulin+alpha_helical+??+??→?→ GAP_unknown1+TM+TM+GAP_unknown2→ HRDC-like?+Fibronectin-like_repeats→ HRDC-like?+Fibronectin-like_repeats→ GAP1-N2+GAP1-M+GAP1-C*→?→?→??+DO-GTPase2.2→	393	Corynebacterium auriscanis	GCA_000767255.1	actinobacteria
WP_035144553.1	GAP1-N2+GAP1-M+GAP1-C	Fibronectin-like_repeats+??→ ZnR+ZnR+DO-GTPase2.2→ GAP1-N2+GAP1-M+GAP1-C*→ TM+TM+TM+TM+ZnR+ZnR+DO-GTPase2.2→ TM+vWA(canonical)+YtKA-like+TM+??→ Tubulin+coiled_coil+??→ Tubulin+coiled_coil+HTH?+??→ TM+TM+TM+vWA(canonical)+TM+TM+TM+polar_tail→ Fibronectin-like_repeats+??→ ZnR+ZnR+DO-GTPase2.2+ZnR+ZnR→	888	Clostridium tetanomorphum	GCA_000647895.1	firmicutes
WP_035377609.1	GAP1-N2+GAP1-M+GAP1-C	GAP1-N2+GAP1-M+GAP1-C*→	853	Fervidicella metallireducens	GCA_000601455.1	firmicutes
WP_035417827.1	GAP1-N2+GAP1-M+GAP1-C	GAP1-N2+GAP1-M+GAP1-C*→	685	Bacillus sp. JCM 19045	GCA_000576325.1	firmicutes
WP_035441099.1	GAP1-N2+GAP1-M+GAP1-C	GAP1-N2+GAP1-M+GAP1-C*→	618	Bacillus sp. JCM 19046	GCA_000576345.1	firmicutes
WP_035847394.1	GAP1-N2+GAP1-M+GAP1-C	TPR_containing_alpha_helical+Fibronectin-like_repeats→ ZnR+ZnR+DO-GTPase2.2→ GAP1-N2+GAP1-M+GAP1-C*→ TM+TM+TM+TM+ZnR+ZnR+DO-GTPase2.2+TM→ vWA(canonical)→ PP2C→ Pkinase+ZnR+beta-rich→	909	Kitasatospora azatica	GCF_000744785.1	actinobacteria
WP_036337168.1	GAP1-N2+GAP1-M+GAP1-C	Pkinase+TM→ ZnR+ZnR+DO-GTPase2.2→ GAP1-N2+GAP1-M+GAP1-C*→	559	Modestobacter caceresii	GCA_000761485.1	actinobacteria
WP_036390676.1	GAP1-N2+GAP1-M+GAP1-C	HRDC-like?+Fibronectin-like_repeats→ GAP1-N2+GAP1-M+GAP1-C*→??+DO-GTPase2.2→	819	Mycolicibacterium farcinogenes	GCA_000723385.1	actinobacteria
WP_036430320.1	GAP1-N2+GAP1-M+GAP1-C	TM+DUF4190+TM→ polar_leader+TM+TM+TM→ PSE→ Tubulin+alpha_helical+??+??→ GAP_unknown1+TM+TM+GAP_unknown2→ HRDC-like?+Fibronectin-like_repeats→ GAP1-N2+GAP1-M+GAP1-C*→ beta-rich+vWA(canonical)→??+DO-GTPase2.2→ TM+DUF4190+TM+PknH_C→ TM+DUF4190+TM+PknH_C→	826	Mycolicibacterium mageritense	GCA_000612825.1	actinobacteria
WP_036443731.1	GAP1-N2+GAP1-M+GAP1-C	HRDC-like?+Fibronectin-like_repeats→ GAP1-N2+GAP1-M+GAP1-C*→??+DO-GTPase2.2→	819	Mycolicibacterium	GCA_000612885.1	actinobacteria
WP_036550689.1	GAP1-N2+GAP1-M+GAP1-C	Pkinase+TM→ ZnR+DO-GTPase2.1→ GAP1-N2+GAP1-M+GAP1-C*→	696	Nocardia seriolae	GCA_000583715.2	actinobacteria
WP_036663727.1	GAP1-N2+GAP1-M+GAP1-C+polar_tail+TM+beta-rich	TM+vWA(canonical)+YtKA-like+TM+??→ Tubulin+coiled_coil+??→ Tubulin+coiled_coil+HTH?+??→ TM+TM+TM+vWA(canonical)+TM+TM+TM+polar_tail→ Fibronectin-like_repeats+??→ ZnR+ZnR+DO-GTPase2.2+ZnR+ZnR→ GAP1-N2+GAP1-M+GAP1-C+polar_tail+TM+beta-rich*→	992	Paenibacillus sp. FSL H8-457	GCA_000517945.1	firmicutes
WP_036667820.1	GAP1-N2+GAP1-M+GAP1-C+polar_tail+TM+beta-rich	TM+vWA(canonical)+YtKA-like+TM+??→ Tubulin+coiled_coil+??→ Tubulin+coiled_coil+HTH?+??→ TM+TM+TM+vWA(canonical)+TM+TM+TM+polar_tail→ Fibronectin-like_repeats+??→ ZnR+ZnR+DO-GTPase2.2+ZnR+ZnR→ GAP1-N2+GAP1-M+GAP1-C+polar_tail+TM+beta-rich*→	1072	Paenibacillus	GCA_000517845.1	firmicutes
WP_036684993.1	GAP1-N2+GAP1-M+GAP1-C+polar_tail+TM+Cadherin-like	TM+vWA(canonical)+YtKA-like+TM+??→ Tubulin+coiled_coil+??→ Tubulin+coiled_coil+HTH?+??→?→ TM+TM+TM+vWA(canonical)+TM+TM+TM+polar_tail→ Fibronectin-like_repeats+??→ ZnR+ZnR+DO-GTPase2.2+ZnR+ZnR→ GAP1-N2+GAP1-M+GAP1-C+polar_tail+TM+Cadherin-like*→	936	Paenibacillus tyrfis	GCA_000722545.1	firmicutes
WP_037285325.1	GAP1-N2+GAP1-M+GAP1-C+polar_tail	Fibronectin-like_repeats+??→ ZnR+ZnR+DO-GTPase2.2→ GAP1-N2+GAP1-M+GAP1-C+polar_tail*→	1075	Saccharibacillus sacchari	GCF_000585395.1	firmicutes
WP_037629188.1	GAP1-N2+GAP1-M+GAP1-C	TPR_containing_alpha_helical+Fibronectin-like_repeats→ ZnR+ZnR+DO-GTPase2.2→ GAP1-N2+GAP1-M+GAP1-C*→ PSE→ vWA(canonical)→ PP2C→ Pkinase+ZnR+beta-rich→	880	Streptomyces	GCF_000716535.1	actinobacteria
WP_037664262.1	GAP1-N2+GAP1-M+GAP1-C	GAP1-N2+GAP1-M+GAP1-C*→ vWA(canonical)→	763	Streptomyces aurantiacus	GCF_000414115.1	actinobacteria
WP_037687853.1	GAP1-N2+GAP1-M+GAP1-C	GAP1-N2+GAP1-M+GAP1-C*→	737	Streptomyces lavendulae	GCA_002803845.1	actinobacteria
WP_037701615.1	GAP1-N2+GAP1-M+GAP1-C	GAP1-N2+GAP1-M+GAP1-C*→	805	Streptomyces atratus	GCF_000745675.1	actinobacteria
WP_037715874.1	GAP1-N2+GAP1-M+GAP1-C	TPR_containing_alpha_helical+Fibronectin-like_repeats→ ZnR+ZnR+DO-GTPase2.2→ GAP1-N2+GAP1-M+GAP1-C*→ TM+TM+TM+TM+ZnR+ZnR+DO-GTPase2.2+TM→ vWA(canonical)→ PP2C→ Pkinase+ZnR+beta-rich→	968	Streptomyces mirabilis	GCF_000745715.1	actinobacteria
WP_037741390.1	GAP1-N2+GAP1-M+GAP1-C	TPR_containing_alpha_helical+Fibronectin-like_repeats→ ZnR+ZnR+DO-GTPase2.2→ GAP1-N2+GAP1-M+GAP1-C*→ TM+TM+TM+TM+ZnR+ZnR+DO-GTPase2.2+TM→ vWA(canonical)→ PP2C→ Pkinase+ZnR+beta-rich→	939	Streptomyces mirabilis	GCF_000746395.1	actinobacteria
WP_037748429.1	GAP1-N2+GAP1-M+GAP1-C	PP2C→ Pkinase→?→?→?→ HSP70_domain_2+low_complexity+domain_betaX→ ZnR+ZnR+DO-GTPase2.2→ GAP1-N2+GAP1-M+GAP1-C*→ TM+TM+TM+TM+ZnR+ZnR+DO-GTPase2.2→	816	Streptomyces sp. CNQ-525	GCA_004217635.1	actinobacteria
WP_037831065.1	GAP1-N2+GAP1-M+GAP1-C	GAP1-N2+GAP1-M+GAP1-C*→	804	Streptomyces sp. NRRL S-325	GCF_000721595.1	actinobacteria
WP_037842919.1	GAP1-N2+GAP1-M+GAP1-C	GAP1-N2+GAP1-M+GAP1-C*→	789	Streptomyces sp. NRRL F-6628	GCF_000721455.1	actinobacteria
WP_037880477.1	GAP1-N2+GAP1-M+GAP1-C	GAP1-N2+GAP1-M+GAP1-C*→	803	Streptomyces sp. NTK 937	GCA_000698495.1	actinobacteria
WP_037911982.1	GAP1-N2+GAP1-M+GAP1-C	TPR_containing_alpha_helical+Fibronectin-like_repeats→ ZnR+ZnR+DO-GTPase2.2→ GAP1-N2+GAP1-M+GAP1-C*→ PSE→ vWA(canonical)→ PP2C→ Pkinase+ZnR+beta-rich→	880	Streptomyces sp. NRRL WC-3744	GCF_000717895.1	actinobacteria
WP_037933696.1	GAP1-N2+GAP1-M+GAP1-C	GAP1-N2+GAP1-M+GAP1-C*→	772	Streptomyces	GCF_900091485.1	actinobacteria
WP_038563339.1	GAP1-N2+GAP1-M+GAP1-C	Tubulin+alpha_helical+??+??→ GAP_unknown1+TM+TM+GAP_unknown2→ HRDC-like?+Fibronectin-like_repeats→ GAP1-N2+GAP1-M+GAP1-C*→ beta-rich+vWA(canonical)→??+DO-GTPase2.2→ TM+DUF4190+TM+PknH_C→ TM+DUF4190+TM+PknH_C→	828	Mycobacteriaceae	GCA_000416365.2	actinobacteria
WP_039273358.1	GAP1-N2+GAP1-M+GAP1-C+polar_tail+TM+beta-rich	TM+vWA(canonical)+YtKA-like+TM+??→ Tubulin+coiled_coil+??→ Tubulin+coiled_coil+HTH?+??→ TM+TM+TM+vWA(canonical)+TM+TM+TM+polar_tail→ Fibronectin-like_repeats+??→ ZnR+ZnR+DO-GTPase2.2+ZnR+ZnR→ GAP1-N2+GAP1-M+GAP1-C+polar_tail+TM+beta-rich*→	1005	Paenibacillus polymyxa	GCA_000714835.1	firmicutes
WP_039326669.1	GAP1-N2+GAP1-M+GAP1-C	HRDC-like?+Fibronectin-like_repeats→ GAP1-N2+GAP1-M+GAP1-C*→??+DO-GTPase2.2→	819	Mycolicibacterium setense	GCA_000805375.1	actinobacteria
WP_039376607.1	GAP1-N2+GAP1-M+GAP1-C	HRDC-like?+Fibronectin-like_repeats→ GAP1-N2+GAP1-M+GAP1-C*→??+DO-GTPase2.2→	819	Mycolicibacterium setense	GCA_000805385.1	actinobacteria
WP_039775456.1	GAP1-N2+GAP1-M+GAP1-C	Tubulin+alpha_helical+??+??→ GAP_unknown1+TM+TM+GAP_unknown2→ HRDC-like?+Fibronectin-like_repeats→ GAP1-N2+GAP1-M+GAP1-C*→?→??+DO-GTPase2.2→	919	Actinomyces oris	GCF_000180155.1	actinobacteria
WP_039870046.1	GAP1-N2+GAP1-M+GAP1-C+polar_tail+TM+beta-rich	TM+vWA(canonical)+YtKA-like+TM+??→ Tubulin+coiled_coil+??→ Tubulin+coiled_coil+HTH?+??→||←?||TM+TM+TM+vWA(canonical)+TM+TM+TM+polar_tail→ Fibronectin-like_repeats+??→ ZnR+ZnR+DO-GTPase2.2+ZnR+ZnR→ GAP1-N2+GAP1-M+GAP1-C+polar_tail+TM+beta-rich*→	1111	Paenibacillus sp. FSL R7-0273	GCA_000758625.1	firmicutes

acc	architecture	operon	len	species	gca	taxend
WP_040101454.1	GAP1-N2+GAP1-M+GAP1-C+polar_tail	Tubulin+coiled_coil+??→ Tubulin+coiled_coil+HTH?+??→ TM+TM+TM+vWA+TM+TM+TM+polar_tail→ Fibronectin-like_repeats+??→ ZnR+ZnR+DO-GTPase2.2→ GAP1-N2+GAP1-M+GAP1-C+polar_tail*→	1009	Paenibacillus polymyxa	GCA_000819665.1	firmicutes
WP_040195797.1	GAP1-N2+GAP1-M+TM+GAP1-C+TM	ZnR+ZnR+DO-GTPase2.2→ GAP1-N2+GAP1-M+TM+GAP1-C+TM*→	776	Candidatus Soleaferrea massiliensis	GCF_000820765.1	firmicutes
WP_040333054.1	GAP1-N2+GAP1-M+TM+GAP1-C	GAP1-N2+GAP1-M+TM+GAP1-C*→||←Pkinase	611	Butyricicoccus pullicaecorum	GCF_900143195.1	firmicutes
WP_040361063.1	GAP1-N2+GAP1-M+GAP1-C	polar_leader+TM+TM+TM→ TM+vWA(canonical)+Fibronectin-like_repeats+TM+beta-rich+lc_tail→ Tubulin+alpha_helical+??+??→ GAP_unknown1+TM+TM+GAP_unknown2→ HRDC-like?+Fibronectin-like_repeats→ GAP1-N2+GAP1-M+GAP1-C*→ beta-rich+vWA(canonical)→??+DO-GTPase2.2→	863	Corynebacterium amycolatum	GCF_000173655.1	actinobacteria
WP_040373124.1	GAP1-N2+GAP1-M+GAP1-C+polar_tail+TM+beta-rich	TM+vWA(canonical)+YtKA-like+TM+??→ PSE→ Tubulin+coiled_coil+HTH?+??→ TM+TM+TM+vWA(canonical)+TM+TM+TM+polar_tail→ Fibronectin-like_repeats+??→ ZnR+ZnR+DO-GTPase2.2+ZnR+ZnR→ GAP1-N2+GAP1-M+GAP1-C+polar_tail+TM+beta-rich*→	868	Bacillus psychrosaccharolyticus	GCF_000305495.1	firmicutes
WP_040705664.1	GAP1-N2+GAP1-M+GAP1-C	GrpE→ HSP70→ TPR_containing_alpha_helical+Fibronectin-like_repeats→ ZnR+ZnR+DO-GTPase2.2→ GAP1-N2+GAP1-M+GAP1-C*→ TM+TM+TM+TM+ZnR+ZnR+DO-GTPase2.2→?→ HSP70→ vWA(canonical)→	805	Nocardia takedensis	GCF_000308695.1	actinobacteria
WP_040816614.1	GAP1-N2+GAP1-M+GAP1-C	Pkinase+TM→ ZnR+DO-GTPase2.1→ GAP1-N2+GAP1-M+GAP1-C*→	705	Nocardia concava	GCF_000308815.1	actinobacteria
WP_040860755.1	GAP1-N2+GAP1-M+GAP1-C	Pkinase+TM→ ZnR+DO-GTPase2.1→ GAP1-N2+GAP1-M+GAP1-C*→	742	Nocardia niigatensis	GCF_000308655.1	actinobacteria
WP_041241565.1	GAP1-N2+GAP1-M+GAP1-C	Pkinase+TM→ ZnR+ZnR+DO-GTPase2.2→ GAP1-N2+GAP1-M+GAP1-C*→||←?←?||?→ Pkinase+low_complexity→	555	Geodermatophilus obscurus	GCF_000025345.1	actinobacteria
WP_041992098.1	GAP1-N2+GAP1-M+GAP1-C	GAP1-N2+GAP1-M+GAP1-C*→	824	Streptomyces sp. AcH 505	GCF_000818175.1	actinobacteria
WP_042229672.1	GAP1-N2+GAP1-M+GAP1-C+polar_tail+TM+Cadherin-like	TM+vWA(canonical)+YtKA-like+TM+??→ Tubulin+coiled_coil+??→ Tubulin+coiled_coil+HTH?+??→ TM+TM+TM+vWA(canonical)+TM+TM+TM+polar_tail→ Fibronectin-like_repeats+??→ ZnR+ZnR+DO-GTPase2.2+ZnR+ZnR→ GAP1-N2+GAP1-M+GAP1-C+polar_tail+TM+Cadherin-like*→	965	Paenibacillus chitinolyticus	GCA_004117095.1	firmicutes
WP_042284210.1	GAP1-N2+GAP1-M+GAP1-C	TPR_containing_alpha_helical+Fibronectin-like_repeats→ ZnR+ZnR+DO-GTPase2.2→ GAP1-N2+GAP1-M+GAP1-C*→ TM+TM+TM+TM+ZnR+ZnR+DO-GTPase2.2→	868	Nocardiopsis alba	GCF_000710955.1	actinobacteria
WP_042495823.1	GAP1-N2+GAP1-M	-	808	Streptomyces griseus	-	actinobacteria
WP_043195346.1	GAP1-N2+GAP1-M+GAP1-C	TPR_containing_alpha_helical+Fibronectin-like_repeats→ ZnR+ZnR+DO-GTPase2.2→ GAP1-N2+GAP1-M+GAP1-C*→ PSE→ vWA(canonical)→ PP2C→ Pkinase+ZnR+beta-rich→	880	Streptomyces sp. NRRL WC-3725	GCF_000720295.1	actinobacteria
WP_043231379.1	GAP1-N2+GAP1-M+GAP1-C	TPR_containing_alpha_helical+Fibronectin-like_repeats→ ZnR+ZnR+DO-GTPase2.2→ GAP1-N2+GAP1-M+GAP1-C*→ TM+TM+TM+TM+ZnR+ZnR+DO-GTPase2.2+TM→?→ vWA(canonical)→ PP2C→ Pkinase+ZnR+beta-rich→	820	Streptomyces sp. NRRL F-5193	GCF_000721515.1	actinobacteria
WP_043454546.1	GAP1-N2+GAP1-M+GAP1-C	TPR_containing_alpha_helical+Fibronectin-like_repeats→ ZnR+ZnR+DO-GTPase2.2→ GAP1-N2+GAP1-M+GAP1-C*→ TM+TM+TM+TM+ZnR+ZnR+DO-GTPase2.2+TM→ vWA(canonical)→ PP2C→ Pkinase+ZnR+beta-rich→	870	Streptomyces fulvoviolaceus	GCF_000718165.1	actinobacteria
WP_043492539.1	GAP1-N2+GAP1-M+GAP1-C	GAP1-N2+GAP1-M+GAP1-C*→	808	Streptomyces bingchenggensis	GCF_000092385.1	actinobacteria
WP_043524316.1	GAP1-N2+GAP1-M+TM+GAP1-C	DO-GTPase1→ GAP1-N2+GAP1-M+TM+GAP1-C*→?→?→ TM+vWA(canonical)+TM+low_complexity→ CASPASE+low_complexity→ TPR_region+CASPASE→	872	Actinoplanes utahensis	GCA_000785985.1	actinobacteria
WP_043930432.1	GAP1-N2+GAP1-M+GAP1-C+polar_tail+TM+beta-rich	TM+vWA(canonical)+YtKA-like+TM+??→ Tubulin+coiled_coil+??→ Tubulin+coiled_coil+HTH?+??→ TM+TM+TM+vWA(canonical)+TM+TM+TM+polar_tail→ Fibronectin-like_repeats+??→ ZnR+ZnR+DO-GTPase2.2+ZnR+ZnR→ GAP1-N2+GAP1-M+GAP1-C+polar_tail+TM+beta-rich*→	725	Bacillus sp. EB01	GCF_000613125.1	firmicutes
WP_044181099.1	GAP1-N2+GAP1-M+GAP1-C	ZnR+ZnR+DO-GTPase2.2→ GAP1-N2+GAP1-M+GAP1-C*→??+TM+??+beta-rich??→ GYF+GYF+TM+TM+TM→	815	Pirellula staleyi	-	planctomycetes
WP_044486087.1	GAP1-N2+GAP1-M+GAP1-C	TM+DUF4190+TM→ TM+DUF4190+TM→ polar_leader+TM+TM+TM→ TM+vWA(canonical)+Fibronectin-like_repeats+TM+beta-rich+lc_tail→ Tubulin+alpha_helical+??+??→ GAP_unknown1+TM+TM+GAP_unknown2→ HRDC-like?+Fibronectin-like_repeats→ GAP1-N2+GAP1-M+GAP1-C*→ beta-rich+vWA(canonical)→??+DO-GTPase2.2→ TM+DUF4190+TM+PknH_C→ TM+DUF4190+TM+PknH_C→ TM+DUF4190+TM+PknH_C→ TM+DUF4190+TM+PknH_C→ TM+DUF4190+TM+PknH_C→ TM+DUF4190+TM+PknH_C→	829	Mycobacterium colombiense	GCA_000661085.1	actinobacteria
WP_044514372.1	GAP1-N2+GAP1-M+GAP1-C	HRDC-like?+Fibronectin-like_repeats→ GAP1-N2+GAP1-M+GAP1-C*→??+DO-GTPase2.2→	819	Mycolicibacterium septicum	GCF_000455325.1	actinobacteria
WP_044647174.1	GAP1-N2+GAP1-M+GAP1-C+polar_tail+TM+beta-rich	GAP1-N2+GAP1-M+GAP1-C+polar_tail+TM+beta-rich*→	1028	Paenibacillus terrae	GCA_000943545.1	firmicutes
WP_044785686.1	GAP1-N2+GAP1-M+GAP1-C+polar_tail+TM+beta-rich	TM+vWA(canonical)+YtKA-like+TM+??→ Tubulin+coiled_coil+??→ Tubulin+coiled_coil+HTH?+??→ TM+TM+TM+vWA(canonical)+TM+TM+TM+polar_tail→ Fibronectin-like_repeats+??→ ZnR+ZnR+DO-GTPase2.2+ZnR+ZnR→ GAP1-N2+GAP1-M+GAP1-C+polar_tail+TM+beta-rich*→	1009	Paenibacillus polymyxa	GCA_000943535.1	firmicutes
WP_045317275.1	GAP1-N2+GAP1-M+SLATT+SLATT	vWA(canonical)+beta-rich+low_complexity→ Tubulin+alpha_helical+??+??→??+TM+TM+TM+TM+??→ Pkinase+TM→ ZnR+ZnR+DO-GTPase2.2→ GAP1-N2+GAP1-M+SLATT+SLATT*→	650	Lechevalieria aerocolonigenes	GCA_000955955.1	actinobacteria
WP_045745420.1	GAP1-N2+GAP1-M+GAP1-C	TPR_containing_alpha_helical+Fibronectin-like_repeats→ ZnR+ZnR+DO-GTPase2.2→ GAP1-N2+GAP1-M+GAP1-C*→	796	Actinoplanes rectilineatus	GCF_000962825.1	actinobacteria
WP_046202033.1	GAP1-N2+GAP1-M+GAP1-C	polar_leader+TM+TM+TM→ TM+vWA(canonical)+Fibronectin-like_repeats+TM+beta-rich+lc_tail→?→?→ GAP_unknown1+TM+TM+GAP_unknown2→ HRDC-like?+Fibronectin-like_repeats→ GAP1-N2+GAP1-M+GAP1-C*→ beta-rich+vWA(canonical)→??+DO-GTPase2.2→	626	Corynebacterium kroppenstedtii	GCA_002871755.1	actinobacteria
WP_046205281.1	GAP1-N2+GAP1-M+GAP1-C	polar_leader+TM+TM+TM→ TM+vWA(canonical)+Fibronectin-like_repeats+TM+beta-rich+lc_tail→ Tubulin+alpha_helical+??+??→ GAP_unknown1+TM+TM+GAP_unknown2→ HRDC-like?+Fibronectin-like_repeats→ GAP1-N2+GAP1-M+GAP1-C*→ beta-rich+vWA(canonical)→??+DO-GTPase2.2→	665	Corynebacterium kroppenstedtii	GCF_000971855.1	actinobacteria
WP_046213785.1	GAP1-N2+GAP1-M+GAP1-C+polar_tail+TM+beta-rich	TM+vWA(canonical)+YtKA-like+TM+??→ Tubulin+coiled_coil+??→ Tubulin+coiled_coil+HTH?+??→ TM+TM+TM+vWA(canonical)+TM+TM+TM+polar_tail→ Fibronectin-like_repeats+??→ ZnR+ZnR+DO-GTPase2.2+ZnR+ZnR→ GAP1-N2+GAP1-M+GAP1-C+polar_tail+TM+beta-rich*→	1125	Paenibacillus wulumuqiensis	GCF_000971965.1	firmicutes
WP_046225918.1	GAP1-N2+GAP1-M+GAP1-C+polar_tail+TM+beta-rich	TM+vWA(canonical)+YtKA-like+TM+??→ Tubulin+coiled_coil+??→ Tubulin+coiled_coil+HTH?+??→ TM+TM+TM+vWA(canonical)+TM+TM+TM+polar_tail→ Fibronectin-like_repeats+??→ ZnR+ZnR+DO-GTPase2.2+ZnR+ZnR→ GAP1-N2+GAP1-M+GAP1-C+polar_tail+TM+beta-rich*→	1143	Paenibacillus dauci	GCF_000971985.1	firmicutes
WP_046260777.1	GAP1-N2+GAP1-M+GAP1-C	TPR_containing_alpha_helical+Fibronectin-like_repeats→ ZnR+ZnR+DO-GTPase2.2→ GAP1-N2+GAP1-M+GAP1-C*→ TM+TM+TM+TM+ZnR+ZnR+DO-GTPase2.2+TM→ vWA(canonical)→ PP2C→ Pkinase+ZnR+beta-rich→	918	Streptomyces sp. WM6386	GCA_000972385.1	actinobacteria
WP_046496261.1	GAP1-N2+GAP1-M	-	845	Streptomyces odonnellii	-	actinobacteria
WP_046704594.1	GAP1-N2+GAP1-M+GAP1-C	GAP1-N2+GAP1-M+GAP1-C*→ vWA(canonical)→ PP2C→ Pkinase+ZnR+beta-rich→	811	Streptomyces europaeiscabiei	GCF_000988945.1	actinobacteria
WP_047040408.1	GAP1-N2+GAP1-M+GAP1-C	HRDC-like?+Fibronectin-like_repeats→ GAP1-N2+GAP1-M+GAP1-C*→??+DO-GTPase2.2→	819	Mycolicibacterium	GCA_001012795.1	actinobacteria
WP_047070091.1	GAP1-N2+GAP1-M+GAP1-C+polar_tail+TM+beta-rich	TM+vWA(canonical)+YtKA-like+TM+??→ Tubulin+coiled_coil+??→ Tubulin+coiled_coil+HTH?+??→ TM+TM+TM+vWA(canonical)+TM+TM+TM+polar_tail→ Fibronectin-like_repeats+??→ ZnR+ZnR+DO-GTPase2.2+ZnR+ZnR→ GAP1-N2+GAP1-M+GAP1-C+polar_tail+TM+beta-rich*→	832	Brevibacillus formosus	GCA_006540185.1	firmicutes
WP_047141722.1	GAP1-N2+GAP1-M+GAP1-C	GAP1-N2+GAP1-M+GAP1-C*→	789	Streptomyces sp. KE1	GCA_001014595.1	actinobacteria
WP_047179414.1	GAP1-N2+GAP1-M+GAP1-C	GAP1-N2+GAP1-M+GAP1-C*→	833	Streptomyces sp. MNU77	-	actinobacteria
WP_047464182.1	GAP1-N2+GAP1-M+GAP1-C	GAP1-N2+GAP1-M+GAP1-C*→	789	Streptomyces sp. M10	GCF_000800535.1	actinobacteria
WP_047840765.1	GAP1-N2+GAP1-M+GAP1-C+polar_tail+TM+beta-rich	TM+vWA(canonical)+YtKA-like+TM+??→ Tubulin+coiled_coil+??→ Tubulin+coiled_coil+HTH?+??→ TM+TM+TM+vWA(canonical)+TM+TM+TM+polar_tail→ Fibronectin-like_repeats+??→ ZnR+ZnR+DO-GTPase2.2+ZnR+ZnR→ GAP1-N2+GAP1-M+GAP1-C+polar_tail+TM+beta-rich*→	1069	Paenibacillus sp. VT-400	GCA_001029205.1	firmicutes
WP_047871140.1	GAP1-N2+GAP1-M+GAP1-C	TPR_containing_alpha_helical+Fibronectin-like_repeats→ ZnR+ZnR+DO-GTPase2.2→ GAP1-N2+GAP1-M+GAP1-C*→	883	Nocardiopsis sp. RV163	GCF_001029525.1	actinobacteria
WP_047943646.1	GAP1-N2+GAP1-M+GAP1-C+polar_tail+TM+beta-rich	GAP1-N2+GAP1-M+GAP1-C+polar_tail+TM+beta-rich*→	746	Bacillus circulans	GCA_001029855.1	firmicutes
WP_048035300.1	GAP1-N2+GAP1-M+GAP1-C+polar_tail+TM+beta-rich	TM+vWA(canonical)+YtKA-like+TM+??→ Tubulin+coiled_coil+??→ Tubulin+coiled_coil+HTH?+??→ TM+TM+TM+vWA(canonical)+TM+TM+TM+polar_tail→ Fibronectin-like_repeats+??→ ZnR+ZnR+DO-GTPase2.2+ZnR+ZnR→ GAP1-N2+GAP1-M+GAP1-C+polar_tail+TM+beta-rich*→	832	Brevibacillus brevis	GCA_001039275.2	firmicutes
WP_048037372.1	GAP1-N2+GAP1-M+GAP1-C	TM+TM+TM+TM+ZnR+ZnR+DO-GTPase2.2→ GrpE→ HSP70→ HSP70+vWA(canonical)→ DnaJ+DnaJ+ZnR+ZnR+coiled_coil+Fibronectin-like_repeats→ ZnR+ZnR+DO-GTPase2.2→ GAP1-N2+GAP1-M+GAP1-C*→	882	Methanosarcina mazei	GCA_000978965.1	euryarchaeota
WP_048041778.1	GAP1-N2+GAP1-M+GAP1-C	TM+TM+TM+TM+ZnR+ZnR+DO-GTPase2.2→ GrpE→ HSP70→ HSP70+vWA(canonical)→ DnaJ+DnaJ+ZnR+ZnR+coiled_coil+Fibronectin-like_repeats→ ZnR+ZnR+DO-GTPase2.2→ GAP1-N2+GAP1-M+GAP1-C*→	882	Methanosarcina mazei	GCA_000979335.1	euryarchaeota
WP_048398625.1	GAP1-N2	-	569	Candidatus Achromatium palustre	-	gammaproteobacteria
WP_048462046.1	GAP1-N2+GAP1-M+GAP1-C	GAP1-N2+GAP1-M+GAP1-C*→	613	Methylobacterium aquaticum	GCA_001043915.1	alphaproteobacteria
WP_048760431.1	GAP1-N2+GAP1-M+GAP1-C	polar_leader+TM+TM+TM→ TM+vWA(canonical)+Fibronectin-like_repeats+TM+beta-rich+lc_tail→ Tubulin+alpha_helical+??+??→ GAP_unknown1+TM+TM+GAP_unknown2→ HRDC-like?+Fibronectin-like_repeats→ GAP1-N2+GAP1-M+GAP1-C*→ beta-rich+vWA(canonical)→??+DO-GTPase2.2→	862	Corynebacterium vitaeruminis	GCF_001054325.1	actinobacteria
WP_049157802.1	GAP1-N2+GAP1-M+GAP1-C	polar_leader+TM+TM+TM→ TM+vWA(canonical)+Fibronectin-like_repeats+TM+beta-rich+lc_tail→ Tubulin+alpha_helical+??+??→ GAP_unknown1+TM+TM+GAP_unknown2→ HRDC-like?+Fibronectin-like_repeats→ GAP1-N2+GAP1-M+GAP1-C*→ beta-rich+vWA(canonical)→??+DO-GTPase2.2→ polar_leader+TM+TM+TM→	565	Corynebacterium aurimucosum	GCF_001059615.1	actinobacteria
WP_049714333.1	GAP1-N2+GAP1-M+GAP1-C	GAP1-N2+GAP1-M+GAP1-C*→	921	Streptomyces caatingaensis	GCF_001187435.1	actinobacteria
WP_049740646.1	GAP1-N2+GAP1-M+GAP1-C+polar_tail+TM+beta-rich	TM+vWA(canonical)+YtKA-like+TM+??→ Tubulin+coiled_coil+??→ Tubulin+coiled_coil+HTH?+??→ TM+TM+TM+vWA(canonical)+TM+TM+TM+polar_tail→ Fibronectin-like_repeats+??→ ZnR+ZnR+DO-GTPase2.2+ZnR+ZnR→ GAP1-N2+GAP1-M+GAP1-C+polar_tail+TM+beta-rich*→	840	Brevibacillus reuszeri	GCF_006540225.1	firmicutes

acc	architecture	operon	len	species	gca	taxend
WP_049744098.1	GAP1-N2+GAP1-M+GAP1-C	HRDC-like?+Fibronectin-like_repeats→ GAP1-N2+GAP1-M+GAP1-C*→??+DO-GTPase2.2→	817	Mycolicibacterium goodii	GCA_001187505.1	actinobacteria
WP_049895018.1	GAP1-N2+GAP1-M+GAP1-C+polar_tail	Tubulin+coiled_coil+??→ Tubulin+coiled_coil+HTH?+??→ TM+TM+TM+vWA+TM+TM+TM+polar_tail→ Fibronectin-like_repeats+??→ ZnR+ZnR+DO-GTPase2.2→ GAP1-N2+GAP1-M+GAP1-C+polar_tail*→	1072	Paenibacillus antibioticophila	GCF_000455265.1	firmicutes
WP_049978494.1	GAP1-N2+GAP1-M+GAP1-C	GAP1-N2+GAP1-M+GAP1-C*→	789	Streptomyces wadayamensis	GCA_000698945.1	actinobacteria
WP_050024924.1	GAP1-N2+GAP1-M+GAP1-C	GrpE→ HSP70→||←?||HSP70+vWA(canonical)→ DnaJ+DnaJ+ZnR+ZnR+coiled_coil+Fibronectin-like_repeats→ ZnR+ZnR+DO-GTPase2.2→ GAP1-N2+GAP1-M+GAP1-C*→?→ TM+TM+ZnR+ZnR+DO-GTPase2.2+TM+PDZ+PDZ→	785	Verrucomicrobium sp. BvORR034	GCF_000739635.1	verrucomicrobia
WP_050054084.1	GAP1-N2+GAP1-M+GAP1-C	GrpE→ HSP70→ TPR_containing_alpha_helical+Fibronectin-like_repeats→ ZnR+ZnR+DO-GTPase2.2→ GAP1-N2+GAP1-M+GAP1-C*→ TM+TM+TM+TM+ZnR+ZnR+DO-GTPase2.2→??→ HSP70→ vWA(canonical)→	791	Pseudarthrobacter siccitolerans	GCA_001046895.1	actinobacteria
WP_050343801.1	GAP1-N2+GAP1-M+GAP1-C	TM+vWA(canonical)+YtKA-like+IG-like+TM+??→ Tubulin+coiled_coil+??→?→?→ ZnR+ZnR+DO-GTPase2.2+ZnR+ZnR→ GAP1-N2+GAP1-M+GAP1-C*→??+Pkinase→?→ Tubulin+coiled_coil+??→ Tubulin+coiled_coil+??→	831	Olsenella sp. oral taxon 807	GCA_001189515.2	actinobacteria
WP_050357186.1	GAP1-N2+GAP1-M+GAP1-C	GAP1-N2+GAP1-M+GAP1-C*→	827	Streptomyces europaeiscabiei	GCA_001189025.1	actinobacteria
WP_050588178.1	GAP1-N2+GAP1-M+GAP1-C	GAP1-N2+GAP1-M+GAP1-C*→	607	Streptomyces sp. HCCB10043	GCF_000498935.1	actinobacteria
WP_050979382.1	GAP1-N2+GAP1-M+GAP1-C+polar_tail	TM+TM+TM+vWA+TM+TM+TM+polar_tail→ Fibronectin-like_repeats+??→ ZnR+ZnR+DO-GTPase2.2→ GAP1-N2+GAP1-M+GAP1-C+polar_tail*→	1022	Paenibacillus dendritiformis	GCF_000245555.1	firmicutes
WP_051154095.1	GAP1-N2+GAP1-M+GAP1-C+polar_tail+TM+beta-rich	TM+vWA(canonical)+YtKA-like+TM+??→ Tubulin+coiled_coil+??→ Tubulin+coiled_coil+HTH?+??→ TM+TM+TM+vWA(canonical)+TM+TM+TM+polar_tail→ Fibronectin-like_repeats+??→ ZnR+ZnR+DO-GTPase2.2+ZnR+ZnR→ GAP1-N2+GAP1-M+GAP1-C+polar_tail+TM+beta-rich*→	1064	Paenibacillus sp. PAMC 26794	GCF_000316035.1	firmicutes
WP_051215300.1	GAP1-N2+GAP1-M+GAP1-C	TM+vWA(canonical)+Fibronectin-like_repeats+TM+beta-rich+lc_tail→||←?||Tubulin+alpha_helical+??+??→ GAP_unknown1+TM+TM+GAP_unknown2→ HRDC-like?+Fibronectin-like_repeats→ GAP1-N2+GAP1-M+GAP1-C*→ beta-rich+vWA(canonical)→??+DO-GTPase2.2→	909	Granulicoccus phenolivorans	GCF_000423085.1	actinobacteria
WP_051217355.1	GAP1-N2+GAP1-M+GAP1-C+polar_tail+TM+beta-rich	TM+vWA(canonical)+YtKA-like+TM+??→ Tubulin+coiled_coil+??→ Tubulin+coiled_coil+HTH?+??→ TM+TM+TM+vWA(canonical)+TM+TM+TM+polar_tail→?→ Fibronectin-like_repeats+??→ ZnR+ZnR+DO-GTPase2.2+ZnR+ZnR→ GAP1-N2+GAP1-M+GAP1-C+polar_tail+TM+beta-rich*→	965	Paenibacillus assamensis	GCF_000422445.1	firmicutes
WP_051235971.1	GAP1-N2+GAP1-M+GAP1-C+polar_tail+TM+beta-rich	TM+vWA(canonical)+YtKA-like+TM+??→ Tubulin+coiled_coil+??→ Tubulin+coiled_coil+HTH?+??→ TM+TM+TM+vWA(canonical)+TM+TM+TM+polar_tail→ Fibronectin-like_repeats+??→ ZnR+ZnR+DO-GTPase2.2+ZnR+ZnR→ GAP1-N2+GAP1-M+GAP1-C+polar_tail+TM+beta-rich*→	977	Paenibacillus pinihumi	GCF_000422505.1	firmicutes
WP_051289601.1	GAP1-N2+GAP1-M+GAP1-C+polar_tail+TM+beta-rich	TM+vWA(canonical)+YtKA-like+TM+??→ Tubulin+coiled_coil+??→ Tubulin+coiled_coil+HTH?+??→ TM+TM+TM+vWA(canonical)+TM+TM+TM+polar_tail→ Fibronectin-like_repeats+??→ ZnR+ZnR+DO-GTPase2.2+ZnR+ZnR→ GAP1-N2+GAP1-M+GAP1-C+polar_tail+TM+beta-rich*→	1000	Paenibacillus panacisoli	GCF_000426545.1	firmicutes
WP_051387685.1	GAP1-N2+GAP1-M+GAP1-C	GAP1-N2+GAP1-M+GAP1-C*→	777	Saccharopolyspora rectivirgula	GCF_000497205.1	actinobacteria
WP_051446081.1	GAP1-N2+GAP1-M+GAP1-C	TM+vWA(canonical)+Fibronectin-like_repeats+TM+beta-rich+lc_tail→ Tubulin+alpha_helical+??+??→ GAP_unknown1+TM+TM+GAP_unknown2→ HRDC-like?+Fibronectin-like_repeats→ GAP1-N2+GAP1-M+GAP1-C*→ beta-rich+vWA(canonical)→??+DO-GTPase2.2→	921	Rhodococcus rhodochrous	GCA_000517665.1	actinobacteria
WP_051447278.1	GAP1-N2+GAP1-M+GAP1-C+polar_tail	Tubulin+coiled_coil+??→ Tubulin+coiled_coil+HTH?+??→ TM+TM+TM+vWA+TM+TM+TM+polar_tail→ Fibronectin-like_repeats+??→ ZnR+ZnR+DO-GTPase2.2→ GAP1-N2+GAP1-M+GAP1-C+polar_tail*→	1068	Paenibacillus sp. FSL H7-689	GCF_000517905.1	firmicutes
WP_051449334.1	GAP1-N2+GAP1-M+GAP1-C+polar_tail+TM+beta-rich	TM+vWA(canonical)+YtKA-like+TM+??→ Tubulin+coiled_coil+??→ Tubulin+coiled_coil+HTH?+??→ TM+TM+TM+vWA(canonical)+TM+TM+TM+polar_tail→ Fibronectin-like_repeats+??→ ZnR+ZnR+DO-GTPase2.2+ZnR+ZnR→ GAP1-N2+GAP1-M+GAP1-C+polar_tail+TM+beta-rich*→	1002	Paenibacillus	GCA_001632305.1	firmicutes
WP_051478511.1	GAP1-N2+GAP1-M+GAP1-C+polar_tail+TM+beta-rich	Tubulin+coiled_coil+??→ Tubulin+coiled_coil+HTH?+??→?→?→ TM+TM+TM+vWA(canonical)+TM+TM+TM+polar_tail→ Fibronectin-like_repeats+??→ ZnR+ZnR+DO-GTPase2.2+ZnR+ZnR→ GAP1-N2+GAP1-M+GAP1-C+polar_tail+TM+beta-rich*→	830	Paenibacillus sp. FSL R7-277	GCF_000517965.1	firmicutes
WP_051480884.1	GAP1-M+GAP1-C	TM+DUF4190+TM→ TM+DUF4190+TM→ polar_leader+TM+TM+TM→ PSE→ PSE→ GAP_unknown1+TM+TM+GAP_unknown2→ GAP_unknown1+TM+TM+GAP_unknown2→ PSE→ GAP1-M+GAP1-C*→ GAP1-N2+GAP1-M+GAP1-C→?→ beta-rich+vWA(canonical)→	150	Mycobacterium kansasii	GCA_000524095.1	actinobacteria
WP_051480886.1	GAP1-M+GAP1-C	TM+DUF4190+TM→ TM+DUF4190+TM→ polar_leader+TM+TM+TM→ PSE→ PSE→ GAP_unknown1+TM+TM+GAP_unknown2→ GAP_unknown1+TM+TM+GAP_unknown2→ PSE→ GAP1-N2+GAP1-M+GAP1-C→ GAP1-M+GAP1-C*→?→ beta-rich+vWA(canonical)→	614	Mycobacterium kansasii	GCF_000524095.1	actinobacteria
WP_051491151.1	GAP1-N2+GAP1-M+GAP1-C+polar_tail+TM+beta-rich	TM+vWA(canonical)+YtKA-like+TM+??→ Tubulin+coiled_coil+??→ Tubulin+coiled_coil+HTH?+??→ TM+TM+TM+vWA(canonical)+TM+TM+TM+polar_tail→ Fibronectin-like_repeats+??→ ZnR+ZnR+DO-GTPase2.2+ZnR+ZnR→ GAP1-N2+GAP1-M+GAP1-C+polar_tail+TM+beta-rich*→	998	Paenibacillus	GCA_000517925.1	firmicutes
WP_051494057.1	GAP1-N2+GAP1-M+GAP1-C+polar_tail+TM+beta-rich	TM+vWA(canonical)+YtKA-like+TM+??→ Tubulin+coiled_coil+??→ Tubulin+coiled_coil+HTH?+??→?→ TM+TM+TM+vWA(canonical)+TM+TM+TM+polar_tail→ Fibronectin-like_repeats+??→ ZnR+ZnR+DO-GTPase2.2+ZnR+ZnR→ GAP1-N2+GAP1-M+GAP1-C+polar_tail+TM+beta-rich*→	789	Paenibacillus sp. FSL R7-269	GCF_000517865.1	firmicutes
WP_051541673.1	GAP1-N2+GAP1-M	TM+TM+TM+TM+ZnR+ZnR+DO-GTPase2.2→ TM+vWA(canonical)+YtKA-like+TM+??→ Tubulin+coiled_coil+??→ Tubulin+coiled_coil+HTH?+??→ TM+TM+TM+vWA(canonical)+TM+TM+TM+polar_tail→ Fibronectin-like_repeats+??→ ZnR+ZnR+DO-GTPase2.2+ZnR+ZnR→ GAP1-N2+GAP1-M*→	389	Clostridium lundense	GCF_000619945.1	firmicutes
WP_051620670.1	GAP1-N2+GAP1-M+GAP1-C+polar_tail+TM+Cadherin-like	TM+vWA(canonical)+YtKA-like+TM+??→ Tubulin+coiled_coil+??→ Tubulin+coiled_coil+HTH?+??→ TM+TM+TM+vWA(canonical)+TM+TM+TM+polar_tail→ Fibronectin-like_repeats+??→ ZnR+ZnR+DO-GTPase2.2+ZnR+ZnR→ GAP1-N2+GAP1-M+GAP1-C+polar_tail+TM+Cadherin-like*→	972	Paenibacillus sp. UNC451MF	GCF_000686845.1	firmicutes
WP_051707761.1	GAP1-N2+GAP1-M+GAP1-C	GAP1-N2+GAP1-M+GAP1-C*→	777	Saccharopolyspora rectivirgula	GCF_000710755.1	actinobacteria
WP_051716628.1	GAP1-N2+GAP1-M+GAP1-C	ZnR+ZnR+DO-GTPase2.2→ GAP1-N2+GAP1-M+GAP1-C*→ TM+TM+TM+TM+ZnR+ZnR+DO-GTPase2.2+TM→?→ vWA(canonical)→ PP2C→ Pkinase+ZnR+beta-rich→	828	Streptomyces sp. NRRL F-5727	GCF_000719555.1	actinobacteria
WP_051730664.1	GAP1-N2+GAP1-M+GAP1-C	polar_leader+TM+TM+TM→ TM+vWA(canonical)+Fibronectin-like_repeats+TM+beta-rich+lc_tail→ Tubulin+alpha_helical+??+??→ GAP_unknown1+TM+TM+GAP_unknown2→ HRDC-like?+Fibronectin-like_repeats→ GAP1-N2+GAP1-M+GAP1-C*→ beta-rich+vWA(canonical)→??+DO-GTPase2.2→	968	Rhodococcus	GCA_000949305.1	actinobacteria
WP_051735597.1	GAP1-N2+GAP1-M+GAP1-C	PP2C→ Pkinase→?→?→?→ HSP70_domain_2+low_complexity+domain_betaX→ ZnR+ZnR+DO-GTPase2.2→ GAP1-N2+GAP1-M+GAP1-C*→ PSE→||←TPR_region+CASPASE	815	Streptomyces sp. NRRL B-3229	GCF_000719505.1	actinobacteria
WP_051735910.1	GAP1-N2+GAP1-M+GAP1-C	GrpE→ HSP70→ TPR_containing_alpha_helical+Fibronectin-like_repeats→ ZnR+ZnR+DO-GTPase2.2→ GAP1-N2+GAP1-M+GAP1-C*→ TM+TM+TM+TM+ZnR+ZnR+DO-GTPase2.2→?→ HSP70→ vWA(canonical)→?→||←HSP70←unknown+CASPASE	827	Amycolatopsis rifamycinica	GCA_000695625.1	actinobacteria
WP_051742302.1	GAP1-N2+GAP1-M+GAP1-C	GAP1-N2+GAP1-M+GAP1-C*→	837	Streptomyces xylophagus	GCF_000716435.1	actinobacteria
WP_051787773.1	GAP1-N2+GAP1-M+GAP1-C	TPR_containing_alpha_helical+Fibronectin-like_repeats→ ZnR+ZnR+DO-GTPase2.2→ GAP1-N2+GAP1-M+GAP1-C*→ TM+TM+TM+TM+ZnR+ZnR+DO-GTPase2.2+TM→?→ vWA(canonical)→ PP2C→ Pkinase+ZnR+beta-rich→	823	Microtetraspora glauca	-	actinobacteria
WP_051807387.1	GAP1-N2+GAP1-M+GAP1-C	TPR_region+CASPASE||GrpE→ HSP70→ TPR_containing_alpha_helical+Fibronectin-like_repeats→ ZnR+ZnR+DO-GTPase2.2→ GAP1-N2+GAP1-M+GAP1-C*→ TM+TM+TM+TM+ZnR+ZnR+DO-GTPase2.2→	792	Actinoplanes subtropicus	GCF_000721705.1	actinobacteria
WP_051985757.1	GAP1-N2+GAP1-M+GAP1-C+polar_tail+TM+beta-rich	TM+vWA(canonical)+YtKA-like+TM+??→ Tubulin+coiled_coil+??→ Tubulin+coiled_coil+HTH?+??→ TM+TM+TM+vWA(canonical)+TM+TM+TM+polar_tail→ Fibronectin-like_repeats+??→ ZnR+ZnR+DO-GTPase2.2+ZnR+ZnR→ GAP1-N2+GAP1-M+GAP1-C+polar_tail+TM+beta-rich*→	1144	Paenibacillus macerans	GCA_000746875.1	firmicutes
WP_052020486.1	GAP1-N2+GAP1-M	Tubulin+coiled_coil+??→ Tubulin+coiled_coil+HTH?+??→ TM+TM+TM+vWA(canonical)+TM+TM+TM+polar_tail→ Fibronectin-like_repeats+??→ PSE→ GAP1-N2+GAP1-M*→	250	Paenibacillus pini	GCA_000576305.1	firmicutes
WP_052038875.1	GAP1-N2+GAP1-M+GAP1-C	GrpE→ HSP70→ TPR_containing_alpha_helical+Fibronectin-like_repeats→ ZnR+ZnR+DO-GTPase2.2→ GAP1-N2+GAP1-M+GAP1-C*→ TM+TM+TM+TM+ZnR+ZnR+DO-GTPase2.2→?→?→?→ HSP70→ vWA(canonical)→	687	Actinomyces sp. S4-C9	GCF_000758775.1	actinobacteria
WP_052047688.1	GAP1-N2+GAP1-M+GAP1-C	polar_leader+TM+TM+TM→ TM+vWA(canonical)+Fibronectin-like_repeats+TM+beta-rich+lc_tail→ Tubulin+alpha_helical+??+??→ GAP_unknown1+TM+TM+GAP_unknown2→ HRDC-like?+Fibronectin-like_repeats→ GAP1-N2+GAP1-M+GAP1-C*→ beta-rich+vWA(canonical)→??+DO-GTPase2.2→	904	Rhodococcus	GCA_002259145.1	actinobacteria
WP_052054083.1	GAP1-N2+GAP1-M+GAP1-C	polar_leader+TM+TM+TM→ TM+vWA(canonical)+Fibronectin-like_repeats+TM+beta-rich+lc_tail→ Tubulin+alpha_helical+??+??→ GAP_unknown1+TM+TM+GAP_unknown2→||←?||HRDC-like?+Fibronectin-like_repeats→ GAP1-N2+GAP1-M+GAP1-C*→ beta-rich+vWA(canonical)→??+DO-GTPase2.2→ polar_leader+TM+TM+TM→	850	Corynebacterium freneyi	GCF_000758965.1	actinobacteria
WP_052063153.1	GAP1-N2+GAP1-M+GAP1-C	polar_leader+TM+TM+TM→ TM+vWA(canonical)+Fibronectin-like_repeats+TM+beta-rich+lc_tail→ Tubulin+alpha_helical+??+??→ GAP_unknown1+TM+TM+GAP_unknown2→ HRDC-like?+Fibronectin-like_repeats→ GAP1-N2+GAP1-M+GAP1-C*→ beta-rich+vWA(canonical)→??+DO-GTPase2.2→	904	Rhodococcus fascians	GCF_000760815.1	actinobacteria
WP_052087819.1	GAP1-N2+GAP1-M+GAP1-C+polar_tail+TM+beta-rich	TM+vWA(canonical)+YtKA-like+TM+??→ Tubulin+coiled_coil+??→ Tubulin+coiled_coil+HTH?+??→ TM+TM+TM+vWA(canonical)+TM+TM+TM+polar_tail→ Fibronectin-like_repeats+??→ ZnR+ZnR+DO-GTPase2.2+ZnR+ZnR→ GAP1-N2+GAP1-M+GAP1-C+polar_tail+TM+beta-rich*→	977	Paenibacillus wynnii	GCF_000757885.1	firmicutes
WP_052091824.1	GAP1-N2+GAP1-M+GAP1-C+polar_tail+TM+beta-rich	TM+vWA(canonical)+YtKA-like+TM+??→ Tubulin+coiled_coil+??→ Tubulin+coiled_coil+HTH?+??→ TM+TM+TM+vWA(canonical)+TM+TM+TM+polar_tail→ Fibronectin-like_repeats+??→ ZnR+ZnR+DO-GTPase2.2+ZnR+ZnR→ GAP1-N2+GAP1-M+GAP1-C+polar_tail+TM+beta-rich*→	1048	Paenibacillus sp. FSL H7-0357	GCF_000758525.1	firmicutes
WP_052096792.1	GAP1-N2+GAP1-M+GAP1-C+polar_tail+TM+beta-rich	TM+vWA(canonical)+YtKA-like+TM+??→ Tubulin+coiled_coil+??→ Tubulin+coiled_coil+HTH?+??→ TM+TM+TM+vWA(canonical)+TM+TM+TM+polar_tail→ Fibronectin-like_repeats+??→ ZnR+ZnR+DO-GTPase2.2+ZnR+ZnR→ GAP1-N2+GAP1-M+GAP1-C+polar_tail+TM+beta-rich*→	998	Paenibacillus odorifer	GCF_000758725.1	firmicutes
WP_052098204.1	GAP1-N2+GAP1-M+GAP1-C+polar_tail+TM+beta-rich	TM+vWA(canonical)+YtKA-like+TM+??→ Tubulin+coiled_coil+??→ Tubulin+coiled_coil+HTH?+??→ TM+TM+TM+vWA(canonical)+TM+TM+TM+polar_tail→ Fibronectin-like_repeats+??→ ZnR+ZnR+DO-GTPase2.2+ZnR+ZnR→ GAP1-N2+GAP1-M+GAP1-C+polar_tail+TM+beta-rich*→	1027	Paenibacillus stellifer	GCF_000758685.1	firmicutes
WP_052146961.1	GAP1-N2+GAP1-M+GAP1-C+polar_tail+TM+beta-rich	TM+vWA(canonical)+YtKA-like+TM+??→ Tubulin+coiled_coil+??→ PSE→ PSE→ Fibronectin-like_repeats+??→ ZnR+ZnR+DO-GTPase2.2+ZnR+ZnR→ GAP1-N2+GAP1-M+GAP1-C+polar_tail+TM+beta-rich*→	1061	Paenibacillus sp. P1XP2	GCA_000787385.1	firmicutes
WP_052205935.1	GAP1-N2+GAP1-M+GAP1-C	polar_leader+TM+TM+TM→ TM+vWA(canonical)+Fibronectin-like_repeats+TM+beta-rich+lc_tail→ Tubulin+alpha_helical+??+??→ GAP_unknown1+TM+TM+GAP_unknown2→ HRDC-like?+Fibronectin-like_repeats→ GAP1-N2+GAP1-M+GAP1-C*→?→??+DO-GTPase2.2→?→||←?←?←HSP70	326	Corynebacterium riegelii	GCA_001263755.1	actinobacteria
WP_052319616.1	GAP1-N2+GAP1-M+GAP1-C	polar_leader+TM+TM+TM→ TM+vWA(canonical)+Fibronectin-like_repeats+TM+beta-rich+lc_tail→ Tubulin+alpha_helical+??+??→ GAP_unknown1+TM+TM+GAP_unknown2→ HRDC-like?+Fibronectin-like_repeats→ GAP1-N2+GAP1-M+GAP1-C*→ beta-rich+vWA(canonical)→??+DO-GTPase2.2→ polar_leader+TM+TM+TM→	559	Corynebacterium minutissimum	GCA_900478045.1	actinobacteria
WP_052339668.1	GAP1-N2+GAP1-M+GAP1-C+polar_tail+TM+beta-rich	TM+vWA(canonical)+YtKA-like+TM+??→ Tubulin+coiled_coil+??→ Tubulin+coiled_coil+HTH?+??→ TM+TM+TM+vWA(canonical)+TM+TM+TM+polar_tail→ Fibronectin-like_repeats+??→ ZnR+ZnR+DO-GTPase2.2+ZnR+ZnR→ GAP1-N2+GAP1-M+GAP1-C+polar_tail+TM+beta-rich*→	911	Gorillibacterium massiliense	GCF_000455485.1	firmicutes
WP_052352962.1	GAP1-N2+GAP1-M+GAP1-C+polar_tail+TM+beta-rich	TM+vWA(canonical)+YtKA-like+TM+??→ Tubulin+coiled_coil+??→ Tubulin+coiled_coil+HTH?+??→ TM+TM+TM+vWA(canonical)+TM+TM+TM+polar_tail→ Fibronectin-like_repeats+??→ ZnR+ZnR+DO-GTPase2.2+ZnR+ZnR→ GAP1-N2+GAP1-M+GAP1-C+polar_tail+TM+beta-rich*→	933	Bacillus dielmoensis	GCF_000612665.1	firmicutes
WP_052353844.1	GAP1-N2+GAP1-M+GAP1-C+polar_tail+TM+beta-rich	TM+vWA(canonical)+YtKA-like+TM+??→ Tubulin+coiled_coil+??→ Tubulin+coiled_coil+HTH?+??→ Fibronectin-like_repeats+??→ ZnR+ZnR+DO-GTPase2.2+ZnR+ZnR→ GAP1-N2+GAP1-M+GAP1-C+polar_tail+TM+beta-rich*→	728	Bacillus	GCF_000612625.1	firmicutes
WP_052380394.1	GAP1-N2+GAP1-M+GAP1-C+polar_tail+TM+beta-rich	TM+vWA(canonical)+YtKA-like+TM+??→ Tubulin+coiled_coil+??→ Tubulin+coiled_coil+HTH?+??→||←?||TM+TM+TM+vWA(canonical)+TM+TM+TM+polar_tail→ Fibronectin-like_repeats+??→ ZnR+ZnR+DO-GTPase2.2+ZnR+ZnR→ GAP1-N2+GAP1-M+GAP1-C+polar_tail+TM+beta-rich*→	1088	Paenibacillus camerounensis	GCF_000723885.1	firmicutes
WP_052404160.1	GAP1-N2+GAP1-M+GAP1-C+polar_tail+TM+beta-rich	TM+vWA(canonical)+YtKA-like+TM+??→ Tubulin+coiled_coil+??→ Tubulin+coiled_coil+HTH?+??→ TM+TM+TM+vWA(canonical)+TM+TM+TM+polar_tail→ Fibronectin-like_repeats+??→ ZnR+ZnR+DO-GTPase2.2+ZnR+ZnR→ GAP1-N2+GAP1-M+GAP1-C+polar_tail+TM+beta-rich*→	986	Paenibacillus sp. FSL H7-0737	GCF_000758545.1	firmicutes
WP_052410072.1	GAP1-N2+GAP1-M+GAP1-C+polar_tail+TM+beta-rich	TM+vWA(canonical)+YtKA-like+TM+??→ Tubulin+coiled_coil+??→ Tubulin+coiled_coil+HTH?+??→ TM+TM+TM+vWA(canonical)+TM+TM+TM+polar_tail→ Fibronectin-like_repeats+??→ ZnR+ZnR+DO-GTPase2.2+ZnR+ZnR→ GAP1-N2+GAP1-M+GAP1-C+polar_tail+TM+beta-rich*→	1028	Paenibacillus durus	GCF_000756615.1	firmicutes

acc	architecture	operon	len	species	gca	taxend
WP_052414459.1	GAP1-N2+GAP1-M+GAP1-C+polar_tail+TM+beta-rich	TM+vWA(canonical)+YtKA-like+TM+??→ Tubulin+coiled_coil+??→ Tubulin+coiled_coil+HTH?+??→ TM+TM+TM+vWA(canonical)+TM+TM+TM+polar_tail→ Fibronectin-like_repeats+??→ ZnR+ZnR+DO-GTPase2.2+ZnR+ZnR→ GAP1-N2+GAP1-M+GAP1-C+polar_tail+TM+beta-rich*→	994	Paenibacillus sp. FSL R5-0345	GCF_000758585.1	firmicutes
WP_052415693.1	GAP1-N2+GAP1-M+GAP1-C+polar_tail+TM+beta-rich	TM+vWA(canonical)+YtKA-like+TM+??→ Tubulin+coiled_coil+??→ Tubulin+coiled_coil+HTH?+??→ TM+TM+TM+vWA(canonical)+TM+TM+TM+polar_tail→ Fibronectin-like_repeats+??→ ZnR+ZnR+DO-GTPase2.2+ZnR+ZnR→ GAP1-N2+GAP1-M+GAP1-C+polar_tail+TM+beta-rich*→	1027	Paenibacillus graminis	GCF_000758705.1	firmicutes
WP_052415985.1	GAP1-N2+GAP1-M+GAP1-C+polar_tail+TM+beta-rich	TM+vWA(canonical)+YtKA-like+TM+??→ Tubulin+coiled_coil+??→ Tubulin+coiled_coil+HTH?+??→ TM+TM+TM+vWA(canonical)+TM+TM+TM+polar_tail→ Fibronectin-like_repeats+??→ ZnR+ZnR+DO-GTPase2.2+ZnR+ZnR→ GAP1-N2+GAP1-M+GAP1-C+polar_tail+TM+beta-rich*→	1075	Paenibacillus sp. FSL R5-0912	GCF_000758605.1	firmicutes
WP_052418904.1	GAP1-N2+GAP1-M+GAP1-C+polar_tail+TM+beta-rich	TM+vWA(canonical)+YtKA-like+TM+??→ Tubulin+coiled_coil+??→ Tubulin+coiled_coil+HTH?+??→ TM+TM+TM+vWA(canonical)+TM+TM+TM+polar_tail→ Fibronectin-like_repeats+??→ ZnR+ZnR+DO-GTPase2.2+ZnR+ZnR→ GAP1-N2+GAP1-M+GAP1-C+polar_tail+TM+beta-rich*→	1087	Paenibacillus sp. FSL R7-0331	GCF_000758645.1	firmicutes
WP_052421677.1	GAP1-N2+GAP1-M+GAP1-C+polar_tail+TM+beta-rich	TM+vWA(canonical)+YtKA-like+TM+??→ Tubulin+coiled_coil+??→ Tubulin+coiled_coil+HTH?+??→ TM+TM+TM+vWA(canonical)+TM+TM+TM+polar_tail→ Fibronectin-like_repeats+??→ ZnR+ZnR+DO-GTPase2.2+ZnR+ZnR→ GAP1-N2+GAP1-M+GAP1-C+polar_tail+TM+beta-rich*→	1070	Paenibacillus sp. FSL P4-0081	GCF_000758565.1	firmicutes
WP_052424850.1	GAP1-N2+GAP1-M+TM+GAP1-C	DO-GTPase1→ GAP1-N2+GAP1-M+TM+GAP1-C*→?→ TM+vWA(canonical)+TM+low_complexity→	839	Streptomyces fulvoviolaceus	GCF_000718165.1	actinobacteria
WP_052429335.1	GAP1-N2+GAP1-M+GAP1-C+polar_tail+TM+beta-rich	TM+vWA(canonical)+YtKA-like+TM+??→ Tubulin+coiled_coil+??→ Tubulin+coiled_coil+HTH?+??→ TM+TM+TM+vWA(canonical)+TM+TM+TM+polar_tail→ Fibronectin-like_repeats+??→ ZnR+ZnR+DO-GTPase2.2+ZnR+ZnR→ GAP1-N2+GAP1-M+GAP1-C+polar_tail+TM+beta-rich*→	1036	Paenibacillus borealis	GCF_000758665.1	firmicutes
WP_052433373.1	GAP1-N2+GAP1-M+GAP1-C	TPR_containing_alpha_helical+Fibronectin-like_repeats→ ZnR+ZnR+DO-GTPase2.2→ GAP1-N2+GAP1-M+GAP1-C*→ TM+TM+DO-GTPase2→ vWA(canonical)→ PP2C→ Pkinase+ZnR+beta-rich→	672	Streptacidiphilus carbonis	GCF_000787775.1	actinobacteria
WP_052437872.1	GAP1-N2+GAP1-M+GAP1-C	polar_leader+TM+TM+TM→ TM+vWA(canonical)+Fibronectin-like_repeats+TM+beta-rich+lc_tail→ Tubulin+alpha_helical+??+??→ GAP_unknown1+TM+TM+GAP_unknown2→ HRDC-like?+Fibronectin-like_repeats→ GAP1-N2+GAP1-M+GAP1-C*→ beta-rich+vWA(canonical)→??+DO-GTPase2.2→ polar_leader+TM+TM+TM→	456	Corynebacterium humireducens	GCA_000819445.1	actinobacteria
WP_052489237.1	GAP1-N2+GAP1-M+GAP1-C	GAP1-N2+GAP1-M+GAP1-C*→	811	Streptomyces sp. 150FB	GCF_000818195.1	actinobacteria
WP_052503686.1	GAP1-N2+GAP1-M+GAP1-C	GrpE→ HSP70→ TPR_containing_alpha_helical+Fibronectin-like_repeats→ ZnR+ZnR+DO-GTPase2.2→ GAP1-N2+GAP1-M+GAP1-C*→ TM+TM+TM+TM+ZnR+ZnR+DO-GTPase2.2→	814	Micromonospora carbonacea	GCF_000876225.1	actinobacteria
WP_052552158.1	GAP1-N2+GAP1-M+TM	GAP1-N2+GAP1-M+TM*→	273	Gemmata sp. SH-PL17	GCA_001610855.1	planctomycetes
WP_052649326.1	GAP1-N2+GAP1-M+GAP1-C+polar_tail+TM+beta-rich	TM+vWA(canonical)+YtKA-like+TM+??→ Tubulin+coiled_coil+??→ Tubulin+coiled_coil+HTH?+??→ TM+TM+TM+vWA(canonical)+TM+TM+TM+polar_tail→ Fibronectin-like_repeats+??→ ZnR+ZnR+DO-GTPase2.2+ZnR+ZnR→ GAP1-N2+GAP1-M+GAP1-C+polar_tail+TM+beta-rich*→	1002	Paenibacillus vortex	GCF_000193415.1	firmicutes
WP_052676055.1	GAP1-N2+GAP1-M+GAP1-C+polar_tail+TM+beta-rich	TM+vWA(canonical)+YtKA-like+TM+??→ Tubulin+coiled_coil+??→ Tubulin+coiled_coil+HTH?+??→ TM+TM+TM+vWA(canonical)+TM+TM+TM+polar_tail→ Fibronectin-like_repeats+??→ ZnR+ZnR+DO-GTPase2.2+ZnR+ZnR→ GAP1-N2+GAP1-M+GAP1-C+polar_tail+TM+beta-rich*→	1011	Paenibacillus sp. IHBB 10380	GCF_000949425.1	firmicutes
WP_052737023.1	GAP1-N2+GAP1-M+GAP1-C+polar_tail+TM+beta-rich	TM+vWA(canonical)+YtKA-like+TM+??→ Tubulin+coiled_coil+??→ Tubulin+coiled_coil+HTH?+??→ TM+TM+TM+vWA(canonical)+TM+TM+TM+polar_tail→ Fibronectin-like_repeats+??→ ZnR+ZnR+DO-GTPase2.2+ZnR+ZnR→ GAP1-N2+GAP1-M+GAP1-C+polar_tail+TM+beta-rich*→	1065	Paenibacillus algorifonticola	GCA_900112925.1	firmicutes
WP_052741416.1	GAP1-N2+GAP1-M+GAP1-C+polar_tail+TM+beta-rich	TM+vWA(canonical)+YtKA-like+TM+??→ Tubulin+coiled_coil+??→ Tubulin+coiled_coil+HTH?+??→ TM+TM+TM+vWA(canonical)+TM+TM+TM+polar_tail→ Fibronectin-like_repeats+??→ ZnR+ZnR+DO-GTPase2.2+ZnR+ZnR→ GAP1-N2+GAP1-M+GAP1-C+polar_tail+TM+beta-rich*→	1037	Paenibacillus riograndensis	GCA_000981585.1	firmicutes
WP_052755973.1	GAP1-N2+GAP1-M+GAP1-C+polar_tail+TM+beta-rich	TM+vWA(canonical)+YtKA-like+TM+??→ Tubulin+coiled_coil+??→ Tubulin+coiled_coil+HTH?+??→ TM+TM+TM+vWA(canonical)+TM+TM+TM+polar_tail→ Fibronectin-like_repeats+??→ ZnR+ZnR+DO-GTPase2.2+ZnR+ZnR→ GAP1-N2+GAP1-M+GAP1-C+polar_tail+TM+beta-rich*→	1030	Paenibacillus durus	GCF_000993825.1	firmicutes
WP_052770211.1	GAP1-N2+GAP1-M+GAP1-C+polar_tail+TM+beta-rich	TM+vWA(canonical)+YtKA-like+TM+??→ Tubulin+coiled_coil+??→ Tubulin+coiled_coil+HTH?+??→ TM+TM+TM+vWA(canonical)+TM+TM+TM+polar_tail→ Fibronectin-like_repeats+??→?→ ZnR+ZnR+DO-GTPase2.2+ZnR+ZnR→ GAP1-N2+GAP1-M+GAP1-C+polar_tail+TM+beta-rich*→	1006	Paenibacillus sp. IHB B 3415	GCF_000802655.2	firmicutes
WP_052844786.1	GAP1-N2+GAP1-M+low_complexity+TM+TM	polar_leader+TM+TM+TM→ TM+vWA(canonical)+Fibronectin-like_repeats+TM+beta-rich+lc_tail→ Tubulin+alpha_helical+??+??→ GAP_unknown1+TM+TM+GAP_unknown2→ HRDC-like?+Fibronectin-like_repeats→ GAP1-N2+GAP1-M+low_complexity+TM+TM*→ beta-rich+vWA(canonical)→??+DO-GTPase2.2→ polar_leader+TM+TM+TM→	511	Corynebacterium mustelae	GCA_001020985.1	actinobacteria
WP_052890323.1	GAP1-N2+GAP1-M+GAP1-C	ZnR+ZnR+DO-GTPase2.2→ GAP1-N2+GAP1-M+GAP1-C*→	973	Thermogemmatispora carboxidivorans	GCF_000702505.1	chloroflexi
WP_052947460.1	GAP1-N2+GAP1-M+GAP1-C+polar_tail+TM+beta-rich	TM+vWA(canonical)+YtKA-like+TM+??→ Tubulin+coiled_coil+??→ Tubulin+coiled_coil+HTH?+??→ TM+TM+TM+vWA(canonical)+TM+TM+TM+polar_tail(frag)→ Fibronectin-like_repeats+??→ ZnR+ZnR+DO-GTPase2.2+ZnR+ZnR→ GAP1-N2+GAP1-M+GAP1-C+polar_tail+TM+beta-rich*→	800	Aneurinibacillus tyrosinisolvens	GCF_001015055.1	firmicutes
WP_052953154.1	GAP1-N2+GAP1-M+GAP1-C	polar_leader+TM+TM+TM→ TM+vWA(canonical)+Fibronectin-like_repeats+TM+beta-rich+lc_tail→ PSE→ GAP_unknown1+TM+TM+GAP_unknown2→ HRDC-like?+Fibronectin-like_repeats→ GAP1-N2+GAP1-M+GAP1-C*→ beta-rich+vWA(canonical)→??+DO-GTPase2.2→ polar_leader+TM+TM+TM→	496	Corynebacterium striatum	GCF_000988225.1	actinobacteria
WP_053084988.1	GAP1-N2+GAP1-M+GAP1-C	polar_leader+TM+TM+TM→ TM+vWA(canonical)+Fibronectin-like_repeats+TM+beta-rich+lc_tail→ Tubulin+alpha_helical+??+??→ GAP_unknown1+TM+TM+GAP_unknown2→ HRDC-like?+Fibronectin-like_repeats→ GAP1-N2+GAP1-M+GAP1-C*→ beta-rich+vWA(canonical)→??+DO-GTPase2.2→ polar_leader+TM+TM+TM→	535	Corynebacterium striatum	GCA_002803965.1	actinobacteria
WP_053086271.1	GAP1-N2+GAP1-M+GAP1-C	GAP1-N2+GAP1-M+GAP1-C*→ beta-rich+vWA(canonical)→??+DO-GTPase2.2→ polar_leader+TM+TM+TM→	535	Corynebacterium striatum	GCF_001059955.1	actinobacteria
WP_053088001.1	GAP1-N2+GAP1-M+GAP1-C	polar_leader+TM+TM+TM→ TM+vWA(canonical)+Fibronectin-like_repeats+TM+beta-rich+lc_tail→ Tubulin+alpha_helical+??+??→ GAP_unknown1+TM+TM+GAP_unknown2→ HRDC-like?+Fibronectin-like_repeats→ GAP1-N2+GAP1-M+GAP1-C*→ beta-rich+vWA(canonical)→??+DO-GTPase2.2→ polar_leader+TM+TM+TM→	535	Corynebacterium striatum	GCF_001058455.1	actinobacteria
WP_053177183.1	GAP1-N2+GAP1-M+GAP1-C	TPR_containing_alpha_helical+Fibronectin-like_repeats→ PSE→ GAP1-N2+GAP1-M+GAP1-C*→ PSE→ vWA(canonical)→ PP2C→ Pkinase+ZnR+beta-rich→	821	Streptomyces virginiae	GCA_001270565.1	actinobacteria
WP_053211495.1	GAP1-N2+GAP1-M+GAP1-C	TPR_containing_alpha_helical+Fibronectin-like_repeats→ ZnR+ZnR+DO-GTPase2.2→ GAP1-N2+GAP1-M+GAP1-C*→ PSE→ vWA(canonical)→ PP2C→ Pkinase+ZnR+beta-rich→	880	Streptomyces antibioticus	GCA_001270635.1	actinobacteria
WP_053240737.1	GAP1-N2+GAP1-M+GAP1-C	GAP1-N2+GAP1-M+GAP1-C*→	883	Clostridium sp. DMHC 10	GCA_001270375.1	firmicutes
WP_053241473.1	GAP1-N2+GAP1-M+GAP1-C	Fibronectin-like_repeats+??→ PSE→ GAP1-N2+GAP1-M+GAP1-C(frag)*→ GAP1-N2+GAP1-M→?→?→ TM+TM+TM+TM+ZnR+ZnR+DO-GTPase2.2→ TM+vWA(canonical)+YtKA-like+TM+??→ PSE→?→ Tubulin+coiled_coil+HTH?+??→	86	Clostridium sp. DMHC 10	GCA_001270375.1	firmicutes
WP_053241474.1	GAP1-N2+GAP1-M	Fibronectin-like_repeats+??→ PSE→ GAP1-N2+GAP1-M+GAP1-C(frag)→ GAP1-N2+GAP1-M*→?→?→ TM+TM+TM+TM+ZnR+ZnR+DO-GTPase2.2→ TM+vWA(canonical)+YtKA-like+TM+??→ PSE→?→ Tubulin+coiled_coil+HTH?+??→ TM+TM+TM+vWA(canonical)+TM+TM+TM+polar_tail→	224	Clostridium sp. DMHC 10	GCA_001270375.1	firmicutes
WP_053241483.1	GAP1-N2+GAP1-M	TM+TM+TM+TM+ZnR+ZnR+DO-GTPase2.2→ TM+vWA(canonical)+YtKA-like+TM+??→ PSE→?→ Tubulin+coiled_coil+HTH?+??→ TM+TM+TM+vWA(canonical)+TM+TM+TM+polar_tail→ Fibronectin-like_repeats+??→ ZnR+ZnR+DO-GTPase2.2+ZnR+ZnR→ GAP1-N2+GAP1-M*→	432	Clostridium sp. DMHC 10	GCF_001270375.1	firmicutes
WP_053325329.1	GAP1-N2+GAP1-M+GAP1-C+polar_tail+TM+beta-rich	TM+vWA(canonical)+YtKA-like+TM+??→ Tubulin+coiled_coil+??→ Tubulin+coiled_coil+HTH?+??→ TM+TM+TM+vWA(canonical)+TM+TM+TM+polar_tail→ Fibronectin-like_repeats+??→ ZnR+ZnR+DO-GTPase2.2+ZnR+ZnR→ GAP1-N2+GAP1-M+GAP1-C+polar_tail+TM+beta-rich*→	1002	Paenibacillus peoriae	GCA_001272655.2	firmicutes
WP_053375319.1	GAP1-N2+GAP1-M+GAP1-C+polar_tail+TM+beta-rich	TM+vWA(canonical)+YtKA-like+TM+??→ Tubulin+coiled_coil+??→ Tubulin+coiled_coil+HTH?+??→ TM+TM+TM+vWA(canonical)+TM+TM+TM+polar_tail→ Fibronectin-like_repeats+??→ ZnR+ZnR+DO-GTPase2.2+ZnR+ZnR→ GAP1-N2+GAP1-M+GAP1-C+polar_tail+TM+beta-rich*→	985	Paenibacillus sp. FJAT-27812	GCF_001273905.1	firmicutes
WP_053478937.1	GAP1-N2+GAP1-M+GAP1-C+polar_tail+TM+beta-rich	TM+vWA(canonical)+YtKA-like+TM+??→ Tubulin+coiled_coil+??→ Tubulin+coiled_coil+HTH?+??→ TM+TM+TM+vWA(canonical)+TM+TM+TM+polar_tail→ Fibronectin-like_repeats+??→ ZnR+ZnR+DO-GTPase2.2+ZnR+ZnR→ GAP1-N2+GAP1-M+GAP1-C+polar_tail+TM+beta-rich*→	908	Bacillus sp. FJAT-21945	GCA_001275655.1	firmicutes
WP_053491362.1	GAP1-N2+GAP1-M+GAP1-C+polar_tail	Tubulin+coiled_coil+??→ Tubulin+coiled_coil+HTH?+??→ TM+TM+TM+vWA+TM+TM+TM+polar_tail→ Fibronectin-like_repeats+??→ ZnR+ZnR+DO-GTPase2.2→ GAP1-N2+GAP1-M+GAP1-C+polar_tail*→	1022	Bacillus sp. FJAT-18019	GCF_001275645.1	firmicutes
WP_053558754.1	GAP1-N2+GAP1-M+GAP1-C	TPR_containing_alpha_helical+Fibronectin-like_repeats→ ZnR+ZnR+DO-GTPase2.2→ GAP1-N2+GAP1-M+GAP1-C*→ TM+TM+TM+TM+ZnR+ZnR+DO-GTPase2.2+TM→ vWA(canonical)→ PP2C→ Pkinase+ZnR+beta-rich→	885	Streptomyces sp. CFMR 7	GCA_001278095.1	actinobacteria
WP_053558761.1	GAP1-N2+GAP1-M+GAP1-C	TM+TM+TM+TM+ZnR+ZnR+DO-GTPase2.2+TM→ vWA(canonical)→ PP2C→ Pkinase+ZnR+beta-rich→||←?←?←?||GAP1-N2+GAP1-M+GAP1-C*→	894	Streptomyces sp. CFMR 7	GCA_001278095.1	actinobacteria
WP_053585907.1	GAP1-N2+GAP1-M+GAP1-C	GrpE→ HSP70→??+Fibronectin→ ZnR+ZnR+DO-GTPase2.2→ GAP1-N2+GAP1-M+GAP1-C*→	808	Actinomyces sp. oral taxon 414	GCA_001278845.1	actinobacteria
WP_053585921.1	GAP1-N2+GAP1-M+GAP1-C	polar_leader+TM+TM+TM→ TM+vWA(canonical)+Fibronectin-like_repeats+TM+beta-rich+lc_tail→ Tubulin+alpha_helical+??+??→ GAP_unknown1+TM+TM+GAP_unknown2→ HRDC-like?+Fibronectin-like_repeats→ GAP1-N2+GAP1-M+GAP1-C*→?→??+DO-GTPase2.2→	901	Actinomyces sp. oral taxon 414	GCA_001278845.1	actinobacteria
WP_053619425.1	GAP1-N2+GAP1-M+GAP1-C	GAP1-N2+GAP1-M+GAP1-C*→	895	Nocardiopsis sp. NRRL B-16309	GCA_001279585.1	actinobacteria
WP_053731464.1	GAP1-N2+GAP1-M+GAP1-C	TM+vWA(canonical)+TM→ HSP70→??+Fibronectin-like_repeats→ ZnR+ZnR+DO-GTPase2.2→ GAP1-N2+GAP1-M+GAP1-C*→ TM+TM+TM+TM+ZnR+ZnR+DO-GTPase2.2→||←GrpE	747	Nocardia sp. NRRL S-836	GCA_001279525.1	actinobacteria
WP_053732589.1	GAP1-N2+GAP1-M+GAP1-C+TM+beta-rich	vWA(canonical)+beta-rich+low_complexity→?→?→ Tubulin+alpha_helical+??+??→??+TM+TM+TM+TM+??→ Pkinase+TM→ ZnR+ZnR+DO-GTPase2.2→ GAP1-N2+GAP1-M+GAP1-C+TM+beta-rich*→?→||←?||Pkinase+TM+Septum_form→	607	Nocardia sp. NRRL S-836	GCA_001279525.1	actinobacteria
WP_053777217.1	GAP1-N2+GAP1-M+GAP1-C	Tubulin+alpha_helical+??+??→ GAP_unknown1+TM+TM+GAP_unknown2→ HRDC-like?+Fibronectin-like_repeats→ HRDC-like?+Fibronectin-like_repeats→ GAP1-N2+GAP1-M+GAP1-C(frag)*→?→ beta-rich+vWA(canonical)→??+DO-GTPase2.2→	367	Gordonia sp. NB4-1Y	GCF_000347295.2	actinobacteria
WP_053782792.1	GAP1-N2+GAP1-M+GAP1-C+polar_tail	TM+TM+TM+vWA+TM+TM+TM+polar_tail→ Fibronectin-like_repeats+??→ ZnR+ZnR+DO-GTPase2.2→ GAP1-N2+GAP1-M+GAP1-C+polar_tail*→	1079	Paenibacillus sp. A59	GCF_001280595.1	firmicutes
WP_054027553.1	GAP1-N2+GAP1-M+GAP1-C+polar_tail+TM+beta-rich	TM+vWA(canonical)+YtKA-like+TM+??→ Tubulin+coiled_coil+??→ Tubulin+coiled_coil+HTH?+??→ TM+TM+TM+vWA(canonical)+TM+TM+TM+polar_tail→ Fibronectin-like_repeats+??→ ZnR+ZnR+DO-GTPase2.2+ZnR+ZnR→ GAP1-N2+GAP1-M+GAP1-C+polar_tail+TM+beta-rich*→	979	Bacillus sp. FJAT-28004	GCF_001280845.1	firmicutes
WP_054245673.1	GAP1-N2+GAP1-M+GAP1-C	polar_leader+TM+TM+TM→ TM+vWA(canonical)+Fibronectin-like_repeats+TM+beta-rich+lc_tail→ Tubulin+alpha_helical+??+??→ GAP_unknown1+TM+TM+GAP_unknown2→ HRDC-like?+Fibronectin-like_repeats→ GAP1-N2+GAP1-M+GAP1-C*→ beta-rich+vWA(canonical)→??+DO-GTPase2.2→	913	Rhodococcus jostii	GCF_001292845.1	actinobacteria
WP_054291506.1	GAP1-N2+GAP1-M	Pkinase+TM→ ZnR+ZnR+DO-GTPase2.2→ GAP1-N2+GAP1-M*→	400	Kibdelosporangium phytohabitans	GCA_001302585.1	actinobacteria
WP_054404307.1	GAP1-N2+GAP1-M+GAP1-C+polar_tail+TM+beta-rich	TM+vWA(canonical)+YtKA-like+TM+??→ Tubulin+coiled_coil+??→ Tubulin+coiled_coil+HTH?+??→ TM+TM+TM+vWA(canonical)+TM+TM+TM+polar_tail→ Fibronectin-like_repeats+??→ ZnR+ZnR+DO-GTPase2.2+ZnR+ZnR→ GAP1-N2+GAP1-M+GAP1-C+polar_tail+TM+beta-rich*→	1023	Paenibacillus solani	GCF_001277345.1	firmicutes
WP_054569407.1	GAP1-N2+GAP1-M+GAP1-C+ZnR+DO-GTPase2	GAP1-N2+GAP1-M+GAP1-C+ZnR+DO-GTPase2*→	984	Frankia sp. R43	GCA_001306465.1	actinobacteria
WP_054600667.1	GAP1-N2+GAP1-M+GAP1-C	HRDC-like?+Fibronectin-like_repeats→ GAP1-N2+GAP1-M+GAP1-C*→??+DO-GTPase2.2→	820	Mycolicibacterium fortuitum	GCA_001307545.1	actinobacteria
WP_054601302.1	GAP1-N2+GAP1-M+GAP1-C	polar_leader+TM+TM+TM→ TM+vWA(canonical)+Fibronectin-like_repeats+TM+beta-rich+lc_tail→ Tubulin+alpha_helical+??+??→ GAP_unknown1+TM+TM+GAP_unknown2→ HRDC-like?+Fibronectin-like_repeats→ GAP1-N2+GAP1-M+GAP1-C*→ beta-rich+vWA(canonical)→??+DO-GTPase2.2→ TM+DUF4190+TM+PknH_C→ TM+DUF4190+TM+PknH_C→	828	Mycolicibacterium fortuitum	GCA_001307545.1	actinobacteria

acc	architecture	operon	len	species	gca	taxend
WP_054706231.1	GAP1-N2+GAP1-M+GAP1-C	Tubulin+coiled_coil+HTH?+??→ TM+TM+TM+vWA+TM+TM+TM+polar_tail→ Fibronectin-like_repeats+??→ ZnR+ZnR+DO-GTPase2.2→ GAP1-N2+GAP1-M+GAP1-C*→	660	Paenibacillus pinihumi	GCF_001312305.1	firmicutes
WP_054716519.1	GAP1-N2+GAP1-M+GAP1-C	GrpE→ HSP70→ DnaJ+DnaJ+ZnR+ZnR+coiled_coil+Fibronectin-like_repeats→ ZnR+ZnR+DO-GTPase2.2→ GAP1-N2+GAP1-M+GAP1-C*→?→ TM+TM+ZnR+ZnR+DO-GTPase2.2+TM+SH3→ FGS-fold_domain→	815	Marinifilum fragile	GCF_001310955.1	bacteroidetes
WP_054792823.1	GAP1-N2+GAP1-M+GAP1-C	GAP1-N2+GAP1-M+GAP1-C(frag)*→	128	Bacillus sp. JCM 19035	GCF_001310595.1	firmicutes
WP_054874870.1	GAP1-N2+GAP1-M+GAP1-C	GAP1-N2+GAP1-M+GAP1-C*→	842	Oxobacter pfennigii	GCA_001317355.1	firmicutes
WP_054875772.1	GAP1-N2+GAP1-M+GAP1-C+polar_tail+TM+beta-rich	Tubulin+coiled_coil+??→ Tubulin+coiled_coil+HTH?+??→ TM+TM+TM+vWA(canonical)+TM+TM+TM+polar_tail→ Fibronectin-like_repeats+??→ ZnR+ZnR+DO-GTPase2.2+ZnR+ZnR→ GAP1-N2+GAP1-M+GAP1-C+polar_tail+TM+beta-rich*→	901	Oxobacter pfennigii	GCA_001317355.1	firmicutes
WP_054938931.1	GAP1-N2+GAP1-M	TM+vWA(canonical)+YtKA-like+TM+??→ PSE→ PSE→ Tubulin+coiled_coil+HTH?+??→ TM+TM+TM+vWA(canonical)+TM+TM+TM+polar_tail→ Fibronectin-like_repeats+??→ ZnR+ZnR+DO-GTPase2.2+ZnR+ZnR→ GAP1-N2+GAP1-M*→ GAP1-N2+GAP1-M+GAP1-C+polar_tail+TM+beta-rich→	206	Paenibacillus ihuae	GCF_001368795.1	firmicutes
WP_054938932.1	GAP1-M+GAP1-C+polar_tail+TM+beta-rich	TM+vWA(canonical)+YtKA-like+TM+??→ PSE→ PSE→ Tubulin+coiled_coil+HTH?+??→ TM+TM+TM+vWA(canonical)+TM+TM+TM+polar_tail→ Fibronectin-like_repeats+??→ ZnR+ZnR+DO-GTPase2.2+ZnR+ZnR→ GAP1-N2+GAP1-M→ GAP1-M+GAP1-C+polar_tail+TM+beta-rich*→	864	Paenibacillus ihuae	GCF_001368795.1	firmicutes
WP_054971920.1	GAP1-N2+GAP1-M+GAP1-C+polar_tail+TM+Cadherin-like	TM+vWA(canonical)+YtKA-like+TM+??→ Tubulin+coiled_coil+??→ Tubulin+coiled_coil+HTH?+??→?→ TM+TM+TM+vWA(canonical)+TM+TM+TM+polar_tail→ Fibronectin-like_repeats+??→ ZnR+ZnR+DO-GTPase2.2+ZnR+ZnR→ GAP1-N2+GAP1-M+GAP1-C+polar_tail+TM+Cadherin-like*→	933	Paenibacillus sp. A3	GCA_001399685.1	firmicutes
WP_055106677.1	GAP1-N2+GAP1-M+GAP1-C+polar_tail+TM+beta-rich	TM+vWA(canonical)+YtKA-like+TM+??→ Tubulin+coiled_coil+??→ Tubulin+coiled_coil+HTH?+??→ TM+TM+TM+vWA(canonical)+TM+TM+TM+polar_tail→ Fibronectin-like_repeats+??→ ZnR+ZnR+DO-GTPase2.2+ZnR+ZnR→ GAP1-N2+GAP1-M+GAP1-C+polar_tail+TM+beta-rich*→	1114	Paenibacillus ihumii	GCF_001403875.1	firmicutes
WP_055117479.1	GAP1-N2+GAP1-M+GAP1-C	polar_leader+TM+TM+TM→ TM+vWA(canonical)+Fibronectin-like_repeats+TM+beta-rich+lc_tail→ Tubulin+alpha_helical+??+??→ GAP_unknown1+TM+TM+GAP_unknown2→ HRDC-like?+Fibronectin-like_repeats→ GAP1-N2+GAP1-M+GAP1-C*→ beta-rich+vWA(canonical)→??+DO-GTPase2.2→ TM+DUF4190+TM+PknH_C→ TM+DUF4190+TM+PknH_C→	828	Mycolicibacterium peregrinum	GCA_002102345.1	actinobacteria
WP_055119270.1	GAP1-N2+GAP1-M+GAP1-C	HRDC-like?+Fibronectin-like_repeats→ GAP1-N2+GAP1-M+GAP1-C*→??+DO-GTPase2.2→	820	Mycolicibacterium peregrinum	GCA_002102345.1	actinobacteria
WP_055544059.1	GAP1-N2+GAP1-M+GAP1-C	GAP1-N2+GAP1-M+GAP1-C*→ vWA(canonical)→ PP2C→ Pkinase+ZnR+beta-rich→||←TPR_containing_alpha_helical+Fibronectin-like_repeats	811	Streptomyces kanamyceticus	GCF_001418465.1	actinobacteria
WP_055702748.1	GAP1-N2+GAP1-M+GAP1-C	GAP1-N2+GAP1-M+GAP1-C*→ vWA(canonical)→ PP2C→ Pkinase+ZnR+beta-rich→	806	Streptomyces silaceus	GCF_001419745.1	actinobacteria
WP_055745894.1	GAP1-N2+GAP1-M+GAP1-C+polar_tail+TM+beta-rich	TM+vWA(canonical)+YtKA-like+TM+??→ Tubulin+coiled_coil+??→ Tubulin+coiled_coil+HTH?+??→ TM+TM+TM+vWA(canonical)+TM+TM+TM+polar_tail→ Fibronectin-like_repeats+??→ ZnR+ZnR+DO-GTPase2.2+ZnR+ZnR→ GAP1-N2+GAP1-M+GAP1-C+polar_tail+TM+beta-rich*→	819	Brevibacillus choshinensis	GCA_001420695.1	firmicutes
WP_056032052.1	GAP1-N2+GAP1-M+GAP1-C+polar_tail	Tubulin+coiled_coil+??→ Tubulin+coiled_coil+HTH?+??→ TM+TM+TM+vWA+TM+TM+TM+polar_tail→ Fibronectin-like_repeats+??→ ZnR+ZnR+DO-GTPase2.2→ GAP1-N2+GAP1-M+GAP1-C+polar_tail*→	1067	Paenibacillus sp. Leaf72	GCA_001422685.1	firmicutes
WP_056489107.1	GAP1-N2+GAP1-M+GAP1-C+polar_tail+TM+beta-rich	TM+vWA(canonical)+YtKA-like+TM+??→ Tubulin+coiled_coil+??→ Tubulin+coiled_coil+HTH?+??→ TM+TM+TM+vWA(canonical)+TM+TM+TM+polar_tail→ Fibronectin-like_repeats+??→ ZnR+ZnR+DO-GTPase2.2+ZnR+ZnR→ GAP1-N2+GAP1-M+GAP1-C+polar_tail+TM+beta-rich*→	832	Brevibacillus sp. Leaf182	GCA_001423865.2	firmicutes
WP_056641465.1	GAP1-N2+GAP1-M+GAP1-C+polar_tail+TM+beta-rich	TM+vWA(canonical)+YtKA-like+TM+??→ Tubulin+coiled_coil+??→ Tubulin+coiled_coil+HTH?+??→ HRDC-like?+Fibronectin-like_repeats→ Fibronectin-like_repeats+??→ ZnR+ZnR+DO-GTPase2.2+ZnR+ZnR→ GAP1-N2+GAP1-M+GAP1-C+polar_tail+TM+beta-rich*→	996	Paenibacillus sp. Soil522	GCA_001428245.1	firmicutes
WP_056682743.1	GAP1-N2+GAP1-M+GAP1-C+polar_tail+TM+beta-rich	TM+vWA(canonical)+YtKA-like+TM+??→ Tubulin+coiled_coil+??→ Tubulin+coiled_coil+HTH?+??→ TM+TM+TM+vWA(canonical)+TM+TM+TM+polar_tail→ Fibronectin-like_repeats+??→ ZnR+ZnR+DO-GTPase2.2+ZnR+ZnR→ GAP1-N2+GAP1-M+GAP1-C+polar_tail+TM+beta-rich*→	908	Bacillus solani	GCA_001420595.1	firmicutes
WP_056698357.1	GAP1-N2+GAP1-M+GAP1-C	GAP1-N2+GAP1-M+GAP1-C*→	827	Streptomyces	GCA_001426325.1	actinobacteria
WP_056702032.1	GAP1-N2+GAP1-M+GAP1-C+polar_tail+TM+beta-rich	TM+vWA(canonical)+YtKA-like+TM+??→ Tubulin+coiled_coil+??→ Tubulin+coiled_coil+HTH?+??→ TM+TM+TM+vWA(canonical)+TM+TM+TM+polar_tail→ Fibronectin-like_repeats+??→ ZnR+ZnR+DO-GTPase2.2+ZnR+ZnR→ GAP1-N2+GAP1-M+GAP1-C+polar_tail+TM+beta-rich*→	1059	Paenibacillus sp. Root52	GCA_001426865.1	firmicutes
WP_056752398.1	GAP1-N2+GAP1-M+GAP1-C	polar_leader+TM+TM+TM→ TM+vWA(canonical)+Fibronectin-like_repeats+TM+beta-rich+lc_tail→ Tubulin+alpha_helical+??+??→ GAP_unknown1+TM+TM+GAP_unknown2→?→ HRDC-like?+Fibronectin-like_repeats→ GAP1-N2+GAP1-M+GAP1-C*→?→??+DO-GTPase2.2→	706	Nocardioides sp. Root190	GCA_001428565.1	actinobacteria
WP_056791870.1	GAP1-N2+GAP1-M+GAP1-C	GAP1-N2+GAP1-M+GAP1-C*→	804	Streptomyces	GCA_001426615.1	actinobacteria
WP_057666511.1	GAP1-N2+GAP1-M+GAP1-C	GAP1-N2+GAP1-M+GAP1-C*→	827	Streptomyces anulatus	GCA_001434355.1	actinobacteria
WP_057760601.1	GAP1-N2+GAP1-M+GAP1-C+polar_tail+TM+beta-rich	TM+vWA(canonical)+YtKA-like+TM+??→ Tubulin+coiled_coil+??→ Tubulin+coiled_coil+HTH?+??→ TM+TM+TM+vWA(canonical)+TM+TM+TM+polar_tail→ Fibronectin-like_repeats+??→ ZnR+ZnR+DO-GTPase2.2+ZnR+ZnR→ GAP1-N2+GAP1-M+GAP1-C+polar_tail+TM+beta-rich*→	908	Bacillus praedii	GCF_001439605.1	firmicutes
WP_058124885.1	GAP1-N2+GAP1-M+GAP1-C	HRDC-like?+Fibronectin-like_repeats→ GAP1-N2+GAP1-M+GAP1-C*→??+DO-GTPase2.2→	822	Mycolicibacterium smegmatis	GCA_001457595.1	actinobacteria
WP_058301504.1	GAP1-N2+GAP1-M+GAP1-C+polar_tail+TM+beta-rich	TM+vWA(canonical)+YtKA-like+TM+??→ Tubulin+coiled_coil+??→ Tubulin+coiled_coil+HTH?+??→ TM+TM+TM+vWA(canonical)+TM+TM+TM+polar_tail→ Fibronectin-like_repeats+??→ ZnR+ZnR+DO-GTPase2.2+ZnR+ZnR→ GAP1-N2+GAP1-M+GAP1-C+polar_tail+TM+beta-rich*→	911	Gorillibacterium timonense	GCF_001457415.1	firmicutes
WP_058554430.1	GAP1-N2+GAP1-M+GAP1-C	GrpE→ HSP70→ HSP70+vWA(canonical)→?→ ZnR+ZnR+DO-GTPase2.2→ GAP1-N2+GAP1-M+GAP1-C*→?→ TIR→ TM+ZnR+ZnR+DO-GTPase2.2+PEGA+PEGA→	859	Thiohalocapsa sp. ML1	GCF_001469165.1	gammaproteobacteria
WP_058624998.1	GAP1-N2+GAP1-M+GAP1-C	GrpE→ HSP70→ TPR_containing_alpha_helical+Fibronectin-like_repeats→ ZnR+ZnR+DO-GTPase2.2→ GAP1-N2+GAP1-M+GAP1-C*→ TM+TM+TM+TM+ZnR+ZnR+DO-GTPase2.2→	670	Microbacterium testaceum	GCA_001476655.1	actinobacteria
WP_058710444.1	GAP1-N2+GAP1-M+GAP1-C+polar_tail+TM+beta-rich	TM+vWA(canonical)+YtKA-like+TM+??→ Tubulin+coiled_coil+??→ Tubulin+coiled_coil+HTH?+??→ TM+TM+TM+vWA(canonical)+TM+TM+TM+polar_tail→ Fibronectin-like_repeats+??→ ZnR+ZnR+DO-GTPase2.2+ZnR+ZnR→ GAP1-N2+GAP1-M+GAP1-C+polar_tail+TM+beta-rich*→	1009	Paenibacillus	GCA_002872435.1	firmicutes
WP_058829597.1	GAP1-N2+GAP1-M+GAP1-C+polar_tail+TM+beta-rich	TM+vWA(canonical)+YtKA-like+TM+??→ Tubulin+coiled_coil+??→ Tubulin+coiled_coil+HTH?+??→ TM+TM+TM+vWA(canonical)+TM+TM+TM+polar_tail→ Fibronectin-like_repeats+??→ ZnR+ZnR+DO-GTPase2.2+ZnR+ZnR→ GAP1-N2+GAP1-M+GAP1-C+polar_tail+TM+beta-rich*→	1009	Paenibacillus	GCF_001049835.1	firmicutes
WP_059013430.1	GAP1-N2+GAP1-M+GAP1-C	GAP1-N2+GAP1-M+GAP1-C*→	460	Streptomyces specialis	GCF_001493375.1	actinobacteria
WP_059036584.1	GAP1-N2+GAP1-M+GAP1-C	Tubulin+alpha_helical+??+??→ GAP_unknown1+TM+TM+GAP_unknown2→ HRDC-like?+Fibronectin-like_repeats→ GAP1-N2+GAP1-M+GAP1-C*→ beta-rich+vWA(canonical)→??+DO-GTPase2.2→	882	Gordonia desulfuricans	GCF_001485495.1	actinobacteria
WP_059061848.1	GAP1-N2+GAP1-M+GAP1-C	GrpE→ HSP70→ TPR_containing_alpha_helical+Fibronectin-like_repeats→ ZnR+ZnR+DO-GTPase2.2→ GAP1-N2+GAP1-M+GAP1-C*→ TM+TM+TM+TM+ZnR+ZnR+DO-GTPase2.2→?→?→ HSP70→ vWA(canonical)→	677	Leucobacter sp. G161	GCA_001482305.1	actinobacteria
WP_059088768.1	GAP1-N2+GAP1-M+GAP1-C	HRDC-like?+Fibronectin-like_repeats→ GAP1-N2+GAP1-M+GAP1-C*→??+DO-GTPase2.2→	824	Mycobacterium sp. GA-2829	GCA_001499965.1	actinobacteria
WP_059096306.1	GAP1-N2+GAP1-M+GAP1-C	HRDC-like?+Fibronectin-like_repeats→ GAP1-N2+GAP1-M+GAP1-C*→??+DO-GTPase2.2→	830	Mycobacterium sp. IS-1742	GCA_001500125.1	actinobacteria
WP_059160459.1	GAP1-N2+GAP1-M+GAP1-C	HRDC-like?+Fibronectin-like_repeats→ GAP1-N2+GAP1-M+GAP1-C*→??+DO-GTPase2.2→	830	Mycobacterium sp. IS-1496	GCA_001499995.1	actinobacteria
WP_059213934.1	GAP1-N2+GAP1-M+GAP1-C	GAP1-N2+GAP1-M+GAP1-C*→	789	Streptomyces griseus	GCA_001509565.1	actinobacteria
WP_059253638.1	GAP1-N2+GAP1-M+GAP1-C	TPR_containing_alpha_helical+Fibronectin-like_repeats→ ZnR+ZnR+DO-GTPase2.2→ GAP1-N2+GAP1-M+GAP1-C*→ TM+TM+TM+TM+ZnR+ZnR+DO-GTPase2.2+TM→ vWA(canonical)→ PP2C→ Pkinase+ZnR+beta-rich→	941	Streptomyces reticuli	GCA_001511815.1	actinobacteria
WP_060532175.1	GAP1-N2+GAP1-M+GAP1-C+polar_tail+TM+beta-rich	Tubulin+coiled_coil+??→ Tubulin+coiled_coil+HTH?+??→||←?||TM+TM+TM+vWA(canonical)+TM+TM+TM+polar_tail→?→ Fibronectin-like_repeats+??→ ZnR+ZnR+DO-GTPase2.2+ZnR+ZnR→ GAP1-N2+GAP1-M*→	363	Paenibacillus bovis	GCA_001421015.2	firmicutes
WP_060625106.1	GAP1-N2+GAP1-M+GAP1-C+polar_tail+TM+beta-rich	TM+vWA(canonical)+YtKA-like+TM+??→ Tubulin+coiled_coil+??→ Tubulin+coiled_coil+HTH?+??→ TM+TM+TM+vWA(canonical)+TM+TM+TM+polar_tail→ Fibronectin-like_repeats+??→ ZnR+ZnR+DO-GTPase2.2+ZnR+ZnR→ GAP1-N2+GAP1-M+GAP1-C+polar_tail+TM+beta-rich*→	998	Paenibacillus etheri	GCA_001012825.2	firmicutes
WP_060651579.1	GAP1-N2+GAP1-M+GAP1-C	polar_leader+TM+TM+TM→ TM+vWA(canonical)+Fibronectin-like_repeats+TM+beta-rich+lc_tail→ Tubulin+alpha_helical+??+??→ GAP_unknown1+TM+TM+GAP_unknown2→ HRDC-like?+Fibronectin-like_repeats→ GAP1-N2+GAP1-M+GAP1-C*→ beta-rich+vWA(canonical)→??+DO-GTPase2.2→	909	Rhodococcus pyridinivorans	GCF_001465325.1	actinobacteria
WP_060729914.1	GAP1-N2+GAP1-M+GAP1-C	TPR_containing_alpha_helical+Fibronectin-like_repeats→ ZnR+ZnR+DO-GTPase2.2→ GAP1-N2+GAP1-M+GAP1-C*→ TM+TM+TM+TM+ZnR+ZnR+DO-GTPase2.2+TM→ vWA(canonical)→ PP2C→ Pkinase+ZnR+beta-rich→	820	Streptomyces albus	GCA_001541145.1	actinobacteria
WP_060862421.1	GAP1-N2+GAP1-M+GAP1-C+polar_tail+TM+beta-rich	TM+vWA(canonical)+YtKA-like+TM+??→ Tubulin+coiled_coil+??→ Tubulin+coiled_coil+HTH?+??→ TM+TM+TM+vWA(canonical)+TM+TM+TM+polar_tail→ Fibronectin-like_repeats+??→ ZnR+ZnR+DO-GTPase2.2+ZnR+ZnR→ GAP1-N2+GAP1-M+GAP1-C+polar_tail+TM+beta-rich*→	1028	Paenibacillus riograndensis	GCF_001546065.1	firmicutes
WP_060893378.1	GAP1-N2+GAP1-M+GAP1-C	GAP1-N2+GAP1-M+GAP1-C*→ vWA(canonical)→ PP2C→ Pkinase+ZnR+beta-rich→	811	Streptomyces europaeiscabiei	GCF_001550375.1	actinobacteria
WP_060941976.1	GAP1-N2+GAP1-M+GAP1-C	polar_leader+TM+TM+TM→ TM+vWA(canonical)+Fibronectin-like_repeats+TM+beta-rich+lc_tail→ Tubulin+alpha_helical+??+??→ GAP_unknown1+TM+TM+GAP_unknown2→ HRDC-like?+Fibronectin-like_repeats→ GAP1-N2+GAP1-M+GAP1-C*→ beta-rich+vWA(canonical)→??+DO-GTPase2.2→	673	Corynebacterium kroppenstedtii	GCA_001552955.1	actinobacteria
WP_060957505.1	GAP1-N2+GAP1-M+GAP1-C	polar_leader+TM+TM+TM→ TM+vWA(canonical)+Fibronectin-like_repeats+TM+beta-rich+lc_tail→ Tubulin+alpha_helical+??+??→ GAP_unknown1+TM+TM+GAP_unknown2→ HRDC-like?+Fibronectin-like_repeats→ GAP1-N2+GAP1-M+GAP1-C*→?→??+DO-GTPase2.2→	933	Actinomyces oris	GCA_001553935.1	actinobacteria
WP_061043383.1	GAP1-N2+GAP1-M+GAP1-C	polar_leader+TM+TM+TM→ TM+vWA(canonical)+Fibronectin-like_repeats+TM+beta-rich+lc_tail→ Tubulin+alpha_helical+??+??→ GAP_unknown1+TM+TM+GAP_unknown2→ HRDC-like?+Fibronectin-like_repeats→ GAP1-N2+GAP1-M+GAP1-C*→ beta-rich+vWA(canonical)→??+DO-GTPase2.2→	925	Rhodococcus sp. SC4	GCA_001555475.1	actinobacteria
WP_061082632.1	GAP1-N2+GAP1-M+GAP1-C	TPR_containing_alpha_helical+Fibronectin-like_repeats+extended_insert+Fibronectin-like_repeats→ ZnR+ZnR+DO-GTPase2.2→ GAP1-N2+GAP1-M+GAP1-C*→ TM+TM+TM+TM+ZnR+ZnR+DO-GTPase2.2→	902	Nocardiopsis dassonvillei	-	actinobacteria
WP_061265397.1	GAP1-N2+GAP1-M+GAP1-C	polar_leader+TM+TM+TM→ TM+vWA(canonical)+Fibronectin-like_repeats+TM+beta-rich+lc_tail→ Tubulin+alpha_helical+??+??→ GAP_unknown1+TM+TM+GAP_unknown2→ HRDC-like?+Fibronectin-like_repeats→ GAP1-N2+GAP1-M+GAP1-C*→ beta-rich+vWA(canonical)→??+DO-GTPase2.2→ TM+DUF4190+TM+PknH_C→ TM+DUF4190+TM+PknH_C→	828	Mycolicibacterium fortuitum	GCA_001570465.1	actinobacteria
WP_061265617.1	GAP1-N2+GAP1-M+GAP1-C	HRDC-like?+Fibronectin-like_repeats→ GAP1-N2+GAP1-M+GAP1-C*→??+DO-GTPase2.2→	820	Mycolicibacterium fortuitum	GCA_001570465.1	actinobacteria
WP_061294805.1	GAP1-N2+GAP1-M+GAP1-C	GrpE→ HSP70→ TPR_containing_alpha_helical+Fibronectin-like_repeats→ ZnR+ZnR+DO-GTPase2.2→ GAP1-N2+GAP1-M+GAP1-C*→ TM+TM+TM+TM+ZnR+ZnR+DO-GTPase2.2+TM→	721	Herbidospora cretacea	GCF_001570605.1	actinobacteria
WP_061300064.1	GAP1-N2+GAP1-M+GAP1-C+TM+low_complexity+TM	vWA(canonical)→ DO-GTPase1→ GAP1-N2+GAP1-M+GAP1-C+TM+low_complexity+TM*→	765	Herbidospora cretacea	GCF_001570605.1	actinobacteria

acc	architecture	operon	len	species	gca	taxend
WP_061403985.1	GAP1-N2+GAP1-M+GAP1-C	GAP1-N2+GAP1-M+GAP1-C*→	789	Streptomyces albus	GCA_001577385.1	actinobacteria
WP_061696939.1	GAP1-N2+GAP1-M+GAP1-C	TM+vWA(canonical)+Fibronectin-like_repeats+TM+beta-rich+lc_tail→ Tubulin+alpha_helical+??+??→ GAP_unknown1+TM+TM+GAP_unknown2→ HRDC-like?+Fibronectin-like_repeats→ GAP1-N2+GAP1-M+GAP1-C*→ beta-rich+vWA(canonical)→??+DO-GTPase2.2→	925	Rhodococcus sp. LB1	GCA_001583455.1	actinobacteria
WP_061798000.1	GAP1-N2+GAP1-M+GAP1-C+polar_tail+TM+beta-rich	GAP1-N2+GAP1-M+GAP1-C+polar_tail+TM+beta-rich*→	746	Bacillus circulans	GCA_900445325.1	firmicutes
WP_061831359.1	GAP1-N2+GAP1-M+GAP1-C+polar_tail	Tubulin+coiled_coil+??→ Tubulin+coiled_coil+HTH?+??→ TM+TM+TM+vWA+TM+TM+TM+polar_tail→ Fibronectin-like_repeats+??→ ZnR+ZnR+DO-GTPase2.2→ GAP1-N2+GAP1-M+GAP1-C+polar_tail*→	1013	Paenibacillus polymyxa	GCA_001593085.1	firmicutes
WP_061920078.1	GAP1-N2+GAP1-M+GAP1-C	polar_leader+TM+TM+TM→ TM+vWA(canonical)+Fibronectin-like_repeats+TM+beta-rich+lc_tail→ Tubulin+alpha_helical+??+??→ GAP_unknown1+TM+TM+GAP_unknown2→ HRDC-like?+Fibronectin-like_repeats→ GAP1-N2+GAP1-M+GAP1-C*→ beta-rich+vWA(canonical)→??+DO-GTPase2.2→ polar_leader+TM+TM+TM→	515	Corynebacterium	GCA_001579275.1	actinobacteria
WP_062036226.1	GAP1-N2+GAP1-M+GAP1-C	polar_leader+TM+TM+TM→ TM+vWA(canonical)+Fibronectin-like_repeats+TM+beta-rich+lc_tail→ Tubulin+alpha_helical+??+??→ GAP_unknown1+TM+TM+GAP_unknown2→ HRDC-like?+Fibronectin-like_repeats→ GAP1-N2+GAP1-M+GAP1-C*→ beta-rich+vWA(canonical)→??+DO-GTPase2.2→ polar_leader+TM+TM+TM→	519	Corynebacterium simulans	GCA_001586215.1	actinobacteria
WP_062042647.1	GAP1-N2+GAP1-M+GAP1-C	polar_leader+TM+TM+TM→ TM+vWA(canonical)+Fibronectin-like_repeats+TM+beta-rich+lc_tail→ Tubulin+alpha_helical+??+??→ GAP_unknown1+TM+TM+GAP_unknown2→ HRDC-like?+Fibronectin-like_repeats→ GAP1-N2+GAP1-M+GAP1-C*→ beta-rich+vWA(canonical)→??+DO-GTPase2.2→ polar_leader+TM+TM+TM→	515	Corynebacterium simulans	GCA_001586235.1	actinobacteria
WP_062149433.1	GAP1-N2+GAP1-M+GAP1-C	TPR_containing_alpha_helical+Fibronectin-like_repeats→ ZnR+ZnR+DO-GTPase2.2→ GAP1-N2+GAP1-M+GAP1-C*→ TM+TM+TM+TM+ZnR+ZnR+DO-GTPase2.2+TM→ vWA(canonical)→ PP2C→ Pkinase+ZnR+beta-rich→	959	Streptomyces curacoi	GCA_001513975.1	actinobacteria
WP_062321561.1	GAP1-N2+GAP1-M+GAP1-C+polar_tail+TM+beta-rich	TM+vWA(canonical)+YtKA-like+TM+??→ Tubulin+coiled_coil+??→ Tubulin+coiled_coil+HTH?+??→ TM+TM+TM+vWA(canonical)+TM+TM+TM+polar_tail→ Fibronectin-like_repeats+??→ ZnR+ZnR+DO-GTPase2.2+ZnR+ZnR→ GAP1-N2+GAP1-M+GAP1-C+polar_tail+TM+beta-rich*→	1077	Paenibacillus pabuli	GCF_001514495.1	firmicutes
WP_062331965.1	GAP1-N2+GAP1-M+GAP1-C	GrpE→ HSP70→ TPR_containing_alpha_helical+Fibronectin-like_repeats→ ZnR+ZnR+DO-GTPase2.2→ GAP1-N2+GAP1-M+TM+GAP1-C*→	733	Herbidospora sakaeratensis	GCF_001570525.1	actinobacteria
WP_062337280.1	GAP1-N2+GAP1-M+GAP1-C+TM+low_complexity+TM	vWA(canonical)→ DO-GTPase1→ GAP1-N2+GAP1-M+GAP1-C+TM+low_complexity+TM*→	753	Herbidospora sakaeratensis	GCF_001570525.1	actinobacteria
WP_062339928.1	GAP1-N2+GAP1-M+GAP1-C	GrpE→ HSP70→ TPR_containing_alpha_helical+Fibronectin-like_repeats→ ZnR+ZnR+DO-GTPase2.2→ GAP1-N2+GAP1-M+GAP1-C*→ TM+TM+TM+TM+ZnR+ZnR+DO-GTPase2.2+TM→	725	Herbidospora sakaeratensis	GCF_001570525.1	actinobacteria
WP_062349315.1	GAP1-N2+GAP1-M+GAP1-C+TM+low_complexity+TM	vWA(canonical)→ DO-GTPase1→ GAP1-N2+GAP1-M+GAP1-C+TM+low_complexity+TM*→	750	Herbidospora yilanensis	GCF_001570565.1	actinobacteria
WP_062357812.1	GAP1-N2+GAP1-M+GAP1-C	GrpE→ HSP70→ TPR_containing_alpha_helical+Fibronectin-like_repeats→ ZnR+ZnR+DO-GTPase2.2→ GAP1-N2+GAP1-M+GAP1-C*→ TM+TM+TM+TM+ZnR+ZnR+DO-GTPase2.2+TM→	725	Herbidospora yilanensis	GCF_001570565.1	actinobacteria
WP_062440995.1	GAP1-N2+GAP1-M+GAP1-C+TM+low_complexity+TM	vWA(canonical)→ DO-GTPase1→ GAP1-N2+GAP1-M+GAP1-C+TM+low_complexity+TM*→	744	Herbidospora daliensis	GCF_001570585.1	actinobacteria
WP_062443634.1	GAP1-N2+GAP1-M+GAP1-C	GrpE→ HSP70→ TPR_containing_alpha_helical+Fibronectin-like_repeats→ ZnR+ZnR+DO-GTPase2.2→ GAP1-N2+GAP1-M+GAP1-C*→ TM+TM+TM+TM+ZnR+ZnR+DO-GTPase2.2+TM→	725	Herbidospora daliensis	GCF_001570585.1	actinobacteria
WP_062519551.1	GAP1-N2+GAP1-M+GAP1-C+polar_tail+TM+beta-rich	TM+vWA(canonical)+YtKA-like+TM+??→ Tubulin+coiled_coil+??→ Tubulin+coiled_coil+HTH?+??→?→ TM+TM+TM+vWA(canonical)+TM+TM+TM+polar_tail→ Fibronectin-like_repeats+??→ ZnR+ZnR+DO-GTPase2.2+ZnR+ZnR→ GAP1-N2+GAP1-M+GAP1-C+polar_tail+TM+beta-rich*→	1037	Paenibacillus jilunlii	GCF_001546055.1	firmicutes
WP_062530245.1	GAP1-N2+GAP1-M+GAP1-C	polar_leader+TM+TM+TM→ TM+vWA(canonical)+Fibronectin-like_repeats+TM+beta-rich+lc_tail→ Tubulin+alpha_helical+??+??→ GAP_unknown1+TM+TM+GAP_unknown2→ HRDC-like?+Fibronectin-like_repeats→ GAP1-N2+GAP1-M+GAP1-C*→ beta-rich+vWA(canonical)→??+DO-GTPase2.2→	965	Lysinimicrobium rhizosphaerae	GCF_000971455.1	actinobacteria
WP_062775528.1	GAP1-N2+GAP1-M+GAP1-C	GAP1-N2+GAP1-M+GAP1-C*→ vWA(canonical)→ PP2C→ Pkinase+ZnR+beta-rich→||←TPR_containing_alpha_helical+Fibronectin-like_repeats	790	Streptomyces sp. NRRL S-1521	GCA_001509505.1	actinobacteria
WP_062795562.1	GAP1-N2+GAP1-M+GAP1-C	polar_leader+TM+TM+TM→ TM+vWA(canonical)+Fibronectin-like_repeats+TM+beta-rich+lc_tail→ Tubulin+alpha_helical+??+??→ GAP_unknown1+TM+TM+GAP_unknown2→ HRDC-like?+Fibronectin-like_repeats→ GAP1-N2+GAP1-M+GAP1-C*→ beta-rich+vWA(canonical)→??+DO-GTPase2.2→	913	Williamsia muralis	GCA_003634525.1	actinobacteria
WP_062829464.1	GAP1-N2+GAP1-M+GAP1-C	TM+DUF4190+TM→ polar_leader+TM+TM+TM→ PSE→ Tubulin+alpha_helical+??+??→ GAP_unknown1+TM+TM+GAP_unknown2→ HRDC-like?+Fibronectin-like_repeats→ GAP1-N2+GAP1-M+GAP1-C*→ beta-rich+vWA(canonical)→??+DO-GTPase2.2→ TM+DUF4190+TM+PknH_C→ TM+DUF4190+TM+PknH_C→ TM+DUF4190+TM+PknH_C→	837	Mycolicibacterium brisbanense	GCA_001570425.1	actinobacteria
WP_062836883.1	GAP1-N2+GAP1-M+GAP1-C+polar_tail+TM+beta-rich	TM+vWA(canonical)+YtKA-like+TM+??→ Tubulin+coiled_coil+??→ Tubulin+coiled_coil+HTH?+??→ TM+TM+TM+vWA(canonical)+TM+TM+TM+polar_tail→ Fibronectin-like_repeats+??→ ZnR+ZnR+DO-GTPase2.2+ZnR+ZnR→ GAP1-N2+GAP1-M+GAP1-C+polar_tail+TM+beta-rich*→	1061	Paenibacillus amylolyticus	GCF_001570725.1	firmicutes
WP_063180107.1	GAP1-N2+GAP1-M+GAP1-C+polar_tail+TM+Cadherin-like	TM+vWA(canonical)+YtKA-like+TM+??→ Tubulin+coiled_coil+??→ Tubulin+coiled_coil+HTH?+??→?→ TM+TM+TM+vWA(canonical)+TM+TM+TM+polar_tail→ Fibronectin-like_repeats+??→ ZnR+ZnR+DO-GTPase2.2+ZnR+ZnR→ GAP1-N2+GAP1-M+GAP1-C+polar_tail+TM+Cadherin-like*→	936	Paenibacillus elgii	GCA_001619725.1	firmicutes
WP_063212701.1	GAP1-N2+GAP1-M+GAP1-C+polar_tail+TM+beta-rich	TM+vWA(canonical)+YtKA-like+TM+??→ Tubulin+coiled_coil+??→ Tubulin+coiled_coil+HTH?+??→ TM+TM+TM+vWA(canonical)+TM+TM+TM+polar_tail→ Fibronectin-like_repeats+??→ ZnR+ZnR+DO-GTPase2.2+ZnR+ZnR→ GAP1-N2+GAP1-M+GAP1-C+polar_tail+TM+beta-rich*→	1009	Paenibacillus jamilae	GCA_001619755.1	firmicutes
WP_063231512.1	GAP1-N2+GAP1-M+GAP1-C+polar_tail+TM+beta-rich	TM+vWA(canonical)+YtKA-like+TM+??→ Tubulin+coiled_coil+??→ Tubulin+coiled_coil+HTH?+??→ TM+TM+TM+vWA(canonical)+TM+TM+TM+polar_tail→ Fibronectin-like_repeats+??→ ZnR+ZnR+DO-GTPase2.2+ZnR+ZnR→ GAP1-N2+GAP1-M+GAP1-C+polar_tail+TM+beta-rich*→	836	Brevibacillus parabrevis	GCA_001619605.1	firmicutes
WP_063477831.1	GAP1-N2+GAP1-M+GAP1-C+polar_tail+TM+beta-rich	TM+vWA(canonical)+YtKA-like+TM+??→ Tubulin+coiled_coil+??→ Tubulin+coiled_coil+HTH?+??→ TM+TM+TM+vWA(canonical)+TM+TM+TM+polar_tail→ Fibronectin-like_repeats+??→ ZnR+ZnR+DO-GTPase2.2+ZnR+ZnR→ GAP1-N2+GAP1-M+GAP1-C+polar_tail+TM+beta-rich*→	1002	Paenibacillus glucanolyticus	GCA_001633025.1	firmicutes
WP_063555795.1	GAP1-N2+GAP1-M+GAP1-C	Fibronectin-like_repeats+??→ PSE→?→ ZnR+ZnR+DO-GTPase2.2→ GAP1-N2+GAP1-M+GAP1-C*→ TM+vWA(canonical)+YtKA-like+TM+??→ Tubulin+coiled_coil+??→ Tubulin+coiled_coil+HTH?+??→ TM+TM+TM+vWA(canonical)+TM+TM+TM+polar_tail→ Fibronectin-like_repeats+??→	870	Clostridium ljungdahlii	GCA_001636845.1	firmicutes
WP_063555805.1	GAP1-N2+GAP1-M	TM+TM+TM+vWA(canonical)+TM+TM+TM+polar_tail→ Fibronectin-like_repeats+??→?→?→?→?→ ZnR+ZnR+DO-GTPase2.2+ZnR+ZnR→ GAP1-N2+GAP1-M*→	351	Clostridium ljungdahlii	GCA_001636845.1	firmicutes
WP_063566909.1	GAP1-N2+GAP1-M+GAP1-C+polar_tail+TM+beta-rich	Tubulin+coiled_coil+HTH?+??→ TM+TM+TM+vWA(canonical)+TM+TM+TM+polar_tail→ Fibronectin-like_repeats+??→ ZnR+ZnR+DO-GTPase2.2+ZnR+ZnR→ GAP1-N2+GAP1-M+GAP1-C+polar_tail+TM+beta-rich*→	1056	Paenibacillus sp. O199	GCF_001636635.1	firmicutes
WP_063601386.1	GAP1-N2+GAP1-M+GAP1-C	Fibronectin-like_repeats+??→ ZnR+ZnR+DO-GTPase2.2→ GAP1-N2+GAP1-M+GAP1-C*→ TM+vWA(canonical)+YtKA-like+TM+??→ Tubulin+coiled_coil+??→ Tubulin+coiled_coil+HTH?+??→ TM+TM+TM+vWA(canonical)+TM+TM+TM+polar_tail→ Fibronectin-like_repeats+??→	868	Clostridium coskatii	GCA_001636835.1	firmicutes
WP_063601394.1	GAP1-N2+GAP1-M	TM+TM+TM+vWA(canonical)+TM+TM+TM+polar_tail→ Fibronectin-like_repeats+??→?→?→?→?→ ZnR+ZnR+DO-GTPase2.2+ZnR+ZnR→ GAP1-N2+GAP1-M*→	351	Clostridium coskatii	GCA_001636835.1	firmicutes
WP_063726900.1	GAP1-N2+GAP1-M+GAP1-C	TPR_containing_alpha_helical+Fibronectin-like_repeats→?→ ZnR+ZnR+DO-GTPase2.2→ GAP1-N2+GAP1-M+GAP1-C*→ TM+TM+TM+TM+ZnR+ZnR+DO-GTPase2.2+TM→ vWA(canonical)→ PP2C→ Pkinase+ZnR+beta-rich→	823	Streptomyces sp. RTd22	GCF_001650215.1	actinobacteria
WP_063792856.1	GAP1-N2+GAP1-M+GAP1-C	TPR_containing_alpha_helical+Fibronectin-like_repeats→ ZnR+ZnR+DO-GTPase2.2→ GAP1-N2+GAP1-M+GAP1-C*→ TM+TM+TM+TM+ZnR+ZnR+DO-GTPase2.2+TM→ vWA(canonical)→ PP2C→ Pkinase+ZnR+beta-rich→	936	Streptomyces aurantiacus	GCF_001418335.1	actinobacteria
WP_063938666.1	GAP1-N2+GAP1-M+GAP1-C	polar_leader+TM+TM+TM→ TM+vWA(canonical)+Fibronectin-like_repeats+TM+beta-rich+lc_tail→ Tubulin+alpha_helical+??+??→ GAP_unknown1+TM+TM+GAP_unknown2→ HRDC-like?+Fibronectin-like_repeats→ GAP1-N2+GAP1-M+GAP1-C*→ beta-rich+vWA(canonical)→??+DO-GTPase2.2→?→||←?←?←HSP70←GrpE	536	Corynebacterium afermentans	GCA_001639025.1	actinobacteria
WP_063973810.1	GAP1-N2+GAP1-M+GAP1-C	GrpE→ HSP70→ TPR_containing_alpha_helical+Fibronectin-like_repeats→ ZnR+ZnR+DO-GTPase2.2→ GAP1-N2+GAP1-M+GAP1-C*→ TM+TM+TM+TM+ZnR+ZnR+DO-GTPase2.2→?→ HSP70→ vWA(canonical)→	806	Dietzia cinnamea	GCA_001643175.1	actinobacteria
WP_064018298.1	GAP1-N2+GAP1-M+GAP1-C+polar_tail+TM+beta-rich	TM+vWA(canonical)+YtKA-like+TM+??→ Tubulin+coiled_coil+??→ Tubulin+coiled_coil+HTH?+??→ TM+TM+TM+vWA(canonical)+TM+TM+TM+polar_tail→ Fibronectin-like_repeats+??→ ZnR+ZnR+DO-GTPase2.2+ZnR+ZnR→ GAP1-N2+GAP1-M+GAP1-C+polar_tail+TM+beta-rich*→	833	Brevibacillus sp. SKDU10	GCA_001645205.1	firmicutes
WP_064063188.1	GAP1-N2+GAP1-M+GAP1-C	polar_leader+TM+TM+TM→ TM+vWA(canonical)+Fibronectin-like_repeats+TM+beta-rich+lc_tail→ Tubulin+alpha_helical+??+??→ GAP_unknown1+TM+TM+GAP_unknown2→ HRDC-like?+Fibronectin-like_repeats→ GAP1-N2+GAP1-M+GAP1-C*→ beta-rich+vWA(canonical)→??+DO-GTPase2.2→	909	Rhodococcus gordoniae	GCA_900455725.1	actinobacteria
WP_064200137.1	GAP1-N2+GAP1-M+GAP1-C+polar_tail+TM+beta-rich	TM+vWA(canonical)+YtKA-like+TM+??→ Tubulin+coiled_coil+??→ Tubulin+coiled_coil+HTH?+??→ TM+TM+TM+vWA(canonical)+TM+TM+TM+polar_tail→ Fibronectin-like_repeats+??→ ZnR+ZnR+DO-GTPase2.2+ZnR+ZnR→ GAP1-N2+GAP1-M+GAP1-C+polar_tail+TM+beta-rich*→	832	Brevibacillus brevis	GCF_001649505.1	firmicutes
WP_064346714.1	GAP1-N2+GAP1-M+GAP1-C	TM+TM+TM+TM+ZnR+ZnR+DO-GTPase2.2→?→ HSP70→ HSP70+vWA(canonical)→ DnaJ+DnaJ+ZnR+ZnR+coiled_coil+Fibronectin-like_repeats→ ZnR+ZnR+DO-GTPase2.2→ GAP1-N2+GAP1-M+GAP1-C*→	914	Gardnerella vaginalis	GCF_001546485.1	actinobacteria
WP_064641835.1	GAP1-N2+GAP1-M+GAP1-C+polar_tail	TM+TM+TM+vWA+TM+TM+TM+polar_tail→ Fibronectin-like_repeats+??→ ZnR+ZnR+DO-GTPase2.2→ GAP1-N2+GAP1-M+GAP1-C+polar_tail*→	1058	Paenibacillus sp. AD87	GCA_001659845.1	firmicutes
WP_064797897.1	GAP1-N2+GAP1-M+GAP1-C+polar_tail	Tubulin+coiled_coil+??→ Tubulin+coiled_coil+HTH?+??→ TM+TM+TM+vWA+TM+TM+TM+polar_tail→ Fibronectin-like_repeats+??→ ZnR+ZnR+DO-GTPase2.2→ GAP1-N2+GAP1-M+GAP1-C+polar_tail*→	1009	Paenibacillus polymyxa	GCA_001663585.1	firmicutes
WP_064850367.1	GAP1-N2+GAP1-M+GAP1-C	HRDC-like?+Fibronectin-like_repeats→ GAP1-N2+GAP1-M+GAP1-C*→??+DO-GTPase2.2→	820	Mycolicibacterium fortuitum	GCA_001665455.1	actinobacteria
WP_064851038.1	GAP1-N2+GAP1-M+GAP1-C	polar_leader+TM+TM+TM→ TM+vWA(canonical)+Fibronectin-like_repeats+TM+beta-rich+lc_tail→ Tubulin+alpha_helical+??+??→ GAP_unknown1+TM+TM+GAP_unknown2→ HRDC-like?+Fibronectin-like_repeats→ GAP1-N2+GAP1-M+GAP1-C*→ beta-rich+vWA(canonical)→??+DO-GTPase2.2→ TM+DUF4190+TM+PknH_C→ TM+DUF4190+TM+PknH_C→	828	Mycolicibacterium fortuitum	GCA_001665455.1	actinobacteria
WP_064865881.1	GAP1-N2+GAP1-M+GAP1-C	HRDC-like?+Fibronectin-like_repeats→ GAP1-N2+GAP1-M+GAP1-C*→??+DO-GTPase2.2→	820	Mycolicibacterium fortuitum	GCA_001665415.1	actinobacteria
WP_064869821.1	GAP1-N2+GAP1-M+GAP1-C	polar_leader+TM+TM+TM→ TM+vWA(canonical)+Fibronectin-like_repeats+TM+beta-rich+lc_tail→ Tubulin+alpha_helical+??+??→ GAP_unknown1+TM+TM+GAP_unknown2→ HRDC-like?+Fibronectin-like_repeats→ GAP1-N2+GAP1-M+GAP1-C*→ beta-rich+vWA(canonical)→??+DO-GTPase2.2→ TM+DUF4190+TM+PknH_C→	828	Mycolicibacterium fortuitum	GCA_001665415.1	actinobacteria
WP_064871995.1	GAP1-N2+GAP1-M+GAP1-C	HRDC-like?+Fibronectin-like_repeats→ GAP1-N2+GAP1-M+GAP1-C*→??+DO-GTPase2.2→	819	Mycolicibacterium setense	GCA_001665445.1	actinobacteria
WP_064872981.1	GAP1-N2+GAP1-M+GAP1-C	HRDC-like?+Fibronectin-like_repeats→ GAP1-N2+GAP1-M+GAP1-C*→??+DO-GTPase2.2→	829	Mycobacterium sp. 852013-50142_SCH4511227	GCA_001665825.1	actinobacteria
WP_064878447.1	GAP1-N2+GAP1-M+GAP1-C	TM+vWA(canonical)+Fibronectin-like_repeats+TM+beta-rich+lc_tail→ Tubulin+alpha_helical+??+??→ GAP_unknown1+TM+TM+GAP_unknown2→ HRDC-like?+Fibronectin-like_repeats→ GAP1-N2+GAP1-M+GAP1-C*→ beta-rich+vWA(canonical)→??+DO-GTPase2.2→ TM+DUF4190+TM+PknH_C→	828	Mycolicibacterium peregrinum	GCA_001665625.1	actinobacteria
WP_064878646.1	GAP1-N2+GAP1-M+GAP1-C	HRDC-like?+Fibronectin-like_repeats→ GAP1-N2+GAP1-M+GAP1-C*→??+DO-GTPase2.2→	820	Mycolicibacterium peregrinum	GCA_001665625.1	actinobacteria
WP_064894838.1	GAP1-N2+GAP1-M+GAP1-C	HRDC-like?+Fibronectin-like_repeats→ GAP1-N2+GAP1-M+GAP1-C*→??+DO-GTPase2.2→	819	Mycolicibacterium conceptionense	GCA_001665335.1	actinobacteria
WP_064900244.1	GAP1-N2+GAP1-M+GAP1-C	HRDC-like?+Fibronectin-like_repeats→ GAP1-N2+GAP1-M+GAP1-C*→??+DO-GTPase2.2→	820	Mycolicibacterium fortuitum	GCA_001665315.1	actinobacteria

acc	architecture	operon	len	species	gca	taxend
WP_064901077.1	GAP1-N2+GAP1-M+GAP1-C	polar_leader+TM+TM+TM→ TM+vWA(canonical)+Fibronectin-like_repeats+TM+beta-rich+lc_tail→ Tubulin+alpha_helical+??+??→ GAP_unknown1+TM+TM+GAP_unknown2→ HRDC-like?+Fibronectin-like_repeats→ GAP1-N2+GAP1-M+GAP1-C*→ beta-rich+vWA(canonical)→??+DO-GTPase2.2→ TM+DUF4190+TM+PknH_C→	828	Mycolicibacterium fortuitum	GCA_001665315.1	actinobacteria
WP_064912096.1	GAP1-N2+GAP1-M+GAP1-C	HRDC-like?+Fibronectin-like_repeats→ GAP1-N2+GAP1-M+GAP1-C*→??+DO-GTPase2.2→	820	Mycolicibacterium fortuitum	GCA_001666795.1	actinobacteria
WP_064917033.1	GAP1-N2+GAP1-M+GAP1-C	HRDC-like?+Fibronectin-like_repeats→ GAP1-N2+GAP1-M+GAP1-C*→??+DO-GTPase2.2→	829	Mycobacterium sp. 852002-10379_SCH5584320	GCA_001667465.1	actinobacteria
WP_064924697.1	GAP1-N2+GAP1-M+GAP1-C	HRDC-like?+Fibronectin-like_repeats→ GAP1-N2+GAP1-M+GAP1-C*→??+DO-GTPase2.2→	820	Mycolicibacterium fortuitum	GCA_001666985.1	actinobacteria
WP_064932157.1	GAP1-N2+GAP1-M+GAP1-C	TM+[lc_insert+]vWA(canonical)+Fibronectin-like_repeats+TM+??+lc_tail→ Tubulin+alpha_helical+??+??→ GAP_unknown1+TM+TM+GAP_unknown2→ HRDC-like?+Fibronectin-like_repeats→ GAP1-N2+GAP1-M+GAP1-C*→ beta-rich+vWA(canonical)→??+DO-GTPase2.2→ TM+DUF4190+TM+PknH_C→	828	Mycolicibacterium peregrinum	GCA_001665785.1	actinobacteria
WP_064932166.1	GAP1-N2+GAP1-M+GAP1-C	HRDC-like?+Fibronectin-like_repeats→ GAP1-N2+GAP1-M+GAP1-C*→??+DO-GTPase2.2→	821	Mycolicibacterium peregrinum	GCA_001665785.1	actinobacteria
WP_064948402.1	GAP1-N2+GAP1-M+GAP1-C	TM+DUF4190+TM→ polar_leader+TM+TM+TM→ PSE→ Tubulin+alpha_helical+??+??→ GAP_unknown1+TM+TM+GAP_unknown2→ HRDC-like?+Fibronectin-like_repeats→ GAP1-N2+GAP1-M+GAP1-C*→ beta-rich+vWA(canonical)→??+DO-GTPase2.2→ TM+DUF4190+TM+PknH_C→ TM+DUF4190+TM+PknH_C→ TM+DUF4190+TM+PknH_C→	828	Mycobacterium sp. 852013-50091_SCH5140682	GCA_001665685.1	actinobacteria
WP_064957532.1	GAP1-N2+GAP1-M+GAP1-C	HRDC-like?+Fibronectin-like_repeats→ GAP1-N2+GAP1-M+GAP1-C*→??+DO-GTPase2.2→	820	Mycolicibacterium peregrinum	GCA_001667205.1	actinobacteria
WP_064958974.1	GAP1-N2+GAP1-M+GAP1-C	polar_leader+TM+TM+TM→ TM+vWA(canonical)+Fibronectin-like_repeats+TM+beta-rich+lc_tail→ Tubulin+alpha_helical+??+??→ GAP_unknown1+TM+TM+GAP_unknown2→ HRDC-like?+Fibronectin-like_repeats→ GAP1-N2+GAP1-M+GAP1-C*→ beta-rich+vWA(canonical)→??+DO-GTPase2.2→ TM+DUF4190+TM+PknH_C→ TM+DUF4190+TM+PknH_C→	828	Mycolicibacterium peregrinum	GCA_001667205.1	actinobacteria
WP_064963236.1	GAP1-N2+GAP1-M+GAP1-C+polar_tail+TM+beta-rich	TM+vWA(canonical)+YtKA-like+TM+??→ Tubulin+coiled_coil+??→ Tubulin+coiled_coil+HTH?+??→ TM+TM+TM+vWA(canonical)+TM+TM+TM+polar_tail→ Fibronectin-like_repeats+??→ ZnR+ZnR+DO-GTPase2.2+ZnR+ZnR→ GAP1-N2+GAP1-M+GAP1-C+polar_tail+TM+beta-rich*→	1009	Paenibacillus polymyxa	GCA_001662815.1	firmicutes
WP_065021493.1	GAP1-N2+GAP1-M+GAP1-C	HRDC-like?+Fibronectin-like_repeats→ GAP1-N2+GAP1-M+GAP1-C*→??+DO-GTPase2.2→	820	Mycolicibacterium fortuitum	GCA_001672855.1	actinobacteria
WP_065030538.1	GAP1-N2+GAP1-M+GAP1-C	TM+DUF4190+TM→ TM+DUF4190+TM→ polar_leader+TM+TM+TM→ TM+vWA(canonical)+Fibronectin-like_repeats+TM+beta-rich+lc_tail→ Tubulin+alpha_helical+??+??→ GAP_unknown1+TM+TM+GAP_unknown2→ HRDC-like?+Fibronectin-like_repeats→ GAP1-N2+GAP1-M+GAP1-C*→ beta-rich+vWA(canonical)→??+DO-GTPase2.2→ TM+DUF4190+TM+PknH_C→ TM+DUF4190+TM+PknH_C→ TM+DUF4190+TM+PknH_C→ TM+DUF4190+TM+PknH_C→	829	Mycobacterium colombiense	GCA_001672755.1	actinobacteria
WP_065052210.1	GAP1-N2+GAP1-M+GAP1-C	polar_leader+TM+TM+TM→ TM+vWA(canonical)+Fibronectin-like_repeats+TM+beta-rich+lc_tail→ Tubulin+alpha_helical+??+??→ GAP_unknown1+TM+TM+GAP_unknown2→ HRDC-like?+Fibronectin-like_repeats→ GAP1-N2+GAP1-M+GAP1-C*→ beta-rich+vWA(canonical)→??+DO-GTPase2.2→ TM+DUF4190+TM+PknH_C→ TM+DUF4190+TM+PknH_C→	828	Mycolicibacterium fortuitum	GCA_001673325.1	actinobacteria
WP_065052315.1	GAP1-N2+GAP1-M+GAP1-C	HRDC-like?+Fibronectin-like_repeats→ GAP1-N2+GAP1-M+GAP1-C*→??+DO-GTPase2.2→	820	Mycolicibacterium fortuitum	GCA_001673325.1	actinobacteria
WP_065063415.1	GAP1-N2+GAP1-M+GAP1-C	HRDC-like?+Fibronectin-like_repeats→ GAP1-N2+GAP1-M+GAP1-C*→??+DO-GTPase2.2→	819	Mycolicibacterium conceptionense	GCA_001673265.1	actinobacteria
WP_065063837.1	GAP1-N2+GAP1-M+GAP1-C	GrpE→ HSP70→ TPR_containing_alpha_helical+Fibronectin-like_repeats→ ZnR+ZnR+DO-GTPase2.2→ GAP1-N2+GAP1-M+GAP1-C*→ TM+TM+TM+TM+ZnR+ZnR+DO-GTPase2.2→ HSP70→ vWA(canonical)→	802	Mycolicibacterium conceptionense	GCA_001673265.1	actinobacteria
WP_065069046.1	GAP1-N2+GAP1-M+GAP1-C	HRDC-like?+Fibronectin-like_repeats→ GAP1-N2+GAP1-M+GAP1-C*→??+DO-GTPase2.2→	820	Mycolicibacterium fortuitum	GCA_001672845.1	actinobacteria
WP_065069842.1	GAP1-N2+GAP1-M+GAP1-C	polar_leader+TM+TM+TM→ TM+vWA(canonical)+Fibronectin-like_repeats+TM+beta-rich+lc_tail→ Tubulin+alpha_helical+??+??→ GAP_unknown1+TM+TM+GAP_unknown2→ HRDC-like?+Fibronectin-like_repeats→ GAP1-N2+GAP1-M+GAP1-C*→ beta-rich+vWA(canonical)→??+DO-GTPase2.2→ TM+DUF4190+TM+PknH_C→ TM+DUF4190+TM+PknH_C→	828	Mycolicibacterium fortuitum	GCA_001672845.1	actinobacteria
WP_065070980.1	GAP1-N2+GAP1-M+GAP1-C	polar_leader+TM+TM+TM→ TM+vWA(canonical)+Fibronectin-like_repeats+TM+beta-rich+lc_tail→ Tubulin+alpha_helical+??+??→ GAP_unknown1+TM+TM+GAP_unknown2→ HRDC-like?+Fibronectin-like_repeats→ GAP1-N2+GAP1-M+GAP1-C*→ beta-rich+vWA(canonical)→??+DO-GTPase2.2→ TM+DUF4190+TM+PknH_C→	828	Mycolicibacterium fortuitum	GCA_001673275.1	actinobacteria
WP_065071663.1	GAP1-N2+GAP1-M+GAP1-C	HRDC-like?+Fibronectin-like_repeats→ GAP1-N2+GAP1-M+GAP1-C*→??+DO-GTPase2.2→	820	Mycolicibacterium fortuitum	GCA_001673275.1	actinobacteria
WP_065077085.1	GAP1-N2+GAP1-M+GAP1-C	Fibronectin-like_repeats+??→ ZnR+ZnR+DO-GTPase2.2→ GAP1-N2+GAP1-M+GAP1-C*→ TM+vWA(canonical)+YtKA-like+TM+??→ Tubulin+coiled_coil+??→ Tubulin+coiled_coil+HTH?+??→ TM+TM+TM+vWA(canonical)+TM+TM+TM+polar_tail→ Fibronectin-like_repeats+??→	868	Clostridium ragsdalei	GCA_001675165.1	firmicutes
WP_065077095.1	GAP1-N2+GAP1-M	TM+TM+TM+vWA(canonical)+TM+TM+TM+polar_tail→ Fibronectin-like_repeats+??→?→?→?→ ZnR+ZnR+DO-GTPase2.2+ZnR+ZnR→ GAP1-N2+GAP1-M*→	351	Clostridium ragsdalei	GCA_001675165.1	firmicutes
WP_065147874.1	GAP1-N2+GAP1-M+GAP1-C	HRDC-like?+Fibronectin-like_repeats→ GAP1-N2+GAP1-M+GAP1-C*→??+DO-GTPase2.2→	820	Mycolicibacterium fortuitum	GCA_001673485.1	actinobacteria
WP_065148828.1	GAP1-N2+GAP1-M+GAP1-C	GAP_unknown1+TM+TM+GAP_unknown2→ HRDC-like?+Fibronectin-like_repeats→ GAP1-N2+GAP1-M+GAP1-C*→ beta-rich+vWA(canonical)→??+DO-GTPase2.2→ TM+DUF4190+TM+PknH_C→	828	Mycolicibacterium fortuitum	GCA_001673485.1	actinobacteria
WP_065294008.1	GAP1-N2+GAP1-M+GAP1-C+polar_tail+TM+beta-rich	TM+vWA(canonical)+YtKA-like+TM+??→ Tubulin+coiled_coil+??→ Tubulin+coiled_coil+HTH?+??→ TM+TM+TM+vWA(canonical)+TM+TM+TM+polar_tail→ Fibronectin-like_repeats+??→ PSE→ GAP1-N2+GAP1-M+GAP1-C+polar_tail+TM+beta-rich*→	976	Paenibacillus sp. KS1	GCA_001680695.1	firmicutes
WP_065361675.1	GAP1-N2+GAP1-M+GAP1-C	polar_leader+TM+TM+TM→ TM+vWA(canonical)+Fibronectin-like_repeats+TM+beta-rich+lc_tail→ Tubulin+alpha_helical+??+??→ GAP_unknown1+TM+TM+GAP_unknown2→ HRDC-like?+Fibronectin-like_repeats→ GAP1-N2+GAP1-M+GAP1-C*→?→??+DO-GTPase2.2→	933	Actinomyces oris	GCA_001682715.1	actinobacteria
WP_065383863.1	GAP1-N2+GAP1-M+GAP1-C+polar_tail+TM+beta-rich	TM+vWA(canonical)+YtKA-like+TM+??→ Tubulin+coiled_coil+??→ Tubulin+coiled_coil+HTH?+??→ TM+TM+TM+vWA(canonical)+TM+TM+TM+polar_tail→ Fibronectin-like_repeats+??→ ZnR+ZnR+DO-GTPase2.2+ZnR+ZnR→ GAP1-N2+GAP1-M+GAP1-C+polar_tail+TM+beta-rich*→	985	Bacillus sp. FJAT-26390	GCA_001682865.1	firmicutes
WP_065458973.1	GAP1-N2+GAP1-M+GAP1-C	HRDC-like?+Fibronectin-like_repeats→ GAP1-N2+GAP1-M+GAP1-C*→??+DO-GTPase2.2→	819	Mycolicibacterium vulneris	GCA_001686835.1	actinobacteria
WP_065509700.1	GAP1-N2+GAP1-M+GAP1-C	HRDC-like?+Fibronectin-like_repeats→ GAP1-N2+GAP1-M+GAP1-C*→??+DO-GTPase2.2→	819	Mycolicibacterium vulneris	GCA_001686755.1	actinobacteria
WP_065513488.1	GAP1-N2+GAP1-M+GAP1-C	HRDC-like?+Fibronectin-like_repeats→ GAP1-N2+GAP1-M+GAP1-C*→??+DO-GTPase2.2→	819	Mycolicibacterium vulneris	GCA_001686675.1	actinobacteria
WP_065847559.1	GAP1-N2+GAP1-M+GAP1-C	GAP1-N2+GAP1-M+GAP1-C*→	804	Streptomyces mutomycini	GCF_001700505.1	actinobacteria
WP_065919640.1	GAP1-N2+GAP1-M+GAP1-C	GrpE→ HSP70→ TPR_containing_alpha_helical+Fibronectin-like_repeats→ TPR_containing_alpha_helical+Fibronectin-like_repeats→ ZnR+ZnR+DO-GTPase2.2→ GAP1-N2+GAP1-M+GAP1-C*→?→ TM+TM+TM+TM+ZnR+ZnR+DO-GTPase2.2→ HSP70→?→?→?→||←PSE←unknown+CASPASE	385	Lentzea guizhouensis	GCA_001701025.1	actinobacteria
WP_066065304.1	GAP1-N2+GAP1-M+GAP1-C+polar_tail+TM+beta-rich	TM+vWA(canonical)+YtKA-like+TM+??→ Tubulin+coiled_coil+??→||←?||Tubulin+coiled_coil+HTH?+??→ TM+TM+TM+vWA(canonical)+TM+TM+TM+polar_tail→ Fibronectin-like_repeats+??→ ZnR+ZnR+DO-GTPase2.2+ZnR+ZnR→ GAP1-N2+GAP1-M+GAP1-C+polar_tail+TM+beta-rich*→	953	Bacillus soli	GCF_001591665.1	firmicutes
WP_066091978.1	GAP1-N2+GAP1-M+GAP1-C+polar_tail+TM+beta-rich	TM+vWA(canonical)+YtKA-like+TM+??→ Tubulin+coiled_coil+??→ Tubulin+coiled_coil+HTH?+??→ TM+TM+TM+vWA(canonical)+TM+TM+TM+polar_tail→ Fibronectin-like_repeats+??→ ZnR+ZnR+DO-GTPase2.2+ZnR+ZnR→ GAP1-N2+GAP1-M+GAP1-C+polar_tail+TM+beta-rich*→	954	Bacillus novalis	GCF_001591805.1	firmicutes
WP_066199613.1	GAP1-N2+GAP1-M+GAP1-C+polar_tail+TM+beta-rich	TM+vWA(canonical)+YtKA-like+TM+??→ Tubulin+coiled_coil+??→ Tubulin+coiled_coil+HTH?+??→ TM+TM+TM+vWA(canonical)+TM+TM+TM+polar_tail→ Fibronectin-like_repeats+??→ ZnR+ZnR+DO-GTPase2.2+ZnR+ZnR→ GAP1-N2+GAP1-M+GAP1-C+polar_tail+TM+beta-rich*→	726	Bacillus sp. FJAT-27225	GCA_001685025.1	firmicutes
WP_066315334.1	GAP1-N2+GAP1-M+GAP1-C+polar_tail+TM+beta-rich	TM+vWA(canonical)+YtKA-like+TM+??→ Tubulin+coiled_coil+??→ Tubulin+coiled_coil+HTH?+??→ TM+TM+TM+vWA(canonical)+TM+TM+TM+polar_tail→ Fibronectin-like_repeats+??→ ZnR+ZnR+DO-GTPase2.2+ZnR+ZnR→ GAP1-N2+GAP1-M+GAP1-C+polar_tail+TM+beta-rich*→	942	Bacillus sp. FJAT-29814	GCF_001510715.1	firmicutes
WP_066362451.1	GAP1-N2+GAP1-M+GAP1-C	GrpE→ HSP70→ TPR_containing_alpha_helical+Fibronectin-like_repeats→ ZnR+ZnR+DO-GTPase2.2→ GAP1-N2+GAP1-M+GAP1-C*→ TM+TM+TM+TM+ZnR+ZnR+DO-GTPase2.2+TM→	717	Herbidospora mongoliensis	GCF_001570545.1	actinobacteria
WP_066375919.1	GAP1-N2+GAP1-M+GAP1-C+TM+low_complexity+TM	vWA(canonical)→ DO-GTPase1→ GAP1-N2+GAP1-M+GAP1-C+TM+low_complexity+TM*→	776	Herbidospora mongoliensis	GCF_001570545.1	actinobacteria
WP_066377148.1	GAP1-N2+GAP1-M+GAP1-C+polar_tail+TM+beta-rich	TM+vWA(canonical)+YtKA-like+TM+??→ Tubulin+coiled_coil+??→ Tubulin+coiled_coil+HTH?+??→ TM+TM+TM+vWA(canonical)+TM+TM+TM+polar_tail→ Fibronectin-like_repeats+??→ ZnR+ZnR+DO-GTPase2.2+ZnR+ZnR→ GAP1-N2+GAP1-M+GAP1-C+polar_tail+TM+beta-rich*→	1010	Bacillus sp. FJAT-27264	GCA_001682855.1	firmicutes
WP_066829668.1	GAP1-N2+GAP1-M+GAP1-C	HRDC-like?+Fibronectin-like_repeats→ GAP1-N2+GAP1-M+GAP1-C*→??+DO-GTPase2.2→	825	Mycobacterium sp. 852013-51886_SCH5428379	GCA_001665575.1	actinobacteria
WP_066869695.1	GAP1-N2+GAP1-M+GAP1-C	GAP_unknown1+TM+TM+GAP_unknown2→ HRDC-like?+Fibronectin-like_repeats→ GAP1-N2+GAP1-M+GAP1-C*→ beta-rich+vWA(canonical)→??+DO-GTPase2.2→ TM+DUF4190+TM+PknH_C→ TM+DUF4190+TM+PknH_C→ TM+DUF4190+TM+PknH_C→ TM+DUF4190+TM+PknH_C→ TM+DUF4190+TM+PknH_C→	829	Mycobacterium sp. 1482292.6	GCA_001672995.1	actinobacteria
WP_066876398.1	GAP1-N2+GAP1-M+GAP1-C	GAP_unknown1+TM+TM+GAP_unknown2→ HRDC-like?+Fibronectin-like_repeats→ GAP1-N2+GAP1-M+GAP1-C*→ beta-rich+vWA(canonical)→??+DO-GTPase2.2→ TM+DUF4190+TM+PknH_C→ TM+DUF4190+TM→	829	Mycobacterium sp. 1245801.1	GCA_001673055.1	actinobacteria
WP_066944787.1	GAP1-N2+GAP1-M+GAP1-C	GAP1-N2+GAP1-M+GAP1-C*→	841	Streptomyces lushanensis	GCF_001700515.1	actinobacteria
WP_066997562.1	GAP1-N2+GAP1-M+GAP1-C	TPR_containing_alpha_helical+Fibronectin-like_repeats→ ZnR+ZnR+DO-GTPase2.2→ GAP1-N2+GAP1-M+GAP1-C*→ TM+TM+TM+TM+ZnR+ZnR+DO-GTPase2.2+TM→ vWA(canonical)→ PP2C→ Pkinase+ZnR+beta-rich→	892	Streptomyces cellostaticus	GCA_001513965.1	actinobacteria
WP_067021906.1	GAP1-N2+GAP1-M+GAP1-C	GAP_unknown1+TM+TM+GAP_unknown2→ HRDC-like?+Fibronectin-like_repeats→ GAP1-N2+GAP1-M+GAP1-C*→ beta-rich+vWA(canonical)→??+DO-GTPase2.2→ TM+DUF4190+TM+PknH_C→ TM+DUF4190+TM+PknH_C→ TM+DUF4190+TM+PknH_C→ PSE→?→ TM+DUF4190+TM+PknH_C→	829	Mycobacterium	GCA_001673415.1	actinobacteria
WP_067040969.1	GAP1-N2+GAP1-M+GAP1-C	GrpE→ HSP70→||←PSE||ZnR+ZnR+DO-GTPase2.2→ GAP1-N2+GAP1-M+GAP1-C*→ TM+TM+TM+TM+ZnR+ZnR+DO-GTPase2.2+Jelly_roll+beta-sandwich→||←STAND_NTPase+TM+TM+TM+TM+TM+TM	478	Streptomyces sp. FXJ1.172	GCA_001636945.1	actinobacteria
WP_067095811.1	GAP1-N2+GAP1-M+GAP1-C	TM+DUF4190+TM→ TM+DUF4190+TM→ polar_leader+TM+TM+TM→ TM+vWA(canonical)+Fibronectin-like_repeats+TM+beta-rich+lc_tail→ Tubulin+alpha_helical+??+??→ GAP_unknown1+TM+TM+GAP_unknown2→ HRDC-like?+Fibronectin-like_repeats→ GAP1-N2+GAP1-M+GAP1-C*→ beta-rich+vWA(canonical)→??+DO-GTPase2.2→ TM+DUF4190+TM+PknH_C→ TM+DUF4190+TM+PknH_C→ TM+DUF4190+TM+PknH_C→ TM+DUF4190+TM+PknH_C→	829	Mycobacterium sp. 852002-40037_SCH5390672	GCA_001665875.1	actinobacteria

acc	architecture	operon	len	species	gca	taxend
WP_067124860.1	GAP1-N2+GAP1-M+GAP1-C	TM+DUF4190+TM→ TM+DUF4190+TM→ polar_leader+TM+TM+TM→ TM+vWA(canonical)+Fibronectin-like_repeats+TM+beta-rich+lc_tail→ Tubulin+alpha_helical+??+??→ GAP_unknown1+TM+TM+GAP_unknown2→ HRDC-like?+Fibronectin-like_repeats→ GAP1-N2+GAP1-M+GAP1-C*→ beta-rich+vWA(canonical)→??+DO-GTPase2.2→ TM+DUF4190+TM+PknH_C→ TM+DUF4190+TM+PknH_C→ TM+DUF4190+TM+PknH_C→	829	Mycobacterium sp. 852002-51971_SCH5477799-a	GCA_001667145.1	actinobacteria
WP_067142841.1	GAP1-N2+GAP1-M+GAP1-C	GAP1-N2+GAP1-M+GAP1-C*→ beta-rich+vWA(canonical)→??+DO-GTPase2.2→ TM+DUF4190+TM+PknH_C→	829	Mycobacterium sp. 1245852.3	GCA_001669335.1	actinobacteria
WP_067348783.1	GAP1-N2+GAP1-M+GAP1-C	polar_leader+TM+TM+TM→ TM+vWA(canonical)+Fibronectin-like_repeats+TM+beta-rich+lc_tail→ Tubulin+alpha_helical+??+??→ GAP_unknown1+TM+TM+GAP_unknown2→ HRDC-like?+Fibronectin-like_repeats→ GAP1-N2+GAP1-M+GAP1-C*→ beta-rich+vWA(canonical)→??+DO-GTPase2.2→ TM+DUF4190+TM+PknH_C→ TM+DUF4190+TM+PknH_C→ TM+DUF4190+TM+PknH_C→	829	Mycobacterium sp. 1165178.9	GCA_001673555.1	actinobacteria
WP_067416587.1	GAP1-N2+GAP1-M+GAP1-C	GAP1-N2+GAP1-M+GAP1-C*→	789	Streptomyces sampsonii	GCA_004195765.1	actinobacteria
WP_067457622.1	GAP1-N2+GAP1-M+GAP1-C	GrpE→ HSP70→ TPR_containing_alpha_helical+Fibronectin-like_repeats→ ZnR+ZnR+DO-GTPase2.2→ GAP1-N2+GAP1-M+GAP1-C*→ TM+TM+TM+TM+ZnR+ZnR+DO-GTPase2.2+TM→	675	Actinomadura macra	GCF_001552215.1	actinobacteria
WP_067491281.1	GAP1-N2+GAP1-M+GAP1-C	GrpE→ HSP70→ TPR_containing_alpha_helical+Fibronectin-like_repeats→ ZnR+ZnR+DO-GTPase2.2→ GAP1-N2+GAP1-M+GAP1-C*→ TM+TM+TM+TM+ZnR+ZnR+DO-GTPase2.2+TM→	795	Actinomadura hibisca	GCF_001552635.1	actinobacteria
WP_067535570.1	GAP1-N2+GAP1-M+GAP1-C	Pkinase+TM→ ZnR+DO-GTPase2.1→ GAP1-N2+GAP1-M+GAP1-C*→	738	Nocardia crassostreae	GCF_001613405.1	actinobacteria
WP_067574519.1	GAP1-N2+GAP1-M+GAP1-C	Pkinase+TM→ ZnR+DO-GTPase2.1→ GAP1-N2+GAP1-M+GAP1-C*→	735	Nocardia acidivorans	GCF_001625085.1	actinobacteria
WP_067597429.1	GAP1-N2+GAP1-M+GAP1-C	TPR_containing_alpha_helical+Fibronectin-like_repeats→ ZnR+ZnR+DO-GTPase2.2→ GAP1-N2+GAP1-M+GAP1-C*→ TM+TM+TM+TM+ZnR+ZnR+DO-GTPase2.2→	653	Nocardiopsis listeri	GCF_001570765.1	actinobacteria
WP_067635518.1	GAP1-N2+GAP1-M+GAP1-C	GrpE→ HSP70→ TPR_containing_alpha_helical+Fibronectin-like_repeats→ ZnR+ZnR+DO-GTPase2.2→ GAP1-N2+GAP1-M+GAP1-C*→ TM+TM+TM+TM+ZnR+ZnR+DO-GTPase2.2+TM→	665	Actinomadura latina	GCF_001552195.1	actinobacteria
WP_067654758.1	GAP1-N2+GAP1-M+GAP1-C	GrpE→ HSP70→ TPR_containing_alpha_helical+Fibronectin-like_repeats→ ZnR+ZnR+DO-GTPase2.2→ GAP1-N2+GAP1-M+GAP1-C*→ TM+TM+TM+TM+ZnR+ZnR+DO-GTPase2.2→?→ HSP70→ vWA(canonical)→	809	Nocardia harenae	GCF_001612885.1	actinobacteria
WP_067687461.1	GAP1-N2+GAP1-M+GAP1-C	Pkinase+TM→ ZnR+DO-GTPase2.1→ GAP1-N2+GAP1-M+GAP1-C*→	739	Nocardia jejuensis	GCF_001613145.1	actinobacteria
WP_067772887.1	GAP1-N2+GAP1-M+GAP1-C	GrpE→ HSP70→ TPR_containing_alpha_helical+Fibronectin-like_repeats→ ZnR+ZnR+DO-GTPase2.2→ GAP1-N2+GAP1-M+GAP1-C*→ TM+TM+TM+TM+ZnR+ZnR+DO-GTPase2.2→ HSP70→ vWA(canonical)→	802	Mycobacterium sp. E802	GCA_001666875.1	actinobacteria
WP_067774504.1	GAP1-N2+GAP1-M+GAP1-C	HRDC-like?+Fibronectin-like_repeats→ GAP1-N2+GAP1-M+GAP1-C*→??+DO-GTPase2.2→	816	Mycobacterium sp. E802	GCA_001666875.1	actinobacteria
WP_067777628.1	GAP1-N2+GAP1-M+GAP1-C	FHA-like?+ZnR+ZnR+DO-GTPase2.2→ GAP1-N2+GAP1-M+GAP1-C*→	807	Akkermansia glycaniphila	GCA_001683795.1	verrucomicrobia
WP_067800015.1	GAP1-N2+GAP1-M+GAP1-C	GrpE→ HSP70→ TPR_containing_alpha_helical+Fibronectin-like_repeats→ ZnR+ZnR+DO-GTPase2.2→ GAP1-N2+GAP1-M+GAP1-C*→ TM+TM+TM+TM+ZnR+ZnR+DO-GTPase2.2+TM→	666	Actinomadura formosensis	GCF_001552155.1	actinobacteria
WP_067803927.1	GAP1-N2+GAP1-M+GAP1-C	TM+DUF4190+TM→ TM+DUF4190+TM→ polar_leader+TM+TM+TM→ PSE→ Tubulin+alpha_helical+??+??→ GAP_unknown1+TM+TM+GAP_unknown2→ HRDC-like?+Fibronectin-like_repeats→ GAP1-N2+GAP1-M+GAP1-C*→ beta-rich+vWA(canonical)→??+DO-GTPase2.2→?→ TM+DUF4190+TM+PknH_C→	807	Mycobacterium sp. ACS1612	GCA_001667505.1	actinobacteria
WP_067811217.1	GAP1-N2+GAP1-M+GAP1-C	GrpE→ HSP70→ TPR_containing_alpha_helical+Fibronectin-like_repeats→ ZnR+ZnR+DO-GTPase2.2→ GAP1-N2+GAP1-M+GAP1-C*→ TM+TM+TM+TM+ZnR+ZnR+DO-GTPase2.2+TM→	818	Actinomadura kijaniata	GCF_001552175.1	actinobacteria
WP_067834668.1	GAP1-N2+GAP1-M+GAP1-C	TM+DUF4190+TM→ TM+DUF4190+TM→ polar_leader+TM+TM+TM→ TM+vWA(canonical)+Fibronectin-like_repeats+TM+beta-rich+lc_tail→ Tubulin+alpha_helical+??+??→ GAP_unknown1+TM+TM+GAP_unknown2→ HRDC-like?+Fibronectin-like_repeats→ GAP1-N2+GAP1-M+GAP1-C*→ beta-rich+vWA(canonical)→??+DO-GTPase2.2→ TM+DUF4190+TM+PknH_C→ TM+DUF4190+TM+PknH_C→ TM+DUF4190+TM+PknH_C→ TM+DUF4190+TM+PknH_C→	829	Mycobacterium sp. E3078	GCA_001667835.1	actinobacteria
WP_067849806.1	GAP1-N2+GAP1-M+GAP1-C	HRDC-like?+Fibronectin-like_repeats→ GAP1-N2+GAP1-M+GAP1-C*→??+DO-GTPase2.2→	820	Mycolicibacterium wolinskyi	GCA_001545135.1	actinobacteria
WP_067890081.1	GAP1-N2+GAP1-M+GAP1-C	GrpE→ HSP70→ TPR_containing_alpha_helical+Fibronectin-like_repeats→ ZnR+ZnR+DO-GTPase2.2→ GAP1-N2+GAP1-M+GAP1-C*→ TM+TM+TM+TM+ZnR+ZnR+DO-GTPase2.2+TM→	738	Actinomadura chibensis	GCF_001552135.1	actinobacteria
WP_067901346.1	GAP1-N2+GAP1-M+GAP1-C+TM	vWA(canonical)→ DO-GTPase1→ GAP1-N2+GAP1-M+GAP1-C+TM*→	683	Actinomadura chibensis	GCF_001552135.1	actinobacteria
WP_067912468.1	GAP1-N2+GAP1-M+GAP1-C	TM+DUF4190+TM→ TM+DUF4190+TM→ polar_leader+TM+TM+TM→ TM+vWA(canonical)+Fibronectin-like_repeats+TM+beta-rich+lc_tail→ Tubulin+alpha_helical+??+??→ GAP_unknown1+TM+TM+GAP_unknown2→ HRDC-like?+Fibronectin-like_repeats→ GAP1-N2+GAP1-M+GAP1-C*→ beta-rich+vWA(canonical)→??+DO-GTPase2.2→ TM+DUF4190+TM+PknH_C→ TM+DUF4190+TM+PknH_C→ TM+DUF4190+TM+PknH_C→ TM+DUF4190+TM+PknH_C→	829	Mycobacterium	GCA_001667865.1	actinobacteria
WP_067913448.1	GAP1-N2+GAP1-M+GAP1-C	GrpE→ HSP70→ TPR_containing_alpha_helical+Fibronectin-like_repeats→ ZnR+ZnR+DO-GTPase2.2→ GAP1-N2+GAP1-M+GAP1-C*→ TM+TM+TM+TM+ZnR+ZnR+DO-GTPase2.2→?→ unknown+CASPASE→ HSP70→	796	Actinomadura	GCA_003001795.1	actinobacteria
WP_067914006.1	GAP1-N2+GAP1-M+GAP1-C	HSP70→ TPR_containing_alpha_helical+Fibronectin-like_repeats→ ZnR+ZnR+DO-GTPase2.2→ GAP1-N2+GAP1-M+GAP1-C*→ TM+TM+TM+TM+ZnR+ZnR+DO-GTPase2.2+TM→	745	Actinomadura	GCA_003001795.1	actinobacteria
WP_068075338.1	GAP1-N2+GAP1-M+GAP1-C	Tubulin+alpha_helical+??+??→ GAP_unknown1+TM+TM+GAP_unknown2→ HRDC-like?+Fibronectin-like_repeats→ GAP1-N2+GAP1-M+GAP1-C*→ beta-rich+vWA(canonical)→	831	Mycobacterium	GCA_001666755.1	actinobacteria
WP_068133906.1	GAP1-N2+GAP1-M	FGS-fold_domain→ ZnR+DO-GTPase2.1→ GAP1-N2+GAP1-M*→	259	Roseimaritima ulvae	GCF_001642915.1	planctomycetes
WP_068245530.1	GAP1-N2+GAP1-M+GAP1-C	Tubulin+alpha_helical+??+??→ GAP_unknown1+TM+TM+GAP_unknown2→ HRDC-like?+Fibronectin-like_repeats→ GAP1-N2+GAP1-M+GAP1-C*→ beta-rich+vWA(canonical)→??+DO-GTPase2.2→ TM+DUF4190+TM+PknH_C→	841	Mycobacterium sp. ACS4331	GCA_001667265.1	actinobacteria
WP_068269433.1	GAP1-N2+GAP1-M+GAP1-C	TM+DUF4190+TM→ TM+DUF4190+TM→ polar_leader+TM+TM+TM→ TM+vWA(canonical)+Fibronectin-like_repeats+TM+beta-rich+lc_tail→ Tubulin+alpha_helical+??+??→ GAP_unknown1+TM+TM+GAP_unknown2→ HRDC-like?+Fibronectin-like_repeats→ GAP1-N2+GAP1-M+GAP1-C*→ beta-rich+vWA(canonical)→??+DO-GTPase2.2→ TM+DUF4190+TM+PknH_C→ TM+DUF4190+TM+PknH_C→ TM+DUF4190+TM+PknH_C→ TM+DUF4190+TM+PknH_C→	829	Mycobacterium sp. E2660	GCA_001667995.1	actinobacteria
WP_068287942.1	GAP1-N2+GAP1-M+GAP1-C	Tubulin+alpha_helical+??+??→ GAP_unknown1+TM+TM+GAP_unknown2→ HRDC-like?+Fibronectin-like_repeats→ GAP1-N2+GAP1-M+GAP1-C*→ beta-rich+vWA(canonical)→??+DO-GTPase2.2→ TM+DUF4190+TM+PknH_C→ TM+DUF4190+TM+PknH_C→ TM+DUF4190+TM+PknH_C→ TM+DUF4190+TM+PknH_C→	831	Mycobacterium sp. E2462	GCA_001672685.1	actinobacteria
WP_068420276.1	GAP1-N2+GAP1-M	GAP1-N2+GAP1-M*→	229	Planctomyces sp. SH-PL62	GCA_001610895.1	planctomycetes
WP_068496114.1	GAP1-N2+GAP1-M+GAP1-C+polar_tail+TM+beta-rich	TM+vWA(canonical)+YtKA-like+TM+??→ Tubulin+coiled_coil+??→ Tubulin+coiled_coil+HTH?+??→ TM+TM+TM+vWA(canonical)+TM+TM+TM+polar_tail→ Fibronectin-like_repeats+??→ ZnR+ZnR+DO-GTPase2.2+ZnR+ZnR→ GAP1-N2+GAP1-M+GAP1-C+polar_tail+TM+beta-rich*→	1001	Paenibacillus kribbensis	GCF_001705305.1	firmicutes
WP_068528494.1	GAP1-N2+GAP1-M+GAP1-C+polar_tail	Tubulin+coiled_coil+HTH?+??→ TM+TM+TM+vWA+TM+TM+TM+polar_tail→ Fibronectin-like_repeats+??→?→ ZnR+ZnR+DO-GTPase2.2→ GAP1-N2+GAP1-M+GAP1-C+polar_tail*→	1052	Paenibacillus glacialis	GCA_001637205.1	firmicutes
WP_068592502.1	GAP1-N2+GAP1-M+GAP1-C+polar_tail+TM+beta-rich	TM+vWA(canonical)+YtKA-like+TM+??→ Tubulin+coiled_coil+??→ Tubulin+coiled_coil+HTH?+??→ TM+TM+TM+vWA(canonical)+TM+TM+TM+polar_tail→ Fibronectin-like_repeats+??→ ZnR+ZnR+DO-GTPase2.2+ZnR+ZnR→ GAP1-N2+GAP1-M+GAP1-C+polar_tail+TM+beta-rich*→	1046	Paenibacillus macquariensis	GCA_001637165.1	firmicutes
WP_068599101.1	GAP1-N2+GAP1-M+GAP1-C+polar_tail+TM+beta-rich	TM+vWA(canonical)+YtKA-like+TM+??→ Tubulin+coiled_coil+??→ Tubulin+coiled_coil+HTH?+??→ TM+TM+TM+vWA(canonical)+TM+TM+TM+polar_tail→ Fibronectin-like_repeats+??→ ZnR+ZnR+DO-GTPase2.2+ZnR+ZnR→ GAP1-N2+GAP1-M+GAP1-C+polar_tail+TM+beta-rich*→	1053	Paenibacillus macquariensis	GCA_001637215.1	firmicutes
WP_068613626.1	GAP1-N2+GAP1-M+GAP1-C+polar_tail	Fibronectin-like_repeats+??→ ZnR+ZnR+DO-GTPase2.2→ GAP1-N2+GAP1-M+GAP1-C+polar_tail*→	1085	Paenibacillus tuaregi	GCF_900086655.1	firmicutes
WP_068650918.1	GAP1-N2+GAP1-M+GAP1-C+polar_tail+TM+beta-rich	TM+vWA(canonical)+YtKA-like+TM+??→ Tubulin+coiled_coil+??→ Tubulin+coiled_coil+HTH?+??→ TM+TM+TM+vWA(canonical)+TM+TM+TM+polar_tail→ Fibronectin-like_repeats+??→ ZnR+ZnR+DO-GTPase2.2+ZnR+ZnR→ GAP1-N2+GAP1-M+GAP1-C+polar_tail+TM+beta-rich*→	1053	Paenibacillus antarcticus	GCA_001637225.1	firmicutes
WP_068660114.1	GAP1-N2+GAP1-M+GAP1-C+polar_tail+TM+beta-rich	TM+vWA(canonical)+YtKA-like+TM+??→ Tubulin+coiled_coil+??→ Tubulin+coiled_coil+HTH?+??→ TM+TM+TM+vWA(canonical)+TM+TM+TM+polar_tail→ Fibronectin-like_repeats+??→ ZnR+ZnR+DO-GTPase2.2+ZnR+ZnR→ GAP1-N2+GAP1-M+GAP1-C+polar_tail+TM+beta-rich*→	1045	Paenibacillus crassostreae	GCA_001857945.1	firmicutes
WP_068687038.1	GAP1-N2+GAP1-M+GAP1-C+polar_tail+TM+beta-rich	TM+vWA(canonical)+YtKA-like+TM+??→ Tubulin+coiled_coil+??→ Tubulin+coiled_coil+HTH?+??→ TM+TM+TM+vWA(canonical)+TM+TM+TM+polar_tail→ Fibronectin-like_repeats+??→ ZnR+ZnR+DO-GTPase2.2+ZnR+ZnR→ GAP1-N2+GAP1-M+GAP1-C+polar_tail+TM+beta-rich*→	915	Paenibacillus oryzae	GCA_001675045.1	firmicutes
WP_068723609.1	GAP1-N2+GAP1-M+GAP1-C+polar_tail+TM+beta-rich	TM+TM+TM+vWA(canonical)+TM+TM+TM+polar_tail→ Fibronectin-like_repeats+??→ ZnR+ZnR+DO-GTPase2.2+ZnR+ZnR→ GAP1-N2+GAP1-M+GAP1-C+polar_tail+TM+beta-rich*→	892	Paenibacillus sp. DMB5	GCA_001517085.1	firmicutes
WP_068779552.1	GAP1-N2+GAP1-M+GAP1-C+polar_tail+TM+beta-rich	TM+vWA(canonical)+YtKA-like+TM+??→ Tubulin+coiled_coil+??→ Tubulin+coiled_coil+HTH?+??→ TM+TM+TM+vWA(canonical)+TM+TM+TM+polar_tail→ Fibronectin-like_repeats+??→ ZnR+ZnR+DO-GTPase2.2+ZnR+ZnR→ GAP1-N2+GAP1-M+GAP1-C+polar_tail+TM+beta-rich*→	1143	Paenibacillus sp. GM2	GCF_900069005.1	firmicutes
WP_068792246.1	GAP1-N2+GAP1-M+GAP1-C+polar_tail+TM+beta-rich	TM+vWA(canonical)+YtKA-like+TM+??→ Tubulin+coiled_coil+??→ Tubulin+coiled_coil+HTH?+??→ TM+TM+TM+vWA(canonical)+TM+TM+TM+polar_tail→ Fibronectin-like_repeats+??→ ZnR+ZnR+DO-GTPase2.2+ZnR+ZnR→ GAP1-N2+GAP1-M+GAP1-C+polar_tail+TM+beta-rich*→	833	Brevibacillus laterosporus	GCF_001696705.1	firmicutes
WP_068940787.1	GAP1-N2+GAP1-M+GAP1-C+polar_tail+TM+beta-rich	TM+vWA(canonical)+YtKA-like+TM+??→ Tubulin+coiled_coil+??→ Tubulin+coiled_coil+HTH?+??→ TM+TM+TM+vWA(canonical)+TM+TM+TM+polar_tail→ Fibronectin-like_repeats+??→ ZnR+ZnR+DO-GTPase2.2+ZnR+ZnR→ GAP1-N2+GAP1-M+GAP1-C+polar_tail+TM+beta-rich*→	1002	Paenibacillus	GCA_001707685.1	firmicutes
WP_069079658.1	GAP1-N2+GAP1-M+GAP1-C+polar_tail+TM+beta-rich	TM+vWA(canonical)+YtKA-like+TM+??→ Tubulin+coiled_coil+??→ Tubulin+coiled_coil+HTH?+??→ TM+TM+TM+vWA(canonical)+TM+TM+TM+polar_tail→ Fibronectin-like_repeats+??→ ZnR+ZnR+DO-GTPase2.2+ZnR+ZnR→ GAP1-N2+GAP1-M+GAP1-C+polar_tail+TM+beta-rich*→	1002	Paenibacillus polymyxa	GCA_001709135.1	firmicutes
WP_069169430.1	GAP1-N2+GAP1-M+GAP1-C	GAP1-N2+GAP1-M+GAP1-C*→	803	Streptomyces griseus	GCF_001715295.1	actinobacteria
WP_069291808.1	GAP1-N2+GAP1-M+GAP1-C+polar_tail	Tubulin+coiled_coil+??→ Tubulin+coiled_coil+HTH?+??→ TM+TM+TM+vWA+TM+TM+TM+polar_tail→ Fibronectin-like_repeats+??→ ZnR+ZnR+DO-GTPase2.2→ GAP1-N2+GAP1-M+GAP1-C+polar_tail*→	1009	Paenibacillus polymyxa	GCA_001719045.1	firmicutes
WP_069329620.1	GAP1-N2+GAP1-M+GAP1-C+polar_tail+TM+beta-rich	TM+vWA(canonical)+YtKA-like+TM+??→ Tubulin+coiled_coil+??→ Tubulin+coiled_coil+HTH?+??→ TM+TM+TM+vWA(canonical)+TM+TM+TM+polar_tail→ Fibronectin-like_repeats+??→ ZnR+ZnR+DO-GTPase2.2+ZnR+ZnR→ GAP1-N2+GAP1-M+GAP1-C+polar_tail+TM+beta-rich*→	1077	Paenibacillus sp. TI45-13ar	GCA_001721045.1	firmicutes
WP_069389143.1	GAP1-N2+GAP1-M+GAP1-C	GrpE→ HSP70→ TPR_containing_alpha_helical+Fibronectin-like_repeats→ ZnR+ZnR+DO-GTPase2.2→ GAP1-N2+GAP1-M+GAP1-C*→ TM+TM+TM+TM+ZnR+ZnR+DO-GTPase2.2→?→ HSP70_domain_1→ HSP70→ vWA(canonical)→	806	Dietzia alimentaria	GCA_001722515.1	actinobacteria

acc	architecture	operon	len	species	gca	taxend
WP_069601145.1	GAP1-N2+GAP1-M+GAP1-C	TM+vWA(canonical)+Fibronectin-like_repeats+TM+beta-rich+lc_tail→ Tubulin+alpha_helical+??+??→ GAP_unknown1+TM+TM+GAP_unknown2→ HRDC-like?+Fibronectin-like_repeats→ GAP1-N2+GAP1-M+GAP1-C*→ beta-rich+vWA(canonical)→??+DO-GTPase2.2→	978	Brevibacterium linens	GCA_001729525.1	actinobacteria
WP_069630652.1	GAP1-N2+GAP1-M+GAP1-C	GAP1-N2+GAP1-M+GAP1-C*→ vWA(canonical)→ PP2C→ Pkinase+ZnR+beta-rich→||←TPR_containing_alpha_helical+Fibronectin-like_repeats	865	Streptomyces niveus	GCF_001723055.1	actinobacteria
WP_069756381.1	GAP1-N2+GAP1-M+GAP1-C	GAP1-N2+GAP1-M+GAP1-C*→	827	Streptomyces sp. EN16	GCF_001746285.1	actinobacteria
WP_069849891.1	GAP1-N2+GAP1-M+GAP1-C+polar_tail+TM+beta-rich	TM+vWA(canonical)+YtKA-like+TM+??→ Tubulin+coiled_coil+??→ Tubulin+coiled_coil+HTH?+??→ TM+TM+TM+vWA(canonical)+TM+TM+TM+polar_tail→ Fibronectin-like_repeats+??→ ZnR+ZnR+DO-GTPase2.2+ZnR+ZnR→ GAP1-N2+GAP1-M+GAP1-C+polar_tail+TM+beta-rich*→	832	Brevibacillus brevis	GCF_001748185.1	firmicutes
WP_069887175.1	GAP1-N2+GAP1-M+GAP1-C	GAP1-N2+GAP1-M+GAP1-C*→ vWA(canonical)→ PP2C→ Pkinase+ZnR+beta-rich→||←TPR_containing_alpha_helical+Fibronectin-like_repeats	803	Streptomyces luteocolor	GCF_001748305.1	actinobacteria
WP_069992599.1	GAP1-N2+GAP1-M+GAP1-C	PP2C→ Pkinase→?→?→?→ HSP70_domain_2+low_complexity+domain_betaX→ ZnR+ZnR+DO-GTPase2.2→ GAP1-N2+GAP1-M+GAP1-C*→ TM+TM+TM+TM+ZnR+ZnR+DO-GTPase2.2→	862	Streptomyces	GCA_001751375.1	actinobacteria
WP_070026458.1	GAP1-N2+GAP1-M+GAP1-C	PP2C→ Pkinase→?→?→?→ HSP70_domain_2+low_complexity+domain_betaX→ ZnR+ZnR+DO-GTPase2.2→ GAP1-N2+GAP1-M+GAP1-C*→ TM+TM+TM+TM+ZnR+ZnR+DO-GTPase2.2→	814	Streptomyces sp. F-1	GCA_900079405.3	actinobacteria
WP_070185344.1	GAP1-N2+GAP1-M+GAP1-C	HRDC-like?+Fibronectin-like_repeats→ GAP1-N2+GAP1-M+GAP1-C*→??+DO-GTPase2.2→	819	Mycolicibacterium sp. (ex Dasyatis americana)	GCA_001756795.1	actinobacteria
WP_070192257.1	GAP1-N2+GAP1-M+GAP1-C	TM+vWA(canonical)+Fibronectin-like_repeats+TM+beta-rich+lc_tail→ Tubulin+alpha_helical+??+??→ GAP_unknown1+TM+TM+GAP_unknown2→?→ HRDC-like?+Fibronectin-like_repeats→ GAP1-N2+GAP1-M+GAP1-C*→?→??+DO-GTPase2.2→	638	Humibacillus sp. DSM 29435	GCA_001758225.1	actinobacteria
WP_070204686.1	GAP1-N2+GAP1-M+GAP1-C	GAP1-N2+GAP1-M+GAP1-C*→	820	Streptomyces nanshensis	GCA_001751305.1	actinobacteria
WP_070420959.1	GAP1-N2+GAP1-M+GAP1-C	polar_leader+TM+TM+TM→ TM+vWA(canonical)+Fibronectin-like_repeats+TM+beta-rich+lc_tail→ Tubulin+alpha_helical+??+??→ GAP_unknown1+TM+TM+GAP_unknown2→ HRDC-like?+Fibronectin-like_repeats→ GAP1-N2+GAP1-M+GAP1-C*→ beta-rich+vWA(canonical)→??+DO-GTPase2.2→ polar_leader+TM+TM+TM→	535	Corynebacterium sp. HMSC06C06	GCA_001807295.1	actinobacteria
WP_070433390.1	GAP1-N2+GAP1-M+GAP1-C	polar_leader+TM+TM+TM→ TM+vWA(canonical)+Fibronectin-like_repeats+TM+beta-rich+lc_tail→ Tubulin+alpha_helical+??+??→ GAP_unknown1+TM+TM+GAP_unknown2→ HRDC-like?+Fibronectin-like_repeats→ GAP1-N2+GAP1-M+GAP1-C*→ beta-rich+vWA(canonical)→??+DO-GTPase2.2→ polar_leader+TM+TM+TM→	515	Corynebacterium sp. HMSC06D04	GCA_001807315.1	actinobacteria
WP_070444258.1	GAP1-N2+GAP1-M+GAP1-C	polar_leader+TM+TM+TM→ TM+vWA(canonical)+Fibronectin-like_repeats+TM+beta-rich+lc_tail→ Tubulin+alpha_helical+??+??→ GAP_unknown1+TM+TM+GAP_unknown2→ HRDC-like?+Fibronectin-like_repeats→ GAP1-N2+GAP1-M+GAP1-C*→ beta-rich+vWA(canonical)→??+DO-GTPase2.2→ polar_leader+TM+TM+TM→	565	Corynebacterium sp. HMSC05D03	GCA_001807205.1	actinobacteria
WP_070455659.1	GAP1-N2+GAP1-M+GAP1-C	polar_leader+TM+TM+TM→ TM+vWA(canonical)+Fibronectin-like_repeats+TM+beta-rich+lc_tail→ Tubulin+alpha_helical+??+??→ PSE→ HRDC-like?+Fibronectin-like_repeats→ GAP1-N2+GAP1-M+GAP1-C*→ beta-rich+vWA(canonical)→??+DO-GTPase2.2→ polar_leader+TM+TM+TM→	515	Corynebacterium	GCA_001810285.1	actinobacteria
WP_070494624.1	GAP1-N2+GAP1-M+GAP1-C	polar_leader+TM+TM+TM→ TM+vWA(canonical)+Fibronectin-like_repeats+TM+beta-rich+lc_tail→ Tubulin+alpha_helical+??+??→ GAP_unknown1+TM+TM+GAP_unknown2→ HRDC-like?+Fibronectin-like_repeats→ GAP1-N2+GAP1-M+GAP1-C*→?→??+DO-GTPase2.2→?→||←?←?←HSP70	326	Corynebacterium sp. HMSC30G07	GCA_001806945.1	actinobacteria
WP_070497539.1	GAP1-N2+GAP1-M+GAP1-C	polar_leader+TM+TM+TM→ TM+vWA(canonical)+Fibronectin-like_repeats+TM+beta-rich+lc_tail→ Tubulin+alpha_helical+??+??→ GAP_unknown1+TM+TM+GAP_unknown2→ HRDC-like?+Fibronectin-like_repeats→ GAP1-N2+GAP1-M+GAP1-C*→ beta-rich+vWA(canonical)→??+DO-GTPase2.2→ polar_leader+TM+TM+TM→	515	Corynebacterium sp. HMSC06G04	GCA_001807365.1	actinobacteria
WP_070508819.1	GAP1-N2+GAP1-M+GAP1-C	polar_leader+TM+TM+TM→ TM+vWA(canonical)+Fibronectin-like_repeats+TM+beta-rich+lc_tail→ Tubulin+alpha_helical+??+??→ GAP_unknown1+TM+TM+GAP_unknown2→ HRDC-like?+Fibronectin-like_repeats→ GAP1-N2+GAP1-M+GAP1-C*→ beta-rich+vWA(canonical)→??+DO-GTPase2.2→ polar_leader+TM+TM+TM→	535	Corynebacterium sp. HMSC05D08	GCA_001807215.1	actinobacteria
WP_070509672.1	GAP1-N2+GAP1-M+GAP1-C	polar_leader+TM+TM+TM→ TM+vWA(canonical)+Fibronectin-like_repeats+TM+beta-rich+lc_tail→ Tubulin+alpha_helical+??+??→ GAP_unknown1+TM+TM+GAP_unknown2→ HRDC-like?+Fibronectin-like_repeats→ GAP1-N2+GAP1-M+GAP1-C*→ beta-rich+vWA(canonical)→??+DO-GTPase2.2→ polar_leader+TM+TM+TM→	560	Corynebacteriaceae	GCA_001811545.1	actinobacteria
WP_070516323.1	GAP1-N2+GAP1-M+GAP1-C	polar_leader+TM+TM+TM→ TM+vWA(canonical)+Fibronectin-like_repeats+TM+beta-rich+lc_tail→ Tubulin+alpha_helical+??+??→ GAP_unknown1+TM+TM+GAP_unknown2→ HRDC-like?+Fibronectin-like_repeats→ GAP1-N2+GAP1-M+GAP1-C*→ beta-rich+vWA(canonical)→??+DO-GTPase2.2→ polar_leader+TM+TM+TM→	560	Turicella sp. HMSC076G08	GCA_001811515.1	actinobacteria
WP_070523249.1	GAP1-N2+GAP1-M+GAP1-C	polar_leader+TM+TM+TM→ TM+vWA(canonical)+Fibronectin-like_repeats+TM+beta-rich+lc_tail→ Tubulin+alpha_helical+??+??→ GAP_unknown1+TM+TM+GAP_unknown2→ HRDC-like?+Fibronectin-like_repeats→ GAP1-N2+GAP1-M+GAP1-C*→ beta-rich+vWA(canonical)→??+DO-GTPase2.2→ polar_leader+TM+TM+TM→	850	Corynebacterium sp. HMSC11E11	GCF_001807025.1	actinobacteria
WP_070524552.1	GAP1-N2+GAP1-M+GAP1-C	polar_leader+TM+TM+TM→ TM+vWA(canonical)+Fibronectin-like_repeats+TM+beta-rich+lc_tail→ Tubulin+alpha_helical+??+??→ GAP_unknown1+TM+TM+GAP_unknown2→ HRDC-like?+Fibronectin-like_repeats→ GAP1-N2+GAP1-M+GAP1-C*→ beta-rich+vWA(canonical)→??+DO-GTPase2.2→ polar_leader+TM+TM+TM→	565	Corynebacteriaceae	GCA_001811845.1	actinobacteria
WP_070525195.1	GAP1-N2+GAP1-M+GAP1-C	polar_leader+TM+TM+TM→ TM+vWA(canonical)+Fibronectin-like_repeats+TM+beta-rich+lc_tail→ Tubulin+alpha_helical+??+??→ GAP_unknown1+TM+TM+GAP_unknown2→ HRDC-like?+Fibronectin-like_repeats→ GAP1-N2+GAP1-M+GAP1-C*→ beta-rich+vWA(canonical)→??+DO-GTPase2.2→ polar_leader+TM+TM+TM→	567	Corynebacterium sp. HMSC074E01	GCA_001808885.1	actinobacteria
WP_070533827.1	GAP1-N2+GAP1-M+GAP1-C	polar_leader+TM+TM+TM→ TM+vWA(canonical)+Fibronectin-like_repeats+TM+beta-rich+lc_tail→ Tubulin+alpha_helical+??+??→ GAP_unknown1+TM+TM+GAP_unknown2→ HRDC-like?+Fibronectin-like_repeats→ GAP1-N2+GAP1-M+GAP1-C*→ beta-rich+vWA(canonical)→??+DO-GTPase2.2→ polar_leader+TM+TM+TM→	577	Corynebacteriaceae	GCA_001811255.1	actinobacteria
WP_070546917.1	GAP1-N2+GAP1-M+GAP1-C	polar_leader+TM+TM+TM→ TM+vWA(canonical)+Fibronectin-like_repeats+TM+beta-rich+lc_tail→ Tubulin+alpha_helical+??+??→ GAP_unknown1+TM+TM+GAP_unknown2→ HRDC-like?+Fibronectin-like_repeats→ GAP1-N2+GAP1-M+GAP1-C*→ beta-rich+vWA(canonical)→??+DO-GTPase2.2→ polar_leader+TM+TM+TM→	560	Corynebacterium sp. HMSC070E08	GCA_001808895.1	actinobacteria
WP_070562458.1	GAP1-N2+GAP1-M+GAP1-C	polar_leader+TM+TM+TM→ TM+vWA(canonical)+Fibronectin-like_repeats+TM+beta-rich+lc_tail→ Tubulin+alpha_helical+??+??→ GAP_unknown1+TM+TM+GAP_unknown2→ HRDC-like?+Fibronectin-like_repeats→ GAP1-N2+GAP1-M+GAP1-C*→ beta-rich+vWA(canonical)→??+DO-GTPase2.2→ polar_leader+TM+TM+TM→	577	Corynebacterium sp. HMSC072A02	GCA_001810085.1	actinobacteria
WP_070564251.1	GAP1-N2+GAP1-M+GAP1-C	polar_leader+TM+TM+TM→ TM+vWA(canonical)+Fibronectin-like_repeats+TM+beta-rich+lc_tail→ Tubulin+alpha_helical+??+??→ GAP_unknown1+TM+TM+GAP_unknown2→ HRDC-like?+Fibronectin-like_repeats→ GAP1-N2+GAP1-M+GAP1-C*→ beta-rich+vWA(canonical)→??+DO-GTPase2.2→ polar_leader+TM+TM+TM→	577	Corynebacterium	GCA_001811565.1	actinobacteria
WP_070662349.1	GAP1-N2+GAP1-M+GAP1-C	polar_leader+TM+TM+TM→ TM+vWA(canonical)+Fibronectin-like_repeats+TM+beta-rich+lc_tail→ Tubulin+alpha_helical+??+??→ GAP_unknown1+TM+TM+GAP_unknown2→ HRDC-like?+Fibronectin-like_repeats→ GAP1-N2+GAP1-M+GAP1-C*→?→??+DO-GTPase2.2→	933	Actinomyces	GCA_001812965.1	actinobacteria
WP_070671429.1	GAP1-N2+GAP1-M+GAP1-C	polar_leader+TM+TM+TM→ TM+vWA(canonical)+Fibronectin-like_repeats+TM+beta-rich+lc_tail→ Tubulin+alpha_helical+??+??→ GAP_unknown1+TM+TM+GAP_unknown2→ HRDC-like?+Fibronectin-like_repeats→ GAP1-N2+GAP1-M+GAP1-C*→ beta-rich+vWA(canonical)→??+DO-GTPase2.2→ polar_leader+TM+TM+TM→	559	Corynebacterium sp. HMSC078H07	GCA_001812805.1	actinobacteria
WP_070679262.1	GAP1-N2+GAP1-M+GAP1-C	polar_leader+TM+TM+TM→ TM+vWA(canonical)+Fibronectin-like_repeats+TM+beta-rich+lc_tail→ Tubulin+alpha_helical+??+??→ GAP_unknown1+TM+TM+GAP_unknown2→ HRDC-like?+Fibronectin-like_repeats→ GAP1-N2+GAP1-M+GAP1-C*→ beta-rich+vWA(canonical)→??+DO-GTPase2.2→ polar_leader+TM+TM+TM→	560	Turicella sp. HMSC034H07	GCA_001815175.1	actinobacteria
WP_070683138.1	GAP1-N2+GAP1-M+GAP1-C	polar_leader+TM+TM+TM→ TM+vWA(canonical)+Fibronectin-like_repeats+TM+beta-rich+lc_tail→ Tubulin+alpha_helical+??+??→ GAP_unknown1+TM+TM+GAP_unknown2→ HRDC-like?+Fibronectin-like_repeats→ GAP1-N2+GAP1-M+GAP1-C*→ beta-rich+vWA(canonical)→??+DO-GTPase2.2→ polar_leader+TM+TM+TM→	577	Corynebacterium sp. HMSC056E09	GCA_001813425.1	actinobacteria
WP_070685436.1	GAP1-N2+GAP1-M+GAP1-C	polar_leader+TM+TM+TM→ TM+vWA(canonical)+Fibronectin-like_repeats+TM+beta-rich+lc_tail→ Tubulin+alpha_helical+??+??→ GAP_unknown1+TM+TM+GAP_unknown2→ HRDC-like?+Fibronectin-like_repeats→ GAP1-N2+GAP1-M+GAP1-C*→ beta-rich+vWA(canonical)→??+DO-GTPase2.2→ polar_leader+TM+TM+TM→	577	Turicella sp. HMSC059E07	GCA_001814395.1	actinobacteria
WP_070701282.1	GAP1-N2+GAP1-M+GAP1-C	polar_leader+TM+TM+TM→ TM+vWA(canonical)+Fibronectin-like_repeats+TM+beta-rich+lc_tail→ Tubulin+alpha_helical+??+??→ GAP_unknown1+TM+TM+GAP_unknown2→ HRDC-like?+Fibronectin-like_repeats→ GAP1-N2+GAP1-M+GAP1-C*→ beta-rich+vWA(canonical)→??+DO-GTPase2.2→ polar_leader+TM+TM+TM→	564	Corynebacterium sp. HMSC068G04	GCA_001814885.1	actinobacteria
WP_070731489.1	GAP1-N2+GAP1-M+GAP1-C	polar_leader+TM+TM+TM→ TM+vWA(canonical)+Fibronectin-like_repeats+TM+beta-rich+lc_tail→ Tubulin+alpha_helical+??+??→ GAP_unknown1+TM+TM+GAP_unknown2→ HRDC-like?+Fibronectin-like_repeats→ GAP1-N2+GAP1-M+GAP1-C*→ beta-rich+vWA(canonical)→??+DO-GTPase2.2→ polar_leader+TM+TM+TM→	565	Corynebacterium sp. HMSC066C02	GCA_001814945.1	actinobacteria
WP_070731802.1	GAP1-N2+GAP1-M+GAP1-C	polar_leader+TM+TM+TM→ PSE→ PSE→ Tubulin+alpha_helical+??+??→ GAP_unknown1+TM+TM+GAP_unknown2→ HRDC-like?+Fibronectin-like_repeats→ GAP1-N2+GAP1-M+GAP1-C*→ beta-rich+vWA(canonical)→??+DO-GTPase2.2→ polar_leader+TM+TM+TM→	515	Corynebacterium sp. HMSC076D02	GCA_001813815.1	actinobacteria
WP_070818055.1	GAP1-N2+GAP1-M+GAP1-C	polar_leader+TM+TM+TM→ TM+vWA(canonical)+Fibronectin-like_repeats+TM+beta-rich+lc_tail→ Tubulin+alpha_helical+??+??→ GAP_unknown1+TM+TM+GAP_unknown2→ HRDC-like?+Fibronectin-like_repeats→ GAP1-N2+GAP1-M+GAP1-C*→ beta-rich+vWA(canonical)→??+DO-GTPase2.2→ polar_leader+TM+TM+TM→	567	Corynebacterium sp. HMSC036D02	GCA_001836165.1	actinobacteria
WP_070823728.1	GAP1-N2+GAP1-M+GAP1-C	polar_leader+TM+TM+TM→ TM+vWA(canonical)+Fibronectin-like_repeats+TM+beta-rich+lc_tail→ Tubulin+alpha_helical+??+??→ GAP_unknown1+TM+TM+GAP_unknown2→ HRDC-like?+Fibronectin-like_repeats→ GAP1-N2+GAP1-M+GAP1-C*→ beta-rich+vWA(canonical)→??+DO-GTPase2.2→ polar_leader+TM+TM+TM→	515	Corynebacterium sp. HMSC036D03	GCA_001836185.1	actinobacteria
WP_070946690.1	GAP1-N2+GAP1-M+GAP1-C	HRDC-like?+Fibronectin-like_repeats→ GAP1-N2+GAP1-M+GAP1-C*→??+DO-GTPase2.2→	819	Mycobacterium sp. 24999	GCA_001853525.1	actinobacteria
WP_071065126.1	GAP1-N2+GAP1-M+GAP1-C	HSP70→ TPR_containing_alpha_helical+Fibronectin-like_repeats→ ZnR+ZnR+DO-GTPase2.2→ GAP1-N2+GAP1-M+GAP1-C*→ TM+TM+TM+TM+ZnR+ZnR+DO-GTPase2.2→	887	Frankia sp. NRRL B-16219	GCA_001854695.1	actinobacteria
WP_071220916.1	GAP1-N2+GAP1-M+GAP1-C+polar_tail+TM+beta-rich	TM+vWA(canonical)+YtKA-like+TM+??→ Tubulin+coiled_coil+??→ Tubulin+coiled_coil+HTH?+??→ TM+TM+TM+vWA(canonical)+TM+TM+TM+polar_tail→ Fibronectin-like_repeats+??→ ZnR+ZnR+DO-GTPase2.2+ZnR+ZnR→ GAP1-N2+GAP1-M+GAP1-C+polar_tail+TM+beta-rich*→	991	Paenibacillus sp. LC231	GCA_001860525.1	firmicutes
WP_071289221.1	GAP1-N2+GAP1-M+GAP1-C	GrpE→ HSP70→ TPR_containing_alpha_helical+Fibronectin-like_repeats→ ZnR+ZnR+DO-GTPase2.2→ GAP1-N2+GAP1-M+GAP1-C*→ TM+TM+TM+TM+ZnR+ZnR+DO-GTPase2.2→??→ HSP70→ vWA(canonical)→	802	Mycolicibacterium llatzerense	GCF_001865165.1	actinobacteria
WP_071339378.1	GAP1-N2+GAP1-M+GAP1-C	GAP1-N2+GAP1-M+GAP1-C*→	789	Streptomyces albidoflavus	GCF_001865795.1	actinobacteria

<table>
<tr><th>acc</th><th>architecture</th><th>operon</th><th>len</th><th>species</th><th>gca</th><th>taxend</th></tr>
<tr><td>WP_071386702.1</td><td>GAP1-N2+GAP1-M+GAP1-C</td><td>ZnR+ZnR+DO-GTPase2.2→ GAP1-N2+GAP1-M+GAP1-C*→ TM+TM+TM+TM+ZnR+ZnR+DO-GTPase2.2→</td><td>832</td><td>Streptomyces sp. MUSC 1</td><td>GCA_001866665.1</td><td>actinobacteria</td></tr>
<tr><td>WP_071409829.1</td><td>GAP1-N2+GAP1-M+GAP1-C</td><td>polar_leader+TM+TM+TM→ TM+vWA(canonical)+Fibronectin-like_repeats+TM+beta-rich+lc_tail→ Tubulin+alpha_helical+??+??→ GAP_unknown1+TM+TM+GAP_unknown2→ HRDC-like?+Fibronectin-like_repeats→ GAP1-N2+GAP1-M+GAP1-C*→ beta-rich+vWA(canonical)→??+DO-GTPase2.2→ polar_leader+TM+TM+TM→</td><td>564</td><td>Corynebacterium sp. HMSC074H12</td><td>GCA_001813755.1</td><td>actinobacteria</td></tr>
<tr><td>WP_071621470.1</td><td>GAP1-N2+GAP1-M+GAP1-C</td><td>TPR_containing_alpha_helical+Fibronectin-like_repeats+extended_insert+Fibronectin-like_repeats→ ZnR+ZnR+DO-GTPase2.2→ GAP1-N2+GAP1-M+GAP1-C*→ TM+TM+TM+TM+ZnR+ZnR+DO-GTPase2.2→</td><td>903</td><td>Nocardiopsis dassonvillei</td><td>GCA_001877055.1</td><td>actinobacteria</td></tr>
<tr><td>WP_071963824.1</td><td>GAP1-N2+GAP1-M+GAP1-C</td><td>GAP1-N2+GAP1-M+GAP1-C*→</td><td>863</td><td>Streptomyces cinnamoneus</td><td>GCF_001885705.1</td><td>actinobacteria</td></tr>
<tr><td>WP_072333059.1</td><td>GAP1-N2+GAP1-M+GAP1-C+polar_tail+TM+Cadherin-like</td><td>TM+vWA(canonical)+YtKA-like+TM+??→ Tubulin+coiled_coil+??→ Tubulin+coiled_coil+HTH?+??→ TM+TM+TM+vWA(canonical)+TM+TM+TM+polar_tail→ Fibronectin-like_repeats+??→ ZnR+ZnR+DO-GTPase2.2+ZnR+ZnR→ GAP1-N2+GAP1-M+GAP1-C+polar_tail+TM+Cadherin-like*→</td><td>919</td><td>Paenibacillus</td><td>GCA_900101595.1</td><td>firmicutes</td></tr>
<tr><td>WP_072417066.1</td><td>GAP1-N2+GAP1-M+GAP1-C</td><td>GrpE→ HSP70→ ZnR+ZnR+DO-GTPase2.2→?→?→ GAP1-N2+GAP1-M+GAP1-C*→</td><td>916</td><td>Ruminococcus</td><td>GCA_900103235.1</td><td>firmicutes</td></tr>
<tr><td>WP_072485992.1</td><td>GAP1-N2+GAP1-M+GAP1-C</td><td>GAP1-N2+GAP1-M+GAP1-C*→</td><td>820</td><td>Streptomyces atratus</td><td>GCA_900119365.1</td><td>actinobacteria</td></tr>
<tr><td>WP_072537017.1</td><td>GAP1-N2+GAP1-M+GAP1-C</td><td>TM+TM+TM+TM+ZnR+ZnR+DO-GTPase2.2→ GrpE→ HSP70→ HSP70+vWA(canonical)→ DnaJ+DnaJ+ZnR+ZnR+coiled_coil+Fibronectin-like_repeats→ ZnR+ZnR+DO-GTPase2.2→ GAP1-N2+GAP1-M+GAP1-C*→ TM+TM+TM+TM+ZnR+ZnR+DO-GTPase2.2→</td><td>866</td><td>Anaerococcus mediterraneensis</td><td>GCF_900128415.1</td><td>firmicutes</td></tr>
<tr><td>WP_072634855.1</td><td>GAP1-N2+GAP1-M+GAP1-C</td><td>polar_leader+TM+TM+TM→ TM+vWA(canonical)+Fibronectin-like_repeats+TM+beta-rich+lc_tail→ Tubulin+alpha_helical+??+??→ GAP_unknown1+TM+TM+GAP_unknown2→ HRDC-like?+Fibronectin-like_repeats→ GAP1-N2+GAP1-M+GAP1-C*→ beta-rich+vWA(canonical)→??+DO-GTPase2.2→</td><td>921</td><td>Rhodococcus sp. M8</td><td>-</td><td>actinobacteria</td></tr>
<tr><td>WP_072728943.1</td><td>GAP1-N2+GAP1-M+GAP1-C+polar_tail+TM+beta-rich</td><td>TM+vWA(canonical)+YtKA-like+TM+??→ Tubulin+coiled_coil+??→ Tubulin+coiled_coil+HTH?+??→ TM+TM+TM+vWA(canonical)+TM+TM+TM+polar_tail→ Fibronectin-like_repeats+??→ ZnR+ZnR+DO-GTPase2.2+ZnR+ZnR→ GAP1-N2+GAP1-M+GAP1-C+polar_tail+TM+beta-rich*→</td><td>935</td><td>Paenibacillus sp. NAIST15-1</td><td>GCF_001894745.1</td><td>firmicutes</td></tr>
<tr><td>WP_072733669.1</td><td>GAP1-N2+GAP1-M+GAP1-C+polar_tail+TM+beta-rich</td><td>TM+vWA(canonical)+YtKA-like+TM+??→ Tubulin+coiled_coil+??→ Tubulin+coiled_coil+HTH?+??→ TM+TM+TM+vWA(canonical)+TM+TM+TM+polar_tail→ Fibronectin-like_repeats+??→ ZnR+ZnR+DO-GTPase2.2+ZnR+ZnR→ GAP1-N2+GAP1-M+GAP1-C+polar_tail+TM+beta-rich*→</td><td>1058</td><td>Paenibacillus sp. ov031</td><td>GCA_900143165.1</td><td>firmicutes</td></tr>
<tr><td>WP_072804085.1</td><td>GAP1-N2+GAP1-M+GAP1-C</td><td>polar_leader+TM+TM+TM→ TM+vWA(canonical)+Fibronectin-like_repeats+TM+beta-rich+lc_tail→ Tubulin+alpha_helical+??+??→ GAP_unknown1+TM+TM+GAP_unknown2→ HRDC-like?+Fibronectin-like_repeats→ GAP1-N2+GAP1-M+GAP1-C*→ beta-rich+vWA(canonical)→??+DO-GTPase2.2→</td><td>906</td><td>Rhodococcus yunnanensis</td><td>GCF_001895005.1</td><td>actinobacteria</td></tr>
<tr><td>WP_072845700.1</td><td>GAP1-N2+GAP1-M+GAP1-C</td><td>Tubulin+alpha_helical+??+??→ GAP_unknown1+TM+TM+GAP_unknown2→ HRDC-like?+Fibronectin-like_repeats→ GAP1-N2+GAP1-M+GAP1-C*→ beta-rich+vWA(canonical)→??+DO-GTPase2.2→</td><td>922</td><td>Rhodococcus tukisamuensis</td><td>GCA_900101735.1</td><td>actinobacteria</td></tr>
<tr><td>WP_072944212.1</td><td>GAP1-N2+GAP1-M+GAP1-C</td><td>polar_leader+TM+TM+TM→ TM+vWA(canonical)+Fibronectin-like_repeats+TM+beta-rich+lc_tail→ Tubulin+alpha_helical+??+??→ GAP_unknown1+TM+TM+GAP_unknown2→ HRDC-like?+Fibronectin-like_repeats→ GAP1-N2+GAP1-M+GAP1-C*→ beta-rich+vWA(canonical)→??+DO-GTPase2.2→</td><td>914</td><td>Rhodococcus koreensis</td><td>GCA_900105905.1</td><td>actinobacteria</td></tr>
<tr><td>WP_072949111.1</td><td>GAP1-N2+GAP1-M+GAP1-C</td><td>HSP70→ HSP70+vWA(canonical)→?→?→ DnaJ+DnaJ+ZnR+ZnR+coiled_coil+Fibronectin-like_repeats→ ZnR+ZnR+DO-GTPase2.2→ ZnR+ZnR+DO-GTPase2.2→ GAP1-N2+GAP1-M+GAP1-C*→</td><td>879</td><td>Ruminococcus flavefaciens</td><td>GCA_900143025.1</td><td>firmicutes</td></tr>
<tr><td>WP_073186675.1</td><td>GAP1-N2+GAP1-M+GAP1-C</td><td>polar_leader+TM+TM+TM→ TM+vWA(canonical)+Fibronectin-like_repeats+TM+beta-rich+lc_tail→ Tubulin+alpha_helical+??+??→ GAP_unknown1+TM+TM+GAP_unknown2→?→ HRDC-like?+Fibronectin-like_repeats→ GAP1-N2+GAP1-M+GAP1-C*→?→??+DO-GTPase2.2→</td><td>452</td><td>Tessaracoccus bendigoensis</td><td>GCA_900141915.1</td><td>actinobacteria</td></tr>
<tr><td>WP_073221137.1</td><td>GAP1-N2+GAP1-M+GAP1-C</td><td>GAP1-N2+GAP1-M+GAP1-C*→</td><td>831</td><td>Streptomyces sp. NBRC 110465</td><td>GCF_001895105.1</td><td>actinobacteria</td></tr>
<tr><td>WP_073366343.1</td><td>GAP1-N2+GAP1-M+GAP1-C</td><td>TM+vWA(canonical)+Fibronectin-like_repeats+TM+beta-rich+lc_tail→ Tubulin+alpha_helical+??+??→ GAP_unknown1+TM+TM+GAP_unknown2→ HRDC-like?+Fibronectin-like_repeats→ GAP1-N2+GAP1-M+GAP1-C*→ beta-rich+vWA(canonical)→??+DO-GTPase2.2→</td><td>924</td><td>Rhodococcus</td><td>GCA_002300155.1</td><td>actinobacteria</td></tr>
<tr><td>WP_073375663.1</td><td>GAP1-N2+GAP1-M+GAP1-C</td><td>GrpE→ HSP70→ TPR_containing_alpha_helical+Fibronectin-like_repeats+extended_insert+Fibronectin-like_repeats→ ZnR+ZnR+DO-GTPase2.2→ GAP1-N2+GAP1-M+GAP1-C*→ TM+TM+TM+TM+ZnR+ZnR+DO-GTPase2.2→</td><td>805</td><td>Nocardiopsis flavescens</td><td>GCA_900141985.1</td><td>actinobacteria</td></tr>
<tr><td>WP_073538748.1</td><td>GAP1-N2+GAP1-M+GAP1-C</td><td>Fibronectin-like_repeats+??→ ZnR+ZnR+DO-GTPase2.2→ GAP1-N2+GAP1-M+GAP1-C*→ TM+TM+TM+TM+ZnR+ZnR+DO-GTPase2.2→ TM+vWA(canonical)+YtKA-like+TM+??→ Tubulin+coiled_coil+??→ Tubulin+coiled_coil+HTH?+??→ Fibronectin-like_repeats+??→ ZnR+ZnR+DO-GTPase2.2+ZnR+ZnR→</td><td>887</td><td>Clostridium kluyveri</td><td>GCA_001902295.1</td><td>firmicutes</td></tr>
<tr><td>WP_073702812.1</td><td>GAP1-N2+GAP1-M+GAP1-C</td><td>TPR_containing_alpha_helical+Fibronectin-like_repeats+extended_insert+Fibronectin-like_repeats→ ZnR+ZnR+DO-GTPase2.2→ GAP1-N2+GAP1-M+GAP1-C*→ TM+TM+TM+TM+ZnR+ZnR+DO-GTPase2.2→</td><td>926</td><td>Nocardiopsis sp. TSRI0078</td><td>GCA_001905145.1</td><td>actinobacteria</td></tr>
<tr><td>WP_073719114.1</td><td>GAP1-N2+GAP1-M+GAP1-C</td><td>GAP1-N2+GAP1-M+GAP1-C*→</td><td>807</td><td>Streptomyces sp. TSRI0281</td><td>GCA_001905125.1</td><td>actinobacteria</td></tr>
<tr><td>WP_073734900.1</td><td>GAP1-N2+GAP1-M+GAP1-C</td><td>GAP1-N2+GAP1-M+GAP1-C*→</td><td>825</td><td>Streptomyces sp. CB02488</td><td>GCA_001905365.1</td><td>actinobacteria</td></tr>
<tr><td>WP_073743189.1</td><td>GAP1-N2+GAP1-M+GAP1-C</td><td>GAP1-N2+GAP1-M+GAP1-C*→</td><td>822</td><td>Streptomyces sp. CB02115</td><td>GCA_001905645.1</td><td>actinobacteria</td></tr>
<tr><td>WP_073748419.1</td><td>GAP1-N2+GAP1-M+GAP1-C</td><td>GAP1-N2+GAP1-M+GAP1-C*→</td><td>804</td><td>Streptomyces sp. CB02058</td><td>GCA_001905505.1</td><td>actinobacteria</td></tr>
<tr><td>WP_073764134.1</td><td>GAP1-N2+GAP1-M+GAP1-C</td><td>TPR_containing_alpha_helical+Fibronectin-like_repeats→ ZnR+ZnR+DO-GTPase2.2→ GAP1-N2+GAP1-M+GAP1-C*→ TM+TM+TM+TM+ZnR+ZnR+DO-GTPase2.2+TM→ vWA(canonical)→ PP2C→ Pkinase+ZnR+beta-rich→</td><td>820</td><td>Streptomyces sp. CB02923</td><td>GCA_001905005.1</td><td>actinobacteria</td></tr>
<tr><td>WP_073778800.1</td><td>GAP1-N2+GAP1-M+GAP1-C</td><td>GAP1-N2+GAP1-M+GAP1-C*→</td><td>826</td><td>Streptomyces sp. TSRI0445</td><td>GCA_001905405.1</td><td>actinobacteria</td></tr>
<tr><td>WP_073785338.1</td><td>GAP1-N2+GAP1-M+GAP1-C</td><td>GAP1-N2+GAP1-M+GAP1-C*→</td><td>836</td><td>Streptomyces sp. CB01580</td><td>GCA_001905795.1</td><td>actinobacteria</td></tr>
<tr><td>WP_073822958.1</td><td>GAP1-N2+GAP1-M+GAP1-C</td><td>GAP1-N2+GAP1-M+GAP1-C*→</td><td>830</td><td>Streptomyces sp. CB02130</td><td>GCA_001905785.1</td><td>actinobacteria</td></tr>
<tr><td>WP_073846654.1</td><td>GAP1-N2+GAP1-M+GAP1-C+TM</td><td>vWA(canonical)+beta-rich+low_complexity→ Tubulin+alpha_helical+??+??→??+TM+TM+TM+TM+??→ Pkinase+TM→ ZnR+ZnR+DO-GTPase2.2→ GAP1-N2+GAP1-M+GAP1-C+TM*→</td><td>476</td><td>Amycolatopsis sp. CB00013</td><td>GCA_001905755.1</td><td>actinobacteria</td></tr>
<tr><td>WP_073861388.1</td><td>GAP1-N2+GAP1-M+GAP1-C</td><td>GAP1-N2+GAP1-M+GAP1-C*→</td><td>815</td><td>Streptomyces sp. CB01249</td><td>GCA_001905735.1</td><td>actinobacteria</td></tr>
<tr><td>WP_073869343.1</td><td>GAP1-N2+GAP1-M+GAP1-C</td><td>TPR_containing_alpha_helical+Fibronectin-like_repeats→ ZnR+ZnR+DO-GTPase2.2→ GAP1-N2+GAP1-M+GAP1-C*→ TM+TM+TM+TM+ZnR+ZnR+DO-GTPase2.2+TM→ vWA(canonical)→ PP2C→ Pkinase+ZnR+beta-rich→||←?←?||GAP1-N2+GAP1-M+GAP1-C→</td><td>821</td><td>Streptomyces sp. CB00072</td><td>GCA_001905575.1</td><td>actinobacteria</td></tr>
<tr><td>WP_073869350.1</td><td>GAP1-N2+GAP1-M+GAP1-C</td><td>GAP1-N2+GAP1-M+GAP1-C→ TM+TM+TM+TM+ZnR+ZnR+DO-GTPase2.2+TM→ vWA(canonical)→ PP2C→ Pkinase+ZnR+beta-rich→||←?←?||GAP1-N2+GAP1-M+GAP1-C*→</td><td>823</td><td>Streptomyces sp. CB00072</td><td>GCA_001905575.1</td><td>actinobacteria</td></tr>
<tr><td>WP_073881706.1</td><td>GAP1-N2+GAP1-M+GAP1-C</td><td>TPR_containing_alpha_helical+Fibronectin-like_repeats→ ZnR+ZnR+DO-GTPase2.2→ GAP1-N2+GAP1-M+GAP1-C*→ TM+TM+TM+TM+ZnR+ZnR+DO-GTPase2.2+TM→ vWA(canonical)→ PP2C→ Pkinase+ZnR+beta-rich→</td><td>855</td><td>Streptomyces sp. CB00316</td><td>GCA_001905595.1</td><td>actinobacteria</td></tr>
<tr><td>WP_073884323.1</td><td>GAP1-N2+GAP1-M+GAP1-C</td><td>polar_leader+TM+TM+TM→ TM+vWA(canonical)+Fibronectin-like_repeats+TM+beta-rich+lc_tail→ Tubulin+alpha_helical+??+??→ GAP_unknown1+TM+TM+GAP_unknown2→ HRDC-like?+Fibronectin-like_repeats→ GAP1-N2+GAP1-M+GAP1-C*→ beta-rich+vWA(canonical)→??+DO-GTPase2.2→</td><td>577</td><td>Corynebacterium variabile</td><td>GCA_900015285.1</td><td>actinobacteria</td></tr>
<tr><td>WP_073906675.1</td><td>GAP1-N2+GAP1-M+GAP1-C</td><td>TPR_containing_alpha_helical+Fibronectin-like_repeats→ ZnR+ZnR+DO-GTPase2.2→ GAP1-N2+GAP1-M+GAP1-C*→ TM+TM+TM+TM+ZnR+ZnR+DO-GTPase2.2+TM→ vWA(canonical)→ PSE→ Pkinase+ZnR+beta-rich→</td><td>851</td><td>Streptomyces sp. CB02009</td><td>GCA_001905825.1</td><td>actinobacteria</td></tr>
<tr><td>WP_073907729.1</td><td>GAP1-N2+GAP1-M+GAP1-C</td><td>GAP_unknown1+TM+TM+GAP_unknown2→ HRDC-like?+Fibronectin-like_repeats→ GAP1-N2+GAP1-M+GAP1-C*→ beta-rich+vWA(canonical)→??+DO-GTPase2.2→ TM+DUF4190+TM+PknH_C→ TM+DUF4190+TM+PknH_C→</td><td>826</td><td>Mycobacterium sp. SWH-M3</td><td>GCA_001907615.1</td><td>actinobacteria</td></tr>
<tr><td>WP_073966110.1</td><td>GAP1-N2+GAP1-M+GAP1-C</td><td>GAP1-N2+GAP1-M+GAP1-C*→</td><td>815</td><td>Streptomyces sp. CB02460</td><td>GCA_001905705.1</td><td>actinobacteria</td></tr>
<tr><td>WP_074082948.1</td><td>GAP1-N2+GAP1-M+GAP1-C+polar_tail+TM+beta-rich</td><td>TM+vWA(canonical)+YtKA-like+TM+??→ Tubulin+coiled_coil+??→ Tubulin+coiled_coil+HTH?+??→ TM+TM+TM+vWA(canonical)+TM+TM+TM+polar_tail→ Fibronectin-like_repeats+??→ ZnR+ZnR+DO-GTPase2.2+ZnR+ZnR→ GAP1-N2+GAP1-M+GAP1-C+polar_tail+TM+beta-rich*→</td><td>1030</td><td>Paenibacillus sp. P32E</td><td>GCA_001909085.1</td><td>firmicutes</td></tr>
<tr><td>WP_074092586.1</td><td>GAP1-N2+GAP1-M+GAP1-C+polar_tail</td><td>Tubulin+coiled_coil+??→ Tubulin+coiled_coil+HTH?+??→ TM+TM+TM+vWA+TM+TM+TM+polar_tail→ Fibronectin-like_repeats+??→ ZnR+ZnR+DO-GTPase2.2→ GAP1-N2+GAP1-M+GAP1-C+polar_tail*→</td><td>1069</td><td>Paenibacillus xylanexedens</td><td>GCA_001908275.1</td><td>firmicutes</td></tr>
<tr><td>WP_074100231.1</td><td>GAP1-N2+GAP1-M+GAP1-C+polar_tail+TM+beta-rich</td><td>TM+vWA(canonical)+YtKA-like+TM+??→ Tubulin+coiled_coil+??→ Tubulin+coiled_coil+HTH?+??→ TM+TM+TM+vWA(canonical)+TM+TM+TM+polar_tail→ Fibronectin-like_repeats+??→ ZnR+ZnR+DO-GTPase2.2+ZnR+ZnR→ GAP1-N2+GAP1-M+GAP1-C+polar_tail+TM+beta-rich*→</td><td>1030</td><td>Paenibacillus sp. P3E</td><td>GCA_001909045.1</td><td>firmicutes</td></tr>
<tr><td>WP_074108094.1</td><td>GAP1-N2+GAP1-M+GAP1-C+polar_tail+TM+beta-rich</td><td>TM+vWA(canonical)+YtKA-like+TM+??→ Tubulin+coiled_coil+??→ Tubulin+coiled_coil+HTH?+??→ TM+TM+TM+vWA(canonical)+TM+TM+TM+polar_tail→ Fibronectin-like_repeats+??→ ZnR+ZnR+DO-GTPase2.2+ZnR+ZnR→ GAP1-N2+GAP1-M+GAP1-C+polar_tail+TM+beta-rich*→</td><td>1030</td><td>Paenibacillus sp. P26E</td><td>GCA_001909055.1</td><td>firmicutes</td></tr>
<tr><td>WP_074110925.1</td><td>GAP1-N2+GAP1-M+GAP1-C+polar_tail+TM+beta-rich</td><td>TM+vWA(canonical)+YtKA-like+TM+??→ Tubulin+coiled_coil+??→ Tubulin+coiled_coil+HTH?+??→ TM+TM+TM+vWA(canonical)+TM+TM+TM+polar_tail→ Fibronectin-like_repeats+??→ ZnR+ZnR+DO-GTPase2.2+ZnR+ZnR→ GAP1-N2+GAP1-M+GAP1-C+polar_tail+TM+beta-rich*→</td><td>1025</td><td>Paenibacillus sp. P46E</td><td>GCA_001909095.1</td><td>firmicutes</td></tr>
<tr><td>WP_074311174.1</td><td>GAP1-N2+GAP1-M+GAP1-C</td><td>DO-GTPase2.1+TM→ GAP1-N2+GAP1-M+GAP1-C*→</td><td>468</td><td>Singulisphaera sp. GP187</td><td>GCF_900129635.1</td><td>planctomycetes</td></tr>
<tr><td>WP_074315831.1</td><td>GAP1-N2+GAP1-M+GAP1-C</td><td>GAP1-N2+GAP1-M+GAP1-C*→</td><td>1117</td><td>Singulisphaera sp. GP187</td><td>GCA_900129635.1</td><td>planctomycetes</td></tr>
<tr><td>WP_074469694.1</td><td>GAP1-N2+GAP1-M+GAP1-C</td><td>polar_leader+TM+TM+TM→ TM+vWA(canonical)+Fibronectin-like_repeats+TM+beta-rich+lc_tail→ Tubulin+alpha_helical+??+??→ GAP_unknown1+TM+TM+GAP_unknown2→ HRDC-like?+Fibronectin-like_repeats→ GAP1-N2+GAP1-M+GAP1-C*→ beta-rich+vWA(canonical)→??+DO-GTPase2.2→</td><td>631</td><td>Corynebacterium</td><td>GCF_000010225.1</td><td>actinobacteria</td></tr>
<tr><td>WP_074492419.1</td><td>GAP1-N2+GAP1-M+GAP1-C</td><td>polar_leader+TM+TM+TM→ TM+vWA(canonical)+Fibronectin-like_repeats+TM+beta-rich+lc_tail→ Tubulin+alpha_helical+??+??→ GAP_unknown1+TM+TM+GAP_unknown2→ HRDC-like?+Fibronectin-like_repeats→ GAP1-N2+GAP1-M+GAP1-C*→ beta-rich+vWA(canonical)→??+DO-GTPase2.2→</td><td>631</td><td>Corynebacterium glutamicum</td><td>GCF_001912725.1</td><td>actinobacteria</td></tr>
<tr><td>WP_074505991.1</td><td>GAP1-N2+GAP1-M+GAP1-C</td><td>polar_leader+TM+TM+TM→ TM+vWA(canonical)+Fibronectin-like_repeats+TM+beta-rich+lc_tail→ Tubulin+alpha_helical+??+??→ GAP_unknown1+TM+TM+GAP_unknown2→ HRDC-like?+Fibronectin-like_repeats→ GAP1-N2+GAP1-M+GAP1-C*→ beta-rich+vWA(canonical)→??+DO-GTPase2.2→</td><td>631</td><td>Corynebacterium glutamicum</td><td>GCF_001912755.2</td><td>actinobacteria</td></tr>
<tr><td>WP_074508404.1</td><td>GAP1-N2+GAP1-M+GAP1-C</td><td>polar_leader+TM+TM+TM→ TM+vWA(canonical)+Fibronectin-like_repeats+TM+beta-rich+lc_tail→ Tubulin+alpha_helical+??+??→ GAP_unknown1+TM+TM+GAP_unknown2→ HRDC-like?+Fibronectin-like_repeats→ GAP1-N2+GAP1-M+GAP1-C*→ beta-rich+vWA(canonical)→??+DO-GTPase2.2→</td><td>631</td><td>Corynebacterium glutamicum</td><td>GCF_000828015.1</td><td>actinobacteria</td></tr>
<tr><td>WP_074648449.1</td><td>GAP1-N2+GAP1-M+GAP1-C+polar_tail+TM+beta-rich</td><td>TM+vWA(canonical)+YtKA-like+TM+??→ Tubulin+coiled_coil+??→ Tubulin+coiled_coil+HTH?+??→ TM+TM+TM+vWA(canonical)+TM+TM+TM+polar_tail→ Fibronectin-like_repeats+??→ ZnR+ZnR+DO-GTPase2.2+ZnR+ZnR→ GAP1-N2+GAP1-M+GAP1-C+polar_tail+TM+beta-rich*→</td><td>1037</td><td>Paenibacillus jilunlii</td><td>GCA_900102965.1</td><td>firmicutes</td></tr>
<tr><td>WP_074991431.1</td><td>GAP1-N2+GAP1-M+GAP1-C</td><td>PP2C→ Pkinase→?→?→?→ HSP70_domain_2+low_complexity+domain_betaX→ ZnR+ZnR+DO-GTPase2.2→ GAP1-N2+GAP1-M+GAP1-C*→ TM+TM+TM+TM+ZnR+ZnR+DO-GTPase2.2→</td><td>814</td><td>Streptomyces misionensis</td><td>GCA_900104815.1</td><td>actinobacteria</td></tr>
</table>

acc	architecture	operon	len	species	gca	taxend
WP_074999863.1	GAP1-N2+GAP1-M+GAP1-C	PP2C→ Pkinase→?→?→?→ HSP70_domain_2+low_complexity+domain_betaX→ ZnR+ZnR+DO-GTPase2.2→ GAP1-N2+GAP1-M+GAP1-C*→ TM+TM+TM+TM+ZnR+ZnR+DO-GTPase2.2→	862	Streptomyces qinglanensis	GCA_900111245.1	actinobacteria
WP_075092843.1	GAP1-N2+GAP1-M+TM+GAP1-C	DO-GTPase2.2→ GAP1-N2+GAP1-M+TM+GAP1-C*→??+TM+??+beta-rich??→ GYF+GYF+Trypsin→	874	Planctomyces sp. SH-PL14	GCF_001610835.1	planctomycetes
WP_075093035.1	GAP1-N2+GAP1-M+TM+GAP1-C	ZnR+ZnR+DO-GTPase2.2→ GAP1-N2+GAP1-M+TM+GAP1-C*→??+TM+??+beta-rich??→ GYF+GYF+Trypsin→ Pkinase+low_complexity+TM→	907	Planctomyces sp. SH-PL14	GCF_001610835.1	planctomycetes
WP_075154132.1	GAP1-N2+GAP1-M+GAP1-C+polar_tail+TM+beta-rich	TM+vWA(canonical)+YtKA-like+TM+??→ Tubulin+coiled_coil+??→ Tubulin+coiled_coil+HTH?+??→ TM+TM+TM+vWA(canonical)+TM+TM+polar_tail→ Fibronectin-like_repeats+??→ ZnR+ZnR+DO-GTPase2.2+ZnR+ZnR→ GAP1-N2+GAP1-M+GAP1-C+polar_tail+TM+beta-rich*→	1002	Paenibacillus polymyxa	GCA_001922145.1	firmicutes
WP_075249976.1	GAP1-N2+GAP1-M+GAP1-C	polar_leader+TM+TM+TM→ TM+vWA(canonical)+Fibronectin-like_repeats+TM+beta-rich+lc_tail→ Tubulin+alpha_helical+??+??→ GAP_unknown1+TM+TM+GAP_unknown2→ HRDC-like?+Fibronectin-like_repeats→ GAP1-N2+GAP1-M+GAP1-C*→?→??+DO-GTPase2.2→	934	Actinomyces oris	GCA_001929375.1	actinobacteria
WP_075348368.1	GAP1-N2+GAP1-M+GAP1-C	polar_leader+TM+TM+TM→ TM+vWA(canonical)+Fibronectin-like_repeats+TM+beta-rich+lc_tail→ Tubulin+alpha_helical+??+??→ GAP_unknown1+TM+TM+GAP_unknown2→ HRDC-like?+Fibronectin-like_repeats→ GAP1-N2+GAP1-M+GAP1-C*→ beta-rich+vWA(canonical)→??+DO-GTPase2.2→	631	Corynebacterium glutamicum	GCF_001936195.1	actinobacteria
WP_075373507.1	GAP1-N2+GAP1-M+GAP1-C	polar_leader+TM+TM+TM→ TM+vWA(canonical)+Fibronectin-like_repeats+TM+beta-rich+lc_tail→ Tubulin+alpha_helical+??+??→ GAP_unknown1+TM+TM+GAP_unknown2→ HRDC-like?+Fibronectin-like_repeats→ GAP1-N2+GAP1-M+GAP1-C*→?→??+DO-GTPase2.2→	919	Actinomyces oris	GCA_001937485.1	actinobacteria
WP_075389620.1	GAP1-N2+GAP1-M+GAP1-C	polar_leader+TM+TM+TM→ TM+vWA(canonical)+Fibronectin-like_repeats+TM+beta-rich+lc_tail→ Tubulin+alpha_helical+??+??→ GAP_unknown1+TM+TM+GAP_unknown2→ HRDC-like?+Fibronectin-like_repeats→ GAP1-N2+GAP1-M+GAP1-C*→?→??+DO-GTPase2.2→	920	Actinomyces oris	GCA_001937415.1	actinobacteria
WP_075392311.1	GAP1-N2+GAP1-M+GAP1-C	polar_leader+TM+TM+TM→ TM+vWA(canonical)+Fibronectin-like_repeats+TM+beta-rich+lc_tail→ Tubulin+alpha_helical+??+??→ GAP_unknown1+TM+TM+GAP_unknown2→ HRDC-like?+Fibronectin-like_repeats→ GAP1-N2+GAP1-M+GAP1-C*→?→??+DO-GTPase2.2→	920	Actinomyces oris	GCA_001937725.1	actinobacteria
WP_075401773.1	GAP1-N2+GAP1-M+GAP1-C	polar_leader+TM+TM+TM→ TM+vWA(canonical)+Fibronectin-like_repeats+TM+beta-rich+lc_tail→ Tubulin+alpha_helical+??+??→||←?||GAP_unknown1+TM+TM+GAP_unknown2→ HRDC-like?+Fibronectin-like_repeats→ GAP1-N2+GAP1-M+GAP1-C*→?→??+DO-GTPase2.2→	933	Actinomyces oris	GCA_001937425.1	actinobacteria
WP_075510382.1	GAP1-N2+GAP1-M+GAP1-C	TM+DUF4190+TM→ TM+DUF4190+TM→ polar_leader+TM+TM+TM→ TM+vWA(canonical)+Fibronectin-like_repeats+TM+beta-rich+lc_tail→ Tubulin+alpha_helical+??+??→ GAP_unknown1+TM+TM+GAP_unknown2→ HRDC-like?+Fibronectin-like_repeats→ GAP1-N2+GAP1-M+GAP1-C*→ beta-rich+vWA(canonical)→??+DO-GTPase2.2→	818	Mycobacterium kansasii	GCA_001632895.1	actinobacteria
WP_075544871.1	GAP1-N2+GAP1-M+GAP1-C	TM+DUF4190+TM→ TM+DUF4190+TM→ polar_leader+TM+TM+TM→ TM+vWA(canonical)+Fibronectin-like_repeats+TM+beta-rich+lc_tail→ Tubulin+alpha_helical+??+??→ GAP_unknown1+TM+TM+GAP_unknown2→ HRDC-like?+Fibronectin-like_repeats→ GAP1-N2+GAP1-M+GAP1-C*→ beta-rich+vWA(canonical)→??+DO-GTPase2.2→ TM+DUF4190+TM+PknH_C→	832	Mycobacterium kansasii	GCA_001632905.1	actinobacteria
WP_075549506.1	GAP1-N2+GAP1-M+GAP1-C	TM+DUF4190+TM→ TM+DUF4190+TM→ polar_leader+TM+TM+TM→ TM+vWA(canonical)+Fibronectin-like_repeats+TM+beta-rich+lc_tail→ Tubulin+alpha_helical+??+??→ GAP_unknown1+TM+TM+GAP_unknown2→ HRDC-like?+Fibronectin-like_repeats→ GAP1-N2+GAP1-M+GAP1-C*→ beta-rich+vWA(canonical)→??+DO-GTPase2.2→	818	Mycobacterium kansasii	GCA_001632975.1	actinobacteria
WP_075690388.1	GAP1-N2+GAP1-M+GAP1-C	SP+vWA+YtKA-like+TM+??→ Tubulin+coiled_coil+??→ Tubulin+coiled_coil+HTH?+??→ Fibronectin-like_repeats+??→ ZnR+ZnR+DO-GTPase2.2→ GAP1-N2+GAP1-M+GAP1-C*→	727	Bacillus sp. MRMR6	GCA_001940785.1	firmicutes
WP_075691160.1	GAP1-N2+GAP1-M+GAP1-C	polar_leader+TM+TM+TM→ TM+vWA(canonical)+Fibronectin-like_repeats+TM+beta-rich+lc_tail→ Tubulin+alpha_helical+??+??→ GAP_unknown1+TM+TM+GAP_unknown2→ HRDC-like?+Fibronectin-like_repeats→ GAP1-N2+GAP1-M+GAP1-C*→ beta-rich+vWA(canonical)→??+DO-GTPase2.2→	792	Corynebacterium sphenisci	GCA_001941505.1	actinobacteria
WP_075727653.1	GAP1-N2+GAP1-M+GAP1-C	polar_leader+TM+TM+TM→ TM+vWA(canonical)+Fibronectin-like_repeats+TM+beta-rich+lc_tail→ Tubulin+alpha_helical+??+??→ GAP_unknown1+TM+TM+GAP_unknown2→ HRDC-like?+Fibronectin-like_repeats→ GAP1-N2+GAP1-M+GAP1-C*→ beta-rich+vWA(canonical)→??+DO-GTPase2.2→	793	Corynebacterium aquilae	GCF_001941445.1	actinobacteria
WP_075728933.1	GAP1-N2+GAP1-M+GAP1-C	polar_leader+TM+TM+TM→ TM+vWA(canonical)+Fibronectin-like_repeats+TM+beta-rich+lc_tail→ Tubulin+alpha_helical+??+??→ GAP_unknown1+TM+TM+GAP_unknown2→ HRDC-like?+Fibronectin-like_repeats→ GAP1-N2+GAP1-M+GAP1-C*→ beta-rich+vWA(canonical)→??+DO-GTPase2.2→ polar_leader+TM+TM+TM→	512	Corynebacterium flavescens	GCA_006539465.1	actinobacteria
WP_075861320.1	GAP1-N2+GAP1-M+GAP1-C	polar_leader+TM+TM+TM→ TM+vWA(canonical)+Fibronectin-like_repeats+TM+beta-rich+lc_tail→ Tubulin+alpha_helical+??+??→ GAP_unknown1+TM+TM+GAP_unknown2→ HRDC-like?+Fibronectin-like_repeats→ GAP1-N2+GAP1-M+GAP1-C*→ beta-rich+vWA(canonical)→??+DO-GTPase2.2→	631	Corynebacterium	GCA_001683055.1	actinobacteria
WP_075910081.1	GAP1-N2+GAP1-M+GAP1-C	HRDC-like?+Fibronectin-like_repeats→ GAP1-N2+GAP1-M+GAP1-C*→??+DO-GTPase2.2→	819	Mycobacterium sp. 27335	GCA_001942625.1	actinobacteria
WP_075924409.1	GAP1-N2+GAP1-M+GAP1-C	HRDC-like?+Fibronectin-like_repeats→ GAP1-N2+GAP1-M+GAP1-C*→??+DO-GTPase2.2→	819	Mycolicibacterium porcinum	GCA_001942045.1	actinobacteria
WP_075986039.1	GAP1-N2+GAP1-M+GAP1-C	GAP1-N2+GAP1-M+GAP1-C*→	789	Streptomyces sp. FR-008	GCA_001431765.1	actinobacteria
WP_076078930.1	GAP1-N2+GAP1-M+GAP1-C+polar_tail+TM+beta-rich	TM+vWA(canonical)+YtKA-like+TM+??→ Tubulin+coiled_coil+??→ Tubulin+coiled_coil+HTH?+??→ TM+TM+TM+vWA(canonical)+TM+TM+TM+polar_tail→ Fibronectin-like_repeats+??→ ZnR+ZnR+DO-GTPase2.2+ZnR+ZnR→ GAP1-N2+GAP1-M+GAP1-C+polar_tail+TM+beta-rich*→	1057	Paenibacillus sp. FSL R7-0333	GCA_001955915.1	firmicutes
WP_076094863.1	GAP1-N2+GAP1-M+GAP1-C	GrpE→ HSP70→ TPR_containing_alpha_helical+Fibronectin-like_repeats→ ZnR+ZnR+DO-GTPase2.2→ GAP1-N2+GAP1-M+GAP1-C*→ TM+TM+TM+TM+ZnR+ZnR+DO-GTPase2.2+Jelly_roll+beta-sandwich→||←STAND_NTPase+TM+TM+TM+TM+TM+TM	477	Streptomyces sp. IMTB 2501	GCA_001953885.1	actinobacteria
WP_076099552.1	GAP1-N2+GAP1-M+GAP1-C+polar_tail+TM+beta-rich	TM+vWA(canonical)+YtKA-like+TM+??→ Tubulin+coiled_coil+??→ Tubulin+coiled_coil+HTH?+??→ TM+TM+TM+vWA(canonical)+TM+TM+TM+polar_tail→ Fibronectin-like_repeats+??→ ZnR+ZnR+DO-GTPase2.2+ZnR+ZnR→ GAP1-N2+GAP1-M+GAP1-C+polar_tail+TM+beta-rich*→	998	Paenibacillus odorifer	GCA_001954285.1	firmicutes
WP_076104210.1	GAP1-N2+GAP1-M+GAP1-C+polar_tail+TM+beta-rich	TM+vWA(canonical)+YtKA-like+TM+??→ Tubulin+coiled_coil+??→ Tubulin+coiled_coil+HTH?+??→ TM+TM+TM+vWA(canonical)+TM+TM+TM+polar_tail→ Fibronectin-like_repeats+??→ ZnR+ZnR+DO-GTPase2.2+ZnR+ZnR→ GAP1-N2+GAP1-M+GAP1-C+polar_tail+TM+beta-rich*→	998	Paenibacillus odorifer	GCA_001954345.1	firmicutes
WP_076110325.1	GAP1-N2+GAP1-M+GAP1-C+polar_tail+TM+beta-rich	TM+vWA(canonical)+YtKA-like+TM+??→ Tubulin+coiled_coil+??→ Tubulin+coiled_coil+HTH?+??→ TM+TM+TM+vWA(canonical)+TM+TM+TM+polar_tail→ Fibronectin-like_repeats+??→?→ ZnR+ZnR+DO-GTPase2.2+ZnR+ZnR→ GAP1-N2+GAP1-M+GAP1-C+polar_tail+TM+beta-rich*→	1050	Paenibacillus borealis	GCA_001954415.1	firmicutes
WP_076119840.1	GAP1-N2+GAP1-M+GAP1-C+polar_tail+TM+beta-rich	TM+vWA(canonical)+YtKA-like+TM+??→ Tubulin+coiled_coil+??→ Tubulin+coiled_coil+HTH?+??→ TM+TM+TM+vWA(canonical)+TM+TM+TM+polar_tail→ Fibronectin-like_repeats+??→ ZnR+ZnR+DO-GTPase2.2+ZnR+ZnR→ GAP1-N2+GAP1-M+GAP1-C+polar_tail+TM+beta-rich*→	994	Paenibacillus odorifer	GCA_001954425.1	firmicutes
WP_076129586.1	GAP1-N2+GAP1-M+GAP1-C+polar_tail+TM+beta-rich	TM+vWA(canonical)+YtKA-like+TM+??→ Tubulin+coiled_coil+??→ Tubulin+coiled_coil+HTH?+??→ TM+TM+TM+vWA(canonical)+TM+TM+TM+polar_tail→ Fibronectin-like_repeats+??→ ZnR+ZnR+DO-GTPase2.2+ZnR+ZnR→ GAP1-N2+GAP1-M+GAP1-C+polar_tail+TM+beta-rich*→	998	Paenibacillus odorifer	GCA_001954575.1	firmicutes
WP_076137120.1	GAP1-N2+GAP1-M+GAP1-C+polar_tail+TM+beta-rich	TM+vWA(canonical)+YtKA-like+TM+??→ Tubulin+coiled_coil+??→ Tubulin+coiled_coil+HTH?+??→ TM+TM+TM+vWA(canonical)+TM+TM+TM+polar_tail→ Fibronectin-like_repeats+??→ ZnR+ZnR+DO-GTPase2.2+ZnR+ZnR→ GAP1-N2+GAP1-M+GAP1-C+polar_tail+TM+beta-rich*→	998	Paenibacillus odorifer	GCA_003255855.1	firmicutes
WP_076141765.1	GAP1-N2+GAP1-M+GAP1-C+polar_tail+TM+beta-rich	TM+vWA(canonical)+YtKA-like+TM+??→ Tubulin+coiled_coil+??→ Tubulin+coiled_coil+HTH?+??→ TM+TM+TM+vWA(canonical)+TM+TM+TM+polar_tail→ Fibronectin-like_repeats+??→ ZnR+ZnR+DO-GTPase2.2+ZnR+ZnR→ GAP1-N2+GAP1-M+GAP1-C+polar_tail+TM+beta-rich*→	998	Paenibacillus odorifer	GCA_001955765.1	firmicutes
WP_076147313.1	GAP1-N2+GAP1-M+GAP1-C+polar_tail+TM+beta-rich	TM+vWA(canonical)+YtKA-like+TM+??→ Tubulin+coiled_coil+??→ Tubulin+coiled_coil+HTH?+??→ TM+TM+TM+vWA(canonical)+TM+TM+TM+polar_tail→ Fibronectin-like_repeats+??→ ZnR+ZnR+DO-GTPase2.2+ZnR+ZnR→ GAP1-N2+GAP1-M+GAP1-C+polar_tail+TM+beta-rich*→	998	Paenibacillus odorifer	GCA_001955785.1	firmicutes
WP_076151715.1	GAP1-N2+GAP1-M+GAP1-C+polar_tail+TM+beta-rich	TM+vWA(canonical)+YtKA-like+TM+??→ Tubulin+coiled_coil+??→ Tubulin+coiled_coil+HTH?+??→ TM+TM+TM+vWA(canonical)+TM+TM+TM+polar_tail→ Fibronectin-like_repeats+??→ ZnR+ZnR+DO-GTPase2.2+ZnR+ZnR→ GAP1-N2+GAP1-M+GAP1-C+polar_tail+TM+beta-rich*→	1002	Paenibacillus glucanolyticus	GCA_001955865.1	firmicutes
WP_076157013.1	GAP1-N2+GAP1-M+GAP1-C+polar_tail	TM+TM+TM+vWA+TM+TM+TM+polar_tail→ Fibronectin-like_repeats+??→ ZnR+ZnR+DO-GTPase2.2→ GAP1-N2+GAP1-M+GAP1-C+polar_tail*→	1055	Paenibacillus sp. FSL R7-0337	GCA_001955925.1	firmicutes
WP_076157214.1	GAP1-N2+GAP1-M+GAP1-C	GrpE→ HSP70→ TPR_containing_alpha_helical+Fibronectin-like_repeats→ ZnR+ZnR+DO-GTPase2.2→ GAP1-N2+GAP1-M+GAP1-C*→ PSE→?→ HSP70→ vWA(canonical)→||←?←?←HSP70←unknown+CASPASE	818	Amycolatopsis coloradensis	GCA_001953865.1	actinobacteria
WP_076160261.1	GAP1-N2+GAP1-M+GAP1-C+polar_tail+TM+beta-rich	TM+vWA(canonical)+YtKA-like+TM+??→ Tubulin+coiled_coil+??→ Tubulin+coiled_coil+HTH?+??→ TM+TM+TM+vWA(canonical)+TM+TM+TM+polar_tail→ Fibronectin-like_repeats+??→ ZnR+ZnR+DO-GTPase2.2+ZnR+ZnR→ GAP1-N2+GAP1-M+GAP1-C+polar_tail+TM+beta-rich*→	1002	Paenibacillus peoriae	GCA_001956115.1	firmicutes
WP_076172998.1	GAP1-N2+GAP1-M+GAP1-C+polar_tail+TM+beta-rich	TM+vWA(canonical)+YtKA-like+TM+??→ Tubulin+coiled_coil+??→ Tubulin+coiled_coil+HTH?+??→ TM+TM+TM+vWA(canonical)+TM+TM+TM+polar_tail→ Fibronectin-like_repeats+??→ ZnR+ZnR+DO-GTPase2.2+ZnR+ZnR→ GAP1-N2+GAP1-M+GAP1-C+polar_tail+TM+beta-rich*→	1087	Paenibacillus rhizosphaerae	GCA_001956185.1	firmicutes
WP_076180470.1	GAP1-N2+GAP1-M+GAP1-C	polar_leader+TM+TM+TM→ TM+vWA(canonical)+Fibronectin-like_repeats+TM+beta-rich+lc_tail→ Tubulin+alpha_helical+??+??→ GAP_unknown1+TM+TM+GAP_unknown2→ HRDC-like?+Fibronectin-like_repeats→ GAP1-N2+GAP1-M+GAP1-C*→ beta-rich+vWA(canonical)→??+DO-GTPase2.2→	828	Mycolicibacterium fortuitum	GCA_001954035.1	actinobacteria
WP_076182326.1	GAP1-N2+GAP1-M+GAP1-C	HRDC-like?+Fibronectin-like_repeats→ GAP1-N2+GAP1-M+GAP1-C*→??+DO-GTPase2.2→	820	Mycolicibacterium fortuitum	GCA_001954035.1	actinobacteria
WP_076185019.1	GAP1-N2+GAP1-M+GAP1-C+polar_tail+TM+beta-rich	TM+vWA(canonical)+YtKA-like+TM+??→ Tubulin+coiled_coil+??→ Tubulin+coiled_coil+HTH?+??→ TM+TM+TM+vWA(canonical)+TM+TM+TM+polar_tail→ Fibronectin-like_repeats+??→ ZnR+ZnR+DO-GTPase2.2+ZnR+ZnR→ GAP1-N2+GAP1-M+GAP1-C+polar_tail+TM+beta-rich*→	998	Paenibacillus odorifer	GCA_001954455.1	firmicutes
WP_076194447.1	GAP1-N2+GAP1-M+GAP1-C+polar_tail+TM+beta-rich	TM+vWA(canonical)+YtKA-like+TM+??→ Tubulin+coiled_coil+??→ Tubulin+coiled_coil+HTH?+??→ TM+TM+TM+vWA(canonical)+TM+TM+TM+polar_tail→ Fibronectin-like_repeats+??→ ZnR+ZnR+DO-GTPase2.2+ZnR+ZnR→ GAP1-N2+GAP1-M+GAP1-C+polar_tail+TM+beta-rich*→	998	Paenibacillus odorifer	GCA_001954515.1	firmicutes
WP_076195840.1	GAP1-N2+GAP1-M+GAP1-C	HRDC-like?+Fibronectin-like_repeats→ GAP1-N2+GAP1-M+GAP1-C*→??+DO-GTPase2.2→	816	Mycobacterium sp. GA-1841	GCA_001954135.1	actinobacteria
WP_076207257.1	GAP1-N2+GAP1-M+GAP1-C	HRDC-like?+Fibronectin-like_repeats→ GAP1-N2+GAP1-M+GAP1-C*→??+DO-GTPase2.2→	820	Mycolicibacterium fortuitum	GCA_001954145.1	actinobacteria
WP_076207875.1	GAP1-N2+GAP1-M+GAP1-C+polar_tail+TM+beta-rich	TM+vWA(canonical)+YtKA-like+TM+??→ Tubulin+coiled_coil+??→ Tubulin+coiled_coil+HTH?+??→ TM+TM+TM+vWA(canonical)+TM+TM+TM+polar_tail→ Fibronectin-like_repeats+??→ ZnR+ZnR+DO-GTPase2.2+ZnR+ZnR→ GAP1-N2+GAP1-M+GAP1-C+polar_tail+TM+beta-rich*→	998	Paenibacillus odorifer	GCA_001954595.1	firmicutes
WP_076209732.1	GAP1-N2+GAP1-M+GAP1-C+polar_tail+TM+beta-rich	TM+vWA(canonical)+YtKA-like+TM+??→ Tubulin+coiled_coil+??→ Tubulin+coiled_coil+HTH?+??→ TM+TM+TM+vWA(canonical)+TM+TM+TM+polar_tail→ Fibronectin-like_repeats+??→ ZnR+ZnR+DO-GTPase2.2+ZnR+ZnR→ GAP1-N2+GAP1-M+GAP1-C+polar_tail+TM+beta-rich*→	1072	Paenibacillus sp. FSL R5-0765	GCA_001955855.1	firmicutes
WP_076210572.1	GAP1-N2+GAP1-M+GAP1-C	HRDC-like?+Fibronectin-like_repeats→ GAP1-N2+GAP1-M+GAP1-C*→??+DO-GTPase2.2→	819	Mycolicibacterium conceptionense	GCA_001954205.1	actinobacteria
WP_076214936.1	GAP1-N2+GAP1-M+GAP1-C+polar_tail+TM+beta-rich	TM+vWA(canonical)+YtKA-like+TM+??→ Tubulin+coiled_coil+??→ Tubulin+coiled_coil+HTH?+??→ TM+TM+TM+vWA(canonical)+TM+TM+TM+polar_tail→ Fibronectin-like_repeats+??→ ZnR+ZnR+DO-GTPase2.2+ZnR+ZnR→ GAP1-N2+GAP1-M+GAP1-C+polar_tail+TM+beta-rich*→	998	Paenibacillus odorifer	GCA_001954655.1	firmicutes
WP_076219742.1	GAP1-N2+GAP1-M+GAP1-C+polar_tail+TM+beta-rich	Tubulin+coiled_coil+??→ Tubulin+coiled_coil+HTH?+??→ TM+TM+TM+vWA(canonical)+TM+TM+TM+polar_tail→ Fibronectin-like_repeats+??→ ZnR+ZnR+DO-GTPase2.2+ZnR+ZnR→ GAP1-N2+GAP1-M+GAP1-C+polar_tail+TM+beta-rich*→	853	Paenibacillus odorifer	GCA_001955595.1	firmicutes
WP_076224971.1	GAP1-N2+GAP1-M+GAP1-C+polar_tail+TM+beta-rich	TM+vWA(canonical)+YtKA-like+TM+??→ Tubulin+coiled_coil+??→ Tubulin+coiled_coil+HTH?+??→ TM+TM+TM+vWA(canonical)+TM+TM+TM+polar_tail→ Fibronectin-like_repeats+??→ ZnR+ZnR+DO-GTPase2.2+ZnR+ZnR→ GAP1-N2+GAP1-M+GAP1-C+polar_tail+TM+beta-rich*→	998	Paenibacillus odorifer	GCA_001955775.1	firmicutes
WP_076229825.1	GAP1-N2+GAP1-M+GAP1-C+polar_tail+TM+Cadherin-like	TM+vWA(canonical)+YtKA-like+TM+??→ Tubulin+coiled_coil+??→ Tubulin+coiled_coil+HTH?+??→ TM+TM+TM+vWA(canonical)+TM+TM+TM+polar_tail→ Fibronectin-like_repeats+??→ ZnR+ZnR+DO-GTPase2.2+ZnR+ZnR→ GAP1-N2+GAP1-M+GAP1-C+polar_tail+TM+Cadherin-like*→	962	Paenibacillus sp. FSL H7-0331	GCA_001956045.1	firmicutes
WP_076240572.1	GAP1-N2+GAP1-M+GAP1-C	HRDC-like?+Fibronectin-like_repeats→ GAP1-N2+GAP1-M+GAP1-C*→??+DO-GTPase2.2→	816	Mycobacterium sp. NS-7484	GCA_001953975.1	actinobacteria
WP_076247349.1	GAP1-N2+GAP1-M+GAP1-C+polar_tail+TM+beta-rich	TM+vWA(canonical)+YtKA-like+TM+??→ Tubulin+coiled_coil+??→ Tubulin+coiled_coil+HTH?+??→ TM+TM+TM+vWA(canonical)+TM+TM+TM+polar_tail→ Fibronectin-like_repeats+??→ ZnR+ZnR+DO-GTPase2.2+ZnR+ZnR→ GAP1-N2+GAP1-M+GAP1-C+polar_tail+TM+beta-rich*→	1070	Paenibacillus sp. FSL H8-0259	GCA_001956105.1	firmicutes
WP_076253673.1	GAP1-N2+GAP1-M+GAP1-C+polar_tail+TM+beta-rich	TM+vWA(canonical)+YtKA-like+TM+??→ Tubulin+coiled_coil+??→ Tubulin+coiled_coil+HTH?+??→ TM+TM+TM+vWA(canonical)+TM+TM+TM+polar_tail→ Fibronectin-like_repeats+??→ ZnR+ZnR+DO-GTPase2.2+ZnR+ZnR→ GAP1-N2+GAP1-M+GAP1-C+polar_tail+TM+beta-rich*→	1067	Paenibacillus	GCA_001956175.1	firmicutes

acc	architecture	operon	len	species	gca	taxend
WP_076265333.1	GAP1-N2+GAP1-M+GAP1-C+polar_tail+TM+beta-rich	TM+vWA(canonical)+YtKA-like+TM+??→ Tubulin+coiled_coil+??→ Tubulin+coiled_coil+HTH?+??→ TM+TM+TM+vWA(canonical)+TM+TM+TM+polar_tail→ Fibronectin-like_repeats+??→ ZnR+ZnR+DO-GTPase2.2+ZnR+ZnR→ GAP1-N2+GAP1-M+GAP1-C+polar_tail+TM+beta-rich*→	1002	Paenibacillus peoriae	GCA_001956225.1	firmicutes
WP_076269554.1	GAP1-N2+GAP1-M+GAP1-C+polar_tail+TM+beta-rich	TM+vWA(canonical)+YtKA-like+TM+??→ Tubulin+coiled_coil+??→ Tubulin+coiled_coil+HTH?+??→ TM+TM+TM+vWA(canonical)+TM+TM+TM+polar_tail→ Fibronectin-like_repeats+??→ ZnR+ZnR+DO-GTPase2.2+ZnR+ZnR→ GAP1-N2+GAP1-M+GAP1-C+polar_tail+TM+beta-rich*→	982	Paenibacillus sp. FSL A5-0031	GCA_001956295.1	firmicutes
WP_076274908.1	GAP1-N2+GAP1-M+GAP1-C+polar_tail+TM+beta-rich	TM+vWA(canonical)+YtKA-like+TM+??→ Tubulin+coiled_coil+??→ Tubulin+coiled_coil+HTH?+??→ TM+TM+TM+vWA(canonical)+TM+TM+TM+polar_tail→ Fibronectin-like_repeats+??→ ZnR+ZnR+DO-GTPase2.2+ZnR+ZnR→ GAP1-N2+GAP1-M+GAP1-C+polar_tail+TM+beta-rich*→	998	Paenibacillus odorifer	GCA_001954335.1	firmicutes
WP_076276824.1	GAP1-N2+GAP1-M+GAP1-C+polar_tail+TM+beta-rich	TM+vWA(canonical)+YtKA-like+TM+??→ Tubulin+coiled_coil+??→ Tubulin+coiled_coil+HTH?+??→ TM+TM+TM+vWA(canonical)+TM+TM+TM+polar_tail→ Fibronectin-like_repeats+??→ ZnR+ZnR+DO-GTPase2.2+ZnR+ZnR→ GAP1-N2+GAP1-M+GAP1-C+polar_tail+TM+beta-rich*→	998	Paenibacillus odorifer	GCA_001954535.1	firmicutes
WP_076279866.1	GAP1-N2+GAP1-M+GAP1-C+polar_tail+TM+beta-rich	TM+vWA(canonical)+YtKA-like+TM+??→ Tubulin+coiled_coil+??→ Tubulin+coiled_coil+HTH?+??→ TM+TM+TM+vWA(canonical)+TM+TM+TM+polar_tail→ Fibronectin-like_repeats+??→ ZnR+ZnR+DO-GTPase2.2+ZnR+ZnR→ GAP1-N2+GAP1-M+GAP1-C+polar_tail+TM+beta-rich*→	998	Paenibacillus odorifer	GCA_001954675.1	firmicutes
WP_076282101.1	GAP1-N2+GAP1-M+GAP1-C+polar_tail+TM+beta-rich	TM+vWA(canonical)+YtKA-like+TM+??→ Tubulin+coiled_coil+??→ Tubulin+coiled_coil+HTH?+??→ TM+TM+TM+vWA(canonical)+TM+TM+TM+polar_tail→ Fibronectin-like_repeats+??→ ZnR+ZnR+DO-GTPase2.2+ZnR+ZnR→ GAP1-N2+GAP1-M+GAP1-C+polar_tail+TM+beta-rich*→	998	Paenibacillus odorifer	GCA_001955555.1	firmicutes
WP_076283866.1	GAP1-N2+GAP1-M+GAP1-C+polar_tail+TM+beta-rich	TM+vWA(canonical)+YtKA-like+TM+??→ Tubulin+coiled_coil+??→ Tubulin+coiled_coil+HTH?+??→ TM+TM+TM+vWA(canonical)+TM+TM+TM+polar_tail→ Fibronectin-like_repeats+??→ ZnR+ZnR+DO-GTPase2.2+ZnR+ZnR→ GAP1-N2+GAP1-M+GAP1-C+polar_tail+TM+beta-rich*→	986	Paenibacillus odorifer	GCA_001955545.1	firmicutes
WP_076287520.1	GAP1-N2+GAP1-M+GAP1-C+polar_tail	TM+TM+TM+vWA+TM+TM+TM+polar_tail→ Fibronectin-like_repeats+??→ ZnR+ZnR+DO-GTPase2.2→ GAP1-N2+GAP1-M+GAP1-C+polar_tail*→	1045	Paenibacillus pabuli	GCF_001955985.1	firmicutes
WP_076294127.1	GAP1-N2+GAP1-M+GAP1-C+polar_tail+TM+beta-rich	TM+vWA(canonical)+YtKA-like+TM+??→ Tubulin+coiled_coil+??→ Tubulin+coiled_coil+HTH?+??→ TM+TM+TM+vWA(canonical)+TM+TM+TM+polar_tail→ Fibronectin-like_repeats+??→ ZnR+ZnR+DO-GTPase2.2+ZnR+ZnR→ GAP1-N2+GAP1-M+GAP1-C+polar_tail+TM+beta-rich*→	1002	Paenibacillus peoriae	GCA_001956155.1	firmicutes
WP_076294845.1	GAP1-N2+GAP1-M+GAP1-C+polar_tail+TM+beta-rich	TM+vWA(canonical)+YtKA-like+TM+??→ Tubulin+coiled_coil+??→ Tubulin+coiled_coil+HTH?+??→ TM+TM+TM+vWA(canonical)+TM+TM+TM+polar_tail→ Fibronectin-like_repeats+??→ ZnR+ZnR+DO-GTPase2.2+ZnR+ZnR→ GAP1-N2+GAP1-M+GAP1-C+polar_tail+TM+beta-rich*→	1002	Paenibacillus peoriae	GCA_001956235.1	firmicutes
WP_076296144.1	GAP1-N2+GAP1-M+GAP1-C+polar_tail+TM+beta-rich	TM+vWA(canonical)+YtKA-like+TM+??→ Tubulin+coiled_coil+??→ Tubulin+coiled_coil+HTH?+??→ TM+TM+TM+vWA(canonical)+TM+TM+TM+polar_tail→ Fibronectin-like_repeats+??→ ZnR+ZnR+DO-GTPase2.2+ZnR+ZnR→ GAP1-N2+GAP1-M+GAP1-C+polar_tail+TM+beta-rich*→	998	Paenibacillus odorifer	GCA_001954375.1	firmicutes
WP_076299117.1	GAP1-N2+GAP1-M+GAP1-C+polar_tail+TM+beta-rich	TM+vWA(canonical)+YtKA-like+TM+??→ Tubulin+coiled_coil+??→ Tubulin+coiled_coil+HTH?+??→ TM+TM+TM+vWA(canonical)+TM+TM+TM+polar_tail→ Fibronectin-like_repeats+??→ ZnR+ZnR+DO-GTPase2.2+ZnR+ZnR→ GAP1-N2+GAP1-M+GAP1-C+polar_tail+TM+beta-rich*→	986	Paenibacillus odorifer	GCA_001954445.1	firmicutes
WP_076302827.1	GAP1-N2+GAP1-M+GAP1-C+polar_tail+TM+beta-rich	TM+vWA(canonical)+YtKA-like+TM+??→ Tubulin+coiled_coil+??→ Tubulin+coiled_coil+HTH?+??→ TM+TM+TM+vWA(canonical)+TM+TM+TM+polar_tail→ Fibronectin-like_repeats+??→ ZnR+ZnR+DO-GTPase2.2+ZnR+ZnR→ GAP1-N2+GAP1-M+GAP1-C+polar_tail+TM+beta-rich*→	998	Paenibacillus odorifer	GCA_001954495.1	firmicutes
WP_076305104.1	GAP1-N2+GAP1-M+GAP1-C+polar_tail+TM+beta-rich	TM+vWA(canonical)+YtKA-like+TM+??→ Tubulin+coiled_coil+??→ Tubulin+coiled_coil+HTH?+??→ TM+TM+TM+vWA(canonical)+TM+TM+TM+polar_tail→ Fibronectin-like_repeats+??→ ZnR+ZnR+DO-GTPase2.2+ZnR+ZnR→ GAP1-N2+GAP1-M+GAP1-C+polar_tail+TM+beta-rich*→	998	Paenibacillus odorifer	GCA_001954505.1	firmicutes
WP_076307182.1	GAP1-N2+GAP1-M+GAP1-C+polar_tail+TM+beta-rich	TM+vWA(canonical)+YtKA-like+TM+??→ Tubulin+coiled_coil+??→ Tubulin+coiled_coil+HTH?+??→ TM+TM+TM+vWA(canonical)+TM+TM+TM+polar_tail→ Fibronectin-like_repeats+??→ ZnR+ZnR+DO-GTPase2.2+ZnR+ZnR→ GAP1-N2+GAP1-M+GAP1-C+polar_tail+TM+beta-rich*→	998	Paenibacillus odorifer	GCA_001954585.1	firmicutes
WP_076308976.1	GAP1-N2+GAP1-M+GAP1-C+polar_tail+TM+beta-rich	TM+vWA(canonical)+YtKA-like+TM+??→ Tubulin+coiled_coil+??→ Tubulin+coiled_coil+HTH?+??→ TM+TM+TM+vWA(canonical)+TM+TM+TM+polar_tail→ Fibronectin-like_repeats+??→ ZnR+ZnR+DO-GTPase2.2+ZnR+ZnR→ GAP1-N2+GAP1-M+GAP1-C+polar_tail+TM+beta-rich*→	998	Paenibacillus odorifer	GCA_001954665.1	firmicutes
WP_076309542.1	GAP1-N2+GAP1-M+GAP1-C+polar_tail+TM+beta-rich	TM+vWA(canonical)+YtKA-like+TM+??→ Tubulin+coiled_coil+??→ Tubulin+coiled_coil+HTH?+??→ TM+TM+TM+vWA(canonical)+TM+TM+TM+polar_tail→ Fibronectin-like_repeats+??→ ZnR+ZnR+DO-GTPase2.2+ZnR+ZnR→ GAP1-N2+GAP1-M+GAP1-C+polar_tail+TM+beta-rich*→	998	Paenibacillus odorifer	GCA_001955495.1	firmicutes
WP_076316998.1	GAP1-N2+GAP1-M+GAP1-C+polar_tail+TM+beta-rich	TM+vWA(canonical)+YtKA-like+TM+??→ Tubulin+coiled_coil+??→ Tubulin+coiled_coil+HTH?+??→ TM+TM+TM+vWA(canonical)+TM+TM+TM+polar_tail→ Fibronectin-like_repeats+??→ ZnR+ZnR+DO-GTPase2.2+ZnR+ZnR→ GAP1-N2+GAP1-M+GAP1-C+polar_tail+TM+beta-rich*→	998	Paenibacillus odorifer	GCA_001955835.1	firmicutes
WP_076319273.1	GAP1-N2+GAP1-M+GAP1-C+polar_tail	Tubulin+coiled_coil+??→ Tubulin+coiled_coil+HTH?+??→ TM+TM+TM+vWA+TM+TM+TM+polar_tail→ Fibronectin-like_repeats+??→ ZnR+ZnR+DO-GTPase2.2→ GAP1-N2+GAP1-M+GAP1-C+polar_tail*→	1067	Paenibacillus amylolyticus	GCA_001955995.1	firmicutes
WP_076325290.1	GAP1-N2+GAP1-M+GAP1-C+polar_tail+TM+beta-rich	TM+vWA(canonical)+YtKA-like+TM+??→?→ Tubulin+coiled_coil+??→ Tubulin+coiled_coil+HTH?+??→ TM+TM+TM+vWA(canonical)+TM+TM+TM+polar_tail→ Fibronectin-like_repeats+??→ ZnR+ZnR+DO-GTPase2.2+ZnR+ZnR→ GAP1-N2+GAP1-M+GAP1-C+polar_tail+TM+beta-rich*→	991	Paenibacillus lautus	GCA_001955975.1	firmicutes
WP_076332474.1	GAP1-N2+GAP1-M+GAP1-C+polar_tail+TM+beta-rich	TM+vWA(canonical)+YtKA-like+TM+??→ Tubulin+coiled_coil+??→ Tubulin+coiled_coil+HTH?+??→ TM+TM+TM+vWA(canonical)+TM+TM+TM+polar_tail→ Fibronectin-like_repeats+??→ ZnR+ZnR+DO-GTPase2.2+ZnR+ZnR→ GAP1-N2+GAP1-M+GAP1-C+polar_tail+TM+beta-rich*→	1061	Paenibacillus amylolyticus	GCA_001956055.1	firmicutes
WP_076335950.1	GAP1-N2+GAP1-M+GAP1-C+polar_tail+TM+beta-rich	TM+vWA(canonical)+YtKA-like+TM+??→ Tubulin+coiled_coil+??→ Tubulin+coiled_coil+HTH?+??→ TM+TM+TM+vWA(canonical)+TM+TM+TM+polar_tail→ Fibronectin-like_repeats+??→ ZnR+ZnR+DO-GTPase2.2+ZnR+ZnR→ GAP1-N2+GAP1-M+GAP1-C+polar_tail+TM+beta-rich*→	1004	Paenibacillus sp. FSL H8-0548	GCA_001956095.1	firmicutes
WP_076344520.1	GAP1-N2+GAP1-M+GAP1-C	ZnR+ZnR+DO-GTPase2.2→ GAP1-N2+GAP1-M+GAP1-C*→??+TM+??+beta-rich??→	788	Paludisphaera borealis	GCA_001956985.1	planctomycetes
WP_076347977.1	GAP1-N2+GAP1-M	GAP1-N2+GAP1-M*→	231	Paludisphaera borealis	GCA_001956985.1	planctomycetes
WP_076683998.1	GAP1-N2+GAP1-M+GAP1-C	GAP1-N2+GAP1-M+GAP1-C*→ vWA(canonical)→ PP2C→ Pkinase+ZnR+beta-rich→	790	Streptomyces alfalfae	GCA_001975025.1	actinobacteria
WP_076780654.1	GAP1-N2+GAP1-M+TM+TM	PP2C→?→ DO-GTPase2.1→ GAP1-N2+GAP1-M+TM+TM*→	389	Lachnoclostridium phocaeense	GCF_900120345.1	firmicutes
WP_076820656.1	GAP1-N2+GAP1-M+GAP1-C	GrpE→ HSP70→ TPR_containing_alpha_helical+Fibronectin-like_repeats→ ZnR+ZnR+DO-GTPase2.2→ GAP1-N2+GAP1-M+GAP1-C*→ TM+TM+TM+TM+ZnR+ZnR+DO-GTPase2.2→	879	Frankia asymbiotica	GCA_001983105.1	actinobacteria
WP_076827099.1	GAP1-N2+GAP1-M+GAP1-C+ZnR+DO-GTPase2	GAP1-N2+GAP1-M+GAP1-C+ZnR+DO-GTPase2*→	984	Frankia sp. CcI49	GCA_001983215.1	actinobacteria
WP_076966755.1	GAP1-N2+GAP1-M+GAP1-C	TPR_containing_alpha_helical+Fibronectin-like_repeats→ ZnR+ZnR+DO-GTPase2.2→ GAP1-N2+GAP1-M+GAP1-C*→ TM+TM+TM+TM+ZnR+ZnR+DO-GTPase2.2+TM→ vWA(canonical)→ PP2C→ Pkinase+ZnR+beta-rich→	846	Streptomyces sp. IB2014 011-1	GCA_001983595.1	actinobacteria
WP_077008701.1	GAP1-N2+GAP1-M+GAP1-C+TM+beta-rich	vWA(canonical)+beta-rich+low_complexity→?→?→ Tubulin+alpha_helical+??+??→??+TM+TM+TM+TM+??→ Pkinase+TM→ ZnR+ZnR+DO-GTPase2.2→ GAP1-N2+GAP1-M+GAP1-C+TM+beta-rich*→	610	Saccharothrix sp. ALI-22-I	GCA_001984175.1	actinobacteria
WP_077026942.1	GAP1-N2+GAP1-M+TM	ZnR+ZnR+DO-GTPase2.2→ GAP1-N2+GAP1-M+TM*→	370	Fuerstia marisgermanicae	GCA_001983935.1	planctomycetes
WP_077061502.1	GAP1-N2+GAP1-M+GAP1-C	GAP1-N2+GAP1-M+GAP1-C*→ vWA(canonical)→ PP2C→ Pkinase+ZnR+beta-rich→||←TPR_containing_alpha_helical+Fibronectin-like_repeats	501	Streptomyces sp. MP131-18	GCA_001984575.1	actinobacteria
WP_077076330.1	GAP1-N2+GAP1-M+GAP1-C	TM+TM+TM+TM+TM+TM+DO-GTPase2.1→ GAP1-N2+GAP1-M+GAP1-C*→??→ TM_region+DO-GTPase1→	556	Cuniculiplasma divulgatum	GCA_900090055.1	euryarchaeota
WP_077092367.1	GAP1-M+GAP1-C	TM+DUF4190+TM→ TM+DUF4190+TM→ polar_leader+TM+TM+TM→ TM+vWA(canonical)+Fibronectin-like_repeats+TM+beta-rich+lc_tail→ PSE→ GAP_unknown1+TM+TM+GAP_unknown2→ HRDC-like?+Fibronectin-like_repeats→ PSE→ GAP1-M+GAP1-C*→ PSE→?→ beta-rich+vWA(canonical)→??+DO-GTPase2.2→ TM+DUF4190+TM+PknH_C→ TM+DUF4190+TM+PknH_C→ TM+DUF4190+TM+PknH_C→ TM+DUF4190+TM+PknH_C→	669	Mycobacterium colombiense	-	actinobacteria
WP_077313322.1	GAP1-N2+GAP1-M+GAP1-C	polar_leader+TM+TM+TM→ TM+vWA(canonical)+Fibronectin-like_repeats+TM+beta-rich+lc_tail→ Tubulin+alpha_helical+??+??→ GAP_unknown1+TM+TM+GAP_unknown2→ HRDC-like?+Fibronectin-like_repeats→ GAP1-N2+GAP1-M+GAP1-C*→ beta-rich+vWA(canonical)→??+DO-GTPase2.2→	631	Corynebacterium glutamicum	GCA_000742735.1	actinobacteria
WP_077343605.1	GAP1-N2+GAP1-M+GAP1-C	polar_leader+TM+TM+TM→ TM+vWA(canonical)+Fibronectin-like_repeats+TM+beta-rich+lc_tail→ Tubulin+alpha_helical+??+??→ GAP_unknown1+TM+TM+GAP_unknown2→ HRDC-like?+Fibronectin-like_repeats→ GAP1-N2+GAP1-M+GAP1-C*→?→??+DO-GTPase2.2→	864	Tessaracoccus flavus	GCA_001997295.1	actinobacteria
WP_077349637.1	GAP1-N2+GAP1-M+GAP1-C	polar_leader+TM+TM+TM→ TM+vWA(canonical)+Fibronectin-like_repeats+TM+beta-rich+lc_tail→ Tubulin+alpha_helical+??+??→ GAP_unknown1+TM+TM+GAP_unknown2→?→ HRDC-like?+Fibronectin-like_repeats→ GAP1-N2+GAP1-M+GAP1-C*→?→??+DO-GTPase2.2→	456	Tessaracoccus flavescens	GCA_001998865.1	actinobacteria
WP_077686749.1	GAP1-N2+GAP1-M+GAP1-C	??+TM+vWA(canonical)+Fibronectin-like_repeats+TM+beta-rich+lc_tail→ Tubulin+alpha_helical+??+??→ GAP_unknown1+TM+TM+GAP_unknown2→?→?→ HRDC-like?+Fibronectin-like_repeats→ GAP1-N2+GAP1-M+GAP1-C*→?→??+DO-GTPase2.2→	455	Tessaracoccus aquimaris	GCA_001997345.1	actinobacteria
WP_077693371.1	GAP1-N2+GAP1-M+GAP1-C	TPR_containing_alpha_helical+Fibronectin-like_repeats+extended_insert+Fibronectin-like_repeats→ ZnR+ZnR+DO-GTPase2.2→ GAP1-N2+GAP1-M+GAP1-C*→	932	Nocardiopsis sinuspersici	GCA_001998325.1	actinobacteria
WP_077739729.1	GAP1-N2+GAP1-M+GAP1-C	HRDC-like?+Fibronectin-like_repeats→ GAP1-N2+GAP1-M+GAP1-C*→??+DO-GTPase2.2→	819	Mycolicibacterium boenickei	GCA_002553535.1	actinobacteria
WP_077936954.1	GAP1-N2+GAP1-M+GAP1-C	GrpE→ HSP70→ TPR_containing_alpha_helical+Fibronectin-like_repeats→ ZnR+ZnR+DO-GTPase2.2→ GAP1-N2+GAP1-M+GAP1-C*→ TM+TM+TM+TM+ZnR+ZnR+DO-GTPase2.2→	814	Micromonospora sp. Rc5	GCA_002006875.1	actinobacteria
WP_078074070.1	GAP1-N2+GAP1-M+GAP1-C	GAP1-N2+GAP1-M+GAP1-C*→ vWA(canonical)→ PP2C→ Pkinase+ZnR+beta-rich→||←TPR_containing_alpha_helical+Fibronectin-like_repeats	866	Streptomyces niveus	GCA_002009175.1	actinobacteria
WP_078501498.1	GAP1-N2+GAP1-M+GAP1-C+polar_tail+TM+beta-rich	TM+vWA(canonical)+YtKA-like+TM+??→ Tubulin+coiled_coil+??→ Tubulin+coiled_coil+HTH?+??→ TM+TM+TM+vWA(canonical)+TM+TM+TM+polar_tail→ Fibronectin-like_repeats+??→ ZnR+ZnR+DO-GTPase2.2+ZnR+ZnR→ GAP1-N2+GAP1-M+GAP1-C+polar_tail+TM+beta-rich*→	968	Paenibacillus selenitireducens	GCA_002021565.1	firmicutes
WP_078533238.1	GAP1-N2+GAP1-M+GAP1-C	TM+TM+TM+TM+ZnR+ZnR+DO-GTPase2.2+TM→ vWA(canonical)→ PP2C→ Pkinase+ZnR+beta-rich→||←?←?←?||GAP1-N2+GAP1-M+GAP1-C*→	813	Streptomyces sp. ScaeMP-e10	GCF_000373405.1	actinobacteria
WP_078543661.1	GAP1-N2+GAP1-M+GAP1-C	GrpE→ HSP70→ HSP70+vWA→ DnaJ+DnaJ+ZnR+ZnR+coiled_coil+Fibronectin-like_repeats→ ZnR+ZnR+DO-GTPase2.2→ GAP1-N2+GAP1-M+GAP1-C*→ TM+TM+TM+TM+TM+TM+TM+TM+ZnR+ZnR+DO-GTPase2.2→	859	Bacillus alkalitelluris	GCF_002019645.1	firmicutes
WP_078567012.1	GAP1-N2+GAP1-M+GAP1-C	GAP1-N2+GAP1-M+GAP1-C*→	835	Streptomyces sp. CNS654	GCF_000702365.1	actinobacteria
WP_078580392.1	GAP1-N2+GAP1-M+GAP1-C	GAP1-N2+GAP1-M+GAP1-C*→	848	Streptomyces	GCF_000717015.1	actinobacteria
WP_078585189.1	GAP1-N2+GAP1-M+GAP1-C	GAP1-N2+GAP1-M+GAP1-C*→	822	Streptomyces anulatus	GCF_000717105.1	actinobacteria
WP_078608964.1	GAP1-N2+GAP1-M+GAP1-C	GAP1-N2+GAP1-M+GAP1-C*→	845	Streptomyces floridae	GCF_000717665.1	actinobacteria
WP_078611209.1	GAP1-N2+GAP1-M+GAP1-C	GAP1-N2+GAP1-M+GAP1-C*→	840	Streptomyces baarnensis	GCF_000716025.1	actinobacteria
WP_078612349.1	GAP1-N2+GAP1-M+GAP1-C	GAP1-N2+GAP1-M+GAP1-C*→	848	Streptomyces vinaceus	GCF_000717215.1	actinobacteria

<table>
<tr><th>acc</th><th>architecture</th><th>operon</th><th>len</th><th>species</th><th>gca</th><th>taxend</th></tr>
<tr><td>WP_078613167.1</td><td>GAP1-N2+GAP1-M</td><td>-</td><td>834</td><td>Streptomyces katrae</td><td>-</td><td>actinobacteria</td></tr>
<tr><td>WP_078615787.1</td><td>GAP1-N2+GAP1-M+GAP1-C</td><td>TM+TM+TM+TM+ZnR+ZnR+DO-GTPase2.2+TM→ vWA(canonical)→ PP2C→ Pkinase+ZnR+beta-rich→||←?←?←?||GAP1-N2+GAP1-M+GAP1-C*→</td><td>813</td><td>Streptomyces cyaneofuscatus</td><td>GCF_000718135.1</td><td>actinobacteria</td></tr>
<tr><td>WP_078621939.1</td><td>GAP1-N2+GAP1-M</td><td>-</td><td>865</td><td>Streptomyces sp. NRRL S-244</td><td>-</td><td>actinobacteria</td></tr>
<tr><td>WP_078625710.1</td><td>GAP1-N2+GAP1-M+GAP1-C</td><td>GAP1-N2+GAP1-M+GAP1-C*→</td><td>837</td><td>Streptomyces sp. CNB091</td><td>GCF_000377965.1</td><td>actinobacteria</td></tr>
<tr><td>WP_078630913.1</td><td>GAP1-N2+GAP1-M+GAP1-C</td><td>GAP1-N2+GAP1-M+GAP1-C*→</td><td>845</td><td>Streptomyces</td><td>GCF_000718915.1</td><td>actinobacteria</td></tr>
<tr><td>WP_078652198.1</td><td>GAP1-N2+GAP1-M+GAP1-C</td><td>GAP1-N2+GAP1-M+GAP1-C*→</td><td>836</td><td>Streptomyces globisporus</td><td>GCF_000718455.1</td><td>actinobacteria</td></tr>
<tr><td>WP_078654581.1</td><td>GAP1-N2+GAP1-M+GAP1-C</td><td>GAP1-N2+GAP1-M+GAP1-C*→</td><td>835</td><td>Streptomyces albus</td><td>GCF_000716945.1</td><td>actinobacteria</td></tr>
<tr><td>WP_078657336.1</td><td>GAP1-N2+GAP1-M+GAP1-C</td><td>GAP1-N2+GAP1-M+GAP1-C*→</td><td>812</td><td>Streptomyces</td><td>GCF_002633395.1</td><td>actinobacteria</td></tr>
<tr><td>WP_078696361.1</td><td>GAP1-N2+GAP1-M+GAP1-C</td><td>PNPase→?→ GAP1-N2+GAP1-M+GAP1-C*→</td><td>826</td><td>Caloramator quimbayensis</td><td>GCA_900167605.1</td><td>firmicutes</td></tr>
<tr><td>WP_078845919.1</td><td>GAP1-N2+GAP1-M+GAP1-C</td><td>GAP1-N2+GAP1-M+GAP1-C*→</td><td>841</td><td>Streptomyces albus</td><td>GCF_000720915.1</td><td>actinobacteria</td></tr>
<tr><td>WP_078859204.1</td><td>GAP1-N2+GAP1-M+GAP1-C</td><td>GAP1-N2+GAP1-M+GAP1-C*→</td><td>845</td><td>Streptomyces sp. NRRL F-2202</td><td>GCF_000719925.1</td><td>actinobacteria</td></tr>
<tr><td>WP_078860453.1</td><td>GAP1-N2+GAP1-M</td><td>-</td><td>841</td><td>Streptomyces sp. NRRL F-2747</td><td>-</td><td>actinobacteria</td></tr>
<tr><td>WP_078866750.1</td><td>GAP1-N2+GAP1-M+GAP1-C</td><td>GAP1-N2+GAP1-M+GAP1-C*→</td><td>845</td><td>Streptomyces griseus group</td><td>GCF_000718695.1</td><td>actinobacteria</td></tr>
<tr><td>WP_078874345.1</td><td>GAP1-N2+GAP1-M+GAP1-C</td><td>GAP1-N2+GAP1-M+GAP1-C*→</td><td>848</td><td>Streptomyces sp. NRRL F-5702</td><td>GCF_000721415.1</td><td>actinobacteria</td></tr>
<tr><td>WP_078879411.1</td><td>GAP1-N2+GAP1-M+GAP1-C</td><td>GAP1-N2+GAP1-M+GAP1-C*→</td><td>848</td><td>Streptomyces sp. NRRL F-5681</td><td>GCF_000719585.1</td><td>actinobacteria</td></tr>
<tr><td>WP_078883114.1</td><td>GAP1-N2+GAP1-M+GAP1-C</td><td>GAP1-N2+GAP1-M+GAP1-C*→</td><td>848</td><td>Streptomyces</td><td>GCF_000720065.1</td><td>actinobacteria</td></tr>
<tr><td>WP_078890683.1</td><td>GAP1-N2+GAP1-M+GAP1-C</td><td>GAP1-N2+GAP1-M+GAP1-C*→</td><td>848</td><td>Streptomyces sp. NRRL B-1381</td><td>GCF_000719655.1</td><td>actinobacteria</td></tr>
<tr><td>WP_078908052.1</td><td>GAP1-N2+GAP1-M+GAP1-C</td><td>GAP1-N2+GAP1-M+GAP1-C*→</td><td>812</td><td>Streptomyces sp. NRRL S-623</td><td>GCF_000725705.1</td><td>actinobacteria</td></tr>
<tr><td>WP_078939583.1</td><td>GAP1-N2+GAP1-M+GAP1-C</td><td>GAP1-N2+GAP1-M+GAP1-C*→</td><td>845</td><td>Streptomyces sp. NRRL F-2295</td><td>GCF_001279425.1</td><td>actinobacteria</td></tr>
<tr><td>WP_078951059.1</td><td>GAP1-N2+GAP1-M+GAP1-C</td><td>GAP1-N2+GAP1-M+GAP1-C*→</td><td>848</td><td>Streptomyces griseus</td><td>GCF_000718235.1</td><td>actinobacteria</td></tr>
<tr><td>WP_078955659.1</td><td>GAP1-N2+GAP1-M+GAP1-C</td><td>GAP1-N2+GAP1-M+GAP1-C*→</td><td>826</td><td>Streptomyces griseus group</td><td>GCA_003147545.1</td><td>actinobacteria</td></tr>
<tr><td>WP_078968860.1</td><td>GAP1-N2+GAP1-M+GAP1-C</td><td>GAP1-N2+GAP1-M+GAP1-C*→</td><td>845</td><td>Streptomyces sp. MMG1522</td><td>GCF_001279735.1</td><td>actinobacteria</td></tr>
<tr><td>WP_078987566.1</td><td>GAP1-N2+GAP1-M</td><td>-</td><td>886</td><td>Streptomyces sp. XY332</td><td>-</td><td>actinobacteria</td></tr>
<tr><td>WP_079036103.1</td><td>GAP1-N2+GAP1-M+GAP1-C</td><td>GAP1-N2+GAP1-M+GAP1-C*→</td><td>811</td><td>Streptomyces luridiscabiei</td><td>GCF_001418625.1</td><td>actinobacteria</td></tr>
<tr><td>WP_079133999.1</td><td>GAP1-N2+GAP1-M+GAP1-C</td><td>GAP1-N2+GAP1-M+GAP1-C*→</td><td>838</td><td>Streptomyces sp. EN23</td><td>GCF_001746305.1</td><td>actinobacteria</td></tr>
<tr><td>WP_079137652.1</td><td>GAP1-N2+GAP1-M+GAP1-C</td><td>GAP1-N2+GAP1-M+GAP1-C*→</td><td>826</td><td>Streptomyces sp. PTY087I2</td><td>GCA_001687325.1</td><td>actinobacteria</td></tr>
<tr><td>WP_079149733.1</td><td>GAP1-N2+GAP1-M+GAP1-C</td><td>GAP1-N2+GAP1-M+GAP1-C*→</td><td>846</td><td>Streptomyces sp. EN27</td><td>GCF_001746315.1</td><td>actinobacteria</td></tr>
<tr><td>WP_079181260.1</td><td>GAP1-N2+GAP1-M+GAP1-C</td><td>TM+TM+TM+TM+ZnR+ZnR+DO-GTPase2.2+TM→ vWA(canonical)→ PP2C→ Pkinase+ZnR+beta-rich→||←?←?←?||GAP1-N2+GAP1-M+GAP1-C*→</td><td>812</td><td>Streptomyces sp. CB00316</td><td>GCF_001905595.1</td><td>actinobacteria</td></tr>
<tr><td>WP_079181421.1</td><td>GAP1-N2+GAP1-M+GAP1-C</td><td>GAP1-N2+GAP1-M+GAP1-C*→</td><td>826</td><td>Streptomyces sp. Tue 6075</td><td>GCF_001931635.1</td><td>actinobacteria</td></tr>
<tr><td>WP_079185375.1</td><td>GAP1-N2+GAP1-M+GAP1-C</td><td>GAP1-N2+GAP1-M+GAP1-C*→</td><td>822</td><td>Streptomyces sp. TSRI0395</td><td>GCF_001905525.1</td><td>actinobacteria</td></tr>
<tr><td>WP_079190473.1</td><td>GAP1-N2+GAP1-M+GAP1-C</td><td>GAP1-N2+GAP1-M+GAP1-C*→</td><td>835</td><td>Streptomyces sp. CB02366</td><td>GCF_001905905.1</td><td>actinobacteria</td></tr>
<tr><td>WP_079251362.1</td><td>GAP1-N2+GAP1-M+GAP1-C</td><td>TM+TM+TM+TM+ZnR+ZnR+DO-GTPase2.2+TM→ vWA(canonical)→ PP2C→ Pkinase+ZnR+beta-rich→||←?←?←?||GAP1-N2+GAP1-M+GAP1-C*→</td><td>812</td><td>Streptomyces sp. IB2014 011-1</td><td>GCA_003367645.1</td><td>actinobacteria</td></tr>
<tr><td>WP_079277259.1</td><td>GAP1-N2+GAP1-M+GAP1-C</td><td>GAP1-N2+GAP1-M+GAP1-C*→</td><td>838</td><td>Streptomyces sp. TSRI0261</td><td>GCA_001905485.1</td><td>actinobacteria</td></tr>
<tr><td>WP_079347757.1</td><td>GAP1-N2+GAP1-M+GAP1-C+polar_tail+TM+beta-rich</td><td>TM+vWA(canonical)+YtKA-like+TM+??→ Tubulin+coiled_coil+??→ PSE→ TM+TM+TM+vWA(canonical)+TM+TM+TM+polar_tail→ Fibronectin-like_repeats+??→ ZnR+ZnR+DO-GTPase2.2+ZnR+ZnR→ GAP1-N2+GAP1-M+GAP1-C+polar_tail+TM+beta-rich*→</td><td>1070</td><td>Paenibacillus sp. VT-16-81</td><td>GCA_002027255.1</td><td>firmicutes</td></tr>
<tr><td>WP_079430303.1</td><td>GAP1-N2+GAP1-M+GAP1-C</td><td>GAP1-N2+GAP1-M+GAP1-C*→</td><td>618</td><td>Streptomyces katrae</td><td>GCA_003814735.1</td><td>actinobacteria</td></tr>
<tr><td>WP_079695570.1</td><td>GAP1-N2+GAP1-M+GAP1-C+polar_tail+TM+beta-rich</td><td>TM+vWA(canonical)+YtKA-like+TM+??→ Tubulin+coiled_coil+??→ Tubulin+coiled_coil+HTH?+??→ TM+TM+TM+vWA(canonical)+TM+TM+TM+polar_tail→ Fibronectin-like_repeats+??→ ZnR+ZnR+DO-GTPase2.2+ZnR+ZnR→ GAP1-N2+GAP1-M+GAP1-C+polar_tail+TM+beta-rich*→</td><td>1057</td><td>Paenibacillus</td><td>GCA_900168305.1</td><td>firmicutes</td></tr>
<tr><td>WP_079913482.1</td><td>GAP1-N2+GAP1-M+GAP1-C+polar_tail+TM+Cadherin-like</td><td>TM+vWA(canonical)+YtKA-like+TM+??→ Tubulin+coiled_coil+??→ Tubulin+coiled_coil+HTH?+??→ TM+TM+TM+vWA(canonical)+TM+TM+TM+polar_tail→ Fibronectin-like_repeats+??→ ZnR+ZnR+DO-GTPase2.2+ZnR+ZnR→ GAP1-N2+GAP1-M+GAP1-C+polar_tail+TM+Cadherin-like*→</td><td>974</td><td>Paenibacillus sp. 32352</td><td>GCF_002042965.1</td><td>firmicutes</td></tr>
<tr><td>WP_080793004.1</td><td>GAP1-N2+GAP1-M+GAP1-C</td><td>TM+TM+TM→ TM+vWA(canonical)+Fibronectin-like_repeats+TM+beta-rich+lc_tail→ Tubulin+alpha_helical+??+??→ GAP_unknown1+TM+TM+GAP_unknown2→ HRDC-like?+Fibronectin-like_repeats→ GAP1-N2+GAP1-M+GAP1-C*→ beta-rich+vWA(canonical)→??+DO-GTPase2.2→ polar_leader+TM+TM+TM→</td><td>529</td><td>Corynebacterium sp.
Marseille-P2417</td><td>GCF_900169525.1</td><td>actinobacteria</td></tr>
<tr><td>WP_080807098.1</td><td>GAP1-N2+GAP1-M+GAP1-C</td><td>GAP1-N2+GAP1-M+GAP1-C*→??→</td><td>984</td><td>Desulfamplus magnetovallimortis</td><td>GCA_900170035.1</td><td>deltaproteobacteria</td></tr>
<tr><td>WP_081099609.1</td><td>GAP1-N2+GAP1-M+GAP1-C</td><td>polar_leader+TM+TM+TM→ TM+vWA(canonical)+Fibronectin-like_repeats+TM+beta-rich+lc_tail→ Tubulin+alpha_helical+??+??→ GAP_unknown1+TM+TM+GAP_unknown2→ HRDC-like?+Fibronectin-like_repeats→ GAP1-N2+GAP1-M+GAP1-C*→ beta-rich+vWA(canonical)→??+DO-GTPase2.2→</td><td>631</td><td>Corynebacterium glutamicum</td><td>GCF_001518935.2</td><td>actinobacteria</td></tr>
<tr><td>WP_081558132.1</td><td>GAP1-N2+GAP1-M+GAP1-C</td><td>polar_leader+TM+TM+TM→ TM+vWA(canonical)+Fibronectin-like_repeats+TM+beta-rich+lc_tail→ Tubulin+alpha_helical+??+??→ GAP_unknown1+TM+TM+GAP_unknown2→ HRDC-like?+Fibronectin-like_repeats→ GAP1-N2+GAP1-M+GAP1-C*→ beta-rich+vWA(canonical)→??+DO-GTPase2.2→</td><td>992</td><td>Rhodococcus sp. BH4</td><td>GCA_002079265.1</td><td>actinobacteria</td></tr>
<tr><td>WP_082276470.1</td><td>GAP1-N2+GAP1-M+GAP1-C</td><td>GAP_unknown1+TM+TM+GAP_unknown2→ HRDC-like?+Fibronectin-like_repeats→ GAP1-N2+GAP1-M+GAP1-C*→ GAP1-M→ GAP1-N2+GAP1-M+GAP1-C→ beta-rich+vWA(canonical)→??+DO-GTPase2.2→</td><td>150</td><td>Mycobacterium kansasii</td><td>GCA_002003625.1</td><td>actinobacteria</td></tr>
<tr><td>WP_082868838.1</td><td>GAP1-N2+GAP1-M+GAP1-C</td><td>TM+TM+TM→ PSE→ Tubulin+alpha_helical+??+??→ GAP_unknown1+TM+TM+GAP_unknown2→ HRDC-like?+Fibronectin-like_repeats→ GAP1-N2+GAP1-M+GAP1-C*→ beta-rich+vWA(canonical)→</td><td>652</td><td>Corynebacterium crudilactis</td><td>GCF_001643015.1</td><td>actinobacteria</td></tr>
<tr><td>WP_083030877.1</td><td>GAP1-N2+GAP1-M+GAP1-C</td><td>TM+DUF4190+TM→ TM+DUF4190+TM→ polar_leader+TM+TM+TM→ PSE→ Tubulin+alpha_helical+??+??→||←PSE||PSE→ GAP1-N2+GAP1-M+GAP1-C*→ beta-rich+vWA(canonical)→??+DO-GTPase2.2→</td><td>827</td><td>Mycolicibacterium insubricum</td><td>GCA_002086255.1</td><td>actinobacteria</td></tr>
<tr><td>WP_083099237.1</td><td>GAP1-N2+GAP1-M+GAP1-C</td><td>TM+DUF4190+TM→ TM+DUF4190+TM→ polar_leader+TM+TM+TM→ TM+vWA(canonical)+Fibronectin-like_repeats+TM+beta-rich+lc_tail→ Tubulin+alpha_helical+??+??→ GAP_unknown1+TM+TM+GAP_unknown2→ HRDC-like?+Fibronectin-like_repeats→ GAP1-N2+GAP1-M+GAP1-C*→ beta-rich+vWA(canonical)→??+DO-GTPase2.2→ TM+DUF4190+TM+PknH_C→ TM+DUF4190+TM+PknH_C→
TM+DUF4190+TM+PknH_C→ TM+DUF4190+TM+PknH_C→</td><td>829</td><td>Mycobacterium mantenii</td><td>GCA_002086335.1</td><td>actinobacteria</td></tr>
<tr><td>WP_083156298.1</td><td>GAP1-N2+GAP1-M+GAP1-C</td><td>GAP1-N2+GAP1-M+GAP1-C*→ beta-rich+vWA(canonical)→??+DO-GTPase2.2→</td><td>818</td><td>Mycobacterium persicum</td><td>GCA_002086675.1</td><td>actinobacteria</td></tr>
<tr><td>WP_083164133.1</td><td>GAP1-N2+GAP1-M+GAP1-C</td><td>TM+DUF4190+TM→ polar_leader+TM+TM+TM→ TM+vWA(canonical)+Fibronectin-like_repeats+TM+beta-rich+lc_tail→ Tubulin+alpha_helical+??+??→ GAP_unknown1+TM+TM+GAP_unknown2→ HRDC-like?+Fibronectin-like_repeats→ GAP1-N2+GAP1-M+GAP1-C*→ beta-rich+vWA(canonical)→??+DO-GTPase2.2→ TM+DUF4190+TM+PknH_C→ TM+DUF4190+TM+PknH_C→ TM+DUF4190+TM+PknH_C→</td><td>828</td><td>Mycobacterium aquaticum</td><td>GCA_002086485.1</td><td>actinobacteria</td></tr>
<tr><td>WP_083193497.1</td><td>GAP1-N2+GAP1-M+GAP1-C</td><td>GAP1-N2+GAP1-M+GAP1-C*→</td><td>845</td><td>Streptomyces violaceoruber</td><td>GCA_002082175.1</td><td>actinobacteria</td></tr>
<tr><td>WP_084131735.1</td><td>GAP1-N2+GAP1-M+GAP1-C+ZnR+DO-GTPase2</td><td>GAP1-N2+GAP1-M+GAP1-C+ZnR+DO-GTPase2*→</td><td>885</td><td>Frankia sp. Cc1.17</td><td>GCF_001854655.1</td><td>actinobacteria</td></tr>
<tr><td>WP_084424213.1</td><td>GAP1-N2+GAP1-M+GAP1-C</td><td>GrpE→ HSP70→ TPR_containing_alpha_helical+Fibronectin-like_repeats→ ZnR+ZnR+DO-GTPase2.2→ GAP1-N2+GAP1-M+GAP1-C*→ TM+TM+TM+TM+ZnR+ZnR+DO-GTPase2.2→?→ HSP70→ vWA(canonical)→ unknown+CASPASE→ HSP70→</td><td>819</td><td>Kibdelosporangium aridum</td><td>GCA_900176515.1</td><td>actinobacteria</td></tr>
</table>

acc	architecture	operon	len	species	gca	taxend
WP_084751128.1	GAP1-N2+GAP1-M+GAP1-C	GAP1-N2+GAP1-M+GAP1-C*→	813	Kitasatospora albolonga	GCA_002082585.1	actinobacteria
WP_084848445.1	GAP1-N2+GAP1-M+GAP1-C	polar_leader+TM+TM+TM→ TM+vWA(canonical)+Fibronectin-like_repeats+TM+beta-rich+lc_tail→ Tubulin+alpha_helical+??+??→ GAP_unknown1+TM+TM+GAP_unknown2→ HRDC-like?+Fibronectin-like_repeats→ GAP1-N2+GAP1-M+GAP1-C*→ beta-rich+vWA(canonical)→??+DO-GTPase2.2→	842	Rhodococcus hoagii	GCA_003013675.1	actinobacteria
WP_084991422.1	GAP1-N2+GAP1-M	-	877	Streptomyces sp. S8	-	actinobacteria
WP_085020611.1	GAP1-N2+GAP1-M+low_complexity+TM+TM	??→ PP2C→ Pkinase+helical_region+TM_region→ ZnR+ZnR+DO-GTPase2.2→ GAP1-N2+GAP1-M+low_complexity+TM+TM*→	536	Cnuibacter physcomitrellae	GCA_002096055.1	actinobacteria
WP_085086797.1	GAP1-N2+GAP1-M+GAP1-C	HSP70→ HSP70+vWA(canonical)→?→ DO-GTPase2.2(frag)→ GAP1-N2+GAP1-M+GAP1-C*→?→ TM+TM+TM+TM+ZnR+ZnR+DO-GTPase2.2→	865	Azospirillum oryzae	GCA_900177515.1	alphaproteobacteria
WP_085096640.1	GAP1-N2+GAP1-M+GAP1-C	TM+DUF4190+TM→ TM+DUF4190+TM→ polar_leader+TM+TM+TM→ TM+vWA(canonical)+Fibronectin-like_repeats+TM+beta-rich+lc_tail→ Tubulin+alpha_helical+??+??→ GAP_unknown1+TM+TM+GAP_unknown2→ HRDC-like?+Fibronectin-like_repeats→ GAP1-N2+GAP1-M+GAP1-C*→ beta-rich+vWA(canonical)→??+DO-GTPase2.2→	807	Mycolicibacterium fallax	GCA_002101995.1	actinobacteria
WP_085140691.1	GAP1-N2+GAP1-M+GAP1-C	HRDC-like?+Fibronectin-like_repeats→ GAP1-N2+GAP1-M+GAP1-C*→??+DO-GTPase2.2→	819	Mycolicibacterium conceptionense	GCA_001052995.1	actinobacteria
WP_085143633.1	GAP1-N2+GAP1-M+GAP1-C	HRDC-like?+Fibronectin-like_repeats→ GAP1-N2+GAP1-M+GAP1-C*→??+DO-GTPase2.2→	820	Mycolicibacterium wolinskyi	GCA_002101965.1	actinobacteria
WP_085151380.1	GAP1-N2+GAP1-M+GAP1-C	Tubulin+alpha_helical+??+??→ GAP_unknown1+TM+TM+GAP_unknown2→ HRDC-like?+Fibronectin-like_repeats→ GAP1-N2+GAP1-M+GAP1-C*→ beta-rich+vWA(canonical)→ ZnR+ZnR+DO-GTPase2.2→	840	Mycolicibacterium confluentis	GCA_002102105.1	actinobacteria
WP_085190682.1	GAP1-N2+GAP1-M+GAP1-C	HRDC-like?+Fibronectin-like_repeats→ GAP1-N2+GAP1-M+GAP1-C*→??+DO-GTPase2.2→	833	Mycolicibacterium doricum	GCA_002102115.1	actinobacteria
WP_085470330.1	GAP1-N2+GAP1-M+GAP1-C	polar_leader+TM+TM+TM→ TM+vWA(canonical)+Fibronectin-like_repeats+TM+beta-rich+lc_tail→ Tubulin+alpha_helical+??+??→ GAP_unknown1+TM+TM+GAP_unknown2→ HRDC-like?+Fibronectin-like_repeats→ GAP1-N2+GAP1-M+GAP1-C*→ beta-rich+vWA(canonical)→??+DO-GTPase2.2→	909	Rhodococcus rhodochrous	GCA_900177695.1	actinobacteria
WP_085478530.1	GAP1-N2+GAP1-M+GAP1-C	GAP1-N2+GAP1-M+GAP1-C*→	789	Streptomyces albidoflavus	GCA_004196125.1	actinobacteria
WP_085494889.1	GAP1-N2+GAP1-M+GAP1-C+polar_tail+TM+beta-rich	TM+vWA(canonical)+YtKA-like+TM+??→ Tubulin+coiled_coil+??→ Tubulin+coiled_coil+HTH?+??→ TM+TM+TM+vWA(canonical)+TM+TM+TM+polar_tail→ Fibronectin-like_repeats+??→ ZnR+ZnR+DO-GTPase2.2+ZnR+ZnR→ GAP1-N2+GAP1-M+GAP1-C+polar_tail+TM+beta-rich*→	974	Paenibacillus aquistagni	GCA_900177815.1	firmicutes
WP_085549323.1	GAP1-N2+GAP1-M+GAP1-C	TM+vWA(canonical)+Fibronectin-like_repeats+TM+beta-rich+lc_tail→ Tubulin+alpha_helical+??+??→ GAP_unknown1+TM+TM+GAP_unknown2→ HRDC-like?+Fibronectin-like_repeats→ GAP1-N2+GAP1-M+GAP1-C*→ beta-rich+vWA(canonical)→??+DO-GTPase2.2→	653	Corynebacterium pollutisoli	GCA_900177745.1	actinobacteria
WP_086160702.1	GAP1-N2+GAP1-M+GAP1-C	GAP1-N2+GAP1-M+GAP1-C*→ PSE→ PP2C→ Pkinase+ZnR+beta-rich→?→?→||←TPR_containing_alpha_helical+Fibronectin-like_repeats	520	Streptomyces sp. SCSIO 03032	GCA_002128305.1	actinobacteria
WP_086665133.1	GAP1-N2+GAP1-M+SLATT+SLATT	vWA(canonical)+beta-rich+low_complexity→ Tubulin+alpha_helical+??+??→??+TM+TM+TM+TM+??→ Pkinase+TM→ ZnR+ZnR+DO-GTPase2.2→ GAP1-N2+GAP1-M+SLATT+SLATT*→ TIR+??→?→?→ STAND_NTPase+TPR_region→	634	Lentzea kentuckyensis	GCF_002150765.1	actinobacteria
WP_086671185.1	GAP1-N2+GAP1-M+GAP1-C	GAP1-N2+GAP1-M+GAP1-C*→	826	Streptomyces albovinaceus	GCF_002154385.1	actinobacteria
WP_086778841.1	GAP1-N2+GAP1-M+GAP1-C	GAP1-N2+GAP1-M+GAP1-C*→	838	Streptomyces fimicarius	GCF_002154345.1	actinobacteria
WP_086817677.1	GAP1-N2+GAP1-M+GAP1-C	GAP1-N2+GAP1-M+GAP1-C*→ PSE→?→||←TPR_containing_alpha_helical+Fibronectin-like_repeats	792	Streptomyces cacaoi	GCA_006539165.1	actinobacteria
WP_086843588.1	GAP1-N2+GAP1-M+GAP1-C+TM+beta-rich	vWA(canonical)+beta-rich+low_complexity→?→?→ Tubulin+alpha_helical+??+??→??+TM+TM+TM+TM+??→ Pkinase+TM→ ZnR+ZnR+DO-GTPase2.2→ GAP1-N2+GAP1-M+GAP1-C+TM+beta-rich*→	578	Amycolatopsis kentuckyensis	GCF_002155975.1	actinobacteria
WP_086860305.1	GAP1-M+GAP1-C	GAP1-M+GAP1-C*→	690	Streptomyces milbemycinicus	GCF_002154605.1	actinobacteria
WP_086892272.1	GAP1-N2+GAP1-M+GAP1-C	polar_leader+TM+TM+TM→ TM+vWA(canonical)+Fibronectin-like_repeats+TM+beta-rich+lc_tail→ Tubulin+alpha_helical+??+??→ GAP_unknown1+TM+TM+GAP_unknown2→ HRDC-like?+Fibronectin-like_repeats→ GAP1-N2+GAP1-M+GAP1-C*→ beta-rich+vWA(canonical)→??+DO-GTPase2.2→ polar_leader+TM+TM+TM→	527	Corynebacterium striatum	GCA_002156805.1	actinobacteria
WP_087063454.1	GAP1-N2+GAP1-M+GAP1-C	polar_leader+TM+TM+TM→ TM+vWA(canonical)+Fibronectin-like_repeats+TM+beta-rich+lc_tail→ Tubulin+alpha_helical+??+??→ GAP_unknown1+TM+TM+GAP_unknown2→ HRDC-like?+Fibronectin-like_repeats→ GAP1-N2+GAP1-M+GAP1-C*→ beta-rich+vWA(canonical)→??+DO-GTPase2.2→	631	Corynebacterium glutamicum	GCF_900163685.1	actinobacteria
WP_087081230.1	GAP1-N2+GAP1-M+GAP1-C	TM+DUF4190+TM→ polar_leader+TM+TM+TM→ PSE→ Tubulin+alpha_helical+??+??→ GAP_unknown1+TM+TM+GAP_unknown2→ HRDC-like?+Fibronectin-like_repeats→ GAP1-N2+GAP1-M+GAP1-C*→ beta-rich+vWA(canonical)→??+DO-GTPase2.2→ TM+DUF4190+TM+PknH_C→ TM+DUF4190+TM+PknH_C→ TM+DUF4190+TM+PknH_C→	828	Mycobacterium dioxanotrophicus	GCA_002157835.1	actinobacteria
WP_087101546.1	GAP1-N2+GAP1-M+GAP1-C	TPR_containing_alpha_helical+Fibronectin-like_repeats→ ZnR+ZnR+DO-GTPase2.2→ GAP1-N2+GAP1-M+GAP1-C*→ TM+TM+TM+TM+ZnR+ZnR+DO-GTPase2.2→	659	Nocardiopsis sp. JB363	GCF_900143625.1	actinobacteria
WP_087117134.1	GAP1-N2+GAP1-M+GAP1-C	Tubulin+alpha_helical+??+??→ GAP_unknown1+TM+TM+GAP_unknown2→ HRDC-like?+Fibronectin-like_repeats→ GAP1-N2+GAP1-M+GAP1-C*→ beta-rich+vWA(canonical)→??+DO-GTPase2.2→	604	Corynebacterium urinapleomorphum	GCF_900155535.1	actinobacteria
WP_087166954.1	GAP1-N2+GAP1-M+GAP1-C	TM+vWA(canonical)+YtKA-like+IG-like+TM+??→ Tubulin+coiled_coil+??→?→?→ ZnR+insert+ZnR+DO-GTPase2.2+ZnR→ GAP1-N2+GAP1-M+GAP1-C*→ Pkinase→	816	Drancourtella sp. An12	GCA_002160935.1	firmicutes
WP_087218558.1	GAP1-N2+GAP1-M+TM+GAP1-C+TM+TM+TM	Tubulin+coiled_coil+??→ Tubulin+coiled_coil+??→?→??→ ZnR+ZnR+DO-GTPase2.2→ GAP1-N2+GAP1-M+TM+GAP1-C+TM+TM+TM*→ TM+TM+TM+vWA(canonical)+TM+TM+TM+polar_tail→	808	Lachnoclostridium sp. An14	GCA_002160755.1	firmicutes
WP_087225396.1	GAP1-N2+GAP1-M+GAP1-C	Tubulin+coiled_coil+??→ Tubulin+coiled_coil+??→?→?→ ZnR+insert+ZnR+DO-GTPase2.2+??→ GAP1-N2+GAP1-M+GAP1-C*→?→?→ Pkinase→	953	Blautia sp. An249	GCA_002159835.1	firmicutes
WP_087230700.1	GAP1-N2+GAP1-M+GAP1-C	GAP1-N2+GAP1-M+GAP1-C*→	923	Pseudoflavonifractor sp. An176	GCA_002160435.1	firmicutes
WP_087348406.1	GAP1-N2+GAP1-M+GAP1-C+polar_tail+TM+beta-rich	TM+vWA(canonical)+YtKA-like+TM+??→ Tubulin+coiled_coil+??→ Tubulin+coiled_coil+HTH?+??→ TM+TM+TM+vWA(canonical)+TM+TM+TM+polar_tail→ Fibronectin-like_repeats+??→ ZnR+ZnR+DO-GTPase2.2+ZnR+ZnR→ GAP1-N2+GAP1-M+GAP1-C+polar_tail+TM+beta-rich*→	832	Brevibacillus brevis	GCA_002161835.1	firmicutes
WP_087435055.1	GAP1-N2+GAP1-M+GAP1-C+polar_tail+TM+beta-rich	TM+vWA(canonical)+YtKA-like+TM+??→ Tubulin+coiled_coil+??→ Tubulin+coiled_coil+HTH?+??→ TM+TM+TM+vWA(canonical)+TM+TM+TM+polar_tail→ Fibronectin-like_repeats+??→ ZnR+ZnR+DO-GTPase2.2+ZnR+ZnR→ GAP1-N2+GAP1-M+GAP1-C+polar_tail+TM+beta-rich*→	971	Paenibacillus apiarius	GCF_002161865.1	firmicutes
WP_087442672.1	GAP1-N2+GAP1-M+GAP1-C+polar_tail+TM+beta-rich	TM+vWA(canonical)+YtKA-like+TM+??→ Tubulin+coiled_coil+??→ Tubulin+coiled_coil+HTH?+??→ TM+TM+TM+vWA(canonical)+TM+TM+TM+polar_tail→ Fibronectin-like_repeats+??→ ZnR+ZnR+DO-GTPase2.2+ZnR+ZnR→ GAP1-N2+GAP1-M+GAP1-C+polar_tail+TM+beta-rich*→	1019	Paenibacillus thiaminolyticus	GCF_004001005.1	firmicutes
WP_087557944.1	GAP1-N2+GAP1-M+GAP1-C	GAP1-N2+GAP1-M+GAP1-C*→ beta-rich+vWA(canonical)→??+DO-GTPase2.2→	914	Rhodococcus sp. NCIMB 12038	GCA_002165735.1	actinobacteria
WP_087764506.1	GAP1-N2+GAP1-M+GAP1-C	GAP1-N2+GAP1-M+GAP1-C*→	833	Streptomyces sp. CS057	GCA_002188345.1	actinobacteria
WP_087772515.1	GAP1-N2+GAP1-M+GAP1-C	GAP1-N2+GAP1-M+GAP1-C*→	789	Streptomyces sp. CS227	GCA_002188355.1	actinobacteria
WP_087799167.1	GAP1-N2+GAP1-M+GAP1-C+polar_tail+TM+beta-rich	TM+vWA(canonical)+YtKA-like+TM+??→ Tubulin+coiled_coil+??→?→ TM+TM+TM+vWA(canonical)+TM+TM+TM+polar_tail→?→ Fibronectin-like_repeats+??→ ZnR+ZnR+DO-GTPase2.2+ZnR+ZnR→ GAP1-N2+GAP1-M+GAP1-C+polar_tail+TM+beta-rich*→	1140	Saccharibacillus sp. O16	GCA_002189105.1	firmicutes
WP_087886241.1	GAP1-N2+GAP1-M+GAP1-C	GAP1-N2+GAP1-M+GAP1-C*→ vWA(canonical)→ PP2C→ Pkinase+ZnR+beta-rich→||←TPR_containing_alpha_helical+Fibronectin-like_repeats	950	Streptomyces alboflavus	GCA_002189675.1	actinobacteria
WP_087919925.1	GAP1-N2+GAP1-M+GAP1-C+polar_tail+TM+beta-rich	TM+vWA(canonical)+YtKA-like+TM+??→ Tubulin+coiled_coil+??→ Tubulin+coiled_coil+HTH?+??→ TM+TM+TM+vWA(canonical)+TM+TM+TM+polar_tail→ Fibronectin-like_repeats+??→ ZnR+ZnR+DO-GTPase2.2+ZnR+ZnR→ GAP1-N2+GAP1-M+GAP1-C+polar_tail+TM+beta-rich*→	1019	Paenibacillus donghaensis	GCA_002192415.1	firmicutes
WP_088089667.1	GAP1-N2+GAP1-M+GAP1-C+polar_tail+TM+beta-rich	TM+vWA(canonical)+YtKA-like+TM+??→ Tubulin+coiled_coil+??→ Tubulin+coiled_coil+HTH?+??→ TM+TM+TM+vWA(canonical)+TM+TM+TM+polar_tail→ Fibronectin-like_repeats+??→ ZnR+ZnR+DO-GTPase2.2+ZnR+ZnR→ GAP1-N2+GAP1-M+GAP1-C+polar_tail+TM+beta-rich*→	939	Bacillus sp. OV166	GCA_900177675.1	firmicutes
WP_088252361.1	GAP1-N2+GAP1-M+TM+low_complexity+TM	GAP1-N2+GAP1-M+TM+low_complexity+TM*→	828	Fimbriiglobus ruber	GCA_002197845.1	planctomycetes
WP_088259456.1	GAP1-N2+GAP1-M+TM+GAP1-C	GAP1-N2+GAP1-M+TM+GAP1-C*→	738	Fimbriiglobus ruber	GCA_002197845.1	planctomycetes
WP_088304970.1	GAP1-N2+GAP1-M+GAP1-C	polar_leader+TM+TM+TM→ TM+vWA(canonical)+Fibronectin-like_repeats+TM+beta-rich+lc_tail→ Tubulin+alpha_helical+??+??→ GAP_unknown1+TM+TM+GAP_unknown2→ HRDC-like?+Fibronectin-like_repeats→ GAP1-N2+GAP1-M+GAP1-C*→ beta-rich+vWA(canonical)→??+DO-GTPase2.2→ TM+DUF4190+TM+PknH_C→ TM+DUF4190+TM+PknH_C→	828	Mycolicibacterium peregrinum	GCA_002198105.1	actinobacteria
WP_088488102.1	GAP1-N2+GAP1-M+GAP1-C+polar_tail+TM+beta-rich	TM+vWA(canonical)+YtKA-like+TM+??→ Tubulin+coiled_coil+??→ Tubulin+coiled_coil+HTH?+??→ TM+TM+TM+vWA(canonical)+TM+TM+TM+polar_tail→?→ Fibronectin-like_repeats+??→ ZnR+ZnR+DO-GTPase2.2+ZnR+ZnR→ GAP1-N2+GAP1-M+GAP1-C+polar_tail+TM+beta-rich*→	1136	Saccharibacillus sp. O23	GCA_002205895.1	firmicutes
WP_088548123.1	GAP1-N2+GAP1-M+GAP1-C+polar_tail+TM+beta-rich	TM+vWA(canonical)+YtKA-like+TM+??→ Tubulin+coiled_coil+??→ Tubulin+coiled_coil+HTH?+??→ TM+TM+TM+vWA(canonical)+TM+TM+TM+polar_tail→ Fibronectin-like_repeats+??→ ZnR+ZnR+DO-GTPase2.2+ZnR+ZnR→ GAP1-N2+GAP1-M+GAP1-C+polar_tail+TM+beta-rich*→	974	Paenibacillus sp. St-s	GCF_900188525.1	firmicutes
WP_088574934.1	GAP1-N2+GAP1-M+GAP1-C	GAP1-N2+GAP1-M+GAP1-C*→	835	Streptomyces sp. PgraA7	GCA_900187925.1	actinobacteria
WP_088831747.1	GAP1-N2+GAP1-M+GAP1-C+polar_tail+TM+Cadherin-like	TM+vWA(canonical)+YtKA-like+TM+??→ Tubulin+coiled_coil+??→ Tubulin+coiled_coil+HTH?+??→?→ TM+TM+TM+vWA(canonical)+TM+TM+TM+polar_tail→ Fibronectin-like_repeats+??→ ZnR+ZnR+DO-GTPase2.2+ZnR+ZnR→ GAP1-N2+GAP1-M+GAP1-C+polar_tail+TM+Cadherin-like*→	936	Paenibacillus elgii	GCF_900188505.1	firmicutes
WP_088896775.1	GAP1-N2+GAP1-M+GAP1-C	polar_leader+TM+TM+TM→ TM+vWA(canonical)+Fibronectin-like_repeats+TM+beta-rich+lc_tail→ Tubulin+alpha_helical+??+??→ GAP_unknown1+TM+TM+GAP_unknown2→ HRDC-like?+Fibronectin-like_repeats→ GAP1-N2+GAP1-M+GAP1-C*→ beta-rich+vWA(canonical)→??+DO-GTPase2.2→	909	Rhodococcus sp. BUPNP1	GCA_002215235.1	actinobacteria
WP_088909953.1	GAP1-N2+GAP1-M+GAP1-C+polar_tail+TM+beta-rich	TM+vWA(canonical)+YtKA-like+TM+??→ Tubulin+coiled_coil+??→ Tubulin+coiled_coil+HTH?+??→ TM+TM+TM+vWA(canonical)+TM+TM+TM+polar_tail→ Fibronectin-like_repeats+??→ ZnR+ZnR+DO-GTPase2.2+ZnR+ZnR→ GAP1-N2+GAP1-M+GAP1-C+polar_tail+TM+beta-rich*→	832	Brevibacillus formosus	GCA_002215075.1	firmicutes
WP_088943649.1	GAP1-N2+GAP1-M+GAP1-C	GrpE→ HSP70→ TPR_containing_alpha_helical+Fibronectin-like_repeats→ ZnR+ZnR+DO-GTPase2.2→ GAP1-N2+GAP1-M+GAP1-C*→ TM+TM+TM+TM+ZnR+ZnR+DO-GTPase2.2→??→ HSP70→	802	Rhodococcus sp. 1168	GCA_002091955.1	actinobacteria
WP_089000279.1	GAP1-N2+GAP1-M+GAP1-C	TPR_containing_alpha_helical+Fibronectin-like_repeats→ ZnR+ZnR+DO-GTPase2.2→ GAP1-N2+GAP1-M+GAP1-C*→ TM+TM+TM+TM+ZnR+ZnR+DO-GTPase2.2→	797	Micromonospora echinofusca	GCA_900091445.1	actinobacteria

acc	architecture	operon	len	species	gca	taxend
WP_089116955.1	GAP1-N2+GAP1-M+GAP1-C	GAP1-N2+GAP1-M+GAP1-C*→	826	Streptomyces sp. SS07	GCF_002217715.1	actinobacteria
WP_089295102.1	GAP1-N2+GAP1-M+GAP1-C	HSP70→ TPR_containing_alpha_helical+Fibronectin-like_repeats→ ZnR+ZnR+DO-GTPase2.2→ GAP1-N2+GAP1-M+GAP1-C*→ TM+TM+TM+TM+ZnR+ZnR+DO-GTPase2.2→ TPR_region+CASPASE→?→||←CASPASE+low_complexity	787	Actinoplanes regularis	GCA_900188005.1	actinobacteria
WP_089306581.1	GAP1-N2+GAP1-M+GAP1-C	Pkinase+TM→ ZnR+ZnR+DO-GTPase2.2→ GAP1-N2+GAP1-M+GAP1-C*→ Pkinase+low_complexity→	556	Geodermatophilus pulveris	GCF_900188375.1	actinobacteria
WP_089315832.1	GAP1-N2+GAP1-M+GAP1-C	GrpE→ HSP70→ TPR_containing_alpha_helical+Fibronectin-like_repeats→ ZnR+ZnR+DO-GTPase2.2→ GAP1-N2+GAP1-M+GAP1-C*→ TM+TM+TM+TM+ZnR+ZnR+DO-GTPase2.2+TM→	740	Actinomadura mexicana	GCA_900188105.1	actinobacteria
WP_089326036.1	GAP1-N2+GAP1-M+GAP1-C	GrpE→ HSP70→ TPR_containing_alpha_helical+Fibronectin-like_repeats→ ZnR+TM+ZnR+DO-GTPase2.2→ GAP1-N2+GAP1-M+GAP1-C*→ TM+TM+TM+TM+ZnR+ZnR+DO-GTPase2.2+TM→	670	Actinomadura meyerae	GCA_900188445.1	actinobacteria
WP_089507784.1	GAP1-N2+GAP1-M+GAP1-C	PP2C→ Pkinase→?→?→?→ HSP70_domain_2+low_complexity+domain_betaX→ ZnR+ZnR+DO-GTPase2.2→ GAP1-N2+GAP1-M+GAP1-C*→ TM+TM+TM+TM+ZnR+ZnR+DO-GTPase2.2→	817	Streptomyces sp. NBS 14/10	GCA_002224125.1	actinobacteria
WP_089509957.1	GAP1-N2+GAP1-M+GAP1-C	GAP1-N2+GAP1-M+GAP1-C*→	808	Streptomyces sp. NBS 14/10	GCA_002224125.1	actinobacteria
WP_089549257.1	GAP1-N2+GAP1-M+GAP1-C+polar_tail+TM+beta-rich	TM+vWA(canonical)+YtKA-like+TM+??→ Tubulin+coiled_coil+??→ Tubulin+coiled_coil+HTH?+??→ TM+TM+TM+vWA(canonical)+TM+TM+TM+polar_tail→ Fibronectin-like_repeats+??→ ZnR+ZnR+DO-GTPase2.2+ZnR+ZnR→ GAP1-N2+GAP1-M+GAP1-C+polar_tail+TM+beta-rich*→	1087	Paenibacillus sp. SSG-1	GCA_002224835.1	firmicutes
WP_090263386.1	GAP1-N2+GAP1-M+GAP1-C	TM+TM+TM+TM+ZnR+ZnR+DO-GTPase2.2→?→ HSP70→ HSP70+vWA(canonical)→ DnaJ+DnaJ+ZnR+ZnR+coiled_coil+Fibronectin-like_repeats→ ZnR+ZnR+DO-GTPase2.2→ GAP1-N2+GAP1-M+GAP1-C*→	916	Eubacteriaceae bacterium CHKCI005	GCA_900067065.1	firmicutes
WP_090429383.1	GAP1-N2+GAP1-M+GAP1-C	HRDC-like?+Fibronectin-like_repeats→ GAP1-N2+GAP1-M+GAP1-C*→??+DO-GTPase2.2→	819	Mycobacterium	GCA_900110825.1	actinobacteria
WP_090516748.1	GAP1-N2+GAP1-M+GAP1-C	HRDC-like?+Fibronectin-like_repeats→ GAP1-N2+GAP1-M+GAP1-C*→??+DO-GTPase2.2→	819	Mycolicibacterium neworleansense	GCA_001245615.1	actinobacteria
WP_090669001.1	GAP1-N2+GAP1-M+GAP1-C+polar_tail+TM+Cadherin-like	TM+vWA(canonical)+YtKA-like+TM+??→ Tubulin+coiled_coil+??→ Tubulin+coiled_coil+HTH?+??→?→ TM+TM+TM+vWA(canonical)+TM+TM+TM+polar_tail→ Fibronectin-like_repeats+??→ ZnR+ZnR+DO-GTPase2.2+ZnR+ZnR→ GAP1-N2+GAP1-M+GAP1-C+polar_tail+TM+Cadherin-like*→	933	Paenibacillus tianmuensis	GCA_900100345.1	firmicutes
WP_090719954.1	GAP1-N2+GAP1-M+GAP1-C+polar_tail+TM+beta-rich	ZnR+ZnR+DO-GTPase2.2+ZnR+ZnR→ GAP1-N2+GAP1-M+GAP1-C+polar_tail+TM+beta-rich*→	1035	Paenibacillus typhae	GCA_900099765.1	firmicutes
WP_090761118.1	GAP1-N2+GAP1-M+GAP1-C+polar_tail+TM+beta-rich	TM+vWA(canonical)+YtKA-like+TM+??→ Tubulin+coiled_coil+??→ Tubulin+coiled_coil+HTH?+??→ TM+TM+TM+vWA(canonical)+TM+TM+TM+polar_tail→ Fibronectin-like_repeats+??→ ZnR+ZnR+DO-GTPase2.2+ZnR+ZnR→ GAP1-N2+GAP1-M+GAP1-C+polar_tail+TM+beta-rich*→	935	Bacillus sp. OK048	GCA_900103525.1	firmicutes
WP_090808992.1	GAP1-N2+GAP1-M+GAP1-C+polar_tail	TM+TM+TM+vWA+TM+TM+TM+polar_tail→ Fibronectin-like_repeats+??→ ZnR+ZnR+DO-GTPase2.2→ GAP1-N2+GAP1-M+GAP1-C+polar_tail*→	1058	Paenibacillus sp. 276b	GCA_900107875.1	firmicutes
WP_090834503.1	GAP1-N2+GAP1-M+GAP1-C+polar_tail+TM+beta-rich	TM+vWA(canonical)+YtKA-like+TM+??→ Tubulin+coiled_coil+??→ Tubulin+coiled_coil+HTH?+??→ TM+TM+TM+vWA(canonical)+TM+TM+TM+polar_tail→ Fibronectin-like_repeats+??→ ZnR+ZnR+DO-GTPase2.2+ZnR+ZnR→ GAP1-N2+GAP1-M+GAP1-C+polar_tail+TM+beta-rich*→	1028	Paenibacillus sophorae	GCA_900110315.1	firmicutes
WP_090886938.1	GAP1-N2+GAP1-M+GAP1-C+polar_tail	Tubulin+coiled_coil+??→ Tubulin+coiled_coil+HTH?+??→ TM+TM+TM+vWA+TM+TM+TM+polar_tail→ Fibronectin-like_repeats+??→ ZnR+ZnR+DO-GTPase2.2→ GAP1-N2+GAP1-M+GAP1-C+polar_tail*→	1009	Paenibacillus sp. 181mfcol5.1	GCA_900109125.1	firmicutes
WP_090903430.1	GAP1-N2+GAP1-M+GAP1-C+polar_tail	TM+TM+TM+vWA+TM+TM+TM+polar_tail→ Fibronectin-like_repeats+??→ ZnR+ZnR+DO-GTPase2.2→ GAP1-N2+GAP1-M+GAP1-C+polar_tail*→	1057	Paenibacillus sp. OK076	GCF_900110055.1	firmicutes
WP_090913398.1	GAP1-N2+GAP1-M+GAP1-C+polar_tail+TM+beta-rich	TM+vWA(canonical)+YtKA-like+TM+??→ Tubulin+coiled_coil+??→ Tubulin+coiled_coil+HTH?+??→ TM+TM+TM+vWA(canonical)+TM+TM+TM+polar_tail→ Fibronectin-like_repeats+??→ ZnR+ZnR+DO-GTPase2.2+ZnR+ZnR→ GAP1-N2+GAP1-M+GAP1-C+polar_tail+TM+beta-rich*→	991	Paenibacillus sp. cl141a	GCA_900109305.1	firmicutes
WP_090924169.1	GAP1-N2+GAP1-M+GAP1-C+polar_tail+TM+beta-rich	TM+vWA(canonical)+YtKA-like+TM+??→ Tubulin+coiled_coil+??→ Tubulin+coiled_coil+HTH?+??→ TM+TM+TM+vWA(canonical)+TM+TM+TM+polar_tail→ Fibronectin-like_repeats+??→ ZnR+ZnR+DO-GTPase2.2+ZnR+ZnR→ GAP1-N2+GAP1-M+GAP1-C+polar_tail+TM+beta-rich*→	1071	Paenibacillus polysaccharolyticus	GCA_900102085.1	firmicutes
WP_090952035.1	GAP1-N2+GAP1-M+GAP1-C+polar_tail+TM+beta-rich	TM+vWA(canonical)+YtKA-like+TM+??→ Tubulin+coiled_coil+??→ Tubulin+coiled_coil+HTH?+??→ TM+TM+TM+vWA(canonical)+TM+TM+TM+polar_tail→ Fibronectin-like_repeats+??→ ZnR+ZnR+DO-GTPase2.2+ZnR+ZnR→ GAP1-N2+GAP1-M+GAP1-C+polar_tail+TM+beta-rich*→	1057	Paenibacillus sp. OK060	GCA_900101205.1	firmicutes
WP_090995055.1	GAP1-N2+GAP1-M+GAP1-C+polar_tail	TM+TM+TM+vWA+TM+TM+TM+polar_tail→ Fibronectin-like_repeats+??→ ZnR+ZnR+DO-GTPase2.2→ GAP1-N2+GAP1-M+GAP1-C+polar_tail*→	1060	Paenibacillus sp. OK003	GCF_900109515.1	firmicutes
WP_091012817.1	GAP1-N2+GAP1-M+GAP1-C+polar_tail	Tubulin+coiled_coil+??→ Tubulin+coiled_coil+HTH?+??→ TM+TM+TM+vWA+TM+TM+TM+polar_tail→ Fibronectin-like_repeats+??→ ZnR+ZnR+DO-GTPase2.2→ GAP1-N2+GAP1-M+GAP1-C+polar_tail*→	1072	Paenibacillus amylolyticus	GCF_900095775.1	firmicutes
WP_091036116.1	GAP1-N2+GAP1-M+GAP1-C+polar_tail	Tubulin+coiled_coil+??→ Tubulin+coiled_coil+HTH?+??→ TM+TM+TM+vWA+TM+TM+TM+polar_tail→ Fibronectin-like_repeats+??→ ZnR+ZnR+DO-GTPase2.2→ GAP1-N2+GAP1-M+GAP1-C+polar_tail*→	1063	Paenibacillus sp. CF095	GCF_900101225.1	firmicutes
WP_091069568.1	GAP1-N2+GAP1-M+GAP1-C+polar_tail+TM+beta-rich	TM+vWA(canonical)+YtKA-like+TM+??→ Tubulin+coiled_coil+??→ Tubulin+coiled_coil+HTH?+??→ TM+TM+TM+vWA(canonical)+TM+TM+TM+polar_tail→ Fibronectin-like_repeats+??→ ZnR+ZnR+DO-GTPase2.2+ZnR+ZnR→ GAP1-N2+GAP1-M+GAP1-C+polar_tail+TM+beta-rich*→	1006	Paenibacillus sp. NFR01	-	firmicutes
WP_091151701.1	GAP1-N2+GAP1-M+GAP1-C	Pkinase+TM→ ZnR+ZnR+DO-GTPase2.2→ GAP1-N2+GAP1-M+GAP1-C*→||←?←?||?→ Pkinase+low_complexity→	559	Geodermatophilus africanus	-	actinobacteria
WP_091171406.1	GAP1-N2+GAP1-M+GAP1-C+polar_tail+TM+Cadherin-like	TM+vWA(canonical)+YtKA-like+TM+??→ Tubulin+coiled_coil+??→ Tubulin+coiled_coil+HTH?+??→ TM+TM+TM+vWA(canonical)+TM+TM+TM+polar_tail→ Fibronectin-like_repeats+??→ ZnR+ZnR+DO-GTPase2.2+ZnR+ZnR→ GAP1-N2+GAP1-M+GAP1-C+polar_tail+TM+Cadherin-like*→	965	Paenibacillus sp. 1_12	GCA_900114475.1	firmicutes
WP_091216627.1	GAP1-N2+GAP1-M+GAP1-C	Pkinase+TM→ ZnR+ZnR+DO-GTPase2.2→ GAP1-N2+GAP1-M+GAP1-C*→||←?←?||?→ Pkinase+low_complexity→	557	Geodermatophilus siccatus	-	actinobacteria
WP_091235374.1	GAP1-N2+GAP1-M+GAP1-C+polar_tail+TM+beta-rich	TM+vWA(canonical)+YtKA-like+TM+??→ Tubulin+coiled_coil+??→ Tubulin+coiled_coil+HTH?+??→ TM+TM+TM+vWA(canonical)+TM+TM+TM+polar_tail→ Fibronectin-like_repeats+??→ ZnR+ZnR+DO-GTPase2.2+ZnR+ZnR→ GAP1-N2+GAP1-M+GAP1-C+polar_tail+TM+beta-rich*→	1024	Fontibacillus panacisegetis	GCA_900102215.1	firmicutes
WP_091280726.1	GAP1-N2+GAP1-M+GAP1-C+ZnR+DO-GTPase2	GAP1-N2+GAP1-M+GAP1-C+ZnR+DO-GTPase2*→	1000	Frankia sp. G2	GCA_001536285.1	actinobacteria
WP_091284384.1	GAP1-N2+GAP1-M+GAP1-C	GrpE→ HSP70→ TPR_containing_alpha_helical+Fibronectin-like_repeats→ ZnR+ZnR+DO-GTPase2.2→ GAP1-N2+GAP1-M+GAP1-C*→ TM+TM+TM+TM+ZnR+ZnR+DO-GTPase2.2→	814	Micromonospora haikouensis	GCA_900091595.1	actinobacteria
WP_091326582.1	GAP1-N2+GAP1-M+GAP1-C	Pkinase+TM→ ZnR+ZnR+DO-GTPase2.2→ GAP1-N2+GAP1-M+GAP1-C*→	567	Geodermatophilus ruber	GCF_900114385.1	actinobacteria
WP_091381672.1	GAP1-N2+GAP1-M+GAP1-C	Pkinase+TM→ ZnR+ZnR+DO-GTPase2.2→ GAP1-N2+GAP1-M+GAP1-C*→||←?←?||?→ Pkinase+low_complexity→	556	Geodermatophilus sp. DSM 45219	GCF_900104025.1	actinobacteria
WP_091442415.1	GAP1-N2+GAP1-M+GAP1-C	Pkinase+TM→ ZnR+ZnR+DO-GTPase2.2→ GAP1-N2+GAP1-M+GAP1-C*→||←?←?||?→ Pkinase+low_complexity→	555	Geodermatophilus poikilotrophus	-	actinobacteria
WP_091450228.1	GAP1-N2+GAP1-M+GAP1-C	GrpE→ HSP70→ TPR_containing_alpha_helical+Fibronectin-like_repeats→ ZnR+ZnR+DO-GTPase2.2→ GAP1-N2+GAP1-M+GAP1-C*→ TM+TM+TM+TM+ZnR+ZnR+DO-GTPase2.2→?→||←?←?←HSP70	801	Alloactinosynnema iranicum	GCA_900101685.1	actinobacteria
WP_091525929.1	GAP1-N2+GAP1-M+GAP1-C	polar_leader+TM+TM+TM→ TM+vWA(canonical)+Fibronectin-like_repeats+TM+beta-rich+lc_tail→ Tubulin+alpha_helical+??+??→ GAP_unknown1+TM+TM+GAP_unknown2→ HRDC-like?+Fibronectin-like_repeats→ GAP1-N2+GAP1-M+GAP1-C*→ beta-rich+vWA(canonical)→??+DO-GTPase2.2→ HSP70_domain_1→	980	Microlunatus soli	GCA_900105385.1	actinobacteria
WP_092053099.1	GAP1-N2+GAP1-M+TM+GAP1-C	ZnR+ZnR+DO-GTPase2.2→ GAP1-N2+GAP1-M+TM+GAP1-C*→??+TM+??+beta-rich??→?→ GYF+GYF+ZnR+TM+TM+TM→	842	Planctomicrobium piriforme	GCA_900113665.1	planctomycetes
WP_092271867.1	GAP1-N2+GAP1-M+GAP1-C+polar_tail+TM+beta-rich	TM+vWA(canonical)+YtKA-like+TM+??→ Tubulin+coiled_coil+??→ Tubulin+coiled_coil+HTH?+??→ TM+TM+TM+vWA(canonical)+TM+TM+TM+polar_tail→ Fibronectin-like_repeats+??→ ZnR+ZnR+DO-GTPase2.2+ZnR+ZnR→ GAP1-N2+GAP1-M+GAP1-C+polar_tail+TM+beta-rich*→	834	Brevibacillus sp. OK042	GCA_900114075.1	firmicutes
WP_092611337.1	GAP1-N2+GAP1-M+low_complexity	ZnR+ZnR+DO-GTPase2.2→ GAP1-N2+GAP1-M+low_complexity*→	541	Raineyella antarctica	GCA_900092135.1	actinobacteria
WP_092783364.1	GAP1-N2+GAP1-M	CASPASE+TPR_region→?→ GAP1-N2+GAP1-M*→	183	Actinokineospora terrae	GCF_900111175.1	actinobacteria
WP_092943636.1	GAP1-N2+GAP1-M+GAP1-C	HSP70→ HSP70+??→ DnaJ+DnaJ+ZnR+ZnR+coiled_coil+Fibronectin-like_repeats→ ZnR+ZnR+DO-GTPase2.2→ GAP1-N2+GAP1-M+GAP1-C*→ TM+TM+TM+TM+ZnR+ZnR+DO-GTPase2.2→	843	Ruminococcaceae bacterium YRB3002	GCA_900099715.1	firmicutes
WP_093149955.1	GAP1-N2+GAP1-M+GAP1-C	GrpE→ HSP70→ TPR_containing_alpha_helical+Fibronectin-like_repeats→ ZnR+ZnR+DO-GTPase2.2→ GAP1-N2+GAP1-M+GAP1-C*→ PSE→?→?→||←?←?←HSP70←unknown+CASPASE	798	Saccharopolyspora antimicrobica	GCA_003635025.1	actinobacteria
WP_093275372.1	GAP1-N2+GAP1-M+GAP1-C	GrpE→ HSP70→ TPR_containing_alpha_helical+Fibronectin-like_repeats→ ZnR+ZnR+DO-GTPase2.2→ GAP1-N2+GAP1-M+GAP1-C*→ TM+TM+TM+TM+ZnR+ZnR+DO-GTPase2.2→ HSP70→ vWA(canonical)→	800	Saccharopolyspora shandongensis	GCA_900106995.1	actinobacteria
WP_093351331.1	GAP1-N2+GAP1-M+GAP1-C	GrpE→ HSP70→ TPR_containing_alpha_helical+Fibronectin-like_repeats→ ZnR+ZnR+DO-GTPase2.2→ GAP1-N2+GAP1-M+GAP1-C*→ PSE→?→?→||←?←?←HSP70←unknown+CASPASE	798	Saccharopolyspora	GCA_003053965.1	actinobacteria
WP_093442214.1	GAP1-N2+GAP1-M+GAP1-C	GAP1-N2+GAP1-M+GAP1-C*→	828	Streptomyces sp. Cmuel-A718b	GCA_900092005.1	actinobacteria
WP_093485969.1	GAP1-N2+GAP1-M+GAP1-C	TPR_containing_alpha_helical+Fibronectin-like_repeats→ ZnR+ZnR+DO-GTPase2.2→ GAP1-N2+GAP1-M+GAP1-C*→ PSE→ vWA(canonical)→ PP2C→ Pkinase+ZnR+beta-rich→	822	Streptomyces	GCA_002797835.1	actinobacteria
WP_093527826.1	GAP1-N2+GAP1-M+GAP1-C	GAP1-N2+GAP1-M+GAP1-C*→	803	Streptomyces	GCA_900091765.1	actinobacteria
WP_093542234.1	GAP1-N2+GAP1-M+GAP1-C	GAP1-N2+GAP1-M+GAP1-C*→	822	Streptomyces sp. DvalAA-43	GCA_900091725.1	actinobacteria
WP_093562468.1	GAP1-N2+GAP1-M+GAP1-C	HSP70_domain_2+low_complexity+domain_betaX→ ZnR+ZnR+DO-GTPase2.2→ GAP1-N2+GAP1-M+GAP1-C*→	843	Streptomyces sp. OspMP-M43	GCA_900091905.1	actinobacteria
WP_093599040.1	GAP1-N2+GAP1-M+GAP1-C	GAP1-N2+GAP1-M+GAP1-C*→	860	Streptomyces sp. MnatMP-M17	GCA_900091995.1	actinobacteria

<table>
<tr><th>acc</th><th>architecture</th><th>operon</th><th>len</th><th>species</th><th>gca</th><th>taxend</th></tr>
<tr><td>WP_093675522.1</td><td>GAP1-N2+GAP1-M+GAP1-C</td><td>GAP1-N2+GAP1-M+GAP1-C*→</td><td>803</td><td>Streptomyces sp. OspMP-M45</td><td>GCA_900090065.1</td><td>actinobacteria</td></tr>
<tr><td>WP_093684623.1</td><td>GAP1-N2+GAP1-M+GAP1-C</td><td>GAP1-N2+GAP1-M+GAP1-C*→</td><td>813</td><td>Streptomyces sp. MnatMP-M77</td><td>GCA_900090075.1</td><td>actinobacteria</td></tr>
<tr><td>WP_093686110.1</td><td>GAP1-N2+GAP1-M+GAP1-C</td><td>GAP1-N2+GAP1-M+GAP1-C*→</td><td>811</td><td>Streptomyces sp. ScaeMP-e83</td><td>GCA_900091775.1</td><td>actinobacteria</td></tr>
<tr><td>WP_093705090.1</td><td>GAP1-N2+GAP1-M+GAP1-C</td><td>GAP1-N2+GAP1-M+GAP1-C*→</td><td>816</td><td>Streptomyces sp. 2131.1</td><td>GCA_900105515.1</td><td>actinobacteria</td></tr>
<tr><td>WP_093755654.1</td><td>GAP1-N2+GAP1-M+GAP1-C</td><td>GAP1-N2+GAP1-M+GAP1-C→||←PSE||vWA(canonical)→ PP2C→ Pkinase+ZnR+beta-rich→ PSE→||←?←?←?||GAP1-N2+GAP1-M+GAP1-C*→</td><td>894</td><td>Streptomyces sp. DvalAA-19</td><td>GCA_900091865.1</td><td>actinobacteria</td></tr>
<tr><td>WP_093755661.1</td><td>GAP1-N2+GAP1-M+GAP1-C</td><td>TPR_containing_alpha_helical+Fibronectin-like_repeats→ ZnR+ZnR+DO-GTPase2.2→ GAP1-N2+GAP1-M+GAP1-C*→||←PSE||vWA(canonical)→ PP2C→ Pkinase+ZnR+beta-rich→ PSE→||←?←?←?||GAP1-N2+GAP1-M+GAP1-C→</td><td>873</td><td>Streptomyces sp. DvalAA-19</td><td>GCA_900091865.1</td><td>actinobacteria</td></tr>
<tr><td>WP_093776162.1</td><td>GAP1-N2+GAP1-M+GAP1-C</td><td>GAP1-N2+GAP1-M+GAP1-C*→</td><td>802</td><td>Streptomyces sp. 136MFCol5.1</td><td>GCA_900101975.1</td><td>actinobacteria</td></tr>
<tr><td>WP_093813336.1</td><td>GAP1-N2+GAP1-M+GAP1-C</td><td>GAP1-N2+GAP1-M+GAP1-C*→</td><td>835</td><td>Streptomyces sp. Ncost-T6T-1</td><td>GCA_900090085.1</td><td>actinobacteria</td></tr>
<tr><td>WP_093831399.1</td><td>GAP1-N2+GAP1-M+GAP1-C</td><td>GAP1-N2+GAP1-M+GAP1-C*→ vWA(canonical)→ PP2C→ Pkinase+ZnR+beta-rich→</td><td>775</td><td>Streptomyces sp. SolWspMP-5a-2</td><td>GCA_900091845.1</td><td>actinobacteria</td></tr>
<tr><td>WP_093845333.1</td><td>GAP1-N2+GAP1-M+GAP1-C</td><td>GAP1-N2+GAP1-M+GAP1-C*→</td><td>789</td><td>Streptomyces sp. ScaeMP-6W</td><td>GCA_900091885.1</td><td>actinobacteria</td></tr>
<tr><td>WP_093899303.1</td><td>GAP1-N2+GAP1-M+GAP1-C</td><td>GAP1-N2+GAP1-M+GAP1-C*→</td><td>814</td><td>Streptomyces sp. Ncost-T10-10d</td><td>GCA_900091955.1</td><td>actinobacteria</td></tr>
<tr><td>WP_093902175.1</td><td>GAP1-N2+GAP1-M+GAP1-C</td><td>GAP1-N2+GAP1-M+GAP1-C*→</td><td>845</td><td>Streptomyces sp. LaPpAH-199</td><td>GCA_900090135.1</td><td>actinobacteria</td></tr>
<tr><td>WP_093910651.1</td><td>GAP1-N2+GAP1-M+GAP1-C</td><td>TPR_containing_alpha_helical+Fibronectin-like_repeats→ ZnR+ZnR+DO-GTPase2.2→ GAP1-N2+GAP1-M+GAP1-C*→ TM+TM+TM+TM+ZnR+ZnR+DO-GTPase2.2+TM→ vWA(canonical)→ PP2C→ Pkinase+ZnR+beta-rich→</td><td>981</td><td>Streptomyces sp. cf386</td><td>GCA_900103585.1</td><td>actinobacteria</td></tr>
<tr><td>WP_093930224.1</td><td>GAP1-N2+GAP1-M+GAP1-C</td><td>GAP1-N2+GAP1-M+GAP1-C*→</td><td>802</td><td>Streptomyces sp. ok210</td><td>GCA_900116325.1</td><td>actinobacteria</td></tr>
<tr><td>WP_094017499.1</td><td>GAP1-N2+GAP1-M+GAP1-C+polar_tail+TM+Cadherin-like</td><td>TM+vWA(canonical)+YtKA-like+TM+??→ Tubulin+coiled_coil+??→ Tubulin+coiled_coil+HTH?+??→ TM+TM+TM+vWA(canonical)+TM+TM+TM+polar_tail→ Fibronectin-like_repeats+??→ ZnR+ZnR+DO-GTPase2.2+ZnR+ZnR→ GAP1-N2+GAP1-M+GAP1-C+polar_tail+TM+Cadherin-like*→</td><td>975</td><td>Paenibacillus rigui</td><td>GCA_002234615.1</td><td>firmicutes</td></tr>
<tr><td>WP_094079172.1</td><td>GAP1-N2+GAP1-M+GAP1-C</td><td>TM+TM+TM+TM+ZnR+ZnR+DO-GTPase2.2→?→ HSP70→ HSP70+vWA(canonical)→ DnaJ+DnaJ+ZnR+ZnR+coiled_coil+Fibronectin-like_repeats→ ZnR+ZnR+DO-GTPase2.2→ GAP1-N2+GAP1-M+GAP1-C*→</td><td>908</td><td>Leptotrichia sp. oral taxon 498</td><td>GCA_002240055.1</td><td>fusobacteria</td></tr>
<tr><td>WP_094155297.1</td><td>GAP1-N2+GAP1-M+GAP1-C+polar_tail+TM+beta-rich</td><td>TM+vWA(canonical)+YtKA-like+TM+??→ Tubulin+coiled_coil+??→ Tubulin+coiled_coil+HTH?+??→ TM+TM+TM+vWA(canonical)+TM+TM+TM+polar_tail→ Fibronectin-like_repeats+??→ ZnR+ZnR+DO-GTPase2.2+ZnR+ZnR→ GAP1-N2+GAP1-M+GAP1-C+polar_tail+TM+beta-rich*→</td><td>1000</td><td>Paenibacillus kribbensis</td><td>GCA_002240415.1</td><td>firmicutes</td></tr>
<tr><td>WP_094213059.1</td><td>GAP1-N2+GAP1-M+GAP1-C</td><td>GAP1-N2+GAP1-M+GAP1-C*→</td><td>812</td><td>Streptomyces sp. 2R</td><td>GCF_002242735.1</td><td>actinobacteria</td></tr>
<tr><td>WP_094274170.1</td><td>GAP1-N2+GAP1-M+GAP1-C</td><td>polar_leader+TM+TM+TM→ TM+vWA(canonical)+Fibronectin-like_repeats+TM+beta-rich+lc_tail→ Tubulin+alpha_helical+??+??→ GAP_unknown1+TM+TM+GAP_unknown2→ HRDC-like?+Fibronectin-like_repeats→ GAP1-N2+GAP1-M+GAP1-C*→ beta-rich+vWA(canonical)→??+DO-GTPase2.2→</td><td>981</td><td>Rhodococcus sp. OK302</td><td>GCA_002245895.1</td><td>actinobacteria</td></tr>
<tr><td>WP_094360273.1</td><td>GAP1-N2+GAP1-M+GAP1-C</td><td>GrpE→ HSP70→ TPR_containing_alpha_helical+Fibronectin-like_repeats→ ZnR+ZnR+DO-GTPase2.2→ GAP1-N2+GAP1-M+GAP1-C*→ TM+TM+TM+TM+ZnR+ZnR+DO-GTPase2.2→??→?→?→?→?→ HSP70→</td><td>800</td><td>Propionibacteriaceae bacterium NML 150272</td><td>GCA_002250525.1</td><td>actinobacteria</td></tr>
<tr><td>WP_094365183.1</td><td>GAP1-N2+GAP1-M+GAP1-C</td><td>TM+vWA(canonical)+Fibronectin-like_repeats+TM+beta-rich+lc_tail→ Tubulin+alpha_helical+??+??→ GAP_unknown1+TM+TM+GAP_unknown2→ HRDC-like?+Fibronectin-like_repeats→ GAP1-N2+GAP1-M+GAP1-C*→ beta-rich+vWA(canonical)→??+DO-GTPase2.2→</td><td>881</td><td>Propionibacteriaceae bacterium NML 130396</td><td>GCA_002250625.1</td><td>actinobacteria</td></tr>
<tr><td>WP_094372855.1</td><td>GAP1-N2+GAP1-M+GAP1-C</td><td>TPR_containing_alpha_helical+Fibronectin-like_repeats→ ZnR+ZnR+DO-GTPase2.2→ GAP1-N2+GAP1-M+GAP1-C*→ TM+TM+TM+TM+ZnR+ZnR+DO-GTPase2.2+TM→ vWA(canonical)→ PSE→||←PSE||Pkinase+ZnR+beta-rich→</td><td>941</td><td>Streptomyces sp. FBKL.4005</td><td>GCA_002251155.1</td><td>actinobacteria</td></tr>
<tr><td>WP_094415864.1</td><td>GAP1-N2+GAP1-M+TM+GAP1-C</td><td>ZnR+ZnR+DO-GTPase2.2→ GAP1-N2+GAP1-M+TM+GAP1-C*→??+TM+??+beta-rich??→ GYF+GYF+TM+TM+TM→</td><td>698</td><td>Rhodopirellula</td><td>GCA_002251265.1</td><td>planctomycetes</td></tr>
<tr><td>WP_094418663.1</td><td>GAP1-N2+GAP1-M</td><td>FGS-fold_domain→ ZnR+DO-GTPase2.1→ GAP1-N2+GAP1-M*→</td><td>257</td><td>Rhodopirellula</td><td>GCA_002251265.1</td><td>planctomycetes</td></tr>
<tr><td>WP_094449561.1</td><td>GAP1-N2+GAP1-M+GAP1-C</td><td>polar_leader+TM+TM+TM→ TM+vWA(canonical)+Fibronectin-like_repeats+TM+beta-rich+lc_tail→ Tubulin+alpha_helical+??+??→ GAP_unknown1+TM+TM+GAP_unknown2→ HRDC-like?+Fibronectin-like_repeats→ GAP1-N2+GAP1-M+GAP1-C*→ beta-rich+vWA(canonical)→??+DO-GTPase2.2→</td><td>904</td><td>Propionibacteriaceae bacterium NML 160184</td><td>GCA_002250475.1</td><td>actinobacteria</td></tr>
<tr><td>WP_094452326.1</td><td>GAP1-N2+GAP1-M+GAP1-C</td><td>polar_leader+TM+TM+TM→ TM+vWA(canonical)+Fibronectin-like_repeats+TM+beta-rich+lc_tail→ Tubulin+alpha_helical+??+??→ GAP_unknown1+TM+TM+GAP_unknown2→ HRDC-like?+Fibronectin-like_repeats→ GAP1-N2+GAP1-M+GAP1-C*→ beta-rich+vWA(canonical)→??+DO-GTPase2.2→</td><td>1040</td><td>Propionibacteriaceae bacterium NML 150081</td><td>GCA_002250565.1</td><td>actinobacteria</td></tr>
<tr><td>WP_094598055.1</td><td>GAP1-N2+GAP1-M+GAP1-C+polar_tail+TM+beta-rich</td><td>TM+vWA(canonical)+YtKA-like+TM+??→ Tubulin+coiled_coil+??→ Tubulin+coiled_coil+HTH?+??→ TM+TM+TM+vWA(canonical)+TM+TM+TM+polar_tail→ Fibronectin-like_repeats+??→?→ ZnR+ZnR+DO-GTPase2.2+ZnR+ZnR→ GAP1-N2+GAP1-M+GAP1-C+polar_tail+TM+beta-rich*→</td><td>1078</td><td>Paenibacillus sp. XY044</td><td>GCA_002257645.1</td><td>firmicutes</td></tr>
<tr><td>WP_094639927.1</td><td>GAP1-N2+GAP1-M+GAP1-C</td><td>polar_leader+TM+TM+TM→ TM+vWA(canonical)+Fibronectin-like_repeats+TM+beta-rich+lc_tail→ Tubulin+alpha_helical+??+??→ GAP_unknown1+TM+TM+GAP_unknown2→ HRDC-like?+Fibronectin-like_repeats→ GAP1-N2+GAP1-M+GAP1-C*→ beta-rich+vWA(canonical)→??+DO-GTPase2.2→</td><td>904</td><td>Rhodococcus</td><td>GCA_002259035.1</td><td>actinobacteria</td></tr>
<tr><td>WP_094661437.1</td><td>GAP1-N2+GAP1-M+GAP1-C</td><td>TM+TM+TM+TM+ZnR+ZnR+DO-GTPase2.2→ GrpE→ HSP70→ DnaJ+DnaJ+ZnR+ZnR+coiled_coil+Fibronectin-like_repeats→ ZnR+ZnR+DO-GTPase2.2→ GAP1-N2+GAP1-M+GAP1-C*→</td><td>933</td><td>Pseudoscardovia radai</td><td>GCA_002259605.1</td><td>actinobacteria</td></tr>
<tr><td>WP_094661875.1</td><td>GAP1-N2+GAP1-M+GAP1-C</td><td>TM+TM+TM+TM+ZnR+ZnR+DO-GTPase2.2→ GrpE→ HSP70→ HSP70+vWA(canonical)→ DnaJ+DnaJ+ZnR+ZnR+coiled_coil+Fibronectin-like_repeats→ ZnR+ZnR+DO-GTPase2.2→ GAP1-N2+GAP1-M+GAP1-C*→</td><td>946</td><td>Bifidobacterium tissieri</td><td>GCA_002259645.1</td><td>actinobacteria</td></tr>
<tr><td>WP_094700136.1</td><td>GAP1-N2+GAP1-M+GAP1-C+polar_tail+TM+beta-rich</td><td>TM+vWA(canonical)+YtKA-like+TM+??→ Tubulin+coiled_coil+??→ Tubulin+coiled_coil+HTH?+??→ TM+TM+TM+vWA(canonical)+TM+TM+TM+polar_tail→ Fibronectin-like_repeats+??→ ZnR+ZnR+DO-GTPase2.2+ZnR+ZnR→ GAP1-N2+GAP1-M+GAP1-C+polar_tail+TM+beta-rich*→</td><td>833</td><td>Brevibacillus laterosporus</td><td>GCF_002259955.1</td><td>firmicutes</td></tr>
<tr><td>WP_094801232.1</td><td>GAP1-N2+GAP1-M+GAP1-C</td><td>TPR_containing_alpha_helical+Fibronectin-like_repeats+extended_insert+Fibronectin-like_repeats→ ZnR+ZnR+DO-GTPase2.2→ GAP1-N2+GAP1-M+GAP1-C*→ TM+TM+TM+TM+ZnR+ZnR+DO-GTPase2.2→</td><td>903</td><td>Nocardiopsis dassonvillei</td><td>GCA_002260685.1</td><td>actinobacteria</td></tr>
<tr><td>WP_094871510.1</td><td>GAP1-N2+GAP1-M+GAP1-C+polar_tail+TM+beta-rich</td><td>TM+vWA(canonical)+YtKA-like+TM+??→ Tubulin+coiled_coil+??→ Tubulin+coiled_coil+HTH?+??→ TM+TM+TM+vWA(canonical)+TM+TM+TM+polar_tail→ Fibronectin-like_repeats+??→ ZnR+ZnR+DO-GTPase2.2+ZnR+ZnR→ GAP1-N2+GAP1-M+GAP1-C+polar_tail+TM+beta-rich*→</td><td>986</td><td>Paenibacillus sp. VTT E-133280</td><td>GCA_002264385.1</td><td>firmicutes</td></tr>
<tr><td>WP_094875450.1</td><td>GAP1-N2+GAP1-M+GAP1-C+polar_tail+TM+beta-rich</td><td>TM+vWA(canonical)+YtKA-like+TM+??→ Tubulin+coiled_coil+??→ Tubulin+coiled_coil+HTH?+??→ TM+TM+TM+vWA(canonical)+TM+TM+TM+polar_tail→ Fibronectin-like_repeats+??→ ZnR+ZnR+DO-GTPase2.2+ZnR+ZnR→ GAP1-N2+GAP1-M+GAP1-C+polar_tail+TM+beta-rich*→</td><td>986</td><td>Paenibacillus sp. VTT E-133291</td><td>GCA_002264395.1</td><td>firmicutes</td></tr>
<tr><td>WP_094903431.1</td><td>GAP1-N2+GAP1-M+GAP1-C+polar_tail+TM+beta-rich</td><td>TM+vWA(canonical)+YtKA-like+TM+??→ Tubulin+coiled_coil+??→ Tubulin+coiled_coil+HTH?+??→ TM+TM+TM+vWA(canonical)+TM+TM+TM+polar_tail→ Fibronectin-like_repeats+??→ ZnR+ZnR+DO-GTPase2.2+ZnR+ZnR→ GAP1-N2+GAP1-M+GAP1-C+polar_tail+TM+beta-rich*→</td><td>998</td><td>Paenibacillus odorifer</td><td>GCA_002264345.1</td><td>firmicutes</td></tr>
<tr><td>WP_094925398.1</td><td>GAP1-N2+GAP1-M+GAP1-C</td><td>PIN+WG_repeats+Fibronectin-like_repeats→ ZnR+ZnR+DO-GTPase2.2→ GAP1-N2+GAP1-M+GAP1-C*→ TM+TM+TM+TM+ZnR+ZnR+DO-GTPase2.2→</td><td>878</td><td>Bacillus sp. SA5d-4</td><td>GCA_002262935.1</td><td>firmicutes</td></tr>
<tr><td>WP_094938700.1</td><td>GAP1-N2+GAP1-M+GAP1-C+polar_tail+TM+beta-rich</td><td>ZnR+ZnR+DO-GTPase2.2+ZnR+ZnR→ GAP1-N2+GAP1-M+GAP1-C+polar_tail+TM+beta-rich*→</td><td>1078</td><td>Paenibacillus taichungensis</td><td>GCF_002264305.1</td><td>firmicutes</td></tr>
<tr><td>WP_095258437.1</td><td>GAP1-N2+GAP1-M+GAP1-C+polar_tail+TM+beta-rich</td><td>GAP1-N2+GAP1-M+GAP1-C+polar_tail+TM+beta-rich*→</td><td>746</td><td>Bacillus circulans</td><td>GCA_002271635.1</td><td>firmicutes</td></tr>
<tr><td>WP_095291104.1</td><td>GAP1-N2+GAP1-M+GAP1-C+polar_tail</td><td>Tubulin+coiled_coil+??→ Tubulin+coiled_coil+HTH?+??→ TM+TM+TM+vWA+TM+TM+TM+polar_tail→ Fibronectin-like_repeats+??→ ZnR+ZnR+DO-GTPase2.2→ GAP1-N2+GAP1-M+GAP1-C+polar_tail*→</td><td>1063</td><td>Paenibacillus sp. 7516</td><td>GCA_002272715.1</td><td>firmicutes</td></tr>
<tr><td>WP_095329927.1</td><td>GAP1-N2+GAP1-M+GAP1-C+polar_tail+TM+beta-rich</td><td>GAP1-N2+GAP1-M+GAP1-C+polar_tail+TM+beta-rich*→</td><td>746</td><td>Bacillus circulans</td><td>GCA_002271985.1</td><td>firmicutes</td></tr>
<tr><td>WP_095360032.1</td><td>GAP1-N2+GAP1-M+GAP1-C+polar_tail</td><td>Tubulin+coiled_coil+??→ Tubulin+coiled_coil+HTH?+??→ TM+TM+TM+vWA+TM+TM+TM+polar_tail→ Fibronectin-like_repeats+??→ ZnR+ZnR+DO-GTPase2.2→ GAP1-N2+GAP1-M+GAP1-C+polar_tail*→</td><td>1062</td><td>Paenibacillus sp. 7523-1</td><td>GCA_002276415.1</td><td>firmicutes</td></tr>
<tr><td>WP_095414977.1</td><td>GAP1-N2+GAP1-M+GAP1-C</td><td>ZnR+ZnR+DO-GTPase2.2→ GAP1-N2+GAP1-M+GAP1-C*→??+TM+??+beta-rich??→ GYF+GYF+TM+TM+TM→</td><td>731</td><td>Thermogutta terrifontis</td><td>GCA_002277955.1</td><td>planctomycetes</td></tr>
<tr><td>WP_095614595.1</td><td>GAP1-N2+GAP1-M+GAP1-C</td><td>TM+TM+TM+TM+ZnR+ZnR+DO-GTPase2.2→ GrpE→ HSP70→ HSP70+vWA(canonical)→ DnaJ+DnaJ+ZnR+ZnR+coiled_coil+Fibronectin-like_repeats→ ZnR+ZnR+DO-GTPase2.2→ GAP1-N2+GAP1-M+GAP1-C*→</td><td>850</td><td>Bifidobacterium criceti</td><td>GCF_002286935.1</td><td>actinobacteria</td></tr>
<tr><td>WP_095681866.1</td><td>GAP1-N2+GAP1-M+GAP1-C</td><td>GAP1-N2+GAP1-M+GAP1-C*→</td><td>772</td><td>Streptomyces sp. CLI2509</td><td>GCA_002288075.1</td><td>actinobacteria</td></tr>
<tr><td>WP_095709542.1</td><td>GAP1-N2+GAP1-M+GAP1-C</td><td>GAP1-N2+GAP1-M+GAP1-C*→</td><td>698</td><td>Streptomyces albidoflavus</td><td>GCA_002289305.1</td><td>actinobacteria</td></tr>
<tr><td>WP_095739059.1</td><td>GAP1-N2+GAP1-M+TM+GAP1-C</td><td>ZnR+ZnR+DO-GTPase2.2→ GAP1-N2+GAP1-M+TM+GAP1-C*→??+TM+??+beta-rich??→ GYF+GYF+TM+TM+TM→</td><td>642</td><td>Rhodopirellula sp. SM50</td><td>GCA_002289565.1</td><td>planctomycetes</td></tr>
<tr><td>WP_095741743.1</td><td>GAP1-N2+GAP1-M</td><td>FGS-fold_domain→ ZnR+DO-GTPase2.1→ GAP1-N2+GAP1-M*→</td><td>257</td><td>Rhodopirellula sp. SM50</td><td>GCA_002289565.1</td><td>planctomycetes</td></tr>
<tr><td>WP_095795750.1</td><td>GAP1-N2+GAP1-M+GAP1-C</td><td>TM+DUF4190+TM→ TM+DUF4190+TM→ polar_leader+TM+TM+TM→ TM+vWA(canonical)+Fibronectin-like_repeats+TM+beta-rich+lc_tail→ Tubulin+alpha_helical+??+??→ GAP_unknown1+TM+TM+GAP_unknown2→ HRDC-like?+Fibronectin-like_repeats→ GAP1-N2+GAP1-M+GAP1-C(frag)*→ beta-rich+vWA(canonical)→??+DO-GTPase2.2→</td><td>363</td><td>Mycobacterium chimaera</td><td>GCA_002292625.1</td><td>actinobacteria</td></tr>
</table>

acc	architecture	operon	len	species	gca	taxend
WP_095865817.1	GAP1-N2+GAP1-M+GAP1-C	polar_leader+TM+TM+TM→ TM+vWA(canonical)+Fibronectin-like_repeats+TM+beta-rich+lc_tail→ Tubulin+alpha_helical+??+??→ GAP_unknown1+TM+TM+GAP_unknown2→ HRDC-like?+Fibronectin-like_repeats→ GAP1-N2+GAP1-M+GAP1-C*→ beta-rich+vWA(canonical)→??+DO-GTPase2.2→	914	Rhodococcus sp. ACS1	GCA_002300155.1	actinobacteria
WP_095969334.1	GAP1-N2+GAP1-M+GAP1-C	GrpE→ HSP70→ TPR_containing_alpha_helical+Fibronectin-like_repeats→ ZnR+ZnR+DO-GTPase2.2→ GAP1-N2+GAP1-M+GAP1-C*→ TM+TM+TM+TM+ZnR+ZnR+DO-GTPase2.2→??→ HSP70→ vWA(canonical)→	798	Rhodococcus erythropolis	GCA_002303875.1	actinobacteria
WP_096095445.1	GAP1-N2+GAP1-M+GAP1-C	GAP1-N2+GAP1-M+GAP1-C*→	751	Streptomyces albidoflavus	GCA_002317035.1	actinobacteria
WP_096096486.1	GAP1-N2+GAP1-M+GAP1-C	GAP1-N2+GAP1-M+GAP1-C*→	789	Streptomyces albidoflavus	GCA_002317005.1	actinobacteria
WP_096168500.1	GAP1-N2+GAP1-M+GAP1-C	TM+vWA(canonical)+Fibronectin-like_repeats+TM+beta-rich+lc_tail→ Tubulin+alpha_helical+??+??→ GAP_unknown1+TM+TM+GAP_unknown2→ HRDC-like?+Fibronectin-like_repeats→ GAP1-N2+GAP1-M+GAP1-C*→ beta-rich+vWA(canonical)→??+DO-GTPase2.2→	978	Brevibacterium linens	GCA_002332445.1	actinobacteria
WP_096179030.1	GAP1-N2+GAP1-M+GAP1-C	GrpE→ HSP70→ TPR_containing_alpha_helical+Fibronectin-like_repeats→ ZnR+ZnR+DO-GTPase2.2→ GAP1-N2+GAP1-M+GAP1-C*→ TM+TM+TM+TM+ZnR+ZnR+DO-GTPase2.2→	693	Brevibacterium	GCA_002332325.1	actinobacteria
WP_096212307.1	GAP1-N2+GAP1-M+GAP1-C	TPR_containing_alpha_helical+Fibronectin-like_repeats→ ZnR+ZnR+DO-GTPase2.2→ GAP1-N2+GAP1-M+GAP1-C*→ PSE→ vWA(canonical)→ PP2C→ Pkinase+ZnR+beta-rich→	822	Streptomyces sp. 2323.1	GCA_900221035.1	actinobacteria
WP_096337284.1	GAP1-N2+GAP1-M+GAP1-C	polar_leader+TM+TM+TM→ TM+vWA(canonical)+Fibronectin-like_repeats+TM+beta-rich+lc_tail→ Tubulin+alpha_helical+??+??→ GAP_unknown1+TM+TM+GAP_unknown2→ HRDC-like?+Fibronectin-like_repeats→ GAP1-N2+GAP1-M+GAP1-C*→ beta-rich+vWA(canonical)→??+DO-GTPase2.2→ polar_leader+TM+TM+TM→	519	Corynebacterium accolens	GCA_002351405.1	actinobacteria
WP_096458547.1	GAP1-N2+GAP1-M+GAP1-C	polar_leader+TM+TM+TM→ TM+vWA(canonical)+Fibronectin-like_repeats+TM+beta-rich+lc_tail→ Tubulin+alpha_helical+??+??→ GAP_unknown1+TM+TM+GAP_unknown2→ HRDC-like?+Fibronectin-like_repeats→ GAP1-N2+GAP1-M+GAP1-C*→ beta-rich+vWA(canonical)→??+DO-GTPase2.2→	631	Corynebacterium glutamicum	GCF_002355155.1	actinobacteria
WP_096626114.1	GAP1-N2+GAP1-M+GAP1-C	GAP1-N2+GAP1-M+GAP1-C*→	811	Streptomyces sp. WZ.A104	GCA_002382885.1	actinobacteria
WP_096776860.1	GAP1-N2+GAP1-M+GAP1-C+polar_tail+TM+beta-rich	TM+vWA(canonical)+YtKA-like+TM+??→ Tubulin+coiled_coil+??→ Tubulin+coiled_coil+HTH?+??→ TM+TM+TM+vWA(canonical)+TM+TM+TM+polar_tail→ Fibronectin-like_repeats+??→ ZnR+ZnR+DO-GTPase2.2+ZnR+ZnR→ GAP1-N2+GAP1-M+GAP1-C+polar_tail+TM+beta-rich*→	992	Paenibacillus lautus	GCA_002407025.1	firmicutes
WP_096886357.1	GAP1-N2+GAP1-M+GAP1-C+polar_tail+TM+beta-rich	TM+vWA(canonical)+YtKA-like+TM+??→ Tubulin+coiled_coil+??→ Tubulin+coiled_coil+HTH?+??→ TM+TM+TM+vWA(canonical)+TM+TM+TM+polar_tail→ Fibronectin-like_repeats+??→ ZnR+ZnR+DO-GTPase2.2+ZnR+ZnR→ GAP1-N2+GAP1-M+GAP1-C+polar_tail+TM+beta-rich*→	833	Brevibacillus laterosporus	GCA_002412145.1	firmicutes
WP_097059724.1	GAP1-N2+GAP1-M+GAP1-C	polar_leader+TM+TM+TM→ TM+vWA(canonical)+Fibronectin-like_repeats+TM+beta-rich+lc_tail→ Tubulin+alpha_helical+??+??→ GAP_unknown1+TM+TM+GAP_unknown2→ HRDC-like?+Fibronectin-like_repeats→ GAP1-N2+GAP1-M+GAP1-C*→ beta-rich+vWA(canonical)→??+DO-GTPase2.2→	891	Salinibacterium xinjiangense	GCA_900230175.1	actinobacteria
WP_097156225.1	GAP1-N2+GAP1-M+GAP1-C+polar_tail+TM+beta-rich	TM+vWA(canonical)+YtKA-like+TM+??→ Tubulin+coiled_coil+??→ Tubulin+coiled_coil+HTH?+??→ TM+TM+TM+vWA(canonical)+TM+TM+TM+polar_tail→ Fibronectin-like_repeats+??→ ZnR+ZnR+DO-GTPase2.2+ZnR+ZnR→ GAP1-N2+GAP1-M+GAP1-C+polar_tail+TM+beta-rich*→	1056	Paenibacillus sp. RU26A	GCF_900221045.1	firmicutes
WP_097207620.1	GAP1-N2+GAP1-M+GAP1-C	Pkinase+TM→ ZnR+ZnR+DO-GTPase2.2→ GAP1-N2+GAP1-M+GAP1-C*→?→||←?←?||?→ Pkinase+low_complexity→	554	Geodermatophilus sabuli	GCA_900215145.1	actinobacteria
WP_097242200.1	GAP1-N2+GAP1-M+GAP1-C	GAP1-N2+GAP1-M+GAP1-C*→	804	Streptomyces microflavus	GCA_003268965.1	actinobacteria
WP_097632881.1	GAP1-N2+GAP1-M+GAP1-C	TPR_containing_alpha_helical+Fibronectin-like_repeats+extended_insert+Fibronectin-like_repeats→ ZnR+ZnR+DO-GTPase2.2→ GAP1-N2+GAP1-M+GAP1-C*→ TM+TM+ZnR+ZnR+DO-GTPase2.2→	947	Glycomyces fuscus	GCA_002529455.1	actinobacteria
WP_097871377.1	GAP1-N2+GAP1-M+GAP1-C	GAP1-N2+GAP1-M+GAP1-C*→	817	Streptomyces sp. rh34	GCF_002551245.1	actinobacteria
WP_097874842.1	GAP1-N2+GAP1-M+GAP1-C	GAP1-N2+GAP1-M+GAP1-C*→	864	Streptomyces sp. ms184	GCF_002551335.1	actinobacteria
WP_097885163.1	GAP1-N2+GAP1-M+GAP1-C	GAP1-N2+GAP1-M+GAP1-C*→	826	Streptomyces sp. st140	GCF_002551135.1	actinobacteria
WP_097894259.1	GAP1-N2+GAP1-M+GAP1-C	GAP1-N2+GAP1-M+GAP1-C*→	598	Streptomyces sp. st170	GCF_002551105.1	actinobacteria
WP_097912485.1	GAP1-N2+GAP1-M+GAP1-C	GAP1-N2+GAP1-M+GAP1-C→ TM+TM+TM+TM+ZnR+ZnR+DO-GTPase2.2+TM→ vWA(canonical)→ PP2C→ Pkinase+ZnR+beta-rich→||←?←?||GAP1-N2+GAP1-M+GAP1-C*→	838	Streptomyces sp. b84	GCF_002551465.1	actinobacteria
WP_097912493.1	GAP1-N2+GAP1-M+GAP1-C	TPR_containing_alpha_helical+Fibronectin-like_repeats→ ZnR+ZnR+DO-GTPase2.2→ GAP1-N2+GAP1-M+GAP1-C*→ TM+TM+TM+TM+ZnR+ZnR+DO-GTPase2.2+TM→ vWA(canonical)→ PP2C→ Pkinase+ZnR+beta-rich→||←?←?||GAP1-N2+GAP1-M+GAP1-C→	821	Streptomyces sp. b84	GCF_002551465.1	actinobacteria
WP_097923466.1	GAP1-N2+GAP1-M+GAP1-C	TPR_containing_alpha_helical+Fibronectin-like_repeats→ ZnR+ZnR+DO-GTPase2.2→ GAP1-N2+GAP1-M+GAP1-C*→ TM+TM+TM+TM+ZnR+ZnR+DO-GTPase2.2+TM→ vWA(canonical)→ PP2C→ Pkinase+ZnR+beta-rich→	821	Streptomyces sp. wa1063	GCF_002551045.1	actinobacteria
WP_097935545.1	GAP1-M+GAP1-C	GAP1-M+GAP1-C*→	708	Streptomyces sp. rh207	GCF_002551215.1	actinobacteria
WP_097946084.1	GAP1-N2+GAP1-M+GAP1-C	GAP1-N2+GAP1-M+GAP1-C*→	830	Streptomyces sp. ms115	GCF_002551345.1	actinobacteria
WP_097957547.1	GAP1-N2+GAP1-M+GAP1-C	ZnR+ZnR+DO-GTPase2.2→ GAP1-N2+GAP1-M+GAP1-C*→ TM+TM+TM+TM+ZnR+ZnR+DO-GTPase2.2+TM→ vWA(canonical)→ PP2C→ Pkinase+ZnR+beta-rich→	821	Streptomyces	GCF_002551015.1	actinobacteria
WP_097965558.1	GAP1-N2+GAP1-M+GAP1-C	GAP1-N2+GAP1-M+GAP1-C*→	828	Streptomyces sp. or20	GCF_002551305.1	actinobacteria
WP_097974788.1	GAP1-N2+GAP1-M+GAP1-C	GAP1-N2+GAP1-M+GAP1-C*→	829	Streptomyces sp. gb14	GCF_002551375.1	actinobacteria
WP_097985987.1	GAP1-N2+GAP1-M+GAP1-C	GAP1-N2+GAP1-M+GAP1-C*→	826	Streptomyces sp. f150	GCF_002551435.1	actinobacteria
WP_097991446.1	GAP1-N2+GAP1-M+GAP1-C	GAP1-N2+GAP1-M+GAP1-C*→	829	Streptomyces sp. f51	GCF_002551415.1	actinobacteria
WP_098009831.1	GAP1-N2+GAP1-M+GAP1-C	GAP1-N2+GAP1-M+GAP1-C*→	848	Streptomyces sp. sk226	GCF_002551185.1	actinobacteria
WP_098014371.1	GAP1-N2+GAP1-M+GAP1-C	GAP1-N2+GAP1-M+GAP1-C*→	828	Streptomyces sp. rh195	GCF_002551235.1	actinobacteria
WP_098018841.1	GAP1-N2+GAP1-M+GAP1-C	GAP1-N2+GAP1-M+GAP1-C*→	826	Streptomyces sp. b62	GCF_002551475.1	actinobacteria
WP_098025836.1	GAP1-N2+GAP1-M+GAP1-C	GAP1-N2+GAP1-M+GAP1-C*→	842	Streptomyces sp. st115	GCF_002551145.1	actinobacteria
WP_098245459.1	GAP1-N2+GAP1-M+GAP1-C	GAP1-N2+GAP1-M+GAP1-C*→ vWA(canonical)→ PP2C→ Pkinase+ZnR+beta-rich→||←TPR_containing_alpha_helical+Fibronectin-like_repeats	813	Streptomyces formicae	GCA_002556545.1	actinobacteria
WP_098530876.1	GAP1-N2+GAP1-M+GAP1-C	TM+vWA(canonical)+YtKA-like+TM+??→ Tubulin+coiled_coil+??→ Tubulin+coiled_coil+HTH?+??→ Fibronectin-like_repeats+??→ ZnR+ZnR+DO-GTPase2.2+ZnR+ZnR→ GAP1-N2+GAP1-M+GAP1-C*→	641	Bacillus sp. AFS076308	GCA_002565135.1	firmicutes
WP_098749063.1	GAP1-N2+GAP1-M+GAP1-C+polar_tail+TM+beta-rich	TM+vWA(canonical)+YtKA-like+TM+??→?→ Tubulin+coiled_coil+??→ Tubulin+coiled_coil+HTH?+??→ TM+TM+TM+vWA(canonical)+TM+TM+TM+polar_tail→ Fibronectin-like_repeats+??→ ZnR+ZnR+DO-GTPase2.2+ZnR+ZnR→ GAP1-N2+GAP1-M+GAP1-C+polar_tail+TM+beta-rich*→	991	Paenibacillus sp. EZ-K15	GCF_002573715.1	firmicutes
WP_098893115.1	GAP1-N2+GAP1-M+GAP1-C	GAP1-N2+GAP1-M+GAP1-C*→	827	Streptomyces sp. or3	GCF_002551295.1	actinobacteria
WP_098895192.1	GAP1-N2+GAP1-M+GAP1-C	GAP1-N2+GAP1-M+GAP1-C*→	787	Streptomyces sp. t99	GCF_002551065.1	actinobacteria
WP_098898971.1	GAP1-N2+GAP1-M+GAP1-C	GAP1-N2+GAP1-M+GAP1-C*→	835	Streptomyces sp. st77	GCF_002551175.1	actinobacteria
WP_098933598.1	GAP1-N2+GAP1-M+GAP1-C+polar_tail+TM+beta-rich	TM+vWA(canonical)+YtKA-like+TM+??→ Tubulin+coiled_coil+??→ Tubulin+coiled_coil+HTH?+??→ TM+TM+TM+vWA(canonical)+TM+TM+TM+polar_tail→ Fibronectin-like_repeats+??→ ZnR+ZnR+DO-GTPase2.2+ZnR+ZnR→ GAP1-N2+GAP1-M+GAP1-C+polar_tail+TM+beta-rich*→	939	Bacillus sp. AFS031507	GCA_002584635.1	firmicutes
WP_098996413.1	GAP1-N2+GAP1-M+GAP1-C	TM+vWA(canonical)+YtKA-like+TM+??→ Tubulin+coiled_coil+??→ Tubulin+coiled_coil+HTH?+??→ Fibronectin-like_repeats+??→ ZnR+ZnR+DO-GTPase2.2+ZnR+ZnR→ GAP1-N2+GAP1-M+GAP1-C*→	641	Bacillus sp. AFS037270	GCA_002585305.1	firmicutes
WP_099174916.1	GAP1-N2+GAP1-M+GAP1-C	GAP1-N2+GAP1-M+GAP1-C*→	804	Streptomyces sp. LamerLS-316	GCA_900090175.1	actinobacteria
WP_099202720.1	GAP1-N2+GAP1-M+GAP1-C	GAP1-N2+GAP1-M+GAP1-C*→	811	Streptomyces cinnamoneus	GCA_002727115.1	actinobacteria
WP_099219868.1	GAP1-N2+GAP1-M+GAP1-C	GAP1-N2+GAP1-M+GAP1-C*→	865	Streptomyces sp. AmelKG-D3	GCA_900090185.1	actinobacteria
WP_099318715.1	GAP1-N2+GAP1-M+GAP1-C	TM+DUF4190+TM→ polar_leader+TM+TM+TM→ TM+vWA(canonical)+Fibronectin-like_repeats+TM+beta-rich+lc_tail→ Tubulin+alpha_helical+??+??→ GAP_unknown1+TM+TM+GAP_unknown2→ HRDC-like?+Fibronectin-like_repeats→ GAP1-N2+GAP1-M+GAP1-C*→ beta-rich+vWA(canonical)→??+DO-GTPase2.2→	818	Mycobacterium kansasii	GCF_002705925.1	actinobacteria
WP_099321913.1	GAP1-N2+GAP1-M+GAP1-C	TM+TM+TM+TM+ZnR+ZnR+DO-GTPase2.2→?→ HSP70→ HSP70+vWA(canonical)→ DnaJ+DnaJ+ZnR+ZnR+coiled_coil+Fibronectin-like_repeats→ ZnR+ZnR+DO-GTPase2.2→ GAP1-N2+GAP1-M+GAP1-C*→	916	Anaeromassilibacillus sp. Marseille-P4683	GCF_900232885.1	firmicutes
WP_099431986.1	GAP1-N2+GAP1-M+GAP1-C	TM+TM+TM+TM+ZnR+ZnR+DO-GTPase2.2→ GrpE→ HSP70→ HSP70+vWA(canonical)→ DnaJ+DnaJ+ZnR+Fibronectin-like_repeats→ ZnR+ZnR+DO-GTPase2.2→ GAP1-N2+GAP1-M+GAP1-C*→	880	Collinsella aerofaciens	GCA_002736145.1	actinobacteria
WP_099517532.1	GAP1-N2+GAP1-M+GAP1-C+polar_tail+TM+beta-rich	TM+vWA(canonical)+YtKA-like+TM+??→ Tubulin+coiled_coil+??→ Tubulin+coiled_coil+HTH?+??→ TM+TM+TM+vWA(canonical)+TM+TM+TM+polar_tail→ Fibronectin-like_repeats+??→ ZnR+ZnR+DO-GTPase2.2+ZnR+ZnR→ GAP1-N2+GAP1-M+GAP1-C+polar_tail+TM+beta-rich*→	1053	Paenibacillus sp. BIHB4019	GCA_002741035.1	firmicutes
WP_099857417.1	GAP1-N2+GAP1-M+GAP1-C+polar_tail+TM+beta-rich	TM+vWA(canonical)+YtKA-like+TM+??→ Tubulin+coiled_coil+??→ Tubulin+coiled_coil+HTH?+??→ TM+TM+TM+vWA(canonical)+TM+TM+TM+polar_tail→ Fibronectin-like_repeats+??→ ZnR+ZnR+DO-GTPase2.2+ZnR+ZnR→ GAP1-N2+GAP1-M+GAP1-C+polar_tail+TM+beta-rich*→	1057	Paenibacillus polymyxa	GCF_002750415.1	firmicutes

acc	architecture	operon	len	species	gca	taxend
WP_100086938.1	GAP1-N2+GAP1-M+GAP1-C	polar_leader+TM+TM+TM→ TM+vWA(canonical)+Fibronectin-like_repeats+TM+beta-rich+lc_tail→ Tubulin+alpha_helical+??+??→ GAP_unknown1+TM+TM+GAP_unknown2→ HRDC-like?+Fibronectin-like_repeats→ GAP1-N2+GAP1-M+GAP1-C*→ beta-rich+vWA(canonical)→??+DO-GTPase2.2→ polar_leader+TM+TM+TM→	535	Corynebacterium striatum	GCA_002775065.1	actinobacteria
WP_100301097.1	GAP1-N2+GAP1-M+GAP1-C	PP2C→ Pkinase→?→?→?→ HSP70_domain_2+low_complexity+domain_betaX→ ZnR+ZnR+DO-GTPase2.2→ GAP1-N2+GAP1-M+GAP1-C*→ TM+TM+TM+TM+ZnR+ZnR+DO-GTPase2.2→	816	Streptomyces sp. CNZ306	GCA_002797655.1	actinobacteria
WP_100453397.1	GAP1-N2+GAP1-M+GAP1-C	GAP1-N2+GAP1-M+GAP1-C*→	789	Streptomyces sp. TSRI0384-2	GCA_002802945.1	actinobacteria
WP_100516541.1	GAP1-N2+GAP1-M+GAP1-C	HRDC-like?+Fibronectin-like_repeats→ GAP1-N2+GAP1-M+GAP1-C*→??+DO-GTPase2.2→	818	Mycolicibacterium goodii	GCA_002798385.1	actinobacteria
WP_100528329.1	GAP1-N2+GAP1-M+GAP1-C+polar_tail	Tubulin+coiled_coil+??→ Tubulin+coiled_coil+HTH?+??→ TM+TM+TM+vWA+TM+TM+TM+polar_tail→ Fibronectin-like_repeats+??→ ZnR+ZnR+DO-GTPase2.2→ GAP1-N2+GAP1-M+GAP1-C+polar_tail*→	1068	Paenibacillus sp. GM1FR	GCA_002803315.1	firmicutes
WP_100541388.1	GAP1-N2+GAP1-M+GAP1-C+polar_tail+TM+beta-rich	TM+vWA(canonical)+YtKA-like+TM+??→ Tubulin+coiled_coil+??→ Tubulin+coiled_coil+HTH?+??→ TM+TM+TM+vWA(canonical)+TM+TM+TM+polar_tail→ Fibronectin-like_repeats+??→ ZnR+ZnR+DO-GTPase2.2+ZnR+ZnR→ GAP1-N2+GAP1-M+GAP1-C+polar_tail+TM+beta-rich*→	989	Paenibacillus sp. GM2FR	GCA_002803325.1	firmicutes
WP_100562496.1	GAP1-N2+GAP1-M+GAP1-C	GAP1-N2+GAP1-M+GAP1-C*→	833	Streptomyces sp. CB02613	GCA_002803175.1	actinobacteria
WP_100619210.1	GAP1-N2+GAP1-M+GAP1-C	polar_leader+TM+TM+TM→ TM+vWA(canonical)+Fibronectin-like_repeats+TM+beta-rich+lc_tail→ Tubulin+alpha_helical+??+??→ GAP_unknown1+TM+TM+GAP_unknown2→ HRDC-like?+Fibronectin-like_repeats→ GAP1-N2+GAP1-M+GAP1-C*→ beta-rich+vWA(canonical)→??+DO-GTPase2.2→ polar_leader+TM+TM+TM→	535	Corynebacterium striatum	GCA_002804085.1	actinobacteria
WP_100862631.1	GAP1-N2+GAP1-M+GAP1-C	GAP1-N2+GAP1-M+GAP1-C*→	612	Streptomyces sp. Ag109_G2-1	GCF_002813225.1	actinobacteria
WP_101281520.1	GAP1-N2+GAP1-M+GAP1-C	GAP1-N2+GAP1-M+GAP1-C*→	789	Streptomyces sp. EAG2	GCA_002844215.1	actinobacteria
WP_101432330.1	GAP1-N2+GAP1-M+GAP1-C	TM+TM+TM+TM+ZnR+ZnR+DO-GTPase2.2→?→ HSP70→ HSP70+vWA(canonical)→ DnaJ+DnaJ+ZnR+ZnR+coiled_coil+Fibronectin-like_repeats→ ZnR+ZnR+DO-GTPase2.2→ GAP1-N2+GAP1-M+GAP1-C*→	916	Bifidobacterium asteroides	GCA_002846895.1	actinobacteria
WP_101557442.1	GAP1-N2+GAP1-M+GAP1-C	TM+vWA(canonical)+Fibronectin-like_repeats+TM+beta-rich+lc_tail→ Tubulin+alpha_helical+??+??→ GAP_unknown1+TM+TM+GAP_unknown2→ HRDC-like?+Fibronectin-like_repeats→ GAP1-N2+GAP1-M+GAP1-C*→ beta-rich+vWA(canonical)→??+DO-GTPase2.2→	978	Brevibacterium aurantiacum	GCA_003335365.1	actinobacteria
WP_101600910.1	GAP1-N2+GAP1-M+GAP1-C	GrpE→ HSP70→ TPR_containing_alpha_helical+Fibronectin-like_repeats→ ZnR+ZnR+DO-GTPase2.2→ GAP1-N2+GAP1-M+GAP1-C*→ TM+TM+TM+TM+ZnR+ZnR+DO-GTPase2.2→	677	Brevibacterium sp. Mu109	GCA_900169375.1	actinobacteria
WP_101623189.1	GAP1-N2+GAP1-M+GAP1-C	TM+TM+TM+TM+ZnR+ZnR+DO-GTPase2.2→ GrpE→ HSP70→ HSP70+vWA(canonical)→ DnaJ+DnaJ+ZnR+ZnR+coiled_coil+Fibronectin-like_repeats→ ZnR+ZnR+DO-GTPase2.2→ GAP1-N2+GAP1-M+GAP1-C*→	969	Bifidobacterium parmae	GCF_002860365.1	actinobacteria
WP_101648094.1	GAP1-N2+GAP1-M+GAP1-C	TM+vWA(canonical)+YtKA-like+TM+??→ Tubulin+coiled_coil+??→ Tubulin+coiled_coil+HTH?+??→ Fibronectin-like_repeats+??→ ZnR+ZnR+DO-GTPase2.2+ZnR+ZnR→ GAP1-N2+GAP1-M+GAP1-C*→	562	Bacillus cucumis	GCA_002860255.1	firmicutes
WP_101669369.1	GAP1-N2+GAP1-M+GAP1-C+polar_tail+TM+beta-rich	TM+vWA(canonical)+YtKA-like+TM+??→ Tubulin+coiled_coil+??→ Tubulin+coiled_coil+HTH?+??→ TM+TM+TM+vWA(canonical)+TM+TM+TM+polar_tail→ Fibronectin-like_repeats+??→ ZnR+ZnR+DO-GTPase2.2+ZnR+ZnR→ GAP1-N2+GAP1-M+GAP1-C+polar_tail+TM+beta-rich*→	833	Brevibacillus laterosporus	GCA_002865525.1	firmicutes
WP_101729374.1	GAP1-N2+GAP1-M+GAP1-C+polar_tail+TM+beta-rich	GAP1-N2+GAP1-M+GAP1-C+polar_tail+TM+beta-rich*→	750	Clostridiales bacterium mt7	GCF_900199695.1	firmicutes
WP_101734776.1	GAP1-N2+GAP1-M+GAP1-C	polar_leader+TM+TM+TM→ TM+vWA(canonical)+Fibronectin-like_repeats+TM+beta-rich+lc_tail→ Tubulin+alpha_helical+??+??→ GAP_unknown1+TM+TM+GAP_unknown2→ HRDC-like?+Fibronectin-like_repeats→ GAP1-N2+GAP1-M+GAP1-C*→?→??+DO-GTPase2.2→?→||←?←?←HSP70	326	Corynebacterium riegelii	GCA_002861325.1	actinobacteria
WP_101736469.1	GAP1-N2+GAP1-M+GAP1-C	polar_leader+TM+TM+TM→ TM+vWA(canonical)+Fibronectin-like_repeats+TM+beta-rich+lc_tail→ Tubulin+alpha_helical+??+??→ GAP_unknown1+TM+TM+GAP_unknown2→ HRDC-like?+Fibronectin-like_repeats→ GAP1-N2+GAP1-M+GAP1-C*→ beta-rich+vWA(canonical)→??+DO-GTPase2.2→ polar_leader+TM+TM+TM→	577	Corynebacterium aurimucosum	GCA_002861385.1	actinobacteria
WP_102234493.1	GAP1-N2+GAP1-M+GAP1-C	polar_leader+TM+TM+TM→ TM+vWA(canonical)+Fibronectin-like_repeats+TM+beta-rich+lc_tail→ Tubulin+alpha_helical+??+??→ GAP_unknown1+TM+TM+GAP_unknown2→ HRDC-like?+Fibronectin-like_repeats→ GAP1-N2+GAP1-M+GAP1-C*→ beta-rich+vWA(canonical)→??+DO-GTPase2.2→ polar_leader+TM+TM+TM→	565	Corynebacterium aurimucosum	GCA_002871735.1	actinobacteria
WP_102239224.1	GAP1-N2+GAP1-M+GAP1-C	polar_leader+TM+TM+TM→ TM+vWA(canonical)+Fibronectin-like_repeats+TM+beta-rich+lc_tail→ Tubulin+alpha_helical+??+??→ GAP_unknown1+TM+TM+GAP_unknown2→ HRDC-like?+Fibronectin-like_repeats→ GAP1-N2+GAP1-M+GAP1-C*→?→??+DO-GTPase2.2→?→||←?←?←TM+TM+vWA(canonical)+TM←TM+TM+vWA(canonical)+TM	962	Brevibacterium paucivorans	GCA_002871875.1	actinobacteria
WP_102240421.1	GAP1-N2+GAP1-M+GAP1-C	GrpE→ HSP70→ TPR_containing_alpha_helical+Fibronectin-like_repeats→ ZnR+ZnR+DO-GTPase2.2→ GAP1-N2+GAP1-M+GAP1-C*→ TM+TM+TM+TM+ZnR+ZnR+DO-GTPase2.2→	808	Actinomyces graevenitzii	GCA_002871975.1	actinobacteria
XP_021300519.1	GAP1-N2+GAP1-M+GAP1-C	GAP1-N2+GAP1-M+GAP1-C(frag)*→	514	Herrania umbratica	GCF_002168275.1	viridiplantae

NucA endonuclease-containing systems

<table>
<thead>
<tr><th>acc</th><th>architecture</th><th>operon</th><th>len</th><th>species</th><th>gca</th><th>taxend</th></tr>
</thead>
<tbody>
<tr><td>WP_053082004.1</td><td>EAD1+Trypsin+EAD1+STAND</td><td>EAD1+Trypsin+EAD1+STAND*→ low_complexity+TM+NucA(inactive)+iSvmX2→||←?←CASPASE+??||Trypsin→</td><td>841</td><td>Methylobacterium aquaticum</td><td>GCF_001043915.1</td><td>alphaproteobacteria</td></tr>
<tr><td>WP_084627253.1</td><td>EAD1+Trypsin+TM+TM+disordered</td><td>EAD1+Trypsin+TM+TM+disordered*→</td><td>465..487&TM</td><td>Mesorhizobium sp. LSHC422A00</td><td>GCF_000503075.1</td><td>-</td></tr>
<tr><td>WP_084629776.1</td><td>EAD1+Trypsin+TM+NPLC_P60</td><td>EAD1+Trypsin+TM+NPLC_P60*→</td><td>768</td><td>Mesorhizobium sp. LSHC412B00</td><td>GCF_000503035.1</td><td>alphaproteobacteria</td></tr>
<tr><td>WP_023715941.1</td><td>EAD1+Trypsin+TM+NPLC_P60</td><td>EAD1+Trypsin+TM+NPLC_P60*→</td><td>757</td><td>Mesorhizobium sp. LSHC422A00</td><td>GCF_000503075.1</td><td>alphaproteobacteria</td></tr>
<tr><td>ESX81659.1</td><td>EAD1+Trypsin+TM+NPLC_P60</td><td>EAD1+Trypsin+TM+NPLC_P60*→</td><td>746</td><td>Mesorhizobium sp. LSHC412B00</td><td>GCA_000503035.1</td><td>alphaproteobacteria</td></tr>
<tr><td>WP_092161432.1</td><td>EAD1+Trypsin</td><td>Trypsin_2+NucA+Calcineurin←?←?||EAD1+Trypsin*→?→??+TM+??+??+iSvmX2→</td><td>409</td><td>Bosea sp. DSM 18164</td><td>GCF_900112185.1</td><td>alphaproteobacteria</td></tr>
<tr><td>WP_083786386.1</td><td>EAD1+Trypsin</td><td>lysozyme+peptidase_MD→||←Trypsin+NucA||EAD1+Trypsin*→?→??+TM+SH3+iSvmX2→</td><td>401</td><td>Methylobacterium nodulans</td><td>GCF_000022085.1</td><td>alphaproteobacteria</td></tr>
<tr><td>WP_012044960.1</td><td>EAD1+Trypsin</td><td>EAD1+Trypsin*→?→ Ploop_NTPase+TM+??→</td><td>382</td><td>Bradyrhizobium sp. BTAi1</td><td>GCF_000015165.1</td><td>alphaproteobacteria</td></tr>
<tr><td>WP_082364706.1</td><td>EAD1+Trypsin</td><td>EAD1+Trypsin*→?→ AAA_ATPase(inactive)+TM+SH3+iSvmX2→||←PSE||?→ Trypsin+NucA→</td><td>381</td><td>Novosphingobium sp. AAP93</td><td>GCF_001296055.1</td><td>alphaproteobacteria</td></tr>
<tr><td>WP_083554684.1</td><td>EAD1+Trypsin</td><td>EAD1+Trypsin*→?→ AAA_ATPase(inactive)+TM+SH3+iSvmX2→||←PSE||?→ Trypsin+NucA→</td><td>381</td><td>Novosphingobium sp. NDB2Meth1</td><td>GCF_900117425.1</td><td>alphaproteobacteria</td></tr>
<tr><td>WP_046612924.1</td><td>EAD1+Trypsin</td><td>Trypsin+NucA||lysozyme+PGBD→ EAD1+Trypsin*→ EADunk+helical_domain→ low_complexity+TM+NucA(inactive)+iSvmX2→ GDSL→</td><td>373</td><td>Neorhizobium galegae</td><td>GCF_000985995.1</td><td>alphaproteobacteria</td></tr>
<tr><td>WP_056528335.1</td><td>EAD1+Trypsin</td><td>Trypsin+NucA→ EAD1+Trypsin*→?→ STAND+TM+iSvmX2→</td><td>373</td><td>Sphingomonas sp. Leaf339</td><td>GCF_001426195.1</td><td>alphaproteobacteria</td></tr>
<tr><td>WP_068292622.1</td><td>EAD1+Trypsin</td><td>EAD1+Trypsin*→ EADunk+helical_domain→??+TM+??→</td><td>372</td><td>Labrys sp. WJW</td><td>GCF_001676615.1</td><td>alphaproteobacteria</td></tr>
<tr><td>WP_015667733.1</td><td>EAD7+helical_domain</td><td>EAD1+Trypsin→ EAD7+helical_domain→ STAND+TM+TPRs→</td><td>370</td><td>Bradyrhizobium oligotrophicum</td><td>GCF_000344805.1</td><td>alphaproteobacteria</td></tr>
<tr><td>PLR28184.1</td><td>EAD1+Trypsin</td><td>EAD1+Trypsin*→?→||←?←?||Trypsin→</td><td>369</td><td>Caulobacter zeae</td><td>GCA_002858925.1</td><td>alphaproteobacteria</td></tr>
<tr><td>WP_068414758.1</td><td>EAD1+Trypsin</td><td>Trypsin_2+NucA+Subtilisin→ Trypsin→ bDLD2+Trypsin+STAND→ STAND+wHTH+TPRs→ EAD1+Trypsin*→?→ low_complexity+Pentapeptide_repeats+TPRs→</td><td>368</td><td>Labrenzia sp. OB1</td><td>GCF_001624695.1</td><td>alphaproteobacteria</td></tr>
<tr><td>WP_003613698.1</td><td>EAD1+Trypsin</td><td>subtilase→ EAD1+Trypsin*→ STAND+TM+TPRs→||←PLD+PLD</td><td>368</td><td>Methylosinus</td><td>GCF_000178815.2</td><td>alphaproteobacteria</td></tr>
<tr><td>WP_096786388.1</td><td>EAD1+Trypsin</td><td>GDSL←lysozyme+PGBD||??+Trypsin→ EAD1+Trypsin*→ EADunk+helical_domain→ low_complexity+TM+NucA(inactive)+iSvmX2→</td><td>368</td><td>Rhodobacter sp. CZR27</td><td>GCF_002407205.1</td><td>alphaproteobacteria</td></tr>
<tr><td>WP_101341742.1</td><td>EAD1+Trypsin</td><td>GDSL←lysozyme+PGBD||??+Trypsin→ EAD1+Trypsin*→ EADunk+helical_domain→ low_complexity+TM+NucA(inactive)+iSvmX2→</td><td>363</td><td>Rhodobacter azotoformans</td><td>GCF_002844445.1</td><td>alphaproteobacteria</td></tr>
<tr><td>WP_011909377.1</td><td>EAD1+Trypsin</td><td>GDSL←lysozyme+PGBD||??+Trypsin→ EAD1+Trypsin*→ EADunk+helical_domain→ low_complexity+TM+NucA(inactive)+iSvmX2→</td><td>363</td><td>Rhodobacter sphaeroides</td><td>GCF_000016405.1</td><td>alphaproteobacteria</td></tr>
<tr><td>WP_091925409.1</td><td>EAD1+Trypsin</td><td>EAD1+Trypsin*→||←?←?←Ploop_NTPase+TM+??</td><td>362</td><td>Phyllobacterium sp. CL33Tsu</td><td>GCF_900113985.1</td><td>alphaproteobacteria</td></tr>
<tr><td>WP_027229487.1</td><td>EAD1+Trypsin</td><td>EAD1+Trypsin*→||←?←?←Ploop_NTPase+TM+??</td><td>362</td><td>Phyllobacterium sp. UNC302MFCol5.2</td><td>GCF_000686005.1</td><td>alphaproteobacteria</td></tr>
<tr><td>WP_091943939.1</td><td>EAD1+Trypsin</td><td>EAD1+Trypsin*→||←?←?←Ploop_NTPase+TM+??</td><td>362</td><td>Phyllobacterium sp. YR620</td><td>GCF_900104165.1</td><td>alphaproteobacteria</td></tr>
<tr><td>WP_018082322.1</td><td>EAD1+Trypsin</td><td>Trypsin+NucA→?→ EAD1+Trypsin→ EAD1+Trypsin→?→ AAA_ATPase(inactive)+TM+SH3+iSvmX2→</td><td>361</td><td>Asticcacaulis benevestitus</td><td>GCF_000376105.1</td><td>alphaproteobacteria</td></tr>
<tr><td>WP_069910982.1</td><td>EAD1+Trypsin</td><td>EAD1+Trypsin*→</td><td>359</td><td>Devosia insulae</td><td>GCF_000970465.2</td><td>alphaproteobacteria</td></tr>
<tr><td>SDW97151.1</td><td>EAD1+Trypsin</td><td>Trypsin+NucA||EAD1+Trypsin*→?→??+TM+SH3+iSvmX2→ GDSL→</td><td>359</td><td>Paracoccus sanguinis</td><td>GCA_900106665.1</td><td>alphaproteobacteria</td></tr>
<tr><td>WP_023690135.1</td><td>EAD1+Trypsin</td><td>EAD1+Trypsin*→?→ STAND+wHTH+TPRs→</td><td>357</td><td>Mesorhizobium sp. LSJC269B00</td><td>GCF_000502815.1</td><td>alphaproteobacteria</td></tr>
<tr><td>WP_066997244.1</td><td>EAD1+Trypsin</td><td>Trypsin←subtilase←?←?←??+GDSL←PSE||EAD1+Trypsin*→?→ STAND+wHTH+TPRs→</td><td>355</td><td>Mesorhizobium sp. WSM3873</td><td>GCF_001672445.1</td><td>alphaproteobacteria</td></tr>
<tr><td>WP_069907699.1</td><td>EAD1+Trypsin</td><td>Trypsin→ EAD1+lysozyme→ EAD1+Trypsin→ EAD1+helical_domain→ low_complexity+TM+NucA(inactive)+iSvmX2→ Trypsin+NucA→</td><td>347</td><td>Devosia insulae</td><td>GCF_000970465.2</td><td>alphaproteobacteria</td></tr>
<tr><td>WP_052172061.1</td><td>EAD1+Trypsin</td><td>Trypsin+NucA||EAD1+Trypsin*→</td><td>347</td><td>Paracoccus sanguinis</td><td>GCF_000763825.1</td><td>alphaproteobacteria</td></tr>
<tr><td>WP_084582114.1</td><td>EAD1+Trypsin</td><td>Trypsin+NucA→?→?→ AAA_ATPase(inactive)+TM+SH3+iSvmX2→?→ EAD1+Trypsin*→</td><td>346</td><td>Sphingomonas azotifigens</td><td>GCF_002091475.1</td><td>alphaproteobacteria</td></tr>
<tr><td>CCD95925.1</td><td>EAD1+Trypsin</td><td>EAD1+Trypsin→ EAD1+??+Ploop_NTPase+??→</td><td>345</td><td>Bradyrhizobium sp. ORS 375</td><td>GCA_000239775.2</td><td>alphaproteobacteria</td></tr>
<tr><td>WP_050995937.1</td><td>EAD1+Trypsin</td><td>EAD1+Trypsin*→||←EAD5+Trypsin←STAND+wHTH+TPRs←?←PLD+PLD←Trypsin</td><td>344</td><td>Bradyrhizobium yuanmingense</td><td>GCF_000261805.1</td><td>alphaproteobacteria</td></tr>
<tr><td>WP_006205238.1</td><td>EAD1+Trypsin</td><td>Trypsin+NucA→ EAD1+Trypsin*→ EADunk+helical_domain→</td><td>327</td><td>Mesorhizobium amorphae</td><td>GCF_000233995.1</td><td>alphaproteobacteria</td></tr>
<tr><td>WP_023829837.1</td><td>EAD1+Trypsin</td><td>Trypsin+NucA→ EAD1+Trypsin*→ EADunk+helical_domain→ low_complexity+TM+NucA(inactive)+iSvmX2→</td><td>327</td><td>Mesorhizobium sp. L103C120A0</td><td>GCF_000502255.1</td><td>alphaproteobacteria</td></tr>
<tr><td>WP_023824922.1</td><td>EAD1+Trypsin</td><td>Trypsin+NucA→ EAD1+Trypsin*→ PSE→ EADunk+helical_domain→ low_complexity+TM+NucA(inactive)+iSvmX2→</td><td>327</td><td>Mesorhizobium sp. L103C565B0</td><td>GCF_000502295.1</td><td>alphaproteobacteria</td></tr>
<tr><td>WP_023730574.1</td><td>EAD1+Trypsin</td><td>Trypsin+NucA→ EAD1+Trypsin*→ EADunk+helical_domain→ low_complexity+TM+NucA(inactive)+iSvmX2→</td><td>327</td><td>Mesorhizobium sp. LNJC405B00</td><td>GCF_000502615.1</td><td>alphaproteobacteria</td></tr>
<tr><td>WP_023702264.1</td><td>EAD1+Trypsin</td><td>Trypsin+NucA→ EAD1+Trypsin*→ EADunk+helical_domain→ low_complexity+TM+NucA(inactive)+iSvmX2→</td><td>327</td><td>Mesorhizobium sp. LSJC264A00</td><td>GCF_000502755.1</td><td>alphaproteobacteria</td></tr>
<tr><td>WP_023696135.1</td><td>EAD1+Trypsin</td><td>Trypsin+NucA→ EAD1+Trypsin*→ EADunk+helical_domain→ low_complexity+TM+NucA(inactive)+iSvmX2→</td><td>327</td><td>Mesorhizobium sp. LSJC265A00</td><td>GCF_000502775.1</td><td>alphaproteobacteria</td></tr>
<tr><td>WP_017270711.1</td><td>EAD1+Trypsin</td><td>GDSL+CASPASE→ subtilase→?→ EAD1+Trypsin*→</td><td>327</td><td>Sinorhizobium meliloti</td><td>GCF_000427585.1</td><td>alphaproteobacteria</td></tr>
<tr><td>WP_023755269.1</td><td>EAD1+Trypsin</td><td>Trypsin+NucA→ EAD1+Trypsin*→ EADunk+helical_domain→ low_complexity+TM+NucA(inactive)+iSvmX2→</td><td>327</td><td>unclassified Mesorhizobium</td><td>GCF_000502475.1</td><td>alphaproteobacteria</td></tr>
<tr><td>WP_023745069.1</td><td>EAD1+Trypsin</td><td>Trypsin+NucA→ EAD1+Trypsin*→ EADunk+helical_domain→ low_complexity+TM+NucA(inactive)+iSvmX2→</td><td>327</td><td>unclassified Mesorhizobium</td><td>GCF_000502555.1</td><td>alphaproteobacteria</td></tr>
<tr><td>WP_023710499.1</td><td>EAD1+Trypsin</td><td>Trypsin+NucA→ EAD1+Trypsin*→ EADunk+helical_domain→ low_complexity+TM+NucA(inactive)+iSvmX2→</td><td>327</td><td>unclassified Mesorhizobium</td><td>GCF_000502695.1</td><td>alphaproteobacteria</td></tr>
</tbody>
</table>

<table>
<tr><th>acc</th><th>architecture</th><th>operon</th><th>len</th><th>species</th><th>gca</th><th>taxend</th></tr>
<tr><td>WP_023691332.1</td><td>EAD1+Trypsin</td><td>NucA→ EAD1+Trypsin*→ EADunk+helical_domain→ low_complexity+TM+NucA(inactive)+iSvmX2→</td><td>327</td><td>unclassified Mesorhizobium</td><td>GCF_000502795.1</td><td>alphaproteobacteria</td></tr>
<tr><td>WP_023688493.1</td><td>EAD1+Trypsin</td><td>Trypsin+NucA→ EAD1+Trypsin*→ EADunk+helical_domain→ low_complexity+TM+NucA(inactive)+iSvmX2→</td><td>327</td><td>unclassified Mesorhizobium</td><td>GCF_000502815.1</td><td>alphaproteobacteria</td></tr>
<tr><td>WP_023738836.1</td><td>EAD1+Trypsin</td><td>Trypsin+NucA→ EAD1+Trypsin*→ EADunk+helical_domain→ low_complexity+TM+NucA(inactive)+iSvmX2→</td><td>327</td><td>unclassified Mesorhizobium</td><td>GCF_000502995.1</td><td>alphaproteobacteria</td></tr>
<tr><td>WP_023726582.1</td><td>EAD1+Trypsin</td><td>Trypsin+NucA→ EAD1+Trypsin*→ EADunk+helical_domain→ low_complexity+TM+NucA(inactive)+iSvmX2→</td><td>327</td><td>unclassified Mesorhizobium</td><td>GCF_000503035.1</td><td>alphaproteobacteria</td></tr>
<tr><td>WP_023668985.1</td><td>EAD1+Trypsin</td><td>Trypsin+NucA→ EAD1+Trypsin*→ EADunk+helical_domain→ low_complexity+TM+NucA(inactive)+iSvmX2→</td><td>327</td><td>unclassified Mesorhizobium</td><td>GCF_000503155.1</td><td>alphaproteobacteria</td></tr>
<tr><td>WP_023832170.1</td><td>EAD1+Trypsin</td><td>Trypsin+NucA→ EAD1+Trypsin*→ EADunk+helical_domain→ low_complexity+TM+NucA(inactive)+iSvmX2→</td><td>325</td><td>Mesorhizobium sp. L103C119B0</td><td>GCF_000502235.1</td><td>alphaproteobacteria</td></tr>
<tr><td>WP_014497152.1</td><td>Trypsin+EAD1+coiled_coil</td><td>calcineurin(low_complexity_insert)→ Trypsin+EAD1+coiled_coil*→ wHTH+TM+PPRs+TPRs→</td><td>643</td><td>Bradyrhizobium japonicum</td><td>GCF_000284375.1</td><td>alphaproteobacteria</td></tr>
<tr><td>WP_049824938.1</td><td>Trypsin+EAD1+coiled_coil</td><td>calcineurin(low_complexity_insert)→ Trypsin+EAD1+coiled_coil*→ wHTH+TM+PPRs+TPRs→</td><td>611</td><td>Bradyrhizobium japonicum</td><td>GCF_000472985.1</td><td>alphaproteobacteria</td></tr>
<tr><td>WP_041955985.1</td><td>Trypsin+EAD1+coiled_coil</td><td>calcineurin(low_complexity_insert)→ Trypsin+EAD1+coiled_coil*→ wHTH+TM+PPRs+TPRs→</td><td>643</td><td>Bradyrhizobium japonicum</td><td>GCF_000773865.1</td><td>alphaproteobacteria</td></tr>
<tr><td>WP_015666606.1</td><td>Trypsin+EAD1+coiled_coil</td><td>calcineurin(low_complexity_insert)→ Trypsin+EAD1+coiled_coil*→ wHTH+TM+PPRs+TPRs→</td><td>674</td><td>Bradyrhizobium oligotrophicum</td><td>GCF_000344805.1</td><td>alphaproteobacteria</td></tr>
<tr><td>WP_065745691.1</td><td>Trypsin+EAD1+coiled_coil</td><td>calcineurin(low_complexity_insert)→ Trypsin+EAD1+coiled_coil*→ wHTH+TM+PPRs+TPRs→</td><td>661</td><td>Bradyrhizobium sp. LMTR 3</td><td>GCF_001693485.1</td><td>alphaproteobacteria</td></tr>
<tr><td>WP_063691614.1</td><td>Trypsin+EAD1+coiled_coil</td><td>calcineurin(low_complexity_insert)→ Trypsin+EAD1+coiled_coil*→ wHTH+TM+PPRs+TPRs→</td><td>643</td><td>Bradyrhizobium stylosanthis</td><td>GCF_001641335.1</td><td>alphaproteobacteria</td></tr>
<tr><td>BAM90641.1</td><td>EAD7+Lysozyme</td><td>EAD1+Trypsin→ EAD7+Lysozyme*→ STAND+TM+TPRs→</td><td>440</td><td>Bradyrhizobium oligotrophicum S58</td><td>GCA_000344805.1</td><td>alphaproteobacteria</td></tr>
<tr><td>WP_091921759.1</td><td>IG-like+EAD1</td><td>IG-like+EAD1*→</td><td>233..255&TM</td><td>Phyllobacterium sp. CL33Tsu</td><td>GCF_900113985.1</td><td>-</td></tr>
<tr><td>WP_027231496.1</td><td>IG-like+EAD1</td><td>IG-like+EAD1*→</td><td>233..255&TM</td><td>Phyllobacterium sp. UNC302MFCol5.2</td><td>GCF_000686005.1</td><td>-</td></tr>
<tr><td>WP_011082909.1</td><td>HNH+PNPase+EAD7</td><td>HNH+PNPase+EAD7*→</td><td>474</td><td>Bradyrhizobium</td><td>GCF_000011365.1</td><td>alphaproteobacteria</td></tr>
<tr><td>WP_069907698.1</td><td>EAD1+TM+coiled_coil</td><td>Trypsin→ EAD1+TM+coiled_coil*→ EAD1+Trypsin→ EAD1+helical_domain→ low_complexity+TM+NucA(inactive)+iSvmX2→ Trypsin+NucA→</td><td>359</td><td>Devosia insulae</td><td>-</td><td>alphaproteobacteria</td></tr>
<tr><td>OWK18362.1</td><td>EAD1</td><td>Trypsin→ Trypsin→ NucA→ EAD1*→ Trypsin→ EAD1→ EADunk+helical_domain→??+TM+??→ NucA→</td><td>108</td><td>Mesorhizobium amorphae CCBAU 01583</td><td>GCA_002200945.1</td><td>alphaproteobacteria</td></tr>
<tr><td>WP_089387726.1</td><td>EAD1+Trypsin</td><td>Trypsin→||←?||EAD1+Trypsin→ EAD1+STAND→ STAND+wHTH+TPRs→</td><td>349</td><td>Paracoccus sediminis</td><td>GCF_900188295.1</td><td>alphaproteobacteria</td></tr>
</table>

Effector Associated Constant Component 1 (EACC1)-containing two gene conflict system

acc	architecture	operon	len	species	gca	taxend
AAZ23093.1	EACC1	EACC1*→ CASPASE+Pkinase+??→	139	Streptomyces fradiae	-	actinobacteria
AEM80885.1	EACC1	TetR_N+TetR_C_6→ EACC1*→ CASPASE+low-complexity+TM+PBPB→	129	Streptomyces violaceusniger Tu 4113	GCA_000147815.3	actinobacteria
AEM84200.1	EACC1	EACC1*→ CASPASE+TM+TM→	127	Streptomyces violaceusniger Tu 4113	GCA_000147815.3	actinobacteria
AEV85592.1	EACC1	EACC1*→ CASPASE+JAB→	115	Actinoplanes sp. SE50/110	GCA_000237145.1	actinobacteria
AGP55504.1	EACC1	EACC1*→ CASPASE+low-complexity+TM+PBPB→	132	Streptomyces rapamycinicus NRRL 5491	GCA_000418455.1	actinobacteria
AGP55984.1	EACC1	EACC1*→	147	Streptomyces rapamycinicus NRRL 5491	GCA_000418455.1	actinobacteria
AGZ40359.1	EACC1	EACC1*→	116	Actinoplanes friuliensis DSM 7358	GCA_000494755.1	actinobacteria
AGZ45935.1	EACC1	EACC1*→ CASPASE+DUF179→ SIR2+??→??→	117	Actinoplanes friuliensis DSM 7358	GCA_000494755.1	actinobacteria
AHH15007.1	EACC1	EACC1*→ TM+TM→	86	Nocardia nova SH22a	GCA_000523235.1	actinobacteria
AHH97879.1	EACC1	polar_lead?+TM+TM+TM+polar_tail?+low-complexity→??→ EACC1*→ CASPASE+TPRs→?→ CASPASE+??+TPRs?→	119	Kutzneria albida DSM 43870	GCA_000525635.1	actinobacteria
AIA00737.1	EACC1	EACC1*→ CASPASE+TM+TM+bPH_domain?→	134	Streptomyces albulus	GCA_000695235.1	actinobacteria
AJE85450.1	EACC1	EACC1*→ CASPASE+HisKA+HATPase_c→	139	Streptomyces albus	GCA_000827005.1	actinobacteria
AJP05873.1	EACC1	EACC1*→	107	Streptomyces cyaneogriseus subsp. noncyanogenus	GCA_000931445.1	actinobacteria
AJT62559.1	EACC1	EACC1*→ CASPASE+low-complexity+TM+PBPB→	155	Streptomyces lydicus	GCA_000952035.2	actinobacteria
AKG44342.1	EACC1	TM+TM+TM+PP2C→ EACC1*→ CASPASE+PBPB+vWA→	152	Streptomyces xiamenensis	GCA_000993785.2	actinobacteria
AKJ15647.1	EACC1	EACC1*→ CASPASE+TPRs→	138	Streptomyces incarnatus	GCA_001027185.1	actinobacteria
AKN69758.1	EACC1	??→??→ EACC1*→ CASPASE+??→	129	Streptomyces sp. PBH53	GCA_001040905.1	actinobacteria
AKN75595.1	EACC1	EACC1*→ CASPASE+STAND+polar_region+TM+BetaPropeller→	128	Streptomyces sp. PBH53	GCA_001040905.1	actinobacteria
ANO41895.1	EACC1	TPRs→ EACC1*→ CASPASE+TPRs→	114	Streptomyces sp. SAT1	-	actinobacteria
AOP46710.1	EACC1	EACC1*→ CASPASE+FGS_fold-domain→??→??→ TM+TM+BaxI_1→?→ NUDIX→	191	Streptomyces lydicus	GCA_001729485.1	actinobacteria
AOR36787.1	EACC1	EACC1*→ CASPASE+STAND+polar_region+TM+BetaPropeller→	121	Streptomyces puniciscabiei	GCA_001735805.1	actinobacteria
AOS63691.1	EACC1	EACC1*→ CASPASE+??+SF1-helicase+Vsr(PDDEXK)?→	126	Actinoalloteichus hymeniacidonis	GCA_001747425.1	actinobacteria
APA99415.1	EACC1	EACC1*→ CASPASE+STAND+TPRs→	123	Nocardia seriolae	GCA_001865855.1	actinobacteria
APE36223.1	EACC1	EACC1*→ CASPASE+TPRs→	121	Nocardia sp. Y48	GCA_001886715.1	actinobacteria
APE38064.1	EACC1	EACC1*→	109	Nocardia sp. Y48	GCA_001886715.1	actinobacteria
APU44471.1	EACC1	EACC1*→ CASPASE+RE_Eco29kI+??→	117	Streptomyces sp. TN58	GCA_001941845.1	actinobacteria
AQU70505.1	EACC1	EACC1*→ CASPASE+??→	130	Streptomyces niveus	GCA_002009175.1	actinobacteria
AQW52911.1	EACC1	TetR_N+TetR_C_6→ EACC1*→ CASPASE+low-complexity+TM+PBPB→	129	Streptomyces hygroscopicus	GCA_002021875.1	actinobacteria
AQY20485.1	EACC1	EACC1*→	87	Streptomyces lydicus	GCA_000952035.2	actinobacteria
ASQ93024.1	EACC1	Pkinase+TPRs→ EACC1*→ CASPASE+TPRs→	141	Streptomyces sp. 11-1-2	GCA_003595545.1	actinobacteria
ASQ93155.1	EACC1	EACC1*→ CASPASE+STAND+polar_region+TM+BetaPropeller→	147	Streptomyces sp. 11-1-2	GCA_003595545.1	actinobacteria
ATL84945.1	EACC1	TetR_N+TetR_C_6→ EACC1*→ CASPASE+low-complexity+TM+PBPB→	131	Streptomyces malaysiensis	GCA_002591335.1	actinobacteria
ATL88100.1	EACC1	EACC1*→ CASPASE+HisKA+HATPase_c→	164	Streptomyces malaysiensis	GCA_002591335.1	actinobacteria
AUA11757.1	EACC1	TetR_N+TetR_C_6→ EACC1*→ CASPASE+low-complexity+TM+PBPB→	141	Streptomyces sp. M56	GCA_002812405.1	actinobacteria
AUG75451.1	EACC1	EACC1*→ CASPASE+STAND+polar_region+TM+BetaPropeller→	125	Kitasatospora sp. MMS16-BH015	GCA_002943525.1	actinobacteria
AUG81298.1	EACC1	EACC1*→	115	Kitasatospora sp. MMS16-BH015	GCA_002943525.1	actinobacteria
AUI64191.1	EACC1	EACC1*→ TM+ZU5+??→	67	Amycolatopsis sp. BJA-103	GCA_002849735.1	actinobacteria
BAL90824.1	EACC1	EACC1*→ CASPASE+TPRs→	118	Actinoplanes missouriensis 431	GCA_000284295.1	actinobacteria
CAJ62793.1	EACC1+EAD1	FHA+??+low-complexity+FHA+ABC-ATPase+TM→||←?||EACC1+EAD1*→||←CASPASE+STAND+polar_region+TM+BetaPropeller	233	Frankia alni ACN14a	GCA_000058485.1	actinobacteria
CCF64831.1	EACC1	RNR_N+Ribonuc_red_lgN+Ribonuc_red_lgC→ EACC1*→ CASPASE+FTSK+FTSK+FTSK+FTSK→	118	Nocardia cyriacigeorgica GUH-2	GCA_000284035.1	actinobacteria

acc	architecture	operon	len	species	gca	taxend
CDR06555.1	EACC1	EACC1*→	113	Streptomyces iranensis	GCA_000938975.1	actinobacteria
CEL16981.1	EACC1	PALP→ Trp_syntA→ TM+LGT→ EACC1*→	132	Kibdelosporangium sp. MJ126-NF4	GCA_000826545.1	actinobacteria
CUW28376.1	EACC1	??→??→ EACC1*→ CASPASE+??→	135	Streptomyces reticuli	GCA_001511815.1	actinobacteria
CUW29406.1	EACC1	EACC1*→ CASPASE+TM?+DUF3558+DUF3558→	145	Streptomyces reticuli	GCA_001511815.1	actinobacteria
CUW32072.1	EACC1	AMP-binding+AMP-binding_C→ AMP-binding+AMP-binding_C→ EACC1*→ CASPASE+STAND+polar_region+TM+BetaPropeller→	156	Streptomyces reticuli	GCA_001511815.1	actinobacteria
EDN65281.1	EACC1	EACC1*→	131	Beggiatoa sp. PS	GCA_000170715.1	gammaproteobacteria
EDY66027.1	EACC1	low-complexity+HD+Tox-RelA+TGS+ACT→ Pkinase+TPRs→ EACC1*→ CASPASE+TPRs→	125	Streptomyces pristinaespiralis ATCC 25486	GCA_000154945.1	actinobacteria
EEP70832.1	EACC1	TM+TM+TM+Peptidase_M48+TM+DUF4864+??→ EACC1*→	191	Micromonospora sp. ATCC 39149	GCA_000158815.1	actinobacteria
EFE74311.1	EACC1	EACC1*→ CASPASE+Beta_rich_repeat→	138	Streptomyces roseosporus NRRL 15998	GCA_000156455.1	actinobacteria
EFF88992.1	EACC1	TPRs→ EACC1*→ CASPASE+TPRs→	121	Streptomyces sp. e14	GCA_000162775.1	actinobacteria
EFL23145.1	EACC1	EACC1*→ CASPASE+TPRs→ Clp_N+Clp_N+Clp_N+Clp_N+ClpABN-AAA→	138	Streptomyces himastatinicus ATCC 53653	GCA_000158915.1	actinobacteria
EFL27052.1	EACC1	HATPase_c_2→ EACC1*→	131	Streptomyces himastatinicus ATCC 53653	GCA_000158915.1	actinobacteria
EFL39449.1	EACC1	MglB→?→ sGTP-MglA→?→ Beta_rich?→ EACC1*→ CASPASE+TPRs→??→??→	133	Streptomyces griseoflavus Tu4000	GCA_000158975.1	actinobacteria
EHM26604.1	EACC1	EACC1*→ CASPASE+PBPB→	116	Streptomyces sp. W007	GCA_000239075.2	actinobacteria
EHR51770.1	EACC1	EACC1*→ CASPASE+TM+TM+TM+TM→	121	Saccharomonospora marina XMU15	GCA_000244955.1	actinobacteria
EMF56432.1	EACC1	EACC1*→ CASPASE+TPRs→	109	Streptomyces bottropensis ATCC 25435	GCA_000340335.2	actinobacteria
EOD64476.1	EACC1	PBPB→?→?→ Aminotran_5+PAS_6+HTH_22→ EACC1*→ CASPASE+??+TM+TM→||←Glyco_trans_4_4+UDPGT	135	Amycolatopsis vancoresmycina DSM 44592	GCA_000388135.1	actinobacteria
EPH45012.1	EACC1	EACC1*→	141	Streptomyces aurantiacus JA 4570	GCA_000414115.1	actinobacteria
EST24898.1	EACC1	EACC1*→ CASPASE+Beta_rich_repeat→	128	Streptomyces niveus NCIMB 11891	GCA_000497425.1	actinobacteria
EST29781.1	EACC1	EACC1*→ CASPASE+BetaPropeller→||←?←?||HATPase_c_2→	139	Streptomyces roseochromogenus subsp. oscitans DS 12.976	GCA_000497445.1	actinobacteria
ESU49840.1	EACC1	EACC1*→ CASPASE+Beta_rich_repeat→	131	Streptomyces sp. HCCB10043	GCA_000498935.1	actinobacteria
EWC63377.1	EACC1	EACC1*→ CASPASE+??+ART→	111	Actinokineospora spheciospongiae	GCA_000564855.1	actinobacteria
EYT80103.1	EACC1	EACC1*→	114	Streptomyces sp. Tu 6176	GCA_000611795.1	actinobacteria
EYT80250.1	EACC1	EACC1*→	134	Streptomyces sp. Tu 6176	GCA_000611795.1	actinobacteria
GAT70183.1	EACC1	EACC1*→ CASPASE+SAD+HNH→	116	Planomonospora sphaerica	GCA_001653075.1	actinobacteria
GAT71384.1	EACC1	EACC1*→ CASPASE+STAND+polar_region+TM+BetaPropeller→	123	Planomonospora sphaerica	GCA_001653075.1	actinobacteria
GAT81834.1	EACC1	EACC1*→ CASPASE+??→	114	Streptomyces sp. F-3	GCA_001653115.1	actinobacteria
KDN73846.1	EACC1	EACC1*→ CASPASE+TM?+BetaPropeller→	153	Streptomyces olindensis	GCA_000696115.1	actinobacteria
KDN73958.1	EACC1	EACC1*→ CASPASE+STAND+polar_region+TM+BetaPropeller→	171	Streptomyces olindensis	GCA_000696115.1	actinobacteria
KDQ68047.1	EACC1	EACC1*→ CASPASE+STAND+PBPB→	138	Streptomyces sp. NTK 937	GCA_000698495.1	actinobacteria
KEF14169.1	EACC1	EACC1*→ CASPASE+??+SF1-helicase+Vsr(PDDEXK)?→ REase+STAND→	138	Streptomyces rimosus R6-500	GCA_000707925.1	actinobacteria
KIA61331.1	EACC1	DUF2510(W3)+BicD+DUF4041+T5orf172→ EACC1*→	122	Nocardia vulneris	GCA_000811985.1	actinobacteria
KIF00454.1	EACC1	EACC1*→ CASPASE+STAND(fragment)→ AAA(fragment-helical-region)+wHTH+TM+BetaPropeller→	135	Streptomyces sp. RSD-27	GCA_000817655.1	actinobacteria
KIX79887.1	EACC1	EACC1*→ CASPASE+STAND+TM+polar_region+TM+BetaPropeller→	80	Streptomyces sp. MBRL 601	GCA_000932205.1	actinobacteria
KJY28680.1	EACC1	FAD_binding_4+FAD-oxidase_C→ DAGK_cat+DAGK_acc→ EACC1*→	132	Streptomyces sp. NRRL S-495	GCA_000966955.1	actinobacteria
KJY43406.1	EACC1	EACC1*→ CASPASE+STAND+polar_region+TM+BetaPropeller→	133	Streptomyces sp. NRRL B-1568	GCA_000967025.1	actinobacteria
KMS82330.1	EACC1	EACC1*→	137	Streptomyces regensis	GCA_001047335.1	actinobacteria
KND30816.1	EACC1	EACC1*→ CASPASE+TM+TM+PBPB+vWA→	116	Streptomyces acidiscabies	GCA_001189015.1	actinobacteria
KND37468.1	EACC1	EACC1*→ CASPASE+Beta_rich_repeat→??+SUKH→	143	Streptomyces europaeiscabiei	GCA_001189025.1	actinobacteria
KOU85740.1	EACC1	EACC1*→ CASPASE+??+SF1-helicase+Vsr(PDDEXK)?→	120	Streptomyces sp. XY593	GCA_001279885.1	actinobacteria
KOV11283.1	EACC1	EACC1*→	110	Streptomyces sp. XY431	GCA_001279805.1	actinobacteria
KOV50628.1	EACC1	EACC1*→	104	Streptomyces sp. AS58	GCA_001279985.1	actinobacteria
KOV61478.1	EACC1	EACC1*→	121	Streptomyces sp. NRRL WC-3618	GCA_001280005.1	actinobacteria
KOV91281.1	EACC1	EACC1*→	145	Streptomyces sp. NRRL B-3648	GCA_001279545.1	actinobacteria
KPH99478.1	EACC1	EACC1*→ CASPASE+TPRs→	130	Actinobacteria bacterium OK006	GCA_001298565.1	actinobacteria

acc	architecture	operon	len	species	gca	taxend
KPI03761.1	EACC1	EACC1*→ CASPASE+STAND+polar_region+TM+BetaPropeller→	186	Actinobacteria bacterium OK006	GCA_001298565.1	actinobacteria
KPI06058.1	EACC1	EACC1*→ CASPASE+STAND+polar_region+TM+BetaPropeller→	147	Actinobacteria bacterium OK074	GCA_001298575.1	actinobacteria
KPI29333.1	EACC1	EACC1*→ CASPASE+low-complexity+BetaPropeller→	115	Actinobacteria bacterium OK074	GCA_001298575.1	actinobacteria
KQU66405.1	EACC1	EACC1*→ CASPASE+??+TM+TM+TM+TM+TM+TM+TM+TM+TM+TM+TM→	135	Phycicoccus sp. Root101	GCA_001426245.1	actinobacteria
KQX58974.1	EACC1	EACC1*→ CASPASE+STAND+polar_region+TM+BetaPropeller→||←MFS_1	174	Streptomyces sp. Root1304	GCA_001426405.1	actinobacteria
KSU68257.1	EACC1	EACC1*→ CASPASE+??→	128	Rhodococcus enclensis	GCA_001456965.1	actinobacteria
KUF13397.1	EACC1	EACC1*→ CASPASE+Type_III_YscG+VHL→	82	Streptomyces silvensis	GCA_001482415.1	actinobacteria
KUJ54561.1	EACC1	EACC1*→ CASPASE+TPRs→	121	Streptomyces albus subsp. albus	GCA_001507455.1	actinobacteria
KUJ66425.1	EACC1	EACC1*→ CASPASE+??→	137	Streptomyces albus subsp. albus	GCA_001507455.1	actinobacteria
KUJ70077.1	EACC1	EACC1*→ CASPASE+PBPB+vWA→	133	Streptomyces albus subsp. albus	GCA_001507455.1	actinobacteria
KUL21378.1	EACC1	EACC1*→ CASPASE+STAND+polar_region+TM+BetaPropeller→	114	Streptomyces regalis	GCA_001509475.1	actinobacteria
KUL31772.1	EACC1	EACC1*→ CASPASE+??→	105	Actinoplanes awajinensis subsp. mycoplanecinus	GCA_001509495.1	actinobacteria
KUM90168.1	EACC1	EACC1*→ CASPASE+low-complexity+BetaPropeller→	141	Streptomyces cellostaticus	GCA_001513965.1	actinobacteria
KWT62307.1	EACC1	EACC1*→	210	Streptomyces albus subsp. albus	GCA_001541145.1	actinobacteria
KZB85498.1	EACC1	EACC1*→	67	Amycolatopsis regifaucium	GCA_001613935.1	actinobacteria
OBG60369.1	EACC1	EACC1*→ TM+TM→	96	Mycobacterium sp. E188	GCA_001666935.1	actinobacteria
OCC10925.1	EACC1	EACC1*→ CASPASE+TPRs→	87	Streptomyces sp. PTY087I2	GCA_001687325.1	actinobacteria
OEJ28906.1	EACC1	EACC1*→ CASPASE+BetaPropeller→	149	Streptomyces agglomeratus	GCA_001746415.1	actinobacteria
OEV32520.1	EACC1	EACC1*→	127	Kitasatospora aureofaciens	GCA_001188955.3	actinobacteria
OIJ66728.1	EACC1	Pkinase+TPRs→ EACC1*→ CASPASE+TPRs→	124	Streptomyces mangrovisoli	GCA_000974985.2	actinobacteria
OIJ93039.1	EACC1	EACC1*→ CASPASE+STAND+polar_region+TM+BetaPropeller→	145	Streptomyces sp. MUSC 14	GCA_001866675.1	actinobacteria
OIK01659.1	EACC1	EACC1*→ CASPASE+STAND+polar_region+TM+BetaPropeller→	131	Streptomyces sp. MUSC 1	GCA_001866665.1	actinobacteria
OIK08128.1	EACC1	EACC1*→	123	Streptomyces sp. MUSC 1	GCA_001866665.1	actinobacteria
OKJ53082.1	EACC1	EACC1*→ CASPASE+Beta_rich_repeat→??+SUKH→	143	Streptomyces sp. CB02115	GCA_001905645.1	actinobacteria
OKJ90725.1	EACC1	EACC1*→	134	Streptomyces sp. TSRI0107	GCA_001905845.1	actinobacteria
OKK02612.1	EACC1	EACC1*→	119	Streptomyces sp. CB03234	GCA_001905885.1	actinobacteria
OKK04987.1	EACC1	FAD_binding_4+??+BBE→ EACC1*→ CASPASE+STAND+polar_region+TM+BetaPropeller→	123	Streptomyces sp. CB03234	GCA_001905885.1	actinobacteria
OKK25374.1	EACC1	MglB→ DUF742→ sGTP-MglA→?→?→ EACC1*→	114	Streptomyces sp. CB02488	GCA_001905365.1	actinobacteria
OLB75938.1	EACC1	EACC1*→	123	Actinobacteria bacterium 13_2_20CM_2_71_6	GCA_001917385.1	actinobacteria
OLB81125.1	EACC1	EACC1*→ CASPASE+FTSK+FTSK+FTSK+FTSK→	127	Actinobacteria bacterium 13_2_20CM_2_71_6	GCA_001917385.1	actinobacteria
OLE22616.1	EACC1+CASPASE+TPR_repeats+low_complexity+TM_region	EACC1+CASPASE+TPR_repeats+low_complexity+TM_region*→	748	Actinobacteria bacterium 13_1_20CM_3_71_11	GCA_001920275.1	actinobacteria
OLE22806.1	EACC1	EACC1*→ CASPASE+PBPB→	123	Catenulispora sp. 13_1_20CM_3_70_7	GCA_001920315.1	actinobacteria
OLE29346.1	EACC1	EACC1*→ CASPASE+DUF179→ SIR2+??→	112	Actinobacteria bacterium 13_1_20CM_3_71_11	GCA_001920275.1	actinobacteria
OLT26847.1	EACC1	EACC1*→	130	Nocardiopsis sp. CNR-923	GCA_001942255.1	actinobacteria
ONK14370.1	EACC1	EACC1*→ CASPASE+TPRs→	154	Streptomyces sp. MP131-18	GCA_001984575.1	actinobacteria
OON81606.1	EACC1	EACC1*→ CASPASE+??+SF1-helicase+Vsr(PDDEXK)?→	138	Streptomyces tsukubensis	GCA_002007125.1	actinobacteria
OPF83920.1	EACC1	EACC1*→ CASPASE+Peripla_BP_5→	122	Streptomyces antioxidans	GCA_000968685.2	actinobacteria
OPG04755.1	EACC1	EACC1*→	114	Streptomyces sp. GKU 895	GCA_002027195.1	actinobacteria
OSC72785.1	EACC1	low-complexity+HD+Tox-RelA+TGS+ACT→ Pkinase+TPRs→ EACC1*→ CASPASE+TPRs→	124	Streptomyces sp. 4F	GCA_002104855.1	actinobacteria
OSY45817.1	EACC1	EACC1*→ CASPASE+Beta_rich_repeat→	133	Streptomyces platensis	GCA_002119195.1	actinobacteria
OXL29487.1	EACC1	low-complexity+HD+Tox-RelA+TGS+ACT→ Pkinase+TPRs→ EACC1*→ CASPASE+TPRs→	131	Streptomyces sp. NBS 14/10	GCA_002224125.1	actinobacteria
OXY99187.1	EACC1	EACC1*→ CASPASE+low-complexity+TM+TM+TM+TM+TM+TM+TM+TM+TM+TM+BetaPropeller→	148	Streptomyces diastatochromogenes	GCA_002242805.1	actinobacteria
OXY99708.1	EACC1	EACC1*→ CASPASE+STAND+polar_region+TM+BetaPropeller→ TM+Acid_phosphat_B→	137	Streptomyces diastatochromogenes	GCA_002242805.1	actinobacteria
PAK26487.1	EACC1	low-complexity+HD+Tox-RelA+TGS+ACT→ Pkinase+TPRs→ EACC1*→ CASPASE+TPRs→	138	Streptomyces sp. alain-838	GCA_002276245.1	actinobacteria
PBC97306.1	EACC1	EACC1*→ CASPASE+BetaPropeller→	205	Streptomyces sp. Ag82_O1-15	GCA_002300485.1	actinobacteria
PFG48271.1	EACC1	GATase→ ABC-ATPase+DUF4162→ cNMP_cyclase+TM+TM+TM+TM+TM+TM→ EACC1*→	138	Amycolatopsis sulphurea	GCA_002564045.1	actinobacteria

acc	architecture	operon	len	species	gca	taxend
PIF83911.1	EACC1	EACC1*→	126	Micromonospora sp. CNZ299	GCA_002754415.1	actinobacteria
PIF88722.1	EACC1	Phage_int_SAM_3+Phage_integrase→ EACC1*→ CASPASE+TPRs→	121	Streptomyces sp. 2233.5	GCA_002754455.1	actinobacteria
PIG15861.1	EACC1	EACC1*→ CASPASE+TM+??→	125	Streptomyces sp. 94	GCA_002754555.1	actinobacteria
PIG17351.1	EACC1	EACC1*→ CASPASE+TPRs+LysM+PG-binding→	120	Streptomyces sp. 94	GCA_002754555.1	actinobacteria
PIG38694.1	EACC1	EACC1*→ CASPASE+STAND+polar_region+TM+BetaPropeller→	147	Streptomyces sp. 93	GCA_002754595.1	actinobacteria
PIG46972.1	EACC1	low-complexity+HD+Tox-RelA+TGS+ACT→ Pkinase+TPRs→ EACC1*→ CASPASE+TPRs→	138	Streptomyces sp. 61	GCA_002754535.1	actinobacteria
PIY08225.1	EACC1	EACC1*→ CASPASE+??→	123	Flexibacter sp. CG_4_10_14_3_um_filter_32_15	GCA_002784125.1	bacteroidetes
PKB35290.1	EACC1	EACC1*→ CASPASE+STAND→ TM+BetaPropeller→ TM+Acid_phosphat_B→	148	Streptomyces sp. 69	GCA_002813405.1	actinobacteria
PKB39967.1	EACC1	EACC1*→ CASPASE+STAND+TPRs→	129	Streptomyces sp. 69	GCA_002813405.1	actinobacteria
PKK11963.1	EACC1	EACC1*→ CASPASE+??+NUDIX→	140	Thermomonospora sp. CIF 1	GCA_001896795.2	actinobacteria
PKT68436.1	EACC1	EACC1*→ CASPASE(fragment)→	120	Streptomyces populi	GCA_002911015.1	actinobacteria
PKV35908.1	EACC1	EACC1*→?→ CASPASE+STAND+polar_region+TM+BetaPropeller→ TM+Acid_phosphat_B→	137	Streptomyces sp. 59	GCA_002846295.1	actinobacteria
PKV38657.1	EACC1	EACC1*→ CASPASE+BetaPropeller→	140	Streptomyces sp. 59	GCA_002846295.1	actinobacteria
PKV55963.1	EACC1	EACC1*→ CASPASE+STAND+polar_region+TM+BetaPropeller→	138	Streptomyces sp. 73	GCA_002846315.1	actinobacteria
PKV70128.1	EACC1	EACC1*→ CASPASE+??+STAND+??→	144	Streptomyces sp. GP55	GCA_002846355.1	actinobacteria
PKV72783.1	EACC1	polar_lead?+TM+TM+TM+polar_tail?+low-complexity→??→ EACC1*→ CASPASE+TPRs→	122	Streptomyces sp. GP55	GCA_002846355.1	actinobacteria
PKV83071.1	EACC1	EACC1*→ CASPASE+low-complexity+BetaPropeller→	157	Streptomyces sp. TLI_146	GCA_002846415.1	actinobacteria
PKV88670.1	EACC1	EACC1*→	157	Streptomyces sp. TLI_146	GCA_002846415.1	actinobacteria
PKW40573.1	EACC1	EACC1*→ CASPASE+TPRs→ Pkinase+??→	141	Streptomyces sp. OK885	GCA_002846625.1	actinobacteria
PLK97651.1	EACC1	EACC1*→	93	Klebsiella pneumoniae	GCA_002855875.1	gammaproteobacteria
POG43333.1	EACC1	Pkinase+TPRs→ EACC1*→ CASPASE+TPRs→	132	Streptomyces sp. ZL-24	GCA_002907485.1	actinobacteria
POX40554.1	EACC1	EACC1*→	90	Streptomyces sp. Ru73	GCA_002920575.1	actinobacteria
POX56155.1	EACC1	EACC1*→ CASPASE+TPRs→ Clp_N+Clp_N+ClpABN-AAA→	59	Streptomyces sp. Ru71	GCA_002920535.1	actinobacteria
PPK63807.1	EACC1	EACC1*→ CASPASE+TM+TM→ TM+PBPB→	117	Actinokineospora auranticolor	GCA_002934265.1	actinobacteria
PPQ61001.1	EACC1	low-complexity+HD+Tox-RelA+TGS+ACT→ Pkinase+TPRs→ EACC1*→ CASPASE+TPRs→	136	Streptomyces sp. QL37	GCA_002941025.1	actinobacteria
PRX04721.1	EACC1+CASPASE+TPR_repeats+low_complexity+TM_region	EACC1+CASPASE+TPR_repeats+low_complexity+TM_region*→ STAND+TPRs→	819	Actinoplanes italicus	GCA_003001815.1	actinobacteria
PRX21968.1	EACC1	Glyco_trans_4_4+UDPGT→ EACC1*→ CASPASE+PBPB+vWA→ TM+PBPB→	141	Actinoplanes italicus	GCA_003001815.1	actinobacteria
PRX63748.1	EACC1	EACC1*→ CASPASE+PBPB→	130	Nonomuraea fuscirosea	GCA_003001935.1	actinobacteria
PRY54979.1	EACC1	EACC1*→ CASPASE+TPRs→	112	Glycomyces artemisiae	GCA_003002955.1	actinobacteria
PTH84465.1	EACC1	EACC1*→ CASPASE+??→	123	Streptomyces sp. A244	GCA_003041235.1	actinobacteria
PWK45066.1	EACC1	EACC1*→ CASPASE+TM+PASTA+PASTA+PASTA→	128	Actinoplanes xinjiangensis	GCA_003148685.1	actinobacteria
PWV79680.1	EACC1	EACC1*→ CASPASE+TPRs→	125	Nocardia neocaledoniensis	GCA_003182135.1	actinobacteria
SAP16349.1	EACC1	EACC1*→ CASPASE→?+BetaPropeller→	119	Nonomuraea gerenzanensis	-	actinobacteria
SBO93207.1	EACC1	ARSR-HTH+RHOD-CDC25→??→??→ EACC1*→	132	Nonomuraea gerenzanensis	-	actinobacteria
SBW28797.1	EACC1	EACC1*→ CASPASE+SF1-helicase→	126	Candidatus Frankia californiensis	GCA_900067225.1	actinobacteria
SCD30855.1	EACC1	EACC1*→ CASPASE+??→	122	Streptomyces sp. DvalAA-14	GCA_900091855.1	actinobacteria
SCD65879.1	low_complexity+EACC1	low_complexity+EACC1*→ CASPASE+GAF+PAS→	245	Streptomyces sp. Termitarium-T10T-6	GCA_900091795.1	actinobacteria
SCD80496.1	EACC1	EACC1*→ CASPASE+STAND+PBPB→	88	Streptomyces sp. PpalLS-921	GCA_900091835.1	actinobacteria
SCF06083.1	EACC1	TM+TM+TM+Peptidase_M48+TM+DUF4864+??→ EACC1*→	153	Micromonospora carbonacea	GCA_900091535.1	actinobacteria
SCF09290.1	EACC1	EACC1*→	123	Micromonospora chokoriensis	GCA_900091505.1	actinobacteria
SCF63949.1	EACC1	EACC1*→	134	Streptomyces sp. Ncost-T6T-2b	GCA_900091965.1	actinobacteria
SCF67305.1	EACC1	TetR_N+TetR_C_6→ EACC1*→ CASPASE+TPRs→	139	Streptomyces sp. MnatMP-M27	GCA_900092015.1	actinobacteria
SCF81128.1	EACC1	EACC1*→ CASPASE+STAND+polar_region+TM+BetaPropeller→	133	Streptomyces sp. Ncost-T10-10d	GCA_900091955.1	actinobacteria
SCK28094.1	EACC1	EACC1*→ CASPASE+Beta_rich_repeat→	163	Streptomyces sp. SceaMP-e96	GCA_900090095.1	actinobacteria
SCK31176.1	EACC1	EACC1*→ TM+TM→	73	Streptomyces sp. SceaMP-e96	GCA_900090095.1	actinobacteria
SCK35005.1	EACC1	EACC1*→ CASPASE+TPRs→	166	Streptomyces sp. AmelKG-E11A	GCA_900090165.1	actinobacteria
SDC39793.1	EACC1+CASPASE(frag)	CASPASE←?←?||HATPase_c_2→ EACC1+CASPASE(frag)*→	235	Streptomyces prasinopilosus	GCA_900101585.1	actinobacteria
SDE31791.1	EACC1	EACC1*→ CASPASE+TPRs→	112	Glycomyces harbinensis	GCA_900101745.1	actinobacteria
SDJ48636.1	EACC1	EACC1*→ CASPASE+low-complexity+DUF4190(has2TM)→	129	Frankineae bacterium MT45	GCA_900100325.1	actinobacteria

acc	architecture	operon	len	species	gca	taxend
SDN31180.1	EACC1	EACC1*→ CASPASE+TPRs→	118	Streptomyces sp. cf386	GCA_900103585.1	actinobacteria
SDQ56450.1	EACC1	EACC1*→ CASPASE+Astro_capsid_N→	119	Thermostaphylospora chromogena	GCA_900099985.1	actinobacteria
SDT41267.1	EACC1	FAD_binding_4+FAD-oxidase_C→ DAGK_cat+DAGK_acc→ EACC1*→ CASPASE+TPRs→ Clp_N+Clp_N+ClpABN-AAA→	141	Streptomyces sp. TLI_053	GCA_900105395.1	actinobacteria
SDW24590.1	EACC1	??+TM+TM+??+TM→ PBPB→ EACC1*→ CASPASE+TPRs→ PknG_rubred+Pkinase+PknG_TPR→ ZnR+PP2C→ vWA+alpha-helical→	113	Amycolatopsis xylanica	GCA_900107045.1	actinobacteria
SDW32577.1	EACC1	EACC1*→ CASPASE+STAND+polar_region+TM+BetaPropeller→	166	Amycolatopsis xylanica	GCA_900107045.1	actinobacteria
SDY41344.1	EACC1	EACC1*→ CASPASE+STAND+polar_region+TM+BetaPropeller→	127	Saccharopolyspora shandongensis	GCA_900106995.1	actinobacteria
SEC47799.1	EACC1	TetR_N+TetR_C_6→ EACC1*→ CASPASE+low-complexity+TM+PBPB→	130	Streptomyces melanosporofaciens	GCA_900105695.1	actinobacteria
SED09207.1	EACC1	PBPB→?→?→? EACC1*→ CASPASE+TM+TM→	130	Amycolatopsis tolypomycina	GCA_900105945.1	actinobacteria
SED41685.1	EACC1	EACC1*→ CASPASE+STAND+polar_region+TM+BetaPropeller→	133	Streptomyces melanosporofaciens	GCA_900105695.1	actinobacteria
SEF56362.1	EACC1	ARSR-HTH+RHOD-CDC25→??→??→ EACC1*→	127	Nonomuraea solani	GCA_900108335.1	actinobacteria
SEG70419.1	EACC1	EACC1*→ CASPASE+BetaPropeller→ BetaPropeller(fragment)→	121	Saccharopolyspora hirsuta	GCA_900108315.1	actinobacteria
SEG73472.1	EACC1	low-complexity+HD+Tox-RelA+TGS+ACT→ Pkinase+TPRs→ EACC1*→ CASPASE+TPRs→	206	Streptomyces yanglinensis	GCA_900107965.1	actinobacteria
SEH02685.1	EACC1	EACC1*→ CASPASE+BetaPropeller→	75	Nonomuraea solani	GCA_900108335.1	actinobacteria
SEJ62319.1	EACC1	EACC1*→ CASPASE+Pkinase→	112	Xiangella phaseoli	GCA_900109115.1	actinobacteria
SES39042.1	EACC1	EACC1*→ CASPASE+??+TM+TM+TM+TM+TM+TM+TM+TM→ CASPASE+??+TM+TM+TM+TM+TM+TM+TM+TM→	123	Actinokineospora terrae	GCA_900111175.1	actinobacteria
SEU35263.1	EACC1	EACC1*→ CASPASE+STAND+polar_region+TM+BetaPropeller→	103	Nonomuraea wenchangensis	GCA_900111685.1	actinobacteria
SEU43169.1	EACC1	TM+TM+vWA+TM→ TM+TM+vWA+TM→ EACC1*→ CASPASE+TPRs→	115	Nonomuraea wenchangensis	GCA_900111685.1	actinobacteria
SFF46366.1	EACC1	EACC1*→	155	Streptomyces alni	GCA_900112845.1	actinobacteria
SFQ15972.1	EACC1	EACC1*→ CASPASE+??+SF1-helicase+Vsr(PDDEXK)?→ vWA→	124	Amycolatopsis rubida	GCA_900115345.1	actinobacteria
SFQ20437.1	EACC1	EACC1*→ CASPASE+??+TM+TM+TM→	126	Actinomadura madurae	GCA_900115095.1	actinobacteria
SFW52991.1	EACC1	MFS_1→||←?||GATase→ ABC-ATPase+DUF4162→ cNMP_cyclase+TM+TM+TM+TM+TM+TM→ EACC1*→	118	Amycolatopsis australiensis	GCA_900119165.1	actinobacteria
SFY01652.1	EACC1	EACC1*→	139	Streptomyces atratus	GCA_900119365.1	actinobacteria
SHM52898.1	EACC1	EACC1*→ CASPASE+TM+MFS_1+TM+TM+TM+TM+TM+TM+TM+TM→	125	Streptomyces yunnanensis	GCA_900142595.1	actinobacteria
SHM78180.1	EACC1	EACC1*→ CASPASE+STAND+polar_region+TM+BetaPropeller→	152	Streptomyces paucisporeus	GCA_900142575.1	actinobacteria
SIM83978.1	EACC1	EACC1*→	131	Micromonospora cremea	GCA_900143515.1	actinobacteria
SNB88651.1	EACC1	EACC1*→	74	Streptomyces sp. PgraA7	GCA_900187925.1	actinobacteria
SNR23901.1	EACC1	EACC1*→ CASPASE+TM+TM→	85	Actinomadura mexicana	GCA_900188105.1	actinobacteria
SNR32426.1	EACC1	EACC1*→ CASPASE+TM+TM+TM+TM+TM+TM+TM+TM→ Aminotran_5→	124	Blastococcus sp. DSM 44272	GCA_900188025.1	actinobacteria
SNR65625.1	EACC1	EACC1*→ CASPASE+TPRs→	114	Actinoplanes regularis	GCA_900188005.1	actinobacteria
SNS54572.1	EACC1	EACC1*→ CASPASE+??+TM+TM→	132	Geodermatophilus saharensis	GCA_900188205.1	actinobacteria
SNS82844.1	EACC1	EACC1*→ CASPASE+??+TM+TM+TM→	130	Actinomadura mexicana	GCA_900188105.1	actinobacteria
SNT19761.1	EACC1	EACC1*→	75	Streptosporangium subroseum	GCA_900188345.1	actinobacteria
SOD66767.1	EACC1	EACC1*→ CASPASE+JAB→	163	Streptomyces zhaozhouensis	GCA_900230195.1	actinobacteria
SOD71875.1	EACC1	EACC1*→ CASPASE+low-complexity+DUF4190(has2TM)→?→ Glyoxalase→	129	Jatrophihabitans sp. GAS493	GCA_900230215.1	actinobacteria
SOD87477.1	EACC1	EACC1*→ CASPASE+STAND+polar_region+TM+BetaPropeller→ TM+Acid_phosphat_B→	137	Streptomyces sp. Ag109_G2-15	GCA_900215565.1	actinobacteria
SOE74808.1	EACC1	EACC1*→ CASPASE+TM+PBPI→	140	Streptomyces sp. OV198	GCA_900230275.1	actinobacteria
SOE81020.1	EACC1	EACC1*→ Tnp_DNA_bind+DDE_Tnp_1+Dimer_Tnp_Tn5→ CASPASE+TPRs→	131	Burkholderia sp. OK806	GCA_900230245.1	betaproteobacteria
SOR77037.1	EACC1	EACC1*→ CASPASE+STAND+polar_region+TM+BetaPropeller→	175	Streptomyces chartreusis NRRL 3882	GCA_900236475.1	actinobacteria
WP_003079032.1	EACC1	GATase→ ABC-ATPase+DUF4162→ cNMP_cyclase+TM+TM+TM+TM+TM+TM→ EACC1*→	104	Amycolatopsis vancoresmycina	GCA_000388135.1	actinobacteria
WP_003962152.1	EACC1	EACC1*→ CASPASE+??+SF1-helicase+Vsr(PDDEXK)?→	141	Streptomyces clavuligerus	GCA_001693675.1	actinobacteria
WP_003966577.1	EACC1	Beta_rich?→ EACC1*→ CASPASE+TPRs→??→??→	133	Streptomyces sp. ACT-1	GCA_000177175.2	actinobacteria
WP_003971296.1	EACC1	EACC1*→ CASPASE+TPRs→	118	Streptomyces	GCA_000177175.2	actinobacteria
WP_003979897.1	EACC1	EACC1*→ CASPASE+TM?+BetaPropeller→	174	Streptomycetaceae	GCA_000331185.1	actinobacteria
WP_004559361.1	EACC1	EACC1*→ CASPASE+Cpn60_TCP1→	112	Amycolatopsis vancoresmycina	GCA_000388135.1	actinobacteria
WP_004945484.1	EACC1	EACC1*→	106	Streptomyces mobaraensis	GCA_000342125.1	actinobacteria
WP_005165699.1	EACC1	CASPASE+TPRs→?→ EACC1*→	131	Amycolatopsis azurea	GCA_000340415.1	actinobacteria
WP_005165783.1	EACC1	EACC1*→ CASPASE+TPRs→	110	Amycolatopsis azurea	GCA_000340415.1	actinobacteria
WP_006134566.1	EACC1	TM+TM+TM+PP2C→??→ EACC1*→ CASPASE+TM+TM+PBPB+vWA→	114	Streptomyces	GCA_000342345.1	actinobacteria
WP_006378561.1	EACC1	EACC1*→ CASPASE+TM+TM+bPH_domain+TM+TM+bPH_domain+TM+TM→	128	Streptomyces turgidiscabies	GCA_000331005.1	actinobacteria
WP_007024301.1	EACC1	CASPASE+??→ EACC1*→	129	Saccharomonospora paurometabolica	GCF_000231035.2	actinobacteria
WP_007032113.1	EACC1	MFS_1→?→||←?||EACC1*→	104	Amycolatopsis decaplanina	GCA_000342005.1	actinobacteria

acc	architecture	operon	len	species	gca	taxend
WP_007385288.1	EACC1	EACC1*→ CASPASE+??+SF1-helicase+Vsr(PDDEXK)?→	129	Streptomyces sviceus	GCA_000154965.1	actinobacteria
WP_007461575.1	EACC1	EACC1*→	137	Micromonospora lupini	GCA_000297395.2	actinobacteria
WP_007498037.1	EACC1	HATPase_c_2→ EACC1*→ CASPASE+??+SF1-helicase+Vsr(PDDEXK)?→	130	Streptomyces zinciresistens	GCA_000225525.2	actinobacteria
WP_007905045.1	EACC1	EACC1*→ CASPASE+STAND+??→	114	Ktedonobacter racemifer	GCA_000178855.1	chloroflexi
WP_008736615.1	EACC1	EACC1*→ CASPASE+STAND+polar_region+TM+BetaPropeller→	154	Streptomyces sp. Mg1	GCA_000154885.1	actinobacteria
WP_009085569.1	EACC1	EACC1*→	108	Actinobacteria	GCA_002796545.1	actinobacteria
WP_009945795.1	EACC1	EACC1*→ CASPASE+??+SF1-helicase+Vsr(PDDEXK)?→	127	Saccharopolyspora erythraea	GCA_000448385.1	actinobacteria
WP_010033024.1	EACC1	EACC1*→ CASPASE+STAND+polar_region+TM+BetaPropeller→	173	Streptomyces chartreusis	GCF_000226455.1	actinobacteria
WP_010311167.1	EACC1	EACC1*→ CASPASE+BetaPropeller→	121	Saccharopolyspora spinosa	GCA_002846475.1	actinobacteria
WP_010354247.1	EACC1	EACC1*→ CASPASE+TM+TM+PBPB+vWA→	115	Streptomyces acidiscabies	GCA_001485105.1	actinobacteria
WP_010692477.1	EACC1	EACC1*→ CASPASE+??+SF1-helicase+Vsr(PDDEXK)?→	131	Saccharopolyspora spinosa	GCA_002846475.1	actinobacteria
WP_011031247.1	EACC1	-	114	Streptomyces coelicolor	GCF_000203835.1	actinobacteria
WP_012379217.1	EACC1	Beta_rich?→ EACC1*→ CASPASE+TPRs→??→??→	133	Streptomyces griseus	GCA_000010605.1	actinobacteria
WP_012894232.1	EACC1+??	EACC1+??*→	456	Streptosporangium roseum	GCA_000024865.1	actinobacteria
WP_013015649.1	EACC1	Clp_N+ClpABN-AAA+CASPASE→ EACC1*→	139	Stackebrandtia nassauensis	GCA_000024545.1	actinobacteria
WP_013016199.1	EACC1	EACC1*→	115	Stackebrandtia nassauensis	GCA_000024545.1	actinobacteria
WP_013153739.1	EACC1	EACC1*→ CASPASE+TM?+BetaPropeller→	126	Nocardiopsis dassonvillei	GCA_000092985.1	actinobacteria
WP_013222002.1	EACC1	GATase→ ABC-ATPase+DUF4162→ cNMP_cyclase+TM+TM+TM+TM+TM+TM→ EACC1*→	105	Amycolatopsis mediterranei	GCA_000196835.1	actinobacteria
WP_013223403.1	EACC1	PBPB→?→?→ Aminotran_5+PAS_6+HTH_22→ EACC1*→||←Glyco_trans_4_4+UDPGT	125	Amycolatopsis mediterranei	GCA_000196835.1	actinobacteria
WP_013227198.1	EACC1	EACC1*→ CASPASE+??→	123	Amycolatopsis mediterranei	GCA_000196835.1	actinobacteria
WP_013423591.1	EACC1	EACC1*→ CASPASE+TIR→	110	Frankia inefficax	GCA_000166135.1	actinobacteria
WP_014142088.1	EACC1	EACC1→ CASPASE+low-complexity+BetaPropeller___*_##→	140	Streptomyces cattleya	GCA_000240165.1	actinobacteria
WP_014144110.1	EACC1	EACC1*→ CASPASE+PBPB+vWA→	151	Streptomyces cattleya	GCA_000240165.1	actinobacteria
WP_014145359.1	EACC1	EACC1*→ CASPASE+??+SF1-helicase+Vsr(PDDEXK)?→	144	Streptomyces cattleya	GCA_000240165.1	actinobacteria
WP_014182282.1	EACC1	EACC1*→ CASPASE+TM+TM+??+TM+TM+??+TM+TM+??→	120	Streptomyces	GCA_000092385.1	actinobacteria
WP_014445973.1	EACC1	EACC1*→ Clp_N+Clp_N+ClpABN-AAA+CASPASE→	118	Actinoplanes missouriensis	GCA_000284295.1	actinobacteria
WP_014670064.1	EACC1	EACC1*→ CASPASE+low-complexity+TM+PBPB→	140	Streptomyces hygroscopicus	GCA_000245355.1	actinobacteria
WP_014673606.1	EACC1	EACC1*→ CASPASE+TM?+BetaPropeller→ MDMPI_N+MDMPI_C→ META→	116	Streptomyces hygroscopicus	GCA_000245355.1	actinobacteria
WP_014796470.1	EACC1	EACC1*→ CASPASE+??→	123	Bernardetia litoralis	GCA_000265505.1	bacteroidetes
WP_015073716.1	EACC1	EACC1*→ CASPASE+GAPEAD1→	113	Gluconobacter oxydans	GCA_000311765.1	alphaproteobacteria
WP_015099360.1	EACC1	TM+TM+TM→ CASPASE+STAND+polar_region+TM+BetaPropeller→ EACC1*→	127	Saccharothrix espanaensis	GCA_000328705.1	actinobacteria
WP_015102978.1	EACC1	EACC1*→ CASPASE+TM+TM→ TM+PBPB→	118	Saccharothrix espanaensis	GCA_000328705.1	actinobacteria
WP_015655483.1	EACC1	EAD2+VMAP-M0+CASPASE→ EACC1*→	134	Streptomyces davaonensis	GCA_000349325.1	actinobacteria
WP_015655816.1	EACC1	EACC1*→	110	Streptomyces davaonensis	GCA_000349325.1	actinobacteria
WP_015656507.1	EACC1	EACC1*→ CASPASE+TM+TM+bPH_domain+TM+TM+bPH_domain+TM+TM→	123	Streptomyces davaonensis	GCA_000349325.1	actinobacteria
WP_015662965.1	EACC1	EACC1*→ CASPASE+TM?+DUF3558+DUF3558→	120	Streptomyces davaonensis	GCA_000349325.1	actinobacteria
WP_015792459.1	EACC1	EACC1*→ CASPASE+low-complexity+BetaPropeller→ CASPASE+low-complexity+BetaPropeller→	115	Catenulispora acidiphila	GCA_000024025.1	actinobacteria
WP_015801011.1	EACC1	EACC1*→	121	Actinosynnema mirum	GCA_000023245.1	actinobacteria
WP_016332521.1	EACC1	EACC1*→	104	Amycolatopsis orientalis	GCA_000400635.2	actinobacteria
WP_016335695.1	EACC1	EACC1*→ CASPASE+DnaJ_ZnR+DnaJ_ZnR+DnaJ_C→	122	Amycolatopsis orientalis	GCA_000400635.2	actinobacteria
WP_016432744.1	EACC1	EACC1*→ CASPASE+TPRs→	75	Streptomyces sp. HGB0020	GCA_000411315.1	actinobacteria
WP_016435707.1	EACC1	EACC1*→ CASPASE+??+TM+??→	118	Streptomyces sp. HGB0020	GCA_000411315.1	actinobacteria
WP_016572868.1	EACC1	EACC1*→	124	Streptomyces albulus	GCA_000963515.1	actinobacteria
WP_016640036.1	EACC1	EACC1*→ CASPASE+STAND+polar_region+TM+BetaPropeller→	142	Streptomyces aurantiacus	GCA_000414115.1	actinobacteria
WP_017301504.1	EACC1	EACC1*→ CASPASE+DUF4062+??+HATPase_c_4+STAND→	125	Nodosilinea nodulosa	GCF_000309385.1	cyanobacteria
WP_017594165.1	EACC1	EACC1*→ CASPASE+TM?+BetaPropeller→	146	Nocardiopsis potens	GCF_000341105.1	actinobacteria
WP_017616278.1	EACC1	EACC1*→	122	Nocardiopsis salina	GCF_000341025.1	actinobacteria
WP_017982730.1	EACC1	EACC1*→ CASPASE+STAND+polar_region+TM+BetaPropeller→	122	Amycolatopsis methanolica	GCA_000739085.1	actinobacteria
WP_018087816.1	EACC1	EACC1*→ CASPASE+FGS_fold-domain→??→??→ TM+TM+BaxI_1→?→ NUDIX→	134	Streptomyces sp. FxanaC1	GCF_000375625.1	actinobacteria
WP_018088837.1	EACC1	EACC1*→ CASPASE+DnaJ_ZnR+DnaJ_ZnR+DnaJ_C→	135	Streptomyces sp. FxanaC1	GCF_000375625.1	actinobacteria
WP_018089822.1	EACC1	-	155	Streptomyces sp. FxanaC1	-	actinobacteria
WP_018348269.1	EACC1	EACC1*→ CASPASE+STAND+polar_region+TM+BetaPropeller→	123	Longispora albida	GCF_000379825.1	actinobacteria

acc	architecture	operon	len	species	gca	taxend
WP_018382060.1	EACC1	EACC1*→ CASPASE+TPRs→	121	Streptomyces vitaminophilus	GCA_001445835.1	actinobacteria
WP_018487316.1	EACC1	EACC1*→ CASPASE+TPRs??+GHKL_ATPase+TPRs??→	121	Streptomyces sp. CcalMP-8W	GCF_000373305.1	actinobacteria
WP_018537276.1	EACC1	??→ EACC1*→ CASPASE+TPRs→	126	Streptomyces sp. MspMP-M5	GCF_000373585.1	actinobacteria
WP_018538327.1	EACC1	EACC1*→ CASPASE+TM+Beta_rich→	116	Streptomyces sp. MspMP-M5	GCF_000373585.1	actinobacteria
WP_018540178.1	EACC1	EACC1*→ CASPASE+TM+??→	78	Streptomyces sp. MspMP-M5	GCF_000373585.1	actinobacteria
WP_018541940.1	EACC1	EACC1*→ CASPASE+TM+BetaPropeller→	117	Streptomyces sp. MspMP-M5	GCF_000373585.1	actinobacteria
WP_018549570.1	EACC1	-	149	Streptomyces sp. LaPpAH-108	-	actinobacteria
WP_018554561.1	EACC1	-	127	Streptomyces sp. ATexAB-D23	-	actinobacteria
WP_018558425.1	EACC1	EACC1*→ CASPASE+PBPB+vWA→	122	Streptomyces sp. BoleA5	GCF_000373665.1	actinobacteria
WP_018569469.1	EACC1	TPRs→ EACC1*→ CASPASE+TPRs→	114	Streptomyces sp. PsTaAH-124	GCF_000373685.1	actinobacteria
WP_018654763.1	EACC1	EACC1*→ CASPASE+SAD+HNH→	123	Actinomadura flavalba	GCF_000374305.1	actinobacteria
WP_018683317.1	EACC1	EACC1*→ CASPASE+TPRs→	139	Actinokineospora enzanensis	GCF_000374445.1	actinobacteria
WP_018842129.1	EACC1	-	135	Streptomyces sp. CNS335	-	actinobacteria
WP_018851576.1	EACC1	EACC1*→ CASPASE+??→	137	Streptomyces sp. CNY243	GCF_000377165.1	actinobacteria
WP_018895946.1	EACC1	TM+REase→ EACC1*→ CASPASE+??+SF1-helicase+Vsr(PDDEXK)?→	132	Streptomyces sp. CNY228	GCF_000377545.1	actinobacteria
WP_018956197.1	EACC1	Beta_rich?→ EACC1*→ CASPASE+TPRs→??→??→	133	Streptomyces sp. CNB091	GCF_000377965.1	actinobacteria
WP_019060213.1	EACC1	polar_lead?+TM+TM+TM+polar_tail?+low-complexity→??→ EACC1*→ CASPASE+TPRs→	159	Streptomyces prunicolor	GCF_000367365.1	actinobacteria
WP_019075768.1	EACC1	EACC1*→ CASPASE+STAND+BetaPropeller→	167	Streptomyces hokutonensis	GCF_000376565.1	actinobacteria
WP_019325097.1	EACC1	EACC1*→ CASPASE+TPRs→	138	Streptomyces sp. TOR3209	GCF_000259895.1	actinobacteria
WP_019329427.1	EACC1	Trypsin+HDxxH+VMAP-M0+CASPASE→ EACC1*→	-	-	GCF_000259895.1	-
WP_019608401.1	EACC1	EACC1*→ CASPASE+TM?+BetaPropeller→	126	Nocardiopsis	GCA_002260685.1	actinobacteria
WP_019750099.1	EACC1	EACC1*→ CASPASE+??→	128	Rhodococcus	GCF_000469645.1	actinobacteria
WP_019885900.1	EACC1	EACC1*→ CASPASE+STAND+polar_region+TM+BetaPropeller→	204	Streptomyces purpureus	GCF_000384175.1	actinobacteria
WP_019927514.1	EACC1	EACC1*→ CASPASE+TM+BetaPropeller→ CASPASE+TM+TM+BetaPropeller→ CASPASE+TM+BetaPropeller→	191	Nocardia sp. BMG111209	GCF_000381925.1	actinobacteria
WP_019929385.1	EACC1	EACC1*→	96	Nocardia sp. BMG111209	GCF_000381925.1	actinobacteria
WP_019930719.1	EACC1	EACC1*→ CASPASE+TPRs→	116	Nocardia sp. BMG111209	GCF_000381925.1	actinobacteria
WP_019983234.1	EACC1	TPRs→ EACC1*→ CASPASE+TPRs→	114	Streptomyces sp. Amel2xE9	GCF_000383935.1	actinobacteria
WP_020115005.1	EACC1	EACC1*→ CASPASE+TPRs→	119	Streptomyces bottropensis	GCF_000340335.1	actinobacteria
WP_020117476.1	EACC1	low-complexity+HD+Tox-RelA+TGS+ACT→ Pkinase+TPRs→ EACC1*→ CASPASE+TPRs→	124	Streptomyces canus	GCF_000383615.1	actinobacteria
WP_020135550.1	EACC1	EACC1*→ CASPASE+TPRs→	151	Streptomyces sp. 351MFTsu5.1	GCF_000383655.1	actinobacteria
WP_020141601.1	EACC1	EACC1*→ CASPASE+??+TM+TM+TM+TM+TM+TM+TM+TM→	124	Terracoccus sp. 273MFTsu3.1	GCF_000383675.1	actinobacteria
WP_020419828.1	EACC1	Pkinase+TM+TM+DUF3298+PSBP→ EACC1*→ CASPASE+STAND+polar_region+TM+BetaPropeller→||←?||?→||←PSE←?←?←FAD_binding_4+??+BBE	125	Amycolatopsis sp. ATCC 39116	GCF_000231075.2	actinobacteria
WP_020464182.1	TIR+EACC1+EAD1	??+TM+BetaPropeller→ TIR+EACC1+EAD1*→	405	Frankia sp. EAN1pec	GCA_000018005.1	actinobacteria
WP_020509053.1	EACC1	EACC1*→ CASPASE+STAND+polar_region+TM+BetaPropeller→	118	Actinoplanes globisporus	GCF_000379645.1	actinobacteria
WP_020629315.1	EACC1	EACC1*→ CASPASE+TPRs→	109	Amycolatopsis alba	GCA_002234385.1	actinobacteria
WP_020631088.1	EACC1	EACC1*→	74	Amycolatopsis alba	GCA_002234385.1	actinobacteria
WP_020642628.1	EACC1	GATase→ ABC-ATPase+DUF4162→ cNMP_cyclase+TM+TM+TM+TM+TM+TM→ EACC1*→	104	Amycolatopsis balhimycina	GCA_003947255.1	actinobacteria
WP_020663538.1	EACC1	GATase→ ABC-ATPase+DUF4162→ cNMP_cyclase+TM+TM+TM+TM+TM+TM→ EACC1*→	108	Amycolatopsis benzoatilytica	GCF_000383915.1	actinobacteria
WP_020666482.1	EACC1	??+TM+TM+??+TM→ PBPB→ EACC1*→ CASPASE+TPRs→ PknG_rubred+Pkinase+PknG_TPR→ ZnR+PP2C→ vWA+alpha-helical→	114	Amycolatopsis nigrescens	GCF_000384315.1	actinobacteria
WP_023539736.1	EACC1	EACC1*→ CASPASE+??+SF1-helicase+Vsr(PDDEXK)?→	138	Streptomyces niveus	GCA_000497425.1	actinobacteria
WP_023560335.1	EACC1	EACC1*→ CASPASE+??→	122	Actinoplanes friuliensis	GCA_000494755.1	actinobacteria
WP_024806583.1	EACC1	EACC1*→ CASPASE+??→	116	Nocardia sp. BMG51109	GCF_000526215.1	actinobacteria
WP_024934407.1	EACC1	EACC1*→ CASPASE+??+TM+TM+TM→	126	Actinomadura madurae	GCF_000468475.2	actinobacteria
WP_025327428.1	EACC1	EACC1*→	119	Aeromonas hydrophila	GCF_000512185.1	gammaproteobacteria
WP_025348143.1	EACC1	DUF2510(W3)+BicD+DUF4041+T5orf172→ EACC1*→ CASPASE+TPRs→	122	Nocardia nova	GCA_000523235.1	actinobacteria
WP_025357936.1	EACC1	polar_lead?+TM+TM+TM+polar_tail?+low-complexity→??→ EACC1*→ CASPASE+TPRs→?→ CASPASE+??+TPRs?→	116	Kutzneria albida	GCF_000525635.1	actinobacteria
WP_026047540.1	EACC1	EACC1*→ CASPASE+??+SF1-helicase+Vsr(PDDEXK)?→	138	Streptomyces	GCA_900116145.1	actinobacteria
WP_026469049.1	EACC1	PBPB→?→?→ Aminotran_5+PAS_6+HTH_22→ EACC1*→	126	Amycolatopsis balhimycina	GCA_003947255.1	actinobacteria
WP_026855269.1	EACC1	EACC1*→ CASPASE+TM+TM+TM+TM+TM+TM+TM+TM→	124	Geodermatophilaceae bacterium URHB0062	GCF_000620185.1	actinobacteria
WP_026931303.1	EACC1	EACC1*→	113	Glycomyces tenuis	GCF_000427885.1	actinobacteria
WP_027003174.1	EACC1	EACC1*→ CASPASE+??→	131	Hugenholtzia roseola	GCF_000422585.1	bacteroidetes

acc	architecture	operon	len	species	gca	taxend
WP_027746727.1	EACC1	EACC1*→ CASPASE+TPRs→	149	Streptomyces sp. CNT371	GCF_000514995.1	actinobacteria
WP_027770853.1	EACC1	EACC1*→ CASPASE+STAND+TPRs→	132	Streptomyces sp. CNQ329	GCF_000482585.1	actinobacteria
WP_027930057.1	EACC1	Pkinase+TM+TM+DUF3298+PSBP→ EACC1*→ CASPASE+STAND+polar_region+TM+BetaPropeller→	125	Amycolatopsis thermoflava	GCF_000473265.1	actinobacteria
WP_028419166.1	EACC1	EACC1*→ CASPASE+TPRs??+GHKL_ATPase+TPRs??→	121	Streptomyces sp. SolWspMP-sol2th	GCF_000647875.1	actinobacteria
WP_028435836.1	EACC1	EACC1*→ CASPASE+TPRs→	119	Streptomyces sp. TAA486	GCF_000426165.1	actinobacteria
WP_028661367.1	EACC1	EACC1*→	128	Nocardioides insulae	GCF_000422825.1	actinobacteria
WP_029721226.1	EACC1	EACC1*→ CASPASE+??+SF1-helicase+Vsr(PDDEXK)?→	130	Saccharopolyspora rectivirgula	GCA_000710755.1	actinobacteria
WP_030029222.1	EACC1	-	118	Streptomyces flavotricini	-	actinobacteria
WP_030076799.1	EACC1	EACC1*→ CASPASE+DnaJ_ZnR+DnaJ_ZnR+DnaJ_C→	135	Streptomyces decoyicus	GCA_001270575.1	actinobacteria
WP_030116247.1	EACC1	low-complexity+HD+Tox-RelA+TGS+ACT→ Pkinase+TPRs→ EACC1*→ CASPASE+TPRs→	135	Streptomyces	GCF_000717015.1	actinobacteria
WP_030125864.1	EACC1	EACC1*→	118	[Kitasatospora] papulosa	GCF_000717245.1	actinobacteria
WP_030154375.1	EACC1	EACC1*→ CASPASE+??+SF1-helicase+Vsr(PDDEXK)?→	136	Streptomyces sp. NRRL S-244	GCF_000717915.1	actinobacteria
WP_030154700.1	EACC1	EACC1*→ CASPASE+TPRs→	112	Glycomyces sp. NRRL B-16210	GCA_004364985.1	actinobacteria
WP_030177526.1	EACC1	??→ EACC1*→	114	Streptomyces sp. NRRL S-813	GCF_000718945.1	actinobacteria
WP_030192630.1	EACC1	EACC1*→ CASPASE+Concanavalin?→	126	Streptomyces	GCA_900090135.1	actinobacteria
WP_030192767.1	EACC1	low-complexity+HD+Tox-RelA+TGS+ACT→ Pkinase+TPRs→ EACC1*→ CASPASE+TPRs→	134	Streptomyces sp. NRRL S-87	GCF_000719335.1	actinobacteria
WP_030195455.1	EACC1	low-complexity+HD+Tox-RelA+TGS+ACT→ Pkinase+TPRs→ EACC1*→ CASPASE+TPRs→	135	Streptomyces	GCA_002082175.1	actinobacteria
WP_030219435.1	EACC1	EACC1*→ CASPASE+STAND+polar_region+TM+BetaPropeller→ HTH+N6_Mtase+Methylase_S→	129	Streptomyces sp. NRRL WC-3626	GCF_000718785.1	actinobacteria
WP_030241573.1	EACC1	EACC1*→ CASPASE+STAND+??+TPRs+??→	78	Streptomyces	GCF_000717875.1	actinobacteria
WP_030253333.1	EACC1	EACC1*→ CASPASE+TM?+BetaPropeller→	139	Streptomyces violens	GCF_000717745.1	actinobacteria
WP_030294927.1	EACC1	EACC1*→ CASPASE+Concanavalin?→	126	Streptomyces	GCA_001279425.1	actinobacteria
WP_030312162.1	EACC1	EACC1*→ CASPASE+??+SF1-helicase+Vsr(PDDEXK)?→ REase+STAND→	138	Streptomycetaceae	GCA_000707945.1	actinobacteria
WP_030334374.1	EACC1	TM+TM+TM+Peptidase_M48+TM+DUF4864+??→ EACC1*→	130	Micromonospora parva	GCF_000718515.1	actinobacteria
WP_030338292.1	EACC1	EACC1*→ CASPASE+TM+TM+bPH_domain?→	109	Streptomyces sp. NRRL S-1022	GCF_000720135.1	actinobacteria
WP_030339067.1	EACC1	EACC1*→ CASPASE+Concanavalin?→	126	Streptomyces floridae	GCF_000717665.1	actinobacteria
WP_030348773.1	EACC1	EACC1*→	119	Streptomyces sp. NRRL S-1022	GCF_000720135.1	actinobacteria
WP_030398398.1	EACC1	EACC1*→ CASPASE+HSP70→	151	Kitasatospora purpeofusca	GCF_000718025.1	actinobacteria
WP_030411720.1	EACC1	EACC1*→ CASPASE+TM+PBPB+vWA→	147	Streptomyces sp. NRRL S-1448	GCF_000719365.1	actinobacteria
WP_030453461.1	EACC1	EACC1*→	156	Herbidospora cretacea	GCF_000719065.1	actinobacteria
WP_030489041.1	EACC1	TM+TM+TM+Peptidase_M48+TM+DUF4864+??→ EACC1*→	134	Micromonospora chokoriensis	GCF_000718555.1	actinobacteria
WP_030490635.1	EACC1	EACC1*→	105	Micromonospora chokoriensis	GCF_000718555.1	actinobacteria
WP_030494645.1	EACC1	HATPase_c_2→ EACC1*→ CASPASE+??+SF1-helicase+Vsr(PDDEXK)?→	141	Microtetraspora glauca	-	actinobacteria
WP_030498129.1	EACC1	EACC1*→ CASPASE+STAND+??+TPRs+??→	162	Microtetraspora glauca	-	actinobacteria
WP_030521461.1	EACC1	EACC1*→ CASPASE+TPRs→	114	Nocardia rhamnosiphila	GCF_001613485.1	actinobacteria
WP_030546253.1	EACC1	EACC1*→ CASPASE+PBPB+vWA→	129	Streptomyces albus	GCF_000719865.1	actinobacteria
WP_030546694.1	EACC1	EACC1*→ CASPASE+TPRs→??→??→	118	Streptomyces albus	GCF_000719865.1	actinobacteria
WP_030575480.1	EACC1	EACC1*→??+vWA→?→ CASPASE+TPRs→	113	Streptomyces sclerotialus	-	actinobacteria
WP_030609229.1	EACC1	EACC1*→ CASPASE+??+SF1-helicase+Vsr(PDDEXK)?→	127	Streptomyces achromogenes	GCF_000720835.1	actinobacteria
WP_030629352.1	EACC1	EACC1*→ CASPASE+??+SF1-helicase+Vsr(PDDEXK)?→	138	Streptomyces	GCA_900090065.1	actinobacteria
WP_030639116.1	EACC1	EACC1*→	118	Streptomyces sp. NRRL B-24051	GCF_000720405.1	actinobacteria
WP_030648078.1	EACC1	EACC1*→ CASPASE+??+SF1-helicase+Vsr(PDDEXK)?→??→	145	Streptomyces rimosus	GCF_000718675.1	actinobacteria
WP_030656920.1	EACC1	EACC1*→ CASPASE+TM?+BetaPropeller→ MDMPI_N+MDMPI_C→ META→	116	Streptomyces	GCA_001047335.1	actinobacteria
WP_030679573.1	EACC1	EACC1*→ CASPASE+TPRs→	119	Streptomyces cellulosae	GCF_000721105.1	actinobacteria
WP_030705286.1	EACC1	Beta_rich?→ EACC1*→ CASPASE+TPRs→??→??→	133	Streptomyces	GCA_900090075.1	actinobacteria
WP_030715834.1	EACC1	EACC1*→ CASPASE+??+SF1-helicase+Vsr(PDDEXK)?→	135	Streptomyces sp. NRRL F-2580	GCF_000720115.1	actinobacteria
WP_030733252.1	EACC1	-	142	Streptomyces sp. NRRL F-2890	-	actinobacteria
WP_030738956.1	EACC1	EACC1*→ CASPASE+TPRs→??→??→	117	Streptomyces sp. NRRL F-2890	GCF_000719915.1	actinobacteria
WP_030741736.1	EACC1	EACC1*→ CASPASE+TPRs→	112	Streptomyces griseus	GCF_000718235.1	actinobacteria
WP_030777398.1	EACC1	EACC1*→ CASPASE+??+SF1-helicase+Vsr(PDDEXK)?→	127	Streptomyces lavenduligriseus	GCF_000718625.1	actinobacteria
WP_030783977.1	EACC1	EACC1*→ CASPASE+TM?+BetaPropeller→	133	Streptomyces lavenduligriseus	GCF_000718625.1	actinobacteria
WP_030815966.1	EACC1	EACC1*→ CASPASE+TM?+BetaPropeller→	156	Streptomyces sp. NRRL S-337	GCF_000720175.1	actinobacteria
WP_030821552.1	EACC1	EACC1*→ CASPASE+??+SF1-helicase+Vsr(PDDEXK)?→	140	Streptomyces	GCA_000966965.1	actinobacteria

acc	architecture	operon	len	species	gca	taxend
WP_030833567.1	EACC1	EACC1*→ CASPASE+STAND+??+TPRs→	133	Streptomyces	GCA_001279005.1	actinobacteria
WP_030850288.1	EACC1	EACC1*→ CASPASE+TPRs→	119	Streptomyces sp. NRRL F-4474	GCF_000719855.1	actinobacteria
WP_030860065.1	EACC1	-	116	Streptomyces sp. NRRL F-2747	-	actinobacteria
WP_030874413.1	EACC1	EACC1*→ CASPASE+Pkinase+TM?+??→	121	Streptomyces varsoviensis	GCA_001270595.1	actinobacteria
WP_030882299.1	EACC1	EACC1*→ CASPASE+TPRs→	165	Streptomyces sp. NRRL S-1868	GCF_000721305.1	actinobacteria
WP_030892741.1	EACC1	EACC1*→ CASPASE+TPRs→	162	Streptomyces sp. NRRL F-5053	GCF_000721445.1	actinobacteria
WP_030924025.1	EACC1	polar_lead?+TM+TM+TM+polar_tail?+low-complexity→??→ EACC1*→ CASPASE+TPRs→	118	Streptomyces sp. NRRL S-384	GCF_000721625.1	actinobacteria
WP_030935869.1	EACC1	EACC1*→	118	Streptomyces sp. NRRL B-24720	GCF_000721715.1	actinobacteria
WP_030968235.1	EACC1	DUF262+DUF1524+DNA_binding_1+??→?→ polar_lead?+TM+TM+TM+polar_tail?+low-complexity→??→ EACC1*→	124	Streptomyces sp. NRRL S-1824	GCF_000719575.1	actinobacteria
WP_030987664.1	EACC1	EACC1*→ CASPASE+STAND+polar_region+TM+BetaPropeller→	111	Streptomyces sp. NRRL S-1813	GCF_000719605.1	actinobacteria
WP_030991892.1	EACC1	EACC1*→ CASPASE+DnaJ_ZnR+DnaJ_ZnR+DnaJ_C→	135	Streptomyces sp. NRRL S-1813	GCF_000719605.1	actinobacteria
WP_031005419.1	EACC1	HATPase_c_2→ EACC1*→ CASPASE+??+SF1-helicase+Vsr(PDDEXK)?→	141	Streptomyces sp. NRRL F-5727	GCF_000719555.1	actinobacteria
WP_031014439.1	EACC1	EACC1*→ CASPASE+STAND+??+TPRs+??→	123	Streptomyces sp. NRRL F-5727	GCF_000719555.1	actinobacteria
WP_031080561.1	EACC1	EACC1*→ CASPASE+TPRs→ TPRs→	118	Streptomyces sp. NRRL WC-3549	GCF_000721935.1	actinobacteria
WP_031084685.1	EACC1	EACC1*→	134	Streptomyces sp. NRRL WC-3549	GCF_000721935.1	actinobacteria
WP_031146689.1	EACC1	EACC1*→ CASPASE+??+SF1-helicase+Vsr(PDDEXK)?→	140	Streptomyces erythrochromogenes	GCF_000725555.1	actinobacteria
WP_031160229.1	EACC1	EACC1*→ CASPASE+??+SF1-helicase+Vsr(PDDEXK)?→	126	Streptomyces durhamensis	GCF_000725475.1	actinobacteria
WP_031484390.1	EACC1	EACC1*→ CASPASE+TM?+BetaPropeller→ MDMPI_N+MDMPI_C→	117	Streptomyces bicolor	GCF_000725465.1	actinobacteria
WP_032291538.1	EACC1	EACC1*→	116	Enterobacteriaceae	GCA_004219385.1	gammaproteobacteria
WP_032773793.1	EACC1	-	118	Streptomyces griseus	-	actinobacteria
WP_032798005.1	EACC1	EACC1*→	131	Streptomyces filamentosus	GCF_000156455.1	actinobacteria
WP_033030163.1	EACC1	EACC1*→ CASPASE+??+SF1-helicase+Vsr(PDDEXK)?→??→	145	Streptomyces rimosus	GCA_003947585.1	actinobacteria
WP_033085337.1	EACC1	EACC1*→ CASPASE+STAND+TPRs→	125	Nocardia seriolae	GCA_000583715.2	actinobacteria
WP_033263563.1	EACC1	PBPB→?→?→ Aminotran_5+PAS_6+HTH_22→ EACC1*→ CASPASE+??+TM+TM→||←Glyco_trans_4_4+UDPGT	125	Amycolatopsis vancoresmycina	GCF_000388135.1	actinobacteria
WP_033263828.1	EACC1	EACC1*→ CASPASE+TPRs→	117	Streptomyces	GCA_002189675.1	actinobacteria
WP_033269153.1	EACC1	Glyoxalase→ EACC1*→ CASPASE+DnaJ_ZnR+DnaJ_ZnR+DnaJ_C→	135	Streptomyces lydicus	GCF_001984445.1	actinobacteria
WP_033291480.1	EACC1	EACC1→ CASPASE+TPRs→?→ EACC1*→	109	Amycolatopsis jejuensis	GCF_000717335.1	actinobacteria
WP_033314394.1	EACC1	EACC1*→ CASPASE+TM+TM+PBPB+vWA→	121	Streptomyces iakyrus	GCF_000717055.1	actinobacteria
WP_033355495.1	EACC1	EACC1*→ CASPASE+STAND+polar_region+TM+BetaPropeller→	131	Kitasatospora aureofaciens	GCF_000716885.1	actinobacteria
WP_033946822.1	EACC1	EACC1*→ CASPASE+??→	154	Streptomyces sp. CNQ431	GCF_000797385.1	actinobacteria
WP_034092777.1	EACC1	EACC1*→ CASPASE+TPRs→ Pkinase+??→	116	Streptacidiphilus albus	GCF_000787755.1	actinobacteria
WP_034262749.1	EACC1	polar_lead?+TM+TM+TM+polar_tail?+low-complexity→??→ EACC1*→ CASPASE+TPRs→	149	Actinospica robiniae	GCF_000504285.1	actinobacteria
WP_034267997.1	EACC1	EACC1*→ CASPASE+??→	113	Actinospica robiniae	GCF_000504285.1	actinobacteria
WP_034484638.1	EACC1	EACC1*→ CASPASE+BetaPropeller→	111	Actinomadura oligospora	GCF_000518265.1	actinobacteria
WP_035279790.1	EACC1	EACC1*→ CASPASE+??+ART→	117	Actinokineospora spheciospongiae	GCA_003182415.1	actinobacteria
WP_035302986.1	EACC1	EACC1*→	118	Actinokineospora inagensis	GCF_000482865.1	actinobacteria
WP_036372545.1	EACC1	TM+TM+TM+Peptidase_M48+TM+DUF4864+??→ EACC1*→	140	Micromonospora sp. ATCC 39149	GCF_000158815.1	actinobacteria
WP_036514812.1	EACC1	EACC1*→ CASPASE+TPRs→	121	Nocardia rhamnosiphila	GCF_001613485.1	actinobacteria
WP_036538668.1	EACC1	RNR_N+Ribonuc_red_lgN+Ribonuc_red_lgC→ EACC1*→	119	Nocardia cyriacigeorgica	GCA_005863225.1	actinobacteria
WP_037320246.1	EACC1	EACC1*→	110	Amycolatopsis orientalis	GCF_000478275.1	actinobacteria
WP_037342923.1	EACC1	EACC1*→	101	Amycolatopsis sp. MJM2582	GCA_000754115.1	actinobacteria
WP_037367462.1	EACC1	EACC1*→	108	Amycolatopsis orientalis	GCF_000411975.1	actinobacteria
WP_037570982.1	EACC1	EACC1*→ CASPASE+TPRs→	122	Streptacidiphilus oryzae	GCF_000744815.1	actinobacteria
WP_037662633.1	EACC1	-	150	Streptomyces griseofuscus	-	actinobacteria
WP_037683222.1	EACC1	EACC1*→ CASPASE+Trypsin+STAND+??+TM→	134	Streptomyces griseus	GCF_000720255.1	actinobacteria
WP_037710889.1	EACC1	EACC1*→ CASPASE+STAND+PBPB→	138	Streptomyces sp. ATexAB-D23	GCF_000373645.1	actinobacteria
WP_037726786.1	EACC1	EACC1*→ CASPASE+STAND+polar_region+TM+BetaPropeller→	147	Streptomyces scopuliridis	GCA_003073355.1	actinobacteria
WP_037735136.1	EACC1	??→ EACC1*→ CASPASE+TM+TM+PBPB+vWA→	126	Streptomyces mirabilis	GCF_000746395.1	actinobacteria
WP_037737687.1	EACC1	EACC1*→ CASPASE+Beta_rich_repeat→	135	Streptomyces sp. CNT371	GCF_000514995.1	actinobacteria
WP_037738220.1	EACC1	EACC1*→ CASPASE+Cpn60_TCP1→	128	Streptomyces mirabilis	GCF_000746395.1	actinobacteria
WP_037738505.1	EACC1	EACC1*→ CASPASE+Beta_rich_repeat→	135	Streptomyces sp. CNQ865	GCF_000424805.1	actinobacteria
WP_037743072.1	EACC1	EACC1*→ CASPASE+STAND+TPRs→	132	Streptomyces mirabilis	GCF_000746395.1	actinobacteria

acc	architecture	operon	len	species	gca	taxend
WP_037743851.1	EACC1	EACC1*→ CASPASE+TPRs→	144	Streptomyces sp. CNQ-525	GCA_004217635.1	actinobacteria
WP_037743995.1	EACC1	polar_lead?+TM+TM+TM+polar_tail?+low-complexity→??→ EACC1*→ CASPASE+TPRs→	150	Streptomyces mirabilis	GCF_000746395.1	actinobacteria
WP_037754092.1	EACC1	EACC1*→ CASPASE+Beta_rich_repeat→	135	Streptomyces sp. CNQ-525	GCF_000701285.1	actinobacteria
WP_037792010.1	EACC1	EACC1*→ CASPASE+??→	132	Streptomyces sp. CNS606	GCF_000426325.1	actinobacteria
WP_037809200.1	EACC1	GATase→ ABC-ATPase+DUF4162→ cNMP_cyclase+TM+TM+TM+TM+TM+TM→ EACC1*→	109	Streptomyces sp. NRRL F-3213	GCF_000720075.1	actinobacteria
WP_037811931.1	EACC1	EACC1*→ CASPASE+??+TM+TM→	189	Streptomyces sp. MspMP-M5	GCF_000373585.1	actinobacteria
WP_037873834.1	EACC1	EACC1*→	120	Streptomyces	GCF_000373685.1	actinobacteria
WP_037877852.1	EACC1	EACC1*→ CASPASE+STAND+PBPB→	127	Streptomyces sp. NTK 937	GCF_000698495.1	actinobacteria
WP_037879140.1	EACC1	EACC1*→ CASPASE+TPRs→	119	Streptomyces sp. NTK 937	GCA_000698495.1	actinobacteria
WP_037891842.1	EACC1	TPRs→ EACC1*→ CASPASE+TPRs→	114	Streptomyces sp. Tu 6176	GCA_000611795.1	actinobacteria
WP_037958524.1	EACC1	EACC1*→ CASPASE+STAND+TPRs→	144	Streptomyces sp. PRh5	GCA_000590515.1	actinobacteria
WP_038508877.1	EACC1	EACC1*→ CASPASE+??→	121	Amycolatopsis japonica	GCA_000732925.1	actinobacteria
WP_038516952.1	EACC1	EACC1*→ CASPASE+TPRs→	110	Amycolatopsis japonica	GCA_000732925.1	actinobacteria
WP_039656817.1	EACC1	HATPase_c_2→ EACC1*→ CASPASE+??+SF1-helicase+Vsr(PDDEXK)?→	127	Streptomyces	GCA_000802245.2	actinobacteria
WP_039795335.1	EACC1	EACC1*→ CASPASE+TM+TM→	124	Nocardia araoensis	GCF_000308435.1	actinobacteria
WP_040694361.1	EACC1	EACC1*→ CASPASE+TM+BetaPropeller→	122	Nocardia vinacea	GCF_000308835.1	actinobacteria
WP_040712577.1	EACC1	EACC1*→	120	Nocardia takedensis	GCF_000308695.1	actinobacteria
WP_040745697.1	EACC1	EACC1*→ CASPASE+TPRs→	119	Nocardia transvalensis	GCF_000308875.1	actinobacteria
WP_040815634.1	EACC1	EACC1*→ CASPASE+BetaPropeller→	139	Nocardia concava	GCF_000308815.1	actinobacteria
WP_040828294.1	EACC1	DUF262+DUF1524+DNA_binding_1+??→?→ EACC1*→ CASPASE+STAND+TPRs+OMPdecase→	128	Nocardia jiangxiensis	GCF_000308615.1	actinobacteria
WP_040836686.1	EACC1	EACC1*→ CASPASE+TM+TM+PBPB+vWA→	111	Streptomyces acidiscabies	GCF_000242715.1	actinobacteria
WP_040857178.1	EACC1	EACC1*→ CASPASE+STAND+TPRs→	132	Nocardia niigatensis	GCF_000308655.1	actinobacteria
WP_041840760.1	EACC1	EACC1*→	119	Actinoplanes friuliensis	GCF_000494755.1	actinobacteria
WP_042150750.1	EACC1	EACC1*→ CASPASE+Beta_rich_repeat→	133	Streptomyces sp. NBRC 110027	GCA_000829715.2	actinobacteria
WP_042157862.1	EACC1	EACC1*→ CASPASE→ TPRs→	114	Streptomyces sp. NBRC 110027	GCA_000829715.2	actinobacteria
WP_042254254.1	EACC1	EACC1*→ CASPASE+??→	122	Nocardia brasiliensis	GCA_000308475.2	actinobacteria
WP_043262060.1	EACC1	TPRs→ EACC1*→ CASPASE+TPRs→	114	Streptomyces sp. e14	GCF_000162775.1	actinobacteria
WP_043450256.1	EACC1	EACC1*→ CASPASE+STAND+polar_region+TM+BetaPropeller→	127	Streptomyces nodosus	GCA_000819545.1	actinobacteria
WP_043453717.1	EACC1	-	149	Streptomyces fulvoviolaceus	-	actinobacteria
WP_043457436.1	EACC1	EACC1*→ CASPASE+TPRs→	120	Streptomyces fulvoviolaceus	GCF_000718165.1	actinobacteria
WP_043464872.1	EACC1	EACC1*→ CASPASE+PBPB+vWA→	129	Streptomyces	GCA_001190165.1	actinobacteria
WP_043631880.1	EACC1	EACC1*→ CASPASE+SAD+HNH→	121	Nonomuraea candida	GCF_000725485.1	actinobacteria
WP_043686591.1	EACC1	EACC1*→ CASPASE+TPRs→	118	Streptomyces xylophagus	GCF_000716435.1	actinobacteria
WP_043735402.1	EACC1	EACC1*→ CASPASE+STAND+BetaPropeller+BetaPropeller→	128	Nocardia asiatica	GCF_000308415.1	actinobacteria
WP_043775607.1	EACC1	PBPB→?→?→ Aminotran_5+PAS_6+HTH_22→ EACC1*→	125	Amycolatopsis rifamycinica	GCA_000695625.1	actinobacteria
WP_043787005.1	EACC1	GATase→ ABC-ATPase+DUF4162→ cNMP_cyclase+TM+TM+TM+TM+TM+TM→ EACC1*→	105	Amycolatopsis rifamycinica	GCA_000695625.1	actinobacteria
WP_043814231.1	EACC1	EACC1*→ CASPASE+DnaJ_ZnR+DnaJ_ZnR+DnaJ_C→	122	Allokutzneria albata	GCA_900103775.1	actinobacteria
WP_043831325.1	EACC1	GATase→ ABC-ATPase+DUF4162→ cNMP_cyclase+TM+TM+TM+TM+TM+TM→ EACC1*→	109	Amycolatopsis orientalis	GCF_000411995.1	actinobacteria
WP_043967418.1	EACC1	??+TM+TM+TM+Peptidase_M48+TM+DUF4864+??→ EACC1*→	141	Micromonospora carbonacea	GCA_000876225.1	actinobacteria
WP_044381661.1	EACC1	EACC1*→ CASPASE+TPRs→ Clp_N+Clp_N+ClpABN-AAA→	80	Streptomyces cyaneogriseus	GCA_000931445.1	actinobacteria
WP_044384776.1	EACC1	EACC1*→ CASPASE+Pkinase→	114	Streptomyces cyaneogriseus	GCF_000931445.1	actinobacteria
WP_044473271.1	EACC1	HTH+CASPASE→ EACC1*→	125	Streptomyces	GCF_000331005.1	actinobacteria
WP_044851652.1	EACC1	EACC1*→	110	Amycolatopsis orientalis	GCA_000943515.2	actinobacteria
WP_045318341.1	EACC1	EACC1*→	113	Lechevalieria aerocolonigenes	GCA_000955955.1	actinobacteria
WP_045558679.1	EACC1	EACC1*→ CASPASE+TM+TM+bPH_domain+TM+TM+bPH_domain+TM+TM→	125	Streptomyces sp. FxanaA7	GCF_000958545.1	actinobacteria
WP_045743884.1	EACC1	EACC1*→ CASPASE+TPRs→	107	Actinoplanes rectilineatus	GCF_000962825.1	actinobacteria
WP_046567869.1	EACC1	EACC1*→	134	Micromonospora sp. HK10	GCA_000982955.1	actinobacteria
WP_046569306.1	EACC1	TM+TM+TM+Peptidase_M48+TM+DUF4864+??→ EACC1*→	131	Micromonospora sp. HK10	GCA_000982955.1	actinobacteria
WP_046724302.1	EACC1	-	150	Streptomyces xiamenensis	-	actinobacteria
WP_046928395.1	EACC1	Phage_int_SAM_3+Phage_integrase→ EACC1*→ CASPASE+TPRs→	121	Streptomyces lydicus	-	actinobacteria
WP_047178561.1	EACC1	EACC1*→ CASPASE+STAND+??+TPRs+??→	119	Streptomyces sp. MNU77	-	actinobacteria
WP_047179412.1	EACC1	HATPase_c_2→ EACC1*→ CASPASE+Beta_rich_repeat→	143	Streptomyces sp. MNU77	-	actinobacteria

acc	architecture	operon	len	species	gca	taxend
WP_047400359.1	EACC1	EACC1*→	116	Enterobacteriaceae	GCA_003687835.1	gammaproteobacteria
WP_048818821.1	EACC1	EACC1*→ CASPASE+TM+TM+bPH_domain+TM+TM+bPH_domain+TM+TM→	124	Streptomyces turgidiscabies	GCF_000331005.1	actinobacteria
WP_048830083.1	EACC1	EACC1*→ CASPASE+FGS_fold-domain→??→??→ TM+TM+BaxI_1→?→ NUDIX→	136	Streptomyces decoyicus	GCA_001270575.1	actinobacteria
WP_048831498.1	EACC1	EACC1*→ CASPASE+Pkinase+TM?+??→	113	Streptomyces varsoviensis	GCF_000718635.1	actinobacteria
WP_048910577.1	EACC1	EACC1*→ PSE→ MDMPI_N+MDMPI_C→ META→	98	Streptomyces	GCA_001280045.1	actinobacteria
WP_049567493.1	EACC1	EACC1*→ CASPASE+low-complexity+BetaPropeller→	97	Nonomuraea sp. SBT364	GCF_001083785.1	actinobacteria
WP_049715171.1	EACC1	EACC1*→ CASPASE+STAND+polar_region+TM+BetaPropeller→	127	Streptomyces caatingaensis	GCA_001187435.1	actinobacteria
WP_049715549.1	EACC1	EACC1*→ CASPASE+TPRs→	124	Streptomyces caatingaensis	GCA_001187435.1	actinobacteria
WP_050008892.1	EACC1	EACC1*→	118	Escherichia coli	GCF_900092705.1	gammaproteobacteria
WP_050360662.1	EACC1	EACC1*→ CASPASE+STAND+??+TPRs+??→	119	Streptomyces	GCA_001189025.1	actinobacteria
WP_050367089.1	EACC1	EACC1*→	133	Kitasatospora aureofaciens	GCF_000719175.1	actinobacteria
WP_050372991.1	EACC1	EACC1*→ CASPASE+TM+TM+PBPB+vWA→	120	Streptomyces acidiscabies	GCF_001189015.1	actinobacteria
WP_050375602.1	EACC1	EACC1*→ CASPASE+low-complexity+BetaPropeller→	120	Streptomyces acidiscabies	GCA_001189015.1	actinobacteria
WP_050500757.1	EACC1	-	172	Streptomyces rimosus	-	actinobacteria
WP_050503036.1	EACC1	EACC1*→	125	Streptomyces monomycini	GCF_000715845.1	actinobacteria
WP_050504262.1	EACC1	EACC1*→ CASPASE+TM?+BetaPropeller→	175	Streptomyces rimosus	GCF_000718675.1	actinobacteria
WP_050506763.1	EACC1	-	185	Streptomyces rimosus	-	actinobacteria
WP_050507927.1	EACC1	-	172	Streptomyces peucetius	-	actinobacteria
WP_050508826.1	EACC1	EACC1*→ CASPASE+TM?+BetaPropeller→	174	Streptomyces rimosus	GCF_000716515.1	actinobacteria
WP_050514958.1	EACC1	-	155	Streptomyces rimosus	-	actinobacteria
WP_050593059.1	EACC1	EACC1*→ CASPASE+STAND+polar_region+TM+BetaPropeller→	122	Salinispora arenicola	GCF_000514555.1	actinobacteria
WP_050782610.1	EACC1	MglB→ DUF742→ sGTP-MglA→?→ Beta_rich?→ EACC1*→ CASPASE+TPRs→??→??→	129	Streptomyces griseoflavus	GCF_000158975.1	actinobacteria
WP_050791603.1	EACC1	low-complexity+HD+Tox-RelA+TGS+ACT→ Pkinase+TPRs→ EACC1*→ CASPASE+TPRs→	120	Streptomyces pristinaespiralis	GCF_000154945.1	actinobacteria
WP_050799858.1	EACC1	EACC1*→ CASPASE+TPRs→ Clp_N+Clp_N+Clp_N+Clp_N+ClpABN-AAA→	118	Streptomyces himastatinicus	GCF_001650215.1	actinobacteria
WP_050997167.1	EACC1+EAD1	FHA+??+low-complexity+FHA+ABC-ATPase+TM→ EACC1+EAD1*→||←CASPASE+STAND+polar_region+TM+BetaPropeller	227	Frankia alni	GCF_000058485.1	actinobacteria
WP_051022391.1	EACC1	EACC1*→ CASPASE+STAND+polar_region+TM+BetaPropeller→	128	Nocardia pneumoniae	GCF_000308755.1	actinobacteria
WP_051042155.1	EACC1	EACC1*→ CASPASE+TPRs→	114	Actinoplanes missouriensis	GCF_000284295.1	actinobacteria
WP_051044348.1	EACC1	EACC1*→ CASPASE+TPRs→??→??→	129	Streptomyces viridosporus	GCF_000316095.1	actinobacteria
WP_051047444.1	EACC1	EACC1*→ CASPASE+STAND→ BetaPropeller→	107	Nocardia asiatica	GCF_000308415.1	actinobacteria
WP_051104136.1	EACC1	EACC1*→ CASPASE+Beta_rich_repeat→	133	Streptomyces sp. FxanaC1	GCF_000375625.1	actinobacteria
WP_051158321.1	EACC1	EACC1*→	123	Nocardia carnea	GCF_000308515.1	actinobacteria
WP_051169530.1	EACC1	EACC1*→ CASPASE+STAND+polar_region+TM+BetaPropeller→	107	Nocardia abscessus	GCF_000308455.1	actinobacteria
WP_051175242.1	EACC1	Pkinase+TM+LppX_LprAFG→ CASPASE+TPRs→ EACC1*→	128	Nocardia exalbida	GCF_000308575.1	actinobacteria
WP_051182412.1	EACC1	EACC1*→ CASPASE+??→	115	Nocardia vinacea	GCF_000308835.1	actinobacteria
WP_051182843.1	EACC1	EACC1*→ CASPASE+EAL→	117	Nocardia vinacea	GCF_000308835.1	actinobacteria
WP_051274390.1	EACC1	EACC1*→ CASPASE+STAND+polar_region+TM+BetaPropeller→	123	Cellulomonas sp. URHD0024	GCF_000426185.1	actinobacteria
WP_051338412.1	EACC1	EACC1*→ CASPASE+TM?+BetaPropeller→	177	Streptomyces flavidovirens	GCF_000429085.1	actinobacteria
WP_051362899.1	EACC1	EACC1*→ CASPASE+TM+TM+TM+TM→	116	Amycolatopsis thermoflava	GCA_003752125.1	actinobacteria
WP_051367990.1	EACC1	PBPB→?→?→?→?→?→ EACC1*→	111	Hamadaea tsunoensis	GCF_000428945.1	actinobacteria
WP_051436354.1	EACC1	EACC1*→ CASPASE→	128	Streptomyces sp. CNT371	GCF_000514995.1	actinobacteria
WP_051450489.1	EACC1	EACC1*→	177	Actinospica robiniae	GCF_000504285.1	actinobacteria
WP_051450899.1	EACC1	EACC1*→ CASPASE+Pkinase+TM?+??→	118	Actinospica robiniae	GCF_000504285.1	actinobacteria
WP_051450927.1	EACC1	EACC1*→ CASPASE+TPRs→	115	Actinospica robiniae	GCF_000504285.1	actinobacteria
WP_051467403.1	EACC1	EACC1*→ CASPASE+SAD+HNH→?→||←METHYLASE	126	Actinomadura oligospora	GCF_000518265.1	actinobacteria
WP_051467905.1	EACC1	EACC1*→ CASPASE+low-complexity+BetaPropeller→	140	Actinomadura oligospora	GCF_000518265.1	actinobacteria
WP_051572740.1	EACC1	EACC1*→	93	Streptomyces sp. PRh5	GCA_000590515.1	actinobacteria
WP_051581547.1	EACC1	EACC1*→ CASPASE+TM+TM+TM+TM→	133	Pseudonocardia acaciae	GCF_000620785.1	actinobacteria
WP_051581839.1	EACC1	EACC1*→ CASPASE+STAND+polar_region+TM+BetaPropeller→	152	Pseudonocardia acaciae	GCF_000620785.1	actinobacteria
WP_051653552.1	EACC1	EACC1*→ CASPASE+??+ZnR+ZnR→	120	Kitasatospora cheerisanensis	GCA_000696185.1	actinobacteria
WP_051661499.1	EACC1	EACC1*→ CASPASE+TM+TM+bPH_domain?→	141	Streptomyces albulus	GCF_000695235.1	actinobacteria
WP_051661885.1	EACC1	EACC1*→	124	Streptomyces albulus	GCA_000695235.1	actinobacteria
WP_051673827.1	EACC1	EACC1*→ CASPASE+STAND+polar_region+TM+BetaPropeller→	132	Streptomyces sp. NTK 937	GCA_000698495.1	actinobacteria

acc	architecture	operon	len	species	gca	taxend
WP_051674065.1	EACC1	EACC1*→ CASPASE+STAND+PBPB→	150	Streptomyces sp. NTK 937	GCA_000698495.1	actinobacteria
WP_051674712.1	EACC1	Pkinase+TPRs→ EACC1*→ CASPASE+TPRs→	172	Streptomyces sp. URHA0041	GCF_000701325.1	actinobacteria
WP_051698691.1	EACC1	EACC1*→ CASPASE+TPRs→	121	Streptomyces sp. NRRL F-5917	GCA_001294325.1	actinobacteria
WP_051707420.1	EACC1	-	133	Streptomyces sp. NRRL B-12105	-	actinobacteria
WP_051708894.1	EACC1	EACC1*→	118	Micromonospora parva	GCF_000718515.1	actinobacteria
WP_051711433.1	EACC1	-	124	Streptomyces sp. NRRL S-350	-	actinobacteria
WP_051715268.1	EACC1	EACC1*→ CASPASE+TPRs→	124	Nocardia rhamnosiphila	GCF_001613485.1	actinobacteria
WP_051715276.1	EACC1	EACC1*→ CASPASE+??+SF1-helicase+Vsr(PDDEXK)?→	138	Nocardia rhamnosiphila	GCF_001613485.1	actinobacteria
WP_051723090.1	EACC1	-	129	Streptomyces albus	-	actinobacteria
WP_051723267.1	EACC1	EACC1*→ CASPASE+STAND+polar_region+TM+BetaPropeller→	138	Streptomyces albus	GCF_000719865.1	actinobacteria
WP_051724817.1	EACC1	EACC1*→ CASPASE+TPRs→ Pkinase+??→	131	Streptacidiphilus jeojiense	GCF_000719095.1	actinobacteria
WP_051727252.1	EACC1	EACC1*→ CASPASE+??→	137	Streptomyces griseus	GCF_000719495.1	actinobacteria
WP_051733700.1	EACC1	polar_lead?+TM+TM+TM+polar_tail?+low-complexity→??→ EACC1*→ CASPASE+TPRs→	126	Streptomyces	GCA_001729485.1	actinobacteria
WP_051737317.1	EACC1	EACC1*→ CASPASE+STAND+polar_region+TM+BetaPropeller→	148	Pseudonocardia autotrophica	GCF_000717175.1	actinobacteria
WP_051740136.1	EACC1	FAD_binding_4+FAD-oxidase_C→ DAGK_cat+DAGK_acc→ EACC1*→ CASPASE+TPRs→?→ Clp_N+Clp_N+ClpABN-AAA→	122	Kitasatospora sp. MBT66	GCF_000710405.2	actinobacteria
WP_051747232.1	EACC1	EACC1*→	133	Streptomyces sp. NRRL B-5680	GCF_000719415.1	actinobacteria
WP_051747308.1	EACC1	EACC1*→ CASPASE+??→	137	Streptomyces sp. NRRL B-5680	GCF_000719415.1	actinobacteria
WP_051751250.1	EACC1	-	123	Streptomyces griseoluteus	-	actinobacteria
WP_051751806.1	EACC1	EACC1*→ BetaPropeller(fragment)→	121	Streptosporangium amethystogenes	GCF_000721075.1	actinobacteria
WP_051753396.1	EACC1	EACC1*→ CASPASE+STAND+polar_region+TM+BetaPropeller→	127	Streptosporangium amethystogenes	GCF_000721075.1	actinobacteria
WP_051753712.1	EACC1	EACC1*→ CASPASE+STAND+polar_region+TM+BetaPropeller→	125	Streptosporangium amethystogenes	GCF_000721075.1	actinobacteria
WP_051754873.1	EACC1	FAD_binding_4+FAD-oxidase_C→ DAGK_cat+DAGK_acc→ EACC1*→ CASPASE+TPRs→ Clp_N+Clp_N+ClpABN-AAA→	122	Kitasatospora purpeofusca	GCF_000718025.1	actinobacteria
WP_051769029.1	EACC1+CASPASE+TPR_repeats+low_complexity+TM_region	TM+TM+BPD_transp_1→ OppC_N+TM+TM+BPD_transp_1→ ABC-ATPase+ABC-ATPase→ PBPB→ TM+Beta_rich+TM+Beta_rich+TM+TM→ EACC1+CASPASE+TPR_repeats+low_complexity+TM_region*→	774	Lentzea albidocapillata	GCA_900176525.1	actinobacteria
WP_051771299.1	EACC1	FAD_binding_4+FAD-oxidase_C→ DAGK_cat+DAGK_acc→ EACC1*→ CASPASE+TPRs→ Clp_N+Clp_N+ClpABN-AAA→	117	Streptomyces sp. NRRL F-6131	GCF_000719695.1	actinobacteria
WP_051789358.1	EACC1	EACC1*→ CASPASE+STAND+polar_region+TM+BetaPropeller→	132	Streptomyces sp. NRRL S-1022	GCF_000720135.1	actinobacteria
WP_051790042.1	EACC1	EACC1*→ CASPASE+low-complexity+BetaPropeller→	141	Streptomyces sp. NRRL S-1022	GCF_000720135.1	actinobacteria
WP_051791754.1	EACC1	EACC1*→ CASPASE+TPRs→?→ EACC1→	73	Amycolatopsis jejuensis	GCF_000717335.1	actinobacteria
WP_051797235.1	EACC1	METHYLASE→ EACC1*→ CASPASE+TM+TM+TM+TM→	122	Catenuloplanes japonicus	GCF_000717135.1	actinobacteria
WP_051797407.1	EACC1	EACC1*→ CASPASE+TM+Beta_rich→	138	Streptomyces sp. NRRL S-337	GCF_000720175.1	actinobacteria
WP_051817603.1	EACC1	EACC1*→ CASPASE+alpha-helical+TM+TM+??+TM+TM→	183	Kitasatospora sp. NRRL B-11411	GCF_000721345.1	actinobacteria
WP_051818683.1	EACC1	EACC1*→ CASPASE+FGS_fold-domain→??→??→	146	Streptomyces sp. NRRL S-1813	GCF_000719605.1	actinobacteria
WP_051820037.1	EACC1	EACC1*→ CASPASE+GAF+PAS+GGDEF→	116	Streptomyces sp. NRRL S-920	GCF_000717945.1	actinobacteria
WP_051820891.1	EACC1	EACC1*→ CASPASE+TPRs+LysM+PG-binding→	120	Streptomyces flavochromogenes	GCF_000717595.1	actinobacteria
WP_051822893.1	EACC1	EACC1*→ CASPASE+TM+Beta_rich→	138	Streptomyces sp. NRRL S-1448	GCF_000719365.1	actinobacteria
WP_051825763.1	EACC1	EACC1*→	133	Kitasatospora aureofaciens	GCF_000716885.1	actinobacteria
WP_051826953.1	EACC1	EACC1*→ CASPASE+TM+TM+bPH_domain?→	129	Streptomyces violaceoruber	GCF_000720805.1	actinobacteria
WP_051827480.1	EACC1	EACC1*→ CASPASE+TM+PBPI→	133	Streptomyces bicolor	GCF_000725465.1	actinobacteria
WP_051828064.1	EACC1	EACC1*→ CASPASE+alpha-helical+TM+TM+??+TM+TM→	155	Streptomyces sp. NRRL S-384	GCF_000721625.1	actinobacteria
WP_051828389.1	EACC1	EACC1*→ CASPASE+STAND+polar_region+TM+BetaPropeller→	143	Streptomyces bicolor	GCF_000725465.1	actinobacteria
WP_051830699.1	EACC1	EACC1*→ CASPASE+TPRs→	136	Streptomyces violens	GCF_000717745.1	actinobacteria
WP_051831085.1	EACC1	-	103	Streptomyces violens	-	actinobacteria
WP_051831682.1	EACC1	EACC1*→ CASPASE+STAND+polar_region+TM+BetaPropeller→	103	Streptomyces violens	GCF_000717745.1	actinobacteria
WP_051832798.1	EACC1	EACC1*→ CASPASE+GAF+PAS→	149	Streptomyces	GCA_001513955.1	actinobacteria
WP_051833041.1	EACC1	EACC1*→ CASPASE+??+TM+??→	120	Streptomyces sp. NRRL S-646	GCF_000720765.1	actinobacteria
WP_051833578.1	EACC1	EACC1*→ CASPASE+??→	116	Streptomyces katrae	GCF_000717655.1	actinobacteria
WP_051837966.1	EACC1	EACC1*→ CASPASE+??+TPRs→	114	Streptomyces sp. NRRL WC-3742	GCF_000720635.1	actinobacteria
WP_051840661.1	EACC1	-	93	Streptomyces sp. NRRL F-5126	-	actinobacteria
WP_051843364.1	EACC1	EACC1*→ CASPASE+TPRs→	132	Streptomyces sp. NRRL S-623	GCF_000725705.1	actinobacteria
WP_051843551.1	EACC1	EACC1*→ CASPASE+STAND+polar_region+TM+BetaPropeller→	130	Streptomyces	GCF_000718375.1	actinobacteria
WP_051850144.1	EACC1	EACC1*→	141	Streptomyces griseofuscus	GCF_000718315.1	actinobacteria
WP_051850290.1	EACC1	EACC1*→ CASPASE+GAF+PAS→	150	Streptomyces griseofuscus	GCA_003933295.1	actinobacteria

acc	architecture	operon	len	species	gca	taxend
WP_051853232.1	EACC1	EACC1*→ CASPASE+PBPB→	120	Streptomyces aureocirculatus	GCF_000720475.1	actinobacteria
WP_051860348.1	EACC1	Beta_rich?→ EACC1*→ CASPASE+TPRs→??→??→	125	Streptomyces anulatus	GCA_003846255.1	actinobacteria
WP_051860360.1	EACC1	-	150	Streptomyces anulatus	-	actinobacteria
WP_051865517.1	EACC1	??→ EACC1*→ CASPASE+TM+TM+PBPB+vWA→	108	Streptomyces griseus	GCF_000720255.1	actinobacteria
WP_051871299.1	EACC1	EACC1*→ CASPASE+STAND+polar_region+TM+BetaPropeller→	141	Streptomyces sclerotialus	-	actinobacteria
WP_051872051.1	EACC1	EACC1*→ CASPASE+TM+PBPB+vWA→	143	Streptomyces sclerotialus	-	actinobacteria
WP_051872372.1	EACC1	EACC1*→ CASPASE+TM?+BetaPropeller→	122	Streptomyces sclerotialus	-	actinobacteria
WP_051874280.1	EACC1	EACC1*→ CASPASE+TPRs→ DUF664→	121	Streptomyces exfoliatus	GCF_000725545.1	actinobacteria
WP_051877232.1	EACC1	EACC1*→ CASPASE+low-complexity+BetaPropeller→	114	Streptomyces natalensis	GCF_000935125.1	actinobacteria
WP_051877321.1	EACC1	PAS+GAF+PP2C+HATPase_c_2→ EACC1*→ CASPASE+TM+Beta_rich→	133	Streptomyces natalensis	GCF_000935125.1	actinobacteria
WP_051919877.1	EACC1	EACC1*→ CASPASE+??→	126	Streptomyces sp. PAN_FS17	GCA_900105465.1	actinobacteria
WP_051927179.1	EACC1	EACC1*→ CASPASE+TM+TM+bPH_domain?→	163	Streptomyces durhamensis	GCF_000725475.1	actinobacteria
WP_051951601.1	EACC1	EACC1*→ CASPASE+TPRs→	123	Streptomyces yeochonensis	GCF_000745345.1	actinobacteria
WP_051969288.1	EACC1	EACC1*→ CASPASE+DnaJ_ZnR+DnaJ_C+DnaJ_C+TM+TM→	106	Kitasatospora azatica	GCF_000744785.1	actinobacteria
WP_052069324.1	EACC1	EACC1*→||←?←?||Pkinase+??→	119	Streptacidiphilus albus	GCF_000744705.1	actinobacteria
WP_052163187.1	EACC1	EACC1*→ CASPASE+??→	118	Actinoplanes utahensis	GCF_000785985.1	actinobacteria
WP_052281097.1	EACC1	DUF2510(W3)+BicD+DUF4041+T5orf172→ EACC1*→ CASPASE+TPRs→	125	Nocardia vulneris	GCF_001613425.1	actinobacteria
WP_052287016.1	EACC1	low-complexity+HD+Tox-RelA+TGS+ACT→ Pkinase+TPRs→ EACC1*→ CASPASE+TPRs→	131	Streptomyces sp. 769	GCA_000816025.1	actinobacteria
WP_052287199.1	EACC1	polar_lead?+TM+TM+TM+polar_tail?+low-complexity→??→ EACC1*→ CASPASE+TPRs→	126	Streptomyces	GCA_000816025.1	actinobacteria
WP_052359567.1	EACC1	EACC1*→ CASPASE+DUF179→ SIR2+??→??→	115	Actinoplanes friuliensis	GCF_000494755.1	actinobacteria
WP_052371933.1	EACC1	EACC1*→ CASPASE+CLP_protease→	120	Amycolatopsis taiwanensis	GCF_000519205.1	actinobacteria
WP_052392347.1	EACC1	-	132	Streptomyces roseoverticillatus	-	actinobacteria
WP_052397909.1	EACC1	EACC1*→ CASPASE+STAND+TPRs→	130	Streptomyces sp. NRRL F-5123	GCF_000721495.1	actinobacteria
WP_052398340.1	EACC1	EACC1*→ CASPASE+BetaPropeller→	93	Streptomyces sp. NRRL F-5123	GCF_000721495.1	actinobacteria
WP_052407300.1	EACC1	EACC1*→	121	Allokutzneria albata	GCA_900103775.1	actinobacteria
WP_052412073.1	EACC1	low-complexity+HD+Tox-RelA+TGS+ACT→ Pkinase+TPRs→ EACC1*→ CASPASE+TPRs→	138	Streptomyces luteus	GCF_000739045.1	actinobacteria
WP_052413571.1	EACC1	EACC1*→ AB_hydrolase→	142	Streptomyces glaucescens	GCA_000761215.1	actinobacteria
WP_052423238.1	EACC1	EACC1*→ CASPASE+low-complexity+BetaPropeller→	125	Nonomuraea candida	GCF_000725485.1	actinobacteria
WP_052423634.1	EACC1	EACC1*→ CASPASE+TM+TM+??→	119	Nonomuraea candida	GCF_000725485.1	actinobacteria
WP_052478239.1	EACC1	PALP→ Trp_syntA→ TM+LGT→ EACC1*→	120	Kibdelosporangium sp. MJ126-NF4	GCF_000826545.1	actinobacteria
WP_052482684.1	EACC1	low-complexity+HD+Tox-RelA+TGS+ACT→ Pkinase+TPRs→ EACC1*→ CASPASE+TPRs→	141	Streptomyces albus	GCA_000827005.1	actinobacteria
WP_052577225.1	EACC1	-	149	Actinosporangium sp. NRRL B-3428	-	actinobacteria
WP_052701361.1	EACC1	EACC1*→	67	Streptomyces iranensis	GCF_000938975.1	actinobacteria
WP_052701406.1	EACC1	EACC1*→ CASPASE+low-complexity+TM+PBPB→	130	Streptomyces iranensis	GCA_000938975.1	actinobacteria
WP_052701431.1	EACC1	EACC1*→ CASPASE+TM+??→	147	Streptomyces iranensis	GCA_000938975.1	actinobacteria
WP_052705912.1	EACC1	EACC1*→ CASPASE+STAND+polar_region+TM+BetaPropeller→	89	Streptomyces sp. NRRL B-1568	GCF_000967025.1	actinobacteria
WP_052709520.1	EACC1	EACC1*→ CASPASE+TPRs→ Clp_N+Clp_N+ClpABN-AAA→	126	Streptomyces sp. NRRL S-495	GCF_000966955.1	actinobacteria
WP_052714810.1	EACC1	EACC1*→	113	Streptomyces	GCF_000720235.1	actinobacteria
WP_052720941.1	EACC1	EACC1*→||←CASPASE+STAND+TM+polar_region+TM+BetaPropeller	88	Actinoplanes rectilineatus	GCF_000962825.1	actinobacteria
WP_052731619.1	EACC1	low-complexity+HD+Tox-RelA+TGS+ACT→ Pkinase+TPRs→ EACC1*→ CASPASE+TPRs→	138	Streptomyces sp. MBT28	GCA_005490965.1	actinobacteria
WP_052742894.1	EACC1	EACC1*→ CASPASE+alpha-helical+TM+TM+??+TM+TM→	67	Streptomyces mangrovisoli	GCA_000974985.2	actinobacteria
WP_052743230.1	EACC1	Pkinase+TPRs→ EACC1*→ CASPASE+TPRs→	125	Streptomyces mangrovisoli	GCF_000974985.2	actinobacteria
WP_052770426.1	EACC1+CASPASE+BetaPropeller	EACC1+CASPASE+BetaPropeller*→ BetaPropeller→	703	Streptomyces sp. CNQ-509	GCF_001011035.1	actinobacteria
WP_052845486.1	EACC1	EACC1*→ CASPASE+TM+??→	78	Streptomyces sp. NRRL S-31	GCF_000718775.1	actinobacteria
WP_053048421.1	EACC1	EACC1*→ CASPASE+TM+??→	141	Streptomyces antioxidans	GCA_000968685.2	actinobacteria
WP_053048662.1	EACC1	EACC1*→ CASPASE+Peripla_BP_5→	127	Streptomyces antioxidans	-	actinobacteria
WP_053080830.1	EACC1	low-complexity+HD+Tox-RelA+TGS+ACT→ Pkinase+TPRs→ EACC1*→ CASPASE+TPRs→	138	Streptomyces roseus	GCF_001044425.1	actinobacteria
WP_053116041.1	EACC1	EACC1*→ CASPASE+TPRs→	109	Streptomyces xiamenensis	GCA_000993785.2	actinobacteria
WP_053164263.1	EACC1	-	134	Streptomyces ahygroscopicus	-	actinobacteria
WP_053164556.1	EACC1	EACC1*→ CASPASE+PBPB→	120	Streptomyces albulus	GCF_000935185.3	actinobacteria
WP_053271602.1	EACC1	EACC1*→	116	Enterobacteriaceae	GCA_002249075.1	gammaproteobacteria
WP_053626492.1	EACC1	EACC1*→ CASPASE+TPRs→	119	Streptomyces	GCA_001279445.1	actinobacteria

acc	architecture	operon	len	species	gca	taxend
WP_053634092.1	EACC1	EACC1*→ CASPASE+TPRs→	119	Streptomyces	GCA_001279855.1	actinobacteria
WP_053650957.1	EACC1	EACC1*→ CASPASE+TPRs→ Clp_N+Clp_N+ClpABN-AAA→	126	Streptomyces sp. XY431	GCF_001279805.1	actinobacteria
WP_053652889.1	EACC1	EACC1*→	117	Micromonospora sp. NRRL B-16802	GCA_001279555.1	actinobacteria
WP_053670302.1	EACC1	??→ EACC1*→ CASPASE+TM+TM+PBPB+vWA→	121	Streptomyces sp. NRRL B-1140	GCA_001280065.1	actinobacteria
WP_053674387.1	EACC1	EACC1*→ CASPASE→ TPRs→	117	Streptomyces sp. NRRL B-1140	GCA_001280065.1	actinobacteria
WP_053676249.1	EACC1	EACC1*→ CASPASE+??+SF1-helicase+Vsr(PDDEXK)?→	139	Streptomyces sp. WM4235	GCF_001279725.1	actinobacteria
WP_053683135.1	EACC1	EACC1*→ CASPASE+TM?+BetaPropeller→	123	Streptomyces sp. WM4235	GCA_001279725.1	actinobacteria
WP_053684084.1	EACC1	EACC1*→ CASPASE+??+SF1-helicase+Vsr(PDDEXK)?→	140	Streptomyces sp. XY593	GCF_001279885.1	actinobacteria
WP_053692927.1	EACC1	EACC1*→ CASPASE+??→	109	Streptomyces sp. WM6372	GCF_001279655.1	actinobacteria
WP_053694030.1	EACC1	low-complexity+HD+Tox-RelA+TGS+ACT→ Pkinase+TPRs→ EACC1*→ CASPASE+TPRs→	137	Streptomyces sp. WM6372	GCF_001279655.1	actinobacteria
WP_053694048.1	EACC1	EACC1*→ CASPASE+??→	116	Streptomyces sp. WM6372	GCA_001279655.1	actinobacteria
WP_053707259.1	EACC1	EACC1*→ CASPASE+STAND+polar_region+TM+BetaPropeller→	147	Streptomyces sp. NRRL B-3648	GCF_001279545.1	actinobacteria
WP_053711694.1	EACC1	EACC1*→ CASPASE+STAND+polar_region+TM+BetaPropeller→	130	Streptomyces sp. NRRL B-3648	GCA_001279545.1	actinobacteria
WP_053711828.1	EACC1	EACC1*→ CASPASE+low-complexity+BetaPropeller→	153	Streptomyces sp. NRRL B-3648	GCF_001279545.1	actinobacteria
WP_053733409.1	EACC1	EACC1*→ CASPASE+Cpn60_TCP1→	111	Nocardia sp. NRRL S-836	GCA_001279525.1	actinobacteria
WP_053751185.1	EACC1	EACC1*→	113	Streptomyces sp. MMG1533	GCA_001279775.1	actinobacteria
WP_053754404.1	EACC1	EACC1*→ CASPASE+STAND+TPRs→	133	Streptomyces sp. MMG1533	GCF_001279775.1	actinobacteria
WP_053763839.1	EACC1	-	116	Streptomyces sp. AS58	-	actinobacteria
WP_053764190.1	EACC1	EACC1*→ CASPASE+TM+TM+bPH_domain+TM+TM+bPH_domain+TM+TM→	76	Streptomyces sp. AS58	GCA_001279985.1	actinobacteria
WP_053790627.1	EACC1	EACC1*→ CASPASE+??→	116	Streptomyces sp. XY332	GCF_001280885.1	actinobacteria
WP_053802205.1	EACC1	EACC1*→	180	Streptomyces rimosus	GCF_001279345.1	actinobacteria
WP_053847006.1	EACC1	EACC1*→ CASPASE+BetaPropeller→	136	Streptomyces sp. NRRL B-24085	GCF_001282115.1	actinobacteria
WP_053923404.1	EACC1	TPRs→ EACC1*→ CASPASE+TPRs→	114	Streptomyces chattanoogensis	GCA_001294335.1	actinobacteria
WP_053926881.1	EACC1	EACC1*→ CASPASE+TM?+BetaPropeller→	156	Streptomyces chattanoogensis	GCA_001294335.1	actinobacteria
WP_053927196.1	EACC1	PAS+GAF+PP2C+HATPase_c_2→ EACC1*→	128	Streptomyces chattanoogensis	GCF_001294335.1	actinobacteria
WP_054001110.1	EACC1	EACC1*→ CASPASE+TM+??→	120	Shimia sp. SK013	GCA_001292625.1	alphaproteobacteria
WP_054048388.1	EACC1	EACC1*→ CASPASE+STAND+TPRs→	106	Alloactinosynnema sp. L-07	GCA_900070365.1	actinobacteria
WP_054066041.1	EACC1	EACC1*→	125	Comamonas kerstersii	GCA_002056725.1	betaproteobacteria
WP_054212290.1	EACC1	EACC1*→ CASPASE+low-complexity+BetaPropeller→	108	Actinobacteria bacterium OK074	GCF_001298575.1	actinobacteria
WP_054217804.1	EACC1	EACC1*→	141	Actinobacteria bacterium OK074	GCF_001298575.1	actinobacteria
WP_054294680.1	EACC1	PALP→ Trp_syntA→ TM+LGT→ EACC1*→	117	Kibdelosporangium phytohabitans	GCA_001302585.1	actinobacteria
WP_054812365.1	EACC1	EACC1*→	97	Nocardia arizonensis	GCF_001618405.1	actinobacteria
WP_055409063.1	EACC1+EAD1	CASPASE+STAND+polar_region+TM+BetaPropeller||EACC1+EAD1*→	291	Frankia sp. ACN1ag	GCA_001414035.1	actinobacteria
WP_055418791.1	EACC1	polar_lead?+TM+TM+TM+polar_tail?+low-complexity→??→ EACC1*→ CASPASE+TPRs→	145	Streptomyces pactum	GCA_002005225.1	actinobacteria
WP_055472090.1	EACC1	EACC1*→ CASPASE+Peripla_BP_5→	122	Streptomyces pathocidini	GCF_001418495.1	actinobacteria
WP_055519506.1	EACC1	EACC1*→	117	Streptomyces ossamyceticus	GCF_001418475.1	actinobacteria
WP_055520371.1	EACC1	EACC1*→ CASPASE→ TPRs→	117	Streptomyces ossamyceticus	GCF_001418475.1	actinobacteria
WP_055529698.1	EACC1	EACC1*→ CASPASE+PBPB→	113	Streptomyces graminilatus	GCF_001418565.1	actinobacteria
WP_055547602.1	EACC1	EACC1*→ CASPASE+TPRs→ Clp_N+Clp_N+Clp_N+Clp_N+ClpABN-AAA→	118	Streptomyces sp. NBRC 110028	GCF_001417735.1	actinobacteria
WP_055554425.1	EACC1	HATPase_c_2→ EACC1*→	118	Streptomyces sp. NBRC 110028	GCF_001417735.1	actinobacteria
WP_055555440.1	EACC1	EACC1*→ CASPASE+STAND+polar_region+TM+BetaPropeller→	150	Streptomyces sp. NBRC 110028	GCF_001417735.1	actinobacteria
WP_055564004.1	EACC1	EACC1*→ CASPASE+TPRs→	138	Streptomyces atriruber	GCF_001418325.1	actinobacteria
WP_055574191.1	EACC1	??+CASPASE→ EACC1*→	132	Streptomyces prasinopilosus	GCA_900101585.1	actinobacteria
WP_055582923.1	EACC1	EACC1*→ CASPASE+TM+TM+bPH_domain?→	141	Streptomyces	GCF_000935185.3	actinobacteria
WP_055591440.1	EACC1	??→ EACC1*→ CASPASE+TPRs→	129	Streptomyces griseoplanus	GCF_001418575.1	actinobacteria
WP_055593759.1	EACC1	EACC1*→ CASPASE+STAND+polar_region+TM+BetaPropeller→	167	Streptomyces hirsutus	GCF_001418415.1	actinobacteria
WP_055607204.1	EACC1	??→ EACC1*→ CASPASE+TPRs→	130	Streptomyces prasinus	-	actinobacteria
WP_055607684.1	EACC1	TIR+CASPASE→ EACC1*→	141	Streptomyces prasinus	GCF_001418165.1	actinobacteria
WP_055628150.1	EACC1	EACC1*→ CASPASE+TPRs→	118	Streptomyces hirsutus	GCF_001418175.1	actinobacteria
WP_055689145.1	EACC1	-	168	Streptomyces prasinus	-	actinobacteria
WP_055690018.1	EACC1	??→ EACC1*→ CASPASE+TPRs→	130	Streptomyces prasinus	GCF_001419705.1	actinobacteria
WP_055690138.1	EACC1	EACC1*→ CASPASE+??→	137	Streptomyces prasinus	GCF_001419705.1	actinobacteria

acc	architecture	operon	len	species	gca	taxend
WP_055693189.1	EACC1	??+CASPASE→ EACC1*→	132	Streptomyces prasinopilosus	GCF_001419695.1	actinobacteria
WP_055698908.1	EACC1	low-complexity+HD+Tox-RelA+TGS+ACT→ Pkinase+TPRs→ EACC1*→ CASPASE+TPRs→	138	Streptomyces	GCF_001748305.1	actinobacteria
WP_055704881.1	EACC1	EACC1*→ CASPASE+??+SF1-helicase+Vsr(PDDEXK)?→	126	Streptomyces puniciscabiei	-	actinobacteria
WP_055721366.1	EACC1	EACC1*→ CASPASE+TM+TM+PBPB+vWA→	105	Streptomyces	GCA_003129505.1	actinobacteria
WP_055751765.1	EACC1+EAD1	FHA+??+low-complexity+FHA+ABC-ATPase+TM→ EACC1+EAD1*→||←CASPASE+STAND+polar_region+TM+BetaPropeller	233	Frankia sp. AvcI1	GCF_001420875.1	actinobacteria
WP_056700872.1	EACC1	EACC1*→ CASPASE+STAND+??+TPRs+??→	119	Streptomyces	GCA_001426325.1	actinobacteria
WP_056780488.1	EACC1	EACC1*→ CASPASE+??+TM+TM+TM+TM+TM+TM+TM+TM→	124	Terrabacter sp. Soil811	GCA_001429655.1	actinobacteria
WP_056790650.1	EACC1	??+CASPASE→ EACC1*→	127	Streptomyces	GCA_001426615.1	actinobacteria
WP_056799763.1	EACC1	EACC1*→ CASPASE+STAND+polar_region+TM+BetaPropeller→	139	Streptomyces sp. Root55	GCA_001427245.1	actinobacteria
WP_056823413.1	EACC1	EACC1*→ CASPASE+??+TM+TM+TM+TM+TM+TM+TM+TM→	124	Terrabacter sp. Root85	GCA_001429185.1	actinobacteria
WP_057235967.1	EACC1	EACC1*→ CASPASE+TPRs→	135	Kitasatospora sp. Root107	GCA_001424875.1	actinobacteria
WP_057238329.1	EACC1	EACC1*→ CASPASE+TPRs→	131	Kitasatospora	GCA_001424875.1	actinobacteria
WP_057240280.1	EACC1	polar_lead?+TM+TM+TM+polar_tail?+low-complexity→??→ EACC1*→ CASPASE+TPRs→	117	Kitasatospora	GCA_001424875.1	actinobacteria
WP_058081073.1	EACC1	EACC1*→ CASPASE+TM?+BetaPropeller→ MDMPI_N+MDMPI_C→ META→	116	Streptomyces hygroscopicus	GCA_001447075.1	actinobacteria
WP_058084346.1	EACC1	EACC1*→ CASPASE+low-complexity+TM+PBPB→	140	Streptomyces hygroscopicus	GCA_001447075.1	actinobacteria
WP_058228238.1	EACC1	EACC1*→ CASPASE+??→	122	Rhodococcus enclensis	GCA_900094765.1	actinobacteria
WP_059065682.1	EACC1	EACC1*→	82	Klebsiella pneumoniae	GCA_002903025.1	gammaproteobacteria
WP_059144748.1	EACC1	TetR_N+TetR_C_6→ EACC1*→	130	Streptomyces	GCA_001509775.1	actinobacteria
WP_059145485.1	EACC1	EACC1*→	133	Streptomyces	GCA_001509775.1	actinobacteria
WP_059193393.1	EACC1	EACC1*→ CASPASE+TM+TM+TM+TM+TM+TM→	136	Streptomyces antibioticus	GCA_001514065.1	actinobacteria
WP_059193417.1	EACC1	EACC1*→ CASPASE+STAND+BetaPropeller→	125	Streptomyces antibioticus	GCA_001514065.1	actinobacteria
WP_059195108.1	EACC1	EACC1*→ CASPASE+STAND+polar_region+TM+BetaPropeller→	133	Streptomyces antibioticus	GCA_001514065.1	actinobacteria
WP_059195220.1	EACC1	EACC1*→ CASPASE+TPRs→	118	Streptomyces antibioticus	GCA_001514065.1	actinobacteria
WP_059249456.1	EACC1	-	133	Streptomyces reticuli	-	actinobacteria
WP_059253791.1	EACC1	AMP-binding+AMP-binding_C→ AMP-binding+AMP-binding_C→ EACC1*→ CASPASE+STAND+polar_region+TM+BetaPropeller→	152	Streptomyces	GCA_002251155.1	actinobacteria
WP_059263747.1	EACC1	EACC1*→ CASPASE+TM?+BetaPropeller→ MDMPI_N+MDMPI_C→ META→	116	Streptomyces corchorusii	GCA_001514055.1	actinobacteria
WP_059265627.1	EACC1	EACC1*→ CASPASE+low-complexity+TM+PBPB→	140	Streptomyces corchorusii	GCA_001514055.1	actinobacteria
WP_059267207.1	EACC1	EACC1*→ CASPASE+TPRs→	119	Streptomyces corchorusii	GCA_001514055.1	actinobacteria
WP_060888019.1	EACC1	EACC1*→ CASPASE+TM+TM+bPH_domain+TM+TM+bPH_domain+TM+TM→	134	Streptomyces	GCA_900114955.1	actinobacteria
WP_060904615.1	EACC1	EACC1*→ CASPASE+TPRs→	127	Streptomyces scabiei	GCF_001550235.1	actinobacteria
WP_061446668.1	EACC1	-	153	Streptomyces sp. CCM_MD2014	-	actinobacteria
WP_061446671.1	EACC1	EACC1*→ CASPASE+TPRs→	119	Streptomyces sp. CCM_MD2014	GCA_000772045.1	actinobacteria
WP_061989687.1	EACC1	EACC1*→	120	Amycolatopsis regifaucium	GCA_001613935.1	actinobacteria
WP_062133880.1	EACC1	EACC1*→ CASPASE(fragment)→	117	Streptomyces sp. CC71	GCA_001595515.1	actinobacteria
WP_062205207.1	EACC1	-	186	Streptomyces sp. NBRC 109706	-	actinobacteria
WP_062208870.1	EACC1	TM+TM+TM+PP2C→ EACC1*→ CASPASE+PBPB+vWA→	135	Streptomyces sp. NBRC 109706	GCF_000974485.1	actinobacteria
WP_062331945.1	EACC1	EACC1*→	119	Herbidospora sakaeratensis	GCF_001570525.1	actinobacteria
WP_062707009.1	EACC1	TPRs→ EACC1*→ CASPASE+TPRs→	114	Streptomyces regalis	GCA_001509475.1	actinobacteria
WP_062720803.1	EACC1	-	135	Streptomyces caeruleatus	GCA_001514235.1	actinobacteria
WP_062752525.1	EACC1	EACC1*→ CASPASE+STAND+polar_region+TM+BetaPropeller→	181	Streptomyces	GCA_002943895.1	actinobacteria
WP_062924605.1	EACC1	EACC1*→ CASPASE+RE_Eco29kI+??→	124	Streptomyces sp. S10(2016)	GCA_001611795.1	actinobacteria
WP_062928059.1	EACC1	TM+TM+TM+PP2C→??→ EACC1*→ CASPASE+TM+TM+PBPB+vWA→	121	Streptomyces sp. S10(2016)	GCA_001611795.1	actinobacteria
WP_062976308.1	EACC1	EACC1*→ CASPASE+TPRs→	147	Nocardia flavorosea	GCF_001613385.1	actinobacteria
WP_062989910.1	EACC1	EACC1*→ CASPASE+TPRs→	117	Nocardia anaemiae	GCF_001612725.1	actinobacteria
WP_063006009.1	EACC1	DUF2510(W3)+BicD+DUF4041+T5orf172→ EACC1→ CASPASE+TPRs→ EACC1*→ CASPASE+EAL→	128	Nocardia cummidelens	GCF_001612865.1	actinobacteria
WP_063006011.1	EACC1	DUF2510(W3)+BicD+DUF4041+T5orf172→ EACC1*→ CASPASE+TPRs→ EACC1→ CASPASE+EAL→	125	Nocardia cummidelens	-	actinobacteria
WP_063038312.1	EACC1	EACC1*→ CASPASE+STAND+polar_region+TM+BetaPropeller→	125	Nocardia pseudovaccinii	GCF_001613225.1	actinobacteria
WP_063038929.1	EACC1	EACC1*→ CASPASE+TPRs→	114	Nocardia grenadensis	GCF_001613445.1	actinobacteria
WP_063044051.1	EACC1	EACC1*→ CASPASE+TPRs→	169	Nocardia pseudovaccinii	GCF_001613225.1	actinobacteria
WP_063047902.1	EACC1	EACC1*→ CASPASE+TPRs→	138	Nocardia pseudovaccinii	GCF_001613225.1	actinobacteria
WP_063057431.1	EACC1	EACC1*→ CASPASE+??+SF1-helicase+Vsr(PDDEXK)?→	138	Nocardia sienata	GCF_001613205.1	actinobacteria
WP_063273430.1	EACC1	EACC1*→	101	Amycolatopsis keratiniphila	-	actinobacteria

acc	architecture	operon	len	species	gca	taxend
WP_063276304.1	EACC1	MFS_1→ EACC1*→ CASPASE+DnaJ_ZnR+DnaJ_ZnR+DnaJ_C→	122	Amycolatopsis keratiniphila	-	actinobacteria
WP_063483685.1	EACC1	EACC1*→ CASPASE+GTP_EFTU+GTP_EFTU_D2+GTP_EFTU_D3→	127	Streptomyces ambofaciens	GCA_001632865.1	actinobacteria
WP_063792803.1	EACC1	EACC1*→	127	Streptomyces aurantiacus	GCF_001418335.1	actinobacteria
WP_063837497.1	EACC1	EACC1*→	98	Streptomyces sp. NRRL WC-3549	GCF_000721935.1	actinobacteria
WP_064068784.1	EACC1	HATPase_c_2→ EACC1*→ CASPASE+??+SF1-helicase+Vsr(PDDEXK)?→	141	Streptomyces albulus	GCA_001646665.1	actinobacteria
WP_064534223.1	EACC1	??→ EACC1*→ CASPASE+TM+TM+PBPB+vWA→	114	Streptomyces sp. SAT1	GCA_001654495.1	actinobacteria
WP_065003909.1	EACC1	??→ EACC1*→ CASPASE+TPRs→	130	Streptomyces sp. H-KF8	GCA_001672315.1	actinobacteria
WP_065479927.1	EACC1	EACC1*→ CASPASE+??+SF1-helicase+Vsr(PDDEXK)?→??→	143	Streptomyces sp. PTY087I2	GCA_001687325.1	actinobacteria
WP_065484169.1	EACC1	EACC1*→ CASPASE+TPRs→	118	Streptomyces sp. PTY087I2	GCF_001687325.1	actinobacteria
WP_065807622.1	EACC1	EACC1*→	93	Enterobacteriaceae	GCA_002844785.1	gammaproteobacteria
WP_066361324.1	EACC1	EACC1*→	81	Herbidospora mongoliensis	GCF_001570545.1	actinobacteria
WP_066890950.1	EACC1	-	131	Streptomyces thermoautotrophicus	-	actinobacteria
WP_066927619.1	EACC1	EACC1*→ CASPASE+TPRs→	114	Streptomyces sp. NBRC 110611	GCA_001590865.1	actinobacteria
WP_066927855.1	EACC1	-	138	Streptomyces sp. NBRC 110611	GCA_001590865.1	actinobacteria
WP_066954369.1	EACC1	EACC1*→	200	Streptomyces lushanensis	GCF_001700515.1	actinobacteria
WP_066956024.1	EACC1	Pkinase+TPRs→ EACC1*→ CASPASE+TPRs→	136	Streptomyces lushanensis	GCF_001700515.1	actinobacteria
WP_066985804.1	EACC1	low-complexity+HD+Tox-RelA+TGS+ACT→ Pkinase+TPRs→ EACC1*→ CASPASE+TPRs→	148	Streptomyces sp. NRRL F-4489	GCA_001509485.1	actinobacteria
WP_067008945.1	EACC1	EACC1*→ CASPASE+PBPB→	152	Streptomyces cellostaticus	GCA_001513965.1	actinobacteria
WP_067118993.1	EACC1	EACC1*→ CASPASE+TPRs→	114	Streptomyces yokosukanensis	GCA_001514035.1	actinobacteria
WP_067155823.1	EACC1	low-complexity+HD+Tox-RelA+TGS+ACT→ Pkinase+TPRs→ EACC1*→ CASPASE+TPRs→	125	Streptomyces sp. ERV7	GCA_001653515.1	actinobacteria
WP_067166979.1	EACC1	EACC1*→ CASPASE+BetaPropeller→||←DUF664	126	Streptomyces sp. ERV7	GCA_001653515.1	actinobacteria
WP_067237622.1	EACC1	EACC1*→ CASPASE+STAND+??→	119	Streptomyces longwoodensis	GCA_001514125.1	actinobacteria
WP_067253610.1	EACC1	EACC1*→ CASPASE+low-complexity+BetaPropeller→	108	Streptomyces sp. DSM 15324	GCA_001514285.1	actinobacteria
WP_067303920.1	EACC1	EACC1*→ CASPASE+wHTH+BetaPropeller→	127	Streptomyces griseochromogenes	GCA_001542625.2	actinobacteria
WP_067304265.1	EACC1	EACC1*→ CASPASE+TM+TM→ PBPB→	145	Micromonospora rifamycinica	GCA_001542325.1	actinobacteria
WP_067309664.1	EACC1	EACC1*→ CASPASE+low-complexity+BetaPropeller→	118	Streptomyces griseochromogenes	GCA_001542625.2	actinobacteria
WP_067312463.1	EACC1	low-complexity+HD+Tox-RelA+TGS+ACT→ Pkinase+TPRs→ EACC1*→ CASPASE+TPRs→	125	Streptomyces griseochromogenes	GCA_001542625.2	actinobacteria
WP_067315441.1	EACC1	TM+TM+TM+Peptidase_M48+TM+DUF4864+??→ EACC1*→	155	Micromonospora rifamycinica	GCA_001542325.1	actinobacteria
WP_067353812.1	EACC1	low-complexity+HD+Tox-RelA+TGS+ACT→ Pkinase+TPRs→ EACC1*→ CASPASE+TPRs→	131	Streptomyces noursei	GCA_001704275.1	actinobacteria
WP_067470360.1	low_complexity+EACC1	low_complexity+EACC1*→	315	Nocardia amamiensis	GCF_001612745.1	actinobacteria
WP_067487973.1	EACC1	EACC1*→ CASPASE+EAL→	122	Nocardia ignorata	GCA_004362495.1	actinobacteria
WP_067499062.1	EACC1	EACC1*→ CASPASE+SF1-helicase→	126	Actinoplanes sp. TFC3	GCF_001553785.1	actinobacteria
WP_067529228.1	EACC1	EACC1*→ CASPASE+??→	128	Nocardia uniformis	GCF_001613345.1	actinobacteria
WP_067578379.1	EACC1	GATase→ ABC-ATPase+DUF4162→ cNMP_cyclase+TM+TM+TM+TM+TM+TM→ EACC1*→	109	Amycolatopsis	GCA_001651785.1	actinobacteria
WP_067670342.1	EACC1	EACC1*→	80	Nocardia miyunensis	GCF_001613065.1	actinobacteria
WP_067690701.1	EACC1	EACC1*→ CASPASE+TPRs→	122	Nocardia jejuensis	GCF_001613145.1	actinobacteria
WP_067694339.1	EACC1	EACC1*→ CASPASE+STAND+BetaPropeller→	156	Nocardia jejuensis	GCF_001613145.1	actinobacteria
WP_067792914.1	EACC1	EACC1*→ CASPASE+TPRs→	75	Nocardia amikacinitolerans	GCF_001612615.1	actinobacteria
WP_067804861.1	EACC1	Pkinase+TM+LppX_LprAFG→ CASPASE+TPRs→ EACC1*→	128	Nocardia beijingensis	GCF_001612785.1	actinobacteria
WP_067806049.1	EACC1	EACC1*→ CASPASE+STAND+polar_region+TM+BetaPropeller→	129	Nocardia beijingensis	GCF_001612785.1	actinobacteria
WP_067827015.1	EACC1	EACC1*→ CASPASE+??→	136	Nocardia inohanensis	GCF_001612945.1	actinobacteria
WP_067915216.1	EACC1	EACC1*→ CASPASE+??+NUDIX→	126	Actinomadura	GCF_001552235.1	actinobacteria
WP_067978771.1	EACC1	EACC1*→ CASPASE+TM+TM+TM+TM+TM+TM+TM+TM→	75	Nocardia caishijiensis	GCF_001612825.1	actinobacteria
WP_068032713.1	EACC1	EACC1*→	157	Nocardia mexicana	GCF_001613165.1	actinobacteria
WP_068902202.1	EACC1	EACC1*→ CASPASE+SAD+HNH→	120	Planomonospora sphaerica	GCF_001653075.1	actinobacteria
WP_068904454.1	EACC1	EACC1*→ CASPASE+STAND+polar_region+TM+BetaPropeller→	119	Planomonospora sphaerica	GCF_001653075.1	actinobacteria
WP_068927089.1	EACC1	EACC1*→	90	Planobispora rosea	GCF_001696485.1	actinobacteria
WP_068928249.1	EACC1	EACC1*→ CASPASE+TM+TM+TM+TM+TM+TM+BetaPropeller→	129	Planobispora rosea	GCF_001696485.1	actinobacteria
WP_069571402.1	EACC1	Glyoxalase→ EACC1*→ CASPASE+DnaJ_ZnR+DnaJ_ZnR+DnaJ_C→	135	Streptomyces lydicus	GCA_001729485.1	actinobacteria
WP_069571866.1	EACC1	EACC1*→ CASPASE+??→	119	Streptomyces lydicus	GCA_001729485.1	actinobacteria
WP_069628874.1	EACC1	EACC1*→?→ PALP→	120	Streptomyces niveus	GCA_004563805.1	actinobacteria
WP_069734965.1	EACC1	EACC1*→ CASPASE+TPRs→	118	Streptomyces sp. EN27	GCA_003353555.1	actinobacteria

<table>
<tr><th>acc</th><th>architecture</th><th>operon</th><th>len</th><th>species</th><th>gca</th><th>taxend</th></tr>
<tr><td>WP_069755251.1</td><td>EACC1</td><td>EACC1*→ CASPASE+STAND+??+TPRs+??→</td><td>119</td><td>Streptomyces sp. EN16</td><td>GCF_001746285.1</td><td>actinobacteria</td></tr>
<tr><td>WP_069756375.1</td><td>EACC1</td><td>EACC1*→ CASPASE+Beta_rich_repeat→??+SUKH→</td><td>137</td><td>Streptomyces sp. EN16</td><td>GCF_001746285.1</td><td>actinobacteria</td></tr>
<tr><td>WP_069767498.1</td><td>EACC1</td><td>EACC1*→ CASPASE+??+SF1-helicase+Vsr(PDDEXK)?→</td><td>126</td><td>Streptomyces sp. LUP30</td><td>GCF_001746365.1</td><td>actinobacteria</td></tr>
<tr><td>WP_069768493.1</td><td>EACC1</td><td>-</td><td>119</td><td>Streptomyces sp. LUP30</td><td>-</td><td>actinobacteria</td></tr>
<tr><td>WP_069776394.1</td><td>EACC1</td><td>EACC1*→ CASPASE+Pkinase+TM?+??→</td><td>138</td><td>Streptomyces puniciscabiei</td><td>GCA_001735805.1</td><td>actinobacteria</td></tr>
<tr><td>WP_069864785.1</td><td>EACC1</td><td>TetR_N+TetR_C_6→ EACC1*→ CASPASE+low-complexity+TM+PBPB→</td><td>140</td><td>Streptomyces sp. SPMA113</td><td>-</td><td>actinobacteria</td></tr>
<tr><td>WP_069865414.1</td><td>EACC1</td><td>EACC1*→ CASPASE+TM+??→</td><td>122</td><td>Streptomyces</td><td>GCA_002812405.1</td><td>actinobacteria</td></tr>
<tr><td>WP_070017995.1</td><td>EACC1</td><td>EACC1*→ CASPASE+STAND+??+TPRs→</td><td>132</td><td>Streptomyces nanshensis</td><td>GCA_001751255.1</td><td>actinobacteria</td></tr>
<tr><td>WP_070025777.1</td><td>EACC1</td><td>EACC1*→ CASPASE+??+ZnR+ZnR→</td><td>119</td><td>Streptomyces</td><td>GCA_900104815.1</td><td>actinobacteria</td></tr>
<tr><td>WP_070029270.1</td><td>EACC1</td><td>EACC1*→ CASPASE+STAND+polar_region+TM+BetaPropeller→</td><td>130</td><td>Streptomyces sp. F-1</td><td>GCA_900079405.3</td><td>actinobacteria</td></tr>
<tr><td>WP_070029920.1</td><td>EACC1</td><td>EACC1*→ CASPASE+STAND+polar_region+TM+BetaPropeller→</td><td>162</td><td>Streptomyces sp. F-1</td><td>GCA_900079405.3</td><td>actinobacteria</td></tr>
<tr><td>WP_070205154.1</td><td>EACC1</td><td>EACC1*→ CASPASE+TPRs→</td><td>119</td><td>Streptomyces</td><td>GCA_001751305.1</td><td>actinobacteria</td></tr>
<tr><td>WP_071284407.1</td><td>EACC1</td><td>EACC1*→</td><td>118</td><td>Enterobacter cloacae complex ‘Hoffmann cluster IV’</td><td>GCA_001858135.1</td><td>gammaproteobacteria</td></tr>
<tr><td>WP_071366246.1</td><td>EACC1</td><td>EACC1*→ CASPASE+??+TM+TM+??→</td><td>139</td><td>Streptomyces sp. MUSC 93</td><td>GCA_001866645.1</td><td>actinobacteria</td></tr>
<tr><td>WP_071386200.1</td><td>EACC1</td><td>Pkinase+TPRs→ EACC1*→ CASPASE+TPRs→</td><td>135</td><td>Streptomyces sp. MUSC 1</td><td>GCA_001866665.1</td><td>actinobacteria</td></tr>
<tr><td>WP_071655689.1</td><td>EACC1</td><td>DUF742→?→?→||←?←?||EACC1*→?→ CASPASE+PBPB+vWA→?→||←?||MFS_1→</td><td>156</td><td>Streptomyces gilvigriseus</td><td>GCA_001879105.1</td><td>actinobacteria</td></tr>
<tr><td>WP_071806911.1</td><td>EACC1</td><td>EACC1*→ CASPASE+DUF179→ SIR2+??→??→</td><td>117</td><td>Couchioplanes caeruleus</td><td>GCA_001884705.1</td><td>actinobacteria</td></tr>
<tr><td>WP_071967634.1</td><td>EACC1</td><td>EACC1*→ CASPASE+TM+TM+PBPB+vWA→</td><td>103</td><td>Streptomyces cinnamoneus</td><td>GCF_001885705.1</td><td>actinobacteria</td></tr>
<tr><td>WP_072475297.1</td><td>EACC1</td><td>MFS_1→||←?||GATase→ ABC-ATPase+DUF4162→ cNMP_cyclase+TM+TM+TM+TM+TM+TM→ EACC1→ CASPASE+TM+TM+TM+TM→ EACC1*→</td><td>105</td><td>Amycolatopsis australiensis</td><td>GCA_900119165.1</td><td>actinobacteria</td></tr>
<tr><td>WP_072475299.1</td><td>EACC1</td><td>MFS_1→||←?||GATase→ ABC-ATPase+DUF4162→ cNMP_cyclase+TM+TM+TM+TM+TM+TM→ EACC1*→ CASPASE+TM+TM+TM+TM→ EACC1→</td><td>109</td><td>Amycolatopsis australiensis</td><td>GCF_900119165.1</td><td>actinobacteria</td></tr>
<tr><td>WP_072480727.1</td><td>EACC1</td><td>EACC1*→ CASPASE+TPRs→</td><td>116</td><td>Amycolatopsis australiensis</td><td>GCA_900119165.1</td><td>actinobacteria</td></tr>
<tr><td>WP_072656118.1</td><td>EACC1</td><td>EACC1*→</td><td>151</td><td>Streptomyces viridifaciens</td><td>GCA_001888885.1</td><td>actinobacteria</td></tr>
<tr><td>WP_073215496.1</td><td>EACC1</td><td>Beta_rich?→ EACC1*→ CASPASE+TPRs→??→??→</td><td>105</td><td>Streptomyces sp. NBRC 110465</td><td>GCF_001895105.1</td><td>actinobacteria</td></tr>
<tr><td>WP_073384278.1</td><td>EACC1</td><td>EACC1*→ CASPASE+STAND+polar_region+TM+BetaPropeller→</td><td>140</td><td>Nocardiopsis flavescens</td><td>GCA_900141985.1</td><td>actinobacteria</td></tr>
<tr><td>WP_073444062.1</td><td>EACC1</td><td>polar_lead?+TM+TM+TM+polar_tail?+low-complexity→??→ EACC1*→ CASPASE+TPRs→</td><td>126</td><td>Streptomyces yunnanensis</td><td>GCA_900142595.1</td><td>actinobacteria</td></tr>
<tr><td>WP_073445437.1</td><td>EACC1</td><td>low-complexity+HD+Tox-RelA+TGS+ACT→ Pkinase+TPRs→ EACC1*→ CASPASE+TPRs→</td><td>131</td><td>Streptomyces yunnanensis</td><td>GCA_900142595.1</td><td>actinobacteria</td></tr>
<tr><td>WP_073500553.1</td><td>EACC1</td><td>EACC1*→ CASPASE+STAND+polar_region+TM+BetaPropeller→</td><td>146</td><td>Streptomyces paucisporeus</td><td>GCF_900142575.1</td><td>actinobacteria</td></tr>
<tr><td>WP_073500803.1</td><td>EACC1</td><td>EACC1*→ CASPASE+HSP70→</td><td>119</td><td>Streptomyces paucisporeus</td><td>GCF_900142575.1</td><td>actinobacteria</td></tr>
<tr><td>WP_073719518.1</td><td>EACC1</td><td>EACC1*→ CASPASE+??+SF1-helicase+Vsr(PDDEXK)?→</td><td>137</td><td>Streptomyces sp. TSRI0281</td><td>GCA_001905125.1</td><td>actinobacteria</td></tr>
<tr><td>WP_073727530.1</td><td>EACC1</td><td>EACC1*→ CASPASE+TPRs→</td><td>118</td><td>Streptomyces sp. CB02414</td><td>GCA_001905385.1</td><td>actinobacteria</td></tr>
<tr><td>WP_073729973.1</td><td>EACC1</td><td>EACC1*→ CASPASE+STAND+polar_region+TM+BetaPropeller→</td><td>173</td><td>Streptomyces sp. CB02414</td><td>GCF_001905385.1</td><td>actinobacteria</td></tr>
<tr><td>WP_073748875.1</td><td>EACC1</td><td>EACC1*→ CASPASE+??→</td><td>119</td><td>Streptomyces sp. CB02058</td><td>GCA_001905505.1</td><td>actinobacteria</td></tr>
<tr><td>WP_073752659.1</td><td>EACC1</td><td>-</td><td>141</td><td>Streptomyces sp. CB02058</td><td>-</td><td>actinobacteria</td></tr>
<tr><td>WP_073753325.1</td><td>EACC1</td><td>EACC1*→ CASPASE+STAND+??+TPRs→</td><td>123</td><td>Streptomyces sp. CB02058</td><td>GCA_001905505.1</td><td>actinobacteria</td></tr>
<tr><td>WP_073760381.1</td><td>EACC1</td><td>EACC1*→</td><td>118</td><td>Streptomyces sp. CB03234</td><td>GCA_001905885.1</td><td>actinobacteria</td></tr>
<tr><td>WP_073762288.1</td><td>EACC1</td><td>SF2-helicase+RecQ_Zn_bind+RQC+HRDC→ EACC1*→</td><td>147</td><td>Streptomyces sp. CB02923</td><td>GCA_001905005.1</td><td>actinobacteria</td></tr>
<tr><td>WP_073781011.1</td><td>EACC1</td><td>EACC1*→ CASPASE+STAND+polar_region+TM+BetaPropeller→</td><td>154</td><td>Streptomyces sp. MJM1172</td><td>GCA_001905425.1</td><td>actinobacteria</td></tr>
<tr><td>WP_073799165.1</td><td>EACC1</td><td>EACC1*→ CASPASE+STAND+polar_region+TM+BetaPropeller→</td><td>154</td><td>Streptomyces</td><td>GCA_001905025.1</td><td>actinobacteria</td></tr>
<tr><td>WP_073800187.1</td><td>EACC1</td><td>EACC1*→ CASPASE+??+SF1-helicase+Vsr(PDDEXK)?→</td><td>138</td><td>Streptomyces sp. TSRI0261</td><td>GCA_001905485.1</td><td>actinobacteria</td></tr>
<tr><td>WP_073802404.1</td><td>EACC1</td><td>Beta_rich?→ EACC1*→ CASPASE+TPRs→??→??→</td><td>122</td><td>Streptomyces sp. TSRI0261</td><td>GCA_001905485.1</td><td>actinobacteria</td></tr>
<tr><td>WP_073803866.1</td><td>EACC1</td><td>EACC1*→ CASPASE+Beta_rich_repeat→??+SUKH→</td><td>143</td><td>Streptomyces sp. TSRI0261</td><td>GCA_001905485.1</td><td>actinobacteria</td></tr>
<tr><td>WP_073808816.1</td><td>EACC1</td><td>EACC1*→ CASPASE+TM+TM+TM+TM→</td><td>143</td><td>Kitasatospora sp. CB01950</td><td>GCA_001905465.1</td><td>actinobacteria</td></tr>
<tr><td>WP_073846533.1</td><td>EACC1</td><td>EACC1*→</td><td>101</td><td>Amycolatopsis sp. CB00013</td><td>GCA_001905755.1</td><td>actinobacteria</td></tr>
<tr><td>WP_073863288.1</td><td>EACC1</td><td>EACC1*→ CASPASE+TPRs→</td><td>121</td><td>Streptomyces sp. CB01249</td><td>GCA_001905735.1</td><td>actinobacteria</td></tr>
<tr><td>WP_073871277.1</td><td>EACC1</td><td>EACC1*→</td><td>153</td><td>Streptomyces sp. CB00072</td><td>GCA_001905575.1</td><td>actinobacteria</td></tr>
<tr><td>WP_073910694.1</td><td>EACC1</td><td>HATPase_c_2→ EACC1*→ CASPASE+??+SF1-helicase+Vsr(PDDEXK)?→</td><td>134</td><td>Streptomyces sp. CB00455</td><td>GCA_001905445.1</td><td>actinobacteria</td></tr>
<tr><td>WP_073920305.1</td><td>EACC1</td><td>low-complexity+HD+Tox-RelA+TGS+ACT→ Pkinase+TPRs→ EACC1*→ CASPASE+TPRs→</td><td>167</td><td>Streptomyces sp. CB00455</td><td>GCA_001905445.1</td><td>actinobacteria</td></tr>
<tr><td>WP_073931364.1</td><td>EACC1</td><td>EACC1*→ CASPASE+STAND+polar_region+TM+BetaPropeller→ HTH+N6_Mtase+Methylase_S→</td><td>156</td><td>Streptomyces sp. CB02400</td><td>GCF_001905725.1</td><td>actinobacteria</td></tr>
<tr><td>WP_073946764.1</td><td>EACC1</td><td>←CASPASE+HATPase_c_4+STAND+TPRs||EACC1*→</td><td>111</td><td>Streptomyces kebangsaanensis</td><td>GCF_001906585.1</td><td>actinobacteria</td></tr>
<tr><td>WP_074266816.1</td><td>EACC1</td><td>EACC1*→ CASPASE+TPRs→</td><td>116</td><td>Paraburkholderia phenazinium</td><td>GCA_900141745.1</td><td>betaproteobacteria</td></tr>
<tr><td>WP_074311352.1</td><td>EACC1</td><td>EACC1*→</td><td>93</td><td>Micromonospora cremea</td><td>GCF_900143515.1</td><td>actinobacteria</td></tr>
</table>

acc	architecture	operon	len	species	gca	taxend
WP_074474396.1	EACC1	TM+TM+TM+Peptidase_M48+TM+DUF4864+??→ EACC1*→	159	Micromonospora carbonacea	-	actinobacteria
WP_074475907.1	EACC1	EACC1*→ CASPASE+STAND+polar_region+TM+BetaPropeller→	130	Micromonospora carbonacea	GCA_900091535.1	actinobacteria
WP_074990165.1	EACC1	EACC1*→ CASPASE+STAND+polar_region+TM+BetaPropeller→	162	Streptomyces misionensis	GCA_900104815.1	actinobacteria
WP_074990255.1	EACC1	EACC1*→ CASPASE+STAND+polar_region+TM+BetaPropeller→	130	Streptomyces misionensis	GCA_900104815.1	actinobacteria
WP_075136975.1	EACC1	EACC1*→ CASPASE+TM+TM→ TM+PBPB→	129	Actinophytocola xinjiangensis	GCA_001921215.1	actinobacteria
WP_075138181.1	EACC1	Beta_rich?→ EACC1*→ CASPASE+TPRs→??→??→	123	Actinophytocola xinjiangensis	GCA_001921215.1	actinobacteria
WP_075733352.1	EACC1	EACC1*→ CASPASE+TM+TM+PBPB+vWA→	111	Streptomyces acidiscabies	GCF_001941275.1	actinobacteria
WP_075735850.1	EACC1	EACC1*→ CASPASE+TPRs→	112	Streptomyces acidiscabies	GCF_001941275.1	actinobacteria
WP_075740780.1	EACC1	EACC1*→ CASPASE+??→	131	Actinoalloteichus	GCA_001941625.1	actinobacteria
WP_075976363.1	EACC1	EACC1*→ CASPASE+GTP_EFTU+GTP_EFTU_D2→	126	Actinokineospora bangkokensis	GCA_001940455.1	actinobacteria
WP_076086048.1	EACC1	EACC1*→ CASPASE+TM+TM+PBPB+vWA→	120	Streptomyces sp. IMTB 2501	GCA_001953885.1	actinobacteria
WP_076166549.1	EACC1	EACC1*→	110	Amycolatopsis coloradensis	GCA_001953865.1	actinobacteria
WP_076818503.1	EACC1	EACC1*→ CASPASE+STAND+polar_region+TM+BetaPropeller→	156	Frankia asymbiotica	GCA_001983105.1	actinobacteria
WP_076978205.1	EACC1	EACC1*→ CASPASE+TPRs→	119	Streptomyces sp. M1013	GCA_001970135.1	actinobacteria
WP_076986698.1	EACC1	EACC1*→	132	Actinosynnema sp. ALI-1.44	GCA_001984155.1	actinobacteria
WP_076994204.1	EACC1	PALP→ Trp_syntA→ TM+LGT→ EACC1*→	117	Actinosynnema sp. ALI-1.44	GCA_001984155.1	actinobacteria
WP_077059651.1	EACC1	EACC1*→ CASPASE+JAB→?→ MFS_1→	128	Streptomyces sp. MP131-18	GCA_001984575.1	actinobacteria
WP_077939109.1	EACC1	??+TM+TM+TM+Peptidase_M48+TM+DUF4864+??→?→ EACC1*→	75	Micromonospora sp. Rc5	GCA_002006875.1	actinobacteria
WP_077964689.1	EACC1	low-complexity+HD+Tox-RelA+TGS+ACT→ Pkinase+TPRs→ EACC1*→ CASPASE+TPRs→	158	Streptomyces tsukubensis	GCA_002007125.1	actinobacteria
WP_077965477.1	EACC1	EACC1*→ CASPASE+TPRs→ Pkinase+??→	141	Streptomyces tsukubensis	GCA_002007125.1	actinobacteria
WP_078630203.1	EACC1	EACC1*→ CASPASE+TPRs→	120	Streptomyces roseochromogenus	GCF_000497445.1	actinobacteria
WP_078644979.1	EACC1	EACC1*→ CASPASE+STAND+polar_region+TM+BetaPropeller→	147	Streptomyces hygroscopicus	GCA_002021875.1	actinobacteria
WP_078884333.1	EACC1	EACC1*→ CASPASE+TM+TM+PBPB+vWA→	114	Streptomyces sp. NRRL S-340	GCF_000720185.1	actinobacteria
WP_078974305.1	EACC1	EACC1*→	123	Streptomyces scabrisporus	GCA_002024165.1	actinobacteria
WP_079076561.1	EACC1	EACC1*→ CASPASE+TPRs→	116	Streptomyces	GCF_001572115.1	actinobacteria
WP_079129943.1	EACC1	EACC1*→ CASPASE+TM+TM+PBPB+vWA→	114	Streptomyces sp. F-3	GCA_001653115.1	actinobacteria
WP_079136578.1	EACC1	-	117	Streptomyces sp. MJM8645	-	actinobacteria
WP_079151662.1	EACC1	EACC1*→ CASPASE+low-complexity+TM+PBPB→	133	Streptomyces sp. RTd22	GCF_001650215.1	actinobacteria
WP_079161065.1	EACC1	EACC1*→ CASPASE+STAND+polar_region+TM+BetaPropeller→	144	Streptomyces puniciscabiei	GCF_001735805.1	actinobacteria
WP_079172843.1	EACC1	EACC1*→	129	Streptomyces sp. MUSC 1	GCF_001866665.1	actinobacteria
WP_079178867.1	EACC1	EACC1*→ CASPASE+TPRs→	122	Streptomyces humi	GCF_001005085.2	actinobacteria
WP_079182070.1	EACC1	EACC1*→ CASPASE+TM+MFS_1+TM+TM+TM+TM+TM+TM+TM+TM→	90	Streptomyces yunnanensis	GCF_900142595.1	actinobacteria
WP_079186679.1	EACC1	-	130	Streptomyces sp. TSRI0107	-	actinobacteria
WP_079189196.1	EACC1	??→ EACC1*→ CASPASE+TM+TM+PBPB+vWA→	114	Streptomyces kebangsaanensis	GCF_001906585.1	actinobacteria
WP_079193145.1	EACC1	MglB→ DUF742→ sGTP-MglA→?→?→ EACC1*→ CASPASE+TPRs→||←?||?→?→ DUF664→	118	Streptomyces sp. CB02488	GCF_001905365.1	actinobacteria
WP_079258527.1	EACC1	EACC1*→ CASPASE+TM+??→	122	Streptomyces autolyticus	GCA_001983975.1	actinobacteria
WP_079273211.1	EACC1	EACC1*→ CASPASE+RE_Eco29kI+??→	119	Streptomyces sp. TN58	GCF_001941845.1	actinobacteria
WP_079277600.1	EACC1	FAD_binding_4+??+BBE→ EACC1*→ CASPASE+STAND+polar_region+TM+BetaPropeller→	125	Streptomyces sp. CB03234	GCF_001905885.1	actinobacteria
WP_079310450.1	EACC1	EACC1*→	120	Streptomyces sp. GKU 895	GCF_002027195.1	actinobacteria
WP_079314406.1	EACC1+CASPASE+TPR_region	EACC1+CASPASE+TPR_region*→	511	Microbispora sp. GKU 823	GCA_002027145.1	actinobacteria
WP_079565090.1	EACC1	EACC1*→ CASPASE+??+TM→	124	Bradyrhizobium erythrophlei	GCA_900129505.1	alphaproteobacteria
WP_079659971.1	EACC1	EACC1*→ CASPASE+??→	137	Streptomyces	GCA_002754515.1	actinobacteria
WP_080042215.1	EACC1	ARSR-HTH+RHOD-CDC25→??→??→ EACC1*→	126	Nonomuraea sp. ATCC 55076	GCA_002057455.1	actinobacteria
WP_081238712.1	EACC1	EACC1*→ CASPASE+TPRs→??→??→	129	Streptomyces viridosporus	GCF_002078235.1	actinobacteria
WP_081546165.1	EACC1	EACC1*→ CASPASE+STAND+polar_region+TM+BetaPropeller→	144	Streptomyces sp. B9173	-	actinobacteria
WP_081550423.1	EACC1	EACC1*→ CASPASE+TPRs→	148	Streptomyces sp. B9173	-	actinobacteria
WP_082310112.1	EACC1	EACC1*→	113	Nonomuraea sp. SBT364	GCF_001083785.1	actinobacteria
WP_082411107.1	EACC1	HATPase_c_2→ EACC1*→ CASPASE+??+SF1-helicase+Vsr(PDDEXK)?→	130	Actinobacteria bacterium OV450	GCA_001298555.1	actinobacteria
WP_082417613.1	low_complexity+EACC1	low_complexity+EACC1*→ CASPASE+STAND+polar_region+TM+BetaPropeller→	255	Actinobacteria bacterium OK006	GCF_001298565.1	actinobacteria
WP_082553406.1	EACC1	EACC1*→ CASPASE+??+TM+TM+TM+TM+TM+TM+TM+TM+TM+TM+TM→	131	Phycicoccus	GCF_001426245.1	actinobacteria
WP_082857637.1	EACC1	EACC1*→	110	Amycolatopsis regifaucium	GCA_900113515.1	actinobacteria
WP_083103658.1	EACC1	EACC1*→ CASPASE+low-complexity+TM+PBPB→	152	Streptomyces gilvosporeus	GCA_002082195.1	actinobacteria

acc	architecture	operon	len	species	gca	taxend
WP_083108843.1	EACC1	PAS+GAF+PP2C+HATPase_c_2→ EACC1*→ CASPASE+TM+Beta_rich→	133	Streptomyces gilvosporeus	GCA_002082195.1	actinobacteria
WP_083971536.1	EACC1	EACC1*→ CASPASE+??→	125	Actinoplanes awajinensis	GCF_001509495.1	actinobacteria
WP_083976228.1	cNMP_cyclase+EACC1	cNMP_cyclase+EACC1*→ CASPASE+TPRs→	608	Kitasatospora azatica	GCF_000744785.1	actinobacteria
WP_083979002.1	EACC1	EACC1*→ CASPASE+TM+TM+TM+TM+TM+TM→	99	Micromonospora rosaria	GCA_001567585.1	actinobacteria
WP_084531937.1	EACC1	EACC1*→ CASPASE+STAND+polar_region+TM+BetaPropeller→	137	Nocardia miyunensis	GCF_001613065.1	actinobacteria
WP_084747233.1	EACC1	EACC1*→ CASPASE+??+SF1-helicase+Vsr(PDDEXK)?→	138	Kitasatospora albolonga	GCA_002082585.1	actinobacteria
WP_084759454.1	EACC1	EACC1*→	130	Nocardia sp. Y48	GCF_001886715.1	actinobacteria
WP_084772597.1	EACC1	polar_lead?+TM+TM+TM+polar_tail?+low-complexity→??→ EACC1*→ CASPASE+TPRs→	126	Streptomyces sp. MOE7	GCA_002090335.1	actinobacteria
WP_084774643.1	EACC1	Glyoxalase→ EACC1*→ CASPASE+DnaJ_ZnR+DnaJ_ZnR+DnaJ_C→	135	Streptomyces sp. MOE7	GCA_002090335.1	actinobacteria
WP_084896276.1	EACC1	EACC1*→ CASPASE→	143	Streptomyces sp. CB03238	GCA_002099185.1	actinobacteria
WP_085565198.1	EACC1	EACC1*→ CASPASE+GAF+PAS→	150	Streptomyces griseofuscus	GCA_002114065.1	actinobacteria
WP_085570882.1	EACC1	EACC1*→ CASPASE+TPRs→	119	Streptomyces sp. 13-12-16	GCA_002114215.1	actinobacteria
WP_085571424.1	EACC1	??→ EACC1*→ CASPASE+TPRs→	169	Streptomyces sp. 13-12-16	GCA_002114215.1	actinobacteria
WP_085922372.1	EACC1	EACC1*→ CASPASE→ TPRs→	114	Streptomyces platensis	GCA_002119195.1	actinobacteria
WP_085924689.1	EACC1	-	127	Streptomyces platensis	-	actinobacteria
WP_086157994.1	EACC1	EACC1*→ CASPASE+STAND+polar_region+TM+BetaPropeller→	128	Streptomyces sp. SCSIO 03032	GCA_002128305.1	actinobacteria
WP_086168353.1	EACC1	EACC1*→ CASPASE(fragment)→ CASPASE+STAND+polar_region+TM+BetaPropeller→	173	Streptomyces pharetrae	GCA_002128465.1	actinobacteria
WP_086172729.1	EACC1	EACC1*→?→ AB_hydrolase→	136	Streptomyces pharetrae	GCA_002128465.1	actinobacteria
WP_086577439.1	EACC1+??	EACC1+??*→	459	Streptosporangium minutum	GCA_002149035.1	actinobacteria
WP_086662191.1	EACC1+CASPASE+TPR_repeats+low_complexity+TM_region	TM+TM+BPD_transp_1→ OppC_N+TM+TM+BPD_transp_1→ ABC-ATPase+ABC-ATPase→ PBPB→ TM+Beta_rich+TM+Beta_rich+TM+TM→ EACC1+CASPASE+TPR_repeats+low_complexity+TM_region*→	984	Lentzea kentuckyensis	GCF_002150765.1	actinobacteria
WP_086676010.1	EACC1	GATase→ ABC-ATPase+DUF4162→ cNMP_cyclase+TM+TM+TM+TM+TM+TM→ EACC1*→	105	Amycolatopsis pretoriensis	GCA_900107925.1	actinobacteria
WP_086696600.1	EACC1	??→??→ EACC1*→ CASPASE+??→	129	Streptomyces tricolor	GCF_002155905.1	actinobacteria
WP_086697136.1	EACC1	AMP-binding+AMP-binding_C→ AMP-binding+AMP-binding_C→ EACC1*→	143	Streptomyces tricolor	GCF_002155905.1	actinobacteria
WP_086721557.1	EACC1	EACC1*→ CASPASE+TPRs→	121	Streptomyces angustmyceticus	GCF_002154415.1	actinobacteria
WP_086727347.1	EACC1	??→ EACC1*→	114	Streptomyces carpinensis	GCF_002154505.1	actinobacteria
WP_086729273.1	EACC1	EACC1*→ CASPASE+TM+TM+bPH_domain?→	128	Streptomyces carpinensis	GCF_002154505.1	actinobacteria
WP_086733927.1	EACC1	EACC1*→ PSE→ AB_hydrolase→	136	Streptomyces glaucescens	GCF_002154375.1	actinobacteria
WP_086735375.1	EACC1	EACC1*→ CASPASE+??+BsuPI→	99	Streptomyces glaucescens	GCF_002154375.1	actinobacteria
WP_086740326.1	EACC1	EACC1*→ CASPASE+STAND+polar_region+TM+BetaPropeller→	173	Streptomyces glaucescens	GCF_002154375.1	actinobacteria
WP_086754885.1	EACC1	EACC1*→ CASPASE+??→	120	Streptomyces scabiei	GCF_002156075.1	actinobacteria
WP_086766728.1	EACC1	low-complexity+HD+Tox-RelA+TGS+ACT→ Pkinase+TPRs→ EACC1*→ CASPASE+TPRs→	143	Streptomyces bobili	GCF_002154575.1	actinobacteria
WP_086796440.1	EACC1	EACC1*→ CASPASE+TPRs→	124	Streptomyces scabiei	GCF_002155725.1	actinobacteria
WP_086816558.1	EACC1	EACC1*→ CASPASE+TPRs→	162	Streptomyces cacaoi	GCA_006539165.1	actinobacteria
WP_086819105.1	EACC1	EACC1*→	121	Allokutzneria sp. NRRL B-24872	GCF_002155965.1	actinobacteria
WP_086825751.1	EACC1	EACC1*→ CASPASE+TPRs→	128	Allokutzneria sp. NRRL B-24872	GCF_002155965.1	actinobacteria
WP_086839407.1	EACC1	EACC1*→	130	Amycolatopsis kentuckyensis	GCF_002155975.1	actinobacteria
WP_086844622.1	EACC1	PBPB→?→?→ Aminotran_5+PAS_6+HTH_22→ EACC1*→	125	Amycolatopsis kentuckyensis	GCF_002155975.1	actinobacteria
WP_086847557.1	EACC1	EACC1*→ CASPASE+TPRs→	110	Amycolatopsis kentuckyensis	GCF_002155975.1	actinobacteria
WP_086848657.1	EACC1	GATase→ ABC-ATPase+DUF4162→ cNMP_cyclase+TM+TM+TM+TM+TM+TM→ EACC1*→	105	Amycolatopsis kentuckyensis	GCF_002155975.1	actinobacteria
WP_086856190.1	EACC1	GATase→ ABC-ATPase+DUF4162→ cNMP_cyclase+TM+TM+TM+TM+TM+TM→ EACC1*→	105	Amycolatopsis lexingtonensis	GCA_003937945.1	actinobacteria
WP_086876208.1	EACC1	low-complexity+HD+Tox-RelA+TGS+ACT→ Pkinase+TPRs→ EACC1*→ CASPASE+TPRs→	138	Streptomyces rochei	GCF_002155895.1	actinobacteria
WP_086885313.1	EACC1	EACC1*→ CASPASE+STAND+TPRs→	144	Streptomyces rhizosphaericus	GCF_002155885.1	actinobacteria
WP_087684270.1	EACC1	TetR_N+TetR_C_6→ EACC1*→ CASPASE+low-complexity+TM+PBPB→	141	Streptomyces autolyticus	GCA_001983975.1	actinobacteria
WP_087764507.1	EACC1	EACC1*→ CASPASE+Beta_rich_repeat→	143	Streptomyces sp. CS057	GCA_002188345.1	actinobacteria
WP_087765136.1	EACC1	EACC1*→ CASPASE+STAND+??+TPRs+??→	119	Streptomyces sp. CS057	GCA_002188345.1	actinobacteria
WP_087765178.1	EACC1	EACC1*→	117	Streptomyces sp. CS057	GCA_002188345.1	actinobacteria
WP_087812330.1	EACC1	EACC1*→ CASPASE+TPRs→	119	Streptomyces sp. CS113	GCA_002188365.1	actinobacteria
WP_088574340.1	EACC1	EACC1*→ CASPASE+STAND+polar_region+TM+BetaPropeller→	95	Streptomyces sp. PgraA7	GCA_900187925.1	actinobacteria
WP_088643096.1	EACC1	TM+TM+TM+Peptidase_M48+TM+DUF4864+??→ EACC1*→	132	Micromonospora wenchangensis	GCA_002210435.1	actinobacteria
WP_088646394.1	EACC1	EACC1*→ CASPASE+STAND+TPRs→||←TM+TM+TM	119	Micromonospora wenchangensis	GCA_002210435.1	actinobacteria
WP_088797080.1	EACC1	EACC1*→ CASPASE+TM+PBPB+vWA→	147	Streptomyces	GCA_002300315.1	actinobacteria

acc	architecture	operon	len	species	gca	taxend
WP_088800827.1	EACC1	EACC1*→	132	Streptomyces	GCA_002300315.1	actinobacteria
WP_088948769.1	EACC1	EACC1*→	126	Micromonospora zamorensis	GCA_900090275.1	actinobacteria
WP_088949309.1	EACC1	TM+TM+TM+Peptidase_M48+TM+DUF4864+??→ EACC1*→	138	Micromonospora zamorensis	GCA_900090275.1	actinobacteria
WP_088961186.1	EACC1	TM+TM+TM+Peptidase_M48+TM+DUF4864+??→ EACC1*→	130	Micromonospora purpureochromogenes	GCA_900091515.1	actinobacteria
WP_088982801.1	EACC1	EACC1*→ CASPASE+TM+TM+TM+TM+TM+TM→	137	Micromonospora echinospora	GCA_002266845.1	actinobacteria
WP_088988490.1	EACC1	TM+TM+TM+Peptidase_M48+TM+DUF4864+??→ EACC1*→	130	Micromonospora chokoriensis	GCA_900091505.1	actinobacteria
WP_088989057.1	EACC1	EACC1*→	126	Micromonospora chokoriensis	GCF_900091505.1	actinobacteria
WP_088992833.1	EACC1	TM+TM+TM+Peptidase_M48+TM+DUF4864+??→ EACC1*→ TPRs+CASPASE→	133	Micromonospora echinaurantiaca	GCA_900090235.1	actinobacteria
WP_089020355.1	EACC1	EACC1*→	128	Micromonospora coriariae	GCA_900091455.1	actinobacteria
WP_089158195.1	EACC1	EACC1*→ CASPASE+STAND+polar_region+TM+BetaPropeller→	129	Micromonospora sp. NBS 11-29	GCF_002140155.1	actinobacteria
WP_089205289.1	EACC1	EACC1*→ CASPASE+??→	115	Streptosporangium subroseum	GCA_900188345.1	actinobacteria
WP_089209902.1	EACC1	EACC1*→	61	Streptosporangium subroseum	GCF_900188345.1	actinobacteria
WP_089222615.1	EACC1	EACC1*→ CASPASE+PBPB+vWA→	122	Streptomyces glauciniger	GCA_900188405.1	actinobacteria
WP_089290989.1	EACC1	EACC1*→ CASPASE+BetaPropeller→	127	Actinoplanes regularis	GCA_900188005.1	actinobacteria
WP_089293414.1	EACC1	EACC1*→ CASPASE+TPRs→	125	Actinoplanes regularis	GCF_900188005.1	actinobacteria
WP_089309665.1	EACC1	EACC1*→ CASPASE+TM+TM→	102	Actinomadura mexicana	GCF_900188105.1	actinobacteria
WP_089316989.1	??+EACC1	??+EACC1*→	227	Actinomadura mexicana	GCA_900188105.1	actinobacteria
WP_089335170.1	EACC1	EACC1*→ CASPASE+TM+TM+TM+TM+TM+TM+TM+TM→ Aminotran_5→	127	Blastococcus sp. DSM 44272	GCF_900188025.1	actinobacteria
WP_089404658.1	EACC1	EACC1*→	134	Geodermatophilus saharensis	-	actinobacteria
WP_089510392.1	EACC1	-	126	Streptomyces sp. NBS 14/10	-	actinobacteria
WP_089513739.1	EACC1	EACC1*→ CASPASE+TM+TM+??+TM+TM+??+TM+TM+??→	124	Streptomyces sp. NBS 14/10	GCA_002224125.1	actinobacteria
WP_089918135.1	EACC1	polar_lead?+TM+TM+TM+polar_tail?+low-complexity→??→ EACC1*→ CASPASE+TPRs→	115	Lentzea albida	GCA_900111005.1	actinobacteria
WP_089919256.1	EACC1+CASPASE+TPR_repeats+low_complexity+TM_region	TM+TM+BPD_transp_1→ OppC_N+TM+TM+BPD_transp_1→ ABC-ATPase+ABC-ATPase→ PBPB→ TM+Beta_rich+TM+Beta_rich+TM+TM→ EACC1+CASPASE+TPR_repeats+low_complexity+TM_region*→	794	Lentzea albida	GCA_900111005.1	actinobacteria
WP_089938606.1	EACC1	RVT_1→23S_rRNA_IVP→ EACC1*→	127	Candidatus Entotheonella palauensis	GCA_000522445.1	nitrospinae/tectomicrobia group
WP_089951186.1	EACC1+CASPASE+TPR_repeats+low_complexity+TM_region	TM+TM+BPD_transp_1→ OppC_N+TM+TM+BPD_transp_1→ ABC-ATPase+ABC-ATPase→ PBPB→ TM+Beta_rich+TM+Beta_rich+TM+TM→ EACC1+CASPASE+TPR_repeats+low_complexity+TM_region*→	817	Lechevalieria xinjiangensis	GCA_900110955.1	actinobacteria
WP_090011727.1	EACC1+CASPASE+TPR_repeats+low_complexity+TM_region	TM+TM+BPD_transp_1→ OppC_N+TM+TM+BPD_transp_1→ ABC-ATPase+ABC-ATPase→ PBPB→ TM+Beta_rich+TM+Beta_rich+TM+TM→ EACC1+CASPASE+TPR_repeats+low_complexity+TM_region*→	772	Lentzea violacea	GCA_900100275.1	actinobacteria
WP_090048966.1	EACC1+CASPASE+TPR_repeats+low_complexity+TM_region	TM+TM+BPD_transp_1→ OppC_N+TM+TM+BPD_transp_1→ ABC-ATPase+ABC-ATPase→ PBPB→ TM+Beta_rich+TM+Beta_rich+TM+TM→ EACC1+CASPASE+TPR_repeats+low_complexity+TM_region*→	828	Lechevalieria fradiae	GCA_900100955.1	actinobacteria
WP_090068728.1	EACC1+CASPASE+TPR_repeats+low_complexity+TM_region	TM+TM+BPD_transp_1→ OppC_N+TM+TM+BPD_transp_1→ ABC-ATPase+ABC-ATPase→ PBPB→ TM+Beta_rich+TM+Beta_rich+TM+TM→ EACC1+CASPASE+TPR_repeats+low_complexity+TM_region*→	895	Lentzea flaviverrucosa	GCA_003350445.1	actinobacteria
WP_090097831.1	EACC1+CASPASE+TPR_repeats+low_complexity+TM_region	TM+TM+BPD_transp_1→ OppC_N+TM+TM+BPD_transp_1→ ABC-ATPase+ABC-ATPase→ PSE→ TM+Beta_rich+TM+Beta_rich+TM+TM→ EACC1+CASPASE+TPR_repeats+low_complexity+TM_region*→	750	Lentzea jiangxiensis	GCA_900104175.1	actinobacteria
WP_090797923.1	EACC1	TPRs+CASPASE→?→?→ EACC1*→?→ CASPASE+TPRs→	128	Asanoa ishikariensis	GCA_900107455.1	actinobacteria
WP_090800042.1	EACC1	EACC1*→ CASPASE+PRTase→	113	Asanoa ishikariensis	GCA_900107455.1	actinobacteria
WP_091039878.1	EACC1	EACC1*→ CASPASE+TPRs→	109	Glycomyces harbinensis	GCF_900101745.1	actinobacteria
WP_091046058.1	EACC1	EACC1*→	121	Glycomyces sambucus	GCA_900102815.1	actinobacteria
WP_091088895.1	EACC1	EACC1*→ CASPASE+STAND+polar_region+TM+BetaPropeller→	113	Nonomuraea wenchangensis	GCF_900111685.1	actinobacteria
WP_091092856.1	EACC1	TM+TM+vWA+TM→ TM+TM+vWA+TM→ EACC1*→ CASPASE+TPRs→	111	Nonomuraea wenchangensis	GCF_900111685.1	actinobacteria
WP_091237393.1	EACC1	TM+TM+TM+Peptidase_M48+TM+DUF4864+??→ EACC1*→	129	Micromonospora matsumotoense	GCA_900091525.1	actinobacteria
WP_091239083.1	EACC1	EACC1*→ CASPASE+STAND+TPRs→||←?←?←?←TM+TM+TM	119	Micromonospora matsumotoense	GCA_900091525.1	actinobacteria
WP_091280673.1	EACC1+EAD1	EACC1+EAD1*→ CASPASE+STAND+polar_region+TM+BetaPropeller→	234	Frankia sp. G2	GCA_001536285.1	actinobacteria
WP_091285508.1	EACC1	EACC1*→ CASPASE+STAND+polar_region+TM+BetaPropeller→	126	Amycolatopsis xylanica	GCF_900107045.1	actinobacteria
WP_091288897.1	EACC1	GATase→ ABC-ATPase+DUF4162→ cNMP_cyclase+TM+TM+TM+TM+TM+TM→ EACC1*→	79	Amycolatopsis xylanica	GCA_900107045.1	actinobacteria
WP_091289305.1	EACC1	EACC1*→ CASPASE+DnaJ_ZnR+DnaJ_ZnR+DnaJ_C→	122	Amycolatopsis xylanica	GCA_900107045.1	actinobacteria
WP_091305099.1	EACC1	GATase→ ABC-ATPase+DUF4162→ cNMP_cyclase+TM+TM+TM+TM+TM+TM→ EACC1*→	105	Amycolatopsis tolypomycina	GCA_900105945.1	actinobacteria
WP_091306292.1	EACC1	EACC1*→	118	Amycolatopsis tolypomycina	GCA_900105945.1	actinobacteria
WP_091360655.1	EACC1	EACC1*→ CASPASE+low-complexity+DUF4190(has2TM)→	132	Frankineae bacterium MT45	GCF_900100325.1	actinobacteria
WP_091399541.1	EACC1	EACC1*→	121	Micromonospora saelicesensis	GCA_900091575.1	actinobacteria

acc	architecture	operon	len	species	gca	taxend
WP_091407867.1	EACC1	TM+TM+TM+Peptidase_M48+TM+DUF4864+??→ EACC1*→	130	Micromonospora saelicesensis	GCA_900091575.1	actinobacteria
WP_091435315.1	EACC1	EACC1*→ CASPASE+SF1-helicase→	125	Micromonospora yangpuensis	GCA_900091615.1	actinobacteria
WP_091447807.1	EACC1	EACC1*→	136	Alloactinosynnema iranicum	GCA_900101685.1	actinobacteria
WP_091453106.1	EACC1	EACC1*→ CASPASE+TPRs→	124	Alloactinosynnema iranicum	GCA_900101685.1	actinobacteria
WP_091516393.1	EACC1	EACC1*→ CASPASE+TM+TM+TM+TM→	120	Amycolatopsis sacchari	GCA_900114035.1	actinobacteria
WP_091597524.1	EACC1	EACC1*→	121	Amycolatopsis lurida	GCA_000749465.3	actinobacteria
WP_091624407.1	EACC1	EACC1*→	114	Amycolatopsis saalfeldensis	GCA_900110575.1	actinobacteria
WP_092380860.1	EACC1	EACC1*→ CASPASE+Pkinase→	67	Xiangella phaseoli	GCF_003253475.1	actinobacteria
WP_092555827.1	??+STAND+EACC1	??+STAND+??→??+STAND+EACC1*→	770	Actinoplanes derwentensis	GCA_900104725.1	actinobacteria
WP_092704916.1	EACC1	EACC1*→	108	Rhodanobacter glycinis	GCF_900114325.1	gammaproteobacteria
WP_092783696.1	EACC1	EACC1*→	113	Actinokineospora terrae	GCA_900111175.1	actinobacteria
WP_092783891.1	EACC1	EACC1*→ CASPASE+??+TM+TM+TM+TM+TM+TM+TM+TM+TM→ CASPASE+??+TM+TM+TM+TM+TM+TM+TM+TM→	128	Actinokineospora terrae	GCF_900111175.1	actinobacteria
WP_093160608.1	EACC1	EACC1*→ CASPASE+FTSK+FTSK+FTSK+FTSK→	128	Saccharopolyspora antimicrobica	GCA_003635025.1	actinobacteria
WP_093258118.1	EACC1	EACC1*→ CASPASE+Astro_capsid_N→	84	Thermostaphylospora chromogena	-	actinobacteria
WP_093344959.1	EACC1	EACC1*→ CASPASE+FTSK+FTSK+FTSK+FTSK→	128	Saccharopolyspora	GCA_003053965.1	actinobacteria
WP_093345617.1	EACC1	EACC1*→ CASPASE+BetaPropeller→	125	Saccharopolyspora	GCF_900112555.1	actinobacteria
WP_093405879.1	EACC1	EACC1*→ CASPASE+TPRs→	123	Verrucosispora sediminis	GCA_900112325.1	actinobacteria
WP_093468834.1	EACC1	EACC1*→ CASPASE+STAND+polar_region+TM+BetaPropeller→	129	Streptomyces melanosporofaciens	GCF_900105695.1	actinobacteria
WP_093485252.1	EACC1	EACC1*→ CASPASE+??→	119	Streptomyces	GCA_002797835.1	actinobacteria
WP_093491400.1	EACC1	EACC1*→	132	Streptomyces sp. 2112.3	GCA_003610145.1	actinobacteria
WP_093497024.1	EACC1	EACC1*→ CASPASE+TM+TM+BetaPropeller→	128	Streptomyces sp. Ag109_O5-10	GCA_900105755.1	actinobacteria
WP_093588272.1	EACC1+CASPASE+TPR_repeats+low_complexity+TM_region	TM+TM+BPD_transp_1→ OppC_N+TM+TM+BPD_transp_1→ ABC-ATPase+ABC-ATPase→ PBPB→ TM+Beta_rich+TM+Beta_rich+TM+TM→ EACC1+CASPASE+TPR_repeats+low_complexity+TM_region*→	831	Lentzea waywayandensis	GCA_900115955.1	actinobacteria
WP_093595983.1	EACC1	EACC1*→ CASPASE+TM+TM+bPH_domain?→	162	Streptomyces sp. MnatMP-M17	GCA_900091995.1	actinobacteria
WP_093598336.1	EACC1	EACC1*→ CASPASE+STAND+polar_region+TM+BetaPropeller→	123	Lentzea waywayandensis	GCA_900115955.1	actinobacteria
WP_093604360.1	EACC1	EACC1*→	145	Streptomyces sp. MnatMP-M17	GCA_900091995.1	actinobacteria
WP_093615170.1	EACC1	EACC1*→ CASPASE+PBPB+vWA→ TM+PBPB→	118	Actinoplanes philippinensis	GCA_900113015.1	actinobacteria
WP_093641700.1	EACC1	EACC1*→ CASPASE+Beta_rich_repeat→	155	Streptomyces sp. SceaMP-e96	GCF_900090095.1	actinobacteria
WP_093643256.1	EACC1	Phage_int_SAM_3+Phage_integrase→ EACC1*→ CASPASE+TPRs→	119	Streptomyces sp. SceaMP-e96	GCF_900090095.1	actinobacteria
WP_093643475.1	EACC1	EACC1*→	135	Streptomyces	GCA_003173275.1	actinobacteria
WP_093647855.1	EACC1	EACC1*→ CASPASE+FGS_fold-domain→??→??→ TM+TM+BaxI_1→?→ NUDIX→	134	Streptomyces sp. SceaMP-e96	GCA_900090095.1	actinobacteria
WP_093700286.1	EACC1	TetR_N+TetR_C_6→ EACC1*→	140	Streptomyces sp. MnatMP-M27	-	actinobacteria
WP_093709954.1	EACC1	EACC1*→ CASPASE+STAND+polar_region+TM+BetaPropeller→	135	Streptomyces sp. 2131.1	GCA_900105515.1	actinobacteria
WP_093715662.1	EACC1	EACC1*→	134	Streptomyces alni	GCF_900112845.1	actinobacteria
WP_093717772.1	EACC1	EACC1*→ CASPASE+low-complexity+BetaPropeller→	117	Streptomyces alni	GCA_900112845.1	actinobacteria
WP_093734734.1	EACC1	EACC1*→	127	Streptomyces sp. DvalAA-14	GCF_900091855.1	actinobacteria
WP_093737778.1	EACC1	EACC1*→ CASPASE+TPRs→	126	Streptomyces sp. DvalAA-14	GCA_900091855.1	actinobacteria
WP_093744297.1	EACC1	EACC1*→ CASPASE+TPRs→	121	Streptomyces sp. PAN_FS17	GCA_900105465.1	actinobacteria
WP_093749236.1	EACC1	EACC1*→	63	Streptomyces sp. PAN_FS17	GCA_900105465.1	actinobacteria
WP_093760390.1	EACC1	EACC1*→ CASPASE+??+SF1-helicase+Vsr(PDDEXK)?→	138	Streptomyces sp. BpilaLS-43	GCA_900091875.1	actinobacteria
WP_093764368.1	EACC1	TM+TM+TM+PP2C→??→ EACC1*→ CASPASE+TM+TM+PBPB+vWA→	114	Streptomyces sp. F-7	GCF_900079415.1	actinobacteria
WP_093782548.1	EACC1	EACC1*→ CASPASE+TPRs→	132	Streptomyces guanduensis	GCA_900103985.1	actinobacteria
WP_093799277.1	EACC1	EACC1*→ CASPASE+TM?+BetaPropeller→	152	Streptomyces sp. Wb2n-11	GCF_001049855.1	actinobacteria
WP_093859836.1	EACC1	FAD_binding_4+FAD-oxidase_C→ DAGK_cat+DAGK_acc→ EACC1*→ CASPASE+TPRs→ Clp_N+Clp_N+ClpABN-AAA→	135	Streptomyces sp. TLI_053	GCF_900105395.1	actinobacteria
WP_093863049.1	EACC1	EACC1*→ CASPASE+HSP70→	139	Streptomyces sp. TLI_053	GCA_900105395.1	actinobacteria
WP_093896825.1	EACC1	EACC1*→ CASPASE+??→	137	Streptomyces sp. Ncost-T10-10d	GCA_900091955.1	actinobacteria
WP_093897822.1	EACC1	EACC1*→ CASPASE+STAND+polar_region+TM+BetaPropeller→	106	Streptomyces sp. Ncost-T10-10d	-	actinobacteria
WP_093905892.1	EACC1	EACC1*→ CASPASE+TPRs→	179	Streptomyces sp. cf386	GCF_900103585.1	actinobacteria
WP_093937741.1	EACC1	EACC1*→	109	Amycolatopsis thailandensis	GCA_002234405.1	actinobacteria
WP_093945735.1	EACC1	GATase→ ABC-ATPase+DUF4162→ cNMP_cyclase+TM+TM+TM+TM+TM+TM→ EACC1→ CASPASE+EAD2+TM+TM+TM+TM→ EACC1*→	104	Amycolatopsis sp. H5	GCA_002234595.1	actinobacteria
WP_093945737.1	EACC1	GATase→ ABC-ATPase+DUF4162→ cNMP_cyclase+TM+TM+TM+TM+TM+TM→ EACC1*→ CASPASE+EAD2+TM+TM+TM+TM→ EACC1→	118	Amycolatopsis sp. H5	GCA_002234595.1	actinobacteria
WP_093950342.1	EACC1	PBPB→?→?→ Aminotran_5+PAS_6+HTH_22→ EACC1*→ CASPASE+??+TM+TM→	126	Amycolatopsis sp. H5	GCA_002234595.1	actinobacteria

acc	architecture	operon	len	species	gca	taxend
WP_094006827.1	EACC1	EACC1*→ CASPASE+low-complexity+BetaPropeller→	113	Amycolatopsis sp. KNN50.9b	GCA_002234605.1	actinobacteria
WP_094103272.1	EACC1	low-complexity+HD+Tox-RelA+TGS+ACT→ Pkinase+TPRs→ EACC1*→ CASPASE+TPRs→	142	Streptomyces phaeoluteigriseus	GCA_001896135.2	actinobacteria
WP_094212107.1	EACC1	EACC1*→ CASPASE+TPRs+LysM+PG-binding→	118	Streptomyces sp. 2R	GCA_002242735.1	actinobacteria
WP_094214728.1	EACC1	EACC1*→ CASPASE+STAND+polar_region+TM+BetaPropeller→ TM+Acid_phosphat_B→	141	Streptomyces diastatochromogenes	GCF_002242805.1	actinobacteria
WP_094215070.1	EACC1	EACC1*→ CASPASE+low-complexity+TM+TM+TM+TM+TM+TM+TM+TM+TM+TM+BetaPropeller→	143	Streptomyces diastatochromogenes	GCF_002242805.1	actinobacteria
WP_094299657.1	EACC1	EACC1*→	120	Salmonella enterica	GCA_002234475.1	gammaproteobacteria
WP_094374006.1	EACC1	??→??→ EACC1*→ CASPASE+??→	129	Streptomyces sp. FBKL.4005	GCA_002251155.1	actinobacteria
WP_095709382.1	EACC1	EACC1*→ CASPASE+Trypsin+TPRs→	132	Streptomyces	GCA_003428925.1	actinobacteria
WP_095751376.1	EACC1	EACC1*→ CASPASE+STAND+polar_region+TM+BetaPropeller→	133	Streptomyces sp. SA15	GCA_002291145.1	actinobacteria
WP_095757213.1	EACC1	EACC1*→ CASPASE+PBPB+vWA→	129	Streptomyces xinghaiensis	GCF_000220705.2	actinobacteria
WP_095853409.1	EACC1	EACC1*→ CASPASE+BetaPropeller→	139	Streptomyces sp. Ag82_O1-15	GCA_003633975.1	actinobacteria
WP_095854532.1	EACC1	EACC1*→ CASPASE+TM+PBPI→	108	Streptomyces	GCA_002300485.1	actinobacteria
WP_096058917.1	EACC1	EACC1*→ CASPASE+STAND+polar_region+TM+BetaPropeller→	135	Streptomyces thermoautotrophicus	GCF_001543895.1	actinobacteria
WP_096302135.1	EACC1	EACC1*→ CASPASE+low-complexity+DUF4190(has2TM)→?→ Glyoxalase→	132	Jatrophihabitans sp. GAS493	GCF_900230215.1	actinobacteria
WP_096492710.1	EACC1	EACC1*→	121	Actinosynnema pretiosum	GCA_002354875.1	actinobacteria
WP_096901987.1	EACC1	EACC1*→	103	Klebsiella pneumoniae	GCA_002411735.1	gammaproteobacteria
WP_097190236.1	EACC1	EACC1→ Tnp_DNA_bind+DDE_Tnp_1+Dimer_Tnp_Tn5→ CASPASE+TPRs→ EACC1→ CASPASE(fragment)→ EACC1*→ CASPASE+CASPASE+TM+TPRs→ CASPASE(fragment)→	135	Burkholderia sp. OK806	GCA_900230245.1	betaproteobacteria
WP_097190238.1	EACC1	EACC1→ Tnp_DNA_bind+DDE_Tnp_1+Dimer_Tnp_Tn5→ CASPASE+TPRs→ EACC1*→ CASPASE(fragment)→ EACC1→ CASPASE+CASPASE+TM+TPRs→ CASPASE(fragment)→	138	Burkholderia sp. OK806	GCA_900230245.1	betaproteobacteria
WP_097190240.1	EACC1	EACC1*→ Tnp_DNA_bind+DDE_Tnp_1+Dimer_Tnp_Tn5→ CASPASE+TPRs→ EACC1→ CASPASE(fragment)→ EACC1→ CASPASE+CASPASE+TM+TPRs→ CASPASE(fragment)→	135	Burkholderia sp. OK806	GCF_900230245.1	betaproteobacteria
WP_097217245.1	EACC1	EACC1*→ CASPASE+??+TM+TM+TM→	125	Streptomyces sp. Ag82_G6-1	GCA_900215615.1	actinobacteria
WP_097229463.1	EACC1	EACC1*→ CASPASE+STAND+polar_region+TM+BetaPropeller→	144	Streptomyces zhaozhouensis	GCA_900230195.1	actinobacteria
WP_097231130.1	EACC1	Pkinase+TPRs→ EACC1*→ CASPASE+TPRs→	149	Streptomyces zhaozhouensis	GCA_900230195.1	actinobacteria
WP_097233812.1	EACC1	EACC1*→ CASPASE+JAB→	130	Streptomyces zhaozhouensis	-	actinobacteria
WP_097244713.1	EACC1	EACC1*→ CASPASE+TPRs→	113	Nocardia amikacinitolerans	GCA_900215415.1	actinobacteria
WP_097255380.1	EACC1	EACC1*→ CASPASE+STAND+polar_region+TM+BetaPropeller→ TM+Acid_phosphat_B→	141	Streptomyces sp. Ag109_G2-15	GCF_900215565.1	actinobacteria
WP_097257754.1	EACC1	EACC1*→ CASPASE+??+TM+TM+TM+TPRs→	112	Streptomyces	GCA_002846295.1	actinobacteria
WP_097317403.1	EACC1	EACC1*→ CASPASE+Pentapeptide_repeats→	112	Actinoplanes atraurantiacus	GCA_900215205.1	actinobacteria
WP_097327188.1	EACC1	EACC1*→ CASPASE+TPRs→	108	Actinoplanes atraurantiacus	GCA_900215205.1	actinobacteria
WP_097478582.1	EACC1	EACC1*→	116	Escherichia coli	GCF_002518865.1	gammaproteobacteria
WP_097868186.1	EACC1	EACC1*→ CASPASE+??+SF1-helicase+Vsr(PDDEXK)?→	138	Streptomyces sp. rh34	GCF_002551245.1	actinobacteria
WP_097873431.1	EACC1	EACC1*→ CASPASE+TPRs→	118	Streptomyces sp. ms184	GCF_002551335.1	actinobacteria
WP_097883263.1	EACC1	EACC1*→ CASPASE+TPRs→	118	Streptomyces sp. st140	GCF_002551135.1	actinobacteria
WP_097932812.1	EACC1	EACC1*→ CASPASE+STAND+??+TPRs+??→	119	Streptomyces	GCF_002551215.1	actinobacteria
WP_097944194.1	EACC1	EACC1*→ CASPASE+TPRs→	121	Streptomyces sp. ms115	GCF_002551345.1	actinobacteria
WP_097965555.1	EACC1	HATPase_c_2→ EACC1*→ CASPASE+Beta_rich_repeat→	143	Streptomyces sp. or20	GCF_002551305.1	actinobacteria
WP_097984112.1	EACC1	EACC1*→ CASPASE+TPRs→	118	Streptomyces sp. f150	GCF_002551435.1	actinobacteria
WP_098020341.1	EACC1	EACC1*→ CASPASE+TPRs→	118	Streptomyces sp. b62	GCF_002551475.1	actinobacteria
WP_098083203.1	EACC1	DUF2510(W3)+BicD+DUF4041+T5orf172→ EACC1*→ CASPASE+TPRs→	121	Nocardia sp. FDAARGOS_372	GCA_002554515.1	actinobacteria
WP_098512379.1	EACC1	GATase→ ABC-ATPase+DUF4162→ cNMP_cyclase+TM+TM+TM+TM+TM+TM→ EACC1*→	110	Amycolatopsis sulphurea	GCA_003600245.1	actinobacteria
WP_098750929.1	EACC1	low-complexity+HD+Tox-RelA+TGS+ACT→ Pkinase+TPRs→ EACC1*→ CASPASE+TPRs→	130	Streptomyces sp. Ru87	GCA_002573955.1	actinobacteria
WP_098752253.1	EACC1	EACC1*→	141	Streptomyces sp. Ru87	GCA_002573955.1	actinobacteria
WP_098753503.1	EACC1	polar_lead?+TM+TM+TM+polar_tail?+low-complexity→??→ EACC1*→ CASPASE+TPRs→	159	Streptomyces sp. Ru87	GCA_002573955.1	actinobacteria
WP_098753773.1	EACC1	EACC1*→ CASPASE+TM?+BetaPropeller→||←DUF664	121	Streptomyces sp. Ru87	GCA_002573955.1	actinobacteria
WP_098891783.1	EACC1	EACC1*→ CASPASE+TPRs→	125	Streptomyces sp. or3	GCF_002551295.1	actinobacteria
WP_098897631.1	EACC1	EACC1*→ CASPASE+STAND+polar_region+TM+BetaPropeller→	95	Streptomyces sp. st77	GCF_002551175.1	actinobacteria
WP_099014516.1	EACC1	EACC1*→ CASPASE+TM+??→	122	Streptomyces malaysiensis	GCA_002591335.1	actinobacteria
WP_099014682.1	EACC1	TetR_N+TetR_C_6→ EACC1*→ CASPASE+low-complexity+TM+PBPB→	137	Streptomyces malaysiensis	GCF_002591335.1	actinobacteria
WP_099127615.1	EACC1	EACC1→ CASPASE+low-complexity+BetaPropeller___*_##→	122	Streptomyces sp. ScaeMP-e48	GCA_900090155.1	actinobacteria
WP_099174769.1	EACC1	polar_lead?+TM+TM+TM+polar_tail?+low-complexity→??→ EACC1*→ CASPASE+TPRs→	130	Streptomyces sp. LamerLS-316	GCA_900090175.1	actinobacteria

<table>
<thead>
<tr><th>acc</th><th>architecture</th><th>operon</th><th>len</th><th>species</th><th>gca</th><th>taxend</th></tr>
</thead>
<tbody>
<tr><td>WP_099198034.1</td><td>EACC1</td><td>EACC1*→ CASPASE+TM+TM+PBPB+vWA→</td><td>99</td><td>Streptomyces cinnamoneus</td><td>GCA_002727115.1</td><td>actinobacteria</td></tr>
<tr><td>WP_099217477.1</td><td>EACC1</td><td>EACC1*→ CASPASE+??+SF1-helicase+Vsr(PDDEXK)?→</td><td>138</td><td>Streptomyces</td><td>GCA_003173635.1</td><td>actinobacteria</td></tr>
<tr><td>WP_099281823.1</td><td>EACC1</td><td>EACC1*→</td><td>129</td><td>Streptomyces sp. AmelKG-E11A</td><td>GCF_002705975.1</td><td>actinobacteria</td></tr>
<tr><td>WP_099502446.1</td><td>EACC1</td><td>EACC1*→ CASPASE+TPRs→</td><td>122</td><td>Streptomyces</td><td>GCA_002946835.1</td><td>actinobacteria</td></tr>
<tr><td>WP_099847282.1</td><td>EACC1</td><td>TM+TM+TM+Peptidase_M48+TM+DUF4864+??→ EACC1*→</td><td>136</td><td>Verrucosispora sp. CNZ293</td><td>GCA_002754655.1</td><td>actinobacteria</td></tr>
<tr><td>WP_099854647.1</td><td>EACC1</td><td>EACC1*→</td><td>120</td><td>Micromonospora sp. CNZ299</td><td>GCF_002754415.1</td><td>actinobacteria</td></tr>
<tr><td>WP_099855908.1</td><td>EACC1</td><td>TM+TM+TM+Peptidase_M48+TM+DUF4864+??→ EACC1*→</td><td>138</td><td>Micromonospora sp. CNZ299</td><td>GCA_002754415.1</td><td>actinobacteria</td></tr>
<tr><td>WP_099871303.1</td><td>EACC1</td><td>Glyoxalase→ EACC1*→ CASPASE+DnaJ_ZnR+DnaJ_ZnR+DnaJ_C→</td><td>136</td><td>Streptomyces sp. 76</td><td>GCA_002754435.1</td><td>actinobacteria</td></tr>
<tr><td>WP_099871939.1</td><td>EACC1</td><td>EACC1*→ CASPASE+??→</td><td>137</td><td>Streptomyces sp. 76</td><td>GCA_002754435.1</td><td>actinobacteria</td></tr>
<tr><td>WP_099892682.1</td><td>EACC1</td><td>EACC1*→ CASPASE+GAF+PAS+GGDEF→</td><td>120</td><td>Streptomyces sp. 61</td><td>GCF_002754535.1</td><td>actinobacteria</td></tr>
<tr><td>WP_099893747.1</td><td>EACC1</td><td>low-complexity+HD+Tox-RelA+TGS+ACT→ Pkinase+TPRs→ EACC1*→ CASPASE+TPRs→</td><td>131</td><td>Streptomyces sp. 61</td><td>GCF_002754535.1</td><td>actinobacteria</td></tr>
<tr><td>WP_099898397.1</td><td>EACC1</td><td>polar_lead?+TM+TM+TM+polar_tail?+low-complexity→??→ EACC1*→ CASPASE+TPRs→</td><td>118</td><td>Streptomyces sp. 1</td><td>GCA_002754615.1</td><td>actinobacteria</td></tr>
<tr><td>WP_099904499.1</td><td>EACC1</td><td>Pkinase+TPRs→ EACC1*→ CASPASE+TPRs→</td><td>148</td><td>Streptomyces sp. 1</td><td>GCA_002754615.1</td><td>actinobacteria</td></tr>
<tr><td>WP_099918141.1</td><td>EACC1</td><td>EACC1*→ CASPASE+TM+??→</td><td>90</td><td>Streptomyces sp. 94</td><td>GCF_002754555.1</td><td>actinobacteria</td></tr>
<tr><td>WP_099919284.1</td><td>EACC1</td><td>EACC1*→ CASPASE+TPRs+LysM+PG-binding→</td><td>127</td><td>Streptomyces sp. 94</td><td>GCF_002754555.1</td><td>actinobacteria</td></tr>
<tr><td>WP_099922540.1</td><td>EACC1</td><td>EACC1*→ CASPASE+TM+PBPI→</td><td>145</td><td>Streptomyces sp. 94</td><td>GCA_002754555.1</td><td>actinobacteria</td></tr>
<tr><td>WP_099943888.1</td><td>EACC1</td><td>EACC1*→</td><td>143</td><td>Streptomyces sp. 93</td><td>GCF_002754595.1</td><td>actinobacteria</td></tr>
<tr><td>WP_100047576.1</td><td>EACC1</td><td>low-complexity+HD+Tox-RelA+TGS+ACT→ Pkinase+TPRs→ EACC1*→ CASPASE+TPRs→</td><td>137</td><td>Streptomyces sp. Alain-F2R5</td><td>GCA_002277855.1</td><td>actinobacteria</td></tr>
<tr><td>WP_100568343.1</td><td>EACC1</td><td>EACC1*→ CASPASE+TM+TM+PBPB+vWA→</td><td>114</td><td>Streptomyces sp. CB01373</td><td>GCA_002803075.1</td><td>actinobacteria</td></tr>
<tr><td>WP_100579415.1</td><td>EACC1</td><td>EACC1*→ CASPASE+STAND+polar_region+TM+BetaPropeller→</td><td>113</td><td>Streptomyces sp. CB01201</td><td>GCA_002803085.1</td><td>actinobacteria</td></tr>
<tr><td>WP_100590194.1</td><td>EACC1</td><td>EACC1*→ CASPASE+PBPB+vWA→</td><td>117</td><td>Kitasatospora sp. CB02891</td><td>GCA_002803145.1</td><td>actinobacteria</td></tr>
<tr><td>WP_100598629.1</td><td>EACC1</td><td>-</td><td>139</td><td>Streptomyces sp. CB01635</td><td>-</td><td>actinobacteria</td></tr>
<tr><td>WP_100602265.1</td><td>EACC1</td><td>low-complexity+HD+Tox-RelA+TGS+ACT→ Pkinase+TPRs→ EACC1*→ CASPASE+TPRs→</td><td>131</td><td>Streptomyces sp. CB02959</td><td>GCA_002803155.1</td><td>actinobacteria</td></tr>
<tr><td>WP_100605450.1</td><td>low_complexity+EACC1</td><td>low_complexity+EACC1*→ CASPASE+TM+TM+bPH_domain?→</td><td>229</td><td>Streptomyces sp. CB02959</td><td>GCA_002803155.1</td><td>actinobacteria</td></tr>
<tr><td>WP_100806405.1</td><td>EACC1</td><td>TetR_N+TetR_C_6→ EACC1*→ CASPASE+low-complexity+TM+PBPB→</td><td>142</td><td>Streptomyces sp. M56</td><td>GCF_002812405.1</td><td>actinobacteria</td></tr>
<tr><td>WP_100825534.1</td><td>EACC1</td><td>EACC1*→ CASPASE+STAND→ TM+BetaPropeller→ TM+Acid_phosphat_B→</td><td>135</td><td>Streptomyces sp. 69</td><td>GCF_002813405.1</td><td>actinobacteria</td></tr>
<tr><td>WP_100841017.1</td><td>EACC1</td><td>EACC1*→ CASPASE+TM+TM+??+TM+TM+??+TM+TM+??→</td><td>110</td><td>Streptomyces sp. 4121.5</td><td>GCA_002813165.1</td><td>actinobacteria</td></tr>
<tr><td>WP_101277357.1</td><td>EACC1</td><td>EACC1*→ CASPASE+TPRs→</td><td>126</td><td>Streptomyces sp. EAG2</td><td>GCA_002844215.1</td><td>actinobacteria</td></tr>
<tr><td>WP_101373080.1</td><td>EACC1</td><td>EACC1*→ CASPASE+STAND+polar_region+TM+BetaPropeller→ TM+Acid_phosphat_B→</td><td>141</td><td>Streptomyces sp. 59</td><td>GCF_002846295.1</td><td>actinobacteria</td></tr>
<tr><td>WP_101373258.1</td><td>EACC1</td><td>Pkinase+??→?→?→||←?←?||HATPase_c_2→ EACC1*→ CASPASE+??+SF1-helicase+Vsr(PDDEXK)?→</td><td>130</td><td>Streptomyces sp. 59</td><td>GCA_002846295.1</td><td>actinobacteria</td></tr>
<tr><td>WP_101378381.1</td><td>EACC1</td><td>EACC1*→ CASPASE+DnaJ_ZnR+DnaJ_C+DnaJ_C+TM+TM→</td><td>106</td><td>Streptomyces sp. GP55</td><td>GCA_002846355.1</td><td>actinobacteria</td></tr>
<tr><td>WP_101380473.1</td><td>EACC1</td><td>EACC1*→ CASPASE+??+STAND+??→</td><td>108</td><td>Streptomyces sp. GP55</td><td>GCF_002846355.1</td><td>actinobacteria</td></tr>
<tr><td>WP_101386063.1</td><td>EACC1</td><td>EACC1*→ CASPASE+low-complexity+BetaPropeller→</td><td>133</td><td>Streptomyces sp. TLI_146</td><td>GCF_002846415.1</td><td>actinobacteria</td></tr>
<tr><td>WP_101387394.1</td><td>EACC1</td><td>low-complexity+HD+Tox-RelA+TGS+ACT→ Pkinase+TPRs→ EACC1*→ CASPASE+TPRs→</td><td>128</td><td>Streptomyces sp. TLI_146</td><td>GCA_002846415.1</td><td>actinobacteria</td></tr>
<tr><td>WP_101407060.1</td><td>EACC1</td><td>EACC1*→ CASPASE+TPRs→ Pkinase+??→</td><td>135</td><td>Streptomyces sp. OK885</td><td>-</td><td>actinobacteria</td></tr>
<tr><td>WP_101417477.1</td><td>EACC1</td><td>EACC1*→ CASPASE+STAND+polar_region+TM+BetaPropeller→</td><td>93</td><td>Streptomyces sp. 73</td><td>GCF_002846315.1</td><td>actinobacteria</td></tr>
<tr><td>WP_101422506.1</td><td>EACC1+CASPASE+BetaPropeller</td><td>EACC1+CASPASE+BetaPropeller*→ BetaPropeller→||←METHYLASE</td><td>703</td><td>Streptomyces sp. CMB-StM0423</td><td>GCA_002847285.1</td><td>actinobacteria</td></tr>
<tr><td>WP_101436672.1</td><td>EACC1</td><td>EACC1*→</td><td>109</td><td>Amycolatopsis niigatensis</td><td>GCA_002846615.1</td><td>actinobacteria</td></tr>
<tr><td>WP_101468971.1</td><td>EACC1</td><td>EACC1*→ CASPASE+FTSK+FTSK+FTSK→</td><td>118</td><td>Nocardia fluminea</td><td>GCA_002846365.1</td><td>actinobacteria</td></tr>
<tr><td>WP_101606153.1</td><td>EACC1</td><td>EACC1*→ TM+ZU5+??→</td><td>107</td><td>Amycolatopsis sp. BJA-103</td><td>GCF_002849735.1</td><td>actinobacteria</td></tr>
<tr><td>WP_101608477.1</td><td>EACC1</td><td>EACC1*→ CASPASE+STAND+polar_region+TM+BetaPropeller→</td><td>128</td><td>Amycolatopsis sp. BJA-103</td><td>GCA_002849735.1</td><td>actinobacteria</td></tr>
<tr><td>WP_102005349.1</td><td>EACC1</td><td>EACC1*→</td><td>103</td><td>Klebsiella quasipneumoniae</td><td>GCA_002853675.1</td><td>gammaproteobacteria</td></tr>
<tr><td>WP_102923658.1</td><td>EACC1</td><td>low-complexity+HD+Tox-RelA+TGS+ACT→ Pkinase+TPRs→ EACC1*→ CASPASE+TPRs→</td><td>131</td><td>Streptomyces noursei</td><td>GCA_002891315.1</td><td>actinobacteria</td></tr>
<tr><td>WP_102937470.1</td><td>EACC1</td><td>TetR_N+TetR_C_6→ EACC1*→ CASPASE+low-complexity+TM+PBPB→</td><td>140</td><td>Streptomyces malaysiensis</td><td>GCA_002891865.1</td><td>actinobacteria</td></tr>
<tr><td>WP_103335469.1</td><td>EACC1</td><td>EACC1*→ CASPASE+HSP70→</td><td>118</td><td>Amycolatopsis sp. CA-126428</td><td>GCF_002904335.1</td><td>actinobacteria</td></tr>
<tr><td>WP_103335615.1</td><td>EACC1</td><td>PBPB→?→?→ Aminotran_5+PAS_6+HTH_22→ EACC1*→||←Glyco_trans_4_4+UDPGT</td><td>125</td><td>Amycolatopsis sp. CA-126428</td><td>GCF_002904335.1</td><td>actinobacteria</td></tr>
<tr><td>WP_103338162.1</td><td>EACC1</td><td>GATase→ ABC-ATPase+DUF4162→ cNMP_cyclase+TM+TM+TM+TM+TM+TM→ EACC1*→</td><td>105</td><td>Amycolatopsis sp. CA-126428</td><td>GCF_002904335.1</td><td>actinobacteria</td></tr>
<tr><td>WP_103353785.1</td><td>EACC1</td><td>GATase→ ABC-ATPase+DUF4162→ cNMP_cyclase+TM+TM+TM+TM+TM+TM→ EACC1*→</td><td>104</td><td>Amycolatopsis sp. CA-128772</td><td>GCF_002904295.1</td><td>actinobacteria</td></tr>
<tr><td>WP_103417476.1</td><td>EACC1</td><td>Beta_rich?→ EACC1*→ CASPASE+TPRs→??→??→</td><td>105</td><td>Streptomyces sp. ZL-24</td><td>GCF_002907485.1</td><td>actinobacteria</td></tr>
<tr><td>WP_103490228.1</td><td>EACC1</td><td>EACC1*→ CASPASE+TPRs→</td><td>118</td><td>Streptomyces</td><td>GCA_002910775.2</td><td>actinobacteria</td></tr>
<tr><td>WP_103506858.1</td><td>EACC1</td><td>TM+REase→ EACC1*→ CASPASE+??+SF1-helicase+Vsr(PDDEXK)?→</td><td>132</td><td>Streptomyces</td><td>GCA_001577385.1</td><td>actinobacteria</td></tr>
<tr><td>WP_103509194.1</td><td>EACC1</td><td>EACC1*→ CASPASE+??+SF1-helicase+Vsr(PDDEXK)?→</td><td>127</td><td>Streptomyces sp. SM13</td><td>GCF_002910875.1</td><td>actinobacteria</td></tr>
</tbody>
</table>

acc	architecture	operon	len	species	gca	taxend
WP_103512661.1	EACC1	EACC1*→ CASPASE+STAND+polar_region+TM+BetaPropeller→	157	Streptomyces sp. SM13	GCF_002910875.1	actinobacteria
WP_103780861.1	EACC1	EACC1*→ CASPASE+TPRs→ Clp_N+Clp_N+ClpABN-AAA→	74	Streptomyces sp. Ru71	GCF_002920535.1	actinobacteria
WP_103817220.1	EACC1	EACC1*→ CASPASE+TM?+BetaPropeller→ MDMPI_N+MDMPI_C→ META→	116	Streptomyces sp. Ru62	GCA_002920615.1	actinobacteria
WP_103832810.1	EACC1	EACC1*→	89	Streptomyces sp. Ru73	GCF_002920575.1	actinobacteria
WP_103908125.1	EACC1	EACC1*→ CASPASE+BetaPropeller→	121	Saccharopolyspora hirsuta	GCA_003053965.1	actinobacteria
WP_103922458.1	EACC1	RVT_1→?→ EACC1*→	138	Thiotrichales bacterium HS_08	GCA_900099115.2	gammaproteobacteria
WP_104380153.1	EACC1	EACC1*→ CASPASE+TPRs→	114	Nocardia nova	GCA_002933535.1	actinobacteria
WP_104477267.1	EACC1	EACC1*→ CASPASE+TPRs→	130	Actinokineospora auranticolor	GCA_002934265.1	actinobacteria
WP_104482422.1	EACC1	EACC1*→ CASPASE+TM+TM→ TM+PBPB→	115	Actinokineospora auranticolor	GCF_002934265.1	actinobacteria
WP_104482994.1	EACC1+CASPASE	EACC1+CASPASE*→?→ BetaPropeller→	201	Actinokineospora auranticolor	GCF_002934265.1	actinobacteria
WP_104790638.1	EACC1	EACC1*→ CASPASE+STAND+polar_region+TM+BetaPropeller→	115	Streptomyces sp. QL37	GCA_002941025.1	actinobacteria
WP_105971700.1	EACC1	EACC1*→ CASPASE+STAND+??+TPRs→	123	Streptomyces geranii	GCF_002954775.1	actinobacteria
WP_105975684.1	TPR_region+EACC1	TPR_region+EACC1*→	355	Streptomyces geranii	GCF_002954775.1	actinobacteria
WP_106126014.1	EACC1	EACC1*→ CASPASE+DUF179→ SIR2+??→??→	117	Pseudosporangium ferrugineum	GCA_003002065.1	actinobacteria
WP_106127751.1	EACC1	EACC1*→ CASPASE+TPRs→	113	Pseudosporangium ferrugineum	GCA_003002065.1	actinobacteria
WP_106188106.1	EACC1	EACC1*→ CASPASE+TM+TM+TM+TM→	117	Umezawaea tangerina	GCA_003002815.1	actinobacteria
WP_106243198.1	EACC1	EACC1*→ CASPASE+PBPB→	123	Nonomuraea fuscirosea	GCF_003001935.1	actinobacteria
WP_106318500.1	EACC1	Glyco_trans_4_4+UDPGT→ EACC1*→ CASPASE+PBPB+vWA→ TM+PBPB→	130	Actinoplanes italicus	GCF_003001815.1	actinobacteria
WP_106331136.1	EACC1+CASPASE+TPR_repeats+low_complexity+TM_region	EACC1+CASPASE+TPR_repeats+low_complexity+TM_region*→ STAND+TPRs→	854	Actinoplanes italicus	GCF_003001815.1	actinobacteria
WP_106677146.1	EACC1	EACC1*→ CASPASE+STAND+polar_region+TM+BetaPropeller→	139	Streptosporangium nondiastaticum	GCA_003011965.1	actinobacteria
WP_106956679.1	EACC1	EACC1*→ CASPASE+TM+TM+TM+TM→	116	Nocardia sp. MDA0666	GCA_003023875.1	actinobacteria
WP_106958984.1	EACC1	EACC1*→	138	Streptomyces	GCF_000156695.2	actinobacteria
WP_107079341.1	EACC1	EACC1*→	117	Micromonospora sp. MH33	GCA_003013775.1	actinobacteria
WP_107082547.1	EACC1	EACC1*→ CASPASE+STAND+polar_region+TM+BetaPropeller→	152	Streptomyces sp. Mg1	GCF_000412265.2	actinobacteria
WP_107089790.1	EACC1	EACC1*→ CASPASE+??+SF1-helicase+Vsr(PDDEXK)?→	133	Streptomyces sp. WM6373	GCF_001279685.1	actinobacteria
WP_107096587.1	EACC1	EACC1*→ CASPASE+TPRs→	126	Actinobacteria bacterium OK006	GCF_001298565.1	actinobacteria
WP_107103604.1	low_complexity+EACC1	low_complexity+EACC1*→ CASPASE+STAND+polar_region+TM+BetaPropeller→||←MFS_1	277	Streptomyces	GCF_001426405.1	actinobacteria
WP_107256709.1	EACC1	EACC1*→ CASPASE+TPRs→	124	Plantactinospora sp. BB1	GCA_003030385.1	actinobacteria
WP_107267269.1	EACC1	EACC1*→ CASPASE+TPRs→	124	Plantactinospora sp. BC1	GCA_003030345.1	actinobacteria
WP_107288274.1	EACC1	EACC1*→	134	Streptomyces sp. NBRC 110611	GCF_001590865.1	actinobacteria
WP_107450456.1	EACC1	EACC1*→ CASPASE+TPRs→	113	Streptomyces sp. LUP30	GCF_001746365.1	actinobacteria
WP_107456389.1	EACC1	EACC1*→ CASPASE+TM+TM+PBPB+vWA→	121	Streptomyces sp. A244	GCA_003041235.1	actinobacteria
WP_107458939.1	EACC1	EACC1*→ CASPASE+??→	117	Streptomyces sp. A244	GCA_003041235.1	actinobacteria
WP_107471257.1	EACC1	EACC1*→ CASPASE+STAND+polar_region+TM+BetaPropeller→	135	Streptomyces sp. MUSC 1	GCF_001866665.1	actinobacteria
WP_107475813.1	EACC1	EACC1*→ CASPASE+TM?+DUF3558+DUF3558→	145	Streptomyces sp. FBKL.4005	GCF_002251155.1	actinobacteria
WP_107657320.1	EACC1	EACC1*→ CASPASE+STAND+BetaPropeller+BetaPropeller→	128	Nocardia suismassiliense	GCF_900269665.1	actinobacteria
WP_108907227.1	EACC1	EACC1*→ CASPASE+STAND+??→	116	Streptomyces sp. HNM0039	GCA_003097515.1	actinobacteria
WP_109174941.1	EACC1	EACC1*→ CASPASE+STAND+polar_region+TM+BetaPropeller→	95	Streptomyces sp. CS147	GCA_003116775.1	actinobacteria
WP_109185243.1	EACC1	EACC1*→ CASPASE+STAND+polar_region+TM+BetaPropeller→	157	Streptomyces sp. CS081A	GCA_003116715.1	actinobacteria
WP_109279769.1	EACC1	EACC1*→ CASPASE+STAND+polar_region+TM+BetaPropeller→	130	Streptomyces orinoci	GCF_003121295.1	actinobacteria
WP_109321412.1	EACC1	EACC1*→ CASPASE+PBPB+vWA→	149	Streptomyces xinghaiensis	GCF_001756925.1	actinobacteria
WP_109378190.1	EACC1	EACC1*→ CASPASE+STAND+polar_region+TM+BetaPropeller→	161	Streptomyces sp. NWU339	GCA_003143855.1	actinobacteria
WP_109494743.1	EACC1	EACC1*→ CASPASE+TPRs→	120	Streptomyces sp. Act143	GCA_003143935.1	actinobacteria
WP_109508239.1	EACC1	EACC1*→	121	Nocardioides sp. YIM ART13	GCF_003121585.1	actinobacteria
WP_109530806.1	EACC1+CASPASE	EACC1+CASPASE*→	475	Nocardia sp. SYSU K10002	GCF_003123685.1	actinobacteria
WP_109593304.1	EACC1	EACC1*→ CASPASE+PBPB+vWA→ TM+PBPB→	140	Actinoplanes xinjiangensis	GCA_003148685.1	actinobacteria
WP_109638580.1	EACC1+CASPASE+TPR_repeats+low_complexity+TM_region	TM+TM+BPD_transp_1→ OppC_N+TM+TM+BPD_transp_1→ ABC-ATPase+ABC-ATPase→ PBPB→ TM+Beta_rich+TM+Beta_rich+TM+TM→ EACC1+CASPASE+TPR_repeats+low_complexity+TM_region*→	802	Lechevalieria deserti	GCA_003148865.1	actinobacteria
WP_109815933.1	EACC1	TM+TM+TM+Peptidase_M48+TM+DUF4864+??→ EACC1*→	131	Micromonospora sp. 5R2A7	GCA_003172955.1	actinobacteria
WP_109818808.1	EACC1	EACC1*→	131	Micromonospora sp. 5R2A7	GCA_003172955.1	actinobacteria
WP_109867070.1	EACC1	EACC1*→	116	Escherichia coli	GCA_006471505.1	gammaproteobacteria

acc	architecture	operon	len	species	gca	taxend
WP_109888687.1	EACC1	EACC1*→ CASPASE+FGS_fold-domain→??→??→ TM+TM+BaxI_1→?→ NUDIX→	134	Streptomyces sp. NEAU-S7GS2	GCA_003173275.1	actinobacteria
WP_109891221.1	EACC1	EACC1*→ CASPASE+Beta_rich_repeat→	155	Streptomyces sp. NEAU-S7GS2	GCA_003173275.1	actinobacteria
WP_109893417.1	EACC1	EACC1*→ CASPASE+??+SF1-helicase+Vsr(PDDEXK)?→	138	Streptomyces sp. NEAU-S7GS2	GCA_003173275.1	actinobacteria
WP_109899578.1	EACC1	TM+TM+TM+Peptidase_M48+TM+DUF4864+??→ EACC1*→ TPRs+CASPASE→	133	Micromonospora sp. S4605	GCA_003176715.1	actinobacteria
WP_109914226.1	EACC1	EACC1*→ CASPASE+TPRs??+GHKL_ATPase+TPRs??→	107	Streptomyces sp. ZEA17I	GCA_003173635.1	actinobacteria
WP_110036763.1	EACC1	EACC1*→	78	Nocardia neocaledoniensis	GCF_003182135.1	actinobacteria
WP_110040833.1	EACC1	DUF742→ EACC1*→ CASPASE+??→	125	Nocardia neocaledoniensis	GCA_003182135.1	actinobacteria

III. Source data - profile-profile comparisons

Comparisons between poorly-understood components of the FtsH- and beta-propeller-containing systems. All comparisons involve construction of a multiple sequence alignment, which is used as the query for profile generation and subsequent comparison with profile databases (including pfam and pdb) using the hhpred program. All significant (< 0.05) hits are reported.

domain query	profile hit	e-value
bpX2	fvmNEFF1-Nterm	8.6e-19
bpX2	fvmX7	1.9e-06
bpX2	bpX5	0.0072
bpX3	fvmX7	0.00036
bpX3	fvmNEFF1-Nterm	0.0032
bpX3	bpX2	0.0051
bpX5	bpX2	0.021
bpX5	fvmNEFF1-Nterm	0.038
fvmX2	fvmX5	4.6e-38
fvmX7	fvmNEFF1-Nterm	2.5e-29
fvmX7	bpX2	0.00012
fvmX5	fvmX2	9.2e-07
fvmX3-analog	fvmX3	0.046
fvmNEFF1-Nterm	bpX2	9e-19
fvmNEFF1-Nterm	fvmX7	5.5e-12

Comparisons between distinct EAD domains and other domains of interest. See above for methodology.

domain query	profile hit	p-value
EAD7	EAD6	2.1e-07
EAD7	EAD1	8.9e-07
EAD7	bDLD2	0.00015
EAD6	EAD7	1.7e-09
EAD6	EAD1	2.3e-07
EAD6	CARD	5.2e-05
EAD1	EAD7	7.1e-09
EAD1	EAD8	1.6e-06
EAD1	bDLD1	8.8e-06
EAD1	EAD6	1.5e-05
EAD1	EAD2	0.00028
EAD8	EAD2	1.3e-06
EAD8	bDLD2	2.5e-06
EAD8	EAD5	4.3e-06
EAD8	EAD1	7.7e-06
EAD2	EAD8	1e-06
EAD2	EAD5	0.00045
EAD2	EAD7	0.00052
EAD5	EAD8	0.00026
EAD5	EAD1	0.00077
bDLD1	EAD1	2.1e-05
bDLD1	DEATH(3OQ9E)	0.00032
bDLD2	EAD8	1.6e-05
bDLD2	CARD(2NZ7B)	0.0005

IV. Source data - pairwise systems comparison in same genomes

system vs system	ov.lap #	fraction
VMAP vs iSTAND	13	0.0317
VMAP vs FtsH	16	0.0403
VMAP vs beta propeller	6	0.0144
VMAP vs GAP1-N1	8	0.0164
VMAP vs GAP1-N2	214	0.2642
VMAP vs NucA	1	0.0026
VMAP vs EACC1	305	0.4398
iSTAND vs FtsH	2	0.022
iSTAND vs beta propeller	4	0.0362
iSTAND vs GAP1-N1	4	0.0219
iSTAND vs GAP1-N2	1	0.002
iSTAND vs NucA	1	0.0135
iSTAND vs EACC1	2	0.0052
FtsH vs beta propeller	17	0.1726
FtsH vs GAP1-N1	2	0.0117
FtsH vs GAP1-N2	13	0.0264
FtsH vs NucA	0	0
FtsH vs EACC1	13	0.0347
beta propeller vs GAP1-N1	5	0.0263
beta propeller vs GAP1-N2	13	0.0254
beta propeller vs NucA	0	0
beta propeller vs EACC1	5	0.0127
GAP1-N1 vs GAP1-N2	4	0.0069
GAP1-N1 vs NucA	2	0.013
GAP1-N1 vs EACC1	8	0.0171
GAP1-N2 vs NucA	1	0.0021
GAP1-N2 vs EACC1	168	0.2133
NucA vs EACC1	0	0

V. Source data - organisms with multiple systems in same genomes

- **VMAP vs iSTAND**:

species	multicellularity
[Scytonema hofmanni] UTEX B 1581	TRUE
Aphanocapsa montana BDHKU210001	TRUE
Calothrix sp. NIES-2098	TRUE
Calothrix sp. PCC 6303	TRUE
Calothrix sp. PCC 7103	TRUE
Fischerella sp. PCC 9431	TRUE
Geitlerinema sp. PCC 7105	TRUE
Mastigocoleus testarum BC008	TRUE
Nostoc punctiforme PCC 73102	TRUE
Rivularia sp. PCC 7116	TRUE
Scytonema sp. HK-05	TRUE
Trichodesmium erythraeum	TRUE
Trichodesmium erythraeum IMS101	TRUE

- **VMAP vs FtsH**:

species	multicellularity
Actinoplanes globisporus	TRUE
Amycolatopsis balhimycina	TRUE
Archangium gephyra	TRUE
Calothrix parietina	TRUE
Frankia sp. QA3	TRUE
Gemmata sp. SH-PL17	FALSE
Moorea producens	TRUE
Streptomyces durhamensis	TRUE
Streptomyces griseus	TRUE
Streptomyces mirabilis	TRUE
Streptomyces puniciscabiei	TRUE
Streptomyces sp. GKU 895	TRUE
Streptomyces sp. MMG1121	TRUE
Streptomyces sp. MUSC 1	TRUE
Streptomyces sp. MUSC 14	TRUE
Streptomyces yokosukanensis	TRUE

- **VMAP vs beta propeller**:

species	multicellularity
Actinoplanes globisporus	TRUE
Frankia sp. QA3	TRUE
Gemmata sp. SH-PL17	FALSE
Streptomyces scabiei	TRUE
Streptomyces sp. MMG1121	TRUE
Streptomyces sp. NRRL F-6131	TRUE

- **VMAP vs GAP1-N1**:

species	multicellularity
Actinomadura sp. CNU-125	TRUE
Archangium violaceum	TRUE
Burkholderia cepacia	FALSE
Burkholderia pseudomallei	FALSE
Pseudomonas	FALSE
Pseudomonas fluorescens	FALSE
Streptomyces canus	TRUE
Streptomyces viridochromogenes	TRUE

- **VMAP vs GAP1-N2**:

species	multicellularity
Actinobacteria bacterium OV450	NA
Actinokineospora inagensis	TRUE
Actinokineospora terrae	TRUE
Actinomadura atramentaria	TRUE
Actinomadura chibensis	TRUE
Actinomadura echinospora	TRUE
Actinomadura formosensis	TRUE
Actinomadura kijaniata	TRUE
Actinomadura latina	TRUE
Actinomadura macra	TRUE
Actinomadura madurae	TRUE
Actinomadura rifamycini	TRUE
Actinoplanes globisporus	TRUE
Actinoplanes rectilineatus	TRUE
Actinoplanes subtropicus	TRUE
Actinoplanes utahensis	TRUE
Amycolatopsis alba	TRUE
Amycolatopsis coloradensis	TRUE
Amycolatopsis rifamycinica	TRUE
Amycolatopsis sp. CB00013	TRUE

species	multicellularity
Beggiatoa sp. 4572_84	TRUE
Frankia sp. CcI49	TRUE
Frankia sp. EAN1pec	TRUE
Frankia sp. EUN1f	TRUE
Frankia sp. G2	TRUE
Frankia sp. QA3	TRUE
Frankia sp. R43	TRUE
Gemmata sp. SH-PL17	FALSE
Hamadaea tsunoensis	TRUE
Kibdelosporangium aridum	TRUE
Kibdelosporangium phytohabitans	TRUE
Kitasatospora azatica	TRUE
Lamprocystis purpurea	TRUE
Lechevalieria aerocolonigenes	TRUE
Lentzea guizhouensis	TRUE
Microcystis sp. T1-4	TRUE
Micromonospora carbonacea	TRUE
Micromonospora echinofusca	TRUE
Micromonospora haikouensis	TRUE
Microtetraspora glauca	TRUE
Mycobacterium tuberculosis	FALSE
Nocardia sp. NRRL S-836	TRUE
Nocardiopsis dassonvillei	TRUE
Nocardiopsis flavescens	TRUE
Nocardiopsis ganjiahuensis	TRUE
Nocardiopsis halotolerans	TRUE
Nocardiopsis kunsanensis	TRUE
Nocardiopsis sinuspersici	TRUE
Nocardiopsis sp. CNS-639	TRUE
Nocardiopsis sp. TSRI0078	TRUE
Nocardiopsis synnemataformans	TRUE
Nocardiopsis xinjiangensis	TRUE
Nonomuraea solani	TRUE
Saccharopolyspora spinosa	TRUE
Saccharothrix sp. ALI-22-I	TRUE
Saccharothrix sp. CB00851	TRUE
Saccharothrix sp. NRRL B-16314	TRUE
Streptomyces	TRUE
Streptomyces albidoflavus	TRUE
Streptomyces albus	TRUE
Streptomyces albus J1074	TRUE
Streptomyces antibioticus	TRUE
Streptomyces anulatus	TRUE
Streptomyces atratus	TRUE

species	multicellularity
Streptomyces atroolivaceus	TRUE
Streptomyces aurantiacus	TRUE
Streptomyces aurantiacus JA 4570	TRUE
Streptomyces baarnensis	TRUE
Streptomyces bicolor	TRUE
Streptomyces bingchenggensis	TRUE
Streptomyces bingchenggensis BCW-1	TRUE
Streptomyces caatingaensis	TRUE
Streptomyces cellostaticus	TRUE
Streptomyces cellulosae	TRUE
Streptomyces curacoi	TRUE
Streptomyces europaeiscabiei	TRUE
Streptomyces filamentosus	TRUE
Streptomyces fulvoviolaceus	TRUE
Streptomyces globisporus	TRUE
Streptomyces griseus	TRUE
Streptomyces griseus subsp. griseus NBRC 13350	TRUE
Streptomyces himastatinicus ATCC 53653	TRUE
Streptomyces hokutonensis	TRUE
Streptomyces kanamyceticus	TRUE
Streptomyces katrae	TRUE
Streptomyces lavendulae	TRUE
Streptomyces lushanensis	TRUE
Streptomyces luteocolor	TRUE
Streptomyces mediolani	TRUE
Streptomyces mirabilis	TRUE
Streptomyces misionensis	TRUE
Streptomyces nanshensis	TRUE
Streptomyces niveus	TRUE
Streptomyces qinglanensis	TRUE
Streptomyces regensis	TRUE
Streptomyces reticuli	TRUE
Streptomyces rimosus	TRUE
Streptomyces roseochromogenus	TRUE
Streptomyces roseochromogenus subsp. oscitans DS 12.976	TRUE
Streptomyces sampsonii	TRUE
Streptomyces scopuliridis	TRUE
Streptomyces silaceus	TRUE
Streptomyces sp. 136MFCol5.1	TRUE
Streptomyces sp. 150FB	TRUE

species	multicellularity
Streptomyces sp. 2131.1	TRUE
Streptomyces sp. AA1529	TRUE
Streptomyces sp. AcH 505	TRUE
Streptomyces sp. ACT-1	TRUE
Streptomyces sp. ATexAB-D23	TRUE
Streptomyces sp. AW19M42	TRUE
Streptomyces sp. BF-3	TRUE
Streptomyces sp. BpilaLS-43	TRUE
Streptomyces sp. C	TRUE
Streptomyces sp. CB00072	TRUE
Streptomyces sp. CB00316	TRUE
Streptomyces sp. CB01249	TRUE
Streptomyces sp. CB01580	TRUE
Streptomyces sp. CB02009	TRUE
Streptomyces sp. CB02058	TRUE
Streptomyces sp. CB02115	TRUE
Streptomyces sp. CB02366	TRUE
Streptomyces sp. CB02460	TRUE
Streptomyces sp. CB02488	TRUE
Streptomyces sp. CB02923	TRUE
Streptomyces sp. CcalMP-8W	TRUE
Streptomyces sp. cf386	TRUE
Streptomyces sp. CFMR 7	TRUE
Streptomyces sp. Cmuel-A718b	TRUE
Streptomyces sp. CNB091	TRUE
Streptomyces sp. CNH099	TRUE
Streptomyces sp. CNQ431	TRUE
Streptomyces sp. CNQ-525	TRUE
Streptomyces sp. CNQ766	TRUE
Streptomyces sp. CNQ865	TRUE
Streptomyces sp. CNS335	TRUE
Streptomyces sp. CNT318	TRUE
Streptomyces sp. CNT371	TRUE
Streptomyces sp. CNY228	TRUE
Streptomyces sp. CNY243	TRUE
Streptomyces sp. DI166	TRUE
Streptomyces sp. DvalAA-19	TRUE
Streptomyces sp. DvalAA-43	TRUE
Streptomyces sp. EN16	TRUE
Streptomyces sp. EN23	TRUE
Streptomyces sp. EN27	TRUE
Streptomyces sp. F-1	TRUE
Streptomyces sp. FR-008	TRUE
Streptomyces sp. FXJ1.172	TRUE

species	multicellularity
Streptomyces sp. GBA 94-10	TRUE
Streptomyces sp. HGB0020	TRUE
Streptomyces sp. IMTB 2501	TRUE
Streptomyces sp. KE1	TRUE
Streptomyces sp. LaPpAH-202	TRUE
Streptomyces sp. M10	TRUE
Streptomyces sp. MnatMP-M17	TRUE
Streptomyces sp. MnatMP-M77	TRUE
Streptomyces sp. MNU77	TRUE
Streptomyces sp. MP131-18	TRUE
Streptomyces sp. MUSC 1	TRUE
Streptomyces sp. NBRC 110465	TRUE
Streptomyces sp. Ncost-T10-10d	TRUE
Streptomyces sp. Ncost-T6T-1	TRUE
Streptomyces sp. NRRL B-1347	TRUE
Streptomyces sp. NRRL B-24720	TRUE
Streptomyces sp. NRRL B-3253	TRUE
Streptomyces sp. NRRL F-2747	TRUE
Streptomyces sp. NRRL F-5053	TRUE
Streptomyces sp. NRRL F-5126	TRUE
Streptomyces sp. NRRL F-5193	TRUE
Streptomyces sp. NRRL F-5630	TRUE
Streptomyces sp. NRRL F-5727	TRUE
Streptomyces sp. NRRL F-6628	TRUE
Streptomyces sp. NRRL S-1824	TRUE
Streptomyces sp. NRRL S-1868	TRUE
Streptomyces sp. NRRL S-237	TRUE
Streptomyces sp. NRRL S-241	TRUE
Streptomyces sp. NRRL S-244	TRUE
Streptomyces sp. NRRL S-444	TRUE
Streptomyces sp. NRRL WC-3549	TRUE
Streptomyces sp. NRRL WC-3723	TRUE
Streptomyces sp. NRRL WC-3725	TRUE
Streptomyces sp. NRRL WC-3744	TRUE
Streptomyces sp. NTK 937	TRUE
Streptomyces sp. ok210	TRUE
Streptomyces sp. or20	TRUE
Streptomyces sp. OspMP-M43	TRUE
Streptomyces sp. OspMP-M45	TRUE
Streptomyces sp. PBH53	TRUE
Streptomyces sp. PTY087I2	TRUE
Streptomyces sp. PVA 94-07	TRUE
Streptomyces sp. RTd22	TRUE
Streptomyces sp. RV15	TRUE

species	multicellularity
Streptomyces sp. S4	TRUE
Streptomyces sp. SA3_actG	TRUE
Streptomyces sp. ScaeMP-6W	TRUE
Streptomyces sp. SCSIO 03032	TRUE
Streptomyces sp. SolWspMP-5a-2	TRUE
Streptomyces sp. TSRI0261	TRUE
Streptomyces sp. TSRI0281	TRUE
Streptomyces sp. TSRI0395	TRUE
Streptomyces sp. TSRI0445	TRUE
Streptomyces sp. Tu6071	TRUE
Streptomyces sp. TverLS-915	TRUE
Streptomyces sp. W007	TRUE
Streptomyces sp. XY332	TRUE
Streptomyces specialis	TRUE
Streptomyces tsukubensis	TRUE
Streptomyces violaceoruber	TRUE
Streptomyces virginiae	TRUE
Streptomyces vitaminophilus	TRUE
Streptomyces wadayamensis	TRUE
Streptomyces xylophagus	TRUE
Streptomyces yerevanensis	TRUE
Thermomonospora curvata	TRUE

- **VMAP vs NucA**:

species	multicellularity
Bradyrhizobium	TRUE

- **VMAP vs EACC1**:

species	multicellularity
Actinobacteria bacterium OK006	NA
Actinobacteria bacterium OK074	NA
Actinobacteria bacterium OV450	NA
Actinokineospora inagensis	TRUE
Actinokineospora terrae	TRUE
Actinomadura madurae	TRUE
Actinophytocola xinjiangensis	TRUE
Actinoplanes awajinensis subsp. mycoplanecinus	TRUE
Actinoplanes globisporus	TRUE
Actinoplanes philippinensis	TRUE

species	multicellularity
Actinoplanes rectilineatus	TRUE
Actinoplanes sp. SE50/110	TRUE
Actinoplanes utahensis	TRUE
Actinospica robiniae	TRUE
Actinosporangium sp. NRRL B-3428	TRUE
Actinosynnema sp. ALI-1.44	TRUE
Amycolatopsis alba	TRUE
Amycolatopsis australiensis	TRUE
Amycolatopsis azurea	TRUE
Amycolatopsis balhimycina	TRUE
Amycolatopsis coloradensis	TRUE
Amycolatopsis decaplanina	TRUE
Amycolatopsis japonica	TRUE
Amycolatopsis keratiniphila	TRUE
Amycolatopsis mediterranei	TRUE
Amycolatopsis orientalis	TRUE
Amycolatopsis pretoriensis	TRUE
Amycolatopsis regifaucium	TRUE
Amycolatopsis rifamycinica	TRUE
Amycolatopsis sp. CB00013	TRUE
Amycolatopsis sp. MJM2582	TRUE
Amycolatopsis tolypomycina	TRUE
Amycolatopsis vancoresmycina	TRUE
Amycolatopsis xylanica	TRUE
Catenulispora acidiphila	TRUE
Catenuloplanes japonicus	TRUE
Frankia alni	TRUE
Frankia inefficax	TRUE
Frankia sp. ACN1ag	TRUE
Frankia sp. AvcI1	TRUE
Frankia sp. EAN1pec	TRUE
Frankia sp. G2	TRUE
Hamadaea tsunoensis	TRUE
Kibdelosporangium phytohabitans	TRUE
Kibdelosporangium sp. MJ126-NF4	TRUE
Kitasatospora	TRUE
Kitasatospora azatica	TRUE
Kitasatospora cheerisanensis	TRUE
Kitasatospora sp. CB02891	TRUE
Kitasatospora sp. MBT66	TRUE
Kitasatospora sp. NRRL B-11411	TRUE
Kitasatospora sp. Root107	TRUE

species	multicellularity
Kutzneria albida	TRUE
Kutzneria albida DSM 43870	TRUE
Lechevalieria aerocolonigenes	TRUE
Lechevalieria fradiae	TRUE
Lechevalieria xinjiangensis	TRUE
Lentzea albida	TRUE
Lentzea albidocapillata	TRUE
Lentzea flaviverrucosa	TRUE
Lentzea violacea	TRUE
Lentzea waywayandensis	TRUE
Longispora albida	TRUE
Micromonospora carbonacea	TRUE
Micromonospora matsumotoense	TRUE
Micromonospora parva	TRUE
Micromonospora sp. HK10	TRUE
Micromonospora sp. NBS 11-29	TRUE
Micromonospora sp. NRRL B-16802	TRUE
Microtetraspora glauca	TRUE
Nocardia mexicana	TRUE
Nocardia sp. NRRL S-836	TRUE
Nocardiopsis dassonvillei	TRUE
Nocardiopsis flavescens	TRUE
Nocardiopsis potens	TRUE
Nocardiopsis salina	TRUE
Nocardiopsis sp. CNR-923	TRUE
Nonomuraea solani	TRUE
Pseudonocardia acaciae	TRUE
Saccharopolyspora spinosa	TRUE
Saccharothrix espanaensis	TRUE
Streptomyces	TRUE
Streptomyces acidiscabies	TRUE
Streptomyces agglomeratus	TRUE
Streptomyces albus	TRUE
Streptomyces albus subsp. albus	TRUE
Streptomyces alni	TRUE
Streptomyces ambofaciens	TRUE
Streptomyces antibioticus	TRUE
Streptomyces antioxidans	TRUE
Streptomyces anulatus	TRUE
Streptomyces atratus	TRUE
Streptomyces atriruber	TRUE
Streptomyces aurantiacus	TRUE
Streptomyces aurantiacus JA 4570	TRUE

species	multicellularity
Streptomyces autolyticus	TRUE
Streptomyces bicolor	TRUE
Streptomyces caatingaensis	TRUE
Streptomyces caeruleatus	TRUE
Streptomyces canus	TRUE
Streptomyces cattleya	TRUE
Streptomyces cellostaticus	TRUE
Streptomyces cellulosae	TRUE
Streptomyces chartreusis	TRUE
Streptomyces chattanoogensis	TRUE
Streptomyces clavuligerus	TRUE
Streptomyces coelicolor	TRUE
Streptomyces corchorusii	TRUE
Streptomyces cyaneogriseus	TRUE
Streptomyces cyaneogriseus subsp. noncyanogenus	TRUE
Streptomyces decoyicus	TRUE
Streptomyces diastatochromogenes	TRUE
Streptomyces durhamensis	TRUE
Streptomyces erythrochromogenes	TRUE
Streptomyces europaeiscabiei	TRUE
Streptomyces exfoliatus	TRUE
Streptomyces filamentosus	TRUE
Streptomyces flavidovirens	TRUE
Streptomyces flavochromogenes	TRUE
Streptomyces fradiae	TRUE
Streptomyces fulvoviolaceus	TRUE
Streptomyces gilvosporeus	TRUE
Streptomyces glaucescens	TRUE
Streptomyces griseochromogenes	TRUE
Streptomyces griseofuscus	TRUE
Streptomyces griseus	TRUE
Streptomyces guanduensis	TRUE
Streptomyces himastatinicus	TRUE
Streptomyces himastatinicus ATCC 53653	TRUE
Streptomyces hokutonensis	TRUE
Streptomyces humi	TRUE
Streptomyces hygroscopicus	TRUE
Streptomyces iakyrus	TRUE
Streptomyces incarnatus	TRUE
Streptomyces iranensis	TRUE
Streptomyces katrae	TRUE

species	multicellularity
Streptomyces longwoodensis	TRUE
Streptomyces lushanensis	TRUE
Streptomyces lydicus	TRUE
Streptomyces mangrovisoli	TRUE
Streptomyces melanosporofaciens	TRUE
Streptomyces mirabilis	TRUE
Streptomyces misionensis	TRUE
Streptomyces monomycini	TRUE
Streptomyces nanshensis	TRUE
Streptomyces natalensis	TRUE
Streptomyces niveus	TRUE
Streptomyces niveus NCIMB 11891	TRUE
Streptomyces nodosus	TRUE
Streptomyces olindensis	TRUE
Streptomyces pactum	TRUE
Streptomyces paucisporeus	TRUE
Streptomyces platensis	TRUE
Streptomyces pristinaespiralis	TRUE
Streptomyces pristinaespiralis ATCC 25486	TRUE
Streptomyces puniciscabiei	TRUE
Streptomyces purpureus	TRUE
Streptomyces rapamycinicus NRRL 5491	TRUE
Streptomyces regalis	TRUE
Streptomyces regensis	TRUE
Streptomyces reticuli	TRUE
Streptomyces rimosus	TRUE
Streptomyces roseochromogenus	TRUE
Streptomyces roseochromogenus subsp. oscitans DS 12.976	TRUE
Streptomyces roseosporus NRRL 15998	TRUE
Streptomyces roseus	TRUE
Streptomyces scabiei	TRUE
Streptomyces scabrisporus	TRUE
Streptomyces sclerotialus	TRUE
Streptomyces scopuliridis	TRUE
Streptomyces silvensis	TRUE
Streptomyces sp. 2112.3	TRUE
Streptomyces sp. 2131.1	TRUE
Streptomyces sp. 4F	TRUE
Streptomyces sp. ACT-1	TRUE

species	multicellularity
Streptomyces sp. Ag82_O1-15	TRUE
Streptomyces sp. AS58	TRUE
Streptomyces sp. ATexAB-D23	TRUE
Streptomyces sp. B9173	TRUE
Streptomyces sp. BoleA5	TRUE
Streptomyces sp. BpilaLS-43	TRUE
Streptomyces sp. CB00072	TRUE
Streptomyces sp. CB00455	TRUE
Streptomyces sp. CB01249	TRUE
Streptomyces sp. CB02058	TRUE
Streptomyces sp. CB02115	TRUE
Streptomyces sp. CB02400	TRUE
Streptomyces sp. CB02414	TRUE
Streptomyces sp. CB02488	TRUE
Streptomyces sp. CB02923	TRUE
Streptomyces sp. CB03234	TRUE
Streptomyces sp. CB03238	TRUE
Streptomyces sp. CC71	TRUE
Streptomyces sp. CcalMP-8W	TRUE
Streptomyces sp. CCM_MD2014	TRUE
Streptomyces sp. cf386	TRUE
Streptomyces sp. CNB091	TRUE
Streptomyces sp. CNQ329	TRUE
Streptomyces sp. CNQ431	TRUE
Streptomyces sp. CNQ-509	TRUE
Streptomyces sp. CNQ-525	TRUE
Streptomyces sp. CNQ865	TRUE
Streptomyces sp. CNS335	TRUE
Streptomyces sp. CNS606	TRUE
Streptomyces sp. CNT371	TRUE
Streptomyces sp. CNY228	TRUE
Streptomyces sp. CNY243	TRUE
Streptomyces sp. DSM 15324	TRUE
Streptomyces sp. DvalAA-14	TRUE
Streptomyces sp. EN16	TRUE
Streptomyces sp. EN27	TRUE
Streptomyces sp. ERV7	TRUE
Streptomyces sp. F-1	TRUE
Streptomyces sp. F-3	TRUE
Streptomyces sp. FxanaC1	TRUE
Streptomyces sp. GKU 895	TRUE
Streptomyces sp. HGB0020	TRUE
Streptomyces sp. IMTB 2501	TRUE
Streptomyces sp. LaPpAH-108	TRUE

species	multicellularity
Streptomyces sp. LUP30	TRUE
Streptomyces sp. M1013	TRUE
Streptomyces sp. MBT28	TRUE
Streptomyces sp. Mg1	TRUE
Streptomyces sp. MJM1172	TRUE
Streptomyces sp. MMG1533	TRUE
Streptomyces sp. MnatMP-M17	TRUE
Streptomyces sp. MNU77	TRUE
Streptomyces sp. MOE7	TRUE
Streptomyces sp. MP131-18	TRUE
Streptomyces sp. MspMP-M5	TRUE
Streptomyces sp. MUSC 1	TRUE
Streptomyces sp. MUSC 14	TRUE
Streptomyces sp. MUSC 93	TRUE
Streptomyces sp. NBRC 109706	TRUE
Streptomyces sp. NBRC 110027	TRUE
Streptomyces sp. NBRC 110028	TRUE
Streptomyces sp. NBRC 110465	TRUE
Streptomyces sp. NBRC 110611	TRUE
Streptomyces sp. Ncost-T10-10d	TRUE
Streptomyces sp. NRRL B-24720	TRUE
Streptomyces sp. NRRL B-3648	TRUE
Streptomyces sp. NRRL F-2580	TRUE
Streptomyces sp. NRRL F-2747	TRUE
Streptomyces sp. NRRL F-2890	TRUE
Streptomyces sp. NRRL F-4474	TRUE
Streptomyces sp. NRRL F-5053	TRUE
Streptomyces sp. NRRL F-5123	TRUE
Streptomyces sp. NRRL F-5126	TRUE
Streptomyces sp. NRRL F-5727	TRUE
Streptomyces sp. NRRL F-6131	TRUE
Streptomyces sp. NRRL S-1022	TRUE
Streptomyces sp. NRRL S-1448	TRUE
Streptomyces sp. NRRL S-1813	TRUE
Streptomyces sp. NRRL S-1824	TRUE
Streptomyces sp. NRRL S-1868	TRUE
Streptomyces sp. NRRL S-244	TRUE
Streptomyces sp. NRRL S-31	TRUE
Streptomyces sp. NRRL S-337	TRUE
Streptomyces sp. NRRL S-340	TRUE
Streptomyces sp. NRRL S-384	TRUE
Streptomyces sp. NRRL S-495	TRUE
Streptomyces sp. NRRL S-646	TRUE
Streptomyces sp. NRRL S-813	TRUE

species	multicellularity
Streptomyces sp. NRRL S-87	TRUE
Streptomyces sp. NRRL WC-3549	TRUE
Streptomyces sp. NRRL WC-3618	TRUE
Streptomyces sp. NRRL WC-3626	TRUE
Streptomyces sp. NRRL WC-3742	TRUE
Streptomyces sp. NTK 937	TRUE
Streptomyces sp. or20	TRUE
Streptomyces sp. OV198	TRUE
Streptomyces sp. PAN_FS17	TRUE
Streptomyces sp. PBH53	TRUE
Streptomyces sp. PpalLS-921	TRUE
Streptomyces sp. PRh5	TRUE
Streptomyces sp. PTY087I2	TRUE
Streptomyces sp. RTd22	TRUE
Streptomyces sp. Ru87	TRUE
Streptomyces sp. S10(2016)	TRUE
Streptomyces sp. SceaMP-e96	TRUE
Streptomyces sp. SCSIO 03032	TRUE
Streptomyces sp. SPMA113	TRUE
Streptomyces sp. TLI_053	TRUE
Streptomyces sp. TN58	TRUE
Streptomyces sp. TOR3209	TRUE
Streptomyces sp. TSRI0107	TRUE
Streptomyces sp. TSRI0261	TRUE
Streptomyces sp. TSRI0281	TRUE
Streptomyces sp. URHA0041	TRUE
Streptomyces sp. W007	TRUE
Streptomyces sp. WM4235	TRUE
Streptomyces sp. WM6372	TRUE
Streptomyces sp. WM6373	TRUE
Streptomyces sp. XY332	TRUE
Streptomyces sp. XY431	TRUE
Streptomyces sp. XY593	TRUE
Streptomyces tsukubensis	TRUE
Streptomyces varsoviensis	TRUE
Streptomyces violaceoruber	TRUE
Streptomyces violens	TRUE
Streptomyces vitaminophilus	TRUE
Streptomyces xiamenensis	TRUE
Streptomyces xylophagus	TRUE
Streptomyces yanglinensis	TRUE
Streptomyces yokosukanensis	TRUE

- **iSTAND vs FtsH**:

species	multicellularity
Candidatus Magnetoglobus multicellularis str. Araruama	TRUE
Gammaproteobacteria bacterium	NA

- **iSTAND vs beta propeller**:

species	multicellularity
Candidatus Magnetoglobus multicellularis str. Araruama	TRUE
Candidatus Thiomargarita nelsonii	TRUE
Gammaproteobacteria bacterium	NA
Haliscomenobacter hydrossis DSM 1100	TRUE

- **iSTAND vs GAP1-N1**:

species	multicellularity
Bradyrhizobium japonicum	TRUE
Candidatus Accumulibacter sp. SK-01	FALSE
Candidatus Magnetoglobus multicellularis str. Araruama	TRUE
Gammaproteobacteria bacterium	NA

- **iSTAND vs GAP1-N2**:

species	multicellularity
Candidatus Thiomargarita nelsonii	TRUE

- **iSTAND vs NucA**:

species	multicellularity
Bradyrhizobium japonicum	TRUE

- **iSTAND vs EACC1**:

species	multicellularity
Beggiatoa sp. PS	TRUE
Thiotrichales bacterium HS_08	TRUE

- **FtsH vs beta propeller**:

species	multicellularity
Actinoplanes derwentensis	TRUE
Actinoplanes globisporus	TRUE
Amycolatopsis sp. H5	TRUE
Candidatus Magnetoglobus multicellularis str. Araruama	TRUE
Dactylosporangium aurantiacum	TRUE
Fimbriiglobus ruber	TRUE
Frankia coriariae	TRUE
Frankia sp. QA3	TRUE
Gammaproteobacteria bacterium	NA
Gemmata sp. SH-PL17	FALSE
Haliangium ochraceum	TRUE
Nostoc sp. T09	TRUE
Sinosporangium album	TRUE
Streptomyces sp. GGCR-6	TRUE
Streptomyces sp. MMG1121	TRUE
Thiohalocapsa sp. ML1	TRUE
Zavarzinella formosa	TRUE

- **FtsH vs GAP1-N1**:

species	multicellularity
Candidatus Magnetoglobus multicellularis str. Araruama	TRUE
Gammaproteobacteria bacterium	NA

- **FtsH vs GAP1-N2**:

species	multicellularity
Actinoplanes globisporus	TRUE
Dactylosporangium aurantiacum	TRUE
Fimbriiglobus ruber	TRUE

species	multicellularity
Frankia sp. QA3	TRUE
Gemmata obscuriglobus	FALSE
Gemmata sp. SH-PL17	FALSE
Isosphaera pallida	TRUE
Streptomyces griseus	TRUE
Streptomyces mirabilis	TRUE
Streptomyces sp. MUSC 1	TRUE
Streptomyces sp. WM6386	TRUE
Thiohalocapsa sp. ML1	TRUE
Zavarzinella formosa	TRUE

- **FtsH vs NucA**:

**species**	**multicellularity**

- **FtsH vs EACC1**:

species	multicellularity
Actinoplanes derwentensis	TRUE
Actinoplanes globisporus	TRUE
Amycolatopsis balhimycina	TRUE
Amycolatopsis sp. H5	TRUE
Streptomyces durhamensis	TRUE
Streptomyces griseus	TRUE
Streptomyces mirabilis	TRUE
Streptomyces puniciscabiei	TRUE
Streptomyces sp. Act143	TRUE
Streptomyces sp. GKU 895	TRUE
Streptomyces sp. MUSC 1	TRUE
Streptomyces sp. MUSC 14	TRUE
Streptomyces yokosukanensis	TRUE

- **beta propeller vs GAP1-N1**:

species	multicellularity
Candidatus Magnetoglobus multicellularis str. Araruama	TRUE
Flavobacterium hydatis	FALSE
Gammaproteobacteria bacterium	NA
Lysobacter enzymogenes	FALSE

species	multicellularity
Singulisphaera sp. GP187	TRUE

- **beta propeller vs GAP1-N2**:

species	multicellularity
Actinoplanes globisporus	TRUE
Blastopirellula marina	TRUE
Candidatus Thiomargarita nelsonii	TRUE
Dactylosporangium aurantiacum	TRUE
Fimbriiglobus ruber	TRUE
Frankia sp. QA3	TRUE
Gemmata sp. SH-PL17	FALSE
Planctomicrobium piriforme	TRUE
Roseimaritima ulvae	TRUE
Schlesneria paludicola	TRUE
Singulisphaera sp. GP187	TRUE
Thiohalocapsa sp. ML1	TRUE
Zavarzinella formosa	TRUE

- **beta propeller vs NucA**:

**species**	**multicellularity**

- **beta propeller vs EACC1**:

species	multicellularity
Actinoplanes derwentensis	TRUE
Actinoplanes globisporus	TRUE
Amycolatopsis sp. H5	TRUE
Streptomyces scabiei	TRUE
Streptomyces sp. NRRL F-6131	TRUE

- **GAP1-N1 vs GAP1-N2**:

species	multicellularity
Achromatium sp. WMS3	FALSE
Candidatus Achromatium palustre	FALSE

species	multicellularity
Rhodococcus fascians	TRUE
Singulisphaera sp. GP187	TRUE

- **GAP1-N1 vs NucA**:

species	multicellularity
Bradyrhizobium japonicum	TRUE
Sinorhizobium meliloti	TRUE

- **GAP1-N1 vs EACC1**:

species	multicellularity
Bradyrhizobium erythrophlei	TRUE
Enterobacteriaceae	FALSE
Gluconobacter oxydans	FALSE
Klebsiella pneumoniae	FALSE
Pseudosporangium ferrugineum	TRUE
Rhodanobacter glycinis	FALSE
Salmonella enterica	FALSE
Streptomyces canus	TRUE

- **GAP1-N2 vs NucA**:

species	multicellularity
Methylobacterium aquaticum	TRUE

- **GAP1-N2 vs EACC1**:

species	multicellularity
Actinobacteria bacterium OV450	NA
Actinokineospora enzanensis	TRUE
Actinokineospora inagensis	TRUE
Actinokineospora terrae	TRUE
Actinomadura	TRUE
Actinomadura flavalba	TRUE
Actinomadura madurae	TRUE
Actinomadura mexicana	TRUE
Actinomadura oligospora	TRUE
Actinoplanes globisporus	TRUE

species	multicellularity
Actinoplanes rectilineatus	TRUE
Actinoplanes regularis	TRUE
Actinoplanes utahensis	TRUE
Alloactinosynnema iranicum	TRUE
Amycolatopsis alba	TRUE
Amycolatopsis coloradensis	TRUE
Amycolatopsis kentuckyensis	TRUE
Amycolatopsis rifamycinica	TRUE
Amycolatopsis sp. CB00013	TRUE
Candidatus Entotheonella palauensis	TRUE
Frankia asymbiotica	TRUE
Frankia sp. EAN1pec	TRUE
Frankia sp. G2	TRUE
Hamadaea tsunoensis	TRUE
Herbidospora cretacea	TRUE
Herbidospora mongoliensis	TRUE
Herbidospora sakaeratensis	TRUE
Kibdelosporangium phytohabitans	TRUE
Kitasatospora albolonga	TRUE
Kitasatospora azatica	TRUE
Kitasatospora sp. MMS16-BH015	TRUE
Lechevalieria aerocolonigenes	TRUE
Lentzea kentuckyensis	TRUE
Micromonospora carbonacea	TRUE
Micromonospora sp. Rc5	TRUE
Microtetraspora glauca	TRUE
Nocardia concava	TRUE
Nocardia jejuensis	TRUE
Nocardia niigatensis	TRUE
Nocardia seriolae	TRUE
Nocardia sp. NRRL S-836	TRUE
Nocardia takedensis	TRUE
Nocardiopsis dassonvillei	TRUE
Nocardiopsis flavescens	TRUE
Nonomuraea solani	TRUE
Rhodococcus	TRUE
Saccharopolyspora	TRUE
Saccharopolyspora antimicrobica	TRUE
Saccharopolyspora erythraea	TRUE
Saccharopolyspora rectivirgula	TRUE
Saccharopolyspora shandongensis	TRUE
Saccharopolyspora spinosa	TRUE
Streptomyces	TRUE

species	multicellularity
Streptomyces albus	TRUE
Streptomyces antibioticus	TRUE
Streptomyces anulatus	TRUE
Streptomyces atratus	TRUE
Streptomyces aurantiacus	TRUE
Streptomyces aurantiacus JA 4570	TRUE
Streptomyces bicolor	TRUE
Streptomyces caatingaensis	TRUE
Streptomyces cacaoi	TRUE
Streptomyces cellostaticus	TRUE
Streptomyces cellulosae	TRUE
Streptomyces cinnamoneus	TRUE
Streptomyces davaonensis	TRUE
Streptomyces europaeiscabiei	TRUE
Streptomyces filamentosus	TRUE
Streptomyces floridae	TRUE
Streptomyces fulvoviolaceus	TRUE
Streptomyces griseus	TRUE
Streptomyces himastatinicus ATCC 53653	TRUE
Streptomyces hokutonensis	TRUE
Streptomyces katrae	TRUE
Streptomyces lushanensis	TRUE
Streptomyces mirabilis	TRUE
Streptomyces misionensis	TRUE
Streptomyces mobaraensis	TRUE
Streptomyces nanshensis	TRUE
Streptomyces niveus	TRUE
Streptomyces prunicolor	TRUE
Streptomyces regensis	TRUE
Streptomyces reticuli	TRUE
Streptomyces rimosus	TRUE
Streptomyces roseochromogenus	TRUE
Streptomyces roseochromogenus subsp. oscitans DS 12.976	TRUE
Streptomyces scopuliridis	TRUE
Streptomyces sp. 2131.1	TRUE
Streptomyces sp. 2R	TRUE
Streptomyces sp. 61	TRUE
Streptomyces sp. ACT-1	TRUE
Streptomyces sp. ATexAB-D23	TRUE
Streptomyces sp. b62	TRUE
Streptomyces sp. BpilaLS-43	TRUE
Streptomyces sp. CB00072	TRUE

species	multicellularity
Streptomyces sp. CB01249	TRUE
Streptomyces sp. CB02058	TRUE
Streptomyces sp. CB02115	TRUE
Streptomyces sp. CB02488	TRUE
Streptomyces sp. CB02923	TRUE
Streptomyces sp. CcalMP-8W	TRUE
Streptomyces sp. cf386	TRUE
Streptomyces sp. CNB091	TRUE
Streptomyces sp. CNQ431	TRUE
Streptomyces sp. CNQ-525	TRUE
Streptomyces sp. CNQ865	TRUE
Streptomyces sp. CNS335	TRUE
Streptomyces sp. CNT371	TRUE
Streptomyces sp. CNY228	TRUE
Streptomyces sp. CNY243	TRUE
Streptomyces sp. CS057	TRUE
Streptomyces sp. EAG2	TRUE
Streptomyces sp. EN16	TRUE
Streptomyces sp. EN27	TRUE
Streptomyces sp. F-1	TRUE
Streptomyces sp. f150	TRUE
Streptomyces sp. FBKL.4005	TRUE
Streptomyces sp. HCCB10043	TRUE
Streptomyces sp. HGB0020	TRUE
Streptomyces sp. IMTB 2501	TRUE
Streptomyces sp. LamerLS-316	TRUE
Streptomyces sp. MBRL 601	TRUE
Streptomyces sp. MnatMP-M17	TRUE
Streptomyces sp. MNU77	TRUE
Streptomyces sp. MP131-18	TRUE
Streptomyces sp. ms115	TRUE
Streptomyces sp. ms184	TRUE
Streptomyces sp. MUSC 1	TRUE
Streptomyces sp. NBRC 110465	TRUE
Streptomyces sp. NBS 14/10	TRUE
Streptomyces sp. Ncost-T10-10d	TRUE
Streptomyces sp. Ncost-T6T-2b	TRUE
Streptomyces sp. NRRL B-24051	TRUE
Streptomyces sp. NRRL B-24720	TRUE
Streptomyces sp. NRRL F-2747	TRUE
Streptomyces sp. NRRL F-5053	TRUE
Streptomyces sp. NRRL F-5126	TRUE
Streptomyces sp. NRRL F-5727	TRUE
Streptomyces sp. NRRL S-1824	TRUE

species	multicellularity
Streptomyces sp. NRRL S-1868	TRUE
Streptomyces sp. NRRL S-244	TRUE
Streptomyces sp. NRRL S-623	TRUE
Streptomyces sp. NRRL S-920	TRUE
Streptomyces sp. NRRL WC-3549	TRUE
Streptomyces sp. NTK 937	TRUE
Streptomyces sp. or20	TRUE
Streptomyces sp. or3	TRUE
Streptomyces sp. PBH53	TRUE
Streptomyces sp. PgraA7	TRUE
Streptomyces sp. PTY087I2	TRUE
Streptomyces sp. rh34	TRUE
Streptomyces sp. RSD-27	TRUE
Streptomyces sp. RTd22	TRUE
Streptomyces sp. ScaeMP-e48	TRUE
Streptomyces sp. SCSIO 03032	TRUE
Streptomyces sp. SolWspMP-sol2th	TRUE
Streptomyces sp. st140	TRUE
Streptomyces sp. st77	TRUE
Streptomyces sp. Termitarium-T10T-6	TRUE
Streptomyces sp. TSRI0261	TRUE
Streptomyces sp. TSRI0281	TRUE
Streptomyces sp. W007	TRUE
Streptomyces sp. XY332	TRUE
Streptomyces tsukubensis	TRUE
Streptomyces violaceoruber	TRUE
Streptomyces vitaminophilus	TRUE
Streptomyces xylophagus	TRUE
Thermomonospora sp. CIF 1	TRUE

- **NucA vs EACC1**:

**species**	**multicellularity**

VI. Source data - genome identifiers and systems for all EAD1-containing organisms

- **GCA_000166135.1; Frankia inefficax; counts= 22; multicellularity status: TRUE**
  - ??+EAD1+cNMP_cyclase+TIR->cNMP_cyclase+EAD1+TIR*->
  - EAD1->EAD1+cNMP_cyclase+TIR*->
  - EAD1*->EAD1+cNMP_cyclase+TIR->
  - EAD1+APATPase+wHTH+TPRs*->
  - EAD1+APATPase+wHTH+TPRs*->
  - EAD1+APATPase+wHTH+TPRs*->
  - EAD1+APATPase+wHTH+TPRs*->
  - EAD1+APATPase+wHTH+TPRs*->
  - EAD1+APATPase+wHTH+TPRs*->
  - EAD1+APATPase+wHTH+TPRs*->
  - EAD1+APATPase+wHTH+TPRs*->
  - EAD1+APATPase+wHTH+TPRs*->
  - EAD1+APATPase+wHTH+TPRs*->
  - EAD1+BetaPropeller*->
  - EAD1+EAD1+TIR*->
  - EAD1+STAND_NTPase+TPRs*->
  - EAD1+TIR*->
  - EAD1+TM+Peripla_BP*->
  - EAD1+TM+TM+STAND_NTPase+TPRs*->
  - EAD1+TPRs->||<-?<-?||EAD1+APATPase+wHTH+TPRs*->
  - EAD2+VMAP-M0+VMAP-C->MoxR->vWA+STAND_NTPase+TPRs->EAD2+EAD1*->
  - HTH+HTH+STAND_NTPase+wHTH+TPRs->EAD1+TPRs*->||<-?<-?||EAD1+APATPase+wHTH+TPRs->
- **GCA_001456025.1; Mastigocoleus testarum BC008; counts= 19; multicellularity status: TRUE**
  - EAD1+??*->
  - EAD1+CASPASE*->
  - EAD1+CASPASE*->
  - EAD1+EAD1-unk4+Pentapeptide_repeats*->
  - EAD1+EAD1-unk4+Pentapeptide_repeats*->
  - EAD1+GUN4*->
  - EAD1+P-loop_NTPase*->
  - EAD1+Pentapeptide_repeats*->
  - EAD1+TIR*->

  - EAD1+TIR*->TIR+TM+GUN4->
  - EAD1+TIR+GUN4*->
  - EAD1+TIR+GUN4*->||<-?||EAD1+GUN4->
  - EAD1+TM+??*->
  - EAD1+TM+??*->
  - EAD1+TM+CHASE2+TM+TM+TM*->
  - EAD1+VMAP-M1+VMAP-C*->
  - EAD1+VMAP-M1+VMAP-C*->MoxR->
  - EAD8+EAD1+PNP_UDP_1*->
  - LRR_region+sGTP_APGTPase+COR+??+EAD1*->

- **GCA_000346485.2; Scytonema hofmannii PCC 7110; counts= 19; multicellularity status: TRUE**
  - ??+EAD1*->GUN4+EAD1->
  - EAD1*->
  - EAD1*->
  - EAD1+??*->
  - EAD1+CASPASE*->
  - EAD1+CASPASE+STAND_NTPase+GUN4*->||<-CASPASE<-STAND_NTPase+BetaPropeller
  - EAD1+EAD9*->
  - EAD1+P-loop_NTPase*->
  - EAD1+Pkinase+globin*->
  - EAD1+TerB+CASPASE+GUN4*->||<-?<-FGS-fold_domain
  - EAD1+TM+TM+TM+TM*->
  - EAD1+TPRs*->
  - EAD1+VMAP-C*->MoxR->
  - EAD1+VMAP-M1+VMAP-C*->MoxR->vWA+Beta-rich+FGS-fold_domain->
  - GUN4+EAD1*->
  - HTH+EAD1+STAND_NTPase+??*->
  - LRR_region+sGTP_APGTPase+COR+??+EAD1*->
  - PNP_UDP_1+EAD1*->
  - TCAD3+EAD1+EAD9*->EAD9+VMAP-M1+VMAP-C->MoxR->vWA+Beta-rich+FGS-fold_domain->

- **GCA_001854645.1; Frankia sp. EUN1h; counts= 18; multicellularity status: TRUE**
  - <-EAD1+EAD1||EAD1+STAND_NTPase+??*->
  - <-EAD1+STAND_NTPase+??||EAD1+EAD1*->
  - EAD1+??*->
  - EAD1+APATPase+wHTH+TPRs*->
  - EAD1+APATPase+wHTH+TPRs*->
  - EAD1+APATPase+wHTH+TPRs*->
  - EAD1+APATPase+wHTH+TPRs*->
  - EAD1+APATPase+wHTH+TPRs*->
  - EAD1+APATPase+wHTH+TPRs*->

  - EAD1+cNMP_cyclase+TIR->cNMP_cyclase+EAD1+TIR*->
  - EAD1+EACC2*->
  - EAD1+EAD1+BetaPropeller*->
  - EAD1+LGFP_repeat_region*->
  - EAD1+STAND_NTPase+TPRs*->
  - EAD1+STAND_NTPase+wHTH+TPRs+BetaPropeller*->
  - low_complexity+EAD1*->
  - pntlT4_fusion_domain+EAD1*->
  - pntlT4_fusion_domain+EAD1*->

- **GCF_000331305.1; Calothrix sp. PCC 7103; counts= 16; multicellularity status: TRUE**
  - DRHyd?+??+EAD1+??*->
  - EAD1*->
  - EAD1+??*->
  - EAD1+APATPase+wHTH+TPRs*->
  - EAD1+CASPASE*->||<-EAD1+TM+Peripla_BP
  - EAD1+LRR_region+sGTP_APGTPase+COR+??->??+Trypsin->?->EAD1+VMAP-M23+VMAP-C*->MoxR->
  - EAD1+LRR_region+sGTP_APGTPase+COR+??*->??+Trypsin->?->EAD1+VMAP-M23+VMAP-C->
  - EAD1+TIR->EAD1+VMAP-M11+VMAP-M1+VMAP-C*->
  - EAD1+TIR*->EAD1+VMAP-M11+VMAP-M1+VMAP-C->
  - EAD1+TM+Peripla_BP*->||<-EAD1+CASPASE
  - EAD1+TPRs*->
  - EAD1+Trypsin->EAD1*->
  - EAD1+Trypsin*->EAD1->
  - HTH+EAD1+FISNA?*->
  - LRR_region+sGTP_APGTPase+COR+EAD1->EAD1+TCAD4+CASPASE*->
  - LRR_region+sGTP_APGTPase+COR+EAD1*->EAD1+TCAD4+CASPASE->

- **GCA_000177675.1; Frankia sp. EUN1f; counts= 15; multicellularity status: TRUE**
  - ??+EAD1*->
  - <-EAD1+TIR||EAD1+P-loop_NTPase(i)*->MoxR->helical_region+vWA->??+EAD1+EAD1+cNMP_cyclase+TIR->
  - <-helical_region+vWA<-MoxR<-EAD1+P-loop_NTPase(i)||EAD1+TIR*->
  - EAD1+??*->
  - EAD1+APATPase+wHTH+TPRs*->
  - EAD1+cNMP_cyclase+TIR*->
  - EAD1+EAD1+TIR*->TIR+STAND_NTPase+BetaPropeller->
  - EAD1+P-loop_NTPase(i)->MoxR->helical_region+vWA->??+EAD1+EAD1+cNMP_cyclase+TIR*->
  - EAD1+PH?*->
  - EAD1+STAND_NTPase+??*->
  - EAD1+TIR*->
  - EAD1+TIR*->
  - EAD1+TIR*->||<-TIR

- EAD2+EAD1*->
- EAD2+EAD1*->EAD2+VMAP-M0+VMAP-C->MoxR->vWA+APATPase+wHTH+TPRs->

- **GCA_001536285.1; Frankia sp. G2; counts= 14; multicellularity status: TRUE**
  - ??+EAD1+??*->?->vWA->
  - <-EAD1+TIR||EAD1+P-loop_NTPase(i)*->MoxR->helical_region+vWA->??+EAD1+EAD1+cNMP_cyclase+TIR->
  - <-helical_region+vWA<-MoxR<-EAD1+P-loop_NTPase(i)||EAD1+TIR*->
  - <-TIR+STAND_NTPase+wHTH+PRs||EACC2+EAD1*->CASPASE+STAND_NTPase+wHTH+BetaPropeller->
  - EAD1+??*->
  - EAD1+??*->
  - EAD1+APATPase+wHTH+TPRs*->
  - EAD1+APATPase+wHTH+TPRs*->
  - EAD1+cNMP_cyclase+TIR*->
  - EAD1+P-loop_NTPase(i)->MoxR->helical_region+vWA->??+EAD1+EAD1+cNMP_cyclase+TIR*->
  - EAD1+TIR*->
  - EAD1+TIR*->||<-TIR
  - EAD1+TIR+STAND_NTPase*->
  - EAD2+EAD1*->EAD2+VMAP-M0+VMAP-C->

- **GCA_002368435.1; Scytonema sp. NIES-4073; counts= 12; multicellularity status: TRUE**
  - EAD1+CASPASE->EAD1+STAND_NTPase+wHTH+TM+TM+TM+TM+TM+TM*->
  - EAD1+CASPASE*->EAD1+STAND_NTPase+wHTH+TM+TM+TM+TM+TM+TM->
  - EAD1+NACHT-like+FGS-fold_domain+GUN4*->
  - EAD1+P-loop_NTPase(i)*->MoxR->helical_region+vWA->
  - EAD1+STAND_NTPase+wHTH+TM+TM+TM+TM+TM+TM->EAD1+CASPASE*->
  - EAD1+STAND_NTPase+wHTH+TM+TM+TM+TM+TM+TM*->EAD1+CASPASE->
  - EAD1+TCAD4+CASPASE->?->EAD1+TerB-like?+GUN4*->
  - EAD1+TCAD4+CASPASE*->
  - EAD1+Trypsin->EAD1+VMAP-M1+VMAP-C*->MoxR->vWA+Beta-rich+FGS-fold_domain->
  - EAD1+Trypsin*->
  - TIR+EAD1*->
  - TIR+EAD1*->

- **GCA_000316665.1; Rivularia sp. PCC 7116; counts= 11; multicellularity status: TRUE**
  - EACC3+CASPASE+LRR_repeats+sGTP_APGTPase->LRR_region+sGTP_APGTPase+COR+??+EAD1*->
  - EAD1*->
  - EAD1+EAD1-unk4+GUN4*->
  - EAD1+EAD10+FGS-fold_domain*->
  - EAD1+GUN4*->
  - EAD1+Trypsin->EAD1+VMAP-M1+VMAP-C*->MoxR->vWA+Beta-rich+FGS-fold_domain->
  - EAD1+Trypsin*->EAD1+VMAP-M1+VMAP-C->MoxR->vWA+Beta-rich+FGS-fold_domain->
  - EAD1+VMAP-M1+VMAP-C*->MoxR->

- LRR_region+sGTP_APGTPase+COR+??+EAD1*->
- LRR_region+sGTP_APGTPase+COR+??+EAD1*->
- LRR_region+sGTP_APGTPase+COR+??+EAD1*->

* **GCA_002368455.1; Calothrix sp. NIES-4071; counts= 11; multicellularity status: TRUE**
  - CASPASE+EAD1->EAD1+??+VMAP-C*->MoxR->vWA+Beta-rich+FGS-fold_domain->
  - CASPASE+EAD1*->EAD1+??+VMAP-C->MoxR->vWA+Beta-rich+FGS-fold_domain->
  - CASPASE+EAD1+CASPASE*->
  - EAD1+??*->
  - EAD1+CASPASE+GUN4*->
  - EAD1+EAD1-unk4+HTH+HTH*->
  - EAD1+TM+??*->
  - EAD1+Trypsin->EAD1+VMAP-M1+VMAP-C*->MoxR->
  - EAD1+Trypsin*->EAD1+VMAP-M1+VMAP-C->MoxR->
  - HTH+EAD1+STAND_NTPase+??*->
  - LRR_region+sGTP_APGTPase+COR+??+EAD1*->

* **GCA_900197875.1; Frankia sp. ARgP5; counts= 11; multicellularity status: NA**
  - *
  - EAD1+??*->
  - EAD1+APATPase+wHTH+TPRs*->
  - EAD1+BetaPropeller->EAD1+EAD1*->
  - EAD1+BetaPropeller*->EAD1+EAD1->
  - EAD1+cNMP_cyclase+extension*->
  - EAD1+cNMP_cyclase+TIR*->
  - EAD1+P-loop_NTPase+TPRs*->
  - EAD1+STAND_NTPase+??*->
  - EAD2+EAD1*->
  - pntlT4_fusion_domain+EAD1*->

* **GCA_001983215.1; Frankia sp. CcI49; counts= 11; multicellularity status: TRUE**
  - <-EAD1+TIR||EAD1+P-loop_NTPase(i)*->MoxR->helical_region+vWA->??+EAD1+EAD1+cNMP_cyclase+TIR->
  - <-helical_region+vWA<-MoxR<-EAD1+P-loop_NTPase(i)||EAD1+TIR*->
  - EAD1+??->EAD1+P-loop_NTPase+TPRs*->
  - EAD1+??*->
  - EAD1+APATPase+wHTH+TPRs*->
  - EAD1+cNMP_cyclase+TIR*->
  - EAD1+P-loop_NTPase(i)->MoxR->helical_region+vWA->??+EAD1+EAD1+cNMP_cyclase+TIR*->
  - EAD1+STAND_NTPase+wHTH*->
  - EAD1+TIR->?->?->EAD1+VMAP-C*->MoxR->
  - EAD1+TIR*->
  - EAD1+TIR*->?->?->EAD1+VMAP-C->

- **GCA_000948395.1; Frankia sp. CpI1-S; counts= 10; multicellularity status: TRUE**
  - ??+EAD1*->
  - ??+EAD1*->
  - EAD1+??*->
  - EAD1+BetaPropeller*->
  - EAD1+cNMP_cyclase+extension*->
  - EAD1+cNMP_cyclase+TIR*->
  - EAD2+EAD1*->
  - EAD2+EAD1*->
  - pntlT4_fusion_domain+EAD1*->
  - pntlT4_fusion_domain+EAD1*->
- **GCA_002368235.1; Scytonema sp. HK-05; counts= 9; multicellularity status: TRUE**
  - EAD1+CASPASE+P-loop_NTPase*->
  - EAD1+P-loop_NTPase(i)*->MoxR->helical_region+vWA->
  - EAD1+TCAD4+CASPASE->PSE->EAD1+TM+TM+STAND_NTPase+FGS-fold_domain+GUN4*->
  - EAD1+TCAD4+CASPASE*->PSE->EAD1+TM+TM+STAND_NTPase+FGS-fold_domain+GUN4->
  - EAD1+Trypsin->EAD1+FGS-fold_domain*->
  - EAD1+Trypsin*->EAD1+FGS-fold_domain->
  - MoxR->helical_region+vWA->PSE->?->TIR+EAD1*->
  - TCAD3+TCAD4+CASPASE+EAD1->EAD1+VMAP-M1+VMAP-C*->MoxR->vWA+Beta-rich+HTH+FGS-fold_domain->
  - TCAD3+TCAD4+CASPASE+EAD1*->EAD1+VMAP-M1+VMAP-C->MoxR->vWA+Beta-rich+HTH+FGS-fold_domain->
- **GCF_000235425.2; Frankia saprophytica; counts= 9; multicellularity status: NA**
  - <-EAD1+EAD1||?->EAD1+STAND_NTPase+??*->
  - EAD1+APATPase+wHTH+TPRs*->
  - EAD1+APATPase+wHTH+TPRs*->
  - EAD1+APATPase+wHTH+TPRs*->
  - EAD1+APATPase+wHTH+TPRs*->
  - EAD1+cNMP_cyclase+TIR*->cNMP_cyclase+EAD1+TIR->
  - EAD1+LGFP_repeat_region*->
  - EAD1+STAND_NTPase+TPRs*->
  - EAD1+TPRs*->
- **GCA_001027285.1; Archangium gephyra; counts= 8; multicellularity status: TRUE**
  - EAD1+STAND_NTPase->STAND_NTPase+wHTH+TPRs+EAD1+EAD5->EAD1+Trypsin*->
  - EAD1+STAND_NTPase->STAND_NTPase+wHTH+TPRs+EAD1+EAD5*->EAD1+Trypsin->
  - EAD1+STAND_NTPase*->STAND_NTPase+wHTH+TPRs+EAD1+EAD5->EAD1+Trypsin->
  - EAD1+TIR*->
  - EAD1+VMAP-M0+VMAP-C*->MoxR->vWA+CASPASE+SLOG+TPRs->
  - EAD1+VMAP-M0+VMAP-C*->vWA+SLOG+TPRs->
  - EAD1+VMAP-M1+VMAP-C*->MoxR->?->vWA+SLOG+SLOG+TPRs->

- MoxR->?->vWA+SLOG+SLOG+TPRs->EAD1+Trypsin*->

- **GCF_000966285.1; Frankia sp. DC12; counts= 8; multicellularity status: NA**
  - ??+EAD1*->
  - EAD1*->
  - EAD1+??*->
  - EAD1+APATPase+wHTH+TPRs*->
  - EAD1+APATPase+wHTH+TPRs*->
  - EAD1+cNMP_cyclase+TIR*->
  - EAD1+VMAP-M2+VMAP-C*->
  - HTH+HTH+STAND_NTPase+wHTH+TPRs->EAD1+TPRs*->
- **GCA_001983105.1; Frankia asymbiotica; counts= 8; multicellularity status: TRUE**
  - EAD1+APATPase+wHTH+TPRs*->
  - EAD1+APATPase+wHTH+TPRs*->
  - EAD1+cNMP_cyclase+TIR*->
  - EAD1+P-loop_NTPase+TPRs*->
  - EAD1+Pentapeptide_repeats*->
  - EAD1+TM+Peripla_BP*->
  - EAD2+EAD1*->
  - pntlT4_fusion_domain+EAD1*->
- **GCA_001767235.1; Moorea producens PAL-8-15-08-1; counts= 7; multicellularity status: NA**
  - EAD1+STAND_NTPase+TM_region*->
  - EAD1+Trypsin->||<-?||PSE->EAD1+VMAP-M1+VMAP-C*->MoxR->vWA+Beta-rich+FGS-fold_domain->
  - EAD1+Trypsin*->||<-?||PSE->EAD1+VMAP-M1+VMAP-C->MoxR->
  - EAD1+VMAP-M1+FGS-fold_domain->EAD1+VMAP-M1+FGS-fold_domain->EAD1*->VMAP-M1+FGS-fold_domain->EAD1+VMAP-M1+FGS-fold_domain->
  - EAD1+VMAP-M1+FGS-fold_domain->EAD1+VMAP-M1+FGS-fold_domain*->EAD1->VMAP-M1+FGS-fold_domain->EAD1+VMAP-M1+FGS-fold_domain->
  - EAD1+VMAP-M1+FGS-fold_domain*->
  - EAD1+VMAP-M1+FGS-fold_domain*->EAD1+VMAP-M1+FGS-fold_domain->EAD1->VMAP-M1+FGS-fold_domain->
- **GCA_000058485.1; Frankia alni ACN14a; counts= 7; multicellularity status: TRUE**
  - ??+EAD1*->
  - EACC2+EAD1*->||<-CASPASE+STAND_NTPase+wHTH+BetaPropeller
  - EAD1*->
  - EAD1+cNMP_cyclase+extension*->
  - EAD1+cNMP_cyclase+TIR*->
  - EAD2+EAD1*->
  - TM+EAD1*->
- **GCA_000760695.2; Tolypothrix bouteillei VB521301; counts= 7; multicellularity status: NA**
  - EAD1*->
  - EAD1+??*->

- EAD1+APATPase+wHTH+TPRs*->
- EAD1+CASPASE*->
- EAD1+STAND_NTPase+TM_region*->
- EAD1+STAND_NTPase+TM_region*->
- Pentapeptide_repeats+EAD1*->

- **GCA_000817735.2; Scytonema millei VB511283; counts= 7; multicellularity status: TRUE**
  - EAD1*->
  - EAD1+??*->
  - EAD1+??*->
  - EAD1+Trypsin->EAD1+FGS-fold_domain*->
  - EAD1+Trypsin*->EAD1+FGS-fold_domain->
  - TIR+EAD1->EAD1+STAND_NTPase+FGS-fold_domain+GUN4*->
  - TIR+EAD1*->EAD1+STAND_NTPase+FGS-fold_domain+GUN4->

- **GCA_001854805.1; Frankia sp. BMG5.36; counts= 7; multicellularity status: NA**
  - EAD1+APATPase+wHTH+TPRs*->
  - EAD1+APATPase+wHTH+TPRs*->
  - EAD1+BetaPropeller*->
  - EAD1+LGFP_repeat_region*->
  - EAD1+STAND_NTPase+TPRs*->
  - SP+TM+TM+TM+EAD1*->
  - TIR+TM+BetaPropeller->?->pntlT4_fusion_domain+EAD1*->CASPASE->

- **GCF_001456025.1; Mastigocoleus testarum; counts= 7; multicellularity status: TRUE**
  - EAD1+??+FGS-fold_domain*->
  - EAD1+APATPase+wHTH+TPRs*->
  - EAD1+FGS-fold_domain*->
  - EAD1+FGS-fold_domain*->
  - EAD1+GUN4*->
  - EAD1+GUN4*->
  - EAD1+TIR+GUN4->||<-?||EAD1+GUN4*->

- **GCF_001306465.1; Frankia sp. R43; counts= 7; multicellularity status: TRUE**
  - EAD1+APATPase+wHTH+TPRs*->
  - EAD1+APATPase+wHTH+TPRs*->
  - EAD1+APATPase+wHTH+TPRs*->
  - EAD1+cNMP_cyclase+TIR*->
  - EAD1+P-loop_NTPase(i)->MoxR->helical_region+vWA->??+EAD1+EAD1+cNMP_cyclase+TIR*->
  - EAD1+TIR*->
  - pntlT4_fusion_domain+EAD1*->?->Lon_C_protease(i)+TM+BetaPropeller->

- **GCA_001854205.1; Moorea producens JHB; counts= 6; multicellularity status: TRUE**

- EAD1+low_complexity+TM+TM+TM*->
- EAD1+Trypsin->EAD1+VMAP-M1+Calcineurin-like->EAD1+VMAP-M1+VMAP-C*->MoxR->vWA+FGS-fold_domain->
- EAD1+Trypsin->EAD1+VMAP-M1+Calcineurin-like*->EAD1+VMAP-M1+VMAP-C->MoxR->vWA+FGS-fold_domain->
- EAD1+Trypsin*->EAD1+VMAP-M1+Calcineurin-like->EAD1+VMAP-M1+VMAP-C->MoxR->
- EAD1+VMAP-M1+FGS-fold_domain*->EAD1+VMAP-M1+FGS-fold_domain->
- VMAP-M1+FGS-fold_domain->EAD1+VMAP-M1+FGS-fold_domain*->

- **GCA_002368195.1; Calothrix sp. NIES-2100; counts= 6; multicellularity status: NA**
  - EAD1+coiled_coil*->
  - EAD1+coiled_coil*->
  - EAD1+low_complexity*->
  - EAD1+TPRs*->
  - EAD1+Trypsin->TM+TM+TM->EAD1+VMAP-M1+VMAP-C*->MoxR->vWA+Beta-rich+FGS-fold_domain->
  - EAD1+Trypsin*->TM+TM+TM->EAD1+VMAP-M1+VMAP-C->MoxR->
- **GCA_000067165.1; Sorangium cellulosum So ce56; counts= 6; multicellularity status: TRUE**
  - bacHORMA->FtsH-like->SAVED+EAD1->EAD1+Trypsin*->
  - bacHORMA->FtsH-like->SAVED+EAD1*->EAD1+Trypsin->
  - EAD1+STAND_NTPase->STAND_NTPase+wHTH+TPRs+EAD1->EAD1+Trypsin*->
  - EAD1+STAND_NTPase*->STAND_NTPase+wHTH+TPRs+EAD1->EAD1+Trypsin->
  - STAND_NTPase+wHTH+TPRs+BetaPropeller+EAD1->EAD1+Trypsin*->
  - STAND_NTPase+wHTH+TPRs+BetaPropeller+EAD1*->EAD1+Trypsin->
- **GCA_001306465.1; Frankia sp. R43; counts= 6; multicellularity status: TRUE**
  - <-EAD1+TIR||EAD1+P-loop_NTPase(i)*->MoxR->helical_region+vWA->??+EAD1+EAD1+cNMP_cyclase+TIR->
  - <-helical_region+vWA<-MoxR<-EAD1+P-loop_NTPase(i)||EAD1+TIR*->
  - EAD1+APATPase+wHTH+TPRs*->
  - EAD1+PH?*->
  - EAD2+EAD1*->EAD2+VMAP-M0+VMAP-C->MoxR->vWA+APATPase+wHTH+TPRs->
  - pntlT4_fusion_domain+EAD1*->?->Lon_C_protease(i)+TM+BetaPropeller->
- **GCA_001421075.1; Frankia sp. CpI1-P; counts= 6; multicellularity status: TRUE**
  - ??+EAD1*->
  - EAD1+??*->
  - EAD1+cNMP_cyclase+TIR*->
  - EAD2+EAD1*->
  - EAD2+EAD1*->
  - pntlT4_fusion_domain+EAD1*->
- **GCA_001589215.1; Sorangium cellulosum; counts= 6; multicellularity status: TRUE**
  - EAD1+Trypsin->EAD1+STAND_NTPase+TPRs*->
  - EAD1+Trypsin*->
  - EAD1+Trypsin*->?->MoxR->

  - EAD1+Trypsin*->EAD1+STAND_NTPase+TPRs->
  - STAND_NTPase+wHTH+TPRs+BetaPropeller+EAD1->EAD1+Trypsin*->
  - STAND_NTPase+wHTH+TPRs+BetaPropeller+EAD1*->EAD1+Trypsin->

- **GCA_001854655.1; Frankia sp. Cc1.17; counts= 6; multicellularity status: TRUE**
  - EAD1*->
  - EAD1+??*->||<-?<-TIR+STAND_NTPase+BetaPropeller
  - EAD1+BetaPropeller*->
  - EAD1+cNMP_cyclase+TIR*->
  - EAD1+TIR*->||<-TIR
  - MoxR->?->??+Pkinase+EAD1+TIR*->

- **GCA_900104725.1; Actinoplanes derwentensis; counts= 6; multicellularity status: TRUE**
  - CASPASE+STAND_NTPase+wHTH->?->AB_hydrolase+TIR->EAD1+TIR*->BetaPropeller+vWA->
  - EAD1+Trypsin->EAD1+P-loop_NTPase(i)*->MoxR->
  - Pkinase->EAD1+Trypsin->EAD1+STAND_NTPase*->?->||<-?<-??+Trypsin
  - Trypsin+EAD1*->BetaPropeller+vWA->
  - vWA->?->Pkinase->EAD1+Trypsin*->EAD1+STAND_NTPase->
  - ZnF->E1(active)+E1-ZnR_C->EAD1+YukDl-Nterm+UB+E2*->JAB-N+JAB->?->CDC48-like->

- **GCA_000018005.1; Frankia sp. EAN1pec; counts= 5; multicellularity status: TRUE**
  - EAD1+??*->
  - EAD1+cNMP_cyclase+TIR*->
  - EAD2+EAD1*->EAD2+VMAP-M0+VMAP-C->MoxR->vWA+APATPase+wHTH+TPRs->
  - pntlT4_fusion_domain+EAD1*->
  - TM+BetaPropeller->TIR+EACC2+EAD1*->

- **GCA_001414035.1; Frankia sp. ACN1ag; counts= 5; multicellularity status: TRUE**
  - <-CASPASE+STAND_NTPase+wHTH+BetaPropeller||EACC2+EAD1*->
  - EAD1+cNMP_cyclase+TIR*->
  - EAD2+EAD1*->
  - EAD2+EAD1*->
  - pntlT4_fusion_domain+EAD1*->

- **GCA_001636565.1; Frankia sp. EI5c; counts= 5; multicellularity status: TRUE**
  - ??+EAD1*->
  - ??+EAD1*->
  - EAD1+cNMP_cyclase+extension*->
  - EAD1+TIR*->
  - pntlT4_fusion_domain+EAD1*->

- **GCA_001904745.1; Calothrix sp. HK-06; counts= 5; multicellularity status: NA**
  - CASPASE+EAD1->EAD1*->

- EAD1*->
- EAD1+??*->
- EAD1+CASPASE+GUN4*->
- EAD1+STAND_NTPase+TM_region*->

- **GCA_001942495.1; Moorea bouillonii PNG; counts= 5; multicellularity status: TRUE**
  - EAD1+low_complexity+TM+TM+TM*->
  - EAD1+Trypsin->EAD1+VMAP-M1+Calcineurin-like->EAD1+VMAP-M1+VMAP-C*->MoxR->vWA+Beta-rich+FGS-fold_domain->
  - EAD1+Trypsin->EAD1+VMAP-M1+Calcineurin-like*->EAD1+VMAP-M1+VMAP-C->MoxR->vWA+Beta-rich+FGS-fold_domain->
  - EAD1+Trypsin*->EAD1+VMAP-M1+Calcineurin-like->EAD1+VMAP-M1+VMAP-C->MoxR->
  - EAD1+VMAP-M1+FGS-fold_domain*->
- **GCF_000374165.1; Frankia elaeagni; counts= 5; multicellularity status: NA**
  - EAD1+??*->
  - EAD1+cNMP_cyclase+TIR*->
  - EAD1+TIR*->||<-TIR
  - pntlT4_fusion_domain+EAD1*->
  - pntlT4_fusion_domain+EAD1*->
- **GCF_000166135.1; Frankia inefficax; counts= 5; multicellularity status: TRUE**
  - ??+EAD1+cNMP_cyclase+TIR*->cNMP_cyclase+EAD1+TIR->
  - EAD1+APATPase+wHTH+TPRs*->
  - EAD1+APATPase+wHTH+TPRs*->
  - EAD1+APATPase+wHTH+TPRs*->
  - EAD1+STAND_NTPase+wHTH+TPRs+BetaPropeller*->
- **GCF_000760695.2; Tolypothrix bouteillei; counts= 5; multicellularity status: NA**
  - EAD1+APATPase+wHTH+TPRs*->
  - EAD1+CASPASE+GUN4*->
  - EAD1+GUN4*->
  - EAD1+STAND_NTPase+HEAT_repeat_region*->
  - Pentapeptide_repeats+EAD1*->
- **GCF_001420875.1; Frankia sp. AvcI1; counts= 5; multicellularity status: TRUE**
  - ??+EAD1*->
  - EACC2+EAD1*->||<-CASPASE+STAND_NTPase+wHTH+BetaPropeller
  - EAD1*->
  - EAD2+EAD1*->
  - TM+EAD1*->
- **GCA_000211815.1; Moorea producens 3L; counts= 4; multicellularity status: NA**
  - EAD1+Trypsin->EAD1+VMAP-M1+Calcineurin-like->EAD1+VMAP-M1+VMAP-C*->MoxR->vWA+TPRs+CASPASE->
  - EAD1+Trypsin->EAD1+VMAP-M1+Calcineurin-like*->EAD1+VMAP-M1+VMAP-C->MoxR->vWA+TPRs+CASPASE->

- EAD1+Trypsin*->EAD1+VMAP-M1+Calcineurin-like->EAD1+VMAP-M1+VMAP-C->MoxR->
- EAD1+VMAP-M1+FGS-fold_domain*->

- **GCA_000262465.1; Frankia sp. QA3; counts= 4; multicellularity status: TRUE**
  - E1(active)+E1_C_unk->EAD1+YukDl-Nterm+UB+E2*->JAB-N+JAB->
  - EAD1+??*->MoxR->
  - EAD1+cNMP_cyclase+extension*->
  - EAD1+cNMP_cyclase+TIR*->
- **GCA_001644415.1; Planctomycetaceae bacterium SCGC AG-212-F19; counts= 4; multicellularity status: NA**
  - EAD1+RRM+STAND_NTPase+FGS-fold_domain->?->EAD1+Trypsin*->
  - EAD1+RRM+STAND_NTPase+FGS-fold_domain*->?->EAD1+Trypsin->
  - EAD1+Trypsin*->
  - EAD1+Trypsin*->
- **GCA_001672105.1; Aphanizomenon flos-aquae WA102; counts= 4; multicellularity status: TRUE**
  - cNMP_cyclase+EAD1->EAD1*->MoxR->vWA+Beta-rich+HTH+FGS-fold_domain->
  - cNMP_cyclase+EAD1*->EAD1->MoxR->vWA+Beta-rich+HTH+FGS-fold_domain->
  - EAD1+Trypsin*->
  - Trypsin->TM+TM+TM->EAD1+P-loop_NTPase*->
- **GCA_001854695.1; Frankia sp. NRRL B-16219; counts= 4; multicellularity status: TRUE**
  - EAD1+cNMP_cyclase+TIR*->
  - EAD1+TM+??*->
  - EAD2+EAD1*->EAD2+VMAP-M0+VMAP-C->
  - pntlT4_fusion_domain+EAD1*->
- **GCF_000373365.1; Frankia discariae; counts= 4; multicellularity status: TRUE**
  - EAD1+??*->
  - EAD1+cNMP_cyclase+TIR*->
  - EAD1+TM+??*->
  - EAD2+EAD1*->EAD2+VMAP-M0+VMAP-C->MoxR->vWA+APATPase+wHTH+TPRs->
- **GCA_001712795.1; Nostoc sp. KVJ20; counts= 4; multicellularity status: TRUE**
  - EAD1+Trypsin->EAD1+VMAP-M1+??*->MoxR->EB1_binding+vWA->
  - EAD1+Trypsin->TM+TM+TM->EAD1+coiled_coil*->
  - EAD1+Trypsin*->EAD1+VMAP-M1+??->MoxR->EB1_binding+vWA->
  - EAD1+Trypsin*->TM+TM+TM->EAD1+coiled_coil->
- **GCA_000970465.2; Devosia insulae; counts= 4; multicellularity status: FALSE**
  - EAD1+Phage_lysozyme->EAD1+Trypsin->EAD1+HExxH+helical_region*->TM+Endonuclease_NS(HNH)+LytR_C/SPOR->Trypsin+Endonuclease_NS(HNH)->
  - EAD1+Phage_lysozyme->EAD1+Trypsin*->EAD1+HExxH+helical_region->TM+Endonuclease_NS(HNH)+LytR_C/SPOR->Trypsin+Endonuclease_NS(HNH)->
  - EAD1+Phage_lysozyme*->EAD1+Trypsin->EAD1+HExxH+helical_region->TM+Endonuclease_NS(HNH)+LytR_C/SPOR->

  - EAD1+Trypsin*->

- **GCF_001870905.1; Geitlerinema sp. PCC 9228; counts= 4; multicellularity status: TRUE**
  - EAD1+??+VMAP-C*->MoxR->
  - EAD1+GUN4+??*->
  - EAD1+Trypsin->TM+TM+TM->EAD1+CASPASE+FGS-fold_domain*->
  - EAD1+Trypsin*->TM+TM+TM->EAD1+CASPASE+FGS-fold_domain->
- **GCA_001610855.1; Gemmata sp. SH-PL17; counts= 3; multicellularity status: FALSE**
  - EAD1+Trypsin->EAD1+HExxH+helical_region*->?->??+Trypsin->
  - EAD1+Trypsin*->EAD1+HExxH+helical_region->?->??+Trypsin->
  - VMAP-C->MoxR->EAD1+HD+vWA+NEFF1*->
- **GCA_000344805.1; Bradyrhizobium oligotrophicum S58; counts= 3; multicellularity status: TRUE**
  - EAD1+Trypsin->EAD1+??*->STAND_NTPase+wHTH+TM+TPRs->
  - EAD1+Trypsin*->EAD1+??->STAND_NTPase+wHTH+TM+TPRs->
  - Trypsin+EAD1+helical_region*->
- **GCA_001589195.1; Sorangium cellulosum; counts= 3; multicellularity status: TRUE**
  - bacHORMA->FtsH-like->SAVED+EAD1->EAD1+Trypsin*->
  - bacHORMA->FtsH-like->SAVED+EAD1*->EAD1+Trypsin->
  - EAD1+Trypsin*->
- **GCA_002148365.1; Litorilinea aerophila; counts= 3; multicellularity status: NA**
  - EAD1*->
  - EAD1+SIR2*->
  - EAD1+Trypsin*->
- **GCF_000426925.1; Dolichospermum circinale; counts= 3; multicellularity status: TRUE**
  - EAD1+Trypsin->TM+TM+TM->EAD1->EAD1+TM*->
  - EAD1+Trypsin->TM+TM+TM->EAD1*->EAD1+TM->
  - EAD1+Trypsin*->TM+TM+TM->EAD1->EAD1+TM->
- **GCF_000716715.1; Dactylosporangium aurantiacum; counts= 3; multicellularity status: TRUE**
  - EAD1+CASPASE->PSE->E1+E1-ZnR_C->EAD1+YukDl-Nterm+UB+E2*->JAB-N+JAB->?->CDC48-like->
  - EAD1+CASPASE*->PSE->E1+E1-ZnR_C->EAD1+YukDl-Nterm+UB+E2->JAB-N+JAB->
  - Trypsin+PDZ+STAND_NTPase+wHTH+TPRs->EAD1+Trypsin*->
- **GCF_000058485.1; Frankia alni; counts= 3; multicellularity status: TRUE**
  - EAD1*->
  - EAD2+EAD1*->
  - TM+EAD1*->
- **GCA_000733295.1; Archangium violaceum; counts= 3; multicellularity status: TRUE**

  - *
  - EAD1+Trypsin->EAD1+??+VMAP-C->MoxR->vWA+SLOG+TPRs+EAD1+TIR*->
  - EAD1+Trypsin*->EAD1+??+VMAP-C->MoxR->vWA+SLOG+TPRs+EAD1+TIR->

- **GCF_001983215.1; Frankia sp. CcI49; counts= 3; multicellularity status: TRUE**
  - EAD1+APATPase+wHTH+TPRs*->
  - EAD1+cNMP_cyclase+TIR*->?->STAND_NTPase+BetaPropeller->
  - EAD1+PNP_UDP_1+STAND_NTPase+wHTH*->
- **GCA_002603685.1; Leptospira phage vb_LkmZ_Bejolso9-LE1; counts= 2; multicellularity status: FALSE**
  - GAP2->ZnR+DO-GTPase2->DO-GTPase1->GAP1-N1+GAP1-M+GAP1-C+EAD1*->PNP_UDP_1+EAD1->
  - ZnR+DO-GTPase2->DO-GTPase1->GAP1-N1+GAP1-M+GAP1-C+EAD1->PNP_UDP_1+EAD1*->
- **GCA_000582515.1; Yersinia similis; counts= 2; multicellularity status: FALSE**
  - GAP2->ZnR+DO-GTPase2->DO-GTPase1->GAP1-N1+GAP1-M+GAP1-C+EAD1*->CASPASE+EAD1->
  - ZnR+DO-GTPase2->DO-GTPase1->GAP1-N1+GAP1-M+GAP1-C+EAD1->CASPASE+EAD1*->
- **GCA_000739375.1; Comamonas testosteroni TK102; counts= 2; multicellularity status: FALSE**
  - CASPASE+EAD1*->
  - DO-GTPase1->GAP1-N1+GAP1-M+GAP1-C+EAD1*->CASPASE+EAD1->
- **GCA_001610875.1; Pirellula sp. SH-Sr6A; counts= 2; multicellularity status: TRUE**
  - EAD1+Trypsin*->||<-GAP1-N1+GAP1-M+GAP1-C+EAD1<-DO-GTPase1<-ZnR+DO-GTPase2
  - ZnR+DO-GTPase2->DO-GTPase1->GAP1-N1+GAP1-M+GAP1-C+EAD1*->||<-EAD1+Trypsin
- **GCA_001931535.1; Minicystis rosea; counts= 2; multicellularity status: TRUE**
  - STAND_NTPase+BetaPropeller+EAD1->EAD1+Trypsin*->
  - STAND_NTPase+BetaPropeller+EAD1*->EAD1+Trypsin->
- **GCA_000959705.2; Aeromonas caviae; counts= 2; multicellularity status: NA**
  - GAP1-N1+GAP1-M+GAP1-C+EAD1->CASPASE+EAD1*->
  - GAP1-N1+GAP1-M+GAP1-C+EAD1*->CASPASE+EAD1->
- **GCA_000239775.2; Bradyrhizobium sp. ORS 375; counts= 2; multicellularity status: TRUE**
  - EAD1+Trypsin->EAD1+Lon_C_protease+STAND_NTPase*->
  - EAD1+Trypsin*->EAD1+Lon_C_protease+STAND_NTPase->
- **GCA_000982975.1; Halomonas sp. A3H3; counts= 2; multicellularity status: FALSE**
  - GAP2->ZnR+DO-GTPase2->DO-GTPase1->GAP1-N1+GAP1-M+GAP1-C+EAD1*->CASPASE+EAD1->
  - ZnR+DO-GTPase2->PSE->GAP1-N1+GAP1-M+GAP1-C+EAD1->CASPASE+EAD1*->
- **GCA_900066015.1; Candidatus Promineofilum breve; counts= 2; multicellularity status: TRUE**
  - EAD1+STAND_NTPase+FGS-fold_domain*->
  - macroglobulin-like+EAD1*->

- **GCA_000244135.3; Leptospira interrogans serovar Grippotyphosa str. UI 08368; counts= 2; multicellularity status: FALSE**
  - EAD1*->PNP_UDP_1+EAD1->
  - PNP_UDP_1+EAD1*->
- **GCA_000316115.1; Leptolyngbya sp. PCC 7375; counts= 2; multicellularity status: TRUE**
  - EAD1+Trypsin*->TM+TM+TM->EAD1+??+VMAP-C->MoxR->
  - EAD1+VMAP-M1+VMAP-C*->MoxR->vWA+BetaPropeller+FGS-fold_domain->FGS-fold_domain->
- **GCA_000368445.1; Acinetobacter sp. CIP 56.2; counts= 2; multicellularity status: FALSE**
  - GAP2->ZnR+DO-GTPase2->DO-GTPase1->PSE->CASPASE+EAD1*->
  - ZnR+DO-GTPase2->DO-GTPase1->?->GAP1-N1+GAP1-M+GAP1-C+EAD1*->CASPASE+EAD1->
- **GCA_000503035.1; Mesorhizobium sp. LSHC412B00; counts= 2; multicellularity status: TRUE**
  - EAD1+Trypsin+??+CarboxypepD-reg-IG+papain-like_peptidoglycan_hydrolase*->
  - Trypsin+Endonuclease_NS(HNH)->EAD1+Trypsin*->
- **GCA_000584955.2; Candidatus Accumulibacter sp. SK-01; counts= 2; multicellularity status: FALSE**
  - GAP2->ZnR+DO-GTPase2->DO-GTPase1->GAP1-N1+GAP1-M+GAP1-C+EAD1*->CASPASE+EAD1->
  - ZnR+DO-GTPase2->DO-GTPase1->GAP1-N1+GAP1-M+GAP1-C+EAD1->CASPASE+EAD1*->
- **GCA_001047325.1; Streptomyces viridochromogenes; counts= 2; multicellularity status: TRUE**
  - GAP1-N1+GAP1-M+GAP1-C+EAD1->CASPASE+EAD1*->
  - GAP2->ZnR+DO-GTPase2->DO-GTPase1->GAP1-N1+GAP1-M+GAP1-C+EAD1*->CASPASE+EAD1->
- **GCA_001314865.1; Phormidesmis priestleyi Ana; counts= 2; multicellularity status: TRUE**
  - EAD1+Trypsin->TM+TM+TM->EAD1+??+VMAP-C*->MoxR->vWA+FGS-fold_domain->FGS-fold_domain->
  - EAD1+Trypsin*->TM+TM+TM->EAD1+??+VMAP-C->MoxR->
- **GCA_001672155.1; Anabaena sp. WA113; counts= 2; multicellularity status: NA**
  - EAD1+Trypsin->TM+TM+TM->EAD1*->
  - EAD1+Trypsin*->TM+TM+TM->EAD1->
- **GCA_001672085.1; Anabaena sp. AL93; counts= 2; multicellularity status: TRUE**
  - EAD1+Trypsin->TM+TM+TM->EAD1*->
  - EAD1+Trypsin*->TM+TM+TM->EAD1->
- **GCA_001672075.1; Anabaena sp. CRKS33; counts= 2; multicellularity status: TRUE**
  - EAD1+Trypsin->TM+TM+TM->EAD1*->
  - EAD1+Trypsin*->TM+TM+TM->EAD1->
- **GCA_001672195.1; Anabaena sp. MDT14b; counts= 2; multicellularity status: TRUE**
  - EAD1+Trypsin->TM+TM+TM->EAD1*->
  - EAD1+Trypsin*->TM+TM+TM->EAD1->

- **GCA_001742065.1; Psychrobacter sp. B29-1; counts= 2; multicellularity status: NA**
  - PNP_UDP_1+EAD1*->?->ZnR+DO-GTPase2->DO-GTPase1->
  - ZnR+DO-GTPase2->DO-GTPase1->GAP1-N1+GAP1-M+GAP1-C+EAD1*->
- **GCA_001897835.1; Bacteroidetes bacterium 46-16; counts= 2; multicellularity status: FALSE**
  - ZnR+DO-GTPase2->DO-GTPase1->GAP1-N1+GAP1-M+GAP1-C+EAD1->PNP_UDP_1+EAD1*->
  - ZnR+DO-GTPase2->DO-GTPase1->GAP1-N1+GAP1-M+GAP1-C+EAD1*->PNP_UDP_1+EAD1->
- **GCA_001898665.1; Burkholderiales bacterium 68-20; counts= 2; multicellularity status: FALSE**
  - DO-GTPase2->DO-GTPase1->GAP1-N1+GAP1-M+GAP1-C+EAD1->CASPASE+EAD1*->
  - DO-GTPase2->DO-GTPase1->GAP1-N1+GAP1-M+GAP1-C+EAD1*->CASPASE+EAD1->
- **GCA_001905545.1; Streptomyces sp. CB02056; counts= 2; multicellularity status: NA**
  - EAD1+Trypsin->EAD1+STAND_NTPase*->
  - EAD1+Trypsin*->||<-?||STAND_NTPase->STAND_NTPase+wHTH+TPRs+AB_hydrolase->
- **GCA_001921865.1; Pseudomonas chlororaphis; counts= 2; multicellularity status: FALSE**
  - GAP1-N1+GAP1-M+GAP1-C+EAD1->CASPASE+EAD1*->
  - GAP2->ZnR+DO-GTPase2->DO-GTPase1->GAP1-N1+GAP1-M+GAP1-C+EAD1*->CASPASE+EAD1->
- **GCA_002280395.1; Flavobacteriales bacterium 32-35-8; counts= 2; multicellularity status: FALSE**
  - DO-GTPase1->GAP1-N1+GAP1-M+GAP1-C+EAD1->?->EAD1*->
  - GAP2->ZnR+DO-GTPase2->DO-GTPase1->GAP1-N1+GAP1-M+GAP1-C+EAD1*->?->EAD1->
- **GCA_002281555.1; Hydrogenophilales bacterium 28-61-23; counts= 2; multicellularity status: FALSE**
  - Calcineurin-like+EAD1*->
  - GAP1-N1+GAP1-M+GAP1-C+EAD1*->
- **GCA_002735705.1; Janthinobacterium sp. BJB426; counts= 2; multicellularity status: FALSE**
  - GAP1-N1+GAP1-M+GAP1-C+EAD1->CASPASE+EAD1*->
  - GAP1-N1+GAP1-M+GAP1-C+EAD1*->CASPASE+EAD1->
- **GCA_002746795.1; Chloroflexi bacterium; counts= 2; multicellularity status: TRUE**
  - EAD1+STAND_NTPase+FGS-fold_domain->?->?->EAD1+Trypsin*->
  - EAD1+STAND_NTPase+FGS-fold_domain*->?->?->EAD1+Trypsin->
- **GCA_002842665.1; Bacteroidetes bacterium HGW-Bacteroidetes-11; counts= 2; multicellularity status: FALSE**
  - GAP2->ZnR+DO-GTPase2->DO-GTPase1->GAP1-N1+GAP1-M+GAP1-C+EAD1*->PNP_UDP_1+EAD1->
  - ZnR+DO-GTPase2->DO-GTPase1->GAP1-N1+GAP1-M+GAP1-C+EAD1->PNP_UDP_1+EAD1*->
- **GCA_900111115.1; Giesbergeria anulus; counts= 2; multicellularity status: FALSE**
  - GAP2->ZnR+DO-GTPase2->DO-GTPase1->GAP1-N1+GAP1-M+GAP1-C+EAD1*->CASPASE+EAD1->
  - ZnR+DO-GTPase2->DO-GTPase1->GAP1-N1+GAP1-M+GAP1-C+EAD1->CASPASE+EAD1*->

- **GCA_900115275.1; Pseudomonas syringae; counts= 2; multicellularity status: FALSE**
  - GAP2->ZnR+DO-GTPase2->DO-GTPase1->GAP1-N1+GAP1-M+GAP1-C+EAD1*->CASPASE+EAD1->
  - ZnR+DO-GTPase2->DO-GTPase1->GAP1-N1+GAP1-M+GAP1-C+EAD1->CASPASE+EAD1*->
- **GCA_900215445.1; Serratia sp. JKS296; counts= 2; multicellularity status: FALSE**
  - CASPASE+EAD1*->
  - GAP2->ZnR+DO-GTPase2->DO-GTPase1->GAP1-N1+GAP1-M+GAP1-C+EAD1*->CASPASE+EAD1->
- **GCA_000368665.1; Acinetobacter junii; counts= 2; multicellularity status: FALSE**
  - GAP2->ZnR+DO-GTPase2->DO-GTPase1->GAP1-N1+GAP1-M+GAP1-C+EAD1*->CASPASE+EAD1->
  - ZnR+DO-GTPase2->DO-GTPase1->GAP1-N1+GAP1-M+GAP1-C+EAD1->CASPASE+EAD1*->
- **GCA_001896005.1; Acinetobacter; counts= 2; multicellularity status: FALSE**
  - PNP_UDP_1+EAD1*->?->ZnR+DO-GTPase2->DO-GTPase1->
  - ZnR+DO-GTPase2->DO-GTPase1->GAP1-N1+GAP1-M+GAP1-C+EAD1*->
- **GCA_000155555.1; Coleofasciculus chthonoplastes; counts= 2; multicellularity status: TRUE**
  - E1->?->EAD1+Trypsin->EAD1+GUN4*->
  - E1->?->EAD1+Trypsin*->EAD1+GUN4->
- **GCA_000190755.3; Methylobacter tundripaludum; counts= 2; multicellularity status: TRUE**
  - PNP_UDP_1+EAD1*->
  - ZnR+DO-GTPase2->DO-GTPase1->GAP1-N1+GAP1-M+GAP1-C+EAD1*->
- **GCA_000282095.2; Rhizobium sp. CF080; counts= 2; multicellularity status: TRUE**
  - GAP1-N1+GAP1-M+GAP1-C+EAD1->CASPASE+EAD1*->
  - GAP1-N1+GAP1-M+GAP1-C+EAD1*->CASPASE+EAD1->
- **GCA_000178855.1; Ktedonobacter racemifer; counts= 2; multicellularity status: TRUE**
  - EAD1+Trypsin->EAD1+STAND_NTPase+STAND_NTPase+wHTH+TPRs+EAD1+Trypsin(i)+PDZ*->
  - EAD1+Trypsin*->EAD1+STAND_NTPase+STAND_NTPase+wHTH+TPRs+EAD1+Trypsin(i)+PDZ->
- **GCF_000332095.2; Leptolyngbya sp. PCC 6406; counts= 2; multicellularity status: TRUE**
  - EAD1+Trypsin->TM+TM+TM->EAD1+VMAP-C*->MoxR->
  - EAD1+Trypsin*->TM+TM+TM->EAD1+VMAP-C->MoxR->
- **GCA_000296815.2; Fibrisoma limi; counts= 2; multicellularity status: TRUE**
  - DO-GTPase1->GAP1-N1+GAP1-M+GAP1-C+EAD1->PNP_UDP_1+EAD1*->
  - DO-GTPase1->GAP1-N1+GAP1-M+GAP1-C+EAD1*->PNP_UDP_1+EAD1->
- **GCA_000314635.1; Acinetobacter baumannii; counts= 2; multicellularity status: FALSE**
  - GAP2->ZnR+DO-GTPase2->DO-GTPase1->GAP1-N1+GAP1-M+GAP1-C+EAD1*->CASPASE+EAD1->
  - ZnR+DO-GTPase2->DO-GTPase1->GAP1-N1+GAP1-M+GAP1-C+EAD1->CASPASE+EAD1*->

- **GCA_000014265.1; Trichodesmium erythraeum; counts= 2; multicellularity status: TRUE**
  - EAD1+Trypsin->TM+TM+TM->EAD1+VMAP-M0+VMAP-C*->MoxR->
  - EAD1+Trypsin*->TM+TM+TM->EAD1+VMAP-M0+VMAP-C->MoxR->
- **GCA_000017645.1; Xanthobacter autotrophicus; counts= 2; multicellularity status: FALSE**
  - GAP2->ZnR+DO-GTPase2->DO-GTPase1->GAP1-N1+GAP1-M+GAP1-C+EAD1*->CASPASE+EAD1->
  - ZnR+DO-GTPase2->DO-GTPase1->GAP1-N1+GAP1-M+GAP1-C+EAD1->CASPASE+EAD1*->
- **GCA_000176855.2; Acidovorax avenae; counts= 2; multicellularity status: FALSE**
  - DO-GTPase2->DO-GTPase1->GAP1-N1+GAP1-M+GAP1-C+EAD1->CASPASE+EAD1*->
  - DO-GTPase2->DO-GTPase1->GAP1-N1+GAP1-M+GAP1-C+EAD1*->CASPASE+EAD1->
- **GCA_000177615.2; Frankia symbiont of Datisca glomerata; counts= 2; multicellularity status: TRUE**
  - EAD1+PH?*->
  - TIR+EAD1*->
- **GCA_000092385.1; Streptomyces; counts= 2; multicellularity status: TRUE**
  - EAD1+Trypsin->EAD1+EAD7*->
  - EAD1+Trypsin*->EAD1+EAD7->
- **GCA_000284295.1; Actinoplanes missouriensis; counts= 2; multicellularity status: TRUE**
  - EAD1+P-loop_NTPase(i)->MoxR->?->EAD1+Trypsin*->
  - EAD1+P-loop_NTPase(i)*->MoxR->?->EAD1+Trypsin->
- **GCA_000317495.1; Crinalium epipsammum; counts= 2; multicellularity status: NA**
  - EAD1->?->EAD1+CASPASE*->
  - Trypsin->EAD1+P-loop_NTPase(i)*->MoxR->helical_region+vWA->
- **GCA_000317435.1; Calothrix parietina; counts= 2; multicellularity status: TRUE**
  - EAD8+EAD1->EAD1+YukDl-Nterm+UB+E2*->JAB-N+JAB->?->CDC48-like->
  - EAD8+EAD1*->EAD1+YukDl-Nterm+UB+E2->JAB-N+JAB->
- **GCA_000320385.2; Sinorhizobium meliloti; counts= 2; multicellularity status: TRUE**
  - GAP2->ZnR+DO-GTPase2->DO-GTPase1->GAP1-N1+GAP1-M+GAP1-C+EAD1*->CASPASE+EAD1->
  - ZnR+DO-GTPase2->DO-GTPase1->GAP1-N1+GAP1-M+GAP1-C+EAD1->CASPASE+EAD1*->
- **GCA_002870665.1; Fischerella muscicola; counts= 2; multicellularity status: NA**
  - EAD1+CASPASE->EAD1*->
  - EAD1+CASPASE*->EAD1->
- **GCA_000287135.2; Vibrio ordalii; counts= 2; multicellularity status: FALSE**
  - GAP2->ZnR+DO-GTPase2->DO-GTPase1->GAP1-N1+GAP1-M+GAP1-C+EAD1*->
  - PNP_UDP_1+EAD1*->GAP2->ZnR+DO-GTPase2->DO-GTPase1->

- **GCA_000495775.1; Asticcacaulis benevestitus; counts= 2; multicellularity status: FALSE**
  - Trypsin+Endonuclease_NS(HNH)->?->EAD1+Trypsin->EAD1+Trypsin*->
  - Trypsin+Endonuclease_NS(HNH)->?->EAD1+Trypsin*->EAD1+Trypsin->
- **GCF_000383615.1; Streptomyces canus; counts= 2; multicellularity status: TRUE**
  - GAP1-N1+GAP1-M+GAP1-C+EAD1->CASPASE+EAD1*->
  - GAP1-N1+GAP1-M+GAP1-C+EAD1*->CASPASE+EAD1->
- **GCF_000383675.1; Terracoccus sp. 273MFTsu3.1; counts= 2; multicellularity status: TRUE**
  - EAD1+Trypsin->EAD1+STAND_NTPase*->STAND_NTPase+wHTH+TPRs->
  - EAD1+Trypsin*->EAD1+STAND_NTPase->STAND_NTPase+wHTH+TPRs->
- **GCF_000379645.1; Actinoplanes globisporus; counts= 2; multicellularity status: TRUE**
  - EAD1+CASPASE*->?->?->ZnF->
  - ZnF->E1(active)+E1_C_unk->EAD1+YukDl-Nterm+UB+E2*->JAB-N+JAB->?->CDC48-like->
- **GCA_002234385.1; Amycolatopsis alba; counts= 2; multicellularity status: TRUE**
  - EAD1+Trypsin->EAD1+STAND_NTPase*->STAND_NTPase+wHTH+TPRs+CASPASE->
  - EAD1+Trypsin*->EAD1+STAND_NTPase->STAND_NTPase+wHTH+TPRs+CASPASE->
- **GCA_003947255.1; Amycolatopsis balhimycina; counts= 2; multicellularity status: TRUE**
  - EAD1+CASPASE->E1(active)+E1_C_unk->EAD1+YukDl-Nterm+UB+E2*->
  - EAD1+CASPASE*->E1(active)+E1_C_unk->EAD1+YukDl-Nterm+UB+E2->
- **GCA_000418325.1; Sorangium cellulosum; counts= 2; multicellularity status: TRUE**
  - EAD1+APATPase+wHTH+TPRs+BetaPropeller+EAD7*->||<-EAD7+CASPASE
  - EAD1+Trypsin*->?->MoxR->
- **GCF_000412185.1; Euryhalocaulis caribicus; counts= 2; multicellularity status: FALSE**
  - ZnR+DO-GTPase2->DO-GTPase1->GAP1-N1+GAP1-M+GAP1-C+DNA-glycosylase+EAD1->CASPASE+EAD1*->
  - ZnR+DO-GTPase2->DO-GTPase1->GAP1-N1+GAP1-M+GAP1-C+DNA-glycosylase+EAD1*->CASPASE+EAD1->
- **GCA_000502815.1; Mesorhizobium; counts= 2; multicellularity status: NA**
  - EAD1+Trypsin*->?->STAND_NTPase+wHTH+TPRs->
  - Trypsin+Endonuclease_NS(HNH)->EAD1+Trypsin*->
- **GCF_000452605.1; Pseudomonas syringae; counts= 2; multicellularity status: FALSE**
  - GAP1-N1+GAP1-M+GAP1-C+EAD1->CASPASE+EAD1*->
  - GAP1-N1+GAP1-M+GAP1-C+EAD1*->CASPASE+EAD1->
- **GCF_000490295.1; Vibrio parahaemolyticus; counts= 2; multicellularity status: FALSE**
  - EAD1->CASPASE+EAD1*->
  - EAD1*->CASPASE+EAD1->

- **GCF_000425065.1; Actinomadura rifamycini; counts= 2; multicellularity status: TRUE**
  - <-EAD1+STAND_NTPase+TPRs||EAD1+Trypsin*->
  - <-EAD1+Trypsin||EAD1+STAND_NTPase+TPRs*->
- **GCF_000515315.1; Asinibacterium sp. OR53; counts= 2; multicellularity status: FALSE**
  - ZnR+DO-GTPase2->DO-GTPase1->GAP1-N1+GAP1-M+GAP1-C+EAD1->PNP_UDP_1+EAD1*->
  - ZnR+DO-GTPase2->DO-GTPase1->GAP1-N1+GAP1-M+GAP1-C+EAD1*->PNP_UDP_1+EAD1->
- **GCA_000769935.1; Flavobacterium subsaxonicum; counts= 2; multicellularity status: NA**
  - EAD1->?->EAD1+Trypsin*->Endonuclease_NS(HNH)->
  - EAD1*->?->EAD1+Trypsin->Endonuclease_NS(HNH)->
- **GCF_000686005.1; Phyllobacterium sp. UNC302MFCol5.2; counts= 2; multicellularity status: TRUE**
  - EAD1+Trypsin*->
  - SP+Ig-like+TM+EAD1*->
- **GCF_000718785.1; Streptomyces sp. NRRL WC-3626; counts= 2; multicellularity status: TRUE**
  - EAD1+Trypsin->EAD1+STAND_NTPase*->STAND_NTPase+wHTH+TPRs+AB_hydrolase->
  - EAD1+Trypsin*->EAD1+STAND_NTPase->STAND_NTPase+wHTH+TPRs+AB_hydrolase->
- **GCF_000719355.1; Streptomyces griseus; counts= 2; multicellularity status: TRUE**
  - EAD1+Trypsin->EAD1+STAND_NTPase*->STAND_NTPase+wHTH+TPRs+AB_hydrolase->
  - EAD1+Trypsin*->EAD1+STAND_NTPase->STAND_NTPase+wHTH+TPRs+AB_hydrolase->
- **GCF_000725475.1; Streptomyces durhamensis; counts= 2; multicellularity status: TRUE**
  - EAD1+CASPASE->ZnF->E1(active)+E1_C_unk->EAD1+YukDl-Nterm+UB+E2*->JAB-N+JAB->?->CDC48-like->
  - EAD1+CASPASE*->ZnF->E1(active)+E1_C_unk->EAD1+YukDl-Nterm+UB+E2->
- **GCF_000761035.1; Rhodococcus fascians; counts= 2; multicellularity status: TRUE**
  - DO-GTPase1->GAP1-N1+GAP1-M+GAP1-C+EAD1->PNP_UDP_1+EAD1*->
  - DO-GTPase1->GAP1-N1+GAP1-M+GAP1-C+EAD1*->PNP_UDP_1+EAD1->
- **GCA_000702925.2; Methylomonas sp. LW13; counts= 2; multicellularity status: FALSE**
  - GAP2->ZnR+DO-GTPase2->DO-GTPase1->GAP1-N1+GAP1-M+GAP1-C+EAD1*->
  - PNP_UDP_1+EAD1*->GAP2->ZnR+DO-GTPase2->DO-GTPase1->
- **GCA_001270515.1; Streptomyces; counts= 2; multicellularity status: TRUE**
  - EAD1+Trypsin->EAD1+STAND_NTPase*->STAND_NTPase+wHTH+TPRs->
  - EAD1+Trypsin*->EAD1+STAND_NTPase->STAND_NTPase+wHTH+TPRs->
- **GCF_000717055.1; Streptomyces iakyrus; counts= 2; multicellularity status: TRUE**
  - EAD1+Trypsin->EAD1+STAND_NTPase*->STAND_NTPase+wHTH+TPRs+AB_hydrolase->
  - EAD1+Trypsin*->EAD1+STAND_NTPase->STAND_NTPase+wHTH+TPRs+AB_hydrolase->

- **GCA_000696185.1; Kitasatospora cheerisanensis; counts= 2; multicellularity status: TRUE**
  - EAD1->Trypsin->EAD1+STAND_NTPase*->
  - EAD1*->Trypsin->EAD1+STAND_NTPase->
- **GCF_000262465.1; Frankia sp. QA3; counts= 2; multicellularity status: TRUE**
  - E1(active)+E1_C_unk->EAD1+YukDl-Nterm+UB+E2*->JAB-N+JAB->
  - MoxR->?->?->EAD1+??+YukD-like_Ub*->||<-E1(active)+E1-ZnR_C
- **GCF_000177675.1; Frankia sp. EUN1f; counts= 2; multicellularity status: TRUE**
  - EAD1*->
  - EAD1+APATPase+wHTH+TPRs*->
- **GCA_000695255.1; Photobacterium galatheae; counts= 2; multicellularity status: FALSE**
  - GAP2->ZnR+DO-GTPase2->DO-GTPase1->GAP1-N1+GAP1-M+GAP1-C+EAD1*->
  - PNP_UDP_1+EAD1*->GAP2->ZnR+DO-GTPase2->DO-GTPase1->
- **GCF_000746395.1; Streptomyces mirabilis; counts= 2; multicellularity status: TRUE**
  - EAD1+CASPASE->ZnF->E1(active)+E1_C_unk->EAD1+YukDl-Nterm+UB+E2*->JAB-N+JAB->?->CDC48-like->
  - EAD1+CASPASE*->ZnF->E1(active)+E1_C_unk->EAD1+YukDl-Nterm+UB+E2->
- **GCA_000788395.1; Pseudoalteromonas piratica; counts= 2; multicellularity status: FALSE**
  - GAP2->ZnR+DO-GTPase2->DO-GTPase1->GAP1-N1+GAP1-M+GAP1-C+EAD1*->
  - PNP_UDP_1+EAD1*->GAP2->ZnR+DO-GTPase2->DO-GTPase1->
- **GCA_000747525.1; Sphingobacterium sp. ML3W; counts= 2; multicellularity status: FALSE**
  - GAP1-N1+GAP1-M+GAP1-C+EAD1->?->?->EAD1+Trypsin*->Endonuclease_NS(HNH)->
  - GAP2->ZnR+DO-GTPase2->DO-GTPase1->GAP1-N1+GAP1-M+GAP1-C+EAD1*->?->?->EAD1+Trypsin->
- **GCA_000773415.1; Burkholderia pseudomallei; counts= 2; multicellularity status: FALSE**
  - ZnR+DO-GTPase2->DO-GTPase1->GAP1-N1+GAP1-M+GAP1-C+EAD1->CASPASE+EAD1*->
  - ZnR+DO-GTPase2->DO-GTPase1->GAP1-N1+GAP1-M+GAP1-C+EAD1*->CASPASE+EAD1->
- **GCF_000813415.1; Plesiomonas shigelloides; counts= 2; multicellularity status: FALSE**
  - GAP2->ZnR+DO-GTPase2->DO-GTPase1->GAP1-N1+GAP1-M+GAP1-C+EAD1*->
  - PNP_UDP_1+EAD1*->GAP2->ZnR+DO-GTPase2->DO-GTPase1->
- **GCA_000746665.1; Vibrio vulnificus; counts= 2; multicellularity status: FALSE**
  - GAP2->ZnR+DO-GTPase2->DO-GTPase1->GAP1-N1+GAP1-M+GAP1-C+EAD1*->
  - PNP_UDP_1+EAD1*->GAP2->ZnR+DO-GTPase2->DO-GTPase1->
- **GCA_000830005.1; Streptomyces vietnamensis; counts= 2; multicellularity status: TRUE**
  - EAD1+Trypsin->EAD1+STAND_NTPase*->STAND_NTPase+wHTH+TPRs->
  - EAD1+Trypsin*->EAD1+STAND_NTPase->STAND_NTPase+wHTH+TPRs->

- **GCF_000018005.1; Frankia sp. EAN1pec; counts= 2; multicellularity status: TRUE**
  - EAD2+EAD1*->EAD2+VMAP-M0+VMAP-C->MoxR->vWA+APATPase+wHTH+TPRs->
  - pntlT4_fusion_domain+EAD1*->
- **GCF_000221965.1; Xanthomonas campestris; counts= 2; multicellularity status: FALSE**
  - ZnR+DO-GTPase2->DO-GTPase1->GAP1-N1+GAP1-M+GAP1-C+EAD1->CASPASE+EAD1*->
  - ZnR+DO-GTPase2->DO-GTPase1->GAP1-N1+GAP1-M+GAP1-C+EAD1*->CASPASE+EAD1->
- **GCA_000601485.1; Chondromyces apiculatus; counts= 2; multicellularity status: NA**
  - STAND_NTPase+wHTH+TPRs+BetaPropeller+EAD1->EAD1+Trypsin*->
  - STAND_NTPase+wHTH+TPRs+BetaPropeller+EAD1*->EAD1+Trypsin->
- **GCF_000818475.1; Vibrio hyugaensis; counts= 2; multicellularity status: FALSE**
  - GAP2->ZnR+DO-GTPase2->DO-GTPase1->GAP1-N1+GAP1-M+GAP1-C+EAD1*->
  - PNP_UDP_1+EAD1*->GAP2->ZnR+DO-GTPase2->DO-GTPase1->
- **GCA_000938735.2; Sphingobacterium sp. PM2-P1-29; counts= 2; multicellularity status: FALSE**
  - DO-GTPase1->GAP1-N1+GAP1-M+GAP1-C+EAD1->?->EAD1+Trypsin*->Endonuclease_NS(HNH)->
  - DO-GTPase1->GAP1-N1+GAP1-M+GAP1-C+EAD1*->?->EAD1+Trypsin->Endonuclease_NS(HNH)->
- **GCA_000968575.1; Pseudomonas kilonensis; counts= 2; multicellularity status: FALSE**
  - ZnR+DO-GTPase2->DO-GTPase1->GAP1-N1+GAP1-M+GAP1-C+EAD1->CASPASE+EAD1*->
  - ZnR+DO-GTPase2->DO-GTPase1->GAP1-N1+GAP1-M+GAP1-C+EAD1*->CASPASE+EAD1->
- **GCA_000974985.2; Streptomyces mangrovisoli; counts= 2; multicellularity status: TRUE**
  - EAD1+Trypsin->EAD1+STAND_NTPase*->STAND_NTPase+wHTH+TPRs+AB_hydrolase->
  - EAD1+Trypsin*->EAD1+STAND_NTPase->STAND_NTPase+wHTH+TPRs+AB_hydrolase->
- **GCA_000952035.2; Streptomyces lydicus; counts= 2; multicellularity status: TRUE**
  - *
  - EAD1+P-loop_helicase+??*->
- **GCA_001013325.1; Vibrio; counts= 2; multicellularity status: NA**
  - GAP2->ZnR+DO-GTPase2->DO-GTPase1->GAP1-N1+GAP1-M+GAP1-C+EAD1*->
  - PNP_UDP_1+EAD1*->GAP2->ZnR+DO-GTPase2->DO-GTPase1->
- **GCF_000798835.1; Shewanella sp. ZOR0012; counts= 2; multicellularity status: FALSE**
  - GAP2->ZnR+DO-GTPase2->DO-GTPase1->GAP1-N1+GAP1-M+GAP1-C+EAD1*->CASPASE+EAD1->
  - ZnR+DO-GTPase2->DO-GTPase1->GAP1-N1+GAP1-M+GAP1-C+EAD1->CASPASE+EAD1*->
- **GCA_001028135.1; Xenorhabdus; counts= 2; multicellularity status: NA**
  - EAD1+P-loop_helicase->CASPASE+EAD1*->
  - EAD1+P-loop_helicase*->CASPASE+EAD1->

- **GCF_001044185.1; Streptomyces sp. HNS054; counts= 2; multicellularity status: NA**
  - EAD1+Trypsin->EAD1+STAND_NTPase*->STAND_NTPase+wHTH+TPRs+AB_hydrolase->
  - EAD1+Trypsin*->EAD1+STAND_NTPase->STAND_NTPase+wHTH+TPRs+AB_hydrolase->
- **GCA_001045445.1; Chryseobacterium sp. BLS98; counts= 2; multicellularity status: FALSE**
  - DO-GTPase1->GAP1-N1+GAP1-M+GAP1-C+EAD1->?->EAD1+Trypsin*->Endonuclease_NS(HNH)->
  - GAP2->ZnR+DO-GTPase2->DO-GTPase1->GAP1-N1+GAP1-M+GAP1-C+EAD1*->?->EAD1+Trypsin->Endonuclease_NS(HNH)->
- **GCA_001189295.1; Chondromyces crocatus; counts= 2; multicellularity status: TRUE**
  - STAND_NTPase+wHTH+TPRs+BetaPropeller+EAD1->EAD1+Trypsin*->
  - STAND_NTPase+wHTH+TPRs+BetaPropeller+EAD1*->EAD1+Trypsin->
- **GCF_000514395.1; Pseudomonas sp. URMO17WK12:I12; counts= 2; multicellularity status: NA**
  - GAP1-N1+GAP1-M+GAP1-C+EAD1->CASPASE+EAD1*->
  - GAP1-N1+GAP1-M+GAP1-C+EAD1*->CASPASE+EAD1->
- **GCF_000828075.2; Tolypothrix campylonemoides; counts= 2; multicellularity status: NA**
  - EAD1+low_complexity*->
  - TIR+EAD1*->
- **GCF_001273715.1; Vibrio alginolyticus; counts= 2; multicellularity status: FALSE**
  - GAP2->ZnR+DO-GTPase2->DO-GTPase1->GAP1-N1+GAP1-M+GAP1-C+EAD1*->
  - PNP_UDP_1+EAD1*->GAP2->ZnR+DO-GTPase2->DO-GTPase1->
- **GCA_001275735.1; Pseudomonas coronafaciens; counts= 2; multicellularity status: FALSE**
  - ZnR+DO-GTPase2->DO-GTPase1->GAP1-N1+GAP1-M+GAP1-C+EAD1->CASPASE+EAD1*->
  - ZnR+DO-GTPase2->DO-GTPase1->GAP1-N1+GAP1-M+GAP1-C+EAD1*->CASPASE+EAD1->
- **GCA_001280885.1; Streptomyces sp. XY332; counts= 2; multicellularity status: TRUE**
  - EAD1+Trypsin->EAD1+STAND_NTPase*->STAND_NTPase+wHTH+TPRs->
  - EAD1+Trypsin*->EAD1+STAND_NTPase->STAND_NTPase+wHTH+TPRs->
- **GCF_001414035.1; Frankia sp. ACN1ag; counts= 2; multicellularity status: TRUE**
  - EAD1+cNMP_cyclase+TIR*->
  - pntlT4_fusion_domain+EAD1*->
- **GCF_000948395.1; Frankia sp. CpI1-P; counts= 2; multicellularity status: TRUE**
  - EAD1+BetaPropeller*->
  - EAD1+cNMP_cyclase+extension*->
- **GCA_001421665.1; Sphingobium sp. Leaf26; counts= 2; multicellularity status: FALSE**
  - GAP2->ZnR+DO-GTPase2->DO-GTPase1->GAP1-N1+GAP1-M+GAP1-C+EAD1*->CASPASE+EAD1->
  - ZnR+DO-GTPase2->DO-GTPase1->GAP1-N1+GAP1-M+GAP1-C+EAD1->CASPASE+EAD1*->

- **GCA_001424875.1; Kitasatospora; counts= 2; multicellularity status: TRUE**
  - EAD1+Trypsin->EAD1+STAND_NTPase*->STAND_NTPase+wHTH+TPRs->
  - EAD1+Trypsin*->EAD1+STAND_NTPase->STAND_NTPase+wHTH+TPRs->
- **GCA_001444405.1; Pseudoalteromonas phenolica; counts= 2; multicellularity status: FALSE**
  - GAP2->ZnR+DO-GTPase2->DO-GTPase1->GAP1-N1+GAP1-M+GAP1-C+EAD1*->
  - PNP_UDP_1+EAD1*->GAP2->ZnR+DO-GTPase2->DO-GTPase1->
- **GCA_001458375.1; Tropicibacter naphthalenivorans; counts= 2; multicellularity status: FALSE**
  - GAP2->ZnR+DO-GTPase2->DO-GTPase1->GAP1-N1+GAP1-M+GAP1-C+EAD1*->CASPASE+EAD1->
  - ZnR+DO-GTPase2->DO-GTPase1->GAP1-N1+GAP1-M+GAP1-C+EAD1->CASPASE+EAD1*->
- **GCA_001467105.1; Pseudomonas syringae; counts= 2; multicellularity status: FALSE**
  - ZnR+DO-GTPase2->DO-GTPase1->GAP1-N1+GAP1-M+GAP1-C+EAD1->CASPASE+EAD1*->
  - ZnR+DO-GTPase2->DO-GTPase1->GAP1-N1+GAP1-M+GAP1-C+EAD1*->CASPASE+EAD1->
- **GCA_001425445.1; Devosia sp. Leaf420; counts= 2; multicellularity status: FALSE**
  - GAP2->ZnR+DO-GTPase2->DO-GTPase1->GAP1-N1+GAP1-M+GAP1-C+EAD1*->CASPASE+EAD1->CASPASE+TPRs->
  - ZnR+DO-GTPase2->DO-GTPase1->GAP1-N1+GAP1-M+GAP1-C+EAD1->CASPASE+EAD1*->CASPASE+TPRs->
- **GCA_001611795.1; Streptomyces sp. S10(2016); counts= 2; multicellularity status: TRUE**
  - *
  - EAD1+Trypsin*->
- **GCF_900079515.1; Shewanella sp. Alg231_23; counts= 2; multicellularity status: FALSE**
  - GAP2->ZnR+DO-GTPase2->DO-GTPase1->GAP1-N1+GAP1-M+GAP1-C+EAD1*->
  - PNP_UDP_1+EAD1*->GAP2->ZnR+DO-GTPase2->DO-GTPase1->
- **GCA_001698815.1; Pseudomonas syringae; counts= 2; multicellularity status: FALSE**
  - ZnR+DO-GTPase2->DO-GTPase1->GAP1-N1+GAP1-M+GAP1-C+EAD1->CASPASE+EAD1*->
  - ZnR+DO-GTPase2->DO-GTPase1->GAP1-N1+GAP1-M+GAP1-C+EAD1*->CASPASE+EAD1->
- **GCA_001969965.1; Streptomyces sparsogenes; counts= 2; multicellularity status: TRUE**
  - EAD1+Trypsin->EAD1+EAD7*->?->?->FGS-fold_domain->
  - EAD1+Trypsin*->EAD1+EAD7->
- **GCF_001685485.1; Formosa haliotis; counts= 2; multicellularity status: FALSE**
  - ??+EAD1->?->?->EAD1+Trypsin*->Endonuclease_NS(HNH)->
  - GAP2->PSE->PSE->?->??+EAD1*->?->?->EAD1+Trypsin->
- **GCF_001571225.1; Hydrogenophaga palleronii; counts= 2; multicellularity status: FALSE**
  - DO-GTPase1->GAP1-N1+GAP1-M+GAP1-C+EAD1->CASPASE+EAD1*->
  - DO-GTPase1->GAP1-N1+GAP1-M+GAP1-C+EAD1*->CASPASE+EAD1->

- **GCF_000346485.2; Scytonema hofmannii; counts= 2; multicellularity status: TRUE**
  - ??+EAD1->GUN4+EAD1*->
  - PNP_UDP_1+EAD1*->
- **GCA_001548375.1; Nostoc sp. NIES-3756; counts= 2; multicellularity status: NA**
  - EAD1+Trypsin->TM+TM+TM->EAD1+STAND_NTPase+TPRs*->
  - EAD1+Trypsin*->TM+TM+TM->EAD1+STAND_NTPase+TPRs->
- **GCF_001746365.1; Streptomyces sp. LUP30; counts= 2; multicellularity status: TRUE**
  - EAD1+CASPASE->EAD1+P-loop_helicase*->
  - EAD1+CASPASE*->EAD1+P-loop_helicase->
- **GCA_001858125.1; Cylindrospermopsis raciborskii; counts= 2; multicellularity status: NA**
  - RHH->EAD1+Trypsin->TM+TM+TM->EAD1+P-loop_NTPase*->MoxR->helical_region+vWA->
  - RHH->EAD1+Trypsin*->TM+TM+TM->EAD1+P-loop_NTPase->MoxR->
- **GCA_900114875.1; Chryseobacterium limigenitum; counts= 2; multicellularity status: FALSE**
  - GAP2->ZnR+DO-GTPase2->DO-GTPase1->GAP1-N1+GAP1-M+GAP1-C+EAD1*->PNP_UDP_1+EAD1->
  - ZnR+DO-GTPase2->DO-GTPase1->GAP1-N1+GAP1-M+GAP1-C+EAD1->PNP_UDP_1+EAD1*->
- **GCA_001904715.1; Nostoc calcicola; counts= 2; multicellularity status: NA**
  - EAD1+Trypsin->EAD1+FGS-fold_domain*->||<-STAND_NTPase+FGS-fold_domain
  - EAD1+Trypsin*->EAD1+FGS-fold_domain->||<-STAND_NTPase+FGS-fold_domain
- **GCA_002217275.1; Flavobacterium; counts= 2; multicellularity status: FALSE**
  - GAP2->ZnR+DO-GTPase2->DO-GTPase1->GAP1-N1+GAP1-M+GAP1-C+EAD1*->PNP_UDP_1+EAD1->
  - ZnR+DO-GTPase2->DO-GTPase1->GAP1-N1+GAP1-M+GAP1-C+EAD1->PNP_UDP_1+EAD1*->
- **GCA_001939115.1; Leptolyngbya sp. 'hensonii'; counts= 2; multicellularity status: NA**
  - EAD1+Trypsin->TM+TM+TM->EAD1+??+VMAP-C*->MoxR->vWA+Beta-rich+FGS-fold_domain->
  - EAD1+Trypsin*->TM+TM+TM->EAD1+??+VMAP-C->MoxR->
- **GCF_001984055.1; Variovorax sp. KK3; counts= 2; multicellularity status: NA**
  - EAD1+Trypsin->EAD1+P-loop_NTPase(i)*->MoxR->helical_region+vWA->STAND_NTPase+TPRs+Lon-N+METALLOPORTEASE->
  - EAD1+Trypsin*->EAD1+P-loop_NTPase(i)->MoxR->helical_region+vWA->
- **GCA_004138325.1; Spirosoma sp. 209; counts= 2; multicellularity status: NA**
  - EAD1+Trypsin->EAD1+SIR2*->STAND_NTPase+wHTH+TM+BetaPropeller->
  - EAD1+Trypsin*->EAD1+SIR2->STAND_NTPase+wHTH+TM+BetaPropeller->
- **GCF_002009845.1; Leptospira alexanderi; counts= 2; multicellularity status: FALSE**
  - GAP2->ZnR+DO-GTPase2->DO-GTPase1->GAP1-N1+GAP1-M+GAP1-C+EAD1*->PNP_UDP_1+EAD1->
  - ZnR+DO-GTPase2->DO-GTPase1->GAP1-N1+GAP1-M+GAP1-C+EAD1->PNP_UDP_1+EAD1*->

- **GCA_900167465.1; Geobacter thiogenes; counts= 2; multicellularity status: FALSE**
  - ??+PNP_UDP_1+EAD1*->GAP2->ZnR+DO-GTPase2->DO-GTPase1->
  - GAP2->ZnR+DO-GTPase2->DO-GTPase1->GAP1-N1+GAP1-M+GAP1-C+EAD1*->
- **GCA_002027345.1; Cylindrospermopsis raciborskii; counts= 2; multicellularity status: NA**
  - RHH->EAD1+Trypsin->TM+TM+TM->EAD1+P-loop_NTPase*->MoxR->helical_region+vWA->
  - RHH->EAD1+Trypsin*->TM+TM+TM->EAD1+P-loop_NTPase->MoxR->
- **GCA_002027195.1; Streptomyces sp. GKU 895; counts= 2; multicellularity status: TRUE**
  - EAD1+CASPASE->E1+E1_C_unk->E1(active)+E1_C->EAD1+YukDl-Nterm+UB+E2*->JAB-N+JAB->?->CDC48-like->
  - EAD1+CASPASE*->E1+E1_C_unk->E1(active)+E1_C->EAD1+YukDl-Nterm+UB+E2->
- **GCF_001854645.1; Frankia sp. EUN1h; counts= 2; multicellularity status: TRUE**
  - EAD1+APATPase+wHTH+TPRs*->
  - EAD1+APATPase+wHTH+TPRs*->
- **GCF_001854655.1; Frankia sp. Cc1.17; counts= 2; multicellularity status: TRUE**
  - EAD1+BetaPropeller*->
  - EAD1+TIR*->
- **GCA_900155475.1; Methylomagnum ishizawai; counts= 2; multicellularity status: FALSE**
  - PNP_UDP_1+EAD1*->||<-?||?->ZnR+DO-GTPase2->
  - ZnR+DO-GTPase2->DO-GTPase1->GAP1-N1+GAP1-M+GAP1-C+EAD1*->
- **GCA_002151545.1; Sphingobacteriaceae bacterium GW460-11-11-14-LB5; counts= 2; multicellularity status: NA**
  - EAD1+Trypsin->EAD1+SIR2*->STAND_NTPase+wHTH+TM+BetaPropeller->
  - EAD1+Trypsin*->EAD1+SIR2->STAND_NTPase+wHTH+TM+BetaPropeller->
- **GCF_002150765.1; Lentzea kentuckyensis; counts= 2; multicellularity status: TRUE**
  - EAD1+Trypsin->EAD1+STAND_NTPase*->STAND_NTPase+wHTH+TPRs+CASPASE->
  - EAD1+Trypsin*->EAD1+STAND_NTPase->STAND_NTPase+wHTH+TPRs+CASPASE->
- **GCA_002154695.1; Nostoc sp. T09; counts= 2; multicellularity status: TRUE**
  - E1(active)+E1_C_unk->?->TCAD3+TCAD4+CASPASE+EAD1->EAD1+YukDl-Nterm+UB+E2*->JAB-N+JAB->?->CDC48-like->
  - ZnF->E1(active)+E1_C_unk->?->TCAD3+TCAD4+CASPASE+EAD1*->EAD1+YukDl-Nterm+UB+E2->JAB-N+JAB->
- **GCA_002157355.1; Variovorax sp. JS1663; counts= 2; multicellularity status: NA**
  - EAD1+Trypsin->EAD1+P-loop_NTPase(i)*->MoxR->helical_region+vWA->STAND_NTPase+TPRs+Lon-N+METALLOPORTEASE->
  - EAD1+Trypsin*->EAD1+P-loop_NTPase(i)->MoxR->helical_region+vWA->
- **GCF_900143175.1; Xanthomonas retroflexus; counts= 2; multicellularity status: FALSE**
  - *
  - ZnR+DO-GTPase2->DO-GTPase1->GAP1-N1+GAP1-M+GAP1-C+EAD1->CASPASE+EAD1*->

- **GCA_002209385.1; Methylovulum psychrotolerans; counts= 2; multicellularity status: FALSE**
  - GAP2->ZnR+DO-GTPase2->DO-GTPase1->GAP1-N1+GAP1-M+GAP1-C+EAD1*->
  - PNP_UDP_1+EAD1*->GAP2->ZnR+DO-GTPase2->DO-GTPase1->
- **GCA_002224125.1; Streptomyces sp. NBS 14/10; counts= 2; multicellularity status: TRUE**
  - EAD1+Trypsin->EAD1+EAD7*->
  - EAD1+Trypsin*->EAD1+EAD7->
- **GCA_900109175.1; Pseudomonas linyingensis; counts= 2; multicellularity status: FALSE**
  - ZnR+DO-GTPase2->DO-GTPase1->GAP1-N1+GAP1-M+GAP1-C+EAD1->CASPASE+EAD1*->
  - ZnR+DO-GTPase2->DO-GTPase1->GAP1-N1+GAP1-M+GAP1-C+EAD1*->CASPASE+EAD1->
- **GCA_900113065.1; Aureimonas phyllosphaerae; counts= 2; multicellularity status: FALSE**
  - GAP1-N1+GAP1-M+GAP1-C+EAD1->CASPASE+EAD1*->
  - GAP1-N1+GAP1-M+GAP1-C+EAD1*->CASPASE+EAD1->
- **GCA_900115505.1; Geodermatophilus sp. DSM 44208; counts= 2; multicellularity status: NA**
  - EAD1+Trypsin->EAD1+P-loop_NTPase(i)*->MoxR->helical_region+vWA->
  - EAD1+Trypsin*->EAD1+P-loop_NTPase(i)->MoxR->helical_region+vWA->
- **GCA_900107105.1; Geodermatophilus africanus; counts= 2; multicellularity status: TRUE**
  - EAD1+Trypsin->EAD1+STAND_NTPase->STAND_NTPase+wHTH+TPRs+EAD1+Trypsin*->
  - EAD1+Trypsin*->EAD1+STAND_NTPase->STAND_NTPase+wHTH+TPRs+EAD1+Trypsin->
- **GCA_900116615.1; Algoriphagus locisalis; counts= 2; multicellularity status: FALSE**
  - GAP2->ZnR+DO-GTPase2->DO-GTPase1->GAP1-N1+GAP1-M+GAP1-C+EAD1*->EAD1+Trypsin->Endonuclease_NS(HNH)->
  - ZnR+DO-GTPase2->DO-GTPase1->GAP1-N1+GAP1-M+GAP1-C+EAD1->EAD1+Trypsin*->Endonuclease_NS(HNH)->
- **GCA_900113985.1; Phyllobacterium sp. CL33Tsu; counts= 2; multicellularity status: TRUE**
  - EAD1+Trypsin*->
  - SP+Ig-like+TM+EAD1*->
- **GCA_900111995.1; Collimonas sp. OK607; counts= 2; multicellularity status: FALSE**
  - ZnR+DO-GTPase2->DO-GTPase1->GAP1-N1+GAP1-M+GAP1-C+EAD1->CASPASE+EAD1*->
  - ZnR+DO-GTPase2->DO-GTPase1->GAP1-N1+GAP1-M+GAP1-C+EAD1*->CASPASE+EAD1->
- **GCF_900104725.1; Actinoplanes derwentensis; counts= 2; multicellularity status: TRUE**
  - EAD1+Trypsin*->EAD1+P-loop_NTPase(i)->MoxR->
  - Trypsin+EAD1*->BetaPropeller+vWA->
- **GCA_002234595.1; Amycolatopsis sp. H5; counts= 2; multicellularity status: TRUE**
  - EAD1+CASPASE->ZnF->E1(active)+E1_C_unk->EAD1+YukDl-Nterm+UB+E2*->JAB-N+JAB->?->CDC48-like->
  - EAD1+CASPASE*->ZnF->E1(active)+E1_C_unk->EAD1+YukDl-Nterm+UB+E2->

- **GCA_002318995.1; Shewanella algae; counts= 2; multicellularity status: FALSE**
  - GAP2->ZnR+DO-GTPase2->DO-GTPase1->GAP1-N1+GAP1-M+GAP1-C+EAD1*->
  - PNP_UDP_1+EAD1*->GAP2->ZnR+DO-GTPase2->DO-GTPase1->
- **GCA_002356095.1; Pseudomonas putida; counts= 2; multicellularity status: FALSE**
  - ZnR+DO-GTPase2->DO-GTPase1->GAP1-N1+GAP1-M+GAP1-C+EAD1->CASPASE+EAD1*->
  - ZnR+DO-GTPase2->DO-GTPase1->GAP1-N1+GAP1-M+GAP1-C+EAD1*->CASPASE+EAD1->
- **GCA_002368335.1; Nostoc sp. NIES-4103; counts= 2; multicellularity status: TRUE**
  - EAD1+CASPASE->EAD1+GUN4*->
  - EAD1+CASPASE*->EAD1+GUN4->
- **GCA_004296455.1; Calothrix sp. NIES-4101; counts= 2; multicellularity status: TRUE**
  - EAD1+Trypsin->TM+TM+TM->EAD1+VMAP-M1+VMAP-C*->
  - EAD1+Trypsin*->TM+TM+TM->EAD1+VMAP-M1+VMAP-C->
- **GCA_002368315.1; Fischerella sp. NIES-4106; counts= 2; multicellularity status: NA**
  - EAD1+Trypsin->TM+TM+TM->EAD1+??+VMAP-C*->MoxR->vWA+Beta-rich+FGS-fold_domain->
  - EAD1+Trypsin*->TM+TM+TM->EAD1+??+VMAP-C->MoxR->
- **GCA_900206255.1; Streptomyces sp. TLI_55; counts= 2; multicellularity status: TRUE**
  - EAD1+CASPASE->ZnF->E1(active)+E1_C_unk->EAD1+YukDl-Nterm+UB+E2*->
  - EAD1+CASPASE*->ZnF->E1(active)+E1_C_unk->EAD1+YukDl-Nterm+UB+E2->
- **GCA_002754245.1; Chryseobacterium sp. 52; counts= 2; multicellularity status: FALSE**
  - DO-GTPase1->GAP1-N1+GAP1-M+GAP1-C+EAD1->?->EAD1+Trypsin*->Endonuclease_NS(HNH)->
  - GAP2->ZnR+DO-GTPase2->DO-GTPase1->GAP1-N1+GAP1-M+GAP1-C+EAD1*->?->EAD1+Trypsin->Endonuclease_NS(HNH)->
- **GCF_002321945.1; Cylindrospermopsis raciborskii; counts= 2; multicellularity status: NA**
  - RHH->EAD1+Trypsin->TM+TM+TM->EAD1*->
  - RHH->EAD1+Trypsin*->TM+TM+TM->EAD1->
- **GCA_002795805.1; Mariprofundus aestuarium; counts= 2; multicellularity status: TRUE**
  - GAP2->ZnR+DO-GTPase2->DO-GTPase1->GAP1-N1+GAP1-M+GAP1-C+EAD1*->PNP_UDP_1+EAD1->
  - ZnR+DO-GTPase2->DO-GTPase1->GAP1-N1+GAP1-M+GAP1-C+EAD1->PNP_UDP_1+EAD1*->
- **GCA_002813455.1; Pseudomonas baetica; counts= 2; multicellularity status: NA**
  - EAD1+P-loop_helicase->CASPASE+EAD1*->
  - EAD1+P-loop_helicase*->CASPASE+EAD1->
- **GCA_000221965.1; Xanthomonas campestris pv. raphani 756C; counts= 1; multicellularity status: FALSE**
  - DO-GTPase1->GAP1-N1+GAP1-M+GAP1-C+EAD1*->
- **GCA_000418455.1; Streptomyces rapamycinicus NRRL 5491; counts= 1; multicellularity status: TRUE**

- EAD1+CASPASE*->

- **GCA_001618865.1; Luteitalea pratensis; counts= 1; multicellularity status: NA**
  - CASPASE->EAD1+Trypsin+TM*->
- **GCA_003595545.1; Streptomyces sp. 11-1-2; counts= 1; multicellularity status: TRUE**
  - EAD1+CASPASE*->
- **GCA_000158915.1; Streptomyces himastatinicus ATCC 53653; counts= 1; multicellularity status: TRUE**
  - EAD1+Trypsin*->
- **GCA_000243895.3; Leptospira interrogans str. FPW2026; counts= 1; multicellularity status: FALSE**
  - helical_region+EAD1->PNP_UDP_1+EAD1*->
- **GCA_000516475.1; Candidatus Magnetoglobus multicellularis str. Araruama; counts= 1; multicellularity status: TRUE**
  - EAD1+??*->
- **GCA_000828085.2; Scytonema tolypothrichoides VB-61278; counts= 1; multicellularity status: NA**
  - EAD1*->
- **GCA_000961745.1; Rhodospirillaceae bacterium BRH_c57; counts= 1; multicellularity status: FALSE**
  - GAP1-N1+GAP1-M+GAP1-C+EAD1*->
- **GCA_001303105.1; Anaerolineae bacterium SG8_19; counts= 1; multicellularity status: NA**
  - EAD1*->
- **GCA_001589205.1; Sorangium cellulosum; counts= 1; multicellularity status: TRUE**
  - EAD1+APATPase+wHTH+TPRs+BetaPropeller+EAD7*->||<-EAD7+CASPASE
- **GCA_001589295.1; Sorangium cellulosum; counts= 1; multicellularity status: TRUE**
  - EAD1+Trypsin*->?->MoxR->
- **GCA_001640045.1; Candidatus Thiomargarita nelsonii; counts= 1; multicellularity status: TRUE**
  - E1(active)+E1_C->EAD1*->
- **GCA_001672255.1; Anabaena sp. AL09; counts= 1; multicellularity status: TRUE**
  - EAD1+CASPASE*->
- **GCA_001772385.1; candidate division WOR-1 bacterium RIFOXYA2_FULL_36_21; counts= 1; multicellularity status: NA**
  - EAD1+EAD1*->
- **GCA_001898145.1; Dyadobacter sp. 50-39; counts= 1; multicellularity status: NA**
  - Trypsin+Endonuclease_NS(HNH)->?->Trypsin(i)+STAND_NTPase+wHTH+TPRs->EAD1+Trypsin*->
- **GCA_001874765.1; Acinetobacter baumannii; counts= 1; multicellularity status: FALSE**

- ZnR+DO-GTPase2->DO-GTPase1->?->??+EAD1*->

- **GCA_001983005.1; Frankia sp. BMG5.30; counts= 1; multicellularity status: TRUE**
  - TIR+EAD1*->
- **GCA_002200945.1; Mesorhizobium amorphae CCBAU 01583; counts= 1; multicellularity status: TRUE**
  - Endonuclease_NS(HNH)->EAD1*->Trypsin->EAD1->
- **GCA_002401655.1; Gammaproteobacteria bacterium; counts= 1; multicellularity status: NA**
  - GAP2->ZnR+DO-GTPase2->DO-GTPase1->GAP1-N1+GAP1-M+GAP1-C+EAD1*->
- **GCA_002858925.1; Caulobacter sp. 410; counts= 1; multicellularity status: NA**
  - EAD1+Trypsin*->
- **GCA_900101685.1; Alloactinosynnema iranicum; counts= 1; multicellularity status: TRUE**
  - EAD1+Trypsin*->STAND_NTPase+TPRs->
- **GCA_900106665.1; Paracoccus sanguinis; counts= 1; multicellularity status: NA**
  - EAD1+Trypsin*->
- **GCA_900169915.1; Acinetobacter baumannii; counts= 1; multicellularity status: FALSE**
  - ??+EAD1*->
- **GCA_000216135.3; Leptospira interrogans; counts= 1; multicellularity status: FALSE**
  - GAP2->ZnR+DO-GTPase2->DO-GTPase1->GAP1-N1+GAP1-M+GAP1-C+EAD1*->PNP_UDP_1+EAD1->
- **GCA_000306495.2; Leptospira interrogans; counts= 1; multicellularity status: FALSE**
  - GAP2->ZnR+DO-GTPase2->DO-GTPase1->GAP1-N1+GAP1-M+GAP1-C+EAD1*->PNP_UDP_1+EAD1->
- **GCA_000164015.3; Pseudomonas savastanoi; counts= 1; multicellularity status: FALSE**
  - ZnR+DO-GTPase2->DO-GTPase1->GAP1-N1+GAP1-M+GAP1-C+EAD1*->?->PSE->CASPASE+EAD1->
- **GCA_000280925.3; Myxococcus hansupus; counts= 1; multicellularity status: TRUE**
  - EAD1+Trypsin*->
- **GCA_002752655.1; Methylosinus; counts= 1; multicellularity status: TRUE**
  - EAD1+Trypsin*->?->STAND_NTPase+wHTH+TM+TPRs->
- **GCA_000368385.1; Acinetobacter sp. NIPH 899; counts= 1; multicellularity status: NA**
  - EAD1+??*->?->ZnR+DO-GTPase2->DO-GTPase1->
- **GCA_000369405.1; Acinetobacter; counts= 1; multicellularity status: FALSE**
  - EAD1+??*->?->ZnR+DO-GTPase2->DO-GTPase1->
- **GCA_000369745.1; Acinetobacter; counts= 1; multicellularity status: FALSE**

  - ZnR+DO-GTPase2->DO-GTPase1->GAP1-N1+GAP1-M+GAP1-C+EAD1*->

- **GCA_001686985.1; Mesorhizobium amorphae; counts= 1; multicellularity status: TRUE**
  - Trypsin+Endonuclease_NS(HNH)->EAD1+Trypsin*->
- **GCA_000350545.1; Desulfotignum phosphitoxidans; counts= 1; multicellularity status: FALSE**
  - ??+PNP_UDP_1+EAD1*->GAP2->ZnR+DO-GTPase2->DO-GTPase1->
- **GCA_000313875.1; Alcaligenes sp. EGD-AK7; counts= 1; multicellularity status: FALSE**
  - ZnR+DO-GTPase2->DO-GTPase1->GAP1-N1+GAP1-M+GAP1-C+EAD1->CASPASE+EAD1*->
- **GCF_000011365.1; Bradyrhizobium; counts= 1; multicellularity status: TRUE**
  - HNH+PNP_UDP_1+EAD1*->
- **GCA_000013345.1; Frankia; counts= 1; multicellularity status: TRUE**
  - EAD1+cNMP_cyclase+TIR*->
- **GCA_000012685.1; Myxococcus xanthus; counts= 1; multicellularity status: TRUE**
  - EAD1+Trypsin*->
- **GCA_000016405.1; Rhodobacter sphaeroides; counts= 1; multicellularity status: FALSE**
  - Trypsin->EAD1+Trypsin*->EAD1+HExxH+helical_region->TM+Endonuclease_NS(HNH)+LytR_C/SPOR->
- **GCA_000015165.1; Bradyrhizobium sp. BTAi1; counts= 1; multicellularity status: TRUE**
  - EAD1+Trypsin*->
- **GCA_000024805.1; Haliangium ochraceum; counts= 1; multicellularity status: TRUE**
  - ST_kinase+??+P-loop_NTPase+EAD1*->
- **GCA_000147335.1; Cyanothece sp. PCC 7822; counts= 1; multicellularity status: NA**
  - EAD1+TerB-like?+GUN4*->
- **GCA_000199675.1; Anaerolinea thermophila; counts= 1; multicellularity status: NA**
  - EAD1+Trypsin*->
- **GCA_000187705.1; Rahnella; counts= 1; multicellularity status: NA**
  - EAD1+??*->
- **GCA_000219105.1; Myxococcus fulvus; counts= 1; multicellularity status: TRUE**
  - EAD1+Trypsin*->
- **GCA_000147815.3; Streptomyces violaceusniger; counts= 1; multicellularity status: TRUE**
  - EAD1+CASPASE*->
- **GCA_000255295.1; Corallococcus coralloides; counts= 1; multicellularity status: NA**

  - EAD1+Trypsin*->
- **GCA_000807315.1; Bradyrhizobium japonicum; counts= 1; multicellularity status: TRUE**
  - Trypsin+EAD1+helical_region*->
- **GCA_000266905.1; Mycolicibacterium chubuense; counts= 1; multicellularity status: NA**
  - EAD1+Trypsin*->EAD1+STAND_NTPase->STAND_NTPase+wHTH+TPRs->
- **GCA_000331735.1; Myxococcus stipitatus; counts= 1; multicellularity status: TRUE**
  - EAD1+Trypsin*->
- **GCF_000427585.1; Sinorhizobium meliloti; counts= 1; multicellularity status: TRUE**
  - EAD1+Trypsin*->
- **GCA_000465875.3; Alcaligenes sp. EGD-AK7; counts= 1; multicellularity status: FALSE**
  - GAP2->ZnR+DO-GTPase2->DO-GTPase1->GAP1-N1+GAP1-M+GAP1-C+EAD1*->CASPASE+EAD1->
- **GCA_000503155.1; Mesorhizobium; counts= 1; multicellularity status: NA**
  - Trypsin+Endonuclease_NS(HNH)->EAD1+Trypsin*->
- **GCA_000502795.1; Mesorhizobium; counts= 1; multicellularity status: NA**
  - EAD1+Trypsin*->
- **GCA_000502775.1; Mesorhizobium sp. LSJC265A00; counts= 1; multicellularity status: TRUE**
  - Trypsin+Endonuclease_NS(HNH)->EAD1+Trypsin*->
- **GCA_000502755.1; Mesorhizobium sp. LSJC264A00; counts= 1; multicellularity status: TRUE**
  - Trypsin+Endonuclease_NS(HNH)->EAD1+Trypsin*->
- **GCA_000502695.1; Mesorhizobium; counts= 1; multicellularity status: NA**
  - Trypsin+Endonuclease_NS(HNH)->EAD1+Trypsin*->
- **GCA_000503075.1; Mesorhizobium sp. LSHC422A00; counts= 1; multicellularity status: TRUE**
  - EAD1+Trypsin+??+CarboxypepD-reg-IG+papain-like_peptidoglycan_hydrolase*->
- **GCA_000502615.1; Mesorhizobium sp. LNJC405B00; counts= 1; multicellularity status: TRUE**
  - Trypsin+Endonuclease_NS(HNH)->EAD1+Trypsin*->
- **GCA_000502995.1; Mesorhizobium; counts= 1; multicellularity status: NA**
  - Trypsin+Endonuclease_NS(HNH)->EAD1+Trypsin*->
- **GCA_000502555.1; Mesorhizobium; counts= 1; multicellularity status: NA**
  - Trypsin+Endonuclease_NS(HNH)->EAD1+Trypsin*->
- **GCA_000502475.1; Mesorhizobium; counts= 1; multicellularity status: NA**

- Trypsin+Endonuclease_NS(HNH)->EAD1+Trypsin*->

- **GCA_000502295.1; Mesorhizobium sp. L103C565B0; counts= 1; multicellularity status: TRUE**
  - Trypsin+Endonuclease_NS(HNH)->EAD1+Trypsin*->
- **GCA_000502255.1; Mesorhizobium sp. L103C120A0; counts= 1; multicellularity status: TRUE**
  - Trypsin+Endonuclease_NS(HNH)->EAD1+Trypsin*->
- **GCA_000502235.1; Mesorhizobium sp. L103C119B0; counts= 1; multicellularity status: TRUE**
  - Trypsin+Endonuclease_NS(HNH)->EAD1+Trypsin*->
- **GCF_000301755.1; Acinetobacter; counts= 1; multicellularity status: FALSE**
  - PNP_UDP_1+EAD1*->?->ZnR+DO-GTPase2->DO-GTPase1->
- **GCF_000428945.1; Hamadaea tsunoensis; counts= 1; multicellularity status: TRUE**
  - vWA->?->Pkinase->EAD1+Trypsin*->EAD1+STAND_NTPase->cNMP_cyclase+STAND_NTPase+wHTH->TM+TM+TM->
- **GCF_000521175.1; Aphanizomenon flos-aquae; counts= 1; multicellularity status: NA**
  - cNMP_cyclase+EAD1*->
- **GCA_004001485.1; Sinorhizobium meliloti; counts= 1; multicellularity status: TRUE**
  - GAP2->ZnR+DO-GTPase2->DO-GTPase1->GAP1-N1+GAP1-M+GAP1-C+EAD1*->CASPASE+EAD1->
- **GCF_000510665.1; Sinorhizobium meliloti; counts= 1; multicellularity status: TRUE**
  - ZnR+DO-GTPase2->DO-GTPase1->GAP1-N1+GAP1-M+GAP1-C+EAD1->CASPASE+EAD1*->
- **GCA_001560865.1; Pseudomonas; counts= 1; multicellularity status: FALSE**
  - ZnR+DO-GTPase2->DO-GTPase1->GAP1-N1+GAP1-M+GAP1-C+EAD1->CASPASE+EAD1*->
- **GCF_000717715.1; Streptomyces sp. NRRL B-24484; counts= 1; multicellularity status: TRUE**
  - EAD1+Trypsin->EAD1+STAND_NTPase*->
- **GCF_000760995.1; Rhodococcus fascians; counts= 1; multicellularity status: TRUE**
  - DO-GTPase1->GAP1-N1+GAP1-M+GAP1-C+EAD1*->PNP_UDP_1+EAD1->
- **GCA_000737695.1; Flavobacterium hydatis; counts= 1; multicellularity status: FALSE**
  - GAP2->ZnR+DO-GTPase2->DO-GTPase1->GAP1-N1+GAP1-M+GAP1-C+EAD1*->?->EAD1+Trypsin->Endonuclease_NS(HNH)->
- **GCA_000732115.1; Frankia sp. CeD; counts= 1; multicellularity status: NA**
  - EAD1+cNMP_cyclase+TIR*->
- **GCF_000316115.1; Leptolyngbya sp. PCC 7375; counts= 1; multicellularity status: TRUE**
  - EAD1+Trypsin->TM+TM+TM->EAD1+??+VMAP-C*->MoxR->vWA+BetaPropeller+FGS-fold_domain->FGS-fold_domain->
- **GCF_000720255.1; Streptomyces griseus; counts= 1; multicellularity status: TRUE**

- CASPASE->?->E1(active)+E1_C_unk->EAD1+YukDl-Nterm+UB+E2*->?->?->CDC48-like->

- **GCA_000738695.1; Streptomyces scabiei; counts= 1; multicellularity status: TRUE**
  - EAD1+TM+??+TM*->
- **GCA_000590515.1; Streptomyces sp. PRh5; counts= 1; multicellularity status: TRUE**
  - EAD1+CASPASE*->
- **GCA_000812465.1; Cupriavidus sp. IDO; counts= 1; multicellularity status: FALSE**
  - ZnR+DO-GTPase2->DO-GTPase1->GAP1-N1+GAP1-M+GAP1-C+EAD1*->
- **GCA_000789435.1; Aphanizomenon flos-aquae; counts= 1; multicellularity status: NA**
  - EAD1+Trypsin*->TM+TM+TM->EAD1+BetaPropeller->
- **GCF_000350545.1; Desulfotignum phosphitoxidans; counts= 1; multicellularity status: FALSE**
  - DO-GTPase1->?->?->GAP1-C+EAD1*->
- **GCF_000317495.1; Crinalium epipsammum; counts= 1; multicellularity status: NA**
  - EAD1*->?->EAD1+CASPASE->
- **GCA_000773865.1; Bradyrhizobium japonicum; counts= 1; multicellularity status: TRUE**
  - Trypsin+EAD1+helical_region*->
- **GCA_000826545.1; Kibdelosporangium sp. MJ126-NF4; counts= 1; multicellularity status: TRUE**
  - EAD1+Trypsin*->EAD1+STAND_NTPase->STAND_NTPase+wHTH+TPRs+CASPASE->
- **GCF_000344805.1; Bradyrhizobium oligotrophicum; counts= 1; multicellularity status: TRUE**
  - EAD1+Trypsin->EAD1+??*->STAND_NTPase+wHTH+TM+TPRs->
- **GCA_000834545.1; Pseudomonas fluorescens; counts= 1; multicellularity status: FALSE**
  - ZnR+DO-GTPase2->DO-GTPase1->GAP1-N1+GAP1-M+GAP1-C+EAD1->CASPASE+EAD1*->
- **GCA_000685765.2; Frankia sp. BMG5.23; counts= 1; multicellularity status: NA**
  - EAD1+cNMP_cyclase+TIR*->
- **GCA_000935305.1; Pseudomonas oryzihabitans; counts= 1; multicellularity status: FALSE**
  - ZnR+DO-GTPase2->DO-GTPase1->GAP1-N1+GAP1-M+GAP1-C+EAD1*->CASPASE+EAD1->
- **GCA_001990805.2; Mastigocladus laminosus; counts= 1; multicellularity status: NA**
  - EAD1+CASPASE*->
- **GCA_000938975.1; Streptomyces iranensis; counts= 1; multicellularity status: TRUE**
  - EAD1+CASPASE*->
- **GCA_000943515.2; Amycolatopsis orientalis; counts= 1; multicellularity status: TRUE**

- EAD1+Trypsin*->EAD1+STAND_NTPase->STAND_NTPase+wHTH+TPRs+CASPASE->

- **GCF_000818715.1; Vibrio jasicida; counts= 1; multicellularity status: FALSE**
  - ZnR+DO-GTPase2->DO-GTPase1->GAP1-N1+GAP1-M+GAP1-C+EAD1*->
- **GCA_000972385.1; Streptomyces sp. WM6386; counts= 1; multicellularity status: TRUE**
  - EAD1+CASPASE->ZnF->E1(active)+E1_C_unk->EAD1+YukDl-Nterm+UB+E2*->JAB-N+JAB->?->CDC48-like->
- **GCF_000981895.1; Streptomyces odonnellii; counts= 1; multicellularity status: TRUE**
  - EAD1+Pkinase*->
- **GCF_000985995.1; Neorhizobium galegae; counts= 1; multicellularity status: FALSE**
  - EAD1+Trypsin*->
- **GCA_000988565.1; Myxococcus fulvus; counts= 1; multicellularity status: TRUE**
  - EAD1+Trypsin*->
- **GCF_001182915.1; Kitasatospora sp. MY 5-36; counts= 1; multicellularity status: NA**
  - EAD1+Trypsin*->||<-?||STAND_NTPase->STAND_NTPase+wHTH+TPRs+AB_hydrolase->
- **GCF_000472985.1; Bradyrhizobium japonicum; counts= 1; multicellularity status: TRUE**
  - Trypsin+EAD1+helical_region*->
- **GCF_000739635.1; Verrucomicrobium sp. BvORR034; counts= 1; multicellularity status: FALSE**
  - VMAP-C->MoxR->vWA+sGTP_APGTPase+COR+EAD1+Trypsin*->
- **GCF_000158915.1; Streptomyces himastatinicus; counts= 1; multicellularity status: TRUE**
  - EAD1+Trypsin*->
- **GCF_000261805.1; Bradyrhizobium yuanmingense; counts= 1; multicellularity status: TRUE**
  - EAD1+Trypsin*->||<-EAD5+Trypsin<-STAND_NTPase+wHTH+TPRs
- **GCA_002217335.1; Flavobacterium hydatis; counts= 1; multicellularity status: FALSE**
  - DO-GTPase1->GAP1-N1+GAP1-M+GAP1-C+EAD1->?->EAD1+Trypsin*->Endonuclease_NS(HNH)->
- **GCF_000763825.1; Paracoccus sanguinis; counts= 1; multicellularity status: NA**
  - EAD1+Trypsin*->
- **GCA_003387095.1; Archangium gephyra; counts= 1; multicellularity status: TRUE**
  - EAD1+TIR*->
- **GCF_001043915.1; Methylobacterium aquaticum; counts= 1; multicellularity status: TRUE**
  - EAD1+Trypsin+??*->TM+Endonuclease_NS(HNH)->
- **GCA_001277295.1; Anabaena sp. WA102; counts= 1; multicellularity status: TRUE**

- EAD1+Trypsin->TM+TM+TM->EAD1+TPRs*->

- **GCA_001280015.1; Streptomyces sp. MMG1121; counts= 1; multicellularity status: TRUE**
  - EAD1+CASPASE->ZnF->E1(active)+E1_C_unk->EAD1+YukDl-Nterm+UB+E2*->JAB-N+JAB->?->CDC48-like->
- **GCF_001421075.1; Frankia sp. CpI1-P; counts= 1; multicellularity status: TRUE**
  - EAD2+EAD1*->
- **GCA_001422775.1; Methylobacterium sp. Leaf87; counts= 1; multicellularity status: TRUE**
  - GAP1-N1+GAP1-M+GAP1-C+EAD1*->CASPASE+EAD1->
- **GCA_001424165.1; Massilia sp. Leaf139; counts= 1; multicellularity status: NA**
  - EAD1+Trypsin->EAD1+P-loop_NTPase*->STAND_NTPase+wHTH+TPRs->
- **GCA_001422925.1; Microbacterium sp. Leaf288; counts= 1; multicellularity status: NA**
  - EAD1+Trypsin*->?->STAND_NTPase+wHTH+TPRs->
- **GCA_001426195.1; Sphingomonas sp. Leaf339; counts= 1; multicellularity status: FALSE**
  - Trypsin+Endonuclease_NS(HNH)->EAD1+Trypsin*->?->STAND_NTPase+TM+Endonuclease_NS(HNH)->
- **GCA_001428505.1; Terrabacter; counts= 1; multicellularity status: NA**
  - EAD1+Trypsin->EAD1+STAND_NTPase*->STAND_NTPase+wHTH+TPRs->
- **GCA_001429185.1; Terrabacter; counts= 1; multicellularity status: NA**
  - EAD1+Trypsin*->EAD1+STAND_NTPase->STAND_NTPase+wHTH+TPRs->
- **GCA_001401405.1; Pseudomonas syringae group genomosp. 3; counts= 1; multicellularity status: FALSE**
  - ZnR+DO-GTPase2->DO-GTPase1->GAP1-N1+GAP1-M+GAP1-C+EAD1*->CASPASE+EAD1->
- **GCA_001485435.1; Deinococcus grandis; counts= 1; multicellularity status: NA**
  - EAD1*->
- **GCA_001509775.1; Streptomyces violaceusniger; counts= 1; multicellularity status: TRUE**
  - EAD1+CASPASE*->
- **GCA_001533325.1; Burkholderia ubonensis; counts= 1; multicellularity status: FALSE**
  - ZnR+DO-GTPase2->DO-GTPase1->GAP1-N1+GAP1-M+GAP1-C+EAD1*->CASPASE+EAD1->
- **GCF_001547895.1; Pseudomonas sp. Os17; counts= 1; multicellularity status: NA**
  - GAP1-N1+GAP1-M+GAP1-C+EAD1*->CASPASE+EAD1->
- **GCA_001547895.1; Pseudomonas sp. Os17; counts= 1; multicellularity status: NA**
  - GAP1-N1+GAP1-M+GAP1-C+EAD1->CASPASE+EAD1*->
- **GCF_001586755.1; Cylindrospermopsis raciborskii; counts= 1; multicellularity status: NA**

  - RHH->EAD1+Trypsin*->
- **GCA_001548455.1; Fischerella sp. NIES-3754; counts= 1; multicellularity status: NA**
  - EAD1+CASPASE->EAD1+TPRs*->
- **GCA_001625455.1; Pseudomonas fluorescens; counts= 1; multicellularity status: FALSE**
  - PNP_UDP_1+EAD1*->GAP2->ZnR+DO-GTPase2->
- **GCA_001636575.1; Frankia casuarinae; counts= 1; multicellularity status: TRUE**
  - EAD1+cNMP_cyclase+TIR*->
- **GCF_001641335.1; Bradyrhizobium stylosanthis; counts= 1; multicellularity status: TRUE**
  - Trypsin+EAD1+helical_region*->
- **GCF_001650215.1; Streptomyces sp. RTd22; counts= 1; multicellularity status: TRUE**
  - EAD1+Trypsin*->VMAP-M0+VMAP-C->MoxR->
- **GCA_001693485.1; Bradyrhizobium sp. LMTR 3; counts= 1; multicellularity status: TRUE**
  - Trypsin+EAD1+helical_region*->
- **GCF_001636565.1; Frankia sp. EI5c; counts= 1; multicellularity status: TRUE**
  - pntlT4_fusion_domain+EAD1*->
- **GCA_001651875.1; Sinorhizobium saheli; counts= 1; multicellularity status: TRUE**
  - GAP1-N1+GAP1-M+GAP1-C+EAD1->CASPASE+EAD1*->
- **GCA_001672445.1; Mesorhizobium sp. WSM3873; counts= 1; multicellularity status: TRUE**
  - EAD1+Trypsin*->?->STAND_NTPase+wHTH+TPRs->
- **GCA_001514035.1; Streptomyces yokosukanensis; counts= 1; multicellularity status: TRUE**
  - EAD1+CASPASE->ZnF->E1(active)+E1_C_unk->EAD1+YukDl-Nterm+UB+E2*->JAB-N+JAB->?->CDC48-like->
- **GCA_001676615.1; Labrys sp. WJW; counts= 1; multicellularity status: NA**
  - EAD1+Trypsin*->
- **GCF_001624695.1; Labrenzia sp. OB1; counts= 1; multicellularity status: FALSE**
  - EAD1+Trypsin*->
- **GCA_001650175.1; Opitutaceae bacterium TSB47; counts= 1; multicellularity status: NA**
  - CASPASE+EAD1*->
- **GCF_001718095.1; Vibrio cholerae; counts= 1; multicellularity status: FALSE**
  - PNP_UDP_1+EAD1*->GAP2->ZnR+DO-GTPase2->DO-GTPase1->
- **GCA_001727475.1; Vibrio parahaemolyticus; counts= 1; multicellularity status: FALSE**

- GAP2->ZnR+DO-GTPase2->DO-GTPase1->GAP1-N1+GAP1-M+GAP1-C+EAD1*->

- **GCA_001735805.1; Streptomyces puniciscabiei; counts= 1; multicellularity status: TRUE**
  - *
- **GCF_001748165.1; Streptomyces sp. SPMA113; counts= 1; multicellularity status: TRUE**
  - EAD1+CASPASE*->
- **GCA_001756285.1; Frankia sp. CgIM4; counts= 1; multicellularity status: NA**
  - EAD1+cNMP_cyclase+TIR*->
- **GCA_001758345.1; Acinetobacter towneri; counts= 1; multicellularity status: NA**
  - EAD1+??*->?->ZnR+DO-GTPase2->DO-GTPase1->
- **GCA_001866675.1; Streptomyces sp. MUSC 14; counts= 1; multicellularity status: TRUE**
  - ZnF->E1(active)+E1_C_unk->EAD1+YukDl-Nterm+UB+E2*->JAB-N+JAB->?->CDC48-like->
- **GCA_001866665.1; Streptomyces sp. MUSC 1; counts= 1; multicellularity status: TRUE**
  - EAD1+CASPASE->ZnF->E1(active)+E1_C_unk->EAD1+YukDl-Nterm+UB+E2*->JAB-N+JAB->?->CDC48-like->
- **GCA_001904645.1; Fischerella major; counts= 1; multicellularity status: NA**
  - EAD1+CASPASE->EAD1+TPRs*->
- **GCA_001904675.1; Scytonema sp. HK-05; counts= 1; multicellularity status: TRUE**
  - TCAD3+TCAD4+CASPASE+EAD1*->EAD1+VMAP-M1+VMAP-C->MoxR->vWA+Beta-rich+HTH+FGS-fold_domain->
- **GCA_900111765.1; Myxococcus fulvus; counts= 1; multicellularity status: TRUE**
  - EAD1+Trypsin*->
- **GCA_001942465.1; Actinomadura sp. CNU-125; counts= 1; multicellularity status: TRUE**
  - ZnR+DO-GTPase2->DO-GTPase1->GAP1-N1+GAP1-M+GAP1-C+EAD1*->CASPASE+EAD1->
- **GCF_001942465.1; Actinomadura sp. CNU-125; counts= 1; multicellularity status: TRUE**
  - ZnR+DO-GTPase2->DO-GTPase1->GAP1-N1+GAP1-M+GAP1-C+EAD1->CASPASE+EAD1*->
- **GCA_001983015.1; Frankia sp. CcI156; counts= 1; multicellularity status: NA**
  - EAD1+cNMP_cyclase+TIR*->
- **GCA_002007105.1; Hymenobacter sp. CRA2; counts= 1; multicellularity status: FALSE**
  - ZnR+DO-GTPase2->?->?->GAP1-N1+GAP1-M+GAP1-C+PNP_UDP_1+EAD1*->
- **GCA_002021875.1; Streptomyces hygroscopicus; counts= 1; multicellularity status: TRUE**
  - EAD1+CASPASE*->
- **GCF_000725495.1; Streptomyces aureus; counts= 1; multicellularity status: TRUE**

- EAD1+Trypsin*->

- **GCA_001983975.1; Streptomyces autolyticus; counts= 1; multicellularity status: TRUE**
  - EAD1+CASPASE*->
- **GCA_002326205.1; Pseudomonas; counts= 1; multicellularity status: FALSE**
  - ZnR+DO-GTPase2->DO-GTPase1->GAP1-N1+GAP1-M+GAP1-C+EAD1*->CASPASE+EAD1->
- **GCF_002071875.1; Burkholderia cenocepacia; counts= 1; multicellularity status: FALSE**
  - ZnR+DO-GTPase2->DO-GTPase1->GAP1-N1+GAP1-M+GAP1-C+EAD1->CASPASE+EAD1*->
- **GCA_002071905.1; Burkholderia cenocepacia; counts= 1; multicellularity status: FALSE**
  - ZnR+DO-GTPase2->DO-GTPase1->GAP1-N1+GAP1-M+GAP1-C+EAD1*->CASPASE+EAD1->
- **GCF_001533325.1; Burkholderia ubonensis; counts= 1; multicellularity status: FALSE**
  - ZnR+DO-GTPase2->DO-GTPase1->GAP1-N1+GAP1-M+GAP1-C+EAD1->CASPASE+EAD1*->
- **GCF_000067165.1; Sorangium cellulosum; counts= 1; multicellularity status: TRUE**
  - STAND_NTPase+wHTH+TPRs+BetaPropeller+EAD1->EAD1+Trypsin*->
- **GCF_000834545.1; Pseudomonas fluorescens; counts= 1; multicellularity status: FALSE**
  - ZnR+DO-GTPase2->DO-GTPase1->GAP1-N1+GAP1-M+GAP1-C+EAD1*->CASPASE+EAD1->
- **GCF_001655155.1; Mycobacteroides immunogenum; counts= 1; multicellularity status: NA**
  - PNP_UDP_1+EAD1*->
- **GCF_001277295.1; Anabaena sp. WA102; counts= 1; multicellularity status: TRUE**
  - EAD1+Trypsin*->TM+TM+TM->EAD1+TPRs->
- **GCF_001589285.1; Sorangium cellulosum; counts= 1; multicellularity status: TRUE**
  - EAD1+APATPase+wHTH+TPRs+BetaPropeller+EAD7*->||<-EAD7+CASPASE
- **GCF_000789435.1; Aphanizomenon flos-aquae; counts= 1; multicellularity status: NA**
  - EAD1+Trypsin->TM+TM+TM->EAD1+BetaPropeller*->
- **GCF_000935305.1; Pseudomonas oryzihabitans; counts= 1; multicellularity status: FALSE**
  - ZnR+DO-GTPase2->DO-GTPase1->GAP1-N1+GAP1-M+GAP1-C+EAD1->CASPASE+EAD1*->
- **GCA_001296055.1; Novosphingobium sp. AAP93; counts= 1; multicellularity status: FALSE**
  - EAD1+Trypsin*->
- **GCF_001422775.1; Methylobacterium sp. Leaf87; counts= 1; multicellularity status: TRUE**
  - GAP1-N1+GAP1-M+GAP1-C+EAD1->CASPASE+EAD1*->
- **GCF_001424165.1; Massilia sp. Leaf139; counts= 1; multicellularity status: NA**

- EAD1+Trypsin*->EAD1+P-loop_NTPase->STAND_NTPase+wHTH+TPRs->

- **GCF_001428505.1; Terrabacter sp. Root181; counts= 1; multicellularity status: TRUE**
  - EAD1+Trypsin*->EAD1+STAND_NTPase->STAND_NTPase+wHTH+TPRs->
- **GCF_001429645.1; Terrabacter sp. Soil810; counts= 1; multicellularity status: NA**
  - EAD1+Trypsin*->EAD1+STAND_NTPase->STAND_NTPase+wHTH+TPRs->
- **GCF_001469165.1; Thiohalocapsa sp. ML1; counts= 1; multicellularity status: TRUE**
  - ZnF->E1(active)+E1_C_unk->TIR->EAD1+YukDl-Nterm+UB+E2*->
- **GCF_001548455.1; Fischerella sp. NIES-3754; counts= 1; multicellularity status: NA**
  - EAD1+CASPASE*->EAD1+TPRs->
- **GCF_001655615.1; Pseudomonas sp. GR 6-02; counts= 1; multicellularity status: NA**
  - EAD1+Trypsin*->?->STAND_NTPase+wHTH+TPRs->
- **GCF_001767235.1; Moorea producens; counts= 1; multicellularity status: TRUE**
  - EAD1+VMAP-M1+FGS-fold_domain->EAD1->VMAP-M1+FGS-fold_domain->EAD1+VMAP-M1+FGS-fold_domain*->
- **GCF_001854205.1; Moorea producens; counts= 1; multicellularity status: TRUE**
  - EAD1+CASPASE+FGS-fold_domain*->
- **GCF_001854805.1; Frankia sp. BMG5.36; counts= 1; multicellularity status: NA**
  - EAD1+APATPase+wHTH+TPRs*->
- **GCF_900117425.1; Novosphingobium sp. NDB2Meth1; counts= 1; multicellularity status: FALSE**
  - EAD1+Trypsin*->
- **GCF_001904745.1; Calothrix sp. HK-06; counts= 1; multicellularity status: NA**
  - CASPASE+EAD1*->EAD1->
- **GCF_000022085.1; Methylobacterium nodulans; counts= 1; multicellularity status: TRUE**
  - <-Trypsin+Endonuclease_NS(HNH)||EAD1+Trypsin*->
- **GCF_000379525.1; Lamprocystis purpurea; counts= 1; multicellularity status: TRUE**
  - EAD1+Trypsin*->EAD1+STAND_NTPase->STAND_NTPase+wHTH+TPRs->
- **GCF_001050195.2; Anaerolinea thermolimosa; counts= 1; multicellularity status: NA**
  - EAD1+Trypsin*->
- **GCF_001651875.1; Sinorhizobium saheli; counts= 1; multicellularity status: TRUE**
  - GAP1-N1+GAP1-M+GAP1-C+EAD1*->CASPASE+EAD1->
- **GCF_001904645.1; Fischerella major; counts= 1; multicellularity status: NA**

  - EAD1+CASPASE*->EAD1+TPRs->
- **GCF_002091475.1; Sphingomonas azotifigens; counts= 1; multicellularity status: FALSE**
  - EAD1+Trypsin*->
- **GCF_000503075.1; Mesorhizobium sp. LSHC422A00; counts= 1; multicellularity status: TRUE**
  - EAD1+Trypsin+??+TM+TM+TM+low_complexity*->
- **GCF_000503035.1; Mesorhizobium sp. LSHC412B00; counts= 1; multicellularity status: TRUE**
  - EAD1+Trypsin+??+CarboxypepD-reg-IG+papain-like_peptidoglycan_hydrolase*->
- **GCA_002099355.1; Frankia casuarinae; counts= 1; multicellularity status: TRUE**
  - EAD1+cNMP_cyclase+TIR*->
- **GCA_002099325.1; Frankia sp. KB5; counts= 1; multicellularity status: NA**
  - EAD1+cNMP_cyclase+TIR*->
- **GCF_001017755.1; Frankia coriariae; counts= 1; multicellularity status: TRUE**
  - TIR+EAD1*->
- **GCA_002143185.1; Acinetobacter baumannii; counts= 1; multicellularity status: FALSE**
  - ZnR+DO-GTPase2->DO-GTPase1->GAP1-N1+GAP1-M+GAP1-C+EAD1*->
- **GCF_002154275.1; Streptomyces castelarensis; counts= 1; multicellularity status: TRUE**
  - EAD1+CASPASE*->
- **GCF_002155885.1; Streptomyces rhizosphaericus; counts= 1; multicellularity status: TRUE**
  - EAD1+CASPASE*->
- **GCF_002157355.1; Variovorax sp. JS1663; counts= 1; multicellularity status: NA**
  - EAD1+Trypsin*->EAD1+STAND_NTPase->STAND_NTPase+wHTH+TPRs->
- **GCA_002163975.1; Nostocales cyanobacterium HT-58-2; counts= 1; multicellularity status: NA**
  - MoxR->helical_region+vWA->?->TIR+EAD1*->
- **GCF_002163975.1; Nostocales cyanobacterium HT-58-2; counts= 1; multicellularity status: NA**
  - EAD1+P-loop_NTPase(i)*->MoxR->helical_region+vWA->
- **GCA_002212765.2; Haliangium sp. UPWRP_2; counts= 1; multicellularity status: TRUE**
  - EAD7+EAD1+Trypsin*->
- **GCA_900188005.1; Actinoplanes regularis; counts= 1; multicellularity status: TRUE**
  - vWA->?->?->EAD1+Trypsin*->
- **GCA_900188295.1; Paracoccus sediminis; counts= 1; multicellularity status: NA**

- EAD1+Trypsin->EAD1+STAND_NTPase*->STAND_NTPase+wHTH+TM+TM->

- **GCA_004310385.1; Paracoccus sediminis; counts= 1; multicellularity status: NA**
  - EAD1+Trypsin*->EAD1+STAND_NTPase->STAND_NTPase+wHTH+TM+TM->
- **GCA_900101905.1; Myxococcus virescens; counts= 1; multicellularity status: TRUE**
  - EAD1+Trypsin*->
- **GCF_900111115.1; Giesbergeria anulus; counts= 1; multicellularity status: FALSE**
  - ZnR+DO-GTPase2->DO-GTPase1->GAP1-N1+GAP1-M+GAP1-C+EAD1*->CASPASE+EAD1->
- **GCA_900104165.1; Phyllobacterium sp. YR620; counts= 1; multicellularity status: TRUE**
  - EAD1+Trypsin*->
- **GCF_900112185.1; Bosea sp. DSM 18164; counts= 1; multicellularity status: TRUE**
  - EAD1+Trypsin*->
- **GCA_900095025.1; Acinetobacter albensis; counts= 1; multicellularity status: NA**
  - EAD1+??*->?->ZnR+DO-GTPase2->DO-GTPase1->
- **GCA_900102195.1; Pseudonocardia oroxyli; counts= 1; multicellularity status: NA**
  - pntlT4_fusion_domain+EAD1+Trypsin*->
- **GCF_900100605.1; Sinosporangium album; counts= 1; multicellularity status: TRUE**
  - EAD1+CASPASE*->E1(active)+E1_C_unk->EAD1+YukDl-Nterm+UB+E2->JAB-N+JAB->
- **GCA_900100605.1; Sinosporangium album; counts= 1; multicellularity status: TRUE**
  - EAD1+CASPASE->E1(active)+E1_C_unk->EAD1+YukDl-Nterm+UB+E2*->JAB-N+JAB->?->CDC48-like->
- **GCA_900099985.1; Thermostaphylospora chromogena; counts= 1; multicellularity status: TRUE**
  - EAD1+CASPASE*->
- **GCA_900105695.1; Streptomyces melanosporofaciens; counts= 1; multicellularity status: TRUE**
  - *
- **GCF_900187555.1; Pseudomonas sp. Irchel s3f19; counts= 1; multicellularity status: NA**
  - PNP_UDP_1+EAD1*->GAP2->
- **GCA_002283555.1; Rubrivirga sp. SAORIC476; counts= 1; multicellularity status: NA**
  - EAD1+Trypsin(frag)*->
- **GCA_002300355.1; Streptomyces sp. TLI_235; counts= 1; multicellularity status: NA**
  - EAD1+Trypsin->EAD1+STAND_NTPase*->STAND_NTPase+wHTH+TPRs->
- **GCF_002300355.1; Streptomyces sp. TLI_235; counts= 1; multicellularity status: NA**

- EAD1+Trypsin*->EAD1+STAND_NTPase->STAND_NTPase+wHTH+TPRs->

- **GCA_002303785.1; Rahnella victoriana; counts= 1; multicellularity status: NA**
  - EAD1+??*->
- **GCA_002305895.1; Myxococcus macrosporus; counts= 1; multicellularity status: TRUE**
  - EAD1+Trypsin*->
- **GCF_900112715.1; Nannocystis exedens; counts= 1; multicellularity status: TRUE**
  - EAD1+??*->
- **GCA_002368415.1; Calothrix sp. NIES-4105; counts= 1; multicellularity status: NA**
  - LRR_region+sGTP_APGTPase+COR+??+EAD1*->
- **GCF_002368455.1; Calothrix sp. NIES-4071; counts= 1; multicellularity status: TRUE**
  - LRR_region+sGTP_APGTPase+COR+??+EAD1*->
- **GCF_002407205.1; Rhodobacter sp. CZR27; counts= 1; multicellularity status: FALSE**
  - Trypsin->EAD1+Trypsin*->EAD1+HExxH+helical_region->TM+Endonuclease_NS(HNH)+LytR_C/SPOR->
- **GCA_900215205.1; Actinoplanes atraurantiacus; counts= 1; multicellularity status: TRUE**
  - Trypsin->EAD1+Trypsin+STAND_NTPase->EAD1+STAND_NTPase+TPRs*->
- **GCA_002591335.1; Streptomyces malaysiensis; counts= 1; multicellularity status: TRUE**
  - EAD1+CASPASE*->
- **GCA_002631205.1; Lewinella marina; counts= 1; multicellularity status: NA**
  - EAD1+Trypsin*->
- **GCA_002812405.1; Streptomyces sp. M56; counts= 1; multicellularity status: TRUE**
  - EAD1+CASPASE*->
- **GCA_002831565.1; Spirosoma sp. HA7; counts= 1; multicellularity status: NA**
  - EAD1+Trypsin*->EAD1+SIR2->STAND_NTPase+wHTH+TM+BetaPropeller->
- **GCF_002844445.1; Rhodobacter azotoformans; counts= 1; multicellularity status: FALSE**
  - Trypsin->EAD1+Trypsin*->EAD1+HExxH+helical_region->TM+Endonuclease_NS(HNH)+LytR_C/SPOR->

VII. Source data - organism abbreviations
VIII. Source data - multiple sequence alignments

================================
1. Organism abbreviations for multiple sequence alignments
2. vWA alignment
3. MoxR alignment
4. VMAP-C alignment
5. VMAP-M0-20 alignments
6. EAD1,2,4,5,6,7,8,9,10 alignments
7. Trypco1 and Trypco2 alignments
8. TCAD1,2,3,7,9 alignments
9. NEFF alignment
10. DO-GTPase1,2 alignments
11. GAAD alignment
12. iSTAND alignment
13. iSvmX1 alignment
14. iSvmX2 alignment
15. ivWA-helicalNterm alignment
16. ivWA alignment
17. EAD11 alignment
18. bpX0,1,2,3,4,5,6 alignments
19. bpMoxR alignment
20. fvmX1,2,3,3-analog,5,6,7 alignments
21. fvmYukDl-Nterm alignment
22. fvmRadSAM-pep(FtsH-associated peptide type) alignment
23. fvmNEFF1-Nterm alignment
24. fvmJAB-N alignment
25. FtsH systems MoxR alignment
26. HAD (effector in vWA systems) alignment
27. EACC1 alignment
28. GAP1-N1,GAP1-N2,GAP1-M,GAP1-C alignments

##
1. Organism abbreviations for multiple sequence alignments
##

Figure 1D (VMAP-C) alignment abbreviations:

Auta : Actinoplanes utahensis; Achi : Actinomadura chibensis; Cysp : Cyanothece sp; Ansp : Anabaena sp; Acsp : Actinoplanes sp; Otri : Oscillochloris trichoides; Gesp : Gemmata sp; Vesp : Verrucomicrobium sp; Nfla : Nocardiopsis flavescens; Nosp : Nocardiopsis sp; Ssca : Streptomyces scabrisporus; Kpho : Kitasatospora phosalacinea; Mpro : Moorea producens; Lpur : Lamprocystis purpurea; Mtes : Mastigocoleus testarum; Psul : Pseudarthrobacter sulfonivorans; Saur : Streptomyces aureus; Bcep : Burkholderia cepacia; Jasp : Janthinobacterium sp; Rrub : Rugamonas rubra; Athi : Azospirillum thiophilum; Aori : Amycolatopsis orientalis; Acsp : Actinomadura sp; Lesp : Leptolyngbya sp; Aglo : Actinoplanes globisporus; Pssp : Pseudomonas sp; Casp : Calothrix sp; Bara : Bradyrhizobium arachidis; Maer : Microcystis aeruginosa; Tery : Trichodesmium erythraeum; Ppri : Phormidesmis priestleyi; Aflo : Aphanizomenon flos-aquae;

Figure 8D (bacterial DEATH domains) alignment abbreviations:

Fmus : Fischerella muscicola; Tery : Trichodesmium erythraeum; Ppau : Planktothrix paucivesiculata; Shof : Scytonema hofmannii; Ansp : Anabaena sp; Mlam : Mastigocladus laminosus; Nosp : Nostoc sp; Ocya : Oscillatoriales cyanobacterium; Mesp : Methanothrix sp; Abac : Anaerolineales bacterium; Psol : Pseudomonas soli; Bpax : Bradyrhizobium paxllaeri; Mesp : Mesorhizobium sp; Hosp : Hoeflea sp; Pchl : Pseudomonas chlororaphis; Lasp : Labrenzia sp; Azsp : Azospirillum sp; Hasp : Hansschlegelia sp;

##
2. vWA alignment
##

Part 1
CATALYTIC ASPARTATE POSITIONS (marked with '*')

[illegible]

```
AGI87916.1        ----ELVARL----------RDAGLDV-------------------DAEHLLDALWLAAR- [illegible]
KIF79231.1        --MPALVARL----------REAGLDP-------------------DAAQLRDALWLARQ- [illegible]
WP_059012194.1    ----TLIRAL---------R-GAGLDP-------------------DWAELADALWLAQF- [illegible]
WP_020277090.1    ----ALVARM----------RDAGIGP-------------------GVEELADALWLARW- [illegible]
WP_079178870.1    ---------M----------RESGLDP-------------------DPEGLCDVLWLAQY- [illegible]
WP_081552946.1    ------------------MR-DVGLDP-------------------DVPQLCDALWLARW- [illegible]
ESP99762.1        ----ELVARL----------RGAGLDV-------------------DAEHLLDALWLAAR- [illegible]
ESQ05820.1        ----ELVARL----------RGAGLDV-------------------DAEHLLDALWLAAR- [illegible]
WP_051715921.1    ----ALVARM----------RDAGIGP-------------------SVEELADALWLAHW- [illegible]
WP_052687086.1    -PLGELVGRL----------TAAGVPA-------------------DARGMADALWLAQW- [illegible]
WP_027753922.1    --------------------------MSP-----------------DARGLADVLWLAEE- [illegible]
WP_051674211.1    ----ELVGRL---------N-GAGLEP-------------------GTGELADALWLARW- [illegible]
WP_053922005.1    -PLGELVGRL----------TAAGVPA-------------------DARGMADALWLAQW- [illegible]
WP_071376575.1    --------------------------MDP-----------------DAEELSDALWLARW- [illegible]
WP_071384258.1    --------------------------MDP-----------------DAEELSDALWLARW- [illegible]
WP_079125114.1    -LLTALVARL----------REAGLDP-------------------DAEQLCDAIWLAQQ- [illegible]
WP_037620923.1    ----ELIARI----------RQTGLDP-------------------DARQLADALWLARW- [illegible]
WP_051700487.1    ----ELAALL----------RAAGLDP-------------------SAEELADALWLAGH- [illegible]
WP_052030314.1    ----ELAALL----------RAAGLDP-------------------SAEELADALWLAGH- [illegible]
WP_053080709.1    ----DLAGLL----------RTAGLDP-------------------TAEELADALWLAGR- [illegible]
WP_079193740.1    ----EIVARL----------RAAGLPA-------------------DARAMADALWLARW- [illegible]
WP_079406085.1    ----ELAALL----------RAVGLDP-------------------SAEEIADALWLAGH- [illegible]
AIV38673.1        ----RLVLRL----------REIGMDP-------------------DAEQLCDALWLARW- [illegible]
WP_030017200.1    ----EIVARL----------RAAGLPA-------------------DARSMADALWLARW- [illegible]
WP_030182699.1    ----EIVARL----------RAAGLPA-------------------DARALADALWLARW- [illegible]
WP_030375332.1    ----EIVARL----------RAAGLPA-------------------DARALADALWLARW- [illegible]
WP_030604157.1    ----EIVARL----------RAAGLPA-------------------DARALADALWLARW- [illegible]
WP_030628104.1    ----EIVARL----------RAAGLPA-------------------DARALADALWLARW- [illegible]
WP_030654968.1    ----EIVARL----------RAAGLPA-------------------DARALADALWLARW- [illegible]
WP_030672371.1    ----EIVARL----------RAAGLPA-------------------DARALADALWLARW- [illegible]
WP_043978690.1    ----EIVARL----------RAAGLPA-------------------DARALADALWLARW- [illegible]
WP_050509157.1    ----EIVARL----------RAAGLPA-------------------DARALADALWLARW- [illegible]
WP_051731479.1    ----EIVARL----------RAAGLPA-------------------DARALADALWLARW- [illegible]
WP_051738500.1    ----EIVARL----------RAAGLPA-------------------DARALADALWLARW- [illegible]
WP_053800883.1    ----EIVARL----------RAAGLPA-------------------DARALADALWLARW- [illegible]
WP_057584733.1    -----MVARL----------RGVGLDP-------------------DVEQLCDALWLARW- [illegible]
WP_060733247.1    ----EIVARL----------RAAGLPA-------------------DARALADALWLARW- [illegible]
WP_081219385.1    ----ELVARM----------RQIGLDP-------------------DAGQLADALWLARW- [illegible]
KND32413.1        ----ELITRL----------RAAGLDP-------------------DQRQLSDALWLARF- [illegible]
WP_052848444.1    -------MGVL---------RDARLDP-------------------DWVGVADALWLAQY- [illegible]
WP_079250460.1    ----ELAALL----------RAAGLDP-------------------SAEELADALWLAGH- [illegible]
WP_031044453.1    ----RLVLRL----------REIGLDP-------------------DAEQLCDALWLARW- [illegible]
WP_060176762.1    ----ELAALL----------RAAGLDP-------------------SAEELADALWLAGH- [illegible]
WP_078095066.1    ----ELAALL----------RAAGLDP-------------------SAEELADALWLAGH- [illegible]
KOG57815.1        -----MASLL----------RAVGLDP-------------------SAEEIADALWLAGH- [illegible]
KOU20535.1        -----MASLL----------RAVGLDP-------------------SAEEIADALWLAGH- [illegible]
WP_031184764.1    ----RLVLKL----------REIGLDP-------------------DAEQLCDALWLARW- [illegible]
WP_037824673.1    ----RLVLKL----------REIGLDP-------------------DAEQLCDALWLARW- [illegible]
[illegible]
```

[illegible]

```
OMI34266.1        VR---V-----R--VPEP--G-----------A--P---N-------------G------T--------WR---------------------------------------SGDGAPWPG--------------------------GE-------------PP-----------------VPVLEL-EA-------------R-W--LGRWARLVTQPSA---------HGRDTLALLPGV
WP_018535458.1    GA---S----NVSLGHGAP--G----------EG--P---D-------------P-----------------LR---------------------------------------PLPEAGDDA--------------------------GE-------------VP-----------------VPVLSL-KP-------------G-S--IAAWADLV---TG------E---RGVR--RALPIV------LA
WP_037691437.1    LD---A------A----GF--G-----------A--L---N-------------R------H----------FL--------------C-------R----------PPHGVPDQAHLLPDQDD------------------------GS-------------VP-----------------VPVLSL-RP-------------G-S--FRAWADLV---TG------E---RGVR--RTLPVV------LA
WP_073719856.1    FR---A------ELDAAGF--G-----------ALNRH--------------------------------FH---------------------------------CRPPHGEGERARRLPDPDD--------------------------GS-------------VP-----------------VPVLSL-RP-------------G-S--FRSWADLI---TG------E---RGVR--RTLPVV------LA
WP_041318577.1    AP---R-------------P--G-----------C--P---N-------------R------A----------LR--------V-AGKTDS--------------------------------------------------------DV-------------IP-----------------VPVLRL-AE-------------D-W--MAGWARLL---TS------P---GAEW---VETT------AA
WP_063820828.1    AV---G------D----GP--G--------NRLLV--A---D-------------P--PPRIP---------WD-----------------------------APPPATDLHGVALPYPD---------------------------NA-------------VP-----------------VPVLQL-DP-------------T-S--LGNWAGLV---AG------T---LPGW---TDLA------AT
WP_076994244.1    SS---P-------------TP--G-----------A--A---N-------------S------------------------------------------------------MLRVDPAPH--------------------------DG-------------VV-----------------VPVLSA-TA-------------E-W--LSGWATLV---AG-----------GATE---TTVI------NA
WP_020126655.1    LTLEAGGFGAGNRGLGIGPPLSG-----------P---D-------------P-----------------VR---------------------------------------PFPDVAHED--------------------------LA-------------IPVLSL-KP-------------G-S--LAAWADLV---VG------A---RGVR--RELPAV------LA
WP_026468526.1    HR---A----LLA----SP--G---------AFQ--P---N-----------------------RRYEFDLPDAWM--------------------------------------TMADPDKAVA--------------------------GA-------------VA-----------------VPVLEL-DG-------------R-W--FSWWVRLV---SR------S---VTDR---VPAT------VL
WP_051807270.1    LR---R------P----RP--G-----------A--A---T-------------G---ELL----------VR-----------Y-------S--------------GMTSVFDPP------------------VRP---------GS-------------LA-----------------VPLIEL-SR-------------R-W--LSWWAQLV---TG-----------PPVW---TAAT------VH
WP_073900317.1    SA---P-------------AP--G-----------A--P---N-------------R------------------LL---------------------------------------RVAGADAG--------------------------GG-------------MA-----------------VPVLGL-SA-------------E-W--MAGWARLI---TT------P---GSEW---VETT------AA
KIH98248.1        LE---A------P----RV--N-----------A--P---N-------------A------L----------LR----------V-------V-------------P--------RDVSAQPAL--------------------------AG-------------VA-----------------VPVVEI-EE-------------R-W--LSVWARLI---GD------P---ALPP---LPGA------AL
OKK13505.1        LE---A------G----GL--G-----------A--A---N-------------D------C----------LA----------L-------R----------PPTQGPDPMRPLPSVGD--------------------------GV-------------AP-----------------VPVLSL-KS-------------G-S--IAAWADLV---------T---GGRGVRRALPAV
WP_040273873.1    LE---A------P----RV--N-----------A--P---N-------------A--LLRVV------------------------------------------P--------RDVSAQPAL--------------------------AG-------------VA-----------------VPVVEI-EE-------------R-W--LSVWARLI---GD------P---ALPP---LPGA------AL
WP_073930805.1    LE---A------G----GL--G-----------A--A---N-------------D----CLA----------LR------------------------------PPTQGPDPMRPLPSVGD--------------------------GV-------------AP-----------------VPVLSL-KS-------------G-S--IAAWADLV---AG---------T---GGRGVRRALPAV
WP_083466592.1    SS---P-------------TP--G-----------A--A---N-------------R-------------------------------------------------------MLRVEPEPH--------------------------DA-------------IA-----------------VPVLAM-SA-------------E-W--LAGWATLV---AG-----------GATETTAIHVSP------AW
WP_082114514.1    AN---L------S----VP--A----------ALR--P---N-------------S------R----------WT-------------F-------------------DLPDAWLDDDPAAALRD---------------------------DA-------------VP-----------------VPVIEL-QP-------------R-Y--LRWWARLI---TA------T---GHRR---PAGG------TV
WP_030212926.1    LE---A------G----GL--G-----------A--A---N------------------------------DRVVLR---------------------------------------PPPQGPDPL--------------------RPLPSTGEGV-------------LP-----------------VPVLSL-KP-------------G-S--LAAWADLV---TG------E---KGEC--RTLPAV------LS
WP_016188158.1    AP---A------N--RHYT--A-----------R--P---F--------------Q-------------------WP---------------------------------------DGLPLPEPE-----------------------------------------------FPHSALSVV-----------LPVLVS-RP-------------E-M--IHAWARFV---MG------T---RQRT---LNAG------AL
SEF26728.1        TT---P-------------GP--F-----------R--P---N-----------------------GRYGFELPDAWM------------------------------------TMADPEKAVA--------------------------GA-------------VP-----------------VPVLEL-DG-------------R-W--FGWWVRLV---SR------S---VGEP---VPGT------VL
WP_065913170.1    LR---------------MPAAG-----------A--P---N---------------------------------------------------------------SRIRYLLTDPSDERPPD--------------------------TA-------------VP-----------------VPVLEL-RP-------------D-W--LEHWTRLV---TA-----------AGPA---VHLT------TF
WP_030328546.1    VR---P------T----RP--A-----------A--P---A-------------------A---QLHPRLR----------------------------------------------DEVPDHLPR--------------------------GA-------------VA-----------------IPVLEL-AP-------------R-W--FAAGAALV---SG------R---TLGP---VAAV------AL
WP_053653628.1    VR---P------T----RP--A-----------A--P---A-------------A--QLHPR----------LR-----------------------------------------DEVPDHLPR--------------------------GA-------------VA-----------------IPVLEL-AP-------------R-W--FAAGAALV---SG------R---ALGP---VAAV------AL
WP_015660488.1    WT---A-------------------G-----------R--P---P-------------------------------WE------------------------------------PAEAVSAVLRARAGLGA---------------------------A-------------TP-----------------VPVLEA-RP-------------E-W--LEPWARFV---GG------D---EPRE---VELA------AV
SF006617.1        AN-----------Q--RLEF--G-----------D--P---T-------------G-----------------------------------------------------------GPDPTRPLPR-----------------------TDHDA-------------LA-----------------VPVLSL-KP-------------G-S--LATWAELV---AG------K---SGLR---ELPV------LT
WP_036013968.1    LR---M------P----DA--G-----------A--P---N-------------A--RIRYR--------LA-------------------------------------------DPSDERPPD--------------------------TA-------------VP-----------------VPVLEL-RP-------------D-W--LGHWTRLV---TG-----------DGPA---VRLT------TL
SDM15376.1        LR---M------P----DA--G-----------A--P---N-------------------A---RIRYRLA------------------------------------------DPSDERPPD--------------------------TA-------------VP-----------------VPVLEL-RP-------------D-W--LGHWTRLV---TG-----------DGPA---VRLT------TL
SEG88246.1        LD---A------H----RP--A-----------L--P---N-------------R---LVRVREIRSHSSWL------------------------------------------SEPSRVPPY--------------------------AP-------------VP-----------------VPLMEL-TP-------------G-S--LGRWAKLV---AT-----------GGHH---DMAV------LL
WP_051766461.1    AP---A-------------P--G-----------T--P---N-------------S------R----------LR---------V-------H---------------------------------------------------------GP--------------ATHATA------------VPVLGL-SA-------------E-W--LAGWTRLL---TA------P---GSEW---VETT------AA
CEL17037.1        LS---A------S----AL--G-----------V--A---N-------------R------E----------LR--------V-------R----------RIEHDIFDDEVPD--------------------------GS-------------IP-----------------VPVLAL-TP-------------E-W--LSGWANLFAASGS----------ASVW---TTAV------ML
WP_042186031.1    SA---S-------------AL--G-----------V--A---N-------------R------E----------LR----------------------------VRRIEHDIFDDEVPD--------------------------GS-------------IP-----------------VPVLAL-TP-------------E-W--LSGWANLFAASGS----------ASVW---TTAV------ML
SEF23786.1        LH---R------V----RP--G-------------------------------------------------------------------------------------------------------------------------TA-------------NP-----------------IPVLEL-DP-------------R-H--VRRWAELI---SG------T---PAVQ---LPVL
SFW79240.1        LR---G------V----LP--G-------------------------------------------------------------------------------------------------------------------------TA-------------NP-----------------IPVLEL-AP-------------R-H--LRRWADLV---SG------T---TGVR---LPVL
WP_072478272.1    ------------------P--G-----------T--A---N-----------------------------------------------------P-------------------------------------------------------------------------------------IPVLEL-AP-------------R-H--LRRWADLV---SG------T---TGVR---LPVL------VP
WP_026401298.1    ----------------QA--G-----------P--P---N-------------S--RLTVR-------------------------------------------SRDGFMLPP--------------------------GT-------------A-------------------IPVLEL-AP-------------R-W--LRPWAEMV---GG-----------GARQ---ITTM------AM
OHV36996.1        YQ---D------D----NP--A-----------E--T---E-------------A-------------------------------------------------------------------------------------------------------------VA-----------------VPVLLA-HP-------------V-S--IGRWADHL---AG------QDV-GEPW---LPVL
WP_007516496.1    QY---Q------D----NP--A-----------E--T---E-------------A-------------------------------------------------------------------------------------------------------------VA-----------------VPVLLA-HP-------------V-S--IGRWADHL---AG------QDV-GEPW---LPVL----PAL
WP_033433400.1    SA---P-------------AP--G-----------T--P---N-------------S------R----------LR--------------------------------------VHGPATHA---------------------------TA-------------------------------VPVLGL-SA-------------E-W--LAGWTRLL---TA------P---GSEW---VETT------AA
WP_063798505.1    SR---V----QLR--VQEA--G-----------A--P---N----------RRIRYRLA---------------------------------------------------DVSDERPPD--------------------------TA-------------VP-----------------VPVLEL-RP-------------A-W--LGHWTQLV---QG-----------DDPV---VRLT------TF
ALG12738.1        LS---A------G----AL--G-----------V--T---N-------------R------E----------LR---------V-------R----------RIEHDIFEDDPAG--------------------------DS-------------IP-----------------VPVLAL-TP-------------E-W--LSGWANLV---AA------A---GAAP---VETT------AV
WP_054294630.1    SA---G-------------AL--G-----------V--T---N-------------R------E----------LR-------------------------------VRRIEHDIFEDDPAG--------------------------DS-------------IP-----------------VPVLAL-TP-------------E-W--LSGWANLV---AA------A---GAAP---VETT------AV
WP_020641866.1    VS---Q----TLR--GVLP--G-----------------------------------------------------------------------------------------------------------------------------TA-------------NP-----------------IPVLEL-EP-------------R-H--LRSWAELI---SG------V---AGVR---LPVL
WP_076994249.1    LS---A------S----AL--G-----------V--A---N-------------R--ELHVR---RIEHDIF--------------------------------------DDDVPG--------------------------DS-------------IP-----------------VPVLAL-TP-------------E-W--LSGWANLV---AA------S---GAAP---VETT------AV
WP_034272253.1    VR---S------P----RA--A-----------A--P---N-------------S----RLA----------VR-------------------------------EADRDHDPFAQPGLRPE--------------------------------------------FP-----------------VPVLEL-NA-------------R-W--IGWWAKLV---TA------P---PDAW---VKAV------LL
WP_021593015.1    FD---A------S----GP--G-----------V--A---N-------------E--QLAVT----------RT--------------------------------------QLQLPDDRP--------------------------QG-------------IP-----------------IPVVEL-SE-------------R-W--LRSWADMV---AR-----------GGRD---VMGA------AL
KYK14919.1        LE---A------G----GF--G-----------A--A---N-------------------D-----GLGHWA----------------------------P--------PGGPDPLRP-----------------LPEADD------GS-------------LP-----------------VPVLSL-KA-------------G-S--LGTWADLV---TG------E---RGVR---RSLPV------VA
SFN86739.1        FD---A------S----GP--G-----------V--A---N-------------E------Q----LAVTRTQ--------------------------------------LQLPDARPE--------------------------G--------------IP-----------------IPVVEL-SE-------------R-W--LRSWAGMV---AR-----------GGRD---VMGA------AL
WP_075020771.1    FD---A------S----GP--G-----------V--A---N-------------E--QLAVT----------RT--------------------------------------QLQLPDARP--------------------------EG-------------IP-----------------IPVVEL-SE-------------R-W--LRSWAGMV---AR-----------GGRD---VMGA------AL
```

[illegible]

[illegible]

```
[illegible]
```

[illegible]

```
[illegible]
```

```
################################################################################
4. VMAP-C alignment
################################################################################
```

Part 1

```
[illegible]
```

[illegible]

[illegible]

[illegible]

SupplementaryDataFile.3.txt

[illegible]

```
PNG91195.1  1  [125..217]        ----------------------------------------G---QLCRTAA-GP-L-Q-D---VPA---VTS----PW-HAFEVLSR-MNAQP-G-----------------G-LPPQLALVEYL-A--AAA---RP-----LH--RADA------LREWADEQARE-LGLT----PQL-----R----SLR---QQV
WP_089510160.1  1  [105..218]    LPTDA-TGRL--RAL-L------EGL-EVA--------Q-LT---RLCQGAA-GP-L-Q-E---VPP---VTG----PW-HAFETLSR-MNARP-D-----------------G-LPPNLALVEYL-A--ASA---RP-----LE--LADH------LRSWADEQAGG-LSLI----SPL-----R----SLR---QQV
WP_033433380.1  1  [104..218]    FPTND-RARL--FTL-L------AGI-AVP--------D-IA---DIYRAIA-GP-S-APN---LY----GPT----TLTEIFRILET-FNAGP-D-----------------G-LRPLVFLEHV-A--TRV---R-----TE--LSIE------LQRWTYEQASK-MGLA----DEL-----V----EAR---EKL
OKI29919.1  1  [106..226]        LPTGD-WDRL--AKA-L------RSV-RLSDDPFEERRE-LR---RLADVAT-DG-H-CDD---LPVR---CRS----VW-SLFLHLAD-HNAGS-G-----------------A-LPPAMVLVDCL-A--GRL---ED-----SA--LAAE------LRRYNWRLAEK-FEVT----DLV-----E-QA-RWR---NET
WP_103348679.1  1  [95..200]     -------EPI--RRM-L------SDV-RLP------Y-K-PK---ALVREAR-LG-L---P---VPD---VDD----HW-QMFLLLSD-FNSAP-G-----------------E-LPPALAFVEVL-A--AKL-------D-QP--VRDE------LKAWVDLQAAR-LRLE----PGL-----A----SLR---ARM
AGP53014.1  1  [107..220]        LPETA-IAKL--RSL-L------EEL-EIE--------Q-LG---QLCRTAA-GP-L-Q-D---VPA---VTG----PW-HAFEVLSR-MNAQP-G-----------------G-LPPGLALVEYL-A--AAA---RP-----LQ--RADA------LRDWADERARE-LGLT----PQL-----R----SLR---QQV
WP_051580014.1  1  [208..318]    LPRQE-MERL--RPW-L------VDI-AVS--------Q-LP---TLVHRAG-GP-G-V-S---PAWN---AAN----AW-EAFSYLAE-FNAAP-D-----------------G-LPPALMFVELA-A--RQI---G-----GE--IGTK------LMQWNDDQARR-LRLT----SVL-----R----ERR---ELV
WP_051735807.1  1  [101..198]    LPSAE-QDRL--REW-L------SGI-VFP--------G-LS---ALMRRAG-GP-G-M-P---RSSG---VHN----AW-DAFSYLAD-FNAGL-D-----------------G-VPPAFAFLELV-A--HRI---G-----GQ--LRGR------LIEWNDFQARR-LRLE----P------------------------
WP_018383822.1  1  [78..185]     YGGRD-WTAL--HRA-L-------DF-HLP--------G-LS---VLVADVT-LG-R-V---R-LPAS---SVT----AW-DAFVHLSV-HNAPP-G-----------------G-LPPGMAFLEHL-L--HQP-------EA-AS--AVGE------IRAWNDHFAQQ-WRLT--A-EVA-----G-----LR---GHL
WP_076994247.1  1  [76..184]     WSPGE-RRQL--FAM-L------TGV-VVP--------D-IA---RIYRFVA-GA-N-APQ---LR----AQA----TCSEVFLALET-LNAGP-D-----------------G-VPKPLILVEHL-A--VRV---R-----PD--LAAE------LRRWVDEQAAR-IGVA----AEA-----R----TVR---KTI
WP_084434767.1  1  [202..314]    LRERS-IDRL--REL-L------DDL-TLP--------Q-LG---LLRARVL-GP---SEP-Q-MSG---RPS----AW-EVAQDLMD-RNTVP-G-----------------E-LRPVLMFVEFL-A--GEV-------H-ES--LSAE------IRAWNDTQAEH-LRQT----ETL-----G----RLR---AET
WP_042196291.1  1  [638..745]    ISPED-RRQL--FTL-L------AGV-VIP--------D-IG---DVYRLVA-GE-A-ALD---LP----EHT----TYQELFRALET-LNSAS-S-----------------G-IPKPVLFVEHL-A--NRV---R-----LD--LALE------LRRWVSVQIGK-LGLD----TEL-----V----QLR---EQL
SDF68729.1  1  [238..349]        TPAEE-HKDL--RGL-L------NEV-KTP--------H-LP---MLVSRAT-RS-A-V-P---AQ-P---FRS----AW-EAYEYLTD-VNAGP-D-----------------G-VPPAVTFVQLL-A--GEV---G-----GD--TGDH------LMEWVERSGRK-LRRT----RVL-----R----EQH---ETR
WP_093783487.1  1  [104..220]    LPPEQ-EERL--RDL-L------KDL-TTP--------R-LD---QLCRDAA-GD-L-H-DPYDLPA---FDD----AW-SAHQALSG-LNARE-D-----------------G-LPPALMFVELV-A--AEV---A-----YDFDRAIA------LRGWNDRQAAA-LGLA----SEL-----G----VLR---QRV
WP_086663819.1  1  [47..167]     LPTNE-WASL--RET-L------LTL-CLA--DDEAT-E-VALLRKLVGIAT-GY-R-VLE---LPRS---HRT----IW-AAFLHLAG-ANAAA-G-----------------S-LPPAMSFLECV-A--EQI-------D-ND--MIAT----D-LRRRVTAWAEQ-FELT----DRL-----A----HTK---ERP
WP_090046758.1  1  [233..344]    TPAEE-HKDL--RGL-L------NEV-KTP--------H-LP---MLVSRAT-RS-A-V-P---AQ-P---FRS----AW-EAYEYLTD-VNAGP-D-----------------G-VPPAVTFVQLL-A--GEV---G-----GD--TGDH------LMEWVERSGRK-LRRT----RVL-----R----EQH---ETR
WP_091388959.1  1  [105..218]    VTEYD-RQEL--LNL-L------GEN-PSS--------R-LA---ELVRAAA-GP-A-A-E-L-TSA---DHR----PA-DALATLER-MNAQP-D-----------------G-IPPLLLFVEYL-A--AAA---DE-----Q---RAEG------LRDWNDRQAAR-LGVT----EQI-----G----AVR---LAR
WP_041312589.1  1  [91..205]     LPTRD-WDRL--REV-L------RSI-RLAEGESAERRL-LR---ELVRTST-VG-K-CEE---LPAG---CRS----TW-TTFLYLAN-RNTSP-N-----------------R-LSPVMVFVAQL-A--DRV---AD-----RA--VAEE------LLRWNWRWAER-FEVT----DLM-----A-AD-RRR---P--
CCH29660.1  1  [108..222]        LPTRD-WDRL--REV-L------RSI-RLAEGESAERRL-LR---ELVRTST-VG-K-CEE---LPAG---CRS----TW-TTFLYLAN-RNTSP-N-----------------R-LSPVMVFVAQL-A--DRV---AD-----RA--VAEE------LLRWNWRWAER-FEVT----DLM-----A-AD-RRR---P--
WP_086843988.1  1  [89..203]     LPDSE-LLWL--RST-L------AGV-APP--------R-LG---ALLRRSA-SP-AVC-P---AG-E---PGD----AW-EAFCLLTE-LNTPA-H-----------------G-LPPALTFVELV-A--AEC---P-----RA--LGDA------LREWNTWQARR-VQGE----ARL-----H----ELR---AEH
WP_086560182.1  1  [932..1050]   LSRED-EASA--RTL-LDKSA--GNP-PGN--------S-LR-D-VLVDELG-GL-D-------LPTG---LSP----V--QLFVHLLE-SNTQP-D-----------------G-LPPAVLLVDCA----AQL---------VAW-DPDH--GVA-LARWARDWAQR-AGLL----TAL-----E----RRR---ADR
WP_099941837.1  1  [124..232]    ------WQFL--GAT-L------GTL-AMS------Y-A-MR-T-ALVRTAT-EA-R-VSA---PPPH---ADT----CW-HDFLHMAG-QGAPR-G-----------------G-LPPWMVYLDRT-A--DAM---------G-HP--VAVE------LLARNQWALR-CGLA----ELL-----D-L--DRA---RTP
EMD29441.1  1  [191..302]        IPRTD-LVTL--RHR-L------TRM-DVP--------H-LA---VMMSRAL-GP-A-V-P---LPRLENVTD----AW-SALLVLSD-YNAGP-D-----------------G-IPPAITFLRLL-A--AQL---D-----GE--AGAS------ITAWINDQARA-LRLG----PML-----A----RQA---ETR
WP_086858624.1  1  [97..190]     LPEPE-LWWL--RAR-L------AGV-VPP--------R-LT---ALLRKAA-GP-A-E-P---PA-D---VRD----AW-DGFCALSD-FNTAA-N-----------------G-LPPALAFVEFV-A--AKC---A-----PA--LAGE------LRHWNSGQARR-LQ------------------------
EWM11540.1  1  [56..159]         LLPAE-RADL--RKL-L------SPL-TSE------V-D-AH---SVYRAAA-GP-L-APP---LESA---PDD----LL-GVADELVN-MMTPP-G-----------------Q-LPPLLNFVEFL-A--AFV---DGR-----GP--SAQA------LRRWNADVARR-CGLP----QQL------------------
WP_043716378.1  1  [93..196]     LLPAE-RADL--RKL-L------SPL-TSE------V-D-AH---SVYRAAA-GP-L-APP---LESA---PDD----LL-GVADELVN-MMTPP-G-----------------Q-LPPLLNFVEFL-A--AFV---DGR-----GP--SAQA------LRRWNADVARR-CGLP----QQL------------------
WP_025356471.1  1  [105..231]    LLPAE-RTAL--RRL-L------TGL-TVE------V-D-VL---ALHRAAV-GP-I-GPP---LTVP---PTD----LL-AVLDELAN-TMCPP-G-----------------Q-LPPLFVFVEHV-A--CAV---DGG-----GY--TAAA------LRQWNSDVAHR-CELP----PQLLAVARE-GA-HSR---VEK
WP_093948307.1  1  [106..215]    VSEAE-QEQF--RRQ-L------EGF-SPP--------G-LR---AAVRRAA-RY-V-V-P---AP-R---YAD----AA-EAFFGLAD-FNAAP-G-----------------E-LPPVVAFAELI-A--AEC---D-----DR--LAAR------LREWTDGQAHH-LRLE----DAL-----R----ELR---AHV
WP_095854147.1  1  [124..232]    ------WQFL--GAT-L------GTL-AMS------Y-A-MR-T-ALVRTAT-EA-R-VSA---PPPH---ADT----CW-HDFLHMAG-QGAPR-G-----------------G-LPPWMVYLDRT-A--DAM---------G-HP--VAVE------LLARNQWALR-CGLA----ELL-----D-L--DRA---RTP
WP_054233091.1  1  [124..232]    ------WQFL--GAT-L------TTL-AMS------Y-A-MR-T-ALVRTAT-EA-R-VSA---PPPH---ADT----CW-HDFLHMAG-QGAPR-G-----------------G-LPPWMVYLDRT-A--DAM---------G-HP--VAVE------LLARNQWALS-CGLA----ELL-----D-L--DRA---RTP
KOX12354.1  1  [107..227]        LPTGD-WDRL--AKA-L------RSV-RLSDDPFEERRE-LR---RLAEVST-DG-H-CDD---LPAR---CNS----VW-SLFLHLAD-HNAGS-G-----------------A-LLPAMVLVDCL-A--GRL---GD-----SA--LAAE------LRRYNWRLAEK-FEVT----DLV-----E-QA-RWR---NET
WP_060900492.1  1  [124..232]    ------WQFL--GAT-L------TTL-AMS------Y-A-MR-T-ALVRTAT-EA-R-VSA---PPPH---ADT----CW-HDFLHMAG-QGAPR-G-----------------G-LPPWMVYLDRT-A--DAM---------G-HP--IAVE------LLARNQWALS-CGLA----ELL-----D-L--DRA---RTP
WP_072478274.1  1  [105..218]    VTEYD-RQEL--LTL-L------GEN-PSS--------R-LA---ELVRAAA-GP-A-A-E-L-TSA---DHR----PA-DALATLER-MNAQP-D-----------------G-VPPLLLFVEYL-A--AAA---DE-----Q---RAEA------LRDWNDRQAAR-LGLT----EQI-----S----AVR---LAQ
SEF89110.1  1  [102..215]        LPPEQ-ESVL--RQL-L------KGL-VTP--------R-LP---QLCQDAA-GE-L-H-DPPDPPP---DD-----AW-SAYEALSG-LNARE-D-----------------R-LPPSLALVEYL-A--AEV---A-----YD--QAIA------LRAWNDRQAAA-LGLV----SEM-----G----VLR---QRV
WP_030495369.1  1  [113..231]    LSPAE-LHQA--RGL-L------ARI-RHL--------D-AP---GLLYAAA-GE-L---P---LPPAP-VTG----LG-EAFDLLVR-VNARP-D-----------------G-LLPVNVLVEHV-I--AAL---DGSDAH-RG--VADD------LRAWNDARAAA-LHLT----PQL-----R----AVR---ADI
WP_103542176.1  1  [121..236]    ISRED-ELGA--RAV-L--SR--GEL-PAT--------R-LR-D-ALVEELN-GR-D-------LPTG---LTA----D--QLFSHVLG-WTAQL-D-----------------G-LPPAVLLVDHA----ARL---------AA--TPAH--HDA-LSGWVGDWARR-AGLT----AQL-----E----GRR---QAA
WP_067376404.1  1  [124..232]    ------WQFL--GAT-L------GTL-AMS------Y-A-MR-T-ALVRTAT-EA-R-VSA---PPPH---ADT----CW-HDFLHMAG-QGAPR-G-----------------G-LPPWMVYLDRT-A--DAM---------G-HP--VAVE------LLARNQWALR-CDLA----ELL-----D-L--DRA---RTP
WP_073939276.1  1  [122..236]    LTAQD-ESTA--RAL-L--ARAQGEI-SAR--------R-LR-D-GLVGDLN-GL-Q-------LPAG---LTP----E--QLFTHVLE-WNVQP-D-----------------G-LPPAVLLLDHA----ARL-------------TPTH--RAA-LTVWADGWAQR-AGLA----DAL-----E----RRR---AER
WP_030825278.1  1  [107..220]    LPGTA-TARL--RPL-L------EEL-EIE--------Q-LG---QLCRMAA-GP-L-Q-D---VPA---VTD----PW-HAFEVLGR-MNAQP-G-----------------G-LPPQLALVEYL-A--AAA---RP-----LR--RADE------LREWADEQARG-LGLT----PQL-----R----SLR---QQV
WP_103348476.1  1  [88..197]     VSQQQ-QEAV--RPL-L------TGL-RPR--------T-MP---LAARQAA-GN-V-V-P---LP-R---FED----AW-SAFCQLAD-LNSAP-G-----------------E-LPPAVSFVELI-S--SEY---Q-----DP--LRAN------LRAWTTAQART-LRQD----AAL-----R----ELR---DHE
WP_060948652.1  1  [107..220]    LPGTA-TARL--RPL-L------EEL-EIE--------Q-LG---QLCRMAA-GP-L-Q-D---VPA---VTD----PW-HAFEVLGR-MNAQP-G-----------------G-LPPQLALVEYL-A--AAA---RP-----LR--RADE------LREWADEQARG-LGLT----PQL-----R----SLR---QQV
WP_060945843.1  1  [107..220]    LPGTA-TARL--RPL-L------EEL-EIE--------Q-LG---QLCRMAA-GP-L-Q-D---VPA---VTD----PW-HAFEVLGR-MNAQP-G-----------------G-LPPQLALVEYL-A--AAA---RP-----LR--RADE------LREWADEQARG-LGLT----PQL-----R----SLR---QQV
WP_052684475.1  1  [217..329]    FPGDD-LKVL--QSL-L------SGV-ETA--------R-LP---MIVSRAT-HS-A-I-P---VP-R---FRS----VW-DAFLHLAD-VNAGP-D-----------------G-IPPALEFLALL-A--ADL---G-----DD--NAAE------LAAWVAQKIHN-LRRT----GEQ-----R----ERQ---AVR
WP_062668507.1  1  [121..234]    ---TDSWDFL--TGT-L------GAL-SMS------H-E-MR-T-ALVRAAT-DN-R-VAR---PPAH---AEN----PW-HDFLHMAG-QGAPR-D-----------------G-LPPWMLYLDRS-T--DSM---------N-TT--LAGE------VLARNQWALR-YGLA----GLL-----D-L--ERAHRFRSP
WP_091292750.1  1  [102..216]    VPERA-RDEL--LGL-I------SGM-SED--------R-LV---DFVHAAV-GPVA-A-E-L-AID---HQR----PE-EALAFLEQ-VNARP-D-----------------G-VPPLLVFVEYL-A--RHL---GD-----Q---RCEQ------LREWNDRQANR-LGIT----EKM-----R----ALR---IEH
WP_064455399.1  1  [107..220]    LPATA-TARL--RPL-L------EEL-EIE--------Q-LG---QLCRMAA-GP-L-Q-D---VPA---VTD----PW-HAFEVLGR-MNAQP-G-----------------G-LPPQLALVEYL-A--AAA---RP-----LR--RADE------LREWADEQARG-LGLT----PQL-----R----SLR---QQV
WP_099870403.1  1  [98..209]     LAESE-LLAL--EGL-L------SQV-PSD--------D-VQ---VIAQASL-QP-L-ASP---LPVH---CTH----AW-SILLHLLR-RNALP-N-----------------G-LPPFMAFLEYL-A--AAV-------D-DG--IGEQ------IRSWTFRHAQA-SGLL----PSL-----R----DCR---KRA
SDY41493.1  1  [104..218]        VPERA-RDEL--LGL-I------SGM-SED--------R-LV---DFVHAAV-GPVA-A-E-L-AID---HQR----PE-EALAFLEQ-VNARP-D-----------------G-VPPLLVFVEYL-A--RHL---GD-----Q---RCEQ------LREWNDRQANR-LGIT----EKM-----R----ALR---IEH
```

[illegible]

```
WP_114950516.1  ILTPLAASLLVLLKKM--AVGNARLHRIYVRS-L-----PNPMREL-GNVSIERWLEDLVDSRRRTESFPSPLIEFAERL----AR-ELKDEKLKNWVCDATCDALNERASLYNALEAE-ALANLPKA-
WP_107142264.1  ------------VDQV--ALGNSTLYRLYVQS-L-----PQAADAQDASVSPAAALRHLSELHPRKPGAPSPLAEFMVRL----AA-ECANTKVDDWINQNVTPQ--ARTQLEQVLAKE-GAAD----
WP_100179618.1  ---QIADDLTELVMAL--GVSRSTLERHFLAT---ARA-MKNDRLL-LAQGISGMIRELNGVGERVPRATYAIVQFVLRLCGDPSN-AKHKSALKDWLSLHATEAVIS--------------------
WP_093391206.1  ---PRLDDALAAIQQV--AITDAQVYRLYVRS-L-----TLSADAVTPPVGLAVVLKLLHDLNPSRPGGPDPMSEFMMRA----GR-EFPDAAIQAWIHKHLTPQF--RAQIDATLDKE-A-------
WP_093036291.1  -GSALFGSVWSSLCAI--PVPDSFYRLHFRRT-VSSLGRPDLDHAQ-GLSGMLRELTSLCDTAGTTQA-PYGLLEFLLRL----SSDARLAEAVEAWLNEHAAAQGNA--------------------
WP_092797644.1  ------ADLLALVRTHIPDVTLARYERLYLRS-L-----PNPTRAV-QQGGAEDWLRHLHDVPVADTHRPPPLAEFAIRL----AK-ETGATVLEDWAASA---------------------------
WP_076913892.1  ---ALFESAWNLLRGL---DAHAFFRRHYERT-V-----ADLGVPA-RAHDLTSALQDLCEMRDDKGAVPHGLMQFMMRL----ATVPALSAPIKNWLDIEAKDQPHSRQEIADKLARE-GLQRLLLVE
WP_064832779.1  ILTPLAASLLVLLKKM--AVGNARLHRIYVRS-L-----PNPMREL-GNVSIERWLEDLVDSRRRTESFPSPLIEFAERL----AR-ELKDEKLKNWVCDATCDALNERASLYNALEAE-ALANLPKA-
WP_048244481.1  -LSSLARDLLSVTAGL--RLNPKSTQRAYLRS-L-----PSSGRSV-TGTDLQAYIKDLDDTRPEFDDAAPPLIEFAKRL----AD-ICNKPELAQWARANAPDG--TYMLLEGKLDAELRVAQRPHA-
WP_045051688.1  AGLAYLDSATNLLRRL--DVTDTEVRFHYERTAI-----AMKFATLPGGTKRQDVLSHLCDLHGDVGLMPFGLLQFMMRL----SRVEDYGKEIANWLDAEAPEQQSARDAVHRLLIEE---------
VBQ80020.1      ---ALFESAWNLLRGL---DAHAFFRRHYERT-V-----ADLGVPA-RAHDLTSALQDLCEMRDDKGAVPHGLMQFMMRL----ATVPALSAPIKNWLDIEAKDQPHSRQEIADKLARE-GLQRLLLVE
ANL87879.1      ILTPLAASLLVLLKKM--AVGNARLHRIYVRS-L-----PNPMREL-GNVSIERWLEDLVDSRRRTESFPSPLIEFAERL----AR-ELKDEKLKNWVCDATCDALNERASLYNALEAE-ALANLPKA-

================================

VMAP-M5
HMM             --HHHHHHHHHHHHHHHHHHHHHHHHHHHHHHHH---HEEHHHHHHHHH--------E---EHHHHHHHHHEEEEE-------EE-------HHHHHHHHHHHH-EEEHHHHHHHHHHHHHHH---HHHHHHHE-----
FREQ            --HHHHHH-HHHHHHHH-----HHEHEEEE--------H--HHHHHHHH--------------------HHH--------EEEE------EEEEEEE-EEEEEHHHH-HHHHHHHH------HHHHHHHHHHH--
PSSM            ----HHHHH----HHHHH----HHHHHHHHHH----HHHH---------------------HHHHHHHEEEE----------------HEHHHHHHHHHH-EEEE--HHHHHHHHHH------EE---------
FINAL           --HHHHHHHHHHHHHHHHHH--HHHHHHHHHH----HHHHHHHHHHHH-------------------EEEEE-------EEEE-------HHHHHHHHH-EEEEEE-HHHHHHHHHH---HHHHHHHHHHH--
WP_006194995.1  KTELKNFYSQRLKAKEVFNKYNFQTLINILQKVNEDKSVINSYNNYLENNDIPKGKINSFDISQAIDDLLMLMCSKSDGDNHISDNPHDSCYLLDFVEKFLCEIVYIQNITEDLQEWIKRNFPEVAFRNKIQIQNL
WP_017804267.1  KTELKNFYSQRLKAKEVFNKYNFQTLINILQKVNEDKSVINSYNNYLENNDIPKGKINSFDISQAIDDLLMLMCSKSDGDNHISDNPHDSCYLLDFVEKFLCEIVYIQNITEDLQEWIKRNFPEVAFRNKIQIQNL

================================

VMAP-M6
ALIGN           ---------EEHHHHHHHH-----HHHHHHHH------------------------HHHHHHHHHHHH-----------------------------EEHHHHHHHHHHH--------HHHHHHHHH-------HHHHHHHH--------------------
HMM             --------EEEEEHHHHHH----HHHHHH-------------------------HHHHHHHHHHHHHH-H----HH-HHH--------------HHHHHHHHHHHHHHH------HHHHHHHHHHH-------EEEEEEE--------------------
FREQ            --------HEE-HHHHHH-----HHHHHHHH-----------------------HHHHHHHHHHHHHH------HEE--------------------EEEEEHHHHHH---------HHHHHHHHHH-------HHHHHHHHH-------------HH---
PSSM            --------EEEEHHHHHH-----HHHHH--------------------------HHHHHHHHHHHHH-----HHHHH------------------HHHHHHHHHHHHHHH------HHHHHHHHHHH-------HHHHHHHH--------------H-----
FINAL           --------EEEEEHHHHHH----HHHHHHHH-----------------------HHHHHHHHHHHHHH------HHHH-----------------HHHHHHHHHHHHHHH------HHHHHHHHHHH-------HHHHHHHH-------------HHHHH--
WP_055491863.1  RDKSPEAGVTWMIPMARLAESWPPLRAALALSEERASARHLPRFPDHPEDDPFDPEAMDDLYRLLLDGAFSLPGLVALRDEALGPVRRPWQGAPEDILGIVHDLQHIAQAGRPVPPLLAFVAALSRRMPGTRGQELARWVRGRAGHCTPVVDPAPLLARRV
WP_055491863.1  RDKSPEAGVTWMIPMARLAESWPPLRAALALSEERASARHLPRFPDHPEDDPFDPEAMDDLYRLLLDGAFSLPGLVALRDEALGPVRRPWQGAPEDILGIVHDLQHIAQAGRPVPPLLAFVAALSRRMPGTRGQELARWVRGRAGHCTPVVDPAPLLARRV
WP_123623414.1  RDKSPEAGVTWMIPLERLARDWPPLRAALALSGDRPAPGPAPGPPD-PDDGPFDDEAMDDLYRLLLDGRLSMPGLVGLRDDALGPVRRPWQGSPEDILGIVHDLRHIAQAGRPVPPLLTFVAALSRRVPGPRGQELARWVRGRAGHCTPAVDPGPLLERRV
WP_121703873.1  RDKSPEAGVTWMIPLERLARDWPPLRAALALSGDRPAPGPAPGPPD-PDDGPFDDEAMDDLYRLLLDGRLAMPGLVGLRDDALGPVRRPWQGSPEDILGIVHDLRHIAQAGRPVPPLLTFVAALSRRVPGPRGQELARWVRGRAGHCTPAVDPGPLLERRV
WP_076973924.1  RDKSPEAGVTWMIPLERLARDWPPLRAALALSGDRPAPGPAPGPPD-PDDGPFDDEAMDDLYRLLLDGRLAMPGLVGLRDDALGPVRRPWQGSPEDILGIVHDLRHIAQAGRPVPPLLTFVAALSRRVPGPRGQELARWVRGRAGHCTPAVDPGPLLERRV
WP_107275064.1  RDKSPEAGVTWMIPLERLARDWPPLRAALALSGDRPAPGPAPGPPD-PDDGPFDDEAMDDLYRLLLDGRLAMPGLVGLRDDALGPVRRPWQGSPEDILGIVHDLRHIAQAGRPVPPLLTFVAALSRRVPGPRGQELARWVRGRAGHCTPAVDPGPLLERRV
WP_087809822.1  RDKSPEAGVTWMIPLERLARDWPPLRAALALSGDRPAPLPAPEPPD-PDDGPFDDEAMDDLYRLLLDGRLSMPGLVGLRDDALGPVRRPWQGSPEDILGIVHDLRHIAQAGRPVPPLLTFVAALSRRVPGPRGQELARWVRGRAGHCTPAVDPGPLLERRV
WP_104636012.1  RDKSPEAGVTWMIPLERLARDWPPLRAALALSGDRPPPPPAPGPPD-PDDGPFDDEAMDDLYRLLLDGRLAMPGLVALRDDALGPVRRPWQGSPEDILGIVHDLRHIAQAGRPVPPLLTFVAALSRRVPGPRGQELARWVRGRAGHCTPAVDPGPLLERRV
PPS76983.1      RDKSPEAGVTWMIPLERLARDWPPLRAALALSGDRPPPPPAPGPPD-PDDGPFDDEAMDDLYRLLLDGRLAMPGLVALRDDALGPVRRPWQGSPEDILGIVHDLRHIAQAGRPVPPLLTFVAALSRRVPGPRGQELARWVRGRAGHCTPAVDPGPLLERRV
WP_030186452.1  RDKSPEAGVTWMIPLERLARDWPPLRAALALSGDRPAPPPGPGPPD-PDDGPFDDEAMDDLYRLLLDGRLAMPGPVALRDDALGPVRRPWQGSPEYILGIVHDLRHIAQAGRPVPPLLTFVAALSRRVPGPRGQELARWVRGRAGHCTPAVDPGPLLERRV

================================

VMAP-M7
ALIGN           ------E----H--------------------HHHHHHHHHHHHHHHHHHHHHHHHHHHHHHHHHHHHH------HHHHHHHH----------HHHHHHHHHH-------------HHHHHHHHHHHHHH-------------------------HHHHHHH----------
HMM             ---------HHHHHHHHHHHHHHHHH------HHHHHHHHHHHHH----HHHEE-HHHHHHHHHHHHH----HHHHHHHHHHHHH-----HHHHHHHHHHHHHHHHHH-HHH----HHHHHHHHHHHHHHH--------------------HHHHHHHHHHH----------
FREQ            ------H-HHHHHH---HHHHHH---------HHHHHHHHHHHHHH--------HHHHHHHHHHHHH------HHHHHHHH-----------HHHHHHHHHHHHHH--HHH------HHHHHHHHHHHHHH---------------------H--HHHHHHHH----------
PSSM            ---------HHHHHHH-HHHHH----------HHHHHHHHHHHHH---HHHH---HH-HHHHHHHHHH-----HHHHHHHHHH---------HHHHHHHHHHHHHH-----------HHHHHHHHHHHHHHHH-----------------------HHHHHHH----------
FINAL           --------HHHHHHHHHHHHHHHH--------HHHHHHHHHHHHHH--------HHHHHHHHHHHHH-----HHHHHHHHHHH---------HHHHHHHHHHHHHHH-HHHH----HHHHHHHHHHHHHH-----------------------HHHHHHHHHHH----------
WP_015655514.1  LPMGGRAIGVSTVRSLFPAVWEANRAPNPLDTELWRLRAALQHEYAPGAVLSLREENALLRAARRSRLAPAALYWRSVHRDYGEPAGPIDTVADALREVADA-PAMLDGPHPLLQFIKQVMDAALPQDSG--------------PLAGLVDLVAQRLGVPTPSPVA
WP_100110114.1  --MGGLALPAAAVREYFPDVWADNQQADRPDDRLWDLVTELRHESAHRRLLSPAEHGELVAAAAGLGMDPHALYWQAVGSLGRAPDKPLEDFTALVRECADAQPARLGAVHPLVRLVELVRAEQIRGDR---ARITRAETTPDPDRLRDLPAVVASRLGQPYTPP--
WP_078951211.1  --LGGLALPASAVRDRFPDVWADNQHADRPDDHLWDLVTELRHESAHRRLLSHAERRELVAAAAGLGMDPNALYWQAVGRLGRAPDKPLEDFTALVRECADAQPAQLGAVHPLVRLVELVRAEQARGSRGHQGRIDPAGTTGVPDRIRDLPAAIASRLGQPYTPP--
WP_005309391.1  --LGGLALPASAVRDRFPDVWADNQHADRPDDHLWDLVTELRHESAHRRLLSHAERRELVAAAAGLGMDPNALYWQAVGRLGRAPDKPLEDFTALVRECADAQPAQLGAVHPLVRLVELVRAEQARGSRGHQGRIDPAGTTGVPDRIRDLPAAIASRLGQPYTPP--

================================

VMAP-M8
ALIGN           ----------HHHHHHHHHHHHHHH-------EE--EEEH--------------HHHHHHHHHH--------H-----------HHHH-------------HHHHHH---------
HMM             -----------HHHHHHHHHHHHHH-----HHHH--HHHHHHH--------HHHHHHHHHHHHHEEE-------EE-----------EEEEE------------HHHHHHHH-------
FREQ            -------------HHHHHHHHH-------------HHHHH----------------EEE-EEEEE-E---HHHH-----------HHHHHH-----------HH-------------
PSSM            -----------HHHHHHHHHHHH-------HHHH--HHHHH---------------HHHHHHHHH-------HHH-----------HHHHH----------HHHHHHHHHH-------
FINAL           -----------HHHHHHHHHHHH------HHHH--HHHHHH-------------HHHHHHHHH-------HHH-----------HHHHH------------HHHHHHHHH--------
WP_125516244.1  DDFLPLDWLPDTPRRELRALFDGVRLDPGTLEL--TVRRAFPTFRR--PLPAFRSLWHAIRYVG-EELTGGER----------LLKLLPELGAVA---RVPVGPWLRRW---MPD-
WP_103938653.1  DDLLPLDWLPPEPRRELRALVTGATSDRDLG---SVFREAFPTFLR--PPETFGSVWEAVRFVG-ETVPGQDR-------LRRFLVTLTPRLDQEA---RPRLADWLRRR---LP--
WP_090768636.1  DDVLDLPWLPQAERRELRGIVAAARSGTDLR--ELVLRVFPGSGA--HPD-FRSVWDAIRHVA-EHLSGDDR-------LRRFLYELVRTLTPAS-A-RSELRQWIREH---L---
WP_089000284.1  LDRICLGPFGAKAYRELHAILHGLELAETHRLLLEVLRQRGPLFPQ--PPAVLSNVLAAVEWLAVGTTFPREE-------VVDLVITLLRRIGQESPQRAALLHEWVGQHGWGAGAA
WP_083946411.1  DDLLPLTWLTAPARRELRGILAGVPFT-------PVLAAELAVVTGRPSPAVFQSAWHSVRHVA-EGF--REEQ-------LVRYLRAVRRASPASA---ARRLTTWSRRH---LPDG
WP_067817610.1  DDHLPLTWLDATARRELRRALAGLPAD-------PAAAARVLDLP---PTRPLRSAWDAARYIA-EGF-DEER-------LARYLAALSPD--------VPRLAGWRRRH---LPAP
WP_067464069.1  DDRLPFDWFSLPARRELRRLLAQAPSRPDPN---DVLRTAFPYLAF--APD-LTSHWEAICYIGEEVMVSVERESLRDEMLAHYLESLSPRLERTG---RRELDTWFQKW---LPDH
WP_027344132.1  GHRLRLGQYDATAYAELRGLLLRATRVRGGNLV-PWLKSRVHGLPELSDPRMLDQPMEVVEWLATQPLGDPEK-------INLYMSTLLRHIGDQENDLRPDLDGWIERH-ARATPA

================================

VMAP-M9
ALIGN           -----------EEEEEEHHH-H-----EEEEEEE-----EEEEEEH-----H-HHHHHH-HHEE------------HHHHHHHHHHH----HHHHHHHHHHH----------HHHHHHHHHHHHH-------HHHHHHHHHH-----
HMM             ------EEEHHEEEHHHHHH-HHHH---EEHHHHH---HHHHHHHHHHHHHHH-----HHHHHHHHH-------HHHHHHHHHHHHHHHHH--HHHHHHHHHHHHHHHH----HHHHHHHHHHHHHHHHHH----HHHHHHHHHHHHHHH-----
FREQ            ---E-EE----EEEEEEEEH-H-----EEEE-EEE----EEEEEEHHHHHHH-HHHHH--EEEEE------------HHHHHHHHHE-----HHHHHHHHHH----------HEEEHHHHHHHHHHHHH------HHHHHHHHHHH------
PSSM            ------H-----EEEHHHHH-------EEEEHHHHH----EEEHHHH--------HHHHHHHHHHHH-----------HHHHHHHHHH-----HHHHHHHHHHH---------HHHHHHHHHHHHHHHHH------HHHHHHHHHH------
FINAL           ---E-EEE---EEEEEHHHH-H-----EEEEHHHHH----EEEEHHHHHHHHHH-HHHHHHHHHHHH------------HHHHHHHHHH-----HHHHHHHHHHH---------HHHHHHHHHHHHHHHHH-----HHHHHHHHHHH------
WP_110578239.1  ENRE-KIQKNQFVPLITEYI-NLKNKSQIINKINVENNDKIKIFEKLNNELK-IKRPMTAAIIKSYLSDDPKIQNSNLKELIEKIKDY-EESFLFSLLHNVAYDQRLDDPLIQLIFEQTVNDLKMQTNISDLDYEQLTSTQLERIELPK
WP_103111423.1  -----KVQKTNFITLIEHWN-AIDNKGTSVENSFVKSKTKINIFTELKNKLSGQQQSELSPIIKSYLPDN---DNANFEEIIDKIINIRDDKDLLRILFRLTQDSRINSDTRSYI-EQTIQELKKLLNINDNAN---FEEIIDKIINIR
WP_079210028.1  ENRE-KIQKNQFVPLITEYI-NLKNKSQIINKINVENNDKIKIFEKLNNELK-IKRPMTAAIIKSYLSDDPKIQNSNLKELIEKIKDY-EESFLFSLLHNVAYDQRLDDPLIQLIFEQTVNDLKMQTNISDLDYEQLTSTQLERIELPK
WP_072926262.1  -----KVQKTNFITLIEHWN-AIDNKGISVENSFVKSKTKIDIFTKLKNKLSGQQQSELNQIIKSYLPDN---DNANFEEIIDKIINLPDDKDLLRILFRLTQDSRINSYNRSYI-EQTIQELKKLLNITDTDYNAWIA----EIPAIN
WP_061432002.1  -----KVQKTNFITLIEHWN-AIDNKGISVENSFVKSKTKINIFTELKNNLSGQQQAELSPIIKSYLPDN---DNANFEEIIDKIINLPDDKDLLRILFRLTQDSRINSYNRSYI-EQTIQELKKLLNITDTDYNAWIA----EIPAIN
WP_052276528.1  ENRE-KIQKNQFVPLITEYI-NLKNKSQIINKMNEENNNKIKIFEKLNNELK-IKRPMTAAIIKSYLSDDPKIQNSNLKELIEKIKDY-EESFLFSLLHNVAYDQRLDDPLIQSIFEQTVNDLKMQTNISDLDYEQLTSTQLERIELPK
WP_016516065.1  ERDIKKVNKDSVISLVEEYNRKISDNGTFLQDKLKENKTKINIFTELKNKLSGQQQAELSPIIKSYLPDN---DNANFEKIIDKIINIQDDKDLLRILFRLTQDSRINSYNRSYI-EQTIQELKKLLNITDTDYNAWIA----EIPAIN
WP_012266974.1  -----KVQKTNFITLIEHWN-AIDNKGISVENSFVKSKTKIDIFTELKNKLSGQQQAELNLIIKSYLLDN---DNANFEEIIDKIINLPDDKDLLRILFRLTQDSRINSYNRSYI-EQTIQELKKLLNITDTDYNAWTA----EITEID
WP_004268273.1  -----EVQKTNFITLIEHWN-AIDNKGISVENSFVKSKTKIDIFTELKNKLSGQQQAELNLIIKSYLPDN---DNANFEEIIDKIINIQDDKDLLRILFRLTQDSRINSYNRSYI-EQTIQELKKPLNITDTDYNAWIA----EIPAIN
WP_004162809.1  -----KVQKTNFITLIEHWN-AIDNKGISVENSFVKSKTKINILTELKNNLSRQQQAELSPIIKSYLPDN---DNANFEEIIDKIINLPDDKDLLRILFRLTQDSRINSYNRSYI-EQTIQELKKLLNITDTDYNAWIA----EIPAIN
WP_002786763.1  -----KVQKTNFITLIEHWN-AIGNKGISVENSLVKSKTKIDIFTKLKNKLSGQQQSELSPIIKSYLPDN---DKANFEEIIDKIINLPDKDSLI-ILFRL---SRVNSSTRSDI-EQTIQELKELLKITDTDYNAWIA----EITEIN
WP_002769615.1  -----KVQKTNFITLIEHWN-AIDDKGISVENSLVQNKTKIDIFTELKNKLSGQQQSELSPIIKSYLPDN---DKANFEEIIDKIINLPNKDSLI-ILFRL---SRVNSSTRSDI-EQTIQELKELLKITDTDYNAWIA----EITETN
WP_002757186.1  -----KVQKTNFITLIEHWN-AIDNKGISVENSFVKSKTKIDIFTELKNNLSGQQQSELNRIIKSYLPDN---DNANFEKIIDKIINIRDDKDLLRILFRLTQDSRINSYNRSYI-EQTIQELKKLLNITDTDYNAWIA----EIPAIN
WP_002755874.1  -----EVQKTNFITLIEHWN-AIDNKGISVENSFVKSKTKIDIFTELKNNLSGQQQKSELNRIIKSYLPDN---DNANFEKIIDKIIDSPDKDSLI-ILFRLTQDSRVNSSTCSKI-EQTIQDLKELLKITDTDYNAWTG----EITEIN
```
[truncated: 263,513 more chars]
